# KAIKObase: An integrated silkworm genome database and data mining tool

**DOI:** 10.1186/1471-2164-10-486

**Published:** 2009-10-21

**Authors:** Michihiko Shimomura, Hiroshi Minami, Yoshitaka Suetsugu, Hajime Ohyanagi, Chikatada Satoh, Baltazar Antonio, Yoshiaki Nagamura, Keiko Kadono-Okuda, Hideyuki Kajiwara, Hideki Sezutsu, Javaregowda Nagaraju, Marian R Goldsmith, Qingyou Xia, Kimiko Yamamoto, Kazuei Mita

**Affiliations:** 1 Mitsubishi Space Software Co. Ltd., Takezono, Tsukuba, Ibaraki 305-0032, Japan; 2 National Institute of Agrobiological Sciences, Owashi, Tsukuba, Ibaraki 305-8634, Japan; 3 National Institute of Agrobiological Sciences, Kannondai, Tsukuba, Ibaraki 305-8602, Japan; 4 Centre for DNA Fingerprinting & Diagnostics, Nampally, Hyderabad-500001, India; 5 Biological Sciences Department, University of Rhode Island, Kingston, Rhode Island 02881-0816, USA; 6 The Institute of Agricultural and Life Sciences, Chongqing University, Chongqing 400030, PR China

## Abstract

**Background:**

The silkworm, *Bombyx mori*, is one of the most economically important insects in many developing countries owing to its large-scale cultivation for silk production. With the development of genomic and biotechnological tools, *B. mori *has also become an important bioreactor for production of various recombinant proteins of biomedical interest. In 2004, two genome sequencing projects for *B. mori *were reported independently by Chinese and Japanese teams; however, the datasets were insufficient for building long genomic scaffolds which are essential for unambiguous annotation of the genome. Now, both the datasets have been merged and assembled through a joint collaboration between the two groups.

**Description:**

Integration of the two data sets of silkworm whole-genome-shotgun sequencing by the Japanese and Chinese groups together with newly obtained fosmid- and BAC-end sequences produced the best continuity (~3.7 Mb in N50 scaffold size) among the sequenced insect genomes and provided a high degree of nucleotide coverage (88%) of all 28 chromosomes. In addition, a physical map of BAC contigs constructed by fingerprinting BAC clones and a SNP linkage map constructed using BAC-end sequences were available. In parallel, proteomic data from two-dimensional polyacrylamide gel electrophoresis in various tissues and developmental stages were compiled into a silkworm proteome database. Finally, a *Bombyx *trap database was constructed for documenting insertion positions and expression data of transposon insertion lines.

**Conclusion:**

For efficient usage of genome information for functional studies, genomic sequences, physical and genetic map information and EST data were compiled into KAIKObase, an integrated silkworm genome database which consists of 4 map viewers, a gene viewer, and sequence, keyword and position search systems to display results and data at the level of nucleotide sequence, gene, scaffold and chromosome. Integration of the silkworm proteome database and the *Bombyx *trap database with KAIKObase led to a high-grade, user-friendly, and comprehensive silkworm genome database which is now available from URL: .

## Background

The silkworm, *Bombyx mori*, has been domesticated for silk production for about 5,000 years from the wild silkworm, *Bombyx mandarina*. As the only truly domesticated insect, it is completely dependent on humans for survival and reproduction. Currently, it is one of the most important economic insects in many developing countries owing to its large-scale propagation and utilization for silk production. Comparison with its wild ancestor *B. mandarina *at the genome level provides an opportunity to examine the effects of artificial selection leading to domestication. In addition, it is the model organism for Lepidoptera, the second largest order of insects, which includes the most destructive agricultural pests. With the development of biotechnology, *B. mori *has come to be used as an important bioreactor for production of recombinant proteins [1,2]. Silkworm genome information not only makes a strong impact on improving sericulture, but also facilitates the development of new methods for pest control.

Genome analyses of insects have moved rapidly in recent years, because insects are the most diverse species on earth and their characteristic biological phenomena are important resources for basic science and industry. Among model insects, complete genome sequences have been published for *Drosophila melanogaster *[3], *Anopheles gambiae *[4], *Apis mellifera *[5] and *Tribolium castaneum *[6]. In 2004, the draft whole genome shotgun (WGS) sequences of the silkworm were reported independently in Japan [7] and China [8], but these produced insufficient genome sequence information because of shallow genome coverage compared with the analyses of the other species. Subsequently, the two independent WGS data sets were merged and assembled together with newly obtained fosmid- and BAC-end sequences. Although these two data sets were derived from two different strains of silkworm, sequence comparison revealed merely 0.2% difference at the nucleotide level. In addition, p50T inbred strain, which was used for WGS by Japanese group, was derived from the same origin of Dazao strain of Chinese group. The RAMEN assembler, which is featured a lookup table generation of seed strings for highly sensitive regions and rapid detection of overlapping reads and precise alignment by efficient banded dynamic programming, was used. Additionally, RAMEN includes a repeat untangling method for transforming a repeat subcontig flanked by two unique subcontigs into one unique contig, thereby circumventing problems associated with the high density of transposable elements in the silkworm genome [7]. Among all the sequenced agriculturally important insect genomes, the silkworm genome assembly (432 Mb) has the best continuity (~3.7 Mb in N50 scaffold size) and provides extensive nucleotide coverage (88%) of all the 28 chromosomes. This was made possible by the availability of a high-density SNP linkage map constructed by the analysis of BAC-end sequences and integrated with a physical map of contigs established by BAC fingerprinting using the FPC program [9]. In a related project, EST data derived from various tissues and different developmental stages were compiled in SilkBase [10] and proteomic data of distinct tissues at different stages were obtained from two-dimensional polyacrylamide gel electrophoresis and mass spectrometry [11]. Finally, a *Bombyx *trap database has been established to provide reporter expression patterns and inserted positions of mutators of enhancer-trap [12] and gene trap lines.

With the tremendous accumulation of genomic information for various organisms, extensive tools have been developed for visualization of data and results. AceDB [13], *C. elegans *[14] was one of the pioneering databases, where genetic map, physical map, genes, clones, markers, and so on, were integrated and displayed in a format easy to see. The technique of placing linkage and physical maps side-by-side, first employed in AceDB was used widely in INE [15], NCBI map viewer [16], Cmap [17,18], and other systems. Subsequently, genome browsers which present extensive genomic information of Mb-order such as chromosomes or scaffolds were developed and available as follows. 1) Ensembl is a portal system developed to handle very large genome and associated requirements of the human genome from sequence analysis to data storage and visualization [19]. 2) GBrowse [20], which was developed for *Drosophila *genome sequences, has the browser software characterized by readily available open source components, and flexible configuration. 3) UTGB (University of Tokyo Genome Browser) was developed for Japanese Medaka (*Oryzias latipes*) [21]. Track showing information used in UTGB is an independent web application that allows enhanced expressibility and expandability of conventional genome browser. In order to construct a user-friendly and efficient genomic database for silkworm, we integrated all of the genomic sequences, map information, ESTs, proteomic data and information on enhancer trap strain into an integrated database called KAIKObase, where all analyzed results and data on nucleotide sequence, scaffold and chromosome are displayed by GBrowse and UTGB.

## Construction and content

### Data sets

#### Genomic sequence

KAIKObase contains a total of 43,462 assembled data [22], including scaffolds (accession no. DF090316-DF092116), and contigs (accession no. BABH01000001-BABH01088672) not used in scaffolds, corresponding to a total genome size of 482 Mb (403 Mb without gaps). Among them, 192 scaffolds were mapped on to 28 chromosomes. An artificial gap of 500 Kb was assigned between scaffolds so that the entire length corresponds to 503 Mb (393 Mb without gaps). In addition, a total of 81,705 BAC-end sequences (accession no. DE283657-DE378560, DE378561-DE420875), 174,222 fosmid-end sequences (accession no. DE143284-DE189151, DE246947-DE248527, DE420876-DE647768), and 166,757 ESTs [22]. The list of cDNA libraries with the corresponding accession numbers of the ESTs is provided in additional files 1 and 2 (ESTs derived from silkworm cDNA libraries).

#### Map information

The genetic and physical maps provide basic information for the silkworm genome. The combined maps contain the following type of data: 16,209 gene models, 1,532 SNP markers, 770 trait markers, and 5,419 FPC contigs. The gene models include 14,622 developed ab initio by the Chinese group using a GLEAN-based algorithm [23], as well as 1,587 genes including GPCR [22], OBP, CSP [22], cuticle protein [22], and tRNA genes [22] developed by automated and manual annotation by the Japanese group. The SNP markers were identifed from BAC-end sequences. Trait markers represent the positions of transposon vectors (mutators) in transposon insertion lines (enhancer-trap lines or gene-trap lines). The FPC contigs represent BAC contigs assembled by BAC fingerprinting.

#### KAIKO2DDB (Proteome database)

Information on the silkworm proteome is provided by the KAIKO2DDB [11]. It contains 116 images of two-dimensional polyacrylamide gel electrophoresis at different developmental stages (such as 4^th ^and 5^th ^larval instars, spinning, and pupation stages) and from various tissues (such as midgut, fat body, middle silkgland, posterior silkgland, Malpighian tubule, ovary, and hemolymph). The spots provide information on products such as molecular weight isoelectric point. Corresponding ESTs and selected gene models are also shown on the gel images.

#### Bombyx trap database

The *Bombyx *trap database contains information of 288 transposon insertion lines, e.g. enhancer-trap lines [12] and gene-trap lines, the positions of insertions in the genomic sequence, expression profiles of genes for various developmental stages, organs and tissues including intensity of expression, and associated photos.

### KAIKObase

**Figure 1:**
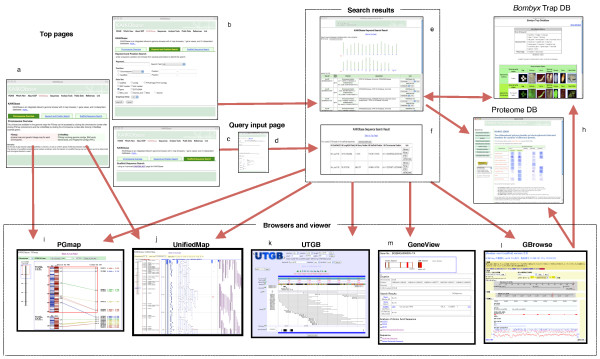
**Flow chart for browsing KAIKObase**. a) KAIKObase top page with links to PGmap and UnifiedMap; b) Keyword and position search function; c) Sequence search function using BLAST; d) Entry of fasta sequence and setting parameters; e) Result of keyword and position search; f) Result of sequence search; g) *Bombyx *trap database top page; h) Proteome database top page; i) PGmap showing an image of the genetic and physical maps; j) UnifiedMap showing the genetic map and various selectable physical map features; k) UTGB showing various selectable physical map functions; l) GBrowse showing various selectable physical map features; m) GeneViewer showing a sample gene profile.

As a portal for silkworm genome information, KAIKObase consists of four map browsers (PGmap, UnifiedMap, GBrowse, and UTGB), a gene viewer (GeneViewer), two independent databases (KAIKO2DDB and *Bombyx *trap database), a sequence search, and keyword and position search systems (Figure 1). The PGmap and UnifiedMap provide an overall view of available information for each chromosome. UTGB and GBrowse provide similar information for each chromosome at the nucleotide level based on scaffolds generated for corresponding regions. The GeneViewer shows the gene models including a description of the genes on each chromosome.

The KAIKO2DDB (Proteome database) consists of proteome data generated from various developmental stages and tissues of silkworm. The system was developed using the make2DDB II (ver. 2.50.1) package [24] from ExPASy [25]. The *Bombyx *trap database provides two data mining approaches, namely, "Keyword search" and "Pictorial search" for information on reporter expression and positions of transposon vectors (mutators) in transposon insertion lines (enhancer-trap lines, or gene-trap lines). The sequence search function provides information on the position in the genome for query sequences using NCBI BLAST software [26]. The keyword and position search function provides information on the position in the genome sequence for a query keyword as well as information for delimiting its range.

#### PGmap

**Figure 2:**
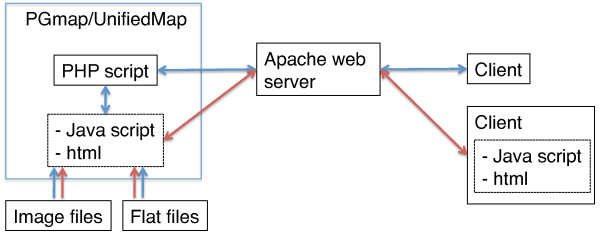
**Communication for PGmap and UnifiedMap**. The blue line represents the flow of information when accessing PGmap and UnifiedMap to select the chromosome number and to change the map scale. The red line represents the flow of information using an asynchronous communication when detailed information from UnifiedMap is selected by positioning the cursor.

The PGmap is an integration of the genetic map and physical map of the silkworm genome. It consists of SNP markers, trait markers using the *Bombyx *trap database, bar charts of repeat sequences, and bar charts of gene sequences in the visible chromosomal range. A visual comparison between the genetic and physical lengths for entire chromosomes or a specified chromosomal region can be generated by specifying a region or position in the genome. In particular, the sequence of the selected region is linked to GBrowse as described below. The web interface of PGmap uses an asynchronous communication written in Javascript (Figure 2).

#### UnifiedMap

The UnifiedMap has a similar function to the PGmap in providing an overview of the genetic map and physical map. Detailed information on scaffolds, contigs, FPC contigs, BAC ends, Fosmid ends, SNP markers, and trait markers within the chromosomal range of interest is provided. Showing or hiding physical map items is selectable by checking a box with four-stage scaling. A marker in the genetic map is linked to a marker on the physical map which is linked to the sequence information in GBrowse. The web interface also uses an asynchronous communication written in Javascript (Figure 2).

#### UTGB

UTGB provides an intermediate coverage which is smaller than the chromosomal range provided by PGmap and UnifiedMap but larger than GBrowse coverage. Track items include FPC contigs, BAC-ends, fosmid-ends, and gene models. Track showing information using in UTGB is an independent web application that allows enhanced expressibility and expandability of conventional genome browser.

#### GBrowse

GBrowse provides a tracking function for restriction sites, FPC-contigs, 6-frame translation, DNA/GC content, contigs, ESTs, transcriptional profile, BAC, BAC-end, fosmid-end, gene models and genes, SNP-markers, and trait-markers. Pop-up balloons in the gene model track show links to: 1) sequence information displayed by the GBrowse function, 2) GeneViewer with detailed information for each gene, and 3) the proteome database. Pop-up balloons associated with the trait-markers show links to 1) sequence information displayed by the GBrowse function and 2) the *Bombyx *trap database. In addition, a BLASTn search without a filter option was used to map ESTs onto the scaffold; the queries were ESTs using the scaffold as the database. Mapped ESTs with the top score for each BLAST result and e-value less than 0.01 were chosen.

#### GeneViewer

The GeneViewer provides an overall profile of the gene models. Display items are: 1) image of nucleotide and spliced nucleotide and results of a domain search in the Pfam database; 2) links to KAIKO2DDB; 3) detailed information on the predicted gene including chromosome number, position of exons and GC content; 4) results of a homology search using BLASTn (top 3 ESTs), BLASTp (top 10 proteins), HMMER and ProfileScan with the alignments of the sequence; 5) results of amino acid analysis in PSORT, SOSUI, MOTIF and Gene ontology (InterProScan and mapping of InterPro entries to GO) with graphical representation of InterProScan and, 6) links to the nucleotide sequence, spliced nucleotide sequence and translated protein sequence of the predicted gene.

#### Software

The software used in KAIKObase was obtained from the public domain and modified to suit specific data. The revised version of GBrowse, originally developed by Stein et al. in 2002 and available in GMOD (Generic Model Organism Database Project) [27], was implemented in KAIKObase using an asynchronous communication written in Javascript. UTGB version 1.0 is a genome browser based on the UTGB (UT Genome Browser) [28] developed by the Morishita Laboratory at the University of Tokyo. The BLAST search engine uses NCBI BLAST [29] version 2.2.17 for sequence search. The database engine uses PostgreSQL [30] version 8.2.1 for keyword and position search. HMMER [31] version 2.1.1, ProfileScan [32] version 2.2, PSORT [33] version 6.4, SOSUI [34] version 1.0, MOTIF [35], and InterProScan [36] version 4.3.1 (data version 14.0) were used in the GeneViewer.

## Utility and discussion

### User interface

**Figure 3:**
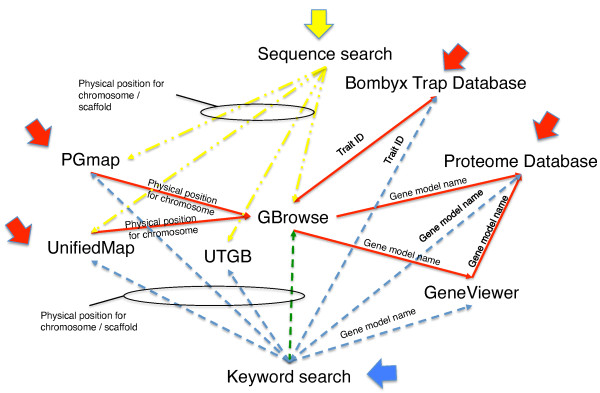
**Links among browsers, viewer, and independent databases**. The large red arrows represent mining from browsers and the pertinent browsers and database. The large blue arrows represent mining from a keyword search and the pertinent browsers and database. The large yellow arrows represent mining from a sequence search and the pertinent browsers and database. The dashed lines represent the flow of information from one database, browser, and viewer to another.

The inter-operability of the databases described above is shown in Figure 3. The red arrows represent the user interface. The user can specify a genomic region using PGmap and UnifiedMap, obtain all related information, and perform data mining through links to GBrowse, UTGB and GeneViewer, thereby providing in-depth information. The user can also specify expression site, intensity, and developmental stage using the *Bombyx *trap database and perform data mining using GBrowse with links to results from inverse PCR. The user can also specify a search to a gene model and related genes to obtain information on the site of expression and developmental stage using the proteome database.

### Keyword and position search

The mining function through a keyword and position search is indicated by the blue arrow in Figure 3. A direct search in KAIKObase for information such as scaffold, contig, FPC-contig, SNP-marker, trait-marker, gene model, EST/cDNA, BAC, BAC-end, fosmid-end, and position on scaffold can be executed. Furthermore, the system provides a search for a specified region on a scaffold or chromosome. Search results are shown as chromosomal images with the retrieved position, and listed with corresponding links to the four browsers, one viewer, and two independent databases.

### Sequence search (BLAST search)

The entry to this class of data mining is shown by the yellow arrow in Figure 3. The query is a nucleic acid or an amino acid sequence; in this way the tool allows finding the location of a scaffold or chromosome. The search result is listed with the data sorted in descending order of bit score with buttons linked to the four browsers. Furthermore, a mechanism was built to show manually entered starting and ending nucleotide positions at the top of the sequence search page.

## Conclusion

We developed KAIKObase to provide effective data mining and efficient utilization of the silkworm genome information for functional and applied genomics. The genomic sequences, map information and EST data are compiled into an integrated silkworm genome database KAIKObase, which consists of 4 map viewers, a gene viewer, sequence search, and keyword and position search systems to display results and data at the level of nucleotide sequence, gene, scaffold and chromosome. In addition, integration of the KAIKO2DDB for proteome data and *Bombyx *trap database for transgene and reporter data further enhance the functionality of KAIKObase. A comprehensive silkworm genome database is indispensable for silkworm research. With introduction of various cutting edge visualization and mining tools, KAIKObase is a powerful data resource and contributes significantly not only to lepidopteran research but also to improve sericulture and to facilitate the development of new pest controls.

## Availability and requirements

Project name: Silkworm Genome Research Program; Project home page: ; Operating system(s): Linux; Programming language: Perl, PHP, JavaScript; Other requirements: Apache, PHP, MySQL, PostgreSQL, GD; License: TBA; Restrictions to use by non-academics: TBA.

## List of abbreviations

BAC: bacterial artificial chromosome; BLAST: Basic Local Alignment Search Tool; CSP: chemosensory protein; EST: expressed sequence tag; FPC: fingerprint contig; GC-content: guanine-cytosine content; GPCR: G protein-coupled receptor; OBP: odorant binding protein; PCR: polymerase chain reaction; SNP: single-nucleotide polymorphism; tRNA: transfer RNA; WGS: whole genome shotgun.

## Authors' contributions

MS was involved in the conception, design, and overall coordination of the database; HM, YS, HO, and CS participated in the programming and implementation of the interfaces, analysis tools, query options etc.; BA, YN, KK-O and JN contributed in the basic design and implementation of various components of the database; HK generated the proteomics data in KAIKO2DDB; HS implemented the *Bombyx *trap database; KY, KM, QX and KK-O were involved in the acquisition and analysis of the genome sequence data; MS, KM, MG, JN and KY contributed in preparing the manuscript. All authors read and approved the final manuscript.

## Supplementary Material

Additional file 1 **Silkworm cDNA libraries and accession number of ESTs derived from each library**. This table provides detailed information on silkworm cDNA libraries such as number of clones, strain, organ/tissue, developmental stage, sex, vector name, cloning site, sequence direction, and the accession number of ESTs in each library. Click here for file

Additional file 2 **Accession number of ESTs from silkworm cDNA libraries**. This table provides a list of clones and accession number for ESTs in the first 38 silkworm libraries described in Additional file 1. Click here for file
