# Supplementary material for: KAIKObase: An integrated silkworm genome database and data mining tool
Source: BMC Genomics. 2009 Oct 21;10:486. doi: 10.1186/1471-2164-10-486 (PMC2770533; doi:10.1186/1471-2164-10-486)
Supplement: Additional file 1 — Silkworm cDNA libraries and accession number of ESTs derived from each library. This table provides detailed information on silkworm cDNA libraries such as number of clones, strain, organ/tissue, developmental stage, sex, vector name, cloning site, sequence direction, and the accession number of ESTs in each library. [file 1471-2164-10-486-S1.PDF]

Additional File 1. Silkworm cDNA libraries and accession number of ESTs derived from each library.

Library ID	Number of clones	Strain/Race	Organ/Tissue	Developmental stage	Sex	Vector	Cloning site	Sequence direction	Accession number
F1mg	2611	F1 (J150 x J203)	Midgut	4th-instar larva day-2	mixed	pBluescript SK-	EcoR1 for 5' Xho1for 3'	sequenced from T3 primer (5' -> 3')	
FJsb	1261	F1-J150	Midgut	4th-instar larva day-2	mixed	pBluescript SK-	EcoR1 for 5' Xho1for 3'	sequenced from T3 primer (5' -> 3')	
JFsb	567	J150-F1	Midgut	4th-instar larva day-3	mixed	pBluescript SK-	EcoR1 for 5' Xho1for 3'	sequenced from T3 primer (5' -> 3')	
n	806	Unknown; BmN cultured cell	Cultured cell (derived from ovary)	Unknown		pBluescript SK-	EcoR1 for 5' Xho1for 3'	sequenced from T3 primer (5' -> 3')	
NV06	313	Unknown; BmN cultured cell	Cultured cell (derived from ovary)	BmNPV infected; 6 hr postinfection		pBluescript SK-	EcoR1 for 5' Xho1for 3'	sequenced from T3 primer (5' -> 3')	
NV12	300	Unknown; BmN cultured cell	Cultured cell (derived from ovary)	BmNPV infected; 12 hr postinfection		pBluescript SK-	EcoR1 for 5' Xho1for 3'	sequenced from T3 primer (5' -> 3')	
Nnor	223	BmN normalized library	Cultured cell (derived from ovary)	Unknown		pBluescript SK-	EcoR1 for 5' Xho1for 3'	sequenced from T3 primer (5' -> 3')	
an--	632	Shuko x Ryuhaku	Antenna	Adult moth		pBluescript SK-	EcoR1 for 5' Xho1for 3'	sequenced from T3 primer (5' -> 3')	
br--	623	p50	Brain	Pupation stege day-0	mixed	pBluescript SK-	EcoR1 for 5' Xho1for 3'	sequenced from T3 primer (5' -> 3')	
brS-	14	p50	Brain	Spinning stage day-0 - day-4	mixed	pBluescript SK-	EcoR1 for 5' Xho1for 3'	sequenced from T3 primer (5' -> 5')	
ceN-	2106	same as ce-- (diff. preparation)	Compound eyes	5th-instar larva - Pupation stage	mixed	pBluescript SK-	EcoR1 for 5' Xho1for 3'	sequenced from T3 primer (5' -> 3')	
e100	7839	p50T	Embryo	100hr after oviposition	mixed	pGCAPI, G-capping		from 5' with M(-21)	
e4	728	p50	Diapause-destined embryo	40 hr after oviposition	mixed	pBluescript SK-	EcoR1 for 5' Xho1for 3'	sequenced from T3 primer (5' -> 3')	
e96h	661	p50	Diapause-cancelled embryo	96 hr after oviposition	mixed	pBluescript SK-	EcoR1 for 5' Xho1for 3'	sequenced from T3 primer (5' -> 3')	
epV3	815	p50	Epidermis	5th-instar larva day-3	mixed	pBluescript SK-	EcoR1 for 5' Xho1for 3'	sequenced from T3 primer (5' -> 3')	
fbS2	425	C202 x J201	Fat body	Spinning stage day-2	male	pBluescript SK-	EcoR1 for 5' Xho1for 3'	sequenced from T3 primer (5' -> 3')	
fbf	213	p50	Fat body	5th-instar larva day-3	female	cloned into pUC18 Smal	Sma!-Blunt-end	sequenced from M4 primer (direction unknown)	
fbm	348	p50	Fat body	5th-instar larva day-3	male	cloned into pUC18 Smal	Sma!-Blunt-end	sequenced from M4 primer (direction unknown)	
fbpv	622	Shuko x Ryuhaku	Fat body	Spinning stage day-2, BmNPV infected 2hr	mixed	pBluescript SK-	EcoR1 for 5' Xho1for 3'	sequenced from T3 primer (5' -> 3')	
fcP8	3357	p50T	Follicle cells	Pupation stege day-8	female	pBluescript SK-	EcoR1 for 5' Xho1for 3'	sequenced from T3 primer (5' -> 3')	Please refer to additional file 2.
heS0	718	C108	Hemocyte	Spinning stage day-0	mixed	pBluescript SK-	EcoR1 for 5' Xho1for 3'	sequenced from T3 primer (5' -> 3')	
heS3	557	C108	Hemocyte	Spinning stage day-3	mixed	pBluescript SK-	EcoR1 for 5' Xho1for 3'	sequenced from T3 primer (5' -> 3')	
maV3	775	p50	Malpighian tubule	5th-instar larva day-3	mixed	pBluescript SK-	EcoR1 for 5' Xho1for 3'	sequenced from T3 primer (5' -> 3')	
mg	778	p50	Midgut	5th-instar larva day-3	mixed	pBluescript SK-	EcoR1 for 5' Xho1for 3'	sequenced from T3 primer (5' -> 3')	
ovS0	4727	p50T	Ovary	Spinning stage day-0	female	pBluescript SK-	EcoR1 for 5' Xho1for 3'	sequenced from T3 primer (5' -> 3')	
ovS3	4860	p50	Ovary	Spinning stage day-3	female	pBluescript SK-	EcoR1 for 5' Xho1for 3'	sequenced from T3 primer (5' -> 3')	
pg--	446	Shuko x Ryuhaku	Pheromone gland	Newly-eclosed adult	female	pBluescript SK-	EcoR1 for 5' Xho1for 3'	sequenced from T3 primer (5' -> 3')	
prW-	2791	p50T	Prothoracic gland	Spinning stage	mixed	pGCAPI, G-capping		sequenced from 5' with T7 primer	
prgv	842	p50	Prothoracic gland	5th-instar larva day-4	mixed	pBluescript SK-	EcoR1 for 5' Xho1for 3'	sequenced from T3 primer (5' -> 3')	
vg4M	4618	p50T	Verson's gland	4th molting stage day-2	male	pBluescript SK-	EcoR1 for 5' Xho1for 3'	sequenced from T3 primer (5' -> 3')	
ws0	864	C108	Wing disc	Spinning stage day-0	mixed	pBluescript SK-	EcoR1 for 5' Xho1for 3'	sequenced from T3 primer (5' -> 3')	
ws2	814	C108	Wing disc	Spinning stage day-2	mixed	pBluescript SK-	EcoR1 for 5' Xho1for 3'	sequenced from T3 primer (5' -> 3')	
wdV1	281	C108	Wing disc	5th-instar larva day-1	mixed	pBluescript SK-	EcoR1 for 5' Xho1for 3'	sequenced from T3 primer (5' -> 3')	
wdV3	672	C108	Wing disc	5th-instar larva day-3	mixed	pBluescript SK-	EcoR1 for 5' Xho1for 3'	sequenced from T3 primer (5' -> 3')	
wv4	823	C108	Wing disc	5th-instar larva day-4	mixed	pBluescript SK-	EcoR1 for 5' Xho1for 3'	sequenced from T3 primer (5' -> 3')	
MSV3	4361	p50T	Middle silkgland	5th-instar larva day-3	mixed	pGADT7		sequenced from 5' with T7, from 3' with RV	
mxg-	3724	p50T	Maxillary galea	5th-instar larva day-3	mixed	pGCAPI, G-capping		sequenced from 5' with T7 primer	
psgV	4740	p50T	Posterior silkgland	5th-instar larva day-3	mixed	pGADT7		sequenced from 5' with T7, from 3' with RV	
BmN-	6391	Unknown; BmN cultured cell	Cultured cell (derived from ovary)	Undefined		Oligo-cap		sequenced from 5'	
swa	9479	P50	silk gland	5th-instar larva day-3	mixed	pBluescript II SK+	EcoR1 for 5' Xho1for 3'	sequenced from T7 primer (5' -> 3')	CK484630-CK494108
swb	7255	P50	midgut	5th-instar larva day-3	mixed	pBluescript II SK+	EcoR1 for 5' Xho1for 3'	sequenced from T7 primer (5' -> 3')	CK494109-CK501363
swc	6105	P50	Fat body	5th-instar larva day-3	female	pBluescript II SK+	EcoR1 for 5' Xho1for 3'	sequenced from T7 primer (5' -> 3')	CK501364-CK507468
swd	6532	P50	Fat body	5th-instar larva day-3	male	pBluescript II SK+	EcoR1 for 5' Xho1for 3'	sequenced from T7 primer (5' -> 3')	CK507469-CK514000
swj	4490	P50	testis	Embryo (72 hours)	mixed	pBluescript II SK+	EcoR1 for 5' Xho1for 3'	sequenced from T7 primer (5' -> 3')	CK514001-CK518490
swe	6424	P50	Hemocyte	5th-instar larva day-3	female	pBluescript II SK+	EcoR1 for 5' Xho1for 3'	sequenced from T7 primer (5' -> 3')	CK518491-CK524914
swf	4993	P50	Hemocyte	5th-instar larva day-3	male	pBluescript II SK+	EcoR1 for 5' Xho1for 3'	sequenced from T7 primer (5' -> 3')	CK524915-CK529907
swg	8407	P50	testis	5th-instar larva day-3	male	pBluescript II SK+	EcoR1 for 5' Xho1for 3'	sequenced from T7 primer (5' -> 3')	CK529908-CK538314
swh	8330	P50	ovary	5th-instar larva day-3	female	pBluescript II SK+	EcoR1 for 5' Xho1for 3'	sequenced from T7 primer (5' -> 3')	CK538315-CK546644
swk	4527	P50		Embryo (nondiapause)	mixed	pBluescript II SK+	EcoR1 for 5' Xho1for 3'	sequenced from T7 primer (5' -> 3')	CK546645-CK551171
swl	6179	P50	Fat body (pupa)	P0	mixed	pBluescript II SK+	EcoR1 for 5' Xho1for 3'	sequenced from T7 primer (5' -> 3')	CK551172-CK557350
swp	7754	P50		Embryo (unfertilized)	mixed	pBluescript II SK+	EcoR1 for 5' Xho1for 3'	sequenced from T7 primer (5' -> 3')	CK557351-CK565104
BmP	1003	Qingsong-Haoyue	whole pupae body excluding skin	metaphase	mixed	pHelix	Site_1 Hind II	M13 Forward	DN236876-DN237878
