# Supplementary material for: KAIKObase: An integrated silkworm genome database and data mining tool
Source: BMC Genomics. 2009 Oct 21;10:486. doi: 10.1186/1471-2164-10-486 (PMC2770533; doi:10.1186/1471-2164-10-486)
Supplement: Additional file 2 — Accession number of ESTs from silkworm cDNA libraries. This table provides a list of clones and accession number for ESTs in the first 38 silkworm libraries described in Additional file 1. [file 1471-2164-10-486-S2.PDF]

Additional File 2. Accession number of ESTs from silkworm cDNA libraries.

Library ID	Clone name	Accession number
br--	br--1002	AV400237
	br--1003	AV400238
	br--1004	AV400239
	br--1005	AV400240
	br--1006	AV400241
	br--1007	AV400242
	br--1009	AV400243
	br--1011	AV400245
	br--1012X	AV400246
	br--1013	AV400247
	br--1014_1	BY914634
	br--1016	AV400250
	br--1017	AV400251
	br--1020	AV400253
	br--1021	AV400254
	br--1023	AV400256
	br--1024X	AV400257
	br--1026	AV400259
	br--1027	AV400260
	br--1028_1	BY914635
	br--1030	AV400262
	br--1032_1	BY914636
	br--1033	AV400265
	br--1034	AV400266
	br--1035	AV400267
	br--1036	AV400268
	br--1037	AV400269
	br--1038X	AV400270
	br--1039X	AV400271
	br--1040X	AV400272
	br--1041_1	BY914637
	br--1042	AV400274
	br--1043X	AV400275
	br--1044X	AV400277
	br--1046X	AV400280
	br--1047	AV400281
	br--1048	AV400282
	br--1049X	AV400283
	br--1050X	AV400284
	br--1053	AV400287
	br--1054X	AV400288
	br--1055	AV400289
	br--1056	AV400290
	br--1060	AV400292
	br--1061	AV400293
	br--1062	AV400294
	br--1063	AV400295
	br--1064	AV400296
	br--1065	AV400297
	br--1066	AV400298
	br--1067	AV400299

br--1068	AV400300
br--1069	AV400301
br--1070	AV400302
br--1071	AV400303
br--1072	AV400304
br--1073X	AV400305
br--1074	AV400306
br--1075	AV400307
br--1076	AV400308
br--1077	AV400309
br--1078	AV400310
br--1079	AV400311
br--1082	AV400313
br--1084	AV400314
br--1086	AV400315
br--1087	AV400316
br--1088	AV400317
br--1089	AV400318
br--1090	AV400319
br--1091	AV400320
br--1092	AV400321
br--1094	AV400322
br--1096	AV400324
br--1097	AV400325
br--1098	AV400326
br--1100X	AV400328
br--1101X	AV400329
br--1102	AV400330
br--1103	AV400331
br--1104	BY914638
br--1105	AV400332
br--1106	AV400333
br--1107X	AV400334
br--1109	AV400336
br--1110	AV400337
br--1111	AV400338
br--1112	AV400339
br--1113	AV400340
br--1114	AV400341
br--1115	AV400342
br--1117	AV400344
br--1118	AV400345
br--1119	AV400346
br--1120	AV400347
br--1121	AV400348
br--1122	AV400349
br--1124	AV400351
br--1125	AV400352
br--1126	AV400353
br--1127	AV400354
br--1128	AV400355
br--1130	AV400357
br--1132	AV400359
br--1134	AV400361

br--1135	AV400362
br--1138	AV400365
br--1139X	AV400366
br--1143	AV400370
br--1146	AV400372
br--1148	AV400373
br--1150	AV400374
br--1151	AV400375
br--1152	AV400376
br--1153	AV400377
br--1154X	AV400378
br--1155	AV400379
br--1156X	AV400380
br--1157	AV400381
br--1158	AV400382
br--1160	AV400383
br--1161X	AV400384
br--1162	AV400385
br--1163	AV400386
br--1164	AV400387
br--1167	AV400388
br--1168	AV400389
br--1169	AV400390
br--1170X	AV400391
br--1172X	AV400393
br--1173	AV400394
br--1174	AV400395
br--1175	AV400396
br--1177	AV400397
br--1178	AV400398
br--1179	AV400399
br--1180	AV400400
br--1181X	AV400401
br--1182X	AV400402
br--1184	AV400404
br--1185	AV400405
br--1187	AV400406
br--1188	AV400407
br--1190	AV400408
br--1191	AV400409
br--1192	AV400410
br--1194X	AV400412
br--1195	AV400413
br--1198X	AV400414
br--1200	AV400415
br--1201	AV400416
br--1204X	AV400417
br--1207	AV400418
br--1208	AV400419
br--1213	AV400420
br--1214	AV400421
br--1215	AV400422
br--1216	AV400423
br--1217X	AV400424

br--1222X	AV400427
br--1223	AV400428
br--1224	AV400429
br--1225X	AV400430
br--1226	AV400431
br--1227	AV400432
br--1228	AV400433
br--1230	AV400434
br--1231	AV400435
br--1236	AV400436
br--1243	AV400439
br--1244X	AV400440
br--1246	AV400441
br--1247	AV400442
br--1248	AV400443
br--1249	AV400444
br--1250	AV400445
br--1252	AV400446
br--1253	AV400447
br--1254	AV400448
br--1256	AV400449
br--1258	AV400450
br--1260	AV400451
br--1261	AV400452
br--1262	AV400453
br--1264	AV400454
br--1265	AV400455
br--1266	AV400456
br--1267	AV400457
br--1269	AV400458
br--1273	AV400459
br--1274	AV400460
br--1276X	AV400461
br--1280	AV400463
br--1281X	AV400464
br--1282	AV400465
br--1283	AV400466
br--1284	AV400467
br--1290X	AV400470
br--1291	AV400471
br--1294	AV400472
br--1295	AV400473
br--1298	AV400474
br--1303	AV400476
br--1304	AV400477
br--1306	AV400478
br--1307	AV400479
br--1308X	AV400480
br--1314	AV400481
br--1315	AV400482
br--1316	AV400483
br--1327X	AV400484
br--1328	AV400485
br--1329	AV400486

br--1332	AV400487
br--1333	AV400488
br--1338X	AV400489
br--1340	AV400490
br--1343	AV400492
br--1345	AV400494
br--1346	AV400495
br--1347X	AV400496
br--1350	AV400499
br--1351	AV400500
br--1352	AV400501
br--1354	AV400502
br--1356	AV400503
br--1357	AV400504
br--1358	AV400505
br--1359	AV400506
br--1360	AV400507
br--1362	AV400509
br--1363	AV400510
br--1364	AV400511
br--1367	AV400512
br--1369	AV400513
br--1372	AV400514
br--1375	AV400516
br--1376	AV400517
br--1378	AV400518
br--1381X	AV400519
br--1389X	AV400520
br--1391	AV400521
br--1392	AV400522
br--1393	AV400523
br--1394	AV400524
br--1397	AV400525
br--1401	AV400526
br--1402	AV400527
br--1405X	AV400528
br--1406	AV400529
br--1407	AV400530
br--1408	AV400531
br--1409	AV400532
br--1411	AV400534
br--1412	AV400535
br--1413	AV400536
br--1414	AV400537
br--1416	AV400539
br--1418X	AV400540
br--1420	AV400541
br--1421	AV400542
br--1427	AV400543
br--1428	AV400544
br--1429	AV400545
br--1430	AV400546
br--1432	AV400547
br--1433X	AV400548

br--1434	AV400549
br--1435	AV400550
br--1436	AV400551
br--1440	AV400552
br--1441	AV400553
br--1443	AV400554
br--1444	AV400555
br--1445	AV400556
br--1447	AV400557
br--1448	AV400558
br--1449	AV400559
br--1450	AV400560
br--1451	AV400561
br--1452X	AV400562
br--1453	AV400563
br--1454	AV400564
br--1455	AV400565
br--1456	AV400566
br--1457	AV400567
br--1459	AV400568
br--1460	AV400569
br--1461	AV400570
br--1462	AV400571
br--1464	AV400572
br--1465	AV400573
br--1466	AV400574
br--1468	AV400575
br--1471	AV400577
br--1472	AV400578
br--1473	AV400579
br--1474	AV400580
br--1475	AV400581
br--1476	AV400582
br--1477	AV400583
br--1478X	AV400584
br--1482	AV400585
br--1485	AV400586
br--1486	AV400587
br--1488	AV400588
br--1491	AV400589
br--1493	AV400590
br--1494X	AV400591
br--1495	AV400592
br--1497	AV400593
br--1498	AV400594
br--1502	AV400595
br--1503	AV400596
br--1508	AV400598
br--1510	AV400599
br--1512	AV400600
br--1513	AV400601
br--1514	AV400602
br--1516	AV400603
br--1517	AV400604

br--1518	AV400605
br--1519	AV400606
br--1520	AV400607
br--1524	AV400608
br--1530	AV400610
br--1532	AV400611
br--1533	AV400612
br--1534	AV400613
br--1535X	AV400614
br--1538	AV400615
br--1540	AV400616
br--1542	AV400617
br--1543	AV400618
br--1545	AV400619
br--1548	AV400620
br--1550X	AV400621
br--1555	AV400622
br--1556X	AV400623
br--1558X	AV400624
br--1559	AV400625
br--1560	AV400626
br--1561	AV400627
br--1563	AV400628
br--1564	AV400629
br--1567X	AV400630
br--1569	AV400631
br--1572	AV400632
br--1573	AV400633
br--1574	AV400634
br--1575	AV400635
br--1576	AV400636
br--1577	AV400637
br--1581X	AV400638
br--1583	AV400639
br--1584	AV400640
br--1586X	AV400641
br--1587	AV400642
br--1593X	AV400643
br--1597	AV400644
br--1601	AV400645
br--1602	AV400646
br--1603	AV400647
br--1604	AV400648
br--1605	AV400649
br--1607	AV400651
br--1608	AV400652
br--1609	AV400653
br--1613	AV400655
br--1614	AV400656
br--1615X	AV400657
br--1616	AV400658
br--1617	AV400659
br--1618	AV400660
br--1620	AV400661

br--1621	AV400662
br--1624	AV400664
br--1625	AV400665
br--1627	AV400667
br--1628	AV400668
br--1629	AV400669
br--1630	AV400670
br--1632	AV400671
br--1634	AV400672
br--1635	AV400673
br--1636	AV400674
br--1637	AV400675
br--1638	AV400676
br--1639X	AV400677
br--1640	AV400678
br--1641	AV400679
br--1642	AV400680
br--1643	AV400681
br--1644	AV400682
br--1646	AV400683
br--1647	AV400684
br--1648	AV400685
br--1649	AV400686
br--1650	AV400687
br--1651	AV400688
br--1652	AV400689
br--1653	AV400690
br--1654	AV400691
br--1656	AV400692
br--1658	AV400693
br--1661	AV400694
br--1662	AV400695
br--1663	AV400696
br--1664	AV400697
br--1665	AV400698
br--1666	AV400699
br--1667	AV400700
br--1668	AV400701
br--1669X	AV400702
br--1670	AV400703
br--1671X	AV400704
br--1672X	AV400705
br--1674X	AV400707
br--1677X	AV400709
br--1678X	AV400710
br--1679	AV400711
br--1684X	AV400712
br--1685	AV400713
br--1687	AV400714
br--1688	AV400715
br--1691	AV400716
br--1693X	AV400717
br--1699	AV400718
br--1700X	AV400719

br--1701	AV400720
br--1703	AV400722
br--1704	AV400723
br--1705	AV400724
br--1706	AV400725
br--1707	AV400726
br--1708X	AV400727
br--1710X	AV400728
br--1711	AV400729
br--1712X	AV400730
br--1713	AV400731
br--1714	AV400732
br--1715	AV400733
br--1717	AV400734
br--1718	AV400735
br--1719	AV400736
br--1723	AV400738
br--1725	AV400739
br--1726	AV400740
br--1728X	AV400741
br--1729	AV400742
br--1730	AV400743
br--1731	AV400744
br--1732	AV400745
br--1733	AV400746
br--1737X	AV400747
br--1738	AV400748
br--1740	AV400749
br--1742	AV400750
br--1745	AV400751
br--1747	AV400753
br--1748	AV400754
br--1749	AV400755
br--1750	AV400756
br--1751	AV400757
br--1756X	AV400759
br--1758	AV400760
br--1759	AV400761
br--1760	AV400762
br--1761	AV400763
br--1763	AV400765
br--1766	AV400766
br--1767	AV400767
br--1769	AV400768
br--1771	AV400769
br--1774X	AV400770
br--1780X	AV400772
br--1781	AV400773
br--1782X	AV400774
br--1783	AV400775
br--1784X	AV400776
br--1788X	AV400778
br--1789	AV400779
br--1791X	AV400780

br--1792	AV400781
br--1794	AV400782
br--1795	AV400783
br--1796	AV400784
br--1799	AV400785
br--1800	AV400786
br--1801	AV400787
br--1803	AV400788
br--1805X	AV400789
br--1811	AV400792
br--1812	AV400793
br--1813	AV400794
br--1814	AV400795
br--1815X	AV400796
br--1816	AV400797
br--1818	AV400799
br--1820	AV400801
br--1821	AV400802
br--1822	AV400803
br--1823X	AV400804
br--1824	AV400805
br--1825	AV400806
br--1826	AV400807
br--1827	AV400808
br--1828	AV400809
br--1829	AV400810
br--1831	AV400811
br--1833	AV400812
br--1834	AV400813
br--1835	AV400814
br--1836	AV400815
br--1838	AV400816
br--1839	AV400817
br--1840	AV400818
br--1841	AV400819
br--1842	AV400820
br--1844X	AV400822
br--1845	AV400823
br--1846	AV400824
br--1847	AV400825
br--1848	AV400826
br--1849X	AV400827
br--1850	AV400828
br--1851	AV400829
br--1853	AV400830
br--1855	AV400831
br--1856	AV400832
br--1857	AV400833
br--1858X	AV400834
br--1859X	AV400835
br--1860	AV400836
br--1861	AV400837
br--1863	AV400838
br--1864X	AV400839

br--1865	AV400840
br--1866	AV400841
br--1868X	AV400842
br--1869	AV400843
br--1870	AV400844
br--1871	AV400845
br--1872	AV400846
br--1873	AV400847
br--1874	AV400848
br--1877	AV400850
br--1879	AV400851
br--1880	AV400852
br--1881	AV400853
br--1882	AV400854
br--1885	AV400855
br--1886	AV400856
br--1887	AV400857
br--1890	AV400858
br--1891	AV400859
br--1892	AV400860
br--1899	AV400861
br--1901	AV400862
br--1902	AV400863
br--1904	AV400864
br--1905	AV400865
br--1907	AV400866
br--1908	AV400867
br--1910	AV400869
br--1912	AV400870
br--1915	AV400872
br--1916	AV400873
br--1918	AV400874
br--1919	AV400875
br--1920	AV400876
br--1922	AV400877
br--1923	AV400878
br--1924	AV400879
br--1925	AV400880
br--1926	AV400881
br--1929	AV400883
br--1931	AV400885
br--1932	AV400886
br--1933	AV400887
br--1934	AV400888
br--1935	AV400889
br--1936	AV400890
br--1937	AV400891
br--1939	AV400892
br--1940	AV400893
br--1941	AV400894
br--1942	AV400895
br--1943	AV400896
br--1948	AV400897
br--1949X	AV400898

	br--1950	AV400899
	br--1951	AV400900
	br--1954	AV400902
	br--1955	AV400903
	br--1959	AV400904
	br--1961	AV400905
	br--1962	AV400906
	br--1963	AV400907
	br--1964	AV400908
	br--1966	AV400910
	br--1967	AV400911
	br--1969X	AV400912
	br--1970	AV400913
	br--1973	AV400914
	br--1974	AV400915
	br--1975	AV400916
	br--1979	AV400917
	br--1980	AV400918
	br--1982	AV400920
	br--1983	AV400921
	br--1984	AV400922
	br--1985	AV400923
	br--1988	AV400924
	br--1989	AV400925
	br--1990	AV400926
	br--1993	AV400928
	br--1994	AV400929
	br--1995	AV400930
	br--1996	AV400931
	br--1997X	AV400932
	br--1998X	AV400933
	br--1999X	AV400934
brS-	brS-2120	BY914639
	brS-2130	BY914640
	brS-2144	BY914641
	brS-2149	BY914642
	brS-2164	BY914643
	brS-2165	BY914644
	brS-2168	BY914645
	brS-2173	BY914646
	brS-2176	BY914647
	brS-2178	BY914648
	brS-2185	BY914649
	brS-2187	BY914650
	brS-2190	BY914651
	brS-2197	BY914652
ceN-	ceN-0001	BP118783
	ceN-0003	BP118784
	ceN-0004	BP118785
	ceN-0005	BP118786
	ceN-0006	BP118787
	ceN-0009	BP118789
	ceN-0010	BP118790
	ceN-0012	BP118791

ceN-0013	BP118792
ceN-0015	BP118793
ceN-0017	BP118794
ceN-0021	BP118795
ceN-0022	BP118796
ceN-0024	BP118797
ceN-0025	BP118798
ceN-0026	BP118799
ceN-0027	BP118800
ceN-0028	BP118801
ceN-0029	BP118802
ceN-0030	BP118803
ceN-0032	BP118804
ceN-0033	BP118805
ceN-0034	BP118806
ceN-0035	BP118807
ceN-0037	BP118808
ceN-0038	BP118809
ceN-0039	BP118810
ceN-0044	BP118812
ceN-0045	BP118813
ceN-0048	BP118814
ceN-0049	BP118815
ceN-0050	BP118816
ceN-0053	BP118817
ceN-0054	BP118818
ceN-0056	BP118819
ceN-0058	BP118821
ceN-0059	BP118822
ceN-0060	BP118823
ceN-0061	BP118824
ceN-0062	BP118825
ceN-0063	BP118826
ceN-0064	BP118827
ceN-0066	BP118828
ceN-0070	BP118829
ceN-0071	BP118830
ceN-0072	BP118831
ceN-0073	BP118832
ceN-0075	BP118833
ceN-0077	BP118834
ceN-0079	BP118835
ceN-0080	BP118836
ceN-0081	BP118837
ceN-0084	BP118838
ceN-0086	BP118840
ceN-0089	BP118841
ceN-0090	BP118842
ceN-0091	BP118843
ceN-0092	BP118844
ceN-0094	BY914653
ceN-0097	BP118845
ceN-0098	BP118846
ceN-0100	BP118847

ceN-0102	BP118848
ceN-0103	BP118849
ceN-0105	BP118851
ceN-0106	BP118852
ceN-0107	BP118853
ceN-0108	BP118854
ceN-0110	BP118856
ceN-0112	BP118857
ceN-0113	BP118858
ceN-0114	BP118859
ceN-0115	BP118860
ceN-0116	BP118861
ceN-0118	BP118862
ceN-0119	BP118863
ceN-0120	BP118864
ceN-0121	BP118865
ceN-0123	BP118867
ceN-0126	BP118868
ceN-0127	BP118869
ceN-0128	BP118870
ceN-0129	BP118871
ceN-0131	BP118873
ceN-0132	BP118874
ceN-0133	BP118875
ceN-0134	BP118876
ceN-0136	BP118877
ceN-0137	BP118878
ceN-0141	BP118879
ceN-0144	BP118880
ceN-0146	BP118882
ceN-0147	BP118883
ceN-0148	BP118884
ceN-0149	BP118885
ceN-0150	BP118886
ceN-0151	BP118887
ceN-0152	BP118888
ceN-0153	BP118889
ceN-0155	BP118891
ceN-0156	BP118892
ceN-0157	BP118893
ceN-0160	BP118896
ceN-0161	BP118897
ceN-0162	BP118898
ceN-0163	BP118899
ceN-0164	BP118900
ceN-0175	BP118903
ceN-0176	BP118904
ceN-0177	BP118905
ceN-0178	BP118906
ceN-0181	BP118907
ceN-0182	BP118908
ceN-0183	BP118909
ceN-0185	BP118911
ceN-0186	BP118912

ceN-0187	BP118913
ceN-0188	BP118914
ceN-0196	BP118918
ceN-0200	BP118920
ceN-0205	BP118921
ceN-0206	BP118922
ceN-0211	BP118923
ceN-0222	BP118925
ceN-0226	BP118926
ceN-0228	BP118927
ceN-0230	BP118928
ceN-0232	BP118929
ceN-0233	BP118930
ceN-0235	BP118931
ceN-0236	BP118932
ceN-0238	BP118933
ceN-0239	BP118934
ceN-0243	BP118936
ceN-0244	BP118937
ceN-0249	BP118940
ceN-0255	BP118943
ceN-0256	BP118944
ceN-0258	BP118945
ceN-0259	BY914654
ceN-0270	BP118949
ceN-0274	BP118951
ceN-0283	BP118955
ceN-0285	BP118957
ceN-0290	BP118960
ceN-0291	BP118961
ceN-0292	BP118962
ceN-0293	BP118963
ceN-0294	BP118964
ceN-0295	BP118965
ceN-0298	BP118968
ceN-0299	BP118969
ceN-0300	BP118970
ceN-0301	BP118971
ceN-0302	BP118972
ceN-0304	BP118973
ceN-0305	BP118974
ceN-0306	BP118975
ceN-0307	BP118976
ceN-0308	BP118977
ceN-0309	BP118978
ceN-0310	BP118979
ceN-0312	BP118981
ceN-0313	BP118982
ceN-0314	BP118983
ceN-0317	BP118984
ceN-0318	BP118985
ceN-0322	BP118987
ceN-0323	BP118988
ceN-0326	BP118990

ceN-0330	BP118992
ceN-0331	BP118993
ceN-0332	BP118994
ceN-0335	BP118995
ceN-0336	BP118996
ceN-0338	BP118998
ceN-0342	BP119000
ceN-0344	BP119002
ceN-0345	BP119003
ceN-0348	BP119004
ceN-0349	BP119005
ceN-0350	BP119006
ceN-0353	BP119007
ceN-0354	BP119008
ceN-0355	BP119009
ceN-0357	BP119011
ceN-0359	BP119012
ceN-0360	BP119013
ceN-0362	BP119015
ceN-0363	BP119016
ceN-0364	BP119017
ceN-0365	BP119018
ceN-0366	BP119019
ceN-0367	BP119020
ceN-0368	BP119021
ceN-0369	BP119022
ceN-0371	BP119023
ceN-0372	BP119024
ceN-0373	BP119025
ceN-0374	BP119026
ceN-0375	BP119027
ceN-0376	BP119028
ceN-0377	BP119029
ceN-0378	BP119030
ceN-0379	BP119031
ceN-0380	BP119032
ceN-0381	BP119033
ceN-0382	BP119034
ceN-0383	BP119035
ceN-0386	BP119038
ceN-0388	BP119040
ceN-0389	BP119041
ceN-0392	BP119043
ceN-0393	BP119044
ceN-0394	BP119045
ceN-0395	BP119046
ceN-0397	BP119047
ceN-0398	BP119048
ceN-0399	BP119049
ceN-0400	BP119050
ceN-0401	BP119051
ceN-0402	BP119052
ceN-0404	BP119054
ceN-0405	BP119055

ceN-0406	BP119056
ceN-0410	BP119059
ceN-0411	BP119060
ceN-0412	BP119061
ceN-0415	BP119063
ceN-0416	BP119064
ceN-0418	BP119065
ceN-0419	BP119066
ceN-0421	BP119067
ceN-0422	BP119068
ceN-0424	BP119069
ceN-0425	BP119070
ceN-0431	BP119071
ceN-0432	BP119072
ceN-0434	BP119073
ceN-0435	BP119074
ceN-0437	BP119075
ceN-0438	BP119076
ceN-0439	BP119077
ceN-0440	BP119078
ceN-0441	BP119079
ceN-0442	BP119080
ceN-0443	BP119081
ceN-0445	BP119083
ceN-0447	BP119085
ceN-0448	BP119086
ceN-0449	BP119087
ceN-0450	BP119088
ceN-0451	BP119089
ceN-0452	BP119090
ceN-0453	BP119091
ceN-0454	BP119092
ceN-0456	BP119094
ceN-0458	BP119096
ceN-0459	BP119097
ceN-0460	BP119098
ceN-0464	BP119100
ceN-0466	BP119101
ceN-0469	BP119102
ceN-0470	BP119103
ceN-0471	BP119104
ceN-0473	BP119105
ceN-0475	BP119106
ceN-0478	BP119107
ceN-0481	BP119108
ceN-0482	BP119109
ceN-0484	BP119111
ceN-0485	BP119112
ceN-0486	BP119113
ceN-0487	BP119114
ceN-0489	BP119116
ceN-0490	BP119117
ceN-0491	BP119118
ceN-0494	BP119120

ceN-0495	BP119121
ceN-0496	BP119122
ceN-0497	BP119123
ceN-0499	BP119124
ceN-0500	BP119125
ceN-0501	BP119126
ceN-0502	BP119127
ceN-0503	BP119128
ceN-0504	BP119129
ceN-0505	BP119130
ceN-0506	BP119131
ceN-0507	BP119132
ceN-0508	BP119133
ceN-0509	BP119134
ceN-0510	BP119135
ceN-0511	BP119136
ceN-0512	BP119137
ceN-0513	BP119138
ceN-0515	BP119139
ceN-0517	BP119140
ceN-0518	BP119141
ceN-0520	BP119142
ceN-0523	BP119143
ceN-0524	BP119144
ceN-0527	BP119146
ceN-0528	BP119147
ceN-0529	BP119148
ceN-0531	BP119150
ceN-0535	BP119152
ceN-0536	BP119153
ceN-0537	BP119154
ceN-0538	BP119155
ceN-0539	BP119156
ceN-0540	BP119157
ceN-0541	BP119158
ceN-0542	BP119159
ceN-0543	BP119160
ceN-0544	BP119161
ceN-0545	BP119162
ceN-0546	BP119163
ceN-0547	BP119164
ceN-0548	BP119165
ceN-0549	BP119166
ceN-0550	BP119167
ceN-0551	BP119168
ceN-0553	BP119169
ceN-0554	BP119170
ceN-0555	BP119171
ceN-0556	BP119172
ceN-0557	BP119173
ceN-0560	BP119175
ceN-0561	BP119176
ceN-0563	BP119178
ceN-0564	BP119179

ceN-0565	BP119180
ceN-0566	BP119181
ceN-0568	BP119183
ceN-0572	BP119184
ceN-0573	BP119185
ceN-0575	BP119186
ceN-0576	BP119187
ceN-0578	BP119188
ceN-0579	BP119189
ceN-0581	BP119191
ceN-0583	BP119193
ceN-0584	BP119194
ceN-0585	BP119195
ceN-0587	BP119196
ceN-0588	BP119197
ceN-0590	BP119198
ceN-0591	BP119199
ceN-0592	BP119200
ceN-0593	BP119201
ceN-0595	BP119203
ceN-0596	BP119204
ceN-0597	BP119205
ceN-0599	BP119206
ceN-0600	BP119207
ceN-0602	BP119208
ceN-0604	BP119209
ceN-0605	BP119210
ceN-0607	BP119211
ceN-0608	BP119212
ceN-0609	BP119213
ceN-0610	BP119214
ceN-0611	BP119215
ceN-0612	BP119216
ceN-0615	BP119217
ceN-0618	BP119219
ceN-0620	BP119221
ceN-0621	BP119222
ceN-0623	BP119223
ceN-0624	BP119224
ceN-0628	BP119226
ceN-0629	BP119227
ceN-0631	BP119229
ceN-0632	BP119230
ceN-0633	BP119231
ceN-0634	BP119232
ceN-0635	BP119233
ceN-0636	BP119234
ceN-0637	BP119235
ceN-0638	BP119236
ceN-0640	BP119237
ceN-0641	BP119238
ceN-0643	BP119239
ceN-0644	BP119240
ceN-0645	BP119241

ceN-0646	BP119242
ceN-0647	BP119243
ceN-0652	BP119247
ceN-0653	BP119248
ceN-0654	BP119249
ceN-0656	BP119250
ceN-0657	BP119251
ceN-0658	BP119252
ceN-0659	BP119253
ceN-0660	BP119254
ceN-0662	BP119256
ceN-0663	BP119257
ceN-0666	BP119258
ceN-0667	BP119259
ceN-0668	BP119260
ceN-0669	BP119261
ceN-0672	BP119263
ceN-0673	BP119264
ceN-0674	BP119265
ceN-0675	BP119266
ceN-0676	BP119267
ceN-0677	BP119268
ceN-0679	BP119269
ceN-0680	BP119270
ceN-0683	BP119271
ceN-0684	BP119272
ceN-0685	BP119273
ceN-0686	BP119274
ceN-0688	BP119276
ceN-0690	BP119277
ceN-0691	BP119278
ceN-0692	BP119279
ceN-0693	BP119280
ceN-0694	BP119281
ceN-0695	BP119282
ceN-0698	BP119284
ceN-0699	BP119285
ceN-0700	BP119286
ceN-0701	BP119287
ceN-0702	BP119288
ceN-0703	BP119289
ceN-0704	BP119290
ceN-0705	BP119291
ceN-0706	BP119292
ceN-0707	BP119293
ceN-0709	BP119294
ceN-0710	BP119295
ceN-0711	BP119296
ceN-0713	BP119297
ceN-0716	BP119299
ceN-0718	BP119300
ceN-0719	BP119301
ceN-0720	BP119302
ceN-0721	BP119303

ceN-0723	BP119304
ceN-0724	BP119305
ceN-0725	BP119306
ceN-0727	BP119307
ceN-0728	BP119308
ceN-0730	BP119310
ceN-0731	BP119311
ceN-0734	BP119313
ceN-0735	BP119314
ceN-0737	BP119315
ceN-0738	BP119316
ceN-0739	BP119317
ceN-0740	BP119318
ceN-0741	BP119319
ceN-0744	BP119321
ceN-0746	BP119322
ceN-0747	BP119323
ceN-0748	BP119324
ceN-0749	BP119325
ceN-0750	BP119326
ceN-0751	BP119327
ceN-0752	BP119328
ceN-0753	BP119329
ceN-0754	BP119330
ceN-0755	BP119331
ceN-0756	BP119332
ceN-0757	BP119333
ceN-0758	BP119334
ceN-0759	BP119335
ceN-0760	BP119336
ceN-0761	BP119337
ceN-0762	BP119338
ceN-0763	BP119339
ceN-0765	BP119341
ceN-0766	BP119342
ceN-0768	BP119343
ceN-0769	BP119344
ceN-0770	BP119345
ceN-0772	BP119347
ceN-0773	BP119348
ceN-0775	BP119349
ceN-0776	BP119350
ceN-0777	BP119351
ceN-0778	BP119352
ceN-0780	BP119354
ceN-0781	BP119355
ceN-0782	BP119356
ceN-0783	BP119357
ceN-0784	BP119358
ceN-0786	BP119359
ceN-0787	BP119360
ceN-0788	BP119361
ceN-0790	BP119362
ceN-0791	BP119363

ceN-0792	BP119364
ceN-0793	BP119365
ceN-0794	BP119366
ceN-0795	BP119367
ceN-0796	BP119368
ceN-0797	BP119369
ceN-0798	BP119370
ceN-0799	BP119371
ceN-0800	BP119372
ceN-0802	BP119373
ceN-0803	BP119374
ceN-0804	BP119375
ceN-0805	BP119376
ceN-0807	BP119378
ceN-0809	BP119379
ceN-0811	BP119381
ceN-0812	BP119382
ceN-0813	BP119383
ceN-0816	BP119384
ceN-0817	BP119385
ceN-0818	BP119386
ceN-0819	BP119387
ceN-0821	BP119388
ceN-0822	BP119389
ceN-0823	BP119390
ceN-0824	BP119391
ceN-0825	BP119392
ceN-0829	BP119393
ceN-0830	BP119394
ceN-0831	BP119395
ceN-0832	BP119396
ceN-0833	BP119397
ceN-0834	BP119398
ceN-0835	BP119399
ceN-0836	BP119400
ceN-0838	BP119401
ceN-0841	BP119403
ceN-0842	BP119404
ceN-0843	BP119405
ceN-0844	BP119406
ceN-0845	BP119407
ceN-0846	BP119408
ceN-0847	BP119409
ceN-0848	BP119410
ceN-0850	BP119412
ceN-0851	BP119413
ceN-0852	BP119414
ceN-0853	BP119415
ceN-0855	BP119416
ceN-0856	BP119417
ceN-0857	BP119418
ceN-0858	BP119419
ceN-0859	BP119420
ceN-0861	BP119422

ceN-0862	BP119423
ceN-0866	BP119426
ceN-0868	BP119427
ceN-0869	BP119428
ceN-0870	BP119429
ceN-0873	BP119430
ceN-0875	BP119432
ceN-0876	BP119433
ceN-0877	BP119434
ceN-0878	BY914655
ceN-0879	BP119435
ceN-0880	BP119436
ceN-0882	BP119437
ceN-0884	BP119438
ceN-0885	BP119439
ceN-0886	BP119440
ceN-0892	BP119443
ceN-0893	BP119444
ceN-0894	BP119445
ceN-0895	BP119446
ceN-0897	BP119448
ceN-0898	BP119449
ceN-0899	BP119450
ceN-0901	BP119451
ceN-0903	BP119452
ceN-0905	BP119453
ceN-0909	BP119454
ceN-0910	BP119455
ceN-0911	BP119456
ceN-0912	BP119457
ceN-0913	BP119458
ceN-0914	BY914656
ceN-0915	BP119459
ceN-0916	BP119460
ceN-0917	BP119461
ceN-0919	BP119463
ceN-0920	BP119464
ceN-0921	BP119465
ceN-0922	BP119466
ceN-0923	BP119467
ceN-0924	BP119468
ceN-0925	BP119469
ceN-0926	BP119470
ceN-0927	BP119471
ceN-0928	BP119472
ceN-0929	BP119473
ceN-0931	BP119474
ceN-0932	BP119475
ceN-0934	BP119477
ceN-0936	BP119478
ceN-0938	BP119480
ceN-0939	BP119481
ceN-0942	BP119482
ceN-0944	BP119483

ceN-0945	BP119484
ceN-0946	BP119485
ceN-0948	BP119487
ceN-0951	BP119489
ceN-0953	BP119491
ceN-0954	BP119492
ceN-0955	BP119493
ceN-0957	BP119494
ceN-0958	BP119495
ceN-0961	BP119497
ceN-0962	BP119498
ceN-0963	BP119499
ceN-0964	BP119500
ceN-0965	BP119501
ceN-0966	BP119502
ceN-0967	BP119503
ceN-0969	BP119505
ceN-0970	BP119506
ceN-0971	BP119507
ceN-0972	BP119508
ceN-0973	BY914657
ceN-0978	BP119511
ceN-0979	BP119512
ceN-0981	BP119513
ceN-0982	BP119514
ceN-0984	BP119515
ceN-0985	BP119516
ceN-0986	BP119517
ceN-0989	BP119518
ceN-0990	BP119519
ceN-0993	BP119520
ceN-0994	BP119521
ceN-0997	BP119522
ceN-0998	BP119523
ceN-0999	BP119524
ceN-1000	BP119525
ceN-1001	BP119526
ceN-1002	BP119527
ceN-1003	BP119528
ceN-1006	BP119529
ceN-1008	BP119530
ceN-1010	BP119532
ceN-1011	BP119533
ceN-1012	BP119534
ceN-1016	BP119535
ceN-1018	BP119536
ceN-1019	BP119537
ceN-1020	BP119538
ceN-1022	BP119539
ceN-1024	BP119541
ceN-1025	BP119542
ceN-1026	BP119543
ceN-1027	BP119544
ceN-1028	BP119545

ceN-1030	BP119546
ceN-1031	BP119547
ceN-1032	BP119548
ceN-1035	BP119550
ceN-1040	BP119552
ceN-1042	BP119553
ceN-1043	BP119554
ceN-1045	BP119555
ceN-1046	BP119556
ceN-1047	BP119557
ceN-1049	BP119558
ceN-1050	BP119559
ceN-1051	BP119560
ceN-1052	BP119561
ceN-1053	BP119562
ceN-1054	BP119563
ceN-1153	BP119647
ceN-1154	BP119648
ceN-1156	BP119649
ceN-1157	BP119650
ceN-1158	BP119651
ceN-1159	BP119652
ceN-1160	BP119653
ceN-1162	BP119654
ceN-1163	BP119655
ceN-1164	BP119656
ceN-1165	BP119657
ceN-1168	BP119658
ceN-1169	BP119659
ceN-1171	BP119660
ceN-1172	BP119661
ceN-1173	BP119662
ceN-1174	BP119663
ceN-1175	BP119664
ceN-1176	BP119665
ceN-1177	BP119666
ceN-1178	BP119667
ceN-1179	BP119668
ceN-1181	BP119670
ceN-1182	BP119671
ceN-1183	BP119672
ceN-1185	BP119673
ceN-1186	BP119674
ceN-1187	BP119675
ceN-1189	BP119676
ceN-1190	BP119677
ceN-1191	BP119678
ceN-1192	BP119679
ceN-1193	BP119680
ceN-1195	BP119682
ceN-1196	BP119683
ceN-1197	BP119684
ceN-1198	BP119685
ceN-1199	BP119686

ceN-1201	BP119688
ceN-1202	BP119689
ceN-1203	BP119690
ceN-1208	BP119694
ceN-1210	BP119696
ceN-1211	BP119697
ceN-1212	BP119698
ceN-1214	BP119700
ceN-1215	BP119701
ceN-1219	BP119704
ceN-1222	BP119706
ceN-1223	BP119707
ceN-1224	BP119708
ceN-1226	BP119710
ceN-1229	BP119712
ceN-1231	BP119714
ceN-1232	BP119715
ceN-1233	BP119716
ceN-1234	BP119717
ceN-1236	BP119718
ceN-1237	BP119719
ceN-1238	BP119720
ceN-1239	BP119721
ceN-1240	BP119722
ceN-1241	BP119723
ceN-1242	BP119724
ceN-1243	BP119725
ceN-1244	BP119726
ceN-1245	BP119727
ceN-1248	BP119729
ceN-1249	BP119730
ceN-1250	BP119731
ceN-1251	BP119732
ceN-1252	BP119733
ceN-1253	BP119734
ceN-1255	BP119735
ceN-1256	BP119736
ceN-1258	BP119737
ceN-1259	BP119738
ceN-1260	BP119739
ceN-1261	BP119740
ceN-1263	BP119741
ceN-1264	BP119742
ceN-1265	BP119743
ceN-1267	BP119745
ceN-1268	BP119746
ceN-1269	BP119747
ceN-1270	BP119748
ceN-1271	BP119749
ceN-1274	BP119751
ceN-1275	BP119752
ceN-1276	BP119753
ceN-1277	BP119754
ceN-1278	BP119755

ceN-1279	BP119756
ceN-1280	BP119757
ceN-1281	BP119758
ceN-1282	BP119759
ceN-1283	BP119760
ceN-1284	BP119761
ceN-1288	BP119762
ceN-1289	BP119763
ceN-1290	BP119764
ceN-1292	BP119765
ceN-1294	BP119767
ceN-1297	BP119768
ceN-1298	BP119769
ceN-1299	BP119770
ceN-1300	BP119771
ceN-1302	BP119772
ceN-1303	BP119773
ceN-1304	BP119774
ceN-1305	BP119775
ceN-1306	BP119776
ceN-1307	BP119777
ceN-1308	BP119778
ceN-1309	BP119779
ceN-1311	BP119781
ceN-1312	BP119782
ceN-1313	BP119783
ceN-1314	BP119784
ceN-1317	BP119786
ceN-1319	BP119788
ceN-1322	BP119790
ceN-1324	BP119792
ceN-1325	BP119793
ceN-1326	BP119794
ceN-1327	BP119795
ceN-1330	BP119796
ceN-1331	BP119797
ceN-1332	BP119798
ceN-1333	BP119799
ceN-1334	BP119800
ceN-1336	BP119801
ceN-1337	BP119802
ceN-1339	BP119804
ceN-1340	BP119805
ceN-1341	BP119806
ceN-1342	BP119807
ceN-1343	BP119808
ceN-1347	BP119811
ceN-1352	BP119812
ceN-1353	BP119813
ceN-1354	BP119814
ceN-1355	BP119815
ceN-1357	BP119817
ceN-1361	BP119819
ceN-1362	BP119820

ceN-1364	BP119821
ceN-1367	BP119822
ceN-1368	BP119823
ceN-1370	BP119824
ceN-1373	BP119826
ceN-1375	BP119827
ceN-1377	BP119828
ceN-1378	BP119829
ceN-1379	BP119830
ceN-1382	BP119833
ceN-1384	BY914658
ceN-1385	BP119834
ceN-1386	BP119835
ceN-1387	BP119836
ceN-1388	BP119837
ceN-1390	BP119838
ceN-1391	BP119839
ceN-1394	BP119840
ceN-1395	BP119841
ceN-1400	BP119842
ceN-1401	BP119843
ceN-1402	BP119844
ceN-1405	BP119845
ceN-1406	BP119846
ceN-1410	BP119847
ceN-1411	BP119848
ceN-1412	BP119849
ceN-1413	BP119850
ceN-1414	BP119851
ceN-1415	BP119852
ceN-1416	BP119853
ceN-1418	BP119854
ceN-1420	BP119855
ceN-1421	BP119856
ceN-1422	BP119857
ceN-1427	BP119859
ceN-1428	BP119860
ceN-1430	BY914659
ceN-1431	BP119861
ceN-1433	BP119862
ceN-1435	BP119863
ceN-1437	BP119864
ceN-1438	BP119865
ceN-1439	BP119866
ceN-1440	BP119867
ceN-1446	BP119870
ceN-1448	BP119871
ceN-1449	BP119872
ceN-1451	BP119874
ceN-1452	BP119875
ceN-1454	BP119877
ceN-1456	BP119878
ceN-1457	BP119879
ceN-1459	BP119880

ceN-1460	BP119881
ceN-1461	BP119882
ceN-1462	BP119883
ceN-1464	BP119885
ceN-1468	BP119886
ceN-1470	BP119887
ceN-1471	BP119888
ceN-1473	BP119890
ceN-1475	BP119891
ceN-1476	BP119892
ceN-1477	BP119893
ceN-1479	BP119895
ceN-1480	BP119896
ceN-1481	BP119897
ceN-1482	BP119898
ceN-1483	BP119899
ceN-1484	BP119900
ceN-1485	BP119901
ceN-1487	BP119903
ceN-1488	BP119904
ceN-1492	BP119908
ceN-1493	BP119909
ceN-1494	BP119910
ceN-1496	BP119912
ceN-1497	BP119913
ceN-1498	BP119914
ceN-1499	BP119915
ceN-1500	BP119916
ceN-1501	BP119917
ceN-1504	BP119919
ceN-1505	BP119920
ceN-1506	BP119921
ceN-1507	BP119922
ceN-1508	BP119923
ceN-1509	BP119924
ceN-1511	BP119926
ceN-1512	BP119927
ceN-1515	BP119930
ceN-1516	BP119931
ceN-1517	BP119932
ceN-1518	BP119933
ceN-1519	BP119934
ceN-1521	BP119935
ceN-1523	BP119936
ceN-1524	BP119937
ceN-1525	BP119938
ceN-1526	BP119939
ceN-1527	BP119940
ceN-1528	BP119941
ceN-1529	BP119942
ceN-1530	BP119943
ceN-1533	BP119944
ceN-1534	BP119945
ceN-1535	BP119946

ceN-1536	BP119947
ceN-1538	BP119948
ceN-1540	BP119949
ceN-1542	BP119951
ceN-1546	BP119953
ceN-1547	BP119954
ceN-1548	BP119955
ceN-1552	BP119959
ceN-1553	BP119960
ceN-1554	BP119961
ceN-1555	BP119962
ceN-1557	BP119964
ceN-1558	BP119965
ceN-1559	BP119966
ceN-1561	BP119967
ceN-1562	BP119968
ceN-1564	BP119969
ceN-1566	BP119970
ceN-1567	BP119971
ceN-1569	BP119973
ceN-1570	BP119974
ceN-1572	BP119975
ceN-1575	BP119978
ceN-1576	BP119979
ceN-1577	BP119980
ceN-1578	BP119981
ceN-1579	BP119982
ceN-1580	BP119983
ceN-1582	BP119984
ceN-1583	BP119985
ceN-1584	BP119986
ceN-1585	BP119987
ceN-1586	BP119988
ceN-1587	BP119989
ceN-1588	BP119990
ceN-1589	BP119991
ceN-1590	BP119992
ceN-1598	BP119994
ceN-1599	BP119995
ceN-1601	BP119996
ceN-1602	BP119997
ceN-1603	BP119998
ceN-1605	BP119999
ceN-1606	BP120000
ceN-1608	BP120001
ceN-1611	BP120003
ceN-1612	BP120004
ceN-1613	BP120005
ceN-1614	BP120006
ceN-1617	BP120008
ceN-1618	BP120009
ceN-1619	BP120010
ceN-1621	BP120012
ceN-1623	BP120013

ceN-1624	BP120014
ceN-1625	BP120015
ceN-1626	BP120016
ceN-1627	BP120017
ceN-1628	BP120018
ceN-1629	BP120019
ceN-1631	BY914660
ceN-1632	BP120021
ceN-1634	BP120022
ceN-1635	BP120023
ceN-1638	BP120025
ceN-1639	BP120026
ceN-1640	BP120027
ceN-1641	BP120028
ceN-1642	BP120029
ceN-1643	BP120030
ceN-1644	BP120031
ceN-1645	BP120032
ceN-1647	BP120034
ceN-1648	BP120035
ceN-1650	BP120037
ceN-1651	BP120038
ceN-1652	BP120039
ceN-1653	BP120040
ceN-1654	BP120041
ceN-1655	BP120042
ceN-1658	BP120044
ceN-1659	BP120045
ceN-1660	BP120046
ceN-1662	BP120047
ceN-1663	BP120048
ceN-1664	BP120049
ceN-1665	BP120050
ceN-1666	BP120051
ceN-1667	BP120052
ceN-1668	BP120053
ceN-1669	BP120054
ceN-1670	BP120055
ceN-1673	BP120056
ceN-1676	BP120058
ceN-1677	BP120059
ceN-1678	BP120060
ceN-1681	BP120061
ceN-1682	BP120062
ceN-1686	BP120064
ceN-1688	BP120066
ceN-1690	BP120067
ceN-1693	BP120069
ceN-1694	BP120070
ceN-1695	BP120071
ceN-1696	BP120072
ceN-1697	BP120073
ceN-1698	BP120074
ceN-1699	BP120075

ceN-1700	BP120076
ceN-1704	BP120077
ceN-1707	BP120078
ceN-1708	BP120079
ceN-1711	BP120080
ceN-1712	BP120081
ceN-1713	BP120082
ceN-1717	BP120084
ceN-1718	BP120085
ceN-1719	BP120086
ceN-1720	BP120087
ceN-1723	BP120089
ceN-1724	BP120090
ceN-1725	BP120091
ceN-1726	BP120092
ceN-1728	BP120093
ceN-1750	BY914661
ceN-1769	BY914662
ceN-1770	BY914663
ceN-1771	BY914664
ceN-1772	BY914665
ceN-1773	BY914666
ceN-1776	BY914667
ceN-1787	BY914668
ceN-1788	BY914669
ceN-1797	BY914670
ceN-1800	BY914671
ceN-1809	BY914672
ceN-1815	BY914673
ceN-1818	BY914674
ceN-1825	BP120094
ceN-1826	BP120095
ceN-1827	BP120096
ceN-1828	BP120097
ceN-1829	BP120098
ceN-1830	BP120099
ceN-1832	BP120100
ceN-1833	BP120101
ceN-1834	BP120102
ceN-1835	BP120103
ceN-1836	BP120104
ceN-1837	BP120105
ceN-1839	BP120107
ceN-1840	BP120108
ceN-1842	BP120109
ceN-1843	BP120110
ceN-1844	BP120111
ceN-1845	BP120112
ceN-1846	BP120113
ceN-1848	BP120114
ceN-1850	BP120116
ceN-1851	BP120117
ceN-1853	BP120118
ceN-1854	BP120119

ceN-1856	BP120120
ceN-1860	BP120122
ceN-1861	BP120123
ceN-1862	BP120124
ceN-1863	BP120125
ceN-1864	BP120126
ceN-1866	BP120127
ceN-1867	BP120128
ceN-1868	BP120129
ceN-1869	BP120130
ceN-1870	BP120131
ceN-1871	BP120132
ceN-1872	BP120133
ceN-1873	BP120134
ceN-1874	BP120135
ceN-1875	BP120136
ceN-1878	BP120137
ceN-1879	BP120138
ceN-1881	BP120139
ceN-1883	BP120140
ceN-1884	BP120141
ceN-1885	BP120142
ceN-1887	BP120143
ceN-1888	BP120144
ceN-1889	BP120145
ceN-1890	BP120146
ceN-1891	BP120147
ceN-1892	BP120148
ceN-1894	BP120149
ceN-1895	BP120150
ceN-1896	BP120151
ceN-1897	BP120152
ceN-1898	BP120153
ceN-1899	BP120154
ceN-1900	BP120155
ceN-1901	BP120156
ceN-1902	BP120157
ceN-1904	BP120159
ceN-1906	BP120161
ceN-1907	BP120162
ceN-1909	BP120163
ceN-1910	BP120164
ceN-1912	BP120166
ceN-1916	BP120168
ceN-1918	BP120170
ceN-1919	BP120171
ceN-1921	BP120173
ceN-1923	BP120174
ceN-1925	BP120175
ceN-1926	BP120176
ceN-1928	BP120178
ceN-1929	BP120179
ceN-1931	BP120180
ceN-1932	BP120181

ceN-1933	BP120182
ceN-1935	BP120184
ceN-1936	BP120185
ceN-1937	BP120186
ceN-1938	BP120187
ceN-1940	BP120188
ceN-1941	BP120189
ceN-1942	BP120190
ceN-1944	BP120192
ceN-1945	BP120193
ceN-1947	BP120194
ceN-1948	BP120195
ceN-1949	BP120196
ceN-1950	BP120197
ceN-1951	BP120198
ceN-1953	BP120200
ceN-1954	BP120201
ceN-1955	BP120202
ceN-1957	BP120203
ceN-1958	BP120204
ceN-1959	BP120205
ceN-1960	BP120206
ceN-1961	BP120207
ceN-1962	BP120208
ceN-1963	BP120209
ceN-1964	BP120210
ceN-1965	BP120211
ceN-1966	BP120212
ceN-1967	BP120213
ceN-1970	BP120214
ceN-1972	BP120215
ceN-1973	BP120216
ceN-1975	BP120217
ceN-1977	BP120218
ceN-1978	BP120219
ceN-1980	BP120220
ceN-1981	BP120221
ceN-1982	BP120222
ceN-1983	BP120223
ceN-1984	BP120224
ceN-1986	BP120225
ceN-1990	BP120227
ceN-1991	BP120228
ceN-1994	BP120230
ceN-1995	BP120231
ceN-1996	BP120232
ceN-2000	BP120233
ceN-2001	BP120234
ceN-2002	BP120235
ceN-2003	BP120236
ceN-2006	BP120238
ceN-2007	BP120239
ceN-2008	BP120240
ceN-2009	BP120241

ceN-2010	BP120242
ceN-2011	BP120243
ceN-2012	BP120244
ceN-2013	BP120245
ceN-2014	BP120246
ceN-2015	BP120247
ceN-2017	BP120248
ceN-2018	BP120249
ceN-2019	BP120250
ceN-2020	BP120251
ceN-2021	BP120252
ceN-2024	BP120254
ceN-2025	BP120255
ceN-2027	BP120256
ceN-2028	BP120257
ceN-2029	BP120258
ceN-2030	BP120259
ceN-2031	BP120260
ceN-2032	BP120261
ceN-2034	BP120263
ceN-2036	BP120265
ceN-2037	BP120266
ceN-2038	BP120267
ceN-2040	BP120268
ceN-2041	BP120269
ceN-2042	BP120270
ceN-2043	BP120271
ceN-2046	BP120272
ceN-2047	BP120273
ceN-2048	BP120274
ceN-2049	BP120275
ceN-2050	BP120276
ceN-2052	BP120278
ceN-2055	BP120280
ceN-2057	BP120281
ceN-2058	BP120282
ceN-2059	BP120283
ceN-2064	BP120284
ceN-2066	BP120286
ceN-2067	BP120287
ceN-2069	BP120288
ceN-2070	BP120289
ceN-2072	BP120291
ceN-2075	BP120293
ceN-2076	BP120294
ceN-2077	BP120295
ceN-2078	BP120296
ceN-2079	BP120297
ceN-2081	BP120298
ceN-2082	BP120299
ceN-2084	BP120301
ceN-2085	BP120302
ceN-2086	BP120303
ceN-2087	BP120304

ceN-2088	BP120305
ceN-2090	BP120307
ceN-2092	BP120308
ceN-2093	BP120309
ceN-2095	BP120310
ceN-2096	BP120311
ceN-2098	BP120312
ceN-2099	BP120313
ceN-2102	BP120314
ceN-2104	BP120315
ceN-2107	BP120316
ceN-2111	BP120317
ceN-2112	BP120318
ceN-2115	BP120321
ceN-2116	BP120322
ceN-2117	BP120323
ceN-2118	BP120324
ceN-2120	BP120325
ceN-2122	BP120327
ceN-2124	BP120328
ceN-2125	BP120329
ceN-2126	BP120330
ceN-2129	BP120331
ceN-2130	BP120332
ceN-2131	BP120333
ceN-2132	BP120334
ceN-2133	BP120335
ceN-2134	BP120336
ceN-2135	BP120337
ceN-2136	BP120338
ceN-2139	BP120341
ceN-2140	BP120342
ceN-2141	BP120343
ceN-2143	BP120344
ceN-2144	BP120345
ceN-2145	BP120346
ceN-2146	BP120347
ceN-2148	BP120349
ceN-2149	BP120350
ceN-2150	BP120351
ceN-2151	BP120352
ceN-2152	BP120353
ceN-2153	BP120354
ceN-2154	BP120355
ceN-2155	BP120356
ceN-2156	BP120357
ceN-2157	BP120358
ceN-2158	BP120359
ceN-2159	BP120360
ceN-2160	BP120361
ceN-2162	BP120363
ceN-2163	BP120364
ceN-2164	BP120365
ceN-2166	BP120367

ceN-2167	BP120368
ceN-2168	BP120369
ceN-2169	BP120370
ceN-2170	BP120371
ceN-2171	BP120372
ceN-2172	BP120373
ceN-2173	BP120374
ceN-2174	BP120375
ceN-2176	BP120377
ceN-2177	BP120378
ceN-2178	BP120379
ceN-2179	BP120380
ceN-2183	BP120382
ceN-2186	BP120384
ceN-2187	BP120385
ceN-2192	BP120388
ceN-2194	BP120390
ceN-2195	BP120391
ceN-2196	BP120392
ceN-2197	BP120393
ceN-2199	BP120395
ceN-2201	BP120397
ceN-2204	BP120398
ceN-2205	BP120399
ceN-2206	BP120400
ceN-2207	BP120401
ceN-2208	BP120402
ceN-2211	BP120404
ceN-2212	BP120405
ceN-2213	BP120406
ceN-2214	BP120407
ceN-2215	BP120408
ceN-2217	BP120409
ceN-2218	BP120410
ceN-2219	BP120411
ceN-2221	BP120413
ceN-2222	BP120414
ceN-2223	BP120415
ceN-2225	BP120417
ceN-2227	BP120418
ceN-2228	BP120419
ceN-2229	BP120420
ceN-2230	BP120421
ceN-2232	BP120423
ceN-2234	BP120424
ceN-2235	BP120425
ceN-2238	BP120426
ceN-2239	BP120427
ceN-2240	BP120428
ceN-2242	BP120429
ceN-2243	BP120430
ceN-2244	BP120431
ceN-2245	BP120432
ceN-2247	BP120433

ceN-2250	BP120434
ceN-2253	BP120436
ceN-2254	BP120437
ceN-2255	BP120438
ceN-2256	BP120439
ceN-2258	BP120440
ceN-2259	BP120441
ceN-2260	BP120442
ceN-2262	BP120443
ceN-2263	BP120444
ceN-2264	BP120445
ceN-2266	BP120447
ceN-2267	BP120448
ceN-2268	BP120449
ceN-2269	BP120450
ceN-2270	BP120451
ceN-2271	BP120452
ceN-2272	BP120453
ceN-2273	BP120454
ceN-2275	BP120455
ceN-2278	BP120457
ceN-2279	BP120458
ceN-2280	BP120459
ceN-2282	BP120461
ceN-2284	BP120462
ceN-2287	BP120463
ceN-2288	BP120464
ceN-2290	BP120466
ceN-2293	BP120468
ceN-2295	BP120470
ceN-2296	BP120471
ceN-2298	BP120472
ceN-2299	BP120473
ceN-2300	BP120474
ceN-2302	BP120476
ceN-2308	BP120478
ceN-2309	BP120479
ceN-2311	BP120480
ceN-2312	BP120481
ceN-2313	BP120482
ceN-2316	BP120483
ceN-2318	BP120485
ceN-2319	BP120486
ceN-2322	BP120488
ceN-2323	BP120489
ceN-2325	BP120491
ceN-2326	BP120492
ceN-2327	BP120493
ceN-2332	BP120496
ceN-2333	BP120497
ceN-2334	BP120498
ceN-2336	BP120499
ceN-2337	BP120500
ceN-2339	BP120501

ceN-2340	BP120502
ceN-2341	BP120503
ceN-2342	BP120504
ceN-2343	BP120505
ceN-2345	BP120507
ceN-2346	BP120508
ceN-2348	BP120510
ceN-2350	BP120511
ceN-2351	BP120512
ceN-2354	BP120515
ceN-2355	BP120516
ceN-2356	BP120517
ceN-2358	BP120518
ceN-2361	BP120520
ceN-2362	BP120521
ceN-2363	BP120522
ceN-2364	BP120523
ceN-2365	BP120524
ceN-2366	BP120525
ceN-2367	BP120526
ceN-2368	BP120527
ceN-2369	BP120528
ceN-2370	BP120529
ceN-2371	BP120530
ceN-2372	BP120531
ceN-2374	BP120533
ceN-2375	BP120534
ceN-2376	BP120535
ceN-2379	BP120537
ceN-2380	BP120538
ceN-2381	BP120539
ceN-2382	BP120540
ceN-2384	BP120541
ceN-2389	BP120544
ceN-2391	BP120546
ceN-2394	BP120547
ceN-2395	BP120548
ceN-2397	BP120549
ceN-2404	BP120554
ceN-2405	BP120555
ceN-2406	BP120556
ceN-2407	BP120557
ceN-2408	BP120558
ceN-2409	BP120559
ceN-2412	BP120560
ceN-2414	BP120561
ceN-2415	BP120562
ceN-2416	BP120563
ceN-2417	BP120564
ceN-2418	BP120565
ceN-2419	BP120566
ceN-2420	BP120567
ceN-2421	BP120568
ceN-2422	BP120569

ceN-2423	BP120570
ceN-2424	BP120571
ceN-2427	BP120573
ceN-2428	BP120574
ceN-2429	BP120575
ceN-2430	BP120576
ceN-2431	BP120577
ceN-2432	BP120578
ceN-2434	BP120580
ceN-2435	BP120581
ceN-2436	BP120582
ceN-2437	BP120583
ceN-2438	BP120584
ceN-2439	BP120585
ceN-2440	BP120586
ceN-2441	BP120587
ceN-2442	BP120588
ceN-2443	BP120589
ceN-2444	BP120590
ceN-2446	BP120591
ceN-2447	BP120592
ceN-2450	BP120594
ceN-2451	BP120595
ceN-2452	BP120596
ceN-2453	BP120597
ceN-2455	BP120598
ceN-2456	BP120599
ceN-2458	BP120600
ceN-2461	BP120603
ceN-2462	BP120604
ceN-2464	BP120606
ceN-2465	BP120607
ceN-2466	BP120608
ceN-2468	BP120609
ceN-2469	BP120610
ceN-2470	BP120611
ceN-2471	BP120612
ceN-2474	BP120614
ceN-2476	BP120616
ceN-2478	BP120618
ceN-2480	BP120619
ceN-2481	BP120620
ceN-2483	BP120621
ceN-2485	BP120623
ceN-2486	BP120624
ceN-2487	BP120625
ceN-2488	BP120626
ceN-2489	BP120627
ceN-2491	BP120628
ceN-2492	BP120629
ceN-2493	BP120630
ceN-2494	BP120631
ceN-2495	BP120632
ceN-2498	BY914675

ceN-2500	BY914676
ceN-2501	BY914677
ceN-2504	BY914678
ceN-2505	BY914679
ceN-2506	BY914680
ceN-2507	BY914681
ceN-2509	BY914682
ceN-2514	BY914683
ceN-2515	BY914684
ceN-2516	BY914685
ceN-2517	BY914686
ceN-2520	BY914687
ceN-2522	BY914688
ceN-2525	BY914689
ceN-2526	BY914690
ceN-2527	BY914691
ceN-2529	BY914692
ceN-2532	BY914693
ceN-2533	BY914694
ceN-2538	BY914695
ceN-2539	BY914696
ceN-2540	BY914697
ceN-2541	BY914698
ceN-2543	BY914699
ceN-2544	BY914700
ceN-2548	BY914701
ceN-2549	BY914702
ceN-2550	BY914703
ceN-2553	BY914704
ceN-2554	BY914705
ceN-2555	BY914706
ceN-2556	BY914707
ceN-2561	BY914708
ceN-2562	BY914709
ceN-2563	BY914710
ceN-2565	BY914711
ceN-2566	BY914712
ceN-2567	BY914713
ceN-2568	BY914714
ceN-2570	BY914715
ceN-2572	BY914716
ceN-2573	BY914717
ceN-2574	BY914718
ceN-2577	BY914719
ceN-2579	BY914720
ceN-2580	BY914721
ceN-2584	BY914722
ceN-2586	BY914723
ceN-2587	BY914724
ceN-2588	BY914725
ceN-2589	BY914726
ceN-2591	BY914727
ceN-2593	BY914728
ceN-2594	BY914729

ceN-2595	BY914730
ceN-2596	BY914731
ceN-2597	BY914732
ceN-2598	BY914733
ceN-2599	BY914734
ceN-2601	BY914735
ceN-2602	BY914736
ceN-2603	BY914737
ceN-2604	BY914738
ceN-2605	BY914739
ceN-2606	BY914740
ceN-2607	BY914741
ceN-2608	BY914742
ceN-2610	BY914743
ceN-2612	BY914744
ceN-2613	BY914745
ceN-2616	BY914746
ceN-2618	BY914747
ceN-2619	BY914748
ceN-2620	BY914749
ceN-2621	BY914750
ceN-2622	BY914751
ceN-2623	BY914752
ceN-2628	BY914753
ceN-2629	BY914754
ceN-2635	BY914755
ceN-2639	BY914756
ceN-2640	BY914757
ceN-2641	BY914758
ceN-2642	BY914759
ceN-2643	BY914760
ceN-2644	BY914761
ceN-2645	BY914762
ceN-2646	BY914763
ceN-2647	BY914764
ceN-2648	BY914765
ceN-2649	BY914766
ceN-2650	BY914767
ceN-2651	BY914768
ceN-2652	BY914769
ceN-2654	BY914770
ceN-2655	BY914771
ceN-2656	BY914772
ceN-2657	BY914773
ceN-2658	BY914774
ceN-2659	BY914775
ceN-2660	BY914776
ceN-2661	BY914777
ceN-2662	BY914778
ceN-2663	BY914779
ceN-2664	BY914780
ceN-2665	BY914781
ceN-2666	BY914782
ceN-2667	BY914783

ceN-2668	BY914784
ceN-2669	BY914785
ceN-2671	BY914786
ceN-2672	BY914787
ceN-2673	BY914788
ceN-2674	BY914789
ceN-2676	BY914790
ceN-2678	BY914791
ceN-2679	BY914792
ceN-2680	BY914793
ceN-2681	BY914794
ceN-2682	BY914795
ceN-2683	BY914796
ceN-2684	BY914797
ceN-2685	BY914798
ceN-2687	BY914799
ceN-2688	BY914800
ceN-2689	BY914801
ceN-2690	BY914802
ceN-2692	BY914803
ceN-2693	BY914804
ceN-2695	BY914805
ceN-2697	BY914806
ceN-2698	BY914807
ceN-2699	BY914808
ceN-2701	BY914809
ceN-2703	BY914810
ceN-2704	BY914811
ceN-2705	BY914812
ceN-2706	BY914813
ceN-2707	BY914814
ceN-2708	BY914815
ceN-2709	BY914816
ceN-2710	BY914817
ceN-2711	BY914818
ceN-2712	BY914819
ceN-2713	BY914820
ceN-2714	BY914821
ceN-2717	BY914822
ceN-2719	BY914823
ceN-2721	BY914824
ceN-2727	BY914825
ceN-2728	BY914826
ceN-2730	BY914827
ceN-2731	BY914828
ceN-2732	BY914829
ceN-2733	BY914830
ceN-2734	BY914831
ceN-2740	BY914832
ceN-2741	BY914833
ceN-2742	BY914834
ceN-2743	BY914835
ceN-2744	BY914836
ceN-2745	BY914837

ceN-2746	BY914838
ceN-2747	BY914839
ceN-2749	BY914840
ceN-2751	BY914841
ceN-2752	BY914842
ceN-2753	BY914843
ceN-2754	BY914844
ceN-2755	BY914845
ceN-2756	BY914846
ceN-2757	BY914847
ceN-2758	BY914848
ceN-2759	BY914849
ceN-2760	BY914850
ceN-2761	BY914851
ceN-2763	BY914852
ceN-2764	BY914853
ceN-2765	BY914854
ceN-2767	BY914855
ceN-2769	BY914856
ceN-2772	BY914857
ceN-2773	BY914858
ceN-2776	BY914859
ceN-2778	BY914860
ceN-2782	BY914861
ceN-2783	BY914862
ceN-2786	BY914863
ceN-2788	BY914864
ceN-2793	BY914865
ceN-2794	BY914866
ceN-2796	BY914867
ceN-2797	BY914868
ceN-2798	BY914869
ceN-2801	BY914870
ceN-2802	BY914871
ceN-2804	BY914872
ceN-2805	BY914873
ceN-2806	BY914874
ceN-2808	BY914875
ceN-2809	BY914876
ceN-2810	BY914877
ceN-2818	BY914878
ceN-2819	BY914879
ceN-2820	BY914880
ceN-2821	BY914881
ceN-2822	BY914882
ceN-2823	BY914883
ceN-2824	BY914884
ceN-2827	BY914885
ceN-2829	BY914886
ceN-2835	BY914887
ceN-2837	BY914888
ceN-2838	BY914889
ceN-2839	BY914890
ceN-2840	BY914891

ceN-2842	BY914892
ceN-2844	BY914893
ceN-2846	BY914894
ceN-2848	BY914895
ceN-2850	BY914896
ceN-2852	BY914897
ceN-2853	BY914898
ceN-2855	BY914899
ceN-2863	BY914900
ceN-2866	BY914901
ceN-2870	BY914902
ceN-2873	BY914903
ceN-2876	BY914904
ceN-2877	BY914905
ceN-2878	BY914906
ceN-2881	BY914907
ceN-2883	BY914908
ceN-2884	BY914909
ceN-2885	BY914910
ceN-2886	BY914911
ceN-2888	BY914912
ceN-2889	BY914913
ceN-2890	BY914914
ceN-2891	BY914915
ceN-2892	BY914916
ceN-2894	BY914917
ceN-2895	BY914918
ceN-2896	BY914919
ceN-2897	BY914920
ceN-2898	BY914921
ceN-2899	BY914922
ceN-2900	BY914923
ceN-2901	BY914924
ceN-2903	BY914925
ceN-2904	BY914926
ceN-2906	BY914927
ceN-2907	BY914928
ceN-2908	BY914929
ceN-2909	BY914930
ceN-2910	BY914931
ceN-2911	BY914932
ceN-2912	BY914933
ceN-2913	BY914934
ceN-2915	BY914935
ceN-2918	BY914936
ceN-2921	BY914937
ceN-2923	BY914938
ceN-2924	BY914939
ceN-2925	BY914940
ceN-2926	BY914941
ceN-2930	BY914942
ceN-2934	BY914943
ceN-2935	BY914944
ceN-2936	BY914945

ceN-2937	BY914946
ceN-2938	BY914947
ceN-2940	BY914948
ceN-2941	BY914949
ceN-2942	BY914950
ceN-2943	BY914951
ceN-2944	BY914952
ceN-2945	BY914953
ceN-2946	BY914954
ceN-2949	BY914955
ceN-2950	BY914956
ceN-2952	BY914957
ceN-2954	BY914958
ceN-2955	BY914959
ceN-2957	BY914960
ceN-2958	BY914961
ceN-2959	BY914962
ceN-2960	BY914963
ceN-2961	BY914964
ceN-2963	BY914965
ceN-2964	BY914966
ceN-2966	BY914967
ceN-2967	BY914968
ceN-2968	BY914969
ceN-2969	BY914970
ceN-2970	BY914971
ceN-2971	BY914972
ceN-2973	BY914973
ceN-2976	BY914974
ceN-2979	BY914975
ceN-2980	BY914976
ceN-2981	BY914977
ceN-2982	BY914978
ceN-2983	BY914979
ceN-2986	BY914980
ceN-2987	BY914981
ceN-2989	BY914982
ceN-2990	BY914983
ceN-2991	BY914984
ceN-2992	BY914985
ceN-2993	BY914986
ceN-2994	BY914987
ceN-2996	BY914988
ceN-2997	BY914989
ceN-2998	BY914990
ceN-3001	BY914991
ceN-3003	BY914992
ceN-3007	BY914993
ceN-3008	BY914994
ceN-3013	BY914995
ceN-3017	BY914996
ceN-3018	BY914997
ceN-3020	BY914998
ceN-3021	BY914999

ceN-3024	BY915000
ceN-3025	BY915001
ceN-3026	BY915002
ceN-3028	BY915003
ceN-3029	BY915004
ceN-3032	BY915005
ceN-3033	BY915006
ceN-3034	BY915007
ceN-3035	BY915008
ceN-3036	BY915009
ceN-3037	BY915010
ceN-3038	BY915011
ceN-3039	BY915012
ceN-3040	BY915013
ceN-3042	BY915014
ceN-3043	BY915015
ceN-3044	BY915016
ceN-3045	BY915017
ceN-3046	BY915018
ceN-3047	BY915019
ceN-3048	BY915020
ceN-3051	BY915021
ceN-3055	BY915022
ceN-3056	BY915023
ceN-3059	BY915024
ceN-3060	BY915025
ceN-3063	BY915026
ceN-3065	BY915027
ceN-3067	BY915028
ceN-3068	BY915029
ceN-3069	BY915030
ceN-3071	BY915031
ceN-3072	BY915032
ceN-3073	BY915033
ceN-3076	BY915034
ceN-3077	BY915035
ceN-3078	BY915036
ceN-3079	BY915037
ceN-3080	BY915038
ceN-3083	BY915039
ceN-3085	BY915040
ceN-3086	BY915041
ceN-3091	BY915042
ceN-3092	BY915043
ceN-3093	BY915044
ceN-3094	BY915045
ceN-3098	BY915046
ceN-3099	BY915047
ceN-3100	BY915048
ceN-3101	BY915049
ceN-3103	BY915050
ceN-3104	BY915051
ceN-3107	BY915052
ceN-3108	BY915053

ceN-3109	BY915054
ceN-3110	BY915055
ceN-3111	BY915056
ceN-3112	BY915057
ceN-3120	BY915058
ceN-3123	BY915059
ceN-3124	BY915060
ceN-3126	BY915061
ceN-3127	BY915062
ceN-3129	BY915063
ceN-3131	BY915064
ceN-3133	BY915065
ceN-3137	BY915066
ceN-3139	BY915067
ceN-3140	BY915068
ceN-3141	BY915069
ceN-3142	BY915070
ceN-3143	BY915071
ceN-3144	BY915072
ceN-3146	BY915073
ceN-3147	BY915074
ceN-3152	BY915075
ceN-3153	BY915076
ceN-3154	BY915077
ceN-3155	BY915078
ceN-3157	BY915079
ceN-3158	BY915080
ceN-3160	BY915081
ceN-3166	BY915082
ceN-3170	BY915083
ceN-3171	BY915084
ceN-3173	BY915085
ceN-3174	BY915086
ceN-3176	BY915087
ceN-3177	BY915088
ceN-3178	BY915089
ceN-3181	BY915090
ceN-3183	BY915091
ceN-3184	BY915092
ceN-3185	BY915093
ceN-3188	BY915094
ceN-3189	BY915095
ceN-3190	BY915096
ceN-3191	BY915097
ceN-3192	BY915098
ceN-3193	BY915099
ceN-3194	BY915100
ceN-3195	BY915101
ceN-3197	BY915102
ceN-3200	BY915103
ceN-3204	BY915104
ceN-3205	BY915105
ceN-3206	BY915106
ceN-3207	BY915107

ceN-3208	BY915108
ceN-3210	BY915109
ceN-3213	BY915110
ceN-3214	BY915111
ceN-3215	BY915112
ceN-3216	BY915113
ceN-3218	BY915114
ceN-3219	BY915115
ceN-3220	BY915116
ceN-3221	BY915117
ceN-3222	BY915118
ceN-3224	BY915119
ceN-3225	BY915120
ceN-3227	BY915121
ceN-3228	BY915122
ceN-3229	BY915123
ceN-3230	BY915124
ceN-3231	BY915125
ceN-3233	BY915126
ceN-3234	BY915127
ceN-3237	BY915128
ceN-3240	BY915129
ceN-3241	BY915130
ceN-3242	BY915131
ceN-3243	BY915132
ceN-3244	BY915133
ceN-3245	BY915134
ceN-3246	BY915135
ceN-3247	BY915136
ceN-3249	BY915137
ceN-3250	BY915138
ceN-3251	BY915139
ceN-3252	BY915140
ceN-3253	BY915141
ceN-3254	BY915142
ceN-3256	BY915143
ceN-3257	BY915144
ceN-3259	BY915145
ceN-3260	BY915146
ceN-3263	BY915147
ceN-3268	BY915148
ceN-3269	BY915149
ceN-3270	BY915150
ceN-3271	BY915151
ceN-3272	BY915152
ceN-3273	BY915153
ceN-3274	BY915154
ceN-3275	BY915155
ceN-3276	BY915156
ceN-3277	BY915157
ceN-3278	BY915158
ceN-3279	BY915159
ceN-3282	BY915160
ceN-3285	BY915161

ceN-3288	BY915162
ceN-3291	BY915163
ceN-3295	BY915164
ceN-3296	BY915165
ceN-3299	BY915166
ceN-3301	BY915167
ceN-3302	BY915168
ceN-3306	BY915169
ceN-3308	BY915170
ceN-3312	BY915171
ceN-3314	BY915172
ceN-3315	BY915173
ceN-3316	BY915174
ceN-3317	BY915175
ceN-3320	BY915176
ceN-3321	BY915177
ceN-3324	BY915178
ceN-3325	BY915179
ceN-3327	BY915180
ceN-3328	BY915181
ceN-3331	BY915182
ceN-3332	BY915183
ceN-3333	BY915184
ceN-3334	BY915185
ceN-3336	BY915186
ceN-3337	BY915187
ceN-3338	BY915188
ceN-3339	BY915189
ceN-3341	BY915190
ceN-3344	BY915191
ceN-3345	BY915192
ceN-3347	BY915193
ceN-3349	BY915194
ceN-3350	BY915195
ceN-3351	BY915196
ceN-3353	BY915197
ceN-3354	BY915198
ceN-3355	BY915199
ceN-3357	BY915200
ceN-3358	BY915201
ceN-3361	BY915202
ceN-3362	BY915203
ceN-3364	BY915204
ceN-3365	BY915205
ceN-3366	BY915206
ceN-3367	BY915207
ceN-3368	BY915208
ceN-3369	BY915209
ceN-3370	BY915210
ceN-3374	BY915211
ceN-3375	BY915212
ceN-3377	BY915213
ceN-3379	BY915214
ceN-3381	BY915215

	ceN-3382	BY915216
	ceN-3385	BY915217
	ceN-3388	BY915218
	ceN-3389	BY915219
	ceN-3390	BY915220
	ceN-3391	BY915221
	ceN-3392	BY915222
	ceN-3395	BY915223
	ceN-3397	BY915224
	ceN-3398	BY915225
	ceN-3399	BY915226
	ceN-3400	BY915227
	ceN-3401	BY915228
	ceN-3402	BY915229
	ceN-3403	BY915230
	ceN-3405	BY915231
	ceN-3407	BY915232
	ceN-3408	BY915233
	ceN-3421	BY915234
	ceN-3422	BY915235
	ceN-3423	BY915236
	ceN-3426	BY915237
	ceN-3428	BY915238
	ceN-3429	BY915239
	ceN-3430	BY915240
	ceN-3431	BY915241
	ceN-3433	BY915242
	ceN-3434	BY915243
	ceN-3435	BY915244
	ceN-3436	BY915245
	ceN-3437	BY915246
	ceN-3438	BY915247
	ceN-3440	BY915248
	ceN-3441	BY915249
	ceN-3442	BY915250
	ceN-3443	BY915251
	ceN-3445	BY915252
	ceN-3446	BY915253
	ceN-3447	BY915254
	ceN-3448	BY915255
	ceN-3450	BY915256
	ceN-3451	BY915257
	ceN-3452	BY915258
	ceN-3453	BY915259
	ceN-3454	BY915260
	ceN-3455	BY915261
e4	e40001	AU000001
	e40002	AU000002
	e40003	AU000003
	e40004	AU000004
	e40005	AU000005
	e40006	AU000006
	e40007	AU000007
	e40008	AU000008

e40009	AU000009
e40010	AU000010
e40011	AU000011
e40012	AU000012
e40013	AU000013
e40015	AU000014
e40016	AU000015
e40017	AU000016
e40018	AU000017
e40019	AU000018
e40020	AU000019
e40021	AU000020
e40022	AU000021
e40023	BY915262
e40024	AU000022
e40025	AU000023
e40026	AU000024
e40027	AU000025
e40028	AU000026
e40029	AU000027
e40030	AU000028
e40031	AU000029
e40033	AU000030
e40034	AU000031
e40035	AU000032
e40036	AU000033
e40037	AU000034
e40039	AU000036
e40040	AU000037
e40041	AU000038
e40044	AU000039
e40045	AU000040
e40046	AU000041
e40047	AU000042
e40048	AU000043
e40049	AU000044
e40050	AU000045
e40051	AU000046
e40052	AU000047
e40056	AU000048
e40057	AU000049
e40058	AU000050
e40059	AU000051
e40060	AU000052
e40061	AU000053
e40062	AU000054
e40063	AU000055
e40064	AU000056
e40065	AU000057
e40066	AU000058
e40067	AU000059
e40068	AU000060
e40069	AU000061
e40070	AU000062

e40071	AU000063
e40073	AU000064
e40074	AU000065
e40076	AU000067
e40078	AU000068
e40079	AU000069
e40080	AU000070
e40081	AU000071
e40082	AU000072
e40083	AU000073
e40084	AU000074
e40085	AU000075
e40086	AU000076
e40087	AU000077
e40088	AU000078
e40089	AU000079
e40090	AU000080
e40091	AU000081
e40092	AU000082
e40093	AU000083
e40094	AU000084
e40095	AU000085
e40096	AU000086
e40097	AU000087
e40098	AU000088
e40099	AU000089
e40100	AU000090
e40101	AU000091
e40103	AU000093
e40104	AU000094
e40105	AU000095
e40106	AU000096
e40107	AU000097
e40109	AU000098
e40110	BY915263
e40111	AU000099
e40112	AU000100
e40113	AU000101
e40115	AU000102
e40116	AU000103
e40117	AU000104
e40118	AU000105
e40120	AU000106
e40121	AU000107
e40122	AU000108
e40123	AU000109
e40124	AU000110
e40125	AU000111
e40128	AU000113
e40129	AU000114
e40130	AU000115
e40131	AU000116
e40132	AU000117
e40133	AU000118

e40135	AU000119
e40137	AU000120
e40138	AU000121
e40139	AU000122
e40140	AU000123
e40142	AU000124
e40143	AU000125
e40144	AU000126
e40145	AU000127
e40146	AU000128
e40147	AU000129
e40148	AU000130
e40149	AU000131
e40151	AU000132
e40152	AU000133
e40153	BY915264
e40154	AU000134
e40156	AU000135
e40157	AU000136
e40158	AU000137
e40159	AU000138
e40160	AU000139
e40161	AU000140
e40163	AU000142
e40164	AU000143
e40165	AU000144
e40166	AU000145
e40167	BY915265
e40169	AU000146
e40170	AU000147
e40172	AU000148
e40173	AU000149
e40175	AU000150
e40176	AU000151
e40178	AU000152
e40179	AU000153
e40180	AU000154
e40181	AU000155
e40182	AU000156
e40183	AU000157
e40184	AU000158
e40185	AU000159
e40186	AU000160
e40188	AU000161
e40189	AU000162
e40190	AU000163
e40192	AU000164
e40193	AU000165
e40194	AU000166
e40195	AU000167
e40196	AU000168
e40197	AU000169
e40198	AU000170
e40199	AU000171

e40200	AU000172
e40201	AU000173
e40202	AU000174
e40203	AU000175
e40204	AU000176
e40205	AU000177
e40206	AU000178
e40207	AU000179
e40208	AU000180
e40209	AU000181
e40210	AU000182
e40212	AU000183
e40213	AU000184
e40214	AU000185
e40215	AU000186
e40216	AU000187
e40218	AU000188
e40219	AU000189
e40221	AU000191
e40222	AU000192
e40223	AU000193
e40224	AU000194
e40225	AU000195
e40227	AU000196
e40228	AU000197
e40229	AU000198
e40230	AU000199
e40231	AU000200
e40232	AU000201
e40233	AU000202
e40234	AU000203
e40235	AU000204
e40236	AU000205
e40237	AU000206
e40238	AU000207
e40239	AU000208
e40240	AU000209
e40241	AU000210
e40242	AU000211
e40243	AU000212
e40244	AU000213
e40249	AU000215
e40250	AU000216
e40252	AU000217
e40253	AU000218
e40254	AU000219
e40255	AU000220
e40258	AU000221
e40259	AU000222
e40260	AU000223
e40261	BY915266
e40262	AU000224
e40263	AU000225
e40265	AU000226

e40266	AU000227
e40269	AU000228
e40272	AU000230
e40273	AU000231
e40277	AU000233
e40278	AU000234
e40280	AU000235
e40282	AU000236
e40283	AU000237
e40284	AU000238
e40285	AU000239
e40286	AU000240
e40287	AU000241
e40288	AU000242
e40289	AU000243
e40291	AU000245
e40292	AU000246
e40293	AU000247
e40295	AU000248
e40296	AU000249
e40297	AU000250
e40298	AU000251
e40299	AU000252
e40300	AU000253
e40301	AU000254
e40302	AU000255
e40303	AU000256
e40305	AU000257
e40308	AU000258
e40310	AU000259
e40312	AU000260
e40314	AU000261
e40315	AU000262
e40316	AU000263
e40317	AU000264
e40318	AU000265
e40319	AU000266
e40320	AU000267
e40321	AU000268
e40323	AU000269
e40324	AU000270
e40327	AU000271
e40331	AU000272
e40333	AU000273
e40334	AU000274
e40336	AU000275
e40339	AU000276
e40340	AU000277
e40342	AU000278
e40343	AU000279
e40344	AU000280
e40346	AU000281
e40347	AU000282
e40348	AU000283

e40350	AU000284
e40351	AU000285
e40352	AU000286
e40353	AU000287
e40354	AU000288
e40356	AU000289
e40357	AU000290
e40363	AU000291
e40365	AU000292
e40366	AU000293
e40367	AU000294
e40368	AU000295
e40369	AU000296
e40370	AU000297
e40371	AU000298
e40372	AU000299
e40374	AU000300
e40375	AU000301
e40377	AU000302
e40379	AU000303
e40381	AU000304
e40382	AU000305
e40384	AU000306
e40385	AU000307
e40386	AU000308
e40387	AU000309
e40389	AU000310
e40390	AU000311
e40392	AU000312
e40393	AU000313
e40394	AU000314
e40395	AU000315
e40396	AU000316
e40397	AU000317
e40398	AU000318
e40399	AU000319
e40400	AU000320
e40401	AU000321
e40402	AU000322
e40405	AU000324
e40406	AU000325
e40408	AU000326
e40409	AU000327
e40410	AU000328
e40411	AU000329
e40412	AU000330
e40413	AU000331
e40414	AU000332
e40415	AU000333
e40416	AU000334
e40417	AU000335
e40419	AU000336
e40420	AU000337
e40423	AU000338

e40425	AU000339
e40426	AU000340
e40427	AU000341
e40428	AU000342
e40429	AU000343
e40431	AU000345
e40432	AU000346
e40433	AU000347
e40434	AU000348
e40435	AU000349
e40436	AU000350
e40437	AU000351
e40438	AU000352
e40439	AU000353
e40440	AU000354
e40441	AU000355
e40444	AU000356
e40445	AU000357
e40446	AU000358
e40448	AU000359
e40449	AU000360
e40450	AU000361
e40451	AU000362
e40452	AU000363
e40454	AU000364
e40456	AU000365
e40457	AU000366
e40458	AU000367
e40460	AU000369
e40461	AU000370
e40462	AU000371
e40463	AU000372
e40464	AU000373
e40465	AU000374
e40467	AU000375
e40468	AU000376
e40470	AU000377
e40471	AU000378
e40472	AU000379
e40473	AU000380
e40474	AU000381
e40475	AU000382
e40477	AU000383
e40478	AU000384
e40479	AU000385
e40481	AU000386
e40483	AU000387
e40485	AU000388
e40486	AU000389
e40487	AU000390
e40488	AU000391
e40489	AU000392
e40490	AU000393
e40492	AU000394

e40493	AU000395
e40494	AU000396
e40495	AU000397
e40496	AU000398
e40497	AU000399
e40498	AU000400
e40500	AU000401
e40501	AU000402
e40503	AU000403
e40504	AU000404
e40505	AU000405
e40506	AU000406
e40507	AU000407
e40508	AU000408
e40510	AU000409
e40511	AU000410
e40513	AU000411
e40514	AU000412
e40517	AU000413
e40518	AU000414
e40519	AU000415
e40521	AU000416
e40522	AU000417
e40523	AU000418
e40524	AU000419
e40525	AU000420
e40526	AU000421
e40527	AU000422
e40528	AU000423
e40529	AU000424
e40530	AU000425
e40531	AU000426
e40533	AU000428
e40534	AU000429
e40535	AU000430
e40537	AU000431
e40538	AU000432
e40540	AU000433
e40542	AU000434
e40543	AU000435
e40544	AU000436
e40547	AU000439
e40548	AU000440
e40549	AU000441
e40550	AU000442
e40551	AU000443
e40552	AU000444
e40553	AU000445
e40554	AU000446
e40555	AU000447
e40556	AU000448
e40557	AU000449
e40558	AU000450
e40561	AU000452

e40562	AU000453
e40563	AU000454
e40565	AU000456
e40567	AU000458
e40568	AU000459
e40569	AU000460
e40570	AU000461
e40571	AU000462
e40573	AU000464
e40574	AU000465
e40575	AU000466
e40576	AU000467
e40578	AU000469
e40579	AU000470
e40580	AU000471
e40583	AU000473
e40584	AU000474
e40585	AU000475
e40587	AU000476
e40588	AU000477
e40590	AU000479
e40591	AU000480
e40593	AU000481
e40595	AU000482
e40597	AU000484
e40598	AU000485
e40599	AU000486
e40602	AU000488
e40603	AU000489
e40604	AU000490
e40605	AU000491
e40606	AU000492
e40607	AU000493
e40608	AU000494
e40609	AU000495
e40610	AU000496
e40611	AU000497
e40612	AU000498
e40614	AU000499
e40615	AU000500
e40616	AU000501
e40617	AU000502
e40618	AU000503
e40619	AU000504
e40620	AU000505
e40621	AU000506
e40622	AU000507
e40623	AU000508
e40624	AU000509
e40625	AU000510
e40626	AU000511
e40628	AU000512
e40630	AU000513
e40632	AU000514

e40633	AU000515
e40635	AU000516
e40636	AU000517
e40637	AU000518
e40638	AU000519
e40639	AU000520
e40640	AU000521
e40641	AU000522
e40642	AU000523
e40643	AU000524
e40645	AU000525
e40646	AU000526
e40647	AU000527
e40648	BY915267
e40650	AU000529
e40651	AU000530
e40652	AU000531
e40654	AU000532
e40655	AU000533
e40656	AU000534
e40658	AU000535
e40661	AU000536
e40662	AU000537
e40663	AU000538
e40664	AU000539
e40667	AU000541
e40668	AU000542
e40669	AU000543
e40672	AU000544
e40676	AU000545
e40677	AU000546
e40678	AU000547
e40681	AU000548
e40682	AU000549
e40683	AU000550
e40687	AU000551
e40688	AU000552
e40689	AU000553
e40692	AU000555
e40693	AU000556
e40694	AU000557
e40695	AU000558
e40699	BY915268
e40700	AU000559
e40701	AU000560
e40702	AU000561
e40703	AU000562
e40705	AU000563
e40707	AU000564
e40710	AU000565
e40711	AU000566
e40712	AU000567
e40713	AU000568
e40714	AU000569

e40715	AU000570
e40716	AU000571
e40718	AU000573
e40719	AU000574
e40720	AU000575
e40724	AU000576
e40725	AU000577
e40726	AU000578
e40727	AU000579
e40728	AU000580
e40730	AU000581
e40731	AU000582
e40736	AU000583
e40737	AU000584
e40738	AU000585
e40739	AU000586
e40741	AU000587
e40742	AU000588
e40743	AU000589
e40748	AU000590
e40750	AU000591
e40751	AU000592
e40753	AU000594
e40754	AU000595
e40755	AU000596
e40756	AU000597
e40761	AU000598
e40762	AU000599
e40767	AU000600
e40770	AU000602
e40772	AU000604
e40773	AU000605
e40774	AU000606
e40775	AU000607
e40776	AU000608
e40777	AU000609
e40778	AU000610
e40779	AU000611
e40780	AU000612
e40781	AU000613
e40782	AU000614
e40783	AU000615
e40784	AU000616
e40785	AU000617
e40791	AU000620
e40794	AU000621
e40796	AU000622
e40798	AU000624
e40800	AU000625
e40801	AU000626
e40803	AU000627
e40804	BY915269
e40805	AU000628
e40806	AU000629

e40807	AU000630
e40808	AU000631
e40809	AU000632
e40810	AU000633
e40815	AU000635
e40816	AU000636
e40817	AU000637
e40818	AU000638
e40819	AU000639
e40821	AU000640
e40822	AU000641
e40823	AU000642
e40824	AU000643
e40825	AU000644
e40826	AU000645
e40827	AU000646
e40828	AU000647
e40829	AU000648
e40831	AU000649
e40832	AU000650
e40834	AU000651
e40835	AU000652
e40836	AU000653
e40837	AU000654
e40838	AU000655
e40839	AU000656
e40840	AU000657
e40841	AU000658
e40842	AU000659
e40843	AU000660
e40844	AU000661
e40845	BY915270
e40846	AU000662
e40848	AU000663
e40849	AU000664
e40850	AU000665
e40852	AU000666
e40853	AU000667
e40854	AU000668
e40855	AU000669
e40856	AU000670
e40857	AU000671
e40858	AU000672
e40859	AU000673
e40860	AU000674
e40863	AU000675
e40864	AU000676
e40865	AU000677
e40866	AU000678
e40868	AU000680
e40869	AU000681
e40870	AU000682
e40871	AU000683
e40872	AU000684

e40873	AU000685
e40874	AU000686
e40875	AU000687
e40876	AU000688
e40877	AU000689
e40878	AU000690
e40879	AU000691
e40880	AU000692
e40881	AU000693
e40884	AU000695
e40885	AU000696
e40886	AU000697
e40887	AU000698
e40888	AU000699
e40889	AU000700
e40891	AU000702
e40892	AU000703
e40893	AU000704
e40894	AU000705
e40896	AU000706
e40897	AU000707
e40898	AU000708
e40899	AU000709
e40900	AU000710
e40901	AU000711
e40902	AU000712
e40904	AU000713
e40905	AU000714
e40906	AU000715
e40907	AU000716
e40908	AU000717
e40909	AU000718
e40910	AU000719
e40911	AU000720
e40913	AU000721
e40914	AU000722
e40915	AU000723
e40916	AU000724
e40917	AU000725
e40918	AU000726
e40919	AU000727
e40920	AU000728
e40921	AU000729
e40922	AU000730
e40923	AU000731
e40924	AU000732
e40925	AU000733
e40927	AU000735
e40928	AU000736
e40929	AU000737
e40930	AU000738
e40931	AU000739
e40932	AU000740
e40933	AU000741

	e40935	AU000742
	e40936	AU000743
	e40937	AU000744
	e40938	AU000745
	e40939	AU000746
	e40942	AU000747
	e40946	AU000751
	e40948	AU000752
	e40949	AU000753
	e40950	AU000754
	e40951	AU000755
	e40952	AU000756
	e40953	AU000757
	e40954	AU000758
	e40955	AU000759
	e40957	AU000760
	e40958	AU000761
	e40960	AU000762
e96h	e96h0001	AV400935
	e96h0002	AV400936
	e96h0003	AV400937
	e96h0004	AV400938
	e96h0007	AV400939
	e96h0008	AV400940
	e96h0010	AV400941
	e96h0011	AV400942
	e96h0013	AV400943
	e96h0016	AV400944
	e96h0021	AV400946
	e96h0022	AV400947
	e96h0023	AV400948
	e96h0024	AV400949
	e96h0025	AV400950
	e96h0028	AV400952
	e96h0029	AV400953
	e96h0030	AV400954
	e96h0031	AV400955
	e96h0032	AV400956
	e96h0033	AV400957
	e96h0034	AV400958
	e96h0036	AV400959
	e96h0037	AV400960
	e96h0038	AV400961
	e96h0040	AV400962
	e96h0041	AV400963
	e96h0042	AV400964
	e96h0044	AV400965
	e96h0045	AV400966
	e96h0046	AV400967
	e96h0047	AV400968
	e96h0048	AV400969
	e96h0051	AV400970
	e96h0052	AV400971
	e96h0053X	AV400972

e96h0054	AV400973
e96h0055	AV400974
e96h0056	AV400975
e96h0057	AV400976
e96h0058	AV400977
e96h0060	AV400978
e96h0061	AV400979
e96h0062	AV400980
e96h0064	AV400981
e96h0066	AV400982
e96h0067	AV400983
e96h0069	AV400985
e96h0070	AV400986
e96h0071	AV400987
e96h0072	AV400988
e96h0073	AV400989
e96h0075	AV400990
e96h0077	AV400991
e96h0081	AV400993
e96h0082	AV400994
e96h0083	AV400995
e96h0084	AV400996
e96h0090	AV400998
e96h0091	AV400999
e96h0093	AV401000
e96h0095	AV401001
e96h0096	AV401002
e96h0097	AV401003
e96h0098	AV401004
e96h0099	AV401005
e96h0100	AV401006
e96h0101	AV401007
e96h0102	AV401008
e96h0103	AV401009
e96h0105	AV401010
e96h0109	AV401012
e96h0110	AV401013
e96h0114	AV401014
e96h0115	AV401015
e96h0117	AV401016
e96h0120	AV401017
e96h0121	AV401018
e96h0122	AV401019
e96h0123	AV401020
e96h0125	AV401021
e96h0126	AV401022
e96h0127	AV401023
e96h0129	AV401024
e96h0130	AV401025
e96h0131	AV401026
e96h0132	AV401027
e96h0134	AV401028
e96h0137	AV401029
e96h0138	AV401030

e96h0140	AV401032
e96h0143	AV401034
e96h0145	AV401035
e96h0146	AV401036
e96h0147	AV401037
e96h0148	AV401038
e96h0149	AV401039
e96h0150	AV401040
e96h0151	AV401041
e96h0152	AV401042
e96h0153	AV401043
e96h0154	AV401044
e96h0156	AV401045
e96h0157	AV401046
e96h0158	AV401047
e96h0159	AV401048
e96h0160	AV401049
e96h0161	AV401050
e96h0162	AV401051
e96h0163	AV401052
e96h0164	AV401053
e96h0165	AV401054
e96h0166	AV401055
e96h0167	AV401056
e96h0168	AV401057
e96h0169	AV401058
e96h0170	AV401059
e96h0171	AV401060
e96h0172	AV401061
e96h0173	AV401062
e96h0174	AV401063
e96h0176	AV401064
e96h0179	AV401065
e96h0180	AV401066
e96h0181	AV401067
e96h0182	AV401068
e96h0183	AV401069
e96h0184	AV401070
e96h0186	AV401071
e96h0188	AV401072
e96h0190	AV401073
e96h0192	AV401074
e96h0195	AV401076
e96h0199	AV401077
e96h0200	AV401078
e96h0201	AV401079
e96h0202	AV401080
e96h0203	AV401081
e96h0205	AV401082
e96h0206	AV401083
e96h0209	AV401085
e96h0210	AV401086
e96h0211	AV401087
e96h0212	AV401088

e96h0213	AV401089
e96h0214	AV401090
e96h0215	AV401091
e96h0217	AV401092
e96h0218	AV401093
e96h0223	AV401095
e96h0224	AV401096
e96h0225	AV401097
e96h0226	AV401098
e96h0227	AV401099
e96h0228	AV401100
e96h0229	AV401101
e96h0231	AV401102
e96h0233	AV401103
e96h0234	AV401104
e96h0235	AV401105
e96h0240	AV401107
e96h0241	AV401108
e96h0243	AV401109
e96h0245	AV401111
e96h0247	AV401113
e96h0248	AV401114
e96h0249	AV401115
e96h0250	AV401116
e96h0251	AV401117
e96h0252	AV401118
e96h0253	AV401119
e96h0254	AV401120
e96h0255	AV401121
e96h0256	AV401122
e96h0257	AV401123
e96h0259	AV401124
e96h0260	AV401125
e96h0262	AV401126
e96h0263	AV401127
e96h0264	AV401128
e96h0265	AV401129
e96h0266	AV401130
e96h0267	AV401131
e96h0269	AV401132
e96h0270	AV401133
e96h0271	AV401134
e96h0272	AV401135
e96h0273	AV401136
e96h0274	AV401137
e96h0275	AV401138
e96h0276	AV401139
e96h0278	AV401140
e96h0279	AV401141
e96h0280	AV401142
e96h0282	AV401144
e96h0283	AV401145
e96h0284	AV401146
e96h0285	AV401147

e96h0286	AV401148
e96h0287	AV401149
e96h0289	AV401150
e96h0290	BY915271
e96h0292	AV401152
e96h0294	AV401153
e96h0295	AV401154
e96h0297	AV401155
e96h0298	AV401156
e96h0299	AV401157
e96h0300	AV401158
e96h0301	AV401159
e96h0302	AV401160
e96h0303	AV401161
e96h0304	AV401162
e96h0305	AV401163
e96h0306	AV401164
e96h0309	AV401166
e96h0310	AV401167
e96h0312	BY915272
e96h0314	AV401168
e96h0315	AV401169
e96h0316	AV401170
e96h0318	AV401171
e96h0321	BY915273
e96h0322	AV401172
e96h0323	AV401173
e96h0324	AV401174
e96h0325	AV401175
e96h0326	BY915274
e96h0327	AV401176
e96h0330	AV401177
e96h0332	AV401179
e96h0334	AV401180
e96h0336	AV401181
e96h0337	AV401182
e96h0338	AV401183
e96h0339	AV401184
e96h0340	AV401185
e96h0341	AV401186
e96h0342	AV401187
e96h0343	AV401188
e96h0345	AV401189
e96h0348	AV401190
e96h0349	AV401191
e96h0350	AV401192
e96h0353	AV401193
e96h0354	AV401194
e96h0356	AV401195
e96h0357	BY915275
e96h0359	AV401196
e96h0360	AV401197
e96h0361	AV401198
e96h0362	AV401199

e96h0364	AV401200
e96h0366	AV401201
e96h0367	AV401202
e96h0369	AV401203
e96h0370	AV401204
e96h0371	AV401205
e96h0372	AV401206
e96h0373	AV401207
e96h0375	AV401208
e96h0376	AV401209
e96h0380	AV401212
e96h0381	AV401213
e96h0383	AV401214
e96h0384_1	BY915276
e96h0386	AV401216
e96h0387	AV401217
e96h0388	AV401218
e96h0389	AV401219
e96h0391	AV401220
e96h0392	AV401221
e96h0393	AV401222
e96h0394	AV401223
e96h0395	AV401224
e96h0398	AV401225
e96h0399	AV401226
e96h0400	AV401227
e96h0402	AV401228
e96h0403	AV401229
e96h0404	AV401230
e96h0406	AV401231
e96h0407	AV401232
e96h0408	AV401233
e96h0409	AV401234
e96h0410	AV401235
e96h0411	AV401236
e96h0412	AV401237
e96h0413	AV401238
e96h0414	AV401239
e96h0416	AV401240
e96h0417	AV401241
e96h0418	AV401242
e96h0419	AV401243
e96h0421	AV401245
e96h0423	AV401246
e96h0424	AV401247
e96h0426	AV401248
e96h0429	AV401249
e96h0430	AV401250
e96h0431	AV401251
e96h0432	AV401252
e96h0434	AV401253
e96h0437	AV401254
e96h0438	AV401255
e96h0439	AV401256

e96h0440	AV401257
e96h0444	AV401258
e96h0445	AV401259
e96h0446	AV401260
e96h0447	AV401261
e96h0449	AV401262
e96h0450	AV401263
e96h0453	AV401264
e96h0454	AV401265
e96h0457	AV401267
e96h0458	AV401268
e96h0461	AV401270
e96h0462	AV401271
e96h0463	AV401272
e96h0465	AV401273
e96h0470	AV401275
e96h0471	AV401276
e96h0472	AV401277
e96h0473	AV401278
e96h0474	AV401279
e96h0475	AV401280
e96h0476	AV401281
e96h0477	AV401282
e96h0478	AV401283
e96h0479	AV401284
e96h0481	AV401285
e96h0482	AV401286
e96h0485	AV401288
e96h0488	AV401289
e96h0495	AV401291
e96h0500	AV401293
e96h0507	AV401295
e96h0509	AV401296
e96h0513	AV401297
e96h0514	AV401298
e96h0515	AV401299
e96h0516	AV401300
e96h0517	AV401301
e96h0518	AV401302
e96h0520	AV401303
e96h0521	AV401304
e96h0523	AV401305
e96h0525	AV401306
e96h0526	AV401307
e96h0528	AV401308
e96h0531	AV401309
e96h0533	AV401310
e96h0537	AV401311
e96h0539	AV401313
e96h0540	AV401314
e96h0541	AV401315
e96h0545	AV401316
e96h0546	AV401317
e96h0548	AV401318

e96h0549	AV401319
e96h0552	AV401320
e96h0553	AV401321
e96h0554	AV401322
e96h0555	AV401323
e96h0556	AV401324
e96h0557	AV401325
e96h0558	AV401326
e96h0559	AV401327
e96h0560	AV401328
e96h0562	AV401329
e96h0565	AV401330
e96h0566	AV401331
e96h0567	AV401332
e96h0568	AV401333
e96h0569	AV401334
e96h0570	AV401335
e96h0571	AV401336
e96h0572	AV401337
e96h0573	AV401338
e96h0574	AV401339
e96h0575	AV401340
e96h0576	AV401341
e96h0580	AV401342
e96h0581	AV401343
e96h0582	AV401344
e96h0584	AV401345
e96h0585	AV401346
e96h0587	AV401347
e96h0590	AV401348
e96h0591	AV401349
e96h0592	AV401350
e96h0593	AV401351
e96h0594	AV401352
e96h0595	AV401353
e96h0596	AV401354
e96h0597	AV401355
e96h0599	AV401357
e96h0600	AV401358
e96h0601	AV401359
e96h0602	AV401360
e96h0603	AV401361
e96h0604	AV401362
e96h0605	AV401363
e96h0606	AV401364
e96h0607	AV401365
e96h0608	AV401366
e96h0609	AV401367
e96h0610	AV401368
e96h0614	AV401371
e96h0615	AV401372
e96h0616	AV401373
e96h0618	AV401374
e96h0619	AV401375

e96h0620	AV401376
e96h0621	AV401377
e96h0622	AV401378
e96h0623	AV401379
e96h0625	AV401380
e96h0627	AV401381
e96h0628	AV401382
e96h0629	AV401383
e96h0630	AV401384
e96h0635	AV401387
e96h0636	AV401388
e96h0649	AV401390
e96h0650	AV401391
e96h0651	AV401392
e96h0652	AV401393
e96h0654	AV401394
e96h0661	AV401395
e96h0664	AV401397
e96h0666	AV401398
e96h0667	AV401399
e96h0668	AV401400
e96h0669	AV401401
e96h0670	AV401402
e96h0672	AV401403
e96h0675	AV401404
e96h0676	AV401405
e96h0677	AV401406
e96h0678	AV401407
e96h0679	AV401408
e96h0680	AV401409
e96h0681	AV401410
e96h0682	AV401411
e96h0683	AV401412
e96h0684	AV401413
e96h0685	AV401414
e96h0687	AV401415
e96h0689	AV401416
e96h0690	AV401417
e96h0691	AV401418
e96h0694	AV401419
e96h0697	AV401421
e96h0698	AV401422
e96h0706	AV401424
e96h0707	AV401425
e96h0708	AV401426
e96h0709	AV401427
e96h0710	AV401428
e96h0711	AV401429
e96h0713	AV401430
e96h0714	AV401431
e96h0715	AV401432
e96h0716	AV401433
e96h0718	AV401434
e96h0723	AV401435

e96h0726	AV401436
e96h0727	AV401437
e96h0729	AV401438
e96h0730	AV401439
e96h0731	AV401440
e96h0734	AV401441
e96h0736	AV401443
e96h0738	AV401444
e96h0740	AV401446
e96h0741	AV401447
e96h0743	AV401449
e96h0744	AV401450
e96h0746	AV401451
e96h0750	AV401452
e96h0752	AV401454
e96h0754	AV401455
e96h0755	AV401456
e96h0758	AV401457
e96h0759	AV401458
e96h0761	AV401459
e96h0763	AV401460
e96h0764	AV401461
e96h0765	AV401462
e96h0766	AV401463
e96h0767	AV401464
e96h0769	AV401466
e96h0770	AV401467
e96h0771	AV401468
e96h0772	AV401469
e96h0773	AV401470
e96h0774	AV401471
e96h0775	AV401472
e96h0777	AV401473
e96h0778	AV401474
e96h0781	AV401475
e96h0782	AV401476
e96h0783	AV401477
e96h0785	AV401478
e96h0786	AV401479
e96h0788	AV401481
e96h0789	AV401482
e96h0790	AV401483
e96h0791	AV401484
e96h0792	AV401485
e96h0793	AV401486
e96h0794	AV401487
e96h0795	AV401488
e96h0796	AV401489
e96h0797	AV401490
e96h0798	AV401491
e96h0799	AV401492
e96h0800	AV401493
e96h0802	AV401495
e96h0803	AV401496

e96h0804	AV401497
e96h0805	AV401498
e96h0806	AV401499
e96h0807	AV401500
e96h0808	AV401501
e96h0809	AV401502
e96h0810	AV401503
e96h0811	AV401504
e96h0812	AV401505
e96h0813	AV401506
e96h0814	AV401507
e96h0815	AV401508
e96h0816	AV401509
e96h0817	AV401510
e96h0818	AV401511
e96h0820	AV401512
e96h0821	AV401513
e96h0823	AV401515
e96h0824	AV401516
e96h0825	AV401517
e96h0826	AV401518
e96h0828	AV401520
e96h0829	AV401521
e96h0830	AV401522
e96h0831	AV401523
e96h0832	AV401524
e96h0833	AV401525
e96h0834	AV401526
e96h0836	AV401527
e96h0837	AV401528
e96h0839	AV401529
e96h0840	AV401530
e96h0841	AV401531
e96h0842	AV401532
e96h0843	AV401533
e96h0844	AV401534
e96h0846	AV401535
e96h0847	AV401536
e96h0848	AV401537
e96h0849	AV401538
e96h0850	AV401539
e96h0851	AV401540
e96h0852	AV401541
e96h0853	AV401542
e96h0855	AV401543
e96h0856	AV401544
e96h0858	AV401545
e96h0860	AV401546
e96h0861	AV401547
e96h0862	AV401548
e96h0863	AV401549
e96h0864	AV401550
e96h0865	AV401551
e96h0866	AV401552

e96h0867	AV401553
e96h0868	AV401554
e96h0869	AV401555
e96h0870	AV401556
e96h0871	AV401557
e96h0872	AV401558
e96h0873	AV401559
e96h0874	AV401560
e96h0875	AV401561
e96h0877	AV401562
e96h0878	AV401563
e96h0880	AV401565
e96h0881	AV401566
e96h0882	AV401567
e96h0883	AV401568
e96h0884	AV401569
e96h0887	AV401570
e96h0888	AV401571
e96h0890	AV401572
e96h0891	AV401573
e96h0892	AV401574
e96h0893	AV401575
e96h0894	AV401576
e96h0896	AV401578
e96h0897	AV401579
e96h0898	AV401580
e96h0902	AV401581
e96h0904	AV401582
e96h0905	AV401583
e96h0906	AV401584
e96h0908	AV401585
e96h0910	AV401586
e96h0911	AV401587
e96h0912	AV401588
e96h0913	AV401589
e96h0914	AV401590
e96h0916	AV401591
e96h0917	AV401592
e96h0918	AV401593
e96h0920	AV401595
e96h0921	AV401596
e96h0922	AV401597
e96h0924	AV401599
e96h0925	AV401600
e96h0926	AV401601
e96h0927	AV401602
e96h0928	AV401603
e96h0929	AV401604
e96h0930	AV401605
e96h0931	AV401606
e96h0934	AV401607
e96h0936	AV401609
e96h0937	AV401610
e96h0938	AV401611

	e96h0939	AV401612
	e96h0941	AV401614
	e96h0942	AV401615
	e96h0944	AV401617
	e96h0945	AV401618
	e96h0947	AV401619
	e96h0948	AV401620
	e96h0949	AV401621
	e96h0952	AV401622
	e96h0953	AV401623
	e96h0954	AV401624
	e96h0955	AV401625
	e96h0956	AV401626
	e96h0957	AV401627
	e96h0967	AV401628
	e96h0968	AV401629
	e96h0969	AV401630
	e96h0970	AV401631
	e96h0975	AV401632
	e96h0978	AV401634
	e96h0979	AV401635
	e96h0980	AV401636
	e96h0982	AV401638
	e96h0983	AV401639
	e96h0986	AV401640
	e96h0987	AV401641
	e96h0988	AV401642
	e96h0992	AV401644
	e96h0993	AV401645
	e96h0995	AV401646
	e96h1000	AV401647
fcP8	E_EL_fcP8_01A01_F_0	BY918786
	E_EL_fcP8_01A01_R_0	BY927072
	E_EL_fcP8_01A02_F_0	BY918787
	E_EL_fcP8_01A02_R_0	BY927073
	E_EL_fcP8_01A03_F_0	BY918788
	E_EL_fcP8_01A03_R_0	BY927074
	E_EL_fcP8_01A04_F_0	BY918789
	E_EL_fcP8_01A04_R_0	BY927075
	E_EL_fcP8_01A05_F_0	BY918790
	E_EL_fcP8_01A05_R_0	BY927076
	E_EL_fcP8_01A06_F_0	BY918791
	E_EL_fcP8_01A06_R_0	BY927077
	E_EL_fcP8_01A07_F_0	BY918792
	E_EL_fcP8_01A07_R_0	BY927078
	E_EL_fcP8_01A08_R_0	BY927079
	E_EL_fcP8_01A09_R_0	BY927080
	E_EL_fcP8_01A10_F_0	BY918793
	E_EL_fcP8_01A10_R_0	BY927081
	E_EL_fcP8_01A11_F_0	BY918794
	E_EL_fcP8_01A11_R_0	BY927082
	E_EL_fcP8_01B01_F_0	BY918795
	E_EL_fcP8_01B01_R_0	BY927083
	E_EL_fcP8_01B02_F_0	BY918796

E_EL_fcP8_01B03_R_0	BY927084
E_EL_fcP8_01B04_F_0	BY918797
E_EL_fcP8_01B04_R_0	BY927085
E_EL_fcP8_01B05_F_0	BY918798
E_EL_fcP8_01B05_R_0	BY927086
E_EL_fcP8_01B06_F_0	BY918799
E_EL_fcP8_01B06_R_0	BY927087
E_EL_fcP8_01B07_R_0	BY927088
E_EL_fcP8_01B09_F_0	BY918800
E_EL_fcP8_01B09_R_0	BY927089
E_EL_fcP8_01B10_F_0	BY918801
E_EL_fcP8_01B10_R_0	BY927090
E_EL_fcP8_01B11_R_0	BY927091
E_EL_fcP8_01B12_F_0	BY918802
E_EL_fcP8_01B12_R_0	BY927092
E_EL_fcP8_01C01_F_0	BY918803
E_EL_fcP8_01C01_R_0	BY927093
E_EL_fcP8_01C02_F_0	BY918804
E_EL_fcP8_01C03_F_0	BY918805
E_EL_fcP8_01C03_R_0	BY927094
E_EL_fcP8_01C04_F_0	BY918806
E_EL_fcP8_01C04_R_0	BY927095
E_EL_fcP8_01C05_F_0	BY918807
E_EL_fcP8_01C05_R_0	BY927096
E_EL_fcP8_01C06_F_0	BY918808
E_EL_fcP8_01C06_R_0	BY927097
E_EL_fcP8_01C07_F_0	BY918809
E_EL_fcP8_01C07_R_0	BY927098
E_EL_fcP8_01C08_F_0	BY918810
E_EL_fcP8_01C08_R_0	BY927099
E_EL_fcP8_01C09_F_0	BY918811
E_EL_fcP8_01C09_R_0	BY927100
E_EL_fcP8_01C10_F_0	BY918812
E_EL_fcP8_01C10_R_0	BY927101
E_EL_fcP8_01C11_F_0	BY918813
E_EL_fcP8_01C11_R_0	BY927102
E_EL_fcP8_01C12_R_0	BY927103
E_EL_fcP8_01D01_F_0	BY918814
E_EL_fcP8_01D01_R_0	BY927104
E_EL_fcP8_01D02_F_0	BY918815
E_EL_fcP8_01D02_R_0	BY927105
E_EL_fcP8_01D03_F_0	BY918816
E_EL_fcP8_01D03_R_0	BY927106
E_EL_fcP8_01D04_F_0	BY918817
E_EL_fcP8_01D04_R_0	BY927107
E_EL_fcP8_01D05_F_0	BY918818
E_EL_fcP8_01D05_R_0	BY927108
E_EL_fcP8_01D06_F_0	BY918819
E_EL_fcP8_01D06_R_0	BY927109
E_EL_fcP8_01D07_F_0	BY918820
E_EL_fcP8_01D07_R_0	BY927110
E_EL_fcP8_01D08_F_0	BY918821
E_EL_fcP8_01D08_R_0	BY927111
E_EL_fcP8_01D09_F_0	BY918822

E_EL_fcP8_01D09_R_0	BY927112
E_EL_fcP8_01D10_F_0	BY918823
E_EL_fcP8_01D10_R_0	BY927113
E_EL_fcP8_01D11_F_0	BY918824
E_EL_fcP8_01D11_R_0	BY927114
E_EL_fcP8_01D12_R_0	BY927115
E_EL_fcP8_01E01_R_0	BY927116
E_EL_fcP8_01E02_F_0	BY918825
E_EL_fcP8_01E02_R_0	BY927117
E_EL_fcP8_01E03_R_0	BY927118
E_EL_fcP8_01E04_F_0	BY918826
E_EL_fcP8_01E04_R_0	BY927119
E_EL_fcP8_01E05_F_0	BY918827
E_EL_fcP8_01E05_R_0	BY927120
E_EL_fcP8_01E06_F_0	BY918828
E_EL_fcP8_01E06_R_0	BY927121
E_EL_fcP8_01E07_R_0	BY927122
E_EL_fcP8_01E08_F_0	BY918829
E_EL_fcP8_01E08_R_0	BY927123
E_EL_fcP8_01E09_F_0	BY918830
E_EL_fcP8_01E09_R_0	BY927124
E_EL_fcP8_01E10_F_0	BY918831
E_EL_fcP8_01E10_R_0	BY927125
E_EL_fcP8_01E11_F_0	BY918832
E_EL_fcP8_01E11_R_0	BY927126
E_EL_fcP8_01E12_F_0	BY918833
E_EL_fcP8_01E12_R_0	BY927127
E_EL_fcP8_01F01_F_0	BY918834
E_EL_fcP8_01F01_R_0	BY927128
E_EL_fcP8_01F02_F_0	BY918835
E_EL_fcP8_01F02_R_0	BY927129
E_EL_fcP8_01F03_F_0	BY918836
E_EL_fcP8_01F03_R_0	BY927130
E_EL_fcP8_01F04_F_0	BY918837
E_EL_fcP8_01F04_R_0	BY927131
E_EL_fcP8_01F05_F_0	BY918838
E_EL_fcP8_01F05_R_0	BY927132
E_EL_fcP8_01F06_F_0	BY918839
E_EL_fcP8_01F06_R_0	BY927133
E_EL_fcP8_01F07_F_0	BY918840
E_EL_fcP8_01F07_R_0	BY927134
E_EL_fcP8_01F08_F_0	BY918841
E_EL_fcP8_01F08_R_0	BY927135
E_EL_fcP8_01F09_F_0	BY918842
E_EL_fcP8_01F09_R_0	BY927136
E_EL_fcP8_01F10_F_0	BY918843
E_EL_fcP8_01F10_R_0	BY927137
E_EL_fcP8_01F11_R_0	BY927138
E_EL_fcP8_01F12_F_0	BY918844
E_EL_fcP8_01F12_R_0	BY927139
E_EL_fcP8_01G01_F_0	BY918845
E_EL_fcP8_01G01_R_0	BY927140
E_EL_fcP8_01G02_F_0	BY918846
E_EL_fcP8_01G02_R_0	BY927141

E_EL_fcP8_01G03_F_0	BY918847
E_EL_fcP8_01G03_R_0	BY927142
E_EL_fcP8_01G04_F_0	BY918848
E_EL_fcP8_01G04_R_0	BY927143
E_EL_fcP8_01G05_F_0	BY918849
E_EL_fcP8_01G05_R_0	BY927144
E_EL_fcP8_01G06_F_0	BY918850
E_EL_fcP8_01G06_R_0	BY927145
E_EL_fcP8_01G07_R_0	BY927146
E_EL_fcP8_01G08_F_0	BY918851
E_EL_fcP8_01G08_R_0	BY927147
E_EL_fcP8_01G09_F_0	BY918852
E_EL_fcP8_01G09_R_0	BY927148
E_EL_fcP8_01G10_R_0	BY927149
E_EL_fcP8_01G11_F_0	BY918853
E_EL_fcP8_01G11_R_0	BY927150
E_EL_fcP8_01H01_F_0	BY918854
E_EL_fcP8_01H01_R_0	BY927151
E_EL_fcP8_01H02_F_0	BY918855
E_EL_fcP8_01H02_R_0	BY927152
E_EL_fcP8_01H03_R_0	BY927153
E_EL_fcP8_01H04_F_0	BY918856
E_EL_fcP8_01H04_R_0	BY927154
E_EL_fcP8_01H05_F_0	BY918857
E_EL_fcP8_01H05_R_0	BY927155
E_EL_fcP8_01H06_F_0	BY918858
E_EL_fcP8_01H06_R_0	BY927156
E_EL_fcP8_01H08_F_0	BY918859
E_EL_fcP8_01H08_R_0	BY927157
E_EL_fcP8_01H09_F_0	BY918860
E_EL_fcP8_01H09_R_0	BY927158
E_EL_fcP8_01H10_F_0	BY918861
E_EL_fcP8_01H10_R_0	BY927159
E_EL_fcP8_01H11_F_0	BY918862
E_EL_fcP8_01H11_R_0	BY927160
E_EL_fcP8_01H12_F_0	BY918863
E_EL_fcP8_01H12_R_0	BY927161
E_EL_fcP8_02A01_F_0	BY918864
E_EL_fcP8_02A01_R_0	BY927162
E_EL_fcP8_02A02_F_0	BY918865
E_EL_fcP8_02A02_R_0	BY927163
E_EL_fcP8_02A03_F_0	BY918866
E_EL_fcP8_02A03_R_0	BY927164
E_EL_fcP8_02A04_F_0	BY918867
E_EL_fcP8_02A04_R_0	BY927165
E_EL_fcP8_02A05_F_0	BY918868
E_EL_fcP8_02A05_R_0	BY927166
E_EL_fcP8_02A06_F_0	BY918869
E_EL_fcP8_02A06_R_0	BY927167
E_EL_fcP8_02A07_F_0	BY918870
E_EL_fcP8_02A07_R_0	BY927168
E_EL_fcP8_02A08_F_0	BY918871
E_EL_fcP8_02A08_R_0	BY927169
E_EL_fcP8_02A09_F_0	BY918872

E_EL_fcP8_02A09_R_0	BY927170
E_EL_fcP8_02A10_F_0	BY918873
E_EL_fcP8_02A10_R_0	BY927171
E_EL_fcP8_02A11_F_0	BY918874
E_EL_fcP8_02A11_R_0	BY927172
E_EL_fcP8_02A12_F_0	BY918875
E_EL_fcP8_02A12_R_0	BY927173
E_EL_fcP8_02B01_F_0	BY918876
E_EL_fcP8_02B01_R_0	BY927174
E_EL_fcP8_02B02_F_0	BY918877
E_EL_fcP8_02B02_R_0	BY927175
E_EL_fcP8_02B04_F_0	BY918878
E_EL_fcP8_02B04_R_0	BY927176
E_EL_fcP8_02B05_R_0	BY927177
E_EL_fcP8_02B06_F_0	BY918879
E_EL_fcP8_02B06_R_0	BY927178
E_EL_fcP8_02B07_F_0	BY918880
E_EL_fcP8_02B07_R_0	BY927179
E_EL_fcP8_02B08_F_0	BY918881
E_EL_fcP8_02B08_R_0	BY927180
E_EL_fcP8_02B09_F_0	BY918882
E_EL_fcP8_02B09_R_0	BY927181
E_EL_fcP8_02B10_F_0	BY918883
E_EL_fcP8_02B10_R_0	BY927182
E_EL_fcP8_02B11_F_0	BY918884
E_EL_fcP8_02B12_R_0	BY927183
E_EL_fcP8_02C01_F_0	BY918885
E_EL_fcP8_02C01_R_0	BY927184
E_EL_fcP8_02C03_F_0	BY918886
E_EL_fcP8_02C03_R_0	BY927185
E_EL_fcP8_02C04_F_0	BY918887
E_EL_fcP8_02C04_R_0	BY927186
E_EL_fcP8_02C05_F_0	BY918888
E_EL_fcP8_02C05_R_0	BY927187
E_EL_fcP8_02C06_F_0	BY918889
E_EL_fcP8_02C06_R_0	BY927188
E_EL_fcP8_02C07_R_0	BY927189
E_EL_fcP8_02C08_F_0	BY918890
E_EL_fcP8_02C08_R_0	BY927190
E_EL_fcP8_02C09_F_0	BY918891
E_EL_fcP8_02C09_R_0	BY927191
E_EL_fcP8_02C10_F_0	BY918892
E_EL_fcP8_02C10_R_0	BY927192
E_EL_fcP8_02C11_R_0	BY927193
E_EL_fcP8_02C12_F_0	BY918893
E_EL_fcP8_02C12_R_0	BY927194
E_EL_fcP8_02D01_F_0	BY918894
E_EL_fcP8_02D01_R_0	BY927195
E_EL_fcP8_02D02_R_0	BY927196
E_EL_fcP8_02D04_F_0	BY918895
E_EL_fcP8_02D04_R_0	BY927197
E_EL_fcP8_02D05_R_0	BY927198
E_EL_fcP8_02D06_F_0	BY918896
E_EL_fcP8_02D06_R_0	BY927199

E_EL_fcP8_02D07_F_0	BY918897
E_EL_fcP8_02D07_R_0	BY927200
E_EL_fcP8_02D08_F_0	BY918898
E_EL_fcP8_02D08_R_0	BY927201
E_EL_fcP8_02D09_F_0	BY918899
E_EL_fcP8_02D09_R_0	BY927202
E_EL_fcP8_02D10_F_0	BY918900
E_EL_fcP8_02D10_R_0	BY927203
E_EL_fcP8_02D11_F_0	BY918901
E_EL_fcP8_02D12_F_0	BY918902
E_EL_fcP8_02D12_R_0	BY927204
E_EL_fcP8_02E01_R_0	BY927205
E_EL_fcP8_02E02_F_0	BY918903
E_EL_fcP8_02E02_R_0	BY927206
E_EL_fcP8_02E03_F_0	BY918904
E_EL_fcP8_02E03_R_0	BY927207
E_EL_fcP8_02E04_F_0	BY918905
E_EL_fcP8_02E04_R_0	BY927208
E_EL_fcP8_02E05_R_0	BY927209
E_EL_fcP8_02E06_F_0	BY918906
E_EL_fcP8_02E06_R_0	BY927210
E_EL_fcP8_02E07_F_0	BY918907
E_EL_fcP8_02E07_R_0	BY927211
E_EL_fcP8_02E08_F_0	BY918908
E_EL_fcP8_02E08_R_0	BY927212
E_EL_fcP8_02E09_F_0	BY918909
E_EL_fcP8_02E09_R_0	BY927213
E_EL_fcP8_02E10_F_0	BY918910
E_EL_fcP8_02E11_F_0	BY918911
E_EL_fcP8_02E11_R_0	BY927214
E_EL_fcP8_02E12_F_0	BY918912
E_EL_fcP8_02E12_R_0	BY927215
E_EL_fcP8_02F01_F_0	BY918913
E_EL_fcP8_02F01_R_0	BY927216
E_EL_fcP8_02F03_F_0	BY918914
E_EL_fcP8_02F03_R_0	BY927217
E_EL_fcP8_02F04_F_0	BY918915
E_EL_fcP8_02F04_R_0	BY927218
E_EL_fcP8_02F05_R_0	BY927219
E_EL_fcP8_02F06_F_0	BY918916
E_EL_fcP8_02F06_R_0	BY927220
E_EL_fcP8_02F07_F_0	BY918917
E_EL_fcP8_02F07_R_0	BY927221
E_EL_fcP8_02F08_F_0	BY918918
E_EL_fcP8_02F08_R_0	BY927222
E_EL_fcP8_02F09_F_0	BY918919
E_EL_fcP8_02F09_R_0	BY927223
E_EL_fcP8_02F10_F_0	BY918920
E_EL_fcP8_02F10_R_0	BY927224
E_EL_fcP8_02F11_F_0	BY918921
E_EL_fcP8_02F11_R_0	BY927225
E_EL_fcP8_02F12_F_0	BY918922
E_EL_fcP8_02F12_R_0	BY927226
E_EL_fcP8_02G01_F_0	BY918923

E_EL_fcP8_02G01_R_0	BY927227
E_EL_fcP8_02G02_F_0	BY918924
E_EL_fcP8_02G02_R_0	BY927228
E_EL_fcP8_02G03_F_0	BY918925
E_EL_fcP8_02G03_R_0	BY927229
E_EL_fcP8_02G04_F_0	BY918926
E_EL_fcP8_02G04_R_0	BY927230
E_EL_fcP8_02G05_R_0	BY927231
E_EL_fcP8_02G06_R_0	BY927232
E_EL_fcP8_02G07_F_0	BY918927
E_EL_fcP8_02G07_R_0	BY927233
E_EL_fcP8_02G08_F_0	BY918928
E_EL_fcP8_02G08_R_0	BY927234
E_EL_fcP8_02G09_F_0	BY918929
E_EL_fcP8_02G09_R_0	BY927235
E_EL_fcP8_02G10_F_0	BY918930
E_EL_fcP8_02G10_R_0	BY927236
E_EL_fcP8_02G11_R_0	BY927237
E_EL_fcP8_02G12_F_0	BY918931
E_EL_fcP8_02G12_R_0	BY927238
E_EL_fcP8_02H01_F_0	BY918932
E_EL_fcP8_02H01_R_0	BY927239
E_EL_fcP8_02H02_F_0	BY918933
E_EL_fcP8_02H02_R_0	BY927240
E_EL_fcP8_02H04_F_0	BY918934
E_EL_fcP8_02H04_R_0	BY927241
E_EL_fcP8_02H05_F_0	BY918935
E_EL_fcP8_02H05_R_0	BY927242
E_EL_fcP8_02H06_F_0	BY918936
E_EL_fcP8_02H06_R_0	BY927243
E_EL_fcP8_02H07_F_0	BY918937
E_EL_fcP8_02H07_R_0	BY927244
E_EL_fcP8_02H08_F_0	BY918938
E_EL_fcP8_02H08_R_0	BY927245
E_EL_fcP8_02H09_F_0	BY918939
E_EL_fcP8_02H09_R_0	BY927246
E_EL_fcP8_02H10_F_0	BY918940
E_EL_fcP8_02H10_R_0	BY927247
E_EL_fcP8_02H12_F_0	BY918941
E_EL_fcP8_02H12_R_0	BY927248
E_EL_fcP8_03A01_R_0	BY927249
E_EL_fcP8_03A02_F_0	BY918942
E_EL_fcP8_03A02_R_0	BY927250
E_EL_fcP8_03A03_F_0	BY918943
E_EL_fcP8_03A03_R_0	BY927251
E_EL_fcP8_03A04_F_0	BY918944
E_EL_fcP8_03A04_R_0	BY927252
E_EL_fcP8_03A06_F_0	BY918945
E_EL_fcP8_03A06_R_0	BY927253
E_EL_fcP8_03A07_F_0	BY918946
E_EL_fcP8_03A07_R_0	BY927254
E_EL_fcP8_03A08_F_0	BY918947
E_EL_fcP8_03A08_R_0	BY927255
E_EL_fcP8_03A10_R_0	BY927256

E_EL_fcP8_03A11_F_0	BY918948
E_EL_fcP8_03A11_R_0	BY927257
E_EL_fcP8_03A12_F_0	BY918949
E_EL_fcP8_03A12_R_0	BY927258
E_EL_fcP8_03B01_F_0	BY918950
E_EL_fcP8_03B01_R_0	BY927259
E_EL_fcP8_03B02_F_0	BY918951
E_EL_fcP8_03B02_R_0	BY927260
E_EL_fcP8_03B03_R_0	BY927261
E_EL_fcP8_03B04_F_0	BY918952
E_EL_fcP8_03B04_R_0	BY927262
E_EL_fcP8_03B05_F_0	BY918953
E_EL_fcP8_03B05_R_0	BY927263
E_EL_fcP8_03B07_F_0	BY918954
E_EL_fcP8_03B07_R_0	BY927264
E_EL_fcP8_03B08_F_0	BY918955
E_EL_fcP8_03B08_R_0	BY927265
E_EL_fcP8_03B09_F_0	BY918956
E_EL_fcP8_03B09_R_0	BY927266
E_EL_fcP8_03B10_F_0	BY918957
E_EL_fcP8_03B10_R_0	BY927267
E_EL_fcP8_03B11_F_0	BY918958
E_EL_fcP8_03B11_R_0	BY927268
E_EL_fcP8_03B12_R_0	BY927269
E_EL_fcP8_03C01_R_0	BY927270
E_EL_fcP8_03C02_F_0	BY918959
E_EL_fcP8_03C02_R_0	BY927271
E_EL_fcP8_03C03_F_0	BY918960
E_EL_fcP8_03C04_F_0	BY918961
E_EL_fcP8_03C04_R_0	BY927272
E_EL_fcP8_03C05_F_0	BY918962
E_EL_fcP8_03C05_R_0	BY927273
E_EL_fcP8_03C06_F_0	BY918963
E_EL_fcP8_03C06_R_0	BY927274
E_EL_fcP8_03C07_F_0	BY918964
E_EL_fcP8_03C07_R_0	BY927275
E_EL_fcP8_03C08_F_0	BY918965
E_EL_fcP8_03C08_R_0	BY927276
E_EL_fcP8_03C09_R_0	BY927277
E_EL_fcP8_03C10_F_0	BY918966
E_EL_fcP8_03C10_R_0	BY927278
E_EL_fcP8_03C11_F_0	BY918967
E_EL_fcP8_03C11_R_0	BY927279
E_EL_fcP8_03C12_F_0	BY918968
E_EL_fcP8_03C12_R_0	BY927280
E_EL_fcP8_03D01_F_0	BY918969
E_EL_fcP8_03D01_R_0	BY927281
E_EL_fcP8_03D03_F_0	BY918970
E_EL_fcP8_03D03_R_0	BY927282
E_EL_fcP8_03D05_F_0	BY918971
E_EL_fcP8_03D05_R_0	BY927283
E_EL_fcP8_03D06_R_0	BY927284
E_EL_fcP8_03D07_F_0	BY918972
E_EL_fcP8_03D07_R_0	BY927285

E_EL_fcP8_03D08_F_0	BY918973
E_EL_fcP8_03D08_R_0	BY927286
E_EL_fcP8_03D09_F_0	BY918974
E_EL_fcP8_03D09_R_0	BY927287
E_EL_fcP8_03D10_F_0	BY918975
E_EL_fcP8_03D10_R_0	BY927288
E_EL_fcP8_03D11_F_0	BY918976
E_EL_fcP8_03D11_R_0	BY927289
E_EL_fcP8_03D12_F_0	BY918977
E_EL_fcP8_03D12_R_0	BY927290
E_EL_fcP8_03E02_F_0	BY918978
E_EL_fcP8_03E02_R_0	BY927291
E_EL_fcP8_03E03_F_0	BY918979
E_EL_fcP8_03E04_F_0	BY918980
E_EL_fcP8_03E04_R_0	BY927292
E_EL_fcP8_03E05_F_0	BY918981
E_EL_fcP8_03E05_R_0	BY927293
E_EL_fcP8_03E06_R_0	BY927294
E_EL_fcP8_03E07_F_0	BY918982
E_EL_fcP8_03E08_F_0	BY918983
E_EL_fcP8_03E08_R_0	BY927295
E_EL_fcP8_03E10_F_0	BY918984
E_EL_fcP8_03E10_R_0	BY927296
E_EL_fcP8_03E11_F_0	BY918985
E_EL_fcP8_03E11_R_0	BY927297
E_EL_fcP8_03E12_F_0	BY918986
E_EL_fcP8_03E12_R_0	BY927298
E_EL_fcP8_03F01_F_0	BY918987
E_EL_fcP8_03F01_R_0	BY927299
E_EL_fcP8_03F02_F_0	BY918988
E_EL_fcP8_03F02_R_0	BY927300
E_EL_fcP8_03F03_F_0	BY918989
E_EL_fcP8_03F03_R_0	BY927301
E_EL_fcP8_03F04_F_0	BY918990
E_EL_fcP8_03F05_F_0	BY918991
E_EL_fcP8_03F05_R_0	BY927302
E_EL_fcP8_03F06_R_0	BY927303
E_EL_fcP8_03F07_F_0	BY918992
E_EL_fcP8_03F07_R_0	BY927304
E_EL_fcP8_03F08_F_0	BY918993
E_EL_fcP8_03F08_R_0	BY927305
E_EL_fcP8_03F09_R_0	BY927306
E_EL_fcP8_03F10_F_0	BY918994
E_EL_fcP8_03F10_R_0	BY927307
E_EL_fcP8_03F11_F_0	BY918995
E_EL_fcP8_03F11_R_0	BY927308
E_EL_fcP8_03F12_F_0	BY918996
E_EL_fcP8_03F12_R_0	BY927309
E_EL_fcP8_03G01_F_0	BY918997
E_EL_fcP8_03G01_R_0	BY927310
E_EL_fcP8_03G02_R_0	BY927311
E_EL_fcP8_03G03_F_0	BY918998
E_EL_fcP8_03G03_R_0	BY927312
E_EL_fcP8_03G04_F_0	BY918999

E_EL_fcP8_03G04_R_0	BY927313
E_EL_fcP8_03G05_R_0	BY927314
E_EL_fcP8_03G06_F_0	BY919000
E_EL_fcP8_03G08_F_0	BY919001
E_EL_fcP8_03G08_R_0	BY927315
E_EL_fcP8_03G09_F_0	BY919002
E_EL_fcP8_03G09_R_0	BY927316
E_EL_fcP8_03G10_F_0	BY919003
E_EL_fcP8_03G10_R_0	BY927317
E_EL_fcP8_03G11_R_0	BY927318
E_EL_fcP8_03G12_F_0	BY919004
E_EL_fcP8_03H01_F_0	BY919005
E_EL_fcP8_03H01_R_0	BY927319
E_EL_fcP8_03H02_F_0	BY919006
E_EL_fcP8_03H02_R_0	BY927320
E_EL_fcP8_03H04_F_0	BY919007
E_EL_fcP8_03H04_R_0	BY927321
E_EL_fcP8_03H05_F_0	BY919008
E_EL_fcP8_03H05_R_0	BY927322
E_EL_fcP8_03H06_F_0	BY919009
E_EL_fcP8_03H06_R_0	BY927323
E_EL_fcP8_03H08_F_0	BY919010
E_EL_fcP8_03H08_R_0	BY927324
E_EL_fcP8_03H09_F_0	BY919011
E_EL_fcP8_03H09_R_0	BY927325
E_EL_fcP8_03H10_R_0	BY927326
E_EL_fcP8_03H11_F_0	BY919012
E_EL_fcP8_03H11_R_0	BY927327
E_EL_fcP8_03H12_R_0	BY927328
E_EL_fcP8_04A02_R_0	BY927329
E_EL_fcP8_04A03_F_0	BY919013
E_EL_fcP8_04A03_R_0	BY927330
E_EL_fcP8_04A05_F_0	BY919014
E_EL_fcP8_04A05_R_0	BY927331
E_EL_fcP8_04A06_F_0	BY919015
E_EL_fcP8_04A06_R_0	BY927332
E_EL_fcP8_04A07_F_0	BY919016
E_EL_fcP8_04A07_R_0	BY927333
E_EL_fcP8_04A08_R_0	BY927334
E_EL_fcP8_04A09_F_0	BY919017
E_EL_fcP8_04A09_R_0	BY927335
E_EL_fcP8_04A10_F_0	BY919018
E_EL_fcP8_04A10_R_0	BY927336
E_EL_fcP8_04A11_F_0	BY919019
E_EL_fcP8_04A11_R_0	BY927337
E_EL_fcP8_04A12_R_0	BY927338
E_EL_fcP8_04B03_F_0	BY919020
E_EL_fcP8_04B04_F_0	BY919021
E_EL_fcP8_04B04_R_0	BY927339
E_EL_fcP8_04B05_F_0	BY919022
E_EL_fcP8_04B05_R_0	BY927340
E_EL_fcP8_04B06_F_0	BY919023
E_EL_fcP8_04B06_R_0	BY927341
E_EL_fcP8_04B07_F_0	BY919024

E_EL_fcP8_04B07_R_0	BY927342
E_EL_fcP8_04B08_F_0	BY919025
E_EL_fcP8_04B08_R_0	BY927343
E_EL_fcP8_04B09_F_0	BY919026
E_EL_fcP8_04B09_R_0	BY927344
E_EL_fcP8_04B10_R_0	BY927345
E_EL_fcP8_04B11_F_0	BY919027
E_EL_fcP8_04B11_R_0	BY927346
E_EL_fcP8_04B12_F_0	BY919028
E_EL_fcP8_04B12_R_0	BY927347
E_EL_fcP8_04C03_F_0	BY919029
E_EL_fcP8_04C04_F_0	BY919030
E_EL_fcP8_04C04_R_0	BY927348
E_EL_fcP8_04C05_F_0	BY919031
E_EL_fcP8_04C05_R_0	BY927349
E_EL_fcP8_04C07_F_0	BY919032
E_EL_fcP8_04C07_R_0	BY927350
E_EL_fcP8_04C08_F_0	BY919033
E_EL_fcP8_04C08_R_0	BY927351
E_EL_fcP8_04C09_R_0	BY927352
E_EL_fcP8_04C10_F_0	BY919034
E_EL_fcP8_04C10_R_0	BY927353
E_EL_fcP8_04C11_F_0	BY919035
E_EL_fcP8_04C12_F_0	BY919036
E_EL_fcP8_04C12_R_0	BY927354
E_EL_fcP8_04D04_F_0	BY919037
E_EL_fcP8_04D04_R_0	BY927355
E_EL_fcP8_04D05_F_0	BY919038
E_EL_fcP8_04D06_F_0	BY919039
E_EL_fcP8_04D06_R_0	BY927356
E_EL_fcP8_04D07_F_0	BY919040
E_EL_fcP8_04D07_R_0	BY927357
E_EL_fcP8_04D08_F_0	BY919041
E_EL_fcP8_04D08_R_0	BY927358
E_EL_fcP8_04D10_F_0	BY919042
E_EL_fcP8_04D10_R_0	BY927359
E_EL_fcP8_04D11_F_0	BY919043
E_EL_fcP8_04D11_R_0	BY927360
E_EL_fcP8_04D12_F_0	BY919044
E_EL_fcP8_04D12_R_0	BY927361
E_EL_fcP8_04E02_F_0	BY919045
E_EL_fcP8_04E03_R_0	BY927362
E_EL_fcP8_04E05_F_0	BY919046
E_EL_fcP8_04E05_R_0	BY927363
E_EL_fcP8_04E06_F_0	BY919047
E_EL_fcP8_04E06_R_0	BY927364
E_EL_fcP8_04E07_F_0	BY919048
E_EL_fcP8_04E07_R_0	BY927365
E_EL_fcP8_04E08_R_0	BY927366
E_EL_fcP8_04E09_F_0	BY919049
E_EL_fcP8_04E09_R_0	BY927367
E_EL_fcP8_04E10_F_0	BY919050
E_EL_fcP8_04E10_R_0	BY927368
E_EL_fcP8_04E11_R_0	BY927369

E_EL_fcP8_04E12_F_0	BY919051
E_EL_fcP8_04F02_F_0	BY919052
E_EL_fcP8_04F03_F_0	BY919053
E_EL_fcP8_04F03_R_0	BY927370
E_EL_fcP8_04F04_F_0	BY919054
E_EL_fcP8_04F04_R_0	BY927371
E_EL_fcP8_04F05_F_0	BY919055
E_EL_fcP8_04F05_R_0	BY927372
E_EL_fcP8_04F06_F_0	BY919056
E_EL_fcP8_04F06_R_0	BY927373
E_EL_fcP8_04F07_F_0	BY919057
E_EL_fcP8_04F07_R_0	BY927374
E_EL_fcP8_04F08_F_0	BY919058
E_EL_fcP8_04F08_R_0	BY927375
E_EL_fcP8_04F09_F_0	BY919059
E_EL_fcP8_04F11_F_0	BY919060
E_EL_fcP8_04F12_R_0	BY927376
E_EL_fcP8_04G03_F_0	BY919061
E_EL_fcP8_04G03_R_0	BY927377
E_EL_fcP8_04G04_F_0	BY919062
E_EL_fcP8_04G04_R_0	BY927378
E_EL_fcP8_04G05_F_0	BY919063
E_EL_fcP8_04G05_R_0	BY927379
E_EL_fcP8_04G06_R_0	BY927380
E_EL_fcP8_04G07_F_0	BY919064
E_EL_fcP8_04G07_R_0	BY927381
E_EL_fcP8_04G08_F_0	BY919065
E_EL_fcP8_04G09_F_0	BY919066
E_EL_fcP8_04G09_R_0	BY927382
E_EL_fcP8_04G10_F_0	BY919067
E_EL_fcP8_04G10_R_0	BY927383
E_EL_fcP8_04G11_F_0	BY919068
E_EL_fcP8_04G11_R_0	BY927384
E_EL_fcP8_04G12_F_0	BY919069
E_EL_fcP8_04G12_R_0	BY927385
E_EL_fcP8_04H04_F_0	BY919070
E_EL_fcP8_04H04_R_0	BY927386
E_EL_fcP8_04H05_R_0	BY927387
E_EL_fcP8_04H06_F_0	BY919071
E_EL_fcP8_04H06_R_0	BY927388
E_EL_fcP8_04H07_F_0	BY919072
E_EL_fcP8_04H07_R_0	BY927389
E_EL_fcP8_04H08_F_0	BY919073
E_EL_fcP8_04H08_R_0	BY927390
E_EL_fcP8_04H11_F_0	BY919074
E_EL_fcP8_04H11_R_0	BY927391
E_EL_fcP8_04H12_F_0	BY919075
E_EL_fcP8_04H12_R_0	BY927392
E_EL_fcP8_05A01_F_0	BY919076
E_EL_fcP8_05A01_R_0	BY927393
E_EL_fcP8_05A03_R_0	BY927394
E_EL_fcP8_05A04_F_0	BY919077
E_EL_fcP8_05A04_R_0	BY927395
E_EL_fcP8_05A05_F_0	BY919078

E_EL_fcP8_05A05_R_0	BY927396
E_EL_fcP8_05A06_F_0	BY919079
E_EL_fcP8_05A06_R_0	BY927397
E_EL_fcP8_05A07_F_0	BY919080
E_EL_fcP8_05A07_R_0	BY927398
E_EL_fcP8_05A08_F_0	BY919081
E_EL_fcP8_05A08_R_0	BY927399
E_EL_fcP8_05A09_F_0	BY919082
E_EL_fcP8_05A09_R_0	BY927400
E_EL_fcP8_05A10_F_0	BY919083
E_EL_fcP8_05A10_R_0	BY927401
E_EL_fcP8_05A11_R_0	BY927402
E_EL_fcP8_05A12_F_0	BY919084
E_EL_fcP8_05A12_R_0	BY927403
E_EL_fcP8_05B01_F_0	BY919085
E_EL_fcP8_05B01_R_0	BY927404
E_EL_fcP8_05B02_R_0	BY927405
E_EL_fcP8_05B03_F_0	BY919086
E_EL_fcP8_05B03_R_0	BY927406
E_EL_fcP8_05B04_F_0	BY919087
E_EL_fcP8_05B04_R_0	BY927407
E_EL_fcP8_05B05_F_0	BY919088
E_EL_fcP8_05B05_R_0	BY927408
E_EL_fcP8_05B06_F_0	BY919089
E_EL_fcP8_05B06_R_0	BY927409
E_EL_fcP8_05B07_F_0	BY919090
E_EL_fcP8_05B07_R_0	BY927410
E_EL_fcP8_05B08_F_0	BY919091
E_EL_fcP8_05B08_R_0	BY927411
E_EL_fcP8_05B09_F_0	BY919092
E_EL_fcP8_05B09_R_0	BY927412
E_EL_fcP8_05B10_F_0	BY919093
E_EL_fcP8_05B10_R_0	BY927413
E_EL_fcP8_05B11_F_0	BY919094
E_EL_fcP8_05B11_R_0	BY927414
E_EL_fcP8_05B12_F_0	BY919095
E_EL_fcP8_05B12_R_0	BY927415
E_EL_fcP8_05C02_F_0	BY919096
E_EL_fcP8_05C02_R_0	BY927416
E_EL_fcP8_05C03_F_0	BY919097
E_EL_fcP8_05C04_F_0	BY919098
E_EL_fcP8_05C04_R_0	BY927417
E_EL_fcP8_05C05_F_0	BY919099
E_EL_fcP8_05C05_R_0	BY927418
E_EL_fcP8_05C06_F_0	BY919100
E_EL_fcP8_05C06_R_0	BY927419
E_EL_fcP8_05C07_F_0	BY919101
E_EL_fcP8_05C07_R_0	BY927420
E_EL_fcP8_05C08_F_0	BY919102
E_EL_fcP8_05C08_R_0	BY927421
E_EL_fcP8_05C09_F_0	BY919103
E_EL_fcP8_05C09_R_0	BY927422
E_EL_fcP8_05C11_F_0	BY919104
E_EL_fcP8_05C11_R_0	BY927423

E_EL_fcP8_05C12_F_0	BY919105
E_EL_fcP8_05C12_R_0	BY927424
E_EL_fcP8_05D01_R_0	BY927425
E_EL_fcP8_05D02_R_0	BY927426
E_EL_fcP8_05D03_F_0	BY919106
E_EL_fcP8_05D03_R_0	BY927427
E_EL_fcP8_05D04_F_0	BY919107
E_EL_fcP8_05D04_R_0	BY927428
E_EL_fcP8_05D05_R_0	BY927429
E_EL_fcP8_05D06_R_0	BY927430
E_EL_fcP8_05D08_F_0	BY919108
E_EL_fcP8_05D08_R_0	BY927431
E_EL_fcP8_05D09_F_0	BY919109
E_EL_fcP8_05D09_R_0	BY927432
E_EL_fcP8_05D10_R_0	BY927433
E_EL_fcP8_05D11_F_0	BY919110
E_EL_fcP8_05D11_R_0	BY927434
E_EL_fcP8_05D12_F_0	BY919111
E_EL_fcP8_05D12_R_0	BY927435
E_EL_fcP8_05E01_F_0	BY919112
E_EL_fcP8_05E01_R_0	BY927436
E_EL_fcP8_05E02_R_0	BY927437
E_EL_fcP8_05E04_F_0	BY919113
E_EL_fcP8_05E04_R_0	BY927438
E_EL_fcP8_05E05_F_0	BY919114
E_EL_fcP8_05E05_R_0	BY927439
E_EL_fcP8_05E06_F_0	BY919115
E_EL_fcP8_05E06_R_0	BY927440
E_EL_fcP8_05E07_F_0	BY919116
E_EL_fcP8_05E07_R_0	BY927441
E_EL_fcP8_05E08_F_0	BY919117
E_EL_fcP8_05E08_R_0	BY927442
E_EL_fcP8_05E09_F_0	BY919118
E_EL_fcP8_05E10_F_0	BY919119
E_EL_fcP8_05E10_R_0	BY927443
E_EL_fcP8_05E11_F_0	BY919120
E_EL_fcP8_05E11_R_0	BY927444
E_EL_fcP8_05E12_R_0	BY927445
E_EL_fcP8_05F02_F_0	BY919121
E_EL_fcP8_05F02_R_0	BY927446
E_EL_fcP8_05F03_F_0	BY919122
E_EL_fcP8_05F03_R_0	BY927447
E_EL_fcP8_05F04_F_0	BY919123
E_EL_fcP8_05F04_R_0	BY927448
E_EL_fcP8_05F05_F_0	BY919124
E_EL_fcP8_05F05_R_0	BY927449
E_EL_fcP8_05F06_F_0	BY919125
E_EL_fcP8_05F06_R_0	BY927450
E_EL_fcP8_05F07_R_0	BY927451
E_EL_fcP8_05F08_R_0	BY927452
E_EL_fcP8_05F09_F_0	BY919126
E_EL_fcP8_05F09_R_0	BY927453
E_EL_fcP8_05F10_F_0	BY919127
E_EL_fcP8_05F10_R_0	BY927454

E_EL_fcP8_05F11_F_0	BY919128
E_EL_fcP8_05F11_R_0	BY927455
E_EL_fcP8_05G01_R_0	BY927456
E_EL_fcP8_05G02_F_0	BY919129
E_EL_fcP8_05G02_R_0	BY927457
E_EL_fcP8_05G03_R_0	BY927458
E_EL_fcP8_05G04_F_0	BY919130
E_EL_fcP8_05G04_R_0	BY927459
E_EL_fcP8_05G05_F_0	BY919131
E_EL_fcP8_05G05_R_0	BY927460
E_EL_fcP8_05G07_F_0	BY919132
E_EL_fcP8_05G07_R_0	BY927461
E_EL_fcP8_05G08_F_0	BY919133
E_EL_fcP8_05G08_R_0	BY927462
E_EL_fcP8_05G09_R_0	BY927463
E_EL_fcP8_05G10_F_0	BY919134
E_EL_fcP8_05G10_R_0	BY927464
E_EL_fcP8_05G11_F_0	BY919135
E_EL_fcP8_05G11_R_0	BY927465
E_EL_fcP8_05G12_F_0	BY919136
E_EL_fcP8_05G12_R_0	BY927466
E_EL_fcP8_05H01_F_0	BY919137
E_EL_fcP8_05H01_R_0	BY927467
E_EL_fcP8_05H02_F_0	BY919138
E_EL_fcP8_05H02_R_0	BY927468
E_EL_fcP8_05H03_F_0	BY919139
E_EL_fcP8_05H03_R_0	BY927469
E_EL_fcP8_05H04_F_0	BY919140
E_EL_fcP8_05H04_R_0	BY927470
E_EL_fcP8_05H05_F_0	BY919141
E_EL_fcP8_05H05_R_0	BY927471
E_EL_fcP8_05H07_F_0	BY919142
E_EL_fcP8_05H07_R_0	BY927472
E_EL_fcP8_05H08_F_0	BY919143
E_EL_fcP8_05H08_R_0	BY927473
E_EL_fcP8_05H09_F_0	BY919144
E_EL_fcP8_05H09_R_0	BY927474
E_EL_fcP8_05H10_F_0	BY919145
E_EL_fcP8_05H10_R_0	BY927475
E_EL_fcP8_05H11_F_0	BY919146
E_EL_fcP8_05H11_R_0	BY927476
E_EL_fcP8_05H12_F_0	BY919147
E_EL_fcP8_05H12_R_0	BY927477
E_EL_fcP8_06A01_F_0	BY919148
E_EL_fcP8_06A01_R_0	BY927478
E_EL_fcP8_06A02_F_0	BY919149
E_EL_fcP8_06A02_R_0	BY927479
E_EL_fcP8_06A03_F_0	BY919150
E_EL_fcP8_06A03_R_0	BY927480
E_EL_fcP8_06A06_F_0	BY919151
E_EL_fcP8_06A06_R_0	BY927481
E_EL_fcP8_06A07_F_0	BY919152
E_EL_fcP8_06A07_R_0	BY927482
E_EL_fcP8_06A08_R_0	BY927483

E_EL_fcP8_06A09_F_0	BY919153
E_EL_fcP8_06A09_R_0	BY927484
E_EL_fcP8_06A10_F_0	BY919154
E_EL_fcP8_06A10_R_0	BY927485
E_EL_fcP8_06A11_F_0	BY919155
E_EL_fcP8_06A11_R_0	BY927486
E_EL_fcP8_06A12_F_0	BY919156
E_EL_fcP8_06A12_R_0	BY927487
E_EL_fcP8_06B01_F_0	BY919157
E_EL_fcP8_06B01_R_0	BY927488
E_EL_fcP8_06B02_F_0	BY919158
E_EL_fcP8_06B02_R_0	BY927489
E_EL_fcP8_06B03_F_0	BY919159
E_EL_fcP8_06B03_R_0	BY927490
E_EL_fcP8_06B05_F_0	BY919160
E_EL_fcP8_06B05_R_0	BY927491
E_EL_fcP8_06B06_F_0	BY919161
E_EL_fcP8_06B06_R_0	BY927492
E_EL_fcP8_06B07_F_0	BY919162
E_EL_fcP8_06B07_R_0	BY927493
E_EL_fcP8_06B08_F_0	BY919163
E_EL_fcP8_06B08_R_0	BY927494
E_EL_fcP8_06B10_R_0	BY927495
E_EL_fcP8_06B11_F_0	BY919164
E_EL_fcP8_06B11_R_0	BY927496
E_EL_fcP8_06B12_F_0	BY919165
E_EL_fcP8_06B12_R_0	BY927497
E_EL_fcP8_06C01_F_0	BY919166
E_EL_fcP8_06C01_R_0	BY927498
E_EL_fcP8_06C02_F_0	BY919167
E_EL_fcP8_06C02_R_0	BY927499
E_EL_fcP8_06C03_R_0	BY927500
E_EL_fcP8_06C04_F_0	BY919168
E_EL_fcP8_06C04_R_0	BY927501
E_EL_fcP8_06C06_F_0	BY919169
E_EL_fcP8_06C06_R_0	BY927502
E_EL_fcP8_06C08_R_0	BY927503
E_EL_fcP8_06C09_F_0	BY919170
E_EL_fcP8_06C09_R_0	BY927504
E_EL_fcP8_06C10_R_0	BY927505
E_EL_fcP8_06C11_F_0	BY919171
E_EL_fcP8_06C11_R_0	BY927506
E_EL_fcP8_06C12_F_0	BY919172
E_EL_fcP8_06C12_R_0	BY927507
E_EL_fcP8_06D02_F_0	BY919173
E_EL_fcP8_06D02_R_0	BY927508
E_EL_fcP8_06D04_F_0	BY919174
E_EL_fcP8_06D04_R_0	BY927509
E_EL_fcP8_06D05_F_0	BY919175
E_EL_fcP8_06D05_R_0	BY927510
E_EL_fcP8_06D07_F_0	BY919176
E_EL_fcP8_06D07_R_0	BY927511
E_EL_fcP8_06D08_F_0	BY919177
E_EL_fcP8_06D08_R_0	BY927512

E_EL_fcP8_06D09_F_0	BY919178
E_EL_fcP8_06D09_R_0	BY927513
E_EL_fcP8_06D11_F_0	BY919179
E_EL_fcP8_06D11_R_0	BY927514
E_EL_fcP8_06D12_F_0	BY919180
E_EL_fcP8_06D12_R_0	BY927515
E_EL_fcP8_06E01_F_0	BY919181
E_EL_fcP8_06E03_F_0	BY919182
E_EL_fcP8_06E03_R_0	BY927516
E_EL_fcP8_06E04_F_0	BY919183
E_EL_fcP8_06E04_R_0	BY927517
E_EL_fcP8_06E05_F_0	BY919184
E_EL_fcP8_06E05_R_0	BY927518
E_EL_fcP8_06E06_F_0	BY919185
E_EL_fcP8_06E06_R_0	BY927519
E_EL_fcP8_06E07_F_0	BY919186
E_EL_fcP8_06E07_R_0	BY927520
E_EL_fcP8_06E08_F_0	BY919187
E_EL_fcP8_06E08_R_0	BY927521
E_EL_fcP8_06E09_F_0	BY919188
E_EL_fcP8_06E09_R_0	BY927522
E_EL_fcP8_06E10_R_0	BY927523
E_EL_fcP8_06E11_F_0	BY919189
E_EL_fcP8_06E11_R_0	BY927524
E_EL_fcP8_06E12_R_0	BY927525
E_EL_fcP8_06F01_F_0	BY919190
E_EL_fcP8_06F01_R_0	BY927526
E_EL_fcP8_06F02_F_0	BY919191
E_EL_fcP8_06F02_R_0	BY927527
E_EL_fcP8_06F03_F_0	BY919192
E_EL_fcP8_06F03_R_0	BY927528
E_EL_fcP8_06F04_F_0	BY919193
E_EL_fcP8_06F04_R_0	BY927529
E_EL_fcP8_06F05_F_0	BY919194
E_EL_fcP8_06F05_R_0	BY927530
E_EL_fcP8_06F06_F_0	BY919195
E_EL_fcP8_06F06_R_0	BY927531
E_EL_fcP8_06F07_F_0	BY919196
E_EL_fcP8_06F07_R_0	BY927532
E_EL_fcP8_06F08_F_0	BY919197
E_EL_fcP8_06F08_R_0	BY927533
E_EL_fcP8_06F09_F_0	BY919198
E_EL_fcP8_06F09_R_0	BY927534
E_EL_fcP8_06F10_F_0	BY919199
E_EL_fcP8_06F10_R_0	BY927535
E_EL_fcP8_06F11_F_0	BY919200
E_EL_fcP8_06F11_R_0	BY927536
E_EL_fcP8_06F12_F_0	BY919201
E_EL_fcP8_06F12_R_0	BY927537
E_EL_fcP8_06G01_F_0	BY919202
E_EL_fcP8_06G01_R_0	BY927538
E_EL_fcP8_06G02_F_0	BY919203
E_EL_fcP8_06G02_R_0	BY927539
E_EL_fcP8_06G03_R_0	BY927540

E_EL_fcP8_06G04_F_0	BY919204
E_EL_fcP8_06G04_R_0	BY927541
E_EL_fcP8_06G05_F_0	BY919205
E_EL_fcP8_06G05_R_0	BY927542
E_EL_fcP8_06G06_F_0	BY919206
E_EL_fcP8_06G06_R_0	BY927543
E_EL_fcP8_06G08_F_0	BY919207
E_EL_fcP8_06G08_R_0	BY927544
E_EL_fcP8_06G09_F_0	BY919208
E_EL_fcP8_06G09_R_0	BY927545
E_EL_fcP8_06G10_F_0	BY919209
E_EL_fcP8_06G10_R_0	BY927546
E_EL_fcP8_06G11_R_0	BY927547
E_EL_fcP8_06G12_F_0	BY919210
E_EL_fcP8_06G12_R_0	BY927548
E_EL_fcP8_06H01_F_0	BY919211
E_EL_fcP8_06H02_F_0	BY919212
E_EL_fcP8_06H02_R_0	BY927549
E_EL_fcP8_06H03_R_0	BY927550
E_EL_fcP8_06H04_F_0	BY919213
E_EL_fcP8_06H04_R_0	BY927551
E_EL_fcP8_06H05_F_0	BY919214
E_EL_fcP8_06H05_R_0	BY927552
E_EL_fcP8_06H06_F_0	BY919215
E_EL_fcP8_06H06_R_0	BY927553
E_EL_fcP8_06H07_R_0	BY927554
E_EL_fcP8_06H08_F_0	BY919216
E_EL_fcP8_06H08_R_0	BY927555
E_EL_fcP8_06H09_R_0	BY927556
E_EL_fcP8_06H10_F_0	BY919217
E_EL_fcP8_06H10_R_0	BY927557
E_EL_fcP8_06H11_F_0	BY919218
E_EL_fcP8_06H11_R_0	BY927558
E_EL_fcP8_06H12_R_0	BY927559
E_EL_fcP8_07A01_F_0	BY919219
E_EL_fcP8_07A01_R_0	BY927560
E_EL_fcP8_07A02_F_0	BY919220
E_EL_fcP8_07A02_R_0	BY927561
E_EL_fcP8_07A04_F_0	BY919221
E_EL_fcP8_07A04_R_0	BY927562
E_EL_fcP8_07A05_F_0	BY919222
E_EL_fcP8_07A05_R_0	BY927563
E_EL_fcP8_07A06_F_0	BY919223
E_EL_fcP8_07A06_R_0	BY927564
E_EL_fcP8_07A07_F_0	BY919224
E_EL_fcP8_07A07_R_0	BY927565
E_EL_fcP8_07A11_F_0	BY919225
E_EL_fcP8_07A11_R_0	BY927566
E_EL_fcP8_07B01_F_0	BY919226
E_EL_fcP8_07B01_R_0	BY927567
E_EL_fcP8_07B02_F_0	BY919227
E_EL_fcP8_07B02_R_0	BY927568
E_EL_fcP8_07B03_F_0	BY919228
E_EL_fcP8_07B03_R_0	BY927569

E_EL_fcP8_07B04_R_0	BY927570
E_EL_fcP8_07B05_F_0	BY919229
E_EL_fcP8_07B05_R_0	BY927571
E_EL_fcP8_07B07_F_0	BY919230
E_EL_fcP8_07B09_F_0	BY919231
E_EL_fcP8_07B09_R_0	BY927572
E_EL_fcP8_07B10_F_0	BY919232
E_EL_fcP8_07B10_R_0	BY927573
E_EL_fcP8_07B11_F_0	BY919233
E_EL_fcP8_07B11_R_0	BY927574
E_EL_fcP8_07B12_F_0	BY919234
E_EL_fcP8_07B12_R_0	BY927575
E_EL_fcP8_07C01_R_0	BY927576
E_EL_fcP8_07C02_F_0	BY919235
E_EL_fcP8_07C02_R_0	BY927577
E_EL_fcP8_07C03_F_0	BY919236
E_EL_fcP8_07C03_R_0	BY927578
E_EL_fcP8_07C04_F_0	BY919237
E_EL_fcP8_07C04_R_0	BY927579
E_EL_fcP8_07C06_F_0	BY919238
E_EL_fcP8_07C06_R_0	BY927580
E_EL_fcP8_07C07_F_0	BY919239
E_EL_fcP8_07C07_R_0	BY927581
E_EL_fcP8_07C08_R_0	BY927582
E_EL_fcP8_07C09_R_0	BY927583
E_EL_fcP8_07C10_F_0	BY919240
E_EL_fcP8_07C10_R_0	BY927584
E_EL_fcP8_07C11_F_0	BY919241
E_EL_fcP8_07C11_R_0	BY927585
E_EL_fcP8_07C12_F_0	BY919242
E_EL_fcP8_07D01_F_0	BY919243
E_EL_fcP8_07D01_R_0	BY927586
E_EL_fcP8_07D02_F_0	BY919244
E_EL_fcP8_07D02_R_0	BY927587
E_EL_fcP8_07D03_F_0	BY919245
E_EL_fcP8_07D03_R_0	BY927588
E_EL_fcP8_07D04_F_0	BY919246
E_EL_fcP8_07D04_R_0	BY927589
E_EL_fcP8_07D05_F_0	BY919247
E_EL_fcP8_07D05_R_0	BY927590
E_EL_fcP8_07D06_F_0	BY919248
E_EL_fcP8_07D06_R_0	BY927591
E_EL_fcP8_07D07_R_0	BY927592
E_EL_fcP8_07D09_F_0	BY919249
E_EL_fcP8_07D09_R_0	BY927593
E_EL_fcP8_07D10_F_0	BY919250
E_EL_fcP8_07D10_R_0	BY927594
E_EL_fcP8_07D11_F_0	BY919251
E_EL_fcP8_07D11_R_0	BY927595
E_EL_fcP8_07D12_F_0	BY919252
E_EL_fcP8_07D12_R_0	BY927596
E_EL_fcP8_07E01_F_0	BY919253
E_EL_fcP8_07E01_R_0	BY927597
E_EL_fcP8_07E02_F_0	BY919254

E_EL_fcP8_07E02_R_0	BY927598
E_EL_fcP8_07E03_F_0	BY919255
E_EL_fcP8_07E03_R_0	BY927599
E_EL_fcP8_07E04_F_0	BY919256
E_EL_fcP8_07E04_R_0	BY927600
E_EL_fcP8_07E05_F_0	BY919257
E_EL_fcP8_07E05_R_0	BY927601
E_EL_fcP8_07E06_F_0	BY919258
E_EL_fcP8_07E06_R_0	BY927602
E_EL_fcP8_07E07_F_0	BY919259
E_EL_fcP8_07E07_R_0	BY927603
E_EL_fcP8_07E08_F_0	BY919260
E_EL_fcP8_07E08_R_0	BY927604
E_EL_fcP8_07E09_F_0	BY919261
E_EL_fcP8_07E09_R_0	BY927605
E_EL_fcP8_07E10_F_0	BY919262
E_EL_fcP8_07E11_R_0	BY927606
E_EL_fcP8_07E12_F_0	BY919263
E_EL_fcP8_07E12_R_0	BY927607
E_EL_fcP8_07F01_F_0	BY919264
E_EL_fcP8_07F01_R_0	BY927608
E_EL_fcP8_07F02_F_0	BY919265
E_EL_fcP8_07F02_R_0	BY927609
E_EL_fcP8_07F03_F_0	BY919266
E_EL_fcP8_07F03_R_0	BY927610
E_EL_fcP8_07F04_F_0	BY919267
E_EL_fcP8_07F04_R_0	BY927611
E_EL_fcP8_07F05_F_0	BY919268
E_EL_fcP8_07F05_R_0	BY927612
E_EL_fcP8_07F06_F_0	BY919269
E_EL_fcP8_07F06_R_0	BY927613
E_EL_fcP8_07F07_F_0	BY919270
E_EL_fcP8_07F07_R_0	BY927614
E_EL_fcP8_07F08_F_0	BY919271
E_EL_fcP8_07F08_R_0	BY927615
E_EL_fcP8_07F09_F_0	BY919272
E_EL_fcP8_07F09_R_0	BY927616
E_EL_fcP8_07F10_F_0	BY919273
E_EL_fcP8_07F10_R_0	BY927617
E_EL_fcP8_07F12_F_0	BY919274
E_EL_fcP8_07F12_R_0	BY927618
E_EL_fcP8_07G01_F_0	BY919275
E_EL_fcP8_07G02_F_0	BY919276
E_EL_fcP8_07G02_R_0	BY927619
E_EL_fcP8_07G03_R_0	BY927620
E_EL_fcP8_07G04_F_0	BY919277
E_EL_fcP8_07G04_R_0	BY927621
E_EL_fcP8_07G05_R_0	BY927622
E_EL_fcP8_07G06_F_0	BY919278
E_EL_fcP8_07G06_R_0	BY927623
E_EL_fcP8_07G07_F_0	BY919279
E_EL_fcP8_07G07_R_0	BY927624
E_EL_fcP8_07G08_F_0	BY919280
E_EL_fcP8_07G08_R_0	BY927625

E_EL_fcP8_07G09_F_0	BY919281
E_EL_fcP8_07G09_R_0	BY927626
E_EL_fcP8_07G10_R_0	BY927627
E_EL_fcP8_07G11_F_0	BY919282
E_EL_fcP8_07G11_R_0	BY927628
E_EL_fcP8_07G12_R_0	BY927629
E_EL_fcP8_07H01_F_0	BY919283
E_EL_fcP8_07H01_R_0	BY927630
E_EL_fcP8_07H02_F_0	BY919284
E_EL_fcP8_07H02_R_0	BY927631
E_EL_fcP8_07H03_F_0	BY919285
E_EL_fcP8_07H03_R_0	BY927632
E_EL_fcP8_07H04_F_0	BY919286
E_EL_fcP8_07H04_R_0	BY927633
E_EL_fcP8_07H05_F_0	BY919287
E_EL_fcP8_07H05_R_0	BY927634
E_EL_fcP8_07H06_F_0	BY919288
E_EL_fcP8_07H06_R_0	BY927635
E_EL_fcP8_07H07_R_0	BY927636
E_EL_fcP8_07H08_F_0	BY919289
E_EL_fcP8_07H08_R_0	BY927637
E_EL_fcP8_07H09_F_0	BY919290
E_EL_fcP8_07H09_R_0	BY927638
E_EL_fcP8_07H10_F_0	BY919291
E_EL_fcP8_07H10_R_0	BY927639
E_EL_fcP8_07H11_F_0	BY919292
E_EL_fcP8_07H11_R_0	BY927640
E_EL_fcP8_07H12_F_0	BY919293
E_EL_fcP8_07H12_R_0	BY927641
E_EL_fcP8_08A02_F_0	BY919294
E_EL_fcP8_08A02_R_0	BY927642
E_EL_fcP8_08A03_F_0	BY919295
E_EL_fcP8_08A03_R_0	BY927643
E_EL_fcP8_08A04_F_0	BY919296
E_EL_fcP8_08A04_R_0	BY927644
E_EL_fcP8_08A05_R_0	BY927645
E_EL_fcP8_08A06_F_0	BY919297
E_EL_fcP8_08A06_R_0	BY927646
E_EL_fcP8_08A07_R_0	BY927647
E_EL_fcP8_08A09_R_0	BY927648
E_EL_fcP8_08A10_R_0	BY927649
E_EL_fcP8_08A11_F_0	BY919298
E_EL_fcP8_08A11_R_0	BY927650
E_EL_fcP8_08A12_F_0	BY919299
E_EL_fcP8_08A12_R_0	BY927651
E_EL_fcP8_08B03_R_0	BY927652
E_EL_fcP8_08B04_F_0	BY919300
E_EL_fcP8_08B04_R_0	BY927653
E_EL_fcP8_08B06_R_0	BY927654
E_EL_fcP8_08B07_R_0	BY927655
E_EL_fcP8_08B08_F_0	BY919301
E_EL_fcP8_08B08_R_0	BY927656
E_EL_fcP8_08B09_F_0	BY919302
E_EL_fcP8_08B09_R_0	BY927657

E_EL_fcP8_08B10_F_0	BY919303
E_EL_fcP8_08B10_R_0	BY927658
E_EL_fcP8_08B11_F_0	BY919304
E_EL_fcP8_08B11_R_0	BY927659
E_EL_fcP8_08B12_F_0	BY919305
E_EL_fcP8_08B12_R_0	BY927660
E_EL_fcP8_08C03_F_0	BY919306
E_EL_fcP8_08C03_R_0	BY927661
E_EL_fcP8_08C04_R_0	BY927662
E_EL_fcP8_08C05_F_0	BY919307
E_EL_fcP8_08C05_R_0	BY927663
E_EL_fcP8_08C06_F_0	BY919308
E_EL_fcP8_08C06_R_0	BY927664
E_EL_fcP8_08C07_R_0	BY927665
E_EL_fcP8_08C08_F_0	BY919309
E_EL_fcP8_08C08_R_0	BY927666
E_EL_fcP8_08C09_F_0	BY919310
E_EL_fcP8_08C09_R_0	BY927667
E_EL_fcP8_08C10_F_0	BY919311
E_EL_fcP8_08C11_F_0	BY919312
E_EL_fcP8_08C11_R_0	BY927668
E_EL_fcP8_08C12_F_0	BY919313
E_EL_fcP8_08C12_R_0	BY927669
E_EL_fcP8_08D04_R_0	BY927670
E_EL_fcP8_08D06_F_0	BY919314
E_EL_fcP8_08D06_R_0	BY927671
E_EL_fcP8_08D07_F_0	BY919315
E_EL_fcP8_08D07_R_0	BY927672
E_EL_fcP8_08D08_F_0	BY919316
E_EL_fcP8_08D08_R_0	BY927673
E_EL_fcP8_08D09_F_0	BY919317
E_EL_fcP8_08D09_R_0	BY927674
E_EL_fcP8_08D10_F_0	BY919318
E_EL_fcP8_08D10_R_0	BY927675
E_EL_fcP8_08D11_F_0	BY919319
E_EL_fcP8_08D11_R_0	BY927676
E_EL_fcP8_08D12_F_0	BY919320
E_EL_fcP8_08D12_R_0	BY927677
E_EL_fcP8_08E03_F_0	BY919321
E_EL_fcP8_08E03_R_0	BY927678
E_EL_fcP8_08E04_F_0	BY919322
E_EL_fcP8_08E04_R_0	BY927679
E_EL_fcP8_08E05_R_0	BY927680
E_EL_fcP8_08E08_F_0	BY919323
E_EL_fcP8_08E08_R_0	BY927681
E_EL_fcP8_08E09_F_0	BY919324
E_EL_fcP8_08E09_R_0	BY927682
E_EL_fcP8_08E10_F_0	BY919325
E_EL_fcP8_08E10_R_0	BY927683
E_EL_fcP8_08E12_F_0	BY919326
E_EL_fcP8_08E12_R_0	BY927684
E_EL_fcP8_08F03_F_0	BY919327
E_EL_fcP8_08F03_R_0	BY927685
E_EL_fcP8_08F04_F_0	BY919328

E_EL_fcP8_08F04_R_0	BY927686
E_EL_fcP8_08F05_F_0	BY919329
E_EL_fcP8_08F05_R_0	BY927687
E_EL_fcP8_08F06_F_0	BY919330
E_EL_fcP8_08F06_R_0	BY927688
E_EL_fcP8_08F07_F_0	BY919331
E_EL_fcP8_08F07_R_0	BY927689
E_EL_fcP8_08F08_F_0	BY919332
E_EL_fcP8_08F08_R_0	BY927690
E_EL_fcP8_08F09_F_0	BY919333
E_EL_fcP8_08F09_R_0	BY927691
E_EL_fcP8_08F11_F_0	BY919334
E_EL_fcP8_08F11_R_0	BY927692
E_EL_fcP8_08F12_F_0	BY919335
E_EL_fcP8_08F12_R_0	BY927693
E_EL_fcP8_08G02_F_0	BY919336
E_EL_fcP8_08G02_R_0	BY927694
E_EL_fcP8_08G03_F_0	BY919337
E_EL_fcP8_08G03_R_0	BY927695
E_EL_fcP8_08G04_F_0	BY919338
E_EL_fcP8_08G04_R_0	BY927696
E_EL_fcP8_08G05_F_0	BY919339
E_EL_fcP8_08G05_R_0	BY927697
E_EL_fcP8_08G07_F_0	BY919340
E_EL_fcP8_08G08_F_0	BY919341
E_EL_fcP8_08G08_R_0	BY927698
E_EL_fcP8_08G09_F_0	BY919342
E_EL_fcP8_08G09_R_0	BY927699
E_EL_fcP8_08G10_R_0	BY927700
E_EL_fcP8_08G11_F_0	BY919343
E_EL_fcP8_08G11_R_0	BY927701
E_EL_fcP8_08G12_F_0	BY919344
E_EL_fcP8_08G12_R_0	BY927702
E_EL_fcP8_08H03_F_0	BY919345
E_EL_fcP8_08H03_R_0	BY927703
E_EL_fcP8_08H04_F_0	BY919346
E_EL_fcP8_08H04_R_0	BY927704
E_EL_fcP8_08H05_F_0	BY919347
E_EL_fcP8_08H05_R_0	BY927705
E_EL_fcP8_08H06_F_0	BY919348
E_EL_fcP8_08H06_R_0	BY927706
E_EL_fcP8_08H07_F_0	BY919349
E_EL_fcP8_08H07_R_0	BY927707
E_EL_fcP8_08H08_F_0	BY919350
E_EL_fcP8_08H08_R_0	BY927708
E_EL_fcP8_08H09_F_0	BY919351
E_EL_fcP8_08H09_R_0	BY927709
E_EL_fcP8_08H11_F_0	BY919352
E_EL_fcP8_08H11_R_0	BY927710
E_EL_fcP8_08H12_F_0	BY919353
E_EL_fcP8_08H12_R_0	BY927711
E_EL_fcP8_09A01_F_0	BY919354
E_EL_fcP8_09A01_R_0	BY927712
E_EL_fcP8_09A02_F_0	BY919355

E_EL_fcP8_09A02_R_0	BY927713
E_EL_fcP8_09A03_F_0	BY919356
E_EL_fcP8_09A03_R_0	BY927714
E_EL_fcP8_09A04_F_0	BY919357
E_EL_fcP8_09A04_R_0	BY927715
E_EL_fcP8_09A06_F_0	BY919358
E_EL_fcP8_09A06_R_0	BY927716
E_EL_fcP8_09A07_F_0	BY919359
E_EL_fcP8_09A07_R_0	BY927717
E_EL_fcP8_09A08_R_0	BY927718
E_EL_fcP8_09A09_F_0	BY919360
E_EL_fcP8_09A09_R_0	BY927719
E_EL_fcP8_09A10_R_0	BY927720
E_EL_fcP8_09A11_F_0	BY919361
E_EL_fcP8_09A11_R_0	BY927721
E_EL_fcP8_09A12_F_0	BY919362
E_EL_fcP8_09A12_R_0	BY927722
E_EL_fcP8_09B01_F_0	BY919363
E_EL_fcP8_09B01_R_0	BY927723
E_EL_fcP8_09B02_F_0	BY919364
E_EL_fcP8_09B03_F_0	BY919365
E_EL_fcP8_09B03_R_0	BY927724
E_EL_fcP8_09B04_F_0	BY919366
E_EL_fcP8_09B04_R_0	BY927725
E_EL_fcP8_09B05_F_0	BY919367
E_EL_fcP8_09B05_R_0	BY927726
E_EL_fcP8_09B06_F_0	BY919368
E_EL_fcP8_09B06_R_0	BY927727
E_EL_fcP8_09B07_F_0	BY919369
E_EL_fcP8_09B07_R_0	BY927728
E_EL_fcP8_09B08_F_0	BY919370
E_EL_fcP8_09B08_R_0	BY927729
E_EL_fcP8_09B09_F_0	BY919371
E_EL_fcP8_09B09_R_0	BY927730
E_EL_fcP8_09B10_F_0	BY919372
E_EL_fcP8_09B10_R_0	BY927731
E_EL_fcP8_09B11_F_0	BY919373
E_EL_fcP8_09B11_R_0	BY927732
E_EL_fcP8_09B12_F_0	BY919374
E_EL_fcP8_09B12_R_0	BY927733
E_EL_fcP8_09C01_F_0	BY919375
E_EL_fcP8_09C01_R_0	BY927734
E_EL_fcP8_09C02_F_0	BY919376
E_EL_fcP8_09C02_R_0	BY927735
E_EL_fcP8_09C03_F_0	BY919377
E_EL_fcP8_09C04_F_0	BY919378
E_EL_fcP8_09C04_R_0	BY927736
E_EL_fcP8_09C05_F_0	BY919379
E_EL_fcP8_09C05_R_0	BY927737
E_EL_fcP8_09C06_F_0	BY919380
E_EL_fcP8_09C06_R_0	BY927738
E_EL_fcP8_09C08_F_0	BY919381
E_EL_fcP8_09C08_R_0	BY927739
E_EL_fcP8_09C09_F_0	BY919382

E_EL_fcP8_09C09_R_0	BY927740
E_EL_fcP8_09C10_F_0	BY919383
E_EL_fcP8_09C10_R_0	BY927741
E_EL_fcP8_09C11_F_0	BY919384
E_EL_fcP8_09C11_R_0	BY927742
E_EL_fcP8_09D01_F_0	BY919385
E_EL_fcP8_09D01_R_0	BY927743
E_EL_fcP8_09D02_R_0	BY927744
E_EL_fcP8_09D03_F_0	BY919386
E_EL_fcP8_09D03_R_0	BY927745
E_EL_fcP8_09D04_F_0	BY919387
E_EL_fcP8_09D04_R_0	BY927746
E_EL_fcP8_09D06_R_0	BY927747
E_EL_fcP8_09D07_F_0	BY919388
E_EL_fcP8_09D07_R_0	BY927748
E_EL_fcP8_09D08_R_0	BY927749
E_EL_fcP8_09D09_F_0	BY919389
E_EL_fcP8_09D09_R_0	BY927750
E_EL_fcP8_09D10_F_0	BY919390
E_EL_fcP8_09D10_R_0	BY927751
E_EL_fcP8_09D11_F_0	BY919391
E_EL_fcP8_09D11_R_0	BY927752
E_EL_fcP8_09D12_F_0	BY919392
E_EL_fcP8_09D12_R_0	BY927753
E_EL_fcP8_09E01_F_0	BY919393
E_EL_fcP8_09E01_R_0	BY927754
E_EL_fcP8_09E02_F_0	BY919394
E_EL_fcP8_09E02_R_0	BY927755
E_EL_fcP8_09E03_F_0	BY919395
E_EL_fcP8_09E03_R_0	BY927756
E_EL_fcP8_09E04_F_0	BY919396
E_EL_fcP8_09E04_R_0	BY927757
E_EL_fcP8_09E05_F_0	BY919397
E_EL_fcP8_09E06_R_0	BY927758
E_EL_fcP8_09E07_F_0	BY919398
E_EL_fcP8_09E07_R_0	BY927759
E_EL_fcP8_09E08_F_0	BY919399
E_EL_fcP8_09E08_R_0	BY927760
E_EL_fcP8_09E09_F_0	BY919400
E_EL_fcP8_09E09_R_0	BY927761
E_EL_fcP8_09E10_F_0	BY919401
E_EL_fcP8_09E10_R_0	BY927762
E_EL_fcP8_09E11_F_0	BY919402
E_EL_fcP8_09E11_R_0	BY927763
E_EL_fcP8_09E12_F_0	BY919403
E_EL_fcP8_09E12_R_0	BY927764
E_EL_fcP8_09F01_F_0	BY919404
E_EL_fcP8_09F01_R_0	BY927765
E_EL_fcP8_09F03_F_0	BY919405
E_EL_fcP8_09F03_R_0	BY927766
E_EL_fcP8_09F04_F_0	BY919406
E_EL_fcP8_09F04_R_0	BY927767
E_EL_fcP8_09F05_R_0	BY927768
E_EL_fcP8_09F06_F_0	BY919407

E_EL_fcP8_09F06_R_0	BY927769
E_EL_fcP8_09F07_F_0	BY919408
E_EL_fcP8_09F07_R_0	BY927770
E_EL_fcP8_09F08_F_0	BY919409
E_EL_fcP8_09F08_R_0	BY927771
E_EL_fcP8_09F09_F_0	BY919410
E_EL_fcP8_09F10_F_0	BY919411
E_EL_fcP8_09F10_R_0	BY927772
E_EL_fcP8_09F11_F_0	BY919412
E_EL_fcP8_09F11_R_0	BY927773
E_EL_fcP8_09F12_F_0	BY919413
E_EL_fcP8_09F12_R_0	BY927774
E_EL_fcP8_09G01_F_0	BY919414
E_EL_fcP8_09G01_R_0	BY927775
E_EL_fcP8_09G02_F_0	BY919415
E_EL_fcP8_09G02_R_0	BY927776
E_EL_fcP8_09G03_F_0	BY919416
E_EL_fcP8_09G03_R_0	BY927777
E_EL_fcP8_09G04_F_0	BY919417
E_EL_fcP8_09G04_R_0	BY927778
E_EL_fcP8_09G05_F_0	BY919418
E_EL_fcP8_09G05_R_0	BY927779
E_EL_fcP8_09G06_F_0	BY919419
E_EL_fcP8_09G06_R_0	BY927780
E_EL_fcP8_09G07_F_0	BY919420
E_EL_fcP8_09G09_F_0	BY919421
E_EL_fcP8_09G09_R_0	BY927781
E_EL_fcP8_09G10_F_0	BY919422
E_EL_fcP8_09G10_R_0	BY927782
E_EL_fcP8_09G11_F_0	BY919423
E_EL_fcP8_09G11_R_0	BY927783
E_EL_fcP8_09G12_F_0	BY919424
E_EL_fcP8_09G12_R_0	BY927784
E_EL_fcP8_09H01_F_0	BY919425
E_EL_fcP8_09H01_R_0	BY927785
E_EL_fcP8_09H03_F_0	BY919426
E_EL_fcP8_09H03_R_0	BY927786
E_EL_fcP8_09H04_F_0	BY919427
E_EL_fcP8_09H04_R_0	BY927787
E_EL_fcP8_09H06_F_0	BY919428
E_EL_fcP8_09H06_R_0	BY927788
E_EL_fcP8_09H07_F_0	BY919429
E_EL_fcP8_09H07_R_0	BY927789
E_EL_fcP8_09H08_F_0	BY919430
E_EL_fcP8_09H08_R_0	BY927790
E_EL_fcP8_09H10_F_0	BY919431
E_EL_fcP8_09H10_R_0	BY927791
E_EL_fcP8_09H11_F_0	BY919432
E_EL_fcP8_09H11_R_0	BY927792
E_EL_fcP8_09H12_F_0	BY919433
E_EL_fcP8_09H12_R_0	BY927793
E_EL_fcP8_10A01_F_0	BY919434
E_EL_fcP8_10A01_R_0	BY927794
E_EL_fcP8_10A02_F_0	BY919435

E_EL_fcP8_10A02_R_0	BY927795
E_EL_fcP8_10A03_F_0	BY919436
E_EL_fcP8_10A03_R_0	BY927796
E_EL_fcP8_10A04_F_0	BY919437
E_EL_fcP8_10A04_R_0	BY927797
E_EL_fcP8_10A05_R_0	BY927798
E_EL_fcP8_10A06_F_0	BY919438
E_EL_fcP8_10A06_R_0	BY927799
E_EL_fcP8_10A07_F_0	BY919439
E_EL_fcP8_10A07_R_0	BY927800
E_EL_fcP8_10A08_F_0	BY919440
E_EL_fcP8_10A08_R_0	BY927801
E_EL_fcP8_10A09_F_0	BY919441
E_EL_fcP8_10A09_R_0	BY927802
E_EL_fcP8_10A10_F_0	BY919442
E_EL_fcP8_10A10_R_0	BY927803
E_EL_fcP8_10A11_F_0	BY919443
E_EL_fcP8_10A11_R_0	BY927804
E_EL_fcP8_10A12_F_0	BY919444
E_EL_fcP8_10A12_R_0	BY927805
E_EL_fcP8_10B01_F_0	BY919445
E_EL_fcP8_10B01_R_0	BY927806
E_EL_fcP8_10B02_F_0	BY919446
E_EL_fcP8_10B03_F_0	BY919447
E_EL_fcP8_10B03_R_0	BY927807
E_EL_fcP8_10B04_F_0	BY919448
E_EL_fcP8_10B04_R_0	BY927808
E_EL_fcP8_10B05_F_0	BY919449
E_EL_fcP8_10B05_R_0	BY927809
E_EL_fcP8_10B06_F_0	BY919450
E_EL_fcP8_10B06_R_0	BY927810
E_EL_fcP8_10B07_F_0	BY919451
E_EL_fcP8_10B07_R_0	BY927811
E_EL_fcP8_10B08_F_0	BY919452
E_EL_fcP8_10B08_R_0	BY927812
E_EL_fcP8_10B09_F_0	BY919453
E_EL_fcP8_10B09_R_0	BY927813
E_EL_fcP8_10B10_F_0	BY919454
E_EL_fcP8_10B10_R_0	BY927814
E_EL_fcP8_10B11_R_0	BY927815
E_EL_fcP8_10B12_F_0	BY919455
E_EL_fcP8_10B12_R_0	BY927816
E_EL_fcP8_10C02_F_0	BY919456
E_EL_fcP8_10C02_R_0	BY927817
E_EL_fcP8_10C03_F_0	BY919457
E_EL_fcP8_10C03_R_0	BY927818
E_EL_fcP8_10C04_F_0	BY919458
E_EL_fcP8_10C04_R_0	BY927819
E_EL_fcP8_10C05_F_0	BY919459
E_EL_fcP8_10C05_R_0	BY927820
E_EL_fcP8_10C06_F_0	BY919460
E_EL_fcP8_10C06_R_0	BY927821
E_EL_fcP8_10C07_F_0	BY919461
E_EL_fcP8_10C07_R_0	BY927822

E_EL_fcP8_10C08_F_0	BY919462
E_EL_fcP8_10C08_R_0	BY927823
E_EL_fcP8_10C09_F_0	BY919463
E_EL_fcP8_10C09_R_0	BY927824
E_EL_fcP8_10C10_F_0	BY919464
E_EL_fcP8_10C10_R_0	BY927825
E_EL_fcP8_10C11_F_0	BY919465
E_EL_fcP8_10C11_R_0	BY927826
E_EL_fcP8_10C12_F_0	BY919466
E_EL_fcP8_10C12_R_0	BY927827
E_EL_fcP8_10D01_F_0	BY919467
E_EL_fcP8_10D01_R_0	BY927828
E_EL_fcP8_10D02_R_0	BY927829
E_EL_fcP8_10D03_R_0	BY927830
E_EL_fcP8_10D04_F_0	BY919468
E_EL_fcP8_10D04_R_0	BY927831
E_EL_fcP8_10D06_F_0	BY919469
E_EL_fcP8_10D06_R_0	BY927832
E_EL_fcP8_10D07_F_0	BY919470
E_EL_fcP8_10D07_R_0	BY927833
E_EL_fcP8_10D08_F_0	BY919471
E_EL_fcP8_10D08_R_0	BY927834
E_EL_fcP8_10D09_F_0	BY919472
E_EL_fcP8_10D09_R_0	BY927835
E_EL_fcP8_10D10_F_0	BY919473
E_EL_fcP8_10D10_R_0	BY927836
E_EL_fcP8_10D11_F_0	BY919474
E_EL_fcP8_10D11_R_0	BY927837
E_EL_fcP8_10D12_F_0	BY919475
E_EL_fcP8_10D12_R_0	BY927838
E_EL_fcP8_10E01_F_0	BY919476
E_EL_fcP8_10E01_R_0	BY927839
E_EL_fcP8_10E02_F_0	BY919477
E_EL_fcP8_10E02_R_0	BY927840
E_EL_fcP8_10E04_F_0	BY919478
E_EL_fcP8_10E04_R_0	BY927841
E_EL_fcP8_10E05_R_0	BY927842
E_EL_fcP8_10E06_R_0	BY927843
E_EL_fcP8_10E08_F_0	BY919479
E_EL_fcP8_10E08_R_0	BY927844
E_EL_fcP8_10E09_F_0	BY919480
E_EL_fcP8_10E09_R_0	BY927845
E_EL_fcP8_10E10_F_0	BY919481
E_EL_fcP8_10E10_R_0	BY927846
E_EL_fcP8_10E11_F_0	BY919482
E_EL_fcP8_10E11_R_0	BY927847
E_EL_fcP8_10E12_F_0	BY919483
E_EL_fcP8_10E12_R_0	BY927848
E_EL_fcP8_10F01_F_0	BY919484
E_EL_fcP8_10F01_R_0	BY927849
E_EL_fcP8_10F02_F_0	BY919485
E_EL_fcP8_10F02_R_0	BY927850
E_EL_fcP8_10F03_F_0	BY919486
E_EL_fcP8_10F03_R_0	BY927851

E_EL_fcP8_10F04_F_0	BY919487
E_EL_fcP8_10F04_R_0	BY927852
E_EL_fcP8_10F05_F_0	BY919488
E_EL_fcP8_10F05_R_0	BY927853
E_EL_fcP8_10F06_F_0	BY919489
E_EL_fcP8_10F06_R_0	BY927854
E_EL_fcP8_10F07_F_0	BY919490
E_EL_fcP8_10F07_R_0	BY927855
E_EL_fcP8_10F08_R_0	BY927856
E_EL_fcP8_10F09_F_0	BY919491
E_EL_fcP8_10F09_R_0	BY927857
E_EL_fcP8_10F10_R_0	BY927858
E_EL_fcP8_10F11_F_0	BY919492
E_EL_fcP8_10F11_R_0	BY927859
E_EL_fcP8_10F12_F_0	BY919493
E_EL_fcP8_10F12_R_0	BY927860
E_EL_fcP8_10G01_F_0	BY919494
E_EL_fcP8_10G01_R_0	BY927861
E_EL_fcP8_10G02_F_0	BY919495
E_EL_fcP8_10G02_R_0	BY927862
E_EL_fcP8_10G03_F_0	BY919496
E_EL_fcP8_10G03_R_0	BY927863
E_EL_fcP8_10G04_F_0	BY919497
E_EL_fcP8_10G04_R_0	BY927864
E_EL_fcP8_10G05_F_0	BY919498
E_EL_fcP8_10G05_R_0	BY927865
E_EL_fcP8_10G06_F_0	BY919499
E_EL_fcP8_10G06_R_0	BY927866
E_EL_fcP8_10G08_F_0	BY919500
E_EL_fcP8_10G08_R_0	BY927867
E_EL_fcP8_10G10_R_0	BY927868
E_EL_fcP8_10G11_F_0	BY919501
E_EL_fcP8_10G11_R_0	BY927869
E_EL_fcP8_11A01_F_0	BY919502
E_EL_fcP8_11A01_R_0	BY927870
E_EL_fcP8_11A02_F_0	BY919503
E_EL_fcP8_11A02_R_0	BY927871
E_EL_fcP8_11A03_F_0	BY919504
E_EL_fcP8_11A04_F_0	BY919505
E_EL_fcP8_11A04_R_0	BY927872
E_EL_fcP8_11A05_F_0	BY919506
E_EL_fcP8_11A05_R_0	BY927873
E_EL_fcP8_11A06_F_0	BY919507
E_EL_fcP8_11A06_R_0	BY927874
E_EL_fcP8_11A07_F_0	BY919508
E_EL_fcP8_11A07_R_0	BY927875
E_EL_fcP8_11A08_F_0	BY919509
E_EL_fcP8_11A08_R_0	BY927876
E_EL_fcP8_11A09_F_0	BY919510
E_EL_fcP8_11A09_R_0	BY927877
E_EL_fcP8_11A10_F_0	BY919511
E_EL_fcP8_11A10_R_0	BY927878
E_EL_fcP8_11A11_R_0	BY927879
E_EL_fcP8_11A12_F_0	BY919512

E_EL_fcP8_11A12_R_0	BY927880
E_EL_fcP8_11B01_F_0	BY919513
E_EL_fcP8_11B02_F_0	BY919514
E_EL_fcP8_11B02_R_0	BY927881
E_EL_fcP8_11B03_F_0	BY919515
E_EL_fcP8_11B04_F_0	BY919516
E_EL_fcP8_11B04_R_0	BY927882
E_EL_fcP8_11B05_F_0	BY919517
E_EL_fcP8_11B05_R_0	BY927883
E_EL_fcP8_11B06_F_0	BY919518
E_EL_fcP8_11B06_R_0	BY927884
E_EL_fcP8_11B07_F_0	BY919519
E_EL_fcP8_11B07_R_0	BY927885
E_EL_fcP8_11B08_F_0	BY919520
E_EL_fcP8_11B08_R_0	BY927886
E_EL_fcP8_11B10_F_0	BY919521
E_EL_fcP8_11B10_R_0	BY927887
E_EL_fcP8_11B11_F_0	BY919522
E_EL_fcP8_11B11_R_0	BY927888
E_EL_fcP8_11B12_F_0	BY919523
E_EL_fcP8_11C01_F_0	BY919524
E_EL_fcP8_11C01_R_0	BY927889
E_EL_fcP8_11C02_F_0	BY919525
E_EL_fcP8_11C02_R_0	BY927890
E_EL_fcP8_11C03_F_0	BY919526
E_EL_fcP8_11C04_F_0	BY919527
E_EL_fcP8_11C04_R_0	BY927891
E_EL_fcP8_11C05_F_0	BY919528
E_EL_fcP8_11C05_R_0	BY927892
E_EL_fcP8_11C06_F_0	BY919529
E_EL_fcP8_11C06_R_0	BY927893
E_EL_fcP8_11C07_F_0	BY919530
E_EL_fcP8_11C07_R_0	BY927894
E_EL_fcP8_11C08_F_0	BY919531
E_EL_fcP8_11C09_F_0	BY919532
E_EL_fcP8_11C09_R_0	BY927895
E_EL_fcP8_11C10_F_0	BY919533
E_EL_fcP8_11C10_R_0	BY927896
E_EL_fcP8_11C11_F_0	BY919534
E_EL_fcP8_11C11_R_0	BY927897
E_EL_fcP8_11C12_F_0	BY919535
E_EL_fcP8_11C12_R_0	BY927898
E_EL_fcP8_11D01_F_0	BY919536
E_EL_fcP8_11D01_R_0	BY927899
E_EL_fcP8_11D02_F_0	BY919537
E_EL_fcP8_11D02_R_0	BY927900
E_EL_fcP8_11D03_F_0	BY919538
E_EL_fcP8_11D05_F_0	BY919539
E_EL_fcP8_11D05_R_0	BY927901
E_EL_fcP8_11D06_F_0	BY919540
E_EL_fcP8_11D06_R_0	BY927902
E_EL_fcP8_11D07_F_0	BY919541
E_EL_fcP8_11D07_R_0	BY927903
E_EL_fcP8_11D08_F_0	BY919542

E_EL_fcP8_11D08_R_0	BY927904
E_EL_fcP8_11D10_F_0	BY919543
E_EL_fcP8_11D10_R_0	BY927905
E_EL_fcP8_11D11_F_0	BY919544
E_EL_fcP8_11D11_R_0	BY927906
E_EL_fcP8_11E01_F_0	BY919545
E_EL_fcP8_11E01_R_0	BY927907
E_EL_fcP8_11E02_F_0	BY919546
E_EL_fcP8_11E02_R_0	BY927908
E_EL_fcP8_11E03_F_0	BY919547
E_EL_fcP8_11E03_R_0	BY927909
E_EL_fcP8_11E04_F_0	BY919548
E_EL_fcP8_11E04_R_0	BY927910
E_EL_fcP8_11E06_F_0	BY919549
E_EL_fcP8_11E06_R_0	BY927911
E_EL_fcP8_11E07_F_0	BY919550
E_EL_fcP8_11E07_R_0	BY927912
E_EL_fcP8_11E08_F_0	BY919551
E_EL_fcP8_11E08_R_0	BY927913
E_EL_fcP8_11E09_R_0	BY927914
E_EL_fcP8_11E10_F_0	BY919552
E_EL_fcP8_11E10_R_0	BY927915
E_EL_fcP8_11E11_F_0	BY919553
E_EL_fcP8_11E11_R_0	BY927916
E_EL_fcP8_11E12_F_0	BY919554
E_EL_fcP8_11F01_F_0	BY919555
E_EL_fcP8_11F01_R_0	BY927917
E_EL_fcP8_11F02_F_0	BY919556
E_EL_fcP8_11F02_R_0	BY927918
E_EL_fcP8_11F04_F_0	BY919557
E_EL_fcP8_11F04_R_0	BY927919
E_EL_fcP8_11F05_F_0	BY919558
E_EL_fcP8_11F05_R_0	BY927920
E_EL_fcP8_11F06_F_0	BY919559
E_EL_fcP8_11F06_R_0	BY927921
E_EL_fcP8_11F07_F_0	BY919560
E_EL_fcP8_11F08_F_0	BY919561
E_EL_fcP8_11F08_R_0	BY927922
E_EL_fcP8_11F09_F_0	BY919562
E_EL_fcP8_11F09_R_0	BY927923
E_EL_fcP8_11F10_F_0	BY919563
E_EL_fcP8_11F10_R_0	BY927924
E_EL_fcP8_11F11_F_0	BY919564
E_EL_fcP8_11F11_R_0	BY927925
E_EL_fcP8_11F12_F_0	BY919565
E_EL_fcP8_11G04_F_0	BY919566
E_EL_fcP8_11G04_R_0	BY927926
E_EL_fcP8_11G05_R_0	BY927927
E_EL_fcP8_11G06_F_0	BY919567
E_EL_fcP8_11G06_R_0	BY927928
E_EL_fcP8_11G07_F_0	BY919568
E_EL_fcP8_11G07_R_0	BY927929
E_EL_fcP8_11G08_F_0	BY919569
E_EL_fcP8_11G09_F_0	BY919570

E_EL_fcP8_11G09_R_0	BY927930
E_EL_fcP8_11G10_F_0	BY919571
E_EL_fcP8_11G10_R_0	BY927931
E_EL_fcP8_11G11_F_0	BY919572
E_EL_fcP8_11G11_R_0	BY927932
E_EL_fcP8_11G12_F_0	BY919573
E_EL_fcP8_11G12_R_0	BY927933
E_EL_fcP8_11H01_F_0	BY919574
E_EL_fcP8_11H01_R_0	BY927934
E_EL_fcP8_11H02_R_0	BY927935
E_EL_fcP8_11H03_F_0	BY919575
E_EL_fcP8_11H03_R_0	BY927936
E_EL_fcP8_11H04_F_0	BY919576
E_EL_fcP8_11H04_R_0	BY927937
E_EL_fcP8_11H05_F_0	BY919577
E_EL_fcP8_11H05_R_0	BY927938
E_EL_fcP8_11H06_F_0	BY919578
E_EL_fcP8_11H06_R_0	BY927939
E_EL_fcP8_11H08_F_0	BY919579
E_EL_fcP8_11H08_R_0	BY927940
E_EL_fcP8_11H09_F_0	BY919580
E_EL_fcP8_11H11_F_0	BY919581
E_EL_fcP8_11H11_R_0	BY927941
E_EL_fcP8_12A01_R_0	BY927942
E_EL_fcP8_12A02_F_0	BY919582
E_EL_fcP8_12A02_R_0	BY927943
E_EL_fcP8_12A04_F_0	BY919583
E_EL_fcP8_12A04_R_0	BY927944
E_EL_fcP8_12A05_F_0	BY919584
E_EL_fcP8_12A05_R_0	BY927945
E_EL_fcP8_12A06_F_0	BY919585
E_EL_fcP8_12A06_R_0	BY927946
E_EL_fcP8_12A07_F_0	BY919586
E_EL_fcP8_12A07_R_0	BY927947
E_EL_fcP8_12A09_F_0	BY919587
E_EL_fcP8_12A09_R_0	BY927948
E_EL_fcP8_12A10_R_0	BY927949
E_EL_fcP8_12A11_F_0	BY919588
E_EL_fcP8_12A12_F_0	BY919589
E_EL_fcP8_12B01_F_0	BY919590
E_EL_fcP8_12B01_R_0	BY927950
E_EL_fcP8_12B02_F_0	BY919591
E_EL_fcP8_12B02_R_0	BY927951
E_EL_fcP8_12B03_F_0	BY919592
E_EL_fcP8_12B03_R_0	BY927952
E_EL_fcP8_12B04_F_0	BY919593
E_EL_fcP8_12B04_R_0	BY927953
E_EL_fcP8_12B05_F_0	BY919594
E_EL_fcP8_12B05_R_0	BY927954
E_EL_fcP8_12B06_F_0	BY919595
E_EL_fcP8_12B06_R_0	BY927955
E_EL_fcP8_12B07_R_0	BY927956
E_EL_fcP8_12B08_R_0	BY927957
E_EL_fcP8_12B09_R_0	BY927958

E_EL_fcP8_12B10_F_0	BY919596
E_EL_fcP8_12B10_R_0	BY927959
E_EL_fcP8_12B11_F_0	BY919597
E_EL_fcP8_12B12_F_0	BY919598
E_EL_fcP8_12B12_R_0	BY927960
E_EL_fcP8_12C01_F_0	BY919599
E_EL_fcP8_12C01_R_0	BY927961
E_EL_fcP8_12C02_F_0	BY919600
E_EL_fcP8_12C02_R_0	BY927962
E_EL_fcP8_12C03_F_0	BY919601
E_EL_fcP8_12C03_R_0	BY927963
E_EL_fcP8_12C04_F_0	BY919602
E_EL_fcP8_12C04_R_0	BY927964
E_EL_fcP8_12C05_F_0	BY919603
E_EL_fcP8_12C05_R_0	BY927965
E_EL_fcP8_12C06_F_0	BY919604
E_EL_fcP8_12C07_F_0	BY919605
E_EL_fcP8_12C07_R_0	BY927966
E_EL_fcP8_12C08_R_0	BY927967
E_EL_fcP8_12C09_F_0	BY919606
E_EL_fcP8_12C09_R_0	BY927968
E_EL_fcP8_12C10_F_0	BY919607
E_EL_fcP8_12C10_R_0	BY927969
E_EL_fcP8_12C11_F_0	BY919608
E_EL_fcP8_12C11_R_0	BY927970
E_EL_fcP8_12C12_F_0	BY919609
E_EL_fcP8_12C12_R_0	BY927971
E_EL_fcP8_12D01_F_0	BY919610
E_EL_fcP8_12D01_R_0	BY927972
E_EL_fcP8_12D02_F_0	BY919611
E_EL_fcP8_12D02_R_0	BY927973
E_EL_fcP8_12D03_R_0	BY927974
E_EL_fcP8_12D04_F_0	BY919612
E_EL_fcP8_12D04_R_0	BY927975
E_EL_fcP8_12D05_F_0	BY919613
E_EL_fcP8_12D05_R_0	BY927976
E_EL_fcP8_12D06_F_0	BY919614
E_EL_fcP8_12D06_R_0	BY927977
E_EL_fcP8_12D07_F_0	BY919615
E_EL_fcP8_12D07_R_0	BY927978
E_EL_fcP8_12D09_F_0	BY919616
E_EL_fcP8_12D09_R_0	BY927979
E_EL_fcP8_12D10_F_0	BY919617
E_EL_fcP8_12D10_R_0	BY927980
E_EL_fcP8_12D11_F_0	BY919618
E_EL_fcP8_12D11_R_0	BY927981
E_EL_fcP8_12D12_R_0	BY927982
E_EL_fcP8_12E01_R_0	BY927983
E_EL_fcP8_12E02_F_0	BY919619
E_EL_fcP8_12E02_R_0	BY927984
E_EL_fcP8_12E03_F_0	BY919620
E_EL_fcP8_12E03_R_0	BY927985
E_EL_fcP8_12E04_F_0	BY919621
E_EL_fcP8_12E04_R_0	BY927986

E_EL_fcP8_12E05_R_0	BY927987
E_EL_fcP8_12E06_F_0	BY919622
E_EL_fcP8_12E06_R_0	BY927988
E_EL_fcP8_12E07_F_0	BY919623
E_EL_fcP8_12E07_R_0	BY927989
E_EL_fcP8_12E08_F_0	BY919624
E_EL_fcP8_12E08_R_0	BY927990
E_EL_fcP8_12E09_F_0	BY919625
E_EL_fcP8_12E09_R_0	BY927991
E_EL_fcP8_12E10_F_0	BY919626
E_EL_fcP8_12E10_R_0	BY927992
E_EL_fcP8_12E11_F_0	BY919627
E_EL_fcP8_12E11_R_0	BY927993
E_EL_fcP8_12E12_F_0	BY919628
E_EL_fcP8_12E12_R_0	BY927994
E_EL_fcP8_12F01_F_0	BY919629
E_EL_fcP8_12F01_R_0	BY927995
E_EL_fcP8_12F02_R_0	BY927996
E_EL_fcP8_12F03_F_0	BY919630
E_EL_fcP8_12F03_R_0	BY927997
E_EL_fcP8_12F04_F_0	BY919631
E_EL_fcP8_12F04_R_0	BY927998
E_EL_fcP8_12F05_F_0	BY919632
E_EL_fcP8_12F06_F_0	BY919633
E_EL_fcP8_12F07_R_0	BY927999
E_EL_fcP8_12F08_F_0	BY919634
E_EL_fcP8_12F08_R_0	BY928000
E_EL_fcP8_12F10_F_0	BY919635
E_EL_fcP8_12F10_R_0	BY928001
E_EL_fcP8_12F12_F_0	BY919636
E_EL_fcP8_12F12_R_0	BY928002
E_EL_fcP8_12G01_F_0	BY919637
E_EL_fcP8_12G01_R_0	BY928003
E_EL_fcP8_12G02_F_0	BY919638
E_EL_fcP8_12G02_R_0	BY928004
E_EL_fcP8_12G03_F_0	BY919639
E_EL_fcP8_12G03_R_0	BY928005
E_EL_fcP8_12G04_F_0	BY919640
E_EL_fcP8_12G04_R_0	BY928006
E_EL_fcP8_12G05_F_0	BY919641
E_EL_fcP8_12G05_R_0	BY928007
E_EL_fcP8_12G06_F_0	BY919642
E_EL_fcP8_12G06_R_0	BY928008
E_EL_fcP8_12G07_F_0	BY919643
E_EL_fcP8_12G07_R_0	BY928009
E_EL_fcP8_12G08_F_0	BY919644
E_EL_fcP8_12G08_R_0	BY928010
E_EL_fcP8_12G09_F_0	BY919645
E_EL_fcP8_12G09_R_0	BY928011
E_EL_fcP8_12G11_F_0	BY919646
E_EL_fcP8_12G11_R_0	BY928012
E_EL_fcP8_12G12_R_0	BY928013
E_EL_fcP8_12H01_F_0	BY919647
E_EL_fcP8_12H01_R_0	BY928014

E_EL_fcP8_12H02_F_0	BY919648
E_EL_fcP8_12H02_R_0	BY928015
E_EL_fcP8_12H03_F_0	BY919649
E_EL_fcP8_12H03_R_0	BY928016
E_EL_fcP8_12H04_F_0	BY919650
E_EL_fcP8_12H04_R_0	BY928017
E_EL_fcP8_12H05_F_0	BY919651
E_EL_fcP8_12H05_R_0	BY928018
E_EL_fcP8_12H06_F_0	BY919652
E_EL_fcP8_12H06_R_0	BY928019
E_EL_fcP8_12H07_F_0	BY919653
E_EL_fcP8_12H07_R_0	BY928020
E_EL_fcP8_12H08_F_0	BY919654
E_EL_fcP8_12H08_R_0	BY928021
E_EL_fcP8_12H09_F_0	BY919655
E_EL_fcP8_12H09_R_0	BY928022
E_EL_fcP8_12H10_R_0	BY928023
E_EL_fcP8_13A01_F_0	BY919656
E_EL_fcP8_13A01_R_0	BY928024
E_EL_fcP8_13A02_F_0	BY919657
E_EL_fcP8_13A02_R_0	BY928025
E_EL_fcP8_13A03_R_0	BY928026
E_EL_fcP8_13A04_F_0	BY919658
E_EL_fcP8_13A04_R_0	BY928027
E_EL_fcP8_13A07_R_0	BY928028
E_EL_fcP8_13A08_F_0	BY919659
E_EL_fcP8_13A08_R_0	BY928029
E_EL_fcP8_13A09_F_0	BY919660
E_EL_fcP8_13A09_R_0	BY928030
E_EL_fcP8_13A10_F_0	BY919661
E_EL_fcP8_13A10_R_0	BY928031
E_EL_fcP8_13A11_F_0	BY919662
E_EL_fcP8_13A11_R_0	BY928032
E_EL_fcP8_13A12_R_0	BY928033
E_EL_fcP8_13B01_F_0	BY919663
E_EL_fcP8_13B01_R_0	BY928034
E_EL_fcP8_13B02_F_0	BY919664
E_EL_fcP8_13B02_R_0	BY928035
E_EL_fcP8_13B03_F_0	BY919665
E_EL_fcP8_13B03_R_0	BY928036
E_EL_fcP8_13B04_F_0	BY919666
E_EL_fcP8_13B04_R_0	BY928037
E_EL_fcP8_13B05_F_0	BY919667
E_EL_fcP8_13B05_R_0	BY928038
E_EL_fcP8_13B06_F_0	BY919668
E_EL_fcP8_13B07_R_0	BY928039
E_EL_fcP8_13B08_F_0	BY919669
E_EL_fcP8_13B08_R_0	BY928040
E_EL_fcP8_13B09_F_0	BY919670
E_EL_fcP8_13B09_R_0	BY928041
E_EL_fcP8_13B10_F_0	BY919671
E_EL_fcP8_13B10_R_0	BY928042
E_EL_fcP8_13B11_F_0	BY919672
E_EL_fcP8_13B11_R_0	BY928043

E_EL_fcP8_13B12_R_0	BY928044
E_EL_fcP8_13C01_R_0	BY928045
E_EL_fcP8_13C02_F_0	BY919673
E_EL_fcP8_13C02_R_0	BY928046
E_EL_fcP8_13C03_F_0	BY919674
E_EL_fcP8_13C03_R_0	BY928047
E_EL_fcP8_13C04_F_0	BY919675
E_EL_fcP8_13C04_R_0	BY928048
E_EL_fcP8_13C05_F_0	BY919676
E_EL_fcP8_13C05_R_0	BY928049
E_EL_fcP8_13C06_F_0	BY919677
E_EL_fcP8_13C06_R_0	BY928050
E_EL_fcP8_13C07_F_0	BY919678
E_EL_fcP8_13C07_R_0	BY928051
E_EL_fcP8_13C08_F_0	BY919679
E_EL_fcP8_13C08_R_0	BY928052
E_EL_fcP8_13C09_F_0	BY919680
E_EL_fcP8_13C09_R_0	BY928053
E_EL_fcP8_13C10_F_0	BY919681
E_EL_fcP8_13C10_R_0	BY928054
E_EL_fcP8_13C11_F_0	BY919682
E_EL_fcP8_13C11_R_0	BY928055
E_EL_fcP8_13C12_F_0	BY919683
E_EL_fcP8_13C12_R_0	BY928056
E_EL_fcP8_13D01_R_0	BY928057
E_EL_fcP8_13D02_F_0	BY919684
E_EL_fcP8_13D02_R_0	BY928058
E_EL_fcP8_13D03_F_0	BY919685
E_EL_fcP8_13D03_R_0	BY928059
E_EL_fcP8_13D04_F_0	BY919686
E_EL_fcP8_13D04_R_0	BY928060
E_EL_fcP8_13D05_F_0	BY919687
E_EL_fcP8_13D05_R_0	BY928061
E_EL_fcP8_13D06_F_0	BY919688
E_EL_fcP8_13D06_R_0	BY928062
E_EL_fcP8_13D07_F_0	BY919689
E_EL_fcP8_13D07_R_0	BY928063
E_EL_fcP8_13D08_F_0	BY919690
E_EL_fcP8_13D08_R_0	BY928064
E_EL_fcP8_13D09_F_0	BY919691
E_EL_fcP8_13D09_R_0	BY928065
E_EL_fcP8_13D10_F_0	BY919692
E_EL_fcP8_13D10_R_0	BY928066
E_EL_fcP8_13D11_F_0	BY919693
E_EL_fcP8_13D11_R_0	BY928067
E_EL_fcP8_13D12_R_0	BY928068
E_EL_fcP8_13E04_F_0	BY919694
E_EL_fcP8_13E04_R_0	BY928069
E_EL_fcP8_13E05_F_0	BY919695
E_EL_fcP8_13E05_R_0	BY928070
E_EL_fcP8_13E06_F_0	BY919696
E_EL_fcP8_13E06_R_0	BY928071
E_EL_fcP8_13E07_F_0	BY919697
E_EL_fcP8_13E07_R_0	BY928072

E_EL_fcP8_13E08_F_0	BY919698
E_EL_fcP8_13E08_R_0	BY928073
E_EL_fcP8_13E10_F_0	BY919699
E_EL_fcP8_13E10_R_0	BY928074
E_EL_fcP8_13E11_F_0	BY919700
E_EL_fcP8_13E11_R_0	BY928075
E_EL_fcP8_13F01_F_0	BY919701
E_EL_fcP8_13F01_R_0	BY928076
E_EL_fcP8_13F02_F_0	BY919702
E_EL_fcP8_13F02_R_0	BY928077
E_EL_fcP8_13F03_F_0	BY919703
E_EL_fcP8_13F03_R_0	BY928078
E_EL_fcP8_13F05_F_0	BY919704
E_EL_fcP8_13F05_R_0	BY928079
E_EL_fcP8_13F06_F_0	BY919705
E_EL_fcP8_13F07_F_0	BY919706
E_EL_fcP8_13F07_R_0	BY928080
E_EL_fcP8_13F08_R_0	BY928081
E_EL_fcP8_13F09_F_0	BY919707
E_EL_fcP8_13F09_R_0	BY928082
E_EL_fcP8_13F10_F_0	BY919708
E_EL_fcP8_13F10_R_0	BY928083
E_EL_fcP8_13F11_F_0	BY919709
E_EL_fcP8_13F11_R_0	BY928084
E_EL_fcP8_13F12_F_0	BY919710
E_EL_fcP8_13F12_R_0	BY928085
E_EL_fcP8_13G02_F_0	BY919711
E_EL_fcP8_13G02_R_0	BY928086
E_EL_fcP8_13G03_F_0	BY919712
E_EL_fcP8_13G03_R_0	BY928087
E_EL_fcP8_13G04_F_0	BY919713
E_EL_fcP8_13G04_R_0	BY928088
E_EL_fcP8_13G05_F_0	BY919714
E_EL_fcP8_13G05_R_0	BY928089
E_EL_fcP8_13G07_F_0	BY919715
E_EL_fcP8_13G07_R_0	BY928090
E_EL_fcP8_13G09_F_0	BY919716
E_EL_fcP8_13G09_R_0	BY928091
E_EL_fcP8_13G10_F_0	BY919717
E_EL_fcP8_13G10_R_0	BY928092
E_EL_fcP8_13G11_F_0	BY919718
E_EL_fcP8_13G11_R_0	BY928093
E_EL_fcP8_13G12_R_0	BY928094
E_EL_fcP8_13H02_F_0	BY919719
E_EL_fcP8_13H02_R_0	BY928095
E_EL_fcP8_13H03_F_0	BY919720
E_EL_fcP8_13H03_R_0	BY928096
E_EL_fcP8_13H04_F_0	BY919721
E_EL_fcP8_13H04_R_0	BY928097
E_EL_fcP8_13H05_F_0	BY919722
E_EL_fcP8_13H05_R_0	BY928098
E_EL_fcP8_13H06_F_0	BY919723
E_EL_fcP8_13H06_R_0	BY928099
E_EL_fcP8_13H07_F_0	BY919724

E_EL_fcP8_13H07_R_0	BY928100
E_EL_fcP8_13H08_F_0	BY919725
E_EL_fcP8_13H08_R_0	BY928101
E_EL_fcP8_13H09_F_0	BY919726
E_EL_fcP8_13H09_R_0	BY928102
E_EL_fcP8_13H10_F_0	BY919727
E_EL_fcP8_13H10_R_0	BY928103
E_EL_fcP8_13H11_R_0	BY928104
E_EL_fcP8_13H12_F_0	BY919728
E_EL_fcP8_13H12_R_0	BY928105
E_EL_fcP8_14A01_F_0	BY919729
E_EL_fcP8_14A01_R_0	BY928106
E_EL_fcP8_14A02_F_0	BY919730
E_EL_fcP8_14A02_R_0	BY928107
E_EL_fcP8_14A03_R_0	BY928108
E_EL_fcP8_14A04_F_0	BY919731
E_EL_fcP8_14A04_R_0	BY928109
E_EL_fcP8_14A05_F_0	BY919732
E_EL_fcP8_14A05_R_0	BY928110
E_EL_fcP8_14A06_F_0	BY919733
E_EL_fcP8_14A06_R_0	BY928111
E_EL_fcP8_14A07_F_0	BY919734
E_EL_fcP8_14A07_R_0	BY928112
E_EL_fcP8_14A08_F_0	BY919735
E_EL_fcP8_14A08_R_0	BY928113
E_EL_fcP8_14A09_F_0	BY919736
E_EL_fcP8_14A09_R_0	BY928114
E_EL_fcP8_14A10_F_0	BY919737
E_EL_fcP8_14A10_R_0	BY928115
E_EL_fcP8_14A11_F_0	BY919738
E_EL_fcP8_14A11_R_0	BY928116
E_EL_fcP8_14A12_F_0	BY919739
E_EL_fcP8_14A12_R_0	BY928117
E_EL_fcP8_14B01_F_0	BY919740
E_EL_fcP8_14B01_R_0	BY928118
E_EL_fcP8_14B02_F_0	BY919741
E_EL_fcP8_14B02_R_0	BY928119
E_EL_fcP8_14B03_F_0	BY919742
E_EL_fcP8_14B03_R_0	BY928120
E_EL_fcP8_14B04_F_0	BY919743
E_EL_fcP8_14B05_F_0	BY919744
E_EL_fcP8_14B05_R_0	BY928121
E_EL_fcP8_14B06_F_0	BY919745
E_EL_fcP8_14B06_R_0	BY928122
E_EL_fcP8_14B07_F_0	BY919746
E_EL_fcP8_14B07_R_0	BY928123
E_EL_fcP8_14B08_F_0	BY919747
E_EL_fcP8_14B08_R_0	BY928124
E_EL_fcP8_14B10_F_0	BY919748
E_EL_fcP8_14B10_R_0	BY928125
E_EL_fcP8_14B11_R_0	BY928126
E_EL_fcP8_14B12_F_0	BY919749
E_EL_fcP8_14B12_R_0	BY928127
E_EL_fcP8_14C01_F_0	BY919750

E_EL_fcP8_14C01_R_0	BY928128
E_EL_fcP8_14C02_F_0	BY919751
E_EL_fcP8_14C02_R_0	BY928129
E_EL_fcP8_14C03_F_0	BY919752
E_EL_fcP8_14C03_R_0	BY928130
E_EL_fcP8_14C04_F_0	BY919753
E_EL_fcP8_14C04_R_0	BY928131
E_EL_fcP8_14C05_F_0	BY919754
E_EL_fcP8_14C05_R_0	BY928132
E_EL_fcP8_14C06_F_0	BY919755
E_EL_fcP8_14C06_R_0	BY928133
E_EL_fcP8_14C07_F_0	BY919756
E_EL_fcP8_14C07_R_0	BY928134
E_EL_fcP8_14C08_F_0	BY919757
E_EL_fcP8_14C08_R_0	BY928135
E_EL_fcP8_14C09_F_0	BY919758
E_EL_fcP8_14C09_R_0	BY928136
E_EL_fcP8_14C10_F_0	BY919759
E_EL_fcP8_14C10_R_0	BY928137
E_EL_fcP8_14C11_F_0	BY919760
E_EL_fcP8_14C11_R_0	BY928138
E_EL_fcP8_14C12_F_0	BY919761
E_EL_fcP8_14C12_R_0	BY928139
E_EL_fcP8_14D01_F_0	BY919762
E_EL_fcP8_14D01_R_0	BY928140
E_EL_fcP8_14D02_F_0	BY919763
E_EL_fcP8_14D02_R_0	BY928141
E_EL_fcP8_14D03_F_0	BY919764
E_EL_fcP8_14D03_R_0	BY928142
E_EL_fcP8_14D04_F_0	BY919765
E_EL_fcP8_14D04_R_0	BY928143
E_EL_fcP8_14D05_F_0	BY919766
E_EL_fcP8_14D05_R_0	BY928144
E_EL_fcP8_14D06_F_0	BY919767
E_EL_fcP8_14D06_R_0	BY928145
E_EL_fcP8_14D07_R_0	BY928146
E_EL_fcP8_14D09_R_0	BY928147
E_EL_fcP8_14D10_F_0	BY919768
E_EL_fcP8_14D11_F_0	BY919769
E_EL_fcP8_14D11_R_0	BY928148
E_EL_fcP8_14D12_F_0	BY919770
E_EL_fcP8_14D12_R_0	BY928149
E_EL_fcP8_14E01_F_0	BY919771
E_EL_fcP8_14E01_R_0	BY928150
E_EL_fcP8_14E02_F_0	BY919772
E_EL_fcP8_14E02_R_0	BY928151
E_EL_fcP8_14E03_F_0	BY919773
E_EL_fcP8_14E03_R_0	BY928152
E_EL_fcP8_14E06_F_0	BY919774
E_EL_fcP8_14E06_R_0	BY928153
E_EL_fcP8_14E07_F_0	BY919775
E_EL_fcP8_14E07_R_0	BY928154
E_EL_fcP8_14E08_F_0	BY919776
E_EL_fcP8_14E08_R_0	BY928155

E_EL_fcP8_14E09_F_0	BY919777
E_EL_fcP8_14E09_R_0	BY928156
E_EL_fcP8_14E10_F_0	BY919778
E_EL_fcP8_14E10_R_0	BY928157
E_EL_fcP8_14E11_F_0	BY919779
E_EL_fcP8_14E11_R_0	BY928158
E_EL_fcP8_14E12_F_0	BY919780
E_EL_fcP8_14E12_R_0	BY928159
E_EL_fcP8_14F01_F_0	BY919781
E_EL_fcP8_14F01_R_0	BY928160
E_EL_fcP8_14F02_F_0	BY919782
E_EL_fcP8_14F02_R_0	BY928161
E_EL_fcP8_14F03_F_0	BY919783
E_EL_fcP8_14F03_R_0	BY928162
E_EL_fcP8_14F04_F_0	BY919784
E_EL_fcP8_14F04_R_0	BY928163
E_EL_fcP8_14F05_F_0	BY919785
E_EL_fcP8_14F05_R_0	BY928164
E_EL_fcP8_14F06_F_0	BY919786
E_EL_fcP8_14F06_R_0	BY928165
E_EL_fcP8_14F07_F_0	BY919787
E_EL_fcP8_14F07_R_0	BY928166
E_EL_fcP8_14F08_F_0	BY919788
E_EL_fcP8_14F08_R_0	BY928167
E_EL_fcP8_14F09_F_0	BY919789
E_EL_fcP8_14F09_R_0	BY928168
E_EL_fcP8_14F11_F_0	BY919790
E_EL_fcP8_14F11_R_0	BY928169
E_EL_fcP8_14F12_F_0	BY919791
E_EL_fcP8_14F12_R_0	BY928170
E_EL_fcP8_14G01_F_0	BY919792
E_EL_fcP8_14G01_R_0	BY928171
E_EL_fcP8_14G02_F_0	BY919793
E_EL_fcP8_14G02_R_0	BY928172
E_EL_fcP8_14G03_F_0	BY919794
E_EL_fcP8_14G03_R_0	BY928173
E_EL_fcP8_14G04_F_0	BY919795
E_EL_fcP8_14G04_R_0	BY928174
E_EL_fcP8_14G05_F_0	BY919796
E_EL_fcP8_14G05_R_0	BY928175
E_EL_fcP8_14G06_F_0	BY919797
E_EL_fcP8_14G06_R_0	BY928176
E_EL_fcP8_14G07_R_0	BY928177
E_EL_fcP8_14G08_F_0	BY919798
E_EL_fcP8_14G08_R_0	BY928178
E_EL_fcP8_14G09_R_0	BY928179
E_EL_fcP8_14G10_F_0	BY919799
E_EL_fcP8_14G10_R_0	BY928180
E_EL_fcP8_14G11_R_0	BY928181
E_EL_fcP8_14G12_F_0	BY919800
E_EL_fcP8_14G12_R_0	BY928182
E_EL_fcP8_14H01_F_0	BY919801
E_EL_fcP8_14H01_R_0	BY928183
E_EL_fcP8_14H02_F_0	BY919802

E_EL_fcP8_14H02_R_0	BY928184
E_EL_fcP8_14H03_R_0	BY928185
E_EL_fcP8_14H04_F_0	BY919803
E_EL_fcP8_14H04_R_0	BY928186
E_EL_fcP8_14H05_F_0	BY919804
E_EL_fcP8_14H05_R_0	BY928187
E_EL_fcP8_14H06_F_0	BY919805
E_EL_fcP8_14H06_R_0	BY928188
E_EL_fcP8_14H07_F_0	BY919806
E_EL_fcP8_14H07_R_0	BY928189
E_EL_fcP8_14H08_F_0	BY919807
E_EL_fcP8_14H08_R_0	BY928190
E_EL_fcP8_14H09_F_0	BY919808
E_EL_fcP8_14H09_R_0	BY928191
E_EL_fcP8_14H11_F_0	BY919809
E_EL_fcP8_14H11_R_0	BY928192
E_EL_fcP8_14H12_F_0	BY919810
E_EL_fcP8_14H12_R_0	BY928193
E_EL_fcP8_15A01_F_0	BY919811
E_EL_fcP8_15A01_R_0	BY928194
E_EL_fcP8_15A02_F_0	BY919812
E_EL_fcP8_15A02_R_0	BY928195
E_EL_fcP8_15A03_F_0	BY919813
E_EL_fcP8_15A03_R_0	BY928196
E_EL_fcP8_15A04_F_0	BY919814
E_EL_fcP8_15A04_R_0	BY928197
E_EL_fcP8_15A05_R_0	BY928198
E_EL_fcP8_15A06_F_0	BY919815
E_EL_fcP8_15A06_R_0	BY928199
E_EL_fcP8_15A07_F_0	BY919816
E_EL_fcP8_15A07_R_0	BY928200
E_EL_fcP8_15A08_F_0	BY919817
E_EL_fcP8_15A08_R_0	BY928201
E_EL_fcP8_15A09_F_0	BY919818
E_EL_fcP8_15A09_R_0	BY928202
E_EL_fcP8_15A10_F_0	BY919819
E_EL_fcP8_15A10_R_0	BY928203
E_EL_fcP8_15A11_F_0	BY919820
E_EL_fcP8_15A11_R_0	BY928204
E_EL_fcP8_15A12_F_0	BY919821
E_EL_fcP8_15A12_R_0	BY928205
E_EL_fcP8_15B01_F_0	BY919822
E_EL_fcP8_15B01_R_0	BY928206
E_EL_fcP8_15B02_F_0	BY919823
E_EL_fcP8_15B02_R_0	BY928207
E_EL_fcP8_15B03_F_0	BY919824
E_EL_fcP8_15B04_F_0	BY919825
E_EL_fcP8_15B04_R_0	BY928208
E_EL_fcP8_15B05_F_0	BY919826
E_EL_fcP8_15B05_R_0	BY928209
E_EL_fcP8_15B06_R_0	BY928210
E_EL_fcP8_15B07_F_0	BY919827
E_EL_fcP8_15B07_R_0	BY928211
E_EL_fcP8_15B08_F_0	BY919828

E_EL_fcP8_15B08_R_0	BY928212
E_EL_fcP8_15B09_F_0	BY919829
E_EL_fcP8_15B10_F_0	BY919830
E_EL_fcP8_15B10_R_0	BY928213
E_EL_fcP8_15B11_F_0	BY919831
E_EL_fcP8_15B11_R_0	BY928214
E_EL_fcP8_15B12_F_0	BY919832
E_EL_fcP8_15B12_R_0	BY928215
E_EL_fcP8_15C01_F_0	BY919833
E_EL_fcP8_15C01_R_0	BY928216
E_EL_fcP8_15C02_F_0	BY919834
E_EL_fcP8_15C02_R_0	BY928217
E_EL_fcP8_15C03_F_0	BY919835
E_EL_fcP8_15C03_R_0	BY928218
E_EL_fcP8_15C04_R_0	BY928219
E_EL_fcP8_15C05_F_0	BY919836
E_EL_fcP8_15C05_R_0	BY928220
E_EL_fcP8_15C06_F_0	BY919837
E_EL_fcP8_15C07_F_0	BY919838
E_EL_fcP8_15C07_R_0	BY928221
E_EL_fcP8_15C08_F_0	BY919839
E_EL_fcP8_15C08_R_0	BY928222
E_EL_fcP8_15C10_F_0	BY919840
E_EL_fcP8_15C10_R_0	BY928223
E_EL_fcP8_15C11_F_0	BY919841
E_EL_fcP8_15C11_R_0	BY928224
E_EL_fcP8_15C12_F_0	BY919842
E_EL_fcP8_15C12_R_0	BY928225
E_EL_fcP8_15D01_F_0	BY919843
E_EL_fcP8_15D01_R_0	BY928226
E_EL_fcP8_15D02_F_0	BY919844
E_EL_fcP8_15D02_R_0	BY928227
E_EL_fcP8_15D03_F_0	BY919845
E_EL_fcP8_15D03_R_0	BY928228
E_EL_fcP8_15D04_F_0	BY919846
E_EL_fcP8_15D04_R_0	BY928229
E_EL_fcP8_15D05_F_0	BY919847
E_EL_fcP8_15D05_R_0	BY928230
E_EL_fcP8_15D06_R_0	BY928231
E_EL_fcP8_15D07_F_0	BY919848
E_EL_fcP8_15D07_R_0	BY928232
E_EL_fcP8_15D09_F_0	BY919849
E_EL_fcP8_15D09_R_0	BY928233
E_EL_fcP8_15D10_F_0	BY919850
E_EL_fcP8_15D10_R_0	BY928234
E_EL_fcP8_15D11_F_0	BY919851
E_EL_fcP8_15D11_R_0	BY928235
E_EL_fcP8_15D12_F_0	BY919852
E_EL_fcP8_15E01_F_0	BY919853
E_EL_fcP8_15E01_R_0	BY928236
E_EL_fcP8_15E02_F_0	BY919854
E_EL_fcP8_15E03_F_0	BY919855
E_EL_fcP8_15E03_R_0	BY928237
E_EL_fcP8_15E04_F_0	BY919856

E_EL_fcP8_15E04_R_0	BY928238
E_EL_fcP8_15E05_F_0	BY919857
E_EL_fcP8_15E05_R_0	BY928239
E_EL_fcP8_15E07_F_0	BY919858
E_EL_fcP8_15E07_R_0	BY928240
E_EL_fcP8_15E08_F_0	BY919859
E_EL_fcP8_15E08_R_0	BY928241
E_EL_fcP8_15E10_F_0	BY919860
E_EL_fcP8_15E10_R_0	BY928242
E_EL_fcP8_15E11_F_0	BY919861
E_EL_fcP8_15E11_R_0	BY928243
E_EL_fcP8_15F01_R_0	BY928244
E_EL_fcP8_15F02_F_0	BY919862
E_EL_fcP8_15F02_R_0	BY928245
E_EL_fcP8_15F04_R_0	BY928246
E_EL_fcP8_15F05_F_0	BY919863
E_EL_fcP8_15F05_R_0	BY928247
E_EL_fcP8_15F06_F_0	BY919864
E_EL_fcP8_15F06_R_0	BY928248
E_EL_fcP8_15F07_F_0	BY919865
E_EL_fcP8_15F07_R_0	BY928249
E_EL_fcP8_15F08_R_0	BY928250
E_EL_fcP8_15F09_F_0	BY919866
E_EL_fcP8_15F09_R_0	BY928251
E_EL_fcP8_15F10_F_0	BY919867
E_EL_fcP8_15F11_F_0	BY919868
E_EL_fcP8_15F11_R_0	BY928252
E_EL_fcP8_15F12_F_0	BY919869
E_EL_fcP8_15F12_R_0	BY928253
E_EL_fcP8_15G01_F_0	BY919870
E_EL_fcP8_15G01_R_0	BY928254
E_EL_fcP8_15G03_F_0	BY919871
E_EL_fcP8_15G03_R_0	BY928255
E_EL_fcP8_15G04_F_0	BY919872
E_EL_fcP8_15G04_R_0	BY928256
E_EL_fcP8_15G05_F_0	BY919873
E_EL_fcP8_15G05_R_0	BY928257
E_EL_fcP8_15G06_F_0	BY919874
E_EL_fcP8_15G06_R_0	BY928258
E_EL_fcP8_15G07_F_0	BY919875
E_EL_fcP8_15G07_R_0	BY928259
E_EL_fcP8_15G08_F_0	BY919876
E_EL_fcP8_15G08_R_0	BY928260
E_EL_fcP8_15G10_F_0	BY919877
E_EL_fcP8_15G10_R_0	BY928261
E_EL_fcP8_15G11_F_0	BY919878
E_EL_fcP8_15G11_R_0	BY928262
E_EL_fcP8_15G12_F_0	BY919879
E_EL_fcP8_15G12_R_0	BY928263
E_EL_fcP8_15H01_F_0	BY919880
E_EL_fcP8_15H01_R_0	BY928264
E_EL_fcP8_15H02_F_0	BY919881
E_EL_fcP8_15H02_R_0	BY928265
E_EL_fcP8_15H03_F_0	BY919882

E_EL_fcP8_15H03_R_0	BY928266
E_EL_fcP8_15H04_F_0	BY919883
E_EL_fcP8_15H04_R_0	BY928267
E_EL_fcP8_15H06_F_0	BY919884
E_EL_fcP8_15H06_R_0	BY928268
E_EL_fcP8_15H07_F_0	BY919885
E_EL_fcP8_15H07_R_0	BY928269
E_EL_fcP8_15H08_F_0	BY919886
E_EL_fcP8_15H08_R_0	BY928270
E_EL_fcP8_15H09_F_0	BY919887
E_EL_fcP8_15H09_R_0	BY928271
E_EL_fcP8_15H10_F_0	BY919888
E_EL_fcP8_15H11_F_0	BY919889
E_EL_fcP8_15H11_R_0	BY928272
E_EL_fcP8_15H12_F_0	BY919890
E_EL_fcP8_16A01_F_0	BY919891
E_EL_fcP8_16A01_R_0	BY928273
E_EL_fcP8_16A02_R_0	BY928274
E_EL_fcP8_16A03_F_0	BY919892
E_EL_fcP8_16A03_R_0	BY928275
E_EL_fcP8_16A04_R_0	BY928276
E_EL_fcP8_16A05_F_0	BY919893
E_EL_fcP8_16A05_R_0	BY928277
E_EL_fcP8_16A06_F_0	BY919894
E_EL_fcP8_16A06_R_0	BY928278
E_EL_fcP8_16A07_F_0	BY919895
E_EL_fcP8_16A07_R_0	BY928279
E_EL_fcP8_16A08_F_0	BY919896
E_EL_fcP8_16A08_R_0	BY928280
E_EL_fcP8_16A09_F_0	BY919897
E_EL_fcP8_16A09_R_0	BY928281
E_EL_fcP8_16A10_F_0	BY919898
E_EL_fcP8_16A10_R_0	BY928282
E_EL_fcP8_16A12_F_0	BY919899
E_EL_fcP8_16A12_R_0	BY928283
E_EL_fcP8_16B02_F_0	BY919900
E_EL_fcP8_16B02_R_0	BY928284
E_EL_fcP8_16B03_R_0	BY928285
E_EL_fcP8_16B04_F_0	BY919901
E_EL_fcP8_16B04_R_0	BY928286
E_EL_fcP8_16B05_F_0	BY919902
E_EL_fcP8_16B05_R_0	BY928287
E_EL_fcP8_16B06_F_0	BY919903
E_EL_fcP8_16B06_R_0	BY928288
E_EL_fcP8_16B07_F_0	BY919904
E_EL_fcP8_16B07_R_0	BY928289
E_EL_fcP8_16B08_F_0	BY919905
E_EL_fcP8_16B08_R_0	BY928290
E_EL_fcP8_16B09_F_0	BY919906
E_EL_fcP8_16B09_R_0	BY928291
E_EL_fcP8_16B10_F_0	BY919907
E_EL_fcP8_16B10_R_0	BY928292
E_EL_fcP8_16B11_F_0	BY919908
E_EL_fcP8_16B11_R_0	BY928293

E_EL_fcP8_16B12_R_0	BY928294
E_EL_fcP8_16C01_F_0	BY919909
E_EL_fcP8_16C01_R_0	BY928295
E_EL_fcP8_16C02_F_0	BY919910
E_EL_fcP8_16C03_F_0	BY919911
E_EL_fcP8_16C03_R_0	BY928296
E_EL_fcP8_16C04_F_0	BY919912
E_EL_fcP8_16C04_R_0	BY928297
E_EL_fcP8_16C05_F_0	BY919913
E_EL_fcP8_16C05_R_0	BY928298
E_EL_fcP8_16C06_F_0	BY919914
E_EL_fcP8_16C06_R_0	BY928299
E_EL_fcP8_16C07_R_0	BY928300
E_EL_fcP8_16C08_R_0	BY928301
E_EL_fcP8_16C09_F_0	BY919915
E_EL_fcP8_16C09_R_0	BY928302
E_EL_fcP8_16C10_F_0	BY919916
E_EL_fcP8_16C10_R_0	BY928303
E_EL_fcP8_16C11_F_0	BY919917
E_EL_fcP8_16C11_R_0	BY928304
E_EL_fcP8_16C12_F_0	BY919918
E_EL_fcP8_16C12_R_0	BY928305
E_EL_fcP8_16D01_F_0	BY919919
E_EL_fcP8_16D01_R_0	BY928306
E_EL_fcP8_16D02_R_0	BY928307
E_EL_fcP8_16D03_R_0	BY928308
E_EL_fcP8_16D04_F_0	BY919920
E_EL_fcP8_16D04_R_0	BY928309
E_EL_fcP8_16D05_F_0	BY919921
E_EL_fcP8_16D05_R_0	BY928310
E_EL_fcP8_16D06_F_0	BY919922
E_EL_fcP8_16D06_R_0	BY928311
E_EL_fcP8_16D07_F_0	BY919923
E_EL_fcP8_16D07_R_0	BY928312
E_EL_fcP8_16D08_F_0	BY919924
E_EL_fcP8_16D08_R_0	BY928313
E_EL_fcP8_16D09_F_0	BY919925
E_EL_fcP8_16D09_R_0	BY928314
E_EL_fcP8_16D10_F_0	BY919926
E_EL_fcP8_16D10_R_0	BY928315
E_EL_fcP8_16D11_F_0	BY919927
E_EL_fcP8_16D11_R_0	BY928316
E_EL_fcP8_16D12_F_0	BY919928
E_EL_fcP8_16D12_R_0	BY928317
E_EL_fcP8_16E01_F_0	BY919929
E_EL_fcP8_16E01_R_0	BY928318
E_EL_fcP8_16E02_F_0	BY919930
E_EL_fcP8_16E02_R_0	BY928319
E_EL_fcP8_16E03_F_0	BY919931
E_EL_fcP8_16E04_F_0	BY919932
E_EL_fcP8_16E04_R_0	BY928320
E_EL_fcP8_16E06_F_0	BY919933
E_EL_fcP8_16E06_R_0	BY928321
E_EL_fcP8_16E07_F_0	BY919934

E_EL_fcP8_16E07_R_0	BY928322
E_EL_fcP8_16E08_F_0	BY919935
E_EL_fcP8_16E08_R_0	BY928323
E_EL_fcP8_16E09_F_0	BY919936
E_EL_fcP8_16E09_R_0	BY928324
E_EL_fcP8_16E10_F_0	BY919937
E_EL_fcP8_16E10_R_0	BY928325
E_EL_fcP8_16E11_F_0	BY919938
E_EL_fcP8_16E11_R_0	BY928326
E_EL_fcP8_16F01_F_0	BY919939
E_EL_fcP8_16F01_R_0	BY928327
E_EL_fcP8_16F02_F_0	BY919940
E_EL_fcP8_16F02_R_0	BY928328
E_EL_fcP8_16F03_F_0	BY919941
E_EL_fcP8_16F03_R_0	BY928329
E_EL_fcP8_16F04_F_0	BY919942
E_EL_fcP8_16F04_R_0	BY928330
E_EL_fcP8_16F05_F_0	BY919943
E_EL_fcP8_16F05_R_0	BY928331
E_EL_fcP8_16F06_F_0	BY919944
E_EL_fcP8_16F07_F_0	BY919945
E_EL_fcP8_16F07_R_0	BY928332
E_EL_fcP8_16F08_F_0	BY919946
E_EL_fcP8_16F08_R_0	BY928333
E_EL_fcP8_16F09_F_0	BY919947
E_EL_fcP8_16F09_R_0	BY928334
E_EL_fcP8_16F10_F_0	BY919948
E_EL_fcP8_16F10_R_0	BY928335
E_EL_fcP8_16F11_F_0	BY919949
E_EL_fcP8_16F11_R_0	BY928336
E_EL_fcP8_16F12_F_0	BY919950
E_EL_fcP8_16F12_R_0	BY928337
E_EL_fcP8_16G01_F_0	BY919951
E_EL_fcP8_16G01_R_0	BY928338
E_EL_fcP8_16G02_R_0	BY928339
E_EL_fcP8_16G03_F_0	BY919952
E_EL_fcP8_16G03_R_0	BY928340
E_EL_fcP8_16G05_R_0	BY928341
E_EL_fcP8_16G06_F_0	BY919953
E_EL_fcP8_16G06_R_0	BY928342
E_EL_fcP8_16G07_F_0	BY919954
E_EL_fcP8_16G07_R_0	BY928343
E_EL_fcP8_16G08_F_0	BY919955
E_EL_fcP8_16G10_F_0	BY919956
E_EL_fcP8_16G10_R_0	BY928344
E_EL_fcP8_16G11_F_0	BY919957
E_EL_fcP8_16G11_R_0	BY928345
E_EL_fcP8_16G12_F_0	BY919958
E_EL_fcP8_16G12_R_0	BY928346
E_EL_fcP8_16H01_F_0	BY919959
E_EL_fcP8_16H01_R_0	BY928347
E_EL_fcP8_16H02_F_0	BY919960
E_EL_fcP8_16H02_R_0	BY928348
E_EL_fcP8_16H03_F_0	BY919961

E_EL_fcP8_16H03_R_0	BY928349
E_EL_fcP8_16H04_F_0	BY919962
E_EL_fcP8_16H04_R_0	BY928350
E_EL_fcP8_16H05_R_0	BY928351
E_EL_fcP8_16H06_F_0	BY919963
E_EL_fcP8_16H06_R_0	BY928352
E_EL_fcP8_16H08_F_0	BY919964
E_EL_fcP8_16H08_R_0	BY928353
E_EL_fcP8_16H09_F_0	BY919965
E_EL_fcP8_16H09_R_0	BY928354
E_EL_fcP8_16H10_F_0	BY919966
E_EL_fcP8_16H10_R_0	BY928355
E_EL_fcP8_16H11_F_0	BY919967
E_EL_fcP8_16H11_R_0	BY928356
E_EL_fcP8_16H12_R_0	BY928357
E_EL_fcP8_17A01_F_0	BY919968
E_EL_fcP8_17A01_R_0	BY928358
E_EL_fcP8_17A02_R_0	BY928359
E_EL_fcP8_17A03_R_0	BY928360
E_EL_fcP8_17A05_R_0	BY928361
E_EL_fcP8_17A07_R_0	BY928362
E_EL_fcP8_17A08_F_0	BY919969
E_EL_fcP8_17A08_R_0	BY928363
E_EL_fcP8_17A09_R_0	BY928364
E_EL_fcP8_17A10_F_0	BY919970
E_EL_fcP8_17A11_F_0	BY919971
E_EL_fcP8_17A11_R_0	BY928365
E_EL_fcP8_17A12_F_0	BY919972
E_EL_fcP8_17A12_R_0	BY928366
E_EL_fcP8_17B02_F_0	BY919973
E_EL_fcP8_17B02_R_0	BY928367
E_EL_fcP8_17B03_F_0	BY919974
E_EL_fcP8_17B03_R_0	BY928368
E_EL_fcP8_17B04_F_0	BY919975
E_EL_fcP8_17B04_R_0	BY928369
E_EL_fcP8_17B05_F_0	BY919976
E_EL_fcP8_17B06_R_0	BY928370
E_EL_fcP8_17B07_F_0	BY919977
E_EL_fcP8_17B07_R_0	BY928371
E_EL_fcP8_17B08_F_0	BY919978
E_EL_fcP8_17B08_R_0	BY928372
E_EL_fcP8_17B09_F_0	BY919979
E_EL_fcP8_17B09_R_0	BY928373
E_EL_fcP8_17B10_F_0	BY919980
E_EL_fcP8_17B10_R_0	BY928374
E_EL_fcP8_17B11_F_0	BY919981
E_EL_fcP8_17B12_R_0	BY928375
E_EL_fcP8_17C01_F_0	BY919982
E_EL_fcP8_17C01_R_0	BY928376
E_EL_fcP8_17C02_F_0	BY919983
E_EL_fcP8_17C02_R_0	BY928377
E_EL_fcP8_17C03_F_0	BY919984
E_EL_fcP8_17C03_R_0	BY928378
E_EL_fcP8_17C04_R_0	BY928379

E_EL_fcP8_17C05_F_0	BY919985
E_EL_fcP8_17C05_R_0	BY928380
E_EL_fcP8_17C06_R_0	BY928381
E_EL_fcP8_17C07_F_0	BY919986
E_EL_fcP8_17C07_R_0	BY928382
E_EL_fcP8_17C08_F_0	BY919987
E_EL_fcP8_17C08_R_0	BY928383
E_EL_fcP8_17C09_F_0	BY919988
E_EL_fcP8_17C09_R_0	BY928384
E_EL_fcP8_17C10_F_0	BY919989
E_EL_fcP8_17C10_R_0	BY928385
E_EL_fcP8_17C11_F_0	BY919990
E_EL_fcP8_17C11_R_0	BY928386
E_EL_fcP8_17C12_R_0	BY928387
E_EL_fcP8_17D01_R_0	BY928388
E_EL_fcP8_17D02_F_0	BY919991
E_EL_fcP8_17D02_R_0	BY928389
E_EL_fcP8_17D03_R_0	BY928390
E_EL_fcP8_17D04_F_0	BY919992
E_EL_fcP8_17D04_R_0	BY928391
E_EL_fcP8_17D05_F_0	BY919993
E_EL_fcP8_17D05_R_0	BY928392
E_EL_fcP8_17D06_F_0	BY919994
E_EL_fcP8_17D06_R_0	BY928393
E_EL_fcP8_17D07_R_0	BY928394
E_EL_fcP8_17D08_R_0	BY928395
E_EL_fcP8_17D09_F_0	BY919995
E_EL_fcP8_17D09_R_0	BY928396
E_EL_fcP8_17D10_F_0	BY919996
E_EL_fcP8_17D10_R_0	BY928397
E_EL_fcP8_17D11_R_0	BY928398
E_EL_fcP8_17D12_R_0	BY928399
E_EL_fcP8_17E01_F_0	BY919997
E_EL_fcP8_17E01_R_0	BY928400
E_EL_fcP8_17E02_F_0	BY919998
E_EL_fcP8_17E02_R_0	BY928401
E_EL_fcP8_17E03_F_0	BY919999
E_EL_fcP8_17E03_R_0	BY928402
E_EL_fcP8_17E04_F_0	BY920000
E_EL_fcP8_17E04_R_0	BY928403
E_EL_fcP8_17E05_F_0	BY920001
E_EL_fcP8_17E05_R_0	BY928404
E_EL_fcP8_17E06_F_0	BY920002
E_EL_fcP8_17E06_R_0	BY928405
E_EL_fcP8_17E07_F_0	BY920003
E_EL_fcP8_17E07_R_0	BY928406
E_EL_fcP8_17E08_F_0	BY920004
E_EL_fcP8_17E08_R_0	BY928407
E_EL_fcP8_17E09_F_0	BY920005
E_EL_fcP8_17E09_R_0	BY928408
E_EL_fcP8_17E10_R_0	BY928409
E_EL_fcP8_17E11_F_0	BY920006
E_EL_fcP8_17E11_R_0	BY928410
E_EL_fcP8_17F01_F_0	BY920007

E_EL_fcP8_17F01_R_0	BY928411
E_EL_fcP8_17F02_F_0	BY920008
E_EL_fcP8_17F02_R_0	BY928412
E_EL_fcP8_17F03_F_0	BY920009
E_EL_fcP8_17F03_R_0	BY928413
E_EL_fcP8_17F04_F_0	BY920010
E_EL_fcP8_17F04_R_0	BY928414
E_EL_fcP8_17F05_R_0	BY928415
E_EL_fcP8_17F06_F_0	BY920011
E_EL_fcP8_17F06_R_0	BY928416
E_EL_fcP8_17F07_R_0	BY928417
E_EL_fcP8_17F08_F_0	BY920012
E_EL_fcP8_17F08_R_0	BY928418
E_EL_fcP8_17F10_F_0	BY920013
E_EL_fcP8_17F10_R_0	BY928419
E_EL_fcP8_17F11_R_0	BY928420
E_EL_fcP8_17F12_F_0	BY920014
E_EL_fcP8_17F12_R_0	BY928421
E_EL_fcP8_17G01_F_0	BY920015
E_EL_fcP8_17G01_R_0	BY928422
E_EL_fcP8_17G02_F_0	BY920016
E_EL_fcP8_17G02_R_0	BY928423
E_EL_fcP8_17G03_R_0	BY928424
E_EL_fcP8_17G04_F_0	BY920017
E_EL_fcP8_17G04_R_0	BY928425
E_EL_fcP8_17G05_F_0	BY920018
E_EL_fcP8_17G05_R_0	BY928426
E_EL_fcP8_17G07_R_0	BY928427
E_EL_fcP8_17G08_F_0	BY920019
E_EL_fcP8_17G08_R_0	BY928428
E_EL_fcP8_17G09_F_0	BY920020
E_EL_fcP8_17G09_R_0	BY928429
E_EL_fcP8_17G10_F_0	BY920021
E_EL_fcP8_17G10_R_0	BY928430
E_EL_fcP8_17G11_F_0	BY920022
E_EL_fcP8_17G11_R_0	BY928431
E_EL_fcP8_17G12_F_0	BY920023
E_EL_fcP8_17G12_R_0	BY928432
E_EL_fcP8_17H01_F_0	BY920024
E_EL_fcP8_17H01_R_0	BY928433
E_EL_fcP8_17H03_F_0	BY920025
E_EL_fcP8_17H03_R_0	BY928434
E_EL_fcP8_17H04_R_0	BY928435
E_EL_fcP8_17H05_F_0	BY920026
E_EL_fcP8_17H05_R_0	BY928436
E_EL_fcP8_17H06_F_0	BY920027
E_EL_fcP8_17H06_R_0	BY928437
E_EL_fcP8_17H07_F_0	BY920028
E_EL_fcP8_17H07_R_0	BY928438
E_EL_fcP8_17H08_R_0	BY928439
E_EL_fcP8_17H09_F_0	BY920029
E_EL_fcP8_17H09_R_0	BY928440
E_EL_fcP8_17H10_F_0	BY920030
E_EL_fcP8_17H10_R_0	BY928441

E_EL_fcP8_17H11_F_0	BY920031
E_EL_fcP8_17H11_R_0	BY928442
E_EL_fcP8_18A01_F_0	BY920032
E_EL_fcP8_18A01_R_0	BY928443
E_EL_fcP8_18A02_F_0	BY920033
E_EL_fcP8_18A02_R_0	BY928444
E_EL_fcP8_18A03_F_0	BY920034
E_EL_fcP8_18A04_F_0	BY920035
E_EL_fcP8_18A04_R_0	BY928445
E_EL_fcP8_18A05_F_0	BY920036
E_EL_fcP8_18A05_R_0	BY928446
E_EL_fcP8_18A06_F_0	BY920037
E_EL_fcP8_18A06_R_0	BY928447
E_EL_fcP8_18A07_F_0	BY920038
E_EL_fcP8_18A07_R_0	BY928448
E_EL_fcP8_18A09_F_0	BY920039
E_EL_fcP8_18A09_R_0	BY928449
E_EL_fcP8_18A10_F_0	BY920040
E_EL_fcP8_18A10_R_0	BY928450
E_EL_fcP8_18A11_F_0	BY920041
E_EL_fcP8_18A11_R_0	BY928451
E_EL_fcP8_18A12_F_0	BY920042
E_EL_fcP8_18A12_R_0	BY928452
E_EL_fcP8_18B01_F_0	BY920043
E_EL_fcP8_18B01_R_0	BY928453
E_EL_fcP8_18B02_F_0	BY920044
E_EL_fcP8_18B02_R_0	BY928454
E_EL_fcP8_18B03_F_0	BY920045
E_EL_fcP8_18B03_R_0	BY928455
E_EL_fcP8_18B04_F_0	BY920046
E_EL_fcP8_18B04_R_0	BY928456
E_EL_fcP8_18B06_F_0	BY920047
E_EL_fcP8_18B06_R_0	BY928457
E_EL_fcP8_18B07_F_0	BY920048
E_EL_fcP8_18B07_R_0	BY928458
E_EL_fcP8_18B08_F_0	BY920049
E_EL_fcP8_18B08_R_0	BY928459
E_EL_fcP8_18B09_F_0	BY920050
E_EL_fcP8_18B09_R_0	BY928460
E_EL_fcP8_18B10_R_0	BY928461
E_EL_fcP8_18B11_R_0	BY928462
E_EL_fcP8_18B12_F_0	BY920051
E_EL_fcP8_18B12_R_0	BY928463
E_EL_fcP8_18C01_F_0	BY920052
E_EL_fcP8_18C01_R_0	BY928464
E_EL_fcP8_18C03_F_0	BY920053
E_EL_fcP8_18C03_R_0	BY928465
E_EL_fcP8_18C04_F_0	BY920054
E_EL_fcP8_18C04_R_0	BY928466
E_EL_fcP8_18C05_F_0	BY920055
E_EL_fcP8_18C05_R_0	BY928467
E_EL_fcP8_18C06_F_0	BY920056
E_EL_fcP8_18C06_R_0	BY928468
E_EL_fcP8_18C07_F_0	BY920057

E_EL_fcP8_18C07_R_0	BY928469
E_EL_fcP8_18C08_F_0	BY920058
E_EL_fcP8_18C08_R_0	BY928470
E_EL_fcP8_18C09_F_0	BY920059
E_EL_fcP8_18C09_R_0	BY928471
E_EL_fcP8_18C10_F_0	BY920060
E_EL_fcP8_18C10_R_0	BY928472
E_EL_fcP8_18C11_F_0	BY920061
E_EL_fcP8_18C11_R_0	BY928473
E_EL_fcP8_18C12_F_0	BY920062
E_EL_fcP8_18C12_R_0	BY928474
E_EL_fcP8_18D01_F_0	BY920063
E_EL_fcP8_18D01_R_0	BY928475
E_EL_fcP8_18D02_F_0	BY920064
E_EL_fcP8_18D02_R_0	BY928476
E_EL_fcP8_18D03_F_0	BY920065
E_EL_fcP8_18D03_R_0	BY928477
E_EL_fcP8_18D04_F_0	BY920066
E_EL_fcP8_18D04_R_0	BY928478
E_EL_fcP8_18D05_F_0	BY920067
E_EL_fcP8_18D05_R_0	BY928479
E_EL_fcP8_18D06_F_0	BY920068
E_EL_fcP8_18D06_R_0	BY928480
E_EL_fcP8_18D07_F_0	BY920069
E_EL_fcP8_18D07_R_0	BY928481
E_EL_fcP8_18D08_F_0	BY920070
E_EL_fcP8_18D08_R_0	BY928482
E_EL_fcP8_18D09_F_0	BY920071
E_EL_fcP8_18D10_F_0	BY920072
E_EL_fcP8_18D10_R_0	BY928483
E_EL_fcP8_18D11_F_0	BY920073
E_EL_fcP8_18D11_R_0	BY928484
E_EL_fcP8_18D12_F_0	BY920074
E_EL_fcP8_18D12_R_0	BY928485
E_EL_fcP8_18E03_F_0	BY920075
E_EL_fcP8_18E03_R_0	BY928486
E_EL_fcP8_18E04_F_0	BY920076
E_EL_fcP8_18E04_R_0	BY928487
E_EL_fcP8_18E06_F_0	BY920077
E_EL_fcP8_18E06_R_0	BY928488
E_EL_fcP8_18E07_F_0	BY920078
E_EL_fcP8_18E07_R_0	BY928489
E_EL_fcP8_18E08_F_0	BY920079
E_EL_fcP8_18E08_R_0	BY928490
E_EL_fcP8_18E09_F_0	BY920080
E_EL_fcP8_18E10_F_0	BY920081
E_EL_fcP8_18E10_R_0	BY928491
E_EL_fcP8_18E11_F_0	BY920082
E_EL_fcP8_18E11_R_0	BY928492
E_EL_fcP8_18E12_F_0	BY920083
E_EL_fcP8_18E12_R_0	BY928493
E_EL_fcP8_18F01_R_0	BY928494
E_EL_fcP8_18F02_F_0	BY920084
E_EL_fcP8_18F02_R_0	BY928495

E_EL_fcP8_18F04_F_0	BY920085
E_EL_fcP8_18F04_R_0	BY928496
E_EL_fcP8_18F05_F_0	BY920086
E_EL_fcP8_18F05_R_0	BY928497
E_EL_fcP8_18F06_R_0	BY928498
E_EL_fcP8_18F07_R_0	BY928499
E_EL_fcP8_18F08_F_0	BY920087
E_EL_fcP8_18F08_R_0	BY928500
E_EL_fcP8_18F09_F_0	BY920088
E_EL_fcP8_18F09_R_0	BY928501
E_EL_fcP8_18F10_F_0	BY920089
E_EL_fcP8_18F10_R_0	BY928502
E_EL_fcP8_18F12_F_0	BY920090
E_EL_fcP8_18F12_R_0	BY928503
E_EL_fcP8_18G01_F_0	BY920091
E_EL_fcP8_18G01_R_0	BY928504
E_EL_fcP8_18G02_F_0	BY920092
E_EL_fcP8_18G02_R_0	BY928505
E_EL_fcP8_18G03_R_0	BY928506
E_EL_fcP8_18G04_R_0	BY928507
E_EL_fcP8_18G05_F_0	BY920093
E_EL_fcP8_18G05_R_0	BY928508
E_EL_fcP8_18G06_F_0	BY920094
E_EL_fcP8_18G06_R_0	BY928509
E_EL_fcP8_18G07_R_0	BY928510
E_EL_fcP8_18G08_F_0	BY920095
E_EL_fcP8_18G08_R_0	BY928511
E_EL_fcP8_18G09_F_0	BY920096
E_EL_fcP8_18G09_R_0	BY928512
E_EL_fcP8_18G10_F_0	BY920097
E_EL_fcP8_18G10_R_0	BY928513
E_EL_fcP8_18G11_F_0	BY920098
E_EL_fcP8_18G11_R_0	BY928514
E_EL_fcP8_18G12_F_0	BY920099
E_EL_fcP8_18G12_R_0	BY928515
E_EL_fcP8_18H01_F_0	BY920100
E_EL_fcP8_18H01_R_0	BY928516
E_EL_fcP8_18H02_F_0	BY920101
E_EL_fcP8_18H02_R_0	BY928517
E_EL_fcP8_18H04_F_0	BY920102
E_EL_fcP8_18H05_F_0	BY920103
E_EL_fcP8_18H05_R_0	BY928518
E_EL_fcP8_18H06_F_0	BY920104
E_EL_fcP8_18H06_R_0	BY928519
E_EL_fcP8_18H07_F_0	BY920105
E_EL_fcP8_18H07_R_0	BY928520
E_EL_fcP8_18H08_F_0	BY920106
E_EL_fcP8_18H08_R_0	BY928521
E_EL_fcP8_18H09_F_0	BY920107
E_EL_fcP8_18H09_R_0	BY928522
E_EL_fcP8_18H10_F_0	BY920108
E_EL_fcP8_18H10_R_0	BY928523
E_EL_fcP8_18H11_F_0	BY920109
E_EL_fcP8_18H11_R_0	BY928524

E_EL_fcP8_18H12_F_0	BY920110
E_EL_fcP8_18H12_R_0	BY928525
E_EL_fcP8_19A01_F_0	BY920111
E_EL_fcP8_19A01_R_0	BY928526
E_EL_fcP8_19A02_R_0	BY928527
E_EL_fcP8_19A03_R_0	BY928528
E_EL_fcP8_19A04_F_0	BY920112
E_EL_fcP8_19A04_R_0	BY928529
E_EL_fcP8_19A05_F_0	BY920113
E_EL_fcP8_19A05_R_0	BY928530
E_EL_fcP8_19A06_F_0	BY920114
E_EL_fcP8_19A06_R_0	BY928531
E_EL_fcP8_19A09_F_0	BY920115
E_EL_fcP8_19A09_R_0	BY928532
E_EL_fcP8_19A10_F_0	BY920116
E_EL_fcP8_19A11_F_0	BY920117
E_EL_fcP8_19B01_F_0	BY920118
E_EL_fcP8_19B01_R_0	BY928533
E_EL_fcP8_19B02_F_0	BY920119
E_EL_fcP8_19B02_R_0	BY928534
E_EL_fcP8_19B03_F_0	BY920120
E_EL_fcP8_19B03_R_0	BY928535
E_EL_fcP8_19B04_R_0	BY928536
E_EL_fcP8_19B05_F_0	BY920121
E_EL_fcP8_19B05_R_0	BY928537
E_EL_fcP8_19B06_F_0	BY920122
E_EL_fcP8_19B06_R_0	BY928538
E_EL_fcP8_19B07_F_0	BY920123
E_EL_fcP8_19B07_R_0	BY928539
E_EL_fcP8_19B09_F_0	BY920124
E_EL_fcP8_19B11_R_0	BY928540
E_EL_fcP8_19B12_R_0	BY928541
E_EL_fcP8_19C02_F_0	BY920125
E_EL_fcP8_19C02_R_0	BY928542
E_EL_fcP8_19C04_F_0	BY920126
E_EL_fcP8_19C04_R_0	BY928543
E_EL_fcP8_19C05_F_0	BY920127
E_EL_fcP8_19C05_R_0	BY928544
E_EL_fcP8_19C06_R_0	BY928545
E_EL_fcP8_19C08_F_0	BY920128
E_EL_fcP8_19C08_R_0	BY928546
E_EL_fcP8_19C09_F_0	BY920129
E_EL_fcP8_19C10_F_0	BY920130
E_EL_fcP8_19C10_R_0	BY928547
E_EL_fcP8_19C11_F_0	BY920131
E_EL_fcP8_19C11_R_0	BY928548
E_EL_fcP8_19C12_R_0	BY928549
E_EL_fcP8_19D01_F_0	BY920132
E_EL_fcP8_19D02_F_0	BY920133
E_EL_fcP8_19D02_R_0	BY928550
E_EL_fcP8_19D03_F_0	BY920134
E_EL_fcP8_19D03_R_0	BY928551
E_EL_fcP8_19D04_F_0	BY920135
E_EL_fcP8_19D04_R_0	BY928552

E_EL_fcP8_19D05_F_0	BY920136
E_EL_fcP8_19D06_F_0	BY920137
E_EL_fcP8_19D06_R_0	BY928553
E_EL_fcP8_19D07_R_0	BY928554
E_EL_fcP8_19D08_F_0	BY920138
E_EL_fcP8_19D09_F_0	BY920139
E_EL_fcP8_19D09_R_0	BY928555
E_EL_fcP8_19D10_F_0	BY920140
E_EL_fcP8_19D10_R_0	BY928556
E_EL_fcP8_19D11_F_0	BY920141
E_EL_fcP8_19D11_R_0	BY928557
E_EL_fcP8_19D12_F_0	BY920142
E_EL_fcP8_19E01_F_0	BY920143
E_EL_fcP8_19E01_R_0	BY928558
E_EL_fcP8_19E02_F_0	BY920144
E_EL_fcP8_19E02_R_0	BY928559
E_EL_fcP8_19E03_R_0	BY928560
E_EL_fcP8_19E04_F_0	BY920145
E_EL_fcP8_19E04_R_0	BY928561
E_EL_fcP8_19E05_F_0	BY920146
E_EL_fcP8_19E05_R_0	BY928562
E_EL_fcP8_19E06_F_0	BY920147
E_EL_fcP8_19E06_R_0	BY928563
E_EL_fcP8_19E08_F_0	BY920148
E_EL_fcP8_19E08_R_0	BY928564
E_EL_fcP8_19E11_F_0	BY920149
E_EL_fcP8_19E11_R_0	BY928565
E_EL_fcP8_19E12_F_0	BY920150
E_EL_fcP8_19E12_R_0	BY928566
E_EL_fcP8_19F01_F_0	BY920151
E_EL_fcP8_19F01_R_0	BY928567
E_EL_fcP8_19F02_R_0	BY928568
E_EL_fcP8_19F04_F_0	BY920152
E_EL_fcP8_19F04_R_0	BY928569
E_EL_fcP8_19F06_F_0	BY920153
E_EL_fcP8_19F06_R_0	BY928570
E_EL_fcP8_19F07_F_0	BY920154
E_EL_fcP8_19F07_R_0	BY928571
E_EL_fcP8_19F08_F_0	BY920155
E_EL_fcP8_19F08_R_0	BY928572
E_EL_fcP8_19F09_F_0	BY920156
E_EL_fcP8_19F09_R_0	BY928573
E_EL_fcP8_19F10_F_0	BY920157
E_EL_fcP8_19F10_R_0	BY928574
E_EL_fcP8_19F12_F_0	BY920158
E_EL_fcP8_19G01_F_0	BY920159
E_EL_fcP8_19G01_R_0	BY928575
E_EL_fcP8_19G02_F_0	BY920160
E_EL_fcP8_19G02_R_0	BY928576
E_EL_fcP8_19G03_F_0	BY920161
E_EL_fcP8_19G03_R_0	BY928577
E_EL_fcP8_19G05_R_0	BY928578
E_EL_fcP8_19G06_F_0	BY920162
E_EL_fcP8_19G06_R_0	BY928579

E_EL_fcP8_19G07_F_0	BY920163
E_EL_fcP8_19G07_R_0	BY928580
E_EL_fcP8_19G08_F_0	BY920164
E_EL_fcP8_19G08_R_0	BY928581
E_EL_fcP8_19G09_F_0	BY920165
E_EL_fcP8_19G09_R_0	BY928582
E_EL_fcP8_19G11_F_0	BY920166
E_EL_fcP8_19G11_R_0	BY928583
E_EL_fcP8_19G12_F_0	BY920167
E_EL_fcP8_19H01_F_0	BY920168
E_EL_fcP8_19H02_R_0	BY928584
E_EL_fcP8_19H03_F_0	BY920169
E_EL_fcP8_19H03_R_0	BY928585
E_EL_fcP8_19H04_F_0	BY920170
E_EL_fcP8_19H04_R_0	BY928586
E_EL_fcP8_19H05_F_0	BY920171
E_EL_fcP8_19H05_R_0	BY928587
E_EL_fcP8_19H06_F_0	BY920172
E_EL_fcP8_19H06_R_0	BY928588
E_EL_fcP8_19H07_F_0	BY920173
E_EL_fcP8_19H07_R_0	BY928589
E_EL_fcP8_19H08_F_0	BY920174
E_EL_fcP8_19H08_R_0	BY928590
E_EL_fcP8_19H09_F_0	BY920175
E_EL_fcP8_19H09_R_0	BY928591
E_EL_fcP8_19H10_F_0	BY920176
E_EL_fcP8_19H10_R_0	BY928592
E_EL_fcP8_19H11_F_0	BY920177
E_EL_fcP8_19H11_R_0	BY928593
E_EL_fcP8_19H12_F_0	BY920178
E_EL_fcP8_19H12_R_0	BY928594
E_EL_fcP8_20A01_R_0	BY928595
E_EL_fcP8_20A02_F_0	BY920179
E_EL_fcP8_20A02_R_0	BY928596
E_EL_fcP8_20A03_F_0	BY920180
E_EL_fcP8_20A03_R_0	BY928597
E_EL_fcP8_20A04_R_0	BY928598
E_EL_fcP8_20A05_F_0	BY920181
E_EL_fcP8_20A05_R_0	BY928599
E_EL_fcP8_20A07_F_0	BY920182
E_EL_fcP8_20A07_R_0	BY928600
E_EL_fcP8_20A09_F_0	BY920183
E_EL_fcP8_20A09_R_0	BY928601
E_EL_fcP8_20A10_F_0	BY920184
E_EL_fcP8_20A10_R_0	BY928602
E_EL_fcP8_20A11_F_0	BY920185
E_EL_fcP8_20A11_R_0	BY928603
E_EL_fcP8_20A12_F_0	BY920186
E_EL_fcP8_20A12_R_0	BY928604
E_EL_fcP8_20B02_F_0	BY920187
E_EL_fcP8_20B02_R_0	BY928605
E_EL_fcP8_20B03_F_0	BY920188
E_EL_fcP8_20B03_R_0	BY928606
E_EL_fcP8_20B04_F_0	BY920189

E_EL_fcP8_20B04_R_0	BY928607
E_EL_fcP8_20B06_F_0	BY920190
E_EL_fcP8_20B06_R_0	BY928608
E_EL_fcP8_20B07_F_0	BY920191
E_EL_fcP8_20B07_R_0	BY928609
E_EL_fcP8_20B08_F_0	BY920192
E_EL_fcP8_20B08_R_0	BY928610
E_EL_fcP8_20B09_F_0	BY920193
E_EL_fcP8_20B09_R_0	BY928611
E_EL_fcP8_20B10_F_0	BY920194
E_EL_fcP8_20B10_R_0	BY928612
E_EL_fcP8_20B11_F_0	BY920195
E_EL_fcP8_20B11_R_0	BY928613
E_EL_fcP8_20B12_F_0	BY920196
E_EL_fcP8_20B12_R_0	BY928614
E_EL_fcP8_20C01_F_0	BY920197
E_EL_fcP8_20C01_R_0	BY928615
E_EL_fcP8_20C02_F_0	BY920198
E_EL_fcP8_20C02_R_0	BY928616
E_EL_fcP8_20C03_F_0	BY920199
E_EL_fcP8_20C03_R_0	BY928617
E_EL_fcP8_20C04_F_0	BY920200
E_EL_fcP8_20C04_R_0	BY928618
E_EL_fcP8_20C05_R_0	BY928619
E_EL_fcP8_20C06_F_0	BY920201
E_EL_fcP8_20C06_R_0	BY928620
E_EL_fcP8_20C07_F_0	BY920202
E_EL_fcP8_20C07_R_0	BY928621
E_EL_fcP8_20C09_R_0	BY928622
E_EL_fcP8_20C11_F_0	BY920203
E_EL_fcP8_20C11_R_0	BY928623
E_EL_fcP8_20C12_F_0	BY920204
E_EL_fcP8_20C12_R_0	BY928624
E_EL_fcP8_20D02_R_0	BY928625
E_EL_fcP8_20D03_F_0	BY920205
E_EL_fcP8_20D03_R_0	BY928626
E_EL_fcP8_20D04_F_0	BY920206
E_EL_fcP8_20D04_R_0	BY928627
E_EL_fcP8_20D05_F_0	BY920207
E_EL_fcP8_20D05_R_0	BY928628
E_EL_fcP8_20D06_F_0	BY920208
E_EL_fcP8_20D06_R_0	BY928629
E_EL_fcP8_20D07_F_0	BY920209
E_EL_fcP8_20D07_R_0	BY928630
E_EL_fcP8_20D08_F_0	BY920210
E_EL_fcP8_20D08_R_0	BY928631
E_EL_fcP8_20D09_F_0	BY920211
E_EL_fcP8_20D09_R_0	BY928632
E_EL_fcP8_20D10_F_0	BY920212
E_EL_fcP8_20D10_R_0	BY928633
E_EL_fcP8_20D11_F_0	BY920213
E_EL_fcP8_20D11_R_0	BY928634
E_EL_fcP8_20D12_F_0	BY920214
E_EL_fcP8_20D12_R_0	BY928635

E_EL_fcP8_20E01_R_0	BY928636
E_EL_fcP8_20E02_F_0	BY920215
E_EL_fcP8_20E02_R_0	BY928637
E_EL_fcP8_20E04_F_0	BY920216
E_EL_fcP8_20E04_R_0	BY928638
E_EL_fcP8_20E05_F_0	BY920217
E_EL_fcP8_20E06_F_0	BY920218
E_EL_fcP8_20E07_F_0	BY920219
E_EL_fcP8_20E07_R_0	BY928639
E_EL_fcP8_20E08_F_0	BY920220
E_EL_fcP8_20E08_R_0	BY928640
E_EL_fcP8_20E10_F_0	BY920221
E_EL_fcP8_20E10_R_0	BY928641
E_EL_fcP8_20E11_F_0	BY920222
E_EL_fcP8_20E11_R_0	BY928642
E_EL_fcP8_20E12_F_0	BY920223
E_EL_fcP8_20E12_R_0	BY928643
E_EL_fcP8_20F01_R_0	BY928644
E_EL_fcP8_20F02_F_0	BY920224
E_EL_fcP8_20F02_R_0	BY928645
E_EL_fcP8_20F03_F_0	BY920225
E_EL_fcP8_20F03_R_0	BY928646
E_EL_fcP8_20F04_F_0	BY920226
E_EL_fcP8_20F04_R_0	BY928647
E_EL_fcP8_20F05_F_0	BY920227
E_EL_fcP8_20F05_R_0	BY928648
E_EL_fcP8_20F06_F_0	BY920228
E_EL_fcP8_20F06_R_0	BY928649
E_EL_fcP8_20F07_F_0	BY920229
E_EL_fcP8_20F07_R_0	BY928650
E_EL_fcP8_20F08_F_0	BY920230
E_EL_fcP8_20F08_R_0	BY928651
E_EL_fcP8_20F10_F_0	BY920231
E_EL_fcP8_20F10_R_0	BY928652
E_EL_fcP8_20F11_F_0	BY920232
E_EL_fcP8_20F12_F_0	BY920233
E_EL_fcP8_20F12_R_0	BY928653
E_EL_fcP8_20G02_F_0	BY920234
E_EL_fcP8_20G02_R_0	BY928654
E_EL_fcP8_20G03_R_0	BY928655
E_EL_fcP8_20G04_F_0	BY920235
E_EL_fcP8_20G04_R_0	BY928656
E_EL_fcP8_20G05_F_0	BY920236
E_EL_fcP8_20G05_R_0	BY928657
E_EL_fcP8_20G06_F_0	BY920237
E_EL_fcP8_20G07_F_0	BY920238
E_EL_fcP8_20G07_R_0	BY928658
E_EL_fcP8_20G09_R_0	BY928659
E_EL_fcP8_20G10_R_0	BY928660
E_EL_fcP8_20G11_F_0	BY920239
E_EL_fcP8_20G11_R_0	BY928661
E_EL_fcP8_20G12_F_0	BY920240
E_EL_fcP8_20G12_R_0	BY928662
E_EL_fcP8_20H01_F_0	BY920241

E_EL_fcP8_20H01_R_0	BY928663
E_EL_fcP8_20H02_F_0	BY920242
E_EL_fcP8_20H02_R_0	BY928664
E_EL_fcP8_20H03_F_0	BY920243
E_EL_fcP8_20H03_R_0	BY928665
E_EL_fcP8_20H04_F_0	BY920244
E_EL_fcP8_20H04_R_0	BY928666
E_EL_fcP8_20H05_F_0	BY920245
E_EL_fcP8_20H05_R_0	BY928667
E_EL_fcP8_20H06_F_0	BY920246
E_EL_fcP8_20H06_R_0	BY928668
E_EL_fcP8_20H07_F_0	BY920247
E_EL_fcP8_20H07_R_0	BY928669
E_EL_fcP8_20H09_F_0	BY920248
E_EL_fcP8_20H09_R_0	BY928670
E_EL_fcP8_20H10_F_0	BY920249
E_EL_fcP8_20H10_R_0	BY928671
E_EL_fcP8_20H11_F_0	BY920250
E_EL_fcP8_20H11_R_0	BY928672
E_EL_fcP8_20H12_F_0	BY920251
E_EL_fcP8_20H12_R_0	BY928673
E_EL_fcP8_21A02_F_0	BY920252
E_EL_fcP8_21A02_R_0	BY928674
E_EL_fcP8_21A03_R_0	BY928675
E_EL_fcP8_21A04_F_0	BY920253
E_EL_fcP8_21A04_R_0	BY928676
E_EL_fcP8_21A06_R_0	BY928677
E_EL_fcP8_21A07_F_0	BY920254
E_EL_fcP8_21A09_R_0	BY928678
E_EL_fcP8_21A10_R_0	BY928679
E_EL_fcP8_21A11_F_0	BY920255
E_EL_fcP8_21A11_R_0	BY928680
E_EL_fcP8_21B01_F_0	BY920256
E_EL_fcP8_21B01_R_0	BY928681
E_EL_fcP8_21B02_F_0	BY920257
E_EL_fcP8_21B02_R_0	BY928682
E_EL_fcP8_21B03_F_0	BY920258
E_EL_fcP8_21B03_R_0	BY928683
E_EL_fcP8_21B04_F_0	BY920259
E_EL_fcP8_21B04_R_0	BY928684
E_EL_fcP8_21B05_F_0	BY920260
E_EL_fcP8_21B05_R_0	BY928685
E_EL_fcP8_21B06_F_0	BY920261
E_EL_fcP8_21B06_R_0	BY928686
E_EL_fcP8_21B07_F_0	BY920262
E_EL_fcP8_21B07_R_0	BY928687
E_EL_fcP8_21B08_R_0	BY928688
E_EL_fcP8_21B09_F_0	BY920263
E_EL_fcP8_21B09_R_0	BY928689
E_EL_fcP8_21B10_F_0	BY920264
E_EL_fcP8_21B10_R_0	BY928690
E_EL_fcP8_21B11_F_0	BY920265
E_EL_fcP8_21B12_R_0	BY928691
E_EL_fcP8_21C01_F_0	BY920266

E_EL_fcP8_21C01_R_0	BY928692
E_EL_fcP8_21C02_F_0	BY920267
E_EL_fcP8_21C02_R_0	BY928693
E_EL_fcP8_21C03_F_0	BY920268
E_EL_fcP8_21C03_R_0	BY928694
E_EL_fcP8_21C04_F_0	BY920269
E_EL_fcP8_21C04_R_0	BY928695
E_EL_fcP8_21C06_F_0	BY920270
E_EL_fcP8_21C06_R_0	BY928696
E_EL_fcP8_21C07_F_0	BY920271
E_EL_fcP8_21C08_F_0	BY920272
E_EL_fcP8_21C08_R_0	BY928697
E_EL_fcP8_21C10_F_0	BY920273
E_EL_fcP8_21C10_R_0	BY928698
E_EL_fcP8_21C11_F_0	BY920274
E_EL_fcP8_21C11_R_0	BY928699
E_EL_fcP8_21D01_F_0	BY920275
E_EL_fcP8_21D01_R_0	BY928700
E_EL_fcP8_21D02_F_0	BY920276
E_EL_fcP8_21D02_R_0	BY928701
E_EL_fcP8_21D03_F_0	BY920277
E_EL_fcP8_21D03_R_0	BY928702
E_EL_fcP8_21D04_F_0	BY920278
E_EL_fcP8_21D04_R_0	BY928703
E_EL_fcP8_21D05_F_0	BY920279
E_EL_fcP8_21D05_R_0	BY928704
E_EL_fcP8_21D06_F_0	BY920280
E_EL_fcP8_21D06_R_0	BY928705
E_EL_fcP8_21D07_F_0	BY920281
E_EL_fcP8_21D07_R_0	BY928706
E_EL_fcP8_21D08_F_0	BY920282
E_EL_fcP8_21D08_R_0	BY928707
E_EL_fcP8_21D09_R_0	BY928708
E_EL_fcP8_21D11_F_0	BY920283
E_EL_fcP8_21D11_R_0	BY928709
E_EL_fcP8_21E01_R_0	BY928710
E_EL_fcP8_21E02_F_0	BY920284
E_EL_fcP8_21E02_R_0	BY928711
E_EL_fcP8_21E03_F_0	BY920285
E_EL_fcP8_21E03_R_0	BY928712
E_EL_fcP8_21E04_F_0	BY920286
E_EL_fcP8_21E04_R_0	BY928713
E_EL_fcP8_21E06_R_0	BY928714
E_EL_fcP8_21E07_R_0	BY928715
E_EL_fcP8_21E09_F_0	BY920287
E_EL_fcP8_21E09_R_0	BY928716
E_EL_fcP8_21E10_F_0	BY920288
E_EL_fcP8_21E10_R_0	BY928717
E_EL_fcP8_21E11_F_0	BY920289
E_EL_fcP8_21E11_R_0	BY928718
E_EL_fcP8_21E12_F_0	BY920290
E_EL_fcP8_21E12_R_0	BY928719
E_EL_fcP8_21F02_R_0	BY928720
E_EL_fcP8_21F03_F_0	BY920291

E_EL_fcP8_21F03_R_0	BY928721
E_EL_fcP8_21F04_R_0	BY928722
E_EL_fcP8_21F06_F_0	BY920292
E_EL_fcP8_21F06_R_0	BY928723
E_EL_fcP8_21F07_F_0	BY920293
E_EL_fcP8_21F07_R_0	BY928724
E_EL_fcP8_21F08_F_0	BY920294
E_EL_fcP8_21F08_R_0	BY928725
E_EL_fcP8_21F09_F_0	BY920295
E_EL_fcP8_21F09_R_0	BY928726
E_EL_fcP8_21F10_F_0	BY920296
E_EL_fcP8_21F10_R_0	BY928727
E_EL_fcP8_21F11_F_0	BY920297
E_EL_fcP8_21F11_R_0	BY928728
E_EL_fcP8_21F12_R_0	BY928729
E_EL_fcP8_21G01_F_0	BY920298
E_EL_fcP8_21G01_R_0	BY928730
E_EL_fcP8_21G02_R_0	BY928731
E_EL_fcP8_21G03_F_0	BY920299
E_EL_fcP8_21G03_R_0	BY928732
E_EL_fcP8_21G04_F_0	BY920300
E_EL_fcP8_21G04_R_0	BY928733
E_EL_fcP8_21G05_R_0	BY928734
E_EL_fcP8_21G06_F_0	BY920301
E_EL_fcP8_21G06_R_0	BY928735
E_EL_fcP8_21G07_F_0	BY920302
E_EL_fcP8_21G07_R_0	BY928736
E_EL_fcP8_21G08_F_0	BY920303
E_EL_fcP8_21G08_R_0	BY928737
E_EL_fcP8_21G09_F_0	BY920304
E_EL_fcP8_21G09_R_0	BY928738
E_EL_fcP8_21G10_F_0	BY920305
E_EL_fcP8_21G11_F_0	BY920306
E_EL_fcP8_21G11_R_0	BY928739
E_EL_fcP8_21G12_F_0	BY920307
E_EL_fcP8_21H01_R_0	BY928740
E_EL_fcP8_21H02_R_0	BY928741
E_EL_fcP8_21H04_F_0	BY920308
E_EL_fcP8_21H04_R_0	BY928742
E_EL_fcP8_21H05_F_0	BY920309
E_EL_fcP8_21H05_R_0	BY928743
E_EL_fcP8_21H06_R_0	BY928744
E_EL_fcP8_21H07_F_0	BY920310
E_EL_fcP8_21H07_R_0	BY928745
E_EL_fcP8_21H08_F_0	BY920311
E_EL_fcP8_21H08_R_0	BY928746
E_EL_fcP8_21H09_F_0	BY920312
E_EL_fcP8_21H09_R_0	BY928747
E_EL_fcP8_21H10_F_0	BY920313
E_EL_fcP8_21H10_R_0	BY928748
E_EL_fcP8_22A01_F_0	BY920314
E_EL_fcP8_22A01_R_0	BY928749
E_EL_fcP8_22A02_F_0	BY920315
E_EL_fcP8_22A02_R_0	BY928750

E_EL_fcP8_22A03_F_0	BY920316
E_EL_fcP8_22A03_R_0	BY928751
E_EL_fcP8_22A04_F_0	BY920317
E_EL_fcP8_22A04_R_0	BY928752
E_EL_fcP8_22A05_F_0	BY920318
E_EL_fcP8_22A05_R_0	BY928753
E_EL_fcP8_22A06_F_0	BY920319
E_EL_fcP8_22A06_R_0	BY928754
E_EL_fcP8_22A07_R_0	BY928755
E_EL_fcP8_22A08_F_0	BY920320
E_EL_fcP8_22A08_R_0	BY928756
E_EL_fcP8_22A09_F_0	BY920321
E_EL_fcP8_22A10_F_0	BY920322
E_EL_fcP8_22A10_R_0	BY928757
E_EL_fcP8_22A11_R_0	BY928758
E_EL_fcP8_22B01_F_0	BY920323
E_EL_fcP8_22B01_R_0	BY928759
E_EL_fcP8_22B02_R_0	BY928760
E_EL_fcP8_22B04_F_0	BY920324
E_EL_fcP8_22B04_R_0	BY928761
E_EL_fcP8_22B05_F_0	BY920325
E_EL_fcP8_22B06_F_0	BY920326
E_EL_fcP8_22B07_R_0	BY928762
E_EL_fcP8_22B09_R_0	BY928763
E_EL_fcP8_22B10_F_0	BY920327
E_EL_fcP8_22B10_R_0	BY928764
E_EL_fcP8_22B11_F_0	BY920328
E_EL_fcP8_22B11_R_0	BY928765
E_EL_fcP8_22B12_F_0	BY920329
E_EL_fcP8_22B12_R_0	BY928766
E_EL_fcP8_22C01_F_0	BY920330
E_EL_fcP8_22C01_R_0	BY928767
E_EL_fcP8_22C02_F_0	BY920331
E_EL_fcP8_22C02_R_0	BY928768
E_EL_fcP8_22C05_F_0	BY920332
E_EL_fcP8_22C05_R_0	BY928769
E_EL_fcP8_22C06_F_0	BY920333
E_EL_fcP8_22C06_R_0	BY928770
E_EL_fcP8_22C07_F_0	BY920334
E_EL_fcP8_22C07_R_0	BY928771
E_EL_fcP8_22C08_F_0	BY920335
E_EL_fcP8_22C08_R_0	BY928772
E_EL_fcP8_22C10_F_0	BY920336
E_EL_fcP8_22C10_R_0	BY928773
E_EL_fcP8_22C12_F_0	BY920337
E_EL_fcP8_22C12_R_0	BY928774
E_EL_fcP8_22D02_F_0	BY920338
E_EL_fcP8_22D03_F_0	BY920339
E_EL_fcP8_22D03_R_0	BY928775
E_EL_fcP8_22D04_F_0	BY920340
E_EL_fcP8_22D04_R_0	BY928776
E_EL_fcP8_22D05_F_0	BY920341
E_EL_fcP8_22D05_R_0	BY928777
E_EL_fcP8_22D06_R_0	BY928778

E_EL_fcP8_22D07_F_0	BY920342
E_EL_fcP8_22D07_R_0	BY928779
E_EL_fcP8_22D08_R_0	BY928780
E_EL_fcP8_22D09_F_0	BY920343
E_EL_fcP8_22D09_R_0	BY928781
E_EL_fcP8_22D10_F_0	BY920344
E_EL_fcP8_22D10_R_0	BY928782
E_EL_fcP8_22D11_F_0	BY920345
E_EL_fcP8_22D11_R_0	BY928783
E_EL_fcP8_22D12_F_0	BY920346
E_EL_fcP8_22E01_F_0	BY920347
E_EL_fcP8_22E01_R_0	BY928784
E_EL_fcP8_22E02_F_0	BY920348
E_EL_fcP8_22E02_R_0	BY928785
E_EL_fcP8_22E03_F_0	BY920349
E_EL_fcP8_22E03_R_0	BY928786
E_EL_fcP8_22E04_F_0	BY920350
E_EL_fcP8_22E04_R_0	BY928787
E_EL_fcP8_22E05_F_0	BY920351
E_EL_fcP8_22E05_R_0	BY928788
E_EL_fcP8_22E06_F_0	BY920352
E_EL_fcP8_22E07_R_0	BY928789
E_EL_fcP8_22E08_F_0	BY920353
E_EL_fcP8_22E08_R_0	BY928790
E_EL_fcP8_22E09_R_0	BY928791
E_EL_fcP8_22E10_F_0	BY920354
E_EL_fcP8_22E10_R_0	BY928792
E_EL_fcP8_22E11_F_0	BY920355
E_EL_fcP8_22E11_R_0	BY928793
E_EL_fcP8_22E12_F_0	BY920356
E_EL_fcP8_22E12_R_0	BY928794
E_EL_fcP8_22F01_F_0	BY920357
E_EL_fcP8_22F01_R_0	BY928795
E_EL_fcP8_22F02_F_0	BY920358
E_EL_fcP8_22F02_R_0	BY928796
E_EL_fcP8_22F03_F_0	BY920359
E_EL_fcP8_22F05_F_0	BY920360
E_EL_fcP8_22F05_R_0	BY928797
E_EL_fcP8_22F06_F_0	BY920361
E_EL_fcP8_22F06_R_0	BY928798
E_EL_fcP8_22F07_F_0	BY920362
E_EL_fcP8_22F07_R_0	BY928799
E_EL_fcP8_22F08_F_0	BY920363
E_EL_fcP8_22F08_R_0	BY928800
E_EL_fcP8_22F09_F_0	BY920364
E_EL_fcP8_22F09_R_0	BY928801
E_EL_fcP8_22F10_F_0	BY920365
E_EL_fcP8_22F10_R_0	BY928802
E_EL_fcP8_22F11_F_0	BY920366
E_EL_fcP8_22F11_R_0	BY928803
E_EL_fcP8_22F12_F_0	BY920367
E_EL_fcP8_22G01_F_0	BY920368
E_EL_fcP8_22G01_R_0	BY928804
E_EL_fcP8_22G02_F_0	BY920369

	E_EL_fcP8_22G02_R_0	BY928805
	E_EL_fcP8_22G03_F_0	BY920370
	E_EL_fcP8_22G03_R_0	BY928806
	E_EL_fcP8_22G05_F_0	BY920371
	E_EL_fcP8_22G05_R_0	BY928807
	E_EL_fcP8_22G06_F_0	BY920372
	E_EL_fcP8_22G06_R_0	BY928808
	E_EL_fcP8_22G07_F_0	BY920373
	E_EL_fcP8_22G07_R_0	BY928809
	E_EL_fcP8_22G08_F_0	BY920374
	E_EL_fcP8_22G08_R_0	BY928810
	E_EL_fcP8_22G09_F_0	BY920375
	E_EL_fcP8_22G09_R_0	BY928811
	E_EL_fcP8_22G10_F_0	BY920376
	E_EL_fcP8_22G10_R_0	BY928812
	E_EL_fcP8_22G11_F_0	BY920377
	E_EL_fcP8_22G11_R_0	BY928813
	E_EL_fcP8_22G12_F_0	BY920378
	E_EL_fcP8_22H01_F_0	BY920379
	E_EL_fcP8_22H01_R_0	BY928814
	E_EL_fcP8_22H02_R_0	BY928815
	E_EL_fcP8_22H03_F_0	BY920380
	E_EL_fcP8_22H03_R_0	BY928816
	E_EL_fcP8_22H04_F_0	BY920381
	E_EL_fcP8_22H04_R_0	BY928817
	E_EL_fcP8_22H05_F_0	BY920382
	E_EL_fcP8_22H05_R_0	BY928818
	E_EL_fcP8_22H06_R_0	BY928819
	E_EL_fcP8_22H07_F_0	BY920383
	E_EL_fcP8_22H07_R_0	BY928820
	E_EL_fcP8_22H08_F_0	BY920384
	E_EL_fcP8_22H08_R_0	BY928821
	E_EL_fcP8_22H09_F_0	BY920385
	E_EL_fcP8_22H09_R_0	BY928822
	E_EL_fcP8_22H10_F_0	BY920386
	E_EL_fcP8_22H10_R_0	BY928823
	E_EL_fcP8_22H11_F_0	BY920387
	E_EL_fcP8_22H11_R_0	BY928824
	E_EL_fcP8_22H12_F_0	BY920388
	E_EL_fcP8_22H12_R_0	BY928825
ovS0	E_EL_ovS0_01A03_F_0	BY921255
	E_EL_ovS0_01A03_R_0	BY929788
	E_EL_ovS0_01A04_F_0	BY921256
	E_EL_ovS0_01A04_R_0	BY929789
	E_EL_ovS0_01A05_F_0	BY921257
	E_EL_ovS0_01A06_F_0	BY921258
	E_EL_ovS0_01A07_F_0	BY921259
	E_EL_ovS0_01A07_R_0	BY929790
	E_EL_ovS0_01A08_F_0	BY921260
	E_EL_ovS0_01A08_R_0	BY929791
	E_EL_ovS0_01A09_F_0	BY921261
	E_EL_ovS0_01A09_R_0	BY929792
	E_EL_ovS0_01A10_F_0	BY921262
	E_EL_ovS0_01A11_F_0	BY921263

E_EL_ovS0_01A11_R_0	BY929793
E_EL_ovS0_01A12_F_0	BY921264
E_EL_ovS0_01A12_R_0	BY929794
E_EL_ovS0_01B01_F_0	BY921265
E_EL_ovS0_01B01_R_0	BY929795
E_EL_ovS0_01B02_F_0	BY921266
E_EL_ovS0_01B02_R_0	BY929796
E_EL_ovS0_01B03_F_0	BY921267
E_EL_ovS0_01B03_R_0	BY929797
E_EL_ovS0_01B04_F_0	BY921268
E_EL_ovS0_01B04_R_0	BY929798
E_EL_ovS0_01B05_F_0	BY921269
E_EL_ovS0_01B05_R_0	BY929799
E_EL_ovS0_01B06_F_0	BY921270
E_EL_ovS0_01B06_R_0	BY929800
E_EL_ovS0_01B07_F_0	BY921271
E_EL_ovS0_01B08_F_0	BY921272
E_EL_ovS0_01B08_R_0	BY929801
E_EL_ovS0_01B09_F_0	BY921273
E_EL_ovS0_01B09_R_0	BY929802
E_EL_ovS0_01B10_F_0	BY921274
E_EL_ovS0_01B10_R_0	BY929803
E_EL_ovS0_01B11_R_0	BY929804
E_EL_ovS0_01C01_F_0	BY921275
E_EL_ovS0_01C01_R_0	BY929805
E_EL_ovS0_01C02_F_0	BY921276
E_EL_ovS0_01C02_R_0	BY929806
E_EL_ovS0_01C04_F_0	BY921277
E_EL_ovS0_01C04_R_0	BY929807
E_EL_ovS0_01C05_F_0	BY921278
E_EL_ovS0_01C06_F_0	BY921279
E_EL_ovS0_01C06_R_0	BY929808
E_EL_ovS0_01C07_F_0	BY921280
E_EL_ovS0_01C07_R_0	BY929809
E_EL_ovS0_01C09_F_0	BY921281
E_EL_ovS0_01C10_F_0	BY921282
E_EL_ovS0_01C10_R_0	BY929810
E_EL_ovS0_01C11_F_0	BY921283
E_EL_ovS0_01C11_R_0	BY929811
E_EL_ovS0_01C12_F_0	BY921284
E_EL_ovS0_01C12_R_0	BY929812
E_EL_ovS0_01D01_F_0	BY921285
E_EL_ovS0_01D01_R_0	BY929813
E_EL_ovS0_01D02_F_0	BY921286
E_EL_ovS0_01D02_R_0	BY929814
E_EL_ovS0_01D03_F_0	BY921287
E_EL_ovS0_01D04_F_0	BY921288
E_EL_ovS0_01D04_R_0	BY929815
E_EL_ovS0_01D05_F_0	BY921289
E_EL_ovS0_01D05_R_0	BY929816
E_EL_ovS0_01D06_R_0	BY929817
E_EL_ovS0_01D07_F_0	BY921290
E_EL_ovS0_01D07_R_0	BY929818
E_EL_ovS0_01D08_F_0	BY921291

E_EL_ovS0_01D09_F_0	BY921292
E_EL_ovS0_01D09_R_0	BY929819
E_EL_ovS0_01D10_F_0	BY921293
E_EL_ovS0_01D11_F_0	BY921294
E_EL_ovS0_01D11_R_0	BY929820
E_EL_ovS0_01D12_F_0	BY921295
E_EL_ovS0_01D12_R_0	BY929821
E_EL_ovS0_01E01_F_0	BY921296
E_EL_ovS0_01E01_R_0	BY929822
E_EL_ovS0_01E02_F_0	BY921297
E_EL_ovS0_01E02_R_0	BY929823
E_EL_ovS0_01E03_R_0	BY929824
E_EL_ovS0_01E04_F_0	BY921298
E_EL_ovS0_01E05_F_0	BY921299
E_EL_ovS0_01E05_R_0	BY929825
E_EL_ovS0_01E06_F_0	BY921300
E_EL_ovS0_01E06_R_0	BY929826
E_EL_ovS0_01E07_F_0	BY921301
E_EL_ovS0_01E08_F_0	BY921302
E_EL_ovS0_01E08_R_0	BY929827
E_EL_ovS0_01E09_F_0	BY921303
E_EL_ovS0_01E10_F_0	BY921304
E_EL_ovS0_01E10_R_0	BY929828
E_EL_ovS0_01E11_F_0	BY921305
E_EL_ovS0_01E11_R_0	BY929829
E_EL_ovS0_01E12_F_0	BY921306
E_EL_ovS0_01E12_R_0	BY929830
E_EL_ovS0_01F01_F_0	BY921307
E_EL_ovS0_01F01_R_0	BY929831
E_EL_ovS0_01F02_R_0	BY929832
E_EL_ovS0_01F03_F_0	BY921308
E_EL_ovS0_01F03_R_0	BY929833
E_EL_ovS0_01F04_F_0	BY921309
E_EL_ovS0_01F04_R_0	BY929834
E_EL_ovS0_01F05_F_0	BY921310
E_EL_ovS0_01F05_R_0	BY929835
E_EL_ovS0_01F06_F_0	BY921311
E_EL_ovS0_01F06_R_0	BY929836
E_EL_ovS0_01F07_F_0	BY921312
E_EL_ovS0_01F07_R_0	BY929837
E_EL_ovS0_01F08_R_0	BY929838
E_EL_ovS0_01F09_F_0	BY921313
E_EL_ovS0_01F09_R_0	BY929839
E_EL_ovS0_01F10_R_0	BY929840
E_EL_ovS0_01F11_R_0	BY929841
E_EL_ovS0_01F12_R_0	BY929842
E_EL_ovS0_01G01_F_0	BY921314
E_EL_ovS0_01G02_F_0	BY921315
E_EL_ovS0_01G02_R_0	BY929843
E_EL_ovS0_01G03_F_0	BY921316
E_EL_ovS0_01G03_R_0	BY929844
E_EL_ovS0_01G04_F_0	BY921317
E_EL_ovS0_01G04_R_0	BY929845
E_EL_ovS0_01G05_F_0	BY921318

E_EL_ovS0_01G05_R_0	BY929846
E_EL_ovS0_01G06_F_0	BY921319
E_EL_ovS0_01G06_R_0	BY929847
E_EL_ovS0_01G07_F_0	BY921320
E_EL_ovS0_01G07_R_0	BY929848
E_EL_ovS0_01G08_F_0	BY921321
E_EL_ovS0_01G08_R_0	BY929849
E_EL_ovS0_01G09_F_0	BY921322
E_EL_ovS0_01G09_R_0	BY929850
E_EL_ovS0_01G10_F_0	BY921323
E_EL_ovS0_01G10_R_0	BY929851
E_EL_ovS0_01G11_F_0	BY921324
E_EL_ovS0_01G11_R_0	BY929852
E_EL_ovS0_01H01_F_0	BY921325
E_EL_ovS0_01H01_R_0	BY929853
E_EL_ovS0_01H02_F_0	BY921326
E_EL_ovS0_01H02_R_0	BY929854
E_EL_ovS0_01H03_F_0	BY921327
E_EL_ovS0_01H03_R_0	BY929855
E_EL_ovS0_01H04_R_0	BY929856
E_EL_ovS0_01H05_F_0	BY921328
E_EL_ovS0_01H05_R_0	BY929857
E_EL_ovS0_01H06_R_0	BY929858
E_EL_ovS0_01H07_F_0	BY921329
E_EL_ovS0_01H07_R_0	BY929859
E_EL_ovS0_01H08_F_0	BY921330
E_EL_ovS0_01H10_F_0	BY921331
E_EL_ovS0_01H10_R_0	BY929860
E_EL_ovS0_01H12_F_0	BY921332
E_EL_ovS0_02A02_F_0	BY921333
E_EL_ovS0_02A02_R_0	BY929861
E_EL_ovS0_02A03_F_0	BY921334
E_EL_ovS0_02A03_R_0	BY929862
E_EL_ovS0_02A04_F_0	BY921335
E_EL_ovS0_02A04_R_0	BY929863
E_EL_ovS0_02A05_F_0	BY921336
E_EL_ovS0_02A07_R_0	BY929864
E_EL_ovS0_02A08_F_0	BY921337
E_EL_ovS0_02A08_R_0	BY929865
E_EL_ovS0_02A11_F_0	BY921338
E_EL_ovS0_02A11_R_0	BY929866
E_EL_ovS0_02A12_R_0	BY929867
E_EL_ovS0_02B01_F_0	BY921339
E_EL_ovS0_02B01_R_0	BY929868
E_EL_ovS0_02B02_F_0	BY921340
E_EL_ovS0_02B02_R_0	BY929869
E_EL_ovS0_02B03_F_0	BY921341
E_EL_ovS0_02B03_R_0	BY929870
E_EL_ovS0_02B04_F_0	BY921342
E_EL_ovS0_02B04_R_0	BY929871
E_EL_ovS0_02B05_F_0	BY921343
E_EL_ovS0_02B05_R_0	BY929872
E_EL_ovS0_02B06_F_0	BY921344
E_EL_ovS0_02B06_R_0	BY929873

E_EL_ovS0_02B07_F_0	BY921345
E_EL_ovS0_02B07_R_0	BY929874
E_EL_ovS0_02B08_F_0	BY921346
E_EL_ovS0_02B08_R_0	BY929875
E_EL_ovS0_02C01_F_0	BY921347
E_EL_ovS0_02C02_F_0	BY921348
E_EL_ovS0_02C02_R_0	BY929876
E_EL_ovS0_02C03_F_0	BY921349
E_EL_ovS0_02C03_R_0	BY929877
E_EL_ovS0_02C04_F_0	BY921350
E_EL_ovS0_02C04_R_0	BY929878
E_EL_ovS0_02C05_R_0	BY929879
E_EL_ovS0_02C07_F_0	BY921351
E_EL_ovS0_02C07_R_0	BY929880
E_EL_ovS0_02C08_F_0	BY921352
E_EL_ovS0_02C08_R_0	BY929881
E_EL_ovS0_02C09_F_0	BY921353
E_EL_ovS0_02C10_R_0	BY929882
E_EL_ovS0_02C11_F_0	BY921354
E_EL_ovS0_02C11_R_0	BY929883
E_EL_ovS0_02C12_F_0	BY921355
E_EL_ovS0_02C12_R_0	BY929884
E_EL_ovS0_02D01_F_0	BY921356
E_EL_ovS0_02D01_R_0	BY929885
E_EL_ovS0_02D02_F_0	BY921357
E_EL_ovS0_02D03_F_0	BY921358
E_EL_ovS0_02D03_R_0	BY929886
E_EL_ovS0_02D04_R_0	BY929887
E_EL_ovS0_02D05_R_0	BY929888
E_EL_ovS0_02D06_F_0	BY921359
E_EL_ovS0_02D06_R_0	BY929889
E_EL_ovS0_02D07_F_0	BY921360
E_EL_ovS0_02D07_R_0	BY929890
E_EL_ovS0_02D08_F_0	BY921361
E_EL_ovS0_02D08_R_0	BY929891
E_EL_ovS0_02D09_F_0	BY921362
E_EL_ovS0_02D09_R_0	BY929892
E_EL_ovS0_02D10_F_0	BY921363
E_EL_ovS0_02D11_F_0	BY921364
E_EL_ovS0_02D11_R_0	BY929893
E_EL_ovS0_02E01_F_0	BY921365
E_EL_ovS0_02E01_R_0	BY929894
E_EL_ovS0_02E02_F_0	BY921366
E_EL_ovS0_02E03_R_0	BY929895
E_EL_ovS0_02E04_F_0	BY921367
E_EL_ovS0_02E04_R_0	BY929896
E_EL_ovS0_02E06_F_0	BY921368
E_EL_ovS0_02E06_R_0	BY929897
E_EL_ovS0_02E07_F_0	BY921369
E_EL_ovS0_02E07_R_0	BY929898
E_EL_ovS0_02E08_F_0	BY921370
E_EL_ovS0_02E08_R_0	BY929899
E_EL_ovS0_02E09_F_0	BY921371
E_EL_ovS0_02E10_F_0	BY921372

E_EL_ovS0_02E10_R_0	BY929900
E_EL_ovS0_02E11_F_0	BY921373
E_EL_ovS0_02E12_R_0	BY929901
E_EL_ovS0_02F01_F_0	BY921374
E_EL_ovS0_02F02_F_0	BY921375
E_EL_ovS0_02F02_R_0	BY929902
E_EL_ovS0_02F03_F_0	BY921376
E_EL_ovS0_02F03_R_0	BY929903
E_EL_ovS0_02F04_F_0	BY921377
E_EL_ovS0_02F04_R_0	BY929904
E_EL_ovS0_02F05_F_0	BY921378
E_EL_ovS0_02F05_R_0	BY929905
E_EL_ovS0_02F06_F_0	BY921379
E_EL_ovS0_02F06_R_0	BY929906
E_EL_ovS0_02F07_F_0	BY921380
E_EL_ovS0_02F07_R_0	BY929907
E_EL_ovS0_02F08_F_0	BY921381
E_EL_ovS0_02F08_R_0	BY929908
E_EL_ovS0_02F09_F_0	BY921382
E_EL_ovS0_02F09_R_0	BY929909
E_EL_ovS0_02F10_F_0	BY921383
E_EL_ovS0_02F10_R_0	BY929910
E_EL_ovS0_02F11_F_0	BY921384
E_EL_ovS0_02F11_R_0	BY929911
E_EL_ovS0_02F12_F_0	BY921385
E_EL_ovS0_02F12_R_0	BY929912
E_EL_ovS0_02G01_F_0	BY921386
E_EL_ovS0_02G01_R_0	BY929913
E_EL_ovS0_02G02_F_0	BY921387
E_EL_ovS0_02G02_R_0	BY929914
E_EL_ovS0_02G03_F_0	BY921388
E_EL_ovS0_02G03_R_0	BY929915
E_EL_ovS0_02G04_F_0	BY921389
E_EL_ovS0_02G04_R_0	BY929916
E_EL_ovS0_02G05_F_0	BY921390
E_EL_ovS0_02G05_R_0	BY929917
E_EL_ovS0_02G06_F_0	BY921391
E_EL_ovS0_02G06_R_0	BY929918
E_EL_ovS0_02G08_R_0	BY929919
E_EL_ovS0_02G09_R_0	BY929920
E_EL_ovS0_02G10_F_0	BY921392
E_EL_ovS0_02G10_R_0	BY929921
E_EL_ovS0_02G11_F_0	BY921393
E_EL_ovS0_02G11_R_0	BY929922
E_EL_ovS0_02G12_F_0	BY921394
E_EL_ovS0_02G12_R_0	BY929923
E_EL_ovS0_02H01_F_0	BY921395
E_EL_ovS0_02H01_R_0	BY929924
E_EL_ovS0_02H02_F_0	BY921396
E_EL_ovS0_02H03_F_0	BY921397
E_EL_ovS0_02H03_R_0	BY929925
E_EL_ovS0_02H04_F_0	BY921398
E_EL_ovS0_02H05_F_0	BY921399
E_EL_ovS0_02H06_F_0	BY921400

E_EL_ovS0_02H07_F_0	BY921401
E_EL_ovS0_02H10_F_0	BY921402
E_EL_ovS0_02H11_F_0	BY921403
E_EL_ovS0_02H11_R_0	BY929926
E_EL_ovS0_02H12_F_0	BY921404
E_EL_ovS0_02H12_R_0	BY929927
E_EL_ovS0_03A01_F_0	BY921405
E_EL_ovS0_03A01_R_0	BY929928
E_EL_ovS0_03A02_F_0	BY921406
E_EL_ovS0_03A02_R_0	BY929929
E_EL_ovS0_03A03_F_0	BY921407
E_EL_ovS0_03A06_R_0	BY929930
E_EL_ovS0_03A07_F_0	BY921408
E_EL_ovS0_03A07_R_0	BY929931
E_EL_ovS0_03A08_F_0	BY921409
E_EL_ovS0_03A08_R_0	BY929932
E_EL_ovS0_03A09_F_0	BY921410
E_EL_ovS0_03A09_R_0	BY929933
E_EL_ovS0_03A11_F_0	BY921411
E_EL_ovS0_03A11_R_0	BY929934
E_EL_ovS0_03A12_F_0	BY921412
E_EL_ovS0_03A12_R_0	BY929935
E_EL_ovS0_03B01_F_0	BY921413
E_EL_ovS0_03B02_F_0	BY921414
E_EL_ovS0_03B02_R_0	BY929936
E_EL_ovS0_03B03_F_0	BY921415
E_EL_ovS0_03B03_R_0	BY929937
E_EL_ovS0_03B04_F_0	BY921416
E_EL_ovS0_03B05_F_0	BY921417
E_EL_ovS0_03B06_F_0	BY921418
E_EL_ovS0_03B07_F_0	BY921419
E_EL_ovS0_03B07_R_0	BY929938
E_EL_ovS0_03B08_F_0	BY921420
E_EL_ovS0_03B08_R_0	BY929939
E_EL_ovS0_03B09_F_0	BY921421
E_EL_ovS0_03B09_R_0	BY929940
E_EL_ovS0_03B11_R_0	BY929941
E_EL_ovS0_03B12_F_0	BY921422
E_EL_ovS0_03B12_R_0	BY929942
E_EL_ovS0_03C01_R_0	BY929943
E_EL_ovS0_03C02_F_0	BY921423
E_EL_ovS0_03C02_R_0	BY929944
E_EL_ovS0_03C03_F_0	BY921424
E_EL_ovS0_03C03_R_0	BY929945
E_EL_ovS0_03C05_F_0	BY921425
E_EL_ovS0_03C05_R_0	BY929946
E_EL_ovS0_03C06_F_0	BY921426
E_EL_ovS0_03C06_R_0	BY929947
E_EL_ovS0_03C08_F_0	BY921427
E_EL_ovS0_03C08_R_0	BY929948
E_EL_ovS0_03C09_F_0	BY921428
E_EL_ovS0_03C09_R_0	BY929949
E_EL_ovS0_03C10_F_0	BY921429
E_EL_ovS0_03C10_R_0	BY929950

E_EL_ovS0_03C11_F_0	BY921430
E_EL_ovS0_03C12_R_0	BY929951
E_EL_ovS0_03D02_F_0	BY921431
E_EL_ovS0_03D02_R_0	BY929952
E_EL_ovS0_03D03_F_0	BY921432
E_EL_ovS0_03D03_R_0	BY929953
E_EL_ovS0_03D04_F_0	BY921433
E_EL_ovS0_03D04_R_0	BY929954
E_EL_ovS0_03D05_F_0	BY921434
E_EL_ovS0_03D05_R_0	BY929955
E_EL_ovS0_03D06_F_0	BY921435
E_EL_ovS0_03D06_R_0	BY929956
E_EL_ovS0_03D07_F_0	BY921436
E_EL_ovS0_03D07_R_0	BY929957
E_EL_ovS0_03D08_F_0	BY921437
E_EL_ovS0_03D08_R_0	BY929958
E_EL_ovS0_03D09_F_0	BY921438
E_EL_ovS0_03D09_R_0	BY929959
E_EL_ovS0_03D10_F_0	BY921439
E_EL_ovS0_03D10_R_0	BY929960
E_EL_ovS0_03D11_F_0	BY921440
E_EL_ovS0_03D11_R_0	BY929961
E_EL_ovS0_03D12_F_0	BY921441
E_EL_ovS0_03D12_R_0	BY929962
E_EL_ovS0_03E01_F_0	BY921442
E_EL_ovS0_03E01_R_0	BY929963
E_EL_ovS0_03E03_F_0	BY921443
E_EL_ovS0_03E04_F_0	BY921444
E_EL_ovS0_03E04_R_0	BY929964
E_EL_ovS0_03E06_F_0	BY921445
E_EL_ovS0_03E06_R_0	BY929965
E_EL_ovS0_03E07_F_0	BY921446
E_EL_ovS0_03E08_F_0	BY921447
E_EL_ovS0_03E08_R_0	BY929966
E_EL_ovS0_03E12_F_0	BY921448
E_EL_ovS0_03E12_R_0	BY929967
E_EL_ovS0_03F01_F_0	BY921449
E_EL_ovS0_03F02_F_0	BY921450
E_EL_ovS0_03F02_R_0	BY929968
E_EL_ovS0_03F03_F_0	BY921451
E_EL_ovS0_03F03_R_0	BY929969
E_EL_ovS0_03F04_F_0	BY921452
E_EL_ovS0_03F04_R_0	BY929970
E_EL_ovS0_03F05_F_0	BY921453
E_EL_ovS0_03F05_R_0	BY929971
E_EL_ovS0_03F07_F_0	BY921454
E_EL_ovS0_03F07_R_0	BY929972
E_EL_ovS0_03F08_F_0	BY921455
E_EL_ovS0_03F09_F_0	BY921456
E_EL_ovS0_03F09_R_0	BY929973
E_EL_ovS0_03F10_F_0	BY921457
E_EL_ovS0_03F10_R_0	BY929974
E_EL_ovS0_03F11_F_0	BY921458
E_EL_ovS0_03F11_R_0	BY929975

E_EL_ovS0_03F12_F_0	BY921459
E_EL_ovS0_03G01_F_0	BY921460
E_EL_ovS0_03G01_R_0	BY929976
E_EL_ovS0_03G02_F_0	BY921461
E_EL_ovS0_03G02_R_0	BY929977
E_EL_ovS0_03G04_F_0	BY921462
E_EL_ovS0_03G05_F_0	BY921463
E_EL_ovS0_03G05_R_0	BY929978
E_EL_ovS0_03G06_F_0	BY921464
E_EL_ovS0_03G06_R_0	BY929979
E_EL_ovS0_03G07_F_0	BY921465
E_EL_ovS0_03G08_F_0	BY921466
E_EL_ovS0_03G08_R_0	BY929980
E_EL_ovS0_03G11_F_0	BY921467
E_EL_ovS0_03G11_R_0	BY929981
E_EL_ovS0_03G12_F_0	BY921468
E_EL_ovS0_03G12_R_0	BY929982
E_EL_ovS0_03H01_F_0	BY921469
E_EL_ovS0_03H01_R_0	BY929983
E_EL_ovS0_03H02_F_0	BY921470
E_EL_ovS0_03H02_R_0	BY929984
E_EL_ovS0_03H03_F_0	BY921471
E_EL_ovS0_03H03_R_0	BY929985
E_EL_ovS0_03H04_R_0	BY929986
E_EL_ovS0_03H05_F_0	BY921472
E_EL_ovS0_03H06_F_0	BY921473
E_EL_ovS0_03H06_R_0	BY929987
E_EL_ovS0_03H07_F_0	BY921474
E_EL_ovS0_03H07_R_0	BY929988
E_EL_ovS0_03H08_F_0	BY921475
E_EL_ovS0_03H09_F_0	BY921476
E_EL_ovS0_03H09_R_0	BY929989
E_EL_ovS0_03H10_F_0	BY921477
E_EL_ovS0_03H10_R_0	BY929990
E_EL_ovS0_03H11_R_0	BY929991
E_EL_ovS0_03H12_R_0	BY929992
E_EL_ovS0_04A01_F_0	BY921478
E_EL_ovS0_04A02_F_0	BY921479
E_EL_ovS0_04A02_R_0	BY929993
E_EL_ovS0_04A03_F_0	BY921480
E_EL_ovS0_04A04_F_0	BY921481
E_EL_ovS0_04A04_R_0	BY929994
E_EL_ovS0_04A06_F_0	BY921482
E_EL_ovS0_04A06_R_0	BY929995
E_EL_ovS0_04A08_F_0	BY921483
E_EL_ovS0_04A08_R_0	BY929996
E_EL_ovS0_04A11_F_0	BY921484
E_EL_ovS0_04A11_R_0	BY929997
E_EL_ovS0_04A12_F_0	BY921485
E_EL_ovS0_04B01_F_0	BY921486
E_EL_ovS0_04B01_R_0	BY929998
E_EL_ovS0_04B02_F_0	BY921487
E_EL_ovS0_04B02_R_0	BY929999
E_EL_ovS0_04B03_R_0	BY930000

E_EL_ovS0_04B04_F_0	BY921488
E_EL_ovS0_04B04_R_0	BY930001
E_EL_ovS0_04B05_R_0	BY930002
E_EL_ovS0_04B07_F_0	BY921489
E_EL_ovS0_04B07_R_0	BY930003
E_EL_ovS0_04B08_F_0	BY921490
E_EL_ovS0_04B10_R_0	BY930004
E_EL_ovS0_04B11_F_0	BY921491
E_EL_ovS0_04B11_R_0	BY930005
E_EL_ovS0_04B12_F_0	BY921492
E_EL_ovS0_04B12_R_0	BY930006
E_EL_ovS0_04C01_F_0	BY921493
E_EL_ovS0_04C01_R_0	BY930007
E_EL_ovS0_04C02_F_0	BY921494
E_EL_ovS0_04C02_R_0	BY930008
E_EL_ovS0_04C04_F_0	BY921495
E_EL_ovS0_04C04_R_0	BY930009
E_EL_ovS0_04C05_F_0	BY921496
E_EL_ovS0_04C05_R_0	BY930010
E_EL_ovS0_04C06_F_0	BY921497
E_EL_ovS0_04C06_R_0	BY930011
E_EL_ovS0_04C07_F_0	BY921498
E_EL_ovS0_04C07_R_0	BY930012
E_EL_ovS0_04C08_F_0	BY921499
E_EL_ovS0_04C11_R_0	BY930013
E_EL_ovS0_04C12_F_0	BY921500
E_EL_ovS0_04C12_R_0	BY930014
E_EL_ovS0_04D02_R_0	BY930015
E_EL_ovS0_04D03_F_0	BY921501
E_EL_ovS0_04D03_R_0	BY930016
E_EL_ovS0_04D04_F_0	BY921502
E_EL_ovS0_04D04_R_0	BY930017
E_EL_ovS0_04D07_F_0	BY921503
E_EL_ovS0_04D08_F_0	BY921504
E_EL_ovS0_04D09_F_0	BY921505
E_EL_ovS0_04D10_F_0	BY921506
E_EL_ovS0_04D12_F_0	BY921507
E_EL_ovS0_04D12_R_0	BY930018
E_EL_ovS0_04E01_F_0	BY921508
E_EL_ovS0_04E01_R_0	BY930019
E_EL_ovS0_04E02_F_0	BY921509
E_EL_ovS0_04E02_R_0	BY930020
E_EL_ovS0_04E03_F_0	BY921510
E_EL_ovS0_04E04_F_0	BY921511
E_EL_ovS0_04E04_R_0	BY930021
E_EL_ovS0_04E06_F_0	BY921512
E_EL_ovS0_04E06_R_0	BY930022
E_EL_ovS0_04E07_R_0	BY930023
E_EL_ovS0_04E08_F_0	BY921513
E_EL_ovS0_04E08_R_0	BY930024
E_EL_ovS0_04E12_F_0	BY921514
E_EL_ovS0_04E12_R_0	BY930025
E_EL_ovS0_04F02_F_0	BY921515
E_EL_ovS0_04F05_F_0	BY921516

E_EL_ovS0_04F05_R_0	BY930026
E_EL_ovS0_04F08_F_0	BY921517
E_EL_ovS0_04F08_R_0	BY930027
E_EL_ovS0_04F09_F_0	BY921518
E_EL_ovS0_04F09_R_0	BY930028
E_EL_ovS0_04F10_F_0	BY921519
E_EL_ovS0_04F10_R_0	BY930029
E_EL_ovS0_04F11_R_0	BY930030
E_EL_ovS0_04F12_F_0	BY921520
E_EL_ovS0_04F12_R_0	BY930031
E_EL_ovS0_04G02_F_0	BY921521
E_EL_ovS0_04G02_R_0	BY930032
E_EL_ovS0_04G04_F_0	BY921522
E_EL_ovS0_04G04_R_0	BY930033
E_EL_ovS0_04G05_F_0	BY921523
E_EL_ovS0_04G05_R_0	BY930034
E_EL_ovS0_04G07_F_0	BY921524
E_EL_ovS0_04G07_R_0	BY930035
E_EL_ovS0_04G08_F_0	BY921525
E_EL_ovS0_04G09_R_0	BY930036
E_EL_ovS0_04G11_F_0	BY921526
E_EL_ovS0_04G11_R_0	BY930037
E_EL_ovS0_04G12_F_0	BY921527
E_EL_ovS0_04G12_R_0	BY930038
E_EL_ovS0_04H01_F_0	BY921528
E_EL_ovS0_04H01_R_0	BY930039
E_EL_ovS0_04H02_F_0	BY921529
E_EL_ovS0_04H02_R_0	BY930040
E_EL_ovS0_04H03_F_0	BY921530
E_EL_ovS0_04H04_F_0	BY921531
E_EL_ovS0_04H04_R_0	BY930041
E_EL_ovS0_04H05_F_0	BY921532
E_EL_ovS0_04H06_F_0	BY921533
E_EL_ovS0_04H06_R_0	BY930042
E_EL_ovS0_04H07_F_0	BY921534
E_EL_ovS0_04H07_R_0	BY930043
E_EL_ovS0_04H08_F_0	BY921535
E_EL_ovS0_04H09_F_0	BY921536
E_EL_ovS0_04H09_R_0	BY930044
E_EL_ovS0_04H10_F_0	BY921537
E_EL_ovS0_04H10_R_0	BY930045
E_EL_ovS0_04H11_R_0	BY930046
E_EL_ovS0_04H12_F_0	BY921538
E_EL_ovS0_04H12_R_0	BY930047
E_EL_ovS0_05A02_F_0	BY921539
E_EL_ovS0_05A02_R_0	BY930048
E_EL_ovS0_05A03_F_0	BY921540
E_EL_ovS0_05A03_R_0	BY930049
E_EL_ovS0_05A04_R_0	BY930050
E_EL_ovS0_05A05_F_0	BY921541
E_EL_ovS0_05A05_R_0	BY930051
E_EL_ovS0_05A06_F_0	BY921542
E_EL_ovS0_05A06_R_0	BY930052
E_EL_ovS0_05A07_F_0	BY921543

E_EL_ovS0_05A07_R_0	BY930053
E_EL_ovS0_05A08_F_0	BY921544
E_EL_ovS0_05A08_R_0	BY930054
E_EL_ovS0_05A09_F_0	BY921545
E_EL_ovS0_05A10_F_0	BY921546
E_EL_ovS0_05A10_R_0	BY930055
E_EL_ovS0_05A11_R_0	BY930056
E_EL_ovS0_05A12_F_0	BY921547
E_EL_ovS0_05A12_R_0	BY930057
E_EL_ovS0_05B01_R_0	BY930058
E_EL_ovS0_05B02_F_0	BY921548
E_EL_ovS0_05B02_R_0	BY930059
E_EL_ovS0_05B03_F_0	BY921549
E_EL_ovS0_05B03_R_0	BY930060
E_EL_ovS0_05B04_F_0	BY921550
E_EL_ovS0_05B04_R_0	BY930061
E_EL_ovS0_05B05_F_0	BY921551
E_EL_ovS0_05B05_R_0	BY930062
E_EL_ovS0_05B06_F_0	BY921552
E_EL_ovS0_05B06_R_0	BY930063
E_EL_ovS0_05B07_F_0	BY921553
E_EL_ovS0_05B07_R_0	BY930064
E_EL_ovS0_05B09_F_0	BY921554
E_EL_ovS0_05B09_R_0	BY930065
E_EL_ovS0_05B10_F_0	BY921555
E_EL_ovS0_05B10_R_0	BY930066
E_EL_ovS0_05B12_F_0	BY921556
E_EL_ovS0_05B12_R_0	BY930067
E_EL_ovS0_05C01_F_0	BY921557
E_EL_ovS0_05C01_R_0	BY930068
E_EL_ovS0_05C02_F_0	BY921558
E_EL_ovS0_05C02_R_0	BY930069
E_EL_ovS0_05C03_F_0	BY921559
E_EL_ovS0_05C03_R_0	BY930070
E_EL_ovS0_05C04_F_0	BY921560
E_EL_ovS0_05C04_R_0	BY930071
E_EL_ovS0_05C05_F_0	BY921561
E_EL_ovS0_05C06_F_0	BY921562
E_EL_ovS0_05C06_R_0	BY930072
E_EL_ovS0_05C08_F_0	BY921563
E_EL_ovS0_05C08_R_0	BY930073
E_EL_ovS0_05C09_F_0	BY921564
E_EL_ovS0_05C09_R_0	BY930074
E_EL_ovS0_05C10_F_0	BY921565
E_EL_ovS0_05C10_R_0	BY930075
E_EL_ovS0_05C11_F_0	BY921566
E_EL_ovS0_05C11_R_0	BY930076
E_EL_ovS0_05D01_F_0	BY921567
E_EL_ovS0_05D01_R_0	BY930077
E_EL_ovS0_05D02_F_0	BY921568
E_EL_ovS0_05D02_R_0	BY930078
E_EL_ovS0_05D03_F_0	BY921569
E_EL_ovS0_05D03_R_0	BY930079
E_EL_ovS0_05D04_F_0	BY921570

E_EL_ovS0_05D04_R_0	BY930080
E_EL_ovS0_05D05_F_0	BY921571
E_EL_ovS0_05D05_R_0	BY930081
E_EL_ovS0_05D06_F_0	BY921572
E_EL_ovS0_05D06_R_0	BY930082
E_EL_ovS0_05D07_F_0	BY921573
E_EL_ovS0_05D07_R_0	BY930083
E_EL_ovS0_05D09_F_0	BY921574
E_EL_ovS0_05D10_F_0	BY921575
E_EL_ovS0_05D10_R_0	BY930084
E_EL_ovS0_05D11_F_0	BY921576
E_EL_ovS0_05D11_R_0	BY930085
E_EL_ovS0_05D12_F_0	BY921577
E_EL_ovS0_05D12_R_0	BY930086
E_EL_ovS0_05E01_F_0	BY921578
E_EL_ovS0_05E02_F_0	BY921579
E_EL_ovS0_05E02_R_0	BY930087
E_EL_ovS0_05E03_F_0	BY921580
E_EL_ovS0_05E03_R_0	BY930088
E_EL_ovS0_05E04_R_0	BY930089
E_EL_ovS0_05E05_F_0	BY921581
E_EL_ovS0_05E06_F_0	BY921582
E_EL_ovS0_05E06_R_0	BY930090
E_EL_ovS0_05E07_F_0	BY921583
E_EL_ovS0_05E07_R_0	BY930091
E_EL_ovS0_05E08_F_0	BY921584
E_EL_ovS0_05E08_R_0	BY930092
E_EL_ovS0_05E09_F_0	BY921585
E_EL_ovS0_05E10_F_0	BY921586
E_EL_ovS0_05E11_F_0	BY921587
E_EL_ovS0_05E12_F_0	BY921588
E_EL_ovS0_05E12_R_0	BY930093
E_EL_ovS0_05F01_F_0	BY921589
E_EL_ovS0_05F01_R_0	BY930094
E_EL_ovS0_05F02_F_0	BY921590
E_EL_ovS0_05F02_R_0	BY930095
E_EL_ovS0_05F03_F_0	BY921591
E_EL_ovS0_05F03_R_0	BY930096
E_EL_ovS0_05F04_F_0	BY921592
E_EL_ovS0_05F04_R_0	BY930097
E_EL_ovS0_05F05_F_0	BY921593
E_EL_ovS0_05F05_R_0	BY930098
E_EL_ovS0_05F06_F_0	BY921594
E_EL_ovS0_05F06_R_0	BY930099
E_EL_ovS0_05F07_F_0	BY921595
E_EL_ovS0_05F08_F_0	BY921596
E_EL_ovS0_05F08_R_0	BY930100
E_EL_ovS0_05F10_F_0	BY921597
E_EL_ovS0_05F10_R_0	BY930101
E_EL_ovS0_05F11_F_0	BY921598
E_EL_ovS0_05F11_R_0	BY930102
E_EL_ovS0_05F12_F_0	BY921599
E_EL_ovS0_05F12_R_0	BY930103
E_EL_ovS0_05G01_F_0	BY921600

E_EL_ovS0_05G01_R_0	BY930104
E_EL_ovS0_05G02_F_0	BY921601
E_EL_ovS0_05G02_R_0	BY930105
E_EL_ovS0_05G03_F_0	BY921602
E_EL_ovS0_05G03_R_0	BY930106
E_EL_ovS0_05G04_F_0	BY921603
E_EL_ovS0_05G04_R_0	BY930107
E_EL_ovS0_05G06_F_0	BY921604
E_EL_ovS0_05G06_R_0	BY930108
E_EL_ovS0_05G07_F_0	BY921605
E_EL_ovS0_05G09_F_0	BY921606
E_EL_ovS0_05G09_R_0	BY930109
E_EL_ovS0_05G10_F_0	BY921607
E_EL_ovS0_05G10_R_0	BY930110
E_EL_ovS0_05G12_F_0	BY921608
E_EL_ovS0_05G12_R_0	BY930111
E_EL_ovS0_05H01_F_0	BY921609
E_EL_ovS0_05H01_R_0	BY930112
E_EL_ovS0_05H02_F_0	BY921610
E_EL_ovS0_05H02_R_0	BY930113
E_EL_ovS0_05H03_F_0	BY921611
E_EL_ovS0_05H03_R_0	BY930114
E_EL_ovS0_05H04_R_0	BY930115
E_EL_ovS0_05H05_F_0	BY921612
E_EL_ovS0_05H05_R_0	BY930116
E_EL_ovS0_05H06_F_0	BY921613
E_EL_ovS0_05H06_R_0	BY930117
E_EL_ovS0_05H07_F_0	BY921614
E_EL_ovS0_05H08_F_0	BY921615
E_EL_ovS0_05H08_R_0	BY930118
E_EL_ovS0_05H09_F_0	BY921616
E_EL_ovS0_05H09_R_0	BY930119
E_EL_ovS0_05H11_F_0	BY921617
E_EL_ovS0_05H11_R_0	BY930120
E_EL_ovS0_05H12_F_0	BY921618
E_EL_ovS0_05H12_R_0	BY930121
E_EL_ovS0_06A01_F_0	BY921619
E_EL_ovS0_06A01_R_0	BY930122
E_EL_ovS0_06A03_F_0	BY921620
E_EL_ovS0_06A03_R_0	BY930123
E_EL_ovS0_06A04_F_0	BY921621
E_EL_ovS0_06A04_R_0	BY930124
E_EL_ovS0_06A05_R_0	BY930125
E_EL_ovS0_06A06_F_0	BY921622
E_EL_ovS0_06A06_R_0	BY930126
E_EL_ovS0_06A07_F_0	BY921623
E_EL_ovS0_06A07_R_0	BY930127
E_EL_ovS0_06A08_F_0	BY921624
E_EL_ovS0_06A08_R_0	BY930128
E_EL_ovS0_06A09_F_0	BY921625
E_EL_ovS0_06A10_F_0	BY921626
E_EL_ovS0_06A10_R_0	BY930129
E_EL_ovS0_06A11_F_0	BY921627
E_EL_ovS0_06A11_R_0	BY930130

E_EL_ovS0_06A12_F_0	BY921628
E_EL_ovS0_06A12_R_0	BY930131
E_EL_ovS0_06B01_F_0	BY921629
E_EL_ovS0_06B01_R_0	BY930132
E_EL_ovS0_06B02_F_0	BY921630
E_EL_ovS0_06B02_R_0	BY930133
E_EL_ovS0_06B03_R_0	BY930134
E_EL_ovS0_06B04_F_0	BY921631
E_EL_ovS0_06B04_R_0	BY930135
E_EL_ovS0_06B05_F_0	BY921632
E_EL_ovS0_06B06_F_0	BY921633
E_EL_ovS0_06B07_F_0	BY921634
E_EL_ovS0_06B07_R_0	BY930136
E_EL_ovS0_06B08_F_0	BY921635
E_EL_ovS0_06B08_R_0	BY930137
E_EL_ovS0_06B09_F_0	BY921636
E_EL_ovS0_06B09_R_0	BY930138
E_EL_ovS0_06B10_F_0	BY921637
E_EL_ovS0_06B10_R_0	BY930139
E_EL_ovS0_06B11_R_0	BY930140
E_EL_ovS0_06B12_R_0	BY930141
E_EL_ovS0_06C01_R_0	BY930142
E_EL_ovS0_06C02_R_0	BY930143
E_EL_ovS0_06C03_F_0	BY921638
E_EL_ovS0_06C03_R_0	BY930144
E_EL_ovS0_06C04_F_0	BY921639
E_EL_ovS0_06C04_R_0	BY930145
E_EL_ovS0_06C07_F_0	BY921640
E_EL_ovS0_06C07_R_0	BY930146
E_EL_ovS0_06C08_F_0	BY921641
E_EL_ovS0_06C08_R_0	BY930147
E_EL_ovS0_06C10_F_0	BY921642
E_EL_ovS0_06C10_R_0	BY930148
E_EL_ovS0_06C11_F_0	BY921643
E_EL_ovS0_06C11_R_0	BY930149
E_EL_ovS0_06C12_F_0	BY921644
E_EL_ovS0_06C12_R_0	BY930150
E_EL_ovS0_06D01_F_0	BY921645
E_EL_ovS0_06D01_R_0	BY930151
E_EL_ovS0_06D02_F_0	BY921646
E_EL_ovS0_06D02_R_0	BY930152
E_EL_ovS0_06D03_F_0	BY921647
E_EL_ovS0_06D03_R_0	BY930153
E_EL_ovS0_06D04_F_0	BY921648
E_EL_ovS0_06D04_R_0	BY930154
E_EL_ovS0_06D05_F_0	BY921649
E_EL_ovS0_06D05_R_0	BY930155
E_EL_ovS0_06D06_F_0	BY921650
E_EL_ovS0_06D06_R_0	BY930156
E_EL_ovS0_06D07_R_0	BY930157
E_EL_ovS0_06D08_F_0	BY921651
E_EL_ovS0_06D08_R_0	BY930158
E_EL_ovS0_06D09_F_0	BY921652
E_EL_ovS0_06D09_R_0	BY930159

E_EL_ovS0_06D10_F_0	BY921653
E_EL_ovS0_06D10_R_0	BY930160
E_EL_ovS0_06D11_F_0	BY921654
E_EL_ovS0_06D11_R_0	BY930161
E_EL_ovS0_06D12_F_0	BY921655
E_EL_ovS0_06E01_F_0	BY921656
E_EL_ovS0_06E02_F_0	BY921657
E_EL_ovS0_06E02_R_0	BY930162
E_EL_ovS0_06E03_F_0	BY921658
E_EL_ovS0_06E03_R_0	BY930163
E_EL_ovS0_06E04_F_0	BY921659
E_EL_ovS0_06E04_R_0	BY930164
E_EL_ovS0_06E05_F_0	BY921660
E_EL_ovS0_06E05_R_0	BY930165
E_EL_ovS0_06E06_F_0	BY921661
E_EL_ovS0_06E06_R_0	BY930166
E_EL_ovS0_06E07_F_0	BY921662
E_EL_ovS0_06E08_F_0	BY921663
E_EL_ovS0_06E08_R_0	BY930167
E_EL_ovS0_06E09_F_0	BY921664
E_EL_ovS0_06E10_F_0	BY921665
E_EL_ovS0_06E10_R_0	BY930168
E_EL_ovS0_06E11_F_0	BY921666
E_EL_ovS0_06E12_F_0	BY921667
E_EL_ovS0_06E12_R_0	BY930169
E_EL_ovS0_06F01_F_0	BY921668
E_EL_ovS0_06F03_F_0	BY921669
E_EL_ovS0_06F03_R_0	BY930170
E_EL_ovS0_06F04_R_0	BY930171
E_EL_ovS0_06F05_F_0	BY921670
E_EL_ovS0_06F05_R_0	BY930172
E_EL_ovS0_06F06_F_0	BY921671
E_EL_ovS0_06F06_R_0	BY930173
E_EL_ovS0_06F07_F_0	BY921672
E_EL_ovS0_06F07_R_0	BY930174
E_EL_ovS0_06F08_F_0	BY921673
E_EL_ovS0_06F08_R_0	BY930175
E_EL_ovS0_06F09_F_0	BY921674
E_EL_ovS0_06F09_R_0	BY930176
E_EL_ovS0_06F10_F_0	BY921675
E_EL_ovS0_06F10_R_0	BY930177
E_EL_ovS0_06F11_R_0	BY930178
E_EL_ovS0_06F12_R_0	BY930179
E_EL_ovS0_06G01_F_0	BY921676
E_EL_ovS0_06G01_R_0	BY930180
E_EL_ovS0_06G02_F_0	BY921677
E_EL_ovS0_06G02_R_0	BY930181
E_EL_ovS0_06G03_F_0	BY921678
E_EL_ovS0_06G03_R_0	BY930182
E_EL_ovS0_06G04_F_0	BY921679
E_EL_ovS0_06G04_R_0	BY930183
E_EL_ovS0_06G05_F_0	BY921680
E_EL_ovS0_06G05_R_0	BY930184
E_EL_ovS0_06G06_R_0	BY930185

E_EL_ovS0_06G07_R_0	BY930186
E_EL_ovS0_06G08_F_0	BY921681
E_EL_ovS0_06G08_R_0	BY930187
E_EL_ovS0_06G09_F_0	BY921682
E_EL_ovS0_06G09_R_0	BY930188
E_EL_ovS0_06G10_F_0	BY921683
E_EL_ovS0_06G10_R_0	BY930189
E_EL_ovS0_06G11_F_0	BY921684
E_EL_ovS0_06G11_R_0	BY930190
E_EL_ovS0_06G12_F_0	BY921685
E_EL_ovS0_06G12_R_0	BY930191
E_EL_ovS0_06H01_F_0	BY921686
E_EL_ovS0_06H01_R_0	BY930192
E_EL_ovS0_06H02_F_0	BY921687
E_EL_ovS0_06H02_R_0	BY930193
E_EL_ovS0_06H03_F_0	BY921688
E_EL_ovS0_06H03_R_0	BY930194
E_EL_ovS0_06H04_F_0	BY921689
E_EL_ovS0_06H05_F_0	BY921690
E_EL_ovS0_06H05_R_0	BY930195
E_EL_ovS0_06H06_F_0	BY921691
E_EL_ovS0_06H06_R_0	BY930196
E_EL_ovS0_06H07_F_0	BY921692
E_EL_ovS0_06H07_R_0	BY930197
E_EL_ovS0_06H08_F_0	BY921693
E_EL_ovS0_06H08_R_0	BY930198
E_EL_ovS0_06H09_R_0	BY930199
E_EL_ovS0_06H10_F_0	BY921694
E_EL_ovS0_06H10_R_0	BY930200
E_EL_ovS0_06H11_F_0	BY921695
E_EL_ovS0_06H11_R_0	BY930201
E_EL_ovS0_06H12_F_0	BY921696
E_EL_ovS0_06H12_R_0	BY930202
E_EL_ovS0_07A01_F_0	BY921697
E_EL_ovS0_07A01_R_0	BY930203
E_EL_ovS0_07A02_F_0	BY921698
E_EL_ovS0_07A03_F_0	BY921699
E_EL_ovS0_07A04_F_0	BY921700
E_EL_ovS0_07A06_F_0	BY921701
E_EL_ovS0_07A07_F_0	BY921702
E_EL_ovS0_07A07_R_0	BY930204
E_EL_ovS0_07A08_F_0	BY921703
E_EL_ovS0_07A08_R_0	BY930205
E_EL_ovS0_07A09_F_0	BY921704
E_EL_ovS0_07A09_R_0	BY930206
E_EL_ovS0_07A10_F_0	BY921705
E_EL_ovS0_07A10_R_0	BY930207
E_EL_ovS0_07A11_F_0	BY921706
E_EL_ovS0_07A11_R_0	BY930208
E_EL_ovS0_07A12_F_0	BY921707
E_EL_ovS0_07A12_R_0	BY930209
E_EL_ovS0_07B01_F_0	BY921708
E_EL_ovS0_07B01_R_0	BY930210
E_EL_ovS0_07B02_F_0	BY921709

E_EL_ovS0_07B04_F_0	BY921710
E_EL_ovS0_07B05_F_0	BY921711
E_EL_ovS0_07B05_R_0	BY930211
E_EL_ovS0_07B06_F_0	BY921712
E_EL_ovS0_07B07_F_0	BY921713
E_EL_ovS0_07B07_R_0	BY930212
E_EL_ovS0_07B08_F_0	BY921714
E_EL_ovS0_07B08_R_0	BY930213
E_EL_ovS0_07B09_F_0	BY921715
E_EL_ovS0_07B09_R_0	BY930214
E_EL_ovS0_07B10_F_0	BY921716
E_EL_ovS0_07B10_R_0	BY930215
E_EL_ovS0_07B11_R_0	BY930216
E_EL_ovS0_07B12_R_0	BY930217
E_EL_ovS0_07C01_F_0	BY921717
E_EL_ovS0_07C01_R_0	BY930218
E_EL_ovS0_07C02_F_0	BY921718
E_EL_ovS0_07C02_R_0	BY930219
E_EL_ovS0_07C03_F_0	BY921719
E_EL_ovS0_07C03_R_0	BY930220
E_EL_ovS0_07C04_R_0	BY930221
E_EL_ovS0_07C05_F_0	BY921720
E_EL_ovS0_07C05_R_0	BY930222
E_EL_ovS0_07C07_F_0	BY921721
E_EL_ovS0_07C07_R_0	BY930223
E_EL_ovS0_07C08_F_0	BY921722
E_EL_ovS0_07C08_R_0	BY930224
E_EL_ovS0_07C09_F_0	BY921723
E_EL_ovS0_07C09_R_0	BY930225
E_EL_ovS0_07C10_F_0	BY921724
E_EL_ovS0_07C10_R_0	BY930226
E_EL_ovS0_07C11_F_0	BY921725
E_EL_ovS0_07C11_R_0	BY930227
E_EL_ovS0_07C12_F_0	BY921726
E_EL_ovS0_07C12_R_0	BY930228
E_EL_ovS0_07D01_F_0	BY921727
E_EL_ovS0_07D02_F_0	BY921728
E_EL_ovS0_07D02_R_0	BY930229
E_EL_ovS0_07D03_F_0	BY921729
E_EL_ovS0_07D03_R_0	BY930230
E_EL_ovS0_07D04_F_0	BY921730
E_EL_ovS0_07D05_F_0	BY921731
E_EL_ovS0_07D05_R_0	BY930231
E_EL_ovS0_07D06_R_0	BY930232
E_EL_ovS0_07D07_F_0	BY921732
E_EL_ovS0_07D07_R_0	BY930233
E_EL_ovS0_07D08_F_0	BY921733
E_EL_ovS0_07D09_F_0	BY921734
E_EL_ovS0_07D09_R_0	BY930234
E_EL_ovS0_07D10_F_0	BY921735
E_EL_ovS0_07D10_R_0	BY930235
E_EL_ovS0_07D11_F_0	BY921736
E_EL_ovS0_07D11_R_0	BY930236
E_EL_ovS0_07D12_F_0	BY921737

E_EL_ovS0_07D12_R_0	BY930237
E_EL_ovS0_07E01_F_0	BY921738
E_EL_ovS0_07E01_R_0	BY930238
E_EL_ovS0_07E03_F_0	BY921739
E_EL_ovS0_07E03_R_0	BY930239
E_EL_ovS0_07E04_F_0	BY921740
E_EL_ovS0_07E04_R_0	BY930240
E_EL_ovS0_07E05_F_0	BY921741
E_EL_ovS0_07E05_R_0	BY930241
E_EL_ovS0_07E06_F_0	BY921742
E_EL_ovS0_07E06_R_0	BY930242
E_EL_ovS0_07E07_F_0	BY921743
E_EL_ovS0_07E07_R_0	BY930243
E_EL_ovS0_07E08_F_0	BY921744
E_EL_ovS0_07E08_R_0	BY930244
E_EL_ovS0_07E09_F_0	BY921745
E_EL_ovS0_07E10_F_0	BY921746
E_EL_ovS0_07E11_F_0	BY921747
E_EL_ovS0_07E11_R_0	BY930245
E_EL_ovS0_07E12_F_0	BY921748
E_EL_ovS0_07F01_F_0	BY921749
E_EL_ovS0_07F01_R_0	BY930246
E_EL_ovS0_07F02_R_0	BY930247
E_EL_ovS0_07F03_F_0	BY921750
E_EL_ovS0_07F03_R_0	BY930248
E_EL_ovS0_07F05_F_0	BY921751
E_EL_ovS0_07F05_R_0	BY930249
E_EL_ovS0_07F06_F_0	BY921752
E_EL_ovS0_07F06_R_0	BY930250
E_EL_ovS0_07F07_F_0	BY921753
E_EL_ovS0_07F07_R_0	BY930251
E_EL_ovS0_07F08_F_0	BY921754
E_EL_ovS0_07F08_R_0	BY930252
E_EL_ovS0_07F09_F_0	BY921755
E_EL_ovS0_07F09_R_0	BY930253
E_EL_ovS0_07F10_F_0	BY921756
E_EL_ovS0_07F10_R_0	BY930254
E_EL_ovS0_07F11_R_0	BY930255
E_EL_ovS0_07F12_R_0	BY930256
E_EL_ovS0_07G01_F_0	BY921757
E_EL_ovS0_07G01_R_0	BY930257
E_EL_ovS0_07G04_F_0	BY921758
E_EL_ovS0_07G04_R_0	BY930258
E_EL_ovS0_07G05_F_0	BY921759
E_EL_ovS0_07G05_R_0	BY930259
E_EL_ovS0_07G06_F_0	BY921760
E_EL_ovS0_07G06_R_0	BY930260
E_EL_ovS0_07G07_F_0	BY921761
E_EL_ovS0_07G07_R_0	BY930261
E_EL_ovS0_07G08_F_0	BY921762
E_EL_ovS0_07G08_R_0	BY930262
E_EL_ovS0_07G09_R_0	BY930263
E_EL_ovS0_07G10_F_0	BY921763
E_EL_ovS0_07G10_R_0	BY930264

E_EL_ovS0_07G11_F_0	BY921764
E_EL_ovS0_07G11_R_0	BY930265
E_EL_ovS0_07H01_F_0	BY921765
E_EL_ovS0_07H01_R_0	BY930266
E_EL_ovS0_07H02_F_0	BY921766
E_EL_ovS0_07H02_R_0	BY930267
E_EL_ovS0_07H03_F_0	BY921767
E_EL_ovS0_07H03_R_0	BY930268
E_EL_ovS0_07H04_F_0	BY921768
E_EL_ovS0_07H04_R_0	BY930269
E_EL_ovS0_07H05_F_0	BY921769
E_EL_ovS0_07H05_R_0	BY930270
E_EL_ovS0_07H06_F_0	BY921770
E_EL_ovS0_07H06_R_0	BY930271
E_EL_ovS0_07H07_F_0	BY921771
E_EL_ovS0_07H07_R_0	BY930272
E_EL_ovS0_07H08_F_0	BY921772
E_EL_ovS0_07H08_R_0	BY930273
E_EL_ovS0_07H09_F_0	BY921773
E_EL_ovS0_07H09_R_0	BY930274
E_EL_ovS0_07H10_F_0	BY921774
E_EL_ovS0_07H10_R_0	BY930275
E_EL_ovS0_07H11_R_0	BY930276
E_EL_ovS0_07H12_F_0	BY921775
E_EL_ovS0_07H12_R_0	BY930277
E_EL_ovS0_08A01_R_0	BY930278
E_EL_ovS0_08A02_R_0	BY930279
E_EL_ovS0_08A03_F_0	BY921776
E_EL_ovS0_08A03_R_0	BY930280
E_EL_ovS0_08A04_F_0	BY921777
E_EL_ovS0_08A04_R_0	BY930281
E_EL_ovS0_08A05_F_0	BY921778
E_EL_ovS0_08A05_R_0	BY930282
E_EL_ovS0_08A06_F_0	BY921779
E_EL_ovS0_08A06_R_0	BY930283
E_EL_ovS0_08A07_F_0	BY921780
E_EL_ovS0_08A07_R_0	BY930284
E_EL_ovS0_08A08_F_0	BY921781
E_EL_ovS0_08A08_R_0	BY930285
E_EL_ovS0_08A10_F_0	BY921782
E_EL_ovS0_08A10_R_0	BY930286
E_EL_ovS0_08A11_F_0	BY921783
E_EL_ovS0_08A12_F_0	BY921784
E_EL_ovS0_08A12_R_0	BY930287
E_EL_ovS0_08B01_F_0	BY921785
E_EL_ovS0_08B01_R_0	BY930288
E_EL_ovS0_08B02_F_0	BY921786
E_EL_ovS0_08B02_R_0	BY930289
E_EL_ovS0_08B03_F_0	BY921787
E_EL_ovS0_08B03_R_0	BY930290
E_EL_ovS0_08B04_F_0	BY921788
E_EL_ovS0_08B04_R_0	BY930291
E_EL_ovS0_08B05_F_0	BY921789
E_EL_ovS0_08B05_R_0	BY930292

E_EL_ovS0_08B06_F_0	BY921790
E_EL_ovS0_08B06_R_0	BY930293
E_EL_ovS0_08B07_F_0	BY921791
E_EL_ovS0_08B09_F_0	BY921792
E_EL_ovS0_08B09_R_0	BY930294
E_EL_ovS0_08B11_R_0	BY930295
E_EL_ovS0_08B12_R_0	BY930296
E_EL_ovS0_08C01_F_0	BY921793
E_EL_ovS0_08C01_R_0	BY930297
E_EL_ovS0_08C02_F_0	BY921794
E_EL_ovS0_08C02_R_0	BY930298
E_EL_ovS0_08C03_F_0	BY921795
E_EL_ovS0_08C03_R_0	BY930299
E_EL_ovS0_08C04_F_0	BY921796
E_EL_ovS0_08C04_R_0	BY930300
E_EL_ovS0_08C05_F_0	BY921797
E_EL_ovS0_08C05_R_0	BY930301
E_EL_ovS0_08C06_F_0	BY921798
E_EL_ovS0_08C07_F_0	BY921799
E_EL_ovS0_08C07_R_0	BY930302
E_EL_ovS0_08C08_R_0	BY930303
E_EL_ovS0_08C09_F_0	BY921800
E_EL_ovS0_08C10_F_0	BY921801
E_EL_ovS0_08C10_R_0	BY930304
E_EL_ovS0_08C11_F_0	BY921802
E_EL_ovS0_08C11_R_0	BY930305
E_EL_ovS0_08C12_F_0	BY921803
E_EL_ovS0_08C12_R_0	BY930306
E_EL_ovS0_08D01_F_0	BY921804
E_EL_ovS0_08D01_R_0	BY930307
E_EL_ovS0_08D02_F_0	BY921805
E_EL_ovS0_08D02_R_0	BY930308
E_EL_ovS0_08D03_F_0	BY921806
E_EL_ovS0_08D03_R_0	BY930309
E_EL_ovS0_08D04_F_0	BY921807
E_EL_ovS0_08D04_R_0	BY930310
E_EL_ovS0_08D05_F_0	BY921808
E_EL_ovS0_08D05_R_0	BY930311
E_EL_ovS0_08D06_R_0	BY930312
E_EL_ovS0_08D07_F_0	BY921809
E_EL_ovS0_08D07_R_0	BY930313
E_EL_ovS0_08D08_F_0	BY921810
E_EL_ovS0_08D08_R_0	BY930314
E_EL_ovS0_08D09_F_0	BY921811
E_EL_ovS0_08D09_R_0	BY930315
E_EL_ovS0_08D10_F_0	BY921812
E_EL_ovS0_08D10_R_0	BY930316
E_EL_ovS0_08D11_F_0	BY921813
E_EL_ovS0_08D11_R_0	BY930317
E_EL_ovS0_08D12_F_0	BY921814
E_EL_ovS0_08D12_R_0	BY930318
E_EL_ovS0_08E01_F_0	BY921815
E_EL_ovS0_08E03_F_0	BY921816
E_EL_ovS0_08E03_R_0	BY930319

E_EL_ovS0_08E04_F_0	BY921817
E_EL_ovS0_08E04_R_0	BY930320
E_EL_ovS0_08E06_F_0	BY921818
E_EL_ovS0_08E06_R_0	BY930321
E_EL_ovS0_08E07_F_0	BY921819
E_EL_ovS0_08E10_F_0	BY921820
E_EL_ovS0_08E10_R_0	BY930322
E_EL_ovS0_08E11_F_0	BY921821
E_EL_ovS0_08E12_F_0	BY921822
E_EL_ovS0_08F01_F_0	BY921823
E_EL_ovS0_08F01_R_0	BY930323
E_EL_ovS0_08F02_R_0	BY930324
E_EL_ovS0_08F04_F_0	BY921824
E_EL_ovS0_08F04_R_0	BY930325
E_EL_ovS0_08F05_F_0	BY921825
E_EL_ovS0_08F05_R_0	BY930326
E_EL_ovS0_08F06_F_0	BY921826
E_EL_ovS0_08F06_R_0	BY930327
E_EL_ovS0_08F07_F_0	BY921827
E_EL_ovS0_08F07_R_0	BY930328
E_EL_ovS0_08F08_F_0	BY921828
E_EL_ovS0_08F08_R_0	BY930329
E_EL_ovS0_08F09_R_0	BY930330
E_EL_ovS0_08F10_F_0	BY921829
E_EL_ovS0_08F10_R_0	BY930331
E_EL_ovS0_08F11_R_0	BY930332
E_EL_ovS0_08F12_R_0	BY930333
E_EL_ovS0_08G01_F_0	BY921830
E_EL_ovS0_08G02_F_0	BY921831
E_EL_ovS0_08G02_R_0	BY930334
E_EL_ovS0_08G03_F_0	BY921832
E_EL_ovS0_08G03_R_0	BY930335
E_EL_ovS0_08G04_F_0	BY921833
E_EL_ovS0_08G04_R_0	BY930336
E_EL_ovS0_08G06_F_0	BY921834
E_EL_ovS0_08G06_R_0	BY930337
E_EL_ovS0_08G08_F_0	BY921835
E_EL_ovS0_08G08_R_0	BY930338
E_EL_ovS0_08G09_F_0	BY921836
E_EL_ovS0_08G11_F_0	BY921837
E_EL_ovS0_08G11_R_0	BY930339
E_EL_ovS0_08G12_F_0	BY921838
E_EL_ovS0_08G12_R_0	BY930340
E_EL_ovS0_08H02_F_0	BY921839
E_EL_ovS0_08H02_R_0	BY930341
E_EL_ovS0_08H03_F_0	BY921840
E_EL_ovS0_08H04_F_0	BY921841
E_EL_ovS0_08H04_R_0	BY930342
E_EL_ovS0_08H05_F_0	BY921842
E_EL_ovS0_08H05_R_0	BY930343
E_EL_ovS0_08H06_F_0	BY921843
E_EL_ovS0_08H06_R_0	BY930344
E_EL_ovS0_08H07_F_0	BY921844
E_EL_ovS0_08H07_R_0	BY930345

E_EL_ovS0_08H08_F_0	BY921845
E_EL_ovS0_08H08_R_0	BY930346
E_EL_ovS0_08H09_F_0	BY921846
E_EL_ovS0_08H09_R_0	BY930347
E_EL_ovS0_08H10_F_0	BY921847
E_EL_ovS0_08H10_R_0	BY930348
E_EL_ovS0_08H11_F_0	BY921848
E_EL_ovS0_08H11_R_0	BY930349
E_EL_ovS0_08H12_F_0	BY921849
E_EL_ovS0_08H12_R_0	BY930350
E_EL_ovS0_09A02_F_0	BY921850
E_EL_ovS0_09A02_R_0	BY930351
E_EL_ovS0_09A03_F_0	BY921851
E_EL_ovS0_09A03_R_0	BY930352
E_EL_ovS0_09A04_R_0	BY930353
E_EL_ovS0_09A05_F_0	BY921852
E_EL_ovS0_09A05_R_0	BY930354
E_EL_ovS0_09A06_F_0	BY921853
E_EL_ovS0_09A06_R_0	BY930355
E_EL_ovS0_09A07_F_0	BY921854
E_EL_ovS0_09A07_R_0	BY930356
E_EL_ovS0_09A08_F_0	BY921855
E_EL_ovS0_09A08_R_0	BY930357
E_EL_ovS0_09A09_F_0	BY921856
E_EL_ovS0_09A09_R_0	BY930358
E_EL_ovS0_09A11_F_0	BY921857
E_EL_ovS0_09A11_R_0	BY930359
E_EL_ovS0_09A12_F_0	BY921858
E_EL_ovS0_09A12_R_0	BY930360
E_EL_ovS0_09B01_F_0	BY921859
E_EL_ovS0_09B01_R_0	BY930361
E_EL_ovS0_09B02_F_0	BY921860
E_EL_ovS0_09B02_R_0	BY930362
E_EL_ovS0_09B03_F_0	BY921861
E_EL_ovS0_09B03_R_0	BY930363
E_EL_ovS0_09B04_F_0	BY921862
E_EL_ovS0_09B04_R_0	BY930364
E_EL_ovS0_09B05_F_0	BY921863
E_EL_ovS0_09B05_R_0	BY930365
E_EL_ovS0_09B06_F_0	BY921864
E_EL_ovS0_09B06_R_0	BY930366
E_EL_ovS0_09B07_R_0	BY930367
E_EL_ovS0_09B08_F_0	BY921865
E_EL_ovS0_09B08_R_0	BY930368
E_EL_ovS0_09B09_F_0	BY921866
E_EL_ovS0_09B09_R_0	BY930369
E_EL_ovS0_09B10_F_0	BY921867
E_EL_ovS0_09B10_R_0	BY930370
E_EL_ovS0_09B11_R_0	BY930371
E_EL_ovS0_09B12_R_0	BY930372
E_EL_ovS0_09C01_R_0	BY930373
E_EL_ovS0_09C02_F_0	BY921868
E_EL_ovS0_09C02_R_0	BY930374
E_EL_ovS0_09C04_F_0	BY921869

E_EL_ovS0_09C04_R_0	BY930375
E_EL_ovS0_09C05_R_0	BY930376
E_EL_ovS0_09C06_R_0	BY930377
E_EL_ovS0_09C07_F_0	BY921870
E_EL_ovS0_09C07_R_0	BY930378
E_EL_ovS0_09C08_F_0	BY921871
E_EL_ovS0_09C08_R_0	BY930379
E_EL_ovS0_09C09_F_0	BY921872
E_EL_ovS0_09C09_R_0	BY930380
E_EL_ovS0_09C10_F_0	BY921873
E_EL_ovS0_09C10_R_0	BY930381
E_EL_ovS0_09C11_F_0	BY921874
E_EL_ovS0_09C11_R_0	BY930382
E_EL_ovS0_09C12_F_0	BY921875
E_EL_ovS0_09D01_F_0	BY921876
E_EL_ovS0_09D01_R_0	BY930383
E_EL_ovS0_09D02_F_0	BY921877
E_EL_ovS0_09D02_R_0	BY930384
E_EL_ovS0_09D03_F_0	BY921878
E_EL_ovS0_09D03_R_0	BY930385
E_EL_ovS0_09D04_F_0	BY921879
E_EL_ovS0_09D04_R_0	BY930386
E_EL_ovS0_09D05_F_0	BY921880
E_EL_ovS0_09D05_R_0	BY930387
E_EL_ovS0_09D06_F_0	BY921881
E_EL_ovS0_09D06_R_0	BY930388
E_EL_ovS0_09D07_R_0	BY930389
E_EL_ovS0_09D08_F_0	BY921882
E_EL_ovS0_09D08_R_0	BY930390
E_EL_ovS0_09D09_R_0	BY930391
E_EL_ovS0_09D10_R_0	BY930392
E_EL_ovS0_09D12_F_0	BY921883
E_EL_ovS0_09D12_R_0	BY930393
E_EL_ovS0_09E01_F_0	BY921884
E_EL_ovS0_09E01_R_0	BY930394
E_EL_ovS0_09E02_F_0	BY921885
E_EL_ovS0_09E02_R_0	BY930395
E_EL_ovS0_09E03_F_0	BY921886
E_EL_ovS0_09E03_R_0	BY930396
E_EL_ovS0_09E04_F_0	BY921887
E_EL_ovS0_09E04_R_0	BY930397
E_EL_ovS0_09E05_F_0	BY921888
E_EL_ovS0_09E05_R_0	BY930398
E_EL_ovS0_09E06_F_0	BY921889
E_EL_ovS0_09E06_R_0	BY930399
E_EL_ovS0_09E07_F_0	BY921890
E_EL_ovS0_09E07_R_0	BY930400
E_EL_ovS0_09E08_F_0	BY921891
E_EL_ovS0_09E08_R_0	BY930401
E_EL_ovS0_09E09_F_0	BY921892
E_EL_ovS0_09E11_F_0	BY921893
E_EL_ovS0_09E11_R_0	BY930402
E_EL_ovS0_09E12_F_0	BY921894
E_EL_ovS0_09E12_R_0	BY930403

E_EL_ovS0_09F01_F_0	BY921895
E_EL_ovS0_09F01_R_0	BY930404
E_EL_ovS0_09F02_R_0	BY930405
E_EL_ovS0_09F03_F_0	BY921896
E_EL_ovS0_09F03_R_0	BY930406
E_EL_ovS0_09F04_F_0	BY921897
E_EL_ovS0_09F04_R_0	BY930407
E_EL_ovS0_09F05_F_0	BY921898
E_EL_ovS0_09F05_R_0	BY930408
E_EL_ovS0_09F06_F_0	BY921899
E_EL_ovS0_09F06_R_0	BY930409
E_EL_ovS0_09F07_F_0	BY921900
E_EL_ovS0_09F07_R_0	BY930410
E_EL_ovS0_09F08_F_0	BY921901
E_EL_ovS0_09F08_R_0	BY930411
E_EL_ovS0_09F09_F_0	BY921902
E_EL_ovS0_09F09_R_0	BY930412
E_EL_ovS0_09F10_F_0	BY921903
E_EL_ovS0_09F10_R_0	BY930413
E_EL_ovS0_09F11_R_0	BY930414
E_EL_ovS0_09F12_R_0	BY930415
E_EL_ovS0_09G01_F_0	BY921904
E_EL_ovS0_09G01_R_0	BY930416
E_EL_ovS0_09G02_R_0	BY930417
E_EL_ovS0_09G03_F_0	BY921905
E_EL_ovS0_09G03_R_0	BY930418
E_EL_ovS0_09G04_F_0	BY921906
E_EL_ovS0_09G04_R_0	BY930419
E_EL_ovS0_09G05_F_0	BY921907
E_EL_ovS0_09G05_R_0	BY930420
E_EL_ovS0_09G06_F_0	BY921908
E_EL_ovS0_09G06_R_0	BY930421
E_EL_ovS0_09G07_F_0	BY921909
E_EL_ovS0_09G07_R_0	BY930422
E_EL_ovS0_09G08_F_0	BY921910
E_EL_ovS0_09G08_R_0	BY930423
E_EL_ovS0_09G09_F_0	BY921911
E_EL_ovS0_09G09_R_0	BY930424
E_EL_ovS0_09G10_R_0	BY930425
E_EL_ovS0_09G11_F_0	BY921912
E_EL_ovS0_09G11_R_0	BY930426
E_EL_ovS0_09G12_F_0	BY921913
E_EL_ovS0_09G12_R_0	BY930427
E_EL_ovS0_09H01_F_0	BY921914
E_EL_ovS0_09H01_R_0	BY930428
E_EL_ovS0_09H02_F_0	BY921915
E_EL_ovS0_09H02_R_0	BY930429
E_EL_ovS0_09H03_F_0	BY921916
E_EL_ovS0_09H04_F_0	BY921917
E_EL_ovS0_09H04_R_0	BY930430
E_EL_ovS0_09H05_F_0	BY921918
E_EL_ovS0_09H05_R_0	BY930431
E_EL_ovS0_09H07_R_0	BY930432
E_EL_ovS0_09H08_F_0	BY921919

E_EL_ovS0_09H08_R_0	BY930433
E_EL_ovS0_09H09_F_0	BY921920
E_EL_ovS0_09H09_R_0	BY930434
E_EL_ovS0_09H10_F_0	BY921921
E_EL_ovS0_09H10_R_0	BY930435
E_EL_ovS0_09H12_F_0	BY921922
E_EL_ovS0_09H12_R_0	BY930436
E_EL_ovS0_10A01_F_0	BY921923
E_EL_ovS0_10A01_R_0	BY930437
E_EL_ovS0_10A02_R_0	BY930438
E_EL_ovS0_10A03_F_0	BY921924
E_EL_ovS0_10A03_R_0	BY930439
E_EL_ovS0_10A04_F_0	BY921925
E_EL_ovS0_10A04_R_0	BY930440
E_EL_ovS0_10A05_F_0	BY921926
E_EL_ovS0_10A05_R_0	BY930441
E_EL_ovS0_10A06_F_0	BY921927
E_EL_ovS0_10A06_R_0	BY930442
E_EL_ovS0_10A07_F_0	BY921928
E_EL_ovS0_10A07_R_0	BY930443
E_EL_ovS0_10A08_F_0	BY921929
E_EL_ovS0_10A09_F_0	BY921930
E_EL_ovS0_10A09_R_0	BY930444
E_EL_ovS0_10A10_F_0	BY921931
E_EL_ovS0_10A10_R_0	BY930445
E_EL_ovS0_10A11_F_0	BY921932
E_EL_ovS0_10A11_R_0	BY930446
E_EL_ovS0_10A12_F_0	BY921933
E_EL_ovS0_10A12_R_0	BY930447
E_EL_ovS0_10B01_F_0	BY921934
E_EL_ovS0_10B02_F_0	BY921935
E_EL_ovS0_10B02_R_0	BY930448
E_EL_ovS0_10B03_F_0	BY921936
E_EL_ovS0_10B03_R_0	BY930449
E_EL_ovS0_10B05_F_0	BY921937
E_EL_ovS0_10B05_R_0	BY930450
E_EL_ovS0_10B06_R_0	BY930451
E_EL_ovS0_10B07_F_0	BY921938
E_EL_ovS0_10B07_R_0	BY930452
E_EL_ovS0_10B08_R_0	BY930453
E_EL_ovS0_10B09_F_0	BY921939
E_EL_ovS0_10B10_F_0	BY921940
E_EL_ovS0_10B10_R_0	BY930454
E_EL_ovS0_10B11_R_0	BY930455
E_EL_ovS0_10B12_R_0	BY930456
E_EL_ovS0_10C01_F_0	BY921941
E_EL_ovS0_10C01_R_0	BY930457
E_EL_ovS0_10C02_F_0	BY921942
E_EL_ovS0_10C02_R_0	BY930458
E_EL_ovS0_10C03_F_0	BY921943
E_EL_ovS0_10C03_R_0	BY930459
E_EL_ovS0_10C04_F_0	BY921944
E_EL_ovS0_10C04_R_0	BY930460
E_EL_ovS0_10C05_F_0	BY921945

E_EL_ovS0_10C06_F_0	BY921946
E_EL_ovS0_10C06_R_0	BY930461
E_EL_ovS0_10C08_F_0	BY921947
E_EL_ovS0_10C08_R_0	BY930462
E_EL_ovS0_10C10_F_0	BY921948
E_EL_ovS0_10C10_R_0	BY930463
E_EL_ovS0_10C11_F_0	BY921949
E_EL_ovS0_10C12_F_0	BY921950
E_EL_ovS0_10C12_R_0	BY930464
E_EL_ovS0_10D01_F_0	BY921951
E_EL_ovS0_10D01_R_0	BY930465
E_EL_ovS0_10D02_F_0	BY921952
E_EL_ovS0_10D02_R_0	BY930466
E_EL_ovS0_10D03_F_0	BY921953
E_EL_ovS0_10D03_R_0	BY930467
E_EL_ovS0_10D04_F_0	BY921954
E_EL_ovS0_10D04_R_0	BY930468
E_EL_ovS0_10D05_F_0	BY921955
E_EL_ovS0_10D05_R_0	BY930469
E_EL_ovS0_10D06_R_0	BY930470
E_EL_ovS0_10D07_F_0	BY921956
E_EL_ovS0_10D07_R_0	BY930471
E_EL_ovS0_10D08_F_0	BY921957
E_EL_ovS0_10D08_R_0	BY930472
E_EL_ovS0_10D09_F_0	BY921958
E_EL_ovS0_10D09_R_0	BY930473
E_EL_ovS0_10D10_F_0	BY921959
E_EL_ovS0_10D10_R_0	BY930474
E_EL_ovS0_10D11_R_0	BY930475
E_EL_ovS0_10D12_F_0	BY921960
E_EL_ovS0_10D12_R_0	BY930476
E_EL_ovS0_10E01_F_0	BY921961
E_EL_ovS0_10E01_R_0	BY930477
E_EL_ovS0_10E02_R_0	BY930478
E_EL_ovS0_10E03_F_0	BY921962
E_EL_ovS0_10E03_R_0	BY930479
E_EL_ovS0_10E04_F_0	BY921963
E_EL_ovS0_10E04_R_0	BY930480
E_EL_ovS0_10E05_F_0	BY921964
E_EL_ovS0_10E05_R_0	BY930481
E_EL_ovS0_10E06_F_0	BY921965
E_EL_ovS0_10E06_R_0	BY930482
E_EL_ovS0_10E07_F_0	BY921966
E_EL_ovS0_10E07_R_0	BY930483
E_EL_ovS0_10E08_F_0	BY921967
E_EL_ovS0_10E08_R_0	BY930484
E_EL_ovS0_10E10_F_0	BY921968
E_EL_ovS0_10E10_R_0	BY930485
E_EL_ovS0_10E11_F_0	BY921969
E_EL_ovS0_10F01_F_0	BY921970
E_EL_ovS0_10F01_R_0	BY930486
E_EL_ovS0_10F02_R_0	BY930487
E_EL_ovS0_10F03_F_0	BY921971
E_EL_ovS0_10F03_R_0	BY930488

E_EL_ovS0_10F05_R_0	BY930489
E_EL_ovS0_10F06_F_0	BY921972
E_EL_ovS0_10F06_R_0	BY930490
E_EL_ovS0_10F07_F_0	BY921973
E_EL_ovS0_10F07_R_0	BY930491
E_EL_ovS0_10F08_F_0	BY921974
E_EL_ovS0_10F08_R_0	BY930492
E_EL_ovS0_10F09_F_0	BY921975
E_EL_ovS0_10F09_R_0	BY930493
E_EL_ovS0_10F10_F_0	BY921976
E_EL_ovS0_10F10_R_0	BY930494
E_EL_ovS0_10F11_R_0	BY930495
E_EL_ovS0_10F12_R_0	BY930496
E_EL_ovS0_10G01_F_0	BY921977
E_EL_ovS0_10G01_R_0	BY930497
E_EL_ovS0_10G02_F_0	BY921978
E_EL_ovS0_10G02_R_0	BY930498
E_EL_ovS0_10G03_F_0	BY921979
E_EL_ovS0_10G03_R_0	BY930499
E_EL_ovS0_10G04_F_0	BY921980
E_EL_ovS0_10G04_R_0	BY930500
E_EL_ovS0_10G05_F_0	BY921981
E_EL_ovS0_10G05_R_0	BY930501
E_EL_ovS0_10G06_F_0	BY921982
E_EL_ovS0_10G06_R_0	BY930502
E_EL_ovS0_10G07_F_0	BY921983
E_EL_ovS0_10G07_R_0	BY930503
E_EL_ovS0_10G08_F_0	BY921984
E_EL_ovS0_10G08_R_0	BY930504
E_EL_ovS0_10G09_F_0	BY921985
E_EL_ovS0_10G09_R_0	BY930505
E_EL_ovS0_10G10_F_0	BY921986
E_EL_ovS0_10G10_R_0	BY930506
E_EL_ovS0_10G11_F_0	BY921987
E_EL_ovS0_10G12_F_0	BY921988
E_EL_ovS0_10G12_R_0	BY930507
E_EL_ovS0_10H01_F_0	BY921989
E_EL_ovS0_10H01_R_0	BY930508
E_EL_ovS0_10H03_F_0	BY921990
E_EL_ovS0_10H03_R_0	BY930509
E_EL_ovS0_10H04_F_0	BY921991
E_EL_ovS0_10H04_R_0	BY930510
E_EL_ovS0_10H05_F_0	BY921992
E_EL_ovS0_10H05_R_0	BY930511
E_EL_ovS0_10H06_F_0	BY921993
E_EL_ovS0_10H06_R_0	BY930512
E_EL_ovS0_10H08_F_0	BY921994
E_EL_ovS0_10H08_R_0	BY930513
E_EL_ovS0_10H09_F_0	BY921995
E_EL_ovS0_10H09_R_0	BY930514
E_EL_ovS0_10H10_F_0	BY921996
E_EL_ovS0_10H10_R_0	BY930515
E_EL_ovS0_10H11_F_0	BY921997
E_EL_ovS0_10H11_R_0	BY930516

E_EL_ovS0_10H12_F_0	BY921998
E_EL_ovS0_10H12_R_0	BY930517
E_EL_ovS0_11A01_F_0	BY921999
E_EL_ovS0_11A02_R_0	BY930518
E_EL_ovS0_11A03_F_0	BY922000
E_EL_ovS0_11A03_R_0	BY930519
E_EL_ovS0_11A04_F_0	BY922001
E_EL_ovS0_11A06_F_0	BY922002
E_EL_ovS0_11A06_R_0	BY930520
E_EL_ovS0_11A07_F_0	BY922003
E_EL_ovS0_11A07_R_0	BY930521
E_EL_ovS0_11A08_F_0	BY922004
E_EL_ovS0_11A08_R_0	BY930522
E_EL_ovS0_11A10_F_0	BY922005
E_EL_ovS0_11A10_R_0	BY930523
E_EL_ovS0_11A11_F_0	BY922006
E_EL_ovS0_11A11_R_0	BY930524
E_EL_ovS0_11A12_F_0	BY922007
E_EL_ovS0_11A12_R_0	BY930525
E_EL_ovS0_11B01_F_0	BY922008
E_EL_ovS0_11B01_R_0	BY930526
E_EL_ovS0_11B02_F_0	BY922009
E_EL_ovS0_11B02_R_0	BY930527
E_EL_ovS0_11B03_F_0	BY922010
E_EL_ovS0_11B03_R_0	BY930528
E_EL_ovS0_11B04_F_0	BY922011
E_EL_ovS0_11B04_R_0	BY930529
E_EL_ovS0_11B05_F_0	BY922012
E_EL_ovS0_11B05_R_0	BY930530
E_EL_ovS0_11B06_F_0	BY922013
E_EL_ovS0_11B06_R_0	BY930531
E_EL_ovS0_11B07_F_0	BY922014
E_EL_ovS0_11B07_R_0	BY930532
E_EL_ovS0_11B08_F_0	BY922015
E_EL_ovS0_11B08_R_0	BY930533
E_EL_ovS0_11B09_F_0	BY922016
E_EL_ovS0_11B09_R_0	BY930534
E_EL_ovS0_11B10_F_0	BY922017
E_EL_ovS0_11B10_R_0	BY930535
E_EL_ovS0_11B11_R_0	BY930536
E_EL_ovS0_11B12_R_0	BY930537
E_EL_ovS0_11C01_F_0	BY922018
E_EL_ovS0_11C01_R_0	BY930538
E_EL_ovS0_11C02_F_0	BY922019
E_EL_ovS0_11C02_R_0	BY930539
E_EL_ovS0_11C03_F_0	BY922020
E_EL_ovS0_11C03_R_0	BY930540
E_EL_ovS0_11C04_R_0	BY930541
E_EL_ovS0_11C05_F_0	BY922021
E_EL_ovS0_11C05_R_0	BY930542
E_EL_ovS0_11C06_F_0	BY922022
E_EL_ovS0_11C06_R_0	BY930543
E_EL_ovS0_11C07_F_0	BY922023
E_EL_ovS0_11C07_R_0	BY930544

E_EL_ovS0_11C08_F_0	BY922024
E_EL_ovS0_11C08_R_0	BY930545
E_EL_ovS0_11C09_F_0	BY922025
E_EL_ovS0_11C09_R_0	BY930546
E_EL_ovS0_11C10_F_0	BY922026
E_EL_ovS0_11C10_R_0	BY930547
E_EL_ovS0_11C11_F_0	BY922027
E_EL_ovS0_11C11_R_0	BY930548
E_EL_ovS0_11C12_R_0	BY930549
E_EL_ovS0_11D01_F_0	BY922028
E_EL_ovS0_11D01_R_0	BY930550
E_EL_ovS0_11D02_F_0	BY922029
E_EL_ovS0_11D02_R_0	BY930551
E_EL_ovS0_11D04_F_0	BY922030
E_EL_ovS0_11D04_R_0	BY930552
E_EL_ovS0_11D05_F_0	BY922031
E_EL_ovS0_11D05_R_0	BY930553
E_EL_ovS0_11D06_F_0	BY922032
E_EL_ovS0_11D07_F_0	BY922033
E_EL_ovS0_11D07_R_0	BY930554
E_EL_ovS0_11D08_F_0	BY922034
E_EL_ovS0_11D08_R_0	BY930555
E_EL_ovS0_11D09_R_0	BY930556
E_EL_ovS0_11D10_F_0	BY922035
E_EL_ovS0_11D10_R_0	BY930557
E_EL_ovS0_11D11_F_0	BY922036
E_EL_ovS0_11D11_R_0	BY930558
E_EL_ovS0_11D12_F_0	BY922037
E_EL_ovS0_11D12_R_0	BY930559
E_EL_ovS0_11E01_F_0	BY922038
E_EL_ovS0_11E01_R_0	BY930560
E_EL_ovS0_11E02_F_0	BY922039
E_EL_ovS0_11E02_R_0	BY930561
E_EL_ovS0_11E04_F_0	BY922040
E_EL_ovS0_11E04_R_0	BY930562
E_EL_ovS0_11E05_F_0	BY922041
E_EL_ovS0_11E06_F_0	BY922042
E_EL_ovS0_11E06_R_0	BY930563
E_EL_ovS0_11E07_F_0	BY922043
E_EL_ovS0_11E08_F_0	BY922044
E_EL_ovS0_11E08_R_0	BY930564
E_EL_ovS0_11E09_R_0	BY930565
E_EL_ovS0_11E10_F_0	BY922045
E_EL_ovS0_11E10_R_0	BY930566
E_EL_ovS0_11E12_F_0	BY922046
E_EL_ovS0_11E12_R_0	BY930567
E_EL_ovS0_11F01_F_0	BY922047
E_EL_ovS0_11F01_R_0	BY930568
E_EL_ovS0_11F02_R_0	BY930569
E_EL_ovS0_11F03_F_0	BY922048
E_EL_ovS0_11F03_R_0	BY930570
E_EL_ovS0_11F04_F_0	BY922049
E_EL_ovS0_11F04_R_0	BY930571
E_EL_ovS0_11F05_F_0	BY922050

E_EL_ovS0_11F05_R_0	BY930572
E_EL_ovS0_11F06_F_0	BY922051
E_EL_ovS0_11F06_R_0	BY930573
E_EL_ovS0_11F07_F_0	BY922052
E_EL_ovS0_11F08_F_0	BY922053
E_EL_ovS0_11F08_R_0	BY930574
E_EL_ovS0_11F09_F_0	BY922054
E_EL_ovS0_11F09_R_0	BY930575
E_EL_ovS0_11F10_F_0	BY922055
E_EL_ovS0_11F10_R_0	BY930576
E_EL_ovS0_11F11_R_0	BY930577
E_EL_ovS0_11F12_R_0	BY930578
E_EL_ovS0_11G01_R_0	BY930579
E_EL_ovS0_11G02_F_0	BY922056
E_EL_ovS0_11G02_R_0	BY930580
E_EL_ovS0_11G03_F_0	BY922057
E_EL_ovS0_11G03_R_0	BY930581
E_EL_ovS0_11G05_F_0	BY922058
E_EL_ovS0_11G05_R_0	BY930582
E_EL_ovS0_11G06_F_0	BY922059
E_EL_ovS0_11G06_R_0	BY930583
E_EL_ovS0_11G07_F_0	BY922060
E_EL_ovS0_11G07_R_0	BY930584
E_EL_ovS0_11G08_F_0	BY922061
E_EL_ovS0_11G08_R_0	BY930585
E_EL_ovS0_11G10_F_0	BY922062
E_EL_ovS0_11G10_R_0	BY930586
E_EL_ovS0_11G11_F_0	BY922063
E_EL_ovS0_11G11_R_0	BY930587
E_EL_ovS0_11G12_F_0	BY922064
E_EL_ovS0_11G12_R_0	BY930588
E_EL_ovS0_11H01_F_0	BY922065
E_EL_ovS0_11H01_R_0	BY930589
E_EL_ovS0_11H03_F_0	BY922066
E_EL_ovS0_11H03_R_0	BY930590
E_EL_ovS0_11H04_F_0	BY922067
E_EL_ovS0_11H04_R_0	BY930591
E_EL_ovS0_11H05_R_0	BY930592
E_EL_ovS0_11H07_F_0	BY922068
E_EL_ovS0_11H07_R_0	BY930593
E_EL_ovS0_11H08_F_0	BY922069
E_EL_ovS0_11H08_R_0	BY930594
E_EL_ovS0_11H09_F_0	BY922070
E_EL_ovS0_11H09_R_0	BY930595
E_EL_ovS0_11H11_F_0	BY922071
E_EL_ovS0_11H11_R_0	BY930596
E_EL_ovS0_11H12_F_0	BY922072
E_EL_ovS0_12A01_F_0	BY922073
E_EL_ovS0_12A01_R_0	BY930597
E_EL_ovS0_12A02_R_0	BY930598
E_EL_ovS0_12A03_F_0	BY922074
E_EL_ovS0_12A03_R_0	BY930599
E_EL_ovS0_12A04_F_0	BY922075
E_EL_ovS0_12A04_R_0	BY930600

E_EL_ovS0_12A05_F_0	BY922076
E_EL_ovS0_12A05_R_0	BY930601
E_EL_ovS0_12A06_F_0	BY922077
E_EL_ovS0_12A06_R_0	BY930602
E_EL_ovS0_12A07_F_0	BY922078
E_EL_ovS0_12A08_F_0	BY922079
E_EL_ovS0_12A08_R_0	BY930603
E_EL_ovS0_12B01_F_0	BY922080
E_EL_ovS0_12B01_R_0	BY930604
E_EL_ovS0_12B02_F_0	BY922081
E_EL_ovS0_12B02_R_0	BY930605
E_EL_ovS0_12B03_F_0	BY922082
E_EL_ovS0_12B03_R_0	BY930606
E_EL_ovS0_12B04_F_0	BY922083
E_EL_ovS0_12B04_R_0	BY930607
E_EL_ovS0_12B06_F_0	BY922084
E_EL_ovS0_12B07_F_0	BY922085
E_EL_ovS0_12B07_R_0	BY930608
E_EL_ovS0_12B08_R_0	BY930609
E_EL_ovS0_12B09_F_0	BY922086
E_EL_ovS0_12B09_R_0	BY930610
E_EL_ovS0_12B10_F_0	BY922087
E_EL_ovS0_12B10_R_0	BY930611
E_EL_ovS0_12B12_F_0	BY922088
E_EL_ovS0_12B12_R_0	BY930612
E_EL_ovS0_12C01_F_0	BY922089
E_EL_ovS0_12C01_R_0	BY930613
E_EL_ovS0_12C02_F_0	BY922090
E_EL_ovS0_12C02_R_0	BY930614
E_EL_ovS0_12C03_F_0	BY922091
E_EL_ovS0_12C03_R_0	BY930615
E_EL_ovS0_12C04_R_0	BY930616
E_EL_ovS0_12C05_F_0	BY922092
E_EL_ovS0_12C05_R_0	BY930617
E_EL_ovS0_12C06_F_0	BY922093
E_EL_ovS0_12C06_R_0	BY930618
E_EL_ovS0_12C07_F_0	BY922094
E_EL_ovS0_12C07_R_0	BY930619
E_EL_ovS0_12C08_F_0	BY922095
E_EL_ovS0_12C08_R_0	BY930620
E_EL_ovS0_12C11_F_0	BY922096
E_EL_ovS0_12C11_R_0	BY930621
E_EL_ovS0_12C12_F_0	BY922097
E_EL_ovS0_12C12_R_0	BY930622
E_EL_ovS0_12D01_F_0	BY922098
E_EL_ovS0_12D01_R_0	BY930623
E_EL_ovS0_12D02_F_0	BY922099
E_EL_ovS0_12D02_R_0	BY930624
E_EL_ovS0_12D03_F_0	BY922100
E_EL_ovS0_12D03_R_0	BY930625
E_EL_ovS0_12D04_F_0	BY922101
E_EL_ovS0_12D04_R_0	BY930626
E_EL_ovS0_12D05_F_0	BY922102
E_EL_ovS0_12D05_R_0	BY930627

E_EL_ovS0_12D06_F_0	BY922103
E_EL_ovS0_12D06_R_0	BY930628
E_EL_ovS0_12D08_R_0	BY930629
E_EL_ovS0_12D09_F_0	BY922104
E_EL_ovS0_12D09_R_0	BY930630
E_EL_ovS0_12D10_F_0	BY922105
E_EL_ovS0_12D10_R_0	BY930631
E_EL_ovS0_12D12_F_0	BY922106
E_EL_ovS0_12D12_R_0	BY930632
E_EL_ovS0_12E01_F_0	BY922107
E_EL_ovS0_12E03_F_0	BY922108
E_EL_ovS0_12E03_R_0	BY930633
E_EL_ovS0_12E04_F_0	BY922109
E_EL_ovS0_12E05_F_0	BY922110
E_EL_ovS0_12E05_R_0	BY930634
E_EL_ovS0_12E06_F_0	BY922111
E_EL_ovS0_12E06_R_0	BY930635
E_EL_ovS0_12E07_F_0	BY922112
E_EL_ovS0_12E07_R_0	BY930636
E_EL_ovS0_12E12_F_0	BY922113
E_EL_ovS0_12E12_R_0	BY930637
E_EL_ovS0_12F01_F_0	BY922114
E_EL_ovS0_12F01_R_0	BY930638
E_EL_ovS0_12F02_F_0	BY922115
E_EL_ovS0_12F02_R_0	BY930639
E_EL_ovS0_12F03_F_0	BY922116
E_EL_ovS0_12F04_F_0	BY922117
E_EL_ovS0_12F04_R_0	BY930640
E_EL_ovS0_12F05_F_0	BY922118
E_EL_ovS0_12F07_R_0	BY930641
E_EL_ovS0_12F08_F_0	BY922119
E_EL_ovS0_12F09_F_0	BY922120
E_EL_ovS0_12F09_R_0	BY930642
E_EL_ovS0_12F10_F_0	BY922121
E_EL_ovS0_12F11_F_0	BY922122
E_EL_ovS0_12F11_R_0	BY930643
E_EL_ovS0_12F12_F_0	BY922123
E_EL_ovS0_12G01_F_0	BY922124
E_EL_ovS0_12G01_R_0	BY930644
E_EL_ovS0_12G02_F_0	BY922125
E_EL_ovS0_12G02_R_0	BY930645
E_EL_ovS0_12G03_F_0	BY922126
E_EL_ovS0_12G03_R_0	BY930646
E_EL_ovS0_12G05_R_0	BY930647
E_EL_ovS0_12G07_F_0	BY922127
E_EL_ovS0_12G07_R_0	BY930648
E_EL_ovS0_12G08_F_0	BY922128
E_EL_ovS0_12G08_R_0	BY930649
E_EL_ovS0_12H01_F_0	BY922129
E_EL_ovS0_12H01_R_0	BY930650
E_EL_ovS0_12H02_F_0	BY922130
E_EL_ovS0_12H02_R_0	BY930651
E_EL_ovS0_12H03_F_0	BY922131
E_EL_ovS0_12H04_F_0	BY922132

E_EL_ovS0_12H04_R_0	BY930652
E_EL_ovS0_12H06_F_0	BY922133
E_EL_ovS0_12H06_R_0	BY930653
E_EL_ovS0_12H07_F_0	BY922134
E_EL_ovS0_12H07_R_0	BY930654
E_EL_ovS0_12H08_F_0	BY922135
E_EL_ovS0_12H08_R_0	BY930655
E_EL_ovS0_12H09_F_0	BY922136
E_EL_ovS0_12H10_R_0	BY930656
E_EL_ovS0_12H11_F_0	BY922137
E_EL_ovS0_12H12_R_0	BY930657
E_EL_ovS0_13A02_F_0	BY922138
E_EL_ovS0_13A02_R_0	BY930658
E_EL_ovS0_13A03_F_0	BY922139
E_EL_ovS0_13A03_R_0	BY930659
E_EL_ovS0_13A04_F_0	BY922140
E_EL_ovS0_13A04_R_0	BY930660
E_EL_ovS0_13A05_F_0	BY922141
E_EL_ovS0_13A05_R_0	BY930661
E_EL_ovS0_13A07_F_0	BY922142
E_EL_ovS0_13A07_R_0	BY930662
E_EL_ovS0_13A08_F_0	BY922143
E_EL_ovS0_13A08_R_0	BY930663
E_EL_ovS0_13A09_F_0	BY922144
E_EL_ovS0_13A10_F_0	BY922145
E_EL_ovS0_13A11_R_0	BY930664
E_EL_ovS0_13A12_F_0	BY922146
E_EL_ovS0_13A12_R_0	BY930665
E_EL_ovS0_13B01_F_0	BY922147
E_EL_ovS0_13B01_R_0	BY930666
E_EL_ovS0_13B02_R_0	BY930667
E_EL_ovS0_13B03_F_0	BY922148
E_EL_ovS0_13B03_R_0	BY930668
E_EL_ovS0_13B04_F_0	BY922149
E_EL_ovS0_13B04_R_0	BY930669
E_EL_ovS0_13B05_F_0	BY922150
E_EL_ovS0_13B05_R_0	BY930670
E_EL_ovS0_13B06_F_0	BY922151
E_EL_ovS0_13B07_F_0	BY922152
E_EL_ovS0_13B07_R_0	BY930671
E_EL_ovS0_13B08_F_0	BY922153
E_EL_ovS0_13B08_R_0	BY930672
E_EL_ovS0_13B09_F_0	BY922154
E_EL_ovS0_13B09_R_0	BY930673
E_EL_ovS0_13B10_F_0	BY922155
E_EL_ovS0_13B10_R_0	BY930674
E_EL_ovS0_13B11_F_0	BY922156
E_EL_ovS0_13B11_R_0	BY930675
E_EL_ovS0_13B12_F_0	BY922157
E_EL_ovS0_13B12_R_0	BY930676
E_EL_ovS0_13C01_F_0	BY922158
E_EL_ovS0_13C01_R_0	BY930677
E_EL_ovS0_13C02_R_0	BY930678
E_EL_ovS0_13C03_F_0	BY922159

E_EL_ovS0_13C03_R_0	BY930679
E_EL_ovS0_13C04_F_0	BY922160
E_EL_ovS0_13C04_R_0	BY930680
E_EL_ovS0_13C05_F_0	BY922161
E_EL_ovS0_13C06_F_0	BY922162
E_EL_ovS0_13C06_R_0	BY930681
E_EL_ovS0_13C07_F_0	BY922163
E_EL_ovS0_13C09_F_0	BY922164
E_EL_ovS0_13C09_R_0	BY930682
E_EL_ovS0_13C10_F_0	BY922165
E_EL_ovS0_13C10_R_0	BY930683
E_EL_ovS0_13C11_F_0	BY922166
E_EL_ovS0_13C11_R_0	BY930684
E_EL_ovS0_13C12_F_0	BY922167
E_EL_ovS0_13D01_F_0	BY922168
E_EL_ovS0_13D01_R_0	BY930685
E_EL_ovS0_13D02_F_0	BY922169
E_EL_ovS0_13D02_R_0	BY930686
E_EL_ovS0_13D03_F_0	BY922170
E_EL_ovS0_13D03_R_0	BY930687
E_EL_ovS0_13D04_F_0	BY922171
E_EL_ovS0_13D04_R_0	BY930688
E_EL_ovS0_13D05_F_0	BY922172
E_EL_ovS0_13D06_R_0	BY930689
E_EL_ovS0_13D07_F_0	BY922173
E_EL_ovS0_13D07_R_0	BY930690
E_EL_ovS0_13D09_F_0	BY922174
E_EL_ovS0_13D09_R_0	BY930691
E_EL_ovS0_13D10_F_0	BY922175
E_EL_ovS0_13D10_R_0	BY930692
E_EL_ovS0_13D11_F_0	BY922176
E_EL_ovS0_13D11_R_0	BY930693
E_EL_ovS0_13D12_F_0	BY922177
E_EL_ovS0_13D12_R_0	BY930694
E_EL_ovS0_13E01_F_0	BY922178
E_EL_ovS0_13E01_R_0	BY930695
E_EL_ovS0_13E02_F_0	BY922179
E_EL_ovS0_13E02_R_0	BY930696
E_EL_ovS0_13E04_F_0	BY922180
E_EL_ovS0_13E04_R_0	BY930697
E_EL_ovS0_13E05_F_0	BY922181
E_EL_ovS0_13E05_R_0	BY930698
E_EL_ovS0_13E06_F_0	BY922182
E_EL_ovS0_13E06_R_0	BY930699
E_EL_ovS0_13E07_F_0	BY922183
E_EL_ovS0_13E07_R_0	BY930700
E_EL_ovS0_13E08_F_0	BY922184
E_EL_ovS0_13E08_R_0	BY930701
E_EL_ovS0_13E09_R_0	BY930702
E_EL_ovS0_13F01_F_0	BY922185
E_EL_ovS0_13F01_R_0	BY930703
E_EL_ovS0_13F02_F_0	BY922186
E_EL_ovS0_13F02_R_0	BY930704
E_EL_ovS0_13F03_F_0	BY922187

E_EL_ovS0_13F03_R_0	BY930705
E_EL_ovS0_13F04_F_0	BY922188
E_EL_ovS0_13F04_R_0	BY930706
E_EL_ovS0_13F05_F_0	BY922189
E_EL_ovS0_13F05_R_0	BY930707
E_EL_ovS0_13F06_F_0	BY922190
E_EL_ovS0_13F06_R_0	BY930708
E_EL_ovS0_13F07_F_0	BY922191
E_EL_ovS0_13F07_R_0	BY930709
E_EL_ovS0_13F08_R_0	BY930710
E_EL_ovS0_13F09_F_0	BY922192
E_EL_ovS0_13F09_R_0	BY930711
E_EL_ovS0_13F10_F_0	BY922193
E_EL_ovS0_13F10_R_0	BY930712
E_EL_ovS0_13F11_F_0	BY922194
E_EL_ovS0_13F11_R_0	BY930713
E_EL_ovS0_13F12_R_0	BY930714
E_EL_ovS0_13G01_R_0	BY930715
E_EL_ovS0_13G02_F_0	BY922195
E_EL_ovS0_13G02_R_0	BY930716
E_EL_ovS0_13G03_F_0	BY922196
E_EL_ovS0_13G03_R_0	BY930717
E_EL_ovS0_13G04_F_0	BY922197
E_EL_ovS0_13G04_R_0	BY930718
E_EL_ovS0_13G05_F_0	BY922198
E_EL_ovS0_13G05_R_0	BY930719
E_EL_ovS0_13G06_F_0	BY922199
E_EL_ovS0_13G06_R_0	BY930720
E_EL_ovS0_13G07_F_0	BY922200
E_EL_ovS0_13G07_R_0	BY930721
E_EL_ovS0_13G08_F_0	BY922201
E_EL_ovS0_13G08_R_0	BY930722
E_EL_ovS0_13G09_F_0	BY922202
E_EL_ovS0_13G09_R_0	BY930723
E_EL_ovS0_13G10_F_0	BY922203
E_EL_ovS0_13G10_R_0	BY930724
E_EL_ovS0_13G11_F_0	BY922204
E_EL_ovS0_13G12_F_0	BY922205
E_EL_ovS0_13G12_R_0	BY930725
E_EL_ovS0_13H01_F_0	BY922206
E_EL_ovS0_13H01_R_0	BY930726
E_EL_ovS0_13H02_F_0	BY922207
E_EL_ovS0_13H02_R_0	BY930727
E_EL_ovS0_13H03_F_0	BY922208
E_EL_ovS0_13H03_R_0	BY930728
E_EL_ovS0_13H04_F_0	BY922209
E_EL_ovS0_13H04_R_0	BY930729
E_EL_ovS0_13H05_F_0	BY922210
E_EL_ovS0_13H06_F_0	BY922211
E_EL_ovS0_13H06_R_0	BY930730
E_EL_ovS0_13H07_F_0	BY922212
E_EL_ovS0_13H08_F_0	BY922213
E_EL_ovS0_13H08_R_0	BY930731
E_EL_ovS0_13H09_F_0	BY922214

E_EL_ovS0_13H09_R_0	BY930732
E_EL_ovS0_13H11_F_0	BY922215
E_EL_ovS0_13H11_R_0	BY930733
E_EL_ovS0_13H12_F_0	BY922216
E_EL_ovS0_13H12_R_0	BY930734
E_EL_ovS0_14A01_F_0	BY922217
E_EL_ovS0_14A01_R_0	BY930735
E_EL_ovS0_14A03_F_0	BY922218
E_EL_ovS0_14A03_R_0	BY930736
E_EL_ovS0_14A04_F_0	BY922219
E_EL_ovS0_14A04_R_0	BY930737
E_EL_ovS0_14A05_F_0	BY922220
E_EL_ovS0_14A05_R_0	BY930738
E_EL_ovS0_14A06_F_0	BY922221
E_EL_ovS0_14A06_R_0	BY930739
E_EL_ovS0_14A07_R_0	BY930740
E_EL_ovS0_14A08_F_0	BY922222
E_EL_ovS0_14A08_R_0	BY930741
E_EL_ovS0_14A09_F_0	BY922223
E_EL_ovS0_14A09_R_0	BY930742
E_EL_ovS0_14A10_F_0	BY922224
E_EL_ovS0_14A10_R_0	BY930743
E_EL_ovS0_14A11_F_0	BY922225
E_EL_ovS0_14A11_R_0	BY930744
E_EL_ovS0_14B01_F_0	BY922226
E_EL_ovS0_14B01_R_0	BY930745
E_EL_ovS0_14B02_F_0	BY922227
E_EL_ovS0_14B02_R_0	BY930746
E_EL_ovS0_14B03_F_0	BY922228
E_EL_ovS0_14B03_R_0	BY930747
E_EL_ovS0_14B04_F_0	BY922229
E_EL_ovS0_14B04_R_0	BY930748
E_EL_ovS0_14B05_F_0	BY922230
E_EL_ovS0_14B05_R_0	BY930749
E_EL_ovS0_14B06_F_0	BY922231
E_EL_ovS0_14B06_R_0	BY930750
E_EL_ovS0_14B07_F_0	BY922232
E_EL_ovS0_14B08_F_0	BY922233
E_EL_ovS0_14B08_R_0	BY930751
E_EL_ovS0_14B09_F_0	BY922234
E_EL_ovS0_14B09_R_0	BY930752
E_EL_ovS0_14B10_F_0	BY922235
E_EL_ovS0_14B10_R_0	BY930753
E_EL_ovS0_14B11_F_0	BY922236
E_EL_ovS0_14B11_R_0	BY930754
E_EL_ovS0_14B12_F_0	BY922237
E_EL_ovS0_14B12_R_0	BY930755
E_EL_ovS0_14C01_F_0	BY922238
E_EL_ovS0_14C03_F_0	BY922239
E_EL_ovS0_14C03_R_0	BY930756
E_EL_ovS0_14C04_F_0	BY922240
E_EL_ovS0_14C05_F_0	BY922241
E_EL_ovS0_14C05_R_0	BY930757
E_EL_ovS0_14C06_F_0	BY922242

E_EL_ovS0_14C06_R_0	BY930758
E_EL_ovS0_14C07_F_0	BY922243
E_EL_ovS0_14C07_R_0	BY930759
E_EL_ovS0_14C08_R_0	BY930760
E_EL_ovS0_14C09_R_0	BY930761
E_EL_ovS0_14C10_F_0	BY922244
E_EL_ovS0_14C10_R_0	BY930762
E_EL_ovS0_14C11_F_0	BY922245
E_EL_ovS0_14C11_R_0	BY930763
E_EL_ovS0_14C12_F_0	BY922246
E_EL_ovS0_14C12_R_0	BY930764
E_EL_ovS0_14D01_F_0	BY922247
E_EL_ovS0_14D02_F_0	BY922248
E_EL_ovS0_14D02_R_0	BY930765
E_EL_ovS0_14D03_F_0	BY922249
E_EL_ovS0_14D03_R_0	BY930766
E_EL_ovS0_14D05_F_0	BY922250
E_EL_ovS0_14D05_R_0	BY930767
E_EL_ovS0_14D06_R_0	BY930768
E_EL_ovS0_14D08_F_0	BY922251
E_EL_ovS0_14D08_R_0	BY930769
E_EL_ovS0_14D09_F_0	BY922252
E_EL_ovS0_14D09_R_0	BY930770
E_EL_ovS0_14D11_F_0	BY922253
E_EL_ovS0_14D11_R_0	BY930771
E_EL_ovS0_14D12_F_0	BY922254
E_EL_ovS0_14D12_R_0	BY930772
E_EL_ovS0_14E01_F_0	BY922255
E_EL_ovS0_14E01_R_0	BY930773
E_EL_ovS0_14E02_R_0	BY930774
E_EL_ovS0_14E03_F_0	BY922256
E_EL_ovS0_14E03_R_0	BY930775
E_EL_ovS0_14E04_F_0	BY922257
E_EL_ovS0_14E04_R_0	BY930776
E_EL_ovS0_14E05_F_0	BY922258
E_EL_ovS0_14E05_R_0	BY930777
E_EL_ovS0_14E06_F_0	BY922259
E_EL_ovS0_14E06_R_0	BY930778
E_EL_ovS0_14E07_R_0	BY930779
E_EL_ovS0_14E08_F_0	BY922260
E_EL_ovS0_14E08_R_0	BY930780
E_EL_ovS0_14E09_F_0	BY922261
E_EL_ovS0_14E09_R_0	BY930781
E_EL_ovS0_14E12_R_0	BY930782
E_EL_ovS0_14F01_R_0	BY930783
E_EL_ovS0_14F02_F_0	BY922262
E_EL_ovS0_14F03_F_0	BY922263
E_EL_ovS0_14F03_R_0	BY930784
E_EL_ovS0_14F04_F_0	BY922264
E_EL_ovS0_14F04_R_0	BY930785
E_EL_ovS0_14F05_R_0	BY930786
E_EL_ovS0_14F06_F_0	BY922265
E_EL_ovS0_14F06_R_0	BY930787
E_EL_ovS0_14F07_F_0	BY922266

E_EL_ovS0_14F07_R_0	BY930788
E_EL_ovS0_14F08_F_0	BY922267
E_EL_ovS0_14F08_R_0	BY930789
E_EL_ovS0_14F09_F_0	BY922268
E_EL_ovS0_14F09_R_0	BY930790
E_EL_ovS0_14F10_F_0	BY922269
E_EL_ovS0_14F10_R_0	BY930791
E_EL_ovS0_14F11_R_0	BY930792
E_EL_ovS0_14F12_R_0	BY930793
E_EL_ovS0_14G02_R_0	BY930794
E_EL_ovS0_14G03_F_0	BY922270
E_EL_ovS0_14G03_R_0	BY930795
E_EL_ovS0_14G04_F_0	BY922271
E_EL_ovS0_14G04_R_0	BY930796
E_EL_ovS0_14G05_F_0	BY922272
E_EL_ovS0_14G05_R_0	BY930797
E_EL_ovS0_14G06_F_0	BY922273
E_EL_ovS0_14G06_R_0	BY930798
E_EL_ovS0_14G07_F_0	BY922274
E_EL_ovS0_14G07_R_0	BY930799
E_EL_ovS0_14G08_F_0	BY922275
E_EL_ovS0_14G08_R_0	BY930800
E_EL_ovS0_14G09_F_0	BY922276
E_EL_ovS0_14G09_R_0	BY930801
E_EL_ovS0_14G11_F_0	BY922277
E_EL_ovS0_14G11_R_0	BY930802
E_EL_ovS0_14G12_F_0	BY922278
E_EL_ovS0_14G12_R_0	BY930803
E_EL_ovS0_14H01_F_0	BY922279
E_EL_ovS0_14H01_R_0	BY930804
E_EL_ovS0_14H02_F_0	BY922280
E_EL_ovS0_14H02_R_0	BY930805
E_EL_ovS0_14H03_F_0	BY922281
E_EL_ovS0_14H03_R_0	BY930806
E_EL_ovS0_14H04_F_0	BY922282
E_EL_ovS0_14H04_R_0	BY930807
E_EL_ovS0_14H05_F_0	BY922283
E_EL_ovS0_14H05_R_0	BY930808
E_EL_ovS0_14H06_F_0	BY922284
E_EL_ovS0_14H07_F_0	BY922285
E_EL_ovS0_14H08_F_0	BY922286
E_EL_ovS0_14H08_R_0	BY930809
E_EL_ovS0_14H09_F_0	BY922287
E_EL_ovS0_14H09_R_0	BY930810
E_EL_ovS0_14H10_F_0	BY922288
E_EL_ovS0_14H10_R_0	BY930811
E_EL_ovS0_15A01_F_0	BY922289
E_EL_ovS0_15A02_R_0	BY930812
E_EL_ovS0_15A03_F_0	BY922290
E_EL_ovS0_15A03_R_0	BY930813
E_EL_ovS0_15A04_F_0	BY922291
E_EL_ovS0_15A04_R_0	BY930814
E_EL_ovS0_15A06_F_0	BY922292
E_EL_ovS0_15A06_R_0	BY930815

E_EL_ovS0_15A07_F_0	BY922293
E_EL_ovS0_15A08_F_0	BY922294
E_EL_ovS0_15A09_F_0	BY922295
E_EL_ovS0_15A12_F_0	BY922296
E_EL_ovS0_15B01_F_0	BY922297
E_EL_ovS0_15B01_R_0	BY930816
E_EL_ovS0_15B02_F_0	BY922298
E_EL_ovS0_15B02_R_0	BY930817
E_EL_ovS0_15B03_F_0	BY922299
E_EL_ovS0_15B03_R_0	BY930818
E_EL_ovS0_15B04_F_0	BY922300
E_EL_ovS0_15B05_F_0	BY922301
E_EL_ovS0_15B05_R_0	BY930819
E_EL_ovS0_15B06_F_0	BY922302
E_EL_ovS0_15B06_R_0	BY930820
E_EL_ovS0_15B07_F_0	BY922303
E_EL_ovS0_15B07_R_0	BY930821
E_EL_ovS0_15B08_F_0	BY922304
E_EL_ovS0_15B08_R_0	BY930822
E_EL_ovS0_15B09_F_0	BY922305
E_EL_ovS0_15B09_R_0	BY930823
E_EL_ovS0_15B10_F_0	BY922306
E_EL_ovS0_15B10_R_0	BY930824
E_EL_ovS0_15B11_F_0	BY922307
E_EL_ovS0_15B11_R_0	BY930825
E_EL_ovS0_15B12_F_0	BY922308
E_EL_ovS0_15B12_R_0	BY930826
E_EL_ovS0_15C01_F_0	BY922309
E_EL_ovS0_15C01_R_0	BY930827
E_EL_ovS0_15C03_F_0	BY922310
E_EL_ovS0_15C03_R_0	BY930828
E_EL_ovS0_15C04_F_0	BY922311
E_EL_ovS0_15C05_F_0	BY922312
E_EL_ovS0_15C05_R_0	BY930829
E_EL_ovS0_15C06_F_0	BY922313
E_EL_ovS0_15C06_R_0	BY930830
E_EL_ovS0_15C07_F_0	BY922314
E_EL_ovS0_15C07_R_0	BY930831
E_EL_ovS0_15C08_F_0	BY922315
E_EL_ovS0_15C08_R_0	BY930832
E_EL_ovS0_15C09_F_0	BY922316
E_EL_ovS0_15C09_R_0	BY930833
E_EL_ovS0_15C10_R_0	BY930834
E_EL_ovS0_15C11_R_0	BY930835
E_EL_ovS0_15C12_F_0	BY922317
E_EL_ovS0_15D01_F_0	BY922318
E_EL_ovS0_15D01_R_0	BY930836
E_EL_ovS0_15D02_F_0	BY922319
E_EL_ovS0_15D03_F_0	BY922320
E_EL_ovS0_15D03_R_0	BY930837
E_EL_ovS0_15D04_F_0	BY922321
E_EL_ovS0_15D04_R_0	BY930838
E_EL_ovS0_15D05_F_0	BY922322
E_EL_ovS0_15D05_R_0	BY930839

E_EL_ovS0_15D06_F_0	BY922323
E_EL_ovS0_15D06_R_0	BY930840
E_EL_ovS0_15D07_F_0	BY922324
E_EL_ovS0_15D07_R_0	BY930841
E_EL_ovS0_15D08_F_0	BY922325
E_EL_ovS0_15D08_R_0	BY930842
E_EL_ovS0_15D10_F_0	BY922326
E_EL_ovS0_15D10_R_0	BY930843
E_EL_ovS0_15D11_F_0	BY922327
E_EL_ovS0_15D11_R_0	BY930844
E_EL_ovS0_15D12_F_0	BY922328
E_EL_ovS0_15E01_F_0	BY922329
E_EL_ovS0_15E02_F_0	BY922330
E_EL_ovS0_15E02_R_0	BY930845
E_EL_ovS0_15E03_F_0	BY922331
E_EL_ovS0_15E03_R_0	BY930846
E_EL_ovS0_15E04_F_0	BY922332
E_EL_ovS0_15E05_F_0	BY922333
E_EL_ovS0_15E06_F_0	BY922334
E_EL_ovS0_15E07_F_0	BY922335
E_EL_ovS0_15E08_F_0	BY922336
E_EL_ovS0_15E08_R_0	BY930847
E_EL_ovS0_15E09_F_0	BY922337
E_EL_ovS0_15E12_F_0	BY922338
E_EL_ovS0_15F01_F_0	BY922339
E_EL_ovS0_15F01_R_0	BY930848
E_EL_ovS0_15F02_F_0	BY922340
E_EL_ovS0_15F02_R_0	BY930849
E_EL_ovS0_15F03_F_0	BY922341
E_EL_ovS0_15F03_R_0	BY930850
E_EL_ovS0_15F04_F_0	BY922342
E_EL_ovS0_15F04_R_0	BY930851
E_EL_ovS0_15F05_F_0	BY922343
E_EL_ovS0_15F05_R_0	BY930852
E_EL_ovS0_15F07_F_0	BY922344
E_EL_ovS0_15F07_R_0	BY930853
E_EL_ovS0_15F08_F_0	BY922345
E_EL_ovS0_15F08_R_0	BY930854
E_EL_ovS0_15F09_F_0	BY922346
E_EL_ovS0_15F09_R_0	BY930855
E_EL_ovS0_15F10_F_0	BY922347
E_EL_ovS0_15F10_R_0	BY930856
E_EL_ovS0_15F11_R_0	BY930857
E_EL_ovS0_15F12_F_0	BY922348
E_EL_ovS0_15F12_R_0	BY930858
E_EL_ovS0_15G01_R_0	BY930859
E_EL_ovS0_15G02_F_0	BY922349
E_EL_ovS0_15G02_R_0	BY930860
E_EL_ovS0_15G03_F_0	BY922350
E_EL_ovS0_15G03_R_0	BY930861
E_EL_ovS0_15G04_F_0	BY922351
E_EL_ovS0_15G04_R_0	BY930862
E_EL_ovS0_15G05_F_0	BY922352
E_EL_ovS0_15G05_R_0	BY930863

E_EL_ovS0_15G06_F_0	BY922353
E_EL_ovS0_15G06_R_0	BY930864
E_EL_ovS0_15G07_F_0	BY922354
E_EL_ovS0_15G07_R_0	BY930865
E_EL_ovS0_15G08_F_0	BY922355
E_EL_ovS0_15G08_R_0	BY930866
E_EL_ovS0_15G09_F_0	BY922356
E_EL_ovS0_15G09_R_0	BY930867
E_EL_ovS0_15G12_F_0	BY922357
E_EL_ovS0_15G12_R_0	BY930868
E_EL_ovS0_15H01_F_0	BY922358
E_EL_ovS0_15H01_R_0	BY930869
E_EL_ovS0_15H02_F_0	BY922359
E_EL_ovS0_15H02_R_0	BY930870
E_EL_ovS0_15H03_F_0	BY922360
E_EL_ovS0_15H04_F_0	BY922361
E_EL_ovS0_15H05_F_0	BY922362
E_EL_ovS0_15H06_F_0	BY922363
E_EL_ovS0_15H06_R_0	BY930871
E_EL_ovS0_15H07_F_0	BY922364
E_EL_ovS0_15H07_R_0	BY930872
E_EL_ovS0_15H08_F_0	BY922365
E_EL_ovS0_15H08_R_0	BY930873
E_EL_ovS0_15H09_F_0	BY922366
E_EL_ovS0_15H10_F_0	BY922367
E_EL_ovS0_15H10_R_0	BY930874
E_EL_ovS0_15H11_F_0	BY922368
E_EL_ovS0_15H11_R_0	BY930875
E_EL_ovS0_15H12_F_0	BY922369
E_EL_ovS0_15H12_R_0	BY930876
E_EL_ovS0_16A01_F_0	BY922370
E_EL_ovS0_16A01_R_0	BY930877
E_EL_ovS0_16A02_F_0	BY922371
E_EL_ovS0_16A02_R_0	BY930878
E_EL_ovS0_16A03_F_0	BY922372
E_EL_ovS0_16A03_R_0	BY930879
E_EL_ovS0_16A04_F_0	BY922373
E_EL_ovS0_16A04_R_0	BY930880
E_EL_ovS0_16A05_F_0	BY922374
E_EL_ovS0_16A06_F_0	BY922375
E_EL_ovS0_16A07_F_0	BY922376
E_EL_ovS0_16A07_R_0	BY930881
E_EL_ovS0_16A08_F_0	BY922377
E_EL_ovS0_16A08_R_0	BY930882
E_EL_ovS0_16A10_F_0	BY922378
E_EL_ovS0_16A11_F_0	BY922379
E_EL_ovS0_16A12_F_0	BY922380
E_EL_ovS0_16A12_R_0	BY930883
E_EL_ovS0_16B01_R_0	BY930884
E_EL_ovS0_16B02_F_0	BY922381
E_EL_ovS0_16B03_F_0	BY922382
E_EL_ovS0_16B04_F_0	BY922383
E_EL_ovS0_16B04_R_0	BY930885
E_EL_ovS0_16B05_F_0	BY922384

E_EL_ovS0_16B05_R_0	BY930886
E_EL_ovS0_16B06_F_0	BY922385
E_EL_ovS0_16B06_R_0	BY930887
E_EL_ovS0_16B07_F_0	BY922386
E_EL_ovS0_16B08_F_0	BY922387
E_EL_ovS0_16B09_F_0	BY922388
E_EL_ovS0_16B09_R_0	BY930888
E_EL_ovS0_16B10_F_0	BY922389
E_EL_ovS0_16B10_R_0	BY930889
E_EL_ovS0_16B11_F_0	BY922390
E_EL_ovS0_16B12_F_0	BY922391
E_EL_ovS0_16B12_R_0	BY930890
E_EL_ovS0_16C01_F_0	BY922392
E_EL_ovS0_16C01_R_0	BY930891
E_EL_ovS0_16C02_F_0	BY922393
E_EL_ovS0_16C02_R_0	BY930892
E_EL_ovS0_16C03_F_0	BY922394
E_EL_ovS0_16C03_R_0	BY930893
E_EL_ovS0_16C04_F_0	BY922395
E_EL_ovS0_16C06_F_0	BY922396
E_EL_ovS0_16C07_F_0	BY922397
E_EL_ovS0_16C07_R_0	BY930894
E_EL_ovS0_16C08_F_0	BY922398
E_EL_ovS0_16C08_R_0	BY930895
E_EL_ovS0_16C09_F_0	BY922399
E_EL_ovS0_16C09_R_0	BY930896
E_EL_ovS0_16C10_F_0	BY922400
E_EL_ovS0_16C10_R_0	BY930897
E_EL_ovS0_16C11_R_0	BY930898
E_EL_ovS0_16C12_F_0	BY922401
E_EL_ovS0_16C12_R_0	BY930899
E_EL_ovS0_16D01_F_0	BY922402
E_EL_ovS0_16D01_R_0	BY930900
E_EL_ovS0_16D03_R_0	BY930901
E_EL_ovS0_16D05_F_0	BY922403
E_EL_ovS0_16D05_R_0	BY930902
E_EL_ovS0_16D06_F_0	BY922404
E_EL_ovS0_16D06_R_0	BY930903
E_EL_ovS0_16D07_F_0	BY922405
E_EL_ovS0_16D07_R_0	BY930904
E_EL_ovS0_16D09_F_0	BY922406
E_EL_ovS0_16D09_R_0	BY930905
E_EL_ovS0_16D10_F_0	BY922407
E_EL_ovS0_16D10_R_0	BY930906
E_EL_ovS0_16D11_F_0	BY922408
E_EL_ovS0_16D11_R_0	BY930907
E_EL_ovS0_16D12_F_0	BY922409
E_EL_ovS0_16D12_R_0	BY930908
E_EL_ovS0_16E01_R_0	BY930909
E_EL_ovS0_16E02_R_0	BY930910
E_EL_ovS0_16E03_F_0	BY922410
E_EL_ovS0_16E03_R_0	BY930911
E_EL_ovS0_16E04_R_0	BY930912
E_EL_ovS0_16E06_F_0	BY922411

E_EL_ovS0_16E06_R_0	BY930913
E_EL_ovS0_16E07_F_0	BY922412
E_EL_ovS0_16E07_R_0	BY930914
E_EL_ovS0_16E08_F_0	BY922413
E_EL_ovS0_16E09_F_0	BY922414
E_EL_ovS0_16E09_R_0	BY930915
E_EL_ovS0_16E10_F_0	BY922415
E_EL_ovS0_16E10_R_0	BY930916
E_EL_ovS0_16E11_F_0	BY922416
E_EL_ovS0_16E12_F_0	BY922417
E_EL_ovS0_16E12_R_0	BY930917
E_EL_ovS0_16F01_F_0	BY922418
E_EL_ovS0_16F01_R_0	BY930918
E_EL_ovS0_16F02_R_0	BY930919
E_EL_ovS0_16F03_F_0	BY922419
E_EL_ovS0_16F04_F_0	BY922420
E_EL_ovS0_16F04_R_0	BY930920
E_EL_ovS0_16F05_F_0	BY922421
E_EL_ovS0_16F05_R_0	BY930921
E_EL_ovS0_16F06_R_0	BY930922
E_EL_ovS0_16F07_F_0	BY922422
E_EL_ovS0_16F09_F_0	BY922423
E_EL_ovS0_16F09_R_0	BY930923
E_EL_ovS0_16F10_F_0	BY922424
E_EL_ovS0_16F11_F_0	BY922425
E_EL_ovS0_16F11_R_0	BY930924
E_EL_ovS0_16F12_F_0	BY922426
E_EL_ovS0_16F12_R_0	BY930925
E_EL_ovS0_16G01_F_0	BY922427
E_EL_ovS0_16G01_R_0	BY930926
E_EL_ovS0_16G03_F_0	BY922428
E_EL_ovS0_16G03_R_0	BY930927
E_EL_ovS0_16G04_R_0	BY930928
E_EL_ovS0_16G05_F_0	BY922429
E_EL_ovS0_16G05_R_0	BY930929
E_EL_ovS0_16G06_F_0	BY922430
E_EL_ovS0_16G06_R_0	BY930930
E_EL_ovS0_16G07_F_0	BY922431
E_EL_ovS0_16G07_R_0	BY930931
E_EL_ovS0_16G08_F_0	BY922432
E_EL_ovS0_16G08_R_0	BY930932
E_EL_ovS0_16G10_F_0	BY922433
E_EL_ovS0_16G10_R_0	BY930933
E_EL_ovS0_16G12_F_0	BY922434
E_EL_ovS0_16G12_R_0	BY930934
E_EL_ovS0_16H01_F_0	BY922435
E_EL_ovS0_16H01_R_0	BY930935
E_EL_ovS0_16H02_F_0	BY922436
E_EL_ovS0_16H02_R_0	BY930936
E_EL_ovS0_16H03_F_0	BY922437
E_EL_ovS0_16H03_R_0	BY930937
E_EL_ovS0_16H04_F_0	BY922438
E_EL_ovS0_16H05_F_0	BY922439
E_EL_ovS0_16H05_R_0	BY930938

E_EL_ovS0_16H06_F_0	BY922440
E_EL_ovS0_16H06_R_0	BY930939
E_EL_ovS0_16H07_F_0	BY922441
E_EL_ovS0_16H07_R_0	BY930940
E_EL_ovS0_16H08_F_0	BY922442
E_EL_ovS0_16H08_R_0	BY930941
E_EL_ovS0_16H09_F_0	BY922443
E_EL_ovS0_16H09_R_0	BY930942
E_EL_ovS0_16H10_F_0	BY922444
E_EL_ovS0_16H10_R_0	BY930943
E_EL_ovS0_16H11_F_0	BY922445
E_EL_ovS0_16H11_R_0	BY930944
E_EL_ovS0_17A01_F_0	BY922446
E_EL_ovS0_17A02_R_0	BY930945
E_EL_ovS0_17A03_F_0	BY922447
E_EL_ovS0_17A03_R_0	BY930946
E_EL_ovS0_17A04_F_0	BY922448
E_EL_ovS0_17A04_R_0	BY930947
E_EL_ovS0_17A05_F_0	BY922449
E_EL_ovS0_17A05_R_0	BY930948
E_EL_ovS0_17A06_F_0	BY922450
E_EL_ovS0_17A06_R_0	BY930949
E_EL_ovS0_17A08_F_0	BY922451
E_EL_ovS0_17A08_R_0	BY930950
E_EL_ovS0_17A09_F_0	BY922452
E_EL_ovS0_17A09_R_0	BY930951
E_EL_ovS0_17A10_F_0	BY922453
E_EL_ovS0_17A10_R_0	BY930952
E_EL_ovS0_17A11_F_0	BY922454
E_EL_ovS0_17A12_R_0	BY930953
E_EL_ovS0_17B01_R_0	BY930954
E_EL_ovS0_17B02_F_0	BY922455
E_EL_ovS0_17B02_R_0	BY930955
E_EL_ovS0_17B03_F_0	BY922456
E_EL_ovS0_17B03_R_0	BY930956
E_EL_ovS0_17B04_F_0	BY922457
E_EL_ovS0_17B04_R_0	BY930957
E_EL_ovS0_17B06_R_0	BY930958
E_EL_ovS0_17B07_F_0	BY922458
E_EL_ovS0_17B07_R_0	BY930959
E_EL_ovS0_17B08_F_0	BY922459
E_EL_ovS0_17B08_R_0	BY930960
E_EL_ovS0_17B09_F_0	BY922460
E_EL_ovS0_17B09_R_0	BY930961
E_EL_ovS0_17B11_F_0	BY922461
E_EL_ovS0_17B11_R_0	BY930962
E_EL_ovS0_17C01_F_0	BY922462
E_EL_ovS0_17C01_R_0	BY930963
E_EL_ovS0_17C02_F_0	BY922463
E_EL_ovS0_17C02_R_0	BY930964
E_EL_ovS0_17C03_R_0	BY930965
E_EL_ovS0_17C04_F_0	BY922464
E_EL_ovS0_17C04_R_0	BY930966
E_EL_ovS0_17C05_F_0	BY922465

E_EL_ovS0_17C05_R_0	BY930967
E_EL_ovS0_17C06_R_0	BY930968
E_EL_ovS0_17C07_F_0	BY922466
E_EL_ovS0_17C07_R_0	BY930969
E_EL_ovS0_17C08_F_0	BY922467
E_EL_ovS0_17C08_R_0	BY930970
E_EL_ovS0_17C09_F_0	BY922468
E_EL_ovS0_17C09_R_0	BY930971
E_EL_ovS0_17C10_F_0	BY922469
E_EL_ovS0_17C10_R_0	BY930972
E_EL_ovS0_17C11_F_0	BY922470
E_EL_ovS0_17C11_R_0	BY930973
E_EL_ovS0_17C12_F_0	BY922471
E_EL_ovS0_17C12_R_0	BY930974
E_EL_ovS0_17D01_F_0	BY922472
E_EL_ovS0_17D01_R_0	BY930975
E_EL_ovS0_17D02_F_0	BY922473
E_EL_ovS0_17D02_R_0	BY930976
E_EL_ovS0_17D03_F_0	BY922474
E_EL_ovS0_17D03_R_0	BY930977
E_EL_ovS0_17D04_F_0	BY922475
E_EL_ovS0_17D04_R_0	BY930978
E_EL_ovS0_17D05_R_0	BY930979
E_EL_ovS0_17D06_F_0	BY922476
E_EL_ovS0_17D06_R_0	BY930980
E_EL_ovS0_17D07_F_0	BY922477
E_EL_ovS0_17D07_R_0	BY930981
E_EL_ovS0_17D08_R_0	BY930982
E_EL_ovS0_17D10_F_0	BY922478
E_EL_ovS0_17D10_R_0	BY930983
E_EL_ovS0_17D11_F_0	BY922479
E_EL_ovS0_17D11_R_0	BY930984
E_EL_ovS0_17D12_F_0	BY922480
E_EL_ovS0_17D12_R_0	BY930985
E_EL_ovS0_17E01_F_0	BY922481
E_EL_ovS0_17E01_R_0	BY930986
E_EL_ovS0_17E02_F_0	BY922482
E_EL_ovS0_17E02_R_0	BY930987
E_EL_ovS0_17E03_F_0	BY922483
E_EL_ovS0_17E03_R_0	BY930988
E_EL_ovS0_17E04_F_0	BY922484
E_EL_ovS0_17E04_R_0	BY930989
E_EL_ovS0_17E06_F_0	BY922485
E_EL_ovS0_17E06_R_0	BY930990
E_EL_ovS0_17E07_F_0	BY922486
E_EL_ovS0_17E07_R_0	BY930991
E_EL_ovS0_17E08_F_0	BY922487
E_EL_ovS0_17E08_R_0	BY930992
E_EL_ovS0_17E09_F_0	BY922488
E_EL_ovS0_17E09_R_0	BY930993
E_EL_ovS0_17E10_R_0	BY930994
E_EL_ovS0_17E11_F_0	BY922489
E_EL_ovS0_17E11_R_0	BY930995
E_EL_ovS0_17E12_F_0	BY922490

E_EL_ovS0_17E12_R_0	BY930996
E_EL_ovS0_17F01_R_0	BY930997
E_EL_ovS0_17F02_F_0	BY922491
E_EL_ovS0_17F02_R_0	BY930998
E_EL_ovS0_17F03_F_0	BY922492
E_EL_ovS0_17F04_F_0	BY922493
E_EL_ovS0_17F04_R_0	BY930999
E_EL_ovS0_17F05_F_0	BY922494
E_EL_ovS0_17F05_R_0	BY931000
E_EL_ovS0_17F06_F_0	BY922495
E_EL_ovS0_17F06_R_0	BY931001
E_EL_ovS0_17F07_F_0	BY922496
E_EL_ovS0_17F07_R_0	BY931002
E_EL_ovS0_17F09_F_0	BY922497
E_EL_ovS0_17F09_R_0	BY931003
E_EL_ovS0_17F10_F_0	BY922498
E_EL_ovS0_17F11_F_0	BY922499
E_EL_ovS0_17F11_R_0	BY931004
E_EL_ovS0_17F12_F_0	BY922500
E_EL_ovS0_17F12_R_0	BY931005
E_EL_ovS0_17G01_F_0	BY922501
E_EL_ovS0_17G01_R_0	BY931006
E_EL_ovS0_17G02_F_0	BY922502
E_EL_ovS0_17G02_R_0	BY931007
E_EL_ovS0_17G03_F_0	BY922503
E_EL_ovS0_17G03_R_0	BY931008
E_EL_ovS0_17G05_F_0	BY922504
E_EL_ovS0_17G06_F_0	BY922505
E_EL_ovS0_17G06_R_0	BY931009
E_EL_ovS0_17G07_F_0	BY922506
E_EL_ovS0_17G07_R_0	BY931010
E_EL_ovS0_17G08_F_0	BY922507
E_EL_ovS0_17G08_R_0	BY931011
E_EL_ovS0_17G10_F_0	BY922508
E_EL_ovS0_17G10_R_0	BY931012
E_EL_ovS0_17G11_F_0	BY922509
E_EL_ovS0_17G11_R_0	BY931013
E_EL_ovS0_17G12_F_0	BY922510
E_EL_ovS0_17G12_R_0	BY931014
E_EL_ovS0_17H01_F_0	BY922511
E_EL_ovS0_17H01_R_0	BY931015
E_EL_ovS0_17H02_F_0	BY922512
E_EL_ovS0_17H02_R_0	BY931016
E_EL_ovS0_17H04_F_0	BY922513
E_EL_ovS0_17H05_R_0	BY931017
E_EL_ovS0_17H06_R_0	BY931018
E_EL_ovS0_17H07_F_0	BY922514
E_EL_ovS0_17H07_R_0	BY931019
E_EL_ovS0_17H08_F_0	BY922515
E_EL_ovS0_17H08_R_0	BY931020
E_EL_ovS0_17H09_F_0	BY922516
E_EL_ovS0_17H09_R_0	BY931021
E_EL_ovS0_17H10_F_0	BY922517
E_EL_ovS0_17H10_R_0	BY931022

E_EL_ovS0_17H11_F_0	BY922518
E_EL_ovS0_17H11_R_0	BY931023
E_EL_ovS0_17H12_F_0	BY922519
E_EL_ovS0_17H12_R_0	BY931024
E_EL_ovS0_18A03_F_0	BY922520
E_EL_ovS0_18A03_R_0	BY931025
E_EL_ovS0_18A04_F_0	BY922521
E_EL_ovS0_18A04_R_0	BY931026
E_EL_ovS0_18A05_F_0	BY922522
E_EL_ovS0_18A05_R_0	BY931027
E_EL_ovS0_18A06_F_0	BY922523
E_EL_ovS0_18A06_R_0	BY931028
E_EL_ovS0_18A07_F_0	BY922524
E_EL_ovS0_18A07_R_0	BY931029
E_EL_ovS0_18A08_F_0	BY922525
E_EL_ovS0_18A08_R_0	BY931030
E_EL_ovS0_18A09_F_0	BY922526
E_EL_ovS0_18A09_R_0	BY931031
E_EL_ovS0_18A10_F_0	BY922527
E_EL_ovS0_18A10_R_0	BY931032
E_EL_ovS0_18A11_F_0	BY922528
E_EL_ovS0_18A11_R_0	BY931033
E_EL_ovS0_18A12_F_0	BY922529
E_EL_ovS0_18A12_R_0	BY931034
E_EL_ovS0_18B01_F_0	BY922530
E_EL_ovS0_18B01_R_0	BY931035
E_EL_ovS0_18B02_R_0	BY931036
E_EL_ovS0_18B03_F_0	BY922531
E_EL_ovS0_18B03_R_0	BY931037
E_EL_ovS0_18B04_F_0	BY922532
E_EL_ovS0_18B04_R_0	BY931038
E_EL_ovS0_18B05_F_0	BY922533
E_EL_ovS0_18B05_R_0	BY931039
E_EL_ovS0_18B06_F_0	BY922534
E_EL_ovS0_18B06_R_0	BY931040
E_EL_ovS0_18B08_F_0	BY922535
E_EL_ovS0_18B08_R_0	BY931041
E_EL_ovS0_18B09_F_0	BY922536
E_EL_ovS0_18B09_R_0	BY931042
E_EL_ovS0_18B10_F_0	BY922537
E_EL_ovS0_18B10_R_0	BY931043
E_EL_ovS0_18B11_F_0	BY922538
E_EL_ovS0_18B11_R_0	BY931044
E_EL_ovS0_18C01_F_0	BY922539
E_EL_ovS0_18C01_R_0	BY931045
E_EL_ovS0_18C02_R_0	BY931046
E_EL_ovS0_18C03_F_0	BY922540
E_EL_ovS0_18C03_R_0	BY931047
E_EL_ovS0_18C04_R_0	BY931048
E_EL_ovS0_18C05_R_0	BY931049
E_EL_ovS0_18C06_F_0	BY922541
E_EL_ovS0_18C06_R_0	BY931050
E_EL_ovS0_18C07_F_0	BY922542
E_EL_ovS0_18C07_R_0	BY931051

E_EL_ovS0_18C08_F_0	BY922543
E_EL_ovS0_18C08_R_0	BY931052
E_EL_ovS0_18C09_F_0	BY922544
E_EL_ovS0_18C09_R_0	BY931053
E_EL_ovS0_18C10_F_0	BY922545
E_EL_ovS0_18C10_R_0	BY931054
E_EL_ovS0_18C12_F_0	BY922546
E_EL_ovS0_18D01_F_0	BY922547
E_EL_ovS0_18D02_F_0	BY922548
E_EL_ovS0_18D02_R_0	BY931055
E_EL_ovS0_18D03_F_0	BY922549
E_EL_ovS0_18D03_R_0	BY931056
E_EL_ovS0_18D04_F_0	BY922550
E_EL_ovS0_18D04_R_0	BY931057
E_EL_ovS0_18D05_R_0	BY931058
E_EL_ovS0_18D06_F_0	BY922551
E_EL_ovS0_18D06_R_0	BY931059
E_EL_ovS0_18D07_F_0	BY922552
E_EL_ovS0_18D07_R_0	BY931060
E_EL_ovS0_18D08_F_0	BY922553
E_EL_ovS0_18D08_R_0	BY931061
E_EL_ovS0_18D09_F_0	BY922554
E_EL_ovS0_18D09_R_0	BY931062
E_EL_ovS0_18D10_F_0	BY922555
E_EL_ovS0_18D10_R_0	BY931063
E_EL_ovS0_18D11_F_0	BY922556
E_EL_ovS0_18D11_R_0	BY931064
E_EL_ovS0_18D12_F_0	BY922557
E_EL_ovS0_18D12_R_0	BY931065
E_EL_ovS0_18E01_F_0	BY922558
E_EL_ovS0_18E02_R_0	BY931066
E_EL_ovS0_18E03_F_0	BY922559
E_EL_ovS0_18E03_R_0	BY931067
E_EL_ovS0_18E04_R_0	BY931068
E_EL_ovS0_18E05_R_0	BY931069
E_EL_ovS0_18E07_F_0	BY922560
E_EL_ovS0_18E07_R_0	BY931070
E_EL_ovS0_18E08_F_0	BY922561
E_EL_ovS0_18E08_R_0	BY931071
E_EL_ovS0_18E10_F_0	BY922562
E_EL_ovS0_18E10_R_0	BY931072
E_EL_ovS0_18E11_F_0	BY922563
E_EL_ovS0_18E11_R_0	BY931073
E_EL_ovS0_18E12_F_0	BY922564
E_EL_ovS0_18E12_R_0	BY931074
E_EL_ovS0_18F01_F_0	BY922565
E_EL_ovS0_18F01_R_0	BY931075
E_EL_ovS0_18F02_F_0	BY922566
E_EL_ovS0_18F03_F_0	BY922567
E_EL_ovS0_18F03_R_0	BY931076
E_EL_ovS0_18F06_R_0	BY931077
E_EL_ovS0_18F07_F_0	BY922568
E_EL_ovS0_18F08_F_0	BY922569
E_EL_ovS0_18F08_R_0	BY931078

E_EL_ovS0_18F09_F_0	BY922570
E_EL_ovS0_18F09_R_0	BY931079
E_EL_ovS0_18F10_F_0	BY922571
E_EL_ovS0_18F10_R_0	BY931080
E_EL_ovS0_18F11_F_0	BY922572
E_EL_ovS0_18F11_R_0	BY931081
E_EL_ovS0_18F12_F_0	BY922573
E_EL_ovS0_18F12_R_0	BY931082
E_EL_ovS0_18G01_F_0	BY922574
E_EL_ovS0_18G01_R_0	BY931083
E_EL_ovS0_18G02_F_0	BY922575
E_EL_ovS0_18G02_R_0	BY931084
E_EL_ovS0_18G03_F_0	BY922576
E_EL_ovS0_18G03_R_0	BY931085
E_EL_ovS0_18G04_F_0	BY922577
E_EL_ovS0_18G04_R_0	BY931086
E_EL_ovS0_18G05_F_0	BY922578
E_EL_ovS0_18G05_R_0	BY931087
E_EL_ovS0_18G06_F_0	BY922579
E_EL_ovS0_18G06_R_0	BY931088
E_EL_ovS0_18G07_R_0	BY931089
E_EL_ovS0_18G08_F_0	BY922580
E_EL_ovS0_18G08_R_0	BY931090
E_EL_ovS0_18G09_F_0	BY922581
E_EL_ovS0_18G09_R_0	BY931091
E_EL_ovS0_18G10_F_0	BY922582
E_EL_ovS0_18G10_R_0	BY931092
E_EL_ovS0_18H01_F_0	BY922583
E_EL_ovS0_18H01_R_0	BY931093
E_EL_ovS0_18H03_R_0	BY931094
E_EL_ovS0_18H04_F_0	BY922584
E_EL_ovS0_18H04_R_0	BY931095
E_EL_ovS0_18H07_F_0	BY922585
E_EL_ovS0_18H07_R_0	BY931096
E_EL_ovS0_18H09_F_0	BY922586
E_EL_ovS0_18H09_R_0	BY931097
E_EL_ovS0_18H10_F_0	BY922587
E_EL_ovS0_18H10_R_0	BY931098
E_EL_ovS0_18H11_F_0	BY922588
E_EL_ovS0_18H11_R_0	BY931099
E_EL_ovS0_18H12_F_0	BY922589
E_EL_ovS0_18H12_R_0	BY931100
E_EL_ovS0_19A02_F_0	BY922590
E_EL_ovS0_19A02_R_0	BY931101
E_EL_ovS0_19A04_F_0	BY922591
E_EL_ovS0_19A04_R_0	BY931102
E_EL_ovS0_19A05_R_0	BY931103
E_EL_ovS0_19A06_F_0	BY922592
E_EL_ovS0_19A06_R_0	BY931104
E_EL_ovS0_19A07_F_0	BY922593
E_EL_ovS0_19A08_F_0	BY922594
E_EL_ovS0_19A08_R_0	BY931105
E_EL_ovS0_19A09_R_0	BY931106
E_EL_ovS0_19A10_F_0	BY922595

E_EL_ovS0_19A10_R_0	BY931107
E_EL_ovS0_19A11_R_0	BY931108
E_EL_ovS0_19A12_F_0	BY922596
E_EL_ovS0_19A12_R_0	BY931109
E_EL_ovS0_19B01_F_0	BY922597
E_EL_ovS0_19B01_R_0	BY931110
E_EL_ovS0_19B02_F_0	BY922598
E_EL_ovS0_19B02_R_0	BY931111
E_EL_ovS0_19B04_F_0	BY922599
E_EL_ovS0_19B04_R_0	BY931112
E_EL_ovS0_19B05_R_0	BY931113
E_EL_ovS0_19B06_R_0	BY931114
E_EL_ovS0_19B07_F_0	BY922600
E_EL_ovS0_19B07_R_0	BY931115
E_EL_ovS0_19B08_F_0	BY922601
E_EL_ovS0_19B08_R_0	BY931116
E_EL_ovS0_19B09_F_0	BY922602
E_EL_ovS0_19B09_R_0	BY931117
E_EL_ovS0_19B10_F_0	BY922603
E_EL_ovS0_19B10_R_0	BY931118
E_EL_ovS0_19B11_F_0	BY922604
E_EL_ovS0_19B11_R_0	BY931119
E_EL_ovS0_19C01_F_0	BY922605
E_EL_ovS0_19C01_R_0	BY931120
E_EL_ovS0_19C02_F_0	BY922606
E_EL_ovS0_19C02_R_0	BY931121
E_EL_ovS0_19C03_F_0	BY922607
E_EL_ovS0_19C03_R_0	BY931122
E_EL_ovS0_19C05_F_0	BY922608
E_EL_ovS0_19C05_R_0	BY931123
E_EL_ovS0_19C06_F_0	BY922609
E_EL_ovS0_19C06_R_0	BY931124
E_EL_ovS0_19C07_R_0	BY931125
E_EL_ovS0_19C08_F_0	BY922610
E_EL_ovS0_19C08_R_0	BY931126
E_EL_ovS0_19C09_F_0	BY922611
E_EL_ovS0_19C09_R_0	BY931127
E_EL_ovS0_19C10_F_0	BY922612
E_EL_ovS0_19C10_R_0	BY931128
E_EL_ovS0_19C11_F_0	BY922613
E_EL_ovS0_19C11_R_0	BY931129
E_EL_ovS0_19C12_R_0	BY931130
E_EL_ovS0_19D01_F_0	BY922614
E_EL_ovS0_19D02_F_0	BY922615
E_EL_ovS0_19D02_R_0	BY931131
E_EL_ovS0_19D03_F_0	BY922616
E_EL_ovS0_19D03_R_0	BY931132
E_EL_ovS0_19D05_F_0	BY922617
E_EL_ovS0_19D05_R_0	BY931133
E_EL_ovS0_19D06_F_0	BY922618
E_EL_ovS0_19D06_R_0	BY931134
E_EL_ovS0_19D07_F_0	BY922619
E_EL_ovS0_19D07_R_0	BY931135
E_EL_ovS0_19D08_R_0	BY931136

E_EL_ovS0_19D09_R_0	BY931137
E_EL_ovS0_19D10_F_0	BY922620
E_EL_ovS0_19D10_R_0	BY931138
E_EL_ovS0_19D11_R_0	BY931139
E_EL_ovS0_19D12_F_0	BY922621
E_EL_ovS0_19D12_R_0	BY931140
E_EL_ovS0_19E01_F_0	BY922622
E_EL_ovS0_19E02_F_0	BY922623
E_EL_ovS0_19E02_R_0	BY931141
E_EL_ovS0_19E03_F_0	BY922624
E_EL_ovS0_19E03_R_0	BY931142
E_EL_ovS0_19E04_F_0	BY922625
E_EL_ovS0_19E06_F_0	BY922626
E_EL_ovS0_19E06_R_0	BY931143
E_EL_ovS0_19E07_F_0	BY922627
E_EL_ovS0_19E07_R_0	BY931144
E_EL_ovS0_19E08_F_0	BY922628
E_EL_ovS0_19E08_R_0	BY931145
E_EL_ovS0_19E09_F_0	BY922629
E_EL_ovS0_19E09_R_0	BY931146
E_EL_ovS0_19E10_F_0	BY922630
E_EL_ovS0_19E10_R_0	BY931147
E_EL_ovS0_19E11_F_0	BY922631
E_EL_ovS0_19E11_R_0	BY931148
E_EL_ovS0_19E12_F_0	BY922632
E_EL_ovS0_19E12_R_0	BY931149
E_EL_ovS0_19F01_R_0	BY931150
E_EL_ovS0_19F02_F_0	BY922633
E_EL_ovS0_19F02_R_0	BY931151
E_EL_ovS0_19F03_F_0	BY922634
E_EL_ovS0_19F03_R_0	BY931152
E_EL_ovS0_19F04_F_0	BY922635
E_EL_ovS0_19F04_R_0	BY931153
E_EL_ovS0_19F05_F_0	BY922636
E_EL_ovS0_19F05_R_0	BY931154
E_EL_ovS0_19F06_F_0	BY922637
E_EL_ovS0_19F06_R_0	BY931155
E_EL_ovS0_19F07_R_0	BY931156
E_EL_ovS0_19F08_F_0	BY922638
E_EL_ovS0_19F08_R_0	BY931157
E_EL_ovS0_19F09_F_0	BY922639
E_EL_ovS0_19F10_F_0	BY922640
E_EL_ovS0_19F11_F_0	BY922641
E_EL_ovS0_19F11_R_0	BY931158
E_EL_ovS0_19F12_F_0	BY922642
E_EL_ovS0_19F12_R_0	BY931159
E_EL_ovS0_19G01_F_0	BY922643
E_EL_ovS0_19G01_R_0	BY931160
E_EL_ovS0_19G02_F_0	BY922644
E_EL_ovS0_19G02_R_0	BY931161
E_EL_ovS0_19G03_F_0	BY922645
E_EL_ovS0_19G03_R_0	BY931162
E_EL_ovS0_19G04_F_0	BY922646
E_EL_ovS0_19G04_R_0	BY931163

E_EL_ovS0_19G05_F_0	BY922647
E_EL_ovS0_19G05_R_0	BY931164
E_EL_ovS0_19G06_F_0	BY922648
E_EL_ovS0_19G06_R_0	BY931165
E_EL_ovS0_19G07_F_0	BY922649
E_EL_ovS0_19G07_R_0	BY931166
E_EL_ovS0_19G08_F_0	BY922650
E_EL_ovS0_19G08_R_0	BY931167
E_EL_ovS0_19G09_F_0	BY922651
E_EL_ovS0_19G09_R_0	BY931168
E_EL_ovS0_19G10_F_0	BY922652
E_EL_ovS0_19G10_R_0	BY931169
E_EL_ovS0_19G11_F_0	BY922653
E_EL_ovS0_19G11_R_0	BY931170
E_EL_ovS0_19G12_F_0	BY922654
E_EL_ovS0_19G12_R_0	BY931171
E_EL_ovS0_19H01_F_0	BY922655
E_EL_ovS0_19H01_R_0	BY931172
E_EL_ovS0_19H02_F_0	BY922656
E_EL_ovS0_19H03_F_0	BY922657
E_EL_ovS0_19H03_R_0	BY931173
E_EL_ovS0_19H04_F_0	BY922658
E_EL_ovS0_19H04_R_0	BY931174
E_EL_ovS0_19H05_F_0	BY922659
E_EL_ovS0_19H05_R_0	BY931175
E_EL_ovS0_19H06_F_0	BY922660
E_EL_ovS0_19H06_R_0	BY931176
E_EL_ovS0_19H07_F_0	BY922661
E_EL_ovS0_19H07_R_0	BY931177
E_EL_ovS0_19H08_F_0	BY922662
E_EL_ovS0_19H08_R_0	BY931178
E_EL_ovS0_19H09_F_0	BY922663
E_EL_ovS0_19H09_R_0	BY931179
E_EL_ovS0_19H10_F_0	BY922664
E_EL_ovS0_19H10_R_0	BY931180
E_EL_ovS0_19H11_F_0	BY922665
E_EL_ovS0_19H11_R_0	BY931181
E_EL_ovS0_20A02_F_0	BY922666
E_EL_ovS0_20A02_R_0	BY931182
E_EL_ovS0_20A03_F_0	BY922667
E_EL_ovS0_20A03_R_0	BY931183
E_EL_ovS0_20A04_F_0	BY922668
E_EL_ovS0_20A04_R_0	BY931184
E_EL_ovS0_20A05_F_0	BY922669
E_EL_ovS0_20A05_R_0	BY931185
E_EL_ovS0_20A06_F_0	BY922670
E_EL_ovS0_20A06_R_0	BY931186
E_EL_ovS0_20A08_F_0	BY922671
E_EL_ovS0_20A08_R_0	BY931187
E_EL_ovS0_20A10_F_0	BY922672
E_EL_ovS0_20A11_F_0	BY922673
E_EL_ovS0_20A11_R_0	BY931188
E_EL_ovS0_20A12_R_0	BY931189
E_EL_ovS0_20B01_F_0	BY922674

E_EL_ovS0_20B01_R_0	BY931190
E_EL_ovS0_20B02_F_0	BY922675
E_EL_ovS0_20B02_R_0	BY931191
E_EL_ovS0_20B03_F_0	BY922676
E_EL_ovS0_20B03_R_0	BY931192
E_EL_ovS0_20B04_F_0	BY922677
E_EL_ovS0_20B04_R_0	BY931193
E_EL_ovS0_20B05_F_0	BY922678
E_EL_ovS0_20B05_R_0	BY931194
E_EL_ovS0_20B06_F_0	BY922679
E_EL_ovS0_20B09_F_0	BY922680
E_EL_ovS0_20B09_R_0	BY931195
E_EL_ovS0_20B10_F_0	BY922681
E_EL_ovS0_20B10_R_0	BY931196
E_EL_ovS0_20B11_F_0	BY922682
E_EL_ovS0_20B11_R_0	BY931197
E_EL_ovS0_20B12_R_0	BY931198
E_EL_ovS0_20C01_R_0	BY931199
E_EL_ovS0_20C03_F_0	BY922683
E_EL_ovS0_20C03_R_0	BY931200
E_EL_ovS0_20C04_F_0	BY922684
E_EL_ovS0_20C04_R_0	BY931201
E_EL_ovS0_20C05_F_0	BY922685
E_EL_ovS0_20C05_R_0	BY931202
E_EL_ovS0_20C06_F_0	BY922686
E_EL_ovS0_20C06_R_0	BY931203
E_EL_ovS0_20C07_F_0	BY922687
E_EL_ovS0_20C07_R_0	BY931204
E_EL_ovS0_20C08_F_0	BY922688
E_EL_ovS0_20C08_R_0	BY931205
E_EL_ovS0_20C09_F_0	BY922689
E_EL_ovS0_20C09_R_0	BY931206
E_EL_ovS0_20C10_F_0	BY922690
E_EL_ovS0_20C10_R_0	BY931207
E_EL_ovS0_20C11_F_0	BY922691
E_EL_ovS0_20C11_R_0	BY931208
E_EL_ovS0_20C12_F_0	BY922692
E_EL_ovS0_20C12_R_0	BY931209
E_EL_ovS0_20D01_F_0	BY922693
E_EL_ovS0_20D01_R_0	BY931210
E_EL_ovS0_20D02_F_0	BY922694
E_EL_ovS0_20D02_R_0	BY931211
E_EL_ovS0_20D03_F_0	BY922695
E_EL_ovS0_20D03_R_0	BY931212
E_EL_ovS0_20D04_F_0	BY922696
E_EL_ovS0_20D04_R_0	BY931213
E_EL_ovS0_20D06_F_0	BY922697
E_EL_ovS0_20D06_R_0	BY931214
E_EL_ovS0_20D07_F_0	BY922698
E_EL_ovS0_20D07_R_0	BY931215
E_EL_ovS0_20D08_F_0	BY922699
E_EL_ovS0_20D09_F_0	BY922700
E_EL_ovS0_20D09_R_0	BY931216
E_EL_ovS0_20D10_F_0	BY922701

E_EL_ovS0_20D10_R_0	BY931217
E_EL_ovS0_20D11_F_0	BY922702
E_EL_ovS0_20D11_R_0	BY931218
E_EL_ovS0_20D12_F_0	BY922703
E_EL_ovS0_20D12_R_0	BY931219
E_EL_ovS0_20E01_R_0	BY931220
E_EL_ovS0_20E02_F_0	BY922704
E_EL_ovS0_20E02_R_0	BY931221
E_EL_ovS0_20E03_F_0	BY922705
E_EL_ovS0_20E03_R_0	BY931222
E_EL_ovS0_20E04_F_0	BY922706
E_EL_ovS0_20E04_R_0	BY931223
E_EL_ovS0_20E05_F_0	BY922707
E_EL_ovS0_20E05_R_0	BY931224
E_EL_ovS0_20E07_F_0	BY922708
E_EL_ovS0_20E07_R_0	BY931225
E_EL_ovS0_20E08_F_0	BY922709
E_EL_ovS0_20E08_R_0	BY931226
E_EL_ovS0_20E09_F_0	BY922710
E_EL_ovS0_20E09_R_0	BY931227
E_EL_ovS0_20E10_F_0	BY922711
E_EL_ovS0_20E11_F_0	BY922712
E_EL_ovS0_20E11_R_0	BY931228
E_EL_ovS0_20E12_F_0	BY922713
E_EL_ovS0_20E12_R_0	BY931229
E_EL_ovS0_20F01_F_0	BY922714
E_EL_ovS0_20F02_F_0	BY922715
E_EL_ovS0_20F02_R_0	BY931230
E_EL_ovS0_20F04_F_0	BY922716
E_EL_ovS0_20F04_R_0	BY931231
E_EL_ovS0_20F05_F_0	BY922717
E_EL_ovS0_20F05_R_0	BY931232
E_EL_ovS0_20F06_F_0	BY922718
E_EL_ovS0_20F06_R_0	BY931233
E_EL_ovS0_20F07_F_0	BY922719
E_EL_ovS0_20F07_R_0	BY931234
E_EL_ovS0_20F09_F_0	BY922720
E_EL_ovS0_20F09_R_0	BY931235
E_EL_ovS0_20F10_F_0	BY922721
E_EL_ovS0_20F10_R_0	BY931236
E_EL_ovS0_20F12_F_0	BY922722
E_EL_ovS0_20F12_R_0	BY931237
E_EL_ovS0_20G01_F_0	BY922723
E_EL_ovS0_20G01_R_0	BY931238
E_EL_ovS0_20G02_F_0	BY922724
E_EL_ovS0_20G02_R_0	BY931239
E_EL_ovS0_20G03_F_0	BY922725
E_EL_ovS0_20G03_R_0	BY931240
E_EL_ovS0_20G04_F_0	BY922726
E_EL_ovS0_20G04_R_0	BY931241
E_EL_ovS0_20G07_F_0	BY922727
E_EL_ovS0_20G07_R_0	BY931242
E_EL_ovS0_20G08_F_0	BY922728
E_EL_ovS0_20G08_R_0	BY931243

E_EL_ovS0_20G09_F_0	BY922729
E_EL_ovS0_20G09_R_0	BY931244
E_EL_ovS0_20G10_F_0	BY922730
E_EL_ovS0_20G10_R_0	BY931245
E_EL_ovS0_20G11_F_0	BY922731
E_EL_ovS0_20G11_R_0	BY931246
E_EL_ovS0_20G12_F_0	BY922732
E_EL_ovS0_20G12_R_0	BY931247
E_EL_ovS0_20H01_F_0	BY922733
E_EL_ovS0_20H01_R_0	BY931248
E_EL_ovS0_20H02_F_0	BY922734
E_EL_ovS0_20H02_R_0	BY931249
E_EL_ovS0_20H03_F_0	BY922735
E_EL_ovS0_20H03_R_0	BY931250
E_EL_ovS0_20H04_F_0	BY922736
E_EL_ovS0_20H04_R_0	BY931251
E_EL_ovS0_20H06_F_0	BY922737
E_EL_ovS0_20H06_R_0	BY931252
E_EL_ovS0_20H07_F_0	BY922738
E_EL_ovS0_20H07_R_0	BY931253
E_EL_ovS0_20H08_F_0	BY922739
E_EL_ovS0_20H08_R_0	BY931254
E_EL_ovS0_20H09_F_0	BY922740
E_EL_ovS0_20H09_R_0	BY931255
E_EL_ovS0_20H10_F_0	BY922741
E_EL_ovS0_20H10_R_0	BY931256
E_EL_ovS0_20H11_F_0	BY922742
E_EL_ovS0_20H11_R_0	BY931257
E_EL_ovS0_21A01_F_0	BY922743
E_EL_ovS0_21A02_F_0	BY922744
E_EL_ovS0_21A02_R_0	BY931258
E_EL_ovS0_21A03_F_0	BY922745
E_EL_ovS0_21A03_R_0	BY931259
E_EL_ovS0_21A04_F_0	BY922746
E_EL_ovS0_21A04_R_0	BY931260
E_EL_ovS0_21A05_F_0	BY922747
E_EL_ovS0_21A05_R_0	BY931261
E_EL_ovS0_21A06_F_0	BY922748
E_EL_ovS0_21A06_R_0	BY931262
E_EL_ovS0_21A07_F_0	BY922749
E_EL_ovS0_21A07_R_0	BY931263
E_EL_ovS0_21A08_F_0	BY922750
E_EL_ovS0_21A08_R_0	BY931264
E_EL_ovS0_21A09_F_0	BY922751
E_EL_ovS0_21A09_R_0	BY931265
E_EL_ovS0_21A10_F_0	BY922752
E_EL_ovS0_21A10_R_0	BY931266
E_EL_ovS0_21A11_F_0	BY922753
E_EL_ovS0_21A11_R_0	BY931267
E_EL_ovS0_21A12_R_0	BY931268
E_EL_ovS0_21B01_F_0	BY922754
E_EL_ovS0_21B01_R_0	BY931269
E_EL_ovS0_21B02_F_0	BY922755
E_EL_ovS0_21B02_R_0	BY931270

E_EL_ovS0_21B03_F_0	BY922756
E_EL_ovS0_21B03_R_0	BY931271
E_EL_ovS0_21B04_F_0	BY922757
E_EL_ovS0_21B04_R_0	BY931272
E_EL_ovS0_21B05_F_0	BY922758
E_EL_ovS0_21B05_R_0	BY931273
E_EL_ovS0_21B06_F_0	BY922759
E_EL_ovS0_21B06_R_0	BY931274
E_EL_ovS0_21B09_F_0	BY922760
E_EL_ovS0_21B09_R_0	BY931275
E_EL_ovS0_21B10_F_0	BY922761
E_EL_ovS0_21B10_R_0	BY931276
E_EL_ovS0_21B11_R_0	BY931277
E_EL_ovS0_21B12_R_0	BY931278
E_EL_ovS0_21C01_F_0	BY922762
E_EL_ovS0_21C01_R_0	BY931279
E_EL_ovS0_21C02_F_0	BY922763
E_EL_ovS0_21C02_R_0	BY931280
E_EL_ovS0_21C03_F_0	BY922764
E_EL_ovS0_21C03_R_0	BY931281
E_EL_ovS0_21C04_F_0	BY922765
E_EL_ovS0_21C04_R_0	BY931282
E_EL_ovS0_21C05_F_0	BY922766
E_EL_ovS0_21C05_R_0	BY931283
E_EL_ovS0_21C06_F_0	BY922767
E_EL_ovS0_21C06_R_0	BY931284
E_EL_ovS0_21C07_F_0	BY922768
E_EL_ovS0_21C07_R_0	BY931285
E_EL_ovS0_21C08_F_0	BY922769
E_EL_ovS0_21C08_R_0	BY931286
E_EL_ovS0_21C09_F_0	BY922770
E_EL_ovS0_21C10_R_0	BY931287
E_EL_ovS0_21C11_R_0	BY931288
E_EL_ovS0_21C12_F_0	BY922771
E_EL_ovS0_21C12_R_0	BY931289
E_EL_ovS0_21D01_F_0	BY922772
E_EL_ovS0_21D01_R_0	BY931290
E_EL_ovS0_21D03_F_0	BY922773
E_EL_ovS0_21D03_R_0	BY931291
E_EL_ovS0_21D04_F_0	BY922774
E_EL_ovS0_21D04_R_0	BY931292
E_EL_ovS0_21D05_F_0	BY922775
E_EL_ovS0_21D05_R_0	BY931293
E_EL_ovS0_21D06_F_0	BY922776
E_EL_ovS0_21D06_R_0	BY931294
E_EL_ovS0_21D07_F_0	BY922777
E_EL_ovS0_21D07_R_0	BY931295
E_EL_ovS0_21D09_F_0	BY922778
E_EL_ovS0_21D09_R_0	BY931296
E_EL_ovS0_21D10_F_0	BY922779
E_EL_ovS0_21D10_R_0	BY931297
E_EL_ovS0_21E01_F_0	BY922780
E_EL_ovS0_21E01_R_0	BY931298
E_EL_ovS0_21E02_F_0	BY922781

E_EL_ovS0_21E03_R_0	BY931299
E_EL_ovS0_21E04_F_0	BY922782
E_EL_ovS0_21E06_F_0	BY922783
E_EL_ovS0_21E06_R_0	BY931300
E_EL_ovS0_21E07_F_0	BY922784
E_EL_ovS0_21E07_R_0	BY931301
E_EL_ovS0_21E08_R_0	BY931302
E_EL_ovS0_21E09_F_0	BY922785
E_EL_ovS0_21E09_R_0	BY931303
E_EL_ovS0_21E10_F_0	BY922786
E_EL_ovS0_21E10_R_0	BY931304
E_EL_ovS0_21E11_R_0	BY931305
E_EL_ovS0_21E12_F_0	BY922787
E_EL_ovS0_21E12_R_0	BY931306
E_EL_ovS0_21F03_F_0	BY922788
E_EL_ovS0_21F03_R_0	BY931307
E_EL_ovS0_21F04_F_0	BY922789
E_EL_ovS0_21F04_R_0	BY931308
E_EL_ovS0_21F05_F_0	BY922790
E_EL_ovS0_21F05_R_0	BY931309
E_EL_ovS0_21F06_F_0	BY922791
E_EL_ovS0_21F06_R_0	BY931310
E_EL_ovS0_21F07_F_0	BY922792
E_EL_ovS0_21F07_R_0	BY931311
E_EL_ovS0_21F08_F_0	BY922793
E_EL_ovS0_21F08_R_0	BY931312
E_EL_ovS0_21F09_F_0	BY922794
E_EL_ovS0_21F09_R_0	BY931313
E_EL_ovS0_21F10_F_0	BY922795
E_EL_ovS0_21F10_R_0	BY931314
E_EL_ovS0_21G01_F_0	BY922796
E_EL_ovS0_21G01_R_0	BY931315
E_EL_ovS0_21G02_F_0	BY922797
E_EL_ovS0_21G02_R_0	BY931316
E_EL_ovS0_21G03_F_0	BY922798
E_EL_ovS0_21G03_R_0	BY931317
E_EL_ovS0_21G04_F_0	BY922799
E_EL_ovS0_21G04_R_0	BY931318
E_EL_ovS0_21G05_F_0	BY922800
E_EL_ovS0_21G05_R_0	BY931319
E_EL_ovS0_21G07_F_0	BY922801
E_EL_ovS0_21G08_F_0	BY922802
E_EL_ovS0_21G08_R_0	BY931320
E_EL_ovS0_21G09_F_0	BY922803
E_EL_ovS0_21G10_F_0	BY922804
E_EL_ovS0_21G10_R_0	BY931321
E_EL_ovS0_21G11_F_0	BY922805
E_EL_ovS0_21G11_R_0	BY931322
E_EL_ovS0_21H02_F_0	BY922806
E_EL_ovS0_21H02_R_0	BY931323
E_EL_ovS0_21H03_F_0	BY922807
E_EL_ovS0_21H03_R_0	BY931324
E_EL_ovS0_21H04_F_0	BY922808
E_EL_ovS0_21H04_R_0	BY931325

E_EL_ovS0_21H05_F_0	BY922809
E_EL_ovS0_21H05_R_0	BY931326
E_EL_ovS0_21H07_F_0	BY922810
E_EL_ovS0_21H07_R_0	BY931327
E_EL_ovS0_21H08_F_0	BY922811
E_EL_ovS0_21H08_R_0	BY931328
E_EL_ovS0_21H09_F_0	BY922812
E_EL_ovS0_21H09_R_0	BY931329
E_EL_ovS0_21H10_F_0	BY922813
E_EL_ovS0_21H10_R_0	BY931330
E_EL_ovS0_21H11_F_0	BY922814
E_EL_ovS0_21H11_R_0	BY931331
E_EL_ovS0_21H12_F_0	BY922815
E_EL_ovS0_21H12_R_0	BY931332
E_EL_ovS0_22A01_F_0	BY922816
E_EL_ovS0_22A03_F_0	BY922817
E_EL_ovS0_22A05_F_0	BY922818
E_EL_ovS0_22A05_R_0	BY931333
E_EL_ovS0_22A06_F_0	BY922819
E_EL_ovS0_22A06_R_0	BY931334
E_EL_ovS0_22A07_F_0	BY922820
E_EL_ovS0_22A07_R_0	BY931335
E_EL_ovS0_22A08_F_0	BY922821
E_EL_ovS0_22A08_R_0	BY931336
E_EL_ovS0_22A09_F_0	BY922822
E_EL_ovS0_22A10_R_0	BY931337
E_EL_ovS0_22A11_F_0	BY922823
E_EL_ovS0_22A11_R_0	BY931338
E_EL_ovS0_22A12_F_0	BY922824
E_EL_ovS0_22A12_R_0	BY931339
E_EL_ovS0_22B09_F_0	BY922825
E_EL_ovS0_22B09_R_0	BY931340
E_EL_ovS0_22B12_R_0	BY931341
E_EL_ovS0_22C01_F_0	BY922826
E_EL_ovS0_22C01_R_0	BY931342
E_EL_ovS0_22C02_F_0	BY922827
E_EL_ovS0_22C02_R_0	BY931343
E_EL_ovS0_22C03_F_0	BY922828
E_EL_ovS0_22C03_R_0	BY931344
E_EL_ovS0_22C04_F_0	BY922829
E_EL_ovS0_22C04_R_0	BY931345
E_EL_ovS0_22C05_F_0	BY922830
E_EL_ovS0_22C05_R_0	BY931346
E_EL_ovS0_22C06_R_0	BY931347
E_EL_ovS0_22C07_F_0	BY922831
E_EL_ovS0_22C07_R_0	BY931348
E_EL_ovS0_22C08_F_0	BY922832
E_EL_ovS0_22C08_R_0	BY931349
E_EL_ovS0_22C09_F_0	BY922833
E_EL_ovS0_22C09_R_0	BY931350
E_EL_ovS0_22C10_F_0	BY922834
E_EL_ovS0_22C11_F_0	BY922835
E_EL_ovS0_22C11_R_0	BY931351
E_EL_ovS0_22C12_F_0	BY922836

E_EL_ovS0_22C12_R_0	BY931352
E_EL_ovS0_22D01_F_0	BY922837
E_EL_ovS0_22D01_R_0	BY931353
E_EL_ovS0_22D02_F_0	BY922838
E_EL_ovS0_22D02_R_0	BY931354
E_EL_ovS0_22D03_F_0	BY922839
E_EL_ovS0_22D03_R_0	BY931355
E_EL_ovS0_22D05_F_0	BY922840
E_EL_ovS0_22D05_R_0	BY931356
E_EL_ovS0_22D06_F_0	BY922841
E_EL_ovS0_22D06_R_0	BY931357
E_EL_ovS0_22D07_F_0	BY922842
E_EL_ovS0_22D07_R_0	BY931358
E_EL_ovS0_22D08_F_0	BY922843
E_EL_ovS0_22D09_F_0	BY922844
E_EL_ovS0_22D09_R_0	BY931359
E_EL_ovS0_22D10_F_0	BY922845
E_EL_ovS0_22D10_R_0	BY931360
E_EL_ovS0_22D12_F_0	BY922846
E_EL_ovS0_22D12_R_0	BY931361
E_EL_ovS0_22E01_F_0	BY922847
E_EL_ovS0_22E01_R_0	BY931362
E_EL_ovS0_22E05_F_0	BY922848
E_EL_ovS0_22E05_R_0	BY931363
E_EL_ovS0_22E06_R_0	BY931364
E_EL_ovS0_22E08_F_0	BY922849
E_EL_ovS0_22E08_R_0	BY931365
E_EL_ovS0_22E09_F_0	BY922850
E_EL_ovS0_22E09_R_0	BY931366
E_EL_ovS0_22E10_F_0	BY922851
E_EL_ovS0_22E10_R_0	BY931367
E_EL_ovS0_22E11_F_0	BY922852
E_EL_ovS0_22E12_F_0	BY922853
E_EL_ovS0_22E12_R_0	BY931368
E_EL_ovS0_22F01_F_0	BY922854
E_EL_ovS0_22F01_R_0	BY931369
E_EL_ovS0_22F03_F_0	BY922855
E_EL_ovS0_22F03_R_0	BY931370
E_EL_ovS0_22F05_R_0	BY931371
E_EL_ovS0_22F06_R_0	BY931372
E_EL_ovS0_22F08_F_0	BY922856
E_EL_ovS0_22F08_R_0	BY931373
E_EL_ovS0_22F09_F_0	BY922857
E_EL_ovS0_22F09_R_0	BY931374
E_EL_ovS0_22F10_F_0	BY922858
E_EL_ovS0_22F10_R_0	BY931375
E_EL_ovS0_22G02_F_0	BY922859
E_EL_ovS0_22G02_R_0	BY931376
E_EL_ovS0_22G03_F_0	BY922860
E_EL_ovS0_22G03_R_0	BY931377
E_EL_ovS0_22G04_F_0	BY922861
E_EL_ovS0_22G04_R_0	BY931378
E_EL_ovS0_22G05_F_0	BY922862
E_EL_ovS0_22G05_R_0	BY931379

E_EL_ovS0_22G06_R_0	BY931380
E_EL_ovS0_22G07_R_0	BY931381
E_EL_ovS0_22G08_F_0	BY922863
E_EL_ovS0_22G08_R_0	BY931382
E_EL_ovS0_22G09_F_0	BY922864
E_EL_ovS0_22G10_F_0	BY922865
E_EL_ovS0_22G10_R_0	BY931383
E_EL_ovS0_22G11_R_0	BY931384
E_EL_ovS0_22G12_F_0	BY922866
E_EL_ovS0_22G12_R_0	BY931385
E_EL_ovS0_22H02_F_0	BY922867
E_EL_ovS0_22H02_R_0	BY931386
E_EL_ovS0_22H03_R_0	BY931387
E_EL_ovS0_22H04_R_0	BY931388
E_EL_ovS0_22H07_F_0	BY922868
E_EL_ovS0_22H07_R_0	BY931389
E_EL_ovS0_22H08_F_0	BY922869
E_EL_ovS0_22H08_R_0	BY931390
E_EL_ovS0_22H09_F_0	BY922870
E_EL_ovS0_22H09_R_0	BY931391
E_EL_ovS0_22H10_F_0	BY922871
E_EL_ovS0_22H10_R_0	BY931392
E_EL_ovS0_22H11_F_0	BY922872
E_EL_ovS0_23A01_F_0	BY922873
E_EL_ovS0_23A01_R_0	BY931393
E_EL_ovS0_23A02_F_0	BY922874
E_EL_ovS0_23A02_R_0	BY931394
E_EL_ovS0_23A03_F_0	BY922875
E_EL_ovS0_23A03_R_0	BY931395
E_EL_ovS0_23A04_F_0	BY922876
E_EL_ovS0_23A04_R_0	BY931396
E_EL_ovS0_23A06_F_0	BY922877
E_EL_ovS0_23A06_R_0	BY931397
E_EL_ovS0_23A07_F_0	BY922878
E_EL_ovS0_23A07_R_0	BY931398
E_EL_ovS0_23A08_F_0	BY922879
E_EL_ovS0_23A08_R_0	BY931399
E_EL_ovS0_23A09_F_0	BY922880
E_EL_ovS0_23A11_F_0	BY922881
E_EL_ovS0_23A11_R_0	BY931400
E_EL_ovS0_23A12_F_0	BY922882
E_EL_ovS0_23A12_R_0	BY931401
E_EL_ovS0_23B01_F_0	BY922883
E_EL_ovS0_23B01_R_0	BY931402
E_EL_ovS0_23B02_F_0	BY922884
E_EL_ovS0_23B02_R_0	BY931403
E_EL_ovS0_23B03_F_0	BY922885
E_EL_ovS0_23B03_R_0	BY931404
E_EL_ovS0_23B04_F_0	BY922886
E_EL_ovS0_23B04_R_0	BY931405
E_EL_ovS0_23B05_F_0	BY922887
E_EL_ovS0_23B05_R_0	BY931406
E_EL_ovS0_23B06_F_0	BY922888
E_EL_ovS0_23B06_R_0	BY931407

E_EL_ovS0_23B07_F_0	BY922889
E_EL_ovS0_23B07_R_0	BY931408
E_EL_ovS0_23B08_R_0	BY931409
E_EL_ovS0_23B09_F_0	BY922890
E_EL_ovS0_23B09_R_0	BY931410
E_EL_ovS0_23B10_F_0	BY922891
E_EL_ovS0_23B10_R_0	BY931411
E_EL_ovS0_23B11_F_0	BY922892
E_EL_ovS0_23B12_F_0	BY922893
E_EL_ovS0_23C01_F_0	BY922894
E_EL_ovS0_23C01_R_0	BY931412
E_EL_ovS0_23C03_F_0	BY922895
E_EL_ovS0_23C03_R_0	BY931413
E_EL_ovS0_23C04_F_0	BY922896
E_EL_ovS0_23C04_R_0	BY931414
E_EL_ovS0_23C05_F_0	BY922897
E_EL_ovS0_23C05_R_0	BY931415
E_EL_ovS0_23C06_F_0	BY922898
E_EL_ovS0_23C06_R_0	BY931416
E_EL_ovS0_23C07_F_0	BY922899
E_EL_ovS0_23C07_R_0	BY931417
E_EL_ovS0_23C08_F_0	BY922900
E_EL_ovS0_23C08_R_0	BY931418
E_EL_ovS0_23C09_F_0	BY922901
E_EL_ovS0_23C09_R_0	BY931419
E_EL_ovS0_23C11_F_0	BY922902
E_EL_ovS0_23C12_F_0	BY922903
E_EL_ovS0_23C12_R_0	BY931420
E_EL_ovS0_23D01_F_0	BY922904
E_EL_ovS0_23D01_R_0	BY931421
E_EL_ovS0_23D02_F_0	BY922905
E_EL_ovS0_23D02_R_0	BY931422
E_EL_ovS0_23D03_F_0	BY922906
E_EL_ovS0_23D03_R_0	BY931423
E_EL_ovS0_23D05_F_0	BY922907
E_EL_ovS0_23D05_R_0	BY931424
E_EL_ovS0_23D06_R_0	BY931425
E_EL_ovS0_23D07_F_0	BY922908
E_EL_ovS0_23D07_R_0	BY931426
E_EL_ovS0_23D08_F_0	BY922909
E_EL_ovS0_23D08_R_0	BY931427
E_EL_ovS0_23D09_F_0	BY922910
E_EL_ovS0_23D09_R_0	BY931428
E_EL_ovS0_23D10_R_0	BY931429
E_EL_ovS0_23D11_F_0	BY922911
E_EL_ovS0_23D11_R_0	BY931430
E_EL_ovS0_23D12_F_0	BY922912
E_EL_ovS0_23D12_R_0	BY931431
E_EL_ovS0_23E01_F_0	BY922913
E_EL_ovS0_23E01_R_0	BY931432
E_EL_ovS0_23E02_F_0	BY922914
E_EL_ovS0_23E02_R_0	BY931433
E_EL_ovS0_23E03_F_0	BY922915
E_EL_ovS0_23E03_R_0	BY931434

E_EL_ovS0_23E04_F_0	BY922916
E_EL_ovS0_23E04_R_0	BY931435
E_EL_ovS0_23E05_F_0	BY922917
E_EL_ovS0_23E05_R_0	BY931436
E_EL_ovS0_23E06_F_0	BY922918
E_EL_ovS0_23E07_R_0	BY931437
E_EL_ovS0_23E08_F_0	BY922919
E_EL_ovS0_23E08_R_0	BY931438
E_EL_ovS0_23E09_R_0	BY931439
E_EL_ovS0_23E10_F_0	BY922920
E_EL_ovS0_23E10_R_0	BY931440
E_EL_ovS0_23E11_R_0	BY931441
E_EL_ovS0_23E12_F_0	BY922921
E_EL_ovS0_23E12_R_0	BY931442
E_EL_ovS0_23F01_F_0	BY922922
E_EL_ovS0_23F01_R_0	BY931443
E_EL_ovS0_23F02_F_0	BY922923
E_EL_ovS0_23F03_F_0	BY922924
E_EL_ovS0_23F03_R_0	BY931444
E_EL_ovS0_23F04_F_0	BY922925
E_EL_ovS0_23F05_R_0	BY931445
E_EL_ovS0_23F06_F_0	BY922926
E_EL_ovS0_23F06_R_0	BY931446
E_EL_ovS0_23F07_R_0	BY931447
E_EL_ovS0_23F08_F_0	BY922927
E_EL_ovS0_23F08_R_0	BY931448
E_EL_ovS0_23F09_F_0	BY922928
E_EL_ovS0_23F09_R_0	BY931449
E_EL_ovS0_23F10_F_0	BY922929
E_EL_ovS0_23F10_R_0	BY931450
E_EL_ovS0_23F11_F_0	BY922930
E_EL_ovS0_23F12_F_0	BY922931
E_EL_ovS0_23G01_F_0	BY922932
E_EL_ovS0_23G01_R_0	BY931451
E_EL_ovS0_23G02_F_0	BY922933
E_EL_ovS0_23G02_R_0	BY931452
E_EL_ovS0_23G03_F_0	BY922934
E_EL_ovS0_23G03_R_0	BY931453
E_EL_ovS0_23G05_F_0	BY922935
E_EL_ovS0_23G05_R_0	BY931454
E_EL_ovS0_23G06_F_0	BY922936
E_EL_ovS0_23G06_R_0	BY931455
E_EL_ovS0_23G07_F_0	BY922937
E_EL_ovS0_23G07_R_0	BY931456
E_EL_ovS0_23G08_F_0	BY922938
E_EL_ovS0_23G08_R_0	BY931457
E_EL_ovS0_23G09_F_0	BY922939
E_EL_ovS0_23G09_R_0	BY931458
E_EL_ovS0_23G10_F_0	BY922940
E_EL_ovS0_23G10_R_0	BY931459
E_EL_ovS0_23G11_R_0	BY931460
E_EL_ovS0_23G12_F_0	BY922941
E_EL_ovS0_23G12_R_0	BY931461
E_EL_ovS0_23H02_F_0	BY922942

E_EL_ovS0_23H02_R_0	BY931462
E_EL_ovS0_23H03_F_0	BY922943
E_EL_ovS0_23H03_R_0	BY931463
E_EL_ovS0_23H05_F_0	BY922944
E_EL_ovS0_23H05_R_0	BY931464
E_EL_ovS0_23H06_F_0	BY922945
E_EL_ovS0_23H06_R_0	BY931465
E_EL_ovS0_23H07_F_0	BY922946
E_EL_ovS0_23H07_R_0	BY931466
E_EL_ovS0_23H08_R_0	BY931467
E_EL_ovS0_23H10_R_0	BY931468
E_EL_ovS0_23H11_R_0	BY931469
E_EL_ovS0_23H12_F_0	BY922947
E_EL_ovS0_23H12_R_0	BY931470
E_EL_ovS0_24A01_F_0	BY922948
E_EL_ovS0_24A01_R_0	BY931471
E_EL_ovS0_24A02_R_0	BY931472
E_EL_ovS0_24A03_F_0	BY922949
E_EL_ovS0_24A03_R_0	BY931473
E_EL_ovS0_24A04_F_0	BY922950
E_EL_ovS0_24A04_R_0	BY931474
E_EL_ovS0_24A05_F_0	BY922951
E_EL_ovS0_24A05_R_0	BY931475
E_EL_ovS0_24A06_F_0	BY922952
E_EL_ovS0_24A06_R_0	BY931476
E_EL_ovS0_24A07_F_0	BY922953
E_EL_ovS0_24A07_R_0	BY931477
E_EL_ovS0_24A08_F_0	BY922954
E_EL_ovS0_24A08_R_0	BY931478
E_EL_ovS0_24A09_F_0	BY922955
E_EL_ovS0_24A09_R_0	BY931479
E_EL_ovS0_24A10_F_0	BY922956
E_EL_ovS0_24A10_R_0	BY931480
E_EL_ovS0_24A11_F_0	BY922957
E_EL_ovS0_24A11_R_0	BY931481
E_EL_ovS0_24A12_F_0	BY922958
E_EL_ovS0_24A12_R_0	BY931482
E_EL_ovS0_24B01_R_0	BY931483
E_EL_ovS0_24B02_F_0	BY922959
E_EL_ovS0_24B02_R_0	BY931484
E_EL_ovS0_24B03_F_0	BY922960
E_EL_ovS0_24B03_R_0	BY931485
E_EL_ovS0_24B04_F_0	BY922961
E_EL_ovS0_24B04_R_0	BY931486
E_EL_ovS0_24B05_R_0	BY931487
E_EL_ovS0_24B06_F_0	BY922962
E_EL_ovS0_24B06_R_0	BY931488
E_EL_ovS0_24B07_F_0	BY922963
E_EL_ovS0_24B08_F_0	BY922964
E_EL_ovS0_24B08_R_0	BY931489
E_EL_ovS0_24B09_F_0	BY922965
E_EL_ovS0_24B09_R_0	BY931490
E_EL_ovS0_24B10_F_0	BY922966
E_EL_ovS0_24B10_R_0	BY931491

E_EL_ovS0_24B11_R_0	BY931492
E_EL_ovS0_24C01_F_0	BY922967
E_EL_ovS0_24C02_F_0	BY922968
E_EL_ovS0_24C03_F_0	BY922969
E_EL_ovS0_24C03_R_0	BY931493
E_EL_ovS0_24C04_F_0	BY922970
E_EL_ovS0_24C04_R_0	BY931494
E_EL_ovS0_24C05_F_0	BY922971
E_EL_ovS0_24C05_R_0	BY931495
E_EL_ovS0_24C06_F_0	BY922972
E_EL_ovS0_24C06_R_0	BY931496
E_EL_ovS0_24C07_F_0	BY922973
E_EL_ovS0_24C07_R_0	BY931497
E_EL_ovS0_24C08_F_0	BY922974
E_EL_ovS0_24C08_R_0	BY931498
E_EL_ovS0_24C09_F_0	BY922975
E_EL_ovS0_24C09_R_0	BY931499
E_EL_ovS0_24C11_F_0	BY922976
E_EL_ovS0_24C11_R_0	BY931500
E_EL_ovS0_24C12_F_0	BY922977
E_EL_ovS0_24C12_R_0	BY931501
E_EL_ovS0_24D01_F_0	BY922978
E_EL_ovS0_24D01_R_0	BY931502
E_EL_ovS0_24D02_F_0	BY922979
E_EL_ovS0_24D02_R_0	BY931503
E_EL_ovS0_24D03_F_0	BY922980
E_EL_ovS0_24D04_F_0	BY922981
E_EL_ovS0_24D04_R_0	BY931504
E_EL_ovS0_24D05_R_0	BY931505
E_EL_ovS0_24D06_F_0	BY922982
E_EL_ovS0_24D06_R_0	BY931506
E_EL_ovS0_24D07_F_0	BY922983
E_EL_ovS0_24D08_F_0	BY922984
E_EL_ovS0_24D08_R_0	BY931507
E_EL_ovS0_24D09_F_0	BY922985
E_EL_ovS0_24D09_R_0	BY931508
E_EL_ovS0_24D10_R_0	BY931509
E_EL_ovS0_24D11_F_0	BY922986
E_EL_ovS0_24D11_R_0	BY931510
E_EL_ovS0_24D12_R_0	BY931511
E_EL_ovS0_24E01_R_0	BY931512
E_EL_ovS0_24E02_R_0	BY931513
E_EL_ovS0_24E03_F_0	BY922987
E_EL_ovS0_24E03_R_0	BY931514
E_EL_ovS0_24E04_F_0	BY922988
E_EL_ovS0_24E04_R_0	BY931515
E_EL_ovS0_24E05_F_0	BY922989
E_EL_ovS0_24E05_R_0	BY931516
E_EL_ovS0_24E06_F_0	BY922990
E_EL_ovS0_24E06_R_0	BY931517
E_EL_ovS0_24E07_F_0	BY922991
E_EL_ovS0_24E07_R_0	BY931518
E_EL_ovS0_24E09_F_0	BY922992
E_EL_ovS0_24E09_R_0	BY931519

E_EL_ovS0_24E10_F_0	BY922993
E_EL_ovS0_24E10_R_0	BY931520
E_EL_ovS0_24E11_F_0	BY922994
E_EL_ovS0_24E11_R_0	BY931521
E_EL_ovS0_24E12_F_0	BY922995
E_EL_ovS0_24E12_R_0	BY931522
E_EL_ovS0_24F01_F_0	BY922996
E_EL_ovS0_24F01_R_0	BY931523
E_EL_ovS0_24F03_F_0	BY922997
E_EL_ovS0_24F03_R_0	BY931524
E_EL_ovS0_24F04_F_0	BY922998
E_EL_ovS0_24F04_R_0	BY931525
E_EL_ovS0_24F05_F_0	BY922999
E_EL_ovS0_24F05_R_0	BY931526
E_EL_ovS0_24F06_F_0	BY923000
E_EL_ovS0_24F06_R_0	BY931527
E_EL_ovS0_24F07_R_0	BY931528
E_EL_ovS0_24F08_F_0	BY923001
E_EL_ovS0_24F08_R_0	BY931529
E_EL_ovS0_24F09_F_0	BY923002
E_EL_ovS0_24F09_R_0	BY931530
E_EL_ovS0_24F10_F_0	BY923003
E_EL_ovS0_24F10_R_0	BY931531
E_EL_ovS0_24F11_F_0	BY923004
E_EL_ovS0_24F11_R_0	BY931532
E_EL_ovS0_24F12_F_0	BY923005
E_EL_ovS0_24F12_R_0	BY931533
E_EL_ovS0_24G01_R_0	BY931534
E_EL_ovS0_24G02_F_0	BY923006
E_EL_ovS0_24G02_R_0	BY931535
E_EL_ovS0_24G03_F_0	BY923007
E_EL_ovS0_24G03_R_0	BY931536
E_EL_ovS0_24G04_F_0	BY923008
E_EL_ovS0_24G04_R_0	BY931537
E_EL_ovS0_24G05_F_0	BY923009
E_EL_ovS0_24G05_R_0	BY931538
E_EL_ovS0_24G07_F_0	BY923010
E_EL_ovS0_24G07_R_0	BY931539
E_EL_ovS0_24G08_F_0	BY923011
E_EL_ovS0_24G08_R_0	BY931540
E_EL_ovS0_24G09_F_0	BY923012
E_EL_ovS0_24G10_F_0	BY923013
E_EL_ovS0_24G10_R_0	BY931541
E_EL_ovS0_24G11_F_0	BY923014
E_EL_ovS0_24G11_R_0	BY931542
E_EL_ovS0_24G12_F_0	BY923015
E_EL_ovS0_24H01_F_0	BY923016
E_EL_ovS0_24H01_R_0	BY931543
E_EL_ovS0_24H02_F_0	BY923017
E_EL_ovS0_24H02_R_0	BY931544
E_EL_ovS0_24H03_F_0	BY923018
E_EL_ovS0_24H03_R_0	BY931545
E_EL_ovS0_24H04_F_0	BY923019
E_EL_ovS0_24H04_R_0	BY931546

E_EL_ovS0_24H05_F_0	BY923020
E_EL_ovS0_24H05_R_0	BY931547
E_EL_ovS0_24H06_R_0	BY931548
E_EL_ovS0_24H07_F_0	BY923021
E_EL_ovS0_24H07_R_0	BY931549
E_EL_ovS0_24H08_R_0	BY931550
E_EL_ovS0_24H11_F_0	BY923022
E_EL_ovS0_24H11_R_0	BY931551
E_EL_ovS0_24H12_F_0	BY923023
E_EL_ovS0_24H12_R_0	BY931552
E_EL_ovS0_25A01_R_0	BY931553
E_EL_ovS0_25A02_F_0	BY923024
E_EL_ovS0_25A03_F_0	BY923025
E_EL_ovS0_25A03_R_0	BY931554
E_EL_ovS0_25A04_F_0	BY923026
E_EL_ovS0_25A04_R_0	BY931555
E_EL_ovS0_25A05_F_0	BY923027
E_EL_ovS0_25A05_R_0	BY931556
E_EL_ovS0_25A06_F_0	BY923028
E_EL_ovS0_25A06_R_0	BY931557
E_EL_ovS0_25A07_F_0	BY923029
E_EL_ovS0_25A07_R_0	BY931558
E_EL_ovS0_25A08_F_0	BY923030
E_EL_ovS0_25A08_R_0	BY931559
E_EL_ovS0_25A09_F_0	BY923031
E_EL_ovS0_25A09_R_0	BY931560
E_EL_ovS0_25A11_F_0	BY923032
E_EL_ovS0_25A11_R_0	BY931561
E_EL_ovS0_25A12_F_0	BY923033
E_EL_ovS0_25A12_R_0	BY931562
E_EL_ovS0_25B01_F_0	BY923034
E_EL_ovS0_25B01_R_0	BY931563
E_EL_ovS0_25B02_F_0	BY923035
E_EL_ovS0_25B04_F_0	BY923036
E_EL_ovS0_25B04_R_0	BY931564
E_EL_ovS0_25B05_F_0	BY923037
E_EL_ovS0_25B05_R_0	BY931565
E_EL_ovS0_25B06_F_0	BY923038
E_EL_ovS0_25B06_R_0	BY931566
E_EL_ovS0_25B07_F_0	BY923039
E_EL_ovS0_25B07_R_0	BY931567
E_EL_ovS0_25B08_F_0	BY923040
E_EL_ovS0_25B08_R_0	BY931568
E_EL_ovS0_25B09_F_0	BY923041
E_EL_ovS0_25B09_R_0	BY931569
E_EL_ovS0_25B10_F_0	BY923042
E_EL_ovS0_25B10_R_0	BY931570
E_EL_ovS0_25B11_F_0	BY923043
E_EL_ovS0_25C01_R_0	BY931571
E_EL_ovS0_25C02_F_0	BY923044
E_EL_ovS0_25C02_R_0	BY931572
E_EL_ovS0_25C03_F_0	BY923045
E_EL_ovS0_25C03_R_0	BY931573
E_EL_ovS0_25C04_F_0	BY923046

E_EL_ovS0_25C04_R_0	BY931574
E_EL_ovS0_25C06_F_0	BY923047
E_EL_ovS0_25C06_R_0	BY931575
E_EL_ovS0_25C07_F_0	BY923048
E_EL_ovS0_25C07_R_0	BY931576
E_EL_ovS0_25C08_F_0	BY923049
E_EL_ovS0_25C08_R_0	BY931577
E_EL_ovS0_25C09_F_0	BY923050
E_EL_ovS0_25C09_R_0	BY931578
E_EL_ovS0_25C10_R_0	BY931579
E_EL_ovS0_25C11_F_0	BY923051
E_EL_ovS0_25C11_R_0	BY931580
E_EL_ovS0_25C12_R_0	BY931581
E_EL_ovS0_25D01_F_0	BY923052
E_EL_ovS0_25D01_R_0	BY931582
E_EL_ovS0_25D02_F_0	BY923053
E_EL_ovS0_25D02_R_0	BY931583
E_EL_ovS0_25D03_F_0	BY923054
E_EL_ovS0_25D03_R_0	BY931584
E_EL_ovS0_25D04_F_0	BY923055
E_EL_ovS0_25D04_R_0	BY931585
E_EL_ovS0_25D05_F_0	BY923056
E_EL_ovS0_25D05_R_0	BY931586
E_EL_ovS0_25D06_F_0	BY923057
E_EL_ovS0_25D06_R_0	BY931587
E_EL_ovS0_25D07_F_0	BY923058
E_EL_ovS0_25D07_R_0	BY931588
E_EL_ovS0_25D08_F_0	BY923059
E_EL_ovS0_25D08_R_0	BY931589
E_EL_ovS0_25D09_F_0	BY923060
E_EL_ovS0_25D09_R_0	BY931590
E_EL_ovS0_25D10_F_0	BY923061
E_EL_ovS0_25D10_R_0	BY931591
E_EL_ovS0_25D11_F_0	BY923062
E_EL_ovS0_25D11_R_0	BY931592
E_EL_ovS0_25D12_F_0	BY923063
E_EL_ovS0_25D12_R_0	BY931593
E_EL_ovS0_25E01_R_0	BY931594
E_EL_ovS0_25E02_F_0	BY923064
E_EL_ovS0_25E02_R_0	BY931595
E_EL_ovS0_25E03_F_0	BY923065
E_EL_ovS0_25E03_R_0	BY931596
E_EL_ovS0_25E04_F_0	BY923066
E_EL_ovS0_25E04_R_0	BY931597
E_EL_ovS0_25E05_F_0	BY923067
E_EL_ovS0_25E05_R_0	BY931598
E_EL_ovS0_25E06_F_0	BY923068
E_EL_ovS0_25E06_R_0	BY931599
E_EL_ovS0_25E07_F_0	BY923069
E_EL_ovS0_25E07_R_0	BY931600
E_EL_ovS0_25E08_F_0	BY923070
E_EL_ovS0_25E08_R_0	BY931601
E_EL_ovS0_25E09_F_0	BY923071
E_EL_ovS0_25E09_R_0	BY931602

E_EL_ovS0_25E10_F_0	BY923072
E_EL_ovS0_25E10_R_0	BY931603
E_EL_ovS0_25E11_F_0	BY923073
E_EL_ovS0_25E11_R_0	BY931604
E_EL_ovS0_25E12_F_0	BY923074
E_EL_ovS0_25E12_R_0	BY931605
E_EL_ovS0_25F01_F_0	BY923075
E_EL_ovS0_25F02_F_0	BY923076
E_EL_ovS0_25F03_F_0	BY923077
E_EL_ovS0_25F04_F_0	BY923078
E_EL_ovS0_25F04_R_0	BY931606
E_EL_ovS0_25F05_F_0	BY923079
E_EL_ovS0_25F05_R_0	BY931607
E_EL_ovS0_25F06_R_0	BY931608
E_EL_ovS0_25F07_F_0	BY923080
E_EL_ovS0_25F07_R_0	BY931609
E_EL_ovS0_25F09_F_0	BY923081
E_EL_ovS0_25F09_R_0	BY931610
E_EL_ovS0_25F10_F_0	BY923082
E_EL_ovS0_25F10_R_0	BY931611
E_EL_ovS0_25F12_F_0	BY923083
E_EL_ovS0_25G01_F_0	BY923084
E_EL_ovS0_25G01_R_0	BY931612
E_EL_ovS0_25G02_F_0	BY923085
E_EL_ovS0_25G02_R_0	BY931613
E_EL_ovS0_25G03_F_0	BY923086
E_EL_ovS0_25G04_F_0	BY923087
E_EL_ovS0_25G04_R_0	BY931614
E_EL_ovS0_25G05_F_0	BY923088
E_EL_ovS0_25G05_R_0	BY931615
E_EL_ovS0_25G06_F_0	BY923089
E_EL_ovS0_25G06_R_0	BY931616
E_EL_ovS0_25G07_F_0	BY923090
E_EL_ovS0_25G07_R_0	BY931617
E_EL_ovS0_25G08_F_0	BY923091
E_EL_ovS0_25G08_R_0	BY931618
E_EL_ovS0_25G09_F_0	BY923092
E_EL_ovS0_25G09_R_0	BY931619
E_EL_ovS0_25G10_F_0	BY923093
E_EL_ovS0_25G10_R_0	BY931620
E_EL_ovS0_25G11_F_0	BY923094
E_EL_ovS0_25G11_R_0	BY931621
E_EL_ovS0_25H01_F_0	BY923095
E_EL_ovS0_25H01_R_0	BY931622
E_EL_ovS0_25H02_F_0	BY923096
E_EL_ovS0_25H02_R_0	BY931623
E_EL_ovS0_25H03_F_0	BY923097
E_EL_ovS0_25H03_R_0	BY931624
E_EL_ovS0_25H04_F_0	BY923098
E_EL_ovS0_25H04_R_0	BY931625
E_EL_ovS0_25H05_F_0	BY923099
E_EL_ovS0_25H05_R_0	BY931626
E_EL_ovS0_25H06_F_0	BY923100
E_EL_ovS0_25H07_F_0	BY923101

E_EL_ovS0_25H07_R_0	BY931627
E_EL_ovS0_25H08_F_0	BY923102
E_EL_ovS0_25H08_R_0	BY931628
E_EL_ovS0_25H09_F_0	BY923103
E_EL_ovS0_25H09_R_0	BY931629
E_EL_ovS0_25H10_F_0	BY923104
E_EL_ovS0_25H10_R_0	BY931630
E_EL_ovS0_25H11_F_0	BY923105
E_EL_ovS0_25H12_R_0	BY931631
E_EL_ovS0_26A03_R_0	BY931632
E_EL_ovS0_26A04_R_0	BY931633
E_EL_ovS0_26A05_R_0	BY931634
E_EL_ovS0_26A06_R_0	BY931635
E_EL_ovS0_26A07_F_0	BY923106
E_EL_ovS0_26A07_R_0	BY931636
E_EL_ovS0_26A08_F_0	BY923107
E_EL_ovS0_26A09_R_0	BY931637
E_EL_ovS0_26A10_R_0	BY931638
E_EL_ovS0_26A11_F_0	BY923108
E_EL_ovS0_26A11_R_0	BY931639
E_EL_ovS0_26B02_R_0	BY931640
E_EL_ovS0_26B03_R_0	BY931641
E_EL_ovS0_26B04_R_0	BY931642
E_EL_ovS0_26B05_F_0	BY923109
E_EL_ovS0_26B06_F_0	BY923110
E_EL_ovS0_26B06_R_0	BY931643
E_EL_ovS0_26B07_F_0	BY923111
E_EL_ovS0_26B07_R_0	BY931644
E_EL_ovS0_26B08_R_0	BY931645
E_EL_ovS0_26B09_R_0	BY931646
E_EL_ovS0_26B10_R_0	BY931647
E_EL_ovS0_26C01_R_0	BY931648
E_EL_ovS0_26C02_R_0	BY931649
E_EL_ovS0_26C04_F_0	BY923112
E_EL_ovS0_26C04_R_0	BY931650
E_EL_ovS0_26C05_F_0	BY923113
E_EL_ovS0_26C05_R_0	BY931651
E_EL_ovS0_26C06_F_0	BY923114
E_EL_ovS0_26C06_R_0	BY931652
E_EL_ovS0_26C08_F_0	BY923115
E_EL_ovS0_26C08_R_0	BY931653
E_EL_ovS0_26C09_F_0	BY923116
E_EL_ovS0_26C09_R_0	BY931654
E_EL_ovS0_26C10_R_0	BY931655
E_EL_ovS0_26C11_R_0	BY931656
E_EL_ovS0_26C12_R_0	BY931657
E_EL_ovS0_26D01_R_0	BY931658
E_EL_ovS0_26D02_R_0	BY931659
E_EL_ovS0_26D03_F_0	BY923117
E_EL_ovS0_26D03_R_0	BY931660
E_EL_ovS0_26D04_F_0	BY923118
E_EL_ovS0_26D04_R_0	BY931661
E_EL_ovS0_26D05_F_0	BY923119
E_EL_ovS0_26D06_F_0	BY923120

E_EL_ovS0_26D06_R_0	BY931662
E_EL_ovS0_26D07_F_0	BY923121
E_EL_ovS0_26D07_R_0	BY931663
E_EL_ovS0_26D08_F_0	BY923122
E_EL_ovS0_26D08_R_0	BY931664
E_EL_ovS0_26D09_F_0	BY923123
E_EL_ovS0_26D09_R_0	BY931665
E_EL_ovS0_26D10_F_0	BY923124
E_EL_ovS0_26D10_R_0	BY931666
E_EL_ovS0_26D12_R_0	BY931667
E_EL_ovS0_26E01_R_0	BY931668
E_EL_ovS0_26E02_R_0	BY931669
E_EL_ovS0_26E03_F_0	BY923125
E_EL_ovS0_26E03_R_0	BY931670
E_EL_ovS0_26E04_R_0	BY931671
E_EL_ovS0_26E05_F_0	BY923126
E_EL_ovS0_26E05_R_0	BY931672
E_EL_ovS0_26E06_R_0	BY931673
E_EL_ovS0_26E08_F_0	BY923127
E_EL_ovS0_26E08_R_0	BY931674
E_EL_ovS0_26E09_F_0	BY923128
E_EL_ovS0_26E09_R_0	BY931675
E_EL_ovS0_26E10_F_0	BY923129
E_EL_ovS0_26E10_R_0	BY931676
E_EL_ovS0_26E12_R_0	BY931677
E_EL_ovS0_26F03_F_0	BY923130
E_EL_ovS0_26F04_F_0	BY923131
E_EL_ovS0_26F04_R_0	BY931678
E_EL_ovS0_26F05_F_0	BY923132
E_EL_ovS0_26F05_R_0	BY931679
E_EL_ovS0_26F06_F_0	BY923133
E_EL_ovS0_26F06_R_0	BY931680
E_EL_ovS0_26F07_F_0	BY923134
E_EL_ovS0_26F07_R_0	BY931681
E_EL_ovS0_26F08_F_0	BY923135
E_EL_ovS0_26F09_R_0	BY931682
E_EL_ovS0_26F10_R_0	BY931683
E_EL_ovS0_26G02_F_0	BY923136
E_EL_ovS0_26G02_R_0	BY931684
E_EL_ovS0_26G03_F_0	BY923137
E_EL_ovS0_26G03_R_0	BY931685
E_EL_ovS0_26G04_F_0	BY923138
E_EL_ovS0_26G04_R_0	BY931686
E_EL_ovS0_26G05_F_0	BY923139
E_EL_ovS0_26G05_R_0	BY931687
E_EL_ovS0_26G06_F_0	BY923140
E_EL_ovS0_26G07_R_0	BY931688
E_EL_ovS0_26G09_R_0	BY931689
E_EL_ovS0_26G10_F_0	BY923141
E_EL_ovS0_26G10_R_0	BY931690
E_EL_ovS0_26G11_R_0	BY931691
E_EL_ovS0_26H01_R_0	BY931692
E_EL_ovS0_26H02_R_0	BY931693
E_EL_ovS0_26H03_R_0	BY931694

E_EL_ovS0_26H04_R_0	BY931695
E_EL_ovS0_26H07_R_0	BY931696
E_EL_ovS0_26H08_F_0	BY923142
E_EL_ovS0_26H09_R_0	BY931697
E_EL_ovS0_26H10_F_0	BY923143
E_EL_ovS0_26H11_F_0	BY923144
E_EL_ovS0_26H11_R_0	BY931698
E_EL_ovS0_27A01_F_0	BY923145
E_EL_ovS0_27A01_R_0	BY931910
E_EL_ovS0_27A02_F_0	BY923146
E_EL_ovS0_27A02_R_0	BY931911
E_EL_ovS0_27A03_F_0	BY923147
E_EL_ovS0_27A03_R_0	BY931912
E_EL_ovS0_27A04_F_0	BY923148
E_EL_ovS0_27A04_R_0	BY931913
E_EL_ovS0_27A05_F_0	BY923149
E_EL_ovS0_27A05_R_0	BY931914
E_EL_ovS0_27A06_F_0	BY923150
E_EL_ovS0_27A06_R_0	BY931915
E_EL_ovS0_27A07_R_0	BY931916
E_EL_ovS0_27A09_F_0	BY923151
E_EL_ovS0_27A09_R_0	BY931917
E_EL_ovS0_27A10_F_0	BY923152
E_EL_ovS0_27A10_R_0	BY931918
E_EL_ovS0_27A11_F_0	BY923153
E_EL_ovS0_27A11_R_0	BY931919
E_EL_ovS0_27A12_F_0	BY923154
E_EL_ovS0_27A12_R_0	BY931920
E_EL_ovS0_27B01_F_0	BY923155
E_EL_ovS0_27B01_R_0	BY931921
E_EL_ovS0_27B02_F_0	BY923156
E_EL_ovS0_27B02_R_0	BY931922
E_EL_ovS0_27B03_F_0	BY923157
E_EL_ovS0_27B03_R_0	BY931923
E_EL_ovS0_27B04_F_0	BY923158
E_EL_ovS0_27B04_R_0	BY931924
E_EL_ovS0_27B05_F_0	BY923159
E_EL_ovS0_27B05_R_0	BY931925
E_EL_ovS0_27B06_F_0	BY923160
E_EL_ovS0_27B06_R_0	BY931926
E_EL_ovS0_27B07_F_0	BY923161
E_EL_ovS0_27B08_F_0	BY923162
E_EL_ovS0_27B08_R_0	BY931927
E_EL_ovS0_27B09_F_0	BY923163
E_EL_ovS0_27B10_F_0	BY923164
E_EL_ovS0_27B10_R_0	BY931928
E_EL_ovS0_27B11_F_0	BY923165
E_EL_ovS0_27B11_R_0	BY931929
E_EL_ovS0_27B12_F_0	BY923166
E_EL_ovS0_27C01_F_0	BY923167
E_EL_ovS0_27C01_R_0	BY931930
E_EL_ovS0_27C02_F_0	BY923168
E_EL_ovS0_27C02_R_0	BY931931
E_EL_ovS0_27C03_F_0	BY923169

E_EL_ovS0_27C03_R_0	BY931932
E_EL_ovS0_27C04_F_0	BY923170
E_EL_ovS0_27C06_F_0	BY923171
E_EL_ovS0_27C06_R_0	BY931933
E_EL_ovS0_27C07_F_0	BY923172
E_EL_ovS0_27C07_R_0	BY931934
E_EL_ovS0_27C08_F_0	BY923173
E_EL_ovS0_27C08_R_0	BY931935
E_EL_ovS0_27C09_F_0	BY923174
E_EL_ovS0_27C10_R_0	BY931936
E_EL_ovS0_27C11_F_0	BY923175
E_EL_ovS0_27C11_R_0	BY931937
E_EL_ovS0_27C12_F_0	BY923176
E_EL_ovS0_27C12_R_0	BY931938
E_EL_ovS0_27D01_F_0	BY923177
E_EL_ovS0_27D01_R_0	BY931939
E_EL_ovS0_27D02_F_0	BY923178
E_EL_ovS0_27D02_R_0	BY931940
E_EL_ovS0_27D03_F_0	BY923179
E_EL_ovS0_27D03_R_0	BY931941
E_EL_ovS0_27D04_F_0	BY923180
E_EL_ovS0_27D04_R_0	BY931942
E_EL_ovS0_27D05_F_0	BY923181
E_EL_ovS0_27D05_R_0	BY931943
E_EL_ovS0_27D07_F_0	BY923182
E_EL_ovS0_27D07_R_0	BY931944
E_EL_ovS0_27D08_F_0	BY923183
E_EL_ovS0_27D09_F_0	BY923184
E_EL_ovS0_27D09_R_0	BY931945
E_EL_ovS0_27D10_F_0	BY923185
E_EL_ovS0_27D10_R_0	BY931946
E_EL_ovS0_27D11_F_0	BY923186
E_EL_ovS0_27D12_F_0	BY923187
E_EL_ovS0_27D12_R_0	BY931947
E_EL_ovS0_27E01_R_0	BY931948
E_EL_ovS0_27E02_R_0	BY931949
E_EL_ovS0_27E03_F_0	BY923188
E_EL_ovS0_27E04_F_0	BY923189
E_EL_ovS0_27E04_R_0	BY931950
E_EL_ovS0_27E06_F_0	BY923190
E_EL_ovS0_27E06_R_0	BY931951
E_EL_ovS0_27E07_F_0	BY923191
E_EL_ovS0_27E07_R_0	BY931952
E_EL_ovS0_27E08_F_0	BY923192
E_EL_ovS0_27E09_F_0	BY923193
E_EL_ovS0_27E09_R_0	BY931953
E_EL_ovS0_27E10_F_0	BY923194
E_EL_ovS0_27E10_R_0	BY931954
E_EL_ovS0_27E11_F_0	BY923195
E_EL_ovS0_27E11_R_0	BY931955
E_EL_ovS0_27E12_F_0	BY923196
E_EL_ovS0_27E12_R_0	BY931956
E_EL_ovS0_27F01_F_0	BY923197
E_EL_ovS0_27F01_R_0	BY931957

E_EL_ovS0_27F02_F_0	BY923198
E_EL_ovS0_27F02_R_0	BY931958
E_EL_ovS0_27F03_F_0	BY923199
E_EL_ovS0_27F03_R_0	BY931959
E_EL_ovS0_27F04_F_0	BY923200
E_EL_ovS0_27F04_R_0	BY931960
E_EL_ovS0_27F06_F_0	BY923201
E_EL_ovS0_27F07_F_0	BY923202
E_EL_ovS0_27F07_R_0	BY931961
E_EL_ovS0_27F08_F_0	BY923203
E_EL_ovS0_27F08_R_0	BY931962
E_EL_ovS0_27F09_F_0	BY923204
E_EL_ovS0_27F09_R_0	BY931963
E_EL_ovS0_27F10_F_0	BY923205
E_EL_ovS0_27F10_R_0	BY931964
E_EL_ovS0_27F11_F_0	BY923206
E_EL_ovS0_27F12_F_0	BY923207
E_EL_ovS0_27F12_R_0	BY931965
E_EL_ovS0_27G01_F_0	BY923208
E_EL_ovS0_27G01_R_0	BY931966
E_EL_ovS0_27G02_R_0	BY931967
E_EL_ovS0_27G03_F_0	BY923209
E_EL_ovS0_27G03_R_0	BY931968
E_EL_ovS0_27G04_F_0	BY923210
E_EL_ovS0_27G04_R_0	BY931969
E_EL_ovS0_27G05_F_0	BY923211
E_EL_ovS0_27G05_R_0	BY931970
E_EL_ovS0_27G06_F_0	BY923212
E_EL_ovS0_27G06_R_0	BY931971
E_EL_ovS0_27G07_F_0	BY923213
E_EL_ovS0_27G07_R_0	BY931972
E_EL_ovS0_27G08_F_0	BY923214
E_EL_ovS0_27G08_R_0	BY931973
E_EL_ovS0_27G09_F_0	BY923215
E_EL_ovS0_27G09_R_0	BY931974
E_EL_ovS0_27G10_F_0	BY923216
E_EL_ovS0_27G10_R_0	BY931975
E_EL_ovS0_27G11_F_0	BY923217
E_EL_ovS0_27G11_R_0	BY931976
E_EL_ovS0_27G12_F_0	BY923218
E_EL_ovS0_27G12_R_0	BY931977
E_EL_ovS0_27H01_F_0	BY923219
E_EL_ovS0_27H01_R_0	BY931978
E_EL_ovS0_27H02_F_0	BY923220
E_EL_ovS0_27H02_R_0	BY931979
E_EL_ovS0_27H03_F_0	BY923221
E_EL_ovS0_27H03_R_0	BY931980
E_EL_ovS0_27H04_F_0	BY923222
E_EL_ovS0_27H04_R_0	BY931981
E_EL_ovS0_27H05_F_0	BY923223
E_EL_ovS0_27H05_R_0	BY931982
E_EL_ovS0_27H06_F_0	BY923224
E_EL_ovS0_27H06_R_0	BY931983
E_EL_ovS0_27H07_F_0	BY923225

E_EL_ovS0_27H07_R_0	BY931984
E_EL_ovS0_27H08_F_0	BY923226
E_EL_ovS0_27H08_R_0	BY931985
E_EL_ovS0_27H09_R_0	BY931986
E_EL_ovS0_27H10_F_0	BY923227
E_EL_ovS0_27H10_R_0	BY931987
E_EL_ovS0_27H11_F_0	BY923228
E_EL_ovS0_27H11_R_0	BY931988
E_EL_ovS0_27H12_F_0	BY923229
E_EL_ovS0_27H12_R_0	BY931989
E_EL_ovS0_28A02_F_0	BY923230
E_EL_ovS0_28A02_R_0	BY931699
E_EL_ovS0_28A03_F_0	BY923231
E_EL_ovS0_28A04_F_0	BY923232
E_EL_ovS0_28A05_F_0	BY923233
E_EL_ovS0_28A05_R_0	BY931700
E_EL_ovS0_28A06_F_0	BY923234
E_EL_ovS0_28A07_F_0	BY923235
E_EL_ovS0_28A08_F_0	BY923236
E_EL_ovS0_28A09_F_0	BY923237
E_EL_ovS0_28A09_R_0	BY931701
E_EL_ovS0_28A10_F_0	BY923238
E_EL_ovS0_28A12_F_0	BY923239
E_EL_ovS0_28A12_R_0	BY931702
E_EL_ovS0_28B01_F_0	BY923240
E_EL_ovS0_28B01_R_0	BY931703
E_EL_ovS0_28B02_R_0	BY931704
E_EL_ovS0_28B03_F_0	BY923241
E_EL_ovS0_28B03_R_0	BY931705
E_EL_ovS0_28B04_F_0	BY923242
E_EL_ovS0_28B04_R_0	BY931706
E_EL_ovS0_28B05_F_0	BY923243
E_EL_ovS0_28B06_F_0	BY923244
E_EL_ovS0_28B06_R_0	BY931707
E_EL_ovS0_28B07_F_0	BY923245
E_EL_ovS0_28B08_F_0	BY923246
E_EL_ovS0_28B09_F_0	BY923247
E_EL_ovS0_28B10_F_0	BY923248
E_EL_ovS0_28B10_R_0	BY931708
E_EL_ovS0_28B11_F_0	BY923249
E_EL_ovS0_28B12_F_0	BY923250
E_EL_ovS0_28C02_F_0	BY923251
E_EL_ovS0_28C02_R_0	BY931709
E_EL_ovS0_28C03_F_0	BY923252
E_EL_ovS0_28C03_R_0	BY931710
E_EL_ovS0_28C04_F_0	BY923253
E_EL_ovS0_28C04_R_0	BY931711
E_EL_ovS0_28C05_F_0	BY923254
E_EL_ovS0_28C05_R_0	BY931712
E_EL_ovS0_28C06_F_0	BY923255
E_EL_ovS0_28C06_R_0	BY931713
E_EL_ovS0_28C07_F_0	BY923256
E_EL_ovS0_28C07_R_0	BY931714
E_EL_ovS0_28C08_F_0	BY923257

E_EL_ovS0_28C08_R_0	BY931715
E_EL_ovS0_28C09_F_0	BY923258
E_EL_ovS0_28C10_F_0	BY923259
E_EL_ovS0_28C10_R_0	BY931716
E_EL_ovS0_28C11_R_0	BY931717
E_EL_ovS0_28C12_R_0	BY931718
E_EL_ovS0_28D01_F_0	BY923260
E_EL_ovS0_28D01_R_0	BY931719
E_EL_ovS0_28D02_F_0	BY923261
E_EL_ovS0_28D02_R_0	BY931720
E_EL_ovS0_28D03_F_0	BY923262
E_EL_ovS0_28D03_R_0	BY931721
E_EL_ovS0_28D04_F_0	BY923263
E_EL_ovS0_28D04_R_0	BY931722
E_EL_ovS0_28D05_F_0	BY923264
E_EL_ovS0_28D05_R_0	BY931723
E_EL_ovS0_28D06_F_0	BY923265
E_EL_ovS0_28D06_R_0	BY931724
E_EL_ovS0_28D07_F_0	BY923266
E_EL_ovS0_28D07_R_0	BY931725
E_EL_ovS0_28D08_F_0	BY923267
E_EL_ovS0_28D08_R_0	BY931726
E_EL_ovS0_28D09_F_0	BY923268
E_EL_ovS0_28D09_R_0	BY931727
E_EL_ovS0_28D10_F_0	BY923269
E_EL_ovS0_28D11_F_0	BY923270
E_EL_ovS0_28D11_R_0	BY931728
E_EL_ovS0_28D12_F_0	BY923271
E_EL_ovS0_28D12_R_0	BY931729
E_EL_ovS0_28E01_F_0	BY923272
E_EL_ovS0_28E01_R_0	BY931730
E_EL_ovS0_28E02_R_0	BY931731
E_EL_ovS0_28E04_F_0	BY923273
E_EL_ovS0_28E04_R_0	BY931732
E_EL_ovS0_28E05_F_0	BY923274
E_EL_ovS0_28E05_R_0	BY931733
E_EL_ovS0_28E06_F_0	BY923275
E_EL_ovS0_28E06_R_0	BY931734
E_EL_ovS0_28E07_R_0	BY931735
E_EL_ovS0_28E08_F_0	BY923276
E_EL_ovS0_28E08_R_0	BY931736
E_EL_ovS0_28E09_F_0	BY923277
E_EL_ovS0_28E09_R_0	BY931737
E_EL_ovS0_28E10_R_0	BY931738
E_EL_ovS0_28E11_F_0	BY923278
E_EL_ovS0_28E11_R_0	BY931739
E_EL_ovS0_28E12_F_0	BY923279
E_EL_ovS0_28E12_R_0	BY931740
E_EL_ovS0_28F01_F_0	BY923280
E_EL_ovS0_28F02_F_0	BY923281
E_EL_ovS0_28F03_F_0	BY923282
E_EL_ovS0_28F04_F_0	BY923283
E_EL_ovS0_28F04_R_0	BY931741
E_EL_ovS0_28F05_F_0	BY923284

E_EL_ovS0_28F05_R_0	BY931742
E_EL_ovS0_28F06_F_0	BY923285
E_EL_ovS0_28F06_R_0	BY931743
E_EL_ovS0_28F07_F_0	BY923286
E_EL_ovS0_28F08_F_0	BY923287
E_EL_ovS0_28F08_R_0	BY931744
E_EL_ovS0_28F09_F_0	BY923288
E_EL_ovS0_28F09_R_0	BY931745
E_EL_ovS0_28F10_R_0	BY931746
E_EL_ovS0_28F12_F_0	BY923289
E_EL_ovS0_28G02_F_0	BY923290
E_EL_ovS0_28G02_R_0	BY931747
E_EL_ovS0_28G03_F_0	BY923291
E_EL_ovS0_28G03_R_0	BY931748
E_EL_ovS0_28G04_F_0	BY923292
E_EL_ovS0_28G04_R_0	BY931749
E_EL_ovS0_28G05_F_0	BY923293
E_EL_ovS0_28G05_R_0	BY931750
E_EL_ovS0_28G06_F_0	BY923294
E_EL_ovS0_28G06_R_0	BY931751
E_EL_ovS0_28G07_F_0	BY923295
E_EL_ovS0_28G07_R_0	BY931752
E_EL_ovS0_28G08_F_0	BY923296
E_EL_ovS0_28G08_R_0	BY931753
E_EL_ovS0_28G09_F_0	BY923297
E_EL_ovS0_28G09_R_0	BY931754
E_EL_ovS0_28G10_F_0	BY923298
E_EL_ovS0_28G10_R_0	BY931755
E_EL_ovS0_28G11_F_0	BY923299
E_EL_ovS0_28G11_R_0	BY931756
E_EL_ovS0_28G12_F_0	BY923300
E_EL_ovS0_28G12_R_0	BY931757
E_EL_ovS0_28H01_F_0	BY923301
E_EL_ovS0_28H01_R_0	BY931758
E_EL_ovS0_28H03_R_0	BY931759
E_EL_ovS0_28H05_F_0	BY923302
E_EL_ovS0_28H05_R_0	BY931760
E_EL_ovS0_28H06_F_0	BY923303
E_EL_ovS0_28H06_R_0	BY931761
E_EL_ovS0_28H07_F_0	BY923304
E_EL_ovS0_28H07_R_0	BY931762
E_EL_ovS0_28H08_R_0	BY931763
E_EL_ovS0_28H09_F_0	BY923305
E_EL_ovS0_28H09_R_0	BY931764
E_EL_ovS0_28H10_F_0	BY923306
E_EL_ovS0_28H10_R_0	BY931765
E_EL_ovS0_28H11_F_0	BY923307
E_EL_ovS0_28H11_R_0	BY931766
E_EL_ovS0_28H12_F_0	BY923308
E_EL_ovS0_28H12_R_0	BY931767
E_EL_ovS0_29A01_F_0	BY923382
E_EL_ovS0_29A01_R_0	BY931768
E_EL_ovS0_29A02_F_0	BY923383
E_EL_ovS0_29A02_R_0	BY931769

E_EL_ovS0_29A04_F_0	BY923384
E_EL_ovS0_29A04_R_0	BY931770
E_EL_ovS0_29A05_F_0	BY923385
E_EL_ovS0_29A05_R_0	BY931771
E_EL_ovS0_29A07_F_0	BY923386
E_EL_ovS0_29A08_F_0	BY923387
E_EL_ovS0_29A08_R_0	BY931772
E_EL_ovS0_29A09_F_0	BY923388
E_EL_ovS0_29A09_R_0	BY931773
E_EL_ovS0_29A10_F_0	BY923389
E_EL_ovS0_29A10_R_0	BY931774
E_EL_ovS0_29A11_F_0	BY923390
E_EL_ovS0_29A11_R_0	BY931775
E_EL_ovS0_29A12_F_0	BY923391
E_EL_ovS0_29A12_R_0	BY931776
E_EL_ovS0_29B01_F_0	BY923392
E_EL_ovS0_29B01_R_0	BY931777
E_EL_ovS0_29B02_F_0	BY923393
E_EL_ovS0_29B03_F_0	BY923394
E_EL_ovS0_29B03_R_0	BY931778
E_EL_ovS0_29B04_F_0	BY923395
E_EL_ovS0_29B04_R_0	BY931779
E_EL_ovS0_29B05_F_0	BY923396
E_EL_ovS0_29B05_R_0	BY931780
E_EL_ovS0_29B06_F_0	BY923397
E_EL_ovS0_29B06_R_0	BY931781
E_EL_ovS0_29B07_F_0	BY923398
E_EL_ovS0_29B07_R_0	BY931782
E_EL_ovS0_29B08_F_0	BY923399
E_EL_ovS0_29B08_R_0	BY931783
E_EL_ovS0_29B09_F_0	BY923400
E_EL_ovS0_29B09_R_0	BY931784
E_EL_ovS0_29B10_F_0	BY923401
E_EL_ovS0_29B10_R_0	BY931785
E_EL_ovS0_29B11_F_0	BY923402
E_EL_ovS0_29B12_F_0	BY923403
E_EL_ovS0_29C01_F_0	BY923404
E_EL_ovS0_29C01_R_0	BY931786
E_EL_ovS0_29C02_F_0	BY923405
E_EL_ovS0_29C02_R_0	BY931787
E_EL_ovS0_29C03_F_0	BY923406
E_EL_ovS0_29C03_R_0	BY931788
E_EL_ovS0_29C04_F_0	BY923407
E_EL_ovS0_29C04_R_0	BY931789
E_EL_ovS0_29C05_F_0	BY923408
E_EL_ovS0_29C05_R_0	BY931790
E_EL_ovS0_29C06_F_0	BY923409
E_EL_ovS0_29C06_R_0	BY931791
E_EL_ovS0_29C09_F_0	BY923410
E_EL_ovS0_29C09_R_0	BY931792
E_EL_ovS0_29C10_F_0	BY923411
E_EL_ovS0_29C10_R_0	BY931793
E_EL_ovS0_29C11_F_0	BY923412
E_EL_ovS0_29C11_R_0	BY931794

E_EL_ovS0_29D01_F_0	BY923413
E_EL_ovS0_29D01_R_0	BY931795
E_EL_ovS0_29D02_F_0	BY923414
E_EL_ovS0_29D02_R_0	BY931796
E_EL_ovS0_29D03_F_0	BY923415
E_EL_ovS0_29D03_R_0	BY931797
E_EL_ovS0_29D05_F_0	BY923416
E_EL_ovS0_29D05_R_0	BY931798
E_EL_ovS0_29D06_F_0	BY923417
E_EL_ovS0_29D06_R_0	BY931799
E_EL_ovS0_29D07_F_0	BY923418
E_EL_ovS0_29D07_R_0	BY931800
E_EL_ovS0_29D08_F_0	BY923419
E_EL_ovS0_29D08_R_0	BY931801
E_EL_ovS0_29D10_F_0	BY923420
E_EL_ovS0_29D10_R_0	BY931802
E_EL_ovS0_29D11_F_0	BY923421
E_EL_ovS0_29D11_R_0	BY931803
E_EL_ovS0_29D12_F_0	BY923422
E_EL_ovS0_29D12_R_0	BY931804
E_EL_ovS0_29E01_F_0	BY923423
E_EL_ovS0_29E01_R_0	BY931805
E_EL_ovS0_29E02_F_0	BY923424
E_EL_ovS0_29E02_R_0	BY931806
E_EL_ovS0_29E03_F_0	BY923425
E_EL_ovS0_29E03_R_0	BY931807
E_EL_ovS0_29E04_F_0	BY923426
E_EL_ovS0_29E05_R_0	BY931808
E_EL_ovS0_29E06_F_0	BY923427
E_EL_ovS0_29E06_R_0	BY931809
E_EL_ovS0_29E07_F_0	BY923428
E_EL_ovS0_29E07_R_0	BY931810
E_EL_ovS0_29E08_F_0	BY923429
E_EL_ovS0_29E08_R_0	BY931811
E_EL_ovS0_29E09_F_0	BY923430
E_EL_ovS0_29E09_R_0	BY931812
E_EL_ovS0_29E10_R_0	BY931813
E_EL_ovS0_29E11_F_0	BY923431
E_EL_ovS0_29E11_R_0	BY931814
E_EL_ovS0_29E12_F_0	BY923432
E_EL_ovS0_29E12_R_0	BY931815
E_EL_ovS0_29F01_F_0	BY923433
E_EL_ovS0_29F02_F_0	BY923434
E_EL_ovS0_29F04_F_0	BY923435
E_EL_ovS0_29F04_R_0	BY931816
E_EL_ovS0_29F05_F_0	BY923436
E_EL_ovS0_29F06_F_0	BY923437
E_EL_ovS0_29F06_R_0	BY931817
E_EL_ovS0_29F07_F_0	BY923438
E_EL_ovS0_29F08_R_0	BY931818
E_EL_ovS0_29F09_F_0	BY923439
E_EL_ovS0_29F09_R_0	BY931819
E_EL_ovS0_29F10_F_0	BY923440
E_EL_ovS0_29F10_R_0	BY931820

E_EL_ovS0_29F11_F_0	BY923441
E_EL_ovS0_29G01_F_0	BY923442
E_EL_ovS0_29G01_R_0	BY931821
E_EL_ovS0_29G02_F_0	BY923443
E_EL_ovS0_29G02_R_0	BY931822
E_EL_ovS0_29G04_F_0	BY923444
E_EL_ovS0_29G05_F_0	BY923445
E_EL_ovS0_29G05_R_0	BY931823
E_EL_ovS0_29G06_F_0	BY923446
E_EL_ovS0_29G06_R_0	BY931824
E_EL_ovS0_29G07_F_0	BY923447
E_EL_ovS0_29G07_R_0	BY931825
E_EL_ovS0_29G08_F_0	BY923448
E_EL_ovS0_29G08_R_0	BY931826
E_EL_ovS0_29G09_F_0	BY923449
E_EL_ovS0_29G09_R_0	BY931827
E_EL_ovS0_29G10_F_0	BY923450
E_EL_ovS0_29G10_R_0	BY931828
E_EL_ovS0_29G11_F_0	BY923451
E_EL_ovS0_29G11_R_0	BY931829
E_EL_ovS0_29G12_F_0	BY923452
E_EL_ovS0_29G12_R_0	BY931830
E_EL_ovS0_29H01_F_0	BY923453
E_EL_ovS0_29H01_R_0	BY931831
E_EL_ovS0_29H02_F_0	BY923454
E_EL_ovS0_29H02_R_0	BY931832
E_EL_ovS0_29H03_F_0	BY923455
E_EL_ovS0_29H03_R_0	BY931833
E_EL_ovS0_29H04_F_0	BY923456
E_EL_ovS0_29H04_R_0	BY931834
E_EL_ovS0_29H05_F_0	BY923457
E_EL_ovS0_29H06_F_0	BY923458
E_EL_ovS0_29H06_R_0	BY931835
E_EL_ovS0_29H07_F_0	BY923459
E_EL_ovS0_29H07_R_0	BY931836
E_EL_ovS0_29H09_F_0	BY923460
E_EL_ovS0_29H09_R_0	BY931837
E_EL_ovS0_29H10_F_0	BY923461
E_EL_ovS0_29H10_R_0	BY931838
E_EL_ovS0_29H11_F_0	BY923462
E_EL_ovS0_29H11_R_0	BY931839
E_EL_ovS0_29H12_F_0	BY923463
E_EL_ovS0_29H12_R_0	BY931840
E_EL_ovS0_30A01_F_0	BY923309
E_EL_ovS0_30A01_R_0	BY931990
E_EL_ovS0_30A02_R_0	BY931991
E_EL_ovS0_30A03_R_0	BY931992
E_EL_ovS0_30A04_F_0	BY923310
E_EL_ovS0_30A04_R_0	BY931993
E_EL_ovS0_30A05_F_0	BY923311
E_EL_ovS0_30A05_R_0	BY931994
E_EL_ovS0_30A06_F_0	BY923312
E_EL_ovS0_30A06_R_0	BY931995
E_EL_ovS0_30A07_F_0	BY923313

E_EL_ovS0_30A07_R_0	BY931996
E_EL_ovS0_30A08_F_0	BY923314
E_EL_ovS0_30A08_R_0	BY931997
E_EL_ovS0_30A09_F_0	BY923315
E_EL_ovS0_30A09_R_0	BY931998
E_EL_ovS0_30A10_R_0	BY931999
E_EL_ovS0_30A11_F_0	BY923316
E_EL_ovS0_30A11_R_0	BY932000
E_EL_ovS0_30B02_F_0	BY923317
E_EL_ovS0_30B02_R_0	BY932001
E_EL_ovS0_30B03_F_0	BY923318
E_EL_ovS0_30B03_R_0	BY932002
E_EL_ovS0_30B04_F_0	BY923319
E_EL_ovS0_30B04_R_0	BY932003
E_EL_ovS0_30B06_F_0	BY923320
E_EL_ovS0_30B06_R_0	BY932004
E_EL_ovS0_30B07_F_0	BY923321
E_EL_ovS0_30B07_R_0	BY932005
E_EL_ovS0_30B08_F_0	BY923322
E_EL_ovS0_30B08_R_0	BY932006
E_EL_ovS0_30B09_R_0	BY932007
E_EL_ovS0_30B11_F_0	BY923323
E_EL_ovS0_30B11_R_0	BY932008
E_EL_ovS0_30B12_F_0	BY923324
E_EL_ovS0_30B12_R_0	BY932009
E_EL_ovS0_30C01_R_0	BY932010
E_EL_ovS0_30C02_F_0	BY923325
E_EL_ovS0_30C02_R_0	BY932011
E_EL_ovS0_30C03_F_0	BY923326
E_EL_ovS0_30C04_R_0	BY932012
E_EL_ovS0_30C05_F_0	BY923327
E_EL_ovS0_30C05_R_0	BY932013
E_EL_ovS0_30C06_F_0	BY923328
E_EL_ovS0_30C06_R_0	BY932014
E_EL_ovS0_30C07_F_0	BY923329
E_EL_ovS0_30C07_R_0	BY932015
E_EL_ovS0_30C08_F_0	BY923330
E_EL_ovS0_30C08_R_0	BY932016
E_EL_ovS0_30C09_F_0	BY923331
E_EL_ovS0_30C09_R_0	BY932017
E_EL_ovS0_30C10_F_0	BY923332
E_EL_ovS0_30C10_R_0	BY932018
E_EL_ovS0_30C11_F_0	BY923333
E_EL_ovS0_30C11_R_0	BY932019
E_EL_ovS0_30C12_F_0	BY923334
E_EL_ovS0_30C12_R_0	BY932020
E_EL_ovS0_30D01_F_0	BY923335
E_EL_ovS0_30D01_R_0	BY932021
E_EL_ovS0_30D02_R_0	BY932022
E_EL_ovS0_30D04_F_0	BY923336
E_EL_ovS0_30D04_R_0	BY932023
E_EL_ovS0_30D05_F_0	BY923337
E_EL_ovS0_30D05_R_0	BY932024
E_EL_ovS0_30D07_F_0	BY923338

E_EL_ovS0_30D07_R_0	BY932025
E_EL_ovS0_30D09_F_0	BY923339
E_EL_ovS0_30D09_R_0	BY932026
E_EL_ovS0_30D10_F_0	BY923340
E_EL_ovS0_30D10_R_0	BY932027
E_EL_ovS0_30D11_F_0	BY923341
E_EL_ovS0_30D11_R_0	BY932028
E_EL_ovS0_30D12_F_0	BY923342
E_EL_ovS0_30D12_R_0	BY932029
E_EL_ovS0_30E01_R_0	BY932030
E_EL_ovS0_30E02_F_0	BY923343
E_EL_ovS0_30E02_R_0	BY932031
E_EL_ovS0_30E03_F_0	BY923344
E_EL_ovS0_30E03_R_0	BY932032
E_EL_ovS0_30E04_F_0	BY923345
E_EL_ovS0_30E04_R_0	BY932033
E_EL_ovS0_30E05_F_0	BY923346
E_EL_ovS0_30E05_R_0	BY932034
E_EL_ovS0_30E06_F_0	BY923347
E_EL_ovS0_30E06_R_0	BY932035
E_EL_ovS0_30E07_F_0	BY923348
E_EL_ovS0_30E07_R_0	BY932036
E_EL_ovS0_30E08_F_0	BY923349
E_EL_ovS0_30E08_R_0	BY932037
E_EL_ovS0_30E09_F_0	BY923350
E_EL_ovS0_30E09_R_0	BY932038
E_EL_ovS0_30E10_F_0	BY923351
E_EL_ovS0_30E10_R_0	BY932039
E_EL_ovS0_30E11_F_0	BY923352
E_EL_ovS0_30E11_R_0	BY932040
E_EL_ovS0_30E12_R_0	BY932041
E_EL_ovS0_30F01_F_0	BY923353
E_EL_ovS0_30F01_R_0	BY932042
E_EL_ovS0_30F02_F_0	BY923354
E_EL_ovS0_30F02_R_0	BY932043
E_EL_ovS0_30F03_F_0	BY923355
E_EL_ovS0_30F03_R_0	BY932044
E_EL_ovS0_30F04_F_0	BY923356
E_EL_ovS0_30F04_R_0	BY932045
E_EL_ovS0_30F05_F_0	BY923357
E_EL_ovS0_30F05_R_0	BY932046
E_EL_ovS0_30F06_F_0	BY923358
E_EL_ovS0_30F06_R_0	BY932047
E_EL_ovS0_30F07_F_0	BY923359
E_EL_ovS0_30F07_R_0	BY932048
E_EL_ovS0_30F08_F_0	BY923360
E_EL_ovS0_30F08_R_0	BY932049
E_EL_ovS0_30F09_R_0	BY932050
E_EL_ovS0_30F10_F_0	BY923361
E_EL_ovS0_30F10_R_0	BY932051
E_EL_ovS0_30F12_F_0	BY923362
E_EL_ovS0_30F12_R_0	BY932052
E_EL_ovS0_30G01_F_0	BY923363
E_EL_ovS0_30G01_R_0	BY932053

E_EL_ovS0_30G02_F_0	BY923364
E_EL_ovS0_30G02_R_0	BY932054
E_EL_ovS0_30G03_F_0	BY923365
E_EL_ovS0_30G03_R_0	BY932055
E_EL_ovS0_30G04_F_0	BY923366
E_EL_ovS0_30G04_R_0	BY932056
E_EL_ovS0_30G05_F_0	BY923367
E_EL_ovS0_30G05_R_0	BY932057
E_EL_ovS0_30G06_F_0	BY923368
E_EL_ovS0_30G06_R_0	BY932058
E_EL_ovS0_30G07_R_0	BY932059
E_EL_ovS0_30G09_F_0	BY923369
E_EL_ovS0_30G09_R_0	BY932060
E_EL_ovS0_30G10_F_0	BY923370
E_EL_ovS0_30G10_R_0	BY932061
E_EL_ovS0_30G11_F_0	BY923371
E_EL_ovS0_30G11_R_0	BY932062
E_EL_ovS0_30G12_F_0	BY923372
E_EL_ovS0_30G12_R_0	BY932063
E_EL_ovS0_30H01_F_0	BY923373
E_EL_ovS0_30H01_R_0	BY932064
E_EL_ovS0_30H02_F_0	BY923374
E_EL_ovS0_30H02_R_0	BY932065
E_EL_ovS0_30H03_F_0	BY923375
E_EL_ovS0_30H03_R_0	BY932066
E_EL_ovS0_30H04_F_0	BY923376
E_EL_ovS0_30H04_R_0	BY932067
E_EL_ovS0_30H05_R_0	BY932068
E_EL_ovS0_30H07_F_0	BY923377
E_EL_ovS0_30H07_R_0	BY932069
E_EL_ovS0_30H08_F_0	BY923378
E_EL_ovS0_30H08_R_0	BY932070
E_EL_ovS0_30H09_F_0	BY923379
E_EL_ovS0_30H09_R_0	BY932071
E_EL_ovS0_30H10_F_0	BY923380
E_EL_ovS0_30H10_R_0	BY932072
E_EL_ovS0_30H11_F_0	BY923381
E_EL_ovS0_30H11_R_0	BY932073
E_EL_ovS0_31A01_F_0	BY923464
E_EL_ovS0_31A01_R_0	BY931841
E_EL_ovS0_31A02_F_0	BY923465
E_EL_ovS0_31A03_F_0	BY923466
E_EL_ovS0_31A04_F_0	BY923467
E_EL_ovS0_31A04_R_0	BY931842
E_EL_ovS0_31A05_F_0	BY923468
E_EL_ovS0_31A05_R_0	BY931843
E_EL_ovS0_31A06_F_0	BY923469
E_EL_ovS0_31A06_R_0	BY931844
E_EL_ovS0_31A07_F_0	BY923470
E_EL_ovS0_31A07_R_0	BY931845
E_EL_ovS0_31A10_F_0	BY923471
E_EL_ovS0_31A10_R_0	BY931846
E_EL_ovS0_31A11_F_0	BY923472
E_EL_ovS0_31A11_R_0	BY931847

E_EL_ovS0_31A12_F_0	BY923473
E_EL_ovS0_31B01_F_0	BY923474
E_EL_ovS0_31B01_R_0	BY931848
E_EL_ovS0_31B02_F_0	BY923475
E_EL_ovS0_31B02_R_0	BY931849
E_EL_ovS0_31B05_F_0	BY923476
E_EL_ovS0_31B05_R_0	BY931850
E_EL_ovS0_31B06_F_0	BY923477
E_EL_ovS0_31B06_R_0	BY931851
E_EL_ovS0_31B07_F_0	BY923478
E_EL_ovS0_31B07_R_0	BY931852
E_EL_ovS0_31B08_F_0	BY923479
E_EL_ovS0_31B08_R_0	BY931853
E_EL_ovS0_31B09_F_0	BY923480
E_EL_ovS0_31B09_R_0	BY931854
E_EL_ovS0_31B10_F_0	BY923481
E_EL_ovS0_31B10_R_0	BY931855
E_EL_ovS0_31B11_F_0	BY923482
E_EL_ovS0_31B12_F_0	BY923483
E_EL_ovS0_31C02_F_0	BY923484
E_EL_ovS0_31C02_R_0	BY931856
E_EL_ovS0_31C03_F_0	BY923485
E_EL_ovS0_31C03_R_0	BY931857
E_EL_ovS0_31C04_F_0	BY923486
E_EL_ovS0_31C04_R_0	BY931858
E_EL_ovS0_31C05_F_0	BY923487
E_EL_ovS0_31C05_R_0	BY931859
E_EL_ovS0_31C06_F_0	BY923488
E_EL_ovS0_31C06_R_0	BY931860
E_EL_ovS0_31C08_F_0	BY923489
E_EL_ovS0_31C08_R_0	BY931861
E_EL_ovS0_31C09_F_0	BY923490
E_EL_ovS0_31C09_R_0	BY931862
E_EL_ovS0_31C10_F_0	BY923491
E_EL_ovS0_31C10_R_0	BY931863
E_EL_ovS0_31C11_F_0	BY923492
E_EL_ovS0_31C11_R_0	BY931864
E_EL_ovS0_31C12_F_0	BY923493
E_EL_ovS0_31C12_R_0	BY931865
E_EL_ovS0_31D01_F_0	BY923494
E_EL_ovS0_31D01_R_0	BY931866
E_EL_ovS0_31D02_F_0	BY923495
E_EL_ovS0_31D02_R_0	BY931867
E_EL_ovS0_31D03_F_0	BY923496
E_EL_ovS0_31D04_F_0	BY923497
E_EL_ovS0_31D04_R_0	BY931868
E_EL_ovS0_31D05_F_0	BY923498
E_EL_ovS0_31D05_R_0	BY931869
E_EL_ovS0_31D07_F_0	BY923499
E_EL_ovS0_31D08_F_0	BY923500
E_EL_ovS0_31D08_R_0	BY931870
E_EL_ovS0_31D09_F_0	BY923501
E_EL_ovS0_31D09_R_0	BY931871
E_EL_ovS0_31D10_F_0	BY923502

E_EL_ovS0_31D10_R_0	BY931872
E_EL_ovS0_31D11_F_0	BY923503
E_EL_ovS0_31D11_R_0	BY931873
E_EL_ovS0_31D12_F_0	BY923504
E_EL_ovS0_31D12_R_0	BY931874
E_EL_ovS0_31E01_F_0	BY923505
E_EL_ovS0_31E01_R_0	BY931875
E_EL_ovS0_31E02_F_0	BY923506
E_EL_ovS0_31E02_R_0	BY931876
E_EL_ovS0_31E03_R_0	BY931877
E_EL_ovS0_31E04_F_0	BY923507
E_EL_ovS0_31E04_R_0	BY931878
E_EL_ovS0_31E05_F_0	BY923508
E_EL_ovS0_31E05_R_0	BY931879
E_EL_ovS0_31E06_F_0	BY923509
E_EL_ovS0_31E06_R_0	BY931880
E_EL_ovS0_31E07_F_0	BY923510
E_EL_ovS0_31E07_R_0	BY931881
E_EL_ovS0_31E08_R_0	BY931882
E_EL_ovS0_31E09_F_0	BY923511
E_EL_ovS0_31E09_R_0	BY931883
E_EL_ovS0_31E10_F_0	BY923512
E_EL_ovS0_31E10_R_0	BY931884
E_EL_ovS0_31E11_F_0	BY923513
E_EL_ovS0_31E11_R_0	BY931885
E_EL_ovS0_31E12_F_0	BY923514
E_EL_ovS0_31F01_F_0	BY923515
E_EL_ovS0_31F02_F_0	BY923516
E_EL_ovS0_31F03_F_0	BY923517
E_EL_ovS0_31F04_F_0	BY923518
E_EL_ovS0_31F04_R_0	BY931886
E_EL_ovS0_31F05_F_0	BY923519
E_EL_ovS0_31F05_R_0	BY931887
E_EL_ovS0_31F06_F_0	BY923520
E_EL_ovS0_31F06_R_0	BY931888
E_EL_ovS0_31F08_F_0	BY923521
E_EL_ovS0_31F08_R_0	BY931889
E_EL_ovS0_31F09_F_0	BY923522
E_EL_ovS0_31F09_R_0	BY931890
E_EL_ovS0_31F10_R_0	BY931891
E_EL_ovS0_31F11_F_0	BY923523
E_EL_ovS0_31F12_F_0	BY923524
E_EL_ovS0_31G01_F_0	BY923525
E_EL_ovS0_31G01_R_0	BY931892
E_EL_ovS0_31G02_F_0	BY923526
E_EL_ovS0_31G02_R_0	BY931893
E_EL_ovS0_31G03_F_0	BY923527
E_EL_ovS0_31G03_R_0	BY931894
E_EL_ovS0_31G04_F_0	BY923528
E_EL_ovS0_31G04_R_0	BY931895
E_EL_ovS0_31G06_F_0	BY923529
E_EL_ovS0_31G06_R_0	BY931896
E_EL_ovS0_31G08_F_0	BY923530
E_EL_ovS0_31G08_R_0	BY931897

E_EL_ovS0_31G09_F_0	BY923531
E_EL_ovS0_31G09_R_0	BY931898
E_EL_ovS0_31G10_F_0	BY923532
E_EL_ovS0_31G10_R_0	BY931899
E_EL_ovS0_31G11_F_0	BY923533
E_EL_ovS0_31G11_R_0	BY931900
E_EL_ovS0_31G12_F_0	BY923534
E_EL_ovS0_31G12_R_0	BY931901
E_EL_ovS0_31H01_R_0	BY931902
E_EL_ovS0_31H02_F_0	BY923535
E_EL_ovS0_31H02_R_0	BY931903
E_EL_ovS0_31H03_F_0	BY923536
E_EL_ovS0_31H03_R_0	BY931904
E_EL_ovS0_31H04_F_0	BY923537
E_EL_ovS0_31H04_R_0	BY931905
E_EL_ovS0_31H05_F_0	BY923538
E_EL_ovS0_31H05_R_0	BY931906
E_EL_ovS0_31H06_F_0	BY923539
E_EL_ovS0_31H09_F_0	BY923540
E_EL_ovS0_31H09_R_0	BY931907
E_EL_ovS0_31H10_F_0	BY923541
E_EL_ovS0_31H10_R_0	BY931908
E_EL_ovS0_31H11_F_0	BY923542
E_EL_ovS0_31H11_R_0	BY931909
E_EL_ovS0_32A01_R_0	BY932074
E_EL_ovS0_32A03_F_0	BY923543
E_EL_ovS0_32A03_R_0	BY932075
E_EL_ovS0_32A05_F_0	BY923544
E_EL_ovS0_32A05_R_0	BY932076
E_EL_ovS0_32A06_F_0	BY923545
E_EL_ovS0_32A06_R_0	BY932077
E_EL_ovS0_32A07_F_0	BY923546
E_EL_ovS0_32A07_R_0	BY932078
E_EL_ovS0_32A08_F_0	BY923547
E_EL_ovS0_32A08_R_0	BY932079
E_EL_ovS0_32A09_F_0	BY923548
E_EL_ovS0_32A10_F_0	BY923549
E_EL_ovS0_32A10_R_0	BY932080
E_EL_ovS0_32A11_F_0	BY923550
E_EL_ovS0_32A11_R_0	BY932081
E_EL_ovS0_32A12_F_0	BY923551
E_EL_ovS0_32A12_R_0	BY932082
E_EL_ovS0_32B01_F_0	BY923552
E_EL_ovS0_32B01_R_0	BY932083
E_EL_ovS0_32B02_F_0	BY923553
E_EL_ovS0_32B02_R_0	BY932084
E_EL_ovS0_32B03_F_0	BY923554
E_EL_ovS0_32B03_R_0	BY932085
E_EL_ovS0_32B04_F_0	BY923555
E_EL_ovS0_32B04_R_0	BY932086
E_EL_ovS0_32B05_F_0	BY923556
E_EL_ovS0_32B05_R_0	BY932087
E_EL_ovS0_32B06_F_0	BY923557
E_EL_ovS0_32B06_R_0	BY932088

E_EL_ovS0_32B07_F_0	BY923558
E_EL_ovS0_32B07_R_0	BY932089
E_EL_ovS0_32B09_F_0	BY923559
E_EL_ovS0_32B10_F_0	BY923560
E_EL_ovS0_32B10_R_0	BY932090
E_EL_ovS0_32B12_F_0	BY923561
E_EL_ovS0_32B12_R_0	BY932091
E_EL_ovS0_32C01_F_0	BY923562
E_EL_ovS0_32C02_F_0	BY923563
E_EL_ovS0_32C02_R_0	BY932092
E_EL_ovS0_32C03_F_0	BY923564
E_EL_ovS0_32C03_R_0	BY932093
E_EL_ovS0_32C04_F_0	BY923565
E_EL_ovS0_32C04_R_0	BY932094
E_EL_ovS0_32C05_F_0	BY923566
E_EL_ovS0_32C05_R_0	BY932095
E_EL_ovS0_32C06_F_0	BY923567
E_EL_ovS0_32C06_R_0	BY932096
E_EL_ovS0_32C07_F_0	BY923568
E_EL_ovS0_32C07_R_0	BY932097
E_EL_ovS0_32C08_F_0	BY923569
E_EL_ovS0_32C08_R_0	BY932098
E_EL_ovS0_32C09_F_0	BY923570
E_EL_ovS0_32C09_R_0	BY932099
E_EL_ovS0_32C10_F_0	BY923571
E_EL_ovS0_32C11_F_0	BY923572
E_EL_ovS0_32C11_R_0	BY932100
E_EL_ovS0_32C12_F_0	BY923573
E_EL_ovS0_32D01_F_0	BY923574
E_EL_ovS0_32D01_R_0	BY932101
E_EL_ovS0_32D02_F_0	BY923575
E_EL_ovS0_32D03_F_0	BY923576
E_EL_ovS0_32D03_R_0	BY932102
E_EL_ovS0_32D04_F_0	BY923577
E_EL_ovS0_32D07_F_0	BY923578
E_EL_ovS0_32D07_R_0	BY932103
E_EL_ovS0_32D08_F_0	BY923579
E_EL_ovS0_32D08_R_0	BY932104
E_EL_ovS0_32D09_F_0	BY923580
E_EL_ovS0_32D09_R_0	BY932105
E_EL_ovS0_32D10_F_0	BY923581
E_EL_ovS0_32D10_R_0	BY932106
E_EL_ovS0_32D12_F_0	BY923582
E_EL_ovS0_32D12_R_0	BY932107
E_EL_ovS0_32E01_F_0	BY923583
E_EL_ovS0_32E02_F_0	BY923584
E_EL_ovS0_32E02_R_0	BY932108
E_EL_ovS0_32E03_F_0	BY923585
E_EL_ovS0_32E03_R_0	BY932109
E_EL_ovS0_32E04_F_0	BY923586
E_EL_ovS0_32E04_R_0	BY932110
E_EL_ovS0_32E05_F_0	BY923587
E_EL_ovS0_32E05_R_0	BY932111
E_EL_ovS0_32E06_F_0	BY923588

E_EL_ovS0_32E06_R_0	BY932112
E_EL_ovS0_32E07_F_0	BY923589
E_EL_ovS0_32E07_R_0	BY932113
E_EL_ovS0_32E08_F_0	BY923590
E_EL_ovS0_32E08_R_0	BY932114
E_EL_ovS0_32E09_F_0	BY923591
E_EL_ovS0_32E09_R_0	BY932115
E_EL_ovS0_32E10_F_0	BY923592
E_EL_ovS0_32E10_R_0	BY932116
E_EL_ovS0_32E11_F_0	BY923593
E_EL_ovS0_32E11_R_0	BY932117
E_EL_ovS0_32E12_F_0	BY923594
E_EL_ovS0_32E12_R_0	BY932118
E_EL_ovS0_32F02_F_0	BY923595
E_EL_ovS0_32F02_R_0	BY932119
E_EL_ovS0_32F03_F_0	BY923596
E_EL_ovS0_32F03_R_0	BY932120
E_EL_ovS0_32F04_F_0	BY923597
E_EL_ovS0_32F04_R_0	BY932121
E_EL_ovS0_32F05_F_0	BY923598
E_EL_ovS0_32F05_R_0	BY932122
E_EL_ovS0_32F06_F_0	BY923599
E_EL_ovS0_32F06_R_0	BY932123
E_EL_ovS0_32F07_F_0	BY923600
E_EL_ovS0_32F07_R_0	BY932124
E_EL_ovS0_32F09_F_0	BY923601
E_EL_ovS0_32F09_R_0	BY932125
E_EL_ovS0_32F10_F_0	BY923602
E_EL_ovS0_32F10_R_0	BY932126
E_EL_ovS0_32F11_F_0	BY923603
E_EL_ovS0_32F11_R_0	BY932127
E_EL_ovS0_32F12_F_0	BY923604
E_EL_ovS0_32F12_R_0	BY932128
E_EL_ovS0_32G01_F_0	BY923605
E_EL_ovS0_32G01_R_0	BY932129
E_EL_ovS0_32G03_F_0	BY923606
E_EL_ovS0_32G03_R_0	BY932130
E_EL_ovS0_32G04_F_0	BY923607
E_EL_ovS0_32G04_R_0	BY932131
E_EL_ovS0_32G05_F_0	BY923608
E_EL_ovS0_32G05_R_0	BY932132
E_EL_ovS0_32G07_F_0	BY923609
E_EL_ovS0_32G07_R_0	BY932133
E_EL_ovS0_32G10_F_0	BY923610
E_EL_ovS0_32G10_R_0	BY932134
E_EL_ovS0_32G11_F_0	BY923611
E_EL_ovS0_32G12_F_0	BY923612
E_EL_ovS0_32G12_R_0	BY932135
E_EL_ovS0_32H01_F_0	BY923613
E_EL_ovS0_32H01_R_0	BY932136
E_EL_ovS0_32H02_F_0	BY923614
E_EL_ovS0_32H02_R_0	BY932137
E_EL_ovS0_32H03_F_0	BY923615
E_EL_ovS0_32H03_R_0	BY932138

	E_EL_ovS0_32H04_F_0	BY923616
	E_EL_ovS0_32H04_R_0	BY932139
	E_EL_ovS0_32H05_F_0	BY923617
	E_EL_ovS0_32H05_R_0	BY932140
	E_EL_ovS0_32H06_F_0	BY923618
	E_EL_ovS0_32H06_R_0	BY932141
	E_EL_ovS0_32H07_F_0	BY923619
	E_EL_ovS0_32H07_R_0	BY932142
	E_EL_ovS0_32H08_F_0	BY923620
	E_EL_ovS0_32H08_R_0	BY932143
	E_EL_ovS0_32H10_F_0	BY923621
	E_EL_ovS0_32H11_F_0	BY923622
	E_EL_ovS0_32H11_R_0	BY932144
	E_EL_ovS0_32H12_F_0	BY923623
	E_EL_ovS0_32H12_R_0	BY932145
vg4M	E_EL_vg4M_01A01_F_0	BY923624
	E_EL_vg4M_01A02_F_0	BY923625
	E_EL_vg4M_01A03_F_0	BY923626
	E_EL_vg4M_01A03_R_0	BY932146
	E_EL_vg4M_01A04_F_0	BY923627
	E_EL_vg4M_01A04_R_0	BY932147
	E_EL_vg4M_01A05_F_0	BY923628
	E_EL_vg4M_01A05_R_0	BY932148
	E_EL_vg4M_01A06_F_0	BY923629
	E_EL_vg4M_01A06_R_0	BY932149
	E_EL_vg4M_01A07_F_0	BY923630
	E_EL_vg4M_01A07_R_0	BY932150
	E_EL_vg4M_01A08_R_0	BY932151
	E_EL_vg4M_01A10_F_0	BY923631
	E_EL_vg4M_01A10_R_0	BY932152
	E_EL_vg4M_01A11_F_0	BY923632
	E_EL_vg4M_01A11_R_0	BY932153
	E_EL_vg4M_01A12_F_0	BY923633
	E_EL_vg4M_01A12_R_0	BY932154
	E_EL_vg4M_01A13_F_0	BY923634
	E_EL_vg4M_01A14_F_0	BY923635
	E_EL_vg4M_01A14_R_0	BY932155
	E_EL_vg4M_01A15_R_0	BY932156
	E_EL_vg4M_01A16_R_0	BY932157
	E_EL_vg4M_01A17_F_0	BY923636
	E_EL_vg4M_01A17_R_0	BY932158
	E_EL_vg4M_01A20_F_0	BY923637
	E_EL_vg4M_01A20_R_0	BY932159
	E_EL_vg4M_01A23_F_0	BY923638
	E_EL_vg4M_01A23_R_0	BY932160
	E_EL_vg4M_01A24_F_0	BY923639
	E_EL_vg4M_01A24_R_0	BY932161
	E_EL_vg4M_01B01_R_0	BY932162
	E_EL_vg4M_01B02_F_0	BY923640
	E_EL_vg4M_01B02_R_0	BY932163
	E_EL_vg4M_01B03_F_0	BY923641
	E_EL_vg4M_01B03_R_0	BY932164
	E_EL_vg4M_01B04_F_0	BY923642
	E_EL_vg4M_01B04_R_0	BY932165

E_EL_vg4M_01B05_F_0	BY923643
E_EL_vg4M_01B06_F_0	BY923644
E_EL_vg4M_01B06_R_0	BY932166
E_EL_vg4M_01B08_R_0	BY932167
E_EL_vg4M_01B09_F_0	BY923645
E_EL_vg4M_01B09_R_0	BY932168
E_EL_vg4M_01B10_F_0	BY923646
E_EL_vg4M_01B10_R_0	BY932169
E_EL_vg4M_01B11_R_0	BY932170
E_EL_vg4M_01B12_R_0	BY932171
E_EL_vg4M_01B13_F_0	BY923647
E_EL_vg4M_01B13_R_0	BY932172
E_EL_vg4M_01B14_F_0	BY923648
E_EL_vg4M_01B14_R_0	BY932173
E_EL_vg4M_01B16_F_0	BY923649
E_EL_vg4M_01B16_R_0	BY932174
E_EL_vg4M_01B17_F_0	BY923650
E_EL_vg4M_01B17_R_0	BY932175
E_EL_vg4M_01B18_F_0	BY923651
E_EL_vg4M_01B18_R_0	BY932176
E_EL_vg4M_01B19_F_0	BY923652
E_EL_vg4M_01B19_R_0	BY932177
E_EL_vg4M_01B20_F_0	BY923653
E_EL_vg4M_01B20_R_0	BY932178
E_EL_vg4M_01B21_R_0	BY932179
E_EL_vg4M_01B22_R_0	BY932180
E_EL_vg4M_01B23_F_0	BY923654
E_EL_vg4M_01B23_R_0	BY932181
E_EL_vg4M_01C01_F_0	BY923655
E_EL_vg4M_01C02_R_0	BY932182
E_EL_vg4M_01C03_F_0	BY923656
E_EL_vg4M_01C03_R_0	BY932183
E_EL_vg4M_01C04_F_0	BY923657
E_EL_vg4M_01C05_F_0	BY923658
E_EL_vg4M_01C07_F_0	BY923659
E_EL_vg4M_01C08_F_0	BY923660
E_EL_vg4M_01C08_R_0	BY932184
E_EL_vg4M_01C09_F_0	BY923661
E_EL_vg4M_01C09_R_0	BY932185
E_EL_vg4M_01C10_F_0	BY923662
E_EL_vg4M_01C10_R_0	BY932186
E_EL_vg4M_01C11_F_0	BY923663
E_EL_vg4M_01C11_R_0	BY932187
E_EL_vg4M_01C13_F_0	BY923664
E_EL_vg4M_01C13_R_0	BY932188
E_EL_vg4M_01C14_R_0	BY932189
E_EL_vg4M_01C15_F_0	BY923665
E_EL_vg4M_01C15_R_0	BY932190
E_EL_vg4M_01C16_R_0	BY932191
E_EL_vg4M_01C17_F_0	BY923666
E_EL_vg4M_01C17_R_0	BY932192
E_EL_vg4M_01C19_F_0	BY923667
E_EL_vg4M_01C19_R_0	BY932193
E_EL_vg4M_01C20_F_0	BY923668

E_EL_vg4M_01C20_R_0	BY932194
E_EL_vg4M_01C21_F_0	BY923669
E_EL_vg4M_01C21_R_0	BY932195
E_EL_vg4M_01C22_F_0	BY923670
E_EL_vg4M_01C22_R_0	BY932196
E_EL_vg4M_01C23_F_0	BY923671
E_EL_vg4M_01C23_R_0	BY932197
E_EL_vg4M_01C24_F_0	BY923672
E_EL_vg4M_01C24_R_0	BY932198
E_EL_vg4M_01D01_F_0	BY923673
E_EL_vg4M_01D01_R_0	BY932199
E_EL_vg4M_01D02_F_0	BY923674
E_EL_vg4M_01D02_R_0	BY932200
E_EL_vg4M_01D03_F_0	BY923675
E_EL_vg4M_01D03_R_0	BY932201
E_EL_vg4M_01D04_F_0	BY923676
E_EL_vg4M_01D04_R_0	BY932202
E_EL_vg4M_01D05_F_0	BY923677
E_EL_vg4M_01D05_R_0	BY932203
E_EL_vg4M_01D06_R_0	BY932204
E_EL_vg4M_01D07_F_0	BY923678
E_EL_vg4M_01D08_F_0	BY923679
E_EL_vg4M_01D08_R_0	BY932205
E_EL_vg4M_01D09_F_0	BY923680
E_EL_vg4M_01D10_F_0	BY923681
E_EL_vg4M_01D10_R_0	BY932206
E_EL_vg4M_01D11_R_0	BY932207
E_EL_vg4M_01D12_R_0	BY932208
E_EL_vg4M_01D13_F_0	BY923682
E_EL_vg4M_01D13_R_0	BY932209
E_EL_vg4M_01D14_F_0	BY923683
E_EL_vg4M_01D14_R_0	BY932210
E_EL_vg4M_01D15_F_0	BY923684
E_EL_vg4M_01D15_R_0	BY932211
E_EL_vg4M_01D16_F_0	BY923685
E_EL_vg4M_01D16_R_0	BY932212
E_EL_vg4M_01D17_R_0	BY932213
E_EL_vg4M_01D18_F_0	BY923686
E_EL_vg4M_01D18_R_0	BY932214
E_EL_vg4M_01D19_F_0	BY923687
E_EL_vg4M_01D19_R_0	BY932215
E_EL_vg4M_01D20_F_0	BY923688
E_EL_vg4M_01D20_R_0	BY932216
E_EL_vg4M_01D21_F_0	BY923689
E_EL_vg4M_01D21_R_0	BY932217
E_EL_vg4M_01D22_F_0	BY923690
E_EL_vg4M_01D22_R_0	BY932218
E_EL_vg4M_01D23_F_0	BY923691
E_EL_vg4M_01D23_R_0	BY932219
E_EL_vg4M_01D24_F_0	BY923692
E_EL_vg4M_01D24_R_0	BY932220
E_EL_vg4M_01E02_F_0	BY923693
E_EL_vg4M_01E02_R_0	BY932221
E_EL_vg4M_01E03_F_0	BY923694

E_EL_vg4M_01E04_F_0	BY923695
E_EL_vg4M_01E05_F_0	BY923696
E_EL_vg4M_01E05_R_0	BY932222
E_EL_vg4M_01E06_F_0	BY923697
E_EL_vg4M_01E06_R_0	BY932223
E_EL_vg4M_01E07_R_0	BY932224
E_EL_vg4M_01E08_R_0	BY932225
E_EL_vg4M_01E09_F_0	BY923698
E_EL_vg4M_01E11_F_0	BY923699
E_EL_vg4M_01E13_F_0	BY923700
E_EL_vg4M_01E13_R_0	BY932226
E_EL_vg4M_01E14_F_0	BY923701
E_EL_vg4M_01E14_R_0	BY932227
E_EL_vg4M_01E15_F_0	BY923702
E_EL_vg4M_01E15_R_0	BY932228
E_EL_vg4M_01E16_F_0	BY923703
E_EL_vg4M_01E17_R_0	BY932229
E_EL_vg4M_01E18_R_0	BY932230
E_EL_vg4M_01E19_F_0	BY923704
E_EL_vg4M_01E19_R_0	BY932231
E_EL_vg4M_01E20_F_0	BY923705
E_EL_vg4M_01E20_R_0	BY932232
E_EL_vg4M_01E21_R_0	BY932233
E_EL_vg4M_01E22_F_0	BY923706
E_EL_vg4M_01E23_F_0	BY923707
E_EL_vg4M_01E23_R_0	BY932234
E_EL_vg4M_01E24_F_0	BY923708
E_EL_vg4M_01E24_R_0	BY932235
E_EL_vg4M_01F01_R_0	BY932236
E_EL_vg4M_01F02_R_0	BY932237
E_EL_vg4M_01F03_F_0	BY923709
E_EL_vg4M_01F04_F_0	BY923710
E_EL_vg4M_01F05_F_0	BY923711
E_EL_vg4M_01F05_R_0	BY932238
E_EL_vg4M_01F06_F_0	BY923712
E_EL_vg4M_01F06_R_0	BY932239
E_EL_vg4M_01F07_F_0	BY923713
E_EL_vg4M_01F07_R_0	BY932240
E_EL_vg4M_01F08_F_0	BY923714
E_EL_vg4M_01F10_F_0	BY923715
E_EL_vg4M_01F10_R_0	BY932241
E_EL_vg4M_01F12_R_0	BY932242
E_EL_vg4M_01F13_F_0	BY923716
E_EL_vg4M_01F13_R_0	BY932243
E_EL_vg4M_01F14_F_0	BY923717
E_EL_vg4M_01F14_R_0	BY932244
E_EL_vg4M_01F15_F_0	BY923718
E_EL_vg4M_01F15_R_0	BY932245
E_EL_vg4M_01F16_F_0	BY923719
E_EL_vg4M_01F16_R_0	BY932246
E_EL_vg4M_01F18_R_0	BY932247
E_EL_vg4M_01F20_F_0	BY923720
E_EL_vg4M_01F20_R_0	BY932248
E_EL_vg4M_01F22_F_0	BY923721

E_EL_vg4M_01F23_F_0	BY923722
E_EL_vg4M_01F23_R_0	BY932249
E_EL_vg4M_01F24_F_0	BY923723
E_EL_vg4M_01F24_R_0	BY932250
E_EL_vg4M_01G02_R_0	BY932251
E_EL_vg4M_01G03_F_0	BY923724
E_EL_vg4M_01G03_R_0	BY932252
E_EL_vg4M_01G04_F_0	BY923725
E_EL_vg4M_01G04_R_0	BY932253
E_EL_vg4M_01G05_F_0	BY923726
E_EL_vg4M_01G05_R_0	BY932254
E_EL_vg4M_01G07_F_0	BY923727
E_EL_vg4M_01G07_R_0	BY932255
E_EL_vg4M_01G08_R_0	BY932256
E_EL_vg4M_01G10_F_0	BY923728
E_EL_vg4M_01G10_R_0	BY932257
E_EL_vg4M_01G11_F_0	BY923729
E_EL_vg4M_01G13_R_0	BY932258
E_EL_vg4M_01G14_R_0	BY932259
E_EL_vg4M_01G15_F_0	BY923730
E_EL_vg4M_01G15_R_0	BY932260
E_EL_vg4M_01G16_F_0	BY923731
E_EL_vg4M_01G16_R_0	BY932261
E_EL_vg4M_01G17_F_0	BY923732
E_EL_vg4M_01G17_R_0	BY932262
E_EL_vg4M_01G19_F_0	BY923733
E_EL_vg4M_01G21_F_0	BY923734
E_EL_vg4M_01G22_F_0	BY923735
E_EL_vg4M_01G22_R_0	BY932263
E_EL_vg4M_01G23_R_0	BY932264
E_EL_vg4M_01G24_F_0	BY923736
E_EL_vg4M_01H01_F_0	BY923737
E_EL_vg4M_01H02_F_0	BY923738
E_EL_vg4M_01H02_R_0	BY932265
E_EL_vg4M_01H03_F_0	BY923739
E_EL_vg4M_01H04_F_0	BY923740
E_EL_vg4M_01H04_R_0	BY932266
E_EL_vg4M_01H05_F_0	BY923741
E_EL_vg4M_01H05_R_0	BY932267
E_EL_vg4M_01H06_F_0	BY923742
E_EL_vg4M_01H06_R_0	BY932268
E_EL_vg4M_01H07_F_0	BY923743
E_EL_vg4M_01H07_R_0	BY932269
E_EL_vg4M_01H08_F_0	BY923744
E_EL_vg4M_01H08_R_0	BY932270
E_EL_vg4M_01H10_R_0	BY932271
E_EL_vg4M_01H11_F_0	BY923745
E_EL_vg4M_01H11_R_0	BY932272
E_EL_vg4M_01H12_F_0	BY923746
E_EL_vg4M_01H12_R_0	BY932273
E_EL_vg4M_01H13_F_0	BY923747
E_EL_vg4M_01H13_R_0	BY932274
E_EL_vg4M_01H14_F_0	BY923748
E_EL_vg4M_01H14_R_0	BY932275

E_EL_vg4M_01H15_F_0	BY923749
E_EL_vg4M_01H15_R_0	BY932276
E_EL_vg4M_01H16_F_0	BY923750
E_EL_vg4M_01H16_R_0	BY932277
E_EL_vg4M_01H19_F_0	BY923751
E_EL_vg4M_01H19_R_0	BY932278
E_EL_vg4M_01H20_F_0	BY923752
E_EL_vg4M_01H20_R_0	BY932279
E_EL_vg4M_01H21_F_0	BY923753
E_EL_vg4M_01H21_R_0	BY932280
E_EL_vg4M_01H22_F_0	BY923754
E_EL_vg4M_01H22_R_0	BY932281
E_EL_vg4M_01H23_R_0	BY932282
E_EL_vg4M_01H24_F_0	BY923755
E_EL_vg4M_01H24_R_0	BY932283
E_EL_vg4M_01I01_F_0	BY923756
E_EL_vg4M_01I02_F_0	BY923757
E_EL_vg4M_01I02_R_0	BY932284
E_EL_vg4M_01I03_F_0	BY923758
E_EL_vg4M_01I03_R_0	BY932285
E_EL_vg4M_01I05_F_0	BY923759
E_EL_vg4M_01I05_R_0	BY932286
E_EL_vg4M_01I06_F_0	BY923760
E_EL_vg4M_01I06_R_0	BY932287
E_EL_vg4M_01I08_F_0	BY923761
E_EL_vg4M_01I08_R_0	BY932288
E_EL_vg4M_01I09_F_0	BY923762
E_EL_vg4M_01I09_R_0	BY932289
E_EL_vg4M_01I10_F_0	BY923763
E_EL_vg4M_01I10_R_0	BY932290
E_EL_vg4M_01I12_R_0	BY932291
E_EL_vg4M_01I13_R_0	BY932292
E_EL_vg4M_01I14_F_0	BY923764
E_EL_vg4M_01I15_F_0	BY923765
E_EL_vg4M_01I15_R_0	BY932293
E_EL_vg4M_01I16_F_0	BY923766
E_EL_vg4M_01I16_R_0	BY932294
E_EL_vg4M_01I17_F_0	BY923767
E_EL_vg4M_01I17_R_0	BY932295
E_EL_vg4M_01I18_R_0	BY932296
E_EL_vg4M_01I20_F_0	BY923768
E_EL_vg4M_01I20_R_0	BY932297
E_EL_vg4M_01I21_F_0	BY923769
E_EL_vg4M_01I21_R_0	BY932298
E_EL_vg4M_01I22_F_0	BY923770
E_EL_vg4M_01I22_R_0	BY932299
E_EL_vg4M_01I23_F_0	BY923771
E_EL_vg4M_01I24_F_0	BY923772
E_EL_vg4M_01I24_R_0	BY932300
E_EL_vg4M_01J01_F_0	BY923773
E_EL_vg4M_01J01_R_0	BY932301
E_EL_vg4M_01J02_R_0	BY932302
E_EL_vg4M_01J03_F_0	BY923774
E_EL_vg4M_01J03_R_0	BY932303

E_EL_vg4M_01J04_F_0	BY923775
E_EL_vg4M_01J04_R_0	BY932304
E_EL_vg4M_01J05_F_0	BY923776
E_EL_vg4M_01J05_R_0	BY932305
E_EL_vg4M_01J06_F_0	BY923777
E_EL_vg4M_01J06_R_0	BY932306
E_EL_vg4M_01J07_F_0	BY923778
E_EL_vg4M_01J07_R_0	BY932307
E_EL_vg4M_01J08_F_0	BY923779
E_EL_vg4M_01J08_R_0	BY932308
E_EL_vg4M_01J09_R_0	BY932309
E_EL_vg4M_01J10_F_0	BY923780
E_EL_vg4M_01J10_R_0	BY932310
E_EL_vg4M_01J11_F_0	BY923781
E_EL_vg4M_01J11_R_0	BY932311
E_EL_vg4M_01J12_F_0	BY923782
E_EL_vg4M_01J12_R_0	BY932312
E_EL_vg4M_01J13_F_0	BY923783
E_EL_vg4M_01J13_R_0	BY932313
E_EL_vg4M_01J14_F_0	BY923784
E_EL_vg4M_01J14_R_0	BY932314
E_EL_vg4M_01J15_R_0	BY932315
E_EL_vg4M_01J18_R_0	BY932316
E_EL_vg4M_01J19_F_0	BY923785
E_EL_vg4M_01J19_R_0	BY932317
E_EL_vg4M_01J20_F_0	BY923786
E_EL_vg4M_01J20_R_0	BY932318
E_EL_vg4M_01J21_F_0	BY923787
E_EL_vg4M_01J22_F_0	BY923788
E_EL_vg4M_01J22_R_0	BY932319
E_EL_vg4M_01J23_F_0	BY923789
E_EL_vg4M_01J23_R_0	BY932320
E_EL_vg4M_01K01_F_0	BY923790
E_EL_vg4M_01K01_R_0	BY932321
E_EL_vg4M_01K02_R_0	BY932322
E_EL_vg4M_01K03_F_0	BY923791
E_EL_vg4M_01K03_R_0	BY932323
E_EL_vg4M_01K04_F_0	BY923792
E_EL_vg4M_01K06_F_0	BY923793
E_EL_vg4M_01K07_F_0	BY923794
E_EL_vg4M_01K08_R_0	BY932324
E_EL_vg4M_01K09_F_0	BY923795
E_EL_vg4M_01K09_R_0	BY932325
E_EL_vg4M_01K10_F_0	BY923796
E_EL_vg4M_01K10_R_0	BY932326
E_EL_vg4M_01K11_F_0	BY923797
E_EL_vg4M_01K11_R_0	BY932327
E_EL_vg4M_01K12_F_0	BY923798
E_EL_vg4M_01K13_F_0	BY923799
E_EL_vg4M_01K13_R_0	BY932328
E_EL_vg4M_01K15_F_0	BY923800
E_EL_vg4M_01K15_R_0	BY932329
E_EL_vg4M_01K16_F_0	BY923801
E_EL_vg4M_01K16_R_0	BY932330

E_EL_vg4M_01K17_F_0	BY923802
E_EL_vg4M_01K17_R_0	BY932331
E_EL_vg4M_01K21_F_0	BY923803
E_EL_vg4M_01K21_R_0	BY932332
E_EL_vg4M_01K22_F_0	BY923804
E_EL_vg4M_01K22_R_0	BY932333
E_EL_vg4M_01K23_F_0	BY923805
E_EL_vg4M_01K23_R_0	BY932334
E_EL_vg4M_01K24_F_0	BY923806
E_EL_vg4M_01K24_R_0	BY932335
E_EL_vg4M_01L01_F_0	BY923807
E_EL_vg4M_01L01_R_0	BY932336
E_EL_vg4M_01L02_F_0	BY923808
E_EL_vg4M_01L02_R_0	BY932337
E_EL_vg4M_01L03_F_0	BY923809
E_EL_vg4M_01L04_F_0	BY923810
E_EL_vg4M_01L04_R_0	BY932338
E_EL_vg4M_01L07_F_0	BY923811
E_EL_vg4M_01L08_F_0	BY923812
E_EL_vg4M_01L08_R_0	BY932339
E_EL_vg4M_01L09_F_0	BY923813
E_EL_vg4M_01L09_R_0	BY932340
E_EL_vg4M_01L10_F_0	BY923814
E_EL_vg4M_01L10_R_0	BY932341
E_EL_vg4M_01L11_R_0	BY932342
E_EL_vg4M_01L12_R_0	BY932343
E_EL_vg4M_01L13_F_0	BY923815
E_EL_vg4M_01L13_R_0	BY932344
E_EL_vg4M_01L14_R_0	BY932345
E_EL_vg4M_01L15_R_0	BY932346
E_EL_vg4M_01L16_F_0	BY923816
E_EL_vg4M_01L16_R_0	BY932347
E_EL_vg4M_01L17_F_0	BY923817
E_EL_vg4M_01L17_R_0	BY932348
E_EL_vg4M_01L19_F_0	BY923818
E_EL_vg4M_01L19_R_0	BY932349
E_EL_vg4M_01L20_F_0	BY923819
E_EL_vg4M_01L20_R_0	BY932350
E_EL_vg4M_01L21_F_0	BY923820
E_EL_vg4M_01L21_R_0	BY932351
E_EL_vg4M_01L22_F_0	BY923821
E_EL_vg4M_01L22_R_0	BY932352
E_EL_vg4M_01L23_F_0	BY923822
E_EL_vg4M_01L23_R_0	BY932353
E_EL_vg4M_01L24_F_0	BY923823
E_EL_vg4M_01L24_R_0	BY932354
E_EL_vg4M_01M01_F_0	BY923824
E_EL_vg4M_01M01_R_0	BY932355
E_EL_vg4M_01M02_F_0	BY923825
E_EL_vg4M_01M02_R_0	BY932356
E_EL_vg4M_01M03_F_0	BY923826
E_EL_vg4M_01M03_R_0	BY932357
E_EL_vg4M_01M04_F_0	BY923827
E_EL_vg4M_01M04_R_0	BY932358

E_EL_vg4M_01M06_F_0	BY923828
E_EL_vg4M_01M06_R_0	BY932359
E_EL_vg4M_01M08_F_0	BY923829
E_EL_vg4M_01M08_R_0	BY932360
E_EL_vg4M_01M09_F_0	BY923830
E_EL_vg4M_01M09_R_0	BY932361
E_EL_vg4M_01M10_F_0	BY923831
E_EL_vg4M_01M10_R_0	BY932362
E_EL_vg4M_01M11_F_0	BY923832
E_EL_vg4M_01M13_F_0	BY923833
E_EL_vg4M_01M13_R_0	BY932363
E_EL_vg4M_01M14_F_0	BY923834
E_EL_vg4M_01M14_R_0	BY932364
E_EL_vg4M_01M15_F_0	BY923835
E_EL_vg4M_01M15_R_0	BY932365
E_EL_vg4M_01M16_F_0	BY923836
E_EL_vg4M_01M16_R_0	BY932366
E_EL_vg4M_01M17_F_0	BY923837
E_EL_vg4M_01M17_R_0	BY932367
E_EL_vg4M_01M19_F_0	BY923838
E_EL_vg4M_01M19_R_0	BY932368
E_EL_vg4M_01M20_F_0	BY923839
E_EL_vg4M_01M20_R_0	BY932369
E_EL_vg4M_01M21_F_0	BY923840
E_EL_vg4M_01M21_R_0	BY932370
E_EL_vg4M_01M22_F_0	BY923841
E_EL_vg4M_01M24_F_0	BY923842
E_EL_vg4M_01M24_R_0	BY932371
E_EL_vg4M_01N01_F_0	BY923843
E_EL_vg4M_01N01_R_0	BY932372
E_EL_vg4M_01N02_F_0	BY923844
E_EL_vg4M_01N02_R_0	BY932373
E_EL_vg4M_01N03_F_0	BY923845
E_EL_vg4M_01N03_R_0	BY932374
E_EL_vg4M_01N04_F_0	BY923846
E_EL_vg4M_01N04_R_0	BY932375
E_EL_vg4M_01N07_F_0	BY923847
E_EL_vg4M_01N08_F_0	BY923848
E_EL_vg4M_01N08_R_0	BY932376
E_EL_vg4M_01N09_F_0	BY923849
E_EL_vg4M_01N09_R_0	BY932377
E_EL_vg4M_01N10_F_0	BY923850
E_EL_vg4M_01N10_R_0	BY932378
E_EL_vg4M_01N11_F_0	BY923851
E_EL_vg4M_01N11_R_0	BY932379
E_EL_vg4M_01N12_F_0	BY923852
E_EL_vg4M_01N13_F_0	BY923853
E_EL_vg4M_01N13_R_0	BY932380
E_EL_vg4M_01N14_F_0	BY923854
E_EL_vg4M_01N14_R_0	BY932381
E_EL_vg4M_01N16_F_0	BY923855
E_EL_vg4M_01N16_R_0	BY932382
E_EL_vg4M_01N17_F_0	BY923856
E_EL_vg4M_01N17_R_0	BY932383

E_EL_vg4M_01N18_R_0	BY932384
E_EL_vg4M_01N19_F_0	BY923857
E_EL_vg4M_01N19_R_0	BY932385
E_EL_vg4M_01N20_F_0	BY923858
E_EL_vg4M_01N20_R_0	BY932386
E_EL_vg4M_01N22_F_0	BY923859
E_EL_vg4M_01N22_R_0	BY932387
E_EL_vg4M_01N23_F_0	BY923860
E_EL_vg4M_01N23_R_0	BY932388
E_EL_vg4M_01N24_F_0	BY923861
E_EL_vg4M_01N24_R_0	BY932389
E_EL_vg4M_01O01_F_0	BY923862
E_EL_vg4M_01O02_F_0	BY923863
E_EL_vg4M_01O02_R_0	BY932390
E_EL_vg4M_01O04_F_0	BY923864
E_EL_vg4M_01O07_F_0	BY923865
E_EL_vg4M_01O07_R_0	BY932391
E_EL_vg4M_01O08_F_0	BY923866
E_EL_vg4M_01O08_R_0	BY932392
E_EL_vg4M_01O09_F_0	BY923867
E_EL_vg4M_01O09_R_0	BY932393
E_EL_vg4M_01O11_R_0	BY932394
E_EL_vg4M_01O12_F_0	BY923868
E_EL_vg4M_01O13_F_0	BY923869
E_EL_vg4M_01O13_R_0	BY932395
E_EL_vg4M_01O14_F_0	BY923870
E_EL_vg4M_01O14_R_0	BY932396
E_EL_vg4M_01O15_F_0	BY923871
E_EL_vg4M_01O15_R_0	BY932397
E_EL_vg4M_01O16_F_0	BY923872
E_EL_vg4M_01O16_R_0	BY932398
E_EL_vg4M_01O19_F_0	BY923873
E_EL_vg4M_01O19_R_0	BY932399
E_EL_vg4M_01O20_F_0	BY923874
E_EL_vg4M_01O20_R_0	BY932400
E_EL_vg4M_01O22_F_0	BY923875
E_EL_vg4M_01O23_F_0	BY923876
E_EL_vg4M_01O23_R_0	BY932401
E_EL_vg4M_01O24_F_0	BY923877
E_EL_vg4M_01P01_F_0	BY923878
E_EL_vg4M_01P01_R_0	BY932402
E_EL_vg4M_01P02_F_0	BY923879
E_EL_vg4M_01P02_R_0	BY932403
E_EL_vg4M_01P04_F_0	BY923880
E_EL_vg4M_01P04_R_0	BY932404
E_EL_vg4M_01P05_F_0	BY923881
E_EL_vg4M_01P06_F_0	BY923882
E_EL_vg4M_01P07_F_0	BY923883
E_EL_vg4M_01P07_R_0	BY932405
E_EL_vg4M_01P08_F_0	BY923884
E_EL_vg4M_01P08_R_0	BY932406
E_EL_vg4M_01P09_F_0	BY923885
E_EL_vg4M_01P09_R_0	BY932407
E_EL_vg4M_01P11_R_0	BY932408

E_EL_vg4M_01P12_R_0	BY932409
E_EL_vg4M_01P13_F_0	BY923886
E_EL_vg4M_01P13_R_0	BY932410
E_EL_vg4M_01P14_F_0	BY923887
E_EL_vg4M_01P14_R_0	BY932411
E_EL_vg4M_01P15_F_0	BY923888
E_EL_vg4M_01P15_R_0	BY932412
E_EL_vg4M_01P16_F_0	BY923889
E_EL_vg4M_01P16_R_0	BY932413
E_EL_vg4M_01P18_F_0	BY923890
E_EL_vg4M_01P18_R_0	BY932414
E_EL_vg4M_01P19_F_0	BY923891
E_EL_vg4M_01P19_R_0	BY932415
E_EL_vg4M_01P21_F_0	BY923892
E_EL_vg4M_01P21_R_0	BY932416
E_EL_vg4M_01P22_F_0	BY923893
E_EL_vg4M_01P22_R_0	BY932417
E_EL_vg4M_01P23_F_0	BY923894
E_EL_vg4M_01P23_R_0	BY932418
E_EL_vg4M_01P24_F_0	BY923895
E_EL_vg4M_01P24_R_0	BY932419
E_EL_vg4M_02A01_F_0	BY923896
E_EL_vg4M_02A01_R_0	BY932420
E_EL_vg4M_02A02_R_0	BY932421
E_EL_vg4M_02A03_F_0	BY923897
E_EL_vg4M_02A03_R_0	BY932422
E_EL_vg4M_02A04_F_0	BY923898
E_EL_vg4M_02A04_R_0	BY932423
E_EL_vg4M_02A05_F_0	BY923899
E_EL_vg4M_02A05_R_0	BY932424
E_EL_vg4M_02A06_F_0	BY923900
E_EL_vg4M_02A06_R_0	BY932425
E_EL_vg4M_02A07_F_0	BY923901
E_EL_vg4M_02A07_R_0	BY932426
E_EL_vg4M_02A08_R_0	BY932427
E_EL_vg4M_02A09_R_0	BY932428
E_EL_vg4M_02A10_F_0	BY923902
E_EL_vg4M_02A10_R_0	BY932429
E_EL_vg4M_02A11_F_0	BY923903
E_EL_vg4M_02A12_F_0	BY923904
E_EL_vg4M_02A12_R_0	BY932430
E_EL_vg4M_02A14_F_0	BY923905
E_EL_vg4M_02A14_R_0	BY932431
E_EL_vg4M_02A15_F_0	BY923906
E_EL_vg4M_02A16_F_0	BY923907
E_EL_vg4M_02A16_R_0	BY932432
E_EL_vg4M_02A17_F_0	BY923908
E_EL_vg4M_02A17_R_0	BY932433
E_EL_vg4M_02A18_F_0	BY923909
E_EL_vg4M_02A18_R_0	BY932434
E_EL_vg4M_02A20_F_0	BY923910
E_EL_vg4M_02A20_R_0	BY932435
E_EL_vg4M_02A21_F_0	BY923911
E_EL_vg4M_02A21_R_0	BY932436

E_EL_vg4M_02A22_F_0	BY923912
E_EL_vg4M_02A22_R_0	BY932437
E_EL_vg4M_02A23_F_0	BY923913
E_EL_vg4M_02A23_R_0	BY932438
E_EL_vg4M_02A24_F_0	BY923914
E_EL_vg4M_02A24_R_0	BY932439
E_EL_vg4M_02B01_F_0	BY923915
E_EL_vg4M_02B01_R_0	BY932440
E_EL_vg4M_02B02_F_0	BY923916
E_EL_vg4M_02B02_R_0	BY932441
E_EL_vg4M_02B03_R_0	BY932442
E_EL_vg4M_02B04_F_0	BY923917
E_EL_vg4M_02B04_R_0	BY932443
E_EL_vg4M_02B05_F_0	BY923918
E_EL_vg4M_02B05_R_0	BY932444
E_EL_vg4M_02B06_F_0	BY923919
E_EL_vg4M_02B06_R_0	BY932445
E_EL_vg4M_02B07_F_0	BY923920
E_EL_vg4M_02B07_R_0	BY932446
E_EL_vg4M_02B08_R_0	BY932447
E_EL_vg4M_02B09_F_0	BY923921
E_EL_vg4M_02B09_R_0	BY932448
E_EL_vg4M_02B10_F_0	BY923922
E_EL_vg4M_02B10_R_0	BY932449
E_EL_vg4M_02B11_F_0	BY923923
E_EL_vg4M_02B11_R_0	BY932450
E_EL_vg4M_02B12_F_0	BY923924
E_EL_vg4M_02B12_R_0	BY932451
E_EL_vg4M_02B13_F_0	BY923925
E_EL_vg4M_02B13_R_0	BY932452
E_EL_vg4M_02B14_F_0	BY923926
E_EL_vg4M_02B14_R_0	BY932453
E_EL_vg4M_02B15_F_0	BY923927
E_EL_vg4M_02B16_F_0	BY923928
E_EL_vg4M_02B16_R_0	BY932454
E_EL_vg4M_02B17_F_0	BY923929
E_EL_vg4M_02B17_R_0	BY932455
E_EL_vg4M_02B18_F_0	BY923930
E_EL_vg4M_02B18_R_0	BY932456
E_EL_vg4M_02B19_F_0	BY923931
E_EL_vg4M_02B19_R_0	BY932457
E_EL_vg4M_02B20_F_0	BY923932
E_EL_vg4M_02B20_R_0	BY932458
E_EL_vg4M_02B21_F_0	BY923933
E_EL_vg4M_02B21_R_0	BY932459
E_EL_vg4M_02B22_F_0	BY923934
E_EL_vg4M_02B23_R_0	BY932460
E_EL_vg4M_02B24_F_0	BY923935
E_EL_vg4M_02C01_F_0	BY923936
E_EL_vg4M_02C02_F_0	BY923937
E_EL_vg4M_02C02_R_0	BY932461
E_EL_vg4M_02C03_F_0	BY923938
E_EL_vg4M_02C03_R_0	BY932462
E_EL_vg4M_02C04_F_0	BY923939

E_EL_vg4M_02C04_R_0	BY932463
E_EL_vg4M_02C06_R_0	BY932464
E_EL_vg4M_02C07_F_0	BY923940
E_EL_vg4M_02C07_R_0	BY932465
E_EL_vg4M_02C08_F_0	BY923941
E_EL_vg4M_02C08_R_0	BY932466
E_EL_vg4M_02C10_F_0	BY923942
E_EL_vg4M_02C10_R_0	BY932467
E_EL_vg4M_02C11_F_0	BY923943
E_EL_vg4M_02C11_R_0	BY932468
E_EL_vg4M_02C12_F_0	BY923944
E_EL_vg4M_02C12_R_0	BY932469
E_EL_vg4M_02C13_F_0	BY923945
E_EL_vg4M_02C13_R_0	BY932470
E_EL_vg4M_02C14_F_0	BY923946
E_EL_vg4M_02C15_F_0	BY923947
E_EL_vg4M_02C15_R_0	BY932471
E_EL_vg4M_02C16_F_0	BY923948
E_EL_vg4M_02C16_R_0	BY932472
E_EL_vg4M_02C18_F_0	BY923949
E_EL_vg4M_02C18_R_0	BY932473
E_EL_vg4M_02C19_F_0	BY923950
E_EL_vg4M_02C19_R_0	BY932474
E_EL_vg4M_02C20_F_0	BY923951
E_EL_vg4M_02C20_R_0	BY932475
E_EL_vg4M_02C21_F_0	BY923952
E_EL_vg4M_02C21_R_0	BY932476
E_EL_vg4M_02C22_F_0	BY923953
E_EL_vg4M_02C22_R_0	BY932477
E_EL_vg4M_02C23_F_0	BY923954
E_EL_vg4M_02C23_R_0	BY932478
E_EL_vg4M_02C24_F_0	BY923955
E_EL_vg4M_02C24_R_0	BY932479
E_EL_vg4M_02D01_F_0	BY923956
E_EL_vg4M_02D01_R_0	BY932480
E_EL_vg4M_02D02_F_0	BY923957
E_EL_vg4M_02D02_R_0	BY932481
E_EL_vg4M_02D03_R_0	BY932482
E_EL_vg4M_02D04_F_0	BY923958
E_EL_vg4M_02D04_R_0	BY932483
E_EL_vg4M_02D05_F_0	BY923959
E_EL_vg4M_02D05_R_0	BY932484
E_EL_vg4M_02D06_F_0	BY923960
E_EL_vg4M_02D06_R_0	BY932485
E_EL_vg4M_02D07_F_0	BY923961
E_EL_vg4M_02D07_R_0	BY932486
E_EL_vg4M_02D08_F_0	BY923962
E_EL_vg4M_02D08_R_0	BY932487
E_EL_vg4M_02D09_F_0	BY923963
E_EL_vg4M_02D09_R_0	BY932488
E_EL_vg4M_02D10_F_0	BY923964
E_EL_vg4M_02D10_R_0	BY932489
E_EL_vg4M_02D11_R_0	BY932490
E_EL_vg4M_02D12_F_0	BY923965

E_EL_vg4M_02D12_R_0	BY932491
E_EL_vg4M_02D13_F_0	BY923966
E_EL_vg4M_02D13_R_0	BY932492
E_EL_vg4M_02D14_F_0	BY923967
E_EL_vg4M_02D14_R_0	BY932493
E_EL_vg4M_02D16_F_0	BY923968
E_EL_vg4M_02D16_R_0	BY932494
E_EL_vg4M_02D17_F_0	BY923969
E_EL_vg4M_02D17_R_0	BY932495
E_EL_vg4M_02D19_F_0	BY923970
E_EL_vg4M_02D19_R_0	BY932496
E_EL_vg4M_02D20_F_0	BY923971
E_EL_vg4M_02D20_R_0	BY932497
E_EL_vg4M_02D21_F_0	BY923972
E_EL_vg4M_02D21_R_0	BY932498
E_EL_vg4M_02D22_F_0	BY923973
E_EL_vg4M_02D22_R_0	BY932499
E_EL_vg4M_02D23_F_0	BY923974
E_EL_vg4M_02D23_R_0	BY932500
E_EL_vg4M_02D24_F_0	BY923975
E_EL_vg4M_02D24_R_0	BY932501
E_EL_vg4M_02E01_R_0	BY932502
E_EL_vg4M_02E02_F_0	BY923976
E_EL_vg4M_02E02_R_0	BY932503
E_EL_vg4M_02E03_F_0	BY923977
E_EL_vg4M_02E03_R_0	BY932504
E_EL_vg4M_02E04_F_0	BY923978
E_EL_vg4M_02E06_F_0	BY923979
E_EL_vg4M_02E06_R_0	BY932505
E_EL_vg4M_02E07_F_0	BY923980
E_EL_vg4M_02E07_R_0	BY932506
E_EL_vg4M_02E08_R_0	BY932507
E_EL_vg4M_02E09_F_0	BY923981
E_EL_vg4M_02E09_R_0	BY932508
E_EL_vg4M_02E10_F_0	BY923982
E_EL_vg4M_02E11_F_0	BY923983
E_EL_vg4M_02E11_R_0	BY932509
E_EL_vg4M_02E12_F_0	BY923984
E_EL_vg4M_02E12_R_0	BY932510
E_EL_vg4M_02E15_F_0	BY923985
E_EL_vg4M_02E15_R_0	BY932511
E_EL_vg4M_02E16_F_0	BY923986
E_EL_vg4M_02E16_R_0	BY932512
E_EL_vg4M_02E17_F_0	BY923987
E_EL_vg4M_02E17_R_0	BY932513
E_EL_vg4M_02E18_F_0	BY923988
E_EL_vg4M_02E18_R_0	BY932514
E_EL_vg4M_02E19_R_0	BY932515
E_EL_vg4M_02E20_F_0	BY923989
E_EL_vg4M_02E20_R_0	BY932516
E_EL_vg4M_02E21_F_0	BY923990
E_EL_vg4M_02E21_R_0	BY932517
E_EL_vg4M_02E22_F_0	BY923991
E_EL_vg4M_02E22_R_0	BY932518

E_EL_vg4M_02E23_F_0	BY923992
E_EL_vg4M_02E23_R_0	BY932519
E_EL_vg4M_02E24_F_0	BY923993
E_EL_vg4M_02E24_R_0	BY932520
E_EL_vg4M_02F01_F_0	BY923994
E_EL_vg4M_02F01_R_0	BY932521
E_EL_vg4M_02F02_F_0	BY923995
E_EL_vg4M_02F02_R_0	BY932522
E_EL_vg4M_02F03_R_0	BY932523
E_EL_vg4M_02F04_F_0	BY923996
E_EL_vg4M_02F04_R_0	BY932524
E_EL_vg4M_02F06_F_0	BY923997
E_EL_vg4M_02F06_R_0	BY932525
E_EL_vg4M_02F07_F_0	BY923998
E_EL_vg4M_02F08_R_0	BY932526
E_EL_vg4M_02F09_F_0	BY923999
E_EL_vg4M_02F09_R_0	BY932527
E_EL_vg4M_02F10_F_0	BY924000
E_EL_vg4M_02F10_R_0	BY932528
E_EL_vg4M_02F11_F_0	BY924001
E_EL_vg4M_02F11_R_0	BY932529
E_EL_vg4M_02F12_F_0	BY924002
E_EL_vg4M_02F13_F_0	BY924003
E_EL_vg4M_02F13_R_0	BY932530
E_EL_vg4M_02F14_F_0	BY924004
E_EL_vg4M_02F14_R_0	BY932531
E_EL_vg4M_02F15_F_0	BY924005
E_EL_vg4M_02F15_R_0	BY932532
E_EL_vg4M_02F16_F_0	BY924006
E_EL_vg4M_02F16_R_0	BY932533
E_EL_vg4M_02F17_F_0	BY924007
E_EL_vg4M_02F17_R_0	BY932534
E_EL_vg4M_02F18_F_0	BY924008
E_EL_vg4M_02F18_R_0	BY932535
E_EL_vg4M_02F19_F_0	BY924009
E_EL_vg4M_02F19_R_0	BY932536
E_EL_vg4M_02F20_R_0	BY932537
E_EL_vg4M_02F22_F_0	BY924010
E_EL_vg4M_02F22_R_0	BY932538
E_EL_vg4M_02F24_F_0	BY924011
E_EL_vg4M_02F24_R_0	BY932539
E_EL_vg4M_02G01_F_0	BY924012
E_EL_vg4M_02G02_F_0	BY924013
E_EL_vg4M_02G02_R_0	BY932540
E_EL_vg4M_02G03_F_0	BY924014
E_EL_vg4M_02G03_R_0	BY932541
E_EL_vg4M_02G04_F_0	BY924015
E_EL_vg4M_02G04_R_0	BY932542
E_EL_vg4M_02G05_R_0	BY932543
E_EL_vg4M_02G06_F_0	BY924016
E_EL_vg4M_02G06_R_0	BY932544
E_EL_vg4M_02G07_F_0	BY924017
E_EL_vg4M_02G07_R_0	BY932545
E_EL_vg4M_02G08_F_0	BY924018

E_EL_vg4M_02G08_R_0	BY932546
E_EL_vg4M_02G10_F_0	BY924019
E_EL_vg4M_02G10_R_0	BY932547
E_EL_vg4M_02G11_F_0	BY924020
E_EL_vg4M_02G11_R_0	BY932548
E_EL_vg4M_02G12_F_0	BY924021
E_EL_vg4M_02G12_R_0	BY932549
E_EL_vg4M_02G13_F_0	BY924022
E_EL_vg4M_02G14_F_0	BY924023
E_EL_vg4M_02G14_R_0	BY932550
E_EL_vg4M_02G15_F_0	BY924024
E_EL_vg4M_02G15_R_0	BY932551
E_EL_vg4M_02G16_F_0	BY924025
E_EL_vg4M_02G16_R_0	BY932552
E_EL_vg4M_02G17_F_0	BY924026
E_EL_vg4M_02G17_R_0	BY932553
E_EL_vg4M_02G19_F_0	BY924027
E_EL_vg4M_02G19_R_0	BY932554
E_EL_vg4M_02G21_F_0	BY924028
E_EL_vg4M_02G21_R_0	BY932555
E_EL_vg4M_02G22_F_0	BY924029
E_EL_vg4M_02G22_R_0	BY932556
E_EL_vg4M_02G24_R_0	BY932557
E_EL_vg4M_02H01_F_0	BY924030
E_EL_vg4M_02H01_R_0	BY932558
E_EL_vg4M_02H02_F_0	BY924031
E_EL_vg4M_02H02_R_0	BY932559
E_EL_vg4M_02H03_F_0	BY924032
E_EL_vg4M_02H03_R_0	BY932560
E_EL_vg4M_02H04_F_0	BY924033
E_EL_vg4M_02H04_R_0	BY932561
E_EL_vg4M_02H05_F_0	BY924034
E_EL_vg4M_02H06_F_0	BY924035
E_EL_vg4M_02H06_R_0	BY932562
E_EL_vg4M_02H07_F_0	BY924036
E_EL_vg4M_02H07_R_0	BY932563
E_EL_vg4M_02H08_F_0	BY924037
E_EL_vg4M_02H08_R_0	BY932564
E_EL_vg4M_02H09_F_0	BY924038
E_EL_vg4M_02H09_R_0	BY932565
E_EL_vg4M_02H11_F_0	BY924039
E_EL_vg4M_02H11_R_0	BY932566
E_EL_vg4M_02H12_F_0	BY924040
E_EL_vg4M_02H12_R_0	BY932567
E_EL_vg4M_02H13_F_0	BY924041
E_EL_vg4M_02H13_R_0	BY932568
E_EL_vg4M_02H14_R_0	BY932569
E_EL_vg4M_02H15_F_0	BY924042
E_EL_vg4M_02H17_F_0	BY924043
E_EL_vg4M_02H17_R_0	BY932570
E_EL_vg4M_02H18_R_0	BY932571
E_EL_vg4M_02H19_F_0	BY924044
E_EL_vg4M_02H19_R_0	BY932572
E_EL_vg4M_02H20_F_0	BY924045

E_EL_vg4M_02H20_R_0	BY932573
E_EL_vg4M_02H21_F_0	BY924046
E_EL_vg4M_02H21_R_0	BY932574
E_EL_vg4M_02H22_F_0	BY924047
E_EL_vg4M_02H22_R_0	BY932575
E_EL_vg4M_02H23_F_0	BY924048
E_EL_vg4M_02H23_R_0	BY932576
E_EL_vg4M_02H24_F_0	BY924049
E_EL_vg4M_02H24_R_0	BY932577
E_EL_vg4M_02I01_F_0	BY924050
E_EL_vg4M_02I02_F_0	BY924051
E_EL_vg4M_02I02_R_0	BY932578
E_EL_vg4M_02I03_F_0	BY924052
E_EL_vg4M_02I03_R_0	BY932579
E_EL_vg4M_02I04_F_0	BY924053
E_EL_vg4M_02I04_R_0	BY932580
E_EL_vg4M_02I05_F_0	BY924054
E_EL_vg4M_02I05_R_0	BY932581
E_EL_vg4M_02I06_F_0	BY924055
E_EL_vg4M_02I07_R_0	BY932582
E_EL_vg4M_02I08_R_0	BY932583
E_EL_vg4M_02I09_F_0	BY924056
E_EL_vg4M_02I10_F_0	BY924057
E_EL_vg4M_02I10_R_0	BY932584
E_EL_vg4M_02I11_F_0	BY924058
E_EL_vg4M_02I11_R_0	BY932585
E_EL_vg4M_02I12_F_0	BY924059
E_EL_vg4M_02I12_R_0	BY932586
E_EL_vg4M_02I14_F_0	BY924060
E_EL_vg4M_02I14_R_0	BY932587
E_EL_vg4M_02I15_F_0	BY924061
E_EL_vg4M_02I15_R_0	BY932588
E_EL_vg4M_02I16_F_0	BY924062
E_EL_vg4M_02I16_R_0	BY932589
E_EL_vg4M_02I18_F_0	BY924063
E_EL_vg4M_02I18_R_0	BY932590
E_EL_vg4M_02I19_F_0	BY924064
E_EL_vg4M_02I19_R_0	BY932591
E_EL_vg4M_02I20_F_0	BY924065
E_EL_vg4M_02I20_R_0	BY932592
E_EL_vg4M_02I21_F_0	BY924066
E_EL_vg4M_02I21_R_0	BY932593
E_EL_vg4M_02I22_F_0	BY924067
E_EL_vg4M_02I22_R_0	BY932594
E_EL_vg4M_02I23_F_0	BY924068
E_EL_vg4M_02I23_R_0	BY932595
E_EL_vg4M_02I24_F_0	BY924069
E_EL_vg4M_02I24_R_0	BY932596
E_EL_vg4M_02J01_F_0	BY924070
E_EL_vg4M_02J01_R_0	BY932597
E_EL_vg4M_02J02_R_0	BY932598
E_EL_vg4M_02J03_R_0	BY932599
E_EL_vg4M_02J04_F_0	BY924071
E_EL_vg4M_02J04_R_0	BY932600

E_EL_vg4M_02J05_F_0	BY924072
E_EL_vg4M_02J05_R_0	BY932601
E_EL_vg4M_02J06_F_0	BY924073
E_EL_vg4M_02J06_R_0	BY932602
E_EL_vg4M_02J07_F_0	BY924074
E_EL_vg4M_02J07_R_0	BY932603
E_EL_vg4M_02J08_F_0	BY924075
E_EL_vg4M_02J08_R_0	BY932604
E_EL_vg4M_02J09_F_0	BY924076
E_EL_vg4M_02J09_R_0	BY932605
E_EL_vg4M_02J11_F_0	BY924077
E_EL_vg4M_02J11_R_0	BY932606
E_EL_vg4M_02J12_F_0	BY924078
E_EL_vg4M_02J12_R_0	BY932607
E_EL_vg4M_02J13_F_0	BY924079
E_EL_vg4M_02J13_R_0	BY932608
E_EL_vg4M_02J14_R_0	BY932609
E_EL_vg4M_02J15_F_0	BY924080
E_EL_vg4M_02J15_R_0	BY932610
E_EL_vg4M_02J16_F_0	BY924081
E_EL_vg4M_02J16_R_0	BY932611
E_EL_vg4M_02J17_F_0	BY924082
E_EL_vg4M_02J18_F_0	BY924083
E_EL_vg4M_02J18_R_0	BY932612
E_EL_vg4M_02J19_F_0	BY924084
E_EL_vg4M_02J19_R_0	BY932613
E_EL_vg4M_02J20_F_0	BY924085
E_EL_vg4M_02J20_R_0	BY932614
E_EL_vg4M_02J21_F_0	BY924086
E_EL_vg4M_02J21_R_0	BY932615
E_EL_vg4M_02J22_F_0	BY924087
E_EL_vg4M_02J22_R_0	BY932616
E_EL_vg4M_02J23_F_0	BY924088
E_EL_vg4M_02J23_R_0	BY932617
E_EL_vg4M_02J24_F_0	BY924089
E_EL_vg4M_02J24_R_0	BY932618
E_EL_vg4M_02K01_F_0	BY924090
E_EL_vg4M_02K02_F_0	BY924091
E_EL_vg4M_02K02_R_0	BY932619
E_EL_vg4M_02K03_F_0	BY924092
E_EL_vg4M_02K03_R_0	BY932620
E_EL_vg4M_02K04_F_0	BY924093
E_EL_vg4M_02K04_R_0	BY932621
E_EL_vg4M_02K05_F_0	BY924094
E_EL_vg4M_02K05_R_0	BY932622
E_EL_vg4M_02K06_F_0	BY924095
E_EL_vg4M_02K06_R_0	BY932623
E_EL_vg4M_02K08_F_0	BY924096
E_EL_vg4M_02K08_R_0	BY932624
E_EL_vg4M_02K09_F_0	BY924097
E_EL_vg4M_02K09_R_0	BY932625
E_EL_vg4M_02K10_R_0	BY932626
E_EL_vg4M_02K11_F_0	BY924098
E_EL_vg4M_02K11_R_0	BY932627

E_EL_vg4M_02K12_F_0	BY924099
E_EL_vg4M_02K12_R_0	BY932628
E_EL_vg4M_02K13_F_0	BY924100
E_EL_vg4M_02K13_R_0	BY932629
E_EL_vg4M_02K14_F_0	BY924101
E_EL_vg4M_02K14_R_0	BY932630
E_EL_vg4M_02K15_F_0	BY924102
E_EL_vg4M_02K15_R_0	BY932631
E_EL_vg4M_02K17_F_0	BY924103
E_EL_vg4M_02K17_R_0	BY932632
E_EL_vg4M_02K18_F_0	BY924104
E_EL_vg4M_02K18_R_0	BY932633
E_EL_vg4M_02K19_F_0	BY924105
E_EL_vg4M_02K19_R_0	BY932634
E_EL_vg4M_02K20_F_0	BY924106
E_EL_vg4M_02K20_R_0	BY932635
E_EL_vg4M_02K21_F_0	BY924107
E_EL_vg4M_02K21_R_0	BY932636
E_EL_vg4M_02K22_F_0	BY924108
E_EL_vg4M_02K22_R_0	BY932637
E_EL_vg4M_02K23_F_0	BY924109
E_EL_vg4M_02K23_R_0	BY932638
E_EL_vg4M_02K24_F_0	BY924110
E_EL_vg4M_02K24_R_0	BY932639
E_EL_vg4M_02L01_F_0	BY924111
E_EL_vg4M_02L01_R_0	BY932640
E_EL_vg4M_02L02_R_0	BY932641
E_EL_vg4M_02L03_F_0	BY924112
E_EL_vg4M_02L03_R_0	BY932642
E_EL_vg4M_02L04_F_0	BY924113
E_EL_vg4M_02L04_R_0	BY932643
E_EL_vg4M_02L05_F_0	BY924114
E_EL_vg4M_02L05_R_0	BY932644
E_EL_vg4M_02L06_R_0	BY932645
E_EL_vg4M_02L08_R_0	BY932646
E_EL_vg4M_02L09_F_0	BY924115
E_EL_vg4M_02L09_R_0	BY932647
E_EL_vg4M_02L10_R_0	BY932648
E_EL_vg4M_02L11_F_0	BY924116
E_EL_vg4M_02L11_R_0	BY932649
E_EL_vg4M_02L12_F_0	BY924117
E_EL_vg4M_02L12_R_0	BY932650
E_EL_vg4M_02L14_F_0	BY924118
E_EL_vg4M_02L14_R_0	BY932651
E_EL_vg4M_02L15_R_0	BY932652
E_EL_vg4M_02L16_F_0	BY924119
E_EL_vg4M_02L16_R_0	BY932653
E_EL_vg4M_02L17_F_0	BY924120
E_EL_vg4M_02L17_R_0	BY932654
E_EL_vg4M_02L18_R_0	BY932655
E_EL_vg4M_02L19_F_0	BY924121
E_EL_vg4M_02L19_R_0	BY932656
E_EL_vg4M_02L20_R_0	BY932657
E_EL_vg4M_02L21_F_0	BY924122

E_EL_vg4M_02L22_F_0	BY924123
E_EL_vg4M_02L22_R_0	BY932658
E_EL_vg4M_02L24_F_0	BY924124
E_EL_vg4M_02L24_R_0	BY932659
E_EL_vg4M_02M02_F_0	BY924125
E_EL_vg4M_02M02_R_0	BY932660
E_EL_vg4M_02M03_F_0	BY924126
E_EL_vg4M_02M03_R_0	BY932661
E_EL_vg4M_02M04_F_0	BY924127
E_EL_vg4M_02M04_R_0	BY932662
E_EL_vg4M_02M05_F_0	BY924128
E_EL_vg4M_02M05_R_0	BY932663
E_EL_vg4M_02M06_F_0	BY924129
E_EL_vg4M_02M06_R_0	BY932664
E_EL_vg4M_02M07_F_0	BY924130
E_EL_vg4M_02M07_R_0	BY932665
E_EL_vg4M_02M08_F_0	BY924131
E_EL_vg4M_02M08_R_0	BY932666
E_EL_vg4M_02M09_F_0	BY924132
E_EL_vg4M_02M09_R_0	BY932667
E_EL_vg4M_02M10_F_0	BY924133
E_EL_vg4M_02M10_R_0	BY932668
E_EL_vg4M_02M11_F_0	BY924134
E_EL_vg4M_02M11_R_0	BY932669
E_EL_vg4M_02M12_R_0	BY932670
E_EL_vg4M_02M13_F_0	BY924135
E_EL_vg4M_02M13_R_0	BY932671
E_EL_vg4M_02M14_R_0	BY932672
E_EL_vg4M_02M15_F_0	BY924136
E_EL_vg4M_02M15_R_0	BY932673
E_EL_vg4M_02M16_F_0	BY924137
E_EL_vg4M_02M17_F_0	BY924138
E_EL_vg4M_02M17_R_0	BY932674
E_EL_vg4M_02M19_F_0	BY924139
E_EL_vg4M_02M20_F_0	BY924140
E_EL_vg4M_02M21_F_0	BY924141
E_EL_vg4M_02M21_R_0	BY932675
E_EL_vg4M_02M22_F_0	BY924142
E_EL_vg4M_02M22_R_0	BY932676
E_EL_vg4M_02M23_F_0	BY924143
E_EL_vg4M_02M23_R_0	BY932677
E_EL_vg4M_02N01_F_0	BY924144
E_EL_vg4M_02N01_R_0	BY932678
E_EL_vg4M_02N02_R_0	BY932679
E_EL_vg4M_02N03_F_0	BY924145
E_EL_vg4M_02N03_R_0	BY932680
E_EL_vg4M_02N04_F_0	BY924146
E_EL_vg4M_02N04_R_0	BY932681
E_EL_vg4M_02N06_F_0	BY924147
E_EL_vg4M_02N06_R_0	BY932682
E_EL_vg4M_02N07_R_0	BY932683
E_EL_vg4M_02N08_F_0	BY924148
E_EL_vg4M_02N08_R_0	BY932684
E_EL_vg4M_02N09_F_0	BY924149

E_EL_vg4M_02N09_R_0	BY932685
E_EL_vg4M_02N10_R_0	BY932686
E_EL_vg4M_02N11_F_0	BY924150
E_EL_vg4M_02N11_R_0	BY932687
E_EL_vg4M_02N12_F_0	BY924151
E_EL_vg4M_02N12_R_0	BY932688
E_EL_vg4M_02N13_F_0	BY924152
E_EL_vg4M_02N13_R_0	BY932689
E_EL_vg4M_02N14_F_0	BY924153
E_EL_vg4M_02N14_R_0	BY932690
E_EL_vg4M_02N15_F_0	BY924154
E_EL_vg4M_02N15_R_0	BY932691
E_EL_vg4M_02N16_F_0	BY924155
E_EL_vg4M_02N16_R_0	BY932692
E_EL_vg4M_02N17_F_0	BY924156
E_EL_vg4M_02N17_R_0	BY932693
E_EL_vg4M_02N18_F_0	BY924157
E_EL_vg4M_02N18_R_0	BY932694
E_EL_vg4M_02N19_F_0	BY924158
E_EL_vg4M_02N19_R_0	BY932695
E_EL_vg4M_02N20_R_0	BY932696
E_EL_vg4M_02N21_F_0	BY924159
E_EL_vg4M_02N21_R_0	BY932697
E_EL_vg4M_02N22_F_0	BY924160
E_EL_vg4M_02N22_R_0	BY932698
E_EL_vg4M_02N23_F_0	BY924161
E_EL_vg4M_02N23_R_0	BY932699
E_EL_vg4M_02N24_F_0	BY924162
E_EL_vg4M_02N24_R_0	BY932700
E_EL_vg4M_02O01_F_0	BY924163
E_EL_vg4M_02O01_R_0	BY932701
E_EL_vg4M_02O03_F_0	BY924164
E_EL_vg4M_02O03_R_0	BY932702
E_EL_vg4M_02O05_F_0	BY924165
E_EL_vg4M_02O05_R_0	BY932703
E_EL_vg4M_02O06_F_0	BY924166
E_EL_vg4M_02O07_F_0	BY924167
E_EL_vg4M_02O07_R_0	BY932704
E_EL_vg4M_02O08_F_0	BY924168
E_EL_vg4M_02O08_R_0	BY932705
E_EL_vg4M_02O09_F_0	BY924169
E_EL_vg4M_02O09_R_0	BY932706
E_EL_vg4M_02O10_F_0	BY924170
E_EL_vg4M_02O13_F_0	BY924171
E_EL_vg4M_02O13_R_0	BY932707
E_EL_vg4M_02O14_R_0	BY932708
E_EL_vg4M_02O15_R_0	BY932709
E_EL_vg4M_02O16_F_0	BY924172
E_EL_vg4M_02O16_R_0	BY932710
E_EL_vg4M_02O17_R_0	BY932711
E_EL_vg4M_02O19_F_0	BY924173
E_EL_vg4M_02O19_R_0	BY932712
E_EL_vg4M_02O20_F_0	BY924174
E_EL_vg4M_02O20_R_0	BY932713

E_EL_vg4M_02O21_F_0	BY924175
E_EL_vg4M_02O21_R_0	BY932714
E_EL_vg4M_02O22_F_0	BY924176
E_EL_vg4M_02O22_R_0	BY932715
E_EL_vg4M_02O23_F_0	BY924177
E_EL_vg4M_02O23_R_0	BY932716
E_EL_vg4M_02P01_F_0	BY924178
E_EL_vg4M_02P01_R_0	BY932717
E_EL_vg4M_02P02_R_0	BY932718
E_EL_vg4M_02P03_F_0	BY924179
E_EL_vg4M_02P03_R_0	BY932719
E_EL_vg4M_02P04_R_0	BY932720
E_EL_vg4M_02P05_R_0	BY932721
E_EL_vg4M_02P06_F_0	BY924180
E_EL_vg4M_02P06_R_0	BY932722
E_EL_vg4M_02P07_F_0	BY924181
E_EL_vg4M_02P07_R_0	BY932723
E_EL_vg4M_02P08_F_0	BY924182
E_EL_vg4M_02P08_R_0	BY932724
E_EL_vg4M_02P09_F_0	BY924183
E_EL_vg4M_02P09_R_0	BY932725
E_EL_vg4M_02P11_F_0	BY924184
E_EL_vg4M_02P11_R_0	BY932726
E_EL_vg4M_02P12_F_0	BY924185
E_EL_vg4M_02P12_R_0	BY932727
E_EL_vg4M_02P13_F_0	BY924186
E_EL_vg4M_02P13_R_0	BY932728
E_EL_vg4M_02P14_F_0	BY924187
E_EL_vg4M_02P14_R_0	BY932729
E_EL_vg4M_02P15_F_0	BY924188
E_EL_vg4M_02P15_R_0	BY932730
E_EL_vg4M_02P16_F_0	BY924189
E_EL_vg4M_02P16_R_0	BY932731
E_EL_vg4M_02P17_F_0	BY924190
E_EL_vg4M_02P17_R_0	BY932732
E_EL_vg4M_02P20_F_0	BY924191
E_EL_vg4M_02P20_R_0	BY932733
E_EL_vg4M_02P21_F_0	BY924192
E_EL_vg4M_02P21_R_0	BY932734
E_EL_vg4M_02P22_F_0	BY924193
E_EL_vg4M_02P22_R_0	BY932735
E_EL_vg4M_02P23_F_0	BY924194
E_EL_vg4M_02P23_R_0	BY932736
E_EL_vg4M_02P24_F_0	BY924195
E_EL_vg4M_02P24_R_0	BY932737
E_EL_vg4M_03A01_F_0	BY924196
E_EL_vg4M_03A02_F_0	BY924197
E_EL_vg4M_03A03_F_0	BY924198
E_EL_vg4M_03A03_R_0	BY932738
E_EL_vg4M_03A04_F_0	BY924199
E_EL_vg4M_03A04_R_0	BY932739
E_EL_vg4M_03A05_F_0	BY924200
E_EL_vg4M_03A05_R_0	BY932740
E_EL_vg4M_03A06_R_0	BY932741

E_EL_vg4M_03A08_F_0	BY924201
E_EL_vg4M_03A08_R_0	BY932742
E_EL_vg4M_03A09_F_0	BY924202
E_EL_vg4M_03A10_F_0	BY924203
E_EL_vg4M_03A10_R_0	BY932743
E_EL_vg4M_03A11_F_0	BY924204
E_EL_vg4M_03A11_R_0	BY932744
E_EL_vg4M_03A12_F_0	BY924205
E_EL_vg4M_03A12_R_0	BY932745
E_EL_vg4M_03A14_F_0	BY924206
E_EL_vg4M_03A14_R_0	BY932746
E_EL_vg4M_03A15_F_0	BY924207
E_EL_vg4M_03A15_R_0	BY932747
E_EL_vg4M_03A16_R_0	BY932748
E_EL_vg4M_03A17_F_0	BY924208
E_EL_vg4M_03A17_R_0	BY932749
E_EL_vg4M_03A18_F_0	BY924209
E_EL_vg4M_03A18_R_0	BY932750
E_EL_vg4M_03A20_F_0	BY924210
E_EL_vg4M_03A20_R_0	BY932751
E_EL_vg4M_03A21_F_0	BY924211
E_EL_vg4M_03A21_R_0	BY932752
E_EL_vg4M_03A22_F_0	BY924212
E_EL_vg4M_03A22_R_0	BY932753
E_EL_vg4M_03A23_R_0	BY932754
E_EL_vg4M_03A24_F_0	BY924213
E_EL_vg4M_03A24_R_0	BY932755
E_EL_vg4M_03B01_F_0	BY924214
E_EL_vg4M_03B01_R_0	BY932756
E_EL_vg4M_03B02_F_0	BY924215
E_EL_vg4M_03B02_R_0	BY932757
E_EL_vg4M_03B03_F_0	BY924216
E_EL_vg4M_03B03_R_0	BY932758
E_EL_vg4M_03B05_R_0	BY932759
E_EL_vg4M_03B07_F_0	BY924217
E_EL_vg4M_03B07_R_0	BY932760
E_EL_vg4M_03B08_F_0	BY924218
E_EL_vg4M_03B08_R_0	BY932761
E_EL_vg4M_03B10_F_0	BY924219
E_EL_vg4M_03B10_R_0	BY932762
E_EL_vg4M_03B11_F_0	BY924220
E_EL_vg4M_03B11_R_0	BY932763
E_EL_vg4M_03B12_F_0	BY924221
E_EL_vg4M_03B12_R_0	BY932764
E_EL_vg4M_03B13_F_0	BY924222
E_EL_vg4M_03B13_R_0	BY932765
E_EL_vg4M_03B14_F_0	BY924223
E_EL_vg4M_03B14_R_0	BY932766
E_EL_vg4M_03B15_F_0	BY924224
E_EL_vg4M_03B15_R_0	BY932767
E_EL_vg4M_03B16_F_0	BY924225
E_EL_vg4M_03B16_R_0	BY932768
E_EL_vg4M_03B18_F_0	BY924226
E_EL_vg4M_03B19_F_0	BY924227

E_EL_vg4M_03B19_R_0	BY932769
E_EL_vg4M_03B20_F_0	BY924228
E_EL_vg4M_03B20_R_0	BY932770
E_EL_vg4M_03B21_F_0	BY924229
E_EL_vg4M_03B21_R_0	BY932771
E_EL_vg4M_03B22_F_0	BY924230
E_EL_vg4M_03B22_R_0	BY932772
E_EL_vg4M_03B23_F_0	BY924231
E_EL_vg4M_03B24_F_0	BY924232
E_EL_vg4M_03B24_R_0	BY932773
E_EL_vg4M_03C01_R_0	BY932774
E_EL_vg4M_03C02_F_0	BY924233
E_EL_vg4M_03C02_R_0	BY932775
E_EL_vg4M_03C03_F_0	BY924234
E_EL_vg4M_03C03_R_0	BY932776
E_EL_vg4M_03C04_F_0	BY924235
E_EL_vg4M_03C04_R_0	BY932777
E_EL_vg4M_03C06_F_0	BY924236
E_EL_vg4M_03C07_R_0	BY932778
E_EL_vg4M_03C08_F_0	BY924237
E_EL_vg4M_03C08_R_0	BY932779
E_EL_vg4M_03C09_F_0	BY924238
E_EL_vg4M_03C09_R_0	BY932780
E_EL_vg4M_03C10_F_0	BY924239
E_EL_vg4M_03C10_R_0	BY932781
E_EL_vg4M_03C11_F_0	BY924240
E_EL_vg4M_03C11_R_0	BY932782
E_EL_vg4M_03C13_F_0	BY924241
E_EL_vg4M_03C13_R_0	BY932783
E_EL_vg4M_03C14_F_0	BY924242
E_EL_vg4M_03C14_R_0	BY932784
E_EL_vg4M_03C15_F_0	BY924243
E_EL_vg4M_03C15_R_0	BY932785
E_EL_vg4M_03C16_F_0	BY924244
E_EL_vg4M_03C16_R_0	BY932786
E_EL_vg4M_03C17_F_0	BY924245
E_EL_vg4M_03C17_R_0	BY932787
E_EL_vg4M_03C18_F_0	BY924246
E_EL_vg4M_03C18_R_0	BY932788
E_EL_vg4M_03C19_F_0	BY924247
E_EL_vg4M_03C20_F_0	BY924248
E_EL_vg4M_03C20_R_0	BY932789
E_EL_vg4M_03C21_R_0	BY932790
E_EL_vg4M_03C22_F_0	BY924249
E_EL_vg4M_03C22_R_0	BY932791
E_EL_vg4M_03C23_F_0	BY924250
E_EL_vg4M_03C23_R_0	BY932792
E_EL_vg4M_03C24_F_0	BY924251
E_EL_vg4M_03C24_R_0	BY932793
E_EL_vg4M_03D01_F_0	BY924252
E_EL_vg4M_03D01_R_0	BY932794
E_EL_vg4M_03D02_F_0	BY924253
E_EL_vg4M_03D03_F_0	BY924254
E_EL_vg4M_03D03_R_0	BY932795

E_EL_vg4M_03D04_F_0	BY924255
E_EL_vg4M_03D04_R_0	BY932796
E_EL_vg4M_03D05_F_0	BY924256
E_EL_vg4M_03D05_R_0	BY932797
E_EL_vg4M_03D06_F_0	BY924257
E_EL_vg4M_03D06_R_0	BY932798
E_EL_vg4M_03D07_F_0	BY924258
E_EL_vg4M_03D07_R_0	BY932799
E_EL_vg4M_03D08_F_0	BY924259
E_EL_vg4M_03D09_F_0	BY924260
E_EL_vg4M_03D10_R_0	BY932800
E_EL_vg4M_03D11_F_0	BY924261
E_EL_vg4M_03D11_R_0	BY932801
E_EL_vg4M_03D12_F_0	BY924262
E_EL_vg4M_03D12_R_0	BY932802
E_EL_vg4M_03D13_F_0	BY924263
E_EL_vg4M_03D13_R_0	BY932803
E_EL_vg4M_03D14_F_0	BY924264
E_EL_vg4M_03D14_R_0	BY932804
E_EL_vg4M_03D15_F_0	BY924265
E_EL_vg4M_03D15_R_0	BY932805
E_EL_vg4M_03D16_F_0	BY924266
E_EL_vg4M_03D16_R_0	BY932806
E_EL_vg4M_03D17_F_0	BY924267
E_EL_vg4M_03D18_F_0	BY924268
E_EL_vg4M_03D19_R_0	BY932807
E_EL_vg4M_03D20_F_0	BY924269
E_EL_vg4M_03D20_R_0	BY932808
E_EL_vg4M_03D21_F_0	BY924270
E_EL_vg4M_03D21_R_0	BY932809
E_EL_vg4M_03D22_F_0	BY924271
E_EL_vg4M_03D22_R_0	BY932810
E_EL_vg4M_03D23_F_0	BY924272
E_EL_vg4M_03D23_R_0	BY932811
E_EL_vg4M_03D24_F_0	BY924273
E_EL_vg4M_03D24_R_0	BY932812
E_EL_vg4M_03E01_R_0	BY932813
E_EL_vg4M_03E02_F_0	BY924274
E_EL_vg4M_03E02_R_0	BY932814
E_EL_vg4M_03E03_F_0	BY924275
E_EL_vg4M_03E03_R_0	BY932815
E_EL_vg4M_03E04_F_0	BY924276
E_EL_vg4M_03E04_R_0	BY932816
E_EL_vg4M_03E05_F_0	BY924277
E_EL_vg4M_03E06_F_0	BY924278
E_EL_vg4M_03E07_R_0	BY932817
E_EL_vg4M_03E08_F_0	BY924279
E_EL_vg4M_03E08_R_0	BY932818
E_EL_vg4M_03E10_F_0	BY924280
E_EL_vg4M_03E10_R_0	BY932819
E_EL_vg4M_03E11_F_0	BY924281
E_EL_vg4M_03E11_R_0	BY932820
E_EL_vg4M_03E13_R_0	BY932821
E_EL_vg4M_03E14_F_0	BY924282

E_EL_vg4M_03E14_R_0	BY932822
E_EL_vg4M_03E15_F_0	BY924283
E_EL_vg4M_03E15_R_0	BY932823
E_EL_vg4M_03E16_F_0	BY924284
E_EL_vg4M_03E16_R_0	BY932824
E_EL_vg4M_03E17_F_0	BY924285
E_EL_vg4M_03E17_R_0	BY932825
E_EL_vg4M_03E18_F_0	BY924286
E_EL_vg4M_03E18_R_0	BY932826
E_EL_vg4M_03E20_F_0	BY924287
E_EL_vg4M_03E20_R_0	BY932827
E_EL_vg4M_03E21_F_0	BY924288
E_EL_vg4M_03E21_R_0	BY932828
E_EL_vg4M_03E22_F_0	BY924289
E_EL_vg4M_03E22_R_0	BY932829
E_EL_vg4M_03E23_F_0	BY924290
E_EL_vg4M_03E23_R_0	BY932830
E_EL_vg4M_03E24_F_0	BY924291
E_EL_vg4M_03F01_F_0	BY924292
E_EL_vg4M_03F01_R_0	BY932831
E_EL_vg4M_03F02_F_0	BY924293
E_EL_vg4M_03F02_R_0	BY932832
E_EL_vg4M_03F03_F_0	BY924294
E_EL_vg4M_03F03_R_0	BY932833
E_EL_vg4M_03F04_F_0	BY924295
E_EL_vg4M_03F04_R_0	BY932834
E_EL_vg4M_03F05_F_0	BY924296
E_EL_vg4M_03F05_R_0	BY932835
E_EL_vg4M_03F06_R_0	BY932836
E_EL_vg4M_03F07_F_0	BY924297
E_EL_vg4M_03F08_R_0	BY932837
E_EL_vg4M_03F09_F_0	BY924298
E_EL_vg4M_03F09_R_0	BY932838
E_EL_vg4M_03F11_F_0	BY924299
E_EL_vg4M_03F11_R_0	BY932839
E_EL_vg4M_03F12_F_0	BY924300
E_EL_vg4M_03F13_F_0	BY924301
E_EL_vg4M_03F13_R_0	BY932840
E_EL_vg4M_03F14_F_0	BY924302
E_EL_vg4M_03F14_R_0	BY932841
E_EL_vg4M_03F15_F_0	BY924303
E_EL_vg4M_03F15_R_0	BY932842
E_EL_vg4M_03F16_F_0	BY924304
E_EL_vg4M_03F16_R_0	BY932843
E_EL_vg4M_03F17_F_0	BY924305
E_EL_vg4M_03F17_R_0	BY932844
E_EL_vg4M_03F19_F_0	BY924306
E_EL_vg4M_03F19_R_0	BY932845
E_EL_vg4M_03F21_F_0	BY924307
E_EL_vg4M_03F21_R_0	BY932846
E_EL_vg4M_03F22_F_0	BY924308
E_EL_vg4M_03F22_R_0	BY932847
E_EL_vg4M_03F23_F_0	BY924309
E_EL_vg4M_03F23_R_0	BY932848

E_EL_vg4M_03F24_F_0	BY924310
E_EL_vg4M_03F24_R_0	BY932849
E_EL_vg4M_03G02_F_0	BY924311
E_EL_vg4M_03G02_R_0	BY932850
E_EL_vg4M_03G03_F_0	BY924312
E_EL_vg4M_03G03_R_0	BY932851
E_EL_vg4M_03G05_F_0	BY924313
E_EL_vg4M_03G05_R_0	BY932852
E_EL_vg4M_03G06_F_0	BY924314
E_EL_vg4M_03G06_R_0	BY932853
E_EL_vg4M_03G07_F_0	BY924315
E_EL_vg4M_03G07_R_0	BY932854
E_EL_vg4M_03G08_F_0	BY924316
E_EL_vg4M_03G09_F_0	BY924317
E_EL_vg4M_03G10_F_0	BY924318
E_EL_vg4M_03G10_R_0	BY932855
E_EL_vg4M_03G11_F_0	BY924319
E_EL_vg4M_03G11_R_0	BY932856
E_EL_vg4M_03G12_F_0	BY924320
E_EL_vg4M_03G12_R_0	BY932857
E_EL_vg4M_03G13_F_0	BY924321
E_EL_vg4M_03G13_R_0	BY932858
E_EL_vg4M_03G14_F_0	BY924322
E_EL_vg4M_03G14_R_0	BY932859
E_EL_vg4M_03G15_F_0	BY924323
E_EL_vg4M_03G15_R_0	BY932860
E_EL_vg4M_03G16_F_0	BY924324
E_EL_vg4M_03G18_F_0	BY924325
E_EL_vg4M_03G19_F_0	BY924326
E_EL_vg4M_03G19_R_0	BY932861
E_EL_vg4M_03G20_F_0	BY924327
E_EL_vg4M_03G21_F_0	BY924328
E_EL_vg4M_03G21_R_0	BY932862
E_EL_vg4M_03G22_F_0	BY924329
E_EL_vg4M_03G23_F_0	BY924330
E_EL_vg4M_03G23_R_0	BY932863
E_EL_vg4M_03G24_F_0	BY924331
E_EL_vg4M_03G24_R_0	BY932864
E_EL_vg4M_03H01_F_0	BY924332
E_EL_vg4M_03H01_R_0	BY932865
E_EL_vg4M_03H02_F_0	BY924333
E_EL_vg4M_03H03_F_0	BY924334
E_EL_vg4M_03H03_R_0	BY932866
E_EL_vg4M_03H04_F_0	BY924335
E_EL_vg4M_03H05_F_0	BY924336
E_EL_vg4M_03H05_R_0	BY932867
E_EL_vg4M_03H07_F_0	BY924337
E_EL_vg4M_03H07_R_0	BY932868
E_EL_vg4M_03H08_F_0	BY924338
E_EL_vg4M_03H08_R_0	BY932869
E_EL_vg4M_03H09_F_0	BY924339
E_EL_vg4M_03H09_R_0	BY932870
E_EL_vg4M_03H10_R_0	BY932871
E_EL_vg4M_03H12_F_0	BY924340

E_EL_vg4M_03H12_R_0	BY932872
E_EL_vg4M_03H13_F_0	BY924341
E_EL_vg4M_03H13_R_0	BY932873
E_EL_vg4M_03H14_F_0	BY924342
E_EL_vg4M_03H14_R_0	BY932874
E_EL_vg4M_03H15_F_0	BY924343
E_EL_vg4M_03H15_R_0	BY932875
E_EL_vg4M_03H16_F_0	BY924344
E_EL_vg4M_03H16_R_0	BY932876
E_EL_vg4M_03H17_F_0	BY924345
E_EL_vg4M_03H17_R_0	BY932877
E_EL_vg4M_03H19_F_0	BY924346
E_EL_vg4M_03H19_R_0	BY932878
E_EL_vg4M_03H20_F_0	BY924347
E_EL_vg4M_03H20_R_0	BY932879
E_EL_vg4M_03H21_F_0	BY924348
E_EL_vg4M_03H21_R_0	BY932880
E_EL_vg4M_03H22_F_0	BY924349
E_EL_vg4M_03H22_R_0	BY932881
E_EL_vg4M_03H23_F_0	BY924350
E_EL_vg4M_03H23_R_0	BY932882
E_EL_vg4M_03H24_F_0	BY924351
E_EL_vg4M_03H24_R_0	BY932883
E_EL_vg4M_03I01_F_0	BY924352
E_EL_vg4M_03I01_R_0	BY932884
E_EL_vg4M_03I02_F_0	BY924353
E_EL_vg4M_03I02_R_0	BY932885
E_EL_vg4M_03I03_F_0	BY924354
E_EL_vg4M_03I04_R_0	BY932886
E_EL_vg4M_03I05_F_0	BY924355
E_EL_vg4M_03I05_R_0	BY932887
E_EL_vg4M_03I06_F_0	BY924356
E_EL_vg4M_03I06_R_0	BY932888
E_EL_vg4M_03I08_R_0	BY932889
E_EL_vg4M_03I09_F_0	BY924357
E_EL_vg4M_03I09_R_0	BY932890
E_EL_vg4M_03I10_F_0	BY924358
E_EL_vg4M_03I10_R_0	BY932891
E_EL_vg4M_03I11_F_0	BY924359
E_EL_vg4M_03I11_R_0	BY932892
E_EL_vg4M_03I12_F_0	BY924360
E_EL_vg4M_03I12_R_0	BY932893
E_EL_vg4M_03I13_F_0	BY924361
E_EL_vg4M_03I15_F_0	BY924362
E_EL_vg4M_03I15_R_0	BY932894
E_EL_vg4M_03I17_F_0	BY924363
E_EL_vg4M_03I17_R_0	BY932895
E_EL_vg4M_03I18_F_0	BY924364
E_EL_vg4M_03I18_R_0	BY932896
E_EL_vg4M_03I19_F_0	BY924365
E_EL_vg4M_03I19_R_0	BY932897
E_EL_vg4M_03I20_F_0	BY924366
E_EL_vg4M_03I20_R_0	BY932898
E_EL_vg4M_03I21_F_0	BY924367

E_EL_vg4M_03I21_R_0	BY932899
E_EL_vg4M_03I22_F_0	BY924368
E_EL_vg4M_03I22_R_0	BY932900
E_EL_vg4M_03I23_F_0	BY924369
E_EL_vg4M_03I24_F_0	BY924370
E_EL_vg4M_03J01_F_0	BY924371
E_EL_vg4M_03J01_R_0	BY932901
E_EL_vg4M_03J02_F_0	BY924372
E_EL_vg4M_03J04_F_0	BY924373
E_EL_vg4M_03J04_R_0	BY932902
E_EL_vg4M_03J05_F_0	BY924374
E_EL_vg4M_03J05_R_0	BY932903
E_EL_vg4M_03J06_F_0	BY924375
E_EL_vg4M_03J06_R_0	BY932904
E_EL_vg4M_03J07_F_0	BY924376
E_EL_vg4M_03J07_R_0	BY932905
E_EL_vg4M_03J08_F_0	BY924377
E_EL_vg4M_03J08_R_0	BY932906
E_EL_vg4M_03J09_F_0	BY924378
E_EL_vg4M_03J09_R_0	BY932907
E_EL_vg4M_03J11_F_0	BY924379
E_EL_vg4M_03J11_R_0	BY932908
E_EL_vg4M_03J12_F_0	BY924380
E_EL_vg4M_03J12_R_0	BY932909
E_EL_vg4M_03J13_F_0	BY924381
E_EL_vg4M_03J13_R_0	BY932910
E_EL_vg4M_03J15_F_0	BY924382
E_EL_vg4M_03J15_R_0	BY932911
E_EL_vg4M_03J16_F_0	BY924383
E_EL_vg4M_03J16_R_0	BY932912
E_EL_vg4M_03J17_R_0	BY932913
E_EL_vg4M_03J18_R_0	BY932914
E_EL_vg4M_03J19_F_0	BY924384
E_EL_vg4M_03J19_R_0	BY932915
E_EL_vg4M_03J20_F_0	BY924385
E_EL_vg4M_03J21_F_0	BY924386
E_EL_vg4M_03J21_R_0	BY932916
E_EL_vg4M_03J22_F_0	BY924387
E_EL_vg4M_03J22_R_0	BY932917
E_EL_vg4M_03J23_F_0	BY924388
E_EL_vg4M_03J23_R_0	BY932918
E_EL_vg4M_03J24_F_0	BY924389
E_EL_vg4M_03J24_R_0	BY932919
E_EL_vg4M_03K01_F_0	BY924390
E_EL_vg4M_03K02_F_0	BY924391
E_EL_vg4M_03K02_R_0	BY932920
E_EL_vg4M_03K03_F_0	BY924392
E_EL_vg4M_03K03_R_0	BY932921
E_EL_vg4M_03K04_F_0	BY924393
E_EL_vg4M_03K04_R_0	BY932922
E_EL_vg4M_03K05_F_0	BY924394
E_EL_vg4M_03K05_R_0	BY932923
E_EL_vg4M_03K06_F_0	BY924395
E_EL_vg4M_03K06_R_0	BY932924

E_EL_vg4M_03K07_R_0	BY932925
E_EL_vg4M_03K08_F_0	BY924396
E_EL_vg4M_03K08_R_0	BY932926
E_EL_vg4M_03K09_F_0	BY924397
E_EL_vg4M_03K09_R_0	BY932927
E_EL_vg4M_03K10_F_0	BY924398
E_EL_vg4M_03K10_R_0	BY932928
E_EL_vg4M_03K11_F_0	BY924399
E_EL_vg4M_03K11_R_0	BY932929
E_EL_vg4M_03K12_F_0	BY924400
E_EL_vg4M_03K13_F_0	BY924401
E_EL_vg4M_03K13_R_0	BY932930
E_EL_vg4M_03K14_F_0	BY924402
E_EL_vg4M_03K14_R_0	BY932931
E_EL_vg4M_03K15_F_0	BY924403
E_EL_vg4M_03K15_R_0	BY932932
E_EL_vg4M_03K16_F_0	BY924404
E_EL_vg4M_03K16_R_0	BY932933
E_EL_vg4M_03K17_F_0	BY924405
E_EL_vg4M_03K17_R_0	BY932934
E_EL_vg4M_03K18_F_0	BY924406
E_EL_vg4M_03K18_R_0	BY932935
E_EL_vg4M_03K19_F_0	BY924407
E_EL_vg4M_03K19_R_0	BY932936
E_EL_vg4M_03K20_F_0	BY924408
E_EL_vg4M_03K20_R_0	BY932937
E_EL_vg4M_03K21_F_0	BY924409
E_EL_vg4M_03K21_R_0	BY932938
E_EL_vg4M_03K22_F_0	BY924410
E_EL_vg4M_03K22_R_0	BY932939
E_EL_vg4M_03K23_F_0	BY924411
E_EL_vg4M_03K24_F_0	BY924412
E_EL_vg4M_03K24_R_0	BY932940
E_EL_vg4M_03L01_F_0	BY924413
E_EL_vg4M_03L03_F_0	BY924414
E_EL_vg4M_03L03_R_0	BY932941
E_EL_vg4M_03L04_F_0	BY924415
E_EL_vg4M_03L04_R_0	BY932942
E_EL_vg4M_03L05_F_0	BY924416
E_EL_vg4M_03L05_R_0	BY932943
E_EL_vg4M_03L07_F_0	BY924417
E_EL_vg4M_03L07_R_0	BY932944
E_EL_vg4M_03L08_F_0	BY924418
E_EL_vg4M_03L08_R_0	BY932945
E_EL_vg4M_03L09_F_0	BY924419
E_EL_vg4M_03L10_R_0	BY932946
E_EL_vg4M_03L11_F_0	BY924420
E_EL_vg4M_03L11_R_0	BY932947
E_EL_vg4M_03L12_F_0	BY924421
E_EL_vg4M_03L13_F_0	BY924422
E_EL_vg4M_03L13_R_0	BY932948
E_EL_vg4M_03L15_F_0	BY924423
E_EL_vg4M_03L15_R_0	BY932949
E_EL_vg4M_03L16_F_0	BY924424

E_EL_vg4M_03L16_R_0	BY932950
E_EL_vg4M_03L17_F_0	BY924425
E_EL_vg4M_03L17_R_0	BY932951
E_EL_vg4M_03L19_F_0	BY924426
E_EL_vg4M_03L20_F_0	BY924427
E_EL_vg4M_03L20_R_0	BY932952
E_EL_vg4M_03L21_F_0	BY924428
E_EL_vg4M_03L21_R_0	BY932953
E_EL_vg4M_03L22_F_0	BY924429
E_EL_vg4M_03L22_R_0	BY932954
E_EL_vg4M_03L23_R_0	BY932955
E_EL_vg4M_03L24_F_0	BY924430
E_EL_vg4M_03L24_R_0	BY932956
E_EL_vg4M_03M01_F_0	BY924431
E_EL_vg4M_03M01_R_0	BY932957
E_EL_vg4M_03M03_F_0	BY924432
E_EL_vg4M_03M03_R_0	BY932958
E_EL_vg4M_03M04_F_0	BY924433
E_EL_vg4M_03M04_R_0	BY932959
E_EL_vg4M_03M05_F_0	BY924434
E_EL_vg4M_03M05_R_0	BY932960
E_EL_vg4M_03M06_F_0	BY924435
E_EL_vg4M_03M06_R_0	BY932961
E_EL_vg4M_03M07_R_0	BY932962
E_EL_vg4M_03M08_F_0	BY924436
E_EL_vg4M_03M08_R_0	BY932963
E_EL_vg4M_03M09_F_0	BY924437
E_EL_vg4M_03M09_R_0	BY932964
E_EL_vg4M_03M10_F_0	BY924438
E_EL_vg4M_03M10_R_0	BY932965
E_EL_vg4M_03M11_F_0	BY924439
E_EL_vg4M_03M11_R_0	BY932966
E_EL_vg4M_03M12_F_0	BY924440
E_EL_vg4M_03M12_R_0	BY932967
E_EL_vg4M_03M13_F_0	BY924441
E_EL_vg4M_03M13_R_0	BY932968
E_EL_vg4M_03M14_F_0	BY924442
E_EL_vg4M_03M14_R_0	BY932969
E_EL_vg4M_03M15_F_0	BY924443
E_EL_vg4M_03M16_F_0	BY924444
E_EL_vg4M_03M17_F_0	BY924445
E_EL_vg4M_03M17_R_0	BY932970
E_EL_vg4M_03M18_F_0	BY924446
E_EL_vg4M_03M19_F_0	BY924447
E_EL_vg4M_03M20_F_0	BY924448
E_EL_vg4M_03M20_R_0	BY932971
E_EL_vg4M_03M21_F_0	BY924449
E_EL_vg4M_03M21_R_0	BY932972
E_EL_vg4M_03M24_F_0	BY924450
E_EL_vg4M_03M24_R_0	BY932973
E_EL_vg4M_03N01_F_0	BY924451
E_EL_vg4M_03N02_R_0	BY932974
E_EL_vg4M_03N03_F_0	BY924452
E_EL_vg4M_03N03_R_0	BY932975

E_EL_vg4M_03N04_F_0	BY924453
E_EL_vg4M_03N05_R_0	BY932976
E_EL_vg4M_03N06_R_0	BY932977
E_EL_vg4M_03N07_F_0	BY924454
E_EL_vg4M_03N07_R_0	BY932978
E_EL_vg4M_03N08_F_0	BY924455
E_EL_vg4M_03N08_R_0	BY932979
E_EL_vg4M_03N09_F_0	BY924456
E_EL_vg4M_03N09_R_0	BY932980
E_EL_vg4M_03N10_F_0	BY924457
E_EL_vg4M_03N10_R_0	BY932981
E_EL_vg4M_03N12_F_0	BY924458
E_EL_vg4M_03N12_R_0	BY932982
E_EL_vg4M_03N13_F_0	BY924459
E_EL_vg4M_03N13_R_0	BY932983
E_EL_vg4M_03N14_F_0	BY924460
E_EL_vg4M_03N15_F_0	BY924461
E_EL_vg4M_03N15_R_0	BY932984
E_EL_vg4M_03N16_F_0	BY924462
E_EL_vg4M_03N16_R_0	BY932985
E_EL_vg4M_03N17_F_0	BY924463
E_EL_vg4M_03N17_R_0	BY932986
E_EL_vg4M_03N18_F_0	BY924464
E_EL_vg4M_03N18_R_0	BY932987
E_EL_vg4M_03N19_F_0	BY924465
E_EL_vg4M_03N19_R_0	BY932988
E_EL_vg4M_03N20_F_0	BY924466
E_EL_vg4M_03N20_R_0	BY932989
E_EL_vg4M_03N21_F_0	BY924467
E_EL_vg4M_03N23_F_0	BY924468
E_EL_vg4M_03N23_R_0	BY932990
E_EL_vg4M_03N24_F_0	BY924469
E_EL_vg4M_03N24_R_0	BY932991
E_EL_vg4M_03O01_F_0	BY924470
E_EL_vg4M_03O01_R_0	BY932992
E_EL_vg4M_03O03_F_0	BY924471
E_EL_vg4M_03O03_R_0	BY932993
E_EL_vg4M_03O04_F_0	BY924472
E_EL_vg4M_03O04_R_0	BY932994
E_EL_vg4M_03O05_F_0	BY924473
E_EL_vg4M_03O06_R_0	BY932995
E_EL_vg4M_03O07_F_0	BY924474
E_EL_vg4M_03O08_F_0	BY924475
E_EL_vg4M_03O08_R_0	BY932996
E_EL_vg4M_03O09_F_0	BY924476
E_EL_vg4M_03O09_R_0	BY932997
E_EL_vg4M_03O10_R_0	BY932998
E_EL_vg4M_03O11_F_0	BY924477
E_EL_vg4M_03O11_R_0	BY932999
E_EL_vg4M_03O12_R_0	BY933000
E_EL_vg4M_03O14_F_0	BY924478
E_EL_vg4M_03O14_R_0	BY933001
E_EL_vg4M_03O15_F_0	BY924479
E_EL_vg4M_03O15_R_0	BY933002

E_EL_vg4M_03O16_F_0	BY924480
E_EL_vg4M_03O16_R_0	BY933003
E_EL_vg4M_03O17_F_0	BY924481
E_EL_vg4M_03O17_R_0	BY933004
E_EL_vg4M_03O19_F_0	BY924482
E_EL_vg4M_03O19_R_0	BY933005
E_EL_vg4M_03O20_F_0	BY924483
E_EL_vg4M_03O20_R_0	BY933006
E_EL_vg4M_03O21_F_0	BY924484
E_EL_vg4M_03O21_R_0	BY933007
E_EL_vg4M_03O22_F_0	BY924485
E_EL_vg4M_03O22_R_0	BY933008
E_EL_vg4M_03O23_F_0	BY924486
E_EL_vg4M_03O23_R_0	BY933009
E_EL_vg4M_03O24_R_0	BY933010
E_EL_vg4M_03P01_F_0	BY924487
E_EL_vg4M_03P01_R_0	BY933011
E_EL_vg4M_03P02_R_0	BY933012
E_EL_vg4M_03P03_F_0	BY924488
E_EL_vg4M_03P05_F_0	BY924489
E_EL_vg4M_03P06_R_0	BY933013
E_EL_vg4M_03P07_F_0	BY924490
E_EL_vg4M_03P07_R_0	BY933014
E_EL_vg4M_03P08_F_0	BY924491
E_EL_vg4M_03P09_R_0	BY933015
E_EL_vg4M_03P10_F_0	BY924492
E_EL_vg4M_03P10_R_0	BY933016
E_EL_vg4M_03P11_F_0	BY924493
E_EL_vg4M_03P11_R_0	BY933017
E_EL_vg4M_03P13_F_0	BY924494
E_EL_vg4M_03P13_R_0	BY933018
E_EL_vg4M_03P14_R_0	BY933019
E_EL_vg4M_03P15_F_0	BY924495
E_EL_vg4M_03P15_R_0	BY933020
E_EL_vg4M_03P16_F_0	BY924496
E_EL_vg4M_03P16_R_0	BY933021
E_EL_vg4M_03P17_F_0	BY924497
E_EL_vg4M_03P17_R_0	BY933022
E_EL_vg4M_03P18_R_0	BY933023
E_EL_vg4M_03P19_F_0	BY924498
E_EL_vg4M_03P19_R_0	BY933024
E_EL_vg4M_03P20_F_0	BY924499
E_EL_vg4M_03P20_R_0	BY933025
E_EL_vg4M_03P21_F_0	BY924500
E_EL_vg4M_03P21_R_0	BY933026
E_EL_vg4M_03P22_F_0	BY924501
E_EL_vg4M_03P22_R_0	BY933027
E_EL_vg4M_03P24_F_0	BY924502
E_EL_vg4M_03P24_R_0	BY933028
E_EL_vg4M_04A01_F_0	BY924503
E_EL_vg4M_04A01_R_0	BY933029
E_EL_vg4M_04A02_F_0	BY924504
E_EL_vg4M_04A02_R_0	BY933030
E_EL_vg4M_04A03_F_0	BY924505

E_EL_vg4M_04A03_R_0	BY933031
E_EL_vg4M_04A04_F_0	BY924506
E_EL_vg4M_04A04_R_0	BY933032
E_EL_vg4M_04A05_F_0	BY924507
E_EL_vg4M_04A05_R_0	BY933033
E_EL_vg4M_04A06_F_0	BY924508
E_EL_vg4M_04A06_R_0	BY933034
E_EL_vg4M_04A07_F_0	BY924509
E_EL_vg4M_04A07_R_0	BY933035
E_EL_vg4M_04A08_F_0	BY924510
E_EL_vg4M_04A08_R_0	BY933036
E_EL_vg4M_04A09_F_0	BY924511
E_EL_vg4M_04A09_R_0	BY933037
E_EL_vg4M_04A10_F_0	BY924512
E_EL_vg4M_04A10_R_0	BY933038
E_EL_vg4M_04A11_F_0	BY924513
E_EL_vg4M_04A11_R_0	BY933039
E_EL_vg4M_04A12_F_0	BY924514
E_EL_vg4M_04A12_R_0	BY933040
E_EL_vg4M_04A14_F_0	BY924515
E_EL_vg4M_04A14_R_0	BY933041
E_EL_vg4M_04A15_F_0	BY924516
E_EL_vg4M_04A15_R_0	BY933042
E_EL_vg4M_04A16_R_0	BY933043
E_EL_vg4M_04A17_F_0	BY924517
E_EL_vg4M_04A17_R_0	BY933044
E_EL_vg4M_04A18_F_0	BY924518
E_EL_vg4M_04A18_R_0	BY933045
E_EL_vg4M_04A20_R_0	BY933046
E_EL_vg4M_04A21_F_0	BY924519
E_EL_vg4M_04A21_R_0	BY933047
E_EL_vg4M_04A22_F_0	BY924520
E_EL_vg4M_04A22_R_0	BY933048
E_EL_vg4M_04A23_F_0	BY924521
E_EL_vg4M_04A23_R_0	BY933049
E_EL_vg4M_04A24_F_0	BY924522
E_EL_vg4M_04A24_R_0	BY933050
E_EL_vg4M_04B02_F_0	BY924523
E_EL_vg4M_04B02_R_0	BY933051
E_EL_vg4M_04B03_F_0	BY924524
E_EL_vg4M_04B03_R_0	BY933052
E_EL_vg4M_04B04_F_0	BY924525
E_EL_vg4M_04B04_R_0	BY933053
E_EL_vg4M_04B05_R_0	BY933054
E_EL_vg4M_04B06_F_0	BY924526
E_EL_vg4M_04B06_R_0	BY933055
E_EL_vg4M_04B07_F_0	BY924527
E_EL_vg4M_04B07_R_0	BY933056
E_EL_vg4M_04B08_F_0	BY924528
E_EL_vg4M_04B08_R_0	BY933057
E_EL_vg4M_04B09_R_0	BY933058
E_EL_vg4M_04B10_F_0	BY924529
E_EL_vg4M_04B10_R_0	BY933059
E_EL_vg4M_04B12_F_0	BY924530

E_EL_vg4M_04B12_R_0	BY933060
E_EL_vg4M_04B14_F_0	BY924531
E_EL_vg4M_04B14_R_0	BY933061
E_EL_vg4M_04B15_R_0	BY933062
E_EL_vg4M_04B16_F_0	BY924532
E_EL_vg4M_04B17_F_0	BY924533
E_EL_vg4M_04B17_R_0	BY933063
E_EL_vg4M_04B18_F_0	BY924534
E_EL_vg4M_04B18_R_0	BY933064
E_EL_vg4M_04B19_F_0	BY924535
E_EL_vg4M_04B19_R_0	BY933065
E_EL_vg4M_04B20_F_0	BY924536
E_EL_vg4M_04B20_R_0	BY933066
E_EL_vg4M_04B21_F_0	BY924537
E_EL_vg4M_04B21_R_0	BY933067
E_EL_vg4M_04B22_F_0	BY924538
E_EL_vg4M_04B22_R_0	BY933068
E_EL_vg4M_04B23_F_0	BY924539
E_EL_vg4M_04B23_R_0	BY933069
E_EL_vg4M_04B24_F_0	BY924540
E_EL_vg4M_04B24_R_0	BY933070
E_EL_vg4M_04C02_F_0	BY924541
E_EL_vg4M_04C02_R_0	BY933071
E_EL_vg4M_04C03_R_0	BY933072
E_EL_vg4M_04C04_R_0	BY933073
E_EL_vg4M_04C05_F_0	BY924542
E_EL_vg4M_04C06_F_0	BY924543
E_EL_vg4M_04C06_R_0	BY933074
E_EL_vg4M_04C08_F_0	BY924544
E_EL_vg4M_04C08_R_0	BY933075
E_EL_vg4M_04C09_R_0	BY933076
E_EL_vg4M_04C11_F_0	BY924545
E_EL_vg4M_04C11_R_0	BY933077
E_EL_vg4M_04C12_R_0	BY933078
E_EL_vg4M_04C13_R_0	BY933079
E_EL_vg4M_04C14_F_0	BY924546
E_EL_vg4M_04C14_R_0	BY933080
E_EL_vg4M_04C15_F_0	BY924547
E_EL_vg4M_04C15_R_0	BY933081
E_EL_vg4M_04C17_R_0	BY933082
E_EL_vg4M_04C18_F_0	BY924548
E_EL_vg4M_04C18_R_0	BY933083
E_EL_vg4M_04C19_F_0	BY924549
E_EL_vg4M_04C19_R_0	BY933084
E_EL_vg4M_04C20_F_0	BY924550
E_EL_vg4M_04C20_R_0	BY933085
E_EL_vg4M_04C21_R_0	BY933086
E_EL_vg4M_04C22_R_0	BY933087
E_EL_vg4M_04C23_F_0	BY924551
E_EL_vg4M_04C23_R_0	BY933088
E_EL_vg4M_04C24_R_0	BY933089
E_EL_vg4M_04D01_R_0	BY933090
E_EL_vg4M_04D02_F_0	BY924552
E_EL_vg4M_04D02_R_0	BY933091

E_EL_vg4M_04D03_F_0	BY924553
E_EL_vg4M_04D03_R_0	BY933092
E_EL_vg4M_04D04_F_0	BY924554
E_EL_vg4M_04D04_R_0	BY933093
E_EL_vg4M_04D05_F_0	BY924555
E_EL_vg4M_04D05_R_0	BY933094
E_EL_vg4M_04D06_R_0	BY933095
E_EL_vg4M_04D07_F_0	BY924556
E_EL_vg4M_04D07_R_0	BY933096
E_EL_vg4M_04D08_F_0	BY924557
E_EL_vg4M_04D08_R_0	BY933097
E_EL_vg4M_04D09_F_0	BY924558
E_EL_vg4M_04D09_R_0	BY933098
E_EL_vg4M_04D10_F_0	BY924559
E_EL_vg4M_04D10_R_0	BY933099
E_EL_vg4M_04D11_F_0	BY924560
E_EL_vg4M_04D11_R_0	BY933100
E_EL_vg4M_04D12_F_0	BY924561
E_EL_vg4M_04D12_R_0	BY933101
E_EL_vg4M_04D13_R_0	BY933102
E_EL_vg4M_04D14_F_0	BY924562
E_EL_vg4M_04D14_R_0	BY933103
E_EL_vg4M_04D15_F_0	BY924563
E_EL_vg4M_04D15_R_0	BY933104
E_EL_vg4M_04D16_R_0	BY933105
E_EL_vg4M_04D17_F_0	BY924564
E_EL_vg4M_04D17_R_0	BY933106
E_EL_vg4M_04D18_R_0	BY933107
E_EL_vg4M_04D21_R_0	BY933108
E_EL_vg4M_04D22_F_0	BY924565
E_EL_vg4M_04D22_R_0	BY933109
E_EL_vg4M_04D23_F_0	BY924566
E_EL_vg4M_04D23_R_0	BY933110
E_EL_vg4M_04D24_F_0	BY924567
E_EL_vg4M_04D24_R_0	BY933111
E_EL_vg4M_04E01_R_0	BY933112
E_EL_vg4M_04E03_F_0	BY924568
E_EL_vg4M_04E03_R_0	BY933113
E_EL_vg4M_04E04_F_0	BY924569
E_EL_vg4M_04E04_R_0	BY933114
E_EL_vg4M_04E05_R_0	BY933115
E_EL_vg4M_04E07_F_0	BY924570
E_EL_vg4M_04E07_R_0	BY933116
E_EL_vg4M_04E08_F_0	BY924571
E_EL_vg4M_04E08_R_0	BY933117
E_EL_vg4M_04E09_F_0	BY924572
E_EL_vg4M_04E10_F_0	BY924573
E_EL_vg4M_04E10_R_0	BY933118
E_EL_vg4M_04E11_F_0	BY924574
E_EL_vg4M_04E11_R_0	BY933119
E_EL_vg4M_04E12_R_0	BY933120
E_EL_vg4M_04E14_F_0	BY924575
E_EL_vg4M_04E14_R_0	BY933121
E_EL_vg4M_04E15_F_0	BY924576

E_EL_vg4M_04E15_R_0	BY933122
E_EL_vg4M_04E16_R_0	BY933123
E_EL_vg4M_04E18_R_0	BY933124
E_EL_vg4M_04E19_F_0	BY924577
E_EL_vg4M_04E19_R_0	BY933125
E_EL_vg4M_04E20_F_0	BY924578
E_EL_vg4M_04E20_R_0	BY933126
E_EL_vg4M_04E21_R_0	BY933127
E_EL_vg4M_04E22_F_0	BY924579
E_EL_vg4M_04E23_F_0	BY924580
E_EL_vg4M_04E24_R_0	BY933128
E_EL_vg4M_04F01_F_0	BY924581
E_EL_vg4M_04F01_R_0	BY933129
E_EL_vg4M_04F02_F_0	BY924582
E_EL_vg4M_04F02_R_0	BY933130
E_EL_vg4M_04F03_F_0	BY924583
E_EL_vg4M_04F03_R_0	BY933131
E_EL_vg4M_04F04_F_0	BY924584
E_EL_vg4M_04F04_R_0	BY933132
E_EL_vg4M_04F05_F_0	BY924585
E_EL_vg4M_04F05_R_0	BY933133
E_EL_vg4M_04F06_F_0	BY924586
E_EL_vg4M_04F06_R_0	BY933134
E_EL_vg4M_04F07_F_0	BY924587
E_EL_vg4M_04F07_R_0	BY933135
E_EL_vg4M_04F08_F_0	BY924588
E_EL_vg4M_04F09_F_0	BY924589
E_EL_vg4M_04F09_R_0	BY933136
E_EL_vg4M_04F10_F_0	BY924590
E_EL_vg4M_04F10_R_0	BY933137
E_EL_vg4M_04F11_F_0	BY924591
E_EL_vg4M_04F11_R_0	BY933138
E_EL_vg4M_04F12_F_0	BY924592
E_EL_vg4M_04F12_R_0	BY933139
E_EL_vg4M_04F13_R_0	BY933140
E_EL_vg4M_04F14_F_0	BY924593
E_EL_vg4M_04F15_F_0	BY924594
E_EL_vg4M_04F15_R_0	BY933141
E_EL_vg4M_04F16_F_0	BY924595
E_EL_vg4M_04F16_R_0	BY933142
E_EL_vg4M_04F17_F_0	BY924596
E_EL_vg4M_04F17_R_0	BY933143
E_EL_vg4M_04F19_F_0	BY924597
E_EL_vg4M_04F19_R_0	BY933144
E_EL_vg4M_04F20_F_0	BY924598
E_EL_vg4M_04F20_R_0	BY933145
E_EL_vg4M_04F21_F_0	BY924599
E_EL_vg4M_04F21_R_0	BY933146
E_EL_vg4M_04F23_F_0	BY924600
E_EL_vg4M_04F23_R_0	BY933147
E_EL_vg4M_04F24_F_0	BY924601
E_EL_vg4M_04F24_R_0	BY933148
E_EL_vg4M_04G01_R_0	BY933149
E_EL_vg4M_04G02_F_0	BY924602

E_EL_vg4M_04G02_R_0	BY933150
E_EL_vg4M_04G03_F_0	BY924603
E_EL_vg4M_04G03_R_0	BY933151
E_EL_vg4M_04G04_F_0	BY924604
E_EL_vg4M_04G04_R_0	BY933152
E_EL_vg4M_04G05_F_0	BY924605
E_EL_vg4M_04G05_R_0	BY933153
E_EL_vg4M_04G06_R_0	BY933154
E_EL_vg4M_04G07_F_0	BY924606
E_EL_vg4M_04G07_R_0	BY933155
E_EL_vg4M_04G08_F_0	BY924607
E_EL_vg4M_04G08_R_0	BY933156
E_EL_vg4M_04G10_F_0	BY924608
E_EL_vg4M_04G10_R_0	BY933157
E_EL_vg4M_04G11_R_0	BY933158
E_EL_vg4M_04G12_R_0	BY933159
E_EL_vg4M_04G13_F_0	BY924609
E_EL_vg4M_04G14_F_0	BY924610
E_EL_vg4M_04G14_R_0	BY933160
E_EL_vg4M_04G16_R_0	BY933161
E_EL_vg4M_04G17_F_0	BY924611
E_EL_vg4M_04G17_R_0	BY933162
E_EL_vg4M_04G19_F_0	BY924612
E_EL_vg4M_04G19_R_0	BY933163
E_EL_vg4M_04G20_F_0	BY924613
E_EL_vg4M_04G21_F_0	BY924614
E_EL_vg4M_04G21_R_0	BY933164
E_EL_vg4M_04G22_R_0	BY933165
E_EL_vg4M_04G23_F_0	BY924615
E_EL_vg4M_04G23_R_0	BY933166
E_EL_vg4M_04G24_R_0	BY933167
E_EL_vg4M_04H01_F_0	BY924616
E_EL_vg4M_04H01_R_0	BY933168
E_EL_vg4M_04H02_F_0	BY924617
E_EL_vg4M_04H02_R_0	BY933169
E_EL_vg4M_04H03_F_0	BY924618
E_EL_vg4M_04H03_R_0	BY933170
E_EL_vg4M_04H04_R_0	BY933171
E_EL_vg4M_04H05_F_0	BY924619
E_EL_vg4M_04H05_R_0	BY933172
E_EL_vg4M_04H06_R_0	BY933173
E_EL_vg4M_04H07_F_0	BY924620
E_EL_vg4M_04H07_R_0	BY933174
E_EL_vg4M_04H08_F_0	BY924621
E_EL_vg4M_04H08_R_0	BY933175
E_EL_vg4M_04H09_R_0	BY933176
E_EL_vg4M_04H10_F_0	BY924622
E_EL_vg4M_04H10_R_0	BY933177
E_EL_vg4M_04H11_F_0	BY924623
E_EL_vg4M_04H11_R_0	BY933178
E_EL_vg4M_04H12_F_0	BY924624
E_EL_vg4M_04H14_F_0	BY924625
E_EL_vg4M_04H14_R_0	BY933179
E_EL_vg4M_04H15_F_0	BY924626

E_EL_vg4M_04H15_R_0	BY933180
E_EL_vg4M_04H16_F_0	BY924627
E_EL_vg4M_04H16_R_0	BY933181
E_EL_vg4M_04H18_F_0	BY924628
E_EL_vg4M_04H18_R_0	BY933182
E_EL_vg4M_04H19_F_0	BY924629
E_EL_vg4M_04H19_R_0	BY933183
E_EL_vg4M_04H20_F_0	BY924630
E_EL_vg4M_04H20_R_0	BY933184
E_EL_vg4M_04H21_F_0	BY924631
E_EL_vg4M_04H21_R_0	BY933185
E_EL_vg4M_04H22_F_0	BY924632
E_EL_vg4M_04H22_R_0	BY933186
E_EL_vg4M_04H24_F_0	BY924633
E_EL_vg4M_04H24_R_0	BY933187
E_EL_vg4M_04I02_F_0	BY924634
E_EL_vg4M_04I02_R_0	BY933188
E_EL_vg4M_04I03_F_0	BY924635
E_EL_vg4M_04I03_R_0	BY933189
E_EL_vg4M_04I04_F_0	BY924636
E_EL_vg4M_04I04_R_0	BY933190
E_EL_vg4M_04I05_F_0	BY924637
E_EL_vg4M_04I06_R_0	BY933191
E_EL_vg4M_04I07_F_0	BY924638
E_EL_vg4M_04I08_F_0	BY924639
E_EL_vg4M_04I08_R_0	BY933192
E_EL_vg4M_04I09_F_0	BY924640
E_EL_vg4M_04I09_R_0	BY933193
E_EL_vg4M_04I10_F_0	BY924641
E_EL_vg4M_04I10_R_0	BY933194
E_EL_vg4M_04I11_F_0	BY924642
E_EL_vg4M_04I11_R_0	BY933195
E_EL_vg4M_04I12_F_0	BY924643
E_EL_vg4M_04I12_R_0	BY933196
E_EL_vg4M_04I13_F_0	BY924644
E_EL_vg4M_04I13_R_0	BY933197
E_EL_vg4M_04I14_F_0	BY924645
E_EL_vg4M_04I14_R_0	BY933198
E_EL_vg4M_04I15_F_0	BY924646
E_EL_vg4M_04I15_R_0	BY933199
E_EL_vg4M_04I16_F_0	BY924647
E_EL_vg4M_04I17_F_0	BY924648
E_EL_vg4M_04I17_R_0	BY933200
E_EL_vg4M_04I18_F_0	BY924649
E_EL_vg4M_04I18_R_0	BY933201
E_EL_vg4M_04I19_R_0	BY933202
E_EL_vg4M_04I20_F_0	BY924650
E_EL_vg4M_04I20_R_0	BY933203
E_EL_vg4M_04I22_R_0	BY933204
E_EL_vg4M_04I24_R_0	BY933205
E_EL_vg4M_04J01_F_0	BY924651
E_EL_vg4M_04J01_R_0	BY933206
E_EL_vg4M_04J02_F_0	BY924652
E_EL_vg4M_04J02_R_0	BY933207

E_EL_vg4M_04J03_F_0	BY924653
E_EL_vg4M_04J03_R_0	BY933208
E_EL_vg4M_04J04_F_0	BY924654
E_EL_vg4M_04J04_R_0	BY933209
E_EL_vg4M_04J05_R_0	BY933210
E_EL_vg4M_04J06_F_0	BY924655
E_EL_vg4M_04J06_R_0	BY933211
E_EL_vg4M_04J07_F_0	BY924656
E_EL_vg4M_04J07_R_0	BY933212
E_EL_vg4M_04J08_F_0	BY924657
E_EL_vg4M_04J08_R_0	BY933213
E_EL_vg4M_04J09_F_0	BY924658
E_EL_vg4M_04J09_R_0	BY933214
E_EL_vg4M_04J10_F_0	BY924659
E_EL_vg4M_04J10_R_0	BY933215
E_EL_vg4M_04J11_F_0	BY924660
E_EL_vg4M_04J11_R_0	BY933216
E_EL_vg4M_04J12_F_0	BY924661
E_EL_vg4M_04J12_R_0	BY933217
E_EL_vg4M_04J13_F_0	BY924662
E_EL_vg4M_04J13_R_0	BY933218
E_EL_vg4M_04J14_F_0	BY924663
E_EL_vg4M_04J14_R_0	BY933219
E_EL_vg4M_04J15_F_0	BY924664
E_EL_vg4M_04J15_R_0	BY933220
E_EL_vg4M_04J16_F_0	BY924665
E_EL_vg4M_04J16_R_0	BY933221
E_EL_vg4M_04J17_F_0	BY924666
E_EL_vg4M_04J17_R_0	BY933222
E_EL_vg4M_04J18_F_0	BY924667
E_EL_vg4M_04J19_R_0	BY933223
E_EL_vg4M_04J20_F_0	BY924668
E_EL_vg4M_04J20_R_0	BY933224
E_EL_vg4M_04J21_R_0	BY933225
E_EL_vg4M_04J22_F_0	BY924669
E_EL_vg4M_04J22_R_0	BY933226
E_EL_vg4M_04J23_R_0	BY933227
E_EL_vg4M_04J24_F_0	BY924670
E_EL_vg4M_04K01_F_0	BY924671
E_EL_vg4M_04K01_R_0	BY933228
E_EL_vg4M_04K02_F_0	BY924672
E_EL_vg4M_04K02_R_0	BY933229
E_EL_vg4M_04K03_F_0	BY924673
E_EL_vg4M_04K03_R_0	BY933230
E_EL_vg4M_04K04_R_0	BY933231
E_EL_vg4M_04K05_F_0	BY924674
E_EL_vg4M_04K05_R_0	BY933232
E_EL_vg4M_04K06_R_0	BY933233
E_EL_vg4M_04K07_F_0	BY924675
E_EL_vg4M_04K07_R_0	BY933234
E_EL_vg4M_04K09_F_0	BY924676
E_EL_vg4M_04K09_R_0	BY933235
E_EL_vg4M_04K10_F_0	BY924677
E_EL_vg4M_04K11_F_0	BY924678

E_EL_vg4M_04K11_R_0	BY933236
E_EL_vg4M_04K12_F_0	BY924679
E_EL_vg4M_04K12_R_0	BY933237
E_EL_vg4M_04K13_F_0	BY924680
E_EL_vg4M_04K13_R_0	BY933238
E_EL_vg4M_04K14_F_0	BY924681
E_EL_vg4M_04K15_F_0	BY924682
E_EL_vg4M_04K15_R_0	BY933239
E_EL_vg4M_04K16_R_0	BY933240
E_EL_vg4M_04K17_F_0	BY924683
E_EL_vg4M_04K19_R_0	BY933241
E_EL_vg4M_04K20_F_0	BY924684
E_EL_vg4M_04K20_R_0	BY933242
E_EL_vg4M_04K21_F_0	BY924685
E_EL_vg4M_04K21_R_0	BY933243
E_EL_vg4M_04K22_F_0	BY924686
E_EL_vg4M_04K22_R_0	BY933244
E_EL_vg4M_04K23_R_0	BY933245
E_EL_vg4M_04L01_F_0	BY924687
E_EL_vg4M_04L01_R_0	BY933246
E_EL_vg4M_04L02_F_0	BY924688
E_EL_vg4M_04L02_R_0	BY933247
E_EL_vg4M_04L03_F_0	BY924689
E_EL_vg4M_04L04_F_0	BY924690
E_EL_vg4M_04L04_R_0	BY933248
E_EL_vg4M_04L05_F_0	BY924691
E_EL_vg4M_04L05_R_0	BY933249
E_EL_vg4M_04L06_F_0	BY924692
E_EL_vg4M_04L07_F_0	BY924693
E_EL_vg4M_04L08_F_0	BY924694
E_EL_vg4M_04L08_R_0	BY933250
E_EL_vg4M_04L09_R_0	BY933251
E_EL_vg4M_04L11_F_0	BY924695
E_EL_vg4M_04L11_R_0	BY933252
E_EL_vg4M_04L12_F_0	BY924696
E_EL_vg4M_04L13_F_0	BY924697
E_EL_vg4M_04L15_F_0	BY924698
E_EL_vg4M_04L16_F_0	BY924699
E_EL_vg4M_04L16_R_0	BY933253
E_EL_vg4M_04L17_F_0	BY924700
E_EL_vg4M_04L19_R_0	BY933254
E_EL_vg4M_04L20_F_0	BY924701
E_EL_vg4M_04L20_R_0	BY933255
E_EL_vg4M_04L21_F_0	BY924702
E_EL_vg4M_04L21_R_0	BY933256
E_EL_vg4M_04L22_F_0	BY924703
E_EL_vg4M_04L22_R_0	BY933257
E_EL_vg4M_04L23_F_0	BY924704
E_EL_vg4M_04L24_F_0	BY924705
E_EL_vg4M_04L24_R_0	BY933258
E_EL_vg4M_04M01_F_0	BY924706
E_EL_vg4M_04M01_R_0	BY933259
E_EL_vg4M_04M02_F_0	BY924707
E_EL_vg4M_04M02_R_0	BY933260

E_EL_vg4M_04M03_F_0	BY924708
E_EL_vg4M_04M03_R_0	BY933261
E_EL_vg4M_04M05_F_0	BY924709
E_EL_vg4M_04M05_R_0	BY933262
E_EL_vg4M_04M06_F_0	BY924710
E_EL_vg4M_04M06_R_0	BY933263
E_EL_vg4M_04M07_R_0	BY933264
E_EL_vg4M_04M08_F_0	BY924711
E_EL_vg4M_04M08_R_0	BY933265
E_EL_vg4M_04M09_F_0	BY924712
E_EL_vg4M_04M09_R_0	BY933266
E_EL_vg4M_04M10_F_0	BY924713
E_EL_vg4M_04M10_R_0	BY933267
E_EL_vg4M_04M11_R_0	BY933268
E_EL_vg4M_04M12_R_0	BY933269
E_EL_vg4M_04M14_F_0	BY924714
E_EL_vg4M_04M15_F_0	BY924715
E_EL_vg4M_04M15_R_0	BY933270
E_EL_vg4M_04M16_F_0	BY924716
E_EL_vg4M_04M16_R_0	BY933271
E_EL_vg4M_04M18_R_0	BY933272
E_EL_vg4M_04M19_R_0	BY933273
E_EL_vg4M_04M20_R_0	BY933274
E_EL_vg4M_04M21_R_0	BY933275
E_EL_vg4M_04M22_R_0	BY933276
E_EL_vg4M_04M23_R_0	BY933277
E_EL_vg4M_04M24_R_0	BY933278
E_EL_vg4M_04N01_F_0	BY924717
E_EL_vg4M_04N01_R_0	BY933279
E_EL_vg4M_04N02_F_0	BY924718
E_EL_vg4M_04N02_R_0	BY933280
E_EL_vg4M_04N03_F_0	BY924719
E_EL_vg4M_04N03_R_0	BY933281
E_EL_vg4M_04N04_R_0	BY933282
E_EL_vg4M_04N05_R_0	BY933283
E_EL_vg4M_04N06_F_0	BY924720
E_EL_vg4M_04N07_F_0	BY924721
E_EL_vg4M_04N08_F_0	BY924722
E_EL_vg4M_04N08_R_0	BY933284
E_EL_vg4M_04N09_F_0	BY924723
E_EL_vg4M_04N09_R_0	BY933285
E_EL_vg4M_04N10_F_0	BY924724
E_EL_vg4M_04N10_R_0	BY933286
E_EL_vg4M_04N11_F_0	BY924725
E_EL_vg4M_04N11_R_0	BY933287
E_EL_vg4M_04N12_R_0	BY933288
E_EL_vg4M_04N13_R_0	BY933289
E_EL_vg4M_04N14_R_0	BY933290
E_EL_vg4M_04N16_R_0	BY933291
E_EL_vg4M_04N17_F_0	BY924726
E_EL_vg4M_04N17_R_0	BY933292
E_EL_vg4M_04N18_F_0	BY924727
E_EL_vg4M_04N19_F_0	BY924728
E_EL_vg4M_04N19_R_0	BY933293

E_EL_vg4M_04N20_F_0	BY924729
E_EL_vg4M_04N20_R_0	BY933294
E_EL_vg4M_04N21_F_0	BY924730
E_EL_vg4M_04N22_F_0	BY924731
E_EL_vg4M_04N22_R_0	BY933295
E_EL_vg4M_04N23_F_0	BY924732
E_EL_vg4M_04O01_F_0	BY924733
E_EL_vg4M_04O02_F_0	BY924734
E_EL_vg4M_04O02_R_0	BY933296
E_EL_vg4M_04O03_F_0	BY924735
E_EL_vg4M_04O03_R_0	BY933297
E_EL_vg4M_04O04_F_0	BY924736
E_EL_vg4M_04O04_R_0	BY933298
E_EL_vg4M_04O05_F_0	BY924737
E_EL_vg4M_04O05_R_0	BY933299
E_EL_vg4M_04O06_R_0	BY933300
E_EL_vg4M_04O07_F_0	BY924738
E_EL_vg4M_04O07_R_0	BY933301
E_EL_vg4M_04O08_F_0	BY924739
E_EL_vg4M_04O08_R_0	BY933302
E_EL_vg4M_04O09_F_0	BY924740
E_EL_vg4M_04O09_R_0	BY933303
E_EL_vg4M_04O10_F_0	BY924741
E_EL_vg4M_04O10_R_0	BY933304
E_EL_vg4M_04O11_F_0	BY924742
E_EL_vg4M_04O11_R_0	BY933305
E_EL_vg4M_04O12_R_0	BY933306
E_EL_vg4M_04O13_R_0	BY933307
E_EL_vg4M_04O14_F_0	BY924743
E_EL_vg4M_04O14_R_0	BY933308
E_EL_vg4M_04O15_F_0	BY924744
E_EL_vg4M_04O15_R_0	BY933309
E_EL_vg4M_04O16_F_0	BY924745
E_EL_vg4M_04O16_R_0	BY933310
E_EL_vg4M_04O17_F_0	BY924746
E_EL_vg4M_04O17_R_0	BY933311
E_EL_vg4M_04O19_R_0	BY933312
E_EL_vg4M_04O20_F_0	BY924747
E_EL_vg4M_04O20_R_0	BY933313
E_EL_vg4M_04O21_F_0	BY924748
E_EL_vg4M_04O21_R_0	BY933314
E_EL_vg4M_04O22_R_0	BY933315
E_EL_vg4M_04O23_F_0	BY924749
E_EL_vg4M_04O23_R_0	BY933316
E_EL_vg4M_04P01_R_0	BY933317
E_EL_vg4M_04P02_R_0	BY933318
E_EL_vg4M_04P03_F_0	BY924750
E_EL_vg4M_04P03_R_0	BY933319
E_EL_vg4M_04P04_F_0	BY924751
E_EL_vg4M_04P04_R_0	BY933320
E_EL_vg4M_04P05_F_0	BY924752
E_EL_vg4M_04P06_F_0	BY924753
E_EL_vg4M_04P07_F_0	BY924754
E_EL_vg4M_04P08_F_0	BY924755

E_EL_vg4M_04P08_R_0	BY933321
E_EL_vg4M_04P09_F_0	BY924756
E_EL_vg4M_04P09_R_0	BY933322
E_EL_vg4M_04P10_F_0	BY924757
E_EL_vg4M_04P11_F_0	BY924758
E_EL_vg4M_04P11_R_0	BY933323
E_EL_vg4M_04P12_F_0	BY924759
E_EL_vg4M_04P12_R_0	BY933324
E_EL_vg4M_04P13_F_0	BY924760
E_EL_vg4M_04P13_R_0	BY933325
E_EL_vg4M_04P14_F_0	BY924761
E_EL_vg4M_04P14_R_0	BY933326
E_EL_vg4M_04P15_F_0	BY924762
E_EL_vg4M_04P15_R_0	BY933327
E_EL_vg4M_04P16_F_0	BY924763
E_EL_vg4M_04P16_R_0	BY933328
E_EL_vg4M_04P17_F_0	BY924764
E_EL_vg4M_04P17_R_0	BY933329
E_EL_vg4M_04P19_F_0	BY924765
E_EL_vg4M_04P19_R_0	BY933330
E_EL_vg4M_04P20_F_0	BY924766
E_EL_vg4M_04P20_R_0	BY933331
E_EL_vg4M_04P21_F_0	BY924767
E_EL_vg4M_04P21_R_0	BY933332
E_EL_vg4M_04P22_F_0	BY924768
E_EL_vg4M_04P23_F_0	BY924769
E_EL_vg4M_04P24_F_0	BY924770
E_EL_vg4M_04P24_R_0	BY933333
E_EL_vg4M_05A01_F_0	BY924771
E_EL_vg4M_05A02_F_0	BY924772
E_EL_vg4M_05A02_R_0	BY933334
E_EL_vg4M_05A03_F_0	BY924773
E_EL_vg4M_05A04_F_0	BY924774
E_EL_vg4M_05A04_R_0	BY933335
E_EL_vg4M_05A05_F_0	BY924775
E_EL_vg4M_05A05_R_0	BY933336
E_EL_vg4M_05A06_F_0	BY924776
E_EL_vg4M_05A06_R_0	BY933337
E_EL_vg4M_05A07_F_0	BY924777
E_EL_vg4M_05A07_R_0	BY933338
E_EL_vg4M_05A08_F_0	BY924778
E_EL_vg4M_05A08_R_0	BY933339
E_EL_vg4M_05A09_F_0	BY924779
E_EL_vg4M_05A09_R_0	BY933340
E_EL_vg4M_05A11_F_0	BY924780
E_EL_vg4M_05A11_R_0	BY933341
E_EL_vg4M_05A13_F_0	BY924781
E_EL_vg4M_05A13_R_0	BY933342
E_EL_vg4M_05A14_F_0	BY924782
E_EL_vg4M_05A14_R_0	BY933343
E_EL_vg4M_05A15_F_0	BY924783
E_EL_vg4M_05A16_F_0	BY924784
E_EL_vg4M_05A16_R_0	BY933344
E_EL_vg4M_05A18_F_0	BY924785

E_EL_vg4M_05A18_R_0	BY933345
E_EL_vg4M_05A19_F_0	BY924786
E_EL_vg4M_05A20_F_0	BY924787
E_EL_vg4M_05A20_R_0	BY933346
E_EL_vg4M_05A21_F_0	BY924788
E_EL_vg4M_05A21_R_0	BY933347
E_EL_vg4M_05A22_F_0	BY924789
E_EL_vg4M_05A22_R_0	BY933348
E_EL_vg4M_05A23_F_0	BY924790
E_EL_vg4M_05A23_R_0	BY933349
E_EL_vg4M_05A24_F_0	BY924791
E_EL_vg4M_05A24_R_0	BY933350
E_EL_vg4M_05B01_F_0	BY924792
E_EL_vg4M_05B01_R_0	BY933351
E_EL_vg4M_05B02_F_0	BY924793
E_EL_vg4M_05B02_R_0	BY933352
E_EL_vg4M_05B03_F_0	BY924794
E_EL_vg4M_05B03_R_0	BY933353
E_EL_vg4M_05B04_F_0	BY924795
E_EL_vg4M_05B04_R_0	BY933354
E_EL_vg4M_05B05_F_0	BY924796
E_EL_vg4M_05B05_R_0	BY933355
E_EL_vg4M_05B07_F_0	BY924797
E_EL_vg4M_05B07_R_0	BY933356
E_EL_vg4M_05B08_F_0	BY924798
E_EL_vg4M_05B08_R_0	BY933357
E_EL_vg4M_05B09_F_0	BY924799
E_EL_vg4M_05B09_R_0	BY933358
E_EL_vg4M_05B10_F_0	BY924800
E_EL_vg4M_05B10_R_0	BY933359
E_EL_vg4M_05B11_F_0	BY924801
E_EL_vg4M_05B11_R_0	BY933360
E_EL_vg4M_05B13_F_0	BY924802
E_EL_vg4M_05B13_R_0	BY933361
E_EL_vg4M_05B14_R_0	BY933362
E_EL_vg4M_05B15_F_0	BY924803
E_EL_vg4M_05B15_R_0	BY933363
E_EL_vg4M_05B17_F_0	BY924804
E_EL_vg4M_05B17_R_0	BY933364
E_EL_vg4M_05B18_F_0	BY924805
E_EL_vg4M_05B18_R_0	BY933365
E_EL_vg4M_05B19_F_0	BY924806
E_EL_vg4M_05B19_R_0	BY933366
E_EL_vg4M_05B20_F_0	BY924807
E_EL_vg4M_05B20_R_0	BY933367
E_EL_vg4M_05B22_F_0	BY924808
E_EL_vg4M_05B22_R_0	BY933368
E_EL_vg4M_05B23_F_0	BY924809
E_EL_vg4M_05B23_R_0	BY933369
E_EL_vg4M_05C01_F_0	BY924810
E_EL_vg4M_05C01_R_0	BY933370
E_EL_vg4M_05C02_F_0	BY924811
E_EL_vg4M_05C02_R_0	BY933371
E_EL_vg4M_05C03_F_0	BY924812

E_EL_vg4M_05C03_R_0	BY933372
E_EL_vg4M_05C04_F_0	BY924813
E_EL_vg4M_05C04_R_0	BY933373
E_EL_vg4M_05C05_F_0	BY924814
E_EL_vg4M_05C05_R_0	BY933374
E_EL_vg4M_05C06_R_0	BY933375
E_EL_vg4M_05C08_R_0	BY933376
E_EL_vg4M_05C09_F_0	BY924815
E_EL_vg4M_05C09_R_0	BY933377
E_EL_vg4M_05C11_F_0	BY924816
E_EL_vg4M_05C11_R_0	BY933378
E_EL_vg4M_05C12_F_0	BY924817
E_EL_vg4M_05C12_R_0	BY933379
E_EL_vg4M_05C14_F_0	BY924818
E_EL_vg4M_05C14_R_0	BY933380
E_EL_vg4M_05C15_R_0	BY933381
E_EL_vg4M_05C16_R_0	BY933382
E_EL_vg4M_05C17_R_0	BY933383
E_EL_vg4M_05C18_R_0	BY933384
E_EL_vg4M_05C20_R_0	BY933385
E_EL_vg4M_05C21_F_0	BY924819
E_EL_vg4M_05C21_R_0	BY933386
E_EL_vg4M_05C22_F_0	BY924820
E_EL_vg4M_05C22_R_0	BY933387
E_EL_vg4M_05C23_F_0	BY924821
E_EL_vg4M_05C23_R_0	BY933388
E_EL_vg4M_05C24_F_0	BY924822
E_EL_vg4M_05C24_R_0	BY933389
E_EL_vg4M_05D01_R_0	BY933390
E_EL_vg4M_05D02_F_0	BY924823
E_EL_vg4M_05D02_R_0	BY933391
E_EL_vg4M_05D03_F_0	BY924824
E_EL_vg4M_05D03_R_0	BY933392
E_EL_vg4M_05D04_F_0	BY924825
E_EL_vg4M_05D04_R_0	BY933393
E_EL_vg4M_05D05_F_0	BY924826
E_EL_vg4M_05D05_R_0	BY933394
E_EL_vg4M_05D06_R_0	BY933395
E_EL_vg4M_05D07_F_0	BY924827
E_EL_vg4M_05D07_R_0	BY933396
E_EL_vg4M_05D08_F_0	BY924828
E_EL_vg4M_05D08_R_0	BY933397
E_EL_vg4M_05D09_F_0	BY924829
E_EL_vg4M_05D09_R_0	BY933398
E_EL_vg4M_05D10_F_0	BY924830
E_EL_vg4M_05D10_R_0	BY933399
E_EL_vg4M_05D12_R_0	BY933400
E_EL_vg4M_05D13_R_0	BY933401
E_EL_vg4M_05D14_R_0	BY933402
E_EL_vg4M_05D15_F_0	BY924831
E_EL_vg4M_05D15_R_0	BY933403
E_EL_vg4M_05D16_F_0	BY924832
E_EL_vg4M_05D16_R_0	BY933404
E_EL_vg4M_05D17_R_0	BY933405

E_EL_vg4M_05D19_R_0	BY933406
E_EL_vg4M_05D20_F_0	BY924833
E_EL_vg4M_05D20_R_0	BY933407
E_EL_vg4M_05D21_R_0	BY933408
E_EL_vg4M_05D22_R_0	BY933409
E_EL_vg4M_05D23_F_0	BY924834
E_EL_vg4M_05D23_R_0	BY933410
E_EL_vg4M_05D24_R_0	BY933411
E_EL_vg4M_05E01_F_0	BY924835
E_EL_vg4M_05E01_R_0	BY933412
E_EL_vg4M_05E02_F_0	BY924836
E_EL_vg4M_05E02_R_0	BY933413
E_EL_vg4M_05E03_F_0	BY924837
E_EL_vg4M_05E03_R_0	BY933414
E_EL_vg4M_05E04_F_0	BY924838
E_EL_vg4M_05E04_R_0	BY933415
E_EL_vg4M_05E05_F_0	BY924839
E_EL_vg4M_05E05_R_0	BY933416
E_EL_vg4M_05E06_R_0	BY933417
E_EL_vg4M_05E07_F_0	BY924840
E_EL_vg4M_05E08_R_0	BY933418
E_EL_vg4M_05E09_F_0	BY924841
E_EL_vg4M_05E09_R_0	BY933419
E_EL_vg4M_05E10_F_0	BY924842
E_EL_vg4M_05E10_R_0	BY933420
E_EL_vg4M_05E11_F_0	BY924843
E_EL_vg4M_05E11_R_0	BY933421
E_EL_vg4M_05E12_F_0	BY924844
E_EL_vg4M_05E12_R_0	BY933422
E_EL_vg4M_05E14_F_0	BY924845
E_EL_vg4M_05E14_R_0	BY933423
E_EL_vg4M_05E15_F_0	BY924846
E_EL_vg4M_05E15_R_0	BY933424
E_EL_vg4M_05E16_F_0	BY924847
E_EL_vg4M_05E16_R_0	BY933425
E_EL_vg4M_05E17_F_0	BY924848
E_EL_vg4M_05E17_R_0	BY933426
E_EL_vg4M_05E18_R_0	BY933427
E_EL_vg4M_05E19_R_0	BY933428
E_EL_vg4M_05E20_F_0	BY924849
E_EL_vg4M_05E20_R_0	BY933429
E_EL_vg4M_05E21_F_0	BY924850
E_EL_vg4M_05E21_R_0	BY933430
E_EL_vg4M_05E22_F_0	BY924851
E_EL_vg4M_05E22_R_0	BY933431
E_EL_vg4M_05E23_F_0	BY924852
E_EL_vg4M_05E23_R_0	BY933432
E_EL_vg4M_05E24_F_0	BY924853
E_EL_vg4M_05E24_R_0	BY933433
E_EL_vg4M_05F02_F_0	BY924854
E_EL_vg4M_05F02_R_0	BY933434
E_EL_vg4M_05F03_F_0	BY924855
E_EL_vg4M_05F03_R_0	BY933435
E_EL_vg4M_05F04_F_0	BY924856

E_EL_vg4M_05F04_R_0	BY933436
E_EL_vg4M_05F05_R_0	BY933437
E_EL_vg4M_05F06_F_0	BY924857
E_EL_vg4M_05F06_R_0	BY933438
E_EL_vg4M_05F08_F_0	BY924858
E_EL_vg4M_05F08_R_0	BY933439
E_EL_vg4M_05F09_R_0	BY933440
E_EL_vg4M_05F10_F_0	BY924859
E_EL_vg4M_05F10_R_0	BY933441
E_EL_vg4M_05F11_F_0	BY924860
E_EL_vg4M_05F11_R_0	BY933442
E_EL_vg4M_05F12_F_0	BY924861
E_EL_vg4M_05F12_R_0	BY933443
E_EL_vg4M_05F13_F_0	BY924862
E_EL_vg4M_05F14_F_0	BY924863
E_EL_vg4M_05F14_R_0	BY933444
E_EL_vg4M_05F15_F_0	BY924864
E_EL_vg4M_05F15_R_0	BY933445
E_EL_vg4M_05F17_F_0	BY924865
E_EL_vg4M_05F17_R_0	BY933446
E_EL_vg4M_05F19_R_0	BY933447
E_EL_vg4M_05F20_F_0	BY924866
E_EL_vg4M_05F20_R_0	BY933448
E_EL_vg4M_05F21_F_0	BY924867
E_EL_vg4M_05F21_R_0	BY933449
E_EL_vg4M_05F22_F_0	BY924868
E_EL_vg4M_05F22_R_0	BY933450
E_EL_vg4M_05F23_F_0	BY924869
E_EL_vg4M_05F23_R_0	BY933451
E_EL_vg4M_05F24_F_0	BY924870
E_EL_vg4M_05F24_R_0	BY933452
E_EL_vg4M_05G01_F_0	BY924871
E_EL_vg4M_05G01_R_0	BY933453
E_EL_vg4M_05G02_R_0	BY933454
E_EL_vg4M_05G03_F_0	BY924872
E_EL_vg4M_05G03_R_0	BY933455
E_EL_vg4M_05G04_R_0	BY933456
E_EL_vg4M_05G05_F_0	BY924873
E_EL_vg4M_05G05_R_0	BY933457
E_EL_vg4M_05G06_R_0	BY933458
E_EL_vg4M_05G07_F_0	BY924874
E_EL_vg4M_05G07_R_0	BY933459
E_EL_vg4M_05G08_F_0	BY924875
E_EL_vg4M_05G08_R_0	BY933460
E_EL_vg4M_05G09_F_0	BY924876
E_EL_vg4M_05G09_R_0	BY933461
E_EL_vg4M_05G11_F_0	BY924877
E_EL_vg4M_05G11_R_0	BY933462
E_EL_vg4M_05G12_F_0	BY924878
E_EL_vg4M_05G12_R_0	BY933463
E_EL_vg4M_05G14_F_0	BY924879
E_EL_vg4M_05G14_R_0	BY933464
E_EL_vg4M_05G15_F_0	BY924880
E_EL_vg4M_05G15_R_0	BY933465

E_EL_vg4M_05G18_F_0	BY924881
E_EL_vg4M_05G18_R_0	BY933466
E_EL_vg4M_05G19_R_0	BY933467
E_EL_vg4M_05G20_F_0	BY924882
E_EL_vg4M_05G20_R_0	BY933468
E_EL_vg4M_05G21_F_0	BY924883
E_EL_vg4M_05G21_R_0	BY933469
E_EL_vg4M_05G22_F_0	BY924884
E_EL_vg4M_05G22_R_0	BY933470
E_EL_vg4M_05G24_F_0	BY924885
E_EL_vg4M_05G24_R_0	BY933471
E_EL_vg4M_05H01_F_0	BY924886
E_EL_vg4M_05H01_R_0	BY933472
E_EL_vg4M_05H02_F_0	BY924887
E_EL_vg4M_05H02_R_0	BY933473
E_EL_vg4M_05H03_F_0	BY924888
E_EL_vg4M_05H03_R_0	BY933474
E_EL_vg4M_05H04_F_0	BY924889
E_EL_vg4M_05H04_R_0	BY933475
E_EL_vg4M_05H05_F_0	BY924890
E_EL_vg4M_05H05_R_0	BY933476
E_EL_vg4M_05H06_R_0	BY933477
E_EL_vg4M_05H07_F_0	BY924891
E_EL_vg4M_05H07_R_0	BY933478
E_EL_vg4M_05H08_F_0	BY924892
E_EL_vg4M_05H09_R_0	BY933479
E_EL_vg4M_05H11_F_0	BY924893
E_EL_vg4M_05H11_R_0	BY933480
E_EL_vg4M_05H12_F_0	BY924894
E_EL_vg4M_05H12_R_0	BY933481
E_EL_vg4M_05H13_F_0	BY924895
E_EL_vg4M_05H13_R_0	BY933482
E_EL_vg4M_05H15_F_0	BY924896
E_EL_vg4M_05H15_R_0	BY933483
E_EL_vg4M_05H16_F_0	BY924897
E_EL_vg4M_05H16_R_0	BY933484
E_EL_vg4M_05H17_F_0	BY924898
E_EL_vg4M_05H17_R_0	BY933485
E_EL_vg4M_05H18_R_0	BY933486
E_EL_vg4M_05H20_F_0	BY924899
E_EL_vg4M_05H20_R_0	BY933487
E_EL_vg4M_05H21_F_0	BY924900
E_EL_vg4M_05H21_R_0	BY933488
E_EL_vg4M_05H22_F_0	BY924901
E_EL_vg4M_05H22_R_0	BY933489
E_EL_vg4M_05H23_F_0	BY924902
E_EL_vg4M_05H23_R_0	BY933490
E_EL_vg4M_05I01_F_0	BY924903
E_EL_vg4M_05I01_R_0	BY933491
E_EL_vg4M_05I02_F_0	BY924904
E_EL_vg4M_05I02_R_0	BY933492
E_EL_vg4M_05I03_F_0	BY924905
E_EL_vg4M_05I03_R_0	BY933493
E_EL_vg4M_05I04_F_0	BY924906

E_EL_vg4M_05I04_R_0	BY933494
E_EL_vg4M_05I05_F_0	BY924907
E_EL_vg4M_05I05_R_0	BY933495
E_EL_vg4M_05I06_F_0	BY924908
E_EL_vg4M_05I06_R_0	BY933496
E_EL_vg4M_05I07_F_0	BY924909
E_EL_vg4M_05I07_R_0	BY933497
E_EL_vg4M_05I08_F_0	BY924910
E_EL_vg4M_05I08_R_0	BY933498
E_EL_vg4M_05I09_F_0	BY924911
E_EL_vg4M_05I09_R_0	BY933499
E_EL_vg4M_05I10_F_0	BY924912
E_EL_vg4M_05I10_R_0	BY933500
E_EL_vg4M_05I11_F_0	BY924913
E_EL_vg4M_05I11_R_0	BY933501
E_EL_vg4M_05I12_F_0	BY924914
E_EL_vg4M_05I12_R_0	BY933502
E_EL_vg4M_05I13_F_0	BY924915
E_EL_vg4M_05I13_R_0	BY933503
E_EL_vg4M_05I14_F_0	BY924916
E_EL_vg4M_05I14_R_0	BY933504
E_EL_vg4M_05I15_F_0	BY924917
E_EL_vg4M_05I15_R_0	BY933505
E_EL_vg4M_05I16_F_0	BY924918
E_EL_vg4M_05I16_R_0	BY933506
E_EL_vg4M_05I17_F_0	BY924919
E_EL_vg4M_05I17_R_0	BY933507
E_EL_vg4M_05I18_R_0	BY933508
E_EL_vg4M_05I19_R_0	BY933509
E_EL_vg4M_05I20_F_0	BY924920
E_EL_vg4M_05I20_R_0	BY933510
E_EL_vg4M_05I21_F_0	BY924921
E_EL_vg4M_05I21_R_0	BY933511
E_EL_vg4M_05I22_F_0	BY924922
E_EL_vg4M_05I22_R_0	BY933512
E_EL_vg4M_05I23_R_0	BY933513
E_EL_vg4M_05I24_R_0	BY933514
E_EL_vg4M_05J01_R_0	BY933515
E_EL_vg4M_05J02_F_0	BY924923
E_EL_vg4M_05J02_R_0	BY933516
E_EL_vg4M_05J03_F_0	BY924924
E_EL_vg4M_05J03_R_0	BY933517
E_EL_vg4M_05J04_R_0	BY933518
E_EL_vg4M_05J05_F_0	BY924925
E_EL_vg4M_05J05_R_0	BY933519
E_EL_vg4M_05J06_F_0	BY924926
E_EL_vg4M_05J06_R_0	BY933520
E_EL_vg4M_05J07_F_0	BY924927
E_EL_vg4M_05J07_R_0	BY933521
E_EL_vg4M_05J08_F_0	BY924928
E_EL_vg4M_05J08_R_0	BY933522
E_EL_vg4M_05J10_R_0	BY933523
E_EL_vg4M_05J11_F_0	BY924929
E_EL_vg4M_05J11_R_0	BY933524

E_EL_vg4M_05J12_F_0	BY924930
E_EL_vg4M_05J12_R_0	BY933525
E_EL_vg4M_05J13_F_0	BY924931
E_EL_vg4M_05J13_R_0	BY933526
E_EL_vg4M_05J14_F_0	BY924932
E_EL_vg4M_05J14_R_0	BY933527
E_EL_vg4M_05J16_R_0	BY933528
E_EL_vg4M_05J17_F_0	BY924933
E_EL_vg4M_05J17_R_0	BY933529
E_EL_vg4M_05J18_R_0	BY933530
E_EL_vg4M_05J19_R_0	BY933531
E_EL_vg4M_05J20_F_0	BY924934
E_EL_vg4M_05J20_R_0	BY933532
E_EL_vg4M_05J21_F_0	BY924935
E_EL_vg4M_05J21_R_0	BY933533
E_EL_vg4M_05J23_F_0	BY924936
E_EL_vg4M_05J23_R_0	BY933534
E_EL_vg4M_05J24_R_0	BY933535
E_EL_vg4M_05K01_F_0	BY924937
E_EL_vg4M_05K01_R_0	BY933536
E_EL_vg4M_05K03_F_0	BY924938
E_EL_vg4M_05K03_R_0	BY933537
E_EL_vg4M_05K04_F_0	BY924939
E_EL_vg4M_05K04_R_0	BY933538
E_EL_vg4M_05K05_F_0	BY924940
E_EL_vg4M_05K05_R_0	BY933539
E_EL_vg4M_05K06_F_0	BY924941
E_EL_vg4M_05K06_R_0	BY933540
E_EL_vg4M_05K07_R_0	BY933541
E_EL_vg4M_05K08_F_0	BY924942
E_EL_vg4M_05K08_R_0	BY933542
E_EL_vg4M_05K09_F_0	BY924943
E_EL_vg4M_05K09_R_0	BY933543
E_EL_vg4M_05K10_F_0	BY924944
E_EL_vg4M_05K10_R_0	BY933544
E_EL_vg4M_05K11_F_0	BY924945
E_EL_vg4M_05K11_R_0	BY933545
E_EL_vg4M_05K12_F_0	BY924946
E_EL_vg4M_05K12_R_0	BY933546
E_EL_vg4M_05K13_F_0	BY924947
E_EL_vg4M_05K13_R_0	BY933547
E_EL_vg4M_05K14_R_0	BY933548
E_EL_vg4M_05K15_R_0	BY933549
E_EL_vg4M_05K16_F_0	BY924948
E_EL_vg4M_05K16_R_0	BY933550
E_EL_vg4M_05K17_R_0	BY933551
E_EL_vg4M_05K18_R_0	BY933552
E_EL_vg4M_05K20_R_0	BY933553
E_EL_vg4M_05K21_R_0	BY933554
E_EL_vg4M_05K22_F_0	BY924949
E_EL_vg4M_05K22_R_0	BY933555
E_EL_vg4M_05K23_F_0	BY924950
E_EL_vg4M_05K23_R_0	BY933556
E_EL_vg4M_05K24_R_0	BY933557

E_EL_vg4M_05L01_F_0	BY924951
E_EL_vg4M_05L01_R_0	BY933558
E_EL_vg4M_05L02_F_0	BY924952
E_EL_vg4M_05L02_R_0	BY933559
E_EL_vg4M_05L04_F_0	BY924953
E_EL_vg4M_05L04_R_0	BY933560
E_EL_vg4M_05L06_F_0	BY924954
E_EL_vg4M_05L06_R_0	BY933561
E_EL_vg4M_05L07_F_0	BY924955
E_EL_vg4M_05L08_F_0	BY924956
E_EL_vg4M_05L08_R_0	BY933562
E_EL_vg4M_05L09_F_0	BY924957
E_EL_vg4M_05L09_R_0	BY933563
E_EL_vg4M_05L10_F_0	BY924958
E_EL_vg4M_05L10_R_0	BY933564
E_EL_vg4M_05L11_R_0	BY933565
E_EL_vg4M_05L12_F_0	BY924959
E_EL_vg4M_05L12_R_0	BY933566
E_EL_vg4M_05L13_R_0	BY933567
E_EL_vg4M_05L16_R_0	BY933568
E_EL_vg4M_05L17_R_0	BY933569
E_EL_vg4M_05L18_F_0	BY924960
E_EL_vg4M_05L18_R_0	BY933570
E_EL_vg4M_05L19_F_0	BY924961
E_EL_vg4M_05L19_R_0	BY933571
E_EL_vg4M_05L20_F_0	BY924962
E_EL_vg4M_05L20_R_0	BY933572
E_EL_vg4M_05L21_F_0	BY924963
E_EL_vg4M_05L21_R_0	BY933573
E_EL_vg4M_05L22_F_0	BY924964
E_EL_vg4M_05L22_R_0	BY933574
E_EL_vg4M_05L23_F_0	BY924965
E_EL_vg4M_05L23_R_0	BY933575
E_EL_vg4M_05L24_F_0	BY924966
E_EL_vg4M_05L24_R_0	BY933576
E_EL_vg4M_05M01_F_0	BY924967
E_EL_vg4M_05M01_R_0	BY933577
E_EL_vg4M_05M02_F_0	BY924968
E_EL_vg4M_05M02_R_0	BY933578
E_EL_vg4M_05M03_F_0	BY924969
E_EL_vg4M_05M03_R_0	BY933579
E_EL_vg4M_05M04_F_0	BY924970
E_EL_vg4M_05M04_R_0	BY933580
E_EL_vg4M_05M05_F_0	BY924971
E_EL_vg4M_05M05_R_0	BY933581
E_EL_vg4M_05M06_F_0	BY924972
E_EL_vg4M_05M06_R_0	BY933582
E_EL_vg4M_05M07_R_0	BY933583
E_EL_vg4M_05M08_F_0	BY924973
E_EL_vg4M_05M08_R_0	BY933584
E_EL_vg4M_05M09_F_0	BY924974
E_EL_vg4M_05M09_R_0	BY933585
E_EL_vg4M_05M10_F_0	BY924975
E_EL_vg4M_05M10_R_0	BY933586

E_EL_vg4M_05M11_R_0	BY933587
E_EL_vg4M_05M12_F_0	BY924976
E_EL_vg4M_05M12_R_0	BY933588
E_EL_vg4M_05M13_F_0	BY924977
E_EL_vg4M_05M14_F_0	BY924978
E_EL_vg4M_05M14_R_0	BY933589
E_EL_vg4M_05M15_F_0	BY924979
E_EL_vg4M_05M15_R_0	BY933590
E_EL_vg4M_05M16_F_0	BY924980
E_EL_vg4M_05M16_R_0	BY933591
E_EL_vg4M_05M17_F_0	BY924981
E_EL_vg4M_05M17_R_0	BY933592
E_EL_vg4M_05M18_R_0	BY933593
E_EL_vg4M_05M20_R_0	BY933594
E_EL_vg4M_05M21_R_0	BY933595
E_EL_vg4M_05M22_R_0	BY933596
E_EL_vg4M_05M24_R_0	BY933597
E_EL_vg4M_05N01_R_0	BY933598
E_EL_vg4M_05N02_F_0	BY924982
E_EL_vg4M_05N02_R_0	BY933599
E_EL_vg4M_05N03_F_0	BY924983
E_EL_vg4M_05N03_R_0	BY933600
E_EL_vg4M_05N04_F_0	BY924984
E_EL_vg4M_05N04_R_0	BY933601
E_EL_vg4M_05N05_F_0	BY924985
E_EL_vg4M_05N05_R_0	BY933602
E_EL_vg4M_05N06_F_0	BY924986
E_EL_vg4M_05N06_R_0	BY933603
E_EL_vg4M_05N07_R_0	BY933604
E_EL_vg4M_05N08_F_0	BY924987
E_EL_vg4M_05N08_R_0	BY933605
E_EL_vg4M_05N10_F_0	BY924988
E_EL_vg4M_05N10_R_0	BY933606
E_EL_vg4M_05N11_F_0	BY924989
E_EL_vg4M_05N11_R_0	BY933607
E_EL_vg4M_05N12_F_0	BY924990
E_EL_vg4M_05N12_R_0	BY933608
E_EL_vg4M_05N14_R_0	BY933609
E_EL_vg4M_05N15_F_0	BY924991
E_EL_vg4M_05N15_R_0	BY933610
E_EL_vg4M_05N16_F_0	BY924992
E_EL_vg4M_05N16_R_0	BY933611
E_EL_vg4M_05N17_F_0	BY924993
E_EL_vg4M_05N17_R_0	BY933612
E_EL_vg4M_05N18_F_0	BY924994
E_EL_vg4M_05N18_R_0	BY933613
E_EL_vg4M_05N19_F_0	BY924995
E_EL_vg4M_05N19_R_0	BY933614
E_EL_vg4M_05N20_R_0	BY933615
E_EL_vg4M_05N21_R_0	BY933616
E_EL_vg4M_05N22_F_0	BY924996
E_EL_vg4M_05N22_R_0	BY933617
E_EL_vg4M_05N23_R_0	BY933618
E_EL_vg4M_05N24_F_0	BY924997

E_EL_vg4M_05N24_R_0	BY933619
E_EL_vg4M_05O01_F_0	BY924998
E_EL_vg4M_05O01_R_0	BY933620
E_EL_vg4M_05O02_F_0	BY924999
E_EL_vg4M_05O02_R_0	BY933621
E_EL_vg4M_05O03_F_0	BY925000
E_EL_vg4M_05O03_R_0	BY933622
E_EL_vg4M_05O04_R_0	BY933623
E_EL_vg4M_05O05_F_0	BY925001
E_EL_vg4M_05O05_R_0	BY933624
E_EL_vg4M_05O06_F_0	BY925002
E_EL_vg4M_05O06_R_0	BY933625
E_EL_vg4M_05O07_F_0	BY925003
E_EL_vg4M_05O07_R_0	BY933626
E_EL_vg4M_05O08_F_0	BY925004
E_EL_vg4M_05O08_R_0	BY933627
E_EL_vg4M_05O09_R_0	BY933628
E_EL_vg4M_05O10_F_0	BY925005
E_EL_vg4M_05O10_R_0	BY933629
E_EL_vg4M_05O12_F_0	BY925006
E_EL_vg4M_05O12_R_0	BY933630
E_EL_vg4M_05O13_F_0	BY925007
E_EL_vg4M_05O13_R_0	BY933631
E_EL_vg4M_05O14_R_0	BY933632
E_EL_vg4M_05O15_F_0	BY925008
E_EL_vg4M_05O15_R_0	BY933633
E_EL_vg4M_05O18_F_0	BY925009
E_EL_vg4M_05O18_R_0	BY933634
E_EL_vg4M_05O19_F_0	BY925010
E_EL_vg4M_05O19_R_0	BY933635
E_EL_vg4M_05O21_F_0	BY925011
E_EL_vg4M_05O21_R_0	BY933636
E_EL_vg4M_05O22_R_0	BY933637
E_EL_vg4M_05O23_F_0	BY925012
E_EL_vg4M_05O23_R_0	BY933638
E_EL_vg4M_05O24_F_0	BY925013
E_EL_vg4M_05O24_R_0	BY933639
E_EL_vg4M_05P01_F_0	BY925014
E_EL_vg4M_05P01_R_0	BY933640
E_EL_vg4M_05P02_F_0	BY925015
E_EL_vg4M_05P02_R_0	BY933641
E_EL_vg4M_05P03_F_0	BY925016
E_EL_vg4M_05P03_R_0	BY933642
E_EL_vg4M_05P05_F_0	BY925017
E_EL_vg4M_05P05_R_0	BY933643
E_EL_vg4M_05P06_F_0	BY925018
E_EL_vg4M_05P06_R_0	BY933644
E_EL_vg4M_05P07_F_0	BY925019
E_EL_vg4M_05P07_R_0	BY933645
E_EL_vg4M_05P08_F_0	BY925020
E_EL_vg4M_05P08_R_0	BY933646
E_EL_vg4M_05P09_F_0	BY925021
E_EL_vg4M_05P09_R_0	BY933647
E_EL_vg4M_05P12_F_0	BY925022

E_EL_vg4M_05P12_R_0	BY933648
E_EL_vg4M_05P13_F_0	BY925023
E_EL_vg4M_05P13_R_0	BY933649
E_EL_vg4M_05P14_F_0	BY925024
E_EL_vg4M_05P14_R_0	BY933650
E_EL_vg4M_05P16_F_0	BY925025
E_EL_vg4M_05P16_R_0	BY933651
E_EL_vg4M_05P17_F_0	BY925026
E_EL_vg4M_05P17_R_0	BY933652
E_EL_vg4M_05P18_F_0	BY925027
E_EL_vg4M_05P18_R_0	BY933653
E_EL_vg4M_05P19_F_0	BY925028
E_EL_vg4M_05P19_R_0	BY933654
E_EL_vg4M_05P20_R_0	BY933655
E_EL_vg4M_05P21_F_0	BY925029
E_EL_vg4M_05P21_R_0	BY933656
E_EL_vg4M_05P22_F_0	BY925030
E_EL_vg4M_05P22_R_0	BY933657
E_EL_vg4M_05P23_F_0	BY925031
E_EL_vg4M_05P23_R_0	BY933658
E_EL_vg4M_05P24_F_0	BY925032
E_EL_vg4M_05P24_R_0	BY933659
E_EL_vg4M_06A02_R_0	BY933660
E_EL_vg4M_06A04_F_0	BY925033
E_EL_vg4M_06A04_R_0	BY933661
E_EL_vg4M_06A06_F_0	BY925034
E_EL_vg4M_06A06_R_0	BY933662
E_EL_vg4M_06A07_F_0	BY925035
E_EL_vg4M_06A07_R_0	BY933663
E_EL_vg4M_06A08_F_0	BY925036
E_EL_vg4M_06A09_F_0	BY925037
E_EL_vg4M_06A10_F_0	BY925038
E_EL_vg4M_06A10_R_0	BY933664
E_EL_vg4M_06A11_F_0	BY925039
E_EL_vg4M_06A11_R_0	BY933665
E_EL_vg4M_06A12_R_0	BY933666
E_EL_vg4M_06A13_F_0	BY925040
E_EL_vg4M_06A13_R_0	BY933667
E_EL_vg4M_06A15_F_0	BY925041
E_EL_vg4M_06A15_R_0	BY933668
E_EL_vg4M_06A17_R_0	BY933669
E_EL_vg4M_06A18_F_0	BY925042
E_EL_vg4M_06A18_R_0	BY933670
E_EL_vg4M_06A19_F_0	BY925043
E_EL_vg4M_06A19_R_0	BY933671
E_EL_vg4M_06A20_F_0	BY925044
E_EL_vg4M_06A20_R_0	BY933672
E_EL_vg4M_06A21_F_0	BY925045
E_EL_vg4M_06A21_R_0	BY933673
E_EL_vg4M_06A22_R_0	BY933674
E_EL_vg4M_06A23_R_0	BY933675
E_EL_vg4M_06A24_F_0	BY925046
E_EL_vg4M_06A24_R_0	BY933676
E_EL_vg4M_06B01_R_0	BY933677

E_EL_vg4M_06B02_F_0	BY925047
E_EL_vg4M_06B02_R_0	BY933678
E_EL_vg4M_06B04_F_0	BY925048
E_EL_vg4M_06B04_R_0	BY933679
E_EL_vg4M_06B05_F_0	BY925049
E_EL_vg4M_06B05_R_0	BY933680
E_EL_vg4M_06B06_R_0	BY933681
E_EL_vg4M_06B07_F_0	BY925050
E_EL_vg4M_06B07_R_0	BY933682
E_EL_vg4M_06B08_F_0	BY925051
E_EL_vg4M_06B08_R_0	BY933683
E_EL_vg4M_06B09_R_0	BY933684
E_EL_vg4M_06B10_R_0	BY933685
E_EL_vg4M_06B11_F_0	BY925052
E_EL_vg4M_06B11_R_0	BY933686
E_EL_vg4M_06B12_R_0	BY933687
E_EL_vg4M_06B13_R_0	BY933688
E_EL_vg4M_06B14_F_0	BY925053
E_EL_vg4M_06B15_R_0	BY933689
E_EL_vg4M_06B16_F_0	BY925054
E_EL_vg4M_06B16_R_0	BY933690
E_EL_vg4M_06B17_R_0	BY933691
E_EL_vg4M_06B18_F_0	BY925055
E_EL_vg4M_06B18_R_0	BY933692
E_EL_vg4M_06B19_F_0	BY925056
E_EL_vg4M_06B19_R_0	BY933693
E_EL_vg4M_06B20_F_0	BY925057
E_EL_vg4M_06B20_R_0	BY933694
E_EL_vg4M_06B21_F_0	BY925058
E_EL_vg4M_06B21_R_0	BY933695
E_EL_vg4M_06B22_R_0	BY933696
E_EL_vg4M_06B23_F_0	BY925059
E_EL_vg4M_06B23_R_0	BY933697
E_EL_vg4M_06B24_F_0	BY925060
E_EL_vg4M_06B24_R_0	BY933698
E_EL_vg4M_06C01_F_0	BY925061
E_EL_vg4M_06C01_R_0	BY933699
E_EL_vg4M_06C02_R_0	BY933700
E_EL_vg4M_06C03_R_0	BY933701
E_EL_vg4M_06C04_F_0	BY925062
E_EL_vg4M_06C04_R_0	BY933702
E_EL_vg4M_06C05_F_0	BY925063
E_EL_vg4M_06C05_R_0	BY933703
E_EL_vg4M_06C06_R_0	BY933704
E_EL_vg4M_06C07_F_0	BY925064
E_EL_vg4M_06C08_F_0	BY925065
E_EL_vg4M_06C10_F_0	BY925066
E_EL_vg4M_06C10_R_0	BY933705
E_EL_vg4M_06C12_R_0	BY933706
E_EL_vg4M_06C13_F_0	BY925067
E_EL_vg4M_06C13_R_0	BY933707
E_EL_vg4M_06C14_R_0	BY933708
E_EL_vg4M_06C16_F_0	BY925068
E_EL_vg4M_06C16_R_0	BY933709

E_EL_vg4M_06C17_R_0	BY933710
E_EL_vg4M_06C18_R_0	BY933711
E_EL_vg4M_06C19_R_0	BY933712
E_EL_vg4M_06C20_R_0	BY933713
E_EL_vg4M_06C21_F_0	BY925069
E_EL_vg4M_06C21_R_0	BY933714
E_EL_vg4M_06C22_F_0	BY925070
E_EL_vg4M_06C22_R_0	BY933715
E_EL_vg4M_06C24_R_0	BY933716
E_EL_vg4M_06D02_F_0	BY925071
E_EL_vg4M_06D03_R_0	BY933717
E_EL_vg4M_06D04_F_0	BY925072
E_EL_vg4M_06D04_R_0	BY933718
E_EL_vg4M_06D06_R_0	BY933719
E_EL_vg4M_06D08_R_0	BY933720
E_EL_vg4M_06D09_R_0	BY933721
E_EL_vg4M_06D10_F_0	BY925073
E_EL_vg4M_06D10_R_0	BY933722
E_EL_vg4M_06D11_F_0	BY925074
E_EL_vg4M_06D11_R_0	BY933723
E_EL_vg4M_06D12_R_0	BY933724
E_EL_vg4M_06D13_R_0	BY933725
E_EL_vg4M_06D15_F_0	BY925075
E_EL_vg4M_06D15_R_0	BY933726
E_EL_vg4M_06D16_R_0	BY933727
E_EL_vg4M_06D19_R_0	BY933728
E_EL_vg4M_06D20_F_0	BY925076
E_EL_vg4M_06D22_F_0	BY925077
E_EL_vg4M_06D22_R_0	BY933729
E_EL_vg4M_06D23_F_0	BY925078
E_EL_vg4M_06D23_R_0	BY933730
E_EL_vg4M_06D24_F_0	BY925079
E_EL_vg4M_06D24_R_0	BY933731
E_EL_vg4M_06E01_F_0	BY925080
E_EL_vg4M_06E02_F_0	BY925081
E_EL_vg4M_06E02_R_0	BY933732
E_EL_vg4M_06E03_F_0	BY925082
E_EL_vg4M_06E03_R_0	BY933733
E_EL_vg4M_06E04_F_0	BY925083
E_EL_vg4M_06E04_R_0	BY933734
E_EL_vg4M_06E05_F_0	BY925084
E_EL_vg4M_06E05_R_0	BY933735
E_EL_vg4M_06E06_R_0	BY933736
E_EL_vg4M_06E07_R_0	BY933737
E_EL_vg4M_06E09_F_0	BY925085
E_EL_vg4M_06E09_R_0	BY933738
E_EL_vg4M_06E10_F_0	BY925086
E_EL_vg4M_06E10_R_0	BY933739
E_EL_vg4M_06E11_R_0	BY933740
E_EL_vg4M_06E14_R_0	BY933741
E_EL_vg4M_06E16_F_0	BY925087
E_EL_vg4M_06E16_R_0	BY933742
E_EL_vg4M_06E17_F_0	BY925088
E_EL_vg4M_06E17_R_0	BY933743

E_EL_vg4M_06E18_F_0	BY925089
E_EL_vg4M_06E18_R_0	BY933744
E_EL_vg4M_06E19_R_0	BY933745
E_EL_vg4M_06E20_F_0	BY925090
E_EL_vg4M_06E20_R_0	BY933746
E_EL_vg4M_06E21_R_0	BY933747
E_EL_vg4M_06E22_F_0	BY925091
E_EL_vg4M_06E22_R_0	BY933748
E_EL_vg4M_06E23_F_0	BY925092
E_EL_vg4M_06E23_R_0	BY933749
E_EL_vg4M_06F01_R_0	BY933750
E_EL_vg4M_06F02_F_0	BY925093
E_EL_vg4M_06F02_R_0	BY933751
E_EL_vg4M_06F03_R_0	BY933752
E_EL_vg4M_06F04_F_0	BY925094
E_EL_vg4M_06F04_R_0	BY933753
E_EL_vg4M_06F05_R_0	BY933754
E_EL_vg4M_06F07_R_0	BY933755
E_EL_vg4M_06F08_F_0	BY925095
E_EL_vg4M_06F08_R_0	BY933756
E_EL_vg4M_06F09_F_0	BY925096
E_EL_vg4M_06F09_R_0	BY933757
E_EL_vg4M_06F11_R_0	BY933758
E_EL_vg4M_06F13_R_0	BY933759
E_EL_vg4M_06F14_F_0	BY925097
E_EL_vg4M_06F14_R_0	BY933760
E_EL_vg4M_06F15_F_0	BY925098
E_EL_vg4M_06F15_R_0	BY933761
E_EL_vg4M_06F16_R_0	BY933762
E_EL_vg4M_06F17_R_0	BY933763
E_EL_vg4M_06F18_F_0	BY925099
E_EL_vg4M_06F20_F_0	BY925100
E_EL_vg4M_06F20_R_0	BY933764
E_EL_vg4M_06F21_F_0	BY925101
E_EL_vg4M_06F21_R_0	BY933765
E_EL_vg4M_06F22_F_0	BY925102
E_EL_vg4M_06F22_R_0	BY933766
E_EL_vg4M_06F23_R_0	BY933767
E_EL_vg4M_06G01_R_0	BY933768
E_EL_vg4M_06G02_R_0	BY933769
E_EL_vg4M_06G03_F_0	BY925103
E_EL_vg4M_06G03_R_0	BY933770
E_EL_vg4M_06G04_R_0	BY933771
E_EL_vg4M_06G05_R_0	BY933772
E_EL_vg4M_06G06_R_0	BY933773
E_EL_vg4M_06G07_F_0	BY925104
E_EL_vg4M_06G08_F_0	BY925105
E_EL_vg4M_06G08_R_0	BY933774
E_EL_vg4M_06G09_F_0	BY925106
E_EL_vg4M_06G09_R_0	BY933775
E_EL_vg4M_06G10_F_0	BY925107
E_EL_vg4M_06G10_R_0	BY933776
E_EL_vg4M_06G12_R_0	BY933777
E_EL_vg4M_06G13_F_0	BY925108

E_EL_vg4M_06G13_R_0	BY933778
E_EL_vg4M_06G14_F_0	BY925109
E_EL_vg4M_06G14_R_0	BY933779
E_EL_vg4M_06G15_R_0	BY933780
E_EL_vg4M_06G16_R_0	BY933781
E_EL_vg4M_06G17_R_0	BY933782
E_EL_vg4M_06G20_F_0	BY925110
E_EL_vg4M_06G20_R_0	BY933783
E_EL_vg4M_06G21_F_0	BY925111
E_EL_vg4M_06G22_R_0	BY933784
E_EL_vg4M_06G23_F_0	BY925112
E_EL_vg4M_06G23_R_0	BY933785
E_EL_vg4M_06G24_F_0	BY925113
E_EL_vg4M_06G24_R_0	BY933786
E_EL_vg4M_06H01_R_0	BY933787
E_EL_vg4M_06H02_F_0	BY925114
E_EL_vg4M_06H02_R_0	BY933788
E_EL_vg4M_06H03_F_0	BY925115
E_EL_vg4M_06H03_R_0	BY933789
E_EL_vg4M_06H04_F_0	BY925116
E_EL_vg4M_06H05_F_0	BY925117
E_EL_vg4M_06H05_R_0	BY933790
E_EL_vg4M_06H07_F_0	BY925118
E_EL_vg4M_06H07_R_0	BY933791
E_EL_vg4M_06H08_F_0	BY925119
E_EL_vg4M_06H08_R_0	BY933792
E_EL_vg4M_06H10_F_0	BY925120
E_EL_vg4M_06H11_R_0	BY933793
E_EL_vg4M_06H13_F_0	BY925121
E_EL_vg4M_06H13_R_0	BY933794
E_EL_vg4M_06H14_F_0	BY925122
E_EL_vg4M_06H15_F_0	BY925123
E_EL_vg4M_06H15_R_0	BY933795
E_EL_vg4M_06H16_F_0	BY925124
E_EL_vg4M_06H16_R_0	BY933796
E_EL_vg4M_06H17_R_0	BY933797
E_EL_vg4M_06H19_F_0	BY925125
E_EL_vg4M_06H19_R_0	BY933798
E_EL_vg4M_06H20_R_0	BY933799
E_EL_vg4M_06H21_F_0	BY925126
E_EL_vg4M_06H21_R_0	BY933800
E_EL_vg4M_06H22_R_0	BY933801
E_EL_vg4M_06H23_F_0	BY925127
E_EL_vg4M_06H23_R_0	BY933802
E_EL_vg4M_06H24_F_0	BY925128
E_EL_vg4M_06H24_R_0	BY933803
E_EL_vg4M_06I01_F_0	BY925129
E_EL_vg4M_06I01_R_0	BY933804
E_EL_vg4M_06I02_R_0	BY933805
E_EL_vg4M_06I03_F_0	BY925130
E_EL_vg4M_06I03_R_0	BY933806
E_EL_vg4M_06I04_F_0	BY925131
E_EL_vg4M_06I04_R_0	BY933807
E_EL_vg4M_06I05_R_0	BY933808

E_EL_vg4M_06I06_F_0	BY925132
E_EL_vg4M_06I06_R_0	BY933809
E_EL_vg4M_06I07_F_0	BY925133
E_EL_vg4M_06I07_R_0	BY933810
E_EL_vg4M_06I08_F_0	BY925134
E_EL_vg4M_06I08_R_0	BY933811
E_EL_vg4M_06I09_F_0	BY925135
E_EL_vg4M_06I09_R_0	BY933812
E_EL_vg4M_06I10_F_0	BY925136
E_EL_vg4M_06I10_R_0	BY933813
E_EL_vg4M_06I11_F_0	BY925137
E_EL_vg4M_06I11_R_0	BY933814
E_EL_vg4M_06I12_F_0	BY925138
E_EL_vg4M_06I12_R_0	BY933815
E_EL_vg4M_06I13_R_0	BY933816
E_EL_vg4M_06I14_F_0	BY925139
E_EL_vg4M_06I14_R_0	BY933817
E_EL_vg4M_06I15_R_0	BY933818
E_EL_vg4M_06I16_R_0	BY933819
E_EL_vg4M_06I18_F_0	BY925140
E_EL_vg4M_06I18_R_0	BY933820
E_EL_vg4M_06I19_F_0	BY925141
E_EL_vg4M_06I19_R_0	BY933821
E_EL_vg4M_06I20_F_0	BY925142
E_EL_vg4M_06I20_R_0	BY933822
E_EL_vg4M_06I21_F_0	BY925143
E_EL_vg4M_06I21_R_0	BY933823
E_EL_vg4M_06I22_R_0	BY933824
E_EL_vg4M_06I23_F_0	BY925144
E_EL_vg4M_06I23_R_0	BY933825
E_EL_vg4M_06I24_R_0	BY933826
E_EL_vg4M_06J01_F_0	BY925145
E_EL_vg4M_06J01_R_0	BY933827
E_EL_vg4M_06J02_F_0	BY925146
E_EL_vg4M_06J03_R_0	BY933828
E_EL_vg4M_06J04_F_0	BY925147
E_EL_vg4M_06J04_R_0	BY933829
E_EL_vg4M_06J05_F_0	BY925148
E_EL_vg4M_06J05_R_0	BY933830
E_EL_vg4M_06J06_F_0	BY925149
E_EL_vg4M_06J06_R_0	BY933831
E_EL_vg4M_06J07_R_0	BY933832
E_EL_vg4M_06J08_F_0	BY925150
E_EL_vg4M_06J08_R_0	BY933833
E_EL_vg4M_06J09_F_0	BY925151
E_EL_vg4M_06J09_R_0	BY933834
E_EL_vg4M_06J10_R_0	BY933835
E_EL_vg4M_06J11_F_0	BY925152
E_EL_vg4M_06J11_R_0	BY933836
E_EL_vg4M_06J12_R_0	BY933837
E_EL_vg4M_06J13_R_0	BY933838
E_EL_vg4M_06J15_F_0	BY925153
E_EL_vg4M_06J15_R_0	BY933839
E_EL_vg4M_06J16_R_0	BY933840

E_EL_vg4M_06J18_R_0	BY933841
E_EL_vg4M_06J19_F_0	BY925154
E_EL_vg4M_06J19_R_0	BY933842
E_EL_vg4M_06J20_F_0	BY925155
E_EL_vg4M_06J21_R_0	BY933843
E_EL_vg4M_06J22_R_0	BY933844
E_EL_vg4M_06J23_F_0	BY925156
E_EL_vg4M_06J23_R_0	BY933845
E_EL_vg4M_06J24_R_0	BY933846
E_EL_vg4M_06K01_F_0	BY925157
E_EL_vg4M_06K01_R_0	BY933847
E_EL_vg4M_06K02_R_0	BY933848
E_EL_vg4M_06K03_F_0	BY925158
E_EL_vg4M_06K03_R_0	BY933849
E_EL_vg4M_06K05_R_0	BY933850
E_EL_vg4M_06K06_F_0	BY925159
E_EL_vg4M_06K06_R_0	BY933851
E_EL_vg4M_06K07_R_0	BY933852
E_EL_vg4M_06K09_F_0	BY925160
E_EL_vg4M_06K09_R_0	BY933853
E_EL_vg4M_06K10_F_0	BY925161
E_EL_vg4M_06K10_R_0	BY933854
E_EL_vg4M_06K11_R_0	BY933855
E_EL_vg4M_06K12_R_0	BY933856
E_EL_vg4M_06K13_R_0	BY933857
E_EL_vg4M_06K14_R_0	BY933858
E_EL_vg4M_06K15_R_0	BY933859
E_EL_vg4M_06K16_R_0	BY933860
E_EL_vg4M_06K17_F_0	BY925162
E_EL_vg4M_06K17_R_0	BY933861
E_EL_vg4M_06K18_R_0	BY933862
E_EL_vg4M_06K19_F_0	BY925163
E_EL_vg4M_06K19_R_0	BY933863
E_EL_vg4M_06K20_F_0	BY925164
E_EL_vg4M_06K20_R_0	BY933864
E_EL_vg4M_06K22_R_0	BY933865
E_EL_vg4M_06L01_R_0	BY933866
E_EL_vg4M_06L02_R_0	BY933867
E_EL_vg4M_06L03_F_0	BY925165
E_EL_vg4M_06L03_R_0	BY933868
E_EL_vg4M_06L04_F_0	BY925166
E_EL_vg4M_06L04_R_0	BY933869
E_EL_vg4M_06L07_R_0	BY933870
E_EL_vg4M_06L08_R_0	BY933871
E_EL_vg4M_06L09_R_0	BY933872
E_EL_vg4M_06L10_R_0	BY933873
E_EL_vg4M_06L11_R_0	BY933874
E_EL_vg4M_06L12_R_0	BY933875
E_EL_vg4M_06L13_F_0	BY925167
E_EL_vg4M_06L13_R_0	BY933876
E_EL_vg4M_06L14_F_0	BY925168
E_EL_vg4M_06L14_R_0	BY933877
E_EL_vg4M_06L15_R_0	BY933878
E_EL_vg4M_06L17_R_0	BY933879

E_EL_vg4M_06L18_R_0	BY933880
E_EL_vg4M_06L19_R_0	BY933881
E_EL_vg4M_06L21_R_0	BY933882
E_EL_vg4M_06L22_F_0	BY925169
E_EL_vg4M_06L22_R_0	BY933883
E_EL_vg4M_06L23_R_0	BY933884
E_EL_vg4M_06L24_F_0	BY925170
E_EL_vg4M_06L24_R_0	BY933885
E_EL_vg4M_06M02_R_0	BY933886
E_EL_vg4M_06M03_F_0	BY925171
E_EL_vg4M_06M03_R_0	BY933887
E_EL_vg4M_06M04_R_0	BY933888
E_EL_vg4M_06M05_F_0	BY925172
E_EL_vg4M_06M05_R_0	BY933889
E_EL_vg4M_06M06_F_0	BY925173
E_EL_vg4M_06M06_R_0	BY933890
E_EL_vg4M_06M07_R_0	BY933891
E_EL_vg4M_06M08_F_0	BY925174
E_EL_vg4M_06M08_R_0	BY933892
E_EL_vg4M_06M09_F_0	BY925175
E_EL_vg4M_06M09_R_0	BY933893
E_EL_vg4M_06M10_F_0	BY925176
E_EL_vg4M_06M10_R_0	BY933894
E_EL_vg4M_06M11_R_0	BY933895
E_EL_vg4M_06M12_R_0	BY933896
E_EL_vg4M_06M13_F_0	BY925177
E_EL_vg4M_06M13_R_0	BY933897
E_EL_vg4M_06M14_R_0	BY933898
E_EL_vg4M_06M15_R_0	BY933899
E_EL_vg4M_06M16_R_0	BY933900
E_EL_vg4M_06M18_R_0	BY933901
E_EL_vg4M_06M19_R_0	BY933902
E_EL_vg4M_06M20_R_0	BY933903
E_EL_vg4M_06M22_R_0	BY933904
E_EL_vg4M_06M23_R_0	BY933905
E_EL_vg4M_06M24_R_0	BY933906
E_EL_vg4M_06N01_R_0	BY933907
E_EL_vg4M_06N02_F_0	BY925178
E_EL_vg4M_06N02_R_0	BY933908
E_EL_vg4M_06N04_F_0	BY925179
E_EL_vg4M_06N04_R_0	BY933909
E_EL_vg4M_06N05_F_0	BY925180
E_EL_vg4M_06N05_R_0	BY933910
E_EL_vg4M_06N06_R_0	BY933911
E_EL_vg4M_06N07_F_0	BY925181
E_EL_vg4M_06N08_F_0	BY925182
E_EL_vg4M_06N08_R_0	BY933912
E_EL_vg4M_06N10_F_0	BY925183
E_EL_vg4M_06N10_R_0	BY933913
E_EL_vg4M_06N11_R_0	BY933914
E_EL_vg4M_06N12_R_0	BY933915
E_EL_vg4M_06N13_R_0	BY933916
E_EL_vg4M_06N14_R_0	BY933917
E_EL_vg4M_06N15_R_0	BY933918

E_EL_vg4M_06N18_F_0	BY925184
E_EL_vg4M_06N18_R_0	BY933919
E_EL_vg4M_06N19_R_0	BY933920
E_EL_vg4M_06N20_R_0	BY933921
E_EL_vg4M_06N21_R_0	BY933922
E_EL_vg4M_06N22_R_0	BY933923
E_EL_vg4M_06N23_F_0	BY925185
E_EL_vg4M_06N23_R_0	BY933924
E_EL_vg4M_06N24_F_0	BY925186
E_EL_vg4M_06N24_R_0	BY933925
E_EL_vg4M_06O01_R_0	BY933926
E_EL_vg4M_06O02_F_0	BY925187
E_EL_vg4M_06O02_R_0	BY933927
E_EL_vg4M_06O03_R_0	BY933928
E_EL_vg4M_06O04_F_0	BY925188
E_EL_vg4M_06O04_R_0	BY933929
E_EL_vg4M_06O05_F_0	BY925189
E_EL_vg4M_06O05_R_0	BY933930
E_EL_vg4M_06O06_F_0	BY925190
E_EL_vg4M_06O06_R_0	BY933931
E_EL_vg4M_06O07_R_0	BY933932
E_EL_vg4M_06O08_F_0	BY925191
E_EL_vg4M_06O08_R_0	BY933933
E_EL_vg4M_06O09_F_0	BY925192
E_EL_vg4M_06O09_R_0	BY933934
E_EL_vg4M_06O10_F_0	BY925193
E_EL_vg4M_06O10_R_0	BY933935
E_EL_vg4M_06O11_R_0	BY933936
E_EL_vg4M_06O12_F_0	BY925194
E_EL_vg4M_06O12_R_0	BY933937
E_EL_vg4M_06O13_R_0	BY933938
E_EL_vg4M_06O14_F_0	BY925195
E_EL_vg4M_06O14_R_0	BY933939
E_EL_vg4M_06O15_F_0	BY925196
E_EL_vg4M_06O15_R_0	BY933940
E_EL_vg4M_06O16_R_0	BY933941
E_EL_vg4M_06O17_F_0	BY925197
E_EL_vg4M_06O17_R_0	BY933942
E_EL_vg4M_06O18_F_0	BY925198
E_EL_vg4M_06O19_F_0	BY925199
E_EL_vg4M_06O20_F_0	BY925200
E_EL_vg4M_06O20_R_0	BY933943
E_EL_vg4M_06O21_F_0	BY925201
E_EL_vg4M_06O21_R_0	BY933944
E_EL_vg4M_06O22_F_0	BY925202
E_EL_vg4M_06O22_R_0	BY933945
E_EL_vg4M_06O23_F_0	BY925203
E_EL_vg4M_06O23_R_0	BY933946
E_EL_vg4M_06O24_F_0	BY925204
E_EL_vg4M_06O24_R_0	BY933947
E_EL_vg4M_06P01_F_0	BY925205
E_EL_vg4M_06P01_R_0	BY933948
E_EL_vg4M_06P02_F_0	BY925206
E_EL_vg4M_06P02_R_0	BY933949

E_EL_vg4M_06P03_F_0	BY925207
E_EL_vg4M_06P03_R_0	BY933950
E_EL_vg4M_06P04_F_0	BY925208
E_EL_vg4M_06P04_R_0	BY933951
E_EL_vg4M_06P05_R_0	BY933952
E_EL_vg4M_06P06_F_0	BY925209
E_EL_vg4M_06P08_F_0	BY925210
E_EL_vg4M_06P08_R_0	BY933953
E_EL_vg4M_06P09_F_0	BY925211
E_EL_vg4M_06P09_R_0	BY933954
E_EL_vg4M_06P11_R_0	BY933955
E_EL_vg4M_06P12_F_0	BY925212
E_EL_vg4M_06P14_R_0	BY933956
E_EL_vg4M_06P15_R_0	BY933957
E_EL_vg4M_06P16_R_0	BY933958
E_EL_vg4M_06P17_F_0	BY925213
E_EL_vg4M_06P17_R_0	BY933959
E_EL_vg4M_06P18_F_0	BY925214
E_EL_vg4M_06P19_F_0	BY925215
E_EL_vg4M_06P19_R_0	BY933960
E_EL_vg4M_06P22_F_0	BY925216
E_EL_vg4M_06P22_R_0	BY933961
E_EL_vg4M_06P23_F_0	BY925217
E_EL_vg4M_06P23_R_0	BY933962
E_EL_vg4M_06P24_F_0	BY925218
E_EL_vg4M_06P24_R_0	BY933963
E_EL_vg4M_07A01_F_0	BY925219
E_EL_vg4M_07A02_F_0	BY925220
E_EL_vg4M_07A02_R_0	BY933964
E_EL_vg4M_07A03_R_0	BY933965
E_EL_vg4M_07A04_F_0	BY925221
E_EL_vg4M_07A04_R_0	BY933966
E_EL_vg4M_07A05_F_0	BY925222
E_EL_vg4M_07A05_R_0	BY933967
E_EL_vg4M_07A06_F_0	BY925223
E_EL_vg4M_07A06_R_0	BY933968
E_EL_vg4M_07A07_F_0	BY925224
E_EL_vg4M_07A08_F_0	BY925225
E_EL_vg4M_07A08_R_0	BY933969
E_EL_vg4M_07A09_F_0	BY925226
E_EL_vg4M_07A09_R_0	BY933970
E_EL_vg4M_07A10_F_0	BY925227
E_EL_vg4M_07A10_R_0	BY933971
E_EL_vg4M_07A12_F_0	BY925228
E_EL_vg4M_07A12_R_0	BY933972
E_EL_vg4M_07A13_F_0	BY925229
E_EL_vg4M_07A13_R_0	BY933973
E_EL_vg4M_07A14_F_0	BY925230
E_EL_vg4M_07A14_R_0	BY933974
E_EL_vg4M_07A15_F_0	BY925231
E_EL_vg4M_07A15_R_0	BY933975
E_EL_vg4M_07A16_F_0	BY925232
E_EL_vg4M_07A16_R_0	BY933976
E_EL_vg4M_07A17_F_0	BY925233

E_EL_vg4M_07A17_R_0	BY933977
E_EL_vg4M_07A18_F_0	BY925234
E_EL_vg4M_07A18_R_0	BY933978
E_EL_vg4M_07A19_F_0	BY925235
E_EL_vg4M_07A19_R_0	BY933979
E_EL_vg4M_07A20_F_0	BY925236
E_EL_vg4M_07A20_R_0	BY933980
E_EL_vg4M_07A21_F_0	BY925237
E_EL_vg4M_07A21_R_0	BY933981
E_EL_vg4M_07A22_F_0	BY925238
E_EL_vg4M_07A22_R_0	BY933982
E_EL_vg4M_07A23_F_0	BY925239
E_EL_vg4M_07A23_R_0	BY933983
E_EL_vg4M_07A24_F_0	BY925240
E_EL_vg4M_07A24_R_0	BY933984
E_EL_vg4M_07B01_F_0	BY925241
E_EL_vg4M_07B01_R_0	BY933985
E_EL_vg4M_07B03_F_0	BY925242
E_EL_vg4M_07B03_R_0	BY933986
E_EL_vg4M_07B04_F_0	BY925243
E_EL_vg4M_07B04_R_0	BY933987
E_EL_vg4M_07B05_F_0	BY925244
E_EL_vg4M_07B05_R_0	BY933988
E_EL_vg4M_07B06_F_0	BY925245
E_EL_vg4M_07B06_R_0	BY933989
E_EL_vg4M_07B08_F_0	BY925246
E_EL_vg4M_07B08_R_0	BY933990
E_EL_vg4M_07B10_F_0	BY925247
E_EL_vg4M_07B10_R_0	BY933991
E_EL_vg4M_07B11_F_0	BY925248
E_EL_vg4M_07B11_R_0	BY933992
E_EL_vg4M_07B12_F_0	BY925249
E_EL_vg4M_07B12_R_0	BY933993
E_EL_vg4M_07B13_F_0	BY925250
E_EL_vg4M_07B13_R_0	BY933994
E_EL_vg4M_07B14_F_0	BY925251
E_EL_vg4M_07B14_R_0	BY933995
E_EL_vg4M_07B15_F_0	BY925252
E_EL_vg4M_07B15_R_0	BY933996
E_EL_vg4M_07B16_F_0	BY925253
E_EL_vg4M_07B16_R_0	BY933997
E_EL_vg4M_07B17_F_0	BY925254
E_EL_vg4M_07B17_R_0	BY933998
E_EL_vg4M_07B19_F_0	BY925255
E_EL_vg4M_07B19_R_0	BY933999
E_EL_vg4M_07B20_F_0	BY925256
E_EL_vg4M_07B20_R_0	BY934000
E_EL_vg4M_07B21_F_0	BY925257
E_EL_vg4M_07B21_R_0	BY934001
E_EL_vg4M_07B22_F_0	BY925258
E_EL_vg4M_07B22_R_0	BY934002
E_EL_vg4M_07B23_F_0	BY925259
E_EL_vg4M_07B23_R_0	BY934003
E_EL_vg4M_07C01_F_0	BY925260

E_EL_vg4M_07C01_R_0	BY934004
E_EL_vg4M_07C02_F_0	BY925261
E_EL_vg4M_07C02_R_0	BY934005
E_EL_vg4M_07C03_F_0	BY925262
E_EL_vg4M_07C03_R_0	BY934006
E_EL_vg4M_07C04_F_0	BY925263
E_EL_vg4M_07C04_R_0	BY934007
E_EL_vg4M_07C05_F_0	BY925264
E_EL_vg4M_07C05_R_0	BY934008
E_EL_vg4M_07C06_F_0	BY925265
E_EL_vg4M_07C06_R_0	BY934009
E_EL_vg4M_07C07_R_0	BY934010
E_EL_vg4M_07C08_F_0	BY925266
E_EL_vg4M_07C08_R_0	BY934011
E_EL_vg4M_07C09_F_0	BY925267
E_EL_vg4M_07C09_R_0	BY934012
E_EL_vg4M_07C10_F_0	BY925268
E_EL_vg4M_07C10_R_0	BY934013
E_EL_vg4M_07C11_F_0	BY925269
E_EL_vg4M_07C12_F_0	BY925270
E_EL_vg4M_07C12_R_0	BY934014
E_EL_vg4M_07C13_R_0	BY934015
E_EL_vg4M_07C14_F_0	BY925271
E_EL_vg4M_07C14_R_0	BY934016
E_EL_vg4M_07C15_F_0	BY925272
E_EL_vg4M_07C15_R_0	BY934017
E_EL_vg4M_07C16_F_0	BY925273
E_EL_vg4M_07C16_R_0	BY934018
E_EL_vg4M_07C17_F_0	BY925274
E_EL_vg4M_07C17_R_0	BY934019
E_EL_vg4M_07C18_F_0	BY925275
E_EL_vg4M_07C18_R_0	BY934020
E_EL_vg4M_07C19_F_0	BY925276
E_EL_vg4M_07C21_F_0	BY925277
E_EL_vg4M_07C21_R_0	BY934021
E_EL_vg4M_07C22_F_0	BY925278
E_EL_vg4M_07C22_R_0	BY934022
E_EL_vg4M_07C23_F_0	BY925279
E_EL_vg4M_07C23_R_0	BY934023
E_EL_vg4M_07C24_F_0	BY925280
E_EL_vg4M_07D01_F_0	BY925281
E_EL_vg4M_07D01_R_0	BY934024
E_EL_vg4M_07D03_F_0	BY925282
E_EL_vg4M_07D03_R_0	BY934025
E_EL_vg4M_07D04_F_0	BY925283
E_EL_vg4M_07D04_R_0	BY934026
E_EL_vg4M_07D05_F_0	BY925284
E_EL_vg4M_07D06_F_0	BY925285
E_EL_vg4M_07D06_R_0	BY934027
E_EL_vg4M_07D07_F_0	BY925286
E_EL_vg4M_07D07_R_0	BY934028
E_EL_vg4M_07D08_F_0	BY925287
E_EL_vg4M_07D08_R_0	BY934029
E_EL_vg4M_07D09_F_0	BY925288

E_EL_vg4M_07D09_R_0	BY934030
E_EL_vg4M_07D10_F_0	BY925289
E_EL_vg4M_07D10_R_0	BY934031
E_EL_vg4M_07D11_F_0	BY925290
E_EL_vg4M_07D11_R_0	BY934032
E_EL_vg4M_07D12_R_0	BY934033
E_EL_vg4M_07D13_F_0	BY925291
E_EL_vg4M_07D13_R_0	BY934034
E_EL_vg4M_07D14_R_0	BY934035
E_EL_vg4M_07D16_F_0	BY925292
E_EL_vg4M_07D16_R_0	BY934036
E_EL_vg4M_07D17_R_0	BY934037
E_EL_vg4M_07D18_F_0	BY925293
E_EL_vg4M_07D18_R_0	BY934038
E_EL_vg4M_07D19_R_0	BY934039
E_EL_vg4M_07D20_F_0	BY925294
E_EL_vg4M_07D20_R_0	BY934040
E_EL_vg4M_07D21_F_0	BY925295
E_EL_vg4M_07D22_F_0	BY925296
E_EL_vg4M_07D22_R_0	BY934041
E_EL_vg4M_07D23_F_0	BY925297
E_EL_vg4M_07D23_R_0	BY934042
E_EL_vg4M_07D24_F_0	BY925298
E_EL_vg4M_07E01_F_0	BY925299
E_EL_vg4M_07E02_F_0	BY925300
E_EL_vg4M_07E02_R_0	BY934043
E_EL_vg4M_07E03_F_0	BY925301
E_EL_vg4M_07E03_R_0	BY934044
E_EL_vg4M_07E04_F_0	BY925302
E_EL_vg4M_07E04_R_0	BY934045
E_EL_vg4M_07E05_F_0	BY925303
E_EL_vg4M_07E05_R_0	BY934046
E_EL_vg4M_07E06_F_0	BY925304
E_EL_vg4M_07E08_F_0	BY925305
E_EL_vg4M_07E08_R_0	BY934047
E_EL_vg4M_07E10_F_0	BY925306
E_EL_vg4M_07E10_R_0	BY934048
E_EL_vg4M_07E11_F_0	BY925307
E_EL_vg4M_07E11_R_0	BY934049
E_EL_vg4M_07E12_F_0	BY925308
E_EL_vg4M_07E12_R_0	BY934050
E_EL_vg4M_07E13_F_0	BY925309
E_EL_vg4M_07E13_R_0	BY934051
E_EL_vg4M_07E14_F_0	BY925310
E_EL_vg4M_07E14_R_0	BY934052
E_EL_vg4M_07E15_F_0	BY925311
E_EL_vg4M_07E15_R_0	BY934053
E_EL_vg4M_07E16_F_0	BY925312
E_EL_vg4M_07E16_R_0	BY934054
E_EL_vg4M_07E17_F_0	BY925313
E_EL_vg4M_07E17_R_0	BY934055
E_EL_vg4M_07E18_F_0	BY925314
E_EL_vg4M_07E18_R_0	BY934056
E_EL_vg4M_07E20_F_0	BY925315

E_EL_vg4M_07E20_R_0	BY934057
E_EL_vg4M_07E21_F_0	BY925316
E_EL_vg4M_07E22_F_0	BY925317
E_EL_vg4M_07E22_R_0	BY934058
E_EL_vg4M_07E23_F_0	BY925318
E_EL_vg4M_07E23_R_0	BY934059
E_EL_vg4M_07E24_F_0	BY925319
E_EL_vg4M_07E24_R_0	BY934060
E_EL_vg4M_07F01_F_0	BY925320
E_EL_vg4M_07F01_R_0	BY934061
E_EL_vg4M_07F02_F_0	BY925321
E_EL_vg4M_07F02_R_0	BY934062
E_EL_vg4M_07F04_F_0	BY925322
E_EL_vg4M_07F04_R_0	BY934063
E_EL_vg4M_07F05_F_0	BY925323
E_EL_vg4M_07F05_R_0	BY934064
E_EL_vg4M_07F06_F_0	BY925324
E_EL_vg4M_07F06_R_0	BY934065
E_EL_vg4M_07F07_F_0	BY925325
E_EL_vg4M_07F07_R_0	BY934066
E_EL_vg4M_07F08_F_0	BY925326
E_EL_vg4M_07F08_R_0	BY934067
E_EL_vg4M_07F10_F_0	BY925327
E_EL_vg4M_07F10_R_0	BY934068
E_EL_vg4M_07F11_F_0	BY925328
E_EL_vg4M_07F11_R_0	BY934069
E_EL_vg4M_07F13_F_0	BY925329
E_EL_vg4M_07F13_R_0	BY934070
E_EL_vg4M_07F14_F_0	BY925330
E_EL_vg4M_07F14_R_0	BY934071
E_EL_vg4M_07F15_F_0	BY925331
E_EL_vg4M_07F16_F_0	BY925332
E_EL_vg4M_07F16_R_0	BY934072
E_EL_vg4M_07F17_F_0	BY925333
E_EL_vg4M_07F17_R_0	BY934073
E_EL_vg4M_07F19_F_0	BY925334
E_EL_vg4M_07F19_R_0	BY934074
E_EL_vg4M_07F20_F_0	BY925335
E_EL_vg4M_07F21_F_0	BY925336
E_EL_vg4M_07F21_R_0	BY934075
E_EL_vg4M_07F22_R_0	BY934076
E_EL_vg4M_07F23_F_0	BY925337
E_EL_vg4M_07F23_R_0	BY934077
E_EL_vg4M_07F24_F_0	BY925338
E_EL_vg4M_07F24_R_0	BY934078
E_EL_vg4M_07G01_F_0	BY925339
E_EL_vg4M_07G01_R_0	BY934079
E_EL_vg4M_07G02_F_0	BY925340
E_EL_vg4M_07G02_R_0	BY934080
E_EL_vg4M_07G03_F_0	BY925341
E_EL_vg4M_07G03_R_0	BY934081
E_EL_vg4M_07G04_F_0	BY925342
E_EL_vg4M_07G04_R_0	BY934082
E_EL_vg4M_07G05_F_0	BY925343

E_EL_vg4M_07G05_R_0	BY934083
E_EL_vg4M_07G06_F_0	BY925344
E_EL_vg4M_07G06_R_0	BY934084
E_EL_vg4M_07G07_F_0	BY925345
E_EL_vg4M_07G08_F_0	BY925346
E_EL_vg4M_07G08_R_0	BY934085
E_EL_vg4M_07G09_F_0	BY925347
E_EL_vg4M_07G09_R_0	BY934086
E_EL_vg4M_07G10_F_0	BY925348
E_EL_vg4M_07G11_F_0	BY925349
E_EL_vg4M_07G11_R_0	BY934087
E_EL_vg4M_07G12_F_0	BY925350
E_EL_vg4M_07G12_R_0	BY934088
E_EL_vg4M_07G13_F_0	BY925351
E_EL_vg4M_07G13_R_0	BY934089
E_EL_vg4M_07G15_F_0	BY925352
E_EL_vg4M_07G15_R_0	BY934090
E_EL_vg4M_07G16_F_0	BY925353
E_EL_vg4M_07G16_R_0	BY934091
E_EL_vg4M_07G17_R_0	BY934092
E_EL_vg4M_07G18_R_0	BY934093
E_EL_vg4M_07G19_F_0	BY925354
E_EL_vg4M_07G19_R_0	BY934094
E_EL_vg4M_07G20_F_0	BY925355
E_EL_vg4M_07G20_R_0	BY934095
E_EL_vg4M_07G21_F_0	BY925356
E_EL_vg4M_07G21_R_0	BY934096
E_EL_vg4M_07G22_F_0	BY925357
E_EL_vg4M_07G23_F_0	BY925358
E_EL_vg4M_07G23_R_0	BY934097
E_EL_vg4M_07G24_R_0	BY934098
E_EL_vg4M_07H01_F_0	BY925359
E_EL_vg4M_07H01_R_0	BY934099
E_EL_vg4M_07H02_F_0	BY925360
E_EL_vg4M_07H02_R_0	BY934100
E_EL_vg4M_07H03_F_0	BY925361
E_EL_vg4M_07H03_R_0	BY934101
E_EL_vg4M_07H04_F_0	BY925362
E_EL_vg4M_07H04_R_0	BY934102
E_EL_vg4M_07H05_R_0	BY934103
E_EL_vg4M_07H07_F_0	BY925363
E_EL_vg4M_07H07_R_0	BY934104
E_EL_vg4M_07H08_F_0	BY925364
E_EL_vg4M_07H09_R_0	BY934105
E_EL_vg4M_07H10_F_0	BY925365
E_EL_vg4M_07H13_F_0	BY925366
E_EL_vg4M_07H13_R_0	BY934106
E_EL_vg4M_07H14_F_0	BY925367
E_EL_vg4M_07H14_R_0	BY934107
E_EL_vg4M_07H15_F_0	BY925368
E_EL_vg4M_07H15_R_0	BY934108
E_EL_vg4M_07H16_F_0	BY925369
E_EL_vg4M_07H16_R_0	BY934109
E_EL_vg4M_07H17_F_0	BY925370

E_EL_vg4M_07H17_R_0	BY934110
E_EL_vg4M_07H18_F_0	BY925371
E_EL_vg4M_07H18_R_0	BY934111
E_EL_vg4M_07H19_F_0	BY925372
E_EL_vg4M_07H19_R_0	BY934112
E_EL_vg4M_07H20_F_0	BY925373
E_EL_vg4M_07H20_R_0	BY934113
E_EL_vg4M_07H21_F_0	BY925374
E_EL_vg4M_07H21_R_0	BY934114
E_EL_vg4M_07H23_F_0	BY925375
E_EL_vg4M_07H23_R_0	BY934115
E_EL_vg4M_07H24_F_0	BY925376
E_EL_vg4M_07H24_R_0	BY934116
E_EL_vg4M_07I01_F_0	BY925377
E_EL_vg4M_07I01_R_0	BY934117
E_EL_vg4M_07I02_F_0	BY925378
E_EL_vg4M_07I03_F_0	BY925379
E_EL_vg4M_07I03_R_0	BY934118
E_EL_vg4M_07I04_F_0	BY925380
E_EL_vg4M_07I04_R_0	BY934119
E_EL_vg4M_07I05_F_0	BY925381
E_EL_vg4M_07I05_R_0	BY934120
E_EL_vg4M_07I06_R_0	BY934121
E_EL_vg4M_07I07_F_0	BY925382
E_EL_vg4M_07I07_R_0	BY934122
E_EL_vg4M_07I09_F_0	BY925383
E_EL_vg4M_07I09_R_0	BY934123
E_EL_vg4M_07I10_F_0	BY925384
E_EL_vg4M_07I10_R_0	BY934124
E_EL_vg4M_07I11_F_0	BY925385
E_EL_vg4M_07I11_R_0	BY934125
E_EL_vg4M_07I12_R_0	BY934126
E_EL_vg4M_07I14_R_0	BY934127
E_EL_vg4M_07I15_F_0	BY925386
E_EL_vg4M_07I15_R_0	BY934128
E_EL_vg4M_07I16_F_0	BY925387
E_EL_vg4M_07I16_R_0	BY934129
E_EL_vg4M_07I17_F_0	BY925388
E_EL_vg4M_07I17_R_0	BY934130
E_EL_vg4M_07I18_F_0	BY925389
E_EL_vg4M_07I18_R_0	BY934131
E_EL_vg4M_07I19_F_0	BY925390
E_EL_vg4M_07I20_F_0	BY925391
E_EL_vg4M_07I20_R_0	BY934132
E_EL_vg4M_07I21_F_0	BY925392
E_EL_vg4M_07I21_R_0	BY934133
E_EL_vg4M_07I22_R_0	BY934134
E_EL_vg4M_07I23_F_0	BY925393
E_EL_vg4M_07I23_R_0	BY934135
E_EL_vg4M_07I24_F_0	BY925394
E_EL_vg4M_07I24_R_0	BY934136
E_EL_vg4M_07J01_F_0	BY925395
E_EL_vg4M_07J01_R_0	BY934137
E_EL_vg4M_07J04_F_0	BY925396

E_EL_vg4M_07J04_R_0	BY934138
E_EL_vg4M_07J05_R_0	BY934139
E_EL_vg4M_07J06_F_0	BY925397
E_EL_vg4M_07J06_R_0	BY934140
E_EL_vg4M_07J07_F_0	BY925398
E_EL_vg4M_07J07_R_0	BY934141
E_EL_vg4M_07J08_F_0	BY925399
E_EL_vg4M_07J08_R_0	BY934142
E_EL_vg4M_07J09_F_0	BY925400
E_EL_vg4M_07J09_R_0	BY934143
E_EL_vg4M_07J10_F_0	BY925401
E_EL_vg4M_07J10_R_0	BY934144
E_EL_vg4M_07J11_F_0	BY925402
E_EL_vg4M_07J11_R_0	BY934145
E_EL_vg4M_07J12_R_0	BY934146
E_EL_vg4M_07J13_F_0	BY925403
E_EL_vg4M_07J13_R_0	BY934147
E_EL_vg4M_07J14_F_0	BY925404
E_EL_vg4M_07J14_R_0	BY934148
E_EL_vg4M_07J15_F_0	BY925405
E_EL_vg4M_07J15_R_0	BY934149
E_EL_vg4M_07J16_F_0	BY925406
E_EL_vg4M_07J16_R_0	BY934150
E_EL_vg4M_07J17_F_0	BY925407
E_EL_vg4M_07J17_R_0	BY934151
E_EL_vg4M_07J19_R_0	BY934152
E_EL_vg4M_07J20_F_0	BY925408
E_EL_vg4M_07J20_R_0	BY934153
E_EL_vg4M_07J21_R_0	BY934154
E_EL_vg4M_07J22_F_0	BY925409
E_EL_vg4M_07J23_F_0	BY925410
E_EL_vg4M_07J23_R_0	BY934155
E_EL_vg4M_07J24_F_0	BY925411
E_EL_vg4M_07J24_R_0	BY934156
E_EL_vg4M_07K01_F_0	BY925412
E_EL_vg4M_07K01_R_0	BY934157
E_EL_vg4M_07K02_F_0	BY925413
E_EL_vg4M_07K02_R_0	BY934158
E_EL_vg4M_07K03_F_0	BY925414
E_EL_vg4M_07K03_R_0	BY934159
E_EL_vg4M_07K04_F_0	BY925415
E_EL_vg4M_07K04_R_0	BY934160
E_EL_vg4M_07K05_F_0	BY925416
E_EL_vg4M_07K05_R_0	BY934161
E_EL_vg4M_07K06_F_0	BY925417
E_EL_vg4M_07K06_R_0	BY934162
E_EL_vg4M_07K07_F_0	BY925418
E_EL_vg4M_07K07_R_0	BY934163
E_EL_vg4M_07K08_F_0	BY925419
E_EL_vg4M_07K08_R_0	BY934164
E_EL_vg4M_07K09_F_0	BY925420
E_EL_vg4M_07K09_R_0	BY934165
E_EL_vg4M_07K10_F_0	BY925421
E_EL_vg4M_07K10_R_0	BY934166

E_EL_vg4M_07K11_F_0	BY925422
E_EL_vg4M_07K11_R_0	BY934167
E_EL_vg4M_07K12_F_0	BY925423
E_EL_vg4M_07K12_R_0	BY934168
E_EL_vg4M_07K13_F_0	BY925424
E_EL_vg4M_07K13_R_0	BY934169
E_EL_vg4M_07K14_F_0	BY925425
E_EL_vg4M_07K14_R_0	BY934170
E_EL_vg4M_07K15_F_0	BY925426
E_EL_vg4M_07K15_R_0	BY934171
E_EL_vg4M_07K16_F_0	BY925427
E_EL_vg4M_07K16_R_0	BY934172
E_EL_vg4M_07K17_R_0	BY934173
E_EL_vg4M_07K18_R_0	BY934174
E_EL_vg4M_07K19_F_0	BY925428
E_EL_vg4M_07K19_R_0	BY934175
E_EL_vg4M_07K20_F_0	BY925429
E_EL_vg4M_07K20_R_0	BY934176
E_EL_vg4M_07K21_F_0	BY925430
E_EL_vg4M_07K21_R_0	BY934177
E_EL_vg4M_07K22_F_0	BY925431
E_EL_vg4M_07K22_R_0	BY934178
E_EL_vg4M_07K23_F_0	BY925432
E_EL_vg4M_07K24_F_0	BY925433
E_EL_vg4M_07K24_R_0	BY934179
E_EL_vg4M_07L02_F_0	BY925434
E_EL_vg4M_07L02_R_0	BY934180
E_EL_vg4M_07L03_F_0	BY925435
E_EL_vg4M_07L05_F_0	BY925436
E_EL_vg4M_07L05_R_0	BY934181
E_EL_vg4M_07L07_F_0	BY925437
E_EL_vg4M_07L07_R_0	BY934182
E_EL_vg4M_07L09_R_0	BY934183
E_EL_vg4M_07L10_F_0	BY925438
E_EL_vg4M_07L11_F_0	BY925439
E_EL_vg4M_07L13_R_0	BY934184
E_EL_vg4M_07L14_F_0	BY925440
E_EL_vg4M_07L14_R_0	BY934185
E_EL_vg4M_07L15_F_0	BY925441
E_EL_vg4M_07L15_R_0	BY934186
E_EL_vg4M_07L16_F_0	BY925442
E_EL_vg4M_07L16_R_0	BY934187
E_EL_vg4M_07L18_R_0	BY934188
E_EL_vg4M_07L21_F_0	BY925443
E_EL_vg4M_07L21_R_0	BY934189
E_EL_vg4M_07L23_R_0	BY934190
E_EL_vg4M_07L24_F_0	BY925444
E_EL_vg4M_07L24_R_0	BY934191
E_EL_vg4M_07M01_F_0	BY925445
E_EL_vg4M_07M01_R_0	BY934192
E_EL_vg4M_07M03_F_0	BY925446
E_EL_vg4M_07M03_R_0	BY934193
E_EL_vg4M_07M04_F_0	BY925447
E_EL_vg4M_07M04_R_0	BY934194

E_EL_vg4M_07M05_F_0	BY925448
E_EL_vg4M_07M05_R_0	BY934195
E_EL_vg4M_07M07_F_0	BY925449
E_EL_vg4M_07M07_R_0	BY934196
E_EL_vg4M_07M08_R_0	BY934197
E_EL_vg4M_07M09_F_0	BY925450
E_EL_vg4M_07M09_R_0	BY934198
E_EL_vg4M_07M10_F_0	BY925451
E_EL_vg4M_07M10_R_0	BY934199
E_EL_vg4M_07M12_F_0	BY925452
E_EL_vg4M_07M12_R_0	BY934200
E_EL_vg4M_07M13_R_0	BY934201
E_EL_vg4M_07M15_F_0	BY925453
E_EL_vg4M_07M15_R_0	BY934202
E_EL_vg4M_07M16_F_0	BY925454
E_EL_vg4M_07M16_R_0	BY934203
E_EL_vg4M_07M18_F_0	BY925455
E_EL_vg4M_07M18_R_0	BY934204
E_EL_vg4M_07M19_F_0	BY925456
E_EL_vg4M_07M20_F_0	BY925457
E_EL_vg4M_07M20_R_0	BY934205
E_EL_vg4M_07M21_F_0	BY925458
E_EL_vg4M_07M21_R_0	BY934206
E_EL_vg4M_07M22_F_0	BY925459
E_EL_vg4M_07M22_R_0	BY934207
E_EL_vg4M_07M23_F_0	BY925460
E_EL_vg4M_07M23_R_0	BY934208
E_EL_vg4M_07M24_F_0	BY925461
E_EL_vg4M_07M24_R_0	BY934209
E_EL_vg4M_07N01_F_0	BY925462
E_EL_vg4M_07N01_R_0	BY934210
E_EL_vg4M_07N02_F_0	BY925463
E_EL_vg4M_07N03_F_0	BY925464
E_EL_vg4M_07N03_R_0	BY934211
E_EL_vg4M_07N04_F_0	BY925465
E_EL_vg4M_07N05_R_0	BY934212
E_EL_vg4M_07N09_F_0	BY925466
E_EL_vg4M_07N09_R_0	BY934213
E_EL_vg4M_07N10_F_0	BY925467
E_EL_vg4M_07N10_R_0	BY934214
E_EL_vg4M_07N11_F_0	BY925468
E_EL_vg4M_07N11_R_0	BY934215
E_EL_vg4M_07N12_F_0	BY925469
E_EL_vg4M_07N13_F_0	BY925470
E_EL_vg4M_07N13_R_0	BY934216
E_EL_vg4M_07N14_F_0	BY925471
E_EL_vg4M_07N14_R_0	BY934217
E_EL_vg4M_07N15_F_0	BY925472
E_EL_vg4M_07N15_R_0	BY934218
E_EL_vg4M_07N16_F_0	BY925473
E_EL_vg4M_07N16_R_0	BY934219
E_EL_vg4M_07N17_F_0	BY925474
E_EL_vg4M_07N17_R_0	BY934220
E_EL_vg4M_07N18_F_0	BY925475

E_EL_vg4M_07N19_F_0	BY925476
E_EL_vg4M_07N19_R_0	BY934221
E_EL_vg4M_07N20_F_0	BY925477
E_EL_vg4M_07N20_R_0	BY934222
E_EL_vg4M_07N21_F_0	BY925478
E_EL_vg4M_07N21_R_0	BY934223
E_EL_vg4M_07N22_F_0	BY925479
E_EL_vg4M_07N22_R_0	BY934224
E_EL_vg4M_07N23_F_0	BY925480
E_EL_vg4M_07N23_R_0	BY934225
E_EL_vg4M_07N24_F_0	BY925481
E_EL_vg4M_07N24_R_0	BY934226
E_EL_vg4M_07O01_F_0	BY925482
E_EL_vg4M_07O01_R_0	BY934227
E_EL_vg4M_07O03_F_0	BY925483
E_EL_vg4M_07O03_R_0	BY934228
E_EL_vg4M_07O04_F_0	BY925484
E_EL_vg4M_07O04_R_0	BY934229
E_EL_vg4M_07O05_F_0	BY925485
E_EL_vg4M_07O05_R_0	BY934230
E_EL_vg4M_07O07_F_0	BY925486
E_EL_vg4M_07O07_R_0	BY934231
E_EL_vg4M_07O08_F_0	BY925487
E_EL_vg4M_07O08_R_0	BY934232
E_EL_vg4M_07O09_F_0	BY925488
E_EL_vg4M_07O09_R_0	BY934233
E_EL_vg4M_07O10_R_0	BY934234
E_EL_vg4M_07O11_R_0	BY934235
E_EL_vg4M_07O12_F_0	BY925489
E_EL_vg4M_07O13_F_0	BY925490
E_EL_vg4M_07O13_R_0	BY934236
E_EL_vg4M_07O14_F_0	BY925491
E_EL_vg4M_07O14_R_0	BY934237
E_EL_vg4M_07O15_F_0	BY925492
E_EL_vg4M_07O15_R_0	BY934238
E_EL_vg4M_07O16_F_0	BY925493
E_EL_vg4M_07O16_R_0	BY934239
E_EL_vg4M_07O17_F_0	BY925494
E_EL_vg4M_07O17_R_0	BY934240
E_EL_vg4M_07O18_F_0	BY925495
E_EL_vg4M_07O19_F_0	BY925496
E_EL_vg4M_07O19_R_0	BY934241
E_EL_vg4M_07O20_R_0	BY934242
E_EL_vg4M_07O21_F_0	BY925497
E_EL_vg4M_07O21_R_0	BY934243
E_EL_vg4M_07O22_R_0	BY934244
E_EL_vg4M_07O23_F_0	BY925498
E_EL_vg4M_07O23_R_0	BY934245
E_EL_vg4M_07O24_F_0	BY925499
E_EL_vg4M_07O24_R_0	BY934246
E_EL_vg4M_07P01_F_0	BY925500
E_EL_vg4M_07P01_R_0	BY934247
E_EL_vg4M_07P02_F_0	BY925501
E_EL_vg4M_07P02_R_0	BY934248

E_EL_vg4M_07P03_F_0	BY925502
E_EL_vg4M_07P03_R_0	BY934249
E_EL_vg4M_07P05_F_0	BY925503
E_EL_vg4M_07P05_R_0	BY934250
E_EL_vg4M_07P06_F_0	BY925504
E_EL_vg4M_07P06_R_0	BY934251
E_EL_vg4M_07P07_F_0	BY925505
E_EL_vg4M_07P07_R_0	BY934252
E_EL_vg4M_07P09_R_0	BY934253
E_EL_vg4M_07P10_F_0	BY925506
E_EL_vg4M_07P10_R_0	BY934254
E_EL_vg4M_07P11_F_0	BY925507
E_EL_vg4M_07P11_R_0	BY934255
E_EL_vg4M_07P12_F_0	BY925508
E_EL_vg4M_07P13_F_0	BY925509
E_EL_vg4M_07P13_R_0	BY934256
E_EL_vg4M_07P14_F_0	BY925510
E_EL_vg4M_07P15_F_0	BY925511
E_EL_vg4M_07P15_R_0	BY934257
E_EL_vg4M_07P16_F_0	BY925512
E_EL_vg4M_07P16_R_0	BY934258
E_EL_vg4M_07P17_F_0	BY925513
E_EL_vg4M_07P17_R_0	BY934259
E_EL_vg4M_07P21_F_0	BY925514
E_EL_vg4M_07P21_R_0	BY934260
E_EL_vg4M_07P22_F_0	BY925515
E_EL_vg4M_07P22_R_0	BY934261
E_EL_vg4M_07P23_F_0	BY925516
E_EL_vg4M_07P23_R_0	BY934262
E_EL_vg4M_07P24_F_0	BY925517
E_EL_vg4M_07P24_R_0	BY934263
E_EL_vg4M_08A02_R_0	BY934264
E_EL_vg4M_08A03_F_0	BY925518
E_EL_vg4M_08A03_R_0	BY934265
E_EL_vg4M_08A04_F_0	BY925519
E_EL_vg4M_08A04_R_0	BY934266
E_EL_vg4M_08A05_R_0	BY934267
E_EL_vg4M_08A06_F_0	BY925520
E_EL_vg4M_08A06_R_0	BY934268
E_EL_vg4M_08A07_F_0	BY925521
E_EL_vg4M_08A07_R_0	BY934269
E_EL_vg4M_08A08_F_0	BY925522
E_EL_vg4M_08A08_R_0	BY934270
E_EL_vg4M_08A09_F_0	BY925523
E_EL_vg4M_08A09_R_0	BY934271
E_EL_vg4M_08A10_F_0	BY925524
E_EL_vg4M_08A10_R_0	BY934272
E_EL_vg4M_08A11_F_0	BY925525
E_EL_vg4M_08A11_R_0	BY934273
E_EL_vg4M_08A12_F_0	BY925526
E_EL_vg4M_08A12_R_0	BY934274
E_EL_vg4M_08A14_R_0	BY934275
E_EL_vg4M_08A15_R_0	BY934276
E_EL_vg4M_08A16_R_0	BY934277

E_EL_vg4M_08A17_R_0	BY934278
E_EL_vg4M_08A19_F_0	BY925527
E_EL_vg4M_08A19_R_0	BY934279
E_EL_vg4M_08A20_F_0	BY925528
E_EL_vg4M_08A21_F_0	BY925529
E_EL_vg4M_08A21_R_0	BY934280
E_EL_vg4M_08A22_F_0	BY925530
E_EL_vg4M_08A24_F_0	BY925531
E_EL_vg4M_08A24_R_0	BY934281
E_EL_vg4M_08B01_R_0	BY934282
E_EL_vg4M_08B02_R_0	BY934283
E_EL_vg4M_08B03_F_0	BY925532
E_EL_vg4M_08B03_R_0	BY934284
E_EL_vg4M_08B04_F_0	BY925533
E_EL_vg4M_08B04_R_0	BY934285
E_EL_vg4M_08B05_F_0	BY925534
E_EL_vg4M_08B05_R_0	BY934286
E_EL_vg4M_08B06_F_0	BY925535
E_EL_vg4M_08B06_R_0	BY934287
E_EL_vg4M_08B07_F_0	BY925536
E_EL_vg4M_08B07_R_0	BY934288
E_EL_vg4M_08B08_F_0	BY925537
E_EL_vg4M_08B08_R_0	BY934289
E_EL_vg4M_08B09_F_0	BY925538
E_EL_vg4M_08B09_R_0	BY934290
E_EL_vg4M_08B10_F_0	BY925539
E_EL_vg4M_08B10_R_0	BY934291
E_EL_vg4M_08B11_F_0	BY925540
E_EL_vg4M_08B11_R_0	BY934292
E_EL_vg4M_08B12_F_0	BY925541
E_EL_vg4M_08B12_R_0	BY934293
E_EL_vg4M_08B13_F_0	BY925542
E_EL_vg4M_08B13_R_0	BY934294
E_EL_vg4M_08B14_F_0	BY925543
E_EL_vg4M_08B14_R_0	BY934295
E_EL_vg4M_08B15_R_0	BY934296
E_EL_vg4M_08B16_R_0	BY934297
E_EL_vg4M_08B17_F_0	BY925544
E_EL_vg4M_08B17_R_0	BY934298
E_EL_vg4M_08B18_F_0	BY925545
E_EL_vg4M_08B18_R_0	BY934299
E_EL_vg4M_08B19_R_0	BY934300
E_EL_vg4M_08B21_F_0	BY925546
E_EL_vg4M_08B21_R_0	BY934301
E_EL_vg4M_08B22_F_0	BY925547
E_EL_vg4M_08B22_R_0	BY934302
E_EL_vg4M_08B23_F_0	BY925548
E_EL_vg4M_08B23_R_0	BY934303
E_EL_vg4M_08B24_F_0	BY925549
E_EL_vg4M_08B24_R_0	BY934304
E_EL_vg4M_08C01_R_0	BY934305
E_EL_vg4M_08C02_F_0	BY925550
E_EL_vg4M_08C02_R_0	BY934306
E_EL_vg4M_08C03_F_0	BY925551

E_EL_vg4M_08C03_R_0	BY934307
E_EL_vg4M_08C04_F_0	BY925552
E_EL_vg4M_08C04_R_0	BY934308
E_EL_vg4M_08C05_F_0	BY925553
E_EL_vg4M_08C05_R_0	BY934309
E_EL_vg4M_08C06_F_0	BY925554
E_EL_vg4M_08C06_R_0	BY934310
E_EL_vg4M_08C07_F_0	BY925555
E_EL_vg4M_08C07_R_0	BY934311
E_EL_vg4M_08C08_F_0	BY925556
E_EL_vg4M_08C08_R_0	BY934312
E_EL_vg4M_08C09_R_0	BY934313
E_EL_vg4M_08C10_F_0	BY925557
E_EL_vg4M_08C10_R_0	BY934314
E_EL_vg4M_08C11_R_0	BY934315
E_EL_vg4M_08C12_F_0	BY925558
E_EL_vg4M_08C12_R_0	BY934316
E_EL_vg4M_08C13_R_0	BY934317
E_EL_vg4M_08C14_F_0	BY925559
E_EL_vg4M_08C14_R_0	BY934318
E_EL_vg4M_08C15_F_0	BY925560
E_EL_vg4M_08C15_R_0	BY934319
E_EL_vg4M_08C16_F_0	BY925561
E_EL_vg4M_08C16_R_0	BY934320
E_EL_vg4M_08C17_F_0	BY925562
E_EL_vg4M_08C17_R_0	BY934321
E_EL_vg4M_08C18_R_0	BY934322
E_EL_vg4M_08C19_R_0	BY934323
E_EL_vg4M_08C20_F_0	BY925563
E_EL_vg4M_08C20_R_0	BY934324
E_EL_vg4M_08C21_R_0	BY934325
E_EL_vg4M_08C22_R_0	BY934326
E_EL_vg4M_08C23_F_0	BY925564
E_EL_vg4M_08C23_R_0	BY934327
E_EL_vg4M_08C24_R_0	BY934328
E_EL_vg4M_08D01_R_0	BY934329
E_EL_vg4M_08D02_F_0	BY925565
E_EL_vg4M_08D02_R_0	BY934330
E_EL_vg4M_08D03_F_0	BY925566
E_EL_vg4M_08D04_F_0	BY925567
E_EL_vg4M_08D04_R_0	BY934331
E_EL_vg4M_08D05_F_0	BY925568
E_EL_vg4M_08D05_R_0	BY934332
E_EL_vg4M_08D06_R_0	BY934333
E_EL_vg4M_08D07_F_0	BY925569
E_EL_vg4M_08D07_R_0	BY934334
E_EL_vg4M_08D08_F_0	BY925570
E_EL_vg4M_08D08_R_0	BY934335
E_EL_vg4M_08D09_F_0	BY925571
E_EL_vg4M_08D09_R_0	BY934336
E_EL_vg4M_08D10_F_0	BY925572
E_EL_vg4M_08D10_R_0	BY934337
E_EL_vg4M_08D11_F_0	BY925573
E_EL_vg4M_08D11_R_0	BY934338

E_EL_vg4M_08D12_F_0	BY925574
E_EL_vg4M_08D13_R_0	BY934339
E_EL_vg4M_08D14_F_0	BY925575
E_EL_vg4M_08D14_R_0	BY934340
E_EL_vg4M_08D15_F_0	BY925576
E_EL_vg4M_08D15_R_0	BY934341
E_EL_vg4M_08D16_F_0	BY925577
E_EL_vg4M_08D16_R_0	BY934342
E_EL_vg4M_08D17_F_0	BY925578
E_EL_vg4M_08D17_R_0	BY934343
E_EL_vg4M_08D18_F_0	BY925579
E_EL_vg4M_08D18_R_0	BY934344
E_EL_vg4M_08D19_R_0	BY934345
E_EL_vg4M_08D20_R_0	BY934346
E_EL_vg4M_08D21_F_0	BY925580
E_EL_vg4M_08D21_R_0	BY934347
E_EL_vg4M_08D22_R_0	BY934348
E_EL_vg4M_08D23_R_0	BY934349
E_EL_vg4M_08D24_R_0	BY934350
E_EL_vg4M_08E01_R_0	BY934351
E_EL_vg4M_08E02_R_0	BY934352
E_EL_vg4M_08E03_F_0	BY925581
E_EL_vg4M_08E03_R_0	BY934353
E_EL_vg4M_08E04_F_0	BY925582
E_EL_vg4M_08E04_R_0	BY934354
E_EL_vg4M_08E05_F_0	BY925583
E_EL_vg4M_08E05_R_0	BY934355
E_EL_vg4M_08E06_F_0	BY925584
E_EL_vg4M_08E06_R_0	BY934356
E_EL_vg4M_08E07_R_0	BY934357
E_EL_vg4M_08E08_R_0	BY934358
E_EL_vg4M_08E09_F_0	BY925585
E_EL_vg4M_08E09_R_0	BY934359
E_EL_vg4M_08E10_F_0	BY925586
E_EL_vg4M_08E10_R_0	BY934360
E_EL_vg4M_08E11_F_0	BY925587
E_EL_vg4M_08E11_R_0	BY934361
E_EL_vg4M_08E12_R_0	BY934362
E_EL_vg4M_08E14_F_0	BY925588
E_EL_vg4M_08E14_R_0	BY934363
E_EL_vg4M_08E15_F_0	BY925589
E_EL_vg4M_08E17_F_0	BY925590
E_EL_vg4M_08E17_R_0	BY934364
E_EL_vg4M_08E18_F_0	BY925591
E_EL_vg4M_08E18_R_0	BY934365
E_EL_vg4M_08E19_R_0	BY934366
E_EL_vg4M_08E20_R_0	BY934367
E_EL_vg4M_08E21_R_0	BY934368
E_EL_vg4M_08E22_F_0	BY925592
E_EL_vg4M_08E22_R_0	BY934369
E_EL_vg4M_08E23_F_0	BY925593
E_EL_vg4M_08E23_R_0	BY934370
E_EL_vg4M_08E24_F_0	BY925594
E_EL_vg4M_08E24_R_0	BY934371

E_EL_vg4M_08F01_F_0	BY925595
E_EL_vg4M_08F01_R_0	BY934372
E_EL_vg4M_08F02_F_0	BY925596
E_EL_vg4M_08F02_R_0	BY934373
E_EL_vg4M_08F03_R_0	BY934374
E_EL_vg4M_08F04_F_0	BY925597
E_EL_vg4M_08F04_R_0	BY934375
E_EL_vg4M_08F05_F_0	BY925598
E_EL_vg4M_08F05_R_0	BY934376
E_EL_vg4M_08F06_F_0	BY925599
E_EL_vg4M_08F06_R_0	BY934377
E_EL_vg4M_08F07_F_0	BY925600
E_EL_vg4M_08F08_F_0	BY925601
E_EL_vg4M_08F08_R_0	BY934378
E_EL_vg4M_08F10_F_0	BY925602
E_EL_vg4M_08F10_R_0	BY934379
E_EL_vg4M_08F11_F_0	BY925603
E_EL_vg4M_08F11_R_0	BY934380
E_EL_vg4M_08F13_R_0	BY934381
E_EL_vg4M_08F14_F_0	BY925604
E_EL_vg4M_08F14_R_0	BY934382
E_EL_vg4M_08F15_F_0	BY925605
E_EL_vg4M_08F15_R_0	BY934383
E_EL_vg4M_08F16_F_0	BY925606
E_EL_vg4M_08F16_R_0	BY934384
E_EL_vg4M_08F17_F_0	BY925607
E_EL_vg4M_08F17_R_0	BY934385
E_EL_vg4M_08F18_R_0	BY934386
E_EL_vg4M_08F19_F_0	BY925608
E_EL_vg4M_08F19_R_0	BY934387
E_EL_vg4M_08F21_F_0	BY925609
E_EL_vg4M_08F21_R_0	BY934388
E_EL_vg4M_08F22_F_0	BY925610
E_EL_vg4M_08F22_R_0	BY934389
E_EL_vg4M_08F23_F_0	BY925611
E_EL_vg4M_08F23_R_0	BY934390
E_EL_vg4M_08F24_F_0	BY925612
E_EL_vg4M_08F24_R_0	BY934391
E_EL_vg4M_08G01_R_0	BY934392
E_EL_vg4M_08G02_F_0	BY925613
E_EL_vg4M_08G02_R_0	BY934393
E_EL_vg4M_08G03_F_0	BY925614
E_EL_vg4M_08G03_R_0	BY934394
E_EL_vg4M_08G05_F_0	BY925615
E_EL_vg4M_08G05_R_0	BY934395
E_EL_vg4M_08G06_F_0	BY925616
E_EL_vg4M_08G06_R_0	BY934396
E_EL_vg4M_08G07_R_0	BY934397
E_EL_vg4M_08G08_F_0	BY925617
E_EL_vg4M_08G08_R_0	BY934398
E_EL_vg4M_08G09_F_0	BY925618
E_EL_vg4M_08G09_R_0	BY934399
E_EL_vg4M_08G10_F_0	BY925619
E_EL_vg4M_08G10_R_0	BY934400

E_EL_vg4M_08G11_F_0	BY925620
E_EL_vg4M_08G11_R_0	BY934401
E_EL_vg4M_08G12_F_0	BY925621
E_EL_vg4M_08G12_R_0	BY934402
E_EL_vg4M_08G13_F_0	BY925622
E_EL_vg4M_08G13_R_0	BY934403
E_EL_vg4M_08G14_F_0	BY925623
E_EL_vg4M_08G14_R_0	BY934404
E_EL_vg4M_08G15_F_0	BY925624
E_EL_vg4M_08G15_R_0	BY934405
E_EL_vg4M_08G16_F_0	BY925625
E_EL_vg4M_08G16_R_0	BY934406
E_EL_vg4M_08G17_R_0	BY934407
E_EL_vg4M_08G21_F_0	BY925626
E_EL_vg4M_08G21_R_0	BY934408
E_EL_vg4M_08G22_F_0	BY925627
E_EL_vg4M_08G22_R_0	BY934409
E_EL_vg4M_08G23_F_0	BY925628
E_EL_vg4M_08G23_R_0	BY934410
E_EL_vg4M_08G24_F_0	BY925629
E_EL_vg4M_08G24_R_0	BY934411
E_EL_vg4M_08H01_R_0	BY934412
E_EL_vg4M_08H02_F_0	BY925630
E_EL_vg4M_08H02_R_0	BY934413
E_EL_vg4M_08H03_F_0	BY925631
E_EL_vg4M_08H03_R_0	BY934414
E_EL_vg4M_08H05_F_0	BY925632
E_EL_vg4M_08H05_R_0	BY934415
E_EL_vg4M_08H06_F_0	BY925633
E_EL_vg4M_08H06_R_0	BY934416
E_EL_vg4M_08H07_F_0	BY925634
E_EL_vg4M_08H07_R_0	BY934417
E_EL_vg4M_08H08_F_0	BY925635
E_EL_vg4M_08H08_R_0	BY934418
E_EL_vg4M_08H09_F_0	BY925636
E_EL_vg4M_08H09_R_0	BY934419
E_EL_vg4M_08H10_F_0	BY925637
E_EL_vg4M_08H10_R_0	BY934420
E_EL_vg4M_08H11_F_0	BY925638
E_EL_vg4M_08H11_R_0	BY934421
E_EL_vg4M_08H12_F_0	BY925639
E_EL_vg4M_08H12_R_0	BY934422
E_EL_vg4M_08H13_F_0	BY925640
E_EL_vg4M_08H13_R_0	BY934423
E_EL_vg4M_08H14_F_0	BY925641
E_EL_vg4M_08H14_R_0	BY934424
E_EL_vg4M_08H15_F_0	BY925642
E_EL_vg4M_08H15_R_0	BY934425
E_EL_vg4M_08H16_F_0	BY925643
E_EL_vg4M_08H16_R_0	BY934426
E_EL_vg4M_08H17_F_0	BY925644
E_EL_vg4M_08H17_R_0	BY934427
E_EL_vg4M_08H18_F_0	BY925645
E_EL_vg4M_08H18_R_0	BY934428

E_EL_vg4M_08H19_F_0	BY925646
E_EL_vg4M_08H19_R_0	BY934429
E_EL_vg4M_08H21_F_0	BY925647
E_EL_vg4M_08H21_R_0	BY934430
E_EL_vg4M_08H22_F_0	BY925648
E_EL_vg4M_08H22_R_0	BY934431
E_EL_vg4M_08H23_R_0	BY934432
E_EL_vg4M_08H24_F_0	BY925649
E_EL_vg4M_08H24_R_0	BY934433
E_EL_vg4M_08I01_F_0	BY925650
E_EL_vg4M_08I01_R_0	BY934434
E_EL_vg4M_08I02_F_0	BY925651
E_EL_vg4M_08I02_R_0	BY934435
E_EL_vg4M_08I03_F_0	BY925652
E_EL_vg4M_08I03_R_0	BY934436
E_EL_vg4M_08I04_F_0	BY925653
E_EL_vg4M_08I04_R_0	BY934437
E_EL_vg4M_08I05_F_0	BY925654
E_EL_vg4M_08I05_R_0	BY934438
E_EL_vg4M_08I06_F_0	BY925655
E_EL_vg4M_08I06_R_0	BY934439
E_EL_vg4M_08I07_F_0	BY925656
E_EL_vg4M_08I08_F_0	BY925657
E_EL_vg4M_08I08_R_0	BY934440
E_EL_vg4M_08I09_F_0	BY925658
E_EL_vg4M_08I09_R_0	BY934441
E_EL_vg4M_08I10_F_0	BY925659
E_EL_vg4M_08I10_R_0	BY934442
E_EL_vg4M_08I11_F_0	BY925660
E_EL_vg4M_08I11_R_0	BY934443
E_EL_vg4M_08I12_R_0	BY934444
E_EL_vg4M_08I13_F_0	BY925661
E_EL_vg4M_08I13_R_0	BY934445
E_EL_vg4M_08I14_F_0	BY925662
E_EL_vg4M_08I14_R_0	BY934446
E_EL_vg4M_08I15_R_0	BY934447
E_EL_vg4M_08I16_R_0	BY934448
E_EL_vg4M_08I17_F_0	BY925663
E_EL_vg4M_08I17_R_0	BY934449
E_EL_vg4M_08I18_F_0	BY925664
E_EL_vg4M_08I18_R_0	BY934450
E_EL_vg4M_08I20_F_0	BY925665
E_EL_vg4M_08I20_R_0	BY934451
E_EL_vg4M_08I21_F_0	BY925666
E_EL_vg4M_08I21_R_0	BY934452
E_EL_vg4M_08I22_F_0	BY925667
E_EL_vg4M_08I22_R_0	BY934453
E_EL_vg4M_08I24_R_0	BY934454
E_EL_vg4M_08J01_F_0	BY925668
E_EL_vg4M_08J01_R_0	BY934455
E_EL_vg4M_08J02_F_0	BY925669
E_EL_vg4M_08J02_R_0	BY934456
E_EL_vg4M_08J03_F_0	BY925670
E_EL_vg4M_08J03_R_0	BY934457

E_EL_vg4M_08J04_F_0	BY925671
E_EL_vg4M_08J04_R_0	BY934458
E_EL_vg4M_08J05_F_0	BY925672
E_EL_vg4M_08J05_R_0	BY934459
E_EL_vg4M_08J06_F_0	BY925673
E_EL_vg4M_08J06_R_0	BY934460
E_EL_vg4M_08J07_R_0	BY934461
E_EL_vg4M_08J08_R_0	BY934462
E_EL_vg4M_08J09_R_0	BY934463
E_EL_vg4M_08J10_F_0	BY925674
E_EL_vg4M_08J10_R_0	BY934464
E_EL_vg4M_08J11_F_0	BY925675
E_EL_vg4M_08J11_R_0	BY934465
E_EL_vg4M_08J12_R_0	BY934466
E_EL_vg4M_08J13_F_0	BY925676
E_EL_vg4M_08J13_R_0	BY934467
E_EL_vg4M_08J14_F_0	BY925677
E_EL_vg4M_08J14_R_0	BY934468
E_EL_vg4M_08J15_F_0	BY925678
E_EL_vg4M_08J15_R_0	BY934469
E_EL_vg4M_08J16_F_0	BY925679
E_EL_vg4M_08J16_R_0	BY934470
E_EL_vg4M_08J17_F_0	BY925680
E_EL_vg4M_08J17_R_0	BY934471
E_EL_vg4M_08J18_F_0	BY925681
E_EL_vg4M_08J18_R_0	BY934472
E_EL_vg4M_08J19_F_0	BY925682
E_EL_vg4M_08J19_R_0	BY934473
E_EL_vg4M_08J21_F_0	BY925683
E_EL_vg4M_08J21_R_0	BY934474
E_EL_vg4M_08J22_F_0	BY925684
E_EL_vg4M_08J22_R_0	BY934475
E_EL_vg4M_08J23_R_0	BY934476
E_EL_vg4M_08J24_F_0	BY925685
E_EL_vg4M_08J24_R_0	BY934477
E_EL_vg4M_08K01_F_0	BY925686
E_EL_vg4M_08K01_R_0	BY934478
E_EL_vg4M_08K02_F_0	BY925687
E_EL_vg4M_08K02_R_0	BY934479
E_EL_vg4M_08K03_F_0	BY925688
E_EL_vg4M_08K03_R_0	BY934480
E_EL_vg4M_08K04_R_0	BY934481
E_EL_vg4M_08K05_F_0	BY925689
E_EL_vg4M_08K05_R_0	BY934482
E_EL_vg4M_08K07_F_0	BY925690
E_EL_vg4M_08K07_R_0	BY934483
E_EL_vg4M_08K08_F_0	BY925691
E_EL_vg4M_08K08_R_0	BY934484
E_EL_vg4M_08K10_F_0	BY925692
E_EL_vg4M_08K10_R_0	BY934485
E_EL_vg4M_08K11_R_0	BY934486
E_EL_vg4M_08K12_R_0	BY934487
E_EL_vg4M_08K13_F_0	BY925693
E_EL_vg4M_08K13_R_0	BY934488

E_EL_vg4M_08K14_F_0	BY925694
E_EL_vg4M_08K14_R_0	BY934489
E_EL_vg4M_08K15_R_0	BY934490
E_EL_vg4M_08K16_F_0	BY925695
E_EL_vg4M_08K16_R_0	BY934491
E_EL_vg4M_08K17_F_0	BY925696
E_EL_vg4M_08K17_R_0	BY934492
E_EL_vg4M_08K18_F_0	BY925697
E_EL_vg4M_08K18_R_0	BY934493
E_EL_vg4M_08K19_F_0	BY925698
E_EL_vg4M_08K19_R_0	BY934494
E_EL_vg4M_08K20_F_0	BY925699
E_EL_vg4M_08K20_R_0	BY934495
E_EL_vg4M_08K21_F_0	BY925700
E_EL_vg4M_08K21_R_0	BY934496
E_EL_vg4M_08K22_R_0	BY934497
E_EL_vg4M_08K23_R_0	BY934498
E_EL_vg4M_08K24_R_0	BY934499
E_EL_vg4M_08L01_F_0	BY925701
E_EL_vg4M_08L01_R_0	BY934500
E_EL_vg4M_08L02_F_0	BY925702
E_EL_vg4M_08L02_R_0	BY934501
E_EL_vg4M_08L03_F_0	BY925703
E_EL_vg4M_08L03_R_0	BY934502
E_EL_vg4M_08L04_R_0	BY934503
E_EL_vg4M_08L05_F_0	BY925704
E_EL_vg4M_08L05_R_0	BY934504
E_EL_vg4M_08L07_F_0	BY925705
E_EL_vg4M_08L07_R_0	BY934505
E_EL_vg4M_08L08_F_0	BY925706
E_EL_vg4M_08L08_R_0	BY934506
E_EL_vg4M_08L10_F_0	BY925707
E_EL_vg4M_08L11_F_0	BY925708
E_EL_vg4M_08L11_R_0	BY934507
E_EL_vg4M_08L12_R_0	BY934508
E_EL_vg4M_08L13_F_0	BY925709
E_EL_vg4M_08L13_R_0	BY934509
E_EL_vg4M_08L15_F_0	BY925710
E_EL_vg4M_08L15_R_0	BY934510
E_EL_vg4M_08L16_F_0	BY925711
E_EL_vg4M_08L16_R_0	BY934511
E_EL_vg4M_08L17_R_0	BY934512
E_EL_vg4M_08L18_F_0	BY925712
E_EL_vg4M_08L18_R_0	BY934513
E_EL_vg4M_08L19_R_0	BY934514
E_EL_vg4M_08L20_R_0	BY934515
E_EL_vg4M_08L21_F_0	BY925713
E_EL_vg4M_08L21_R_0	BY934516
E_EL_vg4M_08L22_R_0	BY934517
E_EL_vg4M_08L23_R_0	BY934518
E_EL_vg4M_08L24_F_0	BY925714
E_EL_vg4M_08L24_R_0	BY934519
E_EL_vg4M_08M01_F_0	BY925715
E_EL_vg4M_08M02_F_0	BY925716

E_EL_vg4M_08M02_R_0	BY934520
E_EL_vg4M_08M03_F_0	BY925717
E_EL_vg4M_08M04_F_0	BY925718
E_EL_vg4M_08M04_R_0	BY934521
E_EL_vg4M_08M05_F_0	BY925719
E_EL_vg4M_08M05_R_0	BY934522
E_EL_vg4M_08M07_R_0	BY934523
E_EL_vg4M_08M08_F_0	BY925720
E_EL_vg4M_08M08_R_0	BY934524
E_EL_vg4M_08M09_R_0	BY934525
E_EL_vg4M_08M10_F_0	BY925721
E_EL_vg4M_08M10_R_0	BY934526
E_EL_vg4M_08M11_F_0	BY925722
E_EL_vg4M_08M11_R_0	BY934527
E_EL_vg4M_08M12_F_0	BY925723
E_EL_vg4M_08M12_R_0	BY934528
E_EL_vg4M_08M13_F_0	BY925724
E_EL_vg4M_08M13_R_0	BY934529
E_EL_vg4M_08M14_R_0	BY934530
E_EL_vg4M_08M15_F_0	BY925725
E_EL_vg4M_08M15_R_0	BY934531
E_EL_vg4M_08M17_F_0	BY925726
E_EL_vg4M_08M17_R_0	BY934532
E_EL_vg4M_08M18_R_0	BY934533
E_EL_vg4M_08M19_R_0	BY934534
E_EL_vg4M_08M20_F_0	BY925727
E_EL_vg4M_08M20_R_0	BY934535
E_EL_vg4M_08M21_F_0	BY925728
E_EL_vg4M_08M21_R_0	BY934536
E_EL_vg4M_08M22_R_0	BY934537
E_EL_vg4M_08M24_R_0	BY934538
E_EL_vg4M_08N01_F_0	BY925729
E_EL_vg4M_08N01_R_0	BY934539
E_EL_vg4M_08N02_F_0	BY925730
E_EL_vg4M_08N02_R_0	BY934540
E_EL_vg4M_08N03_F_0	BY925731
E_EL_vg4M_08N03_R_0	BY934541
E_EL_vg4M_08N04_F_0	BY925732
E_EL_vg4M_08N04_R_0	BY934542
E_EL_vg4M_08N05_F_0	BY925733
E_EL_vg4M_08N05_R_0	BY934543
E_EL_vg4M_08N06_F_0	BY925734
E_EL_vg4M_08N06_R_0	BY934544
E_EL_vg4M_08N07_F_0	BY925735
E_EL_vg4M_08N07_R_0	BY934545
E_EL_vg4M_08N09_F_0	BY925736
E_EL_vg4M_08N09_R_0	BY934546
E_EL_vg4M_08N10_F_0	BY925737
E_EL_vg4M_08N10_R_0	BY934547
E_EL_vg4M_08N11_F_0	BY925738
E_EL_vg4M_08N11_R_0	BY934548
E_EL_vg4M_08N12_R_0	BY934549
E_EL_vg4M_08N13_F_0	BY925739
E_EL_vg4M_08N13_R_0	BY934550

E_EL_vg4M_08N14_R_0	BY934551
E_EL_vg4M_08N15_F_0	BY925740
E_EL_vg4M_08N15_R_0	BY934552
E_EL_vg4M_08N16_F_0	BY925741
E_EL_vg4M_08N16_R_0	BY934553
E_EL_vg4M_08N17_F_0	BY925742
E_EL_vg4M_08N17_R_0	BY934554
E_EL_vg4M_08N19_F_0	BY925743
E_EL_vg4M_08N19_R_0	BY934555
E_EL_vg4M_08N20_F_0	BY925744
E_EL_vg4M_08N21_R_0	BY934556
E_EL_vg4M_08N23_F_0	BY925745
E_EL_vg4M_08N23_R_0	BY934557
E_EL_vg4M_08O01_F_0	BY925746
E_EL_vg4M_08O01_R_0	BY934558
E_EL_vg4M_08O02_R_0	BY934559
E_EL_vg4M_08O03_R_0	BY934560
E_EL_vg4M_08O04_F_0	BY925747
E_EL_vg4M_08O04_R_0	BY934561
E_EL_vg4M_08O06_F_0	BY925748
E_EL_vg4M_08O06_R_0	BY934562
E_EL_vg4M_08O07_F_0	BY925749
E_EL_vg4M_08O07_R_0	BY934563
E_EL_vg4M_08O08_F_0	BY925750
E_EL_vg4M_08O08_R_0	BY934564
E_EL_vg4M_08O09_R_0	BY934565
E_EL_vg4M_08O10_R_0	BY934566
E_EL_vg4M_08O11_F_0	BY925751
E_EL_vg4M_08O11_R_0	BY934567
E_EL_vg4M_08O12_F_0	BY925752
E_EL_vg4M_08O12_R_0	BY934568
E_EL_vg4M_08O13_F_0	BY925753
E_EL_vg4M_08O13_R_0	BY934569
E_EL_vg4M_08O14_F_0	BY925754
E_EL_vg4M_08O14_R_0	BY934570
E_EL_vg4M_08O15_F_0	BY925755
E_EL_vg4M_08O15_R_0	BY934571
E_EL_vg4M_08O16_F_0	BY925756
E_EL_vg4M_08O16_R_0	BY934572
E_EL_vg4M_08O17_R_0	BY934573
E_EL_vg4M_08O18_F_0	BY925757
E_EL_vg4M_08O18_R_0	BY934574
E_EL_vg4M_08O19_F_0	BY925758
E_EL_vg4M_08O19_R_0	BY934575
E_EL_vg4M_08O20_F_0	BY925759
E_EL_vg4M_08O20_R_0	BY934576
E_EL_vg4M_08O21_F_0	BY925760
E_EL_vg4M_08O21_R_0	BY934577
E_EL_vg4M_08O22_F_0	BY925761
E_EL_vg4M_08O22_R_0	BY934578
E_EL_vg4M_08O23_F_0	BY925762
E_EL_vg4M_08O23_R_0	BY934579
E_EL_vg4M_08O24_F_0	BY925763
E_EL_vg4M_08O24_R_0	BY934580

	E_EL_vg4M_08P01_F_0	BY925764
	E_EL_vg4M_08P01_R_0	BY934581
	E_EL_vg4M_08P02_F_0	BY925765
	E_EL_vg4M_08P02_R_0	BY934582
	E_EL_vg4M_08P03_F_0	BY925766
	E_EL_vg4M_08P03_R_0	BY934583
	E_EL_vg4M_08P04_F_0	BY925767
	E_EL_vg4M_08P04_R_0	BY934584
	E_EL_vg4M_08P05_F_0	BY925768
	E_EL_vg4M_08P05_R_0	BY934585
	E_EL_vg4M_08P07_F_0	BY925769
	E_EL_vg4M_08P07_R_0	BY934586
	E_EL_vg4M_08P08_F_0	BY925770
	E_EL_vg4M_08P08_R_0	BY934587
	E_EL_vg4M_08P09_F_0	BY925771
	E_EL_vg4M_08P09_R_0	BY934588
	E_EL_vg4M_08P10_F_0	BY925772
	E_EL_vg4M_08P10_R_0	BY934589
	E_EL_vg4M_08P11_F_0	BY925773
	E_EL_vg4M_08P11_R_0	BY934590
	E_EL_vg4M_08P12_F_0	BY925774
	E_EL_vg4M_08P12_R_0	BY934591
	E_EL_vg4M_08P13_F_0	BY925775
	E_EL_vg4M_08P13_R_0	BY934592
	E_EL_vg4M_08P15_F_0	BY925776
	E_EL_vg4M_08P15_R_0	BY934593
	E_EL_vg4M_08P16_F_0	BY925777
	E_EL_vg4M_08P16_R_0	BY934594
	E_EL_vg4M_08P17_F_0	BY925778
	E_EL_vg4M_08P17_R_0	BY934595
	E_EL_vg4M_08P18_F_0	BY925779
	E_EL_vg4M_08P18_R_0	BY934596
	E_EL_vg4M_08P19_F_0	BY925780
	E_EL_vg4M_08P20_F_0	BY925781
	E_EL_vg4M_08P20_R_0	BY934597
	E_EL_vg4M_08P21_F_0	BY925782
	E_EL_vg4M_08P21_R_0	BY934598
	E_EL_vg4M_08P22_F_0	BY925783
	E_EL_vg4M_08P22_R_0	BY934599
	E_EL_vg4M_08P23_F_0	BY925784
	E_EL_vg4M_08P23_R_0	BY934600
	E_EL_vg4M_08P24_F_0	BY925785
	E_EL_vg4M_08P24_R_0	BY934601
an--	E_ET_an--_01A04_F_0	BY914002
	E_ET_an--_01A06_F_0	BY914003
	E_ET_an--_01A07_F_0	BY914004
	E_ET_an--_01A10_F_0	BY914005
	E_ET_an--_01A11_F_0	BY914006
	E_ET_an--_01A12_F_0	BY914007
	E_ET_an--_01B01_F_0	BY914008
	E_ET_an--_01B04_F_0	BY914009
	E_ET_an--_01B05_F_0	BY914010
	E_ET_an--_01B06_F_0	BY914011
	E_ET_an--_01B08_F_0	BY914012

E_ET_an--_01B09_F_0	BY914013
E_ET_an--_01B10_F_0	BY914014
E_ET_an--_01B12_F_0	BY914015
E_ET_an--_01C01_F_0	BY914016
E_ET_an--_01C03_F_0	BY914017
E_ET_an--_01C05_F_0	BY914018
E_ET_an--_01C06_F_0	BY914019
E_ET_an--_01C09_F_0	BY914020
E_ET_an--_01C10_F_0	BY914021
E_ET_an--_01C12_F_0	BY914022
E_ET_an--_01D01_F_0	BY914023
E_ET_an--_01D02_F_0	BY914024
E_ET_an--_01D03_F_0	BY914025
E_ET_an--_01D04_F_0	BY914026
E_ET_an--_01D06_F_0	BY914027
E_ET_an--_01D07_F_0	BY914028
E_ET_an--_01D08_F_0	BY914029
E_ET_an--_01D09_F_0	BY914030
E_ET_an--_01D10_F_0	BY914031
E_ET_an--_01D11_F_0	BY914032
E_ET_an--_01D12_F_0	BY914033
E_ET_an--_01E01_F_0	BY914034
E_ET_an--_01E02_F_0	BY914035
E_ET_an--_01E04_F_0	BY914036
E_ET_an--_01E05_F_0	BY914037
E_ET_an--_01E07_F_0	BY914038
E_ET_an--_01E08_F_0	BY914039
E_ET_an--_01E09_F_0	BY914040
E_ET_an--_01E12_F_0	BY914041
E_ET_an--_01F01_F_0	BY914042
E_ET_an--_01F02_F_0	BY914043
E_ET_an--_01F05_F_0	BY914044
E_ET_an--_01F06_F_0	BY914045
E_ET_an--_01F07_F_0	BY914046
E_ET_an--_01F08_F_0	BY914047
E_ET_an--_01F10_F_0	BY914048
E_ET_an--_01F11_F_0	BY914049
E_ET_an--_01F12_F_0	BY914050
E_ET_an--_01G01_F_0	BY914051
E_ET_an--_01G03_F_0	BY914052
E_ET_an--_01G06_F_0	BY914053
E_ET_an--_01G07_F_0	BY914054
E_ET_an--_01G10_F_0	BY914055
E_ET_an--_01G11_F_0	BY914056
E_ET_an--_01H02_F_0	BY914057
E_ET_an--_01H04_F_0	BY914058
E_ET_an--_01H08_F_0	BY914059
E_ET_an--_01H09_F_0	BY914060
E_ET_an--_01H10_F_0	BY914061
E_ET_an--_01H12_F_0	BY914062
E_ET_an--_02A01_F_0	BY914063
E_ET_an--_02A02_F_0	BY914064
E_ET_an--_02A04_F_0	BY914065
E_ET_an--_02A05_F_0	BY914066

E_ET_an--_02A06_F_0	BY914067
E_ET_an--_02A07_F_0	BY914068
E_ET_an--_02A08_F_0	BY914069
E_ET_an--_02A09_F_0	BY914070
E_ET_an--_02A11_F_0	BY914071
E_ET_an--_02A12_F_0	BY914072
E_ET_an--_02B01_F_0	BY914073
E_ET_an--_02B02_F_0	BY914074
E_ET_an--_02B03_F_0	BY914075
E_ET_an--_02B04_F_0	BY914076
E_ET_an--_02B05_F_0	BY914077
E_ET_an--_02B06_F_0	BY914078
E_ET_an--_02B08_F_0	BY914079
E_ET_an--_02B09_F_0	BY914080
E_ET_an--_02B10_F_0	BY914081
E_ET_an--_02B11_F_0	BY914082
E_ET_an--_02B12_F_0	BY914083
E_ET_an--_02C01_F_0	BY914084
E_ET_an--_02C02_F_0	BY914085
E_ET_an--_02C03_F_0	BY914086
E_ET_an--_02C04_F_0	BY914087
E_ET_an--_02C08_F_0	BY914088
E_ET_an--_02C09_F_0	BY914089
E_ET_an--_02C11_F_0	BY914090
E_ET_an--_02C12_F_0	BY914091
E_ET_an--_02D01_F_0	BY914092
E_ET_an--_02D02_F_0	BY914093
E_ET_an--_02D03_F_0	BY914094
E_ET_an--_02D04_F_0	BY914095
E_ET_an--_02D06_F_0	BY914096
E_ET_an--_02D07_F_0	BY914097
E_ET_an--_02D08_F_0	BY914098
E_ET_an--_02D10_F_0	BY914099
E_ET_an--_02D11_F_0	BY914100
E_ET_an--_02E01_F_0	BY914101
E_ET_an--_02E02_F_0	BY914102
E_ET_an--_02E03_F_0	BY914103
E_ET_an--_02E04_F_0	BY914104
E_ET_an--_02E05_F_0	BY914105
E_ET_an--_02E06_F_0	BY914106
E_ET_an--_02E08_F_0	BY914107
E_ET_an--_02E09_F_0	BY914108
E_ET_an--_02E10_F_0	BY914109
E_ET_an--_02E11_F_0	BY914110
E_ET_an--_02E12_F_0	BY914111
E_ET_an--_02F02_F_0	BY914112
E_ET_an--_02F03_F_0	BY914113
E_ET_an--_02F05_F_0	BY914114
E_ET_an--_02F06_F_0	BY914115
E_ET_an--_02F08_F_0	BY914116
E_ET_an--_02F09_F_0	BY914117
E_ET_an--_02F10_F_0	BY914118
E_ET_an--_02F12_F_0	BY914119
E_ET_an--_02G01_F_0	BY914120

E_ET_an--_02G02_F_0	BY914121
E_ET_an--_02G03_F_0	BY914122
E_ET_an--_02G04_F_0	BY914123
E_ET_an--_02G06_F_0	BY914124
E_ET_an--_02G07_F_0	BY914125
E_ET_an--_02G08_F_0	BY914126
E_ET_an--_02G09_F_0	BY914127
E_ET_an--_02G10_F_0	BY914128
E_ET_an--_02G11_F_0	BY914129
E_ET_an--_02G12_F_0	BY914130
E_ET_an--_02H01_F_0	BY914131
E_ET_an--_02H02_F_0	BY914132
E_ET_an--_02H03_F_0	BY914133
E_ET_an--_02H04_F_0	BY914134
E_ET_an--_02H05_F_0	BY914135
E_ET_an--_02H06_F_0	BY914136
E_ET_an--_02H07_F_0	BY914137
E_ET_an--_02H09_F_0	BY914138
E_ET_an--_02H10_F_0	BY914139
E_ET_an--_02H12_F_0	BY914140
E_ET_an--_03A02_F_0	BY914141
E_ET_an--_03A11_F_0	BY914142
E_ET_an--_03B06_F_0	BY914143
E_ET_an--_03C07_F_0	BY914144
E_ET_an--_03C10_F_0	BY914145
E_ET_an--_03C11_F_0	BY914146
E_ET_an--_03C12_F_0	BY914147
E_ET_an--_03D01_F_0	BY914148
E_ET_an--_03D02_F_0	BY914149
E_ET_an--_03D03_F_0	BY914150
E_ET_an--_03E06_F_0	BY914151
E_ET_an--_03E08_F_0	BY914152
E_ET_an--_03F08_F_0	BY914153
E_ET_an--_03F10_F_0	BY914154
E_ET_an--_03F11_F_0	BY914155
E_ET_an--_03G02_F_0	BY914156
E_ET_an--_03G03_F_0	BY914157
E_ET_an--_03G05_F_0	BY914158
E_ET_an--_03G07_F_0	BY914159
E_ET_an--_03G08_F_0	BY914160
E_ET_an--_03G09_F_0	BY914161
E_ET_an--_03G10_F_0	BY914162
E_ET_an--_03H01_F_0	BY914163
E_ET_an--_03H02_F_0	BY914164
E_ET_an--_03H03_F_0	BY914165
E_ET_an--_03H04_F_0	BY914166
E_ET_an--_03H05_F_0	BY914167
E_ET_an--_03H06_F_0	BY914168
E_ET_an--_03H07_F_0	BY914169
E_ET_an--_03H08_F_0	BY914170
E_ET_an--_03H09_F_0	BY914171
E_ET_an--_03H10_F_0	BY914172
E_ET_an--_03H12_F_0	BY914173
E_ET_an--_04A01_F_0	BY914174

E_ET_an--_04A02_F_0	BY914175
E_ET_an--_04A03_F_0	BY914176
E_ET_an--_04A04_F_0	BY914177
E_ET_an--_04A05_F_0	BY914178
E_ET_an--_04A06_F_0	BY914179
E_ET_an--_04A07_F_0	BY914180
E_ET_an--_04A08_F_0	BY914181
E_ET_an--_04A09_F_0	BY914182
E_ET_an--_04A10_F_0	BY914183
E_ET_an--_04A11_F_0	BY914184
E_ET_an--_04A12_F_0	BY914185
E_ET_an--_04B02_F_0	BY914186
E_ET_an--_04B03_F_0	BY914187
E_ET_an--_04B04_F_0	BY914188
E_ET_an--_04B05_F_0	BY914189
E_ET_an--_04B06_F_0	BY914190
E_ET_an--_04B07_F_0	BY914191
E_ET_an--_04B08_F_0	BY914192
E_ET_an--_04B09_F_0	BY914193
E_ET_an--_04B10_F_0	BY914194
E_ET_an--_04B11_F_0	BY914195
E_ET_an--_04C01_F_0	BY914196
E_ET_an--_04C02_F_0	BY914197
E_ET_an--_04C03_F_0	BY914198
E_ET_an--_04C04_F_0	BY914199
E_ET_an--_04C05_F_0	BY914200
E_ET_an--_04C06_F_0	BY914201
E_ET_an--_04C07_F_0	BY914202
E_ET_an--_04C08_F_0	BY914203
E_ET_an--_04C09_F_0	BY914204
E_ET_an--_04C10_F_0	BY914205
E_ET_an--_04C11_F_0	BY914206
E_ET_an--_04C12_F_0	BY914207
E_ET_an--_04D01_F_0	BY914208
E_ET_an--_04D02_F_0	BY914209
E_ET_an--_04D03_F_0	BY914210
E_ET_an--_04D04_F_0	BY914211
E_ET_an--_04D05_F_0	BY914212
E_ET_an--_04D06_F_0	BY914213
E_ET_an--_04D07_F_0	BY914214
E_ET_an--_04D09_F_0	BY914215
E_ET_an--_04D10_F_0	BY914216
E_ET_an--_04D11_F_0	BY914217
E_ET_an--_04D12_F_0	BY914218
E_ET_an--_04E01_F_0	BY914219
E_ET_an--_04E02_F_0	BY914220
E_ET_an--_04E03_F_0	BY914221
E_ET_an--_04E04_F_0	BY914222
E_ET_an--_04E05_F_0	BY914223
E_ET_an--_04E06_F_0	BY914224
E_ET_an--_04E07_F_0	BY914225
E_ET_an--_04E08_F_0	BY914226
E_ET_an--_04E10_F_0	BY914227
E_ET_an--_04E11_F_0	BY914228

E_ET_an--_04E12_F_0	BY914229
E_ET_an--_04F01_F_0	BY914230
E_ET_an--_04F02_F_0	BY914231
E_ET_an--_04F03_F_0	BY914232
E_ET_an--_04F04_F_0	BY914233
E_ET_an--_04F05_F_0	BY914234
E_ET_an--_04F06_F_0	BY914235
E_ET_an--_04F07_F_0	BY914236
E_ET_an--_04F08_F_0	BY914237
E_ET_an--_04F10_F_0	BY914238
E_ET_an--_04F12_F_0	BY914239
E_ET_an--_04G01_F_0	BY914240
E_ET_an--_04G02_F_0	BY914241
E_ET_an--_04G03_F_0	BY914242
E_ET_an--_04G04_F_0	BY914243
E_ET_an--_04G05_F_0	BY914244
E_ET_an--_04G06_F_0	BY914245
E_ET_an--_04G07_F_0	BY914246
E_ET_an--_04G08_F_0	BY914247
E_ET_an--_04G09_F_0	BY914248
E_ET_an--_04G10_F_0	BY914249
E_ET_an--_04G11_F_0	BY914250
E_ET_an--_04G12_F_0	BY914251
E_ET_an--_04H01_F_0	BY914252
E_ET_an--_04H02_F_0	BY914253
E_ET_an--_04H03_F_0	BY914254
E_ET_an--_04H04_F_0	BY914255
E_ET_an--_04H05_F_0	BY914256
E_ET_an--_04H06_F_0	BY914257
E_ET_an--_04H07_F_0	BY914258
E_ET_an--_04H08_F_0	BY914259
E_ET_an--_04H09_F_0	BY914260
E_ET_an--_04H10_F_0	BY914261
E_ET_an--_04H11_F_0	BY914262
E_ET_an--_04H12_F_0	BY914263
E_ET_an--_05A01_F_0	BY914264
E_ET_an--_05A02_F_0	BY914265
E_ET_an--_05A03_F_0	BY914266
E_ET_an--_05A06_F_0	BY914267
E_ET_an--_05A07_F_0	BY914268
E_ET_an--_05A08_F_0	BY914269
E_ET_an--_05A09_F_0	BY914270
E_ET_an--_05A11_F_0	BY914271
E_ET_an--_05A12_F_0	BY914272
E_ET_an--_05B01_F_0	BY914273
E_ET_an--_05B02_F_0	BY914274
E_ET_an--_05B03_F_0	BY914275
E_ET_an--_05B04_F_0	BY914276
E_ET_an--_05B05_F_0	BY914277
E_ET_an--_05B06_F_0	BY914278
E_ET_an--_05B07_F_0	BY914279
E_ET_an--_05B08_F_0	BY914280
E_ET_an--_05B09_F_0	BY914281
E_ET_an--_05B10_F_0	BY914282

E_ET_an--_05B11_F_0	BY914283
E_ET_an--_05B12_F_0	BY914284
E_ET_an--_05C01_F_0	BY914285
E_ET_an--_05C02_F_0	BY914286
E_ET_an--_05C03_F_0	BY914287
E_ET_an--_05C04_F_0	BY914288
E_ET_an--_05C05_F_0	BY914289
E_ET_an--_05C06_F_0	BY914290
E_ET_an--_05C07_F_0	BY914291
E_ET_an--_05C08_F_0	BY914292
E_ET_an--_05C09_F_0	BY914293
E_ET_an--_05C10_F_0	BY914294
E_ET_an--_05C11_F_0	BY914295
E_ET_an--_05D02_F_0	BY914296
E_ET_an--_05D03_F_0	BY914297
E_ET_an--_05D04_F_0	BY914298
E_ET_an--_05D05_F_0	BY914299
E_ET_an--_05D06_F_0	BY914300
E_ET_an--_05D07_F_0	BY914301
E_ET_an--_05D08_F_0	BY914302
E_ET_an--_05D09_F_0	BY914303
E_ET_an--_05D10_F_0	BY914304
E_ET_an--_05D11_F_0	BY914305
E_ET_an--_05E01_F_0	BY914306
E_ET_an--_05E02_F_0	BY914307
E_ET_an--_05E04_F_0	BY914308
E_ET_an--_05E05_F_0	BY914309
E_ET_an--_05E06_F_0	BY914310
E_ET_an--_05E07_F_0	BY914311
E_ET_an--_05E08_F_0	BY914312
E_ET_an--_05E09_F_0	BY914313
E_ET_an--_05E10_F_0	BY914314
E_ET_an--_05E11_F_0	BY914315
E_ET_an--_05E12_F_0	BY914316
E_ET_an--_05F01_F_0	BY914317
E_ET_an--_05F02_F_0	BY914318
E_ET_an--_05F03_F_0	BY914319
E_ET_an--_05F04_F_0	BY914320
E_ET_an--_05F05_F_0	BY914321
E_ET_an--_05F06_F_0	BY914322
E_ET_an--_05F07_F_0	BY914323
E_ET_an--_05F08_F_0	BY914324
E_ET_an--_05F09_F_0	BY914325
E_ET_an--_05F10_F_0	BY914326
E_ET_an--_05F11_F_0	BY914327
E_ET_an--_05F12_F_0	BY914328
E_ET_an--_05G01_F_0	BY914329
E_ET_an--_05G02_F_0	BY914330
E_ET_an--_05G03_F_0	BY914331
E_ET_an--_05G04_F_0	BY914332
E_ET_an--_05G06_F_0	BY914333
E_ET_an--_05G07_F_0	BY914334
E_ET_an--_05G08_F_0	BY914335
E_ET_an--_05G09_F_0	BY914336

E_ET_an--_05G10_F_0	BY914337
E_ET_an--_05G11_F_0	BY914338
E_ET_an--_05H01_F_0	BY914339
E_ET_an--_05H02_F_0	BY914340
E_ET_an--_05H03_F_0	BY914341
E_ET_an--_05H04_F_0	BY914342
E_ET_an--_05H05_F_0	BY914343
E_ET_an--_05H06_F_0	BY914344
E_ET_an--_05H07_F_0	BY914345
E_ET_an--_05H08_F_0	BY914346
E_ET_an--_05H10_F_0	BY914347
E_ET_an--_05H12_F_0	BY914348
E_ET_an--_06F08_F_0	BY914349
E_ET_an--_06G06_F_0	BY914350
E_ET_an--_07A01_F_0	BY914351
E_ET_an--_07A02_F_0	BY914352
E_ET_an--_07A03_F_0	BY914353
E_ET_an--_07A04_F_0	BY914354
E_ET_an--_07A06_F_0	BY914355
E_ET_an--_07A07_F_0	BY914356
E_ET_an--_07A08_F_0	BY914357
E_ET_an--_07A09_F_0	BY914358
E_ET_an--_07A10_F_0	BY914359
E_ET_an--_07A11_F_0	BY914360
E_ET_an--_07A12_F_0	BY914361
E_ET_an--_07B01_F_0	BY914362
E_ET_an--_07B03_F_0	BY914363
E_ET_an--_07B05_F_0	BY914364
E_ET_an--_07B06_F_0	BY914365
E_ET_an--_07B07_F_0	BY914366
E_ET_an--_07B08_F_0	BY914367
E_ET_an--_07B09_F_0	BY914368
E_ET_an--_07B10_F_0	BY914369
E_ET_an--_07B11_F_0	BY914370
E_ET_an--_07C01_F_0	BY914371
E_ET_an--_07C03_F_0	BY914372
E_ET_an--_07C04_F_0	BY914373
E_ET_an--_07C05_F_0	BY914374
E_ET_an--_07C07_F_0	BY914375
E_ET_an--_07C08_F_0	BY914376
E_ET_an--_07C10_F_0	BY914377
E_ET_an--_07C11_F_0	BY914378
E_ET_an--_07C12_F_0	BY914379
E_ET_an--_07D01_F_0	BY914380
E_ET_an--_07D03_F_0	BY914381
E_ET_an--_07D05_F_0	BY914382
E_ET_an--_07D07_F_0	BY914383
E_ET_an--_07D08_F_0	BY914384
E_ET_an--_07D09_F_0	BY914385
E_ET_an--_07D10_F_0	BY914386
E_ET_an--_07D12_F_0	BY914387
E_ET_an--_07E01_F_0	BY914388
E_ET_an--_07E04_F_0	BY914389
E_ET_an--_07E05_F_0	BY914390

E_ET_an--_07E06_F_0	BY914391
E_ET_an--_07E07_F_0	BY914392
E_ET_an--_07E08_F_0	BY914393
E_ET_an--_07E09_F_0	BY914394
E_ET_an--_07E10_F_0	BY914395
E_ET_an--_07E11_F_0	BY914396
E_ET_an--_07F02_F_0	BY914397
E_ET_an--_07F03_F_0	BY914398
E_ET_an--_07F04_F_0	BY914399
E_ET_an--_07F05_F_0	BY914400
E_ET_an--_07F06_F_0	BY914401
E_ET_an--_07F07_F_0	BY914402
E_ET_an--_07F09_F_0	BY914403
E_ET_an--_07F10_F_0	BY914404
E_ET_an--_07F12_F_0	BY914405
E_ET_an--_07G01_F_0	BY914406
E_ET_an--_07G02_F_0	BY914407
E_ET_an--_07G03_F_0	BY914408
E_ET_an--_07G04_F_0	BY914409
E_ET_an--_07G07_F_0	BY914410
E_ET_an--_07G08_F_0	BY914411
E_ET_an--_07G10_F_0	BY914412
E_ET_an--_07G11_F_0	BY914413
E_ET_an--_07G12_F_0	BY914414
E_ET_an--_07H02_F_0	BY914415
E_ET_an--_07H03_F_0	BY914416
E_ET_an--_07H04_F_0	BY914417
E_ET_an--_07H06_F_0	BY914418
E_ET_an--_07H07_F_0	BY914419
E_ET_an--_07H08_F_0	BY914420
E_ET_an--_07H09_F_0	BY914421
E_ET_an--_07H12_F_0	BY914422
E_ET_an--_08A01_F_0	BY914423
E_ET_an--_08A02_F_0	BY914424
E_ET_an--_08B04_F_0	BY914425
E_ET_an--_08C06_F_0	BY914426
E_ET_an--_08C07_F_0	BY914427
E_ET_an--_08C08_F_0	BY914428
E_ET_an--_08D08_F_0	BY914429
E_ET_an--_08D11_F_0	BY914430
E_ET_an--_08E02_F_0	BY914431
E_ET_an--_08E03_F_0	BY914432
E_ET_an--_08E06_F_0	BY914433
E_ET_an--_08F05_F_0	BY914434
E_ET_an--_08F07_F_0	BY914435
E_ET_an--_08F08_F_0	BY914436
E_ET_an--_08F10_F_0	BY914437
E_ET_an--_08G09_F_0	BY914438
E_ET_an--_08G11_F_0	BY914439
E_ET_an--_08G12_F_0	BY914440
E_ET_an--_08H01_F_0	BY914441
E_ET_an--_08H05_F_0	BY914442
E_ET_an--_08H06_F_0	BY914443
E_ET_an--_08H07_F_0	BY914444

E_ET_an--_08H10_F_0	BY914445
E_ET_an--_09A01_F_0	BY914446
E_ET_an--_09A04_F_0	BY914447
E_ET_an--_09A06_F_0	BY914448
E_ET_an--_09A07_F_0	BY914449
E_ET_an--_09A09_F_0	BY914450
E_ET_an--_09A11_F_0	BY914451
E_ET_an--_09A12_F_0	BY914452
E_ET_an--_09B01_F_0	BY914453
E_ET_an--_09B02_F_0	BY914454
E_ET_an--_09B03_F_0	BY914455
E_ET_an--_09B04_F_0	BY914456
E_ET_an--_09B06_F_0	BY914457
E_ET_an--_09B07_F_0	BY914458
E_ET_an--_09B09_F_0	BY914459
E_ET_an--_09B10_F_0	BY914460
E_ET_an--_09B11_F_0	BY914461
E_ET_an--_09C02_F_0	BY914462
E_ET_an--_09C03_F_0	BY914463
E_ET_an--_09C04_F_0	BY914464
E_ET_an--_09C05_F_0	BY914465
E_ET_an--_09C07_F_0	BY914466
E_ET_an--_09C08_F_0	BY914467
E_ET_an--_09C10_F_0	BY914468
E_ET_an--_09C11_F_0	BY914469
E_ET_an--_09C12_F_0	BY914470
E_ET_an--_09D01_F_0	BY914471
E_ET_an--_09D02_F_0	BY914472
E_ET_an--_09D04_F_0	BY914473
E_ET_an--_09D07_F_0	BY914474
E_ET_an--_09D09_F_0	BY914475
E_ET_an--_09D10_F_0	BY914476
E_ET_an--_09D11_F_0	BY914477
E_ET_an--_09D12_F_0	BY914478
E_ET_an--_09E01_F_0	BY914479
E_ET_an--_09E02_F_0	BY914480
E_ET_an--_09E03_F_0	BY914481
E_ET_an--_09E05_F_0	BY914482
E_ET_an--_09E06_F_0	BY914483
E_ET_an--_09E07_F_0	BY914484
E_ET_an--_09E08_F_0	BY914485
E_ET_an--_09E09_F_0	BY914486
E_ET_an--_09E10_F_0	BY914487
E_ET_an--_09E11_F_0	BY914488
E_ET_an--_09E12_F_0	BY914489
E_ET_an--_09F01_F_0	BY914490
E_ET_an--_09F02_F_0	BY914491
E_ET_an--_09F03_F_0	BY914492
E_ET_an--_09F04_F_0	BY914493
E_ET_an--_09F05_F_0	BY914494
E_ET_an--_09F06_F_0	BY914495
E_ET_an--_09F07_F_0	BY914496
E_ET_an--_09F08_F_0	BY914497
E_ET_an--_09F09_F_0	BY914498

E_ET_an--_09F10_F_0	BY914499
E_ET_an--_09F11_F_0	BY914500
E_ET_an--_09F12_F_0	BY914501
E_ET_an--_09G01_F_0	BY914502
E_ET_an--_09G02_F_0	BY914503
E_ET_an--_09G03_F_0	BY914504
E_ET_an--_09G04_F_0	BY914505
E_ET_an--_09G06_F_0	BY914506
E_ET_an--_09G07_F_0	BY914507
E_ET_an--_09G08_F_0	BY914508
E_ET_an--_09G09_F_0	BY914509
E_ET_an--_09G10_F_0	BY914510
E_ET_an--_09G11_F_0	BY914511
E_ET_an--_09G12_F_0	BY914512
E_ET_an--_09H01_F_0	BY914513
E_ET_an--_09H03_F_0	BY914514
E_ET_an--_09H04_F_0	BY914515
E_ET_an--_09H05_F_0	BY914516
E_ET_an--_09H06_F_0	BY914517
E_ET_an--_09H08_F_0	BY914518
E_ET_an--_09H09_F_0	BY914519
E_ET_an--_09H10_F_0	BY914520
E_ET_an--_09H11_F_0	BY914521
E_ET_an--_09H12_F_0	BY914522
E_ET_an--_10A01_F_0	BY914523
E_ET_an--_10A02_F_0	BY914524
E_ET_an--_10A03_F_0	BY914525
E_ET_an--_10A04_F_0	BY914526
E_ET_an--_10A05_F_0	BY914527
E_ET_an--_10A06_F_0	BY914528
E_ET_an--_10A08_F_0	BY914529
E_ET_an--_10A09_F_0	BY914530
E_ET_an--_10A10_F_0	BY914531
E_ET_an--_10A11_F_0	BY914532
E_ET_an--_10A12_F_0	BY914533
E_ET_an--_10B02_F_0	BY914534
E_ET_an--_10B04_F_0	BY914535
E_ET_an--_10B06_F_0	BY914536
E_ET_an--_10B08_F_0	BY914537
E_ET_an--_10B09_F_0	BY914538
E_ET_an--_10B11_F_0	BY914539
E_ET_an--_10B12_F_0	BY914540
E_ET_an--_10C01_F_0	BY914541
E_ET_an--_10C03_F_0	BY914542
E_ET_an--_10C05_F_0	BY914543
E_ET_an--_10C06_F_0	BY914544
E_ET_an--_10C07_F_0	BY914545
E_ET_an--_10C09_F_0	BY914546
E_ET_an--_10C10_F_0	BY914547
E_ET_an--_10C11_F_0	BY914548
E_ET_an--_10C12_F_0	BY914549
E_ET_an--_10D02_F_0	BY914550
E_ET_an--_10D03_F_0	BY914551
E_ET_an--_10D04_F_0	BY914552

E_ET_an--_10D05_F_0	BY914553
E_ET_an--_10D06_F_0	BY914554
E_ET_an--_10D07_F_0	BY914555
E_ET_an--_10D09_F_0	BY914556
E_ET_an--_10D10_F_0	BY914557
E_ET_an--_10D11_F_0	BY914558
E_ET_an--_10D12_F_0	BY914559
E_ET_an--_10E02_F_0	BY914560
E_ET_an--_10E03_F_0	BY914561
E_ET_an--_10E04_F_0	BY914562
E_ET_an--_10E06_F_0	BY914563
E_ET_an--_10E07_F_0	BY914564
E_ET_an--_10E09_F_0	BY914565
E_ET_an--_10E10_F_0	BY914566
E_ET_an--_10E11_F_0	BY914567
E_ET_an--_10E12_F_0	BY914568
E_ET_an--_10F01_F_0	BY914569
E_ET_an--_10F02_F_0	BY914570
E_ET_an--_10F03_F_0	BY914571
E_ET_an--_10F05_F_0	BY914572
E_ET_an--_10F06_F_0	BY914573
E_ET_an--_10F07_F_0	BY914574
E_ET_an--_10F08_F_0	BY914575
E_ET_an--_10F09_F_0	BY914576
E_ET_an--_10F10_F_0	BY914577
E_ET_an--_10F12_F_0	BY914578
E_ET_an--_10G01_F_0	BY914579
E_ET_an--_10G02_F_0	BY914580
E_ET_an--_10G04_F_0	BY914581
E_ET_an--_10G05_F_0	BY914582
E_ET_an--_10G07_F_0	BY914583
E_ET_an--_10G08_F_0	BY914584
E_ET_an--_10G09_F_0	BY914585
E_ET_an--_10G10_F_0	BY914586
E_ET_an--_10G12_F_0	BY914587
E_ET_an--_10H01_F_0	BY914588
E_ET_an--_10H02_F_0	BY914589
E_ET_an--_10H03_F_0	BY914590
E_ET_an--_10H04_F_0	BY914591
E_ET_an--_10H05_F_0	BY914592
E_ET_an--_10H06_F_0	BY914593
E_ET_an--_10H07_F_0	BY914594
E_ET_an--_10H08_F_0	BY914595
E_ET_an--_10H09_F_0	BY914596
E_ET_an--_10H10_F_0	BY914597
E_ET_an--_10H11_F_0	BY914598
E_ET_an--_10H12_F_0	BY914599
E_ET_an--_11A01_F_0	BY914600
E_ET_an--_11A02_F_0	BY914601
E_ET_an--_11A03_F_0	BY914602
E_ET_an--_11A04_F_0	BY914603
E_ET_an--_11A05_F_0	BY914604
E_ET_an--_11A06_F_0	BY914605
E_ET_an--_11A07_F_0	BY914606

	E_ET_an--_11A08_F_0	BY914607
	E_ET_an--_11A09_F_0	BY914608
	E_ET_an--_11A10_F_0	BY914609
	E_ET_an--_11A11_F_0	BY914610
	E_ET_an--_11A12_F_0	BY914611
	E_ET_an--_11B01_F_0	BY914612
	E_ET_an--_11B04_F_0	BY914613
	E_ET_an--_11B05_F_0	BY914614
	E_ET_an--_11B06_F_0	BY914615
	E_ET_an--_11B07_F_0	BY914616
	E_ET_an--_11B09_F_0	BY914617
	E_ET_an--_11B10_F_0	BY914618
	E_ET_an--_11B11_F_0	BY914619
	E_ET_an--_11B12_F_0	BY914620
	E_ET_an--_11C01_F_0	BY914621
	E_ET_an--_11C02_F_0	BY914622
	E_ET_an--_11C05_F_0	BY914623
	E_ET_an--_11C06_F_0	BY914624
	E_ET_an--_11C07_F_0	BY914625
	E_ET_an--_11C08_F_0	BY914626
	E_ET_an--_11C10_F_0	BY914627
	E_ET_an--_11C11_F_0	BY914628
	E_ET_an--_11C12_F_0	BY914629
	E_ET_an--_11D01_F_0	BY914630
	E_ET_an--_11D02_F_0	BY914631
	E_ET_an--_11D03_F_0	BY914632
	E_ET_an--_11D04_F_0	BY914633
epV3	E_ET_epV3_09A03_F_1	BB981956
	E_ET_epV3_09A04_F_1	BB981957
	E_ET_epV3_09A05_F_1	BB981958
	E_ET_epV3_09A07_F_1	BB981959
	E_ET_epV3_09A08_F_1	BB981960
	E_ET_epV3_09A10_F_1	BB981961
	E_ET_epV3_09B01_F_1	BB981962
	E_ET_epV3_09B02_F_1	BB981963
	E_ET_epV3_09B03_F_1	BB981964
	E_ET_epV3_09B04_F_1	BB981965
	E_ET_epV3_09B06_F_1	BB981966
	E_ET_epV3_09B08_F_1	BB981967
	E_ET_epV3_09B09_F_1	BB981968
	E_ET_epV3_09B10_F_1	BB981969
	E_ET_epV3_09B11_F_1	BB981970
	E_ET_epV3_09B12_F_1	BB981971
	E_ET_epV3_09C02_F_1	BB981972
	E_ET_epV3_09C04_F_1	BB981973
	E_ET_epV3_09C05_F_1	BB981974
	E_ET_epV3_09C06_F_1	BB981975
	E_ET_epV3_09C07_F_1	BB981976
	E_ET_epV3_09C08_F_1	BB981977
	E_ET_epV3_09C09_F_1	BB981978
	E_ET_epV3_09C11_F_1	BB981979
	E_ET_epV3_09D03_F_1	BB981980
	E_ET_epV3_09D05_F_1	BB981981
	E_ET_epV3_09D06_F_1	BB981982

	E_ET_epV3_09D07_F_1	BB981983
	E_ET_epV3_09D08_F_1	BB981984
	E_ET_epV3_09D09_F_1	BB981985
	E_ET_epV3_09D10_F_1	BB981986
	E_ET_epV3_09D11_F_1	BB981987
	E_ET_epV3_09D12_F_1	BB981988
	E_ET_epV3_09E03_F_1	BB981989
	E_ET_epV3_09E04_F_1	BB981990
	E_ET_epV3_09E07_F_1	BB981991
	E_ET_epV3_09E08_F_1	BB981992
	E_ET_epV3_09E09_F_1	BB981993
	E_ET_epV3_09E10_F_1	BB981994
	E_ET_epV3_09E11_F_1	BB981995
	E_ET_epV3_09E12_F_1	BB981996
	E_ET_epV3_09F01_F_1	BB981997
	E_ET_epV3_09F02_F_1	BB981998
	E_ET_epV3_09F04_F_1	BB981999
	E_ET_epV3_09F05_F_1	BB982000
	E_ET_epV3_09F07_F_1	BB982001
	E_ET_epV3_09F08_F_1	BB982002
	E_ET_epV3_09F11_F_1	BB982003
	E_ET_epV3_09F12_F_1	BB982004
	E_ET_epV3_09G03_F_1	BB982005
	E_ET_epV3_09G04_F_1	BB982006
	E_ET_epV3_09G05_F_1	BB982007
	E_ET_epV3_09G07_F_1	BB982008
	E_ET_epV3_09G08_F_1	BB982009
	E_ET_epV3_09G09_F_1	BB982010
	E_ET_epV3_09G10_F_1	BB982011
	E_ET_epV3_09H01_F_1	BB982012
	E_ET_epV3_09H02_F_1	BB982013
	E_ET_epV3_09H03_F_1	BB982014
	E_ET_epV3_09H07_F_1	BB982015
	E_ET_epV3_09H09_F_1	BB982016
	E_ET_epV3_09H11_F_1	BB982017
	E_ET_epV3_09H12_F_1	BB982018
	E_ET_epV3_13C05_F_1	BB982019
	E_ET_epV3_15E05_F_1	BB982020
	E_ET_epV3_15G11_F_1	BB982021
	E_ET_epV3_15G12_F_1	BB982022
	E_ET_epV3_18E07_F_1	BB982023
	E_ET_epV3_18F04_F_1	BB982024
	E_ET_epV3_19A04_F_1	BB982025
	E_ET_epV3_19B10_F_1	BB982026
	E_ET_epV3_19C09_F_1	BB982027
	E_ET_epV3_21A01_F_1	BB982028
	E_ET_epV3_21B01_F_1	BB982029
	E_ET_epV3_21E07_F_1	BB982030
	E_ET_epV3_22F03_F_1	BB982031
F1mg	E_ET_F1mg_01A01_F_0	BY917461
	E_ET_F1mg_01A01_R_0	BY925786
	E_ET_F1mg_01A02_F_0	BY917462
	E_ET_F1mg_01A02_R_0	BY925787
	E_ET_F1mg_01A03_F_0	BY917463

E_ET_F1mg_01A03_R_0	BY925788
E_ET_F1mg_01A05_F_0	BY917464
E_ET_F1mg_01A05_R_0	BY925789
E_ET_F1mg_01A06_F_0	BY917465
E_ET_F1mg_01A06_R_0	BY925790
E_ET_F1mg_01A07_R_0	BY925791
E_ET_F1mg_01A08_F_0	BY917466
E_ET_F1mg_01A08_R_0	BY925792
E_ET_F1mg_01A09_F_0	BY917467
E_ET_F1mg_01A09_R_0	BY925793
E_ET_F1mg_01A12_F_0	BY917468
E_ET_F1mg_01A12_R_0	BY925794
E_ET_F1mg_01B01_F_0	BY917469
E_ET_F1mg_01B01_R_0	BY925795
E_ET_F1mg_01B02_F_0	BY917470
E_ET_F1mg_01B02_R_0	BY925796
E_ET_F1mg_01B04_F_0	BY917471
E_ET_F1mg_01B04_R_0	BY925797
E_ET_F1mg_01B05_F_0	BY917472
E_ET_F1mg_01B05_R_0	BY925798
E_ET_F1mg_01B07_R_0	BY925799
E_ET_F1mg_01B08_F_0	BY917473
E_ET_F1mg_01B08_R_0	BY925800
E_ET_F1mg_01B09_F_0	BY917474
E_ET_F1mg_01B09_R_0	BY925801
E_ET_F1mg_01B10_F_0	BY917475
E_ET_F1mg_01B10_R_0	BY925802
E_ET_F1mg_01B11_F_0	BY917476
E_ET_F1mg_01B11_R_0	BY925803
E_ET_F1mg_01B12_F_0	BY917477
E_ET_F1mg_01B12_R_0	BY925804
E_ET_F1mg_01C01_F_0	BY917478
E_ET_F1mg_01C01_R_0	BY925805
E_ET_F1mg_01C02_F_0	BY917479
E_ET_F1mg_01C02_R_0	BY925806
E_ET_F1mg_01C05_F_0	BY917480
E_ET_F1mg_01C06_F_0	BY917481
E_ET_F1mg_01C06_R_0	BY925807
E_ET_F1mg_01C08_F_0	BY917482
E_ET_F1mg_01C12_F_0	BY917483
E_ET_F1mg_01C12_R_0	BY925808
E_ET_F1mg_01D02_F_0	BY917484
E_ET_F1mg_01D02_R_0	BY925809
E_ET_F1mg_01D04_F_0	BY917485
E_ET_F1mg_01D04_R_0	BY925810
E_ET_F1mg_01D07_F_0	BY917486
E_ET_F1mg_01D07_R_0	BY925811
E_ET_F1mg_01D09_F_0	BY917487
E_ET_F1mg_01D09_R_0	BY925812
E_ET_F1mg_01D10_F_0	BY917488
E_ET_F1mg_01D10_R_0	BY925813
E_ET_F1mg_01D11_R_0	BY925814
E_ET_F1mg_01E01_F_0	BY917489
E_ET_F1mg_01E01_R_0	BY925815

E_ET_F1mg_01E02_F_0	BY917490
E_ET_F1mg_01E02_R_0	BY925816
E_ET_F1mg_01E03_F_0	BY917491
E_ET_F1mg_01E03_R_0	BY925817
E_ET_F1mg_01E04_R_0	BY925818
E_ET_F1mg_01E07_F_0	BY917492
E_ET_F1mg_01E07_R_0	BY925819
E_ET_F1mg_01E09_F_0	BY917493
E_ET_F1mg_01E09_R_0	BY925820
E_ET_F1mg_01E10_F_0	BY917494
E_ET_F1mg_01E10_R_0	BY925821
E_ET_F1mg_01E12_F_0	BY917495
E_ET_F1mg_01E12_R_0	BY925822
E_ET_F1mg_01F01_F_0	BY917496
E_ET_F1mg_01F01_R_0	BY925823
E_ET_F1mg_01F02_F_0	BY917497
E_ET_F1mg_01F02_R_0	BY925824
E_ET_F1mg_01F03_F_0	BY917498
E_ET_F1mg_01F03_R_0	BY925825
E_ET_F1mg_01F05_F_0	BY917499
E_ET_F1mg_01F05_R_0	BY925826
E_ET_F1mg_01F09_F_0	BY917500
E_ET_F1mg_01F09_R_0	BY925827
E_ET_F1mg_01F10_R_0	BY925828
E_ET_F1mg_01F11_F_0	BY917501
E_ET_F1mg_01F11_R_0	BY925829
E_ET_F1mg_01F12_F_0	BY917502
E_ET_F1mg_01F12_R_0	BY925830
E_ET_F1mg_01G01_F_0	BY917503
E_ET_F1mg_01G01_R_0	BY925831
E_ET_F1mg_01G02_F_0	BY917504
E_ET_F1mg_01G02_R_0	BY925832
E_ET_F1mg_01G03_F_0	BY917505
E_ET_F1mg_01G03_R_0	BY925833
E_ET_F1mg_01G04_F_0	BY917506
E_ET_F1mg_01G04_R_0	BY925834
E_ET_F1mg_01G06_F_0	BY917507
E_ET_F1mg_01G06_R_0	BY925835
E_ET_F1mg_01G07_F_0	BY917508
E_ET_F1mg_01G07_R_0	BY925836
E_ET_F1mg_01G08_F_0	BY917509
E_ET_F1mg_01G08_R_0	BY925837
E_ET_F1mg_01G09_R_0	BY925838
E_ET_F1mg_01G10_F_0	BY917510
E_ET_F1mg_01G11_F_0	BY917511
E_ET_F1mg_01G11_R_0	BY925839
E_ET_F1mg_01G12_F_0	BY917512
E_ET_F1mg_01G12_R_0	BY925840
E_ET_F1mg_01H03_F_0	BY917513
E_ET_F1mg_01H03_R_0	BY925841
E_ET_F1mg_01H04_F_0	BY917514
E_ET_F1mg_01H04_R_0	BY925842
E_ET_F1mg_01H07_R_0	BY925843
E_ET_F1mg_01H08_F_0	BY917515

E_ET_F1mg_01H08_R_0	BY925844
E_ET_F1mg_01H09_R_0	BY925845
E_ET_F1mg_01H10_F_0	BY917516
E_ET_F1mg_01H10_R_0	BY925846
E_ET_F1mg_01H12_F_0	BY917517
E_ET_F1mg_01H12_R_0	BY925847
E_ET_F1mg_02A02_F_0	BY917518
E_ET_F1mg_02A02_R_0	BY925848
E_ET_F1mg_02A03_F_0	BY917519
E_ET_F1mg_02A03_R_0	BY925849
E_ET_F1mg_02A04_F_0	BY917520
E_ET_F1mg_02A04_R_0	BY925850
E_ET_F1mg_02A05_F_0	BY917521
E_ET_F1mg_02A05_R_0	BY925851
E_ET_F1mg_02A07_F_0	BY917522
E_ET_F1mg_02A07_R_0	BY925852
E_ET_F1mg_02A08_F_0	BY917523
E_ET_F1mg_02A08_R_0	BY925853
E_ET_F1mg_02A09_F_0	BY917524
E_ET_F1mg_02A09_R_0	BY925854
E_ET_F1mg_02A10_F_0	BY917525
E_ET_F1mg_02A10_R_0	BY925855
E_ET_F1mg_02A11_F_0	BY917526
E_ET_F1mg_02A12_F_0	BY917527
E_ET_F1mg_02A12_R_0	BY925856
E_ET_F1mg_02B03_F_0	BY917528
E_ET_F1mg_02B03_R_0	BY925857
E_ET_F1mg_02B04_F_0	BY917529
E_ET_F1mg_02B05_F_0	BY917530
E_ET_F1mg_02B05_R_0	BY925858
E_ET_F1mg_02B06_F_0	BY917531
E_ET_F1mg_02B06_R_0	BY925859
E_ET_F1mg_02B08_F_0	BY917532
E_ET_F1mg_02B08_R_0	BY925860
E_ET_F1mg_02B09_F_0	BY917533
E_ET_F1mg_02B09_R_0	BY925861
E_ET_F1mg_02B11_F_0	BY917534
E_ET_F1mg_02B11_R_0	BY925862
E_ET_F1mg_02B12_F_0	BY917535
E_ET_F1mg_02B12_R_0	BY925863
E_ET_F1mg_02C01_F_0	BY917536
E_ET_F1mg_02C01_R_0	BY925864
E_ET_F1mg_02C02_F_0	BY917537
E_ET_F1mg_02C03_F_0	BY917538
E_ET_F1mg_02C03_R_0	BY925865
E_ET_F1mg_02C05_F_0	BY917539
E_ET_F1mg_02C05_R_0	BY925866
E_ET_F1mg_02C06_F_0	BY917540
E_ET_F1mg_02C06_R_0	BY925867
E_ET_F1mg_02C07_F_0	BY917541
E_ET_F1mg_02C07_R_0	BY925868
E_ET_F1mg_02C09_F_0	BY917542
E_ET_F1mg_02C09_R_0	BY925869
E_ET_F1mg_02C10_F_0	BY917543

E_ET_F1mg_02C10_R_0	BY925870
E_ET_F1mg_02C12_F_0	BY917544
E_ET_F1mg_02C12_R_0	BY925871
E_ET_F1mg_02D03_F_0	BY917545
E_ET_F1mg_02D03_R_0	BY925872
E_ET_F1mg_02D06_F_0	BY917546
E_ET_F1mg_02D06_R_0	BY925873
E_ET_F1mg_02D08_F_0	BY917547
E_ET_F1mg_02D09_F_0	BY917548
E_ET_F1mg_02D09_R_0	BY925874
E_ET_F1mg_02D11_F_0	BY917549
E_ET_F1mg_02D11_R_0	BY925875
E_ET_F1mg_02D12_F_0	BY917550
E_ET_F1mg_02D12_R_0	BY925876
E_ET_F1mg_02E06_F_0	BY917551
E_ET_F1mg_02E06_R_0	BY925877
E_ET_F1mg_02E08_F_0	BY917552
E_ET_F1mg_02E08_R_0	BY925878
E_ET_F1mg_02E10_F_0	BY917553
E_ET_F1mg_02E10_R_0	BY925879
E_ET_F1mg_02E12_F_0	BY917554
E_ET_F1mg_02E12_R_0	BY925880
E_ET_F1mg_02F01_F_0	BY917555
E_ET_F1mg_02F01_R_0	BY925881
E_ET_F1mg_02F03_F_0	BY917556
E_ET_F1mg_02F03_R_0	BY925882
E_ET_F1mg_02F06_F_0	BY917557
E_ET_F1mg_02F06_R_0	BY925883
E_ET_F1mg_02F07_F_0	BY917558
E_ET_F1mg_02F07_R_0	BY925884
E_ET_F1mg_02F08_F_0	BY917559
E_ET_F1mg_02F08_R_0	BY925885
E_ET_F1mg_02F09_F_0	BY917560
E_ET_F1mg_02F10_F_0	BY917561
E_ET_F1mg_02F10_R_0	BY925886
E_ET_F1mg_02F11_F_0	BY917562
E_ET_F1mg_02F11_R_0	BY925887
E_ET_F1mg_02F12_F_0	BY917563
E_ET_F1mg_02F12_R_0	BY925888
E_ET_F1mg_02G02_F_0	BY917564
E_ET_F1mg_02G02_R_0	BY925889
E_ET_F1mg_02G03_F_0	BY917565
E_ET_F1mg_02G03_R_0	BY925890
E_ET_F1mg_02G04_F_0	BY917566
E_ET_F1mg_02G04_R_0	BY925891
E_ET_F1mg_02G06_F_0	BY917567
E_ET_F1mg_02G06_R_0	BY925892
E_ET_F1mg_02G08_F_0	BY917568
E_ET_F1mg_02G08_R_0	BY925893
E_ET_F1mg_02G09_F_0	BY917569
E_ET_F1mg_02G09_R_0	BY925894
E_ET_F1mg_02G11_F_0	BY917570
E_ET_F1mg_02G11_R_0	BY925895
E_ET_F1mg_02G12_F_0	BY917571

E_ET_F1mg_02G12_R_0	BY925896
E_ET_F1mg_02H01_F_0	BY917572
E_ET_F1mg_02H01_R_0	BY925897
E_ET_F1mg_02H03_F_0	BY917573
E_ET_F1mg_02H03_R_0	BY925898
E_ET_F1mg_02H06_F_0	BY917574
E_ET_F1mg_02H06_R_0	BY925899
E_ET_F1mg_02H08_F_0	BY917575
E_ET_F1mg_02H09_F_0	BY917576
E_ET_F1mg_02H09_R_0	BY925900
E_ET_F1mg_02H11_F_0	BY917577
E_ET_F1mg_02H11_R_0	BY925901
E_ET_F1mg_02H12_F_0	BY917578
E_ET_F1mg_03A01_R_0	BY925902
E_ET_F1mg_03A04_F_0	BY917579
E_ET_F1mg_03A04_R_0	BY925903
E_ET_F1mg_03A05_F_0	BY917580
E_ET_F1mg_03A05_R_0	BY925904
E_ET_F1mg_03A06_F_0	BY917581
E_ET_F1mg_03A06_R_0	BY925905
E_ET_F1mg_03A11_F_0	BY917582
E_ET_F1mg_03A11_R_0	BY925906
E_ET_F1mg_03B02_F_0	BY917583
E_ET_F1mg_03B02_R_0	BY925907
E_ET_F1mg_03B03_F_0	BY917584
E_ET_F1mg_03B03_R_0	BY925908
E_ET_F1mg_03B04_F_0	BY917585
E_ET_F1mg_03B04_R_0	BY925909
E_ET_F1mg_03B05_F_0	BY917586
E_ET_F1mg_03B05_R_0	BY925910
E_ET_F1mg_03B07_F_0	BY917587
E_ET_F1mg_03B07_R_0	BY925911
E_ET_F1mg_03B08_F_0	BY917588
E_ET_F1mg_03B08_R_0	BY925912
E_ET_F1mg_03B10_F_0	BY917589
E_ET_F1mg_03B10_R_0	BY925913
E_ET_F1mg_03B11_F_0	BY917590
E_ET_F1mg_03B11_R_0	BY925914
E_ET_F1mg_03C01_F_0	BY917591
E_ET_F1mg_03C01_R_0	BY925915
E_ET_F1mg_03C04_F_0	BY917592
E_ET_F1mg_03C04_R_0	BY925916
E_ET_F1mg_03C05_F_0	BY917593
E_ET_F1mg_03C05_R_0	BY925917
E_ET_F1mg_03C06_F_0	BY917594
E_ET_F1mg_03C06_R_0	BY925918
E_ET_F1mg_03C09_F_0	BY917595
E_ET_F1mg_03C11_F_0	BY917596
E_ET_F1mg_03C11_R_0	BY925919
E_ET_F1mg_03C12_F_0	BY917597
E_ET_F1mg_03C12_R_0	BY925920
E_ET_F1mg_03D01_F_0	BY917598
E_ET_F1mg_03D01_R_0	BY925921
E_ET_F1mg_03D02_F_0	BY917599

E_ET_F1mg_03D02_R_0	BY925922
E_ET_F1mg_03D03_F_0	BY917600
E_ET_F1mg_03D03_R_0	BY925923
E_ET_F1mg_03D04_F_0	BY917601
E_ET_F1mg_03D04_R_0	BY925924
E_ET_F1mg_03D05_F_0	BY917602
E_ET_F1mg_03D05_R_0	BY925925
E_ET_F1mg_03D06_F_0	BY917603
E_ET_F1mg_03D06_R_0	BY925926
E_ET_F1mg_03D08_F_0	BY917604
E_ET_F1mg_03D08_R_0	BY925927
E_ET_F1mg_03D12_R_0	BY925928
E_ET_F1mg_03E01_F_0	BY917605
E_ET_F1mg_03E01_R_0	BY925929
E_ET_F1mg_03E04_F_0	BY917606
E_ET_F1mg_03E04_R_0	BY925930
E_ET_F1mg_03E05_F_0	BY917607
E_ET_F1mg_03E05_R_0	BY925931
E_ET_F1mg_03E06_F_0	BY917608
E_ET_F1mg_03E06_R_0	BY925932
E_ET_F1mg_03E07_F_0	BY917609
E_ET_F1mg_03E07_R_0	BY925933
E_ET_F1mg_03E08_F_0	BY917610
E_ET_F1mg_03E08_R_0	BY925934
E_ET_F1mg_03E10_F_0	BY917611
E_ET_F1mg_03E10_R_0	BY925935
E_ET_F1mg_03E11_F_0	BY917612
E_ET_F1mg_03E11_R_0	BY925936
E_ET_F1mg_03E12_F_0	BY917613
E_ET_F1mg_03E12_R_0	BY925937
E_ET_F1mg_03F01_F_0	BY917614
E_ET_F1mg_03F01_R_0	BY925938
E_ET_F1mg_03F02_F_0	BY917615
E_ET_F1mg_03F02_R_0	BY925939
E_ET_F1mg_03F03_F_0	BY917616
E_ET_F1mg_03F03_R_0	BY925940
E_ET_F1mg_03F04_F_0	BY917617
E_ET_F1mg_03F04_R_0	BY925941
E_ET_F1mg_03F05_F_0	BY917618
E_ET_F1mg_03F05_R_0	BY925942
E_ET_F1mg_03F06_F_0	BY917619
E_ET_F1mg_03F06_R_0	BY925943
E_ET_F1mg_03F07_F_0	BY917620
E_ET_F1mg_03F07_R_0	BY925944
E_ET_F1mg_03F08_F_0	BY917621
E_ET_F1mg_03F08_R_0	BY925945
E_ET_F1mg_03F10_F_0	BY917622
E_ET_F1mg_03F10_R_0	BY925946
E_ET_F1mg_03F12_F_0	BY917623
E_ET_F1mg_03F12_R_0	BY925947
E_ET_F1mg_03G01_F_0	BY917624
E_ET_F1mg_03G01_R_0	BY925948
E_ET_F1mg_03G03_F_0	BY917625
E_ET_F1mg_03G03_R_0	BY925949

E_ET_F1mg_03G05_F_0	BY917626
E_ET_F1mg_03G05_R_0	BY925950
E_ET_F1mg_03G09_F_0	BY917627
E_ET_F1mg_03G09_R_0	BY925951
E_ET_F1mg_03G11_F_0	BY917628
E_ET_F1mg_03G11_R_0	BY925952
E_ET_F1mg_03G12_F_0	BY917629
E_ET_F1mg_03G12_R_0	BY925953
E_ET_F1mg_03H02_F_0	BY917630
E_ET_F1mg_03H02_R_0	BY925954
E_ET_F1mg_03H07_F_0	BY917631
E_ET_F1mg_03H08_F_0	BY917632
E_ET_F1mg_03H08_R_0	BY925955
E_ET_F1mg_03H09_F_0	BY917633
E_ET_F1mg_03H09_R_0	BY925956
E_ET_F1mg_03H10_F_0	BY917634
E_ET_F1mg_03H10_R_0	BY925957
E_ET_F1mg_03H11_F_0	BY917635
E_ET_F1mg_03H11_R_0	BY925958
E_ET_F1mg_03H12_F_0	BY917636
E_ET_F1mg_03H12_R_0	BY925959
E_ET_F1mg_04A02_F_0	BY917637
E_ET_F1mg_04A03_F_0	BY917638
E_ET_F1mg_04A04_F_0	BY917639
E_ET_F1mg_04A05_F_0	BY917640
E_ET_F1mg_04A05_R_0	BY925960
E_ET_F1mg_04A08_F_0	BY917641
E_ET_F1mg_04A08_R_0	BY925961
E_ET_F1mg_04A09_F_0	BY917642
E_ET_F1mg_04A10_F_0	BY917643
E_ET_F1mg_04A10_R_0	BY925962
E_ET_F1mg_04A12_F_0	BY917644
E_ET_F1mg_04A12_R_0	BY925963
E_ET_F1mg_04B02_F_0	BY917645
E_ET_F1mg_04B02_R_0	BY925964
E_ET_F1mg_04B03_F_0	BY917646
E_ET_F1mg_04B03_R_0	BY925965
E_ET_F1mg_04B04_R_0	BY925966
E_ET_F1mg_04B05_F_0	BY917647
E_ET_F1mg_04B05_R_0	BY925967
E_ET_F1mg_04B06_F_0	BY917648
E_ET_F1mg_04B06_R_0	BY925968
E_ET_F1mg_04B07_F_0	BY917649
E_ET_F1mg_04B07_R_0	BY925969
E_ET_F1mg_04B08_F_0	BY917650
E_ET_F1mg_04B09_F_0	BY917651
E_ET_F1mg_04B09_R_0	BY925970
E_ET_F1mg_04B10_F_0	BY917652
E_ET_F1mg_04B11_F_0	BY917653
E_ET_F1mg_04B11_R_0	BY925971
E_ET_F1mg_04B12_F_0	BY917654
E_ET_F1mg_04C01_F_0	BY917655
E_ET_F1mg_04C01_R_0	BY925972
E_ET_F1mg_04C02_F_0	BY917656

E_ET_F1mg_04C02_R_0	BY925973
E_ET_F1mg_04C03_F_0	BY917657
E_ET_F1mg_04C04_F_0	BY917658
E_ET_F1mg_04C04_R_0	BY925974
E_ET_F1mg_04C05_F_0	BY917659
E_ET_F1mg_04C06_F_0	BY917660
E_ET_F1mg_04C06_R_0	BY925975
E_ET_F1mg_04C08_F_0	BY917661
E_ET_F1mg_04C09_F_0	BY917662
E_ET_F1mg_04C09_R_0	BY925976
E_ET_F1mg_04C11_F_0	BY917663
E_ET_F1mg_04C11_R_0	BY925977
E_ET_F1mg_04C12_R_0	BY925978
E_ET_F1mg_04D01_R_0	BY925979
E_ET_F1mg_04D02_F_0	BY917664
E_ET_F1mg_04D02_R_0	BY925980
E_ET_F1mg_04D03_R_0	BY925981
E_ET_F1mg_04D05_F_0	BY917665
E_ET_F1mg_04D05_R_0	BY925982
E_ET_F1mg_04D06_R_0	BY925983
E_ET_F1mg_04D07_F_0	BY917666
E_ET_F1mg_04D08_F_0	BY917667
E_ET_F1mg_04D08_R_0	BY925984
E_ET_F1mg_04D09_F_0	BY917668
E_ET_F1mg_04D10_R_0	BY925985
E_ET_F1mg_04D11_F_0	BY917669
E_ET_F1mg_04D11_R_0	BY925986
E_ET_F1mg_04E01_F_0	BY917670
E_ET_F1mg_04E01_R_0	BY925987
E_ET_F1mg_04E02_F_0	BY917671
E_ET_F1mg_04E03_F_0	BY917672
E_ET_F1mg_04E03_R_0	BY925988
E_ET_F1mg_04E04_F_0	BY917673
E_ET_F1mg_04E04_R_0	BY925989
E_ET_F1mg_04E05_F_0	BY917674
E_ET_F1mg_04E05_R_0	BY925990
E_ET_F1mg_04E06_F_0	BY917675
E_ET_F1mg_04E07_F_0	BY917676
E_ET_F1mg_04E07_R_0	BY925991
E_ET_F1mg_04E08_F_0	BY917677
E_ET_F1mg_04E08_R_0	BY925992
E_ET_F1mg_04E09_R_0	BY925993
E_ET_F1mg_04E10_F_0	BY917678
E_ET_F1mg_04E11_F_0	BY917679
E_ET_F1mg_04E12_R_0	BY925994
E_ET_F1mg_04F01_F_0	BY917680
E_ET_F1mg_04F01_R_0	BY925995
E_ET_F1mg_04F02_F_0	BY917681
E_ET_F1mg_04F02_R_0	BY925996
E_ET_F1mg_04F03_F_0	BY917682
E_ET_F1mg_04F04_F_0	BY917683
E_ET_F1mg_04F05_F_0	BY917684
E_ET_F1mg_04F06_F_0	BY917685
E_ET_F1mg_04F06_R_0	BY925997

E_ET_F1mg_04F07_F_0	BY917686
E_ET_F1mg_04F08_R_0	BY925998
E_ET_F1mg_04F10_F_0	BY917687
E_ET_F1mg_04F11_F_0	BY917688
E_ET_F1mg_04F11_R_0	BY925999
E_ET_F1mg_04F12_R_0	BY926000
E_ET_F1mg_04G01_F_0	BY917689
E_ET_F1mg_04G02_R_0	BY926001
E_ET_F1mg_04G03_F_0	BY917690
E_ET_F1mg_04G03_R_0	BY926002
E_ET_F1mg_04G04_F_0	BY917691
E_ET_F1mg_04G04_R_0	BY926003
E_ET_F1mg_04G05_F_0	BY917692
E_ET_F1mg_04G05_R_0	BY926004
E_ET_F1mg_04G06_F_0	BY917693
E_ET_F1mg_04G06_R_0	BY926005
E_ET_F1mg_04G07_F_0	BY917694
E_ET_F1mg_04G08_F_0	BY917695
E_ET_F1mg_04G10_F_0	BY917696
E_ET_F1mg_04G10_R_0	BY926006
E_ET_F1mg_04G11_R_0	BY926007
E_ET_F1mg_04G12_R_0	BY926008
E_ET_F1mg_04H01_R_0	BY926009
E_ET_F1mg_04H02_F_0	BY917697
E_ET_F1mg_04H03_R_0	BY926010
E_ET_F1mg_04H04_F_0	BY917698
E_ET_F1mg_04H04_R_0	BY926011
E_ET_F1mg_04H05_F_0	BY917699
E_ET_F1mg_04H05_R_0	BY926012
E_ET_F1mg_04H06_F_0	BY917700
E_ET_F1mg_04H07_F_0	BY917701
E_ET_F1mg_04H08_F_0	BY917702
E_ET_F1mg_04H09_R_0	BY926013
E_ET_F1mg_04H10_F_0	BY917703
E_ET_F1mg_04H12_F_0	BY917704
E_ET_F1mg_04H12_R_0	BY926014
E_ET_F1mg_05A05_F_0	BY917705
E_ET_F1mg_05A05_R_0	BY926015
E_ET_F1mg_05A08_F_0	BY917706
E_ET_F1mg_05A08_R_0	BY926016
E_ET_F1mg_05A09_F_0	BY917707
E_ET_F1mg_05A09_R_0	BY926017
E_ET_F1mg_05A10_F_0	BY917708
E_ET_F1mg_05A10_R_0	BY926018
E_ET_F1mg_05A12_F_0	BY917709
E_ET_F1mg_05A12_R_0	BY926019
E_ET_F1mg_05B02_F_0	BY917710
E_ET_F1mg_05B02_R_0	BY926020
E_ET_F1mg_05B03_F_0	BY917711
E_ET_F1mg_05B03_R_0	BY926021
E_ET_F1mg_05B04_F_0	BY917712
E_ET_F1mg_05B04_R_0	BY926022
E_ET_F1mg_05B05_F_0	BY917713
E_ET_F1mg_05B05_R_0	BY926023

E_ET_F1mg_05B09_F_0	BY917714
E_ET_F1mg_05B09_R_0	BY926024
E_ET_F1mg_05B11_F_0	BY917715
E_ET_F1mg_05B11_R_0	BY926025
E_ET_F1mg_05C01_F_0	BY917716
E_ET_F1mg_05C01_R_0	BY926026
E_ET_F1mg_05C02_F_0	BY917717
E_ET_F1mg_05C02_R_0	BY926027
E_ET_F1mg_05C04_F_0	BY917718
E_ET_F1mg_05C04_R_0	BY926028
E_ET_F1mg_05C06_F_0	BY917719
E_ET_F1mg_05C06_R_0	BY926029
E_ET_F1mg_05C09_F_0	BY917720
E_ET_F1mg_05C09_R_0	BY926030
E_ET_F1mg_05C11_F_0	BY917721
E_ET_F1mg_05C11_R_0	BY926031
E_ET_F1mg_05C12_F_0	BY917722
E_ET_F1mg_05C12_R_0	BY926032
E_ET_F1mg_05D01_F_0	BY917723
E_ET_F1mg_05D01_R_0	BY926033
E_ET_F1mg_05D02_F_0	BY917724
E_ET_F1mg_05D02_R_0	BY926034
E_ET_F1mg_05D03_F_0	BY917725
E_ET_F1mg_05D03_R_0	BY926035
E_ET_F1mg_05D05_F_0	BY917726
E_ET_F1mg_05D05_R_0	BY926036
E_ET_F1mg_05D06_F_0	BY917727
E_ET_F1mg_05D06_R_0	BY926037
E_ET_F1mg_05D08_F_0	BY917728
E_ET_F1mg_05D08_R_0	BY926038
E_ET_F1mg_05D10_F_0	BY917729
E_ET_F1mg_05D10_R_0	BY926039
E_ET_F1mg_05D11_F_0	BY917730
E_ET_F1mg_05D11_R_0	BY926040
E_ET_F1mg_05E01_R_0	BY926041
E_ET_F1mg_05E03_F_0	BY917731
E_ET_F1mg_05E03_R_0	BY926042
E_ET_F1mg_05E04_F_0	BY917732
E_ET_F1mg_05E04_R_0	BY926043
E_ET_F1mg_05E05_F_0	BY917733
E_ET_F1mg_05E05_R_0	BY926044
E_ET_F1mg_05E07_F_0	BY917734
E_ET_F1mg_05E07_R_0	BY926045
E_ET_F1mg_05E08_F_0	BY917735
E_ET_F1mg_05E08_R_0	BY926046
E_ET_F1mg_05E09_F_0	BY917736
E_ET_F1mg_05E09_R_0	BY926047
E_ET_F1mg_05E12_F_0	BY917737
E_ET_F1mg_05E12_R_0	BY926048
E_ET_F1mg_05F01_F_0	BY917738
E_ET_F1mg_05F01_R_0	BY926049
E_ET_F1mg_05F02_F_0	BY917739
E_ET_F1mg_05F02_R_0	BY926050
E_ET_F1mg_05F06_F_0	BY917740

E_ET_F1mg_05F06_R_0	BY926051
E_ET_F1mg_05F08_F_0	BY917741
E_ET_F1mg_05F08_R_0	BY926052
E_ET_F1mg_05F10_F_0	BY917742
E_ET_F1mg_05F10_R_0	BY926053
E_ET_F1mg_05F11_F_0	BY917743
E_ET_F1mg_05F11_R_0	BY926054
E_ET_F1mg_05F12_F_0	BY917744
E_ET_F1mg_05G02_F_0	BY917745
E_ET_F1mg_05G02_R_0	BY926055
E_ET_F1mg_05G03_F_0	BY917746
E_ET_F1mg_05G03_R_0	BY926056
E_ET_F1mg_05G04_F_0	BY917747
E_ET_F1mg_05G04_R_0	BY926057
E_ET_F1mg_05G05_F_0	BY917748
E_ET_F1mg_05G05_R_0	BY926058
E_ET_F1mg_05G06_F_0	BY917749
E_ET_F1mg_05G06_R_0	BY926059
E_ET_F1mg_05G09_F_0	BY917750
E_ET_F1mg_05G09_R_0	BY926060
E_ET_F1mg_05G10_F_0	BY917751
E_ET_F1mg_05G10_R_0	BY926061
E_ET_F1mg_05G11_F_0	BY917752
E_ET_F1mg_05G11_R_0	BY926062
E_ET_F1mg_05G12_F_0	BY917753
E_ET_F1mg_05G12_R_0	BY926063
E_ET_F1mg_05H01_F_0	BY917754
E_ET_F1mg_05H01_R_0	BY926064
E_ET_F1mg_05H03_F_0	BY917755
E_ET_F1mg_05H03_R_0	BY926065
E_ET_F1mg_05H04_F_0	BY917756
E_ET_F1mg_05H04_R_0	BY926066
E_ET_F1mg_05H05_F_0	BY917757
E_ET_F1mg_05H05_R_0	BY926067
E_ET_F1mg_05H09_F_0	BY917758
E_ET_F1mg_05H09_R_0	BY926068
E_ET_F1mg_05H12_F_0	BY917759
E_ET_F1mg_05H12_R_0	BY926069
E_ET_F1mg_06A01_F_0	BY917760
E_ET_F1mg_06A01_R_0	BY926070
E_ET_F1mg_06A02_F_0	BY917761
E_ET_F1mg_06A02_R_0	BY926071
E_ET_F1mg_06A03_F_0	BY917762
E_ET_F1mg_06A03_R_0	BY926072
E_ET_F1mg_06A04_F_0	BY917763
E_ET_F1mg_06A04_R_0	BY926073
E_ET_F1mg_06A05_F_0	BY917764
E_ET_F1mg_06A05_R_0	BY926074
E_ET_F1mg_06A07_F_0	BY917765
E_ET_F1mg_06A07_R_0	BY926075
E_ET_F1mg_06A08_F_0	BY917766
E_ET_F1mg_06A08_R_0	BY926076
E_ET_F1mg_06A09_F_0	BY917767
E_ET_F1mg_06A10_F_0	BY917768

E_ET_F1mg_06A10_R_0	BY926077
E_ET_F1mg_06A11_F_0	BY917769
E_ET_F1mg_06A11_R_0	BY926078
E_ET_F1mg_06A12_F_0	BY917770
E_ET_F1mg_06A12_R_0	BY926079
E_ET_F1mg_06B01_F_0	BY917771
E_ET_F1mg_06B01_R_0	BY926080
E_ET_F1mg_06B02_F_0	BY917772
E_ET_F1mg_06B02_R_0	BY926081
E_ET_F1mg_06B04_F_0	BY917773
E_ET_F1mg_06B04_R_0	BY926082
E_ET_F1mg_06B05_F_0	BY917774
E_ET_F1mg_06B05_R_0	BY926083
E_ET_F1mg_06B08_F_0	BY917775
E_ET_F1mg_06B08_R_0	BY926084
E_ET_F1mg_06B09_F_0	BY917776
E_ET_F1mg_06B09_R_0	BY926085
E_ET_F1mg_06B12_R_0	BY926086
E_ET_F1mg_06C01_F_0	BY917777
E_ET_F1mg_06C01_R_0	BY926087
E_ET_F1mg_06C02_F_0	BY917778
E_ET_F1mg_06C02_R_0	BY926088
E_ET_F1mg_06C03_R_0	BY926089
E_ET_F1mg_06C04_F_0	BY917779
E_ET_F1mg_06C04_R_0	BY926090
E_ET_F1mg_06C05_F_0	BY917780
E_ET_F1mg_06C05_R_0	BY926091
E_ET_F1mg_06C09_F_0	BY917781
E_ET_F1mg_06C09_R_0	BY926092
E_ET_F1mg_06C10_F_0	BY917782
E_ET_F1mg_06C10_R_0	BY926093
E_ET_F1mg_06C11_F_0	BY917783
E_ET_F1mg_06C11_R_0	BY926094
E_ET_F1mg_06D01_F_0	BY917784
E_ET_F1mg_06D01_R_0	BY926095
E_ET_F1mg_06D03_F_0	BY917785
E_ET_F1mg_06D03_R_0	BY926096
E_ET_F1mg_06D05_F_0	BY917786
E_ET_F1mg_06D05_R_0	BY926097
E_ET_F1mg_06D11_F_0	BY917787
E_ET_F1mg_06D11_R_0	BY926098
E_ET_F1mg_06D12_F_0	BY917788
E_ET_F1mg_06D12_R_0	BY926099
E_ET_F1mg_06E03_F_0	BY917789
E_ET_F1mg_06E03_R_0	BY926100
E_ET_F1mg_06E05_F_0	BY917790
E_ET_F1mg_06E05_R_0	BY926101
E_ET_F1mg_06E06_F_0	BY917791
E_ET_F1mg_06E06_R_0	BY926102
E_ET_F1mg_06E10_F_0	BY917792
E_ET_F1mg_06E10_R_0	BY926103
E_ET_F1mg_06E11_R_0	BY926104
E_ET_F1mg_06F01_F_0	BY917793
E_ET_F1mg_06F01_R_0	BY926105

E_ET_F1mg_06F02_R_0	BY926106
E_ET_F1mg_06F03_F_0	BY917794
E_ET_F1mg_06F03_R_0	BY926107
E_ET_F1mg_06F04_F_0	BY917795
E_ET_F1mg_06F04_R_0	BY926108
E_ET_F1mg_06F06_F_0	BY917796
E_ET_F1mg_06F06_R_0	BY926109
E_ET_F1mg_06F07_R_0	BY926110
E_ET_F1mg_06F09_F_0	BY917797
E_ET_F1mg_06F09_R_0	BY926111
E_ET_F1mg_06F12_F_0	BY917798
E_ET_F1mg_06F12_R_0	BY926112
E_ET_F1mg_06G02_F_0	BY917799
E_ET_F1mg_06G02_R_0	BY926113
E_ET_F1mg_06G03_F_0	BY917800
E_ET_F1mg_06G03_R_0	BY926114
E_ET_F1mg_06G05_F_0	BY917801
E_ET_F1mg_06G05_R_0	BY926115
E_ET_F1mg_06G06_F_0	BY917802
E_ET_F1mg_06G06_R_0	BY926116
E_ET_F1mg_06G07_F_0	BY917803
E_ET_F1mg_06G07_R_0	BY926117
E_ET_F1mg_06G09_F_0	BY917804
E_ET_F1mg_06G09_R_0	BY926118
E_ET_F1mg_06G10_F_0	BY917805
E_ET_F1mg_06G10_R_0	BY926119
E_ET_F1mg_06G11_F_0	BY917806
E_ET_F1mg_06G11_R_0	BY926120
E_ET_F1mg_06G12_F_0	BY917807
E_ET_F1mg_06G12_R_0	BY926121
E_ET_F1mg_06H01_F_0	BY917808
E_ET_F1mg_06H01_R_0	BY926122
E_ET_F1mg_06H02_F_0	BY917809
E_ET_F1mg_06H04_F_0	BY917810
E_ET_F1mg_06H04_R_0	BY926123
E_ET_F1mg_06H05_R_0	BY926124
E_ET_F1mg_06H06_F_0	BY917811
E_ET_F1mg_06H06_R_0	BY926125
E_ET_F1mg_06H07_R_0	BY926126
E_ET_F1mg_06H08_R_0	BY926127
E_ET_F1mg_06H10_F_0	BY917812
E_ET_F1mg_06H10_R_0	BY926128
E_ET_F1mg_06H11_F_0	BY917813
E_ET_F1mg_06H11_R_0	BY926129
E_ET_F1mg_06H12_F_0	BY917814
E_ET_F1mg_07A01_F_0	BY917815
E_ET_F1mg_07A02_F_0	BY917816
E_ET_F1mg_07A03_F_0	BY917817
E_ET_F1mg_07A03_R_0	BY926130
E_ET_F1mg_07A06_F_0	BY917818
E_ET_F1mg_07A06_R_0	BY926131
E_ET_F1mg_07A07_F_0	BY917819
E_ET_F1mg_07A07_R_0	BY926132
E_ET_F1mg_07A08_F_0	BY917820

E_ET_F1mg_07A08_R_0	BY926133
E_ET_F1mg_07A09_F_0	BY917821
E_ET_F1mg_07A09_R_0	BY926134
E_ET_F1mg_07A10_F_0	BY917822
E_ET_F1mg_07A10_R_0	BY926135
E_ET_F1mg_07A11_F_0	BY917823
E_ET_F1mg_07A11_R_0	BY926136
E_ET_F1mg_07A12_F_0	BY917824
E_ET_F1mg_07A12_R_0	BY926137
E_ET_F1mg_07B02_R_0	BY926138
E_ET_F1mg_07B03_F_0	BY917825
E_ET_F1mg_07B05_F_0	BY917826
E_ET_F1mg_07B05_R_0	BY926139
E_ET_F1mg_07B06_F_0	BY917827
E_ET_F1mg_07B06_R_0	BY926140
E_ET_F1mg_07B09_F_0	BY917828
E_ET_F1mg_07B09_R_0	BY926141
E_ET_F1mg_07B11_F_0	BY917829
E_ET_F1mg_07B11_R_0	BY926142
E_ET_F1mg_07B12_F_0	BY917830
E_ET_F1mg_07B12_R_0	BY926143
E_ET_F1mg_07C01_F_0	BY917831
E_ET_F1mg_07C01_R_0	BY926144
E_ET_F1mg_07C03_F_0	BY917832
E_ET_F1mg_07C03_R_0	BY926145
E_ET_F1mg_07C07_F_0	BY917833
E_ET_F1mg_07C07_R_0	BY926146
E_ET_F1mg_07C08_F_0	BY917834
E_ET_F1mg_07C08_R_0	BY926147
E_ET_F1mg_07C09_F_0	BY917835
E_ET_F1mg_07C09_R_0	BY926148
E_ET_F1mg_07C11_F_0	BY917836
E_ET_F1mg_07C11_R_0	BY926149
E_ET_F1mg_07C12_F_0	BY917837
E_ET_F1mg_07C12_R_0	BY926150
E_ET_F1mg_07D01_F_0	BY917838
E_ET_F1mg_07D01_R_0	BY926151
E_ET_F1mg_07D02_F_0	BY917839
E_ET_F1mg_07D02_R_0	BY926152
E_ET_F1mg_07D03_R_0	BY926153
E_ET_F1mg_07D04_F_0	BY917840
E_ET_F1mg_07D04_R_0	BY926154
E_ET_F1mg_07D05_F_0	BY917841
E_ET_F1mg_07D05_R_0	BY926155
E_ET_F1mg_07D06_F_0	BY917842
E_ET_F1mg_07D06_R_0	BY926156
E_ET_F1mg_07D08_F_0	BY917843
E_ET_F1mg_07D08_R_0	BY926157
E_ET_F1mg_07D09_F_0	BY917844
E_ET_F1mg_07D09_R_0	BY926158
E_ET_F1mg_07D10_F_0	BY917845
E_ET_F1mg_07D10_R_0	BY926159
E_ET_F1mg_07D11_F_0	BY917846
E_ET_F1mg_07D11_R_0	BY926160

E_ET_F1mg_07D12_F_0	BY917847
E_ET_F1mg_07D12_R_0	BY926161
E_ET_F1mg_07E01_F_0	BY917848
E_ET_F1mg_07E01_R_0	BY926162
E_ET_F1mg_07E02_F_0	BY917849
E_ET_F1mg_07E02_R_0	BY926163
E_ET_F1mg_07E03_F_0	BY917850
E_ET_F1mg_07E03_R_0	BY926164
E_ET_F1mg_07E04_F_0	BY917851
E_ET_F1mg_07E04_R_0	BY926165
E_ET_F1mg_07E05_F_0	BY917852
E_ET_F1mg_07E05_R_0	BY926166
E_ET_F1mg_07E06_F_0	BY917853
E_ET_F1mg_07E06_R_0	BY926167
E_ET_F1mg_07E07_F_0	BY917854
E_ET_F1mg_07E07_R_0	BY926168
E_ET_F1mg_07E08_F_0	BY917855
E_ET_F1mg_07E10_F_0	BY917856
E_ET_F1mg_07E10_R_0	BY926169
E_ET_F1mg_07E11_F_0	BY917857
E_ET_F1mg_07E11_R_0	BY926170
E_ET_F1mg_07E12_F_0	BY917858
E_ET_F1mg_07E12_R_0	BY926171
E_ET_F1mg_07F01_F_0	BY917859
E_ET_F1mg_07F01_R_0	BY926172
E_ET_F1mg_07F02_F_0	BY917860
E_ET_F1mg_07F02_R_0	BY926173
E_ET_F1mg_07F03_F_0	BY917861
E_ET_F1mg_07F03_R_0	BY926174
E_ET_F1mg_07F04_F_0	BY917862
E_ET_F1mg_07F04_R_0	BY926175
E_ET_F1mg_07F05_F_0	BY917863
E_ET_F1mg_07F05_R_0	BY926176
E_ET_F1mg_07F07_F_0	BY917864
E_ET_F1mg_07F07_R_0	BY926177
E_ET_F1mg_07F08_F_0	BY917865
E_ET_F1mg_07F08_R_0	BY926178
E_ET_F1mg_07F09_F_0	BY917866
E_ET_F1mg_07F09_R_0	BY926179
E_ET_F1mg_07F10_F_0	BY917867
E_ET_F1mg_07F10_R_0	BY926180
E_ET_F1mg_07F11_F_0	BY917868
E_ET_F1mg_07F11_R_0	BY926181
E_ET_F1mg_07G02_F_0	BY917869
E_ET_F1mg_07G02_R_0	BY926182
E_ET_F1mg_07G03_F_0	BY917870
E_ET_F1mg_07G03_R_0	BY926183
E_ET_F1mg_07G04_F_0	BY917871
E_ET_F1mg_07G04_R_0	BY926184
E_ET_F1mg_07G06_F_0	BY917872
E_ET_F1mg_07G06_R_0	BY926185
E_ET_F1mg_07G07_R_0	BY926186
E_ET_F1mg_07G08_F_0	BY917873
E_ET_F1mg_07G08_R_0	BY926187

E_ET_F1mg_07G09_F_0	BY917874
E_ET_F1mg_07G09_R_0	BY926188
E_ET_F1mg_07G10_F_0	BY917875
E_ET_F1mg_07G10_R_0	BY926189
E_ET_F1mg_07G11_F_0	BY917876
E_ET_F1mg_07G11_R_0	BY926190
E_ET_F1mg_07G12_F_0	BY917877
E_ET_F1mg_07G12_R_0	BY926191
E_ET_F1mg_07H02_F_0	BY917878
E_ET_F1mg_07H02_R_0	BY926192
E_ET_F1mg_07H03_F_0	BY917879
E_ET_F1mg_07H03_R_0	BY926193
E_ET_F1mg_07H04_F_0	BY917880
E_ET_F1mg_07H04_R_0	BY926194
E_ET_F1mg_07H06_F_0	BY917881
E_ET_F1mg_07H06_R_0	BY926195
E_ET_F1mg_07H07_F_0	BY917882
E_ET_F1mg_07H07_R_0	BY926196
E_ET_F1mg_07H09_F_0	BY917883
E_ET_F1mg_07H09_R_0	BY926197
E_ET_F1mg_07H10_F_0	BY917884
E_ET_F1mg_07H10_R_0	BY926198
E_ET_F1mg_07H12_F_0	BY917885
E_ET_F1mg_07H12_R_0	BY926199
E_ET_F1mg_08A01_F_0	BY917886
E_ET_F1mg_08A03_R_0	BY926200
E_ET_F1mg_08A04_F_0	BY917887
E_ET_F1mg_08A04_R_0	BY926201
E_ET_F1mg_08A05_F_0	BY917888
E_ET_F1mg_08A05_R_0	BY926202
E_ET_F1mg_08A06_F_0	BY917889
E_ET_F1mg_08A06_R_0	BY926203
E_ET_F1mg_08A08_R_0	BY926204
E_ET_F1mg_08A09_F_0	BY917890
E_ET_F1mg_08A09_R_0	BY926205
E_ET_F1mg_08A11_F_0	BY917891
E_ET_F1mg_08A11_R_0	BY926206
E_ET_F1mg_08A12_F_0	BY917892
E_ET_F1mg_08A12_R_0	BY926207
E_ET_F1mg_08B01_R_0	BY926208
E_ET_F1mg_08B03_F_0	BY917893
E_ET_F1mg_08B03_R_0	BY926209
E_ET_F1mg_08B04_F_0	BY917894
E_ET_F1mg_08B04_R_0	BY926210
E_ET_F1mg_08B06_F_0	BY917895
E_ET_F1mg_08B06_R_0	BY926211
E_ET_F1mg_08B09_F_0	BY917896
E_ET_F1mg_08B09_R_0	BY926212
E_ET_F1mg_08B10_F_0	BY917897
E_ET_F1mg_08B10_R_0	BY926213
E_ET_F1mg_08B12_F_0	BY917898
E_ET_F1mg_08B12_R_0	BY926214
E_ET_F1mg_08C01_F_0	BY917899
E_ET_F1mg_08C01_R_0	BY926215

E_ET_F1mg_08C02_F_0	BY917900
E_ET_F1mg_08C02_R_0	BY926216
E_ET_F1mg_08C03_F_0	BY917901
E_ET_F1mg_08C03_R_0	BY926217
E_ET_F1mg_08C04_F_0	BY917902
E_ET_F1mg_08C04_R_0	BY926218
E_ET_F1mg_08C05_F_0	BY917903
E_ET_F1mg_08C05_R_0	BY926219
E_ET_F1mg_08C06_F_0	BY917904
E_ET_F1mg_08C08_R_0	BY926220
E_ET_F1mg_08C09_F_0	BY917905
E_ET_F1mg_08C09_R_0	BY926221
E_ET_F1mg_08C10_R_0	BY926222
E_ET_F1mg_08C11_F_0	BY917906
E_ET_F1mg_08C11_R_0	BY926223
E_ET_F1mg_08C12_F_0	BY917907
E_ET_F1mg_08C12_R_0	BY926224
E_ET_F1mg_08D02_F_0	BY917908
E_ET_F1mg_08D02_R_0	BY926225
E_ET_F1mg_08D03_F_0	BY917909
E_ET_F1mg_08D03_R_0	BY926226
E_ET_F1mg_08D04_F_0	BY917910
E_ET_F1mg_08D04_R_0	BY926227
E_ET_F1mg_08D06_F_0	BY917911
E_ET_F1mg_08D06_R_0	BY926228
E_ET_F1mg_08D08_F_0	BY917912
E_ET_F1mg_08D09_F_0	BY917913
E_ET_F1mg_08D09_R_0	BY926229
E_ET_F1mg_08D10_R_0	BY926230
E_ET_F1mg_08D11_F_0	BY917914
E_ET_F1mg_08D11_R_0	BY926231
E_ET_F1mg_08D12_F_0	BY917915
E_ET_F1mg_08E02_F_0	BY917916
E_ET_F1mg_08E02_R_0	BY926232
E_ET_F1mg_08E03_F_0	BY917917
E_ET_F1mg_08E03_R_0	BY926233
E_ET_F1mg_08E05_F_0	BY917918
E_ET_F1mg_08E05_R_0	BY926234
E_ET_F1mg_08E08_F_0	BY917919
E_ET_F1mg_08E09_F_0	BY917920
E_ET_F1mg_08E09_R_0	BY926235
E_ET_F1mg_08E11_F_0	BY917921
E_ET_F1mg_08E11_R_0	BY926236
E_ET_F1mg_08E12_F_0	BY917922
E_ET_F1mg_08E12_R_0	BY926237
E_ET_F1mg_08F02_R_0	BY926238
E_ET_F1mg_08F03_F_0	BY917923
E_ET_F1mg_08F04_F_0	BY917924
E_ET_F1mg_08F04_R_0	BY926239
E_ET_F1mg_08F06_F_0	BY917925
E_ET_F1mg_08F06_R_0	BY926240
E_ET_F1mg_08F08_F_0	BY917926
E_ET_F1mg_08F08_R_0	BY926241
E_ET_F1mg_08F09_F_0	BY917927

E_ET_F1mg_08F10_F_0	BY917928
E_ET_F1mg_08F10_R_0	BY926242
E_ET_F1mg_08F11_F_0	BY917929
E_ET_F1mg_08F11_R_0	BY926243
E_ET_F1mg_08F12_F_0	BY917930
E_ET_F1mg_08F12_R_0	BY926244
E_ET_F1mg_08G01_F_0	BY917931
E_ET_F1mg_08G01_R_0	BY926245
E_ET_F1mg_08G02_F_0	BY917932
E_ET_F1mg_08G02_R_0	BY926246
E_ET_F1mg_08G03_F_0	BY917933
E_ET_F1mg_08G03_R_0	BY926247
E_ET_F1mg_08G04_F_0	BY917934
E_ET_F1mg_08G04_R_0	BY926248
E_ET_F1mg_08G05_R_0	BY926249
E_ET_F1mg_08G06_F_0	BY917935
E_ET_F1mg_08G06_R_0	BY926250
E_ET_F1mg_08G08_F_0	BY917936
E_ET_F1mg_08G08_R_0	BY926251
E_ET_F1mg_08G09_F_0	BY917937
E_ET_F1mg_08G09_R_0	BY926252
E_ET_F1mg_08G10_F_0	BY917938
E_ET_F1mg_08G10_R_0	BY926253
E_ET_F1mg_08G12_F_0	BY917939
E_ET_F1mg_08G12_R_0	BY926254
E_ET_F1mg_08H01_F_0	BY917940
E_ET_F1mg_08H01_R_0	BY926255
E_ET_F1mg_08H02_F_0	BY917941
E_ET_F1mg_08H02_R_0	BY926256
E_ET_F1mg_08H05_F_0	BY917942
E_ET_F1mg_08H05_R_0	BY926257
E_ET_F1mg_08H07_F_0	BY917943
E_ET_F1mg_08H07_R_0	BY926258
E_ET_F1mg_08H08_F_0	BY917944
E_ET_F1mg_08H08_R_0	BY926259
E_ET_F1mg_08H09_F_0	BY917945
E_ET_F1mg_08H09_R_0	BY926260
E_ET_F1mg_08H10_F_0	BY917946
E_ET_F1mg_08H10_R_0	BY926261
E_ET_F1mg_08H11_F_0	BY917947
E_ET_F1mg_08H11_R_0	BY926262
E_ET_F1mg_09A03_F_0	BY917948
E_ET_F1mg_09A03_R_0	BY926263
E_ET_F1mg_09A04_F_0	BY917949
E_ET_F1mg_09A04_R_0	BY926264
E_ET_F1mg_09A06_F_0	BY917950
E_ET_F1mg_09A06_R_0	BY926265
E_ET_F1mg_09A08_F_0	BY917951
E_ET_F1mg_09A08_R_0	BY926266
E_ET_F1mg_09A09_F_0	BY917952
E_ET_F1mg_09A09_R_0	BY926267
E_ET_F1mg_09A12_F_0	BY917953
E_ET_F1mg_09A12_R_0	BY926268
E_ET_F1mg_09B01_F_0	BY917954

E_ET_F1mg_09B01_R_0	BY926269
E_ET_F1mg_09B02_F_0	BY917955
E_ET_F1mg_09B02_R_0	BY926270
E_ET_F1mg_09B03_F_0	BY917956
E_ET_F1mg_09B03_R_0	BY926271
E_ET_F1mg_09B04_F_0	BY917957
E_ET_F1mg_09B04_R_0	BY926272
E_ET_F1mg_09B05_F_0	BY917958
E_ET_F1mg_09B05_R_0	BY926273
E_ET_F1mg_09B07_F_0	BY917959
E_ET_F1mg_09B07_R_0	BY926274
E_ET_F1mg_09B08_F_0	BY917960
E_ET_F1mg_09B08_R_0	BY926275
E_ET_F1mg_09C02_F_0	BY917961
E_ET_F1mg_09C02_R_0	BY926276
E_ET_F1mg_09C04_F_0	BY917962
E_ET_F1mg_09C04_R_0	BY926277
E_ET_F1mg_09C05_F_0	BY917963
E_ET_F1mg_09C05_R_0	BY926278
E_ET_F1mg_09C06_F_0	BY917964
E_ET_F1mg_09C06_R_0	BY926279
E_ET_F1mg_09C07_F_0	BY917965
E_ET_F1mg_09C07_R_0	BY926280
E_ET_F1mg_09C08_F_0	BY917966
E_ET_F1mg_09C08_R_0	BY926281
E_ET_F1mg_09C09_F_0	BY917967
E_ET_F1mg_09C09_R_0	BY926282
E_ET_F1mg_09D01_F_0	BY917968
E_ET_F1mg_09D01_R_0	BY926283
E_ET_F1mg_09D02_F_0	BY917969
E_ET_F1mg_09D02_R_0	BY926284
E_ET_F1mg_09D04_F_0	BY917970
E_ET_F1mg_09D04_R_0	BY926285
E_ET_F1mg_09D05_F_0	BY917971
E_ET_F1mg_09D05_R_0	BY926286
E_ET_F1mg_09D09_F_0	BY917972
E_ET_F1mg_09D09_R_0	BY926287
E_ET_F1mg_09D10_F_0	BY917973
E_ET_F1mg_09D10_R_0	BY926288
E_ET_F1mg_09D11_F_0	BY917974
E_ET_F1mg_09D12_F_0	BY917975
E_ET_F1mg_09E01_F_0	BY917976
E_ET_F1mg_09E01_R_0	BY926289
E_ET_F1mg_09E04_F_0	BY917977
E_ET_F1mg_09E04_R_0	BY926290
E_ET_F1mg_09E06_F_0	BY917978
E_ET_F1mg_09E06_R_0	BY926291
E_ET_F1mg_09E07_F_0	BY917979
E_ET_F1mg_09E07_R_0	BY926292
E_ET_F1mg_09E08_F_0	BY917980
E_ET_F1mg_09E08_R_0	BY926293
E_ET_F1mg_09E09_F_0	BY917981
E_ET_F1mg_09E09_R_0	BY926294
E_ET_F1mg_09E10_F_0	BY917982

E_ET_F1mg_09E10_R_0	BY926295
E_ET_F1mg_09E11_F_0	BY917983
E_ET_F1mg_09E11_R_0	BY926296
E_ET_F1mg_09F01_F_0	BY917984
E_ET_F1mg_09F01_R_0	BY926297
E_ET_F1mg_09F02_F_0	BY917985
E_ET_F1mg_09F02_R_0	BY926298
E_ET_F1mg_09F03_R_0	BY926299
E_ET_F1mg_09F04_F_0	BY917986
E_ET_F1mg_09F04_R_0	BY926300
E_ET_F1mg_09F09_F_0	BY917987
E_ET_F1mg_09F09_R_0	BY926301
E_ET_F1mg_09F10_R_0	BY926302
E_ET_F1mg_09F11_F_0	BY917988
E_ET_F1mg_09F11_R_0	BY926303
E_ET_F1mg_09F12_F_0	BY917989
E_ET_F1mg_09G01_F_0	BY917990
E_ET_F1mg_09G01_R_0	BY926304
E_ET_F1mg_09G02_F_0	BY917991
E_ET_F1mg_09G02_R_0	BY926305
E_ET_F1mg_09G03_F_0	BY917992
E_ET_F1mg_09G03_R_0	BY926306
E_ET_F1mg_09G04_F_0	BY917993
E_ET_F1mg_09G04_R_0	BY926307
E_ET_F1mg_09G06_F_0	BY917994
E_ET_F1mg_09G06_R_0	BY926308
E_ET_F1mg_09G09_F_0	BY917995
E_ET_F1mg_09G09_R_0	BY926309
E_ET_F1mg_09G10_F_0	BY917996
E_ET_F1mg_09G10_R_0	BY926310
E_ET_F1mg_09G11_F_0	BY917997
E_ET_F1mg_09G11_R_0	BY926311
E_ET_F1mg_09G12_F_0	BY917998
E_ET_F1mg_09G12_R_0	BY926312
E_ET_F1mg_09H01_F_0	BY917999
E_ET_F1mg_09H01_R_0	BY926313
E_ET_F1mg_09H03_F_0	BY918000
E_ET_F1mg_09H03_R_0	BY926314
E_ET_F1mg_09H04_F_0	BY918001
E_ET_F1mg_09H05_F_0	BY918002
E_ET_F1mg_09H05_R_0	BY926315
E_ET_F1mg_09H08_F_0	BY918003
E_ET_F1mg_09H08_R_0	BY926316
E_ET_F1mg_09H10_F_0	BY918004
E_ET_F1mg_09H10_R_0	BY926317
E_ET_F1mg_09H12_F_0	BY918005
E_ET_F1mg_09H12_R_0	BY926318
E_ET_F1mg_10A03_F_0	BY918006
E_ET_F1mg_10A03_R_0	BY926319
E_ET_F1mg_10A05_F_0	BY918007
E_ET_F1mg_10A05_R_0	BY926320
E_ET_F1mg_10A06_F_0	BY918008
E_ET_F1mg_10A06_R_0	BY926321
E_ET_F1mg_10A07_F_0	BY918009

E_ET_F1mg_10A07_R_0	BY926322
E_ET_F1mg_10A08_F_0	BY918010
E_ET_F1mg_10A08_R_0	BY926323
E_ET_F1mg_10A10_F_0	BY918011
E_ET_F1mg_10A10_R_0	BY926324
E_ET_F1mg_10A11_F_0	BY918012
E_ET_F1mg_10A11_R_0	BY926325
E_ET_F1mg_10A12_F_0	BY918013
E_ET_F1mg_10A12_R_0	BY926326
E_ET_F1mg_10B01_F_0	BY918014
E_ET_F1mg_10B01_R_0	BY926327
E_ET_F1mg_10B02_F_0	BY918015
E_ET_F1mg_10B04_F_0	BY918016
E_ET_F1mg_10B04_R_0	BY926328
E_ET_F1mg_10B05_F_0	BY918017
E_ET_F1mg_10B05_R_0	BY926329
E_ET_F1mg_10B06_F_0	BY918018
E_ET_F1mg_10B06_R_0	BY926330
E_ET_F1mg_10B07_F_0	BY918019
E_ET_F1mg_10B07_R_0	BY926331
E_ET_F1mg_10B09_F_0	BY918020
E_ET_F1mg_10B09_R_0	BY926332
E_ET_F1mg_10B10_F_0	BY918021
E_ET_F1mg_10B10_R_0	BY926333
E_ET_F1mg_10C02_F_0	BY918022
E_ET_F1mg_10C02_R_0	BY926334
E_ET_F1mg_10C03_F_0	BY918023
E_ET_F1mg_10C03_R_0	BY926335
E_ET_F1mg_10C05_F_0	BY918024
E_ET_F1mg_10C05_R_0	BY926336
E_ET_F1mg_10C07_F_0	BY918025
E_ET_F1mg_10C07_R_0	BY926337
E_ET_F1mg_10C10_F_0	BY918026
E_ET_F1mg_10C10_R_0	BY926338
E_ET_F1mg_10C11_F_0	BY918027
E_ET_F1mg_10C12_F_0	BY918028
E_ET_F1mg_10D01_F_0	BY918029
E_ET_F1mg_10D01_R_0	BY926339
E_ET_F1mg_10D02_F_0	BY918030
E_ET_F1mg_10D02_R_0	BY926340
E_ET_F1mg_10D06_F_0	BY918031
E_ET_F1mg_10D06_R_0	BY926341
E_ET_F1mg_10D07_F_0	BY918032
E_ET_F1mg_10D07_R_0	BY926342
E_ET_F1mg_10D08_R_0	BY926343
E_ET_F1mg_10D09_F_0	BY918033
E_ET_F1mg_10D09_R_0	BY926344
E_ET_F1mg_10D12_F_0	BY918034
E_ET_F1mg_10D12_R_0	BY926345
E_ET_F1mg_10E01_F_0	BY918035
E_ET_F1mg_10E01_R_0	BY926346
E_ET_F1mg_10E03_F_0	BY918036
E_ET_F1mg_10E03_R_0	BY926347
E_ET_F1mg_10E04_F_0	BY918037

E_ET_F1mg_10E05_F_0	BY918038
E_ET_F1mg_10E05_R_0	BY926348
E_ET_F1mg_10E06_F_0	BY918039
E_ET_F1mg_10E06_R_0	BY926349
E_ET_F1mg_10E08_F_0	BY918040
E_ET_F1mg_10E08_R_0	BY926350
E_ET_F1mg_10E09_F_0	BY918041
E_ET_F1mg_10E09_R_0	BY926351
E_ET_F1mg_10E10_F_0	BY918042
E_ET_F1mg_10E10_R_0	BY926352
E_ET_F1mg_10E11_F_0	BY918043
E_ET_F1mg_10E11_R_0	BY926353
E_ET_F1mg_10E12_F_0	BY918044
E_ET_F1mg_10E12_R_0	BY926354
E_ET_F1mg_10F02_F_0	BY918045
E_ET_F1mg_10F02_R_0	BY926355
E_ET_F1mg_10F06_R_0	BY926356
E_ET_F1mg_10F07_F_0	BY918046
E_ET_F1mg_10F07_R_0	BY926357
E_ET_F1mg_10F08_F_0	BY918047
E_ET_F1mg_10F08_R_0	BY926358
E_ET_F1mg_10F09_F_0	BY918048
E_ET_F1mg_10F09_R_0	BY926359
E_ET_F1mg_10F10_F_0	BY918049
E_ET_F1mg_10F10_R_0	BY926360
E_ET_F1mg_10F11_F_0	BY918050
E_ET_F1mg_10F11_R_0	BY926361
E_ET_F1mg_10G02_F_0	BY918051
E_ET_F1mg_10G02_R_0	BY926362
E_ET_F1mg_10G04_F_0	BY918052
E_ET_F1mg_10G04_R_0	BY926363
E_ET_F1mg_10G06_F_0	BY918053
E_ET_F1mg_10G08_F_0	BY918054
E_ET_F1mg_10G08_R_0	BY926364
E_ET_F1mg_10G09_F_0	BY918055
E_ET_F1mg_10G09_R_0	BY926365
E_ET_F1mg_10H02_F_0	BY918056
E_ET_F1mg_10H02_R_0	BY926366
E_ET_F1mg_10H07_F_0	BY918057
E_ET_F1mg_10H07_R_0	BY926367
E_ET_F1mg_10H08_F_0	BY918058
E_ET_F1mg_10H08_R_0	BY926368
E_ET_F1mg_10H09_F_0	BY918059
E_ET_F1mg_10H09_R_0	BY926369
E_ET_F1mg_10H10_F_0	BY918060
E_ET_F1mg_10H10_R_0	BY926370
E_ET_F1mg_10H11_R_0	BY926371
E_ET_F1mg_10H12_F_0	BY918061
E_ET_F1mg_10H12_R_0	BY926372
E_ET_F1mg_11A01_F_0	BY918062
E_ET_F1mg_11A01_R_0	BY926373
E_ET_F1mg_11A02_F_0	BY918063
E_ET_F1mg_11A03_F_0	BY918064
E_ET_F1mg_11A03_R_0	BY926374

E_ET_F1mg_11A06_F_0	BY918065
E_ET_F1mg_11A06_R_0	BY926375
E_ET_F1mg_11A09_F_0	BY918066
E_ET_F1mg_11A09_R_0	BY926376
E_ET_F1mg_11A10_F_0	BY918067
E_ET_F1mg_11A10_R_0	BY926377
E_ET_F1mg_11B03_F_0	BY918068
E_ET_F1mg_11B03_R_0	BY926378
E_ET_F1mg_11B05_F_0	BY918069
E_ET_F1mg_11B05_R_0	BY926379
E_ET_F1mg_11B08_F_0	BY918070
E_ET_F1mg_11B08_R_0	BY926380
E_ET_F1mg_11B09_F_0	BY918071
E_ET_F1mg_11B09_R_0	BY926381
E_ET_F1mg_11B12_F_0	BY918072
E_ET_F1mg_11C01_F_0	BY918073
E_ET_F1mg_11C01_R_0	BY926382
E_ET_F1mg_11C02_F_0	BY918074
E_ET_F1mg_11C03_F_0	BY918075
E_ET_F1mg_11C03_R_0	BY926383
E_ET_F1mg_11C05_F_0	BY918076
E_ET_F1mg_11C05_R_0	BY926384
E_ET_F1mg_11C06_F_0	BY918077
E_ET_F1mg_11C06_R_0	BY926385
E_ET_F1mg_11C07_F_0	BY918078
E_ET_F1mg_11C07_R_0	BY926386
E_ET_F1mg_11C08_F_0	BY918079
E_ET_F1mg_11C08_R_0	BY926387
E_ET_F1mg_11C10_F_0	BY918080
E_ET_F1mg_11C10_R_0	BY926388
E_ET_F1mg_11C12_F_0	BY918081
E_ET_F1mg_11C12_R_0	BY926389
E_ET_F1mg_11D01_F_0	BY918082
E_ET_F1mg_11D01_R_0	BY926390
E_ET_F1mg_11D03_F_0	BY918083
E_ET_F1mg_11D03_R_0	BY926391
E_ET_F1mg_11D04_F_0	BY918084
E_ET_F1mg_11D04_R_0	BY926392
E_ET_F1mg_11D06_F_0	BY918085
E_ET_F1mg_11D06_R_0	BY926393
E_ET_F1mg_11D07_F_0	BY918086
E_ET_F1mg_11D07_R_0	BY926394
E_ET_F1mg_11D09_F_0	BY918087
E_ET_F1mg_11D09_R_0	BY926395
E_ET_F1mg_11D10_F_0	BY918088
E_ET_F1mg_11D10_R_0	BY926396
E_ET_F1mg_11E01_F_0	BY918089
E_ET_F1mg_11E01_R_0	BY926397
E_ET_F1mg_11E06_F_0	BY918090
E_ET_F1mg_11E06_R_0	BY926398
E_ET_F1mg_11E07_F_0	BY918091
E_ET_F1mg_11E07_R_0	BY926399
E_ET_F1mg_11E09_F_0	BY918092
E_ET_F1mg_11E09_R_0	BY926400

E_ET_F1mg_11E10_F_0	BY918093
E_ET_F1mg_11E10_R_0	BY926401
E_ET_F1mg_11E11_F_0	BY918094
E_ET_F1mg_11E11_R_0	BY926402
E_ET_F1mg_11F02_F_0	BY918095
E_ET_F1mg_11F02_R_0	BY926403
E_ET_F1mg_11F03_F_0	BY918096
E_ET_F1mg_11F03_R_0	BY926404
E_ET_F1mg_11F05_F_0	BY918097
E_ET_F1mg_11F05_R_0	BY926405
E_ET_F1mg_11F07_F_0	BY918098
E_ET_F1mg_11F07_R_0	BY926406
E_ET_F1mg_11F08_F_0	BY918099
E_ET_F1mg_11F08_R_0	BY926407
E_ET_F1mg_11F11_F_0	BY918100
E_ET_F1mg_11F11_R_0	BY926408
E_ET_F1mg_11G01_F_0	BY918101
E_ET_F1mg_11G01_R_0	BY926409
E_ET_F1mg_11G07_F_0	BY918102
E_ET_F1mg_11G07_R_0	BY926410
E_ET_F1mg_11G08_F_0	BY918103
E_ET_F1mg_11G08_R_0	BY926411
E_ET_F1mg_11G09_F_0	BY918104
E_ET_F1mg_11G09_R_0	BY926412
E_ET_F1mg_11G10_F_0	BY918105
E_ET_F1mg_11G10_R_0	BY926413
E_ET_F1mg_11G11_F_0	BY918106
E_ET_F1mg_11H02_F_0	BY918107
E_ET_F1mg_11H02_R_0	BY926414
E_ET_F1mg_11H03_F_0	BY918108
E_ET_F1mg_11H03_R_0	BY926415
E_ET_F1mg_11H04_F_0	BY918109
E_ET_F1mg_11H04_R_0	BY926416
E_ET_F1mg_11H05_F_0	BY918110
E_ET_F1mg_11H05_R_0	BY926417
E_ET_F1mg_11H06_F_0	BY918111
E_ET_F1mg_11H06_R_0	BY926418
E_ET_F1mg_11H07_F_0	BY918112
E_ET_F1mg_11H07_R_0	BY926419
E_ET_F1mg_11H09_F_0	BY918113
E_ET_F1mg_11H09_R_0	BY926420
E_ET_F1mg_11H12_F_0	BY918114
E_ET_F1mg_12A02_F_0	BY918115
E_ET_F1mg_12A02_R_0	BY926421
E_ET_F1mg_12A03_F_0	BY918116
E_ET_F1mg_12A03_R_0	BY926422
E_ET_F1mg_12A04_F_0	BY918117
E_ET_F1mg_12A06_F_0	BY918118
E_ET_F1mg_12A06_R_0	BY926423
E_ET_F1mg_12A07_F_0	BY918119
E_ET_F1mg_12A07_R_0	BY926424
E_ET_F1mg_12A08_F_0	BY918120
E_ET_F1mg_12A08_R_0	BY926425
E_ET_F1mg_12A09_F_0	BY918121

E_ET_F1mg_12A09_R_0	BY926426
E_ET_F1mg_12A10_F_0	BY918122
E_ET_F1mg_12A10_R_0	BY926427
E_ET_F1mg_12A11_F_0	BY918123
E_ET_F1mg_12A11_R_0	BY926428
E_ET_F1mg_12A12_F_0	BY918124
E_ET_F1mg_12A12_R_0	BY926429
E_ET_F1mg_12B04_F_0	BY918125
E_ET_F1mg_12B04_R_0	BY926430
E_ET_F1mg_12B06_F_0	BY918126
E_ET_F1mg_12B06_R_0	BY926431
E_ET_F1mg_12B07_F_0	BY918127
E_ET_F1mg_12B07_R_0	BY926432
E_ET_F1mg_12B08_F_0	BY918128
E_ET_F1mg_12B08_R_0	BY926433
E_ET_F1mg_12B09_F_0	BY918129
E_ET_F1mg_12B09_R_0	BY926434
E_ET_F1mg_12B11_F_0	BY918130
E_ET_F1mg_12B11_R_0	BY926435
E_ET_F1mg_12C01_F_0	BY918131
E_ET_F1mg_12C01_R_0	BY926436
E_ET_F1mg_12C04_R_0	BY926437
E_ET_F1mg_12C05_F_0	BY918132
E_ET_F1mg_12C06_F_0	BY918133
E_ET_F1mg_12C06_R_0	BY926438
E_ET_F1mg_12C08_F_0	BY918134
E_ET_F1mg_12C08_R_0	BY926439
E_ET_F1mg_12C09_F_0	BY918135
E_ET_F1mg_12C11_F_0	BY918136
E_ET_F1mg_12C11_R_0	BY926440
E_ET_F1mg_12D01_F_0	BY918137
E_ET_F1mg_12D01_R_0	BY926441
E_ET_F1mg_12D02_F_0	BY918138
E_ET_F1mg_12D04_F_0	BY918139
E_ET_F1mg_12D05_F_0	BY918140
E_ET_F1mg_12D05_R_0	BY926442
E_ET_F1mg_12D08_F_0	BY918141
E_ET_F1mg_12D08_R_0	BY926443
E_ET_F1mg_12D11_F_0	BY918142
E_ET_F1mg_12D11_R_0	BY926444
E_ET_F1mg_12D12_F_0	BY918143
E_ET_F1mg_12D12_R_0	BY926445
E_ET_F1mg_12E01_R_0	BY926446
E_ET_F1mg_12E02_F_0	BY918144
E_ET_F1mg_12E02_R_0	BY926447
E_ET_F1mg_12E04_F_0	BY918145
E_ET_F1mg_12E04_R_0	BY926448
E_ET_F1mg_12E05_F_0	BY918146
E_ET_F1mg_12E05_R_0	BY926449
E_ET_F1mg_12E06_F_0	BY918147
E_ET_F1mg_12E06_R_0	BY926450
E_ET_F1mg_12E10_F_0	BY918148
E_ET_F1mg_12E10_R_0	BY926451
E_ET_F1mg_12E11_F_0	BY918149

E_ET_F1mg_12E11_R_0	BY926452
E_ET_F1mg_12E12_F_0	BY918150
E_ET_F1mg_12E12_R_0	BY926453
E_ET_F1mg_12F02_F_0	BY918151
E_ET_F1mg_12F02_R_0	BY926454
E_ET_F1mg_12F05_F_0	BY918152
E_ET_F1mg_12F05_R_0	BY926455
E_ET_F1mg_12F06_F_0	BY918153
E_ET_F1mg_12F06_R_0	BY926456
E_ET_F1mg_12F07_F_0	BY918154
E_ET_F1mg_12F07_R_0	BY926457
E_ET_F1mg_12F08_R_0	BY926458
E_ET_F1mg_12F11_F_0	BY918155
E_ET_F1mg_12F11_R_0	BY926459
E_ET_F1mg_12G02_F_0	BY918156
E_ET_F1mg_12G02_R_0	BY926460
E_ET_F1mg_12G03_F_0	BY918157
E_ET_F1mg_12G03_R_0	BY926461
E_ET_F1mg_12G04_F_0	BY918158
E_ET_F1mg_12G04_R_0	BY926462
E_ET_F1mg_12G05_F_0	BY918159
E_ET_F1mg_12G05_R_0	BY926463
E_ET_F1mg_12G09_F_0	BY918160
E_ET_F1mg_12G09_R_0	BY926464
E_ET_F1mg_12G10_F_0	BY918161
E_ET_F1mg_12G10_R_0	BY926465
E_ET_F1mg_12G12_F_0	BY918162
E_ET_F1mg_12H01_F_0	BY918163
E_ET_F1mg_12H01_R_0	BY926466
E_ET_F1mg_12H03_F_0	BY918164
E_ET_F1mg_12H03_R_0	BY926467
E_ET_F1mg_12H04_F_0	BY918165
E_ET_F1mg_12H04_R_0	BY926468
E_ET_F1mg_12H05_F_0	BY918166
E_ET_F1mg_12H07_F_0	BY918167
E_ET_F1mg_12H07_R_0	BY926469
E_ET_F1mg_13A04_R_0	BY926470
E_ET_F1mg_13A11_F_0	BY918168
E_ET_F1mg_13A11_R_0	BY926471
E_ET_F1mg_13A12_F_0	BY918169
E_ET_F1mg_13A12_R_0	BY926472
E_ET_F1mg_13B01_R_0	BY926473
E_ET_F1mg_13B03_F_0	BY918170
E_ET_F1mg_13B03_R_0	BY926474
E_ET_F1mg_13B06_F_0	BY918171
E_ET_F1mg_13B06_R_0	BY926475
E_ET_F1mg_13B09_F_0	BY918172
E_ET_F1mg_13B09_R_0	BY926476
E_ET_F1mg_13B11_F_0	BY918173
E_ET_F1mg_13B11_R_0	BY926477
E_ET_F1mg_13B12_F_0	BY918174
E_ET_F1mg_13B12_R_0	BY926478
E_ET_F1mg_13C01_F_0	BY918175
E_ET_F1mg_13C01_R_0	BY926479

E_ET_F1mg_13C03_R_0	BY926480
E_ET_F1mg_13C04_F_0	BY918176
E_ET_F1mg_13C04_R_0	BY926481
E_ET_F1mg_13C07_F_0	BY918177
E_ET_F1mg_13C07_R_0	BY926482
E_ET_F1mg_13C11_F_0	BY918178
E_ET_F1mg_13C11_R_0	BY926483
E_ET_F1mg_13C12_F_0	BY918179
E_ET_F1mg_13C12_R_0	BY926484
E_ET_F1mg_13D01_F_0	BY918180
E_ET_F1mg_13D01_R_0	BY926485
E_ET_F1mg_13D03_F_0	BY918181
E_ET_F1mg_13D04_F_0	BY918182
E_ET_F1mg_13D04_R_0	BY926486
E_ET_F1mg_13D06_F_0	BY918183
E_ET_F1mg_13D06_R_0	BY926487
E_ET_F1mg_13D07_F_0	BY918184
E_ET_F1mg_13D07_R_0	BY926488
E_ET_F1mg_13D08_F_0	BY918185
E_ET_F1mg_13D08_R_0	BY926489
E_ET_F1mg_13D09_F_0	BY918186
E_ET_F1mg_13D09_R_0	BY926490
E_ET_F1mg_13D10_F_0	BY918187
E_ET_F1mg_13D10_R_0	BY926491
E_ET_F1mg_13E02_R_0	BY926492
E_ET_F1mg_13E03_F_0	BY918188
E_ET_F1mg_13E03_R_0	BY926493
E_ET_F1mg_13E06_F_0	BY918189
E_ET_F1mg_13E06_R_0	BY926494
E_ET_F1mg_13E08_F_0	BY918190
E_ET_F1mg_13E08_R_0	BY926495
E_ET_F1mg_13E09_F_0	BY918191
E_ET_F1mg_13E09_R_0	BY926496
E_ET_F1mg_13E11_F_0	BY918192
E_ET_F1mg_13E12_F_0	BY918193
E_ET_F1mg_13E12_R_0	BY926497
E_ET_F1mg_13F02_F_0	BY918194
E_ET_F1mg_13F02_R_0	BY926498
E_ET_F1mg_13F03_F_0	BY918195
E_ET_F1mg_13F03_R_0	BY926499
E_ET_F1mg_13F05_F_0	BY918196
E_ET_F1mg_13F05_R_0	BY926500
E_ET_F1mg_13F06_F_0	BY918197
E_ET_F1mg_13F06_R_0	BY926501
E_ET_F1mg_13F10_F_0	BY918198
E_ET_F1mg_13F10_R_0	BY926502
E_ET_F1mg_13F11_F_0	BY918199
E_ET_F1mg_13F11_R_0	BY926503
E_ET_F1mg_13G01_F_0	BY918200
E_ET_F1mg_13G01_R_0	BY926504
E_ET_F1mg_13G02_F_0	BY918201
E_ET_F1mg_13G02_R_0	BY926505
E_ET_F1mg_13G03_F_0	BY918202
E_ET_F1mg_13G03_R_0	BY926506

E_ET_F1mg_13G05_F_0	BY918203
E_ET_F1mg_13G05_R_0	BY926507
E_ET_F1mg_13G08_F_0	BY918204
E_ET_F1mg_13G08_R_0	BY926508
E_ET_F1mg_13G09_F_0	BY918205
E_ET_F1mg_13G09_R_0	BY926509
E_ET_F1mg_13G10_F_0	BY918206
E_ET_F1mg_13G12_F_0	BY918207
E_ET_F1mg_13G12_R_0	BY926510
E_ET_F1mg_13H02_F_0	BY918208
E_ET_F1mg_13H02_R_0	BY926511
E_ET_F1mg_13H04_F_0	BY918209
E_ET_F1mg_13H04_R_0	BY926512
E_ET_F1mg_13H05_F_0	BY918210
E_ET_F1mg_13H05_R_0	BY926513
E_ET_F1mg_13H09_F_0	BY918211
E_ET_F1mg_13H09_R_0	BY926514
E_ET_F1mg_13H12_F_0	BY918212
E_ET_F1mg_13H12_R_0	BY926515
E_ET_F1mg_14A01_F_0	BY918213
E_ET_F1mg_14A01_R_0	BY926516
E_ET_F1mg_14A03_F_0	BY918214
E_ET_F1mg_14A03_R_0	BY926517
E_ET_F1mg_14A05_F_0	BY918215
E_ET_F1mg_14A05_R_0	BY926518
E_ET_F1mg_14A08_F_0	BY918216
E_ET_F1mg_14A08_R_0	BY926519
E_ET_F1mg_14A09_F_0	BY918217
E_ET_F1mg_14A09_R_0	BY926520
E_ET_F1mg_14A10_R_0	BY926521
E_ET_F1mg_14A11_R_0	BY926522
E_ET_F1mg_14B02_F_0	BY918218
E_ET_F1mg_14B03_F_0	BY918219
E_ET_F1mg_14B03_R_0	BY926523
E_ET_F1mg_14B05_F_0	BY918220
E_ET_F1mg_14B05_R_0	BY926524
E_ET_F1mg_14B06_F_0	BY918221
E_ET_F1mg_14B06_R_0	BY926525
E_ET_F1mg_14B07_F_0	BY918222
E_ET_F1mg_14B08_F_0	BY918223
E_ET_F1mg_14B10_F_0	BY918224
E_ET_F1mg_14B10_R_0	BY926526
E_ET_F1mg_14B12_F_0	BY918225
E_ET_F1mg_14B12_R_0	BY926527
E_ET_F1mg_14C02_F_0	BY918226
E_ET_F1mg_14C02_R_0	BY926528
E_ET_F1mg_14C06_R_0	BY926529
E_ET_F1mg_14C07_F_0	BY918227
E_ET_F1mg_14C07_R_0	BY926530
E_ET_F1mg_14C09_F_0	BY918228
E_ET_F1mg_14C09_R_0	BY926531
E_ET_F1mg_14C10_R_0	BY926532
E_ET_F1mg_14C11_F_0	BY918229
E_ET_F1mg_14C11_R_0	BY926533

E_ET_F1mg_14D01_F_0	BY918230
E_ET_F1mg_14D02_F_0	BY918231
E_ET_F1mg_14D02_R_0	BY926534
E_ET_F1mg_14D03_F_0	BY918232
E_ET_F1mg_14D03_R_0	BY926535
E_ET_F1mg_14D07_F_0	BY918233
E_ET_F1mg_14D07_R_0	BY926536
E_ET_F1mg_14D08_F_0	BY918234
E_ET_F1mg_14D08_R_0	BY926537
E_ET_F1mg_14D09_F_0	BY918235
E_ET_F1mg_14D09_R_0	BY926538
E_ET_F1mg_14D10_F_0	BY918236
E_ET_F1mg_14D10_R_0	BY926539
E_ET_F1mg_14D11_F_0	BY918237
E_ET_F1mg_14D11_R_0	BY926540
E_ET_F1mg_14D12_F_0	BY918238
E_ET_F1mg_14D12_R_0	BY926541
E_ET_F1mg_14E01_F_0	BY918239
E_ET_F1mg_14E01_R_0	BY926542
E_ET_F1mg_14E02_R_0	BY926543
E_ET_F1mg_14E04_F_0	BY918240
E_ET_F1mg_14E04_R_0	BY926544
E_ET_F1mg_14E05_F_0	BY918241
E_ET_F1mg_14E05_R_0	BY926545
E_ET_F1mg_14E06_F_0	BY918242
E_ET_F1mg_14E06_R_0	BY926546
E_ET_F1mg_14E07_F_0	BY918243
E_ET_F1mg_14E07_R_0	BY926547
E_ET_F1mg_14E08_F_0	BY918244
E_ET_F1mg_14E08_R_0	BY926548
E_ET_F1mg_14E09_F_0	BY918245
E_ET_F1mg_14E09_R_0	BY926549
E_ET_F1mg_14E11_F_0	BY918246
E_ET_F1mg_14E11_R_0	BY926550
E_ET_F1mg_14F01_F_0	BY918247
E_ET_F1mg_14F01_R_0	BY926551
E_ET_F1mg_14F02_F_0	BY918248
E_ET_F1mg_14F02_R_0	BY926552
E_ET_F1mg_14F05_F_0	BY918249
E_ET_F1mg_14F05_R_0	BY926553
E_ET_F1mg_14F07_F_0	BY918250
E_ET_F1mg_14F07_R_0	BY926554
E_ET_F1mg_14F08_F_0	BY918251
E_ET_F1mg_14F08_R_0	BY926555
E_ET_F1mg_14F09_F_0	BY918252
E_ET_F1mg_14F09_R_0	BY926556
E_ET_F1mg_14F11_R_0	BY926557
E_ET_F1mg_14F12_F_0	BY918253
E_ET_F1mg_14F12_R_0	BY926558
E_ET_F1mg_14G02_F_0	BY918254
E_ET_F1mg_14G02_R_0	BY926559
E_ET_F1mg_14G03_F_0	BY918255
E_ET_F1mg_14G03_R_0	BY926560
E_ET_F1mg_14G04_F_0	BY918256

E_ET_F1mg_14G04_R_0	BY926561
E_ET_F1mg_14G06_F_0	BY918257
E_ET_F1mg_14G06_R_0	BY926562
E_ET_F1mg_14G09_F_0	BY918258
E_ET_F1mg_14G09_R_0	BY926563
E_ET_F1mg_14G11_F_0	BY918259
E_ET_F1mg_14G11_R_0	BY926564
E_ET_F1mg_14H01_F_0	BY918260
E_ET_F1mg_14H01_R_0	BY926565
E_ET_F1mg_14H02_F_0	BY918261
E_ET_F1mg_14H02_R_0	BY926566
E_ET_F1mg_14H03_F_0	BY918262
E_ET_F1mg_14H03_R_0	BY926567
E_ET_F1mg_14H06_F_0	BY918263
E_ET_F1mg_14H06_R_0	BY926568
E_ET_F1mg_14H07_F_0	BY918264
E_ET_F1mg_14H08_F_0	BY918265
E_ET_F1mg_14H08_R_0	BY926569
E_ET_F1mg_14H10_F_0	BY918266
E_ET_F1mg_14H10_R_0	BY926570
E_ET_F1mg_14H11_F_0	BY918267
E_ET_F1mg_14H11_R_0	BY926571
E_ET_F1mg_14H12_F_0	BY918268
E_ET_F1mg_14H12_R_0	BY926572
E_ET_F1mg_15A02_F_0	BY918269
E_ET_F1mg_15A02_R_0	BY926573
E_ET_F1mg_15A03_F_0	BY918270
E_ET_F1mg_15A03_R_0	BY926574
E_ET_F1mg_15A04_F_0	BY918271
E_ET_F1mg_15A04_R_0	BY926575
E_ET_F1mg_15A05_F_0	BY918272
E_ET_F1mg_15A05_R_0	BY926576
E_ET_F1mg_15A06_F_0	BY918273
E_ET_F1mg_15A06_R_0	BY926577
E_ET_F1mg_15A07_F_0	BY918274
E_ET_F1mg_15A07_R_0	BY926578
E_ET_F1mg_15A09_R_0	BY926579
E_ET_F1mg_15A10_F_0	BY918275
E_ET_F1mg_15A10_R_0	BY926580
E_ET_F1mg_15A11_F_0	BY918276
E_ET_F1mg_15A11_R_0	BY926581
E_ET_F1mg_15A12_F_0	BY918277
E_ET_F1mg_15A12_R_0	BY926582
E_ET_F1mg_15B01_R_0	BY926583
E_ET_F1mg_15B02_F_0	BY918278
E_ET_F1mg_15B02_R_0	BY926584
E_ET_F1mg_15B06_F_0	BY918279
E_ET_F1mg_15B07_F_0	BY918280
E_ET_F1mg_15B07_R_0	BY926585
E_ET_F1mg_15B08_F_0	BY918281
E_ET_F1mg_15B08_R_0	BY926586
E_ET_F1mg_15B12_F_0	BY918282
E_ET_F1mg_15B12_R_0	BY926587
E_ET_F1mg_15C02_F_0	BY918283

E_ET_F1mg_15C02_R_0	BY926588
E_ET_F1mg_15C04_F_0	BY918284
E_ET_F1mg_15C04_R_0	BY926589
E_ET_F1mg_15C09_R_0	BY926590
E_ET_F1mg_15C12_F_0	BY918285
E_ET_F1mg_15C12_R_0	BY926591
E_ET_F1mg_15D02_F_0	BY918286
E_ET_F1mg_15D03_F_0	BY918287
E_ET_F1mg_15D03_R_0	BY926592
E_ET_F1mg_15D11_F_0	BY918288
E_ET_F1mg_15E03_F_0	BY918289
E_ET_F1mg_15E03_R_0	BY926593
E_ET_F1mg_15E04_F_0	BY918290
E_ET_F1mg_15E04_R_0	BY926594
E_ET_F1mg_15E05_F_0	BY918291
E_ET_F1mg_15E05_R_0	BY926595
E_ET_F1mg_15E06_F_0	BY918292
E_ET_F1mg_15E06_R_0	BY926596
E_ET_F1mg_15E07_F_0	BY918293
E_ET_F1mg_15E08_F_0	BY918294
E_ET_F1mg_15E08_R_0	BY926597
E_ET_F1mg_15F01_R_0	BY926598
E_ET_F1mg_15F03_F_0	BY918295
E_ET_F1mg_15F03_R_0	BY926599
E_ET_F1mg_15F04_F_0	BY918296
E_ET_F1mg_15F04_R_0	BY926600
E_ET_F1mg_15F07_F_0	BY918297
E_ET_F1mg_15F07_R_0	BY926601
E_ET_F1mg_15F08_F_0	BY918298
E_ET_F1mg_15F08_R_0	BY926602
E_ET_F1mg_15F09_F_0	BY918299
E_ET_F1mg_15F09_R_0	BY926603
E_ET_F1mg_15F12_F_0	BY918300
E_ET_F1mg_15F12_R_0	BY926604
E_ET_F1mg_15G01_F_0	BY918301
E_ET_F1mg_15G01_R_0	BY926605
E_ET_F1mg_15G03_F_0	BY918302
E_ET_F1mg_15G03_R_0	BY926606
E_ET_F1mg_15G05_F_0	BY918303
E_ET_F1mg_15G05_R_0	BY926607
E_ET_F1mg_15G06_F_0	BY918304
E_ET_F1mg_15G06_R_0	BY926608
E_ET_F1mg_15G07_F_0	BY918305
E_ET_F1mg_15G07_R_0	BY926609
E_ET_F1mg_15G08_F_0	BY918306
E_ET_F1mg_15G08_R_0	BY926610
E_ET_F1mg_15G09_F_0	BY918307
E_ET_F1mg_15G09_R_0	BY926611
E_ET_F1mg_15G10_R_0	BY926612
E_ET_F1mg_15G11_F_0	BY918308
E_ET_F1mg_15G11_R_0	BY926613
E_ET_F1mg_15H01_F_0	BY918309
E_ET_F1mg_15H01_R_0	BY926614
E_ET_F1mg_15H02_F_0	BY918310

E_ET_F1mg_15H02_R_0	BY926615
E_ET_F1mg_15H03_F_0	BY918311
E_ET_F1mg_15H03_R_0	BY926616
E_ET_F1mg_15H05_F_0	BY918312
E_ET_F1mg_15H05_R_0	BY926617
E_ET_F1mg_15H07_F_0	BY918313
E_ET_F1mg_15H07_R_0	BY926618
E_ET_F1mg_15H08_F_0	BY918314
E_ET_F1mg_15H08_R_0	BY926619
E_ET_F1mg_15H09_F_0	BY918315
E_ET_F1mg_15H09_R_0	BY926620
E_ET_F1mg_15H11_F_0	BY918316
E_ET_F1mg_15H11_R_0	BY926621
E_ET_F1mg_15H12_F_0	BY918317
E_ET_F1mg_15H12_R_0	BY926622
E_ET_F1mg_16A05_F_0	BY918318
E_ET_F1mg_16A08_F_0	BY918319
E_ET_F1mg_16A08_R_0	BY926623
E_ET_F1mg_16A10_F_0	BY918320
E_ET_F1mg_16A10_R_0	BY926624
E_ET_F1mg_16A11_F_0	BY918321
E_ET_F1mg_16A11_R_0	BY926625
E_ET_F1mg_16A12_F_0	BY918322
E_ET_F1mg_16A12_R_0	BY926626
E_ET_F1mg_16B01_F_0	BY918323
E_ET_F1mg_16B01_R_0	BY926627
E_ET_F1mg_16B02_F_0	BY918324
E_ET_F1mg_16B02_R_0	BY926628
E_ET_F1mg_16B03_F_0	BY918325
E_ET_F1mg_16B03_R_0	BY926629
E_ET_F1mg_16B04_F_0	BY918326
E_ET_F1mg_16B04_R_0	BY926630
E_ET_F1mg_16B09_F_0	BY918327
E_ET_F1mg_16B09_R_0	BY926631
E_ET_F1mg_16B11_F_0	BY918328
E_ET_F1mg_16B11_R_0	BY926632
E_ET_F1mg_16B12_F_0	BY918329
E_ET_F1mg_16B12_R_0	BY926633
E_ET_F1mg_16C02_F_0	BY918330
E_ET_F1mg_16C04_F_0	BY918331
E_ET_F1mg_16C04_R_0	BY926634
E_ET_F1mg_16C05_F_0	BY918332
E_ET_F1mg_16C05_R_0	BY926635
E_ET_F1mg_16C06_F_0	BY918333
E_ET_F1mg_16C06_R_0	BY926636
E_ET_F1mg_16C07_F_0	BY918334
E_ET_F1mg_16C07_R_0	BY926637
E_ET_F1mg_16C09_F_0	BY918335
E_ET_F1mg_16C09_R_0	BY926638
E_ET_F1mg_16C10_F_0	BY918336
E_ET_F1mg_16C10_R_0	BY926639
E_ET_F1mg_16C12_F_0	BY918337
E_ET_F1mg_16C12_R_0	BY926640
E_ET_F1mg_16D01_F_0	BY918338

E_ET_F1mg_16D01_R_0	BY926641
E_ET_F1mg_16D06_F_0	BY918339
E_ET_F1mg_16D06_R_0	BY926642
E_ET_F1mg_16D09_F_0	BY918340
E_ET_F1mg_16D09_R_0	BY926643
E_ET_F1mg_16D10_F_0	BY918341
E_ET_F1mg_16D10_R_0	BY926644
E_ET_F1mg_16D11_F_0	BY918342
E_ET_F1mg_16D11_R_0	BY926645
E_ET_F1mg_16D12_F_0	BY918343
E_ET_F1mg_16D12_R_0	BY926646
E_ET_F1mg_16E03_F_0	BY918344
E_ET_F1mg_16E03_R_0	BY926647
E_ET_F1mg_16E04_F_0	BY918345
E_ET_F1mg_16E04_R_0	BY926648
E_ET_F1mg_16E05_F_0	BY918346
E_ET_F1mg_16E05_R_0	BY926649
E_ET_F1mg_16E06_F_0	BY918347
E_ET_F1mg_16E06_R_0	BY926650
E_ET_F1mg_16E08_F_0	BY918348
E_ET_F1mg_16E08_R_0	BY926651
E_ET_F1mg_16E10_F_0	BY918349
E_ET_F1mg_16E10_R_0	BY926652
E_ET_F1mg_16E11_F_0	BY918350
E_ET_F1mg_16E11_R_0	BY926653
E_ET_F1mg_16E12_F_0	BY918351
E_ET_F1mg_16F02_F_0	BY918352
E_ET_F1mg_16F02_R_0	BY926654
E_ET_F1mg_16F03_F_0	BY918353
E_ET_F1mg_16F03_R_0	BY926655
E_ET_F1mg_16F04_F_0	BY918354
E_ET_F1mg_16F04_R_0	BY926656
E_ET_F1mg_16F06_F_0	BY918355
E_ET_F1mg_16F06_R_0	BY926657
E_ET_F1mg_16F09_F_0	BY918356
E_ET_F1mg_16F09_R_0	BY926658
E_ET_F1mg_16F10_F_0	BY918357
E_ET_F1mg_16F10_R_0	BY926659
E_ET_F1mg_16F12_F_0	BY918358
E_ET_F1mg_16F12_R_0	BY926660
E_ET_F1mg_16G01_F_0	BY918359
E_ET_F1mg_16G06_F_0	BY918360
E_ET_F1mg_16G06_R_0	BY926661
E_ET_F1mg_16G07_F_0	BY918361
E_ET_F1mg_16G07_R_0	BY926662
E_ET_F1mg_16G08_F_0	BY918362
E_ET_F1mg_16G08_R_0	BY926663
E_ET_F1mg_16G09_F_0	BY918363
E_ET_F1mg_16G09_R_0	BY926664
E_ET_F1mg_16G11_F_0	BY918364
E_ET_F1mg_16G11_R_0	BY926665
E_ET_F1mg_16H01_F_0	BY918365
E_ET_F1mg_16H01_R_0	BY926666
E_ET_F1mg_16H02_F_0	BY918366

E_ET_F1mg_16H02_R_0	BY926667
E_ET_F1mg_16H03_F_0	BY918367
E_ET_F1mg_16H03_R_0	BY926668
E_ET_F1mg_16H04_F_0	BY918368
E_ET_F1mg_16H04_R_0	BY926669
E_ET_F1mg_16H06_F_0	BY918369
E_ET_F1mg_16H06_R_0	BY926670
E_ET_F1mg_16H07_F_0	BY918370
E_ET_F1mg_16H07_R_0	BY926671
E_ET_F1mg_16H09_F_0	BY918371
E_ET_F1mg_16H09_R_0	BY926672
E_ET_F1mg_16H10_F_0	BY918372
E_ET_F1mg_16H10_R_0	BY926673
E_ET_F1mg_16H11_F_0	BY918373
E_ET_F1mg_16H11_R_0	BY926674
E_ET_F1mg_17A02_F_0	BY918374
E_ET_F1mg_17A05_F_0	BY918375
E_ET_F1mg_17A05_R_0	BY926675
E_ET_F1mg_17A06_F_0	BY918376
E_ET_F1mg_17A06_R_0	BY926676
E_ET_F1mg_17A07_F_0	BY918377
E_ET_F1mg_17A07_R_0	BY926677
E_ET_F1mg_17A09_F_0	BY918378
E_ET_F1mg_17A09_R_0	BY926678
E_ET_F1mg_17A10_F_0	BY918379
E_ET_F1mg_17A10_R_0	BY926679
E_ET_F1mg_17A11_F_0	BY918380
E_ET_F1mg_17A11_R_0	BY926680
E_ET_F1mg_17B01_R_0	BY926681
E_ET_F1mg_17B04_R_0	BY926682
E_ET_F1mg_17B06_F_0	BY918381
E_ET_F1mg_17B06_R_0	BY926683
E_ET_F1mg_17B09_F_0	BY918382
E_ET_F1mg_17B09_R_0	BY926684
E_ET_F1mg_17C03_R_0	BY926685
E_ET_F1mg_17C07_F_0	BY918383
E_ET_F1mg_17C07_R_0	BY926686
E_ET_F1mg_17C12_F_0	BY918384
E_ET_F1mg_17C12_R_0	BY926687
E_ET_F1mg_17D01_R_0	BY926688
E_ET_F1mg_17D02_R_0	BY926689
E_ET_F1mg_17D03_R_0	BY926690
E_ET_F1mg_17D05_R_0	BY926691
E_ET_F1mg_17D06_R_0	BY926692
E_ET_F1mg_17D07_F_0	BY918385
E_ET_F1mg_17D07_R_0	BY926693
E_ET_F1mg_17D08_F_0	BY918386
E_ET_F1mg_17D08_R_0	BY926694
E_ET_F1mg_17D09_F_0	BY918387
E_ET_F1mg_17D09_R_0	BY926695
E_ET_F1mg_17D10_F_0	BY918388
E_ET_F1mg_17D10_R_0	BY926696
E_ET_F1mg_17D12_F_0	BY918389
E_ET_F1mg_17D12_R_0	BY926697

E_ET_F1mg_17E03_R_0	BY926698
E_ET_F1mg_17E04_R_0	BY926699
E_ET_F1mg_17E08_F_0	BY918390
E_ET_F1mg_17E08_R_0	BY926700
E_ET_F1mg_17E10_F_0	BY918391
E_ET_F1mg_17E10_R_0	BY926701
E_ET_F1mg_17E11_F_0	BY918392
E_ET_F1mg_17E11_R_0	BY926702
E_ET_F1mg_17E12_F_0	BY918393
E_ET_F1mg_17E12_R_0	BY926703
E_ET_F1mg_17F01_R_0	BY926704
E_ET_F1mg_17F02_R_0	BY926705
E_ET_F1mg_17F03_R_0	BY926706
E_ET_F1mg_17F05_R_0	BY926707
E_ET_F1mg_17F10_F_0	BY918394
E_ET_F1mg_17F10_R_0	BY926708
E_ET_F1mg_17F11_F_0	BY918395
E_ET_F1mg_17F12_F_0	BY918396
E_ET_F1mg_17F12_R_0	BY926709
E_ET_F1mg_17G02_F_0	BY918397
E_ET_F1mg_17G02_R_0	BY926710
E_ET_F1mg_17G06_F_0	BY918398
E_ET_F1mg_17G06_R_0	BY926711
E_ET_F1mg_17G07_F_0	BY918399
E_ET_F1mg_17G07_R_0	BY926712
E_ET_F1mg_17G09_F_0	BY918400
E_ET_F1mg_17G10_F_0	BY918401
E_ET_F1mg_17G10_R_0	BY926713
E_ET_F1mg_17G12_F_0	BY918402
E_ET_F1mg_17G12_R_0	BY926714
E_ET_F1mg_17H04_R_0	BY926715
E_ET_F1mg_17H05_F_0	BY918403
E_ET_F1mg_17H05_R_0	BY926716
E_ET_F1mg_17H07_F_0	BY918404
E_ET_F1mg_17H07_R_0	BY926717
E_ET_F1mg_17H08_F_0	BY918405
E_ET_F1mg_17H08_R_0	BY926718
E_ET_F1mg_17H09_F_0	BY918406
E_ET_F1mg_17H09_R_0	BY926719
E_ET_F1mg_17H11_F_0	BY918407
E_ET_F1mg_17H11_R_0	BY926720
E_ET_F1mg_17H12_F_0	BY918408
E_ET_F1mg_17H12_R_0	BY926721
E_ET_F1mg_18A03_F_0	BY918409
E_ET_F1mg_18A03_R_0	BY926722
E_ET_F1mg_18A06_F_0	BY918410
E_ET_F1mg_18A06_R_0	BY926723
E_ET_F1mg_18A09_F_0	BY918411
E_ET_F1mg_18A09_R_0	BY926724
E_ET_F1mg_18B01_F_0	BY918412
E_ET_F1mg_18B01_R_0	BY926725
E_ET_F1mg_18B03_F_0	BY918413
E_ET_F1mg_18B03_R_0	BY926726
E_ET_F1mg_18B04_F_0	BY918414

E_ET_F1mg_18B04_R_0	BY926727
E_ET_F1mg_18B05_F_0	BY918415
E_ET_F1mg_18B05_R_0	BY926728
E_ET_F1mg_18B09_F_0	BY918416
E_ET_F1mg_18B09_R_0	BY926729
E_ET_F1mg_18B11_F_0	BY918417
E_ET_F1mg_18B11_R_0	BY926730
E_ET_F1mg_18B12_F_0	BY918418
E_ET_F1mg_18B12_R_0	BY926731
E_ET_F1mg_18C04_F_0	BY918419
E_ET_F1mg_18C04_R_0	BY926732
E_ET_F1mg_18C05_F_0	BY918420
E_ET_F1mg_18C05_R_0	BY926733
E_ET_F1mg_18C07_F_0	BY918421
E_ET_F1mg_18C07_R_0	BY926734
E_ET_F1mg_18C08_F_0	BY918422
E_ET_F1mg_18C08_R_0	BY926735
E_ET_F1mg_18C10_F_0	BY918423
E_ET_F1mg_18C10_R_0	BY926736
E_ET_F1mg_18C11_F_0	BY918424
E_ET_F1mg_18C11_R_0	BY926737
E_ET_F1mg_18C12_F_0	BY918425
E_ET_F1mg_18C12_R_0	BY926738
E_ET_F1mg_18D01_F_0	BY918426
E_ET_F1mg_18D01_R_0	BY926739
E_ET_F1mg_18D02_F_0	BY918427
E_ET_F1mg_18D02_R_0	BY926740
E_ET_F1mg_18D05_F_0	BY918428
E_ET_F1mg_18D05_R_0	BY926741
E_ET_F1mg_18D08_F_0	BY918429
E_ET_F1mg_18D08_R_0	BY926742
E_ET_F1mg_18D12_F_0	BY918430
E_ET_F1mg_18D12_R_0	BY926743
E_ET_F1mg_18E05_R_0	BY926744
E_ET_F1mg_18E08_F_0	BY918431
E_ET_F1mg_18E08_R_0	BY926745
E_ET_F1mg_18E11_F_0	BY918432
E_ET_F1mg_18E11_R_0	BY926746
E_ET_F1mg_18F03_F_0	BY918433
E_ET_F1mg_18F03_R_0	BY926747
E_ET_F1mg_18F04_F_0	BY918434
E_ET_F1mg_18F04_R_0	BY926748
E_ET_F1mg_18F05_F_0	BY918435
E_ET_F1mg_18F05_R_0	BY926749
E_ET_F1mg_18F06_F_0	BY918436
E_ET_F1mg_18F06_R_0	BY926750
E_ET_F1mg_18F07_F_0	BY918437
E_ET_F1mg_18F07_R_0	BY926751
E_ET_F1mg_18F08_F_0	BY918438
E_ET_F1mg_18F08_R_0	BY926752
E_ET_F1mg_18F09_F_0	BY918439
E_ET_F1mg_18F09_R_0	BY926753
E_ET_F1mg_18F10_F_0	BY918440
E_ET_F1mg_18F10_R_0	BY926754

E_ET_F1mg_18F12_F_0	BY918441
E_ET_F1mg_18F12_R_0	BY926755
E_ET_F1mg_18G01_F_0	BY918442
E_ET_F1mg_18G01_R_0	BY926756
E_ET_F1mg_18G02_F_0	BY918443
E_ET_F1mg_18G02_R_0	BY926757
E_ET_F1mg_18G03_F_0	BY918444
E_ET_F1mg_18G03_R_0	BY926758
E_ET_F1mg_18G04_F_0	BY918445
E_ET_F1mg_18G04_R_0	BY926759
E_ET_F1mg_18G05_F_0	BY918446
E_ET_F1mg_18G05_R_0	BY926760
E_ET_F1mg_18G06_F_0	BY918447
E_ET_F1mg_18G06_R_0	BY926761
E_ET_F1mg_18G07_F_0	BY918448
E_ET_F1mg_18G07_R_0	BY926762
E_ET_F1mg_18G09_F_0	BY918449
E_ET_F1mg_18G09_R_0	BY926763
E_ET_F1mg_18G10_F_0	BY918450
E_ET_F1mg_18G10_R_0	BY926764
E_ET_F1mg_18H01_F_0	BY918451
E_ET_F1mg_18H01_R_0	BY926765
E_ET_F1mg_18H02_F_0	BY918452
E_ET_F1mg_18H02_R_0	BY926766
E_ET_F1mg_18H05_R_0	BY926767
E_ET_F1mg_18H08_F_0	BY918453
E_ET_F1mg_18H08_R_0	BY926768
E_ET_F1mg_18H09_F_0	BY918454
E_ET_F1mg_18H09_R_0	BY926769
E_ET_F1mg_18H11_F_0	BY918455
E_ET_F1mg_18H11_R_0	BY926770
E_ET_F1mg_19A01_F_0	BY918456
E_ET_F1mg_19A01_R_0	BY926771
E_ET_F1mg_19A02_F_0	BY918457
E_ET_F1mg_19A03_F_0	BY918458
E_ET_F1mg_19A03_R_0	BY926772
E_ET_F1mg_19A05_F_0	BY918459
E_ET_F1mg_19A05_R_0	BY926773
E_ET_F1mg_19A06_F_0	BY918460
E_ET_F1mg_19A07_F_0	BY918461
E_ET_F1mg_19A07_R_0	BY926774
E_ET_F1mg_19A10_F_0	BY918462
E_ET_F1mg_19A10_R_0	BY926775
E_ET_F1mg_19B01_F_0	BY918463
E_ET_F1mg_19B01_R_0	BY926776
E_ET_F1mg_19B02_R_0	BY926777
E_ET_F1mg_19B03_F_0	BY918464
E_ET_F1mg_19B03_R_0	BY926778
E_ET_F1mg_19B07_F_0	BY918465
E_ET_F1mg_19B09_F_0	BY918466
E_ET_F1mg_19B09_R_0	BY926779
E_ET_F1mg_19B11_F_0	BY918467
E_ET_F1mg_19B11_R_0	BY926780
E_ET_F1mg_19C01_F_0	BY918468

E_ET_F1mg_19C01_R_0	BY926781
E_ET_F1mg_19C02_F_0	BY918469
E_ET_F1mg_19C02_R_0	BY926782
E_ET_F1mg_19C08_F_0	BY918470
E_ET_F1mg_19C08_R_0	BY926783
E_ET_F1mg_19C12_F_0	BY918471
E_ET_F1mg_19C12_R_0	BY926784
E_ET_F1mg_19D02_F_0	BY918472
E_ET_F1mg_19D02_R_0	BY926785
E_ET_F1mg_19D04_F_0	BY918473
E_ET_F1mg_19D04_R_0	BY926786
E_ET_F1mg_19D05_F_0	BY918474
E_ET_F1mg_19D05_R_0	BY926787
E_ET_F1mg_19D06_F_0	BY918475
E_ET_F1mg_19D06_R_0	BY926788
E_ET_F1mg_19D07_F_0	BY918476
E_ET_F1mg_19D07_R_0	BY926789
E_ET_F1mg_19D08_F_0	BY918477
E_ET_F1mg_19D08_R_0	BY926790
E_ET_F1mg_19D09_F_0	BY918478
E_ET_F1mg_19D09_R_0	BY926791
E_ET_F1mg_19D11_F_0	BY918479
E_ET_F1mg_19D11_R_0	BY926792
E_ET_F1mg_19D12_F_0	BY918480
E_ET_F1mg_19D12_R_0	BY926793
E_ET_F1mg_19E01_F_0	BY918481
E_ET_F1mg_19E01_R_0	BY926794
E_ET_F1mg_19E02_F_0	BY918482
E_ET_F1mg_19E02_R_0	BY926795
E_ET_F1mg_19E03_F_0	BY918483
E_ET_F1mg_19E03_R_0	BY926796
E_ET_F1mg_19E04_F_0	BY918484
E_ET_F1mg_19E04_R_0	BY926797
E_ET_F1mg_19E07_F_0	BY918485
E_ET_F1mg_19E07_R_0	BY926798
E_ET_F1mg_19E10_F_0	BY918486
E_ET_F1mg_19E10_R_0	BY926799
E_ET_F1mg_19E11_F_0	BY918487
E_ET_F1mg_19E11_R_0	BY926800
E_ET_F1mg_19F02_F_0	BY918488
E_ET_F1mg_19F02_R_0	BY926801
E_ET_F1mg_19F03_F_0	BY918489
E_ET_F1mg_19F03_R_0	BY926802
E_ET_F1mg_19F06_F_0	BY918490
E_ET_F1mg_19F06_R_0	BY926803
E_ET_F1mg_19F07_F_0	BY918491
E_ET_F1mg_19F07_R_0	BY926804
E_ET_F1mg_19F09_F_0	BY918492
E_ET_F1mg_19F09_R_0	BY926805
E_ET_F1mg_19G01_F_0	BY918493
E_ET_F1mg_19G01_R_0	BY926806
E_ET_F1mg_19G02_F_0	BY918494
E_ET_F1mg_19G02_R_0	BY926807
E_ET_F1mg_19G07_F_0	BY918495

E_ET_F1mg_19G07_R_0	BY926808
E_ET_F1mg_19G09_F_0	BY918496
E_ET_F1mg_19G09_R_0	BY926809
E_ET_F1mg_19G10_F_0	BY918497
E_ET_F1mg_19G10_R_0	BY926810
E_ET_F1mg_19G11_F_0	BY918498
E_ET_F1mg_19G11_R_0	BY926811
E_ET_F1mg_19G12_F_0	BY918499
E_ET_F1mg_19H05_F_0	BY918500
E_ET_F1mg_19H05_R_0	BY926812
E_ET_F1mg_19H08_F_0	BY918501
E_ET_F1mg_19H08_R_0	BY926813
E_ET_F1mg_20A03_F_0	BY918502
E_ET_F1mg_20A05_F_0	BY918503
E_ET_F1mg_20A05_R_0	BY926814
E_ET_F1mg_20A06_F_0	BY918504
E_ET_F1mg_20A07_F_0	BY918505
E_ET_F1mg_20A07_R_0	BY926815
E_ET_F1mg_20A11_F_0	BY918506
E_ET_F1mg_20A11_R_0	BY926816
E_ET_F1mg_20A12_F_0	BY918507
E_ET_F1mg_20A12_R_0	BY926817
E_ET_F1mg_20B01_F_0	BY918508
E_ET_F1mg_20B01_R_0	BY926818
E_ET_F1mg_20B02_F_0	BY918509
E_ET_F1mg_20B02_R_0	BY926819
E_ET_F1mg_20B04_F_0	BY918510
E_ET_F1mg_20B04_R_0	BY926820
E_ET_F1mg_20B06_F_0	BY918511
E_ET_F1mg_20B06_R_0	BY926821
E_ET_F1mg_20B09_F_0	BY918512
E_ET_F1mg_20B09_R_0	BY926822
E_ET_F1mg_20B10_F_0	BY918513
E_ET_F1mg_20B10_R_0	BY926823
E_ET_F1mg_20B11_F_0	BY918514
E_ET_F1mg_20B11_R_0	BY926824
E_ET_F1mg_20B12_F_0	BY918515
E_ET_F1mg_20B12_R_0	BY926825
E_ET_F1mg_20C01_F_0	BY918516
E_ET_F1mg_20C01_R_0	BY926826
E_ET_F1mg_20C02_R_0	BY926827
E_ET_F1mg_20C03_F_0	BY918517
E_ET_F1mg_20C03_R_0	BY926828
E_ET_F1mg_20C06_F_0	BY918518
E_ET_F1mg_20C06_R_0	BY926829
E_ET_F1mg_20C08_F_0	BY918519
E_ET_F1mg_20C08_R_0	BY926830
E_ET_F1mg_20C09_F_0	BY918520
E_ET_F1mg_20C09_R_0	BY926831
E_ET_F1mg_20C10_F_0	BY918521
E_ET_F1mg_20C10_R_0	BY926832
E_ET_F1mg_20C11_F_0	BY918522
E_ET_F1mg_20C12_F_0	BY918523
E_ET_F1mg_20C12_R_0	BY926833

E_ET_F1mg_20D01_F_0	BY918524
E_ET_F1mg_20D01_R_0	BY926834
E_ET_F1mg_20D02_F_0	BY918525
E_ET_F1mg_20D02_R_0	BY926835
E_ET_F1mg_20D03_F_0	BY918526
E_ET_F1mg_20D03_R_0	BY926836
E_ET_F1mg_20D04_F_0	BY918527
E_ET_F1mg_20D04_R_0	BY926837
E_ET_F1mg_20D05_F_0	BY918528
E_ET_F1mg_20D05_R_0	BY926838
E_ET_F1mg_20D06_F_0	BY918529
E_ET_F1mg_20D06_R_0	BY926839
E_ET_F1mg_20D07_F_0	BY918530
E_ET_F1mg_20D07_R_0	BY926840
E_ET_F1mg_20D09_F_0	BY918531
E_ET_F1mg_20D09_R_0	BY926841
E_ET_F1mg_20D11_F_0	BY918532
E_ET_F1mg_20D11_R_0	BY926842
E_ET_F1mg_20E01_F_0	BY918533
E_ET_F1mg_20E01_R_0	BY926843
E_ET_F1mg_20E02_F_0	BY918534
E_ET_F1mg_20E02_R_0	BY926844
E_ET_F1mg_20E04_F_0	BY918535
E_ET_F1mg_20E04_R_0	BY926845
E_ET_F1mg_20E05_F_0	BY918536
E_ET_F1mg_20E05_R_0	BY926846
E_ET_F1mg_20E06_F_0	BY918537
E_ET_F1mg_20E06_R_0	BY926847
E_ET_F1mg_20E09_F_0	BY918538
E_ET_F1mg_20E09_R_0	BY926848
E_ET_F1mg_20E10_F_0	BY918539
E_ET_F1mg_20E10_R_0	BY926849
E_ET_F1mg_20E11_F_0	BY918540
E_ET_F1mg_20E11_R_0	BY926850
E_ET_F1mg_20E12_F_0	BY918541
E_ET_F1mg_20E12_R_0	BY926851
E_ET_F1mg_20F01_F_0	BY918542
E_ET_F1mg_20F01_R_0	BY926852
E_ET_F1mg_20F02_F_0	BY918543
E_ET_F1mg_20F03_F_0	BY918544
E_ET_F1mg_20F03_R_0	BY926853
E_ET_F1mg_20F04_R_0	BY926854
E_ET_F1mg_20F10_F_0	BY918545
E_ET_F1mg_20F10_R_0	BY926855
E_ET_F1mg_20F11_F_0	BY918546
E_ET_F1mg_20F11_R_0	BY926856
E_ET_F1mg_20F12_F_0	BY918547
E_ET_F1mg_20F12_R_0	BY926857
E_ET_F1mg_20G05_F_0	BY918548
E_ET_F1mg_20G05_R_0	BY926858
E_ET_F1mg_20G07_F_0	BY918549
E_ET_F1mg_20G07_R_0	BY926859
E_ET_F1mg_20G08_F_0	BY918550
E_ET_F1mg_20G08_R_0	BY926860

E_ET_F1mg_20G09_F_0	BY918551
E_ET_F1mg_20G10_F_0	BY918552
E_ET_F1mg_20G10_R_0	BY926861
E_ET_F1mg_20G11_F_0	BY918553
E_ET_F1mg_20G11_R_0	BY926862
E_ET_F1mg_20H02_F_0	BY918554
E_ET_F1mg_20H04_F_0	BY918555
E_ET_F1mg_20H04_R_0	BY926863
E_ET_F1mg_21A02_F_0	BY918556
E_ET_F1mg_21A05_F_0	BY918557
E_ET_F1mg_21A05_R_0	BY926864
E_ET_F1mg_21A07_F_0	BY918558
E_ET_F1mg_21A07_R_0	BY926865
E_ET_F1mg_21A08_F_0	BY918559
E_ET_F1mg_21A08_R_0	BY926866
E_ET_F1mg_21A09_F_0	BY918560
E_ET_F1mg_21A09_R_0	BY926867
E_ET_F1mg_21A12_F_0	BY918561
E_ET_F1mg_21A12_R_0	BY926868
E_ET_F1mg_21B02_F_0	BY918562
E_ET_F1mg_21B02_R_0	BY926869
E_ET_F1mg_21B03_F_0	BY918563
E_ET_F1mg_21B03_R_0	BY926870
E_ET_F1mg_21B06_F_0	BY918564
E_ET_F1mg_21B06_R_0	BY926871
E_ET_F1mg_21B07_F_0	BY918565
E_ET_F1mg_21B07_R_0	BY926872
E_ET_F1mg_21B09_F_0	BY918566
E_ET_F1mg_21B09_R_0	BY926873
E_ET_F1mg_21B10_F_0	BY918567
E_ET_F1mg_21B10_R_0	BY926874
E_ET_F1mg_21B11_F_0	BY918568
E_ET_F1mg_21B11_R_0	BY926875
E_ET_F1mg_21C01_F_0	BY918569
E_ET_F1mg_21C01_R_0	BY926876
E_ET_F1mg_21C02_F_0	BY918570
E_ET_F1mg_21C02_R_0	BY926877
E_ET_F1mg_21C04_F_0	BY918571
E_ET_F1mg_21C04_R_0	BY926878
E_ET_F1mg_21C05_F_0	BY918572
E_ET_F1mg_21C05_R_0	BY926879
E_ET_F1mg_21C06_F_0	BY918573
E_ET_F1mg_21C06_R_0	BY926880
E_ET_F1mg_21C10_F_0	BY918574
E_ET_F1mg_21C10_R_0	BY926881
E_ET_F1mg_21C11_F_0	BY918575
E_ET_F1mg_21C11_R_0	BY926882
E_ET_F1mg_21C12_F_0	BY918576
E_ET_F1mg_21C12_R_0	BY926883
E_ET_F1mg_21D01_F_0	BY918577
E_ET_F1mg_21D01_R_0	BY926884
E_ET_F1mg_21D03_F_0	BY918578
E_ET_F1mg_21D03_R_0	BY926885
E_ET_F1mg_21D04_F_0	BY918579

E_ET_F1mg_21D04_R_0	BY926886
E_ET_F1mg_21D06_F_0	BY918580
E_ET_F1mg_21D06_R_0	BY926887
E_ET_F1mg_21D07_F_0	BY918581
E_ET_F1mg_21D07_R_0	BY926888
E_ET_F1mg_21D08_F_0	BY918582
E_ET_F1mg_21D08_R_0	BY926889
E_ET_F1mg_21D09_F_0	BY918583
E_ET_F1mg_21D09_R_0	BY926890
E_ET_F1mg_21D10_F_0	BY918584
E_ET_F1mg_21D10_R_0	BY926891
E_ET_F1mg_21D11_F_0	BY918585
E_ET_F1mg_21D11_R_0	BY926892
E_ET_F1mg_21D12_F_0	BY918586
E_ET_F1mg_21D12_R_0	BY926893
E_ET_F1mg_21E02_F_0	BY918587
E_ET_F1mg_21E04_F_0	BY918588
E_ET_F1mg_21E04_R_0	BY926894
E_ET_F1mg_21E05_F_0	BY918589
E_ET_F1mg_21E05_R_0	BY926895
E_ET_F1mg_21E06_F_0	BY918590
E_ET_F1mg_21E06_R_0	BY926896
E_ET_F1mg_21E07_F_0	BY918591
E_ET_F1mg_21E07_R_0	BY926897
E_ET_F1mg_21E08_F_0	BY918592
E_ET_F1mg_21E08_R_0	BY926898
E_ET_F1mg_21E09_F_0	BY918593
E_ET_F1mg_21E09_R_0	BY926899
E_ET_F1mg_21E10_R_0	BY926900
E_ET_F1mg_21F02_F_0	BY918594
E_ET_F1mg_21F02_R_0	BY926901
E_ET_F1mg_21F04_F_0	BY918595
E_ET_F1mg_21F04_R_0	BY926902
E_ET_F1mg_21F06_F_0	BY918596
E_ET_F1mg_21F06_R_0	BY926903
E_ET_F1mg_21F07_F_0	BY918597
E_ET_F1mg_21F07_R_0	BY926904
E_ET_F1mg_21F09_F_0	BY918598
E_ET_F1mg_21F09_R_0	BY926905
E_ET_F1mg_21G01_F_0	BY918599
E_ET_F1mg_21G01_R_0	BY926906
E_ET_F1mg_21G03_F_0	BY918600
E_ET_F1mg_21G03_R_0	BY926907
E_ET_F1mg_21G05_F_0	BY918601
E_ET_F1mg_21G05_R_0	BY926908
E_ET_F1mg_21G06_F_0	BY918602
E_ET_F1mg_21G06_R_0	BY926909
E_ET_F1mg_21G09_F_0	BY918603
E_ET_F1mg_21G09_R_0	BY926910
E_ET_F1mg_21G11_R_0	BY926911
E_ET_F1mg_21G12_F_0	BY918604
E_ET_F1mg_21G12_R_0	BY926912
E_ET_F1mg_21H02_F_0	BY918605
E_ET_F1mg_21H02_R_0	BY926913

E_ET_F1mg_21H05_F_0	BY918606
E_ET_F1mg_21H05_R_0	BY926914
E_ET_F1mg_21H09_F_0	BY918607
E_ET_F1mg_21H09_R_0	BY926915
E_ET_F1mg_21H10_F_0	BY918608
E_ET_F1mg_21H10_R_0	BY926916
E_ET_F1mg_21H11_F_0	BY918609
E_ET_F1mg_21H11_R_0	BY926917
E_ET_F1mg_21H12_F_0	BY918610
E_ET_F1mg_21H12_R_0	BY926918
E_ET_F1mg_22A01_F_0	BY918611
E_ET_F1mg_22A01_R_0	BY926919
E_ET_F1mg_22A02_R_0	BY926920
E_ET_F1mg_22A03_F_0	BY918612
E_ET_F1mg_22A03_R_0	BY926921
E_ET_F1mg_22A05_F_0	BY918613
E_ET_F1mg_22A05_R_0	BY926922
E_ET_F1mg_22A06_F_0	BY918614
E_ET_F1mg_22A07_F_0	BY918615
E_ET_F1mg_22A07_R_0	BY926923
E_ET_F1mg_22A09_F_0	BY918616
E_ET_F1mg_22A09_R_0	BY926924
E_ET_F1mg_22A10_F_0	BY918617
E_ET_F1mg_22A10_R_0	BY926925
E_ET_F1mg_22A11_F_0	BY918618
E_ET_F1mg_22A11_R_0	BY926926
E_ET_F1mg_22A12_F_0	BY918619
E_ET_F1mg_22A12_R_0	BY926927
E_ET_F1mg_22B02_F_0	BY918620
E_ET_F1mg_22B02_R_0	BY926928
E_ET_F1mg_22B03_F_0	BY918621
E_ET_F1mg_22B03_R_0	BY926929
E_ET_F1mg_22B04_F_0	BY918622
E_ET_F1mg_22B04_R_0	BY926930
E_ET_F1mg_22B08_F_0	BY918623
E_ET_F1mg_22B08_R_0	BY926931
E_ET_F1mg_22B10_F_0	BY918624
E_ET_F1mg_22B10_R_0	BY926932
E_ET_F1mg_22C02_F_0	BY918625
E_ET_F1mg_22C02_R_0	BY926933
E_ET_F1mg_22C03_F_0	BY918626
E_ET_F1mg_22C03_R_0	BY926934
E_ET_F1mg_22C05_F_0	BY918627
E_ET_F1mg_22C05_R_0	BY926935
E_ET_F1mg_22C06_F_0	BY918628
E_ET_F1mg_22C06_R_0	BY926936
E_ET_F1mg_22C07_F_0	BY918629
E_ET_F1mg_22C07_R_0	BY926937
E_ET_F1mg_22C08_F_0	BY918630
E_ET_F1mg_22C08_R_0	BY926938
E_ET_F1mg_22C09_F_0	BY918631
E_ET_F1mg_22C09_R_0	BY926939
E_ET_F1mg_22C10_F_0	BY918632
E_ET_F1mg_22C10_R_0	BY926940

E_ET_F1mg_22C11_F_0	BY918633
E_ET_F1mg_22C11_R_0	BY926941
E_ET_F1mg_22C12_F_0	BY918634
E_ET_F1mg_22C12_R_0	BY926942
E_ET_F1mg_22D01_F_0	BY918635
E_ET_F1mg_22D01_R_0	BY926943
E_ET_F1mg_22D03_F_0	BY918636
E_ET_F1mg_22D03_R_0	BY926944
E_ET_F1mg_22D04_F_0	BY918637
E_ET_F1mg_22D04_R_0	BY926945
E_ET_F1mg_22D06_F_0	BY918638
E_ET_F1mg_22D06_R_0	BY926946
E_ET_F1mg_22D07_F_0	BY918639
E_ET_F1mg_22D07_R_0	BY926947
E_ET_F1mg_22D08_F_0	BY918640
E_ET_F1mg_22D08_R_0	BY926948
E_ET_F1mg_22D09_F_0	BY918641
E_ET_F1mg_22D09_R_0	BY926949
E_ET_F1mg_22D11_F_0	BY918642
E_ET_F1mg_22D11_R_0	BY926950
E_ET_F1mg_22D12_F_0	BY918643
E_ET_F1mg_22D12_R_0	BY926951
E_ET_F1mg_22E01_F_0	BY918644
E_ET_F1mg_22E04_F_0	BY918645
E_ET_F1mg_22E04_R_0	BY926952
E_ET_F1mg_22E05_F_0	BY918646
E_ET_F1mg_22E05_R_0	BY926953
E_ET_F1mg_22E06_R_0	BY926954
E_ET_F1mg_22E07_F_0	BY918647
E_ET_F1mg_22E07_R_0	BY926955
E_ET_F1mg_22E08_F_0	BY918648
E_ET_F1mg_22E08_R_0	BY926956
E_ET_F1mg_22E09_F_0	BY918649
E_ET_F1mg_22E09_R_0	BY926957
E_ET_F1mg_22E10_F_0	BY918650
E_ET_F1mg_22E10_R_0	BY926958
E_ET_F1mg_22E12_F_0	BY918651
E_ET_F1mg_22F01_F_0	BY918652
E_ET_F1mg_22F01_R_0	BY926959
E_ET_F1mg_22F02_F_0	BY918653
E_ET_F1mg_22F02_R_0	BY926960
E_ET_F1mg_22F03_F_0	BY918654
E_ET_F1mg_22F03_R_0	BY926961
E_ET_F1mg_22F07_F_0	BY918655
E_ET_F1mg_22F07_R_0	BY926962
E_ET_F1mg_22F10_F_0	BY918656
E_ET_F1mg_22F10_R_0	BY926963
E_ET_F1mg_22F11_F_0	BY918657
E_ET_F1mg_22F11_R_0	BY926964
E_ET_F1mg_22F12_F_0	BY918658
E_ET_F1mg_22F12_R_0	BY926965
E_ET_F1mg_22G01_F_0	BY918659
E_ET_F1mg_22G01_R_0	BY926966
E_ET_F1mg_22G02_F_0	BY918660

E_ET_F1mg_22G03_F_0	BY918661
E_ET_F1mg_22G04_F_0	BY918662
E_ET_F1mg_22G08_F_0	BY918663
E_ET_F1mg_22G09_F_0	BY918664
E_ET_F1mg_22G10_F_0	BY918665
E_ET_F1mg_22G11_F_0	BY918666
E_ET_F1mg_22G12_F_0	BY918667
E_ET_F1mg_22H01_F_0	BY918668
E_ET_F1mg_22H02_R_0	BY926967
E_ET_F1mg_22H03_F_0	BY918669
E_ET_F1mg_22H03_R_0	BY926968
E_ET_F1mg_22H04_F_0	BY918670
E_ET_F1mg_22H04_R_0	BY926969
E_ET_F1mg_22H05_F_0	BY918671
E_ET_F1mg_22H05_R_0	BY926970
E_ET_F1mg_22H07_F_0	BY918672
E_ET_F1mg_22H07_R_0	BY926971
E_ET_F1mg_22H08_F_0	BY918673
E_ET_F1mg_22H11_F_0	BY918674
E_ET_F1mg_22H11_R_0	BY926972
E_ET_F1mg_23A01_F_0	BY918675
E_ET_F1mg_23A01_R_0	BY926973
E_ET_F1mg_23A02_F_0	BY918676
E_ET_F1mg_23A02_R_0	BY926974
E_ET_F1mg_23A03_F_0	BY918677
E_ET_F1mg_23A03_R_0	BY926975
E_ET_F1mg_23A08_R_0	BY926976
E_ET_F1mg_23A09_F_0	BY918678
E_ET_F1mg_23A09_R_0	BY926977
E_ET_F1mg_23A10_F_0	BY918679
E_ET_F1mg_23A10_R_0	BY926978
E_ET_F1mg_23A11_F_0	BY918680
E_ET_F1mg_23A11_R_0	BY926979
E_ET_F1mg_23A12_F_0	BY918681
E_ET_F1mg_23B02_F_0	BY918682
E_ET_F1mg_23B02_R_0	BY926980
E_ET_F1mg_23B03_F_0	BY918683
E_ET_F1mg_23B03_R_0	BY926981
E_ET_F1mg_23B04_F_0	BY918684
E_ET_F1mg_23B04_R_0	BY926982
E_ET_F1mg_23B06_F_0	BY918685
E_ET_F1mg_23B06_R_0	BY926983
E_ET_F1mg_23B08_F_0	BY918686
E_ET_F1mg_23B08_R_0	BY926984
E_ET_F1mg_23B10_F_0	BY918687
E_ET_F1mg_23B10_R_0	BY926985
E_ET_F1mg_23B11_F_0	BY918688
E_ET_F1mg_23B11_R_0	BY926986
E_ET_F1mg_23B12_F_0	BY918689
E_ET_F1mg_23B12_R_0	BY926987
E_ET_F1mg_23C01_F_0	BY918690
E_ET_F1mg_23C01_R_0	BY926988
E_ET_F1mg_23C02_F_0	BY918691
E_ET_F1mg_23C04_F_0	BY918692

E_ET_F1mg_23C04_R_0	BY926989
E_ET_F1mg_23C06_F_0	BY918693
E_ET_F1mg_23C07_F_0	BY918694
E_ET_F1mg_23C07_R_0	BY926990
E_ET_F1mg_23C08_F_0	BY918695
E_ET_F1mg_23C08_R_0	BY926991
E_ET_F1mg_23C09_F_0	BY918696
E_ET_F1mg_23C09_R_0	BY926992
E_ET_F1mg_23C11_F_0	BY918697
E_ET_F1mg_23C11_R_0	BY926993
E_ET_F1mg_23D02_F_0	BY918698
E_ET_F1mg_23D02_R_0	BY926994
E_ET_F1mg_23D04_F_0	BY918699
E_ET_F1mg_23D04_R_0	BY926995
E_ET_F1mg_23D05_F_0	BY918700
E_ET_F1mg_23D05_R_0	BY926996
E_ET_F1mg_23D06_F_0	BY918701
E_ET_F1mg_23D06_R_0	BY926997
E_ET_F1mg_23D07_F_0	BY918702
E_ET_F1mg_23D07_R_0	BY926998
E_ET_F1mg_23D08_F_0	BY918703
E_ET_F1mg_23D08_R_0	BY926999
E_ET_F1mg_23D09_F_0	BY918704
E_ET_F1mg_23D09_R_0	BY927000
E_ET_F1mg_23D10_F_0	BY918705
E_ET_F1mg_23D10_R_0	BY927001
E_ET_F1mg_23D11_F_0	BY918706
E_ET_F1mg_23D11_R_0	BY927002
E_ET_F1mg_23D12_F_0	BY918707
E_ET_F1mg_23D12_R_0	BY927003
E_ET_F1mg_23E02_F_0	BY918708
E_ET_F1mg_23E02_R_0	BY927004
E_ET_F1mg_23E03_F_0	BY918709
E_ET_F1mg_23E03_R_0	BY927005
E_ET_F1mg_23E04_F_0	BY918710
E_ET_F1mg_23E04_R_0	BY927006
E_ET_F1mg_23E05_F_0	BY918711
E_ET_F1mg_23E05_R_0	BY927007
E_ET_F1mg_23E06_F_0	BY918712
E_ET_F1mg_23E06_R_0	BY927008
E_ET_F1mg_23E07_F_0	BY918713
E_ET_F1mg_23E07_R_0	BY927009
E_ET_F1mg_23E08_F_0	BY918714
E_ET_F1mg_23E08_R_0	BY927010
E_ET_F1mg_23E09_F_0	BY918715
E_ET_F1mg_23E09_R_0	BY927011
E_ET_F1mg_23E10_F_0	BY918716
E_ET_F1mg_23E10_R_0	BY927012
E_ET_F1mg_23E11_F_0	BY918717
E_ET_F1mg_23E11_R_0	BY927013
E_ET_F1mg_23E12_F_0	BY918718
E_ET_F1mg_23F02_F_0	BY918719
E_ET_F1mg_23F02_R_0	BY927014
E_ET_F1mg_23F03_F_0	BY918720

E_ET_F1mg_23F03_R_0	BY927015
E_ET_F1mg_23F06_F_0	BY918721
E_ET_F1mg_23F09_F_0	BY918722
E_ET_F1mg_23F09_R_0	BY927016
E_ET_F1mg_23F11_F_0	BY918723
E_ET_F1mg_23F11_R_0	BY927017
E_ET_F1mg_23F12_F_0	BY918724
E_ET_F1mg_23F12_R_0	BY927018
E_ET_F1mg_23G02_F_0	BY918725
E_ET_F1mg_23G02_R_0	BY927019
E_ET_F1mg_23G04_F_0	BY918726
E_ET_F1mg_23G04_R_0	BY927020
E_ET_F1mg_23G08_F_0	BY918727
E_ET_F1mg_23G08_R_0	BY927021
E_ET_F1mg_23G09_F_0	BY918728
E_ET_F1mg_23G09_R_0	BY927022
E_ET_F1mg_23G10_F_0	BY918729
E_ET_F1mg_23G10_R_0	BY927023
E_ET_F1mg_23G11_F_0	BY918730
E_ET_F1mg_23G12_F_0	BY918731
E_ET_F1mg_23H03_F_0	BY918732
E_ET_F1mg_23H03_R_0	BY927024
E_ET_F1mg_23H04_F_0	BY918733
E_ET_F1mg_23H06_F_0	BY918734
E_ET_F1mg_23H06_R_0	BY927025
E_ET_F1mg_23H07_F_0	BY918735
E_ET_F1mg_23H07_R_0	BY927026
E_ET_F1mg_23H08_F_0	BY918736
E_ET_F1mg_23H08_R_0	BY927027
E_ET_F1mg_23H09_F_0	BY918737
E_ET_F1mg_23H11_F_0	BY918738
E_ET_F1mg_23H11_R_0	BY927028
E_ET_F1mg_24A04_F_0	BY918739
E_ET_F1mg_24A04_R_0	BY927029
E_ET_F1mg_24A05_F_0	BY918740
E_ET_F1mg_24A05_R_0	BY927030
E_ET_F1mg_24A06_F_0	BY918741
E_ET_F1mg_24A06_R_0	BY927031
E_ET_F1mg_24A07_F_0	BY918742
E_ET_F1mg_24A07_R_0	BY927032
E_ET_F1mg_24A08_F_0	BY918743
E_ET_F1mg_24A08_R_0	BY927033
E_ET_F1mg_24A09_F_0	BY918744
E_ET_F1mg_24A09_R_0	BY927034
E_ET_F1mg_24A11_F_0	BY918745
E_ET_F1mg_24A11_R_0	BY927035
E_ET_F1mg_24B01_F_0	BY918746
E_ET_F1mg_24B01_R_0	BY927036
E_ET_F1mg_24B04_F_0	BY918747
E_ET_F1mg_24B08_F_0	BY918748
E_ET_F1mg_24B08_R_0	BY927037
E_ET_F1mg_24B09_F_0	BY918749
E_ET_F1mg_24C06_F_0	BY918750
E_ET_F1mg_24C06_R_0	BY927038

E_ET_F1mg_24C07_R_0	BY927039
E_ET_F1mg_24C08_R_0	BY927040
E_ET_F1mg_24C10_R_0	BY927041
E_ET_F1mg_24C11_F_0	BY918751
E_ET_F1mg_24D02_F_0	BY918752
E_ET_F1mg_24D08_F_0	BY918753
E_ET_F1mg_24D08_R_0	BY927042
E_ET_F1mg_24D09_F_0	BY918754
E_ET_F1mg_24D09_R_0	BY927043
E_ET_F1mg_24D10_F_0	BY918755
E_ET_F1mg_24D10_R_0	BY927044
E_ET_F1mg_24E02_F_0	BY918756
E_ET_F1mg_24E03_F_0	BY918757
E_ET_F1mg_24E03_R_0	BY927045
E_ET_F1mg_24E07_F_0	BY918758
E_ET_F1mg_24E07_R_0	BY927046
E_ET_F1mg_24E08_F_0	BY918759
E_ET_F1mg_24E08_R_0	BY927047
E_ET_F1mg_24E10_F_0	BY918760
E_ET_F1mg_24E10_R_0	BY927048
E_ET_F1mg_24E11_F_0	BY918761
E_ET_F1mg_24E11_R_0	BY927049
E_ET_F1mg_24F03_F_0	BY918762
E_ET_F1mg_24F04_F_0	BY918763
E_ET_F1mg_24F04_R_0	BY927050
E_ET_F1mg_24F05_F_0	BY918764
E_ET_F1mg_24F05_R_0	BY927051
E_ET_F1mg_24F06_F_0	BY918765
E_ET_F1mg_24F06_R_0	BY927052
E_ET_F1mg_24F07_F_0	BY918766
E_ET_F1mg_24F07_R_0	BY927053
E_ET_F1mg_24F09_F_0	BY918767
E_ET_F1mg_24F09_R_0	BY927054
E_ET_F1mg_24F10_F_0	BY918768
E_ET_F1mg_24F10_R_0	BY927055
E_ET_F1mg_24F11_F_0	BY918769
E_ET_F1mg_24F11_R_0	BY927056
E_ET_F1mg_24F12_F_0	BY918770
E_ET_F1mg_24F12_R_0	BY927057
E_ET_F1mg_24G01_F_0	BY918771
E_ET_F1mg_24G01_R_0	BY927058
E_ET_F1mg_24G02_F_0	BY918772
E_ET_F1mg_24G02_R_0	BY927059
E_ET_F1mg_24G03_F_0	BY918773
E_ET_F1mg_24G03_R_0	BY927060
E_ET_F1mg_24G04_F_0	BY918774
E_ET_F1mg_24G04_R_0	BY927061
E_ET_F1mg_24G07_F_0	BY918775
E_ET_F1mg_24G08_F_0	BY918776
E_ET_F1mg_24G08_R_0	BY927062
E_ET_F1mg_24G09_F_0	BY918777
E_ET_F1mg_24G09_R_0	BY927063
E_ET_F1mg_24G10_F_0	BY918778
E_ET_F1mg_24G10_R_0	BY927064

	E_ET_F1mg_24H01_F_0	BY918779
	E_ET_F1mg_24H01_R_0	BY927065
	E_ET_F1mg_24H02_F_0	BY918780
	E_ET_F1mg_24H02_R_0	BY927066
	E_ET_F1mg_24H03_F_0	BY918781
	E_ET_F1mg_24H04_F_0	BY918782
	E_ET_F1mg_24H04_R_0	BY927067
	E_ET_F1mg_24H06_F_0	BY918783
	E_ET_F1mg_24H06_R_0	BY927068
	E_ET_F1mg_24H08_F_0	BY918784
	E_ET_F1mg_24H08_R_0	BY927069
	E_ET_F1mg_24H09_R_0	BY927070
	E_ET_F1mg_24H11_F_0	BY918785
	E_ET_F1mg_24H11_R_0	BY927071
FJsb	E_ET_FJsb_01A01_F_0	BY920389
	E_ET_FJsb_01A01_R_0	BY928826
	E_ET_FJsb_01A03_F_0	BY920390
	E_ET_FJsb_01A03_R_0	BY928827
	E_ET_FJsb_01A04_F_0	BY920391
	E_ET_FJsb_01A04_R_0	BY928828
	E_ET_FJsb_01A07_F_0	BY920392
	E_ET_FJsb_01A07_R_0	BY928829
	E_ET_FJsb_01A08_F_0	BY920393
	E_ET_FJsb_01A08_R_0	BY928830
	E_ET_FJsb_01A10_F_0	BY920394
	E_ET_FJsb_01A10_R_0	BY928831
	E_ET_FJsb_01A12_F_0	BY920395
	E_ET_FJsb_01A12_R_0	BY928832
	E_ET_FJsb_01B02_F_0	BY920396
	E_ET_FJsb_01B02_R_0	BY928833
	E_ET_FJsb_01B03_R_0	BY928834
	E_ET_FJsb_01B06_F_0	BY920397
	E_ET_FJsb_01B06_R_0	BY928835
	E_ET_FJsb_01B08_F_0	BY920398
	E_ET_FJsb_01B08_R_0	BY928836
	E_ET_FJsb_01B09_F_0	BY920399
	E_ET_FJsb_01B09_R_0	BY928837
	E_ET_FJsb_01B10_R_0	BY928838
	E_ET_FJsb_01B12_F_0	BY920400
	E_ET_FJsb_01B12_R_0	BY928839
	E_ET_FJsb_01C01_R_0	BY928840
	E_ET_FJsb_01C02_F_0	BY920401
	E_ET_FJsb_01C02_R_0	BY928841
	E_ET_FJsb_01C04_F_0	BY920402
	E_ET_FJsb_01C04_R_0	BY928842
	E_ET_FJsb_01C05_F_0	BY920403
	E_ET_FJsb_01C05_R_0	BY928843
	E_ET_FJsb_01C07_F_0	BY920404
	E_ET_FJsb_01C07_R_0	BY928844
	E_ET_FJsb_01D01_F_0	BY920405
	E_ET_FJsb_01D01_R_0	BY928845
	E_ET_FJsb_01D03_F_0	BY920406
	E_ET_FJsb_01D03_R_0	BY928846
	E_ET_FJsb_01D05_R_0	BY928847

E_ET_FJsb_01D06_F_0	BY920407
E_ET_FJsb_01D06_R_0	BY928848
E_ET_FJsb_01D07_F_0	BY920408
E_ET_FJsb_01D07_R_0	BY928849
E_ET_FJsb_01D09_R_0	BY928850
E_ET_FJsb_01D11_F_0	BY920409
E_ET_FJsb_01D11_R_0	BY928851
E_ET_FJsb_01E02_F_0	BY920410
E_ET_FJsb_01E02_R_0	BY928852
E_ET_FJsb_01E03_F_0	BY920411
E_ET_FJsb_01E03_R_0	BY928853
E_ET_FJsb_01E06_F_0	BY920412
E_ET_FJsb_01E06_R_0	BY928854
E_ET_FJsb_01E07_F_0	BY920413
E_ET_FJsb_01E07_R_0	BY928855
E_ET_FJsb_01E08_F_0	BY920414
E_ET_FJsb_01E08_R_0	BY928856
E_ET_FJsb_01E09_F_0	BY920415
E_ET_FJsb_01E09_R_0	BY928857
E_ET_FJsb_01E12_F_0	BY920416
E_ET_FJsb_01E12_R_0	BY928858
E_ET_FJsb_01F01_F_0	BY920417
E_ET_FJsb_01F01_R_0	BY928859
E_ET_FJsb_01F02_F_0	BY920418
E_ET_FJsb_01F02_R_0	BY928860
E_ET_FJsb_01F04_F_0	BY920419
E_ET_FJsb_01F04_R_0	BY928861
E_ET_FJsb_01F05_F_0	BY920420
E_ET_FJsb_01F05_R_0	BY928862
E_ET_FJsb_01F06_F_0	BY920421
E_ET_FJsb_01F06_R_0	BY928863
E_ET_FJsb_01F08_F_0	BY920422
E_ET_FJsb_01F08_R_0	BY928864
E_ET_FJsb_01F09_F_0	BY920423
E_ET_FJsb_01F09_R_0	BY928865
E_ET_FJsb_01F10_F_0	BY920424
E_ET_FJsb_01F10_R_0	BY928866
E_ET_FJsb_01F11_R_0	BY928867
E_ET_FJsb_01F12_F_0	BY920425
E_ET_FJsb_01F12_R_0	BY928868
E_ET_FJsb_01G04_F_0	BY920426
E_ET_FJsb_01G04_R_0	BY928869
E_ET_FJsb_01G05_F_0	BY920427
E_ET_FJsb_01G05_R_0	BY928870
E_ET_FJsb_01G08_R_0	BY928871
E_ET_FJsb_01G09_F_0	BY920428
E_ET_FJsb_01G09_R_0	BY928872
E_ET_FJsb_01G10_F_0	BY920429
E_ET_FJsb_01G10_R_0	BY928873
E_ET_FJsb_01G11_F_0	BY920430
E_ET_FJsb_01H03_F_0	BY920431
E_ET_FJsb_01H03_R_0	BY928874
E_ET_FJsb_01H07_F_0	BY920432
E_ET_FJsb_01H07_R_0	BY928875

E_ET_FJsb_01H08_R_0	BY928876
E_ET_FJsb_01H10_F_0	BY920433
E_ET_FJsb_01H10_R_0	BY928877
E_ET_FJsb_01H12_F_0	BY920434
E_ET_FJsb_01H12_R_0	BY928878
E_ET_FJsb_02A01_F_0	BY920435
E_ET_FJsb_02A01_R_0	BY928879
E_ET_FJsb_02A02_F_0	BY920436
E_ET_FJsb_02A02_R_0	BY928880
E_ET_FJsb_02A03_F_0	BY920437
E_ET_FJsb_02A03_R_0	BY928881
E_ET_FJsb_02A04_F_0	BY920438
E_ET_FJsb_02A04_R_0	BY928882
E_ET_FJsb_02A05_F_0	BY920439
E_ET_FJsb_02A05_R_0	BY928883
E_ET_FJsb_02A06_F_0	BY920440
E_ET_FJsb_02A06_R_0	BY928884
E_ET_FJsb_02A07_F_0	BY920441
E_ET_FJsb_02A07_R_0	BY928885
E_ET_FJsb_02A09_F_0	BY920442
E_ET_FJsb_02A09_R_0	BY928886
E_ET_FJsb_02A10_F_0	BY920443
E_ET_FJsb_02A10_R_0	BY928887
E_ET_FJsb_02A12_F_0	BY920444
E_ET_FJsb_02A12_R_0	BY928888
E_ET_FJsb_02B01_F_0	BY920445
E_ET_FJsb_02B01_R_0	BY928889
E_ET_FJsb_02B02_F_0	BY920446
E_ET_FJsb_02B08_F_0	BY920447
E_ET_FJsb_02B08_R_0	BY928890
E_ET_FJsb_02B09_F_0	BY920448
E_ET_FJsb_02B09_R_0	BY928891
E_ET_FJsb_02B10_F_0	BY920449
E_ET_FJsb_02B10_R_0	BY928892
E_ET_FJsb_02B11_F_0	BY920450
E_ET_FJsb_02B11_R_0	BY928893
E_ET_FJsb_02C02_F_0	BY920451
E_ET_FJsb_02C02_R_0	BY928894
E_ET_FJsb_02C05_F_0	BY920452
E_ET_FJsb_02C05_R_0	BY928895
E_ET_FJsb_02C07_F_0	BY920453
E_ET_FJsb_02C07_R_0	BY928896
E_ET_FJsb_02C08_F_0	BY920454
E_ET_FJsb_02C08_R_0	BY928897
E_ET_FJsb_02C09_F_0	BY920455
E_ET_FJsb_02C09_R_0	BY928898
E_ET_FJsb_02C10_F_0	BY920456
E_ET_FJsb_02C10_R_0	BY928899
E_ET_FJsb_02C11_F_0	BY920457
E_ET_FJsb_02C11_R_0	BY928900
E_ET_FJsb_02D01_F_0	BY920458
E_ET_FJsb_02D01_R_0	BY928901
E_ET_FJsb_02D02_F_0	BY920459
E_ET_FJsb_02D02_R_0	BY928902

E_ET_FJsb_02D05_F_0	BY920460
E_ET_FJsb_02D05_R_0	BY928903
E_ET_FJsb_02D06_F_0	BY920461
E_ET_FJsb_02D07_F_0	BY920462
E_ET_FJsb_02D07_R_0	BY928904
E_ET_FJsb_02D09_F_0	BY920463
E_ET_FJsb_02D09_R_0	BY928905
E_ET_FJsb_02E02_F_0	BY920464
E_ET_FJsb_02E02_R_0	BY928906
E_ET_FJsb_02E03_F_0	BY920465
E_ET_FJsb_02E03_R_0	BY928907
E_ET_FJsb_02E04_R_0	BY928908
E_ET_FJsb_02E06_F_0	BY920466
E_ET_FJsb_02E08_R_0	BY928909
E_ET_FJsb_02E09_F_0	BY920467
E_ET_FJsb_02E09_R_0	BY928910
E_ET_FJsb_02E12_F_0	BY920468
E_ET_FJsb_02E12_R_0	BY928911
E_ET_FJsb_02F01_F_0	BY920469
E_ET_FJsb_02F01_R_0	BY928912
E_ET_FJsb_02F02_R_0	BY928913
E_ET_FJsb_02F04_F_0	BY920470
E_ET_FJsb_02F04_R_0	BY928914
E_ET_FJsb_02F06_F_0	BY920471
E_ET_FJsb_02F06_R_0	BY928915
E_ET_FJsb_02F07_F_0	BY920472
E_ET_FJsb_02F07_R_0	BY928916
E_ET_FJsb_02F10_F_0	BY920473
E_ET_FJsb_02F10_R_0	BY928917
E_ET_FJsb_02F12_F_0	BY920474
E_ET_FJsb_02F12_R_0	BY928918
E_ET_FJsb_02G02_F_0	BY920475
E_ET_FJsb_02G02_R_0	BY928919
E_ET_FJsb_02G04_F_0	BY920476
E_ET_FJsb_02G04_R_0	BY928920
E_ET_FJsb_02G07_F_0	BY920477
E_ET_FJsb_02G07_R_0	BY928921
E_ET_FJsb_02G08_F_0	BY920478
E_ET_FJsb_02G08_R_0	BY928922
E_ET_FJsb_02G10_R_0	BY928923
E_ET_FJsb_02G11_F_0	BY920479
E_ET_FJsb_02G11_R_0	BY928924
E_ET_FJsb_02G12_F_0	BY920480
E_ET_FJsb_02G12_R_0	BY928925
E_ET_FJsb_02H01_F_0	BY920481
E_ET_FJsb_02H01_R_0	BY928926
E_ET_FJsb_02H04_R_0	BY928927
E_ET_FJsb_02H07_F_0	BY920482
E_ET_FJsb_02H07_R_0	BY928928
E_ET_FJsb_02H09_F_0	BY920483
E_ET_FJsb_02H09_R_0	BY928929
E_ET_FJsb_02H10_F_0	BY920484
E_ET_FJsb_02H10_R_0	BY928930
E_ET_FJsb_02H11_F_0	BY920485

E_ET_FJsb_02H11_R_0	BY928931
E_ET_FJsb_02H12_F_0	BY920486
E_ET_FJsb_02H12_R_0	BY928932
E_ET_FJsb_03A02_F_0	BY920487
E_ET_FJsb_03A02_R_0	BY928933
E_ET_FJsb_03A06_R_0	BY928934
E_ET_FJsb_03A07_F_0	BY920488
E_ET_FJsb_03A07_R_0	BY928935
E_ET_FJsb_03A09_F_0	BY920489
E_ET_FJsb_03A09_R_0	BY928936
E_ET_FJsb_03A10_F_0	BY920490
E_ET_FJsb_03A10_R_0	BY928937
E_ET_FJsb_03A11_F_0	BY920491
E_ET_FJsb_03A11_R_0	BY928938
E_ET_FJsb_03A12_F_0	BY920492
E_ET_FJsb_03A12_R_0	BY928939
E_ET_FJsb_03B01_F_0	BY920493
E_ET_FJsb_03B01_R_0	BY928940
E_ET_FJsb_03B02_F_0	BY920494
E_ET_FJsb_03B02_R_0	BY928941
E_ET_FJsb_03B03_F_0	BY920495
E_ET_FJsb_03B03_R_0	BY928942
E_ET_FJsb_03B04_F_0	BY920496
E_ET_FJsb_03B04_R_0	BY928943
E_ET_FJsb_03B05_F_0	BY920497
E_ET_FJsb_03B05_R_0	BY928944
E_ET_FJsb_03B06_F_0	BY920498
E_ET_FJsb_03B06_R_0	BY928945
E_ET_FJsb_03B07_F_0	BY920499
E_ET_FJsb_03B07_R_0	BY928946
E_ET_FJsb_03B08_F_0	BY920500
E_ET_FJsb_03B08_R_0	BY928947
E_ET_FJsb_03B09_F_0	BY920501
E_ET_FJsb_03B09_R_0	BY928948
E_ET_FJsb_03B10_F_0	BY920502
E_ET_FJsb_03B10_R_0	BY928949
E_ET_FJsb_03C02_F_0	BY920503
E_ET_FJsb_03C02_R_0	BY928950
E_ET_FJsb_03C03_F_0	BY920504
E_ET_FJsb_03C03_R_0	BY928951
E_ET_FJsb_03C04_F_0	BY920505
E_ET_FJsb_03C04_R_0	BY928952
E_ET_FJsb_03C06_F_0	BY920506
E_ET_FJsb_03C06_R_0	BY928953
E_ET_FJsb_03C10_F_0	BY920507
E_ET_FJsb_03C10_R_0	BY928954
E_ET_FJsb_03D01_F_0	BY920508
E_ET_FJsb_03D01_R_0	BY928955
E_ET_FJsb_03D02_F_0	BY920509
E_ET_FJsb_03D02_R_0	BY928956
E_ET_FJsb_03D04_F_0	BY920510
E_ET_FJsb_03D04_R_0	BY928957
E_ET_FJsb_03D06_F_0	BY920511
E_ET_FJsb_03D06_R_0	BY928958

E_ET_FJsb_03D11_F_0	BY920512
E_ET_FJsb_03D11_R_0	BY928959
E_ET_FJsb_03D12_F_0	BY920513
E_ET_FJsb_03D12_R_0	BY928960
E_ET_FJsb_03E01_F_0	BY920514
E_ET_FJsb_03E01_R_0	BY928961
E_ET_FJsb_03E03_F_0	BY920515
E_ET_FJsb_03E03_R_0	BY928962
E_ET_FJsb_03E04_R_0	BY928963
E_ET_FJsb_03E05_F_0	BY920516
E_ET_FJsb_03E05_R_0	BY928964
E_ET_FJsb_03E06_R_0	BY928965
E_ET_FJsb_03E07_F_0	BY920517
E_ET_FJsb_03E08_F_0	BY920518
E_ET_FJsb_03E08_R_0	BY928966
E_ET_FJsb_03E09_F_0	BY920519
E_ET_FJsb_03E09_R_0	BY928967
E_ET_FJsb_03E11_F_0	BY920520
E_ET_FJsb_03E11_R_0	BY928968
E_ET_FJsb_03F04_F_0	BY920521
E_ET_FJsb_03F04_R_0	BY928969
E_ET_FJsb_03F09_F_0	BY920522
E_ET_FJsb_03F09_R_0	BY928970
E_ET_FJsb_03F11_R_0	BY928971
E_ET_FJsb_03F12_F_0	BY920523
E_ET_FJsb_03F12_R_0	BY928972
E_ET_FJsb_03G02_F_0	BY920524
E_ET_FJsb_03G02_R_0	BY928973
E_ET_FJsb_03G03_F_0	BY920525
E_ET_FJsb_03G03_R_0	BY928974
E_ET_FJsb_03G04_F_0	BY920526
E_ET_FJsb_03G04_R_0	BY928975
E_ET_FJsb_03G08_F_0	BY920527
E_ET_FJsb_03G08_R_0	BY928976
E_ET_FJsb_03G10_F_0	BY920528
E_ET_FJsb_03G10_R_0	BY928977
E_ET_FJsb_03H03_F_0	BY920529
E_ET_FJsb_03H03_R_0	BY928978
E_ET_FJsb_03H05_F_0	BY920530
E_ET_FJsb_03H05_R_0	BY928979
E_ET_FJsb_03H08_F_0	BY920531
E_ET_FJsb_03H08_R_0	BY928980
E_ET_FJsb_03H10_F_0	BY920532
E_ET_FJsb_03H10_R_0	BY928981
E_ET_FJsb_04A05_F_0	BY920533
E_ET_FJsb_04A05_R_0	BY928982
E_ET_FJsb_04A06_F_0	BY920534
E_ET_FJsb_04A06_R_0	BY928983
E_ET_FJsb_04A08_R_0	BY928984
E_ET_FJsb_04A09_F_0	BY920535
E_ET_FJsb_04A09_R_0	BY928985
E_ET_FJsb_04A10_F_0	BY920536
E_ET_FJsb_04A10_R_0	BY928986
E_ET_FJsb_04A11_R_0	BY928987

E_ET_FJsb_04A12_F_0	BY920537
E_ET_FJsb_04A12_R_0	BY928988
E_ET_FJsb_04B06_F_0	BY920538
E_ET_FJsb_04B06_R_0	BY928989
E_ET_FJsb_04B07_F_0	BY920539
E_ET_FJsb_04B07_R_0	BY928990
E_ET_FJsb_04B08_F_0	BY920540
E_ET_FJsb_04B08_R_0	BY928991
E_ET_FJsb_04B09_F_0	BY920541
E_ET_FJsb_04B09_R_0	BY928992
E_ET_FJsb_04B11_F_0	BY920542
E_ET_FJsb_04B11_R_0	BY928993
E_ET_FJsb_04B12_F_0	BY920543
E_ET_FJsb_04C03_F_0	BY920544
E_ET_FJsb_04C03_R_0	BY928994
E_ET_FJsb_04C06_F_0	BY920545
E_ET_FJsb_04C06_R_0	BY928995
E_ET_FJsb_04C09_F_0	BY920546
E_ET_FJsb_04C09_R_0	BY928996
E_ET_FJsb_04C12_F_0	BY920547
E_ET_FJsb_04C12_R_0	BY928997
E_ET_FJsb_04D01_F_0	BY920548
E_ET_FJsb_04D01_R_0	BY928998
E_ET_FJsb_04D02_F_0	BY920549
E_ET_FJsb_04D09_F_0	BY920550
E_ET_FJsb_04D09_R_0	BY928999
E_ET_FJsb_04D12_F_0	BY920551
E_ET_FJsb_04D12_R_0	BY929000
E_ET_FJsb_04E01_F_0	BY920552
E_ET_FJsb_04E02_F_0	BY920553
E_ET_FJsb_04E02_R_0	BY929001
E_ET_FJsb_04E03_F_0	BY920554
E_ET_FJsb_04E03_R_0	BY929002
E_ET_FJsb_04E04_F_0	BY920555
E_ET_FJsb_04E04_R_0	BY929003
E_ET_FJsb_04E05_F_0	BY920556
E_ET_FJsb_04E08_R_0	BY929004
E_ET_FJsb_04F01_F_0	BY920557
E_ET_FJsb_04F02_F_0	BY920558
E_ET_FJsb_04F02_R_0	BY929005
E_ET_FJsb_04F03_F_0	BY920559
E_ET_FJsb_04F03_R_0	BY929006
E_ET_FJsb_04F04_F_0	BY920560
E_ET_FJsb_04F04_R_0	BY929007
E_ET_FJsb_04F05_F_0	BY920561
E_ET_FJsb_04F05_R_0	BY929008
E_ET_FJsb_04F06_F_0	BY920562
E_ET_FJsb_04F06_R_0	BY929009
E_ET_FJsb_04F07_F_0	BY920563
E_ET_FJsb_04F07_R_0	BY929010
E_ET_FJsb_04F08_F_0	BY920564
E_ET_FJsb_04F09_F_0	BY920565
E_ET_FJsb_04F09_R_0	BY929011
E_ET_FJsb_04G02_F_0	BY920566

E_ET_FJsb_04G02_R_0	BY929012
E_ET_FJsb_04G03_R_0	BY929013
E_ET_FJsb_04G05_F_0	BY920567
E_ET_FJsb_04G05_R_0	BY929014
E_ET_FJsb_04G08_F_0	BY920568
E_ET_FJsb_04G08_R_0	BY929015
E_ET_FJsb_04G09_F_0	BY920569
E_ET_FJsb_04G09_R_0	BY929016
E_ET_FJsb_04G10_F_0	BY920570
E_ET_FJsb_04G10_R_0	BY929017
E_ET_FJsb_04H02_F_0	BY920571
E_ET_FJsb_04H02_R_0	BY929018
E_ET_FJsb_04H03_F_0	BY920572
E_ET_FJsb_04H03_R_0	BY929019
E_ET_FJsb_04H06_R_0	BY929020
E_ET_FJsb_04H07_F_0	BY920573
E_ET_FJsb_04H07_R_0	BY929021
E_ET_FJsb_04H08_R_0	BY929022
E_ET_FJsb_04H10_F_0	BY920574
E_ET_FJsb_04H10_R_0	BY929023
E_ET_FJsb_04H11_F_0	BY920575
E_ET_FJsb_04H11_R_0	BY929024
E_ET_FJsb_05A04_F_0	BY920576
E_ET_FJsb_05A04_R_0	BY929025
E_ET_FJsb_05A05_F_0	BY920577
E_ET_FJsb_05A05_R_0	BY929026
E_ET_FJsb_05A06_F_0	BY920578
E_ET_FJsb_05A06_R_0	BY929027
E_ET_FJsb_05A08_F_0	BY920579
E_ET_FJsb_05A08_R_0	BY929028
E_ET_FJsb_05A11_F_0	BY920580
E_ET_FJsb_05A11_R_0	BY929029
E_ET_FJsb_05A12_F_0	BY920581
E_ET_FJsb_05A12_R_0	BY929030
E_ET_FJsb_05B02_F_0	BY920582
E_ET_FJsb_05B02_R_0	BY929031
E_ET_FJsb_05B03_F_0	BY920583
E_ET_FJsb_05B03_R_0	BY929032
E_ET_FJsb_05B04_F_0	BY920584
E_ET_FJsb_05B04_R_0	BY929033
E_ET_FJsb_05B07_F_0	BY920585
E_ET_FJsb_05B07_R_0	BY929034
E_ET_FJsb_05B08_F_0	BY920586
E_ET_FJsb_05B08_R_0	BY929035
E_ET_FJsb_05B10_F_0	BY920587
E_ET_FJsb_05B10_R_0	BY929036
E_ET_FJsb_05B11_F_0	BY920588
E_ET_FJsb_05B11_R_0	BY929037
E_ET_FJsb_05C01_F_0	BY920589
E_ET_FJsb_05C01_R_0	BY929038
E_ET_FJsb_05C02_R_0	BY929039
E_ET_FJsb_05C03_F_0	BY920590
E_ET_FJsb_05C03_R_0	BY929040
E_ET_FJsb_05C04_F_0	BY920591

E_ET_FJsb_05C04_R_0	BY929041
E_ET_FJsb_05C05_F_0	BY920592
E_ET_FJsb_05C05_R_0	BY929042
E_ET_FJsb_05C06_F_0	BY920593
E_ET_FJsb_05C06_R_0	BY929043
E_ET_FJsb_05C07_F_0	BY920594
E_ET_FJsb_05C07_R_0	BY929044
E_ET_FJsb_05C08_F_0	BY920595
E_ET_FJsb_05C08_R_0	BY929045
E_ET_FJsb_05C09_F_0	BY920596
E_ET_FJsb_05C09_R_0	BY929046
E_ET_FJsb_05C10_F_0	BY920597
E_ET_FJsb_05C10_R_0	BY929047
E_ET_FJsb_05C12_F_0	BY920598
E_ET_FJsb_05C12_R_0	BY929048
E_ET_FJsb_05D01_R_0	BY929049
E_ET_FJsb_05D02_F_0	BY920599
E_ET_FJsb_05D02_R_0	BY929050
E_ET_FJsb_05D04_F_0	BY920600
E_ET_FJsb_05D04_R_0	BY929051
E_ET_FJsb_05D06_F_0	BY920601
E_ET_FJsb_05D06_R_0	BY929052
E_ET_FJsb_05D07_F_0	BY920602
E_ET_FJsb_05D07_R_0	BY929053
E_ET_FJsb_05D09_F_0	BY920603
E_ET_FJsb_05D09_R_0	BY929054
E_ET_FJsb_05D10_F_0	BY920604
E_ET_FJsb_05D10_R_0	BY929055
E_ET_FJsb_05D11_F_0	BY920605
E_ET_FJsb_05D11_R_0	BY929056
E_ET_FJsb_05E03_F_0	BY920606
E_ET_FJsb_05E03_R_0	BY929057
E_ET_FJsb_05E04_F_0	BY920607
E_ET_FJsb_05E04_R_0	BY929058
E_ET_FJsb_05E05_F_0	BY920608
E_ET_FJsb_05E05_R_0	BY929059
E_ET_FJsb_05E06_F_0	BY920609
E_ET_FJsb_05E06_R_0	BY929060
E_ET_FJsb_05E10_F_0	BY920610
E_ET_FJsb_05E10_R_0	BY929061
E_ET_FJsb_05E11_F_0	BY920611
E_ET_FJsb_05E11_R_0	BY929062
E_ET_FJsb_05E12_R_0	BY929063
E_ET_FJsb_05F02_F_0	BY920612
E_ET_FJsb_05F02_R_0	BY929064
E_ET_FJsb_05F03_F_0	BY920613
E_ET_FJsb_05F03_R_0	BY929065
E_ET_FJsb_05F06_F_0	BY920614
E_ET_FJsb_05F06_R_0	BY929066
E_ET_FJsb_05F07_F_0	BY920615
E_ET_FJsb_05F07_R_0	BY929067
E_ET_FJsb_05F08_R_0	BY929068
E_ET_FJsb_05F12_F_0	BY920616
E_ET_FJsb_05F12_R_0	BY929069

E_ET_FJsb_05G03_F_0	BY920617
E_ET_FJsb_05G03_R_0	BY929070
E_ET_FJsb_05G04_F_0	BY920618
E_ET_FJsb_05G04_R_0	BY929071
E_ET_FJsb_05G06_F_0	BY920619
E_ET_FJsb_05G06_R_0	BY929072
E_ET_FJsb_05G08_F_0	BY920620
E_ET_FJsb_05G08_R_0	BY929073
E_ET_FJsb_05G09_F_0	BY920621
E_ET_FJsb_05G09_R_0	BY929074
E_ET_FJsb_05G10_F_0	BY920622
E_ET_FJsb_05G10_R_0	BY929075
E_ET_FJsb_05G11_F_0	BY920623
E_ET_FJsb_05G11_R_0	BY929076
E_ET_FJsb_05H01_R_0	BY929077
E_ET_FJsb_05H03_R_0	BY929078
E_ET_FJsb_05H05_F_0	BY920624
E_ET_FJsb_05H05_R_0	BY929079
E_ET_FJsb_05H07_F_0	BY920625
E_ET_FJsb_05H07_R_0	BY929080
E_ET_FJsb_05H08_F_0	BY920626
E_ET_FJsb_05H08_R_0	BY929081
E_ET_FJsb_05H10_F_0	BY920627
E_ET_FJsb_05H10_R_0	BY929082
E_ET_FJsb_05H12_F_0	BY920628
E_ET_FJsb_05H12_R_0	BY929083
E_ET_FJsb_06A01_R_0	BY929084
E_ET_FJsb_06A02_F_0	BY920629
E_ET_FJsb_06A02_R_0	BY929085
E_ET_FJsb_06A04_F_0	BY920630
E_ET_FJsb_06A04_R_0	BY929086
E_ET_FJsb_06A07_F_0	BY920631
E_ET_FJsb_06A07_R_0	BY929087
E_ET_FJsb_06A08_R_0	BY929088
E_ET_FJsb_06A09_F_0	BY920632
E_ET_FJsb_06A09_R_0	BY929089
E_ET_FJsb_06A10_F_0	BY920633
E_ET_FJsb_06A10_R_0	BY929090
E_ET_FJsb_06A12_F_0	BY920634
E_ET_FJsb_06A12_R_0	BY929091
E_ET_FJsb_06B01_F_0	BY920635
E_ET_FJsb_06B01_R_0	BY929092
E_ET_FJsb_06B03_F_0	BY920636
E_ET_FJsb_06B03_R_0	BY929093
E_ET_FJsb_06B05_F_0	BY920637
E_ET_FJsb_06B05_R_0	BY929094
E_ET_FJsb_06B06_F_0	BY920638
E_ET_FJsb_06B06_R_0	BY929095
E_ET_FJsb_06B07_F_0	BY920639
E_ET_FJsb_06B09_F_0	BY920640
E_ET_FJsb_06B09_R_0	BY929096
E_ET_FJsb_06B10_F_0	BY920641
E_ET_FJsb_06B10_R_0	BY929097
E_ET_FJsb_06B11_F_0	BY920642

E_ET_FJsb_06B11_R_0	BY929098
E_ET_FJsb_06C01_F_0	BY920643
E_ET_FJsb_06C01_R_0	BY929099
E_ET_FJsb_06C04_F_0	BY920644
E_ET_FJsb_06C04_R_0	BY929100
E_ET_FJsb_06C05_F_0	BY920645
E_ET_FJsb_06C05_R_0	BY929101
E_ET_FJsb_06C07_F_0	BY920646
E_ET_FJsb_06C07_R_0	BY929102
E_ET_FJsb_06C08_F_0	BY920647
E_ET_FJsb_06C08_R_0	BY929103
E_ET_FJsb_06C09_F_0	BY920648
E_ET_FJsb_06C09_R_0	BY929104
E_ET_FJsb_06C12_F_0	BY920649
E_ET_FJsb_06C12_R_0	BY929105
E_ET_FJsb_06D05_F_0	BY920650
E_ET_FJsb_06D05_R_0	BY929106
E_ET_FJsb_06D06_F_0	BY920651
E_ET_FJsb_06D06_R_0	BY929107
E_ET_FJsb_06D07_F_0	BY920652
E_ET_FJsb_06D07_R_0	BY929108
E_ET_FJsb_06D08_F_0	BY920653
E_ET_FJsb_06D08_R_0	BY929109
E_ET_FJsb_06D10_F_0	BY920654
E_ET_FJsb_06D10_R_0	BY929110
E_ET_FJsb_06D11_F_0	BY920655
E_ET_FJsb_06D11_R_0	BY929111
E_ET_FJsb_06E03_F_0	BY920656
E_ET_FJsb_06E03_R_0	BY929112
E_ET_FJsb_06E04_F_0	BY920657
E_ET_FJsb_06E04_R_0	BY929113
E_ET_FJsb_06E05_F_0	BY920658
E_ET_FJsb_06E05_R_0	BY929114
E_ET_FJsb_06E06_F_0	BY920659
E_ET_FJsb_06E06_R_0	BY929115
E_ET_FJsb_06E08_F_0	BY920660
E_ET_FJsb_06E08_R_0	BY929116
E_ET_FJsb_06E09_F_0	BY920661
E_ET_FJsb_06E10_F_0	BY920662
E_ET_FJsb_06E10_R_0	BY929117
E_ET_FJsb_06E12_F_0	BY920663
E_ET_FJsb_06E12_R_0	BY929118
E_ET_FJsb_06F01_F_0	BY920664
E_ET_FJsb_06F02_F_0	BY920665
E_ET_FJsb_06F02_R_0	BY929119
E_ET_FJsb_06F04_F_0	BY920666
E_ET_FJsb_06F04_R_0	BY929120
E_ET_FJsb_06F05_F_0	BY920667
E_ET_FJsb_06F05_R_0	BY929121
E_ET_FJsb_06F07_F_0	BY920668
E_ET_FJsb_06F07_R_0	BY929122
E_ET_FJsb_06F08_R_0	BY929123
E_ET_FJsb_06F09_R_0	BY929124
E_ET_FJsb_06F10_F_0	BY920669

E_ET_FJsb_06F10_R_0	BY929125
E_ET_FJsb_06F12_F_0	BY920670
E_ET_FJsb_06F12_R_0	BY929126
E_ET_FJsb_06G01_F_0	BY920671
E_ET_FJsb_06G01_R_0	BY929127
E_ET_FJsb_06G02_F_0	BY920672
E_ET_FJsb_06G02_R_0	BY929128
E_ET_FJsb_06G03_F_0	BY920673
E_ET_FJsb_06G03_R_0	BY929129
E_ET_FJsb_06G12_F_0	BY920674
E_ET_FJsb_06G12_R_0	BY929130
E_ET_FJsb_06H01_F_0	BY920675
E_ET_FJsb_06H01_R_0	BY929131
E_ET_FJsb_06H02_F_0	BY920676
E_ET_FJsb_06H02_R_0	BY929132
E_ET_FJsb_06H03_F_0	BY920677
E_ET_FJsb_06H03_R_0	BY929133
E_ET_FJsb_06H05_F_0	BY920678
E_ET_FJsb_06H05_R_0	BY929134
E_ET_FJsb_06H06_F_0	BY920679
E_ET_FJsb_06H06_R_0	BY929135
E_ET_FJsb_06H07_F_0	BY920680
E_ET_FJsb_06H07_R_0	BY929136
E_ET_FJsb_06H08_F_0	BY920681
E_ET_FJsb_06H08_R_0	BY929137
E_ET_FJsb_06H10_F_0	BY920682
E_ET_FJsb_06H10_R_0	BY929138
E_ET_FJsb_06H11_F_0	BY920683
E_ET_FJsb_06H11_R_0	BY929139
E_ET_FJsb_07A01_R_0	BY929140
E_ET_FJsb_07A02_R_0	BY929141
E_ET_FJsb_07A03_F_0	BY920684
E_ET_FJsb_07A03_R_0	BY929142
E_ET_FJsb_07A04_R_0	BY929143
E_ET_FJsb_07A05_F_0	BY920685
E_ET_FJsb_07A05_R_0	BY929144
E_ET_FJsb_07A09_R_0	BY929145
E_ET_FJsb_07A10_F_0	BY920686
E_ET_FJsb_07A10_R_0	BY929146
E_ET_FJsb_07A11_F_0	BY920687
E_ET_FJsb_07A11_R_0	BY929147
E_ET_FJsb_07B01_R_0	BY929148
E_ET_FJsb_07B03_F_0	BY920688
E_ET_FJsb_07B03_R_0	BY929149
E_ET_FJsb_07B05_F_0	BY920689
E_ET_FJsb_07B07_F_0	BY920690
E_ET_FJsb_07B07_R_0	BY929150
E_ET_FJsb_07B08_F_0	BY920691
E_ET_FJsb_07B08_R_0	BY929151
E_ET_FJsb_07B10_F_0	BY920692
E_ET_FJsb_07B10_R_0	BY929152
E_ET_FJsb_07B11_F_0	BY920693
E_ET_FJsb_07B11_R_0	BY929153
E_ET_FJsb_07B12_F_0	BY920694

E_ET_FJsb_07B12_R_0	BY929154
E_ET_FJsb_07C01_F_0	BY920695
E_ET_FJsb_07C01_R_0	BY929155
E_ET_FJsb_07C03_R_0	BY929156
E_ET_FJsb_07C04_F_0	BY920696
E_ET_FJsb_07C04_R_0	BY929157
E_ET_FJsb_07C06_F_0	BY920697
E_ET_FJsb_07C06_R_0	BY929158
E_ET_FJsb_07C07_F_0	BY920698
E_ET_FJsb_07C07_R_0	BY929159
E_ET_FJsb_07C09_F_0	BY920699
E_ET_FJsb_07C09_R_0	BY929160
E_ET_FJsb_07C10_F_0	BY920700
E_ET_FJsb_07C10_R_0	BY929161
E_ET_FJsb_07C12_F_0	BY920701
E_ET_FJsb_07C12_R_0	BY929162
E_ET_FJsb_07D01_F_0	BY920702
E_ET_FJsb_07D01_R_0	BY929163
E_ET_FJsb_07D02_F_0	BY920703
E_ET_FJsb_07D02_R_0	BY929164
E_ET_FJsb_07D04_F_0	BY920704
E_ET_FJsb_07D04_R_0	BY929165
E_ET_FJsb_07D08_F_0	BY920705
E_ET_FJsb_07D08_R_0	BY929166
E_ET_FJsb_07D11_F_0	BY920706
E_ET_FJsb_07D11_R_0	BY929167
E_ET_FJsb_07E01_R_0	BY929168
E_ET_FJsb_07E03_F_0	BY920707
E_ET_FJsb_07E03_R_0	BY929169
E_ET_FJsb_07E04_F_0	BY920708
E_ET_FJsb_07E04_R_0	BY929170
E_ET_FJsb_07E08_F_0	BY920709
E_ET_FJsb_07E08_R_0	BY929171
E_ET_FJsb_07E09_F_0	BY920710
E_ET_FJsb_07E09_R_0	BY929172
E_ET_FJsb_07E11_F_0	BY920711
E_ET_FJsb_07E11_R_0	BY929173
E_ET_FJsb_07F02_F_0	BY920712
E_ET_FJsb_07F02_R_0	BY929174
E_ET_FJsb_07F05_R_0	BY929175
E_ET_FJsb_07F06_F_0	BY920713
E_ET_FJsb_07F06_R_0	BY929176
E_ET_FJsb_07F09_F_0	BY920714
E_ET_FJsb_07F09_R_0	BY929177
E_ET_FJsb_07F10_F_0	BY920715
E_ET_FJsb_07F10_R_0	BY929178
E_ET_FJsb_07G01_R_0	BY929179
E_ET_FJsb_07G02_F_0	BY920716
E_ET_FJsb_07G02_R_0	BY929180
E_ET_FJsb_07G04_F_0	BY920717
E_ET_FJsb_07G04_R_0	BY929181
E_ET_FJsb_07G07_F_0	BY920718
E_ET_FJsb_07G07_R_0	BY929182
E_ET_FJsb_07G08_F_0	BY920719

E_ET_FJsb_07G08_R_0	BY929183
E_ET_FJsb_07G09_F_0	BY920720
E_ET_FJsb_07G09_R_0	BY929184
E_ET_FJsb_07G11_F_0	BY920721
E_ET_FJsb_07G11_R_0	BY929185
E_ET_FJsb_07G12_F_0	BY920722
E_ET_FJsb_07G12_R_0	BY929186
E_ET_FJsb_07H01_F_0	BY920723
E_ET_FJsb_07H01_R_0	BY929187
E_ET_FJsb_07H03_F_0	BY920724
E_ET_FJsb_07H03_R_0	BY929188
E_ET_FJsb_07H06_F_0	BY920725
E_ET_FJsb_07H06_R_0	BY929189
E_ET_FJsb_07H07_F_0	BY920726
E_ET_FJsb_07H07_R_0	BY929190
E_ET_FJsb_07H09_F_0	BY920727
E_ET_FJsb_07H09_R_0	BY929191
E_ET_FJsb_08A01_R_0	BY929192
E_ET_FJsb_08A02_F_0	BY920728
E_ET_FJsb_08A02_R_0	BY929193
E_ET_FJsb_08A03_R_0	BY929194
E_ET_FJsb_08A04_R_0	BY929195
E_ET_FJsb_08A05_F_0	BY920729
E_ET_FJsb_08A05_R_0	BY929196
E_ET_FJsb_08A06_F_0	BY920730
E_ET_FJsb_08A06_R_0	BY929197
E_ET_FJsb_08A07_F_0	BY920731
E_ET_FJsb_08A07_R_0	BY929198
E_ET_FJsb_08A08_F_0	BY920732
E_ET_FJsb_08A09_R_0	BY929199
E_ET_FJsb_08A10_F_0	BY920733
E_ET_FJsb_08A10_R_0	BY929200
E_ET_FJsb_08A11_F_0	BY920734
E_ET_FJsb_08A11_R_0	BY929201
E_ET_FJsb_08B02_R_0	BY929202
E_ET_FJsb_08B05_F_0	BY920735
E_ET_FJsb_08B05_R_0	BY929203
E_ET_FJsb_08B06_F_0	BY920736
E_ET_FJsb_08B06_R_0	BY929204
E_ET_FJsb_08B07_F_0	BY920737
E_ET_FJsb_08B07_R_0	BY929205
E_ET_FJsb_08B08_F_0	BY920738
E_ET_FJsb_08B08_R_0	BY929206
E_ET_FJsb_08B11_F_0	BY920739
E_ET_FJsb_08B11_R_0	BY929207
E_ET_FJsb_08B12_F_0	BY920740
E_ET_FJsb_08C02_R_0	BY929208
E_ET_FJsb_08C05_F_0	BY920741
E_ET_FJsb_08C05_R_0	BY929209
E_ET_FJsb_08C06_F_0	BY920742
E_ET_FJsb_08C06_R_0	BY929210
E_ET_FJsb_08C07_F_0	BY920743
E_ET_FJsb_08C07_R_0	BY929211
E_ET_FJsb_08C10_R_0	BY929212

E_ET_FJsb_08D01_R_0	BY929213
E_ET_FJsb_08D03_F_0	BY920744
E_ET_FJsb_08D03_R_0	BY929214
E_ET_FJsb_08D07_F_0	BY920745
E_ET_FJsb_08D07_R_0	BY929215
E_ET_FJsb_08D08_F_0	BY920746
E_ET_FJsb_08D08_R_0	BY929216
E_ET_FJsb_08D09_F_0	BY920747
E_ET_FJsb_08D09_R_0	BY929217
E_ET_FJsb_08D11_F_0	BY920748
E_ET_FJsb_08D11_R_0	BY929218
E_ET_FJsb_08D12_F_0	BY920749
E_ET_FJsb_08D12_R_0	BY929219
E_ET_FJsb_08E02_R_0	BY929220
E_ET_FJsb_08E04_R_0	BY929221
E_ET_FJsb_08E07_F_0	BY920750
E_ET_FJsb_08E07_R_0	BY929222
E_ET_FJsb_08E10_F_0	BY920751
E_ET_FJsb_08E10_R_0	BY929223
E_ET_FJsb_08E11_R_0	BY929224
E_ET_FJsb_08E12_F_0	BY920752
E_ET_FJsb_08E12_R_0	BY929225
E_ET_FJsb_08F02_R_0	BY929226
E_ET_FJsb_08F04_F_0	BY920753
E_ET_FJsb_08F04_R_0	BY929227
E_ET_FJsb_08F05_F_0	BY920754
E_ET_FJsb_08F05_R_0	BY929228
E_ET_FJsb_08F06_F_0	BY920755
E_ET_FJsb_08F06_R_0	BY929229
E_ET_FJsb_08F07_F_0	BY920756
E_ET_FJsb_08F07_R_0	BY929230
E_ET_FJsb_08F08_F_0	BY920757
E_ET_FJsb_08F08_R_0	BY929231
E_ET_FJsb_08F10_F_0	BY920758
E_ET_FJsb_08F10_R_0	BY929232
E_ET_FJsb_08F11_F_0	BY920759
E_ET_FJsb_08F11_R_0	BY929233
E_ET_FJsb_08F12_F_0	BY920760
E_ET_FJsb_08G02_R_0	BY929234
E_ET_FJsb_08G04_F_0	BY920761
E_ET_FJsb_08G04_R_0	BY929235
E_ET_FJsb_08G05_F_0	BY920762
E_ET_FJsb_08G05_R_0	BY929236
E_ET_FJsb_08G11_F_0	BY920763
E_ET_FJsb_08H04_F_0	BY920764
E_ET_FJsb_08H04_R_0	BY929237
E_ET_FJsb_08H05_R_0	BY929238
E_ET_FJsb_08H09_F_0	BY920765
E_ET_FJsb_08H09_R_0	BY929239
E_ET_FJsb_09A02_F_0	BY920766
E_ET_FJsb_09A02_R_0	BY929240
E_ET_FJsb_09A03_F_0	BY920767
E_ET_FJsb_09A03_R_0	BY929241
E_ET_FJsb_09A05_F_0	BY920768

E_ET_FJsb_09A05_R_0	BY929242
E_ET_FJsb_09A06_F_0	BY920769
E_ET_FJsb_09A06_R_0	BY929243
E_ET_FJsb_09A09_F_0	BY920770
E_ET_FJsb_09A09_R_0	BY929244
E_ET_FJsb_09A10_F_0	BY920771
E_ET_FJsb_09A10_R_0	BY929245
E_ET_FJsb_09A11_F_0	BY920772
E_ET_FJsb_09A11_R_0	BY929246
E_ET_FJsb_09A12_F_0	BY920773
E_ET_FJsb_09A12_R_0	BY929247
E_ET_FJsb_09B01_F_0	BY920774
E_ET_FJsb_09B02_F_0	BY920775
E_ET_FJsb_09B02_R_0	BY929248
E_ET_FJsb_09B03_F_0	BY920776
E_ET_FJsb_09B03_R_0	BY929249
E_ET_FJsb_09B04_F_0	BY920777
E_ET_FJsb_09B04_R_0	BY929250
E_ET_FJsb_09B05_F_0	BY920778
E_ET_FJsb_09B05_R_0	BY929251
E_ET_FJsb_09B06_F_0	BY920779
E_ET_FJsb_09B06_R_0	BY929252
E_ET_FJsb_09B07_F_0	BY920780
E_ET_FJsb_09B07_R_0	BY929253
E_ET_FJsb_09B08_R_0	BY929254
E_ET_FJsb_09B09_F_0	BY920781
E_ET_FJsb_09B09_R_0	BY929255
E_ET_FJsb_09B11_F_0	BY920782
E_ET_FJsb_09B11_R_0	BY929256
E_ET_FJsb_09B12_F_0	BY920783
E_ET_FJsb_09B12_R_0	BY929257
E_ET_FJsb_09C02_F_0	BY920784
E_ET_FJsb_09C02_R_0	BY929258
E_ET_FJsb_09C05_F_0	BY920785
E_ET_FJsb_09C05_R_0	BY929259
E_ET_FJsb_09C06_R_0	BY929260
E_ET_FJsb_09C07_F_0	BY920786
E_ET_FJsb_09C07_R_0	BY929261
E_ET_FJsb_09C08_F_0	BY920787
E_ET_FJsb_09C09_F_0	BY920788
E_ET_FJsb_09C09_R_0	BY929262
E_ET_FJsb_09C10_F_0	BY920789
E_ET_FJsb_09C10_R_0	BY929263
E_ET_FJsb_09C12_F_0	BY920790
E_ET_FJsb_09D03_F_0	BY920791
E_ET_FJsb_09D03_R_0	BY929264
E_ET_FJsb_09D05_R_0	BY929265
E_ET_FJsb_09D07_F_0	BY920792
E_ET_FJsb_09D07_R_0	BY929266
E_ET_FJsb_09D10_F_0	BY920793
E_ET_FJsb_09D11_F_0	BY920794
E_ET_FJsb_09D11_R_0	BY929267
E_ET_FJsb_09E03_F_0	BY920795
E_ET_FJsb_09E03_R_0	BY929268

E_ET_FJsb_09E04_F_0	BY920796
E_ET_FJsb_09E04_R_0	BY929269
E_ET_FJsb_09E05_F_0	BY920797
E_ET_FJsb_09E05_R_0	BY929270
E_ET_FJsb_09E07_F_0	BY920798
E_ET_FJsb_09E07_R_0	BY929271
E_ET_FJsb_09E08_F_0	BY920799
E_ET_FJsb_09E08_R_0	BY929272
E_ET_FJsb_09E09_F_0	BY920800
E_ET_FJsb_09E09_R_0	BY929273
E_ET_FJsb_09E10_F_0	BY920801
E_ET_FJsb_09E10_R_0	BY929274
E_ET_FJsb_09F02_F_0	BY920802
E_ET_FJsb_09F02_R_0	BY929275
E_ET_FJsb_09F03_F_0	BY920803
E_ET_FJsb_09F03_R_0	BY929276
E_ET_FJsb_09F04_F_0	BY920804
E_ET_FJsb_09F04_R_0	BY929277
E_ET_FJsb_09F05_F_0	BY920805
E_ET_FJsb_09F05_R_0	BY929278
E_ET_FJsb_09F06_F_0	BY920806
E_ET_FJsb_09F06_R_0	BY929279
E_ET_FJsb_09F07_F_0	BY920807
E_ET_FJsb_09F07_R_0	BY929280
E_ET_FJsb_09F08_F_0	BY920808
E_ET_FJsb_09F10_F_0	BY920809
E_ET_FJsb_09F10_R_0	BY929281
E_ET_FJsb_09F11_F_0	BY920810
E_ET_FJsb_09F11_R_0	BY929282
E_ET_FJsb_09G01_F_0	BY920811
E_ET_FJsb_09G01_R_0	BY929283
E_ET_FJsb_09G04_R_0	BY929284
E_ET_FJsb_09G06_F_0	BY920812
E_ET_FJsb_09G06_R_0	BY929285
E_ET_FJsb_09G07_F_0	BY920813
E_ET_FJsb_09G07_R_0	BY929286
E_ET_FJsb_09G08_F_0	BY920814
E_ET_FJsb_09G08_R_0	BY929287
E_ET_FJsb_09G09_F_0	BY920815
E_ET_FJsb_09G09_R_0	BY929288
E_ET_FJsb_09G10_F_0	BY920816
E_ET_FJsb_09G10_R_0	BY929289
E_ET_FJsb_09G11_F_0	BY920817
E_ET_FJsb_09G11_R_0	BY929290
E_ET_FJsb_09H01_F_0	BY920818
E_ET_FJsb_09H01_R_0	BY929291
E_ET_FJsb_09H02_F_0	BY920819
E_ET_FJsb_09H02_R_0	BY929292
E_ET_FJsb_09H04_F_0	BY920820
E_ET_FJsb_09H04_R_0	BY929293
E_ET_FJsb_09H05_F_0	BY920821
E_ET_FJsb_09H05_R_0	BY929294
E_ET_FJsb_09H06_R_0	BY929295
E_ET_FJsb_09H09_F_0	BY920822

E_ET_FJsb_09H09_R_0	BY929296
E_ET_FJsb_09H10_F_0	BY920823
E_ET_FJsb_09H10_R_0	BY929297
E_ET_FJsb_09H11_F_0	BY920824
E_ET_FJsb_09H11_R_0	BY929298
E_ET_FJsb_09H12_F_0	BY920825
E_ET_FJsb_09H12_R_0	BY929299
E_ET_FJsb_10A01_F_0	BY920826
E_ET_FJsb_10A01_R_0	BY929300
E_ET_FJsb_10A02_F_0	BY920827
E_ET_FJsb_10A02_R_0	BY929301
E_ET_FJsb_10A03_F_0	BY920828
E_ET_FJsb_10A03_R_0	BY929302
E_ET_FJsb_10A04_F_0	BY920829
E_ET_FJsb_10A04_R_0	BY929303
E_ET_FJsb_10A07_F_0	BY920830
E_ET_FJsb_10A07_R_0	BY929304
E_ET_FJsb_10A08_F_0	BY920831
E_ET_FJsb_10A08_R_0	BY929305
E_ET_FJsb_10A09_F_0	BY920832
E_ET_FJsb_10A09_R_0	BY929306
E_ET_FJsb_10A10_F_0	BY920833
E_ET_FJsb_10A11_F_0	BY920834
E_ET_FJsb_10A11_R_0	BY929307
E_ET_FJsb_10A12_F_0	BY920835
E_ET_FJsb_10A12_R_0	BY929308
E_ET_FJsb_10B03_F_0	BY920836
E_ET_FJsb_10B03_R_0	BY929309
E_ET_FJsb_10B04_F_0	BY920837
E_ET_FJsb_10B04_R_0	BY929310
E_ET_FJsb_10B06_F_0	BY920838
E_ET_FJsb_10B06_R_0	BY929311
E_ET_FJsb_10B07_F_0	BY920839
E_ET_FJsb_10B07_R_0	BY929312
E_ET_FJsb_10B10_F_0	BY920840
E_ET_FJsb_10B10_R_0	BY929313
E_ET_FJsb_10B12_F_0	BY920841
E_ET_FJsb_10B12_R_0	BY929314
E_ET_FJsb_10C02_R_0	BY929315
E_ET_FJsb_10C05_R_0	BY929316
E_ET_FJsb_10C06_F_0	BY920842
E_ET_FJsb_10C06_R_0	BY929317
E_ET_FJsb_10C07_F_0	BY920843
E_ET_FJsb_10C07_R_0	BY929318
E_ET_FJsb_10C09_F_0	BY920844
E_ET_FJsb_10C09_R_0	BY929319
E_ET_FJsb_10C12_F_0	BY920845
E_ET_FJsb_10C12_R_0	BY929320
E_ET_FJsb_10D01_F_0	BY920846
E_ET_FJsb_10D01_R_0	BY929321
E_ET_FJsb_10D02_F_0	BY920847
E_ET_FJsb_10D02_R_0	BY929322
E_ET_FJsb_10D03_F_0	BY920848
E_ET_FJsb_10D03_R_0	BY929323

E_ET_FJsb_10D05_F_0	BY920849
E_ET_FJsb_10D05_R_0	BY929324
E_ET_FJsb_10D06_F_0	BY920850
E_ET_FJsb_10D06_R_0	BY929325
E_ET_FJsb_10D07_F_0	BY920851
E_ET_FJsb_10D07_R_0	BY929326
E_ET_FJsb_10D08_F_0	BY920852
E_ET_FJsb_10D08_R_0	BY929327
E_ET_FJsb_10D09_F_0	BY920853
E_ET_FJsb_10D09_R_0	BY929328
E_ET_FJsb_10D10_F_0	BY920854
E_ET_FJsb_10D10_R_0	BY929329
E_ET_FJsb_10D11_F_0	BY920855
E_ET_FJsb_10D11_R_0	BY929330
E_ET_FJsb_10D12_F_0	BY920856
E_ET_FJsb_10D12_R_0	BY929331
E_ET_FJsb_10E01_F_0	BY920857
E_ET_FJsb_10E01_R_0	BY929332
E_ET_FJsb_10E04_F_0	BY920858
E_ET_FJsb_10E04_R_0	BY929333
E_ET_FJsb_10E06_F_0	BY920859
E_ET_FJsb_10E06_R_0	BY929334
E_ET_FJsb_10E07_F_0	BY920860
E_ET_FJsb_10E07_R_0	BY929335
E_ET_FJsb_10E08_F_0	BY920861
E_ET_FJsb_10E08_R_0	BY929336
E_ET_FJsb_10E10_F_0	BY920862
E_ET_FJsb_10E10_R_0	BY929337
E_ET_FJsb_10E11_R_0	BY929338
E_ET_FJsb_10F01_F_0	BY920863
E_ET_FJsb_10F01_R_0	BY929339
E_ET_FJsb_10F02_R_0	BY929340
E_ET_FJsb_10F03_F_0	BY920864
E_ET_FJsb_10F03_R_0	BY929341
E_ET_FJsb_10F05_F_0	BY920865
E_ET_FJsb_10F05_R_0	BY929342
E_ET_FJsb_10F06_F_0	BY920866
E_ET_FJsb_10F06_R_0	BY929343
E_ET_FJsb_10F07_F_0	BY920867
E_ET_FJsb_10F07_R_0	BY929344
E_ET_FJsb_10F08_F_0	BY920868
E_ET_FJsb_10F08_R_0	BY929345
E_ET_FJsb_10F12_F_0	BY920869
E_ET_FJsb_10F12_R_0	BY929346
E_ET_FJsb_10G02_F_0	BY920870
E_ET_FJsb_10G02_R_0	BY929347
E_ET_FJsb_10G04_F_0	BY920871
E_ET_FJsb_10G04_R_0	BY929348
E_ET_FJsb_10G07_F_0	BY920872
E_ET_FJsb_10G07_R_0	BY929349
E_ET_FJsb_10G08_F_0	BY920873
E_ET_FJsb_10G08_R_0	BY929350
E_ET_FJsb_10H03_F_0	BY920874
E_ET_FJsb_10H03_R_0	BY929351

E_ET_FJsb_10H04_F_0	BY920875
E_ET_FJsb_10H04_R_0	BY929352
E_ET_FJsb_10H06_F_0	BY920876
E_ET_FJsb_10H06_R_0	BY929353
E_ET_FJsb_10H08_F_0	BY920877
E_ET_FJsb_10H09_F_0	BY920878
E_ET_FJsb_10H09_R_0	BY929354
E_ET_FJsb_10H10_F_0	BY920879
E_ET_FJsb_10H10_R_0	BY929355
E_ET_FJsb_10H11_F_0	BY920880
E_ET_FJsb_10H11_R_0	BY929356
E_ET_FJsb_10H12_F_0	BY920881
E_ET_FJsb_10H12_R_0	BY929357
E_ET_FJsb_11A01_R_0	BY929358
E_ET_FJsb_11A03_F_0	BY920882
E_ET_FJsb_11A03_R_0	BY929359
E_ET_FJsb_11A05_F_0	BY920883
E_ET_FJsb_11A05_R_0	BY929360
E_ET_FJsb_11A06_F_0	BY920884
E_ET_FJsb_11A06_R_0	BY929361
E_ET_FJsb_11A08_F_0	BY920885
E_ET_FJsb_11A08_R_0	BY929362
E_ET_FJsb_11A10_F_0	BY920886
E_ET_FJsb_11A10_R_0	BY929363
E_ET_FJsb_11A12_F_0	BY920887
E_ET_FJsb_11A12_R_0	BY929364
E_ET_FJsb_11B02_F_0	BY920888
E_ET_FJsb_11B03_F_0	BY920889
E_ET_FJsb_11B04_F_0	BY920890
E_ET_FJsb_11B05_F_0	BY920891
E_ET_FJsb_11B07_F_0	BY920892
E_ET_FJsb_11B08_F_0	BY920893
E_ET_FJsb_11B12_F_0	BY920894
E_ET_FJsb_11B12_R_0	BY929365
E_ET_FJsb_11C03_F_0	BY920895
E_ET_FJsb_11C03_R_0	BY929366
E_ET_FJsb_11C05_F_0	BY920896
E_ET_FJsb_11C05_R_0	BY929367
E_ET_FJsb_11C06_F_0	BY920897
E_ET_FJsb_11C06_R_0	BY929368
E_ET_FJsb_11C10_F_0	BY920898
E_ET_FJsb_11C10_R_0	BY929369
E_ET_FJsb_11C11_F_0	BY920899
E_ET_FJsb_11C11_R_0	BY929370
E_ET_FJsb_11C12_F_0	BY920900
E_ET_FJsb_11C12_R_0	BY929371
E_ET_FJsb_11D03_F_0	BY920901
E_ET_FJsb_11D03_R_0	BY929372
E_ET_FJsb_11D04_F_0	BY920902
E_ET_FJsb_11D04_R_0	BY929373
E_ET_FJsb_11D05_F_0	BY920903
E_ET_FJsb_11D05_R_0	BY929374
E_ET_FJsb_11D06_F_0	BY920904
E_ET_FJsb_11D06_R_0	BY929375

E_ET_FJsb_11D09_F_0	BY920905
E_ET_FJsb_11D09_R_0	BY929376
E_ET_FJsb_11E01_F_0	BY920906
E_ET_FJsb_11E01_R_0	BY929377
E_ET_FJsb_11E03_F_0	BY920907
E_ET_FJsb_11E03_R_0	BY929378
E_ET_FJsb_11E07_F_0	BY920908
E_ET_FJsb_11E07_R_0	BY929379
E_ET_FJsb_11E09_F_0	BY920909
E_ET_FJsb_11E09_R_0	BY929380
E_ET_FJsb_11E10_F_0	BY920910
E_ET_FJsb_11E10_R_0	BY929381
E_ET_FJsb_11E11_F_0	BY920911
E_ET_FJsb_11E11_R_0	BY929382
E_ET_FJsb_11E12_F_0	BY920912
E_ET_FJsb_11E12_R_0	BY929383
E_ET_FJsb_11F01_F_0	BY920913
E_ET_FJsb_11F01_R_0	BY929384
E_ET_FJsb_11F05_F_0	BY920914
E_ET_FJsb_11F05_R_0	BY929385
E_ET_FJsb_11F07_F_0	BY920915
E_ET_FJsb_11F07_R_0	BY929386
E_ET_FJsb_11F09_F_0	BY920916
E_ET_FJsb_11F09_R_0	BY929387
E_ET_FJsb_11F10_F_0	BY920917
E_ET_FJsb_11F10_R_0	BY929388
E_ET_FJsb_11F11_F_0	BY920918
E_ET_FJsb_11F11_R_0	BY929389
E_ET_FJsb_11G01_F_0	BY920919
E_ET_FJsb_11G01_R_0	BY929390
E_ET_FJsb_11G04_R_0	BY929391
E_ET_FJsb_11G05_F_0	BY920920
E_ET_FJsb_11G05_R_0	BY929392
E_ET_FJsb_11G06_F_0	BY920921
E_ET_FJsb_11G06_R_0	BY929393
E_ET_FJsb_11G08_F_0	BY920922
E_ET_FJsb_11G08_R_0	BY929394
E_ET_FJsb_11G09_F_0	BY920923
E_ET_FJsb_11G09_R_0	BY929395
E_ET_FJsb_11G10_F_0	BY920924
E_ET_FJsb_11G10_R_0	BY929396
E_ET_FJsb_11G12_F_0	BY920925
E_ET_FJsb_11H01_F_0	BY920926
E_ET_FJsb_11H01_R_0	BY929397
E_ET_FJsb_11H02_F_0	BY920927
E_ET_FJsb_11H02_R_0	BY929398
E_ET_FJsb_11H03_F_0	BY920928
E_ET_FJsb_11H03_R_0	BY929399
E_ET_FJsb_11H05_F_0	BY920929
E_ET_FJsb_11H05_R_0	BY929400
E_ET_FJsb_11H11_F_0	BY920930
E_ET_FJsb_11H11_R_0	BY929401
E_ET_FJsb_11H12_F_0	BY920931
E_ET_FJsb_11H12_R_0	BY929402

E_ET_FJsb_12A02_F_0	BY920932
E_ET_FJsb_12A02_R_0	BY929403
E_ET_FJsb_12A04_F_0	BY920933
E_ET_FJsb_12A04_R_0	BY929404
E_ET_FJsb_12A05_F_0	BY920934
E_ET_FJsb_12A06_F_0	BY920935
E_ET_FJsb_12A07_F_0	BY920936
E_ET_FJsb_12A08_F_0	BY920937
E_ET_FJsb_12A08_R_0	BY929405
E_ET_FJsb_12A09_F_0	BY920938
E_ET_FJsb_12A09_R_0	BY929406
E_ET_FJsb_12A10_F_0	BY920939
E_ET_FJsb_12A10_R_0	BY929407
E_ET_FJsb_12A11_F_0	BY920940
E_ET_FJsb_12A11_R_0	BY929408
E_ET_FJsb_12A12_F_0	BY920941
E_ET_FJsb_12A12_R_0	BY929409
E_ET_FJsb_12B02_F_0	BY920942
E_ET_FJsb_12B02_R_0	BY929410
E_ET_FJsb_12B05_R_0	BY929411
E_ET_FJsb_12B06_F_0	BY920943
E_ET_FJsb_12B06_R_0	BY929412
E_ET_FJsb_12B08_F_0	BY920944
E_ET_FJsb_12B08_R_0	BY929413
E_ET_FJsb_12B10_F_0	BY920945
E_ET_FJsb_12B10_R_0	BY929414
E_ET_FJsb_12C01_F_0	BY920946
E_ET_FJsb_12C01_R_0	BY929415
E_ET_FJsb_12C04_F_0	BY920947
E_ET_FJsb_12C04_R_0	BY929416
E_ET_FJsb_12C05_F_0	BY920948
E_ET_FJsb_12C05_R_0	BY929417
E_ET_FJsb_12C07_F_0	BY920949
E_ET_FJsb_12C07_R_0	BY929418
E_ET_FJsb_12C10_F_0	BY920950
E_ET_FJsb_12C10_R_0	BY929419
E_ET_FJsb_12C11_F_0	BY920951
E_ET_FJsb_12C11_R_0	BY929420
E_ET_FJsb_12C12_F_0	BY920952
E_ET_FJsb_12C12_R_0	BY929421
E_ET_FJsb_12D01_F_0	BY920953
E_ET_FJsb_12D01_R_0	BY929422
E_ET_FJsb_12D03_F_0	BY920954
E_ET_FJsb_12D03_R_0	BY929423
E_ET_FJsb_12D04_F_0	BY920955
E_ET_FJsb_12D04_R_0	BY929424
E_ET_FJsb_12D05_F_0	BY920956
E_ET_FJsb_12D05_R_0	BY929425
E_ET_FJsb_12D09_F_0	BY920957
E_ET_FJsb_12D09_R_0	BY929426
E_ET_FJsb_12E03_F_0	BY920958
E_ET_FJsb_12E04_F_0	BY920959
E_ET_FJsb_12E07_F_0	BY920960
E_ET_FJsb_12E09_F_0	BY920961

E_ET_FJsb_12F01_F_0	BY920962
E_ET_FJsb_12F04_F_0	BY920963
E_ET_FJsb_12F04_R_0	BY929427
E_ET_FJsb_12F06_F_0	BY920964
E_ET_FJsb_12F06_R_0	BY929428
E_ET_FJsb_12F08_F_0	BY920965
E_ET_FJsb_12F08_R_0	BY929429
E_ET_FJsb_12F10_F_0	BY920966
E_ET_FJsb_12F12_F_0	BY920967
E_ET_FJsb_12G01_F_0	BY920968
E_ET_FJsb_12G04_F_0	BY920969
E_ET_FJsb_12G09_F_0	BY920970
E_ET_FJsb_12G09_R_0	BY929430
E_ET_FJsb_12G10_F_0	BY920971
E_ET_FJsb_12G12_F_0	BY920972
E_ET_FJsb_12H01_F_0	BY920973
E_ET_FJsb_12H01_R_0	BY929431
E_ET_FJsb_12H02_F_0	BY920974
E_ET_FJsb_12H02_R_0	BY929432
E_ET_FJsb_12H03_F_0	BY920975
E_ET_FJsb_12H03_R_0	BY929433
E_ET_FJsb_12H04_F_0	BY920976
E_ET_FJsb_12H04_R_0	BY929434
E_ET_FJsb_12H09_F_0	BY920977
E_ET_FJsb_12H09_R_0	BY929435
E_ET_FJsb_13A05_R_0	BY929436
E_ET_FJsb_13A07_F_0	BY920978
E_ET_FJsb_13A07_R_0	BY929437
E_ET_FJsb_13A10_F_0	BY920979
E_ET_FJsb_13A10_R_0	BY929438
E_ET_FJsb_13A12_F_0	BY920980
E_ET_FJsb_13A12_R_0	BY929439
E_ET_FJsb_13B03_R_0	BY929440
E_ET_FJsb_13B04_F_0	BY920981
E_ET_FJsb_13B04_R_0	BY929441
E_ET_FJsb_13B06_R_0	BY929442
E_ET_FJsb_13B07_F_0	BY920982
E_ET_FJsb_13B07_R_0	BY929443
E_ET_FJsb_13B12_F_0	BY920983
E_ET_FJsb_13B12_R_0	BY929444
E_ET_FJsb_13C04_F_0	BY920984
E_ET_FJsb_13C04_R_0	BY929445
E_ET_FJsb_13C05_F_0	BY920985
E_ET_FJsb_13C05_R_0	BY929446
E_ET_FJsb_13C08_R_0	BY929447
E_ET_FJsb_13C09_F_0	BY920986
E_ET_FJsb_13C09_R_0	BY929448
E_ET_FJsb_13C11_F_0	BY920987
E_ET_FJsb_13C11_R_0	BY929449
E_ET_FJsb_13D04_R_0	BY929450
E_ET_FJsb_13D06_R_0	BY929451
E_ET_FJsb_13D11_F_0	BY920988
E_ET_FJsb_13D11_R_0	BY929452
E_ET_FJsb_13E03_R_0	BY929453

	E_ET_FJsb_13E04_R_0	BY929454
	E_ET_FJsb_13E06_F_0	BY920989
	E_ET_FJsb_13E06_R_0	BY929455
	E_ET_FJsb_13E08_F_0	BY920990
	E_ET_FJsb_13E08_R_0	BY929456
	E_ET_FJsb_13E09_F_0	BY920991
	E_ET_FJsb_13E09_R_0	BY929457
	E_ET_FJsb_13E10_R_0	BY929458
	E_ET_FJsb_13E11_F_0	BY920992
	E_ET_FJsb_13E11_R_0	BY929459
	E_ET_FJsb_13E12_R_0	BY929460
	E_ET_FJsb_13F04_F_0	BY920993
	E_ET_FJsb_13F04_R_0	BY929461
	E_ET_FJsb_13F06_R_0	BY929462
	E_ET_FJsb_13F09_F_0	BY920994
	E_ET_FJsb_13F09_R_0	BY929463
	E_ET_FJsb_13G04_R_0	BY929464
	E_ET_FJsb_13G08_F_0	BY920995
	E_ET_FJsb_13G08_R_0	BY929465
	E_ET_FJsb_13G10_F_0	BY920996
	E_ET_FJsb_13G10_R_0	BY929466
	E_ET_FJsb_13G11_F_0	BY920997
	E_ET_FJsb_13G11_R_0	BY929467
	E_ET_FJsb_13G12_F_0	BY920998
	E_ET_FJsb_13G12_R_0	BY929468
	E_ET_FJsb_13H04_F_0	BY920999
	E_ET_FJsb_13H04_R_0	BY929469
	E_ET_FJsb_13H05_F_0	BY921000
	E_ET_FJsb_13H05_R_0	BY929470
	E_ET_FJsb_13H09_F_0	BY921001
	E_ET_FJsb_13H09_R_0	BY929471
	E_ET_FJsb_13H11_F_0	BY921002
	E_ET_FJsb_13H11_R_0	BY929472
JFsb	E_ET_JFsb_01A03_F_0	BY921003
	E_ET_JFsb_01A03_R_0	BY929473
	E_ET_JFsb_01A04_F_0	BY921004
	E_ET_JFsb_01A04_R_0	BY929474
	E_ET_JFsb_01A05_F_0	BY921005
	E_ET_JFsb_01A05_R_0	BY929475
	E_ET_JFsb_01A09_F_0	BY921006
	E_ET_JFsb_01A09_R_0	BY929476
	E_ET_JFsb_01A11_F_0	BY921007
	E_ET_JFsb_01A11_R_0	BY929477
	E_ET_JFsb_01A12_F_0	BY921008
	E_ET_JFsb_01B04_F_0	BY921009
	E_ET_JFsb_01B04_R_0	BY929478
	E_ET_JFsb_01B05_F_0	BY921010
	E_ET_JFsb_01B05_R_0	BY929479
	E_ET_JFsb_01B06_F_0	BY921011
	E_ET_JFsb_01B06_R_0	BY929480
	E_ET_JFsb_01B07_R_0	BY929481
	E_ET_JFsb_01B10_F_0	BY921012
	E_ET_JFsb_01B10_R_0	BY929482
	E_ET_JFsb_01B12_F_0	BY921013

E_ET_JFsb_01C01_R_0	BY929483
E_ET_JFsb_01C02_R_0	BY929484
E_ET_JFsb_01C03_F_0	BY921014
E_ET_JFsb_01C03_R_0	BY929485
E_ET_JFsb_01C04_F_0	BY921015
E_ET_JFsb_01C04_R_0	BY929486
E_ET_JFsb_01C05_F_0	BY921016
E_ET_JFsb_01C05_R_0	BY929487
E_ET_JFsb_01C09_R_0	BY929488
E_ET_JFsb_01C10_R_0	BY929489
E_ET_JFsb_01C11_F_0	BY921017
E_ET_JFsb_01C12_R_0	BY929490
E_ET_JFsb_01D01_R_0	BY929491
E_ET_JFsb_01D02_R_0	BY929492
E_ET_JFsb_01D03_R_0	BY929493
E_ET_JFsb_01D04_R_0	BY929494
E_ET_JFsb_01D06_R_0	BY929495
E_ET_JFsb_01D08_F_0	BY921018
E_ET_JFsb_01D08_R_0	BY929496
E_ET_JFsb_01D09_R_0	BY929497
E_ET_JFsb_01D10_F_0	BY921019
E_ET_JFsb_01D10_R_0	BY929498
E_ET_JFsb_01E02_F_0	BY921020
E_ET_JFsb_01E02_R_0	BY929499
E_ET_JFsb_01E03_F_0	BY921021
E_ET_JFsb_01E03_R_0	BY929500
E_ET_JFsb_01E04_R_0	BY929501
E_ET_JFsb_01E06_F_0	BY921022
E_ET_JFsb_01E10_F_0	BY921023
E_ET_JFsb_01E10_R_0	BY929502
E_ET_JFsb_01E11_F_0	BY921024
E_ET_JFsb_01E11_R_0	BY929503
E_ET_JFsb_01E12_F_0	BY921025
E_ET_JFsb_01E12_R_0	BY929504
E_ET_JFsb_01F02_F_0	BY921026
E_ET_JFsb_01F02_R_0	BY929505
E_ET_JFsb_01F05_F_0	BY921027
E_ET_JFsb_01F05_R_0	BY929506
E_ET_JFsb_01F07_F_0	BY921028
E_ET_JFsb_01F07_R_0	BY929507
E_ET_JFsb_01F09_F_0	BY921029
E_ET_JFsb_01F09_R_0	BY929508
E_ET_JFsb_01F11_F_0	BY921030
E_ET_JFsb_01F11_R_0	BY929509
E_ET_JFsb_01G01_F_0	BY921031
E_ET_JFsb_01G01_R_0	BY929510
E_ET_JFsb_01G02_F_0	BY921032
E_ET_JFsb_01G02_R_0	BY929511
E_ET_JFsb_01G04_F_0	BY921033
E_ET_JFsb_01G04_R_0	BY929512
E_ET_JFsb_01G05_F_0	BY921034
E_ET_JFsb_01G05_R_0	BY929513
E_ET_JFsb_01G06_F_0	BY921035
E_ET_JFsb_01G06_R_0	BY929514

E_ET_JFsb_01G10_F_0	BY921036
E_ET_JFsb_01G10_R_0	BY929515
E_ET_JFsb_01G12_F_0	BY921037
E_ET_JFsb_01G12_R_0	BY929516
E_ET_JFsb_01H01_F_0	BY921038
E_ET_JFsb_01H03_F_0	BY921039
E_ET_JFsb_01H03_R_0	BY929517
E_ET_JFsb_01H06_F_0	BY921040
E_ET_JFsb_01H06_R_0	BY929518
E_ET_JFsb_01H07_F_0	BY921041
E_ET_JFsb_01H07_R_0	BY929519
E_ET_JFsb_01H09_F_0	BY921042
E_ET_JFsb_01H09_R_0	BY929520
E_ET_JFsb_01H10_F_0	BY921043
E_ET_JFsb_01H10_R_0	BY929521
E_ET_JFsb_01H11_F_0	BY921044
E_ET_JFsb_01H12_F_0	BY921045
E_ET_JFsb_02A01_F_0	BY921046
E_ET_JFsb_02A01_R_0	BY929522
E_ET_JFsb_02A04_F_0	BY921047
E_ET_JFsb_02A04_R_0	BY929523
E_ET_JFsb_02A05_F_0	BY921048
E_ET_JFsb_02A05_R_0	BY929524
E_ET_JFsb_02A08_F_0	BY921049
E_ET_JFsb_02A08_R_0	BY929525
E_ET_JFsb_02A10_R_0	BY929526
E_ET_JFsb_02B01_F_0	BY921050
E_ET_JFsb_02B01_R_0	BY929527
E_ET_JFsb_02B03_F_0	BY921051
E_ET_JFsb_02B03_R_0	BY929528
E_ET_JFsb_02B04_F_0	BY921052
E_ET_JFsb_02B04_R_0	BY929529
E_ET_JFsb_02B06_F_0	BY921053
E_ET_JFsb_02B06_R_0	BY929530
E_ET_JFsb_02B07_F_0	BY921054
E_ET_JFsb_02B07_R_0	BY929531
E_ET_JFsb_02B09_F_0	BY921055
E_ET_JFsb_02B09_R_0	BY929532
E_ET_JFsb_02B10_F_0	BY921056
E_ET_JFsb_02B10_R_0	BY929533
E_ET_JFsb_02B11_F_0	BY921057
E_ET_JFsb_02B11_R_0	BY929534
E_ET_JFsb_02B12_F_0	BY921058
E_ET_JFsb_02B12_R_0	BY929535
E_ET_JFsb_02C02_F_0	BY921059
E_ET_JFsb_02C02_R_0	BY929536
E_ET_JFsb_02C05_F_0	BY921060
E_ET_JFsb_02C05_R_0	BY929537
E_ET_JFsb_02C08_F_0	BY921061
E_ET_JFsb_02C08_R_0	BY929538
E_ET_JFsb_02C11_F_0	BY921062
E_ET_JFsb_02C11_R_0	BY929539
E_ET_JFsb_02C12_F_0	BY921063
E_ET_JFsb_02D01_F_0	BY921064

E_ET_JFsb_02D01_R_0	BY929540
E_ET_JFsb_02D02_F_0	BY921065
E_ET_JFsb_02D02_R_0	BY929541
E_ET_JFsb_02D03_F_0	BY921066
E_ET_JFsb_02D03_R_0	BY929542
E_ET_JFsb_02D05_F_0	BY921067
E_ET_JFsb_02D05_R_0	BY929543
E_ET_JFsb_02D09_F_0	BY921068
E_ET_JFsb_02D09_R_0	BY929544
E_ET_JFsb_02D12_F_0	BY921069
E_ET_JFsb_02D12_R_0	BY929545
E_ET_JFsb_02E01_F_0	BY921070
E_ET_JFsb_02E01_R_0	BY929546
E_ET_JFsb_02E04_R_0	BY929547
E_ET_JFsb_02E05_F_0	BY921071
E_ET_JFsb_02E05_R_0	BY929548
E_ET_JFsb_02E07_F_0	BY921072
E_ET_JFsb_02E07_R_0	BY929549
E_ET_JFsb_02E09_F_0	BY921073
E_ET_JFsb_02E09_R_0	BY929550
E_ET_JFsb_02E10_F_0	BY921074
E_ET_JFsb_02E10_R_0	BY929551
E_ET_JFsb_02E12_F_0	BY921075
E_ET_JFsb_02E12_R_0	BY929552
E_ET_JFsb_02F02_F_0	BY921076
E_ET_JFsb_02F02_R_0	BY929553
E_ET_JFsb_02F04_F_0	BY921077
E_ET_JFsb_02F04_R_0	BY929554
E_ET_JFsb_02F05_F_0	BY921078
E_ET_JFsb_02F05_R_0	BY929555
E_ET_JFsb_02F06_R_0	BY929556
E_ET_JFsb_02F09_F_0	BY921079
E_ET_JFsb_02F09_R_0	BY929557
E_ET_JFsb_02G01_R_0	BY929558
E_ET_JFsb_02G03_F_0	BY921080
E_ET_JFsb_02G03_R_0	BY929559
E_ET_JFsb_02G04_F_0	BY921081
E_ET_JFsb_02G04_R_0	BY929560
E_ET_JFsb_02G06_F_0	BY921082
E_ET_JFsb_02G06_R_0	BY929561
E_ET_JFsb_02G07_F_0	BY921083
E_ET_JFsb_02G07_R_0	BY929562
E_ET_JFsb_02G12_R_0	BY929563
E_ET_JFsb_02H02_R_0	BY929564
E_ET_JFsb_02H04_F_0	BY921084
E_ET_JFsb_02H08_F_0	BY921085
E_ET_JFsb_02H08_R_0	BY929565
E_ET_JFsb_02H09_F_0	BY921086
E_ET_JFsb_02H09_R_0	BY929566
E_ET_JFsb_02H10_F_0	BY921087
E_ET_JFsb_02H10_R_0	BY929567
E_ET_JFsb_02H12_F_0	BY921088
E_ET_JFsb_02H12_R_0	BY929568
E_ET_JFsb_03A06_F_0	BY921089

E_ET_JFsb_03A06_R_0	BY929569
E_ET_JFsb_03A07_F_0	BY921090
E_ET_JFsb_03A07_R_0	BY929570
E_ET_JFsb_03A09_F_0	BY921091
E_ET_JFsb_03A09_R_0	BY929571
E_ET_JFsb_03A10_F_0	BY921092
E_ET_JFsb_03B06_F_0	BY921093
E_ET_JFsb_03B06_R_0	BY929572
E_ET_JFsb_03B12_F_0	BY921094
E_ET_JFsb_03B12_R_0	BY929573
E_ET_JFsb_03C02_F_0	BY921095
E_ET_JFsb_03C02_R_0	BY929574
E_ET_JFsb_03C03_F_0	BY921096
E_ET_JFsb_03C03_R_0	BY929575
E_ET_JFsb_03C08_F_0	BY921097
E_ET_JFsb_03C08_R_0	BY929576
E_ET_JFsb_03C10_F_0	BY921098
E_ET_JFsb_03C10_R_0	BY929577
E_ET_JFsb_03D01_F_0	BY921099
E_ET_JFsb_03D01_R_0	BY929578
E_ET_JFsb_03D02_F_0	BY921100
E_ET_JFsb_03D02_R_0	BY929579
E_ET_JFsb_03D04_R_0	BY929580
E_ET_JFsb_03D05_F_0	BY921101
E_ET_JFsb_03D05_R_0	BY929581
E_ET_JFsb_03D06_F_0	BY921102
E_ET_JFsb_03D06_R_0	BY929582
E_ET_JFsb_03D07_F_0	BY921103
E_ET_JFsb_03D07_R_0	BY929583
E_ET_JFsb_03D08_F_0	BY921104
E_ET_JFsb_03D08_R_0	BY929584
E_ET_JFsb_03D10_F_0	BY921105
E_ET_JFsb_03D10_R_0	BY929585
E_ET_JFsb_03D12_F_0	BY921106
E_ET_JFsb_03D12_R_0	BY929586
E_ET_JFsb_03E01_F_0	BY921107
E_ET_JFsb_03E01_R_0	BY929587
E_ET_JFsb_03E02_F_0	BY921108
E_ET_JFsb_03E02_R_0	BY929588
E_ET_JFsb_03E03_R_0	BY929589
E_ET_JFsb_03E04_F_0	BY921109
E_ET_JFsb_03E04_R_0	BY929590
E_ET_JFsb_03E08_F_0	BY921110
E_ET_JFsb_03E08_R_0	BY929591
E_ET_JFsb_03E11_F_0	BY921111
E_ET_JFsb_03E11_R_0	BY929592
E_ET_JFsb_03F01_F_0	BY921112
E_ET_JFsb_03F01_R_0	BY929593
E_ET_JFsb_03F03_F_0	BY921113
E_ET_JFsb_03F03_R_0	BY929594
E_ET_JFsb_03F05_F_0	BY921114
E_ET_JFsb_03F05_R_0	BY929595
E_ET_JFsb_03F06_F_0	BY921115
E_ET_JFsb_03F06_R_0	BY929596

E_ET_JFsb_03F07_F_0	BY921116
E_ET_JFsb_03F07_R_0	BY929597
E_ET_JFsb_03F09_F_0	BY921117
E_ET_JFsb_03F09_R_0	BY929598
E_ET_JFsb_03G04_F_0	BY921118
E_ET_JFsb_03G05_F_0	BY921119
E_ET_JFsb_03G05_R_0	BY929599
E_ET_JFsb_03G07_F_0	BY921120
E_ET_JFsb_03G07_R_0	BY929600
E_ET_JFsb_03G09_F_0	BY921121
E_ET_JFsb_03G09_R_0	BY929601
E_ET_JFsb_03G10_F_0	BY921122
E_ET_JFsb_03G10_R_0	BY929602
E_ET_JFsb_03G11_F_0	BY921123
E_ET_JFsb_03G11_R_0	BY929603
E_ET_JFsb_03H03_F_0	BY921124
E_ET_JFsb_03H03_R_0	BY929604
E_ET_JFsb_03H04_F_0	BY921125
E_ET_JFsb_03H04_R_0	BY929605
E_ET_JFsb_03H07_F_0	BY921126
E_ET_JFsb_03H07_R_0	BY929606
E_ET_JFsb_03H08_F_0	BY921127
E_ET_JFsb_03H08_R_0	BY929607
E_ET_JFsb_04A02_R_0	BY929608
E_ET_JFsb_04A07_R_0	BY929609
E_ET_JFsb_04A09_F_0	BY921128
E_ET_JFsb_04A09_R_0	BY929610
E_ET_JFsb_04A11_R_0	BY929611
E_ET_JFsb_04A12_F_0	BY921129
E_ET_JFsb_04A12_R_0	BY929612
E_ET_JFsb_04B03_R_0	BY929613
E_ET_JFsb_04B04_R_0	BY929614
E_ET_JFsb_04B06_R_0	BY929615
E_ET_JFsb_04B08_R_0	BY929616
E_ET_JFsb_04B09_F_0	BY921130
E_ET_JFsb_04B09_R_0	BY929617
E_ET_JFsb_04B10_F_0	BY921131
E_ET_JFsb_04B10_R_0	BY929618
E_ET_JFsb_04C01_F_0	BY921132
E_ET_JFsb_04C01_R_0	BY929619
E_ET_JFsb_04C02_R_0	BY929620
E_ET_JFsb_04C05_F_0	BY921133
E_ET_JFsb_04C05_R_0	BY929621
E_ET_JFsb_04C12_R_0	BY929622
E_ET_JFsb_04D01_R_0	BY929623
E_ET_JFsb_04D03_R_0	BY929624
E_ET_JFsb_04D06_F_0	BY921134
E_ET_JFsb_04D06_R_0	BY929625
E_ET_JFsb_04D07_F_0	BY921135
E_ET_JFsb_04D07_R_0	BY929626
E_ET_JFsb_04D08_R_0	BY929627
E_ET_JFsb_04D10_F_0	BY921136
E_ET_JFsb_04D10_R_0	BY929628
E_ET_JFsb_04D11_F_0	BY921137

E_ET_JFsb_04D11_R_0	BY929629
E_ET_JFsb_04E01_R_0	BY929630
E_ET_JFsb_04E02_R_0	BY929631
E_ET_JFsb_04E05_R_0	BY929632
E_ET_JFsb_04E06_R_0	BY929633
E_ET_JFsb_04E07_R_0	BY929634
E_ET_JFsb_04E11_F_0	BY921138
E_ET_JFsb_04E11_R_0	BY929635
E_ET_JFsb_04E12_F_0	BY921139
E_ET_JFsb_04E12_R_0	BY929636
E_ET_JFsb_04F02_R_0	BY929637
E_ET_JFsb_04F04_F_0	BY921140
E_ET_JFsb_04F04_R_0	BY929638
E_ET_JFsb_04F05_F_0	BY921141
E_ET_JFsb_04F05_R_0	BY929639
E_ET_JFsb_04F06_R_0	BY929640
E_ET_JFsb_04F07_R_0	BY929641
E_ET_JFsb_04F09_F_0	BY921142
E_ET_JFsb_04F09_R_0	BY929642
E_ET_JFsb_04F11_F_0	BY921143
E_ET_JFsb_04F11_R_0	BY929643
E_ET_JFsb_04G04_R_0	BY929644
E_ET_JFsb_04G06_R_0	BY929645
E_ET_JFsb_04G07_R_0	BY929646
E_ET_JFsb_04G09_F_0	BY921144
E_ET_JFsb_04G09_R_0	BY929647
E_ET_JFsb_04G10_F_0	BY921145
E_ET_JFsb_04G10_R_0	BY929648
E_ET_JFsb_04G11_F_0	BY921146
E_ET_JFsb_04G11_R_0	BY929649
E_ET_JFsb_04G12_R_0	BY929650
E_ET_JFsb_04H01_R_0	BY929651
E_ET_JFsb_04H02_R_0	BY929652
E_ET_JFsb_04H09_F_0	BY921147
E_ET_JFsb_04H09_R_0	BY929653
E_ET_JFsb_04H10_F_0	BY921148
E_ET_JFsb_04H10_R_0	BY929654
E_ET_JFsb_04H12_R_0	BY929655
E_ET_JFsb_05A02_F_0	BY921149
E_ET_JFsb_05A02_R_0	BY929656
E_ET_JFsb_05A03_F_0	BY921150
E_ET_JFsb_05A03_R_0	BY929657
E_ET_JFsb_05A06_F_0	BY921151
E_ET_JFsb_05A06_R_0	BY929658
E_ET_JFsb_05A07_F_0	BY921152
E_ET_JFsb_05A07_R_0	BY929659
E_ET_JFsb_05A10_F_0	BY921153
E_ET_JFsb_05A10_R_0	BY929660
E_ET_JFsb_05B02_F_0	BY921154
E_ET_JFsb_05B02_R_0	BY929661
E_ET_JFsb_05B03_F_0	BY921155
E_ET_JFsb_05B03_R_0	BY929662
E_ET_JFsb_05B04_F_0	BY921156
E_ET_JFsb_05B04_R_0	BY929663

E_ET_JFsb_05B05_F_0	BY921157
E_ET_JFsb_05B05_R_0	BY929664
E_ET_JFsb_05B06_F_0	BY921158
E_ET_JFsb_05B06_R_0	BY929665
E_ET_JFsb_05B08_F_0	BY921159
E_ET_JFsb_05B08_R_0	BY929666
E_ET_JFsb_05B10_F_0	BY921160
E_ET_JFsb_05B10_R_0	BY929667
E_ET_JFsb_05B12_F_0	BY921161
E_ET_JFsb_05B12_R_0	BY929668
E_ET_JFsb_05C02_F_0	BY921162
E_ET_JFsb_05C05_F_0	BY921163
E_ET_JFsb_05C05_R_0	BY929669
E_ET_JFsb_05C07_F_0	BY921164
E_ET_JFsb_05C07_R_0	BY929670
E_ET_JFsb_05C09_F_0	BY921165
E_ET_JFsb_05C09_R_0	BY929671
E_ET_JFsb_05C10_F_0	BY921166
E_ET_JFsb_05C10_R_0	BY929672
E_ET_JFsb_05C12_F_0	BY921167
E_ET_JFsb_05C12_R_0	BY929673
E_ET_JFsb_05D02_F_0	BY921168
E_ET_JFsb_05D02_R_0	BY929674
E_ET_JFsb_05D03_F_0	BY921169
E_ET_JFsb_05D03_R_0	BY929675
E_ET_JFsb_05D06_F_0	BY921170
E_ET_JFsb_05D06_R_0	BY929676
E_ET_JFsb_05D09_F_0	BY921171
E_ET_JFsb_05D09_R_0	BY929677
E_ET_JFsb_05D11_F_0	BY921172
E_ET_JFsb_05D11_R_0	BY929678
E_ET_JFsb_05D12_F_0	BY921173
E_ET_JFsb_05D12_R_0	BY929679
E_ET_JFsb_05E02_R_0	BY929680
E_ET_JFsb_05E04_F_0	BY921174
E_ET_JFsb_05E04_R_0	BY929681
E_ET_JFsb_05E05_F_0	BY921175
E_ET_JFsb_05E05_R_0	BY929682
E_ET_JFsb_05E08_F_0	BY921176
E_ET_JFsb_05E08_R_0	BY929683
E_ET_JFsb_05E10_F_0	BY921177
E_ET_JFsb_05E10_R_0	BY929684
E_ET_JFsb_05E11_R_0	BY929685
E_ET_JFsb_05F01_F_0	BY921178
E_ET_JFsb_05F01_R_0	BY929686
E_ET_JFsb_05F02_F_0	BY921179
E_ET_JFsb_05F02_R_0	BY929687
E_ET_JFsb_05F03_F_0	BY921180
E_ET_JFsb_05F03_R_0	BY929688
E_ET_JFsb_05F05_F_0	BY921181
E_ET_JFsb_05F05_R_0	BY929689
E_ET_JFsb_05F07_F_0	BY921182
E_ET_JFsb_05F07_R_0	BY929690
E_ET_JFsb_05F08_R_0	BY929691

E_ET_JFsb_05F10_F_0	BY921183
E_ET_JFsb_05F10_R_0	BY929692
E_ET_JFsb_05F11_F_0	BY921184
E_ET_JFsb_05F11_R_0	BY929693
E_ET_JFsb_05G01_F_0	BY921185
E_ET_JFsb_05G01_R_0	BY929694
E_ET_JFsb_05G02_F_0	BY921186
E_ET_JFsb_05G02_R_0	BY929695
E_ET_JFsb_05G04_F_0	BY921187
E_ET_JFsb_05G04_R_0	BY929696
E_ET_JFsb_05G07_F_0	BY921188
E_ET_JFsb_05G07_R_0	BY929697
E_ET_JFsb_05G08_R_0	BY929698
E_ET_JFsb_05G09_F_0	BY921189
E_ET_JFsb_05G09_R_0	BY929699
E_ET_JFsb_05G10_F_0	BY921190
E_ET_JFsb_05G10_R_0	BY929700
E_ET_JFsb_05G11_F_0	BY921191
E_ET_JFsb_05G11_R_0	BY929701
E_ET_JFsb_05H02_F_0	BY921192
E_ET_JFsb_05H02_R_0	BY929702
E_ET_JFsb_05H06_F_0	BY921193
E_ET_JFsb_05H06_R_0	BY929703
E_ET_JFsb_05H07_F_0	BY921194
E_ET_JFsb_05H07_R_0	BY929704
E_ET_JFsb_05H10_F_0	BY921195
E_ET_JFsb_05H10_R_0	BY929705
E_ET_JFsb_05H11_F_0	BY921196
E_ET_JFsb_05H11_R_0	BY929706
E_ET_JFsb_05H12_F_0	BY921197
E_ET_JFsb_05H12_R_0	BY929707
E_ET_JFsb_06A04_F_0	BY921198
E_ET_JFsb_06A04_R_0	BY929708
E_ET_JFsb_06A05_R_0	BY929709
E_ET_JFsb_06A06_R_0	BY929710
E_ET_JFsb_06A08_R_0	BY929711
E_ET_JFsb_06A12_F_0	BY921199
E_ET_JFsb_06A12_R_0	BY929712
E_ET_JFsb_06B01_R_0	BY929713
E_ET_JFsb_06B05_R_0	BY929714
E_ET_JFsb_06B11_F_0	BY921200
E_ET_JFsb_06B11_R_0	BY929715
E_ET_JFsb_06C03_R_0	BY929716
E_ET_JFsb_06C04_R_0	BY929717
E_ET_JFsb_06C06_F_0	BY921201
E_ET_JFsb_06C06_R_0	BY929718
E_ET_JFsb_06C07_F_0	BY921202
E_ET_JFsb_06C12_F_0	BY921203
E_ET_JFsb_06C12_R_0	BY929719
E_ET_JFsb_06D01_R_0	BY929720
E_ET_JFsb_06D04_F_0	BY921204
E_ET_JFsb_06D04_R_0	BY929721
E_ET_JFsb_06D05_R_0	BY929722
E_ET_JFsb_06D06_F_0	BY921205

E_ET_JFsb_06D06_R_0	BY929723
E_ET_JFsb_06D07_F_0	BY921206
E_ET_JFsb_06D07_R_0	BY929724
E_ET_JFsb_06D09_R_0	BY929725
E_ET_JFsb_06D10_R_0	BY929726
E_ET_JFsb_06D12_R_0	BY929727
E_ET_JFsb_06E01_R_0	BY929728
E_ET_JFsb_06E02_R_0	BY929729
E_ET_JFsb_06E04_F_0	BY921207
E_ET_JFsb_06E04_R_0	BY929730
E_ET_JFsb_06E05_F_0	BY921208
E_ET_JFsb_06E05_R_0	BY929731
E_ET_JFsb_06E06_R_0	BY929732
E_ET_JFsb_06E09_R_0	BY929733
E_ET_JFsb_06E10_F_0	BY921209
E_ET_JFsb_06E10_R_0	BY929734
E_ET_JFsb_06E12_R_0	BY929735
E_ET_JFsb_06F03_R_0	BY929736
E_ET_JFsb_06F04_F_0	BY921210
E_ET_JFsb_06F04_R_0	BY929737
E_ET_JFsb_06F05_F_0	BY921211
E_ET_JFsb_06F05_R_0	BY929738
E_ET_JFsb_06F07_R_0	BY929739
E_ET_JFsb_06F08_F_0	BY921212
E_ET_JFsb_06F08_R_0	BY929740
E_ET_JFsb_06F11_F_0	BY921213
E_ET_JFsb_06F11_R_0	BY929741
E_ET_JFsb_06F12_F_0	BY921214
E_ET_JFsb_06F12_R_0	BY929742
E_ET_JFsb_06G01_R_0	BY929743
E_ET_JFsb_06G04_R_0	BY929744
E_ET_JFsb_06G06_F_0	BY921215
E_ET_JFsb_06G07_F_0	BY921216
E_ET_JFsb_06G07_R_0	BY929745
E_ET_JFsb_06G09_F_0	BY921217
E_ET_JFsb_06G09_R_0	BY929746
E_ET_JFsb_06G10_R_0	BY929747
E_ET_JFsb_06G11_R_0	BY929748
E_ET_JFsb_06G12_F_0	BY921218
E_ET_JFsb_06G12_R_0	BY929749
E_ET_JFsb_06H01_F_0	BY921219
E_ET_JFsb_06H01_R_0	BY929750
E_ET_JFsb_06H02_R_0	BY929751
E_ET_JFsb_06H04_F_0	BY921220
E_ET_JFsb_06H04_R_0	BY929752
E_ET_JFsb_06H05_F_0	BY921221
E_ET_JFsb_06H05_R_0	BY929753
E_ET_JFsb_06H06_F_0	BY921222
E_ET_JFsb_06H06_R_0	BY929754
E_ET_JFsb_06H10_F_0	BY921223
E_ET_JFsb_06H10_R_0	BY929755
E_ET_JFsb_06H11_F_0	BY921224
E_ET_JFsb_06H11_R_0	BY929756
E_ET_JFsb_07A03_R_0	BY929757

E_ET_JFsb_07A04_F_0	BY921225
E_ET_JFsb_07A04_R_0	BY929758
E_ET_JFsb_07A05_F_0	BY921226
E_ET_JFsb_07A05_R_0	BY929759
E_ET_JFsb_07A09_F_0	BY921227
E_ET_JFsb_07A09_R_0	BY929760
E_ET_JFsb_07A12_F_0	BY921228
E_ET_JFsb_07A12_R_0	BY929761
E_ET_JFsb_07B02_F_0	BY921229
E_ET_JFsb_07B02_R_0	BY929762
E_ET_JFsb_07C04_F_0	BY921230
E_ET_JFsb_07C04_R_0	BY929763
E_ET_JFsb_07C05_F_0	BY921231
E_ET_JFsb_07C05_R_0	BY929764
E_ET_JFsb_07C08_F_0	BY921232
E_ET_JFsb_07C08_R_0	BY929765
E_ET_JFsb_07C10_F_0	BY921233
E_ET_JFsb_07C10_R_0	BY929766
E_ET_JFsb_07D03_F_0	BY921234
E_ET_JFsb_07D03_R_0	BY929767
E_ET_JFsb_07D04_F_0	BY921235
E_ET_JFsb_07D04_R_0	BY929768
E_ET_JFsb_07D08_F_0	BY921236
E_ET_JFsb_07D08_R_0	BY929769
E_ET_JFsb_07D10_F_0	BY921237
E_ET_JFsb_07D10_R_0	BY929770
E_ET_JFsb_07E01_F_0	BY921238
E_ET_JFsb_07E04_F_0	BY921239
E_ET_JFsb_07E04_R_0	BY929771
E_ET_JFsb_07E05_R_0	BY929772
E_ET_JFsb_07E06_F_0	BY921240
E_ET_JFsb_07E06_R_0	BY929773
E_ET_JFsb_07E08_R_0	BY929774
E_ET_JFsb_07F05_F_0	BY921241
E_ET_JFsb_07F05_R_0	BY929775
E_ET_JFsb_07F07_F_0	BY921242
E_ET_JFsb_07F07_R_0	BY929776
E_ET_JFsb_07F08_F_0	BY921243
E_ET_JFsb_07F08_R_0	BY929777
E_ET_JFsb_07F11_F_0	BY921244
E_ET_JFsb_07F12_F_0	BY921245
E_ET_JFsb_07F12_R_0	BY929778
E_ET_JFsb_07G01_F_0	BY921246
E_ET_JFsb_07G01_R_0	BY929779
E_ET_JFsb_07G03_F_0	BY921247
E_ET_JFsb_07G03_R_0	BY929780
E_ET_JFsb_07G06_F_0	BY921248
E_ET_JFsb_07G09_F_0	BY921249
E_ET_JFsb_07G09_R_0	BY929781
E_ET_JFsb_07G12_F_0	BY921250
E_ET_JFsb_07G12_R_0	BY929782
E_ET_JFsb_07H02_F_0	BY921251
E_ET_JFsb_07H02_R_0	BY929783
E_ET_JFsb_07H03_F_0	BY921252

	E_ET_JFsb_07H03_R_0	BY929784
	E_ET_JFsb_07H06_F_0	BY921253
	E_ET_JFsb_07H06_R_0	BY929785
	E_ET_JFsb_07H11_R_0	BY929786
	E_ET_JFsb_07H12_F_0	BY921254
	E_ET_JFsb_07H12_R_0	BY929787
maV3	E_ET_maV3_01A01_F_1	BY915512
	E_ET_maV3_01A02_F_1	BY915513
	E_ET_maV3_01A03_F_1	BY915514
	E_ET_maV3_01A04_F_1	BY915515
	E_ET_maV3_01A05_F_1	BY915516
	E_ET_maV3_01A06_F_1	BY915517
	E_ET_maV3_01A07_F_1	BY915518
	E_ET_maV3_01A08_F_1	BY915519
	E_ET_maV3_01A09_F_1	BY915520
	E_ET_maV3_01A10_F_1	BY915521
	E_ET_maV3_01A11_F_1	BY915522
	E_ET_maV3_01A12_F_1	BY915523
	E_ET_maV3_01B01_F_1	BY915524
	E_ET_maV3_01B02_F_1	BY915525
	E_ET_maV3_01B03_F_1	BY915526
	E_ET_maV3_01B04_F_1	BY915527
	E_ET_maV3_01B05_F_1	BY915528
	E_ET_maV3_01B06_F_1	BY915529
	E_ET_maV3_01B07_F_1	BY915530
	E_ET_maV3_01B08_F_1	BY915531
	E_ET_maV3_01B09_F_1	BY915532
	E_ET_maV3_01B10_F_1	BY915533
	E_ET_maV3_01B11_F_1	BY915534
	E_ET_maV3_01B12_F_1	BY915535
	E_ET_maV3_01C01_F_1	BY915536
	E_ET_maV3_01C02_F_1	BY915537
	E_ET_maV3_01C04_F_1	BY915538
	E_ET_maV3_01C05_F_1	BY915539
	E_ET_maV3_01C06_F_1	BY915540
	E_ET_maV3_01C07_F_1	BY915541
	E_ET_maV3_01C08_F_1	BY915542
	E_ET_maV3_01C09_F_1	BY915543
	E_ET_maV3_01C10_F_1	BY915544
	E_ET_maV3_01C11_F_1	BY915545
	E_ET_maV3_01C12_F_1	BY915546
	E_ET_maV3_01D01_F_1	BY915547
	E_ET_maV3_01D02_F_1	BY915548
	E_ET_maV3_01D05_F_1	BY915549
	E_ET_maV3_01D06_F_1	BY915550
	E_ET_maV3_01D07_F_1	BY915551
	E_ET_maV3_01D08_F_1	BY915552
	E_ET_maV3_01D09_F_1	BY915553
	E_ET_maV3_01D10_F_1	BY915554
	E_ET_maV3_01D11_F_1	BY915555
	E_ET_maV3_01D12_F_1	BY915556
	E_ET_maV3_01E01_F_1	BY915557
	E_ET_maV3_01E03_F_1	BY915558
	E_ET_maV3_01E05_F_1	BY915559

E_ET_maV3_01E06_F_1	BY915560
E_ET_maV3_01E08_F_1	BY915561
E_ET_maV3_01E09_F_1	BY915562
E_ET_maV3_01E10_F_1	BY915563
E_ET_maV3_01E11_F_1	BY915564
E_ET_maV3_01E12_F_1	BY915565
E_ET_maV3_01F01_F_1	BY915566
E_ET_maV3_01F02_F_1	BY915567
E_ET_maV3_01F03_F_1	BY915568
E_ET_maV3_01F04_F_1	BY915569
E_ET_maV3_01F05_F_1	BY915570
E_ET_maV3_01F07_F_1	BY915571
E_ET_maV3_01F08_F_1	BY915572
E_ET_maV3_01F11_F_1	BY915573
E_ET_maV3_01F12_F_1	BY915574
E_ET_maV3_01G02_F_1	BY915575
E_ET_maV3_01G05_F_1	BY915576
E_ET_maV3_01G06_F_1	BY915577
E_ET_maV3_01G08_F_1	BY915578
E_ET_maV3_01H01_F_1	BY915579
E_ET_maV3_01H02_F_1	BY915580
E_ET_maV3_01H03_F_1	BY915581
E_ET_maV3_01H05_F_1	BY915582
E_ET_maV3_01H07_F_1	BY915583
E_ET_maV3_01H10_F_1	BY915584
E_ET_maV3_01H11_F_1	BY915585
E_ET_maV3_01H12_F_1	BY915586
E_ET_maV3_02A01_F_1	BY915587
E_ET_maV3_02A02_F_1	BY915588
E_ET_maV3_02A03_F_1	BY915589
E_ET_maV3_02A05_F_1	BY915590
E_ET_maV3_02A07_F_1	BY915591
E_ET_maV3_02A08_F_1	BY915592
E_ET_maV3_02A09_F_1	BY915593
E_ET_maV3_02A10_F_1	BY915594
E_ET_maV3_02A11_F_1	BY915595
E_ET_maV3_02A12_F_1	BY915596
E_ET_maV3_02B01_F_1	BY915597
E_ET_maV3_02B02_F_1	BY915598
E_ET_maV3_02B03_F_1	BY915599
E_ET_maV3_02B04_F_1	BY915600
E_ET_maV3_02B05_F_1	BY915601
E_ET_maV3_02B06_F_1	BY915602
E_ET_maV3_02B07_F_1	BY915603
E_ET_maV3_02B08_F_1	BY915604
E_ET_maV3_02B09_F_1	BY915605
E_ET_maV3_02B10_F_1	BY915606
E_ET_maV3_02B11_F_1	BY915607
E_ET_maV3_02B12_F_1	BY915608
E_ET_maV3_02C02_F_1	BY915609
E_ET_maV3_02C03_F_1	BY915610
E_ET_maV3_02C04_F_1	BY915611
E_ET_maV3_02C06_F_1	BY915612
E_ET_maV3_02C07_F_1	BY915613

E_ET_maV3_02C09_F_1	BY915614
E_ET_maV3_02C10_F_1	BY915615
E_ET_maV3_02C11_F_1	BY915616
E_ET_maV3_02C12_F_1	BY915617
E_ET_maV3_02D02_F_1	BY915618
E_ET_maV3_02D03_F_1	BY915619
E_ET_maV3_02D04_F_1	BY915620
E_ET_maV3_02D05_F_1	BY915621
E_ET_maV3_02D06_F_1	BY915622
E_ET_maV3_02D07_F_1	BY915623
E_ET_maV3_02D08_F_1	BY915624
E_ET_maV3_02D09_F_1	BY915625
E_ET_maV3_02D11_F_1	BY915626
E_ET_maV3_02D12_F_1	BY915627
E_ET_maV3_02E01_F_1	BY915628
E_ET_maV3_02E03_F_1	BY915629
E_ET_maV3_02E05_F_1	BY915630
E_ET_maV3_02E08_F_1	BY915631
E_ET_maV3_02E09_F_1	BY915632
E_ET_maV3_02E10_F_1	BY915633
E_ET_maV3_02E11_F_1	BY915634
E_ET_maV3_02E12_F_1	BY915635
E_ET_maV3_02F02_F_1	BY915636
E_ET_maV3_02F03_F_1	BY915637
E_ET_maV3_02F04_F_1	BY915638
E_ET_maV3_02F05_F_1	BY915639
E_ET_maV3_02F06_F_1	BY915640
E_ET_maV3_02F07_F_1	BY915641
E_ET_maV3_02F08_F_1	BY915642
E_ET_maV3_02F09_F_1	BY915643
E_ET_maV3_02F10_F_1	BY915644
E_ET_maV3_02F12_F_1	BY915645
E_ET_maV3_02G01_F_1	BY915646
E_ET_maV3_02G02_F_1	BY915647
E_ET_maV3_02G03_F_1	BY915648
E_ET_maV3_02G04_F_1	BY915649
E_ET_maV3_02G05_F_1	BY915650
E_ET_maV3_02G06_F_1	BY915651
E_ET_maV3_02G08_F_1	BY915652
E_ET_maV3_02G09_F_1	BY915653
E_ET_maV3_02G10_F_1	BY915654
E_ET_maV3_02G11_F_1	BY915655
E_ET_maV3_02G12_F_1	BY915656
E_ET_maV3_02H01_F_1	BY915657
E_ET_maV3_02H02_F_1	BY915658
E_ET_maV3_02H03_F_1	BY915659
E_ET_maV3_02H04_F_1	BY915660
E_ET_maV3_02H05_F_1	BY915661
E_ET_maV3_02H06_F_1	BY915662
E_ET_maV3_02H08_F_1	BY915663
E_ET_maV3_02H10_F_1	BY915664
E_ET_maV3_02H11_F_1	BY915665
E_ET_maV3_04A01_F_1	BY915666
E_ET_maV3_04A02_F_1	BY915667

E_ET_maV3_04A04_F_1	BY915668
E_ET_maV3_04A05_F_1	BY915669
E_ET_maV3_04A08_F_1	BY915670
E_ET_maV3_04A09_F_1	BY915671
E_ET_maV3_04A10_F_1	BY915672
E_ET_maV3_04A11_F_1	BY915673
E_ET_maV3_04A12_F_1	BY915674
E_ET_maV3_04B01_F_1	BY915675
E_ET_maV3_04B02_F_1	BY915676
E_ET_maV3_04B03_F_1	BY915677
E_ET_maV3_04B05_F_1	BY915678
E_ET_maV3_04B06_F_1	BY915679
E_ET_maV3_04B07_F_1	BY915680
E_ET_maV3_04B08_F_1	BY915681
E_ET_maV3_04B09_F_1	BY915682
E_ET_maV3_04B10_F_1	BY915683
E_ET_maV3_04B11_F_1	BY915684
E_ET_maV3_04C02_F_1	BY915685
E_ET_maV3_04C03_F_1	BY915686
E_ET_maV3_04C05_F_1	BY915687
E_ET_maV3_04C06_F_1	BY915688
E_ET_maV3_04C07_F_1	BY915689
E_ET_maV3_04C08_F_1	BY915690
E_ET_maV3_04C09_F_1	BY915691
E_ET_maV3_04C10_F_1	BY915692
E_ET_maV3_04C11_F_1	BY915693
E_ET_maV3_04C12_F_1	BY915694
E_ET_maV3_04D01_F_1	BY915695
E_ET_maV3_04D02_F_1	BY915696
E_ET_maV3_04D03_F_1	BY915697
E_ET_maV3_04D04_F_1	BY915698
E_ET_maV3_04D06_F_1	BY915699
E_ET_maV3_04D08_F_1	BY915700
E_ET_maV3_04D09_F_1	BY915701
E_ET_maV3_04D10_F_1	BY915702
E_ET_maV3_04D11_F_1	BY915703
E_ET_maV3_04D12_F_1	BY915704
E_ET_maV3_05A02_F_1	BY915705
E_ET_maV3_05A04_F_1	BY915706
E_ET_maV3_05A06_F_1	BY915707
E_ET_maV3_05A07_F_1	BY915708
E_ET_maV3_05A08_F_1	BY915709
E_ET_maV3_05A09_F_1	BY915710
E_ET_maV3_05B06_F_1	BY915711
E_ET_maV3_05B07_F_1	BY915712
E_ET_maV3_05B09_F_1	BY915713
E_ET_maV3_05C01_F_1	BY915714
E_ET_maV3_05C02_F_1	BY915715
E_ET_maV3_05C03_F_1	BY915716
E_ET_maV3_05C04_F_1	BY915717
E_ET_maV3_05C07_F_1	BY915718
E_ET_maV3_05C08_F_1	BY915719
E_ET_maV3_05C09_F_1	BY915720
E_ET_maV3_05C10_F_1	BY915721

E_ET_maV3_05C11_F_1	BY915722
E_ET_maV3_05D03_F_1	BY915723
E_ET_maV3_05D04_F_1	BY915724
E_ET_maV3_05D05_F_1	BY915725
E_ET_maV3_05D08_F_1	BY915726
E_ET_maV3_05D09_F_1	BY915727
E_ET_maV3_05D11_F_1	BY915728
E_ET_maV3_05E01_F_1	BY915729
E_ET_maV3_05E03_F_1	BY915730
E_ET_maV3_05E04_F_1	BY915731
E_ET_maV3_05E05_F_1	BY915732
E_ET_maV3_05E07_F_1	BY915733
E_ET_maV3_05E08_F_1	BY915734
E_ET_maV3_05E09_F_1	BY915735
E_ET_maV3_05E10_F_1	BY915736
E_ET_maV3_05E11_F_1	BY915737
E_ET_maV3_05E12_F_1	BY915738
E_ET_maV3_05F01_F_1	BY915739
E_ET_maV3_05F02_F_1	BY915740
E_ET_maV3_05F04_F_1	BY915741
E_ET_maV3_05F05_F_1	BY915742
E_ET_maV3_05F06_F_1	BY915743
E_ET_maV3_05F07_F_1	BY915744
E_ET_maV3_05F08_F_1	BY915745
E_ET_maV3_05F10_F_1	BY915746
E_ET_maV3_05G01_F_1	BY915747
E_ET_maV3_05G07_F_1	BY915748
E_ET_maV3_05G08_F_1	BY915749
E_ET_maV3_05G09_F_1	BY915750
E_ET_maV3_05G10_F_1	BY915751
E_ET_maV3_05G11_F_1	BY915752
E_ET_maV3_05H02_F_1	BY915753
E_ET_maV3_05H03_F_1	BY915754
E_ET_maV3_05H04_F_1	BY915755
E_ET_maV3_05H05_F_1	BY915756
E_ET_maV3_05H06_F_1	BY915757
E_ET_maV3_05H07_F_1	BY915758
E_ET_maV3_05H08_F_1	BY915759
E_ET_maV3_05H09_F_1	BY915760
E_ET_maV3_05H10_F_1	BY915761
E_ET_maV3_05H11_F_1	BY915762
E_ET_maV3_05H12_F_1	BY915763
E_ET_maV3_06A01_F_1	BY915764
E_ET_maV3_06A02_F_1	BY915765
E_ET_maV3_06A03_F_1	BY915766
E_ET_maV3_06A04_F_1	BY915767
E_ET_maV3_06A05_F_1	BY915768
E_ET_maV3_06A06_F_1	BY915769
E_ET_maV3_06A07_F_1	BY915770
E_ET_maV3_06A09_F_1	BY915771
E_ET_maV3_06A10_F_1	BY915772
E_ET_maV3_06A11_F_1	BY915773
E_ET_maV3_06B02_F_1	BY915774
E_ET_maV3_06B03_F_1	BY915775

E_ET_maV3_06B04_F_1	BY915776
E_ET_maV3_06B06_F_1	BY915777
E_ET_maV3_06B08_F_1	BY915778
E_ET_maV3_06B09_F_1	BY915779
E_ET_maV3_06B10_F_1	BY915780
E_ET_maV3_06B11_F_1	BY915781
E_ET_maV3_06B12_F_1	BY915782
E_ET_maV3_06C01_F_1	BY915783
E_ET_maV3_06C02_F_1	BY915784
E_ET_maV3_06C03_F_1	BY915785
E_ET_maV3_06C04_F_1	BY915786
E_ET_maV3_06C05_F_1	BY915787
E_ET_maV3_06C07_F_1	BY915788
E_ET_maV3_06C08_F_1	BY915789
E_ET_maV3_06C09_F_1	BY915790
E_ET_maV3_06C10_F_1	BY915791
E_ET_maV3_06C11_F_1	BY915792
E_ET_maV3_06C12_F_1	BY915793
E_ET_maV3_06D01_F_1	BY915794
E_ET_maV3_06D02_F_1	BY915795
E_ET_maV3_06D04_F_1	BY915796
E_ET_maV3_06D05_F_1	BY915797
E_ET_maV3_06D07_F_1	BY915798
E_ET_maV3_06D08_F_1	BY915799
E_ET_maV3_06D09_F_1	BY915800
E_ET_maV3_06D10_F_1	BY915801
E_ET_maV3_06D11_F_1	BY915802
E_ET_maV3_06D12_F_1	BY915803
E_ET_maV3_06E04_F_1	BY915804
E_ET_maV3_06E05_F_1	BY915805
E_ET_maV3_06E06_F_1	BY915806
E_ET_maV3_06E07_F_1	BY915807
E_ET_maV3_06E08_F_1	BY915808
E_ET_maV3_06E09_F_1	BY915809
E_ET_maV3_06E11_F_1	BY915810
E_ET_maV3_06E12_F_1	BY915811
E_ET_maV3_06F01_F_1	BY915812
E_ET_maV3_06F02_F_1	BY915813
E_ET_maV3_06F03_F_1	BY915814
E_ET_maV3_06F04_F_1	BY915815
E_ET_maV3_06F05_F_1	BY915816
E_ET_maV3_06F06_F_1	BY915817
E_ET_maV3_06F07_F_1	BY915818
E_ET_maV3_06F08_F_1	BY915819
E_ET_maV3_06F09_F_1	BY915820
E_ET_maV3_06F10_F_1	BY915821
E_ET_maV3_06F12_F_1	BY915822
E_ET_maV3_06G01_F_1	BY915823
E_ET_maV3_06G03_F_1	BY915824
E_ET_maV3_06G04_F_1	BY915825
E_ET_maV3_06G07_F_1	BY915826
E_ET_maV3_06G09_F_1	BY915827
E_ET_maV3_06G10_F_1	BY915828
E_ET_maV3_06G11_F_1	BY915829

E_ET_maV3_06G12_F_1	BY915830
E_ET_maV3_06H01_F_1	BY915831
E_ET_maV3_06H02_F_1	BY915832
E_ET_maV3_06H03_F_1	BY915833
E_ET_maV3_06H05_F_1	BY915834
E_ET_maV3_06H07_F_1	BY915835
E_ET_maV3_06H09_F_1	BY915836
E_ET_maV3_06H11_F_1	BY915837
E_ET_maV3_06H12_F_1	BY915838
E_ET_maV3_07A01_F_1	BY915839
E_ET_maV3_07A02_F_1	BY915840
E_ET_maV3_07A04_F_1	BY915841
E_ET_maV3_07A05_F_1	BY915842
E_ET_maV3_07A06_F_1	BY915843
E_ET_maV3_07A08_F_1	BY915844
E_ET_maV3_07A09_F_1	BY915845
E_ET_maV3_07A10_F_1	BY915846
E_ET_maV3_07A12_F_1	BY915847
E_ET_maV3_07B01_F_1	BY915848
E_ET_maV3_07B02_F_1	BY915849
E_ET_maV3_07B03_F_1	BY915850
E_ET_maV3_07B04_F_1	BY915851
E_ET_maV3_07B06_F_1	BY915852
E_ET_maV3_07B09_F_1	BY915853
E_ET_maV3_07B10_F_1	BY915854
E_ET_maV3_07B11_F_1	BY915855
E_ET_maV3_07B12_F_1	BY915856
E_ET_maV3_07C01_F_1	BY915857
E_ET_maV3_07C02_F_1	BY915858
E_ET_maV3_07C03_F_1	BY915859
E_ET_maV3_07C04_F_1	BY915860
E_ET_maV3_07C06_F_1	BY915861
E_ET_maV3_07C07_F_1	BY915862
E_ET_maV3_07C08_F_1	BY915863
E_ET_maV3_07C09_F_1	BY915864
E_ET_maV3_07C11_F_1	BY915865
E_ET_maV3_07C12_F_1	BY915866
E_ET_maV3_07D02_F_1	BY915867
E_ET_maV3_07D04_F_1	BY915868
E_ET_maV3_07D05_F_1	BY915869
E_ET_maV3_07D06_F_1	BY915870
E_ET_maV3_07D07_F_1	BY915871
E_ET_maV3_07D09_F_1	BY915872
E_ET_maV3_07D10_F_1	BY915873
E_ET_maV3_07D11_F_1	BY915874
E_ET_maV3_07D12_F_1	BY915875
E_ET_maV3_07E03_F_1	BY915876
E_ET_maV3_07E04_F_1	BY915877
E_ET_maV3_07E06_F_1	BY915878
E_ET_maV3_07E07_F_1	BY915879
E_ET_maV3_07E08_F_1	BY915880
E_ET_maV3_07E09_F_1	BY915881
E_ET_maV3_07E10_F_1	BY915882
E_ET_maV3_07E11_F_1	BY915883

E_ET_maV3_07E12_F_1	BY915884
E_ET_maV3_07F01_F_1	BY915885
E_ET_maV3_07F02_F_1	BY915886
E_ET_maV3_07F03_F_1	BY915887
E_ET_maV3_07F04_F_1	BY915888
E_ET_maV3_07F05_F_1	BY915889
E_ET_maV3_07F06_F_1	BY915890
E_ET_maV3_07F07_F_1	BY915891
E_ET_maV3_07F09_F_1	BY915892
E_ET_maV3_07F10_F_1	BY915893
E_ET_maV3_07F11_F_1	BY915894
E_ET_maV3_07F12_F_1	BY915895
E_ET_maV3_07G01_F_1	BY915896
E_ET_maV3_07G02_F_1	BY915897
E_ET_maV3_07G03_F_1	BY915898
E_ET_maV3_07G04_F_1	BY915899
E_ET_maV3_07G07_F_1	BY915900
E_ET_maV3_07G08_F_1	BY915901
E_ET_maV3_07G10_F_1	BY915902
E_ET_maV3_07G11_F_1	BY915903
E_ET_maV3_07H02_F_1	BY915904
E_ET_maV3_07H03_F_1	BY915905
E_ET_maV3_07H04_F_1	BY915906
E_ET_maV3_07H06_F_1	BY915907
E_ET_maV3_07H07_F_1	BY915908
E_ET_maV3_07H08_F_1	BY915909
E_ET_maV3_07H09_F_1	BY915910
E_ET_maV3_07H10_F_1	BY915911
E_ET_maV3_07H12_F_1	BY915912
E_ET_maV3_08A01_F_1	BY915913
E_ET_maV3_08A04_F_1	BY915914
E_ET_maV3_08A09_F_1	BY915915
E_ET_maV3_08B01_F_1	BY915916
E_ET_maV3_08B02_F_1	BY915917
E_ET_maV3_08B04_F_1	BY915918
E_ET_maV3_08B06_F_1	BY915919
E_ET_maV3_08B07_F_1	BY915920
E_ET_maV3_08B11_F_1	BY915921
E_ET_maV3_08B12_F_1	BY915922
E_ET_maV3_08C02_F_1	BY915923
E_ET_maV3_08C05_F_1	BY915924
E_ET_maV3_08C07_F_1	BY915925
E_ET_maV3_08C11_F_1	BY915926
E_ET_maV3_08D01_F_1	BY915927
E_ET_maV3_08D09_F_1	BY915928
E_ET_maV3_08D10_F_1	BY915929
E_ET_maV3_08D11_F_1	BY915930
E_ET_maV3_08E01_F_1	BY915931
E_ET_maV3_08E03_F_1	BY915932
E_ET_maV3_08E04_F_1	BY915933
E_ET_maV3_08E07_F_1	BY915934
E_ET_maV3_08E08_F_1	BY915935
E_ET_maV3_08E09_F_1	BY915936
E_ET_maV3_08E10_F_1	BY915937

E_ET_maV3_08E11_F_1	BY915938
E_ET_maV3_08E12_F_1	BY915939
E_ET_maV3_08F01_F_1	BY915940
E_ET_maV3_08F02_F_1	BY915941
E_ET_maV3_08F04_F_1	BY915942
E_ET_maV3_08F05_F_1	BY915943
E_ET_maV3_08F06_F_1	BY915944
E_ET_maV3_08F08_F_1	BY915945
E_ET_maV3_08F09_F_1	BY915946
E_ET_maV3_08F10_F_1	BY915947
E_ET_maV3_08F11_F_1	BY915948
E_ET_maV3_08F12_F_1	BY915949
E_ET_maV3_08G02_F_1	BY915950
E_ET_maV3_08G04_F_1	BY915951
E_ET_maV3_08G05_F_1	BY915952
E_ET_maV3_08G06_F_1	BY915953
E_ET_maV3_08G07_F_1	BY915954
E_ET_maV3_08G08_F_1	BY915955
E_ET_maV3_08G12_F_1	BY915956
E_ET_maV3_08H01_F_1	BY915957
E_ET_maV3_08H02_F_1	BY915958
E_ET_maV3_08H05_F_1	BY915959
E_ET_maV3_08H06_F_1	BY915960
E_ET_maV3_08H07_F_1	BY915961
E_ET_maV3_08H09_F_1	BY915962
E_ET_maV3_08H10_F_1	BY915963
E_ET_maV3_08H11_F_1	BY915964
E_ET_maV3_08H12_F_1	BY915965
E_ET_maV3_09A01_F_1	BY915966
E_ET_maV3_09A02_F_1	BY915967
E_ET_maV3_09A03_F_1	BY915968
E_ET_maV3_09A04_F_1	BY915969
E_ET_maV3_09A06_F_1	BY915970
E_ET_maV3_09A07_F_1	BY915971
E_ET_maV3_09A08_F_1	BY915972
E_ET_maV3_09A09_F_1	BY915973
E_ET_maV3_09A10_F_1	BY915974
E_ET_maV3_09A11_F_1	BY915975
E_ET_maV3_09A12_F_1	BY915976
E_ET_maV3_09B01_F_1	BY915977
E_ET_maV3_09B02_F_1	BY915978
E_ET_maV3_09B03_F_1	BY915979
E_ET_maV3_09B04_F_1	BY915980
E_ET_maV3_09B05_F_1	BY915981
E_ET_maV3_09B06_F_1	BY915982
E_ET_maV3_09B07_F_1	BY915983
E_ET_maV3_09B08_F_1	BY915984
E_ET_maV3_09B09_F_1	BY915985
E_ET_maV3_09B10_F_1	BY915986
E_ET_maV3_09B11_F_1	BY915987
E_ET_maV3_09B12_F_1	BY915988
E_ET_maV3_09C01_F_1	BY915989
E_ET_maV3_09C02_F_1	BY915990
E_ET_maV3_09C04_F_1	BY915991

E_ET_maV3_09C05_F_1	BY915992
E_ET_maV3_09C06_F_1	BY915993
E_ET_maV3_09C07_F_1	BY915994
E_ET_maV3_09C08_F_1	BY915995
E_ET_maV3_09C09_F_1	BY915996
E_ET_maV3_09C10_F_1	BY915997
E_ET_maV3_09C12_F_1	BY915998
E_ET_maV3_09D01_F_1	BY915999
E_ET_maV3_09D02_F_1	BY916000
E_ET_maV3_09D03_F_1	BY916001
E_ET_maV3_09D04_F_1	BY916002
E_ET_maV3_09D06_F_1	BY916003
E_ET_maV3_09D07_F_1	BY916004
E_ET_maV3_09D08_F_1	BY916005
E_ET_maV3_09D11_F_1	BY916006
E_ET_maV3_09D12_F_1	BY916007
E_ET_maV3_09E01_F_1	BY916008
E_ET_maV3_09E03_F_1	BY916009
E_ET_maV3_09E05_F_1	BY916010
E_ET_maV3_09E07_F_1	BY916011
E_ET_maV3_09E08_F_1	BY916012
E_ET_maV3_09E11_F_1	BY916013
E_ET_maV3_09E12_F_1	BY916014
E_ET_maV3_09F01_F_1	BY916015
E_ET_maV3_09F03_F_1	BY916016
E_ET_maV3_09F06_F_1	BY916017
E_ET_maV3_09F07_F_1	BY916018
E_ET_maV3_09F08_F_1	BY916019
E_ET_maV3_09F09_F_1	BY916020
E_ET_maV3_09F10_F_1	BY916021
E_ET_maV3_09F11_F_1	BY916022
E_ET_maV3_09F12_F_1	BY916023
E_ET_maV3_09G01_F_1	BY916024
E_ET_maV3_09G02_F_1	BY916025
E_ET_maV3_09G03_F_1	BY916026
E_ET_maV3_09G04_F_1	BY916027
E_ET_maV3_09G05_F_1	BY916028
E_ET_maV3_09G06_F_1	BY916029
E_ET_maV3_09G07_F_1	BY916030
E_ET_maV3_09G08_F_1	BY916031
E_ET_maV3_09G09_F_1	BY916032
E_ET_maV3_09G10_F_1	BY916033
E_ET_maV3_09G11_F_1	BY916034
E_ET_maV3_09G12_F_1	BY916035
E_ET_maV3_09H01_F_1	BY916036
E_ET_maV3_09H02_F_1	BY916037
E_ET_maV3_09H03_F_1	BY916038
E_ET_maV3_09H04_F_1	BY916039
E_ET_maV3_09H05_F_1	BY916040
E_ET_maV3_09H07_F_1	BY916041
E_ET_maV3_09H08_F_1	BY916042
E_ET_maV3_09H09_F_1	BY916043
E_ET_maV3_09H10_F_1	BY916044
E_ET_maV3_09H11_F_1	BY916045

E_ET_maV3_09H12_F_1	BY916046
E_ET_maV3_10A01_F_1	BY916047
E_ET_maV3_10A02_F_1	BY916048
E_ET_maV3_10A03_F_1	BY916049
E_ET_maV3_10A04_F_1	BY916050
E_ET_maV3_10A05_F_1	BY916051
E_ET_maV3_10A06_F_1	BY916052
E_ET_maV3_10A07_F_1	BY916053
E_ET_maV3_10A08_F_1	BY916054
E_ET_maV3_10A09_F_1	BY916055
E_ET_maV3_10A10_F_1	BY916056
E_ET_maV3_10A11_F_1	BY916057
E_ET_maV3_10A12_F_1	BY916058
E_ET_maV3_10B01_F_1	BY916059
E_ET_maV3_10B03_F_1	BY916060
E_ET_maV3_10B04_F_1	BY916061
E_ET_maV3_10B05_F_1	BY916062
E_ET_maV3_10B06_F_1	BY916063
E_ET_maV3_10B07_F_1	BY916064
E_ET_maV3_10B08_F_1	BY916065
E_ET_maV3_10B09_F_1	BY916066
E_ET_maV3_10B10_F_1	BY916067
E_ET_maV3_10B11_F_1	BY916068
E_ET_maV3_10B12_F_1	BY916069
E_ET_maV3_10C01_F_1	BY916070
E_ET_maV3_10C02_F_1	BY916071
E_ET_maV3_10C04_F_1	BY916072
E_ET_maV3_10C05_F_1	BY916073
E_ET_maV3_10C06_F_1	BY916074
E_ET_maV3_10C07_F_1	BY916075
E_ET_maV3_10C08_F_1	BY916076
E_ET_maV3_10C09_F_1	BY916077
E_ET_maV3_10C10_F_1	BY916078
E_ET_maV3_10C11_F_1	BY916079
E_ET_maV3_10C12_F_1	BY916080
E_ET_maV3_10D01_F_1	BY916081
E_ET_maV3_10D02_F_1	BY916082
E_ET_maV3_10D04_F_1	BY916083
E_ET_maV3_10D05_F_1	BY916084
E_ET_maV3_10D06_F_1	BY916085
E_ET_maV3_10D07_F_1	BY916086
E_ET_maV3_10D08_F_1	BY916087
E_ET_maV3_10D10_F_1	BY916088
E_ET_maV3_10D12_F_1	BY916089
E_ET_maV3_11A01_F_1	BY916090
E_ET_maV3_11A02_F_1	BY916091
E_ET_maV3_11A03_F_1	BY916092
E_ET_maV3_11A04_F_1	BY916093
E_ET_maV3_11A05_F_1	BY916094
E_ET_maV3_11A06_F_1	BY916095
E_ET_maV3_11A09_F_1	BY916096
E_ET_maV3_11A10_F_1	BY916097
E_ET_maV3_11A11_F_1	BY916098
E_ET_maV3_11B02_F_1	BY916099

E_ET_maV3_11B04_F_1	BY916100
E_ET_maV3_11B05_F_1	BY916101
E_ET_maV3_11B09_F_1	BY916102
E_ET_maV3_11B10_F_1	BY916103
E_ET_maV3_11B11_F_1	BY916104
E_ET_maV3_11B12_F_1	BY916105
E_ET_maV3_11C01_F_1	BY916106
E_ET_maV3_11C02_F_1	BY916107
E_ET_maV3_11C03_F_1	BY916108
E_ET_maV3_11C04_F_1	BY916109
E_ET_maV3_11C05_F_1	BY916110
E_ET_maV3_11C08_F_1	BY916111
E_ET_maV3_11C09_F_1	BY916112
E_ET_maV3_11C10_F_1	BY916113
E_ET_maV3_11C11_F_1	BY916114
E_ET_maV3_11C12_F_1	BY916115
E_ET_maV3_11D03_F_1	BY916116
E_ET_maV3_11D04_F_1	BY916117
E_ET_maV3_11D05_F_1	BY916118
E_ET_maV3_11D06_F_1	BY916119
E_ET_maV3_11D07_F_1	BY916120
E_ET_maV3_11D08_F_1	BY916121
E_ET_maV3_11D09_F_1	BY916122
E_ET_maV3_11D11_F_1	BY916123
E_ET_maV3_11E01_F_1	BY916124
E_ET_maV3_11E02_F_1	BY916125
E_ET_maV3_11E03_F_1	BY916126
E_ET_maV3_11E04_F_1	BY916127
E_ET_maV3_11E05_F_1	BY916128
E_ET_maV3_11E06_F_1	BY916129
E_ET_maV3_11E07_F_1	BY916130
E_ET_maV3_11E08_F_1	BY916131
E_ET_maV3_11E09_F_1	BY916132
E_ET_maV3_11E10_F_1	BY916133
E_ET_maV3_11E11_F_1	BY916134
E_ET_maV3_11E12_F_1	BY916135
E_ET_maV3_11F03_F_1	BY916136
E_ET_maV3_11F05_F_1	BY916137
E_ET_maV3_11F06_F_1	BY916138
E_ET_maV3_11F07_F_1	BY916139
E_ET_maV3_11F08_F_1	BY916140
E_ET_maV3_11F09_F_1	BY916141
E_ET_maV3_11F10_F_1	BY916142
E_ET_maV3_11F11_F_1	BY916143
E_ET_maV3_11F12_F_1	BY916144
E_ET_maV3_11G01_F_1	BY916145
E_ET_maV3_11G02_F_1	BY916146
E_ET_maV3_11G03_F_1	BY916147
E_ET_maV3_11G04_F_1	BY916148
E_ET_maV3_11G05_F_1	BY916149
E_ET_maV3_11G07_F_1	BY916150
E_ET_maV3_11G08_F_1	BY916151
E_ET_maV3_11G09_F_1	BY916152
E_ET_maV3_11G10_F_1	BY916153

E_ET_maV3_11G11_F_1	BY916154
E_ET_maV3_11G12_F_1	BY916155
E_ET_maV3_11H01_F_1	BY916156
E_ET_maV3_11H02_F_1	BY916157
E_ET_maV3_11H03_F_1	BY916158
E_ET_maV3_11H04_F_1	BY916159
E_ET_maV3_11H05_F_1	BY916160
E_ET_maV3_11H06_F_1	BY916161
E_ET_maV3_11H07_F_1	BY916162
E_ET_maV3_11H08_F_1	BY916163
E_ET_maV3_11H09_F_1	BY916164
E_ET_maV3_11H10_F_1	BY916165
E_ET_maV3_11H11_F_1	BY916166
E_ET_maV3_12A01_F_1	BY916167
E_ET_maV3_12A02_F_1	BY916168
E_ET_maV3_12A03_F_1	BY916169
E_ET_maV3_12A05_F_1	BY916170
E_ET_maV3_12A06_F_1	BY916171
E_ET_maV3_12A07_F_1	BY916172
E_ET_maV3_12A09_F_1	BY916173
E_ET_maV3_12A10_F_1	BY916174
E_ET_maV3_12A11_F_1	BY916175
E_ET_maV3_12A12_F_1	BY916176
E_ET_maV3_12B01_F_1	BY916177
E_ET_maV3_12B02_F_1	BY916178
E_ET_maV3_12B03_F_1	BY916179
E_ET_maV3_12B04_F_1	BY916180
E_ET_maV3_12B05_F_1	BY916181
E_ET_maV3_12B06_F_1	BY916182
E_ET_maV3_12B07_F_1	BY916183
E_ET_maV3_12B08_F_1	BY916184
E_ET_maV3_12B10_F_1	BY916185
E_ET_maV3_12B11_F_1	BY916186
E_ET_maV3_12B12_F_1	BY916187
E_ET_maV3_12C01_F_1	BY916188
E_ET_maV3_12C03_F_1	BY916189
E_ET_maV3_12C05_F_1	BY916190
E_ET_maV3_12C07_F_1	BY916191
E_ET_maV3_12C09_F_1	BY916192
E_ET_maV3_12C11_F_1	BY916193
E_ET_maV3_12C12_F_1	BY916194
E_ET_maV3_12D01_F_1	BY916195
E_ET_maV3_12D03_F_1	BY916196
E_ET_maV3_12D04_F_1	BY916197
E_ET_maV3_12D05_F_1	BY916198
E_ET_maV3_12D06_F_1	BY916199
E_ET_maV3_12D07_F_1	BY916200
E_ET_maV3_12D08_F_1	BY916201
E_ET_maV3_12D09_F_1	BY916202
E_ET_maV3_12D10_F_1	BY916203
E_ET_maV3_12D11_F_1	BY916204
E_ET_maV3_12D12_F_1	BY916205
E_ET_maV3_12E01_F_1	BY916206
E_ET_maV3_12E02_F_1	BY916207

E_ET_maV3_12E04_F_1	BY916208
E_ET_maV3_12E05_F_1	BY916209
E_ET_maV3_12E06_F_1	BY916210
E_ET_maV3_12E07_F_1	BY916211
E_ET_maV3_12E08_F_1	BY916212
E_ET_maV3_12E09_F_1	BY916213
E_ET_maV3_12E10_F_1	BY916214
E_ET_maV3_12E11_F_1	BY916215
E_ET_maV3_12E12_F_1	BY916216
E_ET_maV3_12F01_F_1	BY916217
E_ET_maV3_12F02_F_1	BY916218
E_ET_maV3_12F03_F_1	BY916219
E_ET_maV3_12F04_F_1	BY916220
E_ET_maV3_12F05_F_1	BY916221
E_ET_maV3_12F06_F_1	BY916222
E_ET_maV3_12F08_F_1	BY916223
E_ET_maV3_12F09_F_1	BY916224
E_ET_maV3_12F10_F_1	BY916225
E_ET_maV3_12F11_F_1	BY916226
E_ET_maV3_12F12_F_1	BY916227
E_ET_maV3_12G01_F_1	BY916228
E_ET_maV3_12G02_F_1	BY916229
E_ET_maV3_12G03_F_1	BY916230
E_ET_maV3_12G05_F_1	BY916231
E_ET_maV3_12G06_F_1	BY916232
E_ET_maV3_12G07_F_1	BY916233
E_ET_maV3_12G08_F_1	BY916234
E_ET_maV3_12G09_F_1	BY916235
E_ET_maV3_12G10_F_1	BY916236
E_ET_maV3_12H01_F_1	BY916237
E_ET_maV3_12H02_F_1	BY916238
E_ET_maV3_12H05_F_1	BY916239
E_ET_maV3_12H08_F_1	BY916240
E_ET_maV3_12H09_F_1	BY916241
E_ET_maV3_12H10_F_1	BY916242
E_ET_maV3_12H11_F_1	BY916243
E_ET_maV3_12H12_F_1	BY916244
E_ET_maV3_13A01_F_1	BY916245
E_ET_maV3_13A02_F_1	BY916246
E_ET_maV3_13A03_F_1	BY916247
E_ET_maV3_13A04_F_1	BY916248
E_ET_maV3_13A05_F_1	BY916249
E_ET_maV3_13A07_F_1	BY916250
E_ET_maV3_13A08_F_1	BY916251
E_ET_maV3_13A09_F_1	BY916252
E_ET_maV3_13A10_F_1	BY916253
E_ET_maV3_13A11_F_1	BY916254
E_ET_maV3_13A12_F_1	BY916255
E_ET_maV3_13B01_F_1	BY916256
E_ET_maV3_13B02_F_1	BY916257
E_ET_maV3_13B03_F_1	BY916258
E_ET_maV3_13B04_F_1	BY916259
E_ET_maV3_13B05_F_1	BY916260
E_ET_maV3_13B06_F_1	BY916261

	E_ET_maV3_13B07_F_1	BY916262
	E_ET_maV3_13B08_F_1	BY916263
	E_ET_maV3_13B09_F_1	BY916264
	E_ET_maV3_13B10_F_1	BY916265
	E_ET_maV3_13B11_F_1	BY916266
	E_ET_maV3_13B12_F_1	BY916267
	E_ET_maV3_13C01_F_1	BY916268
	E_ET_maV3_13C02_F_1	BY916269
	E_ET_maV3_13C03_F_1	BY916270
	E_ET_maV3_13C04_F_1	BY916271
	E_ET_maV3_13C05_F_1	BY916272
	E_ET_maV3_13C06_F_1	BY916273
	E_ET_maV3_13C09_F_1	BY916274
	E_ET_maV3_13C10_F_1	BY916275
	E_ET_maV3_13C11_F_1	BY916276
	E_ET_maV3_13D01_F_1	BY916277
	E_ET_maV3_13D03_F_1	BY916278
	E_ET_maV3_13D04_F_1	BY916279
	E_ET_maV3_13D05_F_1	BY916280
	E_ET_maV3_13D06_F_1	BY916281
	E_ET_maV3_13D07_F_1	BY916282
	E_ET_maV3_13D09_F_1	BY916283
	E_ET_maV3_13D10_F_1	BY916284
	E_ET_maV3_13D11_F_1	BY916285
	E_ET_maV3_13D12_F_1	BY916286
MSV3	E_ET_MSV3_01A03_F_0	BB985122
	E_ET_MSV3_01A04_F_0	BB985123
	E_ET_MSV3_01A05_F_0	BB985124
	E_ET_MSV3_01A06_F_0	BB985125
	E_ET_MSV3_01A09_F_0	BB985126
	E_ET_MSV3_01A10_F_0	BB985127
	E_ET_MSV3_01A10_R_0	BB990688
	E_ET_MSV3_01A11_F_0	BB985128
	E_ET_MSV3_01A12_F_0	BB985129
	E_ET_MSV3_01A12_R_0	BB990689
	E_ET_MSV3_01B01_F_0	BB985130
	E_ET_MSV3_01B01_R_0	BB990690
	E_ET_MSV3_01B02_F_0	BB985131
	E_ET_MSV3_01B02_R_0	BB990691
	E_ET_MSV3_01B03_F_0	BB985132
	E_ET_MSV3_01B05_F_0	BB985133
	E_ET_MSV3_01B06_F_0	BB985134
	E_ET_MSV3_01B07_F_0	BB985135
	E_ET_MSV3_01B08_F_0	BB985136
	E_ET_MSV3_01B09_F_0	BB985137
	E_ET_MSV3_01B09_R_0	BB990692
	E_ET_MSV3_01B11_R_0	BB990693
	E_ET_MSV3_01B12_F_0	BB985138
	E_ET_MSV3_01B12_R_0	BB990694
	E_ET_MSV3_01C01_F_0	BB985139
	E_ET_MSV3_01C01_R_0	BB990695
	E_ET_MSV3_01C02_F_0	BB985140
	E_ET_MSV3_01C02_R_0	BB990696
	E_ET_MSV3_01C03_F_0	BB985141

E_ET_MSV3_01C05_F_0	BB985142
E_ET_MSV3_01C07_F_0	BB985143
E_ET_MSV3_01C08_R_0	BB990697
E_ET_MSV3_01C09_F_0	BB985144
E_ET_MSV3_01C09_R_0	BB990698
E_ET_MSV3_01C11_F_0	BB985145
E_ET_MSV3_01C11_R_0	BB990699
E_ET_MSV3_01C12_F_0	BB985146
E_ET_MSV3_01C12_R_0	BB990700
E_ET_MSV3_01D01_F_0	BB985147
E_ET_MSV3_01D02_F_0	BB985148
E_ET_MSV3_01D02_R_0	BB990701
E_ET_MSV3_01D03_F_0	BB985149
E_ET_MSV3_01D03_R_0	BB990702
E_ET_MSV3_01D04_F_0	BB985150
E_ET_MSV3_01D05_F_0	BB985151
E_ET_MSV3_01D05_R_0	BB990703
E_ET_MSV3_01D06_F_0	BB985152
E_ET_MSV3_01D06_R_0	BB990704
E_ET_MSV3_01D07_R_0	BB990705
E_ET_MSV3_01D08_F_0	BB985153
E_ET_MSV3_01D08_R_0	BB990706
E_ET_MSV3_01D09_F_0	BB985154
E_ET_MSV3_01D09_R_0	BB990707
E_ET_MSV3_01D11_F_0	BB985155
E_ET_MSV3_01D11_R_0	BB990708
E_ET_MSV3_01D12_F_0	BB985156
E_ET_MSV3_01D12_R_0	BB990709
E_ET_MSV3_01E02_F_0	BB985157
E_ET_MSV3_01E03_F_0	BB985158
E_ET_MSV3_01E03_R_0	BB990710
E_ET_MSV3_01E04_F_0	BB985159
E_ET_MSV3_01E04_R_0	BB990711
E_ET_MSV3_01E05_F_0	BB985160
E_ET_MSV3_01E05_R_0	BB990712
E_ET_MSV3_01E06_F_0	BB985161
E_ET_MSV3_01E06_R_0	BB990713
E_ET_MSV3_01E07_F_0	BB985162
E_ET_MSV3_01E07_R_0	BB990714
E_ET_MSV3_01E08_F_0	BB985163
E_ET_MSV3_01E09_F_0	BB985164
E_ET_MSV3_01E09_R_0	BB990715
E_ET_MSV3_01E10_R_0	BB990716
E_ET_MSV3_01E11_F_0	BB985165
E_ET_MSV3_01E11_R_0	BB990717
E_ET_MSV3_01E12_F_0	BB985166
E_ET_MSV3_01E12_R_0	BB990718
E_ET_MSV3_01F01_F_0	BB985167
E_ET_MSV3_01F01_R_0	BB990719
E_ET_MSV3_01F02_F_0	BB985168
E_ET_MSV3_01F02_R_0	BB990720
E_ET_MSV3_01F03_F_0	BB985169
E_ET_MSV3_01F05_F_0	BB985170
E_ET_MSV3_01F05_R_0	BB990721

E_ET_MSV3_01F06_F_0	BB985171
E_ET_MSV3_01F06_R_0	BB990722
E_ET_MSV3_01F07_F_0	BB985172
E_ET_MSV3_01F08_F_0	BB985173
E_ET_MSV3_01F08_R_0	BB990723
E_ET_MSV3_01F09_F_0	BB985174
E_ET_MSV3_01F09_R_0	BB990724
E_ET_MSV3_01F10_F_0	BB985175
E_ET_MSV3_01F10_R_0	BB990725
E_ET_MSV3_01F11_F_0	BB985176
E_ET_MSV3_01F11_R_0	BB990726
E_ET_MSV3_01F12_F_0	BB985177
E_ET_MSV3_01G01_F_0	BB985178
E_ET_MSV3_01G01_R_0	BB990727
E_ET_MSV3_01G02_F_0	BB985179
E_ET_MSV3_01G03_F_0	BB985180
E_ET_MSV3_01G05_F_0	BB985181
E_ET_MSV3_01G05_R_0	BB990728
E_ET_MSV3_01G06_F_0	BB985182
E_ET_MSV3_01G07_F_0	BB985183
E_ET_MSV3_01G07_R_0	BB990729
E_ET_MSV3_01G08_F_0	BB985184
E_ET_MSV3_01G09_F_0	BB985185
E_ET_MSV3_01G10_R_0	BB990730
E_ET_MSV3_01G12_F_0	BB985186
E_ET_MSV3_01G12_R_0	BB990731
E_ET_MSV3_01H01_F_0	BB985187
E_ET_MSV3_01H03_F_0	BB985188
E_ET_MSV3_01H03_R_0	BB990732
E_ET_MSV3_01H04_F_0	BB985189
E_ET_MSV3_01H05_F_0	BB985190
E_ET_MSV3_01H06_F_0	BB985191
E_ET_MSV3_01H07_F_0	BB985192
E_ET_MSV3_01H07_R_0	BB990733
E_ET_MSV3_01H08_F_0	BB985193
E_ET_MSV3_01H09_F_0	BB985194
E_ET_MSV3_01H09_R_0	BB990734
E_ET_MSV3_01H10_F_0	BB985195
E_ET_MSV3_01H10_R_0	BB990735
E_ET_MSV3_01H11_F_0	BB985196
E_ET_MSV3_02A01_R_0	BB990736
E_ET_MSV3_02A02_F_0	BB985197
E_ET_MSV3_02A02_R_0	BB990737
E_ET_MSV3_02A03_F_0	BB985198
E_ET_MSV3_02A03_R_0	BB990738
E_ET_MSV3_02A05_R_0	BB990739
E_ET_MSV3_02A06_F_0	BB985199
E_ET_MSV3_02A06_R_0	BB990740
E_ET_MSV3_02A07_F_0	BB985200
E_ET_MSV3_02A07_R_0	BB990741
E_ET_MSV3_02A08_F_0	BB985201
E_ET_MSV3_02A09_R_0	BB990742
E_ET_MSV3_02A10_R_0	BB990743
E_ET_MSV3_02A11_R_0	BB990744

E_ET_MSV3_02A12_R_0	BB990745
E_ET_MSV3_02B01_F_0	BB985202
E_ET_MSV3_02B01_R_0	BB990746
E_ET_MSV3_02B02_F_0	BB985203
E_ET_MSV3_02B02_R_0	BB990747
E_ET_MSV3_02B03_R_0	BB990748
E_ET_MSV3_02B04_F_0	BB985204
E_ET_MSV3_02B04_R_0	BB990749
E_ET_MSV3_02B06_F_0	BB985205
E_ET_MSV3_02B06_R_0	BB990750
E_ET_MSV3_02B07_F_0	BB985206
E_ET_MSV3_02B07_R_0	BB990751
E_ET_MSV3_02B08_R_0	BB990752
E_ET_MSV3_02B09_F_0	BB985207
E_ET_MSV3_02B09_R_0	BB990753
E_ET_MSV3_02B10_R_0	BB990754
E_ET_MSV3_02B11_F_0	BB985208
E_ET_MSV3_02B11_R_0	BB990755
E_ET_MSV3_02B12_R_0	BB990756
E_ET_MSV3_02C01_F_0	BB985209
E_ET_MSV3_02C01_R_0	BB990757
E_ET_MSV3_02C02_F_0	BB985210
E_ET_MSV3_02C02_R_0	BB990758
E_ET_MSV3_02C03_F_0	BB985211
E_ET_MSV3_02C04_F_0	BB985212
E_ET_MSV3_02C04_R_0	BB990759
E_ET_MSV3_02C05_F_0	BB985213
E_ET_MSV3_02C07_F_0	BB985214
E_ET_MSV3_02C07_R_0	BB990760
E_ET_MSV3_02C08_F_0	BB985215
E_ET_MSV3_02C08_R_0	BB990761
E_ET_MSV3_02C09_F_0	BB985216
E_ET_MSV3_02C10_F_0	BB985217
E_ET_MSV3_02C10_R_0	BB990762
E_ET_MSV3_02C11_F_0	BB985218
E_ET_MSV3_02D02_F_0	BB985219
E_ET_MSV3_02D02_R_0	BB990763
E_ET_MSV3_02D03_F_0	BB985220
E_ET_MSV3_02D03_R_0	BB990764
E_ET_MSV3_02D04_F_0	BB985221
E_ET_MSV3_02D04_R_0	BB990765
E_ET_MSV3_02D05_F_0	BB985222
E_ET_MSV3_02D05_R_0	BB990766
E_ET_MSV3_02D06_F_0	BB985223
E_ET_MSV3_02D06_R_0	BB990767
E_ET_MSV3_02D07_F_0	BB985224
E_ET_MSV3_02D08_F_0	BB985225
E_ET_MSV3_02D09_F_0	BB985226
E_ET_MSV3_02D09_R_0	BB990768
E_ET_MSV3_02D10_F_0	BB985227
E_ET_MSV3_02D10_R_0	BB990769
E_ET_MSV3_02D11_F_0	BB985228
E_ET_MSV3_02D11_R_0	BB990770
E_ET_MSV3_02D12_F_0	BB985229

E_ET_MSV3_02D12_R_0	BB990771
E_ET_MSV3_02E01_F_0	BB985230
E_ET_MSV3_02E01_R_0	BB990772
E_ET_MSV3_02E02_F_0	BB985231
E_ET_MSV3_02E02_R_0	BB990773
E_ET_MSV3_02E03_F_0	BB985232
E_ET_MSV3_02E06_F_0	BB985233
E_ET_MSV3_02E07_F_0	BB985234
E_ET_MSV3_02E08_F_0	BB985235
E_ET_MSV3_02E08_R_0	BB990774
E_ET_MSV3_02E09_F_0	BB985236
E_ET_MSV3_02E09_R_0	BB990775
E_ET_MSV3_02E10_F_0	BB985237
E_ET_MSV3_02E10_R_0	BB990776
E_ET_MSV3_02E11_F_0	BB985238
E_ET_MSV3_02E11_R_0	BB990777
E_ET_MSV3_02E12_F_0	BB985239
E_ET_MSV3_02F01_F_0	BB985240
E_ET_MSV3_02F02_F_0	BB985241
E_ET_MSV3_02F02_R_0	BB990778
E_ET_MSV3_02F03_F_0	BB985242
E_ET_MSV3_02F04_R_0	BB990779
E_ET_MSV3_02F05_F_0	BB985243
E_ET_MSV3_02F05_R_0	BB990780
E_ET_MSV3_02F06_F_0	BB985244
E_ET_MSV3_02F06_R_0	BB990781
E_ET_MSV3_02F07_F_0	BB985245
E_ET_MSV3_02F08_F_0	BB985246
E_ET_MSV3_02F08_R_0	BB990782
E_ET_MSV3_02F09_F_0	BB985247
E_ET_MSV3_02F09_R_0	BB990783
E_ET_MSV3_02F10_F_0	BB985248
E_ET_MSV3_02F10_R_0	BB990784
E_ET_MSV3_02F11_R_0	BB990785
E_ET_MSV3_02G01_R_0	BB990786
E_ET_MSV3_02G02_F_0	BB985249
E_ET_MSV3_02G02_R_0	BB990787
E_ET_MSV3_02G03_F_0	BB985250
E_ET_MSV3_02G04_F_0	BB985251
E_ET_MSV3_02G04_R_0	BB990788
E_ET_MSV3_02G05_R_0	BB990789
E_ET_MSV3_02G06_F_0	BB985252
E_ET_MSV3_02G06_R_0	BB990790
E_ET_MSV3_02G07_F_0	BB985253
E_ET_MSV3_02G07_R_0	BB990791
E_ET_MSV3_02G08_F_0	BB985254
E_ET_MSV3_02G08_R_0	BB990792
E_ET_MSV3_02G09_F_0	BB985255
E_ET_MSV3_02G10_F_0	BB985256
E_ET_MSV3_02G10_R_0	BB990793
E_ET_MSV3_02G11_F_0	BB985257
E_ET_MSV3_02G11_R_0	BB990794
E_ET_MSV3_02G12_F_0	BB985258
E_ET_MSV3_02G12_R_0	BB990795

E_ET_MSV3_02H01_F_0	BB985259
E_ET_MSV3_02H01_R_0	BB990796
E_ET_MSV3_02H02_F_0	BB985260
E_ET_MSV3_02H03_F_0	BB985261
E_ET_MSV3_02H03_R_0	BB990797
E_ET_MSV3_02H04_R_0	BB990798
E_ET_MSV3_02H05_F_0	BB985262
E_ET_MSV3_02H06_F_0	BB985263
E_ET_MSV3_02H07_R_0	BB990799
E_ET_MSV3_02H08_R_0	BB990800
E_ET_MSV3_02H09_R_0	BB990801
E_ET_MSV3_02H11_F_0	BB985264
E_ET_MSV3_02H12_F_0	BB985265
E_ET_MSV3_02H12_R_0	BB990802
E_ET_MSV3_03A01_F_0	BB985266
E_ET_MSV3_03A01_R_0	BB990803
E_ET_MSV3_03A02_F_0	BB985267
E_ET_MSV3_03A02_R_0	BB990804
E_ET_MSV3_03A03_F_0	BB985268
E_ET_MSV3_03A03_R_0	BB990805
E_ET_MSV3_03A04_F_0	BB985269
E_ET_MSV3_03A04_R_0	BB990806
E_ET_MSV3_03A05_F_0	BB985270
E_ET_MSV3_03A06_F_0	BB985271
E_ET_MSV3_03A06_R_0	BB990807
E_ET_MSV3_03A07_F_0	BB985272
E_ET_MSV3_03A07_R_0	BB990808
E_ET_MSV3_03A09_F_0	BB985273
E_ET_MSV3_03A09_R_0	BB990809
E_ET_MSV3_03A10_F_0	BB985274
E_ET_MSV3_03A11_F_0	BB985275
E_ET_MSV3_03A11_R_0	BB990810
E_ET_MSV3_03A12_F_0	BB985276
E_ET_MSV3_03A12_R_0	BB990811
E_ET_MSV3_03B01_F_0	BB985277
E_ET_MSV3_03B01_R_0	BB990812
E_ET_MSV3_03B02_F_0	BB985278
E_ET_MSV3_03B02_R_0	BB990813
E_ET_MSV3_03B03_F_0	BB985279
E_ET_MSV3_03B03_R_0	BB990814
E_ET_MSV3_03B04_F_0	BB985280
E_ET_MSV3_03B04_R_0	BB990815
E_ET_MSV3_03B05_F_0	BB985281
E_ET_MSV3_03B05_R_0	BB990816
E_ET_MSV3_03B06_F_0	BB985282
E_ET_MSV3_03B06_R_0	BB990817
E_ET_MSV3_03B07_F_0	BB985283
E_ET_MSV3_03B08_F_0	BB985284
E_ET_MSV3_03B08_R_0	BB990818
E_ET_MSV3_03B09_F_0	BB985285
E_ET_MSV3_03B09_R_0	BB990819
E_ET_MSV3_03B11_F_0	BB985286
E_ET_MSV3_03B11_R_0	BB990820
E_ET_MSV3_03B12_F_0	BB985287

E_ET_MSV3_03C02_F_0	BB985288
E_ET_MSV3_03C02_R_0	BB990821
E_ET_MSV3_03C03_F_0	BB985289
E_ET_MSV3_03C03_R_0	BB990822
E_ET_MSV3_03C06_F_0	BB985290
E_ET_MSV3_03C06_R_0	BB990823
E_ET_MSV3_03C07_F_0	BB985291
E_ET_MSV3_03C08_F_0	BB985292
E_ET_MSV3_03C08_R_0	BB990824
E_ET_MSV3_03C09_F_0	BB985293
E_ET_MSV3_03C09_R_0	BB990825
E_ET_MSV3_03C10_F_0	BB985294
E_ET_MSV3_03C10_R_0	BB990826
E_ET_MSV3_03D01_F_0	BB985295
E_ET_MSV3_03D01_R_0	BB990827
E_ET_MSV3_03D02_F_0	BB985296
E_ET_MSV3_03D03_F_0	BB985297
E_ET_MSV3_03D04_F_0	BB985298
E_ET_MSV3_03D05_F_0	BB985299
E_ET_MSV3_03D05_R_0	BB990828
E_ET_MSV3_03D06_F_0	BB985300
E_ET_MSV3_03D06_R_0	BB990829
E_ET_MSV3_03D07_F_0	BB985301
E_ET_MSV3_03D07_R_0	BB990830
E_ET_MSV3_03D08_F_0	BB985302
E_ET_MSV3_03D08_R_0	BB990831
E_ET_MSV3_03D09_F_0	BB985303
E_ET_MSV3_03D10_F_0	BB985304
E_ET_MSV3_03D11_F_0	BB985305
E_ET_MSV3_03D11_R_0	BB990832
E_ET_MSV3_03D12_F_0	BB985306
E_ET_MSV3_03D12_R_0	BB990833
E_ET_MSV3_03E01_F_0	BB985307
E_ET_MSV3_03E01_R_0	BB990834
E_ET_MSV3_03E02_F_0	BB985308
E_ET_MSV3_03E02_R_0	BB990835
E_ET_MSV3_03E03_F_0	BB985309
E_ET_MSV3_03E04_F_0	BB985310
E_ET_MSV3_03E04_R_0	BB990836
E_ET_MSV3_03E05_F_0	BB985311
E_ET_MSV3_03E06_F_0	BB985312
E_ET_MSV3_03E06_R_0	BB990837
E_ET_MSV3_03E08_F_0	BB985313
E_ET_MSV3_03E09_F_0	BB985314
E_ET_MSV3_03E09_R_0	BB990838
E_ET_MSV3_03E12_F_0	BB985315
E_ET_MSV3_03F01_F_0	BB985316
E_ET_MSV3_03F01_R_0	BB990839
E_ET_MSV3_03F02_F_0	BB985317
E_ET_MSV3_03F02_R_0	BB990840
E_ET_MSV3_03F03_F_0	BB985318
E_ET_MSV3_03F03_R_0	BB990841
E_ET_MSV3_03F04_F_0	BB985319
E_ET_MSV3_03F04_R_0	BB990842

E_ET_MSV3_03F05_F_0	BB985320
E_ET_MSV3_03F05_R_0	BB990843
E_ET_MSV3_03F06_F_0	BB985321
E_ET_MSV3_03F06_R_0	BB990844
E_ET_MSV3_03F07_F_0	BB985322
E_ET_MSV3_03F07_R_0	BB990845
E_ET_MSV3_03F08_F_0	BB985323
E_ET_MSV3_03F08_R_0	BB990846
E_ET_MSV3_03F09_F_0	BB985324
E_ET_MSV3_03F09_R_0	BB990847
E_ET_MSV3_03F10_F_0	BB985325
E_ET_MSV3_03F10_R_0	BB990848
E_ET_MSV3_03F11_F_0	BB985326
E_ET_MSV3_03F12_F_0	BB985327
E_ET_MSV3_03G01_F_0	BB985328
E_ET_MSV3_03G01_R_0	BB990849
E_ET_MSV3_03G02_F_0	BB985329
E_ET_MSV3_03G02_R_0	BB990850
E_ET_MSV3_03G03_F_0	BB985330
E_ET_MSV3_03G04_F_0	BB985331
E_ET_MSV3_03G04_R_0	BB990851
E_ET_MSV3_03G05_F_0	BB985332
E_ET_MSV3_03G05_R_0	BB990852
E_ET_MSV3_03G06_F_0	BB985333
E_ET_MSV3_03G06_R_0	BB990853
E_ET_MSV3_03G07_F_0	BB985334
E_ET_MSV3_03G08_F_0	BB985335
E_ET_MSV3_03G08_R_0	BB990854
E_ET_MSV3_03G09_F_0	BB985336
E_ET_MSV3_03G09_R_0	BB990855
E_ET_MSV3_03G10_F_0	BB985337
E_ET_MSV3_03G11_F_0	BB985338
E_ET_MSV3_03G11_R_0	BB990856
E_ET_MSV3_03G12_F_0	BB985339
E_ET_MSV3_03G12_R_0	BB990857
E_ET_MSV3_03H01_F_0	BB985340
E_ET_MSV3_03H01_R_0	BB990858
E_ET_MSV3_03H02_F_0	BB985341
E_ET_MSV3_03H02_R_0	BB990859
E_ET_MSV3_03H03_F_0	BB985342
E_ET_MSV3_03H03_R_0	BB990860
E_ET_MSV3_03H05_F_0	BB985343
E_ET_MSV3_03H06_F_0	BB985344
E_ET_MSV3_03H06_R_0	BB990861
E_ET_MSV3_03H07_F_0	BB985345
E_ET_MSV3_03H07_R_0	BB990862
E_ET_MSV3_03H08_F_0	BB985346
E_ET_MSV3_03H08_R_0	BB990863
E_ET_MSV3_03H09_R_0	BB990864
E_ET_MSV3_03H11_R_0	BB990865
E_ET_MSV3_03H12_F_0	BB985347
E_ET_MSV3_03H12_R_0	BB990866
E_ET_MSV3_04A01_F_0	BB985348
E_ET_MSV3_04A01_R_0	BB990867

E_ET_MSV3_04A02_F_0	BB985349
E_ET_MSV3_04A02_R_0	BB990868
E_ET_MSV3_04A03_F_0	BB985350
E_ET_MSV3_04A03_R_0	BB990869
E_ET_MSV3_04A04_F_0	BB985351
E_ET_MSV3_04A05_F_0	BB985352
E_ET_MSV3_04A06_F_0	BB985353
E_ET_MSV3_04A06_R_0	BB990870
E_ET_MSV3_04A07_F_0	BB985354
E_ET_MSV3_04A08_F_0	BB985355
E_ET_MSV3_04A08_R_0	BB990871
E_ET_MSV3_04A09_F_0	BB985356
E_ET_MSV3_04A09_R_0	BB990872
E_ET_MSV3_04A10_F_0	BB985357
E_ET_MSV3_04A11_F_0	BB985358
E_ET_MSV3_04A12_F_0	BB985359
E_ET_MSV3_04A12_R_0	BB990873
E_ET_MSV3_04B02_F_0	BB985360
E_ET_MSV3_04B02_R_0	BB990874
E_ET_MSV3_04B03_F_0	BB985361
E_ET_MSV3_04B04_F_0	BB985362
E_ET_MSV3_04B04_R_0	BB990875
E_ET_MSV3_04B05_F_0	BB985363
E_ET_MSV3_04B05_R_0	BB990876
E_ET_MSV3_04B06_F_0	BB985364
E_ET_MSV3_04B06_R_0	BB990877
E_ET_MSV3_04B07_F_0	BB985365
E_ET_MSV3_04B07_R_0	BB990878
E_ET_MSV3_04B08_R_0	BB990879
E_ET_MSV3_04B09_F_0	BB985366
E_ET_MSV3_04B10_F_0	BB985367
E_ET_MSV3_04B10_R_0	BB990880
E_ET_MSV3_04B11_F_0	BB985368
E_ET_MSV3_04B11_R_0	BB990881
E_ET_MSV3_04B12_F_0	BB985369
E_ET_MSV3_04B12_R_0	BB990882
E_ET_MSV3_04C01_R_0	BB990883
E_ET_MSV3_04C02_R_0	BB990884
E_ET_MSV3_04C03_F_0	BB985370
E_ET_MSV3_04C03_R_0	BB990885
E_ET_MSV3_04C04_F_0	BB985371
E_ET_MSV3_04C04_R_0	BB990886
E_ET_MSV3_04C05_F_0	BB985372
E_ET_MSV3_04C05_R_0	BB990887
E_ET_MSV3_04C06_F_0	BB985373
E_ET_MSV3_04C06_R_0	BB990888
E_ET_MSV3_04C07_F_0	BB985374
E_ET_MSV3_04C07_R_0	BB990889
E_ET_MSV3_04C08_F_0	BB985375
E_ET_MSV3_04C08_R_0	BB990890
E_ET_MSV3_04C09_F_0	BB985376
E_ET_MSV3_04C09_R_0	BB990891
E_ET_MSV3_04C10_F_0	BB985377
E_ET_MSV3_04C10_R_0	BB990892

E_ET_MSV3_04C11_F_0	BB985378
E_ET_MSV3_04C11_R_0	BB990893
E_ET_MSV3_04D01_F_0	BB985379
E_ET_MSV3_04D02_F_0	BB985380
E_ET_MSV3_04D03_F_0	BB985381
E_ET_MSV3_04D05_F_0	BB985382
E_ET_MSV3_04D06_F_0	BB985383
E_ET_MSV3_04D07_F_0	BB985384
E_ET_MSV3_04D07_R_0	BB990894
E_ET_MSV3_04D08_F_0	BB985385
E_ET_MSV3_04D09_F_0	BB985386
E_ET_MSV3_04D09_R_0	BB990895
E_ET_MSV3_04D10_F_0	BB985387
E_ET_MSV3_04D11_F_0	BB985388
E_ET_MSV3_04D12_F_0	BB985389
E_ET_MSV3_04D12_R_0	BB990896
E_ET_MSV3_04E01_F_0	BB985390
E_ET_MSV3_04E01_R_0	BB990897
E_ET_MSV3_04E02_F_0	BB985391
E_ET_MSV3_04E02_R_0	BB990898
E_ET_MSV3_04E03_F_0	BB985392
E_ET_MSV3_04E04_F_0	BB985393
E_ET_MSV3_04E04_R_0	BB990899
E_ET_MSV3_04E05_F_0	BB985394
E_ET_MSV3_04E06_F_0	BB985395
E_ET_MSV3_04E06_R_0	BB990900
E_ET_MSV3_04E07_F_0	BB985396
E_ET_MSV3_04E08_F_0	BB985397
E_ET_MSV3_04E09_F_0	BB985398
E_ET_MSV3_04E10_F_0	BB985399
E_ET_MSV3_04E11_F_0	BB985400
E_ET_MSV3_04E12_F_0	BB985401
E_ET_MSV3_04F01_F_0	BB985402
E_ET_MSV3_04F01_R_0	BB990901
E_ET_MSV3_04F02_F_0	BB985403
E_ET_MSV3_04F02_R_0	BB990902
E_ET_MSV3_04F03_F_0	BB985404
E_ET_MSV3_04F03_R_0	BB990903
E_ET_MSV3_04F05_F_0	BB985405
E_ET_MSV3_04F05_R_0	BB990904
E_ET_MSV3_04F06_F_0	BB985406
E_ET_MSV3_04F06_R_0	BB990905
E_ET_MSV3_04F07_F_0	BB985407
E_ET_MSV3_04F07_R_0	BB990906
E_ET_MSV3_04F08_F_0	BB985408
E_ET_MSV3_04F08_R_0	BB990907
E_ET_MSV3_04F09_F_0	BB985409
E_ET_MSV3_04F09_R_0	BB990908
E_ET_MSV3_04F10_F_0	BB985410
E_ET_MSV3_04F10_R_0	BB990909
E_ET_MSV3_04F12_F_0	BB985411
E_ET_MSV3_04G01_F_0	BB985412
E_ET_MSV3_04G01_R_0	BB990910
E_ET_MSV3_04G02_F_0	BB985413

E_ET_MSV3_04G02_R_0	BB990911
E_ET_MSV3_04G03_F_0	BB985414
E_ET_MSV3_04G03_R_0	BB990912
E_ET_MSV3_04G04_F_0	BB985415
E_ET_MSV3_04G04_R_0	BB990913
E_ET_MSV3_04G05_R_0	BB990914
E_ET_MSV3_04G06_F_0	BB985416
E_ET_MSV3_04G06_R_0	BB990915
E_ET_MSV3_04G07_F_0	BB985417
E_ET_MSV3_04G07_R_0	BB990916
E_ET_MSV3_04G08_F_0	BB985418
E_ET_MSV3_04G09_F_0	BB985419
E_ET_MSV3_04G10_F_0	BB985420
E_ET_MSV3_04G12_F_0	BB985421
E_ET_MSV3_04G12_R_0	BB990917
E_ET_MSV3_04H01_F_0	BB985422
E_ET_MSV3_04H01_R_0	BB990918
E_ET_MSV3_04H03_F_0	BB985423
E_ET_MSV3_04H03_R_0	BB990919
E_ET_MSV3_04H05_R_0	BB990920
E_ET_MSV3_04H07_F_0	BB985424
E_ET_MSV3_04H08_R_0	BB990921
E_ET_MSV3_04H10_F_0	BB985425
E_ET_MSV3_04H10_R_0	BB990922
E_ET_MSV3_05A01_F_0	BB985426
E_ET_MSV3_05A01_R_0	BB990923
E_ET_MSV3_05A02_F_0	BB985427
E_ET_MSV3_05A02_R_0	BB990924
E_ET_MSV3_05A04_F_0	BB985428
E_ET_MSV3_05A04_R_0	BB990925
E_ET_MSV3_05A05_F_0	BB985429
E_ET_MSV3_05A05_R_0	BB990926
E_ET_MSV3_05A06_F_0	BB985430
E_ET_MSV3_05A06_R_0	BB990927
E_ET_MSV3_05A07_F_0	BB985431
E_ET_MSV3_05A07_R_0	BB990928
E_ET_MSV3_05A08_F_0	BB985432
E_ET_MSV3_05A08_R_0	BB990929
E_ET_MSV3_05A09_F_0	BB985433
E_ET_MSV3_05A09_R_0	BB990930
E_ET_MSV3_05A10_F_0	BB985434
E_ET_MSV3_05A10_R_0	BB990931
E_ET_MSV3_05A11_F_0	BB985435
E_ET_MSV3_05A12_F_0	BB985436
E_ET_MSV3_05A12_R_0	BB990932
E_ET_MSV3_05B01_F_0	BB985437
E_ET_MSV3_05B01_R_0	BB990933
E_ET_MSV3_05B02_F_0	BB985438
E_ET_MSV3_05B02_R_0	BB990934
E_ET_MSV3_05B03_F_0	BB985439
E_ET_MSV3_05B03_R_0	BB990935
E_ET_MSV3_05B05_F_0	BB985440
E_ET_MSV3_05B05_R_0	BB990936
E_ET_MSV3_05B06_F_0	BB985441

E_ET_MSV3_05B06_R_0	BB990937
E_ET_MSV3_05B07_F_0	BB985442
E_ET_MSV3_05B08_F_0	BB985443
E_ET_MSV3_05B09_F_0	BB985444
E_ET_MSV3_05B09_R_0	BB990938
E_ET_MSV3_05B10_F_0	BB985445
E_ET_MSV3_05B10_R_0	BB990939
E_ET_MSV3_05B11_F_0	BB985446
E_ET_MSV3_05B12_F_0	BB985447
E_ET_MSV3_05B12_R_0	BB990940
E_ET_MSV3_05C01_F_0	BB985448
E_ET_MSV3_05C01_R_0	BB990941
E_ET_MSV3_05C02_F_0	BB985449
E_ET_MSV3_05C02_R_0	BB990942
E_ET_MSV3_05C03_F_0	BB985450
E_ET_MSV3_05C03_R_0	BB990943
E_ET_MSV3_05C04_F_0	BB985451
E_ET_MSV3_05C05_F_0	BB985452
E_ET_MSV3_05C05_R_0	BB990944
E_ET_MSV3_05C06_F_0	BB985453
E_ET_MSV3_05C06_R_0	BB990945
E_ET_MSV3_05C07_F_0	BB985454
E_ET_MSV3_05C07_R_0	BB990946
E_ET_MSV3_05C08_F_0	BB985455
E_ET_MSV3_05C09_F_0	BB985456
E_ET_MSV3_05C10_F_0	BB985457
E_ET_MSV3_05C10_R_0	BB990947
E_ET_MSV3_05C11_F_0	BB985458
E_ET_MSV3_05C11_R_0	BB990948
E_ET_MSV3_05D01_F_0	BB985459
E_ET_MSV3_05D01_R_0	BB990949
E_ET_MSV3_05D03_F_0	BB985460
E_ET_MSV3_05D03_R_0	BB990950
E_ET_MSV3_05D04_F_0	BB985461
E_ET_MSV3_05D06_F_0	BB985462
E_ET_MSV3_05D06_R_0	BB990951
E_ET_MSV3_05D07_F_0	BB985463
E_ET_MSV3_05D08_F_0	BB985464
E_ET_MSV3_05D10_F_0	BB985465
E_ET_MSV3_05D10_R_0	BB990952
E_ET_MSV3_05D11_F_0	BB985466
E_ET_MSV3_05D11_R_0	BB990953
E_ET_MSV3_05D12_F_0	BB985467
E_ET_MSV3_05E01_F_0	BB985468
E_ET_MSV3_05E02_F_0	BB985469
E_ET_MSV3_05E03_F_0	BB985470
E_ET_MSV3_05E03_R_0	BB990954
E_ET_MSV3_05E04_F_0	BB985471
E_ET_MSV3_05E05_F_0	BB985472
E_ET_MSV3_05E06_F_0	BB985473
E_ET_MSV3_05E07_F_0	BB985474
E_ET_MSV3_05E12_F_0	BB985475
E_ET_MSV3_05F01_F_0	BB985476
E_ET_MSV3_05F01_R_0	BB990955

E_ET_MSV3_05F02_F_0	BB985477
E_ET_MSV3_05F03_F_0	BB985478
E_ET_MSV3_05F03_R_0	BB990956
E_ET_MSV3_05F04_F_0	BB985479
E_ET_MSV3_05F04_R_0	BB990957
E_ET_MSV3_05F05_F_0	BB985480
E_ET_MSV3_05F05_R_0	BB990958
E_ET_MSV3_05F06_F_0	BB985481
E_ET_MSV3_05F06_R_0	BB990959
E_ET_MSV3_05F07_F_0	BB985482
E_ET_MSV3_05F07_R_0	BB990960
E_ET_MSV3_05F08_F_0	BB985483
E_ET_MSV3_05F08_R_0	BB990961
E_ET_MSV3_05F09_F_0	BB985484
E_ET_MSV3_05F09_R_0	BB990962
E_ET_MSV3_05F10_F_0	BB985485
E_ET_MSV3_05F10_R_0	BB990963
E_ET_MSV3_05F11_F_0	BB985486
E_ET_MSV3_05F11_R_0	BB990964
E_ET_MSV3_05G01_R_0	BB990965
E_ET_MSV3_05G02_F_0	BB985487
E_ET_MSV3_05G02_R_0	BB990966
E_ET_MSV3_05G03_R_0	BB990967
E_ET_MSV3_05G04_F_0	BB985488
E_ET_MSV3_05G04_R_0	BB990968
E_ET_MSV3_05G05_F_0	BB985489
E_ET_MSV3_05G05_R_0	BB990969
E_ET_MSV3_05G06_F_0	BB985490
E_ET_MSV3_05G07_F_0	BB985491
E_ET_MSV3_05G08_F_0	BB985492
E_ET_MSV3_05G08_R_0	BB990970
E_ET_MSV3_05G09_F_0	BB985493
E_ET_MSV3_05G10_F_0	BB985494
E_ET_MSV3_05G10_R_0	BB990971
E_ET_MSV3_05G11_F_0	BB985495
E_ET_MSV3_05G11_R_0	BB990972
E_ET_MSV3_05G12_F_0	BB985496
E_ET_MSV3_05G12_R_0	BB990973
E_ET_MSV3_05H02_F_0	BB985497
E_ET_MSV3_05H03_F_0	BB985498
E_ET_MSV3_05H03_R_0	BB990974
E_ET_MSV3_05H05_F_0	BB985499
E_ET_MSV3_05H08_F_0	BB985500
E_ET_MSV3_05H09_F_0	BB985501
E_ET_MSV3_05H09_R_0	BB990975
E_ET_MSV3_05H10_R_0	BB990976
E_ET_MSV3_05H11_F_0	BB985502
E_ET_MSV3_05H12_F_0	BB985503
E_ET_MSV3_05H12_R_0	BB990977
E_ET_MSV3_06A01_F_0	BB985504
E_ET_MSV3_06A02_F_0	BB985505
E_ET_MSV3_06A02_R_0	BB990978
E_ET_MSV3_06A03_F_0	BB985506
E_ET_MSV3_06A03_R_0	BB990979

E_ET_MSV3_06A04_F_0	BB985507
E_ET_MSV3_06A06_F_0	BB985508
E_ET_MSV3_06A07_F_0	BB985509
E_ET_MSV3_06A07_R_0	BB990980
E_ET_MSV3_06A08_F_0	BB985510
E_ET_MSV3_06A08_R_0	BB990981
E_ET_MSV3_06A09_F_0	BB985511
E_ET_MSV3_06A09_R_0	BB990982
E_ET_MSV3_06A10_F_0	BB985512
E_ET_MSV3_06A10_R_0	BB990983
E_ET_MSV3_06A11_F_0	BB985513
E_ET_MSV3_06A11_R_0	BB990984
E_ET_MSV3_06A12_F_0	BB985514
E_ET_MSV3_06B01_F_0	BB985515
E_ET_MSV3_06B02_F_0	BB985516
E_ET_MSV3_06B02_R_0	BB990985
E_ET_MSV3_06B03_F_0	BB985517
E_ET_MSV3_06B03_R_0	BB990986
E_ET_MSV3_06B04_F_0	BB985518
E_ET_MSV3_06B04_R_0	BB990987
E_ET_MSV3_06B06_F_0	BB985519
E_ET_MSV3_06B06_R_0	BB990988
E_ET_MSV3_06B07_F_0	BB985520
E_ET_MSV3_06B07_R_0	BB990989
E_ET_MSV3_06B08_F_0	BB985521
E_ET_MSV3_06B08_R_0	BB990990
E_ET_MSV3_06B09_F_0	BB985522
E_ET_MSV3_06B09_R_0	BB990991
E_ET_MSV3_06B10_F_0	BB985523
E_ET_MSV3_06B10_R_0	BB990992
E_ET_MSV3_06B11_F_0	BB985524
E_ET_MSV3_06B11_R_0	BB990993
E_ET_MSV3_06B12_F_0	BB985525
E_ET_MSV3_06B12_R_0	BB990994
E_ET_MSV3_06C01_F_0	BB985526
E_ET_MSV3_06C01_R_0	BB990995
E_ET_MSV3_06C02_F_0	BB985527
E_ET_MSV3_06C02_R_0	BB990996
E_ET_MSV3_06C03_F_0	BB985528
E_ET_MSV3_06C03_R_0	BB990997
E_ET_MSV3_06C04_F_0	BB985529
E_ET_MSV3_06C04_R_0	BB990998
E_ET_MSV3_06C05_F_0	BB985530
E_ET_MSV3_06C05_R_0	BB990999
E_ET_MSV3_06C06_F_0	BB985531
E_ET_MSV3_06C06_R_0	BB991000
E_ET_MSV3_06C07_F_0	BB985532
E_ET_MSV3_06C07_R_0	BB991001
E_ET_MSV3_06C08_F_0	BB985533
E_ET_MSV3_06C08_R_0	BB991002
E_ET_MSV3_06C11_F_0	BB985534
E_ET_MSV3_06C11_R_0	BB991003
E_ET_MSV3_06C12_F_0	BB985535
E_ET_MSV3_06C12_R_0	BB991004

E_ET_MSV3_06D01_F_0	BB985536
E_ET_MSV3_06D01_R_0	BB991005
E_ET_MSV3_06D02_F_0	BB985537
E_ET_MSV3_06D03_F_0	BB985538
E_ET_MSV3_06D04_F_0	BB985539
E_ET_MSV3_06D04_R_0	BB991006
E_ET_MSV3_06D05_F_0	BB985540
E_ET_MSV3_06D06_F_0	BB985541
E_ET_MSV3_06D06_R_0	BB991007
E_ET_MSV3_06D07_F_0	BB985542
E_ET_MSV3_06D08_F_0	BB985543
E_ET_MSV3_06D08_R_0	BB991008
E_ET_MSV3_06D09_F_0	BB985544
E_ET_MSV3_06D10_F_0	BB985545
E_ET_MSV3_06D10_R_0	BB991009
E_ET_MSV3_06D12_F_0	BB985546
E_ET_MSV3_06E01_R_0	BB991010
E_ET_MSV3_06E02_F_0	BB985547
E_ET_MSV3_06E03_F_0	BB985548
E_ET_MSV3_06E04_F_0	BB985549
E_ET_MSV3_06E05_F_0	BB985550
E_ET_MSV3_06E06_F_0	BB985551
E_ET_MSV3_06E07_F_0	BB985552
E_ET_MSV3_06E08_F_0	BB985553
E_ET_MSV3_06E08_R_0	BB991011
E_ET_MSV3_06E09_F_0	BB985554
E_ET_MSV3_06E10_F_0	BB985555
E_ET_MSV3_06E11_F_0	BB985556
E_ET_MSV3_06E12_F_0	BB985557
E_ET_MSV3_06F01_F_0	BB985558
E_ET_MSV3_06F01_R_0	BB991012
E_ET_MSV3_06F02_R_0	BB991013
E_ET_MSV3_06F03_F_0	BB985559
E_ET_MSV3_06F03_R_0	BB991014
E_ET_MSV3_06F04_F_0	BB985560
E_ET_MSV3_06F04_R_0	BB991015
E_ET_MSV3_06F05_F_0	BB985561
E_ET_MSV3_06F05_R_0	BB991016
E_ET_MSV3_06F06_F_0	BB985562
E_ET_MSV3_06F06_R_0	BB991017
E_ET_MSV3_06F07_R_0	BB991018
E_ET_MSV3_06F08_F_0	BB985563
E_ET_MSV3_06F08_R_0	BB991019
E_ET_MSV3_06F09_F_0	BB985564
E_ET_MSV3_06F09_R_0	BB991020
E_ET_MSV3_06F10_F_0	BB985565
E_ET_MSV3_06F10_R_0	BB991021
E_ET_MSV3_06F11_F_0	BB985566
E_ET_MSV3_06F12_F_0	BB985567
E_ET_MSV3_06G01_F_0	BB985568
E_ET_MSV3_06G01_R_0	BB991022
E_ET_MSV3_06G02_F_0	BB985569
E_ET_MSV3_06G03_F_0	BB985570
E_ET_MSV3_06G03_R_0	BB991023

E_ET_MSV3_06G04_F_0	BB985571
E_ET_MSV3_06G04_R_0	BB991024
E_ET_MSV3_06G05_F_0	BB985572
E_ET_MSV3_06G05_R_0	BB991025
E_ET_MSV3_06G06_F_0	BB985573
E_ET_MSV3_06G06_R_0	BB991026
E_ET_MSV3_06G07_F_0	BB985574
E_ET_MSV3_06G07_R_0	BB991027
E_ET_MSV3_06G08_F_0	BB985575
E_ET_MSV3_06G08_R_0	BB991028
E_ET_MSV3_06G10_F_0	BB985576
E_ET_MSV3_06G10_R_0	BB991029
E_ET_MSV3_06G11_F_0	BB985577
E_ET_MSV3_06G11_R_0	BB991030
E_ET_MSV3_06G12_F_0	BB985578
E_ET_MSV3_06G12_R_0	BB991031
E_ET_MSV3_06H01_F_0	BB985579
E_ET_MSV3_06H01_R_0	BB991032
E_ET_MSV3_06H02_F_0	BB985580
E_ET_MSV3_06H03_F_0	BB985581
E_ET_MSV3_06H04_F_0	BB985582
E_ET_MSV3_06H05_F_0	BB985583
E_ET_MSV3_06H06_F_0	BB985584
E_ET_MSV3_06H07_F_0	BB985585
E_ET_MSV3_06H08_F_0	BB985586
E_ET_MSV3_06H10_F_0	BB985587
E_ET_MSV3_06H11_F_0	BB985588
E_ET_MSV3_06H12_F_0	BB985589
E_ET_MSV3_07A01_F_0	BB985590
E_ET_MSV3_07A01_R_0	BB991033
E_ET_MSV3_07A02_R_0	BB991034
E_ET_MSV3_07A04_F_0	BB985591
E_ET_MSV3_07A04_R_0	BB991035
E_ET_MSV3_07A05_F_0	BB985592
E_ET_MSV3_07A05_R_0	BB991036
E_ET_MSV3_07A06_F_0	BB985593
E_ET_MSV3_07A07_F_0	BB985594
E_ET_MSV3_07A08_F_0	BB985595
E_ET_MSV3_07A08_R_0	BB991037
E_ET_MSV3_07A09_F_0	BB985596
E_ET_MSV3_07A09_R_0	BB991038
E_ET_MSV3_07A11_R_0	BB991039
E_ET_MSV3_07A12_R_0	BB991040
E_ET_MSV3_07B02_R_0	BB991041
E_ET_MSV3_07B03_F_0	BB985597
E_ET_MSV3_07B03_R_0	BB991042
E_ET_MSV3_07B04_F_0	BB985598
E_ET_MSV3_07B04_R_0	BB991043
E_ET_MSV3_07B05_F_0	BB985599
E_ET_MSV3_07B06_F_0	BB985600
E_ET_MSV3_07B06_R_0	BB991044
E_ET_MSV3_07B07_F_0	BB985601
E_ET_MSV3_07B07_R_0	BB991045
E_ET_MSV3_07B08_F_0	BB985602

E_ET_MSV3_07B08_R_0	BB991046
E_ET_MSV3_07B09_F_0	BB985603
E_ET_MSV3_07B09_R_0	BB991047
E_ET_MSV3_07B10_R_0	BB991048
E_ET_MSV3_07B11_R_0	BB991049
E_ET_MSV3_07B12_F_0	BB985604
E_ET_MSV3_07B12_R_0	BB991050
E_ET_MSV3_07C01_F_0	BB985605
E_ET_MSV3_07C01_R_0	BB991051
E_ET_MSV3_07C02_F_0	BB985606
E_ET_MSV3_07C03_F_0	BB985607
E_ET_MSV3_07C03_R_0	BB991052
E_ET_MSV3_07C04_R_0	BB991053
E_ET_MSV3_07C05_F_0	BB985608
E_ET_MSV3_07C05_R_0	BB991054
E_ET_MSV3_07C06_R_0	BB991055
E_ET_MSV3_07C07_F_0	BB985609
E_ET_MSV3_07C07_R_0	BB991056
E_ET_MSV3_07C08_F_0	BB985610
E_ET_MSV3_07C08_R_0	BB991057
E_ET_MSV3_07C09_R_0	BB991058
E_ET_MSV3_07C10_R_0	BB991059
E_ET_MSV3_07C11_R_0	BB991060
E_ET_MSV3_07C12_F_0	BB985611
E_ET_MSV3_07C12_R_0	BB991061
E_ET_MSV3_07D01_F_0	BB985612
E_ET_MSV3_07D01_R_0	BB991062
E_ET_MSV3_07D02_R_0	BB991063
E_ET_MSV3_07D03_F_0	BB985613
E_ET_MSV3_07D03_R_0	BB991064
E_ET_MSV3_07D04_F_0	BB985614
E_ET_MSV3_07D04_R_0	BB991065
E_ET_MSV3_07D05_R_0	BB991066
E_ET_MSV3_07D06_R_0	BB991067
E_ET_MSV3_07D07_R_0	BB991068
E_ET_MSV3_07D08_R_0	BB991069
E_ET_MSV3_07D12_R_0	BB991070
E_ET_MSV3_07E01_F_0	BB985615
E_ET_MSV3_07E02_F_0	BB985616
E_ET_MSV3_07E03_R_0	BB991071
E_ET_MSV3_07E04_R_0	BB991072
E_ET_MSV3_07E05_F_0	BB985617
E_ET_MSV3_07E05_R_0	BB991073
E_ET_MSV3_07E06_F_0	BB985618
E_ET_MSV3_07E07_F_0	BB985619
E_ET_MSV3_07E07_R_0	BB991074
E_ET_MSV3_07E08_F_0	BB985620
E_ET_MSV3_07E08_R_0	BB991075
E_ET_MSV3_07E09_R_0	BB991076
E_ET_MSV3_07E10_R_0	BB991077
E_ET_MSV3_07E11_R_0	BB991078
E_ET_MSV3_07E12_F_0	BB985621
E_ET_MSV3_07E12_R_0	BB991079
E_ET_MSV3_07F01_F_0	BB985622

E_ET_MSV3_07F02_F_0	BB985623
E_ET_MSV3_07F02_R_0	BB991080
E_ET_MSV3_07F04_F_0	BB985624
E_ET_MSV3_07F04_R_0	BB991081
E_ET_MSV3_07F05_F_0	BB985625
E_ET_MSV3_07F05_R_0	BB991082
E_ET_MSV3_07F06_F_0	BB985626
E_ET_MSV3_07F06_R_0	BB991083
E_ET_MSV3_07F08_F_0	BB985627
E_ET_MSV3_07F08_R_0	BB991084
E_ET_MSV3_07F09_F_0	BB985628
E_ET_MSV3_07F09_R_0	BB991085
E_ET_MSV3_07F12_R_0	BB991086
E_ET_MSV3_07G01_F_0	BB985629
E_ET_MSV3_07G01_R_0	BB991087
E_ET_MSV3_07G02_F_0	BB985630
E_ET_MSV3_07G03_R_0	BB991088
E_ET_MSV3_07G04_R_0	BB991089
E_ET_MSV3_07G05_F_0	BB985631
E_ET_MSV3_07G05_R_0	BB991090
E_ET_MSV3_07G08_F_0	BB985632
E_ET_MSV3_07G08_R_0	BB991091
E_ET_MSV3_07G10_R_0	BB991092
E_ET_MSV3_07G11_R_0	BB991093
E_ET_MSV3_07G12_R_0	BB991094
E_ET_MSV3_07H01_F_0	BB985633
E_ET_MSV3_07H01_R_0	BB991095
E_ET_MSV3_07H03_F_0	BB985634
E_ET_MSV3_07H03_R_0	BB991096
E_ET_MSV3_07H05_R_0	BB991097
E_ET_MSV3_07H06_R_0	BB991098
E_ET_MSV3_07H07_R_0	BB991099
E_ET_MSV3_07H08_F_0	BB985635
E_ET_MSV3_07H08_R_0	BB991100
E_ET_MSV3_07H09_F_0	BB985636
E_ET_MSV3_07H10_R_0	BB991101
E_ET_MSV3_07H11_R_0	BB991102
E_ET_MSV3_07H12_R_0	BB991103
E_ET_MSV3_08A01_F_0	BB985637
E_ET_MSV3_08A01_R_0	BB991104
E_ET_MSV3_08A02_F_0	BB985638
E_ET_MSV3_08A02_R_0	BB991105
E_ET_MSV3_08A03_F_0	BB985639
E_ET_MSV3_08A03_R_0	BB991106
E_ET_MSV3_08A04_F_0	BB985640
E_ET_MSV3_08A05_F_0	BB985641
E_ET_MSV3_08A05_R_0	BB991107
E_ET_MSV3_08A06_R_0	BB991108
E_ET_MSV3_08A07_F_0	BB985642
E_ET_MSV3_08A07_R_0	BB991109
E_ET_MSV3_08A08_F_0	BB985643
E_ET_MSV3_08A08_R_0	BB991110
E_ET_MSV3_08A09_F_0	BB985644
E_ET_MSV3_08A11_F_0	BB985645

E_ET_MSV3_08A12_F_0	BB985646
E_ET_MSV3_08A12_R_0	BB991111
E_ET_MSV3_08B01_F_0	BB985647
E_ET_MSV3_08B01_R_0	BB991112
E_ET_MSV3_08B02_F_0	BB985648
E_ET_MSV3_08B03_R_0	BB991113
E_ET_MSV3_08B04_F_0	BB985649
E_ET_MSV3_08B04_R_0	BB991114
E_ET_MSV3_08B06_F_0	BB985650
E_ET_MSV3_08B06_R_0	BB991115
E_ET_MSV3_08B07_F_0	BB985651
E_ET_MSV3_08B09_F_0	BB985652
E_ET_MSV3_08B09_R_0	BB991116
E_ET_MSV3_08B10_F_0	BB985653
E_ET_MSV3_08B11_F_0	BB985654
E_ET_MSV3_08B11_R_0	BB991117
E_ET_MSV3_08B12_F_0	BB985655
E_ET_MSV3_08B12_R_0	BB991118
E_ET_MSV3_08C01_F_0	BB985656
E_ET_MSV3_08C01_R_0	BB991119
E_ET_MSV3_08C02_F_0	BB985657
E_ET_MSV3_08C03_F_0	BB985658
E_ET_MSV3_08C03_R_0	BB991120
E_ET_MSV3_08C04_F_0	BB985659
E_ET_MSV3_08C04_R_0	BB991121
E_ET_MSV3_08C05_F_0	BB985660
E_ET_MSV3_08C05_R_0	BB991122
E_ET_MSV3_08C07_F_0	BB985661
E_ET_MSV3_08C07_R_0	BB991123
E_ET_MSV3_08C08_F_0	BB985662
E_ET_MSV3_08C09_F_0	BB985663
E_ET_MSV3_08C09_R_0	BB991124
E_ET_MSV3_08C11_F_0	BB985664
E_ET_MSV3_08C12_F_0	BB985665
E_ET_MSV3_08C12_R_0	BB991125
E_ET_MSV3_08D01_F_0	BB985666
E_ET_MSV3_08D01_R_0	BB991126
E_ET_MSV3_08D02_F_0	BB985667
E_ET_MSV3_08D03_F_0	BB985668
E_ET_MSV3_08D03_R_0	BB991127
E_ET_MSV3_08D04_F_0	BB985669
E_ET_MSV3_08D04_R_0	BB991128
E_ET_MSV3_08D05_F_0	BB985670
E_ET_MSV3_08D05_R_0	BB991129
E_ET_MSV3_08D06_F_0	BB985671
E_ET_MSV3_08D07_F_0	BB985672
E_ET_MSV3_08D07_R_0	BB991130
E_ET_MSV3_08D09_F_0	BB985673
E_ET_MSV3_08D09_R_0	BB991131
E_ET_MSV3_08D10_F_0	BB985674
E_ET_MSV3_08D10_R_0	BB991132
E_ET_MSV3_08D11_F_0	BB985675
E_ET_MSV3_08D11_R_0	BB991133
E_ET_MSV3_08D12_F_0	BB985676

E_ET_MSV3_08D12_R_0	BB991134
E_ET_MSV3_08E01_F_0	BB985677
E_ET_MSV3_08E01_R_0	BB991135
E_ET_MSV3_08E02_F_0	BB985678
E_ET_MSV3_08E02_R_0	BB991136
E_ET_MSV3_08E03_F_0	BB985679
E_ET_MSV3_08E03_R_0	BB991137
E_ET_MSV3_08E05_F_0	BB985680
E_ET_MSV3_08E05_R_0	BB991138
E_ET_MSV3_08E07_F_0	BB985681
E_ET_MSV3_08E08_R_0	BB991139
E_ET_MSV3_08E09_F_0	BB985682
E_ET_MSV3_08E09_R_0	BB991140
E_ET_MSV3_08E10_F_0	BB985683
E_ET_MSV3_08E10_R_0	BB991141
E_ET_MSV3_08E11_F_0	BB985684
E_ET_MSV3_08E12_F_0	BB985685
E_ET_MSV3_08E12_R_0	BB991142
E_ET_MSV3_08F02_F_0	BB985686
E_ET_MSV3_08F02_R_0	BB991143
E_ET_MSV3_08F03_F_0	BB985687
E_ET_MSV3_08F04_F_0	BB985688
E_ET_MSV3_08F04_R_0	BB991144
E_ET_MSV3_08F05_F_0	BB985689
E_ET_MSV3_08F05_R_0	BB991145
E_ET_MSV3_08F07_F_0	BB985690
E_ET_MSV3_08F07_R_0	BB991146
E_ET_MSV3_08F08_F_0	BB985691
E_ET_MSV3_08F08_R_0	BB991147
E_ET_MSV3_08F09_F_0	BB985692
E_ET_MSV3_08F09_R_0	BB991148
E_ET_MSV3_08F10_F_0	BB985693
E_ET_MSV3_08F10_R_0	BB991149
E_ET_MSV3_08G01_F_0	BB985694
E_ET_MSV3_08G02_F_0	BB985695
E_ET_MSV3_08G03_F_0	BB985696
E_ET_MSV3_08G03_R_0	BB991150
E_ET_MSV3_08G04_F_0	BB985697
E_ET_MSV3_08G04_R_0	BB991151
E_ET_MSV3_08G05_F_0	BB985698
E_ET_MSV3_08G05_R_0	BB991152
E_ET_MSV3_08G06_F_0	BB985699
E_ET_MSV3_08G06_R_0	BB991153
E_ET_MSV3_08G07_F_0	BB985700
E_ET_MSV3_08G08_F_0	BB985701
E_ET_MSV3_08G08_R_0	BB991154
E_ET_MSV3_08G09_F_0	BB985702
E_ET_MSV3_08G09_R_0	BB991155
E_ET_MSV3_08G10_F_0	BB985703
E_ET_MSV3_08G10_R_0	BB991156
E_ET_MSV3_08G11_F_0	BB985704
E_ET_MSV3_08G12_F_0	BB985705
E_ET_MSV3_08G12_R_0	BB991157
E_ET_MSV3_08H01_F_0	BB985706

E_ET_MSV3_08H04_F_0	BB985707
E_ET_MSV3_08H05_F_0	BB985708
E_ET_MSV3_08H06_F_0	BB985709
E_ET_MSV3_08H06_R_0	BB991158
E_ET_MSV3_08H07_F_0	BB985710
E_ET_MSV3_08H08_F_0	BB985711
E_ET_MSV3_08H11_F_0	BB985712
E_ET_MSV3_08H12_F_0	BB985713
E_ET_MSV3_09A02_F_0	BB985714
E_ET_MSV3_09A02_R_0	BB991159
E_ET_MSV3_09A04_F_0	BB985715
E_ET_MSV3_09A04_R_0	BB991160
E_ET_MSV3_09A05_F_0	BB985716
E_ET_MSV3_09A05_R_0	BB991161
E_ET_MSV3_09A06_F_0	BB985717
E_ET_MSV3_09A06_R_0	BB991162
E_ET_MSV3_09A07_F_0	BB985718
E_ET_MSV3_09A08_F_0	BB985719
E_ET_MSV3_09A08_R_0	BB991163
E_ET_MSV3_09A09_F_0	BB985720
E_ET_MSV3_09A09_R_0	BB991164
E_ET_MSV3_09A10_F_0	BB985721
E_ET_MSV3_09A10_R_0	BB991165
E_ET_MSV3_09A11_F_0	BB985722
E_ET_MSV3_09A11_R_0	BB991166
E_ET_MSV3_09A12_F_0	BB985723
E_ET_MSV3_09A12_R_0	BB991167
E_ET_MSV3_09B01_F_0	BB985724
E_ET_MSV3_09B01_R_0	BB991168
E_ET_MSV3_09B02_F_0	BB985725
E_ET_MSV3_09B02_R_0	BB991169
E_ET_MSV3_09B03_F_0	BB985726
E_ET_MSV3_09B03_R_0	BB991170
E_ET_MSV3_09B04_F_0	BB985727
E_ET_MSV3_09B04_R_0	BB991171
E_ET_MSV3_09B05_F_0	BB985728
E_ET_MSV3_09B05_R_0	BB991172
E_ET_MSV3_09B06_F_0	BB985729
E_ET_MSV3_09B06_R_0	BB991173
E_ET_MSV3_09B07_F_0	BB985730
E_ET_MSV3_09B07_R_0	BB991174
E_ET_MSV3_09B08_F_0	BB985731
E_ET_MSV3_09B08_R_0	BB991175
E_ET_MSV3_09B09_F_0	BB985732
E_ET_MSV3_09B09_R_0	BB991176
E_ET_MSV3_09B10_F_0	BB985733
E_ET_MSV3_09B12_F_0	BB985734
E_ET_MSV3_09B12_R_0	BB991177
E_ET_MSV3_09C01_F_0	BB985735
E_ET_MSV3_09C01_R_0	BB991178
E_ET_MSV3_09C02_F_0	BB985736
E_ET_MSV3_09C02_R_0	BB991179
E_ET_MSV3_09C03_F_0	BB985737
E_ET_MSV3_09C03_R_0	BB991180

E_ET_MSV3_09C04_F_0	BB985738
E_ET_MSV3_09C05_F_0	BB985739
E_ET_MSV3_09C05_R_0	BB991181
E_ET_MSV3_09C07_F_0	BB985740
E_ET_MSV3_09C08_F_0	BB985741
E_ET_MSV3_09C08_R_0	BB991182
E_ET_MSV3_09C09_F_0	BB985742
E_ET_MSV3_09C09_R_0	BB991183
E_ET_MSV3_09C10_F_0	BB985743
E_ET_MSV3_09C11_F_0	BB985744
E_ET_MSV3_09C12_F_0	BB985745
E_ET_MSV3_09D01_F_0	BB985746
E_ET_MSV3_09D01_R_0	BB991184
E_ET_MSV3_09D02_R_0	BB991185
E_ET_MSV3_09D03_F_0	BB985747
E_ET_MSV3_09D03_R_0	BB991186
E_ET_MSV3_09D04_F_0	BB985748
E_ET_MSV3_09D04_R_0	BB991187
E_ET_MSV3_09D05_F_0	BB985749
E_ET_MSV3_09D05_R_0	BB991188
E_ET_MSV3_09D07_F_0	BB985750
E_ET_MSV3_09D08_F_0	BB985751
E_ET_MSV3_09D08_R_0	BB991189
E_ET_MSV3_09D10_F_0	BB985752
E_ET_MSV3_09D10_R_0	BB991190
E_ET_MSV3_09D11_F_0	BB985753
E_ET_MSV3_09D11_R_0	BB991191
E_ET_MSV3_09E01_F_0	BB985754
E_ET_MSV3_09E01_R_0	BB991192
E_ET_MSV3_09E02_F_0	BB985755
E_ET_MSV3_09E03_F_0	BB985756
E_ET_MSV3_09E03_R_0	BB991193
E_ET_MSV3_09E04_F_0	BB985757
E_ET_MSV3_09E04_R_0	BB991194
E_ET_MSV3_09E05_R_0	BB991195
E_ET_MSV3_09E06_F_0	BB985758
E_ET_MSV3_09E06_R_0	BB991196
E_ET_MSV3_09E07_F_0	BB985759
E_ET_MSV3_09E07_R_0	BB991197
E_ET_MSV3_09E08_F_0	BB985760
E_ET_MSV3_09E08_R_0	BB991198
E_ET_MSV3_09E09_F_0	BB985761
E_ET_MSV3_09E09_R_0	BB991199
E_ET_MSV3_09E10_F_0	BB985762
E_ET_MSV3_09E10_R_0	BB991200
E_ET_MSV3_09E11_F_0	BB985763
E_ET_MSV3_09E11_R_0	BB991201
E_ET_MSV3_09F01_F_0	BB985764
E_ET_MSV3_09F01_R_0	BB991202
E_ET_MSV3_09F02_F_0	BB985765
E_ET_MSV3_09F02_R_0	BB991203
E_ET_MSV3_09F03_F_0	BB985766
E_ET_MSV3_09F03_R_0	BB991204
E_ET_MSV3_09F04_F_0	BB985767

E_ET_MSV3_09F05_F_0	BB985768
E_ET_MSV3_09F05_R_0	BB991205
E_ET_MSV3_09F06_F_0	BB985769
E_ET_MSV3_09F07_F_0	BB985770
E_ET_MSV3_09F08_F_0	BB985771
E_ET_MSV3_09F09_F_0	BB985772
E_ET_MSV3_09F09_R_0	BB991206
E_ET_MSV3_09F10_F_0	BB985773
E_ET_MSV3_09F10_R_0	BB991207
E_ET_MSV3_09F11_R_0	BB991208
E_ET_MSV3_09F12_F_0	BB985774
E_ET_MSV3_09G03_F_0	BB985775
E_ET_MSV3_09G03_R_0	BB991209
E_ET_MSV3_09G04_F_0	BB985776
E_ET_MSV3_09G04_R_0	BB991210
E_ET_MSV3_09G05_F_0	BB985777
E_ET_MSV3_09G06_F_0	BB985778
E_ET_MSV3_09G06_R_0	BB991211
E_ET_MSV3_09G07_F_0	BB985779
E_ET_MSV3_09G07_R_0	BB991212
E_ET_MSV3_09G08_F_0	BB985780
E_ET_MSV3_09G09_F_0	BB985781
E_ET_MSV3_09G10_F_0	BB985782
E_ET_MSV3_09G10_R_0	BB991213
E_ET_MSV3_09G11_F_0	BB985783
E_ET_MSV3_09G11_R_0	BB991214
E_ET_MSV3_09G12_F_0	BB985784
E_ET_MSV3_09H01_F_0	BB985785
E_ET_MSV3_09H01_R_0	BB991215
E_ET_MSV3_09H02_F_0	BB985786
E_ET_MSV3_09H02_R_0	BB991216
E_ET_MSV3_09H04_F_0	BB985787
E_ET_MSV3_09H05_F_0	BB985788
E_ET_MSV3_09H06_F_0	BB985789
E_ET_MSV3_09H07_F_0	BB985790
E_ET_MSV3_09H08_F_0	BB985791
E_ET_MSV3_09H08_R_0	BB991217
E_ET_MSV3_09H10_F_0	BB985792
E_ET_MSV3_09H10_R_0	BB991218
E_ET_MSV3_09H11_F_0	BB985793
E_ET_MSV3_09H11_R_0	BB991219
E_ET_MSV3_09H12_F_0	BB985794
E_ET_MSV3_10A01_R_0	BB991220
E_ET_MSV3_10A02_F_0	BB985795
E_ET_MSV3_10A03_F_0	BB985796
E_ET_MSV3_10A04_F_0	BB985797
E_ET_MSV3_10A04_R_0	BB991221
E_ET_MSV3_10A05_F_0	BB985798
E_ET_MSV3_10A05_R_0	BB991222
E_ET_MSV3_10A06_R_0	BB991223
E_ET_MSV3_10A07_F_0	BB985799
E_ET_MSV3_10A07_R_0	BB991224
E_ET_MSV3_10A08_R_0	BB991225
E_ET_MSV3_10A10_F_0	BB985800

E_ET_MSV3_10A10_R_0	BB991226
E_ET_MSV3_10A11_F_0	BB985801
E_ET_MSV3_10A11_R_0	BB991227
E_ET_MSV3_10A12_R_0	BB991228
E_ET_MSV3_10B01_F_0	BB985802
E_ET_MSV3_10B01_R_0	BB991229
E_ET_MSV3_10B02_F_0	BB985803
E_ET_MSV3_10B02_R_0	BB991230
E_ET_MSV3_10B03_F_0	BB985804
E_ET_MSV3_10B03_R_0	BB991231
E_ET_MSV3_10B04_F_0	BB985805
E_ET_MSV3_10B04_R_0	BB991232
E_ET_MSV3_10B05_F_0	BB985806
E_ET_MSV3_10B06_F_0	BB985807
E_ET_MSV3_10B06_R_0	BB991233
E_ET_MSV3_10B07_F_0	BB985808
E_ET_MSV3_10B07_R_0	BB991234
E_ET_MSV3_10B08_F_0	BB985809
E_ET_MSV3_10B08_R_0	BB991235
E_ET_MSV3_10B09_F_0	BB985810
E_ET_MSV3_10B09_R_0	BB991236
E_ET_MSV3_10B11_F_0	BB985811
E_ET_MSV3_10B11_R_0	BB991237
E_ET_MSV3_10C02_F_0	BB985812
E_ET_MSV3_10C02_R_0	BB991238
E_ET_MSV3_10C03_F_0	BB985813
E_ET_MSV3_10C04_F_0	BB985814
E_ET_MSV3_10C04_R_0	BB991239
E_ET_MSV3_10C05_F_0	BB985815
E_ET_MSV3_10C06_F_0	BB985816
E_ET_MSV3_10C06_R_0	BB991240
E_ET_MSV3_10C07_F_0	BB985817
E_ET_MSV3_10C08_F_0	BB985818
E_ET_MSV3_10C09_F_0	BB985819
E_ET_MSV3_10C09_R_0	BB991241
E_ET_MSV3_10C10_F_0	BB985820
E_ET_MSV3_10C10_R_0	BB991242
E_ET_MSV3_10C11_F_0	BB985821
E_ET_MSV3_10C11_R_0	BB991243
E_ET_MSV3_10C12_F_0	BB985822
E_ET_MSV3_10D01_F_0	BB985823
E_ET_MSV3_10D02_F_0	BB985824
E_ET_MSV3_10D02_R_0	BB991244
E_ET_MSV3_10D03_F_0	BB985825
E_ET_MSV3_10D04_F_0	BB985826
E_ET_MSV3_10D04_R_0	BB991245
E_ET_MSV3_10D05_F_0	BB985827
E_ET_MSV3_10D05_R_0	BB991246
E_ET_MSV3_10D06_R_0	BB991247
E_ET_MSV3_10D07_F_0	BB985828
E_ET_MSV3_10D07_R_0	BB991248
E_ET_MSV3_10D08_F_0	BB985829
E_ET_MSV3_10D08_R_0	BB991249
E_ET_MSV3_10D09_F_0	BB985830

E_ET_MSV3_10D09_R_0	BB991250
E_ET_MSV3_10D10_R_0	BB991251
E_ET_MSV3_10D11_F_0	BB985831
E_ET_MSV3_10D11_R_0	BB991252
E_ET_MSV3_10D12_F_0	BB985832
E_ET_MSV3_10D12_R_0	BB991253
E_ET_MSV3_10E01_R_0	BB991254
E_ET_MSV3_10E02_F_0	BB985833
E_ET_MSV3_10E02_R_0	BB991255
E_ET_MSV3_10E03_F_0	BB985834
E_ET_MSV3_10E03_R_0	BB991256
E_ET_MSV3_10E04_F_0	BB985835
E_ET_MSV3_10E04_R_0	BB991257
E_ET_MSV3_10E05_F_0	BB985836
E_ET_MSV3_10E05_R_0	BB991258
E_ET_MSV3_10E06_F_0	BB985837
E_ET_MSV3_10E07_F_0	BB985838
E_ET_MSV3_10E07_R_0	BB991259
E_ET_MSV3_10E08_F_0	BB985839
E_ET_MSV3_10E08_R_0	BB991260
E_ET_MSV3_10E09_F_0	BB985840
E_ET_MSV3_10E09_R_0	BB991261
E_ET_MSV3_10E10_F_0	BB985841
E_ET_MSV3_10E10_R_0	BB991262
E_ET_MSV3_10E11_F_0	BB985842
E_ET_MSV3_10E11_R_0	BB991263
E_ET_MSV3_10E12_F_0	BB985843
E_ET_MSV3_10E12_R_0	BB991264
E_ET_MSV3_10F01_F_0	BB985844
E_ET_MSV3_10F01_R_0	BB991265
E_ET_MSV3_10F02_F_0	BB985845
E_ET_MSV3_10F03_F_0	BB985846
E_ET_MSV3_10F03_R_0	BB991266
E_ET_MSV3_10F04_F_0	BB985847
E_ET_MSV3_10F04_R_0	BB991267
E_ET_MSV3_10F05_F_0	BB985848
E_ET_MSV3_10F05_R_0	BB991268
E_ET_MSV3_10F06_F_0	BB985849
E_ET_MSV3_10F06_R_0	BB991269
E_ET_MSV3_10F07_F_0	BB985850
E_ET_MSV3_10F07_R_0	BB991270
E_ET_MSV3_10F08_F_0	BB985851
E_ET_MSV3_10F08_R_0	BB991271
E_ET_MSV3_10F09_R_0	BB991272
E_ET_MSV3_10F10_F_0	BB985852
E_ET_MSV3_10F10_R_0	BB991273
E_ET_MSV3_10F11_F_0	BB985853
E_ET_MSV3_10F11_R_0	BB991274
E_ET_MSV3_10F12_R_0	BB991275
E_ET_MSV3_10G01_F_0	BB985854
E_ET_MSV3_10G02_R_0	BB991276
E_ET_MSV3_10G03_F_0	BB985855
E_ET_MSV3_10G03_R_0	BB991277
E_ET_MSV3_10G04_F_0	BB985856

E_ET_MSV3_10G04_R_0	BB991278
E_ET_MSV3_10G05_F_0	BB985857
E_ET_MSV3_10G05_R_0	BB991279
E_ET_MSV3_10G06_F_0	BB985858
E_ET_MSV3_10G06_R_0	BB991280
E_ET_MSV3_10G07_F_0	BB985859
E_ET_MSV3_10G07_R_0	BB991281
E_ET_MSV3_10G08_F_0	BB985860
E_ET_MSV3_10G09_F_0	BB985861
E_ET_MSV3_10G10_F_0	BB985862
E_ET_MSV3_10G11_F_0	BB985863
E_ET_MSV3_10G11_R_0	BB991282
E_ET_MSV3_10H01_F_0	BB985864
E_ET_MSV3_10H01_R_0	BB991283
E_ET_MSV3_10H02_F_0	BB985865
E_ET_MSV3_10H03_F_0	BB985866
E_ET_MSV3_10H03_R_0	BB991284
E_ET_MSV3_10H04_F_0	BB985867
E_ET_MSV3_10H05_F_0	BB985868
E_ET_MSV3_10H05_R_0	BB991285
E_ET_MSV3_10H06_F_0	BB985869
E_ET_MSV3_10H07_F_0	BB985870
E_ET_MSV3_10H07_R_0	BB991286
E_ET_MSV3_10H08_F_0	BB985871
E_ET_MSV3_10H09_F_0	BB985872
E_ET_MSV3_10H09_R_0	BB991287
E_ET_MSV3_10H10_F_0	BB985873
E_ET_MSV3_10H10_R_0	BB991288
E_ET_MSV3_10H11_F_0	BB985874
E_ET_MSV3_10H12_F_0	BB985875
E_ET_MSV3_10H12_R_0	BB991289
E_ET_MSV3_11A01_F_0	BB985876
E_ET_MSV3_11A03_F_0	BB985877
E_ET_MSV3_11A04_F_0	BB985878
E_ET_MSV3_11A06_F_0	BB985879
E_ET_MSV3_11A08_F_0	BB985880
E_ET_MSV3_11A09_F_0	BB985881
E_ET_MSV3_11A10_F_0	BB985882
E_ET_MSV3_11A11_F_0	BB985883
E_ET_MSV3_11A12_F_0	BB985884
E_ET_MSV3_11B01_F_0	BB985885
E_ET_MSV3_11B02_F_0	BB985886
E_ET_MSV3_11B03_F_0	BB985887
E_ET_MSV3_11B04_F_0	BB985888
E_ET_MSV3_11B05_F_0	BB985889
E_ET_MSV3_11B06_F_0	BB985890
E_ET_MSV3_11B07_F_0	BB985891
E_ET_MSV3_11B08_F_0	BB985892
E_ET_MSV3_11B10_F_0	BB985893
E_ET_MSV3_11B11_F_0	BB985894
E_ET_MSV3_11B12_F_0	BB985895
E_ET_MSV3_11C01_F_0	BB985896
E_ET_MSV3_11C02_F_0	BB985897
E_ET_MSV3_11C03_F_0	BB985898

E_ET_MSV3_11C04_F_0	BB985899
E_ET_MSV3_11C05_F_0	BB985900
E_ET_MSV3_11C06_F_0	BB985901
E_ET_MSV3_11C06_R_0	BB991290
E_ET_MSV3_11C07_F_0	BB985902
E_ET_MSV3_11C08_R_0	BB991291
E_ET_MSV3_11C09_F_0	BB985903
E_ET_MSV3_11C10_R_0	BB991292
E_ET_MSV3_11C11_F_0	BB985904
E_ET_MSV3_11C11_R_0	BB991293
E_ET_MSV3_11C12_F_0	BB985905
E_ET_MSV3_11D01_F_0	BB985906
E_ET_MSV3_11D02_F_0	BB985907
E_ET_MSV3_11D03_F_0	BB985908
E_ET_MSV3_11D05_F_0	BB985909
E_ET_MSV3_11D06_F_0	BB985910
E_ET_MSV3_11D07_F_0	BB985911
E_ET_MSV3_11D08_F_0	BB985912
E_ET_MSV3_11D09_F_0	BB985913
E_ET_MSV3_11D10_F_0	BB985914
E_ET_MSV3_11D11_F_0	BB985915
E_ET_MSV3_11D12_F_0	BB985916
E_ET_MSV3_11E01_F_0	BB985917
E_ET_MSV3_11E02_F_0	BB985918
E_ET_MSV3_11E03_F_0	BB985919
E_ET_MSV3_11E04_F_0	BB985920
E_ET_MSV3_11E05_F_0	BB985921
E_ET_MSV3_11E06_F_0	BB985922
E_ET_MSV3_11E06_R_0	BB991294
E_ET_MSV3_11E07_F_0	BB985923
E_ET_MSV3_11E08_F_0	BB985924
E_ET_MSV3_11E09_F_0	BB985925
E_ET_MSV3_11E09_R_0	BB991295
E_ET_MSV3_11E10_F_0	BB985926
E_ET_MSV3_11E11_F_0	BB985927
E_ET_MSV3_11E11_R_0	BB991296
E_ET_MSV3_11E12_F_0	BB985928
E_ET_MSV3_11E12_R_0	BB991297
E_ET_MSV3_11F01_F_0	BB985929
E_ET_MSV3_11F01_R_0	BB991298
E_ET_MSV3_11F02_F_0	BB985930
E_ET_MSV3_11F03_F_0	BB985931
E_ET_MSV3_11F04_F_0	BB985932
E_ET_MSV3_11F05_F_0	BB985933
E_ET_MSV3_11F07_F_0	BB985934
E_ET_MSV3_11F08_F_0	BB985935
E_ET_MSV3_11F09_R_0	BB991299
E_ET_MSV3_11F10_F_0	BB985936
E_ET_MSV3_11F11_F_0	BB985937
E_ET_MSV3_11F12_F_0	BB985938
E_ET_MSV3_11G01_F_0	BB985939
E_ET_MSV3_11G02_F_0	BB985940
E_ET_MSV3_11G03_F_0	BB985941
E_ET_MSV3_11G05_F_0	BB985942

E_ET_MSV3_11G06_F_0	BB985943
E_ET_MSV3_11G07_F_0	BB985944
E_ET_MSV3_11G10_F_0	BB985945
E_ET_MSV3_11G11_F_0	BB985946
E_ET_MSV3_11G12_F_0	BB985947
E_ET_MSV3_11H01_F_0	BB985948
E_ET_MSV3_11H02_F_0	BB985949
E_ET_MSV3_11H03_F_0	BB985950
E_ET_MSV3_11H04_F_0	BB985951
E_ET_MSV3_11H05_F_0	BB985952
E_ET_MSV3_11H06_F_0	BB985953
E_ET_MSV3_11H07_F_0	BB985954
E_ET_MSV3_11H08_F_0	BB985955
E_ET_MSV3_11H09_F_0	BB985956
E_ET_MSV3_11H10_F_0	BB985957
E_ET_MSV3_11H11_F_0	BB985958
E_ET_MSV3_11H12_F_0	BB985959
E_ET_MSV3_12A01_F_0	BB985960
E_ET_MSV3_12A02_F_0	BB985961
E_ET_MSV3_12A03_F_0	BB985962
E_ET_MSV3_12A03_R_0	BB991300
E_ET_MSV3_12A04_F_0	BB985963
E_ET_MSV3_12A06_F_0	BB985964
E_ET_MSV3_12A06_R_0	BB991301
E_ET_MSV3_12A07_F_0	BB985965
E_ET_MSV3_12A07_R_0	BB991302
E_ET_MSV3_12A08_F_0	BB985966
E_ET_MSV3_12A08_R_0	BB991303
E_ET_MSV3_12A09_F_0	BB985967
E_ET_MSV3_12A10_F_0	BB985968
E_ET_MSV3_12A10_R_0	BB991304
E_ET_MSV3_12A12_F_0	BB985969
E_ET_MSV3_12A12_R_0	BB991305
E_ET_MSV3_12B01_F_0	BB985970
E_ET_MSV3_12B01_R_0	BB991306
E_ET_MSV3_12B02_F_0	BB985971
E_ET_MSV3_12B02_R_0	BB991307
E_ET_MSV3_12B03_F_0	BB985972
E_ET_MSV3_12B03_R_0	BB991308
E_ET_MSV3_12B04_F_0	BB985973
E_ET_MSV3_12B04_R_0	BB991309
E_ET_MSV3_12B05_R_0	BB991310
E_ET_MSV3_12B06_F_0	BB985974
E_ET_MSV3_12B07_F_0	BB985975
E_ET_MSV3_12B08_F_0	BB985976
E_ET_MSV3_12B08_R_0	BB991311
E_ET_MSV3_12B09_F_0	BB985977
E_ET_MSV3_12B09_R_0	BB991312
E_ET_MSV3_12B10_F_0	BB985978
E_ET_MSV3_12B10_R_0	BB991313
E_ET_MSV3_12B11_F_0	BB985979
E_ET_MSV3_12B11_R_0	BB991314
E_ET_MSV3_12B12_F_0	BB985980
E_ET_MSV3_12B12_R_0	BB991315

E_ET_MSV3_12C01_F_0	BB985981
E_ET_MSV3_12C01_R_0	BB991316
E_ET_MSV3_12C02_F_0	BB985982
E_ET_MSV3_12C02_R_0	BB991317
E_ET_MSV3_12C03_F_0	BB985983
E_ET_MSV3_12C03_R_0	BB991318
E_ET_MSV3_12C04_F_0	BB985984
E_ET_MSV3_12C04_R_0	BB991319
E_ET_MSV3_12C05_F_0	BB985985
E_ET_MSV3_12C06_F_0	BB985986
E_ET_MSV3_12C06_R_0	BB991320
E_ET_MSV3_12C07_F_0	BB985987
E_ET_MSV3_12C08_F_0	BB985988
E_ET_MSV3_12C08_R_0	BB991321
E_ET_MSV3_12C09_F_0	BB985989
E_ET_MSV3_12C09_R_0	BB991322
E_ET_MSV3_12C10_F_0	BB985990
E_ET_MSV3_12C10_R_0	BB991323
E_ET_MSV3_12C11_F_0	BB985991
E_ET_MSV3_12C11_R_0	BB991324
E_ET_MSV3_12C12_F_0	BB985992
E_ET_MSV3_12C12_R_0	BB991325
E_ET_MSV3_12D01_F_0	BB985993
E_ET_MSV3_12D02_F_0	BB985994
E_ET_MSV3_12D03_F_0	BB985995
E_ET_MSV3_12D04_F_0	BB985996
E_ET_MSV3_12D04_R_0	BB991326
E_ET_MSV3_12D05_F_0	BB985997
E_ET_MSV3_12D05_R_0	BB991327
E_ET_MSV3_12D06_F_0	BB985998
E_ET_MSV3_12D06_R_0	BB991328
E_ET_MSV3_12D07_F_0	BB985999
E_ET_MSV3_12D07_R_0	BB991329
E_ET_MSV3_12D08_F_0	BB986000
E_ET_MSV3_12D09_F_0	BB986001
E_ET_MSV3_12D09_R_0	BB991330
E_ET_MSV3_12D10_F_0	BB986002
E_ET_MSV3_12D10_R_0	BB991331
E_ET_MSV3_12D11_F_0	BB986003
E_ET_MSV3_12D11_R_0	BB991332
E_ET_MSV3_12D12_F_0	BB986004
E_ET_MSV3_12D12_R_0	BB991333
E_ET_MSV3_12E01_F_0	BB986005
E_ET_MSV3_12E02_F_0	BB986006
E_ET_MSV3_12E03_F_0	BB986007
E_ET_MSV3_12E04_F_0	BB986008
E_ET_MSV3_12E05_F_0	BB986009
E_ET_MSV3_12E06_F_0	BB986010
E_ET_MSV3_12E06_R_0	BB991334
E_ET_MSV3_12E07_F_0	BB986011
E_ET_MSV3_12E07_R_0	BB991335
E_ET_MSV3_12E08_F_0	BB986012
E_ET_MSV3_12E08_R_0	BB991336
E_ET_MSV3_12E09_F_0	BB986013

E_ET_MSV3_12E09_R_0	BB991337
E_ET_MSV3_12E10_F_0	BB986014
E_ET_MSV3_12E10_R_0	BB991338
E_ET_MSV3_12E11_F_0	BB986015
E_ET_MSV3_12E11_R_0	BB991339
E_ET_MSV3_12E12_F_0	BB986016
E_ET_MSV3_12E12_R_0	BB991340
E_ET_MSV3_12F02_F_0	BB986017
E_ET_MSV3_12F02_R_0	BB991341
E_ET_MSV3_12F03_F_0	BB986018
E_ET_MSV3_12F03_R_0	BB991342
E_ET_MSV3_12F04_F_0	BB986019
E_ET_MSV3_12F05_F_0	BB986020
E_ET_MSV3_12F05_R_0	BB991343
E_ET_MSV3_12F06_F_0	BB986021
E_ET_MSV3_12F06_R_0	BB991344
E_ET_MSV3_12F07_F_0	BB986022
E_ET_MSV3_12F07_R_0	BB991345
E_ET_MSV3_12F08_F_0	BB986023
E_ET_MSV3_12F09_F_0	BB986024
E_ET_MSV3_12F09_R_0	BB991346
E_ET_MSV3_12F10_F_0	BB986025
E_ET_MSV3_12F10_R_0	BB991347
E_ET_MSV3_12G01_F_0	BB986026
E_ET_MSV3_12G01_R_0	BB991348
E_ET_MSV3_12G02_F_0	BB986027
E_ET_MSV3_12G02_R_0	BB991349
E_ET_MSV3_12G03_F_0	BB986028
E_ET_MSV3_12G03_R_0	BB991350
E_ET_MSV3_12G04_F_0	BB986029
E_ET_MSV3_12G04_R_0	BB991351
E_ET_MSV3_12G05_F_0	BB986030
E_ET_MSV3_12G05_R_0	BB991352
E_ET_MSV3_12G06_F_0	BB986031
E_ET_MSV3_12G06_R_0	BB991353
E_ET_MSV3_12G08_F_0	BB986032
E_ET_MSV3_12G08_R_0	BB991354
E_ET_MSV3_12G09_F_0	BB986033
E_ET_MSV3_12G09_R_0	BB991355
E_ET_MSV3_12G10_F_0	BB986034
E_ET_MSV3_12G10_R_0	BB991356
E_ET_MSV3_12G11_F_0	BB986035
E_ET_MSV3_12G12_F_0	BB986036
E_ET_MSV3_12H01_F_0	BB986037
E_ET_MSV3_12H01_R_0	BB991357
E_ET_MSV3_12H02_F_0	BB986038
E_ET_MSV3_12H03_F_0	BB986039
E_ET_MSV3_12H03_R_0	BB991358
E_ET_MSV3_12H04_F_0	BB986040
E_ET_MSV3_12H04_R_0	BB991359
E_ET_MSV3_12H05_F_0	BB986041
E_ET_MSV3_12H05_R_0	BB991360
E_ET_MSV3_12H06_F_0	BB986042
E_ET_MSV3_12H06_R_0	BB991361

E_ET_MSV3_12H07_F_0	BB986043
E_ET_MSV3_12H07_R_0	BB991362
E_ET_MSV3_12H08_F_0	BB986044
E_ET_MSV3_12H08_R_0	BB991363
E_ET_MSV3_12H09_F_0	BB986045
E_ET_MSV3_12H09_R_0	BB991364
E_ET_MSV3_12H10_F_0	BB986046
E_ET_MSV3_12H11_F_0	BB986047
E_ET_MSV3_12H12_F_0	BB986048
E_ET_MSV3_13A01_F_0	BB986049
E_ET_MSV3_13A01_R_0	BB991365
E_ET_MSV3_13A02_F_0	BB986050
E_ET_MSV3_13A04_F_0	BB986051
E_ET_MSV3_13A05_F_0	BB986052
E_ET_MSV3_13A05_R_0	BB991366
E_ET_MSV3_13A06_F_0	BB986053
E_ET_MSV3_13A06_R_0	BB991367
E_ET_MSV3_13A07_F_0	BB986054
E_ET_MSV3_13A07_R_0	BB991368
E_ET_MSV3_13A08_F_0	BB986055
E_ET_MSV3_13A09_R_0	BB991369
E_ET_MSV3_13A10_F_0	BB986056
E_ET_MSV3_13A10_R_0	BB991370
E_ET_MSV3_13A11_F_0	BB986057
E_ET_MSV3_13A12_R_0	BB991371
E_ET_MSV3_13B01_F_0	BB986058
E_ET_MSV3_13B01_R_0	BB991372
E_ET_MSV3_13B02_F_0	BB986059
E_ET_MSV3_13B02_R_0	BB991373
E_ET_MSV3_13B04_F_0	BB986060
E_ET_MSV3_13B05_F_0	BB986061
E_ET_MSV3_13B05_R_0	BB991374
E_ET_MSV3_13B06_F_0	BB986062
E_ET_MSV3_13B07_F_0	BB986063
E_ET_MSV3_13B07_R_0	BB991375
E_ET_MSV3_13B08_F_0	BB986064
E_ET_MSV3_13B08_R_0	BB991376
E_ET_MSV3_13B09_F_0	BB986065
E_ET_MSV3_13B09_R_0	BB991377
E_ET_MSV3_13B10_F_0	BB986066
E_ET_MSV3_13B10_R_0	BB991378
E_ET_MSV3_13B11_F_0	BB986067
E_ET_MSV3_13B12_F_0	BB986068
E_ET_MSV3_13C01_R_0	BB991379
E_ET_MSV3_13C02_F_0	BB986069
E_ET_MSV3_13C03_F_0	BB986070
E_ET_MSV3_13C03_R_0	BB991380
E_ET_MSV3_13C04_F_0	BB986071
E_ET_MSV3_13C04_R_0	BB991381
E_ET_MSV3_13C05_F_0	BB986072
E_ET_MSV3_13C05_R_0	BB991382
E_ET_MSV3_13C06_F_0	BB986073
E_ET_MSV3_13C06_R_0	BB991383
E_ET_MSV3_13C07_F_0	BB986074

E_ET_MSV3_13C07_R_0	BB991384
E_ET_MSV3_13C08_F_0	BB986075
E_ET_MSV3_13C08_R_0	BB991385
E_ET_MSV3_13C09_F_0	BB986076
E_ET_MSV3_13C09_R_0	BB991386
E_ET_MSV3_13C10_F_0	BB986077
E_ET_MSV3_13C11_F_0	BB986078
E_ET_MSV3_13C11_R_0	BB991387
E_ET_MSV3_13C12_F_0	BB986079
E_ET_MSV3_13D01_F_0	BB986080
E_ET_MSV3_13D01_R_0	BB991388
E_ET_MSV3_13D02_F_0	BB986081
E_ET_MSV3_13D02_R_0	BB991389
E_ET_MSV3_13D03_F_0	BB986082
E_ET_MSV3_13D03_R_0	BB991390
E_ET_MSV3_13D04_F_0	BB986083
E_ET_MSV3_13D05_F_0	BB986084
E_ET_MSV3_13D05_R_0	BB991391
E_ET_MSV3_13D07_F_0	BB986085
E_ET_MSV3_13D07_R_0	BB991392
E_ET_MSV3_13D08_F_0	BB986086
E_ET_MSV3_13D09_F_0	BB986087
E_ET_MSV3_13D09_R_0	BB991393
E_ET_MSV3_13D10_F_0	BB986088
E_ET_MSV3_13D11_F_0	BB986089
E_ET_MSV3_13D11_R_0	BB991394
E_ET_MSV3_13D12_F_0	BB986090
E_ET_MSV3_13E01_F_0	BB986091
E_ET_MSV3_13E01_R_0	BB991395
E_ET_MSV3_13E02_F_0	BB986092
E_ET_MSV3_13E02_R_0	BB991396
E_ET_MSV3_13E03_F_0	BB986093
E_ET_MSV3_13E04_F_0	BB986094
E_ET_MSV3_13E05_F_0	BB986095
E_ET_MSV3_13E06_F_0	BB986096
E_ET_MSV3_13E06_R_0	BB991397
E_ET_MSV3_13E07_F_0	BB986097
E_ET_MSV3_13E08_F_0	BB986098
E_ET_MSV3_13E09_F_0	BB986099
E_ET_MSV3_13E09_R_0	BB991398
E_ET_MSV3_13E10_F_0	BB986100
E_ET_MSV3_13E11_F_0	BB986101
E_ET_MSV3_13E11_R_0	BB991399
E_ET_MSV3_13E12_F_0	BB986102
E_ET_MSV3_13E12_R_0	BB991400
E_ET_MSV3_13F01_F_0	BB986103
E_ET_MSV3_13F01_R_0	BB991401
E_ET_MSV3_13F03_F_0	BB986104
E_ET_MSV3_13F04_F_0	BB986105
E_ET_MSV3_13F05_F_0	BB986106
E_ET_MSV3_13F06_F_0	BB986107
E_ET_MSV3_13F06_R_0	BB991402
E_ET_MSV3_13F07_F_0	BB986108
E_ET_MSV3_13F08_F_0	BB986109

E_ET_MSV3_13F09_F_0	BB986110
E_ET_MSV3_13F09_R_0	BB991403
E_ET_MSV3_13F10_R_0	BB991404
E_ET_MSV3_13F11_F_0	BB986111
E_ET_MSV3_13F11_R_0	BB991405
E_ET_MSV3_13F12_F_0	BB986112
E_ET_MSV3_13G01_F_0	BB986113
E_ET_MSV3_13G01_R_0	BB991406
E_ET_MSV3_13G02_F_0	BB986114
E_ET_MSV3_13G02_R_0	BB991407
E_ET_MSV3_13G03_F_0	BB986115
E_ET_MSV3_13G03_R_0	BB991408
E_ET_MSV3_13G04_F_0	BB986116
E_ET_MSV3_13G05_F_0	BB986117
E_ET_MSV3_13G06_F_0	BB986118
E_ET_MSV3_13G07_F_0	BB986119
E_ET_MSV3_13G08_F_0	BB986120
E_ET_MSV3_13G08_R_0	BB991409
E_ET_MSV3_13G09_F_0	BB986121
E_ET_MSV3_13G09_R_0	BB991410
E_ET_MSV3_13G10_F_0	BB986122
E_ET_MSV3_13G10_R_0	BB991411
E_ET_MSV3_13G11_F_0	BB986123
E_ET_MSV3_13G11_R_0	BB991412
E_ET_MSV3_13H01_F_0	BB986124
E_ET_MSV3_13H01_R_0	BB991413
E_ET_MSV3_13H02_F_0	BB986125
E_ET_MSV3_13H02_R_0	BB991414
E_ET_MSV3_13H03_F_0	BB986126
E_ET_MSV3_13H03_R_0	BB991415
E_ET_MSV3_13H04_F_0	BB986127
E_ET_MSV3_13H05_F_0	BB986128
E_ET_MSV3_13H06_F_0	BB986129
E_ET_MSV3_13H07_F_0	BB986130
E_ET_MSV3_13H08_F_0	BB986131
E_ET_MSV3_13H08_R_0	BB991416
E_ET_MSV3_13H09_F_0	BB986132
E_ET_MSV3_13H10_F_0	BB986133
E_ET_MSV3_13H10_R_0	BB991417
E_ET_MSV3_13H11_F_0	BB986134
E_ET_MSV3_13H12_F_0	BB986135
E_ET_MSV3_13H12_R_0	BB991418
E_ET_MSV3_14A01_F_0	BB986136
E_ET_MSV3_14A02_F_0	BB986137
E_ET_MSV3_14A02_R_0	BB991419
E_ET_MSV3_14A03_F_0	BB986138
E_ET_MSV3_14A03_R_0	BB991420
E_ET_MSV3_14A04_F_0	BB986139
E_ET_MSV3_14A04_R_0	BB991421
E_ET_MSV3_14A05_F_0	BB986140
E_ET_MSV3_14A05_R_0	BB991422
E_ET_MSV3_14A06_F_0	BB986141
E_ET_MSV3_14A06_R_0	BB991423
E_ET_MSV3_14A07_F_0	BB986142

E_ET_MSV3_14A07_R_0	BB991424
E_ET_MSV3_14A08_F_0	BB986143
E_ET_MSV3_14A08_R_0	BB991425
E_ET_MSV3_14A09_F_0	BB986144
E_ET_MSV3_14A09_R_0	BB991426
E_ET_MSV3_14A10_F_0	BB986145
E_ET_MSV3_14A10_R_0	BB991427
E_ET_MSV3_14A11_F_0	BB986146
E_ET_MSV3_14A11_R_0	BB991428
E_ET_MSV3_14A12_F_0	BB986147
E_ET_MSV3_14A12_R_0	BB991429
E_ET_MSV3_14B01_F_0	BB986148
E_ET_MSV3_14B01_R_0	BB991430
E_ET_MSV3_14B02_F_0	BB986149
E_ET_MSV3_14B02_R_0	BB991431
E_ET_MSV3_14B03_F_0	BB986150
E_ET_MSV3_14B03_R_0	BB991432
E_ET_MSV3_14B04_R_0	BB991433
E_ET_MSV3_14B05_F_0	BB986151
E_ET_MSV3_14B06_F_0	BB986152
E_ET_MSV3_14B06_R_0	BB991434
E_ET_MSV3_14B07_F_0	BB986153
E_ET_MSV3_14B07_R_0	BB991435
E_ET_MSV3_14B08_F_0	BB986154
E_ET_MSV3_14B08_R_0	BB991436
E_ET_MSV3_14B09_F_0	BB986155
E_ET_MSV3_14B09_R_0	BB991437
E_ET_MSV3_14B10_F_0	BB986156
E_ET_MSV3_14B11_F_0	BB986157
E_ET_MSV3_14B11_R_0	BB991438
E_ET_MSV3_14B12_F_0	BB986158
E_ET_MSV3_14C01_F_0	BB986159
E_ET_MSV3_14C01_R_0	BB991439
E_ET_MSV3_14C03_F_0	BB986160
E_ET_MSV3_14C03_R_0	BB991440
E_ET_MSV3_14C04_F_0	BB986161
E_ET_MSV3_14C05_F_0	BB986162
E_ET_MSV3_14C05_R_0	BB991441
E_ET_MSV3_14C06_F_0	BB986163
E_ET_MSV3_14C06_R_0	BB991442
E_ET_MSV3_14C07_F_0	BB986164
E_ET_MSV3_14C07_R_0	BB991443
E_ET_MSV3_14C08_F_0	BB986165
E_ET_MSV3_14C08_R_0	BB991444
E_ET_MSV3_14C09_F_0	BB986166
E_ET_MSV3_14C09_R_0	BB991445
E_ET_MSV3_14C11_F_0	BB986167
E_ET_MSV3_14C11_R_0	BB991446
E_ET_MSV3_14C12_F_0	BB986168
E_ET_MSV3_14C12_R_0	BB991447
E_ET_MSV3_14D01_F_0	BB986169
E_ET_MSV3_14D01_R_0	BB991448
E_ET_MSV3_14D03_F_0	BB986170
E_ET_MSV3_14D03_R_0	BB991449

E_ET_MSV3_14D05_F_0	BB986171
E_ET_MSV3_14D05_R_0	BB991450
E_ET_MSV3_14D06_F_0	BB986172
E_ET_MSV3_14D06_R_0	BB991451
E_ET_MSV3_14D07_R_0	BB991452
E_ET_MSV3_14D08_F_0	BB986173
E_ET_MSV3_14D09_F_0	BB986174
E_ET_MSV3_14D09_R_0	BB991453
E_ET_MSV3_14D10_F_0	BB986175
E_ET_MSV3_14E01_F_0	BB986176
E_ET_MSV3_14E01_R_0	BB991454
E_ET_MSV3_14E02_F_0	BB986177
E_ET_MSV3_14E02_R_0	BB991455
E_ET_MSV3_14E04_F_0	BB986178
E_ET_MSV3_14E04_R_0	BB991456
E_ET_MSV3_14E05_R_0	BB991457
E_ET_MSV3_14E06_F_0	BB986179
E_ET_MSV3_14E06_R_0	BB991458
E_ET_MSV3_14E07_F_0	BB986180
E_ET_MSV3_14E07_R_0	BB991459
E_ET_MSV3_14E08_F_0	BB986181
E_ET_MSV3_14E08_R_0	BB991460
E_ET_MSV3_14E09_F_0	BB986182
E_ET_MSV3_14E09_R_0	BB991461
E_ET_MSV3_14E10_R_0	BB991462
E_ET_MSV3_14E11_F_0	BB986183
E_ET_MSV3_14E11_R_0	BB991463
E_ET_MSV3_14E12_F_0	BB986184
E_ET_MSV3_14F01_F_0	BB986185
E_ET_MSV3_14F01_R_0	BB991464
E_ET_MSV3_14F02_F_0	BB986186
E_ET_MSV3_14F02_R_0	BB991465
E_ET_MSV3_14F03_F_0	BB986187
E_ET_MSV3_14F03_R_0	BB991466
E_ET_MSV3_14F04_F_0	BB986188
E_ET_MSV3_14F04_R_0	BB991467
E_ET_MSV3_14F05_F_0	BB986189
E_ET_MSV3_14F05_R_0	BB991468
E_ET_MSV3_14F06_F_0	BB986190
E_ET_MSV3_14F07_F_0	BB986191
E_ET_MSV3_14F07_R_0	BB991469
E_ET_MSV3_14F09_F_0	BB986192
E_ET_MSV3_14F09_R_0	BB991470
E_ET_MSV3_14F10_F_0	BB986193
E_ET_MSV3_14F10_R_0	BB991471
E_ET_MSV3_14F11_F_0	BB986194
E_ET_MSV3_14F12_F_0	BB986195
E_ET_MSV3_14G01_F_0	BB986196
E_ET_MSV3_14G01_R_0	BB991472
E_ET_MSV3_14G02_F_0	BB986197
E_ET_MSV3_14G02_R_0	BB991473
E_ET_MSV3_14G04_F_0	BB986198
E_ET_MSV3_14G04_R_0	BB991474
E_ET_MSV3_14G05_R_0	BB991475

E_ET_MSV3_14G06_F_0	BB986199
E_ET_MSV3_14G06_R_0	BB991476
E_ET_MSV3_14G07_F_0	BB986200
E_ET_MSV3_14G07_R_0	BB991477
E_ET_MSV3_14G08_F_0	BB986201
E_ET_MSV3_14G09_F_0	BB986202
E_ET_MSV3_14G09_R_0	BB991478
E_ET_MSV3_14G10_F_0	BB986203
E_ET_MSV3_14G10_R_0	BB991479
E_ET_MSV3_14G11_F_0	BB986204
E_ET_MSV3_14G11_R_0	BB991480
E_ET_MSV3_14G12_F_0	BB986205
E_ET_MSV3_14G12_R_0	BB991481
E_ET_MSV3_14H01_F_0	BB986206
E_ET_MSV3_14H02_F_0	BB986207
E_ET_MSV3_14H03_F_0	BB986208
E_ET_MSV3_14H03_R_0	BB991482
E_ET_MSV3_14H04_F_0	BB986209
E_ET_MSV3_14H05_F_0	BB986210
E_ET_MSV3_14H06_F_0	BB986211
E_ET_MSV3_14H07_F_0	BB986212
E_ET_MSV3_14H07_R_0	BB991483
E_ET_MSV3_14H08_F_0	BB986213
E_ET_MSV3_14H08_R_0	BB991484
E_ET_MSV3_14H09_F_0	BB986214
E_ET_MSV3_14H09_R_0	BB991485
E_ET_MSV3_14H10_F_0	BB986215
E_ET_MSV3_14H10_R_0	BB991486
E_ET_MSV3_14H11_F_0	BB986216
E_ET_MSV3_14H11_R_0	BB991487
E_ET_MSV3_14H12_F_0	BB986217
E_ET_MSV3_15A01_F_0	BB986218
E_ET_MSV3_15A01_R_0	BB991488
E_ET_MSV3_15A02_F_0	BB986219
E_ET_MSV3_15A02_R_0	BB991489
E_ET_MSV3_15A03_R_0	BB991490
E_ET_MSV3_15A04_F_0	BB986220
E_ET_MSV3_15A04_R_0	BB991491
E_ET_MSV3_15A05_F_0	BB986221
E_ET_MSV3_15A06_F_0	BB986222
E_ET_MSV3_15A07_F_0	BB986223
E_ET_MSV3_15A07_R_0	BB991492
E_ET_MSV3_15A08_F_0	BB986224
E_ET_MSV3_15A08_R_0	BB991493
E_ET_MSV3_15A09_F_0	BB986225
E_ET_MSV3_15A09_R_0	BB991494
E_ET_MSV3_15A10_F_0	BB986226
E_ET_MSV3_15A11_F_0	BB986227
E_ET_MSV3_15A12_F_0	BB986228
E_ET_MSV3_15A12_R_0	BB991495
E_ET_MSV3_15B01_F_0	BB986229
E_ET_MSV3_15B01_R_0	BB991496
E_ET_MSV3_15B02_F_0	BB986230
E_ET_MSV3_15B02_R_0	BB991497

E_ET_MSV3_15B03_F_0	BB986231
E_ET_MSV3_15B03_R_0	BB991498
E_ET_MSV3_15B04_F_0	BB986232
E_ET_MSV3_15B04_R_0	BB991499
E_ET_MSV3_15B05_F_0	BB986233
E_ET_MSV3_15B06_F_0	BB986234
E_ET_MSV3_15B06_R_0	BB991500
E_ET_MSV3_15B07_F_0	BB986235
E_ET_MSV3_15B07_R_0	BB991501
E_ET_MSV3_15B08_F_0	BB986236
E_ET_MSV3_15B09_R_0	BB991502
E_ET_MSV3_15B10_F_0	BB986237
E_ET_MSV3_15B10_R_0	BB991503
E_ET_MSV3_15B11_F_0	BB986238
E_ET_MSV3_15B11_R_0	BB991504
E_ET_MSV3_15B12_F_0	BB986239
E_ET_MSV3_15C01_F_0	BB986240
E_ET_MSV3_15C01_R_0	BB991505
E_ET_MSV3_15C02_F_0	BB986241
E_ET_MSV3_15C02_R_0	BB991506
E_ET_MSV3_15C03_F_0	BB986242
E_ET_MSV3_15C03_R_0	BB991507
E_ET_MSV3_15C04_F_0	BB986243
E_ET_MSV3_15C04_R_0	BB991508
E_ET_MSV3_15C05_F_0	BB986244
E_ET_MSV3_15C05_R_0	BB991509
E_ET_MSV3_15C06_F_0	BB986245
E_ET_MSV3_15C06_R_0	BB991510
E_ET_MSV3_15C07_F_0	BB986246
E_ET_MSV3_15C07_R_0	BB991511
E_ET_MSV3_15C08_F_0	BB986247
E_ET_MSV3_15C08_R_0	BB991512
E_ET_MSV3_15C09_F_0	BB986248
E_ET_MSV3_15C10_R_0	BB991513
E_ET_MSV3_15C11_F_0	BB986249
E_ET_MSV3_15C11_R_0	BB991514
E_ET_MSV3_15C12_F_0	BB986250
E_ET_MSV3_15D01_F_0	BB986251
E_ET_MSV3_15D01_R_0	BB991515
E_ET_MSV3_15D02_F_0	BB986252
E_ET_MSV3_15D02_R_0	BB991516
E_ET_MSV3_15D03_F_0	BB986253
E_ET_MSV3_15D03_R_0	BB991517
E_ET_MSV3_15D04_R_0	BB991518
E_ET_MSV3_15D05_F_0	BB986254
E_ET_MSV3_15D05_R_0	BB991519
E_ET_MSV3_15D06_F_0	BB986255
E_ET_MSV3_15D07_F_0	BB986256
E_ET_MSV3_15D08_F_0	BB986257
E_ET_MSV3_15D08_R_0	BB991520
E_ET_MSV3_15D09_F_0	BB986258
E_ET_MSV3_15D10_F_0	BB986259
E_ET_MSV3_15D10_R_0	BB991521
E_ET_MSV3_15D12_F_0	BB986260

E_ET_MSV3_15D12_R_0	BB991522
E_ET_MSV3_15E01_F_0	BB986261
E_ET_MSV3_15E01_R_0	BB991523
E_ET_MSV3_15E02_F_0	BB986262
E_ET_MSV3_15E02_R_0	BB991524
E_ET_MSV3_15E03_F_0	BB986263
E_ET_MSV3_15E04_F_0	BB986264
E_ET_MSV3_15E04_R_0	BB991525
E_ET_MSV3_15E05_F_0	BB986265
E_ET_MSV3_15E05_R_0	BB991526
E_ET_MSV3_15E06_F_0	BB986266
E_ET_MSV3_15E06_R_0	BB991527
E_ET_MSV3_15E07_R_0	BB991528
E_ET_MSV3_15E08_F_0	BB986267
E_ET_MSV3_15E08_R_0	BB991529
E_ET_MSV3_15E09_R_0	BB991530
E_ET_MSV3_15E11_F_0	BB986268
E_ET_MSV3_15E11_R_0	BB991531
E_ET_MSV3_15E12_F_0	BB986269
E_ET_MSV3_15E12_R_0	BB991532
E_ET_MSV3_15F01_F_0	BB986270
E_ET_MSV3_15F01_R_0	BB991533
E_ET_MSV3_15F02_F_0	BB986271
E_ET_MSV3_15F02_R_0	BB991534
E_ET_MSV3_15F03_F_0	BB986272
E_ET_MSV3_15F03_R_0	BB991535
E_ET_MSV3_15F04_F_0	BB986273
E_ET_MSV3_15F04_R_0	BB991536
E_ET_MSV3_15F05_F_0	BB986274
E_ET_MSV3_15F05_R_0	BB991537
E_ET_MSV3_15F06_F_0	BB986275
E_ET_MSV3_15F07_R_0	BB991538
E_ET_MSV3_15F08_R_0	BB991539
E_ET_MSV3_15F09_F_0	BB986276
E_ET_MSV3_15F09_R_0	BB991540
E_ET_MSV3_15F10_F_0	BB986277
E_ET_MSV3_15F10_R_0	BB991541
E_ET_MSV3_15F11_F_0	BB986278
E_ET_MSV3_15G01_F_0	BB986279
E_ET_MSV3_15G01_R_0	BB991542
E_ET_MSV3_15G02_F_0	BB986280
E_ET_MSV3_15G02_R_0	BB991543
E_ET_MSV3_15G03_R_0	BB991544
E_ET_MSV3_15G04_F_0	BB986281
E_ET_MSV3_15G04_R_0	BB991545
E_ET_MSV3_15G05_F_0	BB986282
E_ET_MSV3_15G05_R_0	BB991546
E_ET_MSV3_15G06_F_0	BB986283
E_ET_MSV3_15G06_R_0	BB991547
E_ET_MSV3_15G07_F_0	BB986284
E_ET_MSV3_15G07_R_0	BB991548
E_ET_MSV3_15G08_F_0	BB986285
E_ET_MSV3_15G08_R_0	BB991549
E_ET_MSV3_15G09_F_0	BB986286

E_ET_MSV3_15G09_R_0	BB991550
E_ET_MSV3_15G10_R_0	BB991551
E_ET_MSV3_15G11_F_0	BB986287
E_ET_MSV3_15G11_R_0	BB991552
E_ET_MSV3_15G12_F_0	BB986288
E_ET_MSV3_15G12_R_0	BB991553
E_ET_MSV3_15H01_F_0	BB986289
E_ET_MSV3_15H01_R_0	BB991554
E_ET_MSV3_15H02_F_0	BB986290
E_ET_MSV3_15H02_R_0	BB991555
E_ET_MSV3_15H03_F_0	BB986291
E_ET_MSV3_15H03_R_0	BB991556
E_ET_MSV3_15H04_F_0	BB986292
E_ET_MSV3_15H04_R_0	BB991557
E_ET_MSV3_15H06_F_0	BB986293
E_ET_MSV3_15H06_R_0	BB991558
E_ET_MSV3_15H07_F_0	BB986294
E_ET_MSV3_15H07_R_0	BB991559
E_ET_MSV3_15H08_F_0	BB986295
E_ET_MSV3_15H08_R_0	BB991560
E_ET_MSV3_15H09_R_0	BB991561
E_ET_MSV3_15H10_F_0	BB986296
E_ET_MSV3_15H11_F_0	BB986297
E_ET_MSV3_15H11_R_0	BB991562
E_ET_MSV3_15H12_F_0	BB986298
E_ET_MSV3_15H12_R_0	BB991563
E_ET_MSV3_16A02_F_0	BB986299
E_ET_MSV3_16A02_R_0	BB991564
E_ET_MSV3_16A03_F_0	BB986300
E_ET_MSV3_16A03_R_0	BB991565
E_ET_MSV3_16A04_F_0	BB986301
E_ET_MSV3_16A04_R_0	BB991566
E_ET_MSV3_16A05_F_0	BB986302
E_ET_MSV3_16A05_R_0	BB991567
E_ET_MSV3_16A06_F_0	BB986303
E_ET_MSV3_16A06_R_0	BB991568
E_ET_MSV3_16A07_F_0	BB986304
E_ET_MSV3_16A08_F_0	BB986305
E_ET_MSV3_16A09_F_0	BB986306
E_ET_MSV3_16A09_R_0	BB991569
E_ET_MSV3_16A10_F_0	BB986307
E_ET_MSV3_16A11_F_0	BB986308
E_ET_MSV3_16A11_R_0	BB991570
E_ET_MSV3_16A12_F_0	BB986309
E_ET_MSV3_16A12_R_0	BB991571
E_ET_MSV3_16B01_F_0	BB986310
E_ET_MSV3_16B01_R_0	BB991572
E_ET_MSV3_16B02_F_0	BB986311
E_ET_MSV3_16B02_R_0	BB991573
E_ET_MSV3_16B03_F_0	BB986312
E_ET_MSV3_16B03_R_0	BB991574
E_ET_MSV3_16B04_F_0	BB986313
E_ET_MSV3_16B04_R_0	BB991575
E_ET_MSV3_16B05_R_0	BB991576

E_ET_MSV3_16B06_F_0	BB986314
E_ET_MSV3_16B07_F_0	BB986315
E_ET_MSV3_16B07_R_0	BB991577
E_ET_MSV3_16B08_F_0	BB986316
E_ET_MSV3_16B09_F_0	BB986317
E_ET_MSV3_16B10_F_0	BB986318
E_ET_MSV3_16B10_R_0	BB991578
E_ET_MSV3_16B11_F_0	BB986319
E_ET_MSV3_16B11_R_0	BB991579
E_ET_MSV3_16B12_R_0	BB991580
E_ET_MSV3_16C01_F_0	BB986320
E_ET_MSV3_16C01_R_0	BB991581
E_ET_MSV3_16C02_F_0	BB986321
E_ET_MSV3_16C02_R_0	BB991582
E_ET_MSV3_16C03_F_0	BB986322
E_ET_MSV3_16C03_R_0	BB991583
E_ET_MSV3_16C04_F_0	BB986323
E_ET_MSV3_16C04_R_0	BB991584
E_ET_MSV3_16C05_F_0	BB986324
E_ET_MSV3_16C05_R_0	BB991585
E_ET_MSV3_16C06_F_0	BB986325
E_ET_MSV3_16C06_R_0	BB991586
E_ET_MSV3_16C07_F_0	BB986326
E_ET_MSV3_16C07_R_0	BB991587
E_ET_MSV3_16C08_F_0	BB986327
E_ET_MSV3_16C08_R_0	BB991588
E_ET_MSV3_16C09_F_0	BB986328
E_ET_MSV3_16C09_R_0	BB991589
E_ET_MSV3_16C10_F_0	BB986329
E_ET_MSV3_16C10_R_0	BB991590
E_ET_MSV3_16C11_F_0	BB986330
E_ET_MSV3_16C11_R_0	BB991591
E_ET_MSV3_16C12_F_0	BB986331
E_ET_MSV3_16D01_F_0	BB986332
E_ET_MSV3_16D02_F_0	BB986333
E_ET_MSV3_16D02_R_0	BB991592
E_ET_MSV3_16D03_F_0	BB986334
E_ET_MSV3_16D03_R_0	BB991593
E_ET_MSV3_16D04_F_0	BB986335
E_ET_MSV3_16D04_R_0	BB991594
E_ET_MSV3_16D05_F_0	BB986336
E_ET_MSV3_16D05_R_0	BB991595
E_ET_MSV3_16D06_F_0	BB986337
E_ET_MSV3_16D06_R_0	BB991596
E_ET_MSV3_16D07_F_0	BB986338
E_ET_MSV3_16D07_R_0	BB991597
E_ET_MSV3_16D08_F_0	BB986339
E_ET_MSV3_16D08_R_0	BB991598
E_ET_MSV3_16D09_F_0	BB986340
E_ET_MSV3_16D09_R_0	BB991599
E_ET_MSV3_16D10_F_0	BB986341
E_ET_MSV3_16D11_F_0	BB986342
E_ET_MSV3_16D11_R_0	BB991600
E_ET_MSV3_16E01_F_0	BB986343

E_ET_MSV3_16E02_F_0	BB986344
E_ET_MSV3_16E04_F_0	BB986345
E_ET_MSV3_16E04_R_0	BB991601
E_ET_MSV3_16E05_F_0	BB986346
E_ET_MSV3_16E05_R_0	BB991602
E_ET_MSV3_16E06_F_0	BB986347
E_ET_MSV3_16E06_R_0	BB991603
E_ET_MSV3_16E07_F_0	BB986348
E_ET_MSV3_16E07_R_0	BB991604
E_ET_MSV3_16E08_F_0	BB986349
E_ET_MSV3_16E08_R_0	BB991605
E_ET_MSV3_16E09_F_0	BB986350
E_ET_MSV3_16E10_F_0	BB986351
E_ET_MSV3_16E10_R_0	BB991606
E_ET_MSV3_16E11_F_0	BB986352
E_ET_MSV3_16E11_R_0	BB991607
E_ET_MSV3_16E12_F_0	BB986353
E_ET_MSV3_16F01_F_0	BB986354
E_ET_MSV3_16F02_F_0	BB986355
E_ET_MSV3_16F02_R_0	BB991608
E_ET_MSV3_16F03_F_0	BB986356
E_ET_MSV3_16F03_R_0	BB991609
E_ET_MSV3_16F04_F_0	BB986357
E_ET_MSV3_16F04_R_0	BB991610
E_ET_MSV3_16F05_F_0	BB986358
E_ET_MSV3_16F05_R_0	BB991611
E_ET_MSV3_16F06_F_0	BB986359
E_ET_MSV3_16F06_R_0	BB991612
E_ET_MSV3_16F08_F_0	BB986360
E_ET_MSV3_16F08_R_0	BB991613
E_ET_MSV3_16F09_F_0	BB986361
E_ET_MSV3_16F09_R_0	BB991614
E_ET_MSV3_16F10_F_0	BB986362
E_ET_MSV3_16F10_R_0	BB991615
E_ET_MSV3_16F12_F_0	BB986363
E_ET_MSV3_16F12_R_0	BB991616
E_ET_MSV3_16G01_F_0	BB986364
E_ET_MSV3_16G01_R_0	BB991617
E_ET_MSV3_16G02_F_0	BB986365
E_ET_MSV3_16G02_R_0	BB991618
E_ET_MSV3_16G03_F_0	BB986366
E_ET_MSV3_16G03_R_0	BB991619
E_ET_MSV3_16G04_F_0	BB986367
E_ET_MSV3_16G04_R_0	BB991620
E_ET_MSV3_16G05_R_0	BB991621
E_ET_MSV3_16G06_F_0	BB986368
E_ET_MSV3_16G06_R_0	BB991622
E_ET_MSV3_16G07_F_0	BB986369
E_ET_MSV3_16G07_R_0	BB991623
E_ET_MSV3_16G08_F_0	BB986370
E_ET_MSV3_16G08_R_0	BB991624
E_ET_MSV3_16G09_F_0	BB986371
E_ET_MSV3_16G09_R_0	BB991625
E_ET_MSV3_16G10_F_0	BB986372

E_ET_MSV3_16G11_F_0	BB986373
E_ET_MSV3_16G12_F_0	BB986374
E_ET_MSV3_16G12_R_0	BB991626
E_ET_MSV3_16H01_F_0	BB986375
E_ET_MSV3_16H02_R_0	BB991627
E_ET_MSV3_16H03_F_0	BB986376
E_ET_MSV3_16H03_R_0	BB991628
E_ET_MSV3_16H04_F_0	BB986377
E_ET_MSV3_16H04_R_0	BB991629
E_ET_MSV3_16H05_F_0	BB986378
E_ET_MSV3_16H06_F_0	BB986379
E_ET_MSV3_16H07_R_0	BB991630
E_ET_MSV3_16H09_R_0	BB991631
E_ET_MSV3_16H10_F_0	BB986380
E_ET_MSV3_16H10_R_0	BB991632
E_ET_MSV3_16H11_F_0	BB986381
E_ET_MSV3_16H11_R_0	BB991633
E_ET_MSV3_17A03_R_0	BB991634
E_ET_MSV3_17A04_F_0	BB986382
E_ET_MSV3_17A04_R_0	BB991635
E_ET_MSV3_17A06_F_0	BB986383
E_ET_MSV3_17A07_F_0	BB986384
E_ET_MSV3_17A07_R_0	BB991636
E_ET_MSV3_17A09_F_0	BB986385
E_ET_MSV3_17A09_R_0	BB991637
E_ET_MSV3_17A10_F_0	BB986386
E_ET_MSV3_17A10_R_0	BB991638
E_ET_MSV3_17A12_F_0	BB986387
E_ET_MSV3_17B02_R_0	BB991639
E_ET_MSV3_17B04_F_0	BB986388
E_ET_MSV3_17B04_R_0	BB991640
E_ET_MSV3_17B05_F_0	BB986389
E_ET_MSV3_17B05_R_0	BB991641
E_ET_MSV3_17B06_F_0	BB986390
E_ET_MSV3_17B06_R_0	BB991642
E_ET_MSV3_17B07_F_0	BB986391
E_ET_MSV3_17B07_R_0	BB991643
E_ET_MSV3_17B08_F_0	BB986392
E_ET_MSV3_17B08_R_0	BB991644
E_ET_MSV3_17B09_F_0	BB986393
E_ET_MSV3_17B09_R_0	BB991645
E_ET_MSV3_17B10_F_0	BB986394
E_ET_MSV3_17B10_R_0	BB991646
E_ET_MSV3_17B11_F_0	BB986395
E_ET_MSV3_17B12_F_0	BB986396
E_ET_MSV3_17C01_R_0	BB991647
E_ET_MSV3_17C02_R_0	BB991648
E_ET_MSV3_17C03_R_0	BB991649
E_ET_MSV3_17C06_F_0	BB986397
E_ET_MSV3_17C06_R_0	BB991650
E_ET_MSV3_17C07_F_0	BB986398
E_ET_MSV3_17C07_R_0	BB991651
E_ET_MSV3_17C08_F_0	BB986399
E_ET_MSV3_17C08_R_0	BB991652

E_ET_MSV3_17C09_F_0	BB986400
E_ET_MSV3_17C09_R_0	BB991653
E_ET_MSV3_17C10_F_0	BB986401
E_ET_MSV3_17C11_F_0	BB986402
E_ET_MSV3_17C11_R_0	BB991654
E_ET_MSV3_17C12_F_0	BB986403
E_ET_MSV3_17C12_R_0	BB991655
E_ET_MSV3_17D01_F_0	BB986404
E_ET_MSV3_17D02_F_0	BB986405
E_ET_MSV3_17D02_R_0	BB991656
E_ET_MSV3_17D03_F_0	BB986406
E_ET_MSV3_17D03_R_0	BB991657
E_ET_MSV3_17D04_F_0	BB986407
E_ET_MSV3_17D05_F_0	BB986408
E_ET_MSV3_17D06_F_0	BB986409
E_ET_MSV3_17D06_R_0	BB991658
E_ET_MSV3_17D07_F_0	BB986410
E_ET_MSV3_17D08_F_0	BB986411
E_ET_MSV3_17D08_R_0	BB991659
E_ET_MSV3_17D09_F_0	BB986412
E_ET_MSV3_17D10_F_0	BB986413
E_ET_MSV3_17D11_F_0	BB986414
E_ET_MSV3_17D11_R_0	BB991660
E_ET_MSV3_17E02_R_0	BB991661
E_ET_MSV3_17E04_F_0	BB986415
E_ET_MSV3_17E04_R_0	BB991662
E_ET_MSV3_17E06_R_0	BB991663
E_ET_MSV3_17E07_R_0	BB991664
E_ET_MSV3_17E08_F_0	BB986416
E_ET_MSV3_17E08_R_0	BB991665
E_ET_MSV3_17E09_F_0	BB986417
E_ET_MSV3_17E10_F_0	BB986418
E_ET_MSV3_17E10_R_0	BB991666
E_ET_MSV3_17E11_F_0	BB986419
E_ET_MSV3_17E11_R_0	BB991667
E_ET_MSV3_17E12_F_0	BB986420
E_ET_MSV3_17F01_R_0	BB991668
E_ET_MSV3_17F02_F_0	BB986421
E_ET_MSV3_17F02_R_0	BB991669
E_ET_MSV3_17F04_F_0	BB986422
E_ET_MSV3_17F04_R_0	BB991670
E_ET_MSV3_17F05_F_0	BB986423
E_ET_MSV3_17F05_R_0	BB991671
E_ET_MSV3_17F06_F_0	BB986424
E_ET_MSV3_17F07_F_0	BB986425
E_ET_MSV3_17F07_R_0	BB991672
E_ET_MSV3_17F08_F_0	BB986426
E_ET_MSV3_17F09_F_0	BB986427
E_ET_MSV3_17F09_R_0	BB991673
E_ET_MSV3_17F10_F_0	BB986428
E_ET_MSV3_17F10_R_0	BB991674
E_ET_MSV3_17F11_F_0	BB986429
E_ET_MSV3_17F11_R_0	BB991675
E_ET_MSV3_17G02_F_0	BB986430

E_ET_MSV3_17G02_R_0	BB991676
E_ET_MSV3_17G03_F_0	BB986431
E_ET_MSV3_17G04_F_0	BB986432
E_ET_MSV3_17G05_F_0	BB986433
E_ET_MSV3_17G05_R_0	BB991677
E_ET_MSV3_17G06_F_0	BB986434
E_ET_MSV3_17G06_R_0	BB991678
E_ET_MSV3_17G07_F_0	BB986435
E_ET_MSV3_17G08_F_0	BB986436
E_ET_MSV3_17G09_F_0	BB986437
E_ET_MSV3_17G09_R_0	BB991679
E_ET_MSV3_17G10_F_0	BB986438
E_ET_MSV3_17G11_F_0	BB986439
E_ET_MSV3_17G11_R_0	BB991680
E_ET_MSV3_17G12_F_0	BB986440
E_ET_MSV3_17G12_R_0	BB991681
E_ET_MSV3_17H01_F_0	BB986441
E_ET_MSV3_17H01_R_0	BB991682
E_ET_MSV3_17H02_F_0	BB986442
E_ET_MSV3_17H02_R_0	BB991683
E_ET_MSV3_17H03_F_0	BB986443
E_ET_MSV3_17H03_R_0	BB991684
E_ET_MSV3_17H04_F_0	BB986444
E_ET_MSV3_17H04_R_0	BB991685
E_ET_MSV3_17H05_F_0	BB986445
E_ET_MSV3_17H05_R_0	BB991686
E_ET_MSV3_17H06_F_0	BB986446
E_ET_MSV3_17H06_R_0	BB991687
E_ET_MSV3_17H07_F_0	BB986447
E_ET_MSV3_17H07_R_0	BB991688
E_ET_MSV3_17H08_F_0	BB986448
E_ET_MSV3_17H09_F_0	BB986449
E_ET_MSV3_17H09_R_0	BB991689
E_ET_MSV3_17H10_F_0	BB986450
E_ET_MSV3_17H10_R_0	BB991690
E_ET_MSV3_17H11_F_0	BB986451
E_ET_MSV3_17H11_R_0	BB991691
E_ET_MSV3_17H12_F_0	BB986452
E_ET_MSV3_17H12_R_0	BB991692
E_ET_MSV3_18A01_F_0	BB986453
E_ET_MSV3_18A03_F_0	BB986454
E_ET_MSV3_18A04_F_0	BB986455
E_ET_MSV3_18A04_R_0	BB991693
E_ET_MSV3_18A05_R_0	BB991694
E_ET_MSV3_18A06_F_0	BB986456
E_ET_MSV3_18A06_R_0	BB991695
E_ET_MSV3_18A07_F_0	BB986457
E_ET_MSV3_18A07_R_0	BB991696
E_ET_MSV3_18A08_F_0	BB986458
E_ET_MSV3_18A09_F_0	BB986459
E_ET_MSV3_18A09_R_0	BB991697
E_ET_MSV3_18A10_F_0	BB986460
E_ET_MSV3_18A10_R_0	BB991698
E_ET_MSV3_18A11_R_0	BB991699

E_ET_MSV3_18A12_F_0	BB986461
E_ET_MSV3_18B01_F_0	BB986462
E_ET_MSV3_18B02_F_0	BB986463
E_ET_MSV3_18B03_F_0	BB986464
E_ET_MSV3_18B03_R_0	BB991700
E_ET_MSV3_18B04_F_0	BB986465
E_ET_MSV3_18B05_F_0	BB986466
E_ET_MSV3_18B06_F_0	BB986467
E_ET_MSV3_18B06_R_0	BB991701
E_ET_MSV3_18B07_F_0	BB986468
E_ET_MSV3_18B09_F_0	BB986469
E_ET_MSV3_18B10_F_0	BB986470
E_ET_MSV3_18B11_F_0	BB986471
E_ET_MSV3_18B11_R_0	BB991702
E_ET_MSV3_18B12_F_0	BB986472
E_ET_MSV3_18B12_R_0	BB991703
E_ET_MSV3_18C01_F_0	BB986473
E_ET_MSV3_18C02_F_0	BB986474
E_ET_MSV3_18C02_R_0	BB991704
E_ET_MSV3_18C03_F_0	BB986475
E_ET_MSV3_18C04_F_0	BB986476
E_ET_MSV3_18C04_R_0	BB991705
E_ET_MSV3_18C05_F_0	BB986477
E_ET_MSV3_18C05_R_0	BB991706
E_ET_MSV3_18C06_F_0	BB986478
E_ET_MSV3_18C06_R_0	BB991707
E_ET_MSV3_18C07_F_0	BB986479
E_ET_MSV3_18C07_R_0	BB991708
E_ET_MSV3_18C08_F_0	BB986480
E_ET_MSV3_18C09_F_0	BB986481
E_ET_MSV3_18C10_F_0	BB986482
E_ET_MSV3_18C10_R_0	BB991709
E_ET_MSV3_18C11_F_0	BB986483
E_ET_MSV3_18C11_R_0	BB991710
E_ET_MSV3_18C12_F_0	BB986484
E_ET_MSV3_18C12_R_0	BB991711
E_ET_MSV3_18D02_F_0	BB986485
E_ET_MSV3_18D03_F_0	BB986486
E_ET_MSV3_18D03_R_0	BB991712
E_ET_MSV3_18D04_F_0	BB986487
E_ET_MSV3_18D05_F_0	BB986488
E_ET_MSV3_18D06_F_0	BB986489
E_ET_MSV3_18D06_R_0	BB991713
E_ET_MSV3_18D08_F_0	BB986490
E_ET_MSV3_18D09_F_0	BB986491
E_ET_MSV3_18D09_R_0	BB991714
E_ET_MSV3_18D10_F_0	BB986492
E_ET_MSV3_18D10_R_0	BB991715
E_ET_MSV3_18D12_F_0	BB986493
E_ET_MSV3_18E03_F_0	BB986494
E_ET_MSV3_18E03_R_0	BB991716
E_ET_MSV3_18E04_F_0	BB986495
E_ET_MSV3_18E05_F_0	BB986496
E_ET_MSV3_18E05_R_0	BB991717

E_ET_MSV3_18E07_F_0	BB986497
E_ET_MSV3_18E09_F_0	BB986498
E_ET_MSV3_18E10_F_0	BB986499
E_ET_MSV3_18E10_R_0	BB991718
E_ET_MSV3_18E11_F_0	BB986500
E_ET_MSV3_18E11_R_0	BB991719
E_ET_MSV3_18E12_F_0	BB986501
E_ET_MSV3_18E12_R_0	BB991720
E_ET_MSV3_18F01_F_0	BB986502
E_ET_MSV3_18F02_F_0	BB986503
E_ET_MSV3_18F03_F_0	BB986504
E_ET_MSV3_18F03_R_0	BB991721
E_ET_MSV3_18F04_F_0	BB986505
E_ET_MSV3_18F04_R_0	BB991722
E_ET_MSV3_18F05_F_0	BB986506
E_ET_MSV3_18F05_R_0	BB991723
E_ET_MSV3_18F07_F_0	BB986507
E_ET_MSV3_18F08_F_0	BB986508
E_ET_MSV3_18F08_R_0	BB991724
E_ET_MSV3_18F09_F_0	BB986509
E_ET_MSV3_18F09_R_0	BB991725
E_ET_MSV3_18F10_F_0	BB986510
E_ET_MSV3_18F11_F_0	BB986511
E_ET_MSV3_18F11_R_0	BB991726
E_ET_MSV3_18F12_F_0	BB986512
E_ET_MSV3_18G01_F_0	BB986513
E_ET_MSV3_18G02_F_0	BB986514
E_ET_MSV3_18G03_F_0	BB986515
E_ET_MSV3_18G03_R_0	BB991727
E_ET_MSV3_18G04_F_0	BB986516
E_ET_MSV3_18G04_R_0	BB991728
E_ET_MSV3_18G05_F_0	BB986517
E_ET_MSV3_18G06_F_0	BB986518
E_ET_MSV3_18G06_R_0	BB991729
E_ET_MSV3_18G07_F_0	BB986519
E_ET_MSV3_18G08_F_0	BB986520
E_ET_MSV3_18G09_F_0	BB986521
E_ET_MSV3_18G10_F_0	BB986522
E_ET_MSV3_18G11_F_0	BB986523
E_ET_MSV3_18G11_R_0	BB991730
E_ET_MSV3_18G12_F_0	BB986524
E_ET_MSV3_18H02_F_0	BB986525
E_ET_MSV3_18H03_F_0	BB986526
E_ET_MSV3_18H05_F_0	BB986527
E_ET_MSV3_18H06_F_0	BB986528
E_ET_MSV3_18H07_F_0	BB986529
E_ET_MSV3_18H07_R_0	BB991731
E_ET_MSV3_18H08_F_0	BB986530
E_ET_MSV3_18H09_F_0	BB986531
E_ET_MSV3_18H09_R_0	BB991732
E_ET_MSV3_18H10_F_0	BB986532
E_ET_MSV3_18H10_R_0	BB991733
E_ET_MSV3_18H11_F_0	BB986533
E_ET_MSV3_18H12_F_0	BB986534

E_ET_MSV3_19A01_F_0	BB986535
E_ET_MSV3_19A01_R_0	BB991734
E_ET_MSV3_19A02_F_0	BB986536
E_ET_MSV3_19A02_R_0	BB991735
E_ET_MSV3_19A03_F_0	BB986537
E_ET_MSV3_19A04_F_0	BB986538
E_ET_MSV3_19A05_F_0	BB986539
E_ET_MSV3_19A06_F_0	BB986540
E_ET_MSV3_19A07_F_0	BB986541
E_ET_MSV3_19A07_R_0	BB991736
E_ET_MSV3_19A08_F_0	BB986542
E_ET_MSV3_19A09_F_0	BB986543
E_ET_MSV3_19A09_R_0	BB991737
E_ET_MSV3_19A10_F_0	BB986544
E_ET_MSV3_19A10_R_0	BB991738
E_ET_MSV3_19A11_R_0	BB991739
E_ET_MSV3_19A12_F_0	BB986545
E_ET_MSV3_19B02_F_0	BB986546
E_ET_MSV3_19B04_F_0	BB986547
E_ET_MSV3_19B05_F_0	BB986548
E_ET_MSV3_19B05_R_0	BB991740
E_ET_MSV3_19B06_F_0	BB986549
E_ET_MSV3_19B06_R_0	BB991741
E_ET_MSV3_19B07_F_0	BB986550
E_ET_MSV3_19B07_R_0	BB991742
E_ET_MSV3_19B09_F_0	BB986551
E_ET_MSV3_19B09_R_0	BB991743
E_ET_MSV3_19B10_F_0	BB986552
E_ET_MSV3_19B10_R_0	BB991744
E_ET_MSV3_19B11_F_0	BB986553
E_ET_MSV3_19B12_F_0	BB986554
E_ET_MSV3_19C01_F_0	BB986555
E_ET_MSV3_19C01_R_0	BB991745
E_ET_MSV3_19C02_R_0	BB991746
E_ET_MSV3_19C03_F_0	BB986556
E_ET_MSV3_19C03_R_0	BB991747
E_ET_MSV3_19C04_F_0	BB986557
E_ET_MSV3_19C04_R_0	BB991748
E_ET_MSV3_19C05_F_0	BB986558
E_ET_MSV3_19C06_F_0	BB986559
E_ET_MSV3_19C06_R_0	BB991749
E_ET_MSV3_19C07_F_0	BB986560
E_ET_MSV3_19C07_R_0	BB991750
E_ET_MSV3_19C08_F_0	BB986561
E_ET_MSV3_19C08_R_0	BB991751
E_ET_MSV3_19C09_F_0	BB986562
E_ET_MSV3_19C09_R_0	BB991752
E_ET_MSV3_19C10_F_0	BB986563
E_ET_MSV3_19C11_F_0	BB986564
E_ET_MSV3_19C11_R_0	BB991753
E_ET_MSV3_19C12_F_0	BB986565
E_ET_MSV3_19D01_F_0	BB986566
E_ET_MSV3_19D01_R_0	BB991754
E_ET_MSV3_19D02_F_0	BB986567

E_ET_MSV3_19D02_R_0	BB991755
E_ET_MSV3_19D03_F_0	BB986568
E_ET_MSV3_19D03_R_0	BB991756
E_ET_MSV3_19D04_F_0	BB986569
E_ET_MSV3_19D05_F_0	BB986570
E_ET_MSV3_19D05_R_0	BB991757
E_ET_MSV3_19D06_F_0	BB986571
E_ET_MSV3_19D06_R_0	BB991758
E_ET_MSV3_19D07_F_0	BB986572
E_ET_MSV3_19D08_R_0	BB991759
E_ET_MSV3_19D09_F_0	BB986573
E_ET_MSV3_19D09_R_0	BB991760
E_ET_MSV3_19D10_F_0	BB986574
E_ET_MSV3_19D10_R_0	BB991761
E_ET_MSV3_19D11_F_0	BB986575
E_ET_MSV3_19D11_R_0	BB991762
E_ET_MSV3_19D12_F_0	BB986576
E_ET_MSV3_19E01_F_0	BB986577
E_ET_MSV3_19E01_R_0	BB991763
E_ET_MSV3_19E03_F_0	BB986578
E_ET_MSV3_19E03_R_0	BB991764
E_ET_MSV3_19E04_F_0	BB986579
E_ET_MSV3_19E04_R_0	BB991765
E_ET_MSV3_19E05_F_0	BB986580
E_ET_MSV3_19E05_R_0	BB991766
E_ET_MSV3_19E06_F_0	BB986581
E_ET_MSV3_19E06_R_0	BB991767
E_ET_MSV3_19E08_F_0	BB986582
E_ET_MSV3_19E08_R_0	BB991768
E_ET_MSV3_19E09_F_0	BB986583
E_ET_MSV3_19E09_R_0	BB991769
E_ET_MSV3_19E10_F_0	BB986584
E_ET_MSV3_19E10_R_0	BB991770
E_ET_MSV3_19E11_R_0	BB991771
E_ET_MSV3_19E12_F_0	BB986585
E_ET_MSV3_19E12_R_0	BB991772
E_ET_MSV3_19F02_F_0	BB986586
E_ET_MSV3_19F02_R_0	BB991773
E_ET_MSV3_19F03_R_0	BB991774
E_ET_MSV3_19F04_F_0	BB986587
E_ET_MSV3_19F04_R_0	BB991775
E_ET_MSV3_19F06_F_0	BB986588
E_ET_MSV3_19F06_R_0	BB991776
E_ET_MSV3_19F07_F_0	BB986589
E_ET_MSV3_19F07_R_0	BB991777
E_ET_MSV3_19F08_F_0	BB986590
E_ET_MSV3_19F08_R_0	BB991778
E_ET_MSV3_19F09_F_0	BB986591
E_ET_MSV3_19F09_R_0	BB991779
E_ET_MSV3_19F11_F_0	BB986592
E_ET_MSV3_19F12_F_0	BB986593
E_ET_MSV3_19G01_F_0	BB986594
E_ET_MSV3_19G01_R_0	BB991780
E_ET_MSV3_19G02_F_0	BB986595

E_ET_MSV3_19G02_R_0	BB991781
E_ET_MSV3_19G03_F_0	BB986596
E_ET_MSV3_19G03_R_0	BB991782
E_ET_MSV3_19G04_F_0	BB986597
E_ET_MSV3_19G04_R_0	BB991783
E_ET_MSV3_19G06_F_0	BB986598
E_ET_MSV3_19G07_F_0	BB986599
E_ET_MSV3_19G09_F_0	BB986600
E_ET_MSV3_19G09_R_0	BB991784
E_ET_MSV3_19G10_F_0	BB986601
E_ET_MSV3_19G10_R_0	BB991785
E_ET_MSV3_19G11_F_0	BB986602
E_ET_MSV3_19G12_F_0	BB986603
E_ET_MSV3_19H01_F_0	BB986604
E_ET_MSV3_19H01_R_0	BB991786
E_ET_MSV3_19H02_F_0	BB986605
E_ET_MSV3_19H03_F_0	BB986606
E_ET_MSV3_19H03_R_0	BB991787
E_ET_MSV3_19H05_F_0	BB986607
E_ET_MSV3_19H06_R_0	BB991788
E_ET_MSV3_19H07_R_0	BB991789
E_ET_MSV3_19H08_F_0	BB986608
E_ET_MSV3_19H08_R_0	BB991790
E_ET_MSV3_19H09_F_0	BB986609
E_ET_MSV3_19H09_R_0	BB991791
E_ET_MSV3_19H10_R_0	BB991792
E_ET_MSV3_19H11_R_0	BB991793
E_ET_MSV3_20A01_F_0	BB986610
E_ET_MSV3_20A01_R_0	BB991794
E_ET_MSV3_20A02_F_0	BB986611
E_ET_MSV3_20A03_F_0	BB986612
E_ET_MSV3_20A03_R_0	BB991795
E_ET_MSV3_20A04_F_0	BB986613
E_ET_MSV3_20A05_F_0	BB986614
E_ET_MSV3_20A05_R_0	BB991796
E_ET_MSV3_20A07_F_0	BB986615
E_ET_MSV3_20A09_F_0	BB986616
E_ET_MSV3_20A09_R_0	BB991797
E_ET_MSV3_20A10_F_0	BB986617
E_ET_MSV3_20A11_F_0	BB986618
E_ET_MSV3_20A11_R_0	BB991798
E_ET_MSV3_20A12_F_0	BB986619
E_ET_MSV3_20B01_F_0	BB986620
E_ET_MSV3_20B02_F_0	BB986621
E_ET_MSV3_20B03_F_0	BB986622
E_ET_MSV3_20B04_F_0	BB986623
E_ET_MSV3_20B04_R_0	BB991799
E_ET_MSV3_20B05_F_0	BB986624
E_ET_MSV3_20B06_F_0	BB986625
E_ET_MSV3_20B07_F_0	BB986626
E_ET_MSV3_20B07_R_0	BB991800
E_ET_MSV3_20B08_F_0	BB986627
E_ET_MSV3_20B09_F_0	BB986628
E_ET_MSV3_20B09_R_0	BB991801

E_ET_MSV3_20B10_F_0	BB986629
E_ET_MSV3_20B11_F_0	BB986630
E_ET_MSV3_20B11_R_0	BB991802
E_ET_MSV3_20B12_F_0	BB986631
E_ET_MSV3_20C01_F_0	BB986632
E_ET_MSV3_20C02_F_0	BB986633
E_ET_MSV3_20C04_F_0	BB986634
E_ET_MSV3_20C04_R_0	BB991803
E_ET_MSV3_20C05_F_0	BB986635
E_ET_MSV3_20C05_R_0	BB991804
E_ET_MSV3_20C06_F_0	BB986636
E_ET_MSV3_20C07_F_0	BB986637
E_ET_MSV3_20C07_R_0	BB991805
E_ET_MSV3_20C08_F_0	BB986638
E_ET_MSV3_20C09_R_0	BB991806
E_ET_MSV3_20C10_F_0	BB986639
E_ET_MSV3_20C10_R_0	BB991807
E_ET_MSV3_20C11_F_0	BB986640
E_ET_MSV3_20C12_F_0	BB986641
E_ET_MSV3_20C12_R_0	BB991808
E_ET_MSV3_20D01_F_0	BB986642
E_ET_MSV3_20D01_R_0	BB991809
E_ET_MSV3_20D02_F_0	BB986643
E_ET_MSV3_20D02_R_0	BB991810
E_ET_MSV3_20D03_F_0	BB986644
E_ET_MSV3_20D03_R_0	BB991811
E_ET_MSV3_20D04_F_0	BB986645
E_ET_MSV3_20D05_F_0	BB986646
E_ET_MSV3_20D06_F_0	BB986647
E_ET_MSV3_20D06_R_0	BB991812
E_ET_MSV3_20D07_F_0	BB986648
E_ET_MSV3_20D07_R_0	BB991813
E_ET_MSV3_20D09_F_0	BB986649
E_ET_MSV3_20D09_R_0	BB991814
E_ET_MSV3_20D10_R_0	BB991815
E_ET_MSV3_20D11_F_0	BB986650
E_ET_MSV3_20D12_F_0	BB986651
E_ET_MSV3_20E01_F_0	BB986652
E_ET_MSV3_20E03_F_0	BB986653
E_ET_MSV3_20E04_F_0	BB986654
E_ET_MSV3_20E05_F_0	BB986655
E_ET_MSV3_20E05_R_0	BB991816
E_ET_MSV3_20E06_F_0	BB986656
E_ET_MSV3_20E07_R_0	BB991817
E_ET_MSV3_20E08_F_0	BB986657
E_ET_MSV3_20E09_F_0	BB986658
E_ET_MSV3_20E09_R_0	BB991818
E_ET_MSV3_20E10_R_0	BB991819
E_ET_MSV3_20E11_F_0	BB986659
E_ET_MSV3_20E11_R_0	BB991820
E_ET_MSV3_20E12_F_0	BB986660
E_ET_MSV3_20F02_F_0	BB986661
E_ET_MSV3_20F03_F_0	BB986662
E_ET_MSV3_20F03_R_0	BB991821

E_ET_MSV3_20F04_F_0	BB986663
E_ET_MSV3_20F04_R_0	BB991822
E_ET_MSV3_20F05_F_0	BB986664
E_ET_MSV3_20F06_F_0	BB986665
E_ET_MSV3_20F07_F_0	BB986666
E_ET_MSV3_20F08_F_0	BB986667
E_ET_MSV3_20F09_F_0	BB986668
E_ET_MSV3_20F10_F_0	BB986669
E_ET_MSV3_20F10_R_0	BB991823
E_ET_MSV3_20F11_F_0	BB986670
E_ET_MSV3_20F12_F_0	BB986671
E_ET_MSV3_20G01_F_0	BB986672
E_ET_MSV3_20G03_F_0	BB986673
E_ET_MSV3_20G03_R_0	BB991824
E_ET_MSV3_20G04_R_0	BB991825
E_ET_MSV3_20G05_F_0	BB986674
E_ET_MSV3_20G05_R_0	BB991826
E_ET_MSV3_20G06_F_0	BB986675
E_ET_MSV3_20G07_F_0	BB986676
E_ET_MSV3_20G08_R_0	BB991827
E_ET_MSV3_20G09_F_0	BB986677
E_ET_MSV3_20G10_F_0	BB986678
E_ET_MSV3_20G10_R_0	BB991828
E_ET_MSV3_20G11_F_0	BB986679
E_ET_MSV3_20G11_R_0	BB991829
E_ET_MSV3_20H01_F_0	BB986680
E_ET_MSV3_20H02_F_0	BB986681
E_ET_MSV3_20H03_F_0	BB986682
E_ET_MSV3_20H03_R_0	BB991830
E_ET_MSV3_20H04_F_0	BB986683
E_ET_MSV3_20H05_F_0	BB986684
E_ET_MSV3_20H06_F_0	BB986685
E_ET_MSV3_20H08_F_0	BB986686
E_ET_MSV3_20H09_F_0	BB986687
E_ET_MSV3_20H10_F_0	BB986688
E_ET_MSV3_20H11_F_0	BB986689
E_ET_MSV3_20H12_F_0	BB986690
E_ET_MSV3_21A01_F_0	BB986691
E_ET_MSV3_21A01_R_0	BB991831
E_ET_MSV3_21A02_F_0	BB986692
E_ET_MSV3_21A02_R_0	BB991832
E_ET_MSV3_21A03_F_0	BB986693
E_ET_MSV3_21A03_R_0	BB991833
E_ET_MSV3_21A05_F_0	BB986694
E_ET_MSV3_21A05_R_0	BB991834
E_ET_MSV3_21A06_F_0	BB986695
E_ET_MSV3_21A06_R_0	BB991835
E_ET_MSV3_21A08_F_0	BB986696
E_ET_MSV3_21A09_F_0	BB986697
E_ET_MSV3_21A09_R_0	BB991836
E_ET_MSV3_21A10_F_0	BB986698
E_ET_MSV3_21A10_R_0	BB991837
E_ET_MSV3_21A11_F_0	BB986699
E_ET_MSV3_21A11_R_0	BB991838

E_ET_MSV3_21A12_F_0	BB986700
E_ET_MSV3_21A12_R_0	BB991839
E_ET_MSV3_21B01_F_0	BB986701
E_ET_MSV3_21B01_R_0	BB991840
E_ET_MSV3_21B03_F_0	BB986702
E_ET_MSV3_21B03_R_0	BB991841
E_ET_MSV3_21B04_F_0	BB986703
E_ET_MSV3_21B04_R_0	BB991842
E_ET_MSV3_21B06_F_0	BB986704
E_ET_MSV3_21B06_R_0	BB991843
E_ET_MSV3_21B07_F_0	BB986705
E_ET_MSV3_21B07_R_0	BB991844
E_ET_MSV3_21B08_F_0	BB986706
E_ET_MSV3_21B08_R_0	BB991845
E_ET_MSV3_21B09_F_0	BB986707
E_ET_MSV3_21B09_R_0	BB991846
E_ET_MSV3_21B10_F_0	BB986708
E_ET_MSV3_21B10_R_0	BB991847
E_ET_MSV3_21B11_F_0	BB986709
E_ET_MSV3_21B11_R_0	BB991848
E_ET_MSV3_21B12_F_0	BB986710
E_ET_MSV3_21B12_R_0	BB991849
E_ET_MSV3_21C01_F_0	BB986711
E_ET_MSV3_21C03_F_0	BB986712
E_ET_MSV3_21C03_R_0	BB991850
E_ET_MSV3_21C04_F_0	BB986713
E_ET_MSV3_21C04_R_0	BB991851
E_ET_MSV3_21C05_F_0	BB986714
E_ET_MSV3_21C06_F_0	BB986715
E_ET_MSV3_21C06_R_0	BB991852
E_ET_MSV3_21C07_R_0	BB991853
E_ET_MSV3_21C08_F_0	BB986716
E_ET_MSV3_21C08_R_0	BB991854
E_ET_MSV3_21C09_F_0	BB986717
E_ET_MSV3_21C09_R_0	BB991855
E_ET_MSV3_21C10_F_0	BB986718
E_ET_MSV3_21C10_R_0	BB991856
E_ET_MSV3_21C11_F_0	BB986719
E_ET_MSV3_21C11_R_0	BB991857
E_ET_MSV3_21C12_F_0	BB986720
E_ET_MSV3_21C12_R_0	BB991858
E_ET_MSV3_21D01_F_0	BB986721
E_ET_MSV3_21D02_F_0	BB986722
E_ET_MSV3_21D02_R_0	BB991859
E_ET_MSV3_21D03_R_0	BB991860
E_ET_MSV3_21D04_F_0	BB986723
E_ET_MSV3_21D04_R_0	BB991861
E_ET_MSV3_21D05_R_0	BB991862
E_ET_MSV3_21D08_R_0	BB991863
E_ET_MSV3_21D10_F_0	BB986724
E_ET_MSV3_21D10_R_0	BB991864
E_ET_MSV3_21D11_F_0	BB986725
E_ET_MSV3_21D11_R_0	BB991865
E_ET_MSV3_21D12_F_0	BB986726

E_ET_MSV3_21D12_R_0	BB991866
E_ET_MSV3_21E01_F_0	BB986727
E_ET_MSV3_21E01_R_0	BB991867
E_ET_MSV3_21E02_F_0	BB986728
E_ET_MSV3_21E02_R_0	BB991868
E_ET_MSV3_21E04_F_0	BB986729
E_ET_MSV3_21E04_R_0	BB991869
E_ET_MSV3_21E05_F_0	BB986730
E_ET_MSV3_21E05_R_0	BB991870
E_ET_MSV3_21E06_F_0	BB986731
E_ET_MSV3_21E06_R_0	BB991871
E_ET_MSV3_21E07_R_0	BB991872
E_ET_MSV3_21E09_F_0	BB986732
E_ET_MSV3_21E09_R_0	BB991873
E_ET_MSV3_21E10_F_0	BB986733
E_ET_MSV3_21E10_R_0	BB991874
E_ET_MSV3_21E11_R_0	BB991875
E_ET_MSV3_21E12_F_0	BB986734
E_ET_MSV3_21E12_R_0	BB991876
E_ET_MSV3_21F01_F_0	BB986735
E_ET_MSV3_21F01_R_0	BB991877
E_ET_MSV3_21F02_F_0	BB986736
E_ET_MSV3_21F02_R_0	BB991878
E_ET_MSV3_21F03_F_0	BB986737
E_ET_MSV3_21F03_R_0	BB991879
E_ET_MSV3_21F04_F_0	BB986738
E_ET_MSV3_21F04_R_0	BB991880
E_ET_MSV3_21F06_F_0	BB986739
E_ET_MSV3_21F07_F_0	BB986740
E_ET_MSV3_21F08_F_0	BB986741
E_ET_MSV3_21F08_R_0	BB991881
E_ET_MSV3_21F09_F_0	BB986742
E_ET_MSV3_21F09_R_0	BB991882
E_ET_MSV3_21F10_R_0	BB991883
E_ET_MSV3_21F11_F_0	BB986743
E_ET_MSV3_21F12_F_0	BB986744
E_ET_MSV3_21F12_R_0	BB991884
E_ET_MSV3_21G02_F_0	BB986745
E_ET_MSV3_21G02_R_0	BB991885
E_ET_MSV3_21G03_F_0	BB986746
E_ET_MSV3_21G03_R_0	BB991886
E_ET_MSV3_21G04_F_0	BB986747
E_ET_MSV3_21G04_R_0	BB991887
E_ET_MSV3_21G06_F_0	BB986748
E_ET_MSV3_21G06_R_0	BB991888
E_ET_MSV3_21G07_F_0	BB986749
E_ET_MSV3_21G07_R_0	BB991889
E_ET_MSV3_21G08_F_0	BB986750
E_ET_MSV3_21G09_F_0	BB986751
E_ET_MSV3_21G09_R_0	BB991890
E_ET_MSV3_21G10_F_0	BB986752
E_ET_MSV3_21G10_R_0	BB991891
E_ET_MSV3_21G11_F_0	BB986753
E_ET_MSV3_21G11_R_0	BB991892

E_ET_MSV3_21G12_F_0	BB986754
E_ET_MSV3_21G12_R_0	BB991893
E_ET_MSV3_21H01_R_0	BB991894
E_ET_MSV3_21H02_F_0	BB986755
E_ET_MSV3_21H02_R_0	BB991895
E_ET_MSV3_21H03_F_0	BB986756
E_ET_MSV3_21H03_R_0	BB991896
E_ET_MSV3_21H04_F_0	BB986757
E_ET_MSV3_21H04_R_0	BB991897
E_ET_MSV3_21H05_F_0	BB986758
E_ET_MSV3_21H05_R_0	BB991898
E_ET_MSV3_21H07_F_0	BB986759
E_ET_MSV3_21H07_R_0	BB991899
E_ET_MSV3_21H08_F_0	BB986760
E_ET_MSV3_21H08_R_0	BB991900
E_ET_MSV3_21H09_F_0	BB986761
E_ET_MSV3_21H09_R_0	BB991901
E_ET_MSV3_21H10_F_0	BB986762
E_ET_MSV3_21H11_R_0	BB991902
E_ET_MSV3_21H12_F_0	BB986763
E_ET_MSV3_21H12_R_0	BB991903
E_ET_MSV3_22A01_F_0	BB986764
E_ET_MSV3_22A02_F_0	BB986765
E_ET_MSV3_22A02_R_0	BB991904
E_ET_MSV3_22A04_F_0	BB986766
E_ET_MSV3_22A05_F_0	BB986767
E_ET_MSV3_22A05_R_0	BB991905
E_ET_MSV3_22A06_F_0	BB986768
E_ET_MSV3_22A06_R_0	BB991906
E_ET_MSV3_22A07_F_0	BB986769
E_ET_MSV3_22A07_R_0	BB991907
E_ET_MSV3_22A08_F_0	BB986770
E_ET_MSV3_22A08_R_0	BB991908
E_ET_MSV3_22A09_F_0	BB986771
E_ET_MSV3_22A09_R_0	BB991909
E_ET_MSV3_22A10_F_0	BB986772
E_ET_MSV3_22A10_R_0	BB991910
E_ET_MSV3_22A12_F_0	BB986773
E_ET_MSV3_22A12_R_0	BB991911
E_ET_MSV3_22B01_F_0	BB986774
E_ET_MSV3_22B01_R_0	BB991912
E_ET_MSV3_22B02_R_0	BB991913
E_ET_MSV3_22B03_F_0	BB986775
E_ET_MSV3_22B03_R_0	BB991914
E_ET_MSV3_22B04_F_0	BB986776
E_ET_MSV3_22B04_R_0	BB991915
E_ET_MSV3_22B05_F_0	BB986777
E_ET_MSV3_22B05_R_0	BB991916
E_ET_MSV3_22B07_F_0	BB986778
E_ET_MSV3_22B08_F_0	BB986779
E_ET_MSV3_22B08_R_0	BB991917
E_ET_MSV3_22B09_F_0	BB986780
E_ET_MSV3_22B11_F_0	BB986781
E_ET_MSV3_22B11_R_0	BB991918

E_ET_MSV3_22B12_F_0	BB986782
E_ET_MSV3_22B12_R_0	BB991919
E_ET_MSV3_22C01_F_0	BB986783
E_ET_MSV3_22C01_R_0	BB991920
E_ET_MSV3_22C02_F_0	BB986784
E_ET_MSV3_22C02_R_0	BB991921
E_ET_MSV3_22C03_F_0	BB986785
E_ET_MSV3_22C03_R_0	BB991922
E_ET_MSV3_22C04_F_0	BB986786
E_ET_MSV3_22C04_R_0	BB991923
E_ET_MSV3_22C05_F_0	BB986787
E_ET_MSV3_22C05_R_0	BB991924
E_ET_MSV3_22C06_F_0	BB986788
E_ET_MSV3_22C07_F_0	BB986789
E_ET_MSV3_22C07_R_0	BB991925
E_ET_MSV3_22C08_F_0	BB986790
E_ET_MSV3_22C08_R_0	BB991926
E_ET_MSV3_22C09_F_0	BB986791
E_ET_MSV3_22C09_R_0	BB991927
E_ET_MSV3_22C10_F_0	BB986792
E_ET_MSV3_22C10_R_0	BB991928
E_ET_MSV3_22C12_F_0	BB986793
E_ET_MSV3_22D01_F_0	BB986794
E_ET_MSV3_22D01_R_0	BB991929
E_ET_MSV3_22D02_F_0	BB986795
E_ET_MSV3_22D02_R_0	BB991930
E_ET_MSV3_22D03_F_0	BB986796
E_ET_MSV3_22D03_R_0	BB991931
E_ET_MSV3_22D04_F_0	BB986797
E_ET_MSV3_22D04_R_0	BB991932
E_ET_MSV3_22D05_F_0	BB986798
E_ET_MSV3_22D05_R_0	BB991933
E_ET_MSV3_22D06_F_0	BB986799
E_ET_MSV3_22D07_R_0	BB991934
E_ET_MSV3_22D08_F_0	BB986800
E_ET_MSV3_22D08_R_0	BB991935
E_ET_MSV3_22D09_R_0	BB991936
E_ET_MSV3_22D10_F_0	BB986801
E_ET_MSV3_22D10_R_0	BB991937
E_ET_MSV3_22D11_R_0	BB991938
E_ET_MSV3_22D12_F_0	BB986802
E_ET_MSV3_22D12_R_0	BB991939
E_ET_MSV3_22E01_F_0	BB986803
E_ET_MSV3_22E01_R_0	BB991940
E_ET_MSV3_22E02_F_0	BB986804
E_ET_MSV3_22E02_R_0	BB991941
E_ET_MSV3_22E03_F_0	BB986805
E_ET_MSV3_22E03_R_0	BB991942
E_ET_MSV3_22E04_R_0	BB991943
E_ET_MSV3_22E05_F_0	BB986806
E_ET_MSV3_22E05_R_0	BB991944
E_ET_MSV3_22E06_F_0	BB986807
E_ET_MSV3_22E06_R_0	BB991945
E_ET_MSV3_22E07_F_0	BB986808

E_ET_MSV3_22E07_R_0	BB991946
E_ET_MSV3_22E09_F_0	BB986809
E_ET_MSV3_22E09_R_0	BB991947
E_ET_MSV3_22E10_F_0	BB986810
E_ET_MSV3_22E10_R_0	BB991948
E_ET_MSV3_22E11_F_0	BB986811
E_ET_MSV3_22E11_R_0	BB991949
E_ET_MSV3_22E12_F_0	BB986812
E_ET_MSV3_22F01_F_0	BB986813
E_ET_MSV3_22F01_R_0	BB991950
E_ET_MSV3_22F02_F_0	BB986814
E_ET_MSV3_22F02_R_0	BB991951
E_ET_MSV3_22F03_F_0	BB986815
E_ET_MSV3_22F04_F_0	BB986816
E_ET_MSV3_22F04_R_0	BB991952
E_ET_MSV3_22F05_F_0	BB986817
E_ET_MSV3_22F05_R_0	BB991953
E_ET_MSV3_22F06_R_0	BB991954
E_ET_MSV3_22F07_R_0	BB991955
E_ET_MSV3_22F08_F_0	BB986818
E_ET_MSV3_22F09_F_0	BB986819
E_ET_MSV3_22F09_R_0	BB991956
E_ET_MSV3_22F11_F_0	BB986820
E_ET_MSV3_22F11_R_0	BB991957
E_ET_MSV3_22F12_F_0	BB986821
E_ET_MSV3_22F12_R_0	BB991958
E_ET_MSV3_22G01_F_0	BB986822
E_ET_MSV3_22G01_R_0	BB991959
E_ET_MSV3_22G02_F_0	BB986823
E_ET_MSV3_22G02_R_0	BB991960
E_ET_MSV3_22G03_F_0	BB986824
E_ET_MSV3_22G03_R_0	BB991961
E_ET_MSV3_22G04_F_0	BB986825
E_ET_MSV3_22G04_R_0	BB991962
E_ET_MSV3_22G05_F_0	BB986826
E_ET_MSV3_22G05_R_0	BB991963
E_ET_MSV3_22G07_F_0	BB986827
E_ET_MSV3_22G07_R_0	BB991964
E_ET_MSV3_22G08_F_0	BB986828
E_ET_MSV3_22G08_R_0	BB991965
E_ET_MSV3_22G09_F_0	BB986829
E_ET_MSV3_22G10_F_0	BB986830
E_ET_MSV3_22G10_R_0	BB991966
E_ET_MSV3_22G11_F_0	BB986831
E_ET_MSV3_22G12_F_0	BB986832
E_ET_MSV3_22G12_R_0	BB991967
E_ET_MSV3_22H01_F_0	BB986833
E_ET_MSV3_22H01_R_0	BB991968
E_ET_MSV3_22H02_F_0	BB986834
E_ET_MSV3_22H02_R_0	BB991969
E_ET_MSV3_22H04_F_0	BB986835
E_ET_MSV3_22H04_R_0	BB991970
E_ET_MSV3_22H05_F_0	BB986836
E_ET_MSV3_22H05_R_0	BB991971

E_ET_MSV3_22H06_F_0	BB986837
E_ET_MSV3_22H06_R_0	BB991972
E_ET_MSV3_22H07_F_0	BB986838
E_ET_MSV3_22H07_R_0	BB991973
E_ET_MSV3_22H08_F_0	BB986839
E_ET_MSV3_22H08_R_0	BB991974
E_ET_MSV3_22H09_F_0	BB986840
E_ET_MSV3_22H09_R_0	BB991975
E_ET_MSV3_22H10_F_0	BB986841
E_ET_MSV3_22H10_R_0	BB991976
E_ET_MSV3_22H11_F_0	BB986842
E_ET_MSV3_22H11_R_0	BB991977
E_ET_MSV3_22H12_F_0	BB986843
E_ET_MSV3_22H12_R_0	BB991978
E_ET_MSV3_23A01_F_0	BB986844
E_ET_MSV3_23A01_R_0	BB991979
E_ET_MSV3_23A02_F_0	BB986845
E_ET_MSV3_23A03_F_0	BB986846
E_ET_MSV3_23A03_R_0	BB991980
E_ET_MSV3_23A04_F_0	BB986847
E_ET_MSV3_23A04_R_0	BB991981
E_ET_MSV3_23A05_F_0	BB986848
E_ET_MSV3_23A05_R_0	BB991982
E_ET_MSV3_23A06_F_0	BB986849
E_ET_MSV3_23A06_R_0	BB991983
E_ET_MSV3_23A07_F_0	BB986850
E_ET_MSV3_23A07_R_0	BB991984
E_ET_MSV3_23A08_F_0	BB986851
E_ET_MSV3_23A08_R_0	BB991985
E_ET_MSV3_23A09_F_0	BB986852
E_ET_MSV3_23A10_F_0	BB986853
E_ET_MSV3_23A10_R_0	BB991986
E_ET_MSV3_23A11_F_0	BB986854
E_ET_MSV3_23A11_R_0	BB991987
E_ET_MSV3_23A12_F_0	BB986855
E_ET_MSV3_23B01_F_0	BB986856
E_ET_MSV3_23B01_R_0	BB991988
E_ET_MSV3_23B02_F_0	BB986857
E_ET_MSV3_23B02_R_0	BB991989
E_ET_MSV3_23B03_F_0	BB986858
E_ET_MSV3_23B05_F_0	BB986859
E_ET_MSV3_23B05_R_0	BB991990
E_ET_MSV3_23B06_F_0	BB986860
E_ET_MSV3_23B07_F_0	BB986861
E_ET_MSV3_23B08_F_0	BB986862
E_ET_MSV3_23B08_R_0	BB991991
E_ET_MSV3_23B09_F_0	BB986863
E_ET_MSV3_23B10_F_0	BB986864
E_ET_MSV3_23B11_R_0	BB991992
E_ET_MSV3_23B12_F_0	BB986865
E_ET_MSV3_23B12_R_0	BB991993
E_ET_MSV3_23C01_F_0	BB986866
E_ET_MSV3_23C01_R_0	BB991994
E_ET_MSV3_23C02_F_0	BB986867

E_ET_MSV3_23C02_R_0	BB991995
E_ET_MSV3_23C03_F_0	BB986868
E_ET_MSV3_23C03_R_0	BB991996
E_ET_MSV3_23C04_F_0	BB986869
E_ET_MSV3_23C04_R_0	BB991997
E_ET_MSV3_23C05_F_0	BB986870
E_ET_MSV3_23C05_R_0	BB991998
E_ET_MSV3_23C06_F_0	BB986871
E_ET_MSV3_23C07_F_0	BB986872
E_ET_MSV3_23C07_R_0	BB991999
E_ET_MSV3_23C08_F_0	BB986873
E_ET_MSV3_23C08_R_0	BB992000
E_ET_MSV3_23C09_R_0	BB992001
E_ET_MSV3_23C10_F_0	BB986874
E_ET_MSV3_23C10_R_0	BB992002
E_ET_MSV3_23C11_F_0	BB986875
E_ET_MSV3_23C11_R_0	BB992003
E_ET_MSV3_23C12_F_0	BB986876
E_ET_MSV3_23C12_R_0	BB992004
E_ET_MSV3_23D01_F_0	BB986877
E_ET_MSV3_23D02_F_0	BB986878
E_ET_MSV3_23D02_R_0	BB992005
E_ET_MSV3_23D03_F_0	BB986879
E_ET_MSV3_23D03_R_0	BB992006
E_ET_MSV3_23D04_F_0	BB986880
E_ET_MSV3_23D04_R_0	BB992007
E_ET_MSV3_23D05_F_0	BB986881
E_ET_MSV3_23D05_R_0	BB992008
E_ET_MSV3_23D06_F_0	BB986882
E_ET_MSV3_23D06_R_0	BB992009
E_ET_MSV3_23D07_F_0	BB986883
E_ET_MSV3_23D07_R_0	BB992010
E_ET_MSV3_23D10_F_0	BB986884
E_ET_MSV3_23D10_R_0	BB992011
E_ET_MSV3_23D11_F_0	BB986885
E_ET_MSV3_23D11_R_0	BB992012
E_ET_MSV3_23D12_F_0	BB986886
E_ET_MSV3_23E01_F_0	BB986887
E_ET_MSV3_23E01_R_0	BB992013
E_ET_MSV3_23E02_F_0	BB986888
E_ET_MSV3_23E02_R_0	BB992014
E_ET_MSV3_23E03_F_0	BB986889
E_ET_MSV3_23E04_F_0	BB986890
E_ET_MSV3_23E04_R_0	BB992015
E_ET_MSV3_23E05_F_0	BB986891
E_ET_MSV3_23E05_R_0	BB992016
E_ET_MSV3_23E06_F_0	BB986892
E_ET_MSV3_23E06_R_0	BB992017
E_ET_MSV3_23E07_F_0	BB986893
E_ET_MSV3_23E08_F_0	BB986894
E_ET_MSV3_23E08_R_0	BB992018
E_ET_MSV3_23E10_F_0	BB986895
E_ET_MSV3_23E10_R_0	BB992019
E_ET_MSV3_23E11_F_0	BB986896

E_ET_MSV3_23E11_R_0	BB992020
E_ET_MSV3_23E12_F_0	BB986897
E_ET_MSV3_23F02_F_0	BB986898
E_ET_MSV3_23F02_R_0	BB992021
E_ET_MSV3_23F03_F_0	BB986899
E_ET_MSV3_23F03_R_0	BB992022
E_ET_MSV3_23F04_F_0	BB986900
E_ET_MSV3_23F04_R_0	BB992023
E_ET_MSV3_23F05_F_0	BB986901
E_ET_MSV3_23F06_F_0	BB986902
E_ET_MSV3_23F06_R_0	BB992024
E_ET_MSV3_23F07_F_0	BB986903
E_ET_MSV3_23F08_F_0	BB986904
E_ET_MSV3_23F08_R_0	BB992025
E_ET_MSV3_23F09_F_0	BB986905
E_ET_MSV3_23F09_R_0	BB992026
E_ET_MSV3_23F10_F_0	BB986906
E_ET_MSV3_23F10_R_0	BB992027
E_ET_MSV3_23F12_F_0	BB986907
E_ET_MSV3_23G01_F_0	BB986908
E_ET_MSV3_23G01_R_0	BB992028
E_ET_MSV3_23G02_F_0	BB986909
E_ET_MSV3_23G02_R_0	BB992029
E_ET_MSV3_23G03_F_0	BB986910
E_ET_MSV3_23G03_R_0	BB992030
E_ET_MSV3_23G04_F_0	BB986911
E_ET_MSV3_23G04_R_0	BB992031
E_ET_MSV3_23G05_F_0	BB986912
E_ET_MSV3_23G05_R_0	BB992032
E_ET_MSV3_23G06_F_0	BB986913
E_ET_MSV3_23G08_F_0	BB986914
E_ET_MSV3_23G08_R_0	BB992033
E_ET_MSV3_23G10_F_0	BB986915
E_ET_MSV3_23G10_R_0	BB992034
E_ET_MSV3_23G11_F_0	BB986916
E_ET_MSV3_23G11_R_0	BB992035
E_ET_MSV3_23G12_F_0	BB986917
E_ET_MSV3_23G12_R_0	BB992036
E_ET_MSV3_23H01_F_0	BB986918
E_ET_MSV3_23H01_R_0	BB992037
E_ET_MSV3_23H02_F_0	BB986919
E_ET_MSV3_23H02_R_0	BB992038
E_ET_MSV3_23H03_F_0	BB986920
E_ET_MSV3_23H03_R_0	BB992039
E_ET_MSV3_23H04_F_0	BB986921
E_ET_MSV3_23H04_R_0	BB992040
E_ET_MSV3_23H05_F_0	BB986922
E_ET_MSV3_23H06_F_0	BB986923
E_ET_MSV3_23H06_R_0	BB992041
E_ET_MSV3_23H07_F_0	BB986924
E_ET_MSV3_23H07_R_0	BB992042
E_ET_MSV3_23H08_F_0	BB986925
E_ET_MSV3_23H08_R_0	BB992043
E_ET_MSV3_23H09_R_0	BB992044

E_ET_MSV3_23H10_F_0	BB986926
E_ET_MSV3_23H12_F_0	BB986927
E_ET_MSV3_24A01_F_0	BB986928
E_ET_MSV3_24A01_R_0	BB992045
E_ET_MSV3_24A02_F_0	BB986929
E_ET_MSV3_24A02_R_0	BB992046
E_ET_MSV3_24A03_F_0	BB986930
E_ET_MSV3_24A03_R_0	BB992047
E_ET_MSV3_24A04_F_0	BB986931
E_ET_MSV3_24A05_F_0	BB986932
E_ET_MSV3_24A05_R_0	BB992048
E_ET_MSV3_24A06_F_0	BB986933
E_ET_MSV3_24A06_R_0	BB992049
E_ET_MSV3_24A07_R_0	BB992050
E_ET_MSV3_24A08_R_0	BB992051
E_ET_MSV3_24A09_F_0	BB986934
E_ET_MSV3_24A09_R_0	BB992052
E_ET_MSV3_24A10_F_0	BB986935
E_ET_MSV3_24A10_R_0	BB992053
E_ET_MSV3_24A11_F_0	BB986936
E_ET_MSV3_24A11_R_0	BB992054
E_ET_MSV3_24A12_R_0	BB992055
E_ET_MSV3_24B01_F_0	BB986937
E_ET_MSV3_24B01_R_0	BB992056
E_ET_MSV3_24B02_F_0	BB986938
E_ET_MSV3_24B02_R_0	BB992057
E_ET_MSV3_24B03_F_0	BB986939
E_ET_MSV3_24B03_R_0	BB992058
E_ET_MSV3_24B04_F_0	BB986940
E_ET_MSV3_24B04_R_0	BB992059
E_ET_MSV3_24B05_F_0	BB986941
E_ET_MSV3_24B06_R_0	BB992060
E_ET_MSV3_24B07_F_0	BB986942
E_ET_MSV3_24B07_R_0	BB992061
E_ET_MSV3_24B08_F_0	BB986943
E_ET_MSV3_24B08_R_0	BB992062
E_ET_MSV3_24B10_F_0	BB986944
E_ET_MSV3_24B10_R_0	BB992063
E_ET_MSV3_24C01_F_0	BB986945
E_ET_MSV3_24C01_R_0	BB992064
E_ET_MSV3_24C02_F_0	BB986946
E_ET_MSV3_24C02_R_0	BB992065
E_ET_MSV3_24C03_F_0	BB986947
E_ET_MSV3_24C03_R_0	BB992066
E_ET_MSV3_24C04_F_0	BB986948
E_ET_MSV3_24C04_R_0	BB992067
E_ET_MSV3_24C05_R_0	BB992068
E_ET_MSV3_24C06_F_0	BB986949
E_ET_MSV3_24C08_F_0	BB986950
E_ET_MSV3_24C08_R_0	BB992069
E_ET_MSV3_24C09_F_0	BB986951
E_ET_MSV3_24C10_F_0	BB986952
E_ET_MSV3_24C10_R_0	BB992070
E_ET_MSV3_24C11_F_0	BB986953

E_ET_MSV3_24D01_F_0	BB986954
E_ET_MSV3_24D01_R_0	BB992071
E_ET_MSV3_24D02_F_0	BB986955
E_ET_MSV3_24D02_R_0	BB992072
E_ET_MSV3_24D03_F_0	BB986956
E_ET_MSV3_24D03_R_0	BB992073
E_ET_MSV3_24D04_F_0	BB986957
E_ET_MSV3_24D04_R_0	BB992074
E_ET_MSV3_24D05_F_0	BB986958
E_ET_MSV3_24D06_F_0	BB986959
E_ET_MSV3_24D06_R_0	BB992075
E_ET_MSV3_24D07_F_0	BB986960
E_ET_MSV3_24D07_R_0	BB992076
E_ET_MSV3_24D08_F_0	BB986961
E_ET_MSV3_24D08_R_0	BB992077
E_ET_MSV3_24D09_F_0	BB986962
E_ET_MSV3_24D09_R_0	BB992078
E_ET_MSV3_24D10_F_0	BB986963
E_ET_MSV3_24D11_F_0	BB986964
E_ET_MSV3_24D11_R_0	BB992079
E_ET_MSV3_24D12_R_0	BB992080
E_ET_MSV3_24E01_F_0	BB986965
E_ET_MSV3_24E01_R_0	BB992081
E_ET_MSV3_24E02_F_0	BB986966
E_ET_MSV3_24E02_R_0	BB992082
E_ET_MSV3_24E04_F_0	BB986967
E_ET_MSV3_24E04_R_0	BB992083
E_ET_MSV3_24E06_F_0	BB986968
E_ET_MSV3_24E06_R_0	BB992084
E_ET_MSV3_24E07_F_0	BB986969
E_ET_MSV3_24E08_F_0	BB986970
E_ET_MSV3_24E08_R_0	BB992085
E_ET_MSV3_24E09_F_0	BB986971
E_ET_MSV3_24E09_R_0	BB992086
E_ET_MSV3_24E10_F_0	BB986972
E_ET_MSV3_24E10_R_0	BB992087
E_ET_MSV3_24E11_F_0	BB986973
E_ET_MSV3_24E11_R_0	BB992088
E_ET_MSV3_24E12_R_0	BB992089
E_ET_MSV3_24F01_F_0	BB986974
E_ET_MSV3_24F02_F_0	BB986975
E_ET_MSV3_24F02_R_0	BB992090
E_ET_MSV3_24F03_F_0	BB986976
E_ET_MSV3_24F03_R_0	BB992091
E_ET_MSV3_24F04_F_0	BB986977
E_ET_MSV3_24F04_R_0	BB992092
E_ET_MSV3_24F05_F_0	BB986978
E_ET_MSV3_24F05_R_0	BB992093
E_ET_MSV3_24F07_F_0	BB986979
E_ET_MSV3_24F07_R_0	BB992094
E_ET_MSV3_24F08_F_0	BB986980
E_ET_MSV3_24F08_R_0	BB992095
E_ET_MSV3_24F09_F_0	BB986981
E_ET_MSV3_24F09_R_0	BB992096

E_ET_MSV3_24F10_F_0	BB986982
E_ET_MSV3_24F11_R_0	BB992097
E_ET_MSV3_24F12_R_0	BB992098
E_ET_MSV3_24G01_F_0	BB986983
E_ET_MSV3_24G01_R_0	BB992099
E_ET_MSV3_24G02_F_0	BB986984
E_ET_MSV3_24G02_R_0	BB992100
E_ET_MSV3_24G03_F_0	BB986985
E_ET_MSV3_24G04_F_0	BB986986
E_ET_MSV3_24G04_R_0	BB992101
E_ET_MSV3_24G05_F_0	BB986987
E_ET_MSV3_24G07_F_0	BB986988
E_ET_MSV3_24G07_R_0	BB992102
E_ET_MSV3_24G10_F_0	BB986989
E_ET_MSV3_24G10_R_0	BB992103
E_ET_MSV3_24G11_F_0	BB986990
E_ET_MSV3_24G11_R_0	BB992104
E_ET_MSV3_24G12_R_0	BB992105
E_ET_MSV3_24H01_F_0	BB986991
E_ET_MSV3_24H01_R_0	BB992106
E_ET_MSV3_24H02_F_0	BB986992
E_ET_MSV3_24H02_R_0	BB992107
E_ET_MSV3_24H03_F_0	BB986993
E_ET_MSV3_24H04_F_0	BB986994
E_ET_MSV3_24H04_R_0	BB992108
E_ET_MSV3_24H05_R_0	BB992109
E_ET_MSV3_24H06_F_0	BB986995
E_ET_MSV3_24H06_R_0	BB992110
E_ET_MSV3_24H07_R_0	BB992111
E_ET_MSV3_24H08_F_0	BB986996
E_ET_MSV3_24H10_F_0	BB986997
E_ET_MSV3_24H10_R_0	BB992112
E_ET_MSV3_24H11_F_0	BB986998
E_ET_MSV3_24H12_R_0	BB992113
E_ET_MSV3_25A01_F_0	BB986999
E_ET_MSV3_25A01_R_0	BB992114
E_ET_MSV3_25A03_F_0	BB987000
E_ET_MSV3_25A03_R_0	BB992115
E_ET_MSV3_25A04_F_0	BB987001
E_ET_MSV3_25A05_R_0	BB992116
E_ET_MSV3_25A06_F_0	BB987002
E_ET_MSV3_25A06_R_0	BB992117
E_ET_MSV3_25A07_F_0	BB987003
E_ET_MSV3_25A07_R_0	BB992118
E_ET_MSV3_25A09_F_0	BB987004
E_ET_MSV3_25A09_R_0	BB992119
E_ET_MSV3_25A10_F_0	BB987005
E_ET_MSV3_25A10_R_0	BB992120
E_ET_MSV3_25A11_F_0	BB987006
E_ET_MSV3_25A11_R_0	BB992121
E_ET_MSV3_25A12_F_0	BB987007
E_ET_MSV3_25A12_R_0	BB992122
E_ET_MSV3_25B02_R_0	BB992123
E_ET_MSV3_25B03_R_0	BB992124

E_ET_MSV3_25B04_R_0	BB992125
E_ET_MSV3_25B05_F_0	BB987008
E_ET_MSV3_25B05_R_0	BB992126
E_ET_MSV3_25B06_R_0	BB992127
E_ET_MSV3_25B07_F_0	BB987009
E_ET_MSV3_25B07_R_0	BB992128
E_ET_MSV3_25B08_F_0	BB987010
E_ET_MSV3_25B08_R_0	BB992129
E_ET_MSV3_25B09_F_0	BB987011
E_ET_MSV3_25B09_R_0	BB992130
E_ET_MSV3_25B10_F_0	BB987012
E_ET_MSV3_25B11_F_0	BB987013
E_ET_MSV3_25B11_R_0	BB992131
E_ET_MSV3_25B12_F_0	BB987014
E_ET_MSV3_25B12_R_0	BB992132
E_ET_MSV3_25C01_F_0	BB987015
E_ET_MSV3_25C02_F_0	BB987016
E_ET_MSV3_25C02_R_0	BB992133
E_ET_MSV3_25C03_R_0	BB992134
E_ET_MSV3_25C04_F_0	BB987017
E_ET_MSV3_25C04_R_0	BB992135
E_ET_MSV3_25C05_F_0	BB987018
E_ET_MSV3_25C06_F_0	BB987019
E_ET_MSV3_25C06_R_0	BB992136
E_ET_MSV3_25C07_F_0	BB987020
E_ET_MSV3_25C07_R_0	BB992137
E_ET_MSV3_25C09_F_0	BB987021
E_ET_MSV3_25C09_R_0	BB992138
E_ET_MSV3_25C10_F_0	BB987022
E_ET_MSV3_25C10_R_0	BB992139
E_ET_MSV3_25C11_F_0	BB987023
E_ET_MSV3_25C11_R_0	BB992140
E_ET_MSV3_25C12_F_0	BB987024
E_ET_MSV3_25C12_R_0	BB992141
E_ET_MSV3_25D01_F_0	BB987025
E_ET_MSV3_25D02_F_0	BB987026
E_ET_MSV3_25D02_R_0	BB992142
E_ET_MSV3_25D03_F_0	BB987027
E_ET_MSV3_25D03_R_0	BB992143
E_ET_MSV3_25D04_F_0	BB987028
E_ET_MSV3_25D04_R_0	BB992144
E_ET_MSV3_25D05_R_0	BB992145
E_ET_MSV3_25D06_F_0	BB987029
E_ET_MSV3_25D06_R_0	BB992146
E_ET_MSV3_25D08_R_0	BB992147
E_ET_MSV3_25D09_F_0	BB987030
E_ET_MSV3_25D09_R_0	BB992148
E_ET_MSV3_25D10_F_0	BB987031
E_ET_MSV3_25D10_R_0	BB992149
E_ET_MSV3_25D11_F_0	BB987032
E_ET_MSV3_25D11_R_0	BB992150
E_ET_MSV3_25E01_F_0	BB987033
E_ET_MSV3_25E01_R_0	BB992151
E_ET_MSV3_25E02_F_0	BB987034

E_ET_MSV3_25E02_R_0	BB992152
E_ET_MSV3_25E03_F_0	BB987035
E_ET_MSV3_25E04_R_0	BB992153
E_ET_MSV3_25E06_F_0	BB987036
E_ET_MSV3_25E06_R_0	BB992154
E_ET_MSV3_25E07_R_0	BB992155
E_ET_MSV3_25E08_F_0	BB987037
E_ET_MSV3_25E09_F_0	BB987038
E_ET_MSV3_25E09_R_0	BB992156
E_ET_MSV3_25E10_F_0	BB987039
E_ET_MSV3_25E10_R_0	BB992157
E_ET_MSV3_25E11_F_0	BB987040
E_ET_MSV3_25E11_R_0	BB992158
E_ET_MSV3_25F01_F_0	BB987041
E_ET_MSV3_25F02_F_0	BB987042
E_ET_MSV3_25F02_R_0	BB992159
E_ET_MSV3_25F05_R_0	BB992160
E_ET_MSV3_25F06_F_0	BB987043
E_ET_MSV3_25F06_R_0	BB992161
E_ET_MSV3_25F07_F_0	BB987044
E_ET_MSV3_25F07_R_0	BB992162
E_ET_MSV3_25F08_F_0	BB987045
E_ET_MSV3_25F08_R_0	BB992163
E_ET_MSV3_25F09_F_0	BB987046
E_ET_MSV3_25F09_R_0	BB992164
E_ET_MSV3_25F10_F_0	BB987047
E_ET_MSV3_25F10_R_0	BB992165
E_ET_MSV3_25F11_F_0	BB987048
E_ET_MSV3_25F11_R_0	BB992166
E_ET_MSV3_25F12_F_0	BB987049
E_ET_MSV3_25F12_R_0	BB992167
E_ET_MSV3_25G01_F_0	BB987050
E_ET_MSV3_25G01_R_0	BB992168
E_ET_MSV3_25G02_F_0	BB987051
E_ET_MSV3_25G03_F_0	BB987052
E_ET_MSV3_25G03_R_0	BB992169
E_ET_MSV3_25G04_F_0	BB987053
E_ET_MSV3_25G04_R_0	BB992170
E_ET_MSV3_25G05_F_0	BB987054
E_ET_MSV3_25G05_R_0	BB992171
E_ET_MSV3_25G06_F_0	BB987055
E_ET_MSV3_25G07_F_0	BB987056
E_ET_MSV3_25G07_R_0	BB992172
E_ET_MSV3_25G08_F_0	BB987057
E_ET_MSV3_25G09_R_0	BB992173
E_ET_MSV3_25G10_R_0	BB992174
E_ET_MSV3_25H01_F_0	BB987058
E_ET_MSV3_25H01_R_0	BB992175
E_ET_MSV3_25H02_F_0	BB987059
E_ET_MSV3_25H02_R_0	BB992176
E_ET_MSV3_25H03_F_0	BB987060
E_ET_MSV3_25H03_R_0	BB992177
E_ET_MSV3_25H04_F_0	BB987061
E_ET_MSV3_25H04_R_0	BB992178

E_ET_MSV3_25H05_R_0	BB992179
E_ET_MSV3_25H06_F_0	BB987062
E_ET_MSV3_25H06_R_0	BB992180
E_ET_MSV3_25H07_F_0	BB987063
E_ET_MSV3_25H07_R_0	BB992181
E_ET_MSV3_25H08_R_0	BB992182
E_ET_MSV3_25H09_F_0	BB987064
E_ET_MSV3_25H09_R_0	BB992183
E_ET_MSV3_25H10_F_0	BB987065
E_ET_MSV3_25H10_R_0	BB992184
E_ET_MSV3_25H11_F_0	BB987066
E_ET_MSV3_25H11_R_0	BB992185
E_ET_MSV3_25H12_F_0	BB987067
E_ET_MSV3_25H12_R_0	BB992186
E_ET_MSV3_26A01_F_0	BB987068
E_ET_MSV3_26A01_R_0	BB992187
E_ET_MSV3_26A02_F_0	BB987069
E_ET_MSV3_26A02_R_0	BB992188
E_ET_MSV3_26A03_F_0	BB987070
E_ET_MSV3_26A03_R_0	BB992189
E_ET_MSV3_26A04_R_0	BB992190
E_ET_MSV3_26A06_F_0	BB987071
E_ET_MSV3_26A06_R_0	BB992191
E_ET_MSV3_26A07_F_0	BB987072
E_ET_MSV3_26A08_R_0	BB992192
E_ET_MSV3_26A09_F_0	BB987073
E_ET_MSV3_26A09_R_0	BB992193
E_ET_MSV3_26A10_F_0	BB987074
E_ET_MSV3_26A10_R_0	BB992194
E_ET_MSV3_26A11_F_0	BB987075
E_ET_MSV3_26A11_R_0	BB992195
E_ET_MSV3_26A12_F_0	BB987076
E_ET_MSV3_26A12_R_0	BB992196
E_ET_MSV3_26B01_F_0	BB987077
E_ET_MSV3_26B01_R_0	BB992197
E_ET_MSV3_26B02_R_0	BB992198
E_ET_MSV3_26B03_F_0	BB987078
E_ET_MSV3_26B03_R_0	BB992199
E_ET_MSV3_26B05_F_0	BB987079
E_ET_MSV3_26B06_F_0	BB987080
E_ET_MSV3_26B06_R_0	BB992200
E_ET_MSV3_26B07_F_0	BB987081
E_ET_MSV3_26B07_R_0	BB992201
E_ET_MSV3_26B08_F_0	BB987082
E_ET_MSV3_26B08_R_0	BB992202
E_ET_MSV3_26B09_F_0	BB987083
E_ET_MSV3_26B09_R_0	BB992203
E_ET_MSV3_26B10_F_0	BB987084
E_ET_MSV3_26B10_R_0	BB992204
E_ET_MSV3_26B11_F_0	BB987085
E_ET_MSV3_26B11_R_0	BB992205
E_ET_MSV3_26B12_F_0	BB987086
E_ET_MSV3_26B12_R_0	BB992206
E_ET_MSV3_26C01_F_0	BB987087

E_ET_MSV3_26C01_R_0	BB992207
E_ET_MSV3_26C02_F_0	BB987088
E_ET_MSV3_26C02_R_0	BB992208
E_ET_MSV3_26C03_F_0	BB987089
E_ET_MSV3_26C04_F_0	BB987090
E_ET_MSV3_26C04_R_0	BB992209
E_ET_MSV3_26C05_F_0	BB987091
E_ET_MSV3_26C05_R_0	BB992210
E_ET_MSV3_26C06_F_0	BB987092
E_ET_MSV3_26C08_F_0	BB987093
E_ET_MSV3_26C08_R_0	BB992211
E_ET_MSV3_26C09_F_0	BB987094
E_ET_MSV3_26C09_R_0	BB992212
E_ET_MSV3_26C10_F_0	BB987095
E_ET_MSV3_26C10_R_0	BB992213
E_ET_MSV3_26C11_R_0	BB992214
E_ET_MSV3_26C12_F_0	BB987096
E_ET_MSV3_26D01_F_0	BB987097
E_ET_MSV3_26D01_R_0	BB992215
E_ET_MSV3_26D02_R_0	BB992216
E_ET_MSV3_26D03_F_0	BB987098
E_ET_MSV3_26D03_R_0	BB992217
E_ET_MSV3_26D05_F_0	BB987099
E_ET_MSV3_26D06_F_0	BB987100
E_ET_MSV3_26D07_F_0	BB987101
E_ET_MSV3_26D07_R_0	BB992218
E_ET_MSV3_26D09_F_0	BB987102
E_ET_MSV3_26D09_R_0	BB992219
E_ET_MSV3_26D10_F_0	BB987103
E_ET_MSV3_26D10_R_0	BB992220
E_ET_MSV3_26D11_F_0	BB987104
E_ET_MSV3_26D11_R_0	BB992221
E_ET_MSV3_26D12_F_0	BB987105
E_ET_MSV3_26D12_R_0	BB992222
E_ET_MSV3_26E01_F_0	BB987106
E_ET_MSV3_26E01_R_0	BB992223
E_ET_MSV3_26E02_F_0	BB987107
E_ET_MSV3_26E02_R_0	BB992224
E_ET_MSV3_26E03_F_0	BB987108
E_ET_MSV3_26E03_R_0	BB992225
E_ET_MSV3_26E05_F_0	BB987109
E_ET_MSV3_26E05_R_0	BB992226
E_ET_MSV3_26E06_R_0	BB992227
E_ET_MSV3_26E07_R_0	BB992228
E_ET_MSV3_26E08_F_0	BB987110
E_ET_MSV3_26E08_R_0	BB992229
E_ET_MSV3_26E09_R_0	BB992230
E_ET_MSV3_26E10_F_0	BB987111
E_ET_MSV3_26E10_R_0	BB992231
E_ET_MSV3_26E11_F_0	BB987112
E_ET_MSV3_26E11_R_0	BB992232
E_ET_MSV3_26E12_F_0	BB987113
E_ET_MSV3_26E12_R_0	BB992233
E_ET_MSV3_26F01_F_0	BB987114

E_ET_MSV3_26F01_R_0	BB992234
E_ET_MSV3_26F02_F_0	BB987115
E_ET_MSV3_26F02_R_0	BB992235
E_ET_MSV3_26F03_F_0	BB987116
E_ET_MSV3_26F03_R_0	BB992236
E_ET_MSV3_26F04_F_0	BB987117
E_ET_MSV3_26F04_R_0	BB992237
E_ET_MSV3_26F05_F_0	BB987118
E_ET_MSV3_26F05_R_0	BB992238
E_ET_MSV3_26F06_R_0	BB992239
E_ET_MSV3_26F07_F_0	BB987119
E_ET_MSV3_26F07_R_0	BB992240
E_ET_MSV3_26F08_F_0	BB987120
E_ET_MSV3_26F10_F_0	BB987121
E_ET_MSV3_26F10_R_0	BB992241
E_ET_MSV3_26F11_F_0	BB987122
E_ET_MSV3_26G01_F_0	BB987123
E_ET_MSV3_26G01_R_0	BB992242
E_ET_MSV3_26G02_F_0	BB987124
E_ET_MSV3_26G02_R_0	BB992243
E_ET_MSV3_26G03_R_0	BB992244
E_ET_MSV3_26G04_F_0	BB987125
E_ET_MSV3_26G04_R_0	BB992245
E_ET_MSV3_26G05_R_0	BB992246
E_ET_MSV3_26G06_R_0	BB992247
E_ET_MSV3_26G07_F_0	BB987126
E_ET_MSV3_26G08_F_0	BB987127
E_ET_MSV3_26G09_F_0	BB987128
E_ET_MSV3_26G09_R_0	BB992248
E_ET_MSV3_26G10_F_0	BB987129
E_ET_MSV3_26G10_R_0	BB992249
E_ET_MSV3_26G11_F_0	BB987130
E_ET_MSV3_26G12_R_0	BB992250
E_ET_MSV3_26H01_F_0	BB987131
E_ET_MSV3_26H01_R_0	BB992251
E_ET_MSV3_26H02_R_0	BB992252
E_ET_MSV3_26H03_R_0	BB992253
E_ET_MSV3_26H04_R_0	BB992254
E_ET_MSV3_26H05_R_0	BB992255
E_ET_MSV3_26H08_F_0	BB987132
E_ET_MSV3_26H08_R_0	BB992256
E_ET_MSV3_26H09_F_0	BB987133
E_ET_MSV3_26H10_R_0	BB992257
E_ET_MSV3_26H11_R_0	BB992258
E_ET_MSV3_26H12_F_0	BB987134
E_ET_MSV3_27A01_F_0	BB987135
E_ET_MSV3_27A01_R_0	BB992259
E_ET_MSV3_27A02_F_0	BB987136
E_ET_MSV3_27A03_F_0	BB987137
E_ET_MSV3_27A03_R_0	BB992260
E_ET_MSV3_27A04_F_0	BB987138
E_ET_MSV3_27A05_F_0	BB987139
E_ET_MSV3_27A05_R_0	BB992261
E_ET_MSV3_27A06_F_0	BB987140

E_ET_MSV3_27A06_R_0	BB992262
E_ET_MSV3_27A07_F_0	BB987141
E_ET_MSV3_27A08_F_0	BB987142
E_ET_MSV3_27A08_R_0	BB992263
E_ET_MSV3_27A09_F_0	BB987143
E_ET_MSV3_27A09_R_0	BB992264
E_ET_MSV3_27A10_F_0	BB987144
E_ET_MSV3_27A10_R_0	BB992265
E_ET_MSV3_27A11_F_0	BB987145
E_ET_MSV3_27A11_R_0	BB992266
E_ET_MSV3_27A12_F_0	BB987146
E_ET_MSV3_27A12_R_0	BB992267
E_ET_MSV3_27B01_F_0	BB987147
E_ET_MSV3_27B01_R_0	BB992268
E_ET_MSV3_27B03_F_0	BB987148
E_ET_MSV3_27B03_R_0	BB992269
E_ET_MSV3_27B04_F_0	BB987149
E_ET_MSV3_27B05_F_0	BB987150
E_ET_MSV3_27B05_R_0	BB992270
E_ET_MSV3_27B06_F_0	BB987151
E_ET_MSV3_27B06_R_0	BB992271
E_ET_MSV3_27B07_F_0	BB987152
E_ET_MSV3_27B07_R_0	BB992272
E_ET_MSV3_27B08_F_0	BB987153
E_ET_MSV3_27B08_R_0	BB992273
E_ET_MSV3_27B09_F_0	BB987154
E_ET_MSV3_27B09_R_0	BB992274
E_ET_MSV3_27B10_F_0	BB987155
E_ET_MSV3_27B11_F_0	BB987156
E_ET_MSV3_27C01_F_0	BB987157
E_ET_MSV3_27C01_R_0	BB992275
E_ET_MSV3_27C02_F_0	BB987158
E_ET_MSV3_27C02_R_0	BB992276
E_ET_MSV3_27C03_F_0	BB987159
E_ET_MSV3_27C03_R_0	BB992277
E_ET_MSV3_27C04_F_0	BB987160
E_ET_MSV3_27C04_R_0	BB992278
E_ET_MSV3_27C05_F_0	BB987161
E_ET_MSV3_27C05_R_0	BB992279
E_ET_MSV3_27C06_F_0	BB987162
E_ET_MSV3_27C06_R_0	BB992280
E_ET_MSV3_27C07_F_0	BB987163
E_ET_MSV3_27C07_R_0	BB992281
E_ET_MSV3_27C08_F_0	BB987164
E_ET_MSV3_27C09_F_0	BB987165
E_ET_MSV3_27C09_R_0	BB992282
E_ET_MSV3_27C11_F_0	BB987166
E_ET_MSV3_27C11_R_0	BB992283
E_ET_MSV3_27C12_R_0	BB992284
E_ET_MSV3_27D01_F_0	BB987167
E_ET_MSV3_27D01_R_0	BB992285
E_ET_MSV3_27D02_R_0	BB992286
E_ET_MSV3_27D03_F_0	BB987168
E_ET_MSV3_27D03_R_0	BB992287

E_ET_MSV3_27D04_R_0	BB992288
E_ET_MSV3_27D05_F_0	BB987169
E_ET_MSV3_27D06_F_0	BB987170
E_ET_MSV3_27D06_R_0	BB992289
E_ET_MSV3_27D07_F_0	BB987171
E_ET_MSV3_27D07_R_0	BB992290
E_ET_MSV3_27D08_F_0	BB987172
E_ET_MSV3_27D08_R_0	BB992291
E_ET_MSV3_27D09_F_0	BB987173
E_ET_MSV3_27D09_R_0	BB992292
E_ET_MSV3_27D10_F_0	BB987174
E_ET_MSV3_27D10_R_0	BB992293
E_ET_MSV3_27D11_F_0	BB987175
E_ET_MSV3_27D11_R_0	BB992294
E_ET_MSV3_27D12_F_0	BB987176
E_ET_MSV3_27D12_R_0	BB992295
E_ET_MSV3_27E01_F_0	BB987177
E_ET_MSV3_27E02_R_0	BB992296
E_ET_MSV3_27E03_R_0	BB992297
E_ET_MSV3_27E05_R_0	BB992298
E_ET_MSV3_27E06_F_0	BB987178
E_ET_MSV3_27E06_R_0	BB992299
E_ET_MSV3_27E07_F_0	BB987179
E_ET_MSV3_27E07_R_0	BB992300
E_ET_MSV3_27E09_F_0	BB987180
E_ET_MSV3_27E09_R_0	BB992301
E_ET_MSV3_27E10_R_0	BB992302
E_ET_MSV3_27E11_F_0	BB987181
E_ET_MSV3_27E11_R_0	BB992303
E_ET_MSV3_27F02_F_0	BB987182
E_ET_MSV3_27F03_F_0	BB987183
E_ET_MSV3_27F03_R_0	BB992304
E_ET_MSV3_27F04_R_0	BB992305
E_ET_MSV3_27F05_F_0	BB987184
E_ET_MSV3_27F06_F_0	BB987185
E_ET_MSV3_27F06_R_0	BB992306
E_ET_MSV3_27F07_F_0	BB987186
E_ET_MSV3_27F08_F_0	BB987187
E_ET_MSV3_27F08_R_0	BB992307
E_ET_MSV3_27F09_F_0	BB987188
E_ET_MSV3_27F10_F_0	BB987189
E_ET_MSV3_27F10_R_0	BB992308
E_ET_MSV3_27F11_F_0	BB987190
E_ET_MSV3_27F12_F_0	BB987191
E_ET_MSV3_27F12_R_0	BB992309
E_ET_MSV3_27G02_F_0	BB987192
E_ET_MSV3_27G02_R_0	BB992310
E_ET_MSV3_27G03_F_0	BB987193
E_ET_MSV3_27G03_R_0	BB992311
E_ET_MSV3_27G04_R_0	BB992312
E_ET_MSV3_27G05_F_0	BB987194
E_ET_MSV3_27G06_F_0	BB987195
E_ET_MSV3_27G06_R_0	BB992313
E_ET_MSV3_27G07_F_0	BB987196

E_ET_MSV3_27G07_R_0	BB992314
E_ET_MSV3_27G08_F_0	BB987197
E_ET_MSV3_27G08_R_0	BB992315
E_ET_MSV3_27G09_F_0	BB987198
E_ET_MSV3_27G10_F_0	BB987199
E_ET_MSV3_27G10_R_0	BB992316
E_ET_MSV3_27G12_F_0	BB987200
E_ET_MSV3_27G12_R_0	BB992317
E_ET_MSV3_27H01_F_0	BB987201
E_ET_MSV3_27H01_R_0	BB992318
E_ET_MSV3_27H02_F_0	BB987202
E_ET_MSV3_27H02_R_0	BB992319
E_ET_MSV3_27H04_R_0	BB992320
E_ET_MSV3_27H05_F_0	BB987203
E_ET_MSV3_27H06_F_0	BB987204
E_ET_MSV3_27H07_F_0	BB987205
E_ET_MSV3_27H07_R_0	BB992321
E_ET_MSV3_27H08_F_0	BB987206
E_ET_MSV3_27H08_R_0	BB992322
E_ET_MSV3_27H09_F_0	BB987207
E_ET_MSV3_27H09_R_0	BB992323
E_ET_MSV3_27H10_F_0	BB987208
E_ET_MSV3_27H10_R_0	BB992324
E_ET_MSV3_27H11_F_0	BB987209
E_ET_MSV3_27H11_R_0	BB992325
E_ET_MSV3_27H12_F_0	BB987210
E_ET_MSV3_27H12_R_0	BB992326
E_ET_MSV3_28A01_F_0	BB987211
E_ET_MSV3_28A01_R_0	BB992327
E_ET_MSV3_28A02_R_0	BB992328
E_ET_MSV3_28A03_F_0	BB987212
E_ET_MSV3_28A03_R_0	BB992329
E_ET_MSV3_28A04_F_0	BB987213
E_ET_MSV3_28A04_R_0	BB992330
E_ET_MSV3_28A05_F_0	BB987214
E_ET_MSV3_28A05_R_0	BB992331
E_ET_MSV3_28A07_F_0	BB987215
E_ET_MSV3_28A07_R_0	BB992332
E_ET_MSV3_28A08_F_0	BB987216
E_ET_MSV3_28A08_R_0	BB992333
E_ET_MSV3_28A09_F_0	BB987217
E_ET_MSV3_28A09_R_0	BB992334
E_ET_MSV3_28A10_F_0	BB987218
E_ET_MSV3_28A10_R_0	BB992335
E_ET_MSV3_28A11_R_0	BB992336
E_ET_MSV3_28A12_F_0	BB987219
E_ET_MSV3_28B01_F_0	BB987220
E_ET_MSV3_28B01_R_0	BB992337
E_ET_MSV3_28B02_R_0	BB992338
E_ET_MSV3_28B04_F_0	BB987221
E_ET_MSV3_28B04_R_0	BB992339
E_ET_MSV3_28B05_F_0	BB987222
E_ET_MSV3_28B05_R_0	BB992340
E_ET_MSV3_28B07_R_0	BB992341

E_ET_MSV3_28B08_R_0	BB992342
E_ET_MSV3_28B09_F_0	BB987223
E_ET_MSV3_28B10_R_0	BB992343
E_ET_MSV3_28B11_F_0	BB987224
E_ET_MSV3_28B12_R_0	BB992344
E_ET_MSV3_28C01_F_0	BB987225
E_ET_MSV3_28C02_F_0	BB987226
E_ET_MSV3_28C03_R_0	BB992345
E_ET_MSV3_28C04_R_0	BB992346
E_ET_MSV3_28C05_F_0	BB987227
E_ET_MSV3_28C05_R_0	BB992347
E_ET_MSV3_28C06_F_0	BB987228
E_ET_MSV3_28C06_R_0	BB992348
E_ET_MSV3_28C07_F_0	BB987229
E_ET_MSV3_28C08_F_0	BB987230
E_ET_MSV3_28C08_R_0	BB992349
E_ET_MSV3_28C09_F_0	BB987231
E_ET_MSV3_28C09_R_0	BB992350
E_ET_MSV3_28C10_F_0	BB987232
E_ET_MSV3_28C11_F_0	BB987233
E_ET_MSV3_28C12_F_0	BB987234
E_ET_MSV3_28C12_R_0	BB992351
E_ET_MSV3_28D01_F_0	BB987235
E_ET_MSV3_28D01_R_0	BB992352
E_ET_MSV3_28D03_F_0	BB987236
E_ET_MSV3_28D03_R_0	BB992353
E_ET_MSV3_28D04_F_0	BB987237
E_ET_MSV3_28D05_R_0	BB992354
E_ET_MSV3_28D09_F_0	BB987238
E_ET_MSV3_28D10_F_0	BB987239
E_ET_MSV3_28D10_R_0	BB992355
E_ET_MSV3_28D11_F_0	BB987240
E_ET_MSV3_28D11_R_0	BB992356
E_ET_MSV3_28D12_R_0	BB992357
E_ET_MSV3_28E01_F_0	BB987241
E_ET_MSV3_28E01_R_0	BB992358
E_ET_MSV3_28E02_F_0	BB987242
E_ET_MSV3_28E02_R_0	BB992359
E_ET_MSV3_28E03_F_0	BB987243
E_ET_MSV3_28E03_R_0	BB992360
E_ET_MSV3_28E05_F_0	BB987244
E_ET_MSV3_28E05_R_0	BB992361
E_ET_MSV3_28E06_F_0	BB987245
E_ET_MSV3_28E06_R_0	BB992362
E_ET_MSV3_28E07_F_0	BB987246
E_ET_MSV3_28E09_F_0	BB987247
E_ET_MSV3_28E09_R_0	BB992363
E_ET_MSV3_28E10_F_0	BB987248
E_ET_MSV3_28E10_R_0	BB992364
E_ET_MSV3_28E11_R_0	BB992365
E_ET_MSV3_28F01_F_0	BB987249
E_ET_MSV3_28F01_R_0	BB992366
E_ET_MSV3_28F03_F_0	BB987250
E_ET_MSV3_28F03_R_0	BB992367

E_ET_MSV3_28F04_R_0	BB992368
E_ET_MSV3_28F05_R_0	BB992369
E_ET_MSV3_28F06_F_0	BB987251
E_ET_MSV3_28F06_R_0	BB992370
E_ET_MSV3_28F07_F_0	BB987252
E_ET_MSV3_28F07_R_0	BB992371
E_ET_MSV3_28F08_F_0	BB987253
E_ET_MSV3_28F08_R_0	BB992372
E_ET_MSV3_28F09_F_0	BB987254
E_ET_MSV3_28F09_R_0	BB992373
E_ET_MSV3_28F10_F_0	BB987255
E_ET_MSV3_28F10_R_0	BB992374
E_ET_MSV3_28F12_F_0	BB987256
E_ET_MSV3_28G01_F_0	BB987257
E_ET_MSV3_28G02_F_0	BB987258
E_ET_MSV3_28G02_R_0	BB992375
E_ET_MSV3_28G03_F_0	BB987259
E_ET_MSV3_28G03_R_0	BB992376
E_ET_MSV3_28G04_F_0	BB987260
E_ET_MSV3_28G04_R_0	BB992377
E_ET_MSV3_28G05_F_0	BB987261
E_ET_MSV3_28G05_R_0	BB992378
E_ET_MSV3_28G06_F_0	BB987262
E_ET_MSV3_28G07_F_0	BB987263
E_ET_MSV3_28G07_R_0	BB992379
E_ET_MSV3_28G08_F_0	BB987264
E_ET_MSV3_28G08_R_0	BB992380
E_ET_MSV3_28G09_F_0	BB987265
E_ET_MSV3_28G09_R_0	BB992381
E_ET_MSV3_28G10_F_0	BB987266
E_ET_MSV3_28G10_R_0	BB992382
E_ET_MSV3_28G11_F_0	BB987267
E_ET_MSV3_28G11_R_0	BB992383
E_ET_MSV3_28G12_R_0	BB992384
E_ET_MSV3_28H01_R_0	BB992385
E_ET_MSV3_28H02_R_0	BB992386
E_ET_MSV3_28H03_F_0	BB987268
E_ET_MSV3_28H03_R_0	BB992387
E_ET_MSV3_28H04_R_0	BB992388
E_ET_MSV3_28H05_R_0	BB992389
E_ET_MSV3_28H06_F_0	BB987269
E_ET_MSV3_28H06_R_0	BB992390
E_ET_MSV3_28H07_F_0	BB987270
E_ET_MSV3_28H07_R_0	BB992391
E_ET_MSV3_28H08_R_0	BB992392
E_ET_MSV3_28H10_F_0	BB987271
E_ET_MSV3_28H10_R_0	BB992393
E_ET_MSV3_28H11_F_0	BB987272
E_ET_MSV3_28H12_F_0	BB987273
E_ET_MSV3_28H12_R_0	BB992394
E_ET_MSV3_29A01_F_0	BB987274
E_ET_MSV3_29A01_R_0	BB992395
E_ET_MSV3_29A02_R_0	BB992396
E_ET_MSV3_29A03_F_0	BB987275

E_ET_MSV3_29A03_R_0	BB992397
E_ET_MSV3_29A04_F_0	BB987276
E_ET_MSV3_29A04_R_0	BB992398
E_ET_MSV3_29A05_F_0	BB987277
E_ET_MSV3_29A06_F_0	BB987278
E_ET_MSV3_29A06_R_0	BB992399
E_ET_MSV3_29A07_F_0	BB987279
E_ET_MSV3_29A07_R_0	BB992400
E_ET_MSV3_29A08_F_0	BB987280
E_ET_MSV3_29A08_R_0	BB992401
E_ET_MSV3_29A09_F_0	BB987281
E_ET_MSV3_29A09_R_0	BB992402
E_ET_MSV3_29A11_F_0	BB987282
E_ET_MSV3_29A11_R_0	BB992403
E_ET_MSV3_29A12_F_0	BB987283
E_ET_MSV3_29A12_R_0	BB992404
E_ET_MSV3_29B01_F_0	BB987284
E_ET_MSV3_29B01_R_0	BB992405
E_ET_MSV3_29B02_F_0	BB987285
E_ET_MSV3_29B02_R_0	BB992406
E_ET_MSV3_29B03_F_0	BB987286
E_ET_MSV3_29B04_F_0	BB987287
E_ET_MSV3_29B04_R_0	BB992407
E_ET_MSV3_29B05_F_0	BB987288
E_ET_MSV3_29B05_R_0	BB992408
E_ET_MSV3_29B07_F_0	BB987289
E_ET_MSV3_29B07_R_0	BB992409
E_ET_MSV3_29B08_F_0	BB987290
E_ET_MSV3_29B08_R_0	BB992410
E_ET_MSV3_29B09_F_0	BB987291
E_ET_MSV3_29B09_R_0	BB992411
E_ET_MSV3_29B11_F_0	BB987292
E_ET_MSV3_29B11_R_0	BB992412
E_ET_MSV3_29C01_F_0	BB987293
E_ET_MSV3_29C01_R_0	BB992413
E_ET_MSV3_29C02_F_0	BB987294
E_ET_MSV3_29C03_F_0	BB987295
E_ET_MSV3_29C03_R_0	BB992414
E_ET_MSV3_29C04_F_0	BB987296
E_ET_MSV3_29C04_R_0	BB992415
E_ET_MSV3_29C05_R_0	BB992416
E_ET_MSV3_29C06_F_0	BB987297
E_ET_MSV3_29C06_R_0	BB992417
E_ET_MSV3_29C07_R_0	BB992418
E_ET_MSV3_29C08_F_0	BB987298
E_ET_MSV3_29C08_R_0	BB992419
E_ET_MSV3_29C09_F_0	BB987299
E_ET_MSV3_29C09_R_0	BB992420
E_ET_MSV3_29C10_F_0	BB987300
E_ET_MSV3_29C10_R_0	BB992421
E_ET_MSV3_29C11_F_0	BB987301
E_ET_MSV3_29C11_R_0	BB992422
E_ET_MSV3_29C12_F_0	BB987302
E_ET_MSV3_29C12_R_0	BB992423

E_ET_MSV3_29D01_F_0	BB987303
E_ET_MSV3_29D01_R_0	BB992424
E_ET_MSV3_29D02_R_0	BB992425
E_ET_MSV3_29D03_F_0	BB987304
E_ET_MSV3_29D03_R_0	BB992426
E_ET_MSV3_29D04_F_0	BB987305
E_ET_MSV3_29D04_R_0	BB992427
E_ET_MSV3_29D05_F_0	BB987306
E_ET_MSV3_29D05_R_0	BB992428
E_ET_MSV3_29D06_F_0	BB987307
E_ET_MSV3_29D08_F_0	BB987308
E_ET_MSV3_29D08_R_0	BB992429
E_ET_MSV3_29D09_F_0	BB987309
E_ET_MSV3_29D09_R_0	BB992430
E_ET_MSV3_29D10_F_0	BB987310
E_ET_MSV3_29D11_F_0	BB987311
E_ET_MSV3_29D11_R_0	BB992431
E_ET_MSV3_29D12_R_0	BB992432
E_ET_MSV3_29E01_F_0	BB987312
E_ET_MSV3_29E01_R_0	BB992433
E_ET_MSV3_29E02_R_0	BB992434
E_ET_MSV3_29E03_R_0	BB992435
E_ET_MSV3_29E04_R_0	BB992436
E_ET_MSV3_29E05_F_0	BB987313
E_ET_MSV3_29E05_R_0	BB992437
E_ET_MSV3_29E06_F_0	BB987314
E_ET_MSV3_29E06_R_0	BB992438
E_ET_MSV3_29E07_F_0	BB987315
E_ET_MSV3_29E07_R_0	BB992439
E_ET_MSV3_29E08_F_0	BB987316
E_ET_MSV3_29E08_R_0	BB992440
E_ET_MSV3_29E09_F_0	BB987317
E_ET_MSV3_29E09_R_0	BB992441
E_ET_MSV3_29E10_F_0	BB987318
E_ET_MSV3_29E11_F_0	BB987319
E_ET_MSV3_29E11_R_0	BB992442
E_ET_MSV3_29E12_F_0	BB987320
E_ET_MSV3_29F01_F_0	BB987321
E_ET_MSV3_29F01_R_0	BB992443
E_ET_MSV3_29F02_F_0	BB987322
E_ET_MSV3_29F02_R_0	BB992444
E_ET_MSV3_29F03_F_0	BB987323
E_ET_MSV3_29F03_R_0	BB992445
E_ET_MSV3_29F04_F_0	BB987324
E_ET_MSV3_29F04_R_0	BB992446
E_ET_MSV3_29F06_F_0	BB987325
E_ET_MSV3_29F06_R_0	BB992447
E_ET_MSV3_29F07_R_0	BB992448
E_ET_MSV3_29F08_R_0	BB992449
E_ET_MSV3_29F09_F_0	BB987326
E_ET_MSV3_29F09_R_0	BB992450
E_ET_MSV3_29F10_F_0	BB987327
E_ET_MSV3_29F11_F_0	BB987328
E_ET_MSV3_29F11_R_0	BB992451

E_ET_MSV3_29F12_F_0	BB987329
E_ET_MSV3_29F12_R_0	BB992452
E_ET_MSV3_29G01_F_0	BB987330
E_ET_MSV3_29G01_R_0	BB992453
E_ET_MSV3_29G02_F_0	BB987331
E_ET_MSV3_29G03_F_0	BB987332
E_ET_MSV3_29G03_R_0	BB992454
E_ET_MSV3_29G05_R_0	BB992455
E_ET_MSV3_29G06_F_0	BB987333
E_ET_MSV3_29G06_R_0	BB992456
E_ET_MSV3_29G08_F_0	BB987334
E_ET_MSV3_29G09_F_0	BB987335
E_ET_MSV3_29G09_R_0	BB992457
E_ET_MSV3_29G10_R_0	BB992458
E_ET_MSV3_29G11_F_0	BB987336
E_ET_MSV3_29G11_R_0	BB992459
E_ET_MSV3_29G12_F_0	BB987337
E_ET_MSV3_29G12_R_0	BB992460
E_ET_MSV3_29H01_F_0	BB987338
E_ET_MSV3_29H02_F_0	BB987339
E_ET_MSV3_29H02_R_0	BB992461
E_ET_MSV3_29H03_F_0	BB987340
E_ET_MSV3_29H03_R_0	BB992462
E_ET_MSV3_29H04_R_0	BB992463
E_ET_MSV3_29H05_F_0	BB987341
E_ET_MSV3_29H07_F_0	BB987342
E_ET_MSV3_29H07_R_0	BB992464
E_ET_MSV3_29H09_F_0	BB987343
E_ET_MSV3_29H09_R_0	BB992465
E_ET_MSV3_29H10_F_0	BB987344
E_ET_MSV3_29H10_R_0	BB992466
E_ET_MSV3_29H12_F_0	BB987345
E_ET_MSV3_29H12_R_0	BB992467
E_ET_MSV3_30A01_F_0	BB987346
E_ET_MSV3_30A02_F_0	BB987347
E_ET_MSV3_30A02_R_0	BB992468
E_ET_MSV3_30A03_F_0	BB987348
E_ET_MSV3_30A03_R_0	BB992469
E_ET_MSV3_30A04_F_0	BB987349
E_ET_MSV3_30A04_R_0	BB992470
E_ET_MSV3_30A05_F_0	BB987350
E_ET_MSV3_30A05_R_0	BB992471
E_ET_MSV3_30A06_F_0	BB987351
E_ET_MSV3_30A06_R_0	BB992472
E_ET_MSV3_30A08_F_0	BB987352
E_ET_MSV3_30A09_R_0	BB992473
E_ET_MSV3_30A10_F_0	BB987353
E_ET_MSV3_30A11_F_0	BB987354
E_ET_MSV3_30A12_F_0	BB987355
E_ET_MSV3_30B01_F_0	BB987356
E_ET_MSV3_30B02_F_0	BB987357
E_ET_MSV3_30B03_F_0	BB987358
E_ET_MSV3_30B04_F_0	BB987359
E_ET_MSV3_30B04_R_0	BB992474

E_ET_MSV3_30B05_F_0	BB987360
E_ET_MSV3_30B08_F_0	BB987361
E_ET_MSV3_30B08_R_0	BB992475
E_ET_MSV3_30B09_F_0	BB987362
E_ET_MSV3_30B09_R_0	BB992476
E_ET_MSV3_30B10_R_0	BB992477
E_ET_MSV3_30B11_F_0	BB987363
E_ET_MSV3_30B11_R_0	BB992478
E_ET_MSV3_30B12_R_0	BB992479
E_ET_MSV3_30C01_F_0	BB987364
E_ET_MSV3_30C02_F_0	BB987365
E_ET_MSV3_30C03_F_0	BB987366
E_ET_MSV3_30C03_R_0	BB992480
E_ET_MSV3_30C04_R_0	BB992481
E_ET_MSV3_30C05_F_0	BB987367
E_ET_MSV3_30C05_R_0	BB992482
E_ET_MSV3_30C06_F_0	BB987368
E_ET_MSV3_30C06_R_0	BB992483
E_ET_MSV3_30C08_F_0	BB987369
E_ET_MSV3_30C08_R_0	BB992484
E_ET_MSV3_30C09_F_0	BB987370
E_ET_MSV3_30C10_F_0	BB987371
E_ET_MSV3_30C10_R_0	BB992485
E_ET_MSV3_30C11_F_0	BB987372
E_ET_MSV3_30C11_R_0	BB992486
E_ET_MSV3_30C12_F_0	BB987373
E_ET_MSV3_30C12_R_0	BB992487
E_ET_MSV3_30D01_R_0	BB992488
E_ET_MSV3_30D02_F_0	BB987374
E_ET_MSV3_30D02_R_0	BB992489
E_ET_MSV3_30D03_F_0	BB987375
E_ET_MSV3_30D03_R_0	BB992490
E_ET_MSV3_30D04_F_0	BB987376
E_ET_MSV3_30D04_R_0	BB992491
E_ET_MSV3_30D05_F_0	BB987377
E_ET_MSV3_30D05_R_0	BB992492
E_ET_MSV3_30D06_F_0	BB987378
E_ET_MSV3_30D06_R_0	BB992493
E_ET_MSV3_30D08_F_0	BB987379
E_ET_MSV3_30D08_R_0	BB992494
E_ET_MSV3_30D09_F_0	BB987380
E_ET_MSV3_30D09_R_0	BB992495
E_ET_MSV3_30D10_F_0	BB987381
E_ET_MSV3_30D10_R_0	BB992496
E_ET_MSV3_30D11_F_0	BB987382
E_ET_MSV3_30D11_R_0	BB992497
E_ET_MSV3_30D12_F_0	BB987383
E_ET_MSV3_30E01_F_0	BB987384
E_ET_MSV3_30E01_R_0	BB992498
E_ET_MSV3_30E02_F_0	BB987385
E_ET_MSV3_30E02_R_0	BB992499
E_ET_MSV3_30E03_F_0	BB987386
E_ET_MSV3_30E04_F_0	BB987387
E_ET_MSV3_30E04_R_0	BB992500

E_ET_MSV3_30E05_F_0	BB987388
E_ET_MSV3_30E05_R_0	BB992501
E_ET_MSV3_30E06_F_0	BB987389
E_ET_MSV3_30E06_R_0	BB992502
E_ET_MSV3_30E07_F_0	BB987390
E_ET_MSV3_30E07_R_0	BB992503
E_ET_MSV3_30E09_F_0	BB987391
E_ET_MSV3_30E09_R_0	BB992504
E_ET_MSV3_30E10_F_0	BB987392
E_ET_MSV3_30E10_R_0	BB992505
E_ET_MSV3_30E11_F_0	BB987393
E_ET_MSV3_30E11_R_0	BB992506
E_ET_MSV3_30E12_F_0	BB987394
E_ET_MSV3_30E12_R_0	BB992507
E_ET_MSV3_30F01_F_0	BB987395
E_ET_MSV3_30F02_F_0	BB987396
E_ET_MSV3_30F02_R_0	BB992508
E_ET_MSV3_30F03_F_0	BB987397
E_ET_MSV3_30F03_R_0	BB992509
E_ET_MSV3_30F04_F_0	BB987398
E_ET_MSV3_30F04_R_0	BB992510
E_ET_MSV3_30F05_F_0	BB987399
E_ET_MSV3_30F05_R_0	BB992511
E_ET_MSV3_30F07_F_0	BB987400
E_ET_MSV3_30F08_F_0	BB987401
E_ET_MSV3_30F09_F_0	BB987402
E_ET_MSV3_30F09_R_0	BB992512
E_ET_MSV3_30F10_F_0	BB987403
E_ET_MSV3_30F12_F_0	BB987404
E_ET_MSV3_30G01_F_0	BB987405
E_ET_MSV3_30G01_R_0	BB992513
E_ET_MSV3_30G03_F_0	BB987406
E_ET_MSV3_30G04_F_0	BB987407
E_ET_MSV3_30G04_R_0	BB992514
E_ET_MSV3_30G05_F_0	BB987408
E_ET_MSV3_30G06_F_0	BB987409
E_ET_MSV3_30G06_R_0	BB992515
E_ET_MSV3_30G07_F_0	BB987410
E_ET_MSV3_30G07_R_0	BB992516
E_ET_MSV3_30G08_F_0	BB987411
E_ET_MSV3_30G09_F_0	BB987412
E_ET_MSV3_30G09_R_0	BB992517
E_ET_MSV3_30G10_F_0	BB987413
E_ET_MSV3_30G10_R_0	BB992518
E_ET_MSV3_30G11_F_0	BB987414
E_ET_MSV3_30G11_R_0	BB992519
E_ET_MSV3_30G12_F_0	BB987415
E_ET_MSV3_30G12_R_0	BB992520
E_ET_MSV3_30H01_F_0	BB987416
E_ET_MSV3_30H01_R_0	BB992521
E_ET_MSV3_30H03_F_0	BB987417
E_ET_MSV3_30H03_R_0	BB992522
E_ET_MSV3_30H04_F_0	BB987418
E_ET_MSV3_30H05_F_0	BB987419

E_ET_MSV3_30H06_F_0	BB987420
E_ET_MSV3_30H06_R_0	BB992523
E_ET_MSV3_30H07_F_0	BB987421
E_ET_MSV3_30H09_F_0	BB987422
E_ET_MSV3_30H09_R_0	BB992524
E_ET_MSV3_30H10_R_0	BB992525
E_ET_MSV3_30H11_F_0	BB987423
E_ET_MSV3_30H12_R_0	BB992526
E_ET_MSV3_31A01_F_0	BB987424
E_ET_MSV3_31A02_R_0	BB992527
E_ET_MSV3_31A03_R_0	BB992528
E_ET_MSV3_31A05_R_0	BB992529
E_ET_MSV3_31A06_R_0	BB992530
E_ET_MSV3_31A07_R_0	BB992531
E_ET_MSV3_31A08_R_0	BB992532
E_ET_MSV3_31A09_R_0	BB992533
E_ET_MSV3_31A10_R_0	BB992534
E_ET_MSV3_31A11_R_0	BB992535
E_ET_MSV3_31B01_F_0	BB987425
E_ET_MSV3_31B01_R_0	BB992536
E_ET_MSV3_31B04_R_0	BB992537
E_ET_MSV3_31B07_R_0	BB992538
E_ET_MSV3_31B09_R_0	BB992539
E_ET_MSV3_31B10_R_0	BB992540
E_ET_MSV3_31B11_R_0	BB992541
E_ET_MSV3_31C01_F_0	BB987426
E_ET_MSV3_31C01_R_0	BB992542
E_ET_MSV3_31C02_F_0	BB987427
E_ET_MSV3_31C02_R_0	BB992543
E_ET_MSV3_31C05_R_0	BB992544
E_ET_MSV3_31C06_R_0	BB992545
E_ET_MSV3_31C07_F_0	BB987428
E_ET_MSV3_31C08_R_0	BB992546
E_ET_MSV3_31C09_F_0	BB987429
E_ET_MSV3_31C10_R_0	BB992547
E_ET_MSV3_31C11_R_0	BB992548
E_ET_MSV3_31C12_R_0	BB992549
E_ET_MSV3_31D01_F_0	BB987430
E_ET_MSV3_31D01_R_0	BB992550
E_ET_MSV3_31D03_R_0	BB992551
E_ET_MSV3_31D05_R_0	BB992552
E_ET_MSV3_31D09_R_0	BB992553
E_ET_MSV3_31E01_F_0	BB987431
E_ET_MSV3_31E01_R_0	BB992554
E_ET_MSV3_31E02_F_0	BB987432
E_ET_MSV3_31E02_R_0	BB992555
E_ET_MSV3_31E03_F_0	BB987433
E_ET_MSV3_31E03_R_0	BB992556
E_ET_MSV3_31E04_F_0	BB987434
E_ET_MSV3_31E04_R_0	BB992557
E_ET_MSV3_31E05_F_0	BB987435
E_ET_MSV3_31E05_R_0	BB992558
E_ET_MSV3_31E06_F_0	BB987436
E_ET_MSV3_31E06_R_0	BB992559

E_ET_MSV3_31E07_F_0	BB987437
E_ET_MSV3_31E07_R_0	BB992560
E_ET_MSV3_31E08_F_0	BB987438
E_ET_MSV3_31E08_R_0	BB992561
E_ET_MSV3_31E09_F_0	BB987439
E_ET_MSV3_31E09_R_0	BB992562
E_ET_MSV3_31E10_R_0	BB992563
E_ET_MSV3_31E11_F_0	BB987440
E_ET_MSV3_31E11_R_0	BB992564
E_ET_MSV3_31F01_F_0	BB987441
E_ET_MSV3_31F01_R_0	BB992565
E_ET_MSV3_31F03_R_0	BB992566
E_ET_MSV3_31F04_R_0	BB992567
E_ET_MSV3_31F06_R_0	BB992568
E_ET_MSV3_31F07_R_0	BB992569
E_ET_MSV3_31G01_F_0	BB987442
E_ET_MSV3_31G03_R_0	BB992570
E_ET_MSV3_31G04_R_0	BB992571
E_ET_MSV3_31G05_R_0	BB992572
E_ET_MSV3_31G06_R_0	BB992573
E_ET_MSV3_31G07_R_0	BB992574
E_ET_MSV3_31G09_R_0	BB992575
E_ET_MSV3_31G11_R_0	BB992576
E_ET_MSV3_31H01_F_0	BB987443
E_ET_MSV3_31H06_R_0	BB992577
E_ET_MSV3_31H09_R_0	BB992578
E_ET_MSV3_32A01_F_0	BB987444
E_ET_MSV3_32A01_R_0	BB992579
E_ET_MSV3_32A02_F_0	BB987445
E_ET_MSV3_32A02_R_0	BB992580
E_ET_MSV3_32A03_R_0	BB992581
E_ET_MSV3_32A04_F_0	BB987446
E_ET_MSV3_32A06_F_0	BB987447
E_ET_MSV3_32A06_R_0	BB992582
E_ET_MSV3_32A07_F_0	BB987448
E_ET_MSV3_32A07_R_0	BB992583
E_ET_MSV3_32A08_F_0	BB987449
E_ET_MSV3_32A09_F_0	BB987450
E_ET_MSV3_32A09_R_0	BB992584
E_ET_MSV3_32A10_F_0	BB987451
E_ET_MSV3_32A10_R_0	BB992585
E_ET_MSV3_32A11_F_0	BB987452
E_ET_MSV3_32A11_R_0	BB992586
E_ET_MSV3_32A12_F_0	BB987453
E_ET_MSV3_32A12_R_0	BB992587
E_ET_MSV3_32B02_F_0	BB987454
E_ET_MSV3_32B03_F_0	BB987455
E_ET_MSV3_32B03_R_0	BB992588
E_ET_MSV3_32B04_F_0	BB987456
E_ET_MSV3_32B04_R_0	BB992589
E_ET_MSV3_32B05_F_0	BB987457
E_ET_MSV3_32B05_R_0	BB992590
E_ET_MSV3_32B06_F_0	BB987458
E_ET_MSV3_32B06_R_0	BB992591

E_ET_MSV3_32B08_F_0	BB987459
E_ET_MSV3_32B09_F_0	BB987460
E_ET_MSV3_32B09_R_0	BB992592
E_ET_MSV3_32B11_F_0	BB987461
E_ET_MSV3_32B11_R_0	BB992593
E_ET_MSV3_32B12_F_0	BB987462
E_ET_MSV3_32B12_R_0	BB992594
E_ET_MSV3_32C01_F_0	BB987463
E_ET_MSV3_32C02_R_0	BB992595
E_ET_MSV3_32C03_F_0	BB987464
E_ET_MSV3_32C03_R_0	BB992596
E_ET_MSV3_32C04_F_0	BB987465
E_ET_MSV3_32C04_R_0	BB992597
E_ET_MSV3_32C05_F_0	BB987466
E_ET_MSV3_32C05_R_0	BB992598
E_ET_MSV3_32C06_F_0	BB987467
E_ET_MSV3_32C06_R_0	BB992599
E_ET_MSV3_32C07_R_0	BB992600
E_ET_MSV3_32C08_F_0	BB987468
E_ET_MSV3_32C08_R_0	BB992601
E_ET_MSV3_32C09_F_0	BB987469
E_ET_MSV3_32C09_R_0	BB992602
E_ET_MSV3_32C10_F_0	BB987470
E_ET_MSV3_32C10_R_0	BB992603
E_ET_MSV3_32C11_F_0	BB987471
E_ET_MSV3_32C11_R_0	BB992604
E_ET_MSV3_32D01_F_0	BB987472
E_ET_MSV3_32D02_F_0	BB987473
E_ET_MSV3_32D02_R_0	BB992605
E_ET_MSV3_32D03_F_0	BB987474
E_ET_MSV3_32D03_R_0	BB992606
E_ET_MSV3_32D04_F_0	BB987475
E_ET_MSV3_32D04_R_0	BB992607
E_ET_MSV3_32D05_F_0	BB987476
E_ET_MSV3_32D05_R_0	BB992608
E_ET_MSV3_32D06_F_0	BB987477
E_ET_MSV3_32D06_R_0	BB992609
E_ET_MSV3_32D07_F_0	BB987478
E_ET_MSV3_32D07_R_0	BB992610
E_ET_MSV3_32D08_F_0	BB987479
E_ET_MSV3_32D08_R_0	BB992611
E_ET_MSV3_32D09_F_0	BB987480
E_ET_MSV3_32D09_R_0	BB992612
E_ET_MSV3_32D10_F_0	BB987481
E_ET_MSV3_32D10_R_0	BB992613
E_ET_MSV3_32D11_F_0	BB987482
E_ET_MSV3_32D11_R_0	BB992614
E_ET_MSV3_32D12_F_0	BB987483
E_ET_MSV3_32E01_F_0	BB987484
E_ET_MSV3_32E01_R_0	BB992615
E_ET_MSV3_32E02_F_0	BB987485
E_ET_MSV3_32E02_R_0	BB992616
E_ET_MSV3_32E05_F_0	BB987486
E_ET_MSV3_32E06_F_0	BB987487

E_ET_MSV3_32E06_R_0	BB992617
E_ET_MSV3_32E07_F_0	BB987488
E_ET_MSV3_32E07_R_0	BB992618
E_ET_MSV3_32E08_F_0	BB987489
E_ET_MSV3_32E08_R_0	BB992619
E_ET_MSV3_32E09_F_0	BB987490
E_ET_MSV3_32E10_F_0	BB987491
E_ET_MSV3_32E10_R_0	BB992620
E_ET_MSV3_32E11_F_0	BB987492
E_ET_MSV3_32E12_F_0	BB987493
E_ET_MSV3_32E12_R_0	BB992621
E_ET_MSV3_32F01_F_0	BB987494
E_ET_MSV3_32F01_R_0	BB992622
E_ET_MSV3_32F03_F_0	BB987495
E_ET_MSV3_32F03_R_0	BB992623
E_ET_MSV3_32F04_F_0	BB987496
E_ET_MSV3_32F04_R_0	BB992624
E_ET_MSV3_32F05_F_0	BB987497
E_ET_MSV3_32F05_R_0	BB992625
E_ET_MSV3_32F06_F_0	BB987498
E_ET_MSV3_32F06_R_0	BB992626
E_ET_MSV3_32F07_F_0	BB987499
E_ET_MSV3_32F09_F_0	BB987500
E_ET_MSV3_32F09_R_0	BB992627
E_ET_MSV3_32F10_F_0	BB987501
E_ET_MSV3_32F10_R_0	BB992628
E_ET_MSV3_32F11_F_0	BB987502
E_ET_MSV3_32F12_F_0	BB987503
E_ET_MSV3_32F12_R_0	BB992629
E_ET_MSV3_32G01_R_0	BB992630
E_ET_MSV3_32G02_F_0	BB987504
E_ET_MSV3_32G02_R_0	BB992631
E_ET_MSV3_32G03_F_0	BB987505
E_ET_MSV3_32G03_R_0	BB992632
E_ET_MSV3_32G04_F_0	BB987506
E_ET_MSV3_32G04_R_0	BB992633
E_ET_MSV3_32G05_F_0	BB987507
E_ET_MSV3_32G05_R_0	BB992634
E_ET_MSV3_32G06_F_0	BB987508
E_ET_MSV3_32G07_F_0	BB987509
E_ET_MSV3_32G08_F_0	BB987510
E_ET_MSV3_32G08_R_0	BB992635
E_ET_MSV3_32G09_F_0	BB987511
E_ET_MSV3_32G10_F_0	BB987512
E_ET_MSV3_32G10_R_0	BB992636
E_ET_MSV3_32G11_F_0	BB987513
E_ET_MSV3_32G11_R_0	BB992637
E_ET_MSV3_32G12_F_0	BB987514
E_ET_MSV3_32H01_F_0	BB987515
E_ET_MSV3_32H01_R_0	BB992638
E_ET_MSV3_32H05_F_0	BB987516
E_ET_MSV3_32H05_R_0	BB992639
E_ET_MSV3_32H06_F_0	BB987517
E_ET_MSV3_32H06_R_0	BB992640

	E_ET_MSV3_32H07_F_0	BB987518
	E_ET_MSV3_32H07_R_0	BB992641
	E_ET_MSV3_32H08_F_0	BB987519
	E_ET_MSV3_32H08_R_0	BB992642
	E_ET_MSV3_32H09_F_0	BB987520
	E_ET_MSV3_32H09_R_0	BB992643
	E_ET_MSV3_32H10_F_0	BB987521
	E_ET_MSV3_32H10_R_0	BB992644
	E_ET_MSV3_32H11_F_0	BB987522
	E_ET_MSV3_32H11_R_0	BB992645
	E_ET_MSV3_32H12_F_0	BB987523
	E_ET_MSV3_32H12_R_0	BB992646
Nnor	E_ET_Nnor_01A04_F_0	BY916644
	E_ET_Nnor_01A06_F_0	BY916645
	E_ET_Nnor_01A07_F_0	BY916646
	E_ET_Nnor_01A08_F_0	BY916647
	E_ET_Nnor_01A09_F_0	BY916648
	E_ET_Nnor_01B06_F_0	BY916649
	E_ET_Nnor_01B10_F_0	BY916650
	E_ET_Nnor_01C02_F_0	BY916651
	E_ET_Nnor_01C07_F_0	BY916652
	E_ET_Nnor_01C09_F_0	BY916653
	E_ET_Nnor_01C10_F_0	BY916654
	E_ET_Nnor_01C12_F_0	BY916655
	E_ET_Nnor_01C12_F_1	BY916656
	E_ET_Nnor_01E06_F_0	BY916657
	E_ET_Nnor_01E07_F_0	BY916658
	E_ET_Nnor_01E11_F_0	BY916659
	E_ET_Nnor_01E12_F_0	BY916660
	E_ET_Nnor_01F02_F_0	BY916661
	E_ET_Nnor_01F03_F_0	BY916662
	E_ET_Nnor_01F05_F_0	BY916663
	E_ET_Nnor_01F09_F_0	BY916664
	E_ET_Nnor_01F10_F_0	BY916665
	E_ET_Nnor_01G03_F_0	BY916666
	E_ET_Nnor_01G04_F_0	BY916667
	E_ET_Nnor_01G09_F_0	BY916668
	E_ET_Nnor_01G10_F_0	BY916669
	E_ET_Nnor_01H01_F_0	BY916670
	E_ET_Nnor_01H02_F_0	BY916671
	E_ET_Nnor_01H04_F_0	BY916672
	E_ET_Nnor_01H05_F_0	BY916673
	E_ET_Nnor_01H08_F_0	BY916674
	E_ET_Nnor_02A04_F_0	BY916675
	E_ET_Nnor_02A10_F_0	BY916676
	E_ET_Nnor_02A12_F_0	BY916677
	E_ET_Nnor_02B01_F_0	BY916678
	E_ET_Nnor_02B02_F_0	BY916679
	E_ET_Nnor_02B03_F_0	BY916680
	E_ET_Nnor_02B04_F_0	BY916681
	E_ET_Nnor_02B06_F_0	BY916682
	E_ET_Nnor_02B08_F_0	BY916683
	E_ET_Nnor_02B09_F_0	BY916684
	E_ET_Nnor_02B10_F_0	BY916685

E_ET_Nnor_02B11_F_0	BY916686
E_ET_Nnor_02B12_F_0	BY916687
E_ET_Nnor_02C01_F_0	BY916688
E_ET_Nnor_02C03_F_0	BY916689
E_ET_Nnor_02C04_F_0	BY916690
E_ET_Nnor_02C05_F_0	BY916691
E_ET_Nnor_02C07_F_0	BY916692
E_ET_Nnor_02C08_F_0	BY916693
E_ET_Nnor_02C09_F_0	BY916694
E_ET_Nnor_02C11_F_0	BY916695
E_ET_Nnor_02C12_F_0	BY916696
E_ET_Nnor_02D01_F_0	BY916697
E_ET_Nnor_02D04_F_0	BY916698
E_ET_Nnor_02D06_F_0	BY916699
E_ET_Nnor_02D08_F_0	BY916700
E_ET_Nnor_02D10_F_0	BY916701
E_ET_Nnor_02D12_F_0	BY916702
E_ET_Nnor_02E02_F_0	BY916703
E_ET_Nnor_02E04_F_0	BY916704
E_ET_Nnor_02E05_F_0	BY916705
E_ET_Nnor_02E07_F_0	BY916706
E_ET_Nnor_02E09_F_0	BY916707
E_ET_Nnor_02E10_F_0	BY916708
E_ET_Nnor_02E11_F_0	BY916709
E_ET_Nnor_02F01_F_0	BY916710
E_ET_Nnor_02F05_F_0	BY916711
E_ET_Nnor_02F06_F_0	BY916712
E_ET_Nnor_02F07_F_0	BY916713
E_ET_Nnor_02F08_F_0	BY916714
E_ET_Nnor_02F09_F_0	BY916715
E_ET_Nnor_02G01_F_0	BY916716
E_ET_Nnor_02G05_F_0	BY916717
E_ET_Nnor_02G07_F_0	BY916718
E_ET_Nnor_02G09_F_0	BY916719
E_ET_Nnor_02G10_F_0	BY916720
E_ET_Nnor_02G11_F_0	BY916721
E_ET_Nnor_02H03_F_0	BY916722
E_ET_Nnor_02H04_F_0	BY916723
E_ET_Nnor_02H05_F_0	BY916724
E_ET_Nnor_02H06_F_0	BY916725
E_ET_Nnor_02H07_F_0	BY916726
E_ET_Nnor_02H08_F_0	BY916727
E_ET_Nnor_03A02_F_0	BY916728
E_ET_Nnor_03A03_F_0	BY916729
E_ET_Nnor_03A04_F_0	BY916730
E_ET_Nnor_03A06_F_0	BY916731
E_ET_Nnor_03A10_F_0	BY916732
E_ET_Nnor_03A12_F_0	BY916733
E_ET_Nnor_03B01_F_0	BY916734
E_ET_Nnor_03B02_F_0	BY916735
E_ET_Nnor_03B06_F_0	BY916736
E_ET_Nnor_03B07_F_0	BY916737
E_ET_Nnor_03B08_F_0	BY916738
E_ET_Nnor_03B09_F_0	BY916739

E_ET_Nnor_03B10_F_0	BY916740
E_ET_Nnor_03B11_F_0	BY916741
E_ET_Nnor_03C01_F_0	BY916742
E_ET_Nnor_03C02_F_0	BY916743
E_ET_Nnor_03C07_F_0	BY916744
E_ET_Nnor_03C09_F_0	BY916745
E_ET_Nnor_03C10_F_0	BY916746
E_ET_Nnor_03C11_F_0	BY916747
E_ET_Nnor_03C12_F_0	BY916748
E_ET_Nnor_03D04_F_0	BY916749
E_ET_Nnor_03D05_F_0	BY916750
E_ET_Nnor_03D06_F_0	BY916751
E_ET_Nnor_03D08_F_0	BY916752
E_ET_Nnor_03D09_F_0	BY916753
E_ET_Nnor_03D10_F_0	BY916754
E_ET_Nnor_03D12_F_0	BY916755
E_ET_Nnor_03E02_F_0	BY916756
E_ET_Nnor_03E06_F_0	BY916757
E_ET_Nnor_03E10_F_0	BY916758
E_ET_Nnor_03E11_F_0	BY916759
E_ET_Nnor_03F01_F_0	BY916760
E_ET_Nnor_03F02_F_0	BY916761
E_ET_Nnor_03F04_F_0	BY916762
E_ET_Nnor_03F07_F_0	BY916763
E_ET_Nnor_03F09_F_0	BY916764
E_ET_Nnor_03G02_F_0	BY916765
E_ET_Nnor_03G03_F_0	BY916766
E_ET_Nnor_03G04_F_0	BY916767
E_ET_Nnor_03G06_F_0	BY916768
E_ET_Nnor_03G07_F_0	BY916769
E_ET_Nnor_03G10_F_0	BY916770
E_ET_Nnor_03G11_F_0	BY916771
E_ET_Nnor_03H01_F_0	BY916772
E_ET_Nnor_03H03_F_0	BY916773
E_ET_Nnor_03H04_F_0	BY916774
E_ET_Nnor_03H07_F_0	BY916775
E_ET_Nnor_04A02_F_0	BY916776
E_ET_Nnor_04A06_F_0	BY916777
E_ET_Nnor_04B06_F_0	BY916778
E_ET_Nnor_04C02_F_0	BY916779
E_ET_Nnor_04C07_F_0	BY916780
E_ET_Nnor_04C09_F_0	BY916781
E_ET_Nnor_04C10_F_0	BY916782
E_ET_Nnor_04D03_F_0	BY916783
E_ET_Nnor_04D05_F_0	BY916784
E_ET_Nnor_04D06_F_0	BY916785
E_ET_Nnor_04D08_F_0	BY916786
E_ET_Nnor_04E03_F_0	BY916787
E_ET_Nnor_04E05_F_0	BY916788
E_ET_Nnor_04E07_F_0	BY916789
E_ET_Nnor_04F05_F_0	BY916790
E_ET_Nnor_04F06_F_0	BY916791
E_ET_Nnor_04G07_F_0	BY916792
E_ET_Nnor_04H03_F_0	BY916793

E_ET_Nnor_05A02_F_0	BY916794
E_ET_Nnor_05A04_F_0	BY916795
E_ET_Nnor_05A05_F_0	BY916796
E_ET_Nnor_05A07_F_0	BY916797
E_ET_Nnor_05A08_F_0	BY916798
E_ET_Nnor_05A09_F_0	BY916799
E_ET_Nnor_05A10_F_0	BY916800
E_ET_Nnor_05A11_F_0	BY916801
E_ET_Nnor_05B05_F_0	BY916802
E_ET_Nnor_05B06_F_0	BY916803
E_ET_Nnor_05B09_F_0	BY916804
E_ET_Nnor_05B10_F_0	BY916805
E_ET_Nnor_05B11_F_0	BY916806
E_ET_Nnor_05B12_F_0	BY916807
E_ET_Nnor_05C02_F_0	BY916808
E_ET_Nnor_05C08_F_0	BY916809
E_ET_Nnor_05C11_F_0	BY916810
E_ET_Nnor_05D03_F_0	BY916811
E_ET_Nnor_05D05_F_0	BY916812
E_ET_Nnor_05D06_F_0	BY916813
E_ET_Nnor_05D07_F_0	BY916814
E_ET_Nnor_05D09_F_0	BY916815
E_ET_Nnor_05D10_F_0	BY916816
E_ET_Nnor_05D11_F_0	BY916817
E_ET_Nnor_05E01_F_0	BY916818
E_ET_Nnor_05E05_F_0	BY916819
E_ET_Nnor_05E06_F_0	BY916820
E_ET_Nnor_05E07_F_0	BY916821
E_ET_Nnor_05E08_F_0	BY916822
E_ET_Nnor_05E10_F_0	BY916823
E_ET_Nnor_05E11_F_0	BY916824
E_ET_Nnor_05E12_F_0	BY916825
E_ET_Nnor_05F01_F_0	BY916826
E_ET_Nnor_05F04_F_0	BY916827
E_ET_Nnor_05F05_F_0	BY916828
E_ET_Nnor_05F06_F_0	BY916829
E_ET_Nnor_05F10_F_0	BY916830
E_ET_Nnor_05F12_F_0	BY916831
E_ET_Nnor_05G01_F_0	BY916832
E_ET_Nnor_05G03_F_0	BY916833
E_ET_Nnor_05G04_F_0	BY916834
E_ET_Nnor_05G05_F_0	BY916835
E_ET_Nnor_05G06_F_0	BY916836
E_ET_Nnor_05G07_F_0	BY916837
E_ET_Nnor_05G08_F_0	BY916838
E_ET_Nnor_05G09_F_0	BY916839
E_ET_Nnor_05G11_F_0	BY916840
E_ET_Nnor_05H01_F_0	BY916841
E_ET_Nnor_05H03_F_0	BY916842
E_ET_Nnor_05H04_F_0	BY916843
E_ET_Nnor_05H05_F_0	BY916844
E_ET_Nnor_05H06_F_0	BY916845
E_ET_Nnor_05H08_F_0	BY916846
E_ET_Nnor_05H10_F_0	BY916847

	E_ET_Nnor_06A04_F_0	BY916848
	E_ET_Nnor_06A05_F_0	BY916849
	E_ET_Nnor_06A06_F_0	BY916850
	E_ET_Nnor_06A07_F_0	BY916851
	E_ET_Nnor_06A08_F_0	BY916852
	E_ET_Nnor_06A09_F_0	BY916853
	E_ET_Nnor_06A12_F_0	BY916854
	E_ET_Nnor_06B01_F_0	BY916855
	E_ET_Nnor_06B03_F_0	BY916856
	E_ET_Nnor_06B04_F_0	BY916857
	E_ET_Nnor_06B05_F_0	BY916858
	E_ET_Nnor_06B06_F_0	BY916859
	E_ET_Nnor_06B08_F_0	BY916860
	E_ET_Nnor_08F07_F_0	BY916861
	E_ET_Nnor_14A07_F_0	BY916862
	E_ET_Nnor_16F12_F_0	BY916863
	E_ET_Nnor_16G01_F_0	BY916864
	E_ET_Nnor_16G02_F_0	BY916865
	E_ET_Nnor_16G03_F_0	BY916866
psgV	E_ET_psgV_01A01_F_0	BB987524
	E_ET_psgV_01A03_F_0	BB987525
	E_ET_psgV_01A04_F_0	BB987526
	E_ET_psgV_01A04_R_0	BB992647
	E_ET_psgV_01A05_F_0	BB987527
	E_ET_psgV_01A05_R_0	BB992648
	E_ET_psgV_01A06_F_0	BB987528
	E_ET_psgV_01A06_R_0	BB992649
	E_ET_psgV_01A07_F_0	BB987529
	E_ET_psgV_01A07_R_0	BB992650
	E_ET_psgV_01A09_F_0	BB987530
	E_ET_psgV_01A09_R_0	BB992651
	E_ET_psgV_01A11_F_0	BB987531
	E_ET_psgV_01A11_R_0	BB992652
	E_ET_psgV_01A12_F_0	BB987532
	E_ET_psgV_01A12_R_0	BB992653
	E_ET_psgV_01B01_R_0	BB992654
	E_ET_psgV_01B02_F_0	BB987533
	E_ET_psgV_01B02_R_0	BB992655
	E_ET_psgV_01B03_F_0	BB987534
	E_ET_psgV_01B04_F_0	BB987535
	E_ET_psgV_01B04_R_0	BB992656
	E_ET_psgV_01B05_F_0	BB987536
	E_ET_psgV_01B05_R_0	BB992657
	E_ET_psgV_01B06_F_0	BB987537
	E_ET_psgV_01B07_F_0	BB987538
	E_ET_psgV_01B07_R_0	BB992658
	E_ET_psgV_01B08_F_0	BB987539
	E_ET_psgV_01B08_R_0	BB992659
	E_ET_psgV_01B09_F_0	BB987540
	E_ET_psgV_01B09_R_0	BB992660
	E_ET_psgV_01B10_F_0	BB987541
	E_ET_psgV_01B10_R_0	BB992661
	E_ET_psgV_01B11_F_0	BB987542
	E_ET_psgV_01B11_R_0	BB992662

E_ET_psgV_01B12_F_0	BB987543
E_ET_psgV_01B12_R_0	BB992663
E_ET_psgV_01C01_F_0	BB987544
E_ET_psgV_01C01_R_0	BB992664
E_ET_psgV_01C02_F_0	BB987545
E_ET_psgV_01C02_R_0	BB992665
E_ET_psgV_01C03_F_0	BB987546
E_ET_psgV_01C03_R_0	BB992666
E_ET_psgV_01C04_F_0	BB987547
E_ET_psgV_01C04_R_0	BB992667
E_ET_psgV_01C05_R_0	BB992668
E_ET_psgV_01C06_F_0	BB987548
E_ET_psgV_01C06_R_0	BB992669
E_ET_psgV_01C07_R_0	BB992670
E_ET_psgV_01C09_F_0	BB987549
E_ET_psgV_01C09_R_0	BB992671
E_ET_psgV_01C10_F_0	BB987550
E_ET_psgV_01C10_R_0	BB992672
E_ET_psgV_01C11_F_0	BB987551
E_ET_psgV_01C11_R_0	BB992673
E_ET_psgV_01C12_F_0	BB987552
E_ET_psgV_01D01_F_0	BB987553
E_ET_psgV_01D01_R_0	BB992674
E_ET_psgV_01D03_F_0	BB987554
E_ET_psgV_01D03_R_0	BB992675
E_ET_psgV_01D04_F_0	BB987555
E_ET_psgV_01D05_F_0	BB987556
E_ET_psgV_01D05_R_0	BB992676
E_ET_psgV_01D06_F_0	BB987557
E_ET_psgV_01D07_F_0	BB987558
E_ET_psgV_01D07_R_0	BB992677
E_ET_psgV_01D08_F_0	BB987559
E_ET_psgV_01D08_R_0	BB992678
E_ET_psgV_01D09_F_0	BB987560
E_ET_psgV_01D09_R_0	BB992679
E_ET_psgV_01D10_F_0	BB987561
E_ET_psgV_01D10_R_0	BB992680
E_ET_psgV_01D11_F_0	BB987562
E_ET_psgV_01D11_R_0	BB992681
E_ET_psgV_01D12_F_0	BB987563
E_ET_psgV_01D12_R_0	BB992682
E_ET_psgV_01E01_F_0	BB987564
E_ET_psgV_01E02_F_0	BB987565
E_ET_psgV_01E02_R_0	BB992683
E_ET_psgV_01E03_F_0	BB987566
E_ET_psgV_01E03_R_0	BB992684
E_ET_psgV_01E04_F_0	BB987567
E_ET_psgV_01E04_R_0	BB992685
E_ET_psgV_01E05_F_0	BB987568
E_ET_psgV_01E05_R_0	BB992686
E_ET_psgV_01E06_R_0	BB992687
E_ET_psgV_01E07_R_0	BB992688
E_ET_psgV_01E08_R_0	BB992689
E_ET_psgV_01E09_F_0	BB987569

E_ET_psgV_01E09_R_0	BB992690
E_ET_psgV_01E10_F_0	BB987570
E_ET_psgV_01E10_R_0	BB992691
E_ET_psgV_01E11_F_0	BB987571
E_ET_psgV_01E11_R_0	BB992692
E_ET_psgV_01E12_F_0	BB987572
E_ET_psgV_01E12_R_0	BB992693
E_ET_psgV_01F02_F_0	BB987573
E_ET_psgV_01F03_F_0	BB987574
E_ET_psgV_01F03_R_0	BB992694
E_ET_psgV_01F04_F_0	BB987575
E_ET_psgV_01F05_F_0	BB987576
E_ET_psgV_01F06_F_0	BB987577
E_ET_psgV_01F06_R_0	BB992695
E_ET_psgV_01F07_F_0	BB987578
E_ET_psgV_01F07_R_0	BB992696
E_ET_psgV_01F08_F_0	BB987579
E_ET_psgV_01F08_R_0	BB992697
E_ET_psgV_01F09_F_0	BB987580
E_ET_psgV_01F10_F_0	BB987581
E_ET_psgV_01F10_R_0	BB992698
E_ET_psgV_01F11_F_0	BB987582
E_ET_psgV_01F11_R_0	BB992699
E_ET_psgV_01F12_F_0	BB987583
E_ET_psgV_01F12_R_0	BB992700
E_ET_psgV_01G01_F_0	BB987584
E_ET_psgV_01G01_R_0	BB992701
E_ET_psgV_01G02_F_0	BB987585
E_ET_psgV_01G02_R_0	BB992702
E_ET_psgV_01G03_F_0	BB987586
E_ET_psgV_01G03_R_0	BB992703
E_ET_psgV_01G04_F_0	BB987587
E_ET_psgV_01G04_R_0	BB992704
E_ET_psgV_01G08_F_0	BB987588
E_ET_psgV_01G08_R_0	BB992705
E_ET_psgV_01G09_F_0	BB987589
E_ET_psgV_01G09_R_0	BB992706
E_ET_psgV_01G10_F_0	BB987590
E_ET_psgV_01G10_R_0	BB992707
E_ET_psgV_01G11_F_0	BB987591
E_ET_psgV_01G11_R_0	BB992708
E_ET_psgV_01G12_F_0	BB987592
E_ET_psgV_01G12_R_0	BB992709
E_ET_psgV_01H01_F_0	BB987593
E_ET_psgV_01H01_R_0	BB992710
E_ET_psgV_01H02_F_0	BB987594
E_ET_psgV_01H02_R_0	BB992711
E_ET_psgV_01H04_F_0	BB987595
E_ET_psgV_01H04_R_0	BB992712
E_ET_psgV_01H05_F_0	BB987596
E_ET_psgV_01H05_R_0	BB992713
E_ET_psgV_01H06_R_0	BB992714
E_ET_psgV_01H07_F_0	BB987597
E_ET_psgV_01H08_F_0	BB987598

E_ET_psgV_01H08_R_0	BB992715
E_ET_psgV_01H09_F_0	BB987599
E_ET_psgV_01H09_R_0	BB992716
E_ET_psgV_01H10_F_0	BB987600
E_ET_psgV_01H10_R_0	BB992717
E_ET_psgV_01H11_F_0	BB987601
E_ET_psgV_01H11_R_0	BB992718
E_ET_psgV_01H12_F_0	BB987602
E_ET_psgV_01H12_R_0	BB992719
E_ET_psgV_02A01_F_0	BB987603
E_ET_psgV_02A02_F_0	BB987604
E_ET_psgV_02A02_R_0	BB992720
E_ET_psgV_02A03_F_0	BB987605
E_ET_psgV_02A04_F_0	BB987606
E_ET_psgV_02A05_F_0	BB987607
E_ET_psgV_02A05_R_0	BB992721
E_ET_psgV_02A06_F_0	BB987608
E_ET_psgV_02A08_F_0	BB987609
E_ET_psgV_02A09_F_0	BB987610
E_ET_psgV_02A09_R_0	BB992722
E_ET_psgV_02A10_F_0	BB987611
E_ET_psgV_02A10_R_0	BB992723
E_ET_psgV_02A11_F_0	BB987612
E_ET_psgV_02A11_R_0	BB992724
E_ET_psgV_02A12_F_0	BB987613
E_ET_psgV_02B01_F_0	BB987614
E_ET_psgV_02B01_R_0	BB992725
E_ET_psgV_02B02_F_0	BB987615
E_ET_psgV_02B02_R_0	BB992726
E_ET_psgV_02B03_F_0	BB987616
E_ET_psgV_02B03_R_0	BB992727
E_ET_psgV_02B05_F_0	BB987617
E_ET_psgV_02B05_R_0	BB992728
E_ET_psgV_02B06_F_0	BB987618
E_ET_psgV_02B07_F_0	BB987619
E_ET_psgV_02B07_R_0	BB992729
E_ET_psgV_02B08_F_0	BB987620
E_ET_psgV_02B08_R_0	BB992730
E_ET_psgV_02B09_F_0	BB987621
E_ET_psgV_02B09_R_0	BB992731
E_ET_psgV_02B10_F_0	BB987622
E_ET_psgV_02B10_R_0	BB992732
E_ET_psgV_02B11_F_0	BB987623
E_ET_psgV_02B11_R_0	BB992733
E_ET_psgV_02B12_F_0	BB987624
E_ET_psgV_02B12_R_0	BB992734
E_ET_psgV_02C01_F_0	BB987625
E_ET_psgV_02C01_R_0	BB992735
E_ET_psgV_02C02_F_0	BB987626
E_ET_psgV_02C02_R_0	BB992736
E_ET_psgV_02C03_F_0	BB987627
E_ET_psgV_02C03_R_0	BB992737
E_ET_psgV_02C04_F_0	BB987628
E_ET_psgV_02C04_R_0	BB992738

E_ET_psgV_02C05_F_0	BB987629
E_ET_psgV_02C05_R_0	BB992739
E_ET_psgV_02C06_F_0	BB987630
E_ET_psgV_02C07_F_0	BB987631
E_ET_psgV_02C07_R_0	BB992740
E_ET_psgV_02C08_F_0	BB987632
E_ET_psgV_02C08_R_0	BB992741
E_ET_psgV_02C09_F_0	BB987633
E_ET_psgV_02C09_R_0	BB992742
E_ET_psgV_02C10_F_0	BB987634
E_ET_psgV_02C10_R_0	BB992743
E_ET_psgV_02C12_F_0	BB987635
E_ET_psgV_02D01_F_0	BB987636
E_ET_psgV_02D01_R_0	BB992744
E_ET_psgV_02D02_F_0	BB987637
E_ET_psgV_02D02_R_0	BB992745
E_ET_psgV_02D04_F_0	BB987638
E_ET_psgV_02D05_F_0	BB987639
E_ET_psgV_02D06_F_0	BB987640
E_ET_psgV_02D06_R_0	BB992746
E_ET_psgV_02D07_F_0	BB987641
E_ET_psgV_02D07_R_0	BB992747
E_ET_psgV_02D08_F_0	BB987642
E_ET_psgV_02D08_R_0	BB992748
E_ET_psgV_02D09_F_0	BB987643
E_ET_psgV_02D09_R_0	BB992749
E_ET_psgV_02D10_F_0	BB987644
E_ET_psgV_02D10_R_0	BB992750
E_ET_psgV_02D11_F_0	BB987645
E_ET_psgV_02D11_R_0	BB992751
E_ET_psgV_02E01_F_0	BB987646
E_ET_psgV_02E01_R_0	BB992752
E_ET_psgV_02E02_F_0	BB987647
E_ET_psgV_02E02_R_0	BB992753
E_ET_psgV_02E03_F_0	BB987648
E_ET_psgV_02E03_R_0	BB992754
E_ET_psgV_02E04_F_0	BB987649
E_ET_psgV_02E04_R_0	BB992755
E_ET_psgV_02E05_F_0	BB987650
E_ET_psgV_02E05_R_0	BB992756
E_ET_psgV_02E06_F_0	BB987651
E_ET_psgV_02E07_F_0	BB987652
E_ET_psgV_02E07_R_0	BB992757
E_ET_psgV_02E08_F_0	BB987653
E_ET_psgV_02E08_R_0	BB992758
E_ET_psgV_02E09_F_0	BB987654
E_ET_psgV_02E09_R_0	BB992759
E_ET_psgV_02E10_F_0	BB987655
E_ET_psgV_02E10_R_0	BB992760
E_ET_psgV_02E11_F_0	BB987656
E_ET_psgV_02E12_F_0	BB987657
E_ET_psgV_02E12_R_0	BB992761
E_ET_psgV_02F01_F_0	BB987658
E_ET_psgV_02F01_R_0	BB992762

E_ET_psgV_02F02_F_0	BB987659
E_ET_psgV_02F02_R_0	BB992763
E_ET_psgV_02F03_F_0	BB987660
E_ET_psgV_02F03_R_0	BB992764
E_ET_psgV_02F04_F_0	BB987661
E_ET_psgV_02F04_R_0	BB992765
E_ET_psgV_02F05_F_0	BB987662
E_ET_psgV_02F05_R_0	BB992766
E_ET_psgV_02F06_F_0	BB987663
E_ET_psgV_02F06_R_0	BB992767
E_ET_psgV_02F08_F_0	BB987664
E_ET_psgV_02F08_R_0	BB992768
E_ET_psgV_02F09_F_0	BB987665
E_ET_psgV_02F09_R_0	BB992769
E_ET_psgV_02F10_F_0	BB987666
E_ET_psgV_02F11_F_0	BB987667
E_ET_psgV_02F11_R_0	BB992770
E_ET_psgV_02F12_F_0	BB987668
E_ET_psgV_02F12_R_0	BB992771
E_ET_psgV_02G01_F_0	BB987669
E_ET_psgV_02G01_R_0	BB992772
E_ET_psgV_02G02_F_0	BB987670
E_ET_psgV_02G02_R_0	BB992773
E_ET_psgV_02G03_F_0	BB987671
E_ET_psgV_02G03_R_0	BB992774
E_ET_psgV_02G04_F_0	BB987672
E_ET_psgV_02G05_F_0	BB987673
E_ET_psgV_02G05_R_0	BB992775
E_ET_psgV_02G06_F_0	BB987674
E_ET_psgV_02G08_F_0	BB987675
E_ET_psgV_02G09_F_0	BB987676
E_ET_psgV_02G09_R_0	BB992776
E_ET_psgV_02G10_F_0	BB987677
E_ET_psgV_02G11_F_0	BB987678
E_ET_psgV_02G11_R_0	BB992777
E_ET_psgV_02G12_F_0	BB987679
E_ET_psgV_02H01_F_0	BB987680
E_ET_psgV_02H02_F_0	BB987681
E_ET_psgV_02H03_F_0	BB987682
E_ET_psgV_02H03_R_0	BB992778
E_ET_psgV_02H04_F_0	BB987683
E_ET_psgV_02H04_R_0	BB992779
E_ET_psgV_02H05_F_0	BB987684
E_ET_psgV_02H05_R_0	BB992780
E_ET_psgV_02H06_F_0	BB987685
E_ET_psgV_02H06_R_0	BB992781
E_ET_psgV_02H07_F_0	BB987686
E_ET_psgV_02H07_R_0	BB992782
E_ET_psgV_02H08_F_0	BB987687
E_ET_psgV_02H08_R_0	BB992783
E_ET_psgV_02H09_F_0	BB987688
E_ET_psgV_02H09_R_0	BB992784
E_ET_psgV_02H10_F_0	BB987689
E_ET_psgV_02H10_R_0	BB992785

E_ET_psgV_02H11_R_0	BB992786
E_ET_psgV_02H12_F_0	BB987690
E_ET_psgV_03A01_F_0	BB987691
E_ET_psgV_03A01_R_0	BB992787
E_ET_psgV_03A02_F_0	BB987692
E_ET_psgV_03A02_R_0	BB992788
E_ET_psgV_03A03_F_0	BB987693
E_ET_psgV_03A03_R_0	BB992789
E_ET_psgV_03A04_F_0	BB987694
E_ET_psgV_03A04_R_0	BB992790
E_ET_psgV_03A05_F_0	BB987695
E_ET_psgV_03A05_R_0	BB992791
E_ET_psgV_03A06_R_0	BB992792
E_ET_psgV_03A07_F_0	BB987696
E_ET_psgV_03A07_R_0	BB992793
E_ET_psgV_03A08_R_0	BB992794
E_ET_psgV_03A11_R_0	BB992795
E_ET_psgV_03B01_F_0	BB987697
E_ET_psgV_03B01_R_0	BB992796
E_ET_psgV_03B02_F_0	BB987698
E_ET_psgV_03B02_R_0	BB992797
E_ET_psgV_03B03_F_0	BB987699
E_ET_psgV_03B05_F_0	BB987700
E_ET_psgV_03B07_F_0	BB987701
E_ET_psgV_03B07_R_0	BB992798
E_ET_psgV_03B08_F_0	BB987702
E_ET_psgV_03B08_R_0	BB992799
E_ET_psgV_03B09_F_0	BB987703
E_ET_psgV_03B09_R_0	BB992800
E_ET_psgV_03B10_F_0	BB987704
E_ET_psgV_03B10_R_0	BB992801
E_ET_psgV_03B11_F_0	BB987705
E_ET_psgV_03C02_F_0	BB987706
E_ET_psgV_03C04_F_0	BB987707
E_ET_psgV_03C04_R_0	BB992802
E_ET_psgV_03C05_F_0	BB987708
E_ET_psgV_03C05_R_0	BB992803
E_ET_psgV_03C06_F_0	BB987709
E_ET_psgV_03C07_F_0	BB987710
E_ET_psgV_03C07_R_0	BB992804
E_ET_psgV_03C08_F_0	BB987711
E_ET_psgV_03C09_F_0	BB987712
E_ET_psgV_03C09_R_0	BB992805
E_ET_psgV_03C10_F_0	BB987713
E_ET_psgV_03C11_F_0	BB987714
E_ET_psgV_03C11_R_0	BB992806
E_ET_psgV_03D01_R_0	BB992807
E_ET_psgV_03D02_F_0	BB987715
E_ET_psgV_03D02_R_0	BB992808
E_ET_psgV_03D03_R_0	BB992809
E_ET_psgV_03D04_R_0	BB992810
E_ET_psgV_03D05_F_0	BB987716
E_ET_psgV_03D06_F_0	BB987717
E_ET_psgV_03D06_R_0	BB992811

E_ET_psgV_03D07_R_0	BB992812
E_ET_psgV_03D09_R_0	BB992813
E_ET_psgV_03D10_F_0	BB987718
E_ET_psgV_03D11_F_0	BB987719
E_ET_psgV_03D11_R_0	BB992814
E_ET_psgV_03E03_F_0	BB987720
E_ET_psgV_03E03_R_0	BB992815
E_ET_psgV_03E04_R_0	BB992816
E_ET_psgV_03E05_R_0	BB992817
E_ET_psgV_03E06_R_0	BB992818
E_ET_psgV_03E07_F_0	BB987721
E_ET_psgV_03E08_F_0	BB987722
E_ET_psgV_03E08_R_0	BB992819
E_ET_psgV_03E09_R_0	BB992820
E_ET_psgV_03E11_F_0	BB987723
E_ET_psgV_03E11_R_0	BB992821
E_ET_psgV_03F01_F_0	BB987724
E_ET_psgV_03F01_R_0	BB992822
E_ET_psgV_03F02_F_0	BB987725
E_ET_psgV_03F02_R_0	BB992823
E_ET_psgV_03F03_F_0	BB987726
E_ET_psgV_03F04_F_0	BB987727
E_ET_psgV_03F04_R_0	BB992824
E_ET_psgV_03F05_F_0	BB987728
E_ET_psgV_03F06_F_0	BB987729
E_ET_psgV_03F06_R_0	BB992825
E_ET_psgV_03F08_F_0	BB987730
E_ET_psgV_03F08_R_0	BB992826
E_ET_psgV_03F09_F_0	BB987731
E_ET_psgV_03F09_R_0	BB992827
E_ET_psgV_03F10_F_0	BB987732
E_ET_psgV_03F10_R_0	BB992828
E_ET_psgV_03F11_F_0	BB987733
E_ET_psgV_03G01_F_0	BB987734
E_ET_psgV_03G01_R_0	BB992829
E_ET_psgV_03G02_F_0	BB987735
E_ET_psgV_03G02_R_0	BB992830
E_ET_psgV_03G03_R_0	BB992831
E_ET_psgV_03G04_F_0	BB987736
E_ET_psgV_03G04_R_0	BB992832
E_ET_psgV_03G05_F_0	BB987737
E_ET_psgV_03G05_R_0	BB992833
E_ET_psgV_03G08_F_0	BB987738
E_ET_psgV_03G08_R_0	BB992834
E_ET_psgV_03G09_F_0	BB987739
E_ET_psgV_03G09_R_0	BB992835
E_ET_psgV_03G10_F_0	BB987740
E_ET_psgV_03G10_R_0	BB992836
E_ET_psgV_03G11_F_0	BB987741
E_ET_psgV_03G11_R_0	BB992837
E_ET_psgV_03H01_F_0	BB987742
E_ET_psgV_03H01_R_0	BB992838
E_ET_psgV_03H03_R_0	BB992839
E_ET_psgV_03H05_R_0	BB992840

E_ET_psgV_03H06_R_0	BB992841
E_ET_psgV_03H09_R_0	BB992842
E_ET_psgV_03H10_R_0	BB992843
E_ET_psgV_03H11_R_0	BB992844
E_ET_psgV_04A01_F_0	BB987743
E_ET_psgV_04A01_R_0	BB992845
E_ET_psgV_04A02_F_0	BB987744
E_ET_psgV_04A02_R_0	BB992846
E_ET_psgV_04A03_F_0	BB987745
E_ET_psgV_04A05_F_0	BB987746
E_ET_psgV_04A05_R_0	BB992847
E_ET_psgV_04A06_F_0	BB987747
E_ET_psgV_04A06_R_0	BB992848
E_ET_psgV_04A07_F_0	BB987748
E_ET_psgV_04A07_R_0	BB992849
E_ET_psgV_04A08_F_0	BB987749
E_ET_psgV_04A09_F_0	BB987750
E_ET_psgV_04A09_R_0	BB992850
E_ET_psgV_04A10_F_0	BB987751
E_ET_psgV_04A10_R_0	BB992851
E_ET_psgV_04A11_F_0	BB987752
E_ET_psgV_04A12_R_0	BB992852
E_ET_psgV_04B01_F_0	BB987753
E_ET_psgV_04B01_R_0	BB992853
E_ET_psgV_04B02_F_0	BB987754
E_ET_psgV_04B02_R_0	BB992854
E_ET_psgV_04B03_F_0	BB987755
E_ET_psgV_04B04_F_0	BB987756
E_ET_psgV_04B04_R_0	BB992855
E_ET_psgV_04B05_F_0	BB987757
E_ET_psgV_04B05_R_0	BB992856
E_ET_psgV_04B07_F_0	BB987758
E_ET_psgV_04B07_R_0	BB992857
E_ET_psgV_04B09_F_0	BB987759
E_ET_psgV_04B09_R_0	BB992858
E_ET_psgV_04B10_F_0	BB987760
E_ET_psgV_04B10_R_0	BB992859
E_ET_psgV_04B11_F_0	BB987761
E_ET_psgV_04B12_R_0	BB992860
E_ET_psgV_04C01_F_0	BB987762
E_ET_psgV_04C01_R_0	BB992861
E_ET_psgV_04C02_F_0	BB987763
E_ET_psgV_04C02_R_0	BB992862
E_ET_psgV_04C03_F_0	BB987764
E_ET_psgV_04C03_R_0	BB992863
E_ET_psgV_04C04_F_0	BB987765
E_ET_psgV_04C04_R_0	BB992864
E_ET_psgV_04C05_F_0	BB987766
E_ET_psgV_04C06_F_0	BB987767
E_ET_psgV_04C06_R_0	BB992865
E_ET_psgV_04C07_F_0	BB987768
E_ET_psgV_04C09_F_0	BB987769
E_ET_psgV_04C09_R_0	BB992866
E_ET_psgV_04C10_F_0	BB987770

E_ET_psgV_04C11_F_0	BB987771
E_ET_psgV_04C12_F_0	BB987772
E_ET_psgV_04D01_F_0	BB987773
E_ET_psgV_04D01_R_0	BB992867
E_ET_psgV_04D02_F_0	BB987774
E_ET_psgV_04D02_R_0	BB992868
E_ET_psgV_04D03_F_0	BB987775
E_ET_psgV_04D03_R_0	BB992869
E_ET_psgV_04D04_F_0	BB987776
E_ET_psgV_04D04_R_0	BB992870
E_ET_psgV_04D05_F_0	BB987777
E_ET_psgV_04D05_R_0	BB992871
E_ET_psgV_04D06_F_0	BB987778
E_ET_psgV_04D06_R_0	BB992872
E_ET_psgV_04D07_F_0	BB987779
E_ET_psgV_04D07_R_0	BB992873
E_ET_psgV_04D08_F_0	BB987780
E_ET_psgV_04D09_F_0	BB987781
E_ET_psgV_04D09_R_0	BB992874
E_ET_psgV_04D10_F_0	BB987782
E_ET_psgV_04D11_F_0	BB987783
E_ET_psgV_04D11_R_0	BB992875
E_ET_psgV_04D12_F_0	BB987784
E_ET_psgV_04D12_R_0	BB992876
E_ET_psgV_04E01_F_0	BB987785
E_ET_psgV_04E01_R_0	BB992877
E_ET_psgV_04E02_F_0	BB987786
E_ET_psgV_04E02_R_0	BB992878
E_ET_psgV_04E04_F_0	BB987787
E_ET_psgV_04E04_R_0	BB992879
E_ET_psgV_04E05_F_0	BB987788
E_ET_psgV_04E05_R_0	BB992880
E_ET_psgV_04E06_F_0	BB987789
E_ET_psgV_04E06_R_0	BB992881
E_ET_psgV_04E07_F_0	BB987790
E_ET_psgV_04E08_F_0	BB987791
E_ET_psgV_04E08_R_0	BB992882
E_ET_psgV_04E09_F_0	BB987792
E_ET_psgV_04E09_R_0	BB992883
E_ET_psgV_04E10_F_0	BB987793
E_ET_psgV_04E10_R_0	BB992884
E_ET_psgV_04E11_F_0	BB987794
E_ET_psgV_04E11_R_0	BB992885
E_ET_psgV_04E12_F_0	BB987795
E_ET_psgV_04F01_F_0	BB987796
E_ET_psgV_04F01_R_0	BB992886
E_ET_psgV_04F02_F_0	BB987797
E_ET_psgV_04F02_R_0	BB992887
E_ET_psgV_04F04_F_0	BB987798
E_ET_psgV_04F04_R_0	BB992888
E_ET_psgV_04F05_F_0	BB987799
E_ET_psgV_04F05_R_0	BB992889
E_ET_psgV_04F06_R_0	BB992890
E_ET_psgV_04F07_F_0	BB987800

E_ET_psgV_04F08_F_0	BB987801
E_ET_psgV_04F08_R_0	BB992891
E_ET_psgV_04F09_F_0	BB987802
E_ET_psgV_04F09_R_0	BB992892
E_ET_psgV_04F10_F_0	BB987803
E_ET_psgV_04F10_R_0	BB992893
E_ET_psgV_04F11_F_0	BB987804
E_ET_psgV_04F12_R_0	BB992894
E_ET_psgV_04G01_F_0	BB987805
E_ET_psgV_04G01_R_0	BB992895
E_ET_psgV_04G02_F_0	BB987806
E_ET_psgV_04G02_R_0	BB992896
E_ET_psgV_04G03_F_0	BB987807
E_ET_psgV_04G03_R_0	BB992897
E_ET_psgV_04G04_F_0	BB987808
E_ET_psgV_04G04_R_0	BB992898
E_ET_psgV_04G05_F_0	BB987809
E_ET_psgV_04G06_F_0	BB987810
E_ET_psgV_04G06_R_0	BB992899
E_ET_psgV_04G07_F_0	BB987811
E_ET_psgV_04G07_R_0	BB992900
E_ET_psgV_04G08_F_0	BB987812
E_ET_psgV_04G08_R_0	BB992901
E_ET_psgV_04G09_F_0	BB987813
E_ET_psgV_04G09_R_0	BB992902
E_ET_psgV_04G11_F_0	BB987814
E_ET_psgV_04G11_R_0	BB992903
E_ET_psgV_04H01_F_0	BB987815
E_ET_psgV_04H03_F_0	BB987816
E_ET_psgV_04H03_R_0	BB992904
E_ET_psgV_04H04_F_0	BB987817
E_ET_psgV_04H05_F_0	BB987818
E_ET_psgV_04H06_F_0	BB987819
E_ET_psgV_04H07_F_0	BB987820
E_ET_psgV_04H07_R_0	BB992905
E_ET_psgV_04H08_F_0	BB987821
E_ET_psgV_04H09_F_0	BB987822
E_ET_psgV_04H10_F_0	BB987823
E_ET_psgV_04H10_R_0	BB992906
E_ET_psgV_04H12_F_0	BB987824
E_ET_psgV_04H12_R_0	BB992907
E_ET_psgV_05A01_F_0	BB987825
E_ET_psgV_05A01_R_0	BB992908
E_ET_psgV_05A02_F_0	BB987826
E_ET_psgV_05A03_F_0	BB987827
E_ET_psgV_05A03_R_0	BB992909
E_ET_psgV_05A04_F_0	BB987828
E_ET_psgV_05A04_R_0	BB992910
E_ET_psgV_05A05_F_0	BB987829
E_ET_psgV_05A05_R_0	BB992911
E_ET_psgV_05A06_F_0	BB987830
E_ET_psgV_05A06_R_0	BB992912
E_ET_psgV_05A08_R_0	BB992913
E_ET_psgV_05A09_F_0	BB987831

E_ET_psgV_05A10_F_0	BB987832
E_ET_psgV_05A10_R_0	BB992914
E_ET_psgV_05A11_F_0	BB987833
E_ET_psgV_05A11_R_0	BB992915
E_ET_psgV_05A12_F_0	BB987834
E_ET_psgV_05A12_R_0	BB992916
E_ET_psgV_05B01_F_0	BB987835
E_ET_psgV_05B01_R_0	BB992917
E_ET_psgV_05B02_F_0	BB987836
E_ET_psgV_05B02_R_0	BB992918
E_ET_psgV_05B03_F_0	BB987837
E_ET_psgV_05B03_R_0	BB992919
E_ET_psgV_05B04_F_0	BB987838
E_ET_psgV_05B05_F_0	BB987839
E_ET_psgV_05B05_R_0	BB992920
E_ET_psgV_05B06_F_0	BB987840
E_ET_psgV_05B06_R_0	BB992921
E_ET_psgV_05B07_F_0	BB987841
E_ET_psgV_05B08_F_0	BB987842
E_ET_psgV_05B08_R_0	BB992922
E_ET_psgV_05B09_F_0	BB987843
E_ET_psgV_05B09_R_0	BB992923
E_ET_psgV_05B10_R_0	BB992924
E_ET_psgV_05B11_F_0	BB987844
E_ET_psgV_05B12_F_0	BB987845
E_ET_psgV_05B12_R_0	BB992925
E_ET_psgV_05C01_R_0	BB992926
E_ET_psgV_05C02_F_0	BB987846
E_ET_psgV_05C02_R_0	BB992927
E_ET_psgV_05C04_F_0	BB987847
E_ET_psgV_05C04_R_0	BB992928
E_ET_psgV_05C05_F_0	BB987848
E_ET_psgV_05C05_R_0	BB992929
E_ET_psgV_05C06_F_0	BB987849
E_ET_psgV_05C07_F_0	BB987850
E_ET_psgV_05C07_R_0	BB992930
E_ET_psgV_05C09_F_0	BB987851
E_ET_psgV_05C09_R_0	BB992931
E_ET_psgV_05C10_F_0	BB987852
E_ET_psgV_05C10_R_0	BB992932
E_ET_psgV_05C11_F_0	BB987853
E_ET_psgV_05C11_R_0	BB992933
E_ET_psgV_05C12_F_0	BB987854
E_ET_psgV_05C12_R_0	BB992934
E_ET_psgV_05D01_F_0	BB987855
E_ET_psgV_05D01_R_0	BB992935
E_ET_psgV_05D02_R_0	BB992936
E_ET_psgV_05D03_F_0	BB987856
E_ET_psgV_05D04_F_0	BB987857
E_ET_psgV_05D04_R_0	BB992937
E_ET_psgV_05D05_F_0	BB987858
E_ET_psgV_05D05_R_0	BB992938
E_ET_psgV_05D06_F_0	BB987859
E_ET_psgV_05D07_F_0	BB987860

E_ET_psgV_05D07_R_0	BB992939
E_ET_psgV_05D08_F_0	BB987861
E_ET_psgV_05D08_R_0	BB992940
E_ET_psgV_05D09_F_0	BB987862
E_ET_psgV_05D09_R_0	BB992941
E_ET_psgV_05D11_F_0	BB987863
E_ET_psgV_05D12_F_0	BB987864
E_ET_psgV_05D12_R_0	BB992942
E_ET_psgV_05E01_F_0	BB987865
E_ET_psgV_05E01_R_0	BB992943
E_ET_psgV_05E02_F_0	BB987866
E_ET_psgV_05E03_F_0	BB987867
E_ET_psgV_05E03_R_0	BB992944
E_ET_psgV_05E04_F_0	BB987868
E_ET_psgV_05E04_R_0	BB992945
E_ET_psgV_05E05_F_0	BB987869
E_ET_psgV_05E06_R_0	BB992946
E_ET_psgV_05E07_F_0	BB987870
E_ET_psgV_05E07_R_0	BB992947
E_ET_psgV_05E08_F_0	BB987871
E_ET_psgV_05E09_F_0	BB987872
E_ET_psgV_05E09_R_0	BB992948
E_ET_psgV_05E10_F_0	BB987873
E_ET_psgV_05E10_R_0	BB992949
E_ET_psgV_05F01_F_0	BB987874
E_ET_psgV_05F01_R_0	BB992950
E_ET_psgV_05F02_F_0	BB987875
E_ET_psgV_05F04_F_0	BB987876
E_ET_psgV_05F04_R_0	BB992951
E_ET_psgV_05F05_F_0	BB987877
E_ET_psgV_05F05_R_0	BB992952
E_ET_psgV_05F06_F_0	BB987878
E_ET_psgV_05F07_F_0	BB987879
E_ET_psgV_05F07_R_0	BB992953
E_ET_psgV_05F08_F_0	BB987880
E_ET_psgV_05F09_F_0	BB987881
E_ET_psgV_05F09_R_0	BB992954
E_ET_psgV_05F10_F_0	BB987882
E_ET_psgV_05F10_R_0	BB992955
E_ET_psgV_05F11_F_0	BB987883
E_ET_psgV_05F11_R_0	BB992956
E_ET_psgV_05F12_F_0	BB987884
E_ET_psgV_05F12_R_0	BB992957
E_ET_psgV_05G02_F_0	BB987885
E_ET_psgV_05G02_R_0	BB992958
E_ET_psgV_05G03_F_0	BB987886
E_ET_psgV_05G04_F_0	BB987887
E_ET_psgV_05G05_F_0	BB987888
E_ET_psgV_05G05_R_0	BB992959
E_ET_psgV_05G07_F_0	BB987889
E_ET_psgV_05G08_F_0	BB987890
E_ET_psgV_05G08_R_0	BB992960
E_ET_psgV_05G09_F_0	BB987891
E_ET_psgV_05G09_R_0	BB992961

E_ET_psgV_05G11_F_0	BB987892
E_ET_psgV_05G11_R_0	BB992962
E_ET_psgV_05G12_F_0	BB987893
E_ET_psgV_05H01_F_0	BB987894
E_ET_psgV_05H01_R_0	BB992963
E_ET_psgV_05H02_F_0	BB987895
E_ET_psgV_05H02_R_0	BB992964
E_ET_psgV_05H03_F_0	BB987896
E_ET_psgV_05H03_R_0	BB992965
E_ET_psgV_05H04_F_0	BB987897
E_ET_psgV_05H04_R_0	BB992966
E_ET_psgV_05H05_R_0	BB992967
E_ET_psgV_05H06_F_0	BB987898
E_ET_psgV_05H06_R_0	BB992968
E_ET_psgV_05H07_R_0	BB992969
E_ET_psgV_05H08_F_0	BB987899
E_ET_psgV_05H10_F_0	BB987900
E_ET_psgV_05H11_F_0	BB987901
E_ET_psgV_05H11_R_0	BB992970
E_ET_psgV_05H12_F_0	BB987902
E_ET_psgV_05H12_R_0	BB992971
E_ET_psgV_06A01_F_0	BB987903
E_ET_psgV_06A01_R_0	BB992972
E_ET_psgV_06A02_R_0	BB992973
E_ET_psgV_06A03_F_0	BB987904
E_ET_psgV_06A03_R_0	BB992974
E_ET_psgV_06A05_F_0	BB987905
E_ET_psgV_06A07_F_0	BB987906
E_ET_psgV_06A08_R_0	BB992975
E_ET_psgV_06A11_F_0	BB987907
E_ET_psgV_06A11_R_0	BB992976
E_ET_psgV_06A12_F_0	BB987908
E_ET_psgV_06A12_R_0	BB992977
E_ET_psgV_06B01_F_0	BB987909
E_ET_psgV_06B01_R_0	BB992978
E_ET_psgV_06B02_F_0	BB987910
E_ET_psgV_06B02_R_0	BB992979
E_ET_psgV_06B03_F_0	BB987911
E_ET_psgV_06B03_R_0	BB992980
E_ET_psgV_06B04_F_0	BB987912
E_ET_psgV_06B04_R_0	BB992981
E_ET_psgV_06B05_R_0	BB992982
E_ET_psgV_06B06_F_0	BB987913
E_ET_psgV_06B06_R_0	BB992983
E_ET_psgV_06B07_F_0	BB987914
E_ET_psgV_06B07_R_0	BB992984
E_ET_psgV_06B08_F_0	BB987915
E_ET_psgV_06B08_R_0	BB992985
E_ET_psgV_06B10_F_0	BB987916
E_ET_psgV_06B10_R_0	BB992986
E_ET_psgV_06B11_F_0	BB987917
E_ET_psgV_06B12_F_0	BB987918
E_ET_psgV_06B12_R_0	BB992987
E_ET_psgV_06C01_F_0	BB987919

E_ET_psgV_06C01_R_0	BB992988
E_ET_psgV_06C02_F_0	BB987920
E_ET_psgV_06C02_R_0	BB992989
E_ET_psgV_06C03_F_0	BB987921
E_ET_psgV_06C04_F_0	BB987922
E_ET_psgV_06C04_R_0	BB992990
E_ET_psgV_06C05_F_0	BB987923
E_ET_psgV_06C05_R_0	BB992991
E_ET_psgV_06C06_F_0	BB987924
E_ET_psgV_06C06_R_0	BB992992
E_ET_psgV_06C07_F_0	BB987925
E_ET_psgV_06C07_R_0	BB992993
E_ET_psgV_06C08_F_0	BB987926
E_ET_psgV_06C09_R_0	BB992994
E_ET_psgV_06C10_F_0	BB987927
E_ET_psgV_06C10_R_0	BB992995
E_ET_psgV_06C11_F_0	BB987928
E_ET_psgV_06C11_R_0	BB992996
E_ET_psgV_06C12_F_0	BB987929
E_ET_psgV_06C12_R_0	BB992997
E_ET_psgV_06D01_F_0	BB987930
E_ET_psgV_06D01_R_0	BB992998
E_ET_psgV_06D02_F_0	BB987931
E_ET_psgV_06D02_R_0	BB992999
E_ET_psgV_06D03_R_0	BB993000
E_ET_psgV_06D04_F_0	BB987932
E_ET_psgV_06D04_R_0	BB993001
E_ET_psgV_06D05_F_0	BB987933
E_ET_psgV_06D06_F_0	BB987934
E_ET_psgV_06D06_R_0	BB993002
E_ET_psgV_06D07_F_0	BB987935
E_ET_psgV_06D07_R_0	BB993003
E_ET_psgV_06D09_F_0	BB987936
E_ET_psgV_06D09_R_0	BB993004
E_ET_psgV_06D10_F_0	BB987937
E_ET_psgV_06D10_R_0	BB993005
E_ET_psgV_06D11_F_0	BB987938
E_ET_psgV_06D12_F_0	BB987939
E_ET_psgV_06E01_F_0	BB987940
E_ET_psgV_06E01_R_0	BB993006
E_ET_psgV_06E02_F_0	BB987941
E_ET_psgV_06E02_R_0	BB993007
E_ET_psgV_06E04_F_0	BB987942
E_ET_psgV_06E06_F_0	BB987943
E_ET_psgV_06E07_F_0	BB987944
E_ET_psgV_06E08_F_0	BB987945
E_ET_psgV_06E08_R_0	BB993008
E_ET_psgV_06E09_F_0	BB987946
E_ET_psgV_06E09_R_0	BB993009
E_ET_psgV_06E10_F_0	BB987947
E_ET_psgV_06E11_F_0	BB987948
E_ET_psgV_06E11_R_0	BB993010
E_ET_psgV_06E12_F_0	BB987949
E_ET_psgV_06E12_R_0	BB993011

E_ET_psgV_06F01_F_0	BB987950
E_ET_psgV_06F01_R_0	BB993012
E_ET_psgV_06F02_F_0	BB987951
E_ET_psgV_06F03_F_0	BB987952
E_ET_psgV_06F03_R_0	BB993013
E_ET_psgV_06F04_F_0	BB987953
E_ET_psgV_06F04_R_0	BB993014
E_ET_psgV_06F05_F_0	BB987954
E_ET_psgV_06F05_R_0	BB993015
E_ET_psgV_06F06_F_0	BB987955
E_ET_psgV_06F06_R_0	BB993016
E_ET_psgV_06F07_F_0	BB987956
E_ET_psgV_06F07_R_0	BB993017
E_ET_psgV_06F08_F_0	BB987957
E_ET_psgV_06F08_R_0	BB993018
E_ET_psgV_06F09_F_0	BB987958
E_ET_psgV_06F09_R_0	BB993019
E_ET_psgV_06F10_F_0	BB987959
E_ET_psgV_06F10_R_0	BB993020
E_ET_psgV_06F11_F_0	BB987960
E_ET_psgV_06F11_R_0	BB993021
E_ET_psgV_06F12_F_0	BB987961
E_ET_psgV_06F12_R_0	BB993022
E_ET_psgV_06G02_F_0	BB987962
E_ET_psgV_06G02_R_0	BB993023
E_ET_psgV_06G03_F_0	BB987963
E_ET_psgV_06G03_R_0	BB993024
E_ET_psgV_06G04_F_0	BB987964
E_ET_psgV_06G04_R_0	BB993025
E_ET_psgV_06G05_F_0	BB987965
E_ET_psgV_06G05_R_0	BB993026
E_ET_psgV_06G06_F_0	BB987966
E_ET_psgV_06G07_F_0	BB987967
E_ET_psgV_06G07_R_0	BB993027
E_ET_psgV_06G08_F_0	BB987968
E_ET_psgV_06G08_R_0	BB993028
E_ET_psgV_06G10_F_0	BB987969
E_ET_psgV_06G10_R_0	BB993029
E_ET_psgV_06G11_F_0	BB987970
E_ET_psgV_06G11_R_0	BB993030
E_ET_psgV_06G12_F_0	BB987971
E_ET_psgV_06H01_F_0	BB987972
E_ET_psgV_06H01_R_0	BB993031
E_ET_psgV_06H02_F_0	BB987973
E_ET_psgV_06H03_F_0	BB987974
E_ET_psgV_06H03_R_0	BB993032
E_ET_psgV_06H04_F_0	BB987975
E_ET_psgV_06H05_F_0	BB987976
E_ET_psgV_06H05_R_0	BB993033
E_ET_psgV_06H06_F_0	BB987977
E_ET_psgV_06H06_R_0	BB993034
E_ET_psgV_06H07_F_0	BB987978
E_ET_psgV_06H07_R_0	BB993035
E_ET_psgV_06H08_F_0	BB987979

E_ET_psgV_06H08_R_0	BB993036
E_ET_psgV_06H09_F_0	BB987980
E_ET_psgV_06H10_F_0	BB987981
E_ET_psgV_06H10_R_0	BB993037
E_ET_psgV_06H11_F_0	BB987982
E_ET_psgV_06H11_R_0	BB993038
E_ET_psgV_06H12_F_0	BB987983
E_ET_psgV_07A02_F_0	BB987984
E_ET_psgV_07A02_R_0	BB993039
E_ET_psgV_07A03_F_0	BB987985
E_ET_psgV_07A03_R_0	BB993040
E_ET_psgV_07A04_F_0	BB987986
E_ET_psgV_07A05_F_0	BB987987
E_ET_psgV_07A05_R_0	BB993041
E_ET_psgV_07A07_F_0	BB987988
E_ET_psgV_07A07_R_0	BB993042
E_ET_psgV_07A08_F_0	BB987989
E_ET_psgV_07A08_R_0	BB993043
E_ET_psgV_07A09_F_0	BB987990
E_ET_psgV_07A10_F_0	BB987991
E_ET_psgV_07A10_R_0	BB993044
E_ET_psgV_07A11_F_0	BB987992
E_ET_psgV_07A12_F_0	BB987993
E_ET_psgV_07A12_R_0	BB993045
E_ET_psgV_07B01_F_0	BB987994
E_ET_psgV_07B02_F_0	BB987995
E_ET_psgV_07B02_R_0	BB993046
E_ET_psgV_07B03_F_0	BB987996
E_ET_psgV_07B03_R_0	BB993047
E_ET_psgV_07B04_F_0	BB987997
E_ET_psgV_07B04_R_0	BB993048
E_ET_psgV_07B06_F_0	BB987998
E_ET_psgV_07B07_F_0	BB987999
E_ET_psgV_07B07_R_0	BB993049
E_ET_psgV_07B08_F_0	BB988000
E_ET_psgV_07B08_R_0	BB993050
E_ET_psgV_07B09_F_0	BB988001
E_ET_psgV_07B09_R_0	BB993051
E_ET_psgV_07B10_F_0	BB988002
E_ET_psgV_07B10_R_0	BB993052
E_ET_psgV_07B12_R_0	BB993053
E_ET_psgV_07C01_R_0	BB993054
E_ET_psgV_07C02_F_0	BB988003
E_ET_psgV_07C02_R_0	BB993055
E_ET_psgV_07C03_F_0	BB988004
E_ET_psgV_07C03_R_0	BB993056
E_ET_psgV_07C04_F_0	BB988005
E_ET_psgV_07C04_R_0	BB993057
E_ET_psgV_07C05_F_0	BB988006
E_ET_psgV_07C05_R_0	BB993058
E_ET_psgV_07C06_F_0	BB988007
E_ET_psgV_07C06_R_0	BB993059
E_ET_psgV_07C07_F_0	BB988008
E_ET_psgV_07C08_F_0	BB988009

E_ET_psgV_07C08_R_0	BB993060
E_ET_psgV_07C09_F_0	BB988010
E_ET_psgV_07C09_R_0	BB993061
E_ET_psgV_07C10_F_0	BB988011
E_ET_psgV_07C10_R_0	BB993062
E_ET_psgV_07C11_F_0	BB988012
E_ET_psgV_07C11_R_0	BB993063
E_ET_psgV_07C12_R_0	BB993064
E_ET_psgV_07D01_F_0	BB988013
E_ET_psgV_07D01_R_0	BB993065
E_ET_psgV_07D02_F_0	BB988014
E_ET_psgV_07D02_R_0	BB993066
E_ET_psgV_07D03_F_0	BB988015
E_ET_psgV_07D03_R_0	BB993067
E_ET_psgV_07D04_F_0	BB988016
E_ET_psgV_07D04_R_0	BB993068
E_ET_psgV_07D05_F_0	BB988017
E_ET_psgV_07D05_R_0	BB993069
E_ET_psgV_07D06_F_0	BB988018
E_ET_psgV_07D07_F_0	BB988019
E_ET_psgV_07D08_F_0	BB988020
E_ET_psgV_07D09_F_0	BB988021
E_ET_psgV_07D09_R_0	BB993070
E_ET_psgV_07D10_F_0	BB988022
E_ET_psgV_07D11_F_0	BB988023
E_ET_psgV_07D11_R_0	BB993071
E_ET_psgV_07D12_F_0	BB988024
E_ET_psgV_07D12_R_0	BB993072
E_ET_psgV_07E01_F_0	BB988025
E_ET_psgV_07E01_R_0	BB993073
E_ET_psgV_07E02_F_0	BB988026
E_ET_psgV_07E02_R_0	BB993074
E_ET_psgV_07E03_F_0	BB988027
E_ET_psgV_07E03_R_0	BB993075
E_ET_psgV_07E04_F_0	BB988028
E_ET_psgV_07E04_R_0	BB993076
E_ET_psgV_07E05_R_0	BB993077
E_ET_psgV_07E06_F_0	BB988029
E_ET_psgV_07E06_R_0	BB993078
E_ET_psgV_07E07_F_0	BB988030
E_ET_psgV_07E07_R_0	BB993079
E_ET_psgV_07E08_F_0	BB988031
E_ET_psgV_07E08_R_0	BB993080
E_ET_psgV_07E09_F_0	BB988032
E_ET_psgV_07E09_R_0	BB993081
E_ET_psgV_07E10_F_0	BB988033
E_ET_psgV_07E10_R_0	BB993082
E_ET_psgV_07E11_F_0	BB988034
E_ET_psgV_07E11_R_0	BB993083
E_ET_psgV_07E12_F_0	BB988035
E_ET_psgV_07E12_R_0	BB993084
E_ET_psgV_07F01_F_0	BB988036
E_ET_psgV_07F01_R_0	BB993085
E_ET_psgV_07F02_F_0	BB988037

E_ET_psgV_07F02_R_0	BB993086
E_ET_psgV_07F03_R_0	BB993087
E_ET_psgV_07F05_F_0	BB988038
E_ET_psgV_07F06_F_0	BB988039
E_ET_psgV_07F06_R_0	BB993088
E_ET_psgV_07F07_F_0	BB988040
E_ET_psgV_07F07_R_0	BB993089
E_ET_psgV_07F08_F_0	BB988041
E_ET_psgV_07F08_R_0	BB993090
E_ET_psgV_07F09_F_0	BB988042
E_ET_psgV_07F09_R_0	BB993091
E_ET_psgV_07F10_F_0	BB988043
E_ET_psgV_07F10_R_0	BB993092
E_ET_psgV_07F11_F_0	BB988044
E_ET_psgV_07F12_F_0	BB988045
E_ET_psgV_07G01_F_0	BB988046
E_ET_psgV_07G02_F_0	BB988047
E_ET_psgV_07G02_R_0	BB993093
E_ET_psgV_07G03_F_0	BB988048
E_ET_psgV_07G03_R_0	BB993094
E_ET_psgV_07G04_F_0	BB988049
E_ET_psgV_07G04_R_0	BB993095
E_ET_psgV_07G05_F_0	BB988050
E_ET_psgV_07G05_R_0	BB993096
E_ET_psgV_07G06_F_0	BB988051
E_ET_psgV_07G06_R_0	BB993097
E_ET_psgV_07G07_F_0	BB988052
E_ET_psgV_07G07_R_0	BB993098
E_ET_psgV_07G08_F_0	BB988053
E_ET_psgV_07G09_F_0	BB988054
E_ET_psgV_07G09_R_0	BB993099
E_ET_psgV_07G10_F_0	BB988055
E_ET_psgV_07G10_R_0	BB993100
E_ET_psgV_07G11_F_0	BB988056
E_ET_psgV_07G11_R_0	BB993101
E_ET_psgV_07G12_F_0	BB988057
E_ET_psgV_07H01_F_0	BB988058
E_ET_psgV_07H02_F_0	BB988059
E_ET_psgV_07H02_R_0	BB993102
E_ET_psgV_07H03_F_0	BB988060
E_ET_psgV_07H05_F_0	BB988061
E_ET_psgV_07H05_R_0	BB993103
E_ET_psgV_07H06_F_0	BB988062
E_ET_psgV_07H07_R_0	BB993104
E_ET_psgV_07H08_F_0	BB988063
E_ET_psgV_07H08_R_0	BB993105
E_ET_psgV_07H09_F_0	BB988064
E_ET_psgV_07H09_R_0	BB993106
E_ET_psgV_07H10_F_0	BB988065
E_ET_psgV_07H11_F_0	BB988066
E_ET_psgV_07H11_R_0	BB993107
E_ET_psgV_07H12_F_0	BB988067
E_ET_psgV_07H12_R_0	BB993108
E_ET_psgV_08A01_F_0	BB988068

E_ET_psgV_08A01_R_0	BB993109
E_ET_psgV_08A02_F_0	BB988069
E_ET_psgV_08A02_R_0	BB993110
E_ET_psgV_08A03_F_0	BB988070
E_ET_psgV_08A04_F_0	BB988071
E_ET_psgV_08A04_R_0	BB993111
E_ET_psgV_08A05_F_0	BB988072
E_ET_psgV_08A05_R_0	BB993112
E_ET_psgV_08A06_F_0	BB988073
E_ET_psgV_08A07_F_0	BB988074
E_ET_psgV_08A07_R_0	BB993113
E_ET_psgV_08A08_F_0	BB988075
E_ET_psgV_08A08_R_0	BB993114
E_ET_psgV_08A09_F_0	BB988076
E_ET_psgV_08A09_R_0	BB993115
E_ET_psgV_08A10_F_0	BB988077
E_ET_psgV_08A10_R_0	BB993116
E_ET_psgV_08A11_F_0	BB988078
E_ET_psgV_08A11_R_0	BB993117
E_ET_psgV_08A12_F_0	BB988079
E_ET_psgV_08A12_R_0	BB993118
E_ET_psgV_08B01_F_0	BB988080
E_ET_psgV_08B01_R_0	BB993119
E_ET_psgV_08B02_F_0	BB988081
E_ET_psgV_08B02_R_0	BB993120
E_ET_psgV_08B03_F_0	BB988082
E_ET_psgV_08B03_R_0	BB993121
E_ET_psgV_08B04_F_0	BB988083
E_ET_psgV_08B04_R_0	BB993122
E_ET_psgV_08B05_F_0	BB988084
E_ET_psgV_08B05_R_0	BB993123
E_ET_psgV_08B06_F_0	BB988085
E_ET_psgV_08B06_R_0	BB993124
E_ET_psgV_08B07_F_0	BB988086
E_ET_psgV_08B07_R_0	BB993125
E_ET_psgV_08B08_F_0	BB988087
E_ET_psgV_08B08_R_0	BB993126
E_ET_psgV_08B09_F_0	BB988088
E_ET_psgV_08B09_R_0	BB993127
E_ET_psgV_08B10_F_0	BB988089
E_ET_psgV_08B11_F_0	BB988090
E_ET_psgV_08B11_R_0	BB993128
E_ET_psgV_08B12_F_0	BB988091
E_ET_psgV_08C01_F_0	BB988092
E_ET_psgV_08C01_R_0	BB993129
E_ET_psgV_08C02_F_0	BB988093
E_ET_psgV_08C02_R_0	BB993130
E_ET_psgV_08C03_F_0	BB988094
E_ET_psgV_08C03_R_0	BB993131
E_ET_psgV_08C05_F_0	BB988095
E_ET_psgV_08C05_R_0	BB993132
E_ET_psgV_08C06_F_0	BB988096
E_ET_psgV_08C07_F_0	BB988097
E_ET_psgV_08C08_F_0	BB988098

E_ET_psgV_08C08_R_0	BB993133
E_ET_psgV_08C09_F_0	BB988099
E_ET_psgV_08C09_R_0	BB993134
E_ET_psgV_08C10_F_0	BB988100
E_ET_psgV_08C10_R_0	BB993135
E_ET_psgV_08C11_F_0	BB988101
E_ET_psgV_08C11_R_0	BB993136
E_ET_psgV_08C12_F_0	BB988102
E_ET_psgV_08C12_R_0	BB993137
E_ET_psgV_08D01_R_0	BB993138
E_ET_psgV_08D02_F_0	BB988103
E_ET_psgV_08D02_R_0	BB993139
E_ET_psgV_08D03_F_0	BB988104
E_ET_psgV_08D03_R_0	BB993140
E_ET_psgV_08D04_F_0	BB988105
E_ET_psgV_08D04_R_0	BB993141
E_ET_psgV_08D05_F_0	BB988106
E_ET_psgV_08D05_R_0	BB993142
E_ET_psgV_08D06_F_0	BB988107
E_ET_psgV_08D07_F_0	BB988108
E_ET_psgV_08D07_R_0	BB993143
E_ET_psgV_08D09_F_0	BB988109
E_ET_psgV_08D09_R_0	BB993144
E_ET_psgV_08D10_F_0	BB988110
E_ET_psgV_08D11_F_0	BB988111
E_ET_psgV_08D11_R_0	BB993145
E_ET_psgV_08D12_F_0	BB988112
E_ET_psgV_08D12_R_0	BB993146
E_ET_psgV_08E03_F_0	BB988113
E_ET_psgV_08E03_R_0	BB993147
E_ET_psgV_08E04_F_0	BB988114
E_ET_psgV_08E04_R_0	BB993148
E_ET_psgV_08E05_F_0	BB988115
E_ET_psgV_08E05_R_0	BB993149
E_ET_psgV_08E06_F_0	BB988116
E_ET_psgV_08E06_R_0	BB993150
E_ET_psgV_08E08_F_0	BB988117
E_ET_psgV_08E08_R_0	BB993151
E_ET_psgV_08E09_F_0	BB988118
E_ET_psgV_08E09_R_0	BB993152
E_ET_psgV_08E10_F_0	BB988119
E_ET_psgV_08E10_R_0	BB993153
E_ET_psgV_08E11_F_0	BB988120
E_ET_psgV_08E11_R_0	BB993154
E_ET_psgV_08E12_F_0	BB988121
E_ET_psgV_08F01_F_0	BB988122
E_ET_psgV_08F01_R_0	BB993155
E_ET_psgV_08F02_F_0	BB988123
E_ET_psgV_08F02_R_0	BB993156
E_ET_psgV_08F03_F_0	BB988124
E_ET_psgV_08F03_R_0	BB993157
E_ET_psgV_08F04_F_0	BB988125
E_ET_psgV_08F05_F_0	BB988126
E_ET_psgV_08F06_F_0	BB988127

E_ET_psgV_08F06_R_0	BB993158
E_ET_psgV_08F07_F_0	BB988128
E_ET_psgV_08F07_R_0	BB993159
E_ET_psgV_08F08_F_0	BB988129
E_ET_psgV_08F08_R_0	BB993160
E_ET_psgV_08F10_F_0	BB988130
E_ET_psgV_08F10_R_0	BB993161
E_ET_psgV_08F11_F_0	BB988131
E_ET_psgV_08F11_R_0	BB993162
E_ET_psgV_08F12_F_0	BB988132
E_ET_psgV_08F12_R_0	BB993163
E_ET_psgV_08G01_F_0	BB988133
E_ET_psgV_08G02_F_0	BB988134
E_ET_psgV_08G02_R_0	BB993164
E_ET_psgV_08G03_F_0	BB988135
E_ET_psgV_08G03_R_0	BB993165
E_ET_psgV_08G05_F_0	BB988136
E_ET_psgV_08G05_R_0	BB993166
E_ET_psgV_08G06_R_0	BB993167
E_ET_psgV_08G07_F_0	BB988137
E_ET_psgV_08G07_R_0	BB993168
E_ET_psgV_08G09_F_0	BB988138
E_ET_psgV_08G09_R_0	BB993169
E_ET_psgV_08G10_F_0	BB988139
E_ET_psgV_08G10_R_0	BB993170
E_ET_psgV_08G11_F_0	BB988140
E_ET_psgV_08G11_R_0	BB993171
E_ET_psgV_08G12_F_0	BB988141
E_ET_psgV_08G12_R_0	BB993172
E_ET_psgV_08H01_F_0	BB988142
E_ET_psgV_08H02_F_0	BB988143
E_ET_psgV_08H03_F_0	BB988144
E_ET_psgV_08H03_R_0	BB993173
E_ET_psgV_08H04_F_0	BB988145
E_ET_psgV_08H04_R_0	BB993174
E_ET_psgV_08H05_F_0	BB988146
E_ET_psgV_08H05_R_0	BB993175
E_ET_psgV_08H06_F_0	BB988147
E_ET_psgV_08H06_R_0	BB993176
E_ET_psgV_08H08_F_0	BB988148
E_ET_psgV_08H08_R_0	BB993177
E_ET_psgV_08H09_F_0	BB988149
E_ET_psgV_08H09_R_0	BB993178
E_ET_psgV_08H11_F_0	BB988150
E_ET_psgV_08H11_R_0	BB993179
E_ET_psgV_08H12_F_0	BB988151
E_ET_psgV_08H12_R_0	BB993180
E_ET_psgV_09A01_F_0	BB988152
E_ET_psgV_09A01_R_0	BB993181
E_ET_psgV_09A03_F_0	BB988153
E_ET_psgV_09A04_F_0	BB988154
E_ET_psgV_09A04_R_0	BB993182
E_ET_psgV_09A06_F_0	BB988155
E_ET_psgV_09A08_F_0	BB988156

E_ET_psgV_09A08_R_0	BB993183
E_ET_psgV_09A09_F_0	BB988157
E_ET_psgV_09A10_F_0	BB988158
E_ET_psgV_09A10_R_0	BB993184
E_ET_psgV_09A11_F_0	BB988159
E_ET_psgV_09A11_R_0	BB993185
E_ET_psgV_09A12_F_0	BB988160
E_ET_psgV_09B01_F_0	BB988161
E_ET_psgV_09B01_R_0	BB993186
E_ET_psgV_09B03_F_0	BB988162
E_ET_psgV_09B03_R_0	BB993187
E_ET_psgV_09B04_F_0	BB988163
E_ET_psgV_09B04_R_0	BB993188
E_ET_psgV_09B05_F_0	BB988164
E_ET_psgV_09B06_F_0	BB988165
E_ET_psgV_09B07_F_0	BB988166
E_ET_psgV_09B07_R_0	BB993189
E_ET_psgV_09B08_F_0	BB988167
E_ET_psgV_09B08_R_0	BB993190
E_ET_psgV_09B09_F_0	BB988168
E_ET_psgV_09B09_R_0	BB993191
E_ET_psgV_09B10_F_0	BB988169
E_ET_psgV_09B10_R_0	BB993192
E_ET_psgV_09B11_F_0	BB988170
E_ET_psgV_09B12_F_0	BB988171
E_ET_psgV_09B12_R_0	BB993193
E_ET_psgV_09C01_F_0	BB988172
E_ET_psgV_09C01_R_0	BB993194
E_ET_psgV_09C02_F_0	BB988173
E_ET_psgV_09C02_R_0	BB993195
E_ET_psgV_09C03_F_0	BB988174
E_ET_psgV_09C03_R_0	BB993196
E_ET_psgV_09C04_F_0	BB988175
E_ET_psgV_09C04_R_0	BB993197
E_ET_psgV_09C05_F_0	BB988176
E_ET_psgV_09C05_R_0	BB993198
E_ET_psgV_09C06_F_0	BB988177
E_ET_psgV_09C06_R_0	BB993199
E_ET_psgV_09C07_F_0	BB988178
E_ET_psgV_09C07_R_0	BB993200
E_ET_psgV_09C08_F_0	BB988179
E_ET_psgV_09C09_F_0	BB988180
E_ET_psgV_09C09_R_0	BB993201
E_ET_psgV_09C10_F_0	BB988181
E_ET_psgV_09C10_R_0	BB993202
E_ET_psgV_09C11_F_0	BB988182
E_ET_psgV_09C11_R_0	BB993203
E_ET_psgV_09C12_F_0	BB988183
E_ET_psgV_09C12_R_0	BB993204
E_ET_psgV_09D01_F_0	BB988184
E_ET_psgV_09D01_R_0	BB993205
E_ET_psgV_09D02_R_0	BB993206
E_ET_psgV_09D03_F_0	BB988185
E_ET_psgV_09D03_R_0	BB993207

E_ET_psgV_09D04_F_0	BB988186
E_ET_psgV_09D04_R_0	BB993208
E_ET_psgV_09D05_F_0	BB988187
E_ET_psgV_09D05_R_0	BB993209
E_ET_psgV_09D06_F_0	BB988188
E_ET_psgV_09D06_R_0	BB993210
E_ET_psgV_09D07_F_0	BB988189
E_ET_psgV_09D08_F_0	BB988190
E_ET_psgV_09D08_R_0	BB993211
E_ET_psgV_09D09_F_0	BB988191
E_ET_psgV_09D10_F_0	BB988192
E_ET_psgV_09D10_R_0	BB993212
E_ET_psgV_09D11_F_0	BB988193
E_ET_psgV_09D11_R_0	BB993213
E_ET_psgV_09D12_F_0	BB988194
E_ET_psgV_09D12_R_0	BB993214
E_ET_psgV_09E01_F_0	BB988195
E_ET_psgV_09E02_F_0	BB988196
E_ET_psgV_09E02_R_0	BB993215
E_ET_psgV_09E03_F_0	BB988197
E_ET_psgV_09E03_R_0	BB993216
E_ET_psgV_09E04_F_0	BB988198
E_ET_psgV_09E04_R_0	BB993217
E_ET_psgV_09E05_F_0	BB988199
E_ET_psgV_09E05_R_0	BB993218
E_ET_psgV_09E06_F_0	BB988200
E_ET_psgV_09E07_F_0	BB988201
E_ET_psgV_09E07_R_0	BB993219
E_ET_psgV_09E08_R_0	BB993220
E_ET_psgV_09E09_F_0	BB988202
E_ET_psgV_09E09_R_0	BB993221
E_ET_psgV_09E11_F_0	BB988203
E_ET_psgV_09E11_R_0	BB993222
E_ET_psgV_09E12_F_0	BB988204
E_ET_psgV_09E12_R_0	BB993223
E_ET_psgV_09F01_F_0	BB988205
E_ET_psgV_09F01_R_0	BB993224
E_ET_psgV_09F02_F_0	BB988206
E_ET_psgV_09F02_R_0	BB993225
E_ET_psgV_09F03_F_0	BB988207
E_ET_psgV_09F03_R_0	BB993226
E_ET_psgV_09F04_F_0	BB988208
E_ET_psgV_09F04_R_0	BB993227
E_ET_psgV_09F05_F_0	BB988209
E_ET_psgV_09F06_F_0	BB988210
E_ET_psgV_09F06_R_0	BB993228
E_ET_psgV_09F07_F_0	BB988211
E_ET_psgV_09F07_R_0	BB993229
E_ET_psgV_09F08_F_0	BB988212
E_ET_psgV_09F08_R_0	BB993230
E_ET_psgV_09F09_F_0	BB988213
E_ET_psgV_09F09_R_0	BB993231
E_ET_psgV_09F10_F_0	BB988214
E_ET_psgV_09F10_R_0	BB993232

E_ET_psgV_09F11_F_0	BB988215
E_ET_psgV_09F12_F_0	BB988216
E_ET_psgV_09G01_F_0	BB988217
E_ET_psgV_09G01_R_0	BB993233
E_ET_psgV_09G02_F_0	BB988218
E_ET_psgV_09G02_R_0	BB993234
E_ET_psgV_09G03_F_0	BB988219
E_ET_psgV_09G03_R_0	BB993235
E_ET_psgV_09G04_F_0	BB988220
E_ET_psgV_09G04_R_0	BB993236
E_ET_psgV_09G05_F_0	BB988221
E_ET_psgV_09G05_R_0	BB993237
E_ET_psgV_09G06_F_0	BB988222
E_ET_psgV_09G06_R_0	BB993238
E_ET_psgV_09G07_F_0	BB988223
E_ET_psgV_09G08_F_0	BB988224
E_ET_psgV_09G09_F_0	BB988225
E_ET_psgV_09G09_R_0	BB993239
E_ET_psgV_09G10_F_0	BB988226
E_ET_psgV_09G11_F_0	BB988227
E_ET_psgV_09G11_R_0	BB993240
E_ET_psgV_09G12_F_0	BB988228
E_ET_psgV_09G12_R_0	BB993241
E_ET_psgV_09H01_R_0	BB993242
E_ET_psgV_09H02_F_0	BB988229
E_ET_psgV_09H02_R_0	BB993243
E_ET_psgV_09H03_F_0	BB988230
E_ET_psgV_09H03_R_0	BB993244
E_ET_psgV_09H04_F_0	BB988231
E_ET_psgV_09H06_F_0	BB988232
E_ET_psgV_09H07_F_0	BB988233
E_ET_psgV_09H07_R_0	BB993245
E_ET_psgV_09H08_F_0	BB988234
E_ET_psgV_09H09_F_0	BB988235
E_ET_psgV_09H09_R_0	BB993246
E_ET_psgV_09H10_F_0	BB988236
E_ET_psgV_09H11_F_0	BB988237
E_ET_psgV_09H11_R_0	BB993247
E_ET_psgV_09H12_F_0	BB988238
E_ET_psgV_09H12_R_0	BB993248
E_ET_psgV_10A01_F_0	BB988239
E_ET_psgV_10A01_R_0	BB993249
E_ET_psgV_10A02_F_0	BB988240
E_ET_psgV_10A02_R_0	BB993250
E_ET_psgV_10A03_F_0	BB988241
E_ET_psgV_10A03_R_0	BB993251
E_ET_psgV_10A04_F_0	BB988242
E_ET_psgV_10A04_R_0	BB993252
E_ET_psgV_10A05_F_0	BB988243
E_ET_psgV_10A06_F_0	BB988244
E_ET_psgV_10A06_R_0	BB993253
E_ET_psgV_10A08_F_0	BB988245
E_ET_psgV_10A08_R_0	BB993254
E_ET_psgV_10A09_F_0	BB988246

E_ET_psgV_10A10_F_0	BB988247
E_ET_psgV_10A10_R_0	BB993255
E_ET_psgV_10A11_F_0	BB988248
E_ET_psgV_10A11_R_0	BB993256
E_ET_psgV_10A12_F_0	BB988249
E_ET_psgV_10A12_R_0	BB993257
E_ET_psgV_10B01_F_0	BB988250
E_ET_psgV_10B02_F_0	BB988251
E_ET_psgV_10B03_F_0	BB988252
E_ET_psgV_10B03_R_0	BB993258
E_ET_psgV_10B04_F_0	BB988253
E_ET_psgV_10B04_R_0	BB993259
E_ET_psgV_10B05_F_0	BB988254
E_ET_psgV_10B05_R_0	BB993260
E_ET_psgV_10B06_F_0	BB988255
E_ET_psgV_10B06_R_0	BB993261
E_ET_psgV_10B07_F_0	BB988256
E_ET_psgV_10B08_F_0	BB988257
E_ET_psgV_10B08_R_0	BB993262
E_ET_psgV_10B09_F_0	BB988258
E_ET_psgV_10B09_R_0	BB993263
E_ET_psgV_10B10_F_0	BB988259
E_ET_psgV_10B10_R_0	BB993264
E_ET_psgV_10B11_F_0	BB988260
E_ET_psgV_10B11_R_0	BB993265
E_ET_psgV_10B12_F_0	BB988261
E_ET_psgV_10B12_R_0	BB993266
E_ET_psgV_10C01_F_0	BB988262
E_ET_psgV_10C02_F_0	BB988263
E_ET_psgV_10C02_R_0	BB993267
E_ET_psgV_10C03_F_0	BB988264
E_ET_psgV_10C03_R_0	BB993268
E_ET_psgV_10C04_F_0	BB988265
E_ET_psgV_10C04_R_0	BB993269
E_ET_psgV_10C05_F_0	BB988266
E_ET_psgV_10C05_R_0	BB993270
E_ET_psgV_10C07_F_0	BB988267
E_ET_psgV_10C07_R_0	BB993271
E_ET_psgV_10C08_F_0	BB988268
E_ET_psgV_10C08_R_0	BB993272
E_ET_psgV_10C09_F_0	BB988269
E_ET_psgV_10C09_R_0	BB993273
E_ET_psgV_10C10_F_0	BB988270
E_ET_psgV_10C11_F_0	BB988271
E_ET_psgV_10C11_R_0	BB993274
E_ET_psgV_10C12_F_0	BB988272
E_ET_psgV_10C12_R_0	BB993275
E_ET_psgV_10D01_F_0	BB988273
E_ET_psgV_10D01_R_0	BB993276
E_ET_psgV_10D02_F_0	BB988274
E_ET_psgV_10D02_R_0	BB993277
E_ET_psgV_10D03_F_0	BB988275
E_ET_psgV_10D03_R_0	BB993278
E_ET_psgV_10D04_F_0	BB988276

E_ET_psgV_10D04_R_0	BB993279
E_ET_psgV_10D05_F_0	BB988277
E_ET_psgV_10D05_R_0	BB993280
E_ET_psgV_10D06_F_0	BB988278
E_ET_psgV_10D06_R_0	BB993281
E_ET_psgV_10D07_F_0	BB988279
E_ET_psgV_10D07_R_0	BB993282
E_ET_psgV_10D08_F_0	BB988280
E_ET_psgV_10D08_R_0	BB993283
E_ET_psgV_10D09_R_0	BB993284
E_ET_psgV_10D10_F_0	BB988281
E_ET_psgV_10D10_R_0	BB993285
E_ET_psgV_10D11_F_0	BB988282
E_ET_psgV_10D11_R_0	BB993286
E_ET_psgV_10D12_F_0	BB988283
E_ET_psgV_10D12_R_0	BB993287
E_ET_psgV_10E01_F_0	BB988284
E_ET_psgV_10E01_R_0	BB993288
E_ET_psgV_10E02_F_0	BB988285
E_ET_psgV_10E02_R_0	BB993289
E_ET_psgV_10E03_F_0	BB988286
E_ET_psgV_10E03_R_0	BB993290
E_ET_psgV_10E05_F_0	BB988287
E_ET_psgV_10E05_R_0	BB993291
E_ET_psgV_10E06_F_0	BB988288
E_ET_psgV_10E07_F_0	BB988289
E_ET_psgV_10E07_R_0	BB993292
E_ET_psgV_10E08_F_0	BB988290
E_ET_psgV_10E08_R_0	BB993293
E_ET_psgV_10E09_F_0	BB988291
E_ET_psgV_10E10_F_0	BB988292
E_ET_psgV_10E10_R_0	BB993294
E_ET_psgV_10E11_F_0	BB988293
E_ET_psgV_10E11_R_0	BB993295
E_ET_psgV_10E12_F_0	BB988294
E_ET_psgV_10E12_R_0	BB993296
E_ET_psgV_10F01_F_0	BB988295
E_ET_psgV_10F01_R_0	BB993297
E_ET_psgV_10F02_F_0	BB988296
E_ET_psgV_10F02_R_0	BB993298
E_ET_psgV_10F03_F_0	BB988297
E_ET_psgV_10F03_R_0	BB993299
E_ET_psgV_10F04_F_0	BB988298
E_ET_psgV_10F04_R_0	BB993300
E_ET_psgV_10F05_F_0	BB988299
E_ET_psgV_10F05_R_0	BB993301
E_ET_psgV_10F06_F_0	BB988300
E_ET_psgV_10F07_F_0	BB988301
E_ET_psgV_10F07_R_0	BB993302
E_ET_psgV_10F08_F_0	BB988302
E_ET_psgV_10F08_R_0	BB993303
E_ET_psgV_10F09_F_0	BB988303
E_ET_psgV_10F09_R_0	BB993304
E_ET_psgV_10F10_F_0	BB988304

E_ET_psgV_10F10_R_0	BB993305
E_ET_psgV_10F11_F_0	BB988305
E_ET_psgV_10F11_R_0	BB993306
E_ET_psgV_10F12_F_0	BB988306
E_ET_psgV_10F12_R_0	BB993307
E_ET_psgV_10G01_F_0	BB988307
E_ET_psgV_10G01_R_0	BB993308
E_ET_psgV_10G02_F_0	BB988308
E_ET_psgV_10G02_R_0	BB993309
E_ET_psgV_10G03_F_0	BB988309
E_ET_psgV_10G03_R_0	BB993310
E_ET_psgV_10G04_F_0	BB988310
E_ET_psgV_10G04_R_0	BB993311
E_ET_psgV_10G05_F_0	BB988311
E_ET_psgV_10G05_R_0	BB993312
E_ET_psgV_10G06_F_0	BB988312
E_ET_psgV_10G06_R_0	BB993313
E_ET_psgV_10G07_F_0	BB988313
E_ET_psgV_10G07_R_0	BB993314
E_ET_psgV_10G08_F_0	BB988314
E_ET_psgV_10G08_R_0	BB993315
E_ET_psgV_10G09_F_0	BB988315
E_ET_psgV_10G10_F_0	BB988316
E_ET_psgV_10G10_R_0	BB993316
E_ET_psgV_10G11_F_0	BB988317
E_ET_psgV_10G11_R_0	BB993317
E_ET_psgV_10G12_R_0	BB993318
E_ET_psgV_10H01_F_0	BB988318
E_ET_psgV_10H01_R_0	BB993319
E_ET_psgV_10H02_F_0	BB988319
E_ET_psgV_10H02_R_0	BB993320
E_ET_psgV_10H03_F_0	BB988320
E_ET_psgV_10H03_R_0	BB993321
E_ET_psgV_10H04_F_0	BB988321
E_ET_psgV_10H05_F_0	BB988322
E_ET_psgV_10H05_R_0	BB993322
E_ET_psgV_10H06_F_0	BB988323
E_ET_psgV_10H06_R_0	BB993323
E_ET_psgV_10H07_F_0	BB988324
E_ET_psgV_10H07_R_0	BB993324
E_ET_psgV_10H09_F_0	BB988325
E_ET_psgV_10H09_R_0	BB993325
E_ET_psgV_10H10_F_0	BB988326
E_ET_psgV_10H10_R_0	BB993326
E_ET_psgV_10H11_F_0	BB988327
E_ET_psgV_10H11_R_0	BB993327
E_ET_psgV_10H12_F_0	BB988328
E_ET_psgV_10H12_R_0	BB993328
E_ET_psgV_11A01_F_0	BB988329
E_ET_psgV_11A01_R_0	BB993329
E_ET_psgV_11A02_F_0	BB988330
E_ET_psgV_11A03_F_0	BB988331
E_ET_psgV_11A03_R_0	BB993330
E_ET_psgV_11A04_F_0	BB988332

E_ET_psgV_11A04_R_0	BB993331
E_ET_psgV_11A05_F_0	BB988333
E_ET_psgV_11A05_R_0	BB993332
E_ET_psgV_11A06_F_0	BB988334
E_ET_psgV_11A06_R_0	BB993333
E_ET_psgV_11A07_F_0	BB988335
E_ET_psgV_11A08_F_0	BB988336
E_ET_psgV_11A08_R_0	BB993334
E_ET_psgV_11A09_F_0	BB988337
E_ET_psgV_11A09_R_0	BB993335
E_ET_psgV_11A10_F_0	BB988338
E_ET_psgV_11A10_R_0	BB993336
E_ET_psgV_11A11_F_0	BB988339
E_ET_psgV_11A11_R_0	BB993337
E_ET_psgV_11A12_F_0	BB988340
E_ET_psgV_11A12_R_0	BB993338
E_ET_psgV_11B01_F_0	BB988341
E_ET_psgV_11B01_R_0	BB993339
E_ET_psgV_11B02_F_0	BB988342
E_ET_psgV_11B02_R_0	BB993340
E_ET_psgV_11B03_F_0	BB988343
E_ET_psgV_11B03_R_0	BB993341
E_ET_psgV_11B05_F_0	BB988344
E_ET_psgV_11B05_R_0	BB993342
E_ET_psgV_11B06_F_0	BB988345
E_ET_psgV_11B06_R_0	BB993343
E_ET_psgV_11B09_F_0	BB988346
E_ET_psgV_11B09_R_0	BB993344
E_ET_psgV_11B10_F_0	BB988347
E_ET_psgV_11B10_R_0	BB993345
E_ET_psgV_11B11_F_0	BB988348
E_ET_psgV_11B12_F_0	BB988349
E_ET_psgV_11B12_R_0	BB993346
E_ET_psgV_11C01_F_0	BB988350
E_ET_psgV_11C01_R_0	BB993347
E_ET_psgV_11C02_F_0	BB988351
E_ET_psgV_11C02_R_0	BB993348
E_ET_psgV_11C03_F_0	BB988352
E_ET_psgV_11C03_R_0	BB993349
E_ET_psgV_11C04_F_0	BB988353
E_ET_psgV_11C05_F_0	BB988354
E_ET_psgV_11C05_R_0	BB993350
E_ET_psgV_11C06_F_0	BB988355
E_ET_psgV_11C06_R_0	BB993351
E_ET_psgV_11C07_F_0	BB988356
E_ET_psgV_11C07_R_0	BB993352
E_ET_psgV_11C08_F_0	BB988357
E_ET_psgV_11C09_F_0	BB988358
E_ET_psgV_11C09_R_0	BB993353
E_ET_psgV_11C10_F_0	BB988359
E_ET_psgV_11C10_R_0	BB993354
E_ET_psgV_11C11_F_0	BB988360
E_ET_psgV_11C11_R_0	BB993355
E_ET_psgV_11C12_F_0	BB988361

E_ET_psgV_11C12_R_0	BB993356
E_ET_psgV_11D01_F_0	BB988362
E_ET_psgV_11D02_F_0	BB988363
E_ET_psgV_11D02_R_0	BB993357
E_ET_psgV_11D03_F_0	BB988364
E_ET_psgV_11D03_R_0	BB993358
E_ET_psgV_11D04_F_0	BB988365
E_ET_psgV_11D04_R_0	BB993359
E_ET_psgV_11D05_F_0	BB988366
E_ET_psgV_11D05_R_0	BB993360
E_ET_psgV_11D06_F_0	BB988367
E_ET_psgV_11D06_R_0	BB993361
E_ET_psgV_11D07_F_0	BB988368
E_ET_psgV_11D08_F_0	BB988369
E_ET_psgV_11D08_R_0	BB993362
E_ET_psgV_11D09_F_0	BB988370
E_ET_psgV_11D09_R_0	BB993363
E_ET_psgV_11D10_F_0	BB988371
E_ET_psgV_11D10_R_0	BB993364
E_ET_psgV_11D11_F_0	BB988372
E_ET_psgV_11D11_R_0	BB993365
E_ET_psgV_11D12_F_0	BB988373
E_ET_psgV_11D12_R_0	BB993366
E_ET_psgV_11E01_F_0	BB988374
E_ET_psgV_11E01_R_0	BB993367
E_ET_psgV_11E02_F_0	BB988375
E_ET_psgV_11E02_R_0	BB993368
E_ET_psgV_11E03_F_0	BB988376
E_ET_psgV_11E04_F_0	BB988377
E_ET_psgV_11E05_F_0	BB988378
E_ET_psgV_11E05_R_0	BB993369
E_ET_psgV_11E06_F_0	BB988379
E_ET_psgV_11E06_R_0	BB993370
E_ET_psgV_11E07_F_0	BB988380
E_ET_psgV_11E07_R_0	BB993371
E_ET_psgV_11E08_F_0	BB988381
E_ET_psgV_11E09_F_0	BB988382
E_ET_psgV_11E09_R_0	BB993372
E_ET_psgV_11E10_F_0	BB988383
E_ET_psgV_11E10_R_0	BB993373
E_ET_psgV_11E11_F_0	BB988384
E_ET_psgV_11E11_R_0	BB993374
E_ET_psgV_11E12_F_0	BB988385
E_ET_psgV_11E12_R_0	BB993375
E_ET_psgV_11F01_F_0	BB988386
E_ET_psgV_11F01_R_0	BB993376
E_ET_psgV_11F02_F_0	BB988387
E_ET_psgV_11F02_R_0	BB993377
E_ET_psgV_11F03_F_0	BB988388
E_ET_psgV_11F03_R_0	BB993378
E_ET_psgV_11F04_F_0	BB988389
E_ET_psgV_11F04_R_0	BB993379
E_ET_psgV_11F05_F_0	BB988390
E_ET_psgV_11F05_R_0	BB993380

E_ET_psgV_11F07_F_0	BB988391
E_ET_psgV_11F07_R_0	BB993381
E_ET_psgV_11F08_F_0	BB988392
E_ET_psgV_11F08_R_0	BB993382
E_ET_psgV_11F09_F_0	BB988393
E_ET_psgV_11F09_R_0	BB993383
E_ET_psgV_11F10_F_0	BB988394
E_ET_psgV_11F10_R_0	BB993384
E_ET_psgV_11F11_F_0	BB988395
E_ET_psgV_11F12_F_0	BB988396
E_ET_psgV_11F12_R_0	BB993385
E_ET_psgV_11G01_F_0	BB988397
E_ET_psgV_11G01_R_0	BB993386
E_ET_psgV_11G02_F_0	BB988398
E_ET_psgV_11G02_R_0	BB993387
E_ET_psgV_11G03_R_0	BB993388
E_ET_psgV_11G04_F_0	BB988399
E_ET_psgV_11G04_R_0	BB993389
E_ET_psgV_11G05_F_0	BB988400
E_ET_psgV_11G06_F_0	BB988401
E_ET_psgV_11G06_R_0	BB993390
E_ET_psgV_11G07_F_0	BB988402
E_ET_psgV_11G08_F_0	BB988403
E_ET_psgV_11G09_F_0	BB988404
E_ET_psgV_11G09_R_0	BB993391
E_ET_psgV_11G10_F_0	BB988405
E_ET_psgV_11G11_F_0	BB988406
E_ET_psgV_11G11_R_0	BB993392
E_ET_psgV_11G12_F_0	BB988407
E_ET_psgV_11G12_R_0	BB993393
E_ET_psgV_11H01_R_0	BB993394
E_ET_psgV_11H02_F_0	BB988408
E_ET_psgV_11H02_R_0	BB993395
E_ET_psgV_11H03_R_0	BB993396
E_ET_psgV_11H04_F_0	BB988409
E_ET_psgV_11H04_R_0	BB993397
E_ET_psgV_11H05_F_0	BB988410
E_ET_psgV_11H06_F_0	BB988411
E_ET_psgV_11H06_R_0	BB993398
E_ET_psgV_11H07_F_0	BB988412
E_ET_psgV_11H07_R_0	BB993399
E_ET_psgV_11H08_F_0	BB988413
E_ET_psgV_11H08_R_0	BB993400
E_ET_psgV_11H09_F_0	BB988414
E_ET_psgV_11H09_R_0	BB993401
E_ET_psgV_11H10_F_0	BB988415
E_ET_psgV_11H10_R_0	BB993402
E_ET_psgV_11H11_F_0	BB988416
E_ET_psgV_11H11_R_0	BB993403
E_ET_psgV_11H12_F_0	BB988417
E_ET_psgV_11H12_R_0	BB993404
E_ET_psgV_12A01_F_0	BB988418
E_ET_psgV_12A01_R_0	BB993405
E_ET_psgV_12A02_F_0	BB988419

E_ET_psgV_12A02_R_0	BB993406
E_ET_psgV_12A03_F_0	BB988420
E_ET_psgV_12A04_F_0	BB988421
E_ET_psgV_12A05_F_0	BB988422
E_ET_psgV_12A05_R_0	BB993407
E_ET_psgV_12A06_F_0	BB988423
E_ET_psgV_12A06_R_0	BB993408
E_ET_psgV_12A07_F_0	BB988424
E_ET_psgV_12A07_R_0	BB993409
E_ET_psgV_12A09_F_0	BB988425
E_ET_psgV_12A09_R_0	BB993410
E_ET_psgV_12A10_F_0	BB988426
E_ET_psgV_12A11_F_0	BB988427
E_ET_psgV_12A11_R_0	BB993411
E_ET_psgV_12A12_F_0	BB988428
E_ET_psgV_12A12_R_0	BB993412
E_ET_psgV_12B01_F_0	BB988429
E_ET_psgV_12B01_R_0	BB993413
E_ET_psgV_12B02_F_0	BB988430
E_ET_psgV_12B02_R_0	BB993414
E_ET_psgV_12B03_F_0	BB988431
E_ET_psgV_12B03_R_0	BB993415
E_ET_psgV_12B04_F_0	BB988432
E_ET_psgV_12B04_R_0	BB993416
E_ET_psgV_12B05_F_0	BB988433
E_ET_psgV_12B05_R_0	BB993417
E_ET_psgV_12B06_F_0	BB988434
E_ET_psgV_12B07_F_0	BB988435
E_ET_psgV_12B07_R_0	BB993418
E_ET_psgV_12B08_F_0	BB988436
E_ET_psgV_12B09_F_0	BB988437
E_ET_psgV_12B09_R_0	BB993419
E_ET_psgV_12B10_F_0	BB988438
E_ET_psgV_12B10_R_0	BB993420
E_ET_psgV_12B11_F_0	BB988439
E_ET_psgV_12B11_R_0	BB993421
E_ET_psgV_12B12_F_0	BB988440
E_ET_psgV_12B12_R_0	BB993422
E_ET_psgV_12C01_F_0	BB988441
E_ET_psgV_12C02_F_0	BB988442
E_ET_psgV_12C02_R_0	BB993423
E_ET_psgV_12C03_F_0	BB988443
E_ET_psgV_12C03_R_0	BB993424
E_ET_psgV_12C04_F_0	BB988444
E_ET_psgV_12C04_R_0	BB993425
E_ET_psgV_12C05_F_0	BB988445
E_ET_psgV_12C05_R_0	BB993426
E_ET_psgV_12C06_F_0	BB988446
E_ET_psgV_12C07_F_0	BB988447
E_ET_psgV_12C08_F_0	BB988448
E_ET_psgV_12C08_R_0	BB993427
E_ET_psgV_12C09_F_0	BB988449
E_ET_psgV_12C10_F_0	BB988450
E_ET_psgV_12C11_F_0	BB988451

E_ET_psgV_12C12_F_0	BB988452
E_ET_psgV_12C12_R_0	BB993428
E_ET_psgV_12D01_F_0	BB988453
E_ET_psgV_12D01_R_0	BB993429
E_ET_psgV_12D02_F_0	BB988454
E_ET_psgV_12D02_R_0	BB993430
E_ET_psgV_12D04_F_0	BB988455
E_ET_psgV_12D04_R_0	BB993431
E_ET_psgV_12D05_F_0	BB988456
E_ET_psgV_12D05_R_0	BB993432
E_ET_psgV_12D07_R_0	BB993433
E_ET_psgV_12D08_F_0	BB988457
E_ET_psgV_12D08_R_0	BB993434
E_ET_psgV_12D09_F_0	BB988458
E_ET_psgV_12D09_R_0	BB993435
E_ET_psgV_12D10_F_0	BB988459
E_ET_psgV_12D10_R_0	BB993436
E_ET_psgV_12D11_F_0	BB988460
E_ET_psgV_12D11_R_0	BB993437
E_ET_psgV_12D12_F_0	BB988461
E_ET_psgV_12D12_R_0	BB993438
E_ET_psgV_12E01_F_0	BB988462
E_ET_psgV_12E02_F_0	BB988463
E_ET_psgV_12E02_R_0	BB993439
E_ET_psgV_12E03_F_0	BB988464
E_ET_psgV_12E03_R_0	BB993440
E_ET_psgV_12E04_F_0	BB988465
E_ET_psgV_12E04_R_0	BB993441
E_ET_psgV_12E05_F_0	BB988466
E_ET_psgV_12E06_F_0	BB988467
E_ET_psgV_12E06_R_0	BB993442
E_ET_psgV_12E07_F_0	BB988468
E_ET_psgV_12E07_R_0	BB993443
E_ET_psgV_12E08_F_0	BB988469
E_ET_psgV_12E08_R_0	BB993444
E_ET_psgV_12E09_F_0	BB988470
E_ET_psgV_12E09_R_0	BB993445
E_ET_psgV_12E10_F_0	BB988471
E_ET_psgV_12E10_R_0	BB993446
E_ET_psgV_12E11_F_0	BB988472
E_ET_psgV_12E11_R_0	BB993447
E_ET_psgV_12E12_F_0	BB988473
E_ET_psgV_12E12_R_0	BB993448
E_ET_psgV_12F01_F_0	BB988474
E_ET_psgV_12F02_F_0	BB988475
E_ET_psgV_12F02_R_0	BB993449
E_ET_psgV_12F03_F_0	BB988476
E_ET_psgV_12F03_R_0	BB993450
E_ET_psgV_12F04_F_0	BB988477
E_ET_psgV_12F04_R_0	BB993451
E_ET_psgV_12F06_F_0	BB988478
E_ET_psgV_12F06_R_0	BB993452
E_ET_psgV_12F07_F_0	BB988479
E_ET_psgV_12F07_R_0	BB993453

E_ET_psgV_12F08_F_0	BB988480
E_ET_psgV_12F08_R_0	BB993454
E_ET_psgV_12F09_R_0	BB993455
E_ET_psgV_12F11_F_0	BB988481
E_ET_psgV_12F11_R_0	BB993456
E_ET_psgV_12F12_F_0	BB988482
E_ET_psgV_12F12_R_0	BB993457
E_ET_psgV_12G01_F_0	BB988483
E_ET_psgV_12G01_R_0	BB993458
E_ET_psgV_12G02_F_0	BB988484
E_ET_psgV_12G02_R_0	BB993459
E_ET_psgV_12G03_F_0	BB988485
E_ET_psgV_12G04_F_0	BB988486
E_ET_psgV_12G04_R_0	BB993460
E_ET_psgV_12G05_F_0	BB988487
E_ET_psgV_12G05_R_0	BB993461
E_ET_psgV_12G06_F_0	BB988488
E_ET_psgV_12G06_R_0	BB993462
E_ET_psgV_12G07_F_0	BB988489
E_ET_psgV_12G07_R_0	BB993463
E_ET_psgV_12G08_F_0	BB988490
E_ET_psgV_12G08_R_0	BB993464
E_ET_psgV_12G09_F_0	BB988491
E_ET_psgV_12G10_F_0	BB988492
E_ET_psgV_12G10_R_0	BB993465
E_ET_psgV_12G11_F_0	BB988493
E_ET_psgV_12G11_R_0	BB993466
E_ET_psgV_12G12_F_0	BB988494
E_ET_psgV_12G12_R_0	BB993467
E_ET_psgV_12H01_F_0	BB988495
E_ET_psgV_12H01_R_0	BB993468
E_ET_psgV_12H02_F_0	BB988496
E_ET_psgV_12H02_R_0	BB993469
E_ET_psgV_12H03_F_0	BB988497
E_ET_psgV_12H03_R_0	BB993470
E_ET_psgV_12H05_F_0	BB988498
E_ET_psgV_12H05_R_0	BB993471
E_ET_psgV_12H06_F_0	BB988499
E_ET_psgV_12H06_R_0	BB993472
E_ET_psgV_12H07_F_0	BB988500
E_ET_psgV_12H07_R_0	BB993473
E_ET_psgV_12H08_F_0	BB988501
E_ET_psgV_12H08_R_0	BB993474
E_ET_psgV_12H09_R_0	BB993475
E_ET_psgV_12H10_F_0	BB988502
E_ET_psgV_12H10_R_0	BB993476
E_ET_psgV_12H11_F_0	BB988503
E_ET_psgV_12H11_R_0	BB993477
E_ET_psgV_12H12_F_0	BB988504
E_ET_psgV_12H12_R_0	BB993478
E_ET_psgV_13A02_F_0	BB988505
E_ET_psgV_13A04_F_0	BB988506
E_ET_psgV_13A05_F_0	BB988507
E_ET_psgV_13A07_F_0	BB988508

E_ET_psgV_13A08_F_0	BB988509
E_ET_psgV_13A09_F_0	BB988510
E_ET_psgV_13A10_F_0	BB988511
E_ET_psgV_13A11_F_0	BB988512
E_ET_psgV_13A12_F_0	BB988513
E_ET_psgV_13B01_F_0	BB988514
E_ET_psgV_13B03_F_0	BB988515
E_ET_psgV_13B04_F_0	BB988516
E_ET_psgV_13B04_R_0	BB993479
E_ET_psgV_13B05_F_0	BB988517
E_ET_psgV_13B07_F_0	BB988518
E_ET_psgV_13B08_F_0	BB988519
E_ET_psgV_13B09_F_0	BB988520
E_ET_psgV_13B10_F_0	BB988521
E_ET_psgV_13B11_F_0	BB988522
E_ET_psgV_13B12_F_0	BB988523
E_ET_psgV_13C02_F_0	BB988524
E_ET_psgV_13C03_F_0	BB988525
E_ET_psgV_13C04_F_0	BB988526
E_ET_psgV_13C05_F_0	BB988527
E_ET_psgV_13C06_F_0	BB988528
E_ET_psgV_13C07_F_0	BB988529
E_ET_psgV_13C08_F_0	BB988530
E_ET_psgV_13C09_F_0	BB988531
E_ET_psgV_13C10_F_0	BB988532
E_ET_psgV_13C11_F_0	BB988533
E_ET_psgV_13C12_F_0	BB988534
E_ET_psgV_13D01_F_0	BB988535
E_ET_psgV_13D02_F_0	BB988536
E_ET_psgV_13D02_R_0	BB993480
E_ET_psgV_13D03_F_0	BB988537
E_ET_psgV_13D04_F_0	BB988538
E_ET_psgV_13D05_F_0	BB988539
E_ET_psgV_13D06_F_0	BB988540
E_ET_psgV_13D08_F_0	BB988541
E_ET_psgV_13D09_F_0	BB988542
E_ET_psgV_13D09_R_0	BB993481
E_ET_psgV_13D10_F_0	BB988543
E_ET_psgV_13D11_F_0	BB988544
E_ET_psgV_13D12_F_0	BB988545
E_ET_psgV_13E01_F_0	BB988546
E_ET_psgV_13E01_R_0	BB993482
E_ET_psgV_13E02_R_0	BB993483
E_ET_psgV_13E03_F_0	BB988547
E_ET_psgV_13E04_F_0	BB988548
E_ET_psgV_13E05_F_0	BB988549
E_ET_psgV_13E06_F_0	BB988550
E_ET_psgV_13E07_F_0	BB988551
E_ET_psgV_13E08_F_0	BB988552
E_ET_psgV_13E09_F_0	BB988553
E_ET_psgV_13E09_R_0	BB993484
E_ET_psgV_13E10_F_0	BB988554
E_ET_psgV_13E11_F_0	BB988555
E_ET_psgV_13E12_F_0	BB988556

E_ET_psgV_13F01_R_0	BB993485
E_ET_psgV_13F02_R_0	BB993486
E_ET_psgV_13F03_F_0	BB988557
E_ET_psgV_13F03_R_0	BB993487
E_ET_psgV_13F04_F_0	BB988558
E_ET_psgV_13F05_F_0	BB988559
E_ET_psgV_13F06_F_0	BB988560
E_ET_psgV_13F07_F_0	BB988561
E_ET_psgV_13F08_F_0	BB988562
E_ET_psgV_13F10_F_0	BB988563
E_ET_psgV_13F12_F_0	BB988564
E_ET_psgV_13F12_R_0	BB993488
E_ET_psgV_13G02_F_0	BB988565
E_ET_psgV_13G02_R_0	BB993489
E_ET_psgV_13G03_F_0	BB988566
E_ET_psgV_13G04_F_0	BB988567
E_ET_psgV_13G06_F_0	BB988568
E_ET_psgV_13G07_F_0	BB988569
E_ET_psgV_13G08_F_0	BB988570
E_ET_psgV_13G09_F_0	BB988571
E_ET_psgV_13G10_F_0	BB988572
E_ET_psgV_13G11_R_0	BB993490
E_ET_psgV_13G12_F_0	BB988573
E_ET_psgV_13H01_F_0	BB988574
E_ET_psgV_13H01_R_0	BB993491
E_ET_psgV_13H02_F_0	BB988575
E_ET_psgV_13H02_R_0	BB993492
E_ET_psgV_13H03_F_0	BB988576
E_ET_psgV_13H03_R_0	BB993493
E_ET_psgV_13H04_F_0	BB988577
E_ET_psgV_13H05_F_0	BB988578
E_ET_psgV_13H06_F_0	BB988579
E_ET_psgV_13H07_F_0	BB988580
E_ET_psgV_13H08_F_0	BB988581
E_ET_psgV_13H09_F_0	BB988582
E_ET_psgV_13H10_F_0	BB988583
E_ET_psgV_13H11_F_0	BB988584
E_ET_psgV_13H11_R_0	BB993494
E_ET_psgV_13H12_F_0	BB988585
E_ET_psgV_13H12_R_0	BB993495
E_ET_psgV_14A01_F_0	BB988586
E_ET_psgV_14A01_R_0	BB993496
E_ET_psgV_14A02_F_0	BB988587
E_ET_psgV_14A02_R_0	BB993497
E_ET_psgV_14A03_F_0	BB988588
E_ET_psgV_14A03_R_0	BB993498
E_ET_psgV_14A04_F_0	BB988589
E_ET_psgV_14A05_F_0	BB988590
E_ET_psgV_14A05_R_0	BB993499
E_ET_psgV_14A06_F_0	BB988591
E_ET_psgV_14A06_R_0	BB993500
E_ET_psgV_14A08_F_0	BB988592
E_ET_psgV_14A08_R_0	BB993501
E_ET_psgV_14A09_F_0	BB988593

E_ET_psgV_14A09_R_0	BB993502
E_ET_psgV_14A10_R_0	BB993503
E_ET_psgV_14A11_F_0	BB988594
E_ET_psgV_14A11_R_0	BB993504
E_ET_psgV_14A12_F_0	BB988595
E_ET_psgV_14A12_R_0	BB993505
E_ET_psgV_14B01_F_0	BB988596
E_ET_psgV_14B01_R_0	BB993506
E_ET_psgV_14B02_F_0	BB988597
E_ET_psgV_14B02_R_0	BB993507
E_ET_psgV_14B03_F_0	BB988598
E_ET_psgV_14B03_R_0	BB993508
E_ET_psgV_14B04_R_0	BB993509
E_ET_psgV_14B05_F_0	BB988599
E_ET_psgV_14B05_R_0	BB993510
E_ET_psgV_14B06_F_0	BB988600
E_ET_psgV_14B07_F_0	BB988601
E_ET_psgV_14B07_R_0	BB993511
E_ET_psgV_14B08_F_0	BB988602
E_ET_psgV_14B08_R_0	BB993512
E_ET_psgV_14B09_F_0	BB988603
E_ET_psgV_14B09_R_0	BB993513
E_ET_psgV_14B10_F_0	BB988604
E_ET_psgV_14B10_R_0	BB993514
E_ET_psgV_14B11_F_0	BB988605
E_ET_psgV_14B11_R_0	BB993515
E_ET_psgV_14B12_F_0	BB988606
E_ET_psgV_14B12_R_0	BB993516
E_ET_psgV_14C01_F_0	BB988607
E_ET_psgV_14C02_F_0	BB988608
E_ET_psgV_14C03_F_0	BB988609
E_ET_psgV_14C04_F_0	BB988610
E_ET_psgV_14C04_R_0	BB993517
E_ET_psgV_14C06_F_0	BB988611
E_ET_psgV_14C07_F_0	BB988612
E_ET_psgV_14C07_R_0	BB993518
E_ET_psgV_14C09_F_0	BB988613
E_ET_psgV_14C09_R_0	BB993519
E_ET_psgV_14C10_F_0	BB988614
E_ET_psgV_14C10_R_0	BB993520
E_ET_psgV_14C11_F_0	BB988615
E_ET_psgV_14C11_R_0	BB993521
E_ET_psgV_14C12_F_0	BB988616
E_ET_psgV_14D01_F_0	BB988617
E_ET_psgV_14D01_R_0	BB993522
E_ET_psgV_14D03_F_0	BB988618
E_ET_psgV_14D03_R_0	BB993523
E_ET_psgV_14D04_F_0	BB988619
E_ET_psgV_14D04_R_0	BB993524
E_ET_psgV_14D05_F_0	BB988620
E_ET_psgV_14D05_R_0	BB993525
E_ET_psgV_14D06_F_0	BB988621
E_ET_psgV_14D06_R_0	BB993526
E_ET_psgV_14D07_F_0	BB988622

E_ET_psgV_14D07_R_0	BB993527
E_ET_psgV_14D08_F_0	BB988623
E_ET_psgV_14D08_R_0	BB993528
E_ET_psgV_14D10_F_0	BB988624
E_ET_psgV_14D10_R_0	BB993529
E_ET_psgV_14D11_R_0	BB993530
E_ET_psgV_14D12_F_0	BB988625
E_ET_psgV_14D12_R_0	BB993531
E_ET_psgV_14E01_F_0	BB988626
E_ET_psgV_14E02_F_0	BB988627
E_ET_psgV_14E03_F_0	BB988628
E_ET_psgV_14E03_R_0	BB993532
E_ET_psgV_14E04_F_0	BB988629
E_ET_psgV_14E04_R_0	BB993533
E_ET_psgV_14E05_F_0	BB988630
E_ET_psgV_14E05_R_0	BB993534
E_ET_psgV_14E06_F_0	BB988631
E_ET_psgV_14E06_R_0	BB993535
E_ET_psgV_14E07_F_0	BB988632
E_ET_psgV_14E08_F_0	BB988633
E_ET_psgV_14E08_R_0	BB993536
E_ET_psgV_14E10_F_0	BB988634
E_ET_psgV_14E10_R_0	BB993537
E_ET_psgV_14E11_R_0	BB993538
E_ET_psgV_14E12_F_0	BB988635
E_ET_psgV_14E12_R_0	BB993539
E_ET_psgV_14F01_F_0	BB988636
E_ET_psgV_14F01_R_0	BB993540
E_ET_psgV_14F02_F_0	BB988637
E_ET_psgV_14F03_F_0	BB988638
E_ET_psgV_14F03_R_0	BB993541
E_ET_psgV_14F04_F_0	BB988639
E_ET_psgV_14F04_R_0	BB993542
E_ET_psgV_14F05_F_0	BB988640
E_ET_psgV_14F05_R_0	BB993543
E_ET_psgV_14F06_F_0	BB988641
E_ET_psgV_14F06_R_0	BB993544
E_ET_psgV_14F07_F_0	BB988642
E_ET_psgV_14F07_R_0	BB993545
E_ET_psgV_14F08_F_0	BB988643
E_ET_psgV_14F09_F_0	BB988644
E_ET_psgV_14F11_F_0	BB988645
E_ET_psgV_14F12_R_0	BB993546
E_ET_psgV_14G01_F_0	BB988646
E_ET_psgV_14G02_R_0	BB993547
E_ET_psgV_14G03_F_0	BB988647
E_ET_psgV_14G04_F_0	BB988648
E_ET_psgV_14G04_R_0	BB993548
E_ET_psgV_14G05_F_0	BB988649
E_ET_psgV_14G05_R_0	BB993549
E_ET_psgV_14G06_R_0	BB993550
E_ET_psgV_14G07_F_0	BB988650
E_ET_psgV_14G07_R_0	BB993551
E_ET_psgV_14G09_F_0	BB988651

E_ET_psgV_14G09_R_0	BB993552
E_ET_psgV_14G10_F_0	BB988652
E_ET_psgV_14G10_R_0	BB993553
E_ET_psgV_14G11_F_0	BB988653
E_ET_psgV_14G12_R_0	BB993554
E_ET_psgV_14H01_F_0	BB988654
E_ET_psgV_14H01_R_0	BB993555
E_ET_psgV_14H02_F_0	BB988655
E_ET_psgV_14H02_R_0	BB993556
E_ET_psgV_14H03_F_0	BB988656
E_ET_psgV_14H03_R_0	BB993557
E_ET_psgV_14H04_F_0	BB988657
E_ET_psgV_14H04_R_0	BB993558
E_ET_psgV_14H05_F_0	BB988658
E_ET_psgV_14H05_R_0	BB993559
E_ET_psgV_14H06_F_0	BB988659
E_ET_psgV_14H06_R_0	BB993560
E_ET_psgV_14H07_F_0	BB988660
E_ET_psgV_14H07_R_0	BB993561
E_ET_psgV_14H08_F_0	BB988661
E_ET_psgV_14H09_R_0	BB993562
E_ET_psgV_14H11_R_0	BB993563
E_ET_psgV_14H12_F_0	BB988662
E_ET_psgV_14H12_R_0	BB993564
E_ET_psgV_15A01_F_0	BB988663
E_ET_psgV_15A02_F_0	BB988664
E_ET_psgV_15A02_R_0	BB993565
E_ET_psgV_15A03_R_0	BB993566
E_ET_psgV_15A04_F_0	BB988665
E_ET_psgV_15A04_R_0	BB993567
E_ET_psgV_15A05_F_0	BB988666
E_ET_psgV_15A05_R_0	BB993568
E_ET_psgV_15A06_F_0	BB988667
E_ET_psgV_15A06_R_0	BB993569
E_ET_psgV_15A08_F_0	BB988668
E_ET_psgV_15A08_R_0	BB993570
E_ET_psgV_15A09_F_0	BB988669
E_ET_psgV_15A09_R_0	BB993571
E_ET_psgV_15A10_F_0	BB988670
E_ET_psgV_15A10_R_0	BB993572
E_ET_psgV_15A11_F_0	BB988671
E_ET_psgV_15A11_R_0	BB993573
E_ET_psgV_15A12_F_0	BB988672
E_ET_psgV_15A12_R_0	BB993574
E_ET_psgV_15B01_F_0	BB988673
E_ET_psgV_15B01_R_0	BB993575
E_ET_psgV_15B02_F_0	BB988674
E_ET_psgV_15B02_R_0	BB993576
E_ET_psgV_15B03_F_0	BB988675
E_ET_psgV_15B04_F_0	BB988676
E_ET_psgV_15B04_R_0	BB993577
E_ET_psgV_15B05_F_0	BB988677
E_ET_psgV_15B05_R_0	BB993578
E_ET_psgV_15B07_F_0	BB988678

E_ET_psgV_15B07_R_0	BB993579
E_ET_psgV_15B08_F_0	BB988679
E_ET_psgV_15B08_R_0	BB993580
E_ET_psgV_15B09_F_0	BB988680
E_ET_psgV_15B09_R_0	BB993581
E_ET_psgV_15B10_F_0	BB988681
E_ET_psgV_15B11_F_0	BB988682
E_ET_psgV_15B11_R_0	BB993582
E_ET_psgV_15B12_F_0	BB988683
E_ET_psgV_15B12_R_0	BB993583
E_ET_psgV_15C01_F_0	BB988684
E_ET_psgV_15C02_F_0	BB988685
E_ET_psgV_15C02_R_0	BB993584
E_ET_psgV_15C03_F_0	BB988686
E_ET_psgV_15C04_F_0	BB988687
E_ET_psgV_15C04_R_0	BB993585
E_ET_psgV_15C05_F_0	BB988688
E_ET_psgV_15C06_F_0	BB988689
E_ET_psgV_15C06_R_0	BB993586
E_ET_psgV_15C07_F_0	BB988690
E_ET_psgV_15C08_F_0	BB988691
E_ET_psgV_15C08_R_0	BB993587
E_ET_psgV_15C09_F_0	BB988692
E_ET_psgV_15C09_R_0	BB993588
E_ET_psgV_15C10_F_0	BB988693
E_ET_psgV_15C10_R_0	BB993589
E_ET_psgV_15C11_F_0	BB988694
E_ET_psgV_15C11_R_0	BB993590
E_ET_psgV_15C12_F_0	BB988695
E_ET_psgV_15C12_R_0	BB993591
E_ET_psgV_15D01_F_0	BB988696
E_ET_psgV_15D02_R_0	BB993592
E_ET_psgV_15D03_R_0	BB993593
E_ET_psgV_15D04_R_0	BB993594
E_ET_psgV_15D05_F_0	BB988697
E_ET_psgV_15D05_R_0	BB993595
E_ET_psgV_15D06_F_0	BB988698
E_ET_psgV_15D07_F_0	BB988699
E_ET_psgV_15D08_F_0	BB988700
E_ET_psgV_15D08_R_0	BB993596
E_ET_psgV_15D09_F_0	BB988701
E_ET_psgV_15D09_R_0	BB993597
E_ET_psgV_15D10_F_0	BB988702
E_ET_psgV_15D10_R_0	BB993598
E_ET_psgV_15D11_F_0	BB988703
E_ET_psgV_15D11_R_0	BB993599
E_ET_psgV_15D12_F_0	BB988704
E_ET_psgV_15D12_R_0	BB993600
E_ET_psgV_15E01_R_0	BB993601
E_ET_psgV_15E02_F_0	BB988705
E_ET_psgV_15E02_R_0	BB993602
E_ET_psgV_15E03_F_0	BB988706
E_ET_psgV_15E03_R_0	BB993603
E_ET_psgV_15E04_F_0	BB988707

E_ET_psgV_15E04_R_0	BB993604
E_ET_psgV_15E05_F_0	BB988708
E_ET_psgV_15E05_R_0	BB993605
E_ET_psgV_15E06_F_0	BB988709
E_ET_psgV_15E06_R_0	BB993606
E_ET_psgV_15E07_F_0	BB988710
E_ET_psgV_15E07_R_0	BB993607
E_ET_psgV_15E08_F_0	BB988711
E_ET_psgV_15E08_R_0	BB993608
E_ET_psgV_15E09_F_0	BB988712
E_ET_psgV_15E09_R_0	BB993609
E_ET_psgV_15E10_F_0	BB988713
E_ET_psgV_15E10_R_0	BB993610
E_ET_psgV_15E11_F_0	BB988714
E_ET_psgV_15E11_R_0	BB993611
E_ET_psgV_15E12_F_0	BB988715
E_ET_psgV_15F01_F_0	BB988716
E_ET_psgV_15F01_R_0	BB993612
E_ET_psgV_15F04_R_0	BB993613
E_ET_psgV_15F05_R_0	BB993614
E_ET_psgV_15F06_F_0	BB988717
E_ET_psgV_15F06_R_0	BB993615
E_ET_psgV_15F07_F_0	BB988718
E_ET_psgV_15F08_F_0	BB988719
E_ET_psgV_15F08_R_0	BB993616
E_ET_psgV_15F10_F_0	BB988720
E_ET_psgV_15F10_R_0	BB993617
E_ET_psgV_15F11_F_0	BB988721
E_ET_psgV_15F11_R_0	BB993618
E_ET_psgV_15F12_F_0	BB988722
E_ET_psgV_15G01_F_0	BB988723
E_ET_psgV_15G01_R_0	BB993619
E_ET_psgV_15G02_F_0	BB988724
E_ET_psgV_15G02_R_0	BB993620
E_ET_psgV_15G03_F_0	BB988725
E_ET_psgV_15G03_R_0	BB993621
E_ET_psgV_15G04_F_0	BB988726
E_ET_psgV_15G04_R_0	BB993622
E_ET_psgV_15G05_F_0	BB988727
E_ET_psgV_15G05_R_0	BB993623
E_ET_psgV_15G06_F_0	BB988728
E_ET_psgV_15G06_R_0	BB993624
E_ET_psgV_15G07_F_0	BB988729
E_ET_psgV_15G07_R_0	BB993625
E_ET_psgV_15G08_F_0	BB988730
E_ET_psgV_15G08_R_0	BB993626
E_ET_psgV_15G09_F_0	BB988731
E_ET_psgV_15G10_F_0	BB988732
E_ET_psgV_15G10_R_0	BB993627
E_ET_psgV_15G11_F_0	BB988733
E_ET_psgV_15G11_R_0	BB993628
E_ET_psgV_15H01_F_0	BB988734
E_ET_psgV_15H01_R_0	BB993629
E_ET_psgV_15H02_F_0	BB988735

E_ET_psgV_15H02_R_0	BB993630
E_ET_psgV_15H03_F_0	BB988736
E_ET_psgV_15H03_R_0	BB993631
E_ET_psgV_15H04_F_0	BB988737
E_ET_psgV_15H04_R_0	BB993632
E_ET_psgV_15H05_F_0	BB988738
E_ET_psgV_15H05_R_0	BB993633
E_ET_psgV_15H06_F_0	BB988739
E_ET_psgV_15H06_R_0	BB993634
E_ET_psgV_15H07_F_0	BB988740
E_ET_psgV_15H07_R_0	BB993635
E_ET_psgV_15H08_F_0	BB988741
E_ET_psgV_15H08_R_0	BB993636
E_ET_psgV_15H09_F_0	BB988742
E_ET_psgV_15H09_R_0	BB993637
E_ET_psgV_15H11_F_0	BB988743
E_ET_psgV_15H11_R_0	BB993638
E_ET_psgV_15H12_F_0	BB988744
E_ET_psgV_15H12_R_0	BB993639
E_ET_psgV_16A01_F_0	BB988745
E_ET_psgV_16A01_R_0	BB993640
E_ET_psgV_16A02_R_0	BB993641
E_ET_psgV_16A03_F_0	BB988746
E_ET_psgV_16A03_R_0	BB993642
E_ET_psgV_16A05_F_0	BB988747
E_ET_psgV_16A05_R_0	BB993643
E_ET_psgV_16A06_F_0	BB988748
E_ET_psgV_16A06_R_0	BB993644
E_ET_psgV_16A07_F_0	BB988749
E_ET_psgV_16A07_R_0	BB993645
E_ET_psgV_16A08_F_0	BB988750
E_ET_psgV_16A08_R_0	BB993646
E_ET_psgV_16A09_F_0	BB988751
E_ET_psgV_16A09_R_0	BB993647
E_ET_psgV_16A10_F_0	BB988752
E_ET_psgV_16A12_F_0	BB988753
E_ET_psgV_16B01_F_0	BB988754
E_ET_psgV_16B01_R_0	BB993648
E_ET_psgV_16B02_F_0	BB988755
E_ET_psgV_16B03_F_0	BB988756
E_ET_psgV_16B03_R_0	BB993649
E_ET_psgV_16B04_F_0	BB988757
E_ET_psgV_16B04_R_0	BB993650
E_ET_psgV_16B05_F_0	BB988758
E_ET_psgV_16B05_R_0	BB993651
E_ET_psgV_16B06_F_0	BB988759
E_ET_psgV_16B06_R_0	BB993652
E_ET_psgV_16B07_F_0	BB988760
E_ET_psgV_16B07_R_0	BB993653
E_ET_psgV_16B08_F_0	BB988761
E_ET_psgV_16B08_R_0	BB993654
E_ET_psgV_16B09_F_0	BB988762
E_ET_psgV_16B10_F_0	BB988763
E_ET_psgV_16B10_R_0	BB993655

E_ET_psgV_16B11_F_0	BB988764
E_ET_psgV_16B11_R_0	BB993656
E_ET_psgV_16C01_F_0	BB988765
E_ET_psgV_16C01_R_0	BB993657
E_ET_psgV_16C02_F_0	BB988766
E_ET_psgV_16C02_R_0	BB993658
E_ET_psgV_16C03_F_0	BB988767
E_ET_psgV_16C03_R_0	BB993659
E_ET_psgV_16C04_F_0	BB988768
E_ET_psgV_16C05_R_0	BB993660
E_ET_psgV_16C06_R_0	BB993661
E_ET_psgV_16C08_F_0	BB988769
E_ET_psgV_16C08_R_0	BB993662
E_ET_psgV_16C10_F_0	BB988770
E_ET_psgV_16C11_F_0	BB988771
E_ET_psgV_16C11_R_0	BB993663
E_ET_psgV_16C12_F_0	BB988772
E_ET_psgV_16D01_F_0	BB988773
E_ET_psgV_16D01_R_0	BB993664
E_ET_psgV_16D02_F_0	BB988774
E_ET_psgV_16D02_R_0	BB993665
E_ET_psgV_16D03_F_0	BB988775
E_ET_psgV_16D03_R_0	BB993666
E_ET_psgV_16D04_F_0	BB988776
E_ET_psgV_16D04_R_0	BB993667
E_ET_psgV_16D05_F_0	BB988777
E_ET_psgV_16D05_R_0	BB993668
E_ET_psgV_16D06_F_0	BB988778
E_ET_psgV_16D06_R_0	BB993669
E_ET_psgV_16D07_F_0	BB988779
E_ET_psgV_16D07_R_0	BB993670
E_ET_psgV_16D08_F_0	BB988780
E_ET_psgV_16D08_R_0	BB993671
E_ET_psgV_16D10_F_0	BB988781
E_ET_psgV_16D11_F_0	BB988782
E_ET_psgV_16D12_F_0	BB988783
E_ET_psgV_16E01_F_0	BB988784
E_ET_psgV_16E01_R_0	BB993672
E_ET_psgV_16E02_F_0	BB988785
E_ET_psgV_16E02_R_0	BB993673
E_ET_psgV_16E03_F_0	BB988786
E_ET_psgV_16E03_R_0	BB993674
E_ET_psgV_16E04_F_0	BB988787
E_ET_psgV_16E04_R_0	BB993675
E_ET_psgV_16E05_F_0	BB988788
E_ET_psgV_16E05_R_0	BB993676
E_ET_psgV_16E06_F_0	BB988789
E_ET_psgV_16E06_R_0	BB993677
E_ET_psgV_16E08_F_0	BB988790
E_ET_psgV_16E09_F_0	BB988791
E_ET_psgV_16E09_R_0	BB993678
E_ET_psgV_16E10_R_0	BB993679
E_ET_psgV_16E11_R_0	BB993680
E_ET_psgV_16E12_F_0	BB988792

E_ET_psgV_16E12_R_0	BB993681
E_ET_psgV_16F01_F_0	BB988793
E_ET_psgV_16F02_F_0	BB988794
E_ET_psgV_16F02_R_0	BB993682
E_ET_psgV_16F03_F_0	BB988795
E_ET_psgV_16F04_F_0	BB988796
E_ET_psgV_16F04_R_0	BB993683
E_ET_psgV_16F05_F_0	BB988797
E_ET_psgV_16F07_F_0	BB988798
E_ET_psgV_16F07_R_0	BB993684
E_ET_psgV_16F08_F_0	BB988799
E_ET_psgV_16F08_R_0	BB993685
E_ET_psgV_16F10_R_0	BB993686
E_ET_psgV_16F11_F_0	BB988800
E_ET_psgV_16F11_R_0	BB993687
E_ET_psgV_16F12_F_0	BB988801
E_ET_psgV_16F12_R_0	BB993688
E_ET_psgV_16G01_F_0	BB988802
E_ET_psgV_16G01_R_0	BB993689
E_ET_psgV_16G02_F_0	BB988803
E_ET_psgV_16G02_R_0	BB993690
E_ET_psgV_16G03_F_0	BB988804
E_ET_psgV_16G03_R_0	BB993691
E_ET_psgV_16G04_F_0	BB988805
E_ET_psgV_16G04_R_0	BB993692
E_ET_psgV_16G05_F_0	BB988806
E_ET_psgV_16G05_R_0	BB993693
E_ET_psgV_16G06_F_0	BB988807
E_ET_psgV_16G07_F_0	BB988808
E_ET_psgV_16G07_R_0	BB993694
E_ET_psgV_16G08_F_0	BB988809
E_ET_psgV_16G08_R_0	BB993695
E_ET_psgV_16G09_F_0	BB988810
E_ET_psgV_16G09_R_0	BB993696
E_ET_psgV_16G10_F_0	BB988811
E_ET_psgV_16G10_R_0	BB993697
E_ET_psgV_16G11_F_0	BB988812
E_ET_psgV_16G11_R_0	BB993698
E_ET_psgV_16H01_F_0	BB988813
E_ET_psgV_16H01_R_0	BB993699
E_ET_psgV_16H02_F_0	BB988814
E_ET_psgV_16H03_F_0	BB988815
E_ET_psgV_16H03_R_0	BB993700
E_ET_psgV_16H05_F_0	BB988816
E_ET_psgV_16H06_F_0	BB988817
E_ET_psgV_16H07_F_0	BB988818
E_ET_psgV_16H07_R_0	BB993701
E_ET_psgV_16H08_F_0	BB988819
E_ET_psgV_16H08_R_0	BB993702
E_ET_psgV_16H10_R_0	BB993703
E_ET_psgV_16H11_F_0	BB988820
E_ET_psgV_17A01_F_0	BB988821
E_ET_psgV_17A01_R_0	BB993704
E_ET_psgV_17A02_F_0	BB988822

E_ET_psgV_17A02_R_0	BB993705
E_ET_psgV_17A03_F_0	BB988823
E_ET_psgV_17A03_R_0	BB993706
E_ET_psgV_17A04_F_0	BB988824
E_ET_psgV_17A04_R_0	BB993707
E_ET_psgV_17A05_F_0	BB988825
E_ET_psgV_17A06_F_0	BB988826
E_ET_psgV_17A07_F_0	BB988827
E_ET_psgV_17A09_F_0	BB988828
E_ET_psgV_17A10_F_0	BB988829
E_ET_psgV_17A12_F_0	BB988830
E_ET_psgV_17B01_F_0	BB988831
E_ET_psgV_17B02_F_0	BB988832
E_ET_psgV_17B02_R_0	BB993708
E_ET_psgV_17B03_F_0	BB988833
E_ET_psgV_17B03_R_0	BB993709
E_ET_psgV_17B04_F_0	BB988834
E_ET_psgV_17B05_F_0	BB988835
E_ET_psgV_17B05_R_0	BB993710
E_ET_psgV_17B06_F_0	BB988836
E_ET_psgV_17B06_R_0	BB993711
E_ET_psgV_17B07_F_0	BB988837
E_ET_psgV_17B07_R_0	BB993712
E_ET_psgV_17B08_F_0	BB988838
E_ET_psgV_17B08_R_0	BB993713
E_ET_psgV_17B09_F_0	BB988839
E_ET_psgV_17B10_F_0	BB988840
E_ET_psgV_17B10_R_0	BB993714
E_ET_psgV_17B11_F_0	BB988841
E_ET_psgV_17B11_R_0	BB993715
E_ET_psgV_17B12_F_0	BB988842
E_ET_psgV_17B12_R_0	BB993716
E_ET_psgV_17C01_F_0	BB988843
E_ET_psgV_17C01_R_0	BB993717
E_ET_psgV_17C02_F_0	BB988844
E_ET_psgV_17C03_F_0	BB988845
E_ET_psgV_17C03_R_0	BB993718
E_ET_psgV_17C04_F_0	BB988846
E_ET_psgV_17C05_F_0	BB988847
E_ET_psgV_17C05_R_0	BB993719
E_ET_psgV_17C06_F_0	BB988848
E_ET_psgV_17C06_R_0	BB993720
E_ET_psgV_17C07_R_0	BB993721
E_ET_psgV_17C08_F_0	BB988849
E_ET_psgV_17C09_F_0	BB988850
E_ET_psgV_17C09_R_0	BB993722
E_ET_psgV_17C10_F_0	BB988851
E_ET_psgV_17C11_F_0	BB988852
E_ET_psgV_17C11_R_0	BB993723
E_ET_psgV_17C12_F_0	BB988853
E_ET_psgV_17C12_R_0	BB993724
E_ET_psgV_17D01_F_0	BB988854
E_ET_psgV_17D01_R_0	BB993725
E_ET_psgV_17D02_F_0	BB988855

E_ET_psgV_17D02_R_0	BB993726
E_ET_psgV_17D03_F_0	BB988856
E_ET_psgV_17D03_R_0	BB993727
E_ET_psgV_17D04_F_0	BB988857
E_ET_psgV_17D04_R_0	BB993728
E_ET_psgV_17D05_F_0	BB988858
E_ET_psgV_17D05_R_0	BB993729
E_ET_psgV_17D07_R_0	BB993730
E_ET_psgV_17D08_R_0	BB993731
E_ET_psgV_17D09_F_0	BB988859
E_ET_psgV_17D09_R_0	BB993732
E_ET_psgV_17D10_F_0	BB988860
E_ET_psgV_17D10_R_0	BB993733
E_ET_psgV_17D11_F_0	BB988861
E_ET_psgV_17D11_R_0	BB993734
E_ET_psgV_17E01_F_0	BB988862
E_ET_psgV_17E01_R_0	BB993735
E_ET_psgV_17E02_F_0	BB988863
E_ET_psgV_17E02_R_0	BB993736
E_ET_psgV_17E03_F_0	BB988864
E_ET_psgV_17E03_R_0	BB993737
E_ET_psgV_17E04_F_0	BB988865
E_ET_psgV_17E05_F_0	BB988866
E_ET_psgV_17E05_R_0	BB993738
E_ET_psgV_17E06_F_0	BB988867
E_ET_psgV_17E06_R_0	BB993739
E_ET_psgV_17E07_F_0	BB988868
E_ET_psgV_17E07_R_0	BB993740
E_ET_psgV_17E08_F_0	BB988869
E_ET_psgV_17E08_R_0	BB993741
E_ET_psgV_17E09_F_0	BB988870
E_ET_psgV_17E09_R_0	BB993742
E_ET_psgV_17E10_F_0	BB988871
E_ET_psgV_17E10_R_0	BB993743
E_ET_psgV_17E11_F_0	BB988872
E_ET_psgV_17E12_F_0	BB988873
E_ET_psgV_17E12_R_0	BB993744
E_ET_psgV_17F01_F_0	BB988874
E_ET_psgV_17F01_R_0	BB993745
E_ET_psgV_17F02_F_0	BB988875
E_ET_psgV_17F02_R_0	BB993746
E_ET_psgV_17F03_F_0	BB988876
E_ET_psgV_17F03_R_0	BB993747
E_ET_psgV_17F04_F_0	BB988877
E_ET_psgV_17F04_R_0	BB993748
E_ET_psgV_17F05_F_0	BB988878
E_ET_psgV_17F05_R_0	BB993749
E_ET_psgV_17F06_F_0	BB988879
E_ET_psgV_17F07_F_0	BB988880
E_ET_psgV_17F07_R_0	BB993750
E_ET_psgV_17F08_F_0	BB988881
E_ET_psgV_17F08_R_0	BB993751
E_ET_psgV_17F09_R_0	BB993752
E_ET_psgV_17F10_F_0	BB988882

E_ET_psgV_17F10_R_0	BB993753
E_ET_psgV_17F11_F_0	BB988883
E_ET_psgV_17F11_R_0	BB993754
E_ET_psgV_17G01_F_0	BB988884
E_ET_psgV_17G01_R_0	BB993755
E_ET_psgV_17G02_R_0	BB993756
E_ET_psgV_17G03_F_0	BB988885
E_ET_psgV_17G03_R_0	BB993757
E_ET_psgV_17G04_F_0	BB988886
E_ET_psgV_17G05_F_0	BB988887
E_ET_psgV_17G05_R_0	BB993758
E_ET_psgV_17G06_F_0	BB988888
E_ET_psgV_17G07_F_0	BB988889
E_ET_psgV_17G07_R_0	BB993759
E_ET_psgV_17G08_F_0	BB988890
E_ET_psgV_17G08_R_0	BB993760
E_ET_psgV_17G09_F_0	BB988891
E_ET_psgV_17G09_R_0	BB993761
E_ET_psgV_17G10_F_0	BB988892
E_ET_psgV_17G10_R_0	BB993762
E_ET_psgV_17G11_F_0	BB988893
E_ET_psgV_17H01_F_0	BB988894
E_ET_psgV_17H01_R_0	BB993763
E_ET_psgV_17H02_F_0	BB988895
E_ET_psgV_17H03_F_0	BB988896
E_ET_psgV_17H03_R_0	BB993764
E_ET_psgV_17H04_F_0	BB988897
E_ET_psgV_17H05_F_0	BB988898
E_ET_psgV_17H05_R_0	BB993765
E_ET_psgV_17H06_F_0	BB988899
E_ET_psgV_17H07_F_0	BB988900
E_ET_psgV_17H08_F_0	BB988901
E_ET_psgV_17H09_F_0	BB988902
E_ET_psgV_17H09_R_0	BB993766
E_ET_psgV_17H10_F_0	BB988903
E_ET_psgV_17H11_F_0	BB988904
E_ET_psgV_17H11_R_0	BB993767
E_ET_psgV_17H12_F_0	BB988905
E_ET_psgV_17H12_R_0	BB993768
E_ET_psgV_18A01_F_0	BB988906
E_ET_psgV_18A01_R_0	BB993769
E_ET_psgV_18A03_F_0	BB988907
E_ET_psgV_18A03_R_0	BB993770
E_ET_psgV_18A04_F_0	BB988908
E_ET_psgV_18A04_R_0	BB993771
E_ET_psgV_18A05_F_0	BB988909
E_ET_psgV_18A05_R_0	BB993772
E_ET_psgV_18A07_F_0	BB988910
E_ET_psgV_18A07_R_0	BB993773
E_ET_psgV_18A08_F_0	BB988911
E_ET_psgV_18A08_R_0	BB993774
E_ET_psgV_18A10_F_0	BB988912
E_ET_psgV_18A10_R_0	BB993775
E_ET_psgV_18A11_F_0	BB988913

E_ET_psgV_18A11_R_0	BB993776
E_ET_psgV_18B01_F_0	BB988914
E_ET_psgV_18B01_R_0	BB993777
E_ET_psgV_18B02_R_0	BB993778
E_ET_psgV_18B03_F_0	BB988915
E_ET_psgV_18B03_R_0	BB993779
E_ET_psgV_18B04_R_0	BB993780
E_ET_psgV_18B05_R_0	BB993781
E_ET_psgV_18B06_F_0	BB988916
E_ET_psgV_18B06_R_0	BB993782
E_ET_psgV_18B08_F_0	BB988917
E_ET_psgV_18B08_R_0	BB993783
E_ET_psgV_18B09_F_0	BB988918
E_ET_psgV_18B09_R_0	BB993784
E_ET_psgV_18B10_F_0	BB988919
E_ET_psgV_18B11_F_0	BB988920
E_ET_psgV_18B11_R_0	BB993785
E_ET_psgV_18B12_F_0	BB988921
E_ET_psgV_18B12_R_0	BB993786
E_ET_psgV_18C01_F_0	BB988922
E_ET_psgV_18C01_R_0	BB993787
E_ET_psgV_18C02_F_0	BB988923
E_ET_psgV_18C02_R_0	BB993788
E_ET_psgV_18C03_F_0	BB988924
E_ET_psgV_18C03_R_0	BB993789
E_ET_psgV_18C04_F_0	BB988925
E_ET_psgV_18C05_F_0	BB988926
E_ET_psgV_18C05_R_0	BB993790
E_ET_psgV_18C06_F_0	BB988927
E_ET_psgV_18C07_F_0	BB988928
E_ET_psgV_18C07_R_0	BB993791
E_ET_psgV_18C08_F_0	BB988929
E_ET_psgV_18C08_R_0	BB993792
E_ET_psgV_18C09_F_0	BB988930
E_ET_psgV_18C11_F_0	BB988931
E_ET_psgV_18C11_R_0	BB993793
E_ET_psgV_18C12_F_0	BB988932
E_ET_psgV_18C12_R_0	BB993794
E_ET_psgV_18D01_F_0	BB988933
E_ET_psgV_18D02_F_0	BB988934
E_ET_psgV_18D02_R_0	BB993795
E_ET_psgV_18D03_F_0	BB988935
E_ET_psgV_18D03_R_0	BB993796
E_ET_psgV_18D05_F_0	BB988936
E_ET_psgV_18D05_R_0	BB993797
E_ET_psgV_18D06_F_0	BB988937
E_ET_psgV_18D06_R_0	BB993798
E_ET_psgV_18D07_F_0	BB988938
E_ET_psgV_18D08_F_0	BB988939
E_ET_psgV_18D08_R_0	BB993799
E_ET_psgV_18D09_F_0	BB988940
E_ET_psgV_18D10_R_0	BB993800
E_ET_psgV_18D12_F_0	BB988941
E_ET_psgV_18D12_R_0	BB993801

E_ET_psgV_18E01_F_0	BB988942
E_ET_psgV_18E01_R_0	BB993802
E_ET_psgV_18E02_F_0	BB988943
E_ET_psgV_18E03_F_0	BB988944
E_ET_psgV_18E03_R_0	BB993803
E_ET_psgV_18E04_F_0	BB988945
E_ET_psgV_18E04_R_0	BB993804
E_ET_psgV_18E05_F_0	BB988946
E_ET_psgV_18E05_R_0	BB993805
E_ET_psgV_18E06_F_0	BB988947
E_ET_psgV_18E07_R_0	BB993806
E_ET_psgV_18E08_F_0	BB988948
E_ET_psgV_18E08_R_0	BB993807
E_ET_psgV_18E09_F_0	BB988949
E_ET_psgV_18E09_R_0	BB993808
E_ET_psgV_18E10_F_0	BB988950
E_ET_psgV_18E10_R_0	BB993809
E_ET_psgV_18E11_F_0	BB988951
E_ET_psgV_18E12_F_0	BB988952
E_ET_psgV_18E12_R_0	BB993810
E_ET_psgV_18F01_F_0	BB988953
E_ET_psgV_18F01_R_0	BB993811
E_ET_psgV_18F02_F_0	BB988954
E_ET_psgV_18F02_R_0	BB993812
E_ET_psgV_18F03_F_0	BB988955
E_ET_psgV_18F03_R_0	BB993813
E_ET_psgV_18F04_F_0	BB988956
E_ET_psgV_18F05_F_0	BB988957
E_ET_psgV_18F05_R_0	BB993814
E_ET_psgV_18F06_F_0	BB988958
E_ET_psgV_18F06_R_0	BB993815
E_ET_psgV_18F08_F_0	BB988959
E_ET_psgV_18F08_R_0	BB993816
E_ET_psgV_18F09_F_0	BB988960
E_ET_psgV_18F09_R_0	BB993817
E_ET_psgV_18F10_F_0	BB988961
E_ET_psgV_18F10_R_0	BB993818
E_ET_psgV_18F11_F_0	BB988962
E_ET_psgV_18F11_R_0	BB993819
E_ET_psgV_18F12_F_0	BB988963
E_ET_psgV_18F12_R_0	BB993820
E_ET_psgV_18G01_F_0	BB988964
E_ET_psgV_18G01_R_0	BB993821
E_ET_psgV_18G02_F_0	BB988965
E_ET_psgV_18G02_R_0	BB993822
E_ET_psgV_18G03_F_0	BB988966
E_ET_psgV_18G03_R_0	BB993823
E_ET_psgV_18G04_F_0	BB988967
E_ET_psgV_18G04_R_0	BB993824
E_ET_psgV_18G05_F_0	BB988968
E_ET_psgV_18G05_R_0	BB993825
E_ET_psgV_18G06_F_0	BB988969
E_ET_psgV_18G06_R_0	BB993826
E_ET_psgV_18G07_F_0	BB988970

E_ET_psgV_18G07_R_0	BB993827
E_ET_psgV_18G08_F_0	BB988971
E_ET_psgV_18G09_F_0	BB988972
E_ET_psgV_18G09_R_0	BB993828
E_ET_psgV_18G10_F_0	BB988973
E_ET_psgV_18G11_F_0	BB988974
E_ET_psgV_18G11_R_0	BB993829
E_ET_psgV_18G12_R_0	BB993830
E_ET_psgV_18H01_F_0	BB988975
E_ET_psgV_18H01_R_0	BB993831
E_ET_psgV_18H02_F_0	BB988976
E_ET_psgV_18H02_R_0	BB993832
E_ET_psgV_18H03_F_0	BB988977
E_ET_psgV_18H03_R_0	BB993833
E_ET_psgV_18H04_F_0	BB988978
E_ET_psgV_18H04_R_0	BB993834
E_ET_psgV_18H05_F_0	BB988979
E_ET_psgV_18H05_R_0	BB993835
E_ET_psgV_18H06_F_0	BB988980
E_ET_psgV_18H06_R_0	BB993836
E_ET_psgV_18H07_F_0	BB988981
E_ET_psgV_18H07_R_0	BB993837
E_ET_psgV_18H08_F_0	BB988982
E_ET_psgV_18H08_R_0	BB993838
E_ET_psgV_18H10_F_0	BB988983
E_ET_psgV_18H10_R_0	BB993839
E_ET_psgV_18H11_F_0	BB988984
E_ET_psgV_18H11_R_0	BB993840
E_ET_psgV_18H12_F_0	BB988985
E_ET_psgV_18H12_R_0	BB993841
E_ET_psgV_19A02_F_0	BB988986
E_ET_psgV_19A03_F_0	BB988987
E_ET_psgV_19A04_R_0	BB993842
E_ET_psgV_19A05_F_0	BB988988
E_ET_psgV_19A05_R_0	BB993843
E_ET_psgV_19A06_F_0	BB988989
E_ET_psgV_19A07_F_0	BB988990
E_ET_psgV_19A07_R_0	BB993844
E_ET_psgV_19A08_F_0	BB988991
E_ET_psgV_19A09_F_0	BB988992
E_ET_psgV_19A10_F_0	BB988993
E_ET_psgV_19A11_F_0	BB988994
E_ET_psgV_19A12_F_0	BB988995
E_ET_psgV_19A12_R_0	BB993845
E_ET_psgV_19B01_F_0	BB988996
E_ET_psgV_19B01_R_0	BB993846
E_ET_psgV_19B02_F_0	BB988997
E_ET_psgV_19B02_R_0	BB993847
E_ET_psgV_19B03_R_0	BB993848
E_ET_psgV_19B04_F_0	BB988998
E_ET_psgV_19B04_R_0	BB993849
E_ET_psgV_19B05_F_0	BB988999
E_ET_psgV_19B05_R_0	BB993850
E_ET_psgV_19B06_F_0	BB989000

E_ET_psgV_19B07_F_0	BB989001
E_ET_psgV_19B08_F_0	BB989002
E_ET_psgV_19B09_F_0	BB989003
E_ET_psgV_19B09_R_0	BB993851
E_ET_psgV_19B10_F_0	BB989004
E_ET_psgV_19B10_R_0	BB993852
E_ET_psgV_19B11_F_0	BB989005
E_ET_psgV_19B12_F_0	BB989006
E_ET_psgV_19B12_R_0	BB993853
E_ET_psgV_19C01_F_0	BB989007
E_ET_psgV_19C03_F_0	BB989008
E_ET_psgV_19C03_R_0	BB993854
E_ET_psgV_19C04_F_0	BB989009
E_ET_psgV_19C04_R_0	BB993855
E_ET_psgV_19C05_F_0	BB989010
E_ET_psgV_19C05_R_0	BB993856
E_ET_psgV_19C06_F_0	BB989011
E_ET_psgV_19C06_R_0	BB993857
E_ET_psgV_19C07_F_0	BB989012
E_ET_psgV_19C07_R_0	BB993858
E_ET_psgV_19C08_F_0	BB989013
E_ET_psgV_19C09_F_0	BB989014
E_ET_psgV_19C09_R_0	BB993859
E_ET_psgV_19C10_F_0	BB989015
E_ET_psgV_19C11_F_0	BB989016
E_ET_psgV_19C12_F_0	BB989017
E_ET_psgV_19C12_R_0	BB993860
E_ET_psgV_19D01_F_0	BB989018
E_ET_psgV_19D02_F_0	BB989019
E_ET_psgV_19D03_F_0	BB989020
E_ET_psgV_19D03_R_0	BB993861
E_ET_psgV_19D04_F_0	BB989021
E_ET_psgV_19D05_F_0	BB989022
E_ET_psgV_19D05_R_0	BB993862
E_ET_psgV_19D06_F_0	BB989023
E_ET_psgV_19D06_R_0	BB993863
E_ET_psgV_19D07_F_0	BB989024
E_ET_psgV_19D07_R_0	BB993864
E_ET_psgV_19D08_F_0	BB989025
E_ET_psgV_19D08_R_0	BB993865
E_ET_psgV_19D09_F_0	BB989026
E_ET_psgV_19D09_R_0	BB993866
E_ET_psgV_19D10_F_0	BB989027
E_ET_psgV_19D10_R_0	BB993867
E_ET_psgV_19D11_F_0	BB989028
E_ET_psgV_19D11_R_0	BB993868
E_ET_psgV_19D12_F_0	BB989029
E_ET_psgV_19D12_R_0	BB993869
E_ET_psgV_19E01_F_0	BB989030
E_ET_psgV_19E01_R_0	BB993870
E_ET_psgV_19E02_F_0	BB989031
E_ET_psgV_19E03_F_0	BB989032
E_ET_psgV_19E03_R_0	BB993871
E_ET_psgV_19E04_F_0	BB989033

E_ET_psgV_19E04_R_0	BB993872
E_ET_psgV_19E05_F_0	BB989034
E_ET_psgV_19E05_R_0	BB993873
E_ET_psgV_19E06_R_0	BB993874
E_ET_psgV_19E07_F_0	BB989035
E_ET_psgV_19E08_F_0	BB989036
E_ET_psgV_19E08_R_0	BB993875
E_ET_psgV_19E09_F_0	BB989037
E_ET_psgV_19E09_R_0	BB993876
E_ET_psgV_19E10_F_0	BB989038
E_ET_psgV_19E10_R_0	BB993877
E_ET_psgV_19E11_F_0	BB989039
E_ET_psgV_19E11_R_0	BB993878
E_ET_psgV_19E12_F_0	BB989040
E_ET_psgV_19F01_F_0	BB989041
E_ET_psgV_19F01_R_0	BB993879
E_ET_psgV_19F02_F_0	BB989042
E_ET_psgV_19F02_R_0	BB993880
E_ET_psgV_19F03_F_0	BB989043
E_ET_psgV_19F03_R_0	BB993881
E_ET_psgV_19F04_F_0	BB989044
E_ET_psgV_19F04_R_0	BB993882
E_ET_psgV_19F05_F_0	BB989045
E_ET_psgV_19F05_R_0	BB993883
E_ET_psgV_19F06_F_0	BB989046
E_ET_psgV_19F06_R_0	BB993884
E_ET_psgV_19F07_F_0	BB989047
E_ET_psgV_19F07_R_0	BB993885
E_ET_psgV_19F08_F_0	BB989048
E_ET_psgV_19F08_R_0	BB993886
E_ET_psgV_19F09_F_0	BB989049
E_ET_psgV_19F09_R_0	BB993887
E_ET_psgV_19F10_F_0	BB989050
E_ET_psgV_19F10_R_0	BB993888
E_ET_psgV_19F11_F_0	BB989051
E_ET_psgV_19F11_R_0	BB993889
E_ET_psgV_19F12_R_0	BB993890
E_ET_psgV_19G01_F_0	BB989052
E_ET_psgV_19G01_R_0	BB993891
E_ET_psgV_19G02_F_0	BB989053
E_ET_psgV_19G02_R_0	BB993892
E_ET_psgV_19G03_F_0	BB989054
E_ET_psgV_19G03_R_0	BB993893
E_ET_psgV_19G04_F_0	BB989055
E_ET_psgV_19G04_R_0	BB993894
E_ET_psgV_19G05_F_0	BB989056
E_ET_psgV_19G05_R_0	BB993895
E_ET_psgV_19G06_F_0	BB989057
E_ET_psgV_19G07_F_0	BB989058
E_ET_psgV_19G07_R_0	BB993896
E_ET_psgV_19G08_F_0	BB989059
E_ET_psgV_19G08_R_0	BB993897
E_ET_psgV_19G09_F_0	BB989060
E_ET_psgV_19G09_R_0	BB993898

E_ET_psgV_19G10_F_0	BB989061
E_ET_psgV_19G11_F_0	BB989062
E_ET_psgV_19G11_R_0	BB993899
E_ET_psgV_19G12_F_0	BB989063
E_ET_psgV_19G12_R_0	BB993900
E_ET_psgV_19H01_F_0	BB989064
E_ET_psgV_19H01_R_0	BB993901
E_ET_psgV_19H02_R_0	BB993902
E_ET_psgV_19H03_F_0	BB989065
E_ET_psgV_19H03_R_0	BB993903
E_ET_psgV_19H04_F_0	BB989066
E_ET_psgV_19H04_R_0	BB993904
E_ET_psgV_19H05_R_0	BB993905
E_ET_psgV_19H06_R_0	BB993906
E_ET_psgV_19H07_F_0	BB989067
E_ET_psgV_19H07_R_0	BB993907
E_ET_psgV_19H08_F_0	BB989068
E_ET_psgV_19H08_R_0	BB993908
E_ET_psgV_19H10_F_0	BB989069
E_ET_psgV_19H11_F_0	BB989070
E_ET_psgV_19H11_R_0	BB993909
E_ET_psgV_20A01_R_0	BB993910
E_ET_psgV_20A02_F_0	BB989071
E_ET_psgV_20A02_R_0	BB993911
E_ET_psgV_20A03_F_0	BB989072
E_ET_psgV_20A03_R_0	BB993912
E_ET_psgV_20A04_F_0	BB989073
E_ET_psgV_20A04_R_0	BB993913
E_ET_psgV_20A05_F_0	BB989074
E_ET_psgV_20A05_R_0	BB993914
E_ET_psgV_20A06_F_0	BB989075
E_ET_psgV_20A06_R_0	BB993915
E_ET_psgV_20A07_F_0	BB989076
E_ET_psgV_20A07_R_0	BB993916
E_ET_psgV_20A08_F_0	BB989077
E_ET_psgV_20A09_R_0	BB993917
E_ET_psgV_20A10_F_0	BB989078
E_ET_psgV_20A11_R_0	BB993918
E_ET_psgV_20A12_F_0	BB989079
E_ET_psgV_20B01_F_0	BB989080
E_ET_psgV_20B01_R_0	BB993919
E_ET_psgV_20B02_F_0	BB989081
E_ET_psgV_20B02_R_0	BB993920
E_ET_psgV_20B03_F_0	BB989082
E_ET_psgV_20B03_R_0	BB993921
E_ET_psgV_20B04_F_0	BB989083
E_ET_psgV_20B04_R_0	BB993922
E_ET_psgV_20B05_F_0	BB989084
E_ET_psgV_20B06_F_0	BB989085
E_ET_psgV_20B06_R_0	BB993923
E_ET_psgV_20B08_F_0	BB989086
E_ET_psgV_20B08_R_0	BB993924
E_ET_psgV_20B09_F_0	BB989087
E_ET_psgV_20B09_R_0	BB993925

E_ET_psgV_20B10_F_0	BB989088
E_ET_psgV_20B10_R_0	BB993926
E_ET_psgV_20B11_F_0	BB989089
E_ET_psgV_20B11_R_0	BB993927
E_ET_psgV_20C01_R_0	BB993928
E_ET_psgV_20C02_F_0	BB989090
E_ET_psgV_20C02_R_0	BB993929
E_ET_psgV_20C03_F_0	BB989091
E_ET_psgV_20C03_R_0	BB993930
E_ET_psgV_20C04_F_0	BB989092
E_ET_psgV_20C05_F_0	BB989093
E_ET_psgV_20C06_F_0	BB989094
E_ET_psgV_20C06_R_0	BB993931
E_ET_psgV_20C07_F_0	BB989095
E_ET_psgV_20C08_F_0	BB989096
E_ET_psgV_20C08_R_0	BB993932
E_ET_psgV_20C09_F_0	BB989097
E_ET_psgV_20C09_R_0	BB993933
E_ET_psgV_20C10_F_0	BB989098
E_ET_psgV_20C10_R_0	BB993934
E_ET_psgV_20C12_F_0	BB989099
E_ET_psgV_20C12_R_0	BB993935
E_ET_psgV_20D02_F_0	BB989100
E_ET_psgV_20D02_R_0	BB993936
E_ET_psgV_20D03_R_0	BB993937
E_ET_psgV_20D04_F_0	BB989101
E_ET_psgV_20D04_R_0	BB993938
E_ET_psgV_20D05_F_0	BB989102
E_ET_psgV_20D05_R_0	BB993939
E_ET_psgV_20D06_F_0	BB989103
E_ET_psgV_20D06_R_0	BB993940
E_ET_psgV_20D07_F_0	BB989104
E_ET_psgV_20D07_R_0	BB993941
E_ET_psgV_20D08_F_0	BB989105
E_ET_psgV_20D08_R_0	BB993942
E_ET_psgV_20D09_F_0	BB989106
E_ET_psgV_20D09_R_0	BB993943
E_ET_psgV_20D10_F_0	BB989107
E_ET_psgV_20D10_R_0	BB993944
E_ET_psgV_20D11_F_0	BB989108
E_ET_psgV_20D11_R_0	BB993945
E_ET_psgV_20D12_F_0	BB989109
E_ET_psgV_20D12_R_0	BB993946
E_ET_psgV_20E01_F_0	BB989110
E_ET_psgV_20E01_R_0	BB993947
E_ET_psgV_20E02_F_0	BB989111
E_ET_psgV_20E02_R_0	BB993948
E_ET_psgV_20E03_F_0	BB989112
E_ET_psgV_20E03_R_0	BB993949
E_ET_psgV_20E04_F_0	BB989113
E_ET_psgV_20E04_R_0	BB993950
E_ET_psgV_20E05_F_0	BB989114
E_ET_psgV_20E05_R_0	BB993951
E_ET_psgV_20E07_F_0	BB989115

E_ET_psgV_20E07_R_0	BB993952
E_ET_psgV_20E08_F_0	BB989116
E_ET_psgV_20E08_R_0	BB993953
E_ET_psgV_20E09_F_0	BB989117
E_ET_psgV_20E09_R_0	BB993954
E_ET_psgV_20E10_F_0	BB989118
E_ET_psgV_20E10_R_0	BB993955
E_ET_psgV_20E11_F_0	BB989119
E_ET_psgV_20E11_R_0	BB993956
E_ET_psgV_20E12_F_0	BB989120
E_ET_psgV_20E12_R_0	BB993957
E_ET_psgV_20F01_F_0	BB989121
E_ET_psgV_20F01_R_0	BB993958
E_ET_psgV_20F02_F_0	BB989122
E_ET_psgV_20F02_R_0	BB993959
E_ET_psgV_20F03_F_0	BB989123
E_ET_psgV_20F03_R_0	BB993960
E_ET_psgV_20F04_F_0	BB989124
E_ET_psgV_20F04_R_0	BB993961
E_ET_psgV_20F05_F_0	BB989125
E_ET_psgV_20F05_R_0	BB993962
E_ET_psgV_20F06_F_0	BB989126
E_ET_psgV_20F06_R_0	BB993963
E_ET_psgV_20F07_F_0	BB989127
E_ET_psgV_20F07_R_0	BB993964
E_ET_psgV_20F08_F_0	BB989128
E_ET_psgV_20F08_R_0	BB993965
E_ET_psgV_20F10_F_0	BB989129
E_ET_psgV_20F10_R_0	BB993966
E_ET_psgV_20F11_F_0	BB989130
E_ET_psgV_20F11_R_0	BB993967
E_ET_psgV_20G01_F_0	BB989131
E_ET_psgV_20G01_R_0	BB993968
E_ET_psgV_20G02_F_0	BB989132
E_ET_psgV_20G02_R_0	BB993969
E_ET_psgV_20G03_F_0	BB989133
E_ET_psgV_20G04_F_0	BB989134
E_ET_psgV_20G04_R_0	BB993970
E_ET_psgV_20G05_F_0	BB989135
E_ET_psgV_20G05_R_0	BB993971
E_ET_psgV_20G06_F_0	BB989136
E_ET_psgV_20G07_F_0	BB989137
E_ET_psgV_20G07_R_0	BB993972
E_ET_psgV_20G08_F_0	BB989138
E_ET_psgV_20G08_R_0	BB993973
E_ET_psgV_20G09_F_0	BB989139
E_ET_psgV_20G10_F_0	BB989140
E_ET_psgV_20G10_R_0	BB993974
E_ET_psgV_20G11_F_0	BB989141
E_ET_psgV_20G12_F_0	BB989142
E_ET_psgV_20H01_F_0	BB989143
E_ET_psgV_20H01_R_0	BB993975
E_ET_psgV_20H02_F_0	BB989144
E_ET_psgV_20H02_R_0	BB993976

E_ET_psgV_20H03_F_0	BB989145
E_ET_psgV_20H03_R_0	BB993977
E_ET_psgV_20H04_F_0	BB989146
E_ET_psgV_20H05_F_0	BB989147
E_ET_psgV_20H05_R_0	BB993978
E_ET_psgV_20H06_F_0	BB989148
E_ET_psgV_20H06_R_0	BB993979
E_ET_psgV_20H07_F_0	BB989149
E_ET_psgV_20H07_R_0	BB993980
E_ET_psgV_20H08_F_0	BB989150
E_ET_psgV_20H08_R_0	BB993981
E_ET_psgV_20H09_F_0	BB989151
E_ET_psgV_20H10_F_0	BB989152
E_ET_psgV_20H10_R_0	BB993982
E_ET_psgV_20H12_F_0	BB989153
E_ET_psgV_20H12_R_0	BB993983
E_ET_psgV_21A01_R_0	BB993984
E_ET_psgV_21A02_R_0	BB993985
E_ET_psgV_21A05_F_0	BB989154
E_ET_psgV_21A05_R_0	BB993986
E_ET_psgV_21A06_F_0	BB989155
E_ET_psgV_21A06_R_0	BB993987
E_ET_psgV_21A07_F_0	BB989156
E_ET_psgV_21A08_F_0	BB989157
E_ET_psgV_21A08_R_0	BB993988
E_ET_psgV_21A10_F_0	BB989158
E_ET_psgV_21A10_R_0	BB993989
E_ET_psgV_21A11_F_0	BB989159
E_ET_psgV_21A12_F_0	BB989160
E_ET_psgV_21A12_R_0	BB993990
E_ET_psgV_21B02_F_0	BB989161
E_ET_psgV_21B02_R_0	BB993991
E_ET_psgV_21B03_F_0	BB989162
E_ET_psgV_21B03_R_0	BB993992
E_ET_psgV_21B04_F_0	BB989163
E_ET_psgV_21B04_R_0	BB993993
E_ET_psgV_21B05_F_0	BB989164
E_ET_psgV_21B05_R_0	BB993994
E_ET_psgV_21B06_F_0	BB989165
E_ET_psgV_21B06_R_0	BB993995
E_ET_psgV_21B07_F_0	BB989166
E_ET_psgV_21B07_R_0	BB993996
E_ET_psgV_21B08_F_0	BB989167
E_ET_psgV_21B08_R_0	BB993997
E_ET_psgV_21B09_F_0	BB989168
E_ET_psgV_21B09_R_0	BB993998
E_ET_psgV_21B10_F_0	BB989169
E_ET_psgV_21B10_R_0	BB993999
E_ET_psgV_21B11_F_0	BB989170
E_ET_psgV_21B12_F_0	BB989171
E_ET_psgV_21C01_F_0	BB989172
E_ET_psgV_21C01_R_0	BB994000
E_ET_psgV_21C02_F_0	BB989173
E_ET_psgV_21C02_R_0	BB994001

E_ET_psgV_21C04_F_0	BB989174
E_ET_psgV_21C04_R_0	BB994002
E_ET_psgV_21C05_F_0	BB989175
E_ET_psgV_21C05_R_0	BB994003
E_ET_psgV_21C06_F_0	BB989176
E_ET_psgV_21C06_R_0	BB994004
E_ET_psgV_21C07_F_0	BB989177
E_ET_psgV_21C07_R_0	BB994005
E_ET_psgV_21C09_F_0	BB989178
E_ET_psgV_21C09_R_0	BB994006
E_ET_psgV_21C10_F_0	BB989179
E_ET_psgV_21C10_R_0	BB994007
E_ET_psgV_21C11_F_0	BB989180
E_ET_psgV_21C12_F_0	BB989181
E_ET_psgV_21C12_R_0	BB994008
E_ET_psgV_21D01_F_0	BB989182
E_ET_psgV_21D01_R_0	BB994009
E_ET_psgV_21D02_F_0	BB989183
E_ET_psgV_21D02_R_0	BB994010
E_ET_psgV_21D03_F_0	BB989184
E_ET_psgV_21D04_F_0	BB989185
E_ET_psgV_21D04_R_0	BB994011
E_ET_psgV_21D05_F_0	BB989186
E_ET_psgV_21D06_F_0	BB989187
E_ET_psgV_21D06_R_0	BB994012
E_ET_psgV_21D07_F_0	BB989188
E_ET_psgV_21D07_R_0	BB994013
E_ET_psgV_21D08_F_0	BB989189
E_ET_psgV_21D08_R_0	BB994014
E_ET_psgV_21D09_F_0	BB989190
E_ET_psgV_21D09_R_0	BB994015
E_ET_psgV_21D10_F_0	BB989191
E_ET_psgV_21D10_R_0	BB994016
E_ET_psgV_21D11_F_0	BB989192
E_ET_psgV_21D12_R_0	BB994017
E_ET_psgV_21E01_F_0	BB989193
E_ET_psgV_21E01_R_0	BB994018
E_ET_psgV_21E02_R_0	BB994019
E_ET_psgV_21E03_F_0	BB989194
E_ET_psgV_21E03_R_0	BB994020
E_ET_psgV_21E04_F_0	BB989195
E_ET_psgV_21E05_F_0	BB989196
E_ET_psgV_21E06_F_0	BB989197
E_ET_psgV_21E08_R_0	BB994021
E_ET_psgV_21E09_F_0	BB989198
E_ET_psgV_21E11_F_0	BB989199
E_ET_psgV_21E11_R_0	BB994022
E_ET_psgV_21E12_F_0	BB989200
E_ET_psgV_21E12_R_0	BB994023
E_ET_psgV_21F01_F_0	BB989201
E_ET_psgV_21F01_R_0	BB994024
E_ET_psgV_21F02_F_0	BB989202
E_ET_psgV_21F03_F_0	BB989203
E_ET_psgV_21F03_R_0	BB994025

E_ET_psgV_21F04_F_0	BB989204
E_ET_psgV_21F04_R_0	BB994026
E_ET_psgV_21F05_F_0	BB989205
E_ET_psgV_21F05_R_0	BB994027
E_ET_psgV_21F06_F_0	BB989206
E_ET_psgV_21F06_R_0	BB994028
E_ET_psgV_21F07_F_0	BB989207
E_ET_psgV_21F08_F_0	BB989208
E_ET_psgV_21F08_R_0	BB994029
E_ET_psgV_21F09_F_0	BB989209
E_ET_psgV_21F09_R_0	BB994030
E_ET_psgV_21F10_F_0	BB989210
E_ET_psgV_21F11_F_0	BB989211
E_ET_psgV_21F11_R_0	BB994031
E_ET_psgV_21F12_F_0	BB989212
E_ET_psgV_21F12_R_0	BB994032
E_ET_psgV_21G01_F_0	BB989213
E_ET_psgV_21G02_F_0	BB989214
E_ET_psgV_21G02_R_0	BB994033
E_ET_psgV_21G03_F_0	BB989215
E_ET_psgV_21G03_R_0	BB994034
E_ET_psgV_21G04_F_0	BB989216
E_ET_psgV_21G04_R_0	BB994035
E_ET_psgV_21G05_F_0	BB989217
E_ET_psgV_21G05_R_0	BB994036
E_ET_psgV_21G06_F_0	BB989218
E_ET_psgV_21G06_R_0	BB994037
E_ET_psgV_21G07_F_0	BB989219
E_ET_psgV_21G08_F_0	BB989220
E_ET_psgV_21G08_R_0	BB994038
E_ET_psgV_21G09_F_0	BB989221
E_ET_psgV_21G09_R_0	BB994039
E_ET_psgV_21G10_F_0	BB989222
E_ET_psgV_21G11_F_0	BB989223
E_ET_psgV_21G11_R_0	BB994040
E_ET_psgV_21G12_F_0	BB989224
E_ET_psgV_21G12_R_0	BB994041
E_ET_psgV_21H01_F_0	BB989225
E_ET_psgV_21H01_R_0	BB994042
E_ET_psgV_21H02_F_0	BB989226
E_ET_psgV_21H02_R_0	BB994043
E_ET_psgV_21H03_F_0	BB989227
E_ET_psgV_21H04_F_0	BB989228
E_ET_psgV_21H05_F_0	BB989229
E_ET_psgV_21H05_R_0	BB994044
E_ET_psgV_21H06_F_0	BB989230
E_ET_psgV_21H07_F_0	BB989231
E_ET_psgV_21H07_R_0	BB994045
E_ET_psgV_21H08_F_0	BB989232
E_ET_psgV_21H10_F_0	BB989233
E_ET_psgV_21H11_F_0	BB989234
E_ET_psgV_21H12_F_0	BB989235
E_ET_psgV_22A01_F_0	BB989236
E_ET_psgV_22A01_R_0	BB994046

E_ET_psgV_22A02_F_0	BB989237
E_ET_psgV_22A04_F_0	BB989238
E_ET_psgV_22A04_R_0	BB994047
E_ET_psgV_22A05_F_0	BB989239
E_ET_psgV_22A05_R_0	BB994048
E_ET_psgV_22A06_F_0	BB989240
E_ET_psgV_22A06_R_0	BB994049
E_ET_psgV_22A07_F_0	BB989241
E_ET_psgV_22A07_R_0	BB994050
E_ET_psgV_22A08_F_0	BB989242
E_ET_psgV_22A08_R_0	BB994051
E_ET_psgV_22A09_R_0	BB994052
E_ET_psgV_22A10_F_0	BB989243
E_ET_psgV_22A11_F_0	BB989244
E_ET_psgV_22A11_R_0	BB994053
E_ET_psgV_22B01_F_0	BB989245
E_ET_psgV_22B01_R_0	BB994054
E_ET_psgV_22B02_F_0	BB989246
E_ET_psgV_22B02_R_0	BB994055
E_ET_psgV_22B03_F_0	BB989247
E_ET_psgV_22B04_F_0	BB989248
E_ET_psgV_22B04_R_0	BB994056
E_ET_psgV_22B05_F_0	BB989249
E_ET_psgV_22B05_R_0	BB994057
E_ET_psgV_22B06_F_0	BB989250
E_ET_psgV_22B06_R_0	BB994058
E_ET_psgV_22B07_F_0	BB989251
E_ET_psgV_22B07_R_0	BB994059
E_ET_psgV_22B08_F_0	BB989252
E_ET_psgV_22B08_R_0	BB994060
E_ET_psgV_22B09_F_0	BB989253
E_ET_psgV_22B09_R_0	BB994061
E_ET_psgV_22B10_F_0	BB989254
E_ET_psgV_22B10_R_0	BB994062
E_ET_psgV_22B11_F_0	BB989255
E_ET_psgV_22B11_R_0	BB994063
E_ET_psgV_22B12_F_0	BB989256
E_ET_psgV_22B12_R_0	BB994064
E_ET_psgV_22C01_F_0	BB989257
E_ET_psgV_22C01_R_0	BB994065
E_ET_psgV_22C02_F_0	BB989258
E_ET_psgV_22C03_F_0	BB989259
E_ET_psgV_22C03_R_0	BB994066
E_ET_psgV_22C04_F_0	BB989260
E_ET_psgV_22C05_F_0	BB989261
E_ET_psgV_22C05_R_0	BB994067
E_ET_psgV_22C06_F_0	BB989262
E_ET_psgV_22C07_F_0	BB989263
E_ET_psgV_22C07_R_0	BB994068
E_ET_psgV_22C08_F_0	BB989264
E_ET_psgV_22C08_R_0	BB994069
E_ET_psgV_22C09_F_0	BB989265
E_ET_psgV_22C10_F_0	BB989266
E_ET_psgV_22C12_F_0	BB989267

E_ET_psgV_22C12_R_0	BB994070
E_ET_psgV_22D01_F_0	BB989268
E_ET_psgV_22D02_F_0	BB989269
E_ET_psgV_22D02_R_0	BB994071
E_ET_psgV_22D03_F_0	BB989270
E_ET_psgV_22D03_R_0	BB994072
E_ET_psgV_22D04_F_0	BB989271
E_ET_psgV_22D04_R_0	BB994073
E_ET_psgV_22D05_F_0	BB989272
E_ET_psgV_22D06_F_0	BB989273
E_ET_psgV_22D07_F_0	BB989274
E_ET_psgV_22D07_R_0	BB994074
E_ET_psgV_22D09_F_0	BB989275
E_ET_psgV_22D09_R_0	BB994075
E_ET_psgV_22D10_F_0	BB989276
E_ET_psgV_22D10_R_0	BB994076
E_ET_psgV_22E01_F_0	BB989277
E_ET_psgV_22E01_R_0	BB994077
E_ET_psgV_22E02_F_0	BB989278
E_ET_psgV_22E03_F_0	BB989279
E_ET_psgV_22E03_R_0	BB994078
E_ET_psgV_22E04_F_0	BB989280
E_ET_psgV_22E05_F_0	BB989281
E_ET_psgV_22E06_F_0	BB989282
E_ET_psgV_22E06_R_0	BB994079
E_ET_psgV_22E07_F_0	BB989283
E_ET_psgV_22E07_R_0	BB994080
E_ET_psgV_22E08_F_0	BB989284
E_ET_psgV_22E08_R_0	BB994081
E_ET_psgV_22E09_F_0	BB989285
E_ET_psgV_22E09_R_0	BB994082
E_ET_psgV_22E10_F_0	BB989286
E_ET_psgV_22E10_R_0	BB994083
E_ET_psgV_22E11_F_0	BB989287
E_ET_psgV_22E11_R_0	BB994084
E_ET_psgV_22E12_F_0	BB989288
E_ET_psgV_22E12_R_0	BB994085
E_ET_psgV_22F01_F_0	BB989289
E_ET_psgV_22F01_R_0	BB994086
E_ET_psgV_22F02_R_0	BB994087
E_ET_psgV_22F04_F_0	BB989290
E_ET_psgV_22F04_R_0	BB994088
E_ET_psgV_22F05_F_0	BB989291
E_ET_psgV_22F06_F_0	BB989292
E_ET_psgV_22F06_R_0	BB994089
E_ET_psgV_22F07_F_0	BB989293
E_ET_psgV_22F07_R_0	BB994090
E_ET_psgV_22F08_F_0	BB989294
E_ET_psgV_22F08_R_0	BB994091
E_ET_psgV_22F09_R_0	BB994092
E_ET_psgV_22F10_F_0	BB989295
E_ET_psgV_22F10_R_0	BB994093
E_ET_psgV_22F11_F_0	BB989296
E_ET_psgV_22F11_R_0	BB994094

E_ET_psgV_22F12_F_0	BB989297
E_ET_psgV_22F12_R_0	BB994095
E_ET_psgV_22G01_F_0	BB989298
E_ET_psgV_22G01_R_0	BB994096
E_ET_psgV_22G02_F_0	BB989299
E_ET_psgV_22G02_R_0	BB994097
E_ET_psgV_22G03_F_0	BB989300
E_ET_psgV_22G04_F_0	BB989301
E_ET_psgV_22G05_F_0	BB989302
E_ET_psgV_22G06_F_0	BB989303
E_ET_psgV_22G07_F_0	BB989304
E_ET_psgV_22G07_R_0	BB994098
E_ET_psgV_22G08_F_0	BB989305
E_ET_psgV_22G08_R_0	BB994099
E_ET_psgV_22G09_F_0	BB989306
E_ET_psgV_22G09_R_0	BB994100
E_ET_psgV_22G10_F_0	BB989307
E_ET_psgV_22G10_R_0	BB994101
E_ET_psgV_22G11_F_0	BB989308
E_ET_psgV_22G11_R_0	BB994102
E_ET_psgV_22G12_F_0	BB989309
E_ET_psgV_22G12_R_0	BB994103
E_ET_psgV_22H01_F_0	BB989310
E_ET_psgV_22H02_F_0	BB989311
E_ET_psgV_22H02_R_0	BB994104
E_ET_psgV_22H03_F_0	BB989312
E_ET_psgV_22H03_R_0	BB994105
E_ET_psgV_22H05_F_0	BB989313
E_ET_psgV_22H05_R_0	BB994106
E_ET_psgV_22H06_F_0	BB989314
E_ET_psgV_22H07_F_0	BB989315
E_ET_psgV_22H07_R_0	BB994107
E_ET_psgV_22H08_F_0	BB989316
E_ET_psgV_22H08_R_0	BB994108
E_ET_psgV_22H09_F_0	BB989317
E_ET_psgV_22H11_F_0	BB989318
E_ET_psgV_22H11_R_0	BB994109
E_ET_psgV_22H12_R_0	BB994110
E_ET_psgV_23A01_F_0	BB989319
E_ET_psgV_23A01_R_0	BB994111
E_ET_psgV_23A02_F_0	BB989320
E_ET_psgV_23A02_R_0	BB994112
E_ET_psgV_23A03_F_0	BB989321
E_ET_psgV_23A03_R_0	BB994113
E_ET_psgV_23A04_F_0	BB989322
E_ET_psgV_23A04_R_0	BB994114
E_ET_psgV_23A05_F_0	BB989323
E_ET_psgV_23A05_R_0	BB994115
E_ET_psgV_23A06_F_0	BB989324
E_ET_psgV_23A07_F_0	BB989325
E_ET_psgV_23A07_R_0	BB994116
E_ET_psgV_23A08_F_0	BB989326
E_ET_psgV_23A08_R_0	BB994117
E_ET_psgV_23A09_F_0	BB989327

E_ET_psgV_23A09_R_0	BB994118
E_ET_psgV_23A10_F_0	BB989328
E_ET_psgV_23A11_F_0	BB989329
E_ET_psgV_23B01_F_0	BB989330
E_ET_psgV_23B01_R_0	BB994119
E_ET_psgV_23B02_F_0	BB989331
E_ET_psgV_23B02_R_0	BB994120
E_ET_psgV_23B03_F_0	BB989332
E_ET_psgV_23B05_F_0	BB989333
E_ET_psgV_23B05_R_0	BB994121
E_ET_psgV_23B06_F_0	BB989334
E_ET_psgV_23B08_F_0	BB989335
E_ET_psgV_23B09_F_0	BB989336
E_ET_psgV_23B10_F_0	BB989337
E_ET_psgV_23B10_R_0	BB994122
E_ET_psgV_23B11_F_0	BB989338
E_ET_psgV_23B11_R_0	BB994123
E_ET_psgV_23B12_F_0	BB989339
E_ET_psgV_23B12_R_0	BB994124
E_ET_psgV_23C01_F_0	BB989340
E_ET_psgV_23C01_R_0	BB994125
E_ET_psgV_23C03_F_0	BB989341
E_ET_psgV_23C03_R_0	BB994126
E_ET_psgV_23C04_F_0	BB989342
E_ET_psgV_23C04_R_0	BB994127
E_ET_psgV_23C06_F_0	BB989343
E_ET_psgV_23C06_R_0	BB994128
E_ET_psgV_23C07_F_0	BB989344
E_ET_psgV_23C07_R_0	BB994129
E_ET_psgV_23C08_F_0	BB989345
E_ET_psgV_23C08_R_0	BB994130
E_ET_psgV_23C09_F_0	BB989346
E_ET_psgV_23C10_F_0	BB989347
E_ET_psgV_23C10_R_0	BB994131
E_ET_psgV_23C11_F_0	BB989348
E_ET_psgV_23C11_R_0	BB994132
E_ET_psgV_23C12_F_0	BB989349
E_ET_psgV_23C12_R_0	BB994133
E_ET_psgV_23D01_F_0	BB989350
E_ET_psgV_23D02_F_0	BB989351
E_ET_psgV_23D02_R_0	BB994134
E_ET_psgV_23D04_F_0	BB989352
E_ET_psgV_23D05_F_0	BB989353
E_ET_psgV_23D05_R_0	BB994135
E_ET_psgV_23D06_F_0	BB989354
E_ET_psgV_23D06_R_0	BB994136
E_ET_psgV_23D07_F_0	BB989355
E_ET_psgV_23D07_R_0	BB994137
E_ET_psgV_23D10_F_0	BB989356
E_ET_psgV_23D10_R_0	BB994138
E_ET_psgV_23D12_F_0	BB989357
E_ET_psgV_23D12_R_0	BB994139
E_ET_psgV_23E01_F_0	BB989358
E_ET_psgV_23E01_R_0	BB994140

E_ET_psgV_23E02_F_0	BB989359
E_ET_psgV_23E03_F_0	BB989360
E_ET_psgV_23E05_F_0	BB989361
E_ET_psgV_23E05_R_0	BB994141
E_ET_psgV_23E07_F_0	BB989362
E_ET_psgV_23E07_R_0	BB994142
E_ET_psgV_23E08_F_0	BB989363
E_ET_psgV_23E09_F_0	BB989364
E_ET_psgV_23E09_R_0	BB994143
E_ET_psgV_23E11_F_0	BB989365
E_ET_psgV_23E11_R_0	BB994144
E_ET_psgV_23E12_F_0	BB989366
E_ET_psgV_23E12_R_0	BB994145
E_ET_psgV_23F01_F_0	BB989367
E_ET_psgV_23F01_R_0	BB994146
E_ET_psgV_23F02_F_0	BB989368
E_ET_psgV_23F02_R_0	BB994147
E_ET_psgV_23F03_F_0	BB989369
E_ET_psgV_23F04_F_0	BB989370
E_ET_psgV_23F04_R_0	BB994148
E_ET_psgV_23F05_F_0	BB989371
E_ET_psgV_23F05_R_0	BB994149
E_ET_psgV_23F07_F_0	BB989372
E_ET_psgV_23F08_F_0	BB989373
E_ET_psgV_23F10_F_0	BB989374
E_ET_psgV_23F10_R_0	BB994150
E_ET_psgV_23F11_F_0	BB989375
E_ET_psgV_23F11_R_0	BB994151
E_ET_psgV_23G01_F_0	BB989376
E_ET_psgV_23G01_R_0	BB994152
E_ET_psgV_23G02_F_0	BB989377
E_ET_psgV_23G02_R_0	BB994153
E_ET_psgV_23G03_F_0	BB989378
E_ET_psgV_23G03_R_0	BB994154
E_ET_psgV_23G04_F_0	BB989379
E_ET_psgV_23G04_R_0	BB994155
E_ET_psgV_23G05_F_0	BB989380
E_ET_psgV_23G05_R_0	BB994156
E_ET_psgV_23G06_F_0	BB989381
E_ET_psgV_23G06_R_0	BB994157
E_ET_psgV_23G07_F_0	BB989382
E_ET_psgV_23G07_R_0	BB994158
E_ET_psgV_23G08_F_0	BB989383
E_ET_psgV_23G08_R_0	BB994159
E_ET_psgV_23G09_F_0	BB989384
E_ET_psgV_23G09_R_0	BB994160
E_ET_psgV_23G10_F_0	BB989385
E_ET_psgV_23G11_F_0	BB989386
E_ET_psgV_23G11_R_0	BB994161
E_ET_psgV_23G12_F_0	BB989387
E_ET_psgV_23H01_F_0	BB989388
E_ET_psgV_23H01_R_0	BB994162
E_ET_psgV_23H02_F_0	BB989389
E_ET_psgV_23H02_R_0	BB994163

E_ET_psgV_23H03_F_0	BB989390
E_ET_psgV_23H03_R_0	BB994164
E_ET_psgV_23H04_F_0	BB989391
E_ET_psgV_23H04_R_0	BB994165
E_ET_psgV_23H05_F_0	BB989392
E_ET_psgV_23H07_F_0	BB989393
E_ET_psgV_23H07_R_0	BB994166
E_ET_psgV_23H08_F_0	BB989394
E_ET_psgV_23H08_R_0	BB994167
E_ET_psgV_23H09_R_0	BB994168
E_ET_psgV_23H10_F_0	BB989395
E_ET_psgV_23H10_R_0	BB994169
E_ET_psgV_23H11_F_0	BB989396
E_ET_psgV_23H12_F_0	BB989397
E_ET_psgV_23H12_R_0	BB994170
E_ET_psgV_24A01_F_0	BB989398
E_ET_psgV_24A01_R_0	BB994171
E_ET_psgV_24A02_F_0	BB989399
E_ET_psgV_24A02_R_0	BB994172
E_ET_psgV_24A03_F_0	BB989400
E_ET_psgV_24A03_R_0	BB994173
E_ET_psgV_24A04_F_0	BB989401
E_ET_psgV_24A05_F_0	BB989402
E_ET_psgV_24A05_R_0	BB994174
E_ET_psgV_24A07_R_0	BB994175
E_ET_psgV_24A08_F_0	BB989403
E_ET_psgV_24A08_R_0	BB994176
E_ET_psgV_24A09_F_0	BB989404
E_ET_psgV_24A10_F_0	BB989405
E_ET_psgV_24A10_R_0	BB994177
E_ET_psgV_24A11_F_0	BB989406
E_ET_psgV_24A11_R_0	BB994178
E_ET_psgV_24A12_F_0	BB989407
E_ET_psgV_24A12_R_0	BB994179
E_ET_psgV_24B02_R_0	BB994180
E_ET_psgV_24B03_F_0	BB989408
E_ET_psgV_24B03_R_0	BB994181
E_ET_psgV_24B06_F_0	BB989409
E_ET_psgV_24B07_F_0	BB989410
E_ET_psgV_24B07_R_0	BB994182
E_ET_psgV_24B08_F_0	BB989411
E_ET_psgV_24B08_R_0	BB994183
E_ET_psgV_24B09_F_0	BB989412
E_ET_psgV_24B09_R_0	BB994184
E_ET_psgV_24B10_F_0	BB989413
E_ET_psgV_24B10_R_0	BB994185
E_ET_psgV_24B11_F_0	BB989414
E_ET_psgV_24B11_R_0	BB994186
E_ET_psgV_24B12_R_0	BB994187
E_ET_psgV_24C01_R_0	BB994188
E_ET_psgV_24C02_F_0	BB989415
E_ET_psgV_24C02_R_0	BB994189
E_ET_psgV_24C03_F_0	BB989416
E_ET_psgV_24C03_R_0	BB994190

E_ET_psgV_24C04_F_0	BB989417
E_ET_psgV_24C04_R_0	BB994191
E_ET_psgV_24C05_F_0	BB989418
E_ET_psgV_24C05_R_0	BB994192
E_ET_psgV_24C06_F_0	BB989419
E_ET_psgV_24C06_R_0	BB994193
E_ET_psgV_24C07_F_0	BB989420
E_ET_psgV_24C07_R_0	BB994194
E_ET_psgV_24C08_F_0	BB989421
E_ET_psgV_24C08_R_0	BB994195
E_ET_psgV_24C09_R_0	BB994196
E_ET_psgV_24C11_F_0	BB989422
E_ET_psgV_24C11_R_0	BB994197
E_ET_psgV_24C12_F_0	BB989423
E_ET_psgV_24C12_R_0	BB994198
E_ET_psgV_24D01_F_0	BB989424
E_ET_psgV_24D01_R_0	BB994199
E_ET_psgV_24D02_F_0	BB989425
E_ET_psgV_24D02_R_0	BB994200
E_ET_psgV_24D03_F_0	BB989426
E_ET_psgV_24D03_R_0	BB994201
E_ET_psgV_24D04_F_0	BB989427
E_ET_psgV_24D04_R_0	BB994202
E_ET_psgV_24D05_F_0	BB989428
E_ET_psgV_24D05_R_0	BB994203
E_ET_psgV_24D06_F_0	BB989429
E_ET_psgV_24D07_F_0	BB989430
E_ET_psgV_24D07_R_0	BB994204
E_ET_psgV_24D08_F_0	BB989431
E_ET_psgV_24D09_F_0	BB989432
E_ET_psgV_24D09_R_0	BB994205
E_ET_psgV_24D10_F_0	BB989433
E_ET_psgV_24D10_R_0	BB994206
E_ET_psgV_24D11_F_0	BB989434
E_ET_psgV_24D11_R_0	BB994207
E_ET_psgV_24D12_F_0	BB989435
E_ET_psgV_24D12_R_0	BB994208
E_ET_psgV_24E01_R_0	BB994209
E_ET_psgV_24E02_F_0	BB989436
E_ET_psgV_24E02_R_0	BB994210
E_ET_psgV_24E03_F_0	BB989437
E_ET_psgV_24E03_R_0	BB994211
E_ET_psgV_24E04_R_0	BB994212
E_ET_psgV_24E05_R_0	BB994213
E_ET_psgV_24E06_R_0	BB994214
E_ET_psgV_24E07_F_0	BB989438
E_ET_psgV_24E11_F_0	BB989439
E_ET_psgV_24E11_R_0	BB994215
E_ET_psgV_24E12_F_0	BB989440
E_ET_psgV_24E12_R_0	BB994216
E_ET_psgV_24F01_F_0	BB989441
E_ET_psgV_24F01_R_0	BB994217
E_ET_psgV_24F02_F_0	BB989442
E_ET_psgV_24F02_R_0	BB994218

E_ET_psgV_24F03_F_0	BB989443
E_ET_psgV_24F03_R_0	BB994219
E_ET_psgV_24F04_F_0	BB989444
E_ET_psgV_24F04_R_0	BB994220
E_ET_psgV_24F05_F_0	BB989445
E_ET_psgV_24F06_R_0	BB994221
E_ET_psgV_24F07_F_0	BB989446
E_ET_psgV_24F07_R_0	BB994222
E_ET_psgV_24F08_F_0	BB989447
E_ET_psgV_24F08_R_0	BB994223
E_ET_psgV_24F09_F_0	BB989448
E_ET_psgV_24F11_F_0	BB989449
E_ET_psgV_24F11_R_0	BB994224
E_ET_psgV_24F12_R_0	BB994225
E_ET_psgV_24G01_R_0	BB994226
E_ET_psgV_24G02_F_0	BB989450
E_ET_psgV_24G02_R_0	BB994227
E_ET_psgV_24G03_F_0	BB989451
E_ET_psgV_24G03_R_0	BB994228
E_ET_psgV_24G04_F_0	BB989452
E_ET_psgV_24G04_R_0	BB994229
E_ET_psgV_24G05_F_0	BB989453
E_ET_psgV_24G05_R_0	BB994230
E_ET_psgV_24G06_F_0	BB989454
E_ET_psgV_24G06_R_0	BB994231
E_ET_psgV_24G07_F_0	BB989455
E_ET_psgV_24G08_F_0	BB989456
E_ET_psgV_24G08_R_0	BB994232
E_ET_psgV_24G09_F_0	BB989457
E_ET_psgV_24G09_R_0	BB994233
E_ET_psgV_24G10_F_0	BB989458
E_ET_psgV_24G10_R_0	BB994234
E_ET_psgV_24G11_F_0	BB989459
E_ET_psgV_24G11_R_0	BB994235
E_ET_psgV_24G12_F_0	BB989460
E_ET_psgV_24G12_R_0	BB994236
E_ET_psgV_24H01_F_0	BB989461
E_ET_psgV_24H01_R_0	BB994237
E_ET_psgV_24H02_F_0	BB989462
E_ET_psgV_24H03_F_0	BB989463
E_ET_psgV_24H03_R_0	BB994238
E_ET_psgV_24H05_F_0	BB989464
E_ET_psgV_24H05_R_0	BB994239
E_ET_psgV_24H06_F_0	BB989465
E_ET_psgV_24H06_R_0	BB994240
E_ET_psgV_24H07_F_0	BB989466
E_ET_psgV_24H07_R_0	BB994241
E_ET_psgV_24H08_F_0	BB989467
E_ET_psgV_24H09_R_0	BB994242
E_ET_psgV_24H10_F_0	BB989468
E_ET_psgV_24H10_R_0	BB994243
E_ET_psgV_24H11_F_0	BB989469
E_ET_psgV_24H11_R_0	BB994244
E_ET_psgV_24H12_F_0	BB989470

E_ET_psgV_24H12_R_0	BB994245
E_ET_psgV_25A01_F_0	BB989471
E_ET_psgV_25A01_R_0	BB994246
E_ET_psgV_25A02_F_0	BB989472
E_ET_psgV_25A03_F_0	BB989473
E_ET_psgV_25A03_R_0	BB994247
E_ET_psgV_25A04_F_0	BB989474
E_ET_psgV_25A04_R_0	BB994248
E_ET_psgV_25A05_F_0	BB989475
E_ET_psgV_25A06_F_0	BB989476
E_ET_psgV_25A06_R_0	BB994249
E_ET_psgV_25A07_F_0	BB989477
E_ET_psgV_25A07_R_0	BB994250
E_ET_psgV_25A09_F_0	BB989478
E_ET_psgV_25A09_R_0	BB994251
E_ET_psgV_25A10_F_0	BB989479
E_ET_psgV_25A10_R_0	BB994252
E_ET_psgV_25A11_F_0	BB989480
E_ET_psgV_25A11_R_0	BB994253
E_ET_psgV_25A12_F_0	BB989481
E_ET_psgV_25B01_F_0	BB989482
E_ET_psgV_25B01_R_0	BB994254
E_ET_psgV_25B02_F_0	BB989483
E_ET_psgV_25B03_F_0	BB989484
E_ET_psgV_25B03_R_0	BB994255
E_ET_psgV_25B04_F_0	BB989485
E_ET_psgV_25B04_R_0	BB994256
E_ET_psgV_25B05_F_0	BB989486
E_ET_psgV_25B05_R_0	BB994257
E_ET_psgV_25B06_F_0	BB989487
E_ET_psgV_25B06_R_0	BB994258
E_ET_psgV_25B07_F_0	BB989488
E_ET_psgV_25B08_F_0	BB989489
E_ET_psgV_25B09_F_0	BB989490
E_ET_psgV_25B09_R_0	BB994259
E_ET_psgV_25B10_F_0	BB989491
E_ET_psgV_25B10_R_0	BB994260
E_ET_psgV_25B11_F_0	BB989492
E_ET_psgV_25B12_F_0	BB989493
E_ET_psgV_25B12_R_0	BB994261
E_ET_psgV_25C01_F_0	BB989494
E_ET_psgV_25C01_R_0	BB994262
E_ET_psgV_25C02_F_0	BB989495
E_ET_psgV_25C02_R_0	BB994263
E_ET_psgV_25C03_F_0	BB989496
E_ET_psgV_25C03_R_0	BB994264
E_ET_psgV_25C04_F_0	BB989497
E_ET_psgV_25C04_R_0	BB994265
E_ET_psgV_25C05_F_0	BB989498
E_ET_psgV_25C05_R_0	BB994266
E_ET_psgV_25C06_F_0	BB989499
E_ET_psgV_25C06_R_0	BB994267
E_ET_psgV_25C07_F_0	BB989500
E_ET_psgV_25C07_R_0	BB994268

E_ET_psgV_25C08_F_0	BB989501
E_ET_psgV_25C08_R_0	BB994269
E_ET_psgV_25C09_F_0	BB989502
E_ET_psgV_25C09_R_0	BB994270
E_ET_psgV_25C10_F_0	BB989503
E_ET_psgV_25C11_F_0	BB989504
E_ET_psgV_25C12_F_0	BB989505
E_ET_psgV_25C12_R_0	BB994271
E_ET_psgV_25D01_F_0	BB989506
E_ET_psgV_25D02_F_0	BB989507
E_ET_psgV_25D02_R_0	BB994272
E_ET_psgV_25D03_F_0	BB989508
E_ET_psgV_25D03_R_0	BB994273
E_ET_psgV_25D04_F_0	BB989509
E_ET_psgV_25D04_R_0	BB994274
E_ET_psgV_25D05_F_0	BB989510
E_ET_psgV_25D05_R_0	BB994275
E_ET_psgV_25D06_F_0	BB989511
E_ET_psgV_25D06_R_0	BB994276
E_ET_psgV_25D07_F_0	BB989512
E_ET_psgV_25D09_F_0	BB989513
E_ET_psgV_25D09_R_0	BB994277
E_ET_psgV_25D10_F_0	BB989514
E_ET_psgV_25D10_R_0	BB994278
E_ET_psgV_25D11_F_0	BB989515
E_ET_psgV_25E01_F_0	BB989516
E_ET_psgV_25E01_R_0	BB994279
E_ET_psgV_25E02_F_0	BB989517
E_ET_psgV_25E02_R_0	BB994280
E_ET_psgV_25E03_F_0	BB989518
E_ET_psgV_25E03_R_0	BB994281
E_ET_psgV_25E04_F_0	BB989519
E_ET_psgV_25E04_R_0	BB994282
E_ET_psgV_25E06_F_0	BB989520
E_ET_psgV_25E06_R_0	BB994283
E_ET_psgV_25E07_F_0	BB989521
E_ET_psgV_25E07_R_0	BB994284
E_ET_psgV_25E08_F_0	BB989522
E_ET_psgV_25E08_R_0	BB994285
E_ET_psgV_25E09_F_0	BB989523
E_ET_psgV_25E09_R_0	BB994286
E_ET_psgV_25E10_F_0	BB989524
E_ET_psgV_25E10_R_0	BB994287
E_ET_psgV_25E11_R_0	BB994288
E_ET_psgV_25E12_F_0	BB989525
E_ET_psgV_25E12_R_0	BB994289
E_ET_psgV_25F01_F_0	BB989526
E_ET_psgV_25F01_R_0	BB994290
E_ET_psgV_25F04_F_0	BB989527
E_ET_psgV_25F04_R_0	BB994291
E_ET_psgV_25F05_R_0	BB994292
E_ET_psgV_25F06_F_0	BB989528
E_ET_psgV_25F06_R_0	BB994293
E_ET_psgV_25F07_F_0	BB989529

E_ET_psgV_25F07_R_0	BB994294
E_ET_psgV_25F08_F_0	BB989530
E_ET_psgV_25F08_R_0	BB994295
E_ET_psgV_25F09_F_0	BB989531
E_ET_psgV_25F09_R_0	BB994296
E_ET_psgV_25F10_R_0	BB994297
E_ET_psgV_25F11_F_0	BB989532
E_ET_psgV_25F11_R_0	BB994298
E_ET_psgV_25F12_F_0	BB989533
E_ET_psgV_25F12_R_0	BB994299
E_ET_psgV_25G01_F_0	BB989534
E_ET_psgV_25G01_R_0	BB994300
E_ET_psgV_25G02_F_0	BB989535
E_ET_psgV_25G02_R_0	BB994301
E_ET_psgV_25G03_F_0	BB989536
E_ET_psgV_25G03_R_0	BB994302
E_ET_psgV_25G04_F_0	BB989537
E_ET_psgV_25G04_R_0	BB994303
E_ET_psgV_25G05_F_0	BB989538
E_ET_psgV_25G05_R_0	BB994304
E_ET_psgV_25G06_F_0	BB989539
E_ET_psgV_25G06_R_0	BB994305
E_ET_psgV_25G07_F_0	BB989540
E_ET_psgV_25G07_R_0	BB994306
E_ET_psgV_25G08_F_0	BB989541
E_ET_psgV_25G09_F_0	BB989542
E_ET_psgV_25G10_F_0	BB989543
E_ET_psgV_25G10_R_0	BB994307
E_ET_psgV_25G11_F_0	BB989544
E_ET_psgV_25G11_R_0	BB994308
E_ET_psgV_25G12_F_0	BB989545
E_ET_psgV_25G12_R_0	BB994309
E_ET_psgV_25H01_F_0	BB989546
E_ET_psgV_25H01_R_0	BB994310
E_ET_psgV_25H02_F_0	BB989547
E_ET_psgV_25H02_R_0	BB994311
E_ET_psgV_25H04_F_0	BB989548
E_ET_psgV_25H04_R_0	BB994312
E_ET_psgV_25H05_F_0	BB989549
E_ET_psgV_25H06_F_0	BB989550
E_ET_psgV_25H06_R_0	BB994313
E_ET_psgV_25H07_F_0	BB989551
E_ET_psgV_25H07_R_0	BB994314
E_ET_psgV_25H08_F_0	BB989552
E_ET_psgV_25H08_R_0	BB994315
E_ET_psgV_25H09_F_0	BB989553
E_ET_psgV_25H09_R_0	BB994316
E_ET_psgV_25H10_F_0	BB989554
E_ET_psgV_25H10_R_0	BB994317
E_ET_psgV_25H11_F_0	BB989555
E_ET_psgV_25H11_R_0	BB994318
E_ET_psgV_25H12_F_0	BB989556
E_ET_psgV_26A01_F_0	BB989557
E_ET_psgV_26A01_R_0	BB994319

E_ET_psgV_26A02_F_0	BB989558
E_ET_psgV_26A03_F_0	BB989559
E_ET_psgV_26A04_F_0	BB989560
E_ET_psgV_26A04_R_0	BB994320
E_ET_psgV_26A05_F_0	BB989561
E_ET_psgV_26A05_R_0	BB994321
E_ET_psgV_26A06_F_0	BB989562
E_ET_psgV_26A06_R_0	BB994322
E_ET_psgV_26A07_F_0	BB989563
E_ET_psgV_26A08_F_0	BB989564
E_ET_psgV_26A08_R_0	BB994323
E_ET_psgV_26A09_F_0	BB989565
E_ET_psgV_26A10_F_0	BB989566
E_ET_psgV_26A11_F_0	BB989567
E_ET_psgV_26A11_R_0	BB994324
E_ET_psgV_26A12_F_0	BB989568
E_ET_psgV_26A12_R_0	BB994325
E_ET_psgV_26B01_F_0	BB989569
E_ET_psgV_26B03_F_0	BB989570
E_ET_psgV_26B03_R_0	BB994326
E_ET_psgV_26B04_F_0	BB989571
E_ET_psgV_26B04_R_0	BB994327
E_ET_psgV_26B06_F_0	BB989572
E_ET_psgV_26B06_R_0	BB994328
E_ET_psgV_26B07_F_0	BB989573
E_ET_psgV_26B07_R_0	BB994329
E_ET_psgV_26B08_F_0	BB989574
E_ET_psgV_26B08_R_0	BB994330
E_ET_psgV_26B09_F_0	BB989575
E_ET_psgV_26B10_F_0	BB989576
E_ET_psgV_26B10_R_0	BB994331
E_ET_psgV_26B11_F_0	BB989577
E_ET_psgV_26B11_R_0	BB994332
E_ET_psgV_26B12_F_0	BB989578
E_ET_psgV_26B12_R_0	BB994333
E_ET_psgV_26C01_F_0	BB989579
E_ET_psgV_26C01_R_0	BB994334
E_ET_psgV_26C02_F_0	BB989580
E_ET_psgV_26C02_R_0	BB994335
E_ET_psgV_26C03_F_0	BB989581
E_ET_psgV_26C03_R_0	BB994336
E_ET_psgV_26C04_R_0	BB994337
E_ET_psgV_26C05_F_0	BB989582
E_ET_psgV_26C05_R_0	BB994338
E_ET_psgV_26C06_F_0	BB989583
E_ET_psgV_26C06_R_0	BB994339
E_ET_psgV_26C07_R_0	BB994340
E_ET_psgV_26C08_F_0	BB989584
E_ET_psgV_26C09_F_0	BB989585
E_ET_psgV_26C09_R_0	BB994341
E_ET_psgV_26C10_F_0	BB989586
E_ET_psgV_26C11_F_0	BB989587
E_ET_psgV_26C11_R_0	BB994342
E_ET_psgV_26C12_F_0	BB989588

E_ET_psgV_26C12_R_0	BB994343
E_ET_psgV_26D01_R_0	BB994344
E_ET_psgV_26D03_F_0	BB989589
E_ET_psgV_26D03_R_0	BB994345
E_ET_psgV_26D04_F_0	BB989590
E_ET_psgV_26D04_R_0	BB994346
E_ET_psgV_26D05_F_0	BB989591
E_ET_psgV_26D05_R_0	BB994347
E_ET_psgV_26D06_F_0	BB989592
E_ET_psgV_26D06_R_0	BB994348
E_ET_psgV_26D07_R_0	BB994349
E_ET_psgV_26D08_F_0	BB989593
E_ET_psgV_26D08_R_0	BB994350
E_ET_psgV_26D09_F_0	BB989594
E_ET_psgV_26D10_F_0	BB989595
E_ET_psgV_26D11_F_0	BB989596
E_ET_psgV_26D11_R_0	BB994351
E_ET_psgV_26D12_F_0	BB989597
E_ET_psgV_26D12_R_0	BB994352
E_ET_psgV_26E01_F_0	BB989598
E_ET_psgV_26E02_F_0	BB989599
E_ET_psgV_26E02_R_0	BB994353
E_ET_psgV_26E03_F_0	BB989600
E_ET_psgV_26E04_F_0	BB989601
E_ET_psgV_26E04_R_0	BB994354
E_ET_psgV_26E05_F_0	BB989602
E_ET_psgV_26E05_R_0	BB994355
E_ET_psgV_26E06_F_0	BB989603
E_ET_psgV_26E06_R_0	BB994356
E_ET_psgV_26E08_F_0	BB989604
E_ET_psgV_26E09_F_0	BB989605
E_ET_psgV_26E10_F_0	BB989606
E_ET_psgV_26E12_F_0	BB989607
E_ET_psgV_26E12_R_0	BB994357
E_ET_psgV_26F01_F_0	BB989608
E_ET_psgV_26F01_R_0	BB994358
E_ET_psgV_26F02_F_0	BB989609
E_ET_psgV_26F03_F_0	BB989610
E_ET_psgV_26F03_R_0	BB994359
E_ET_psgV_26F04_F_0	BB989611
E_ET_psgV_26F04_R_0	BB994360
E_ET_psgV_26F05_F_0	BB989612
E_ET_psgV_26F05_R_0	BB994361
E_ET_psgV_26F06_F_0	BB989613
E_ET_psgV_26F06_R_0	BB994362
E_ET_psgV_26F07_F_0	BB989614
E_ET_psgV_26F07_R_0	BB994363
E_ET_psgV_26F08_F_0	BB989615
E_ET_psgV_26F08_R_0	BB994364
E_ET_psgV_26F09_F_0	BB989616
E_ET_psgV_26F10_F_0	BB989617
E_ET_psgV_26F10_R_0	BB994365
E_ET_psgV_26F11_F_0	BB989618
E_ET_psgV_26F11_R_0	BB994366

E_ET_psgV_26F12_F_0	BB989619
E_ET_psgV_26G01_F_0	BB989620
E_ET_psgV_26G03_F_0	BB989621
E_ET_psgV_26G03_R_0	BB994367
E_ET_psgV_26G04_F_0	BB989622
E_ET_psgV_26G04_R_0	BB994368
E_ET_psgV_26G05_R_0	BB994369
E_ET_psgV_26G06_F_0	BB989623
E_ET_psgV_26G06_R_0	BB994370
E_ET_psgV_26G07_F_0	BB989624
E_ET_psgV_26G07_R_0	BB994371
E_ET_psgV_26G08_F_0	BB989625
E_ET_psgV_26G08_R_0	BB994372
E_ET_psgV_26G09_R_0	BB994373
E_ET_psgV_26G10_F_0	BB989626
E_ET_psgV_26G10_R_0	BB994374
E_ET_psgV_26G11_F_0	BB989627
E_ET_psgV_26G11_R_0	BB994375
E_ET_psgV_26H01_F_0	BB989628
E_ET_psgV_26H01_R_0	BB994376
E_ET_psgV_26H02_F_0	BB989629
E_ET_psgV_26H03_F_0	BB989630
E_ET_psgV_26H03_R_0	BB994377
E_ET_psgV_26H04_F_0	BB989631
E_ET_psgV_26H04_R_0	BB994378
E_ET_psgV_26H05_F_0	BB989632
E_ET_psgV_26H05_R_0	BB994379
E_ET_psgV_26H06_F_0	BB989633
E_ET_psgV_26H06_R_0	BB994380
E_ET_psgV_26H07_F_0	BB989634
E_ET_psgV_26H07_R_0	BB994381
E_ET_psgV_26H08_F_0	BB989635
E_ET_psgV_26H08_R_0	BB994382
E_ET_psgV_26H09_F_0	BB989636
E_ET_psgV_26H10_F_0	BB989637
E_ET_psgV_26H11_F_0	BB989638
E_ET_psgV_26H11_R_0	BB994383
E_ET_psgV_26H12_F_0	BB989639
E_ET_psgV_27A02_F_0	BB989640
E_ET_psgV_27A03_F_0	BB989641
E_ET_psgV_27A03_R_0	BB994384
E_ET_psgV_27A04_F_0	BB989642
E_ET_psgV_27A04_R_0	BB994385
E_ET_psgV_27A05_F_0	BB989643
E_ET_psgV_27A05_R_0	BB994386
E_ET_psgV_27A06_F_0	BB989644
E_ET_psgV_27A06_R_0	BB994387
E_ET_psgV_27A07_F_0	BB989645
E_ET_psgV_27A07_R_0	BB994388
E_ET_psgV_27A08_F_0	BB989646
E_ET_psgV_27A08_R_0	BB994389
E_ET_psgV_27A09_F_0	BB989647
E_ET_psgV_27A09_R_0	BB994390
E_ET_psgV_27A10_F_0	BB989648

E_ET_psgV_27A10_R_0	BB994391
E_ET_psgV_27A12_F_0	BB989649
E_ET_psgV_27A12_R_0	BB994392
E_ET_psgV_27B01_F_0	BB989650
E_ET_psgV_27B01_R_0	BB994393
E_ET_psgV_27B02_F_0	BB989651
E_ET_psgV_27B02_R_0	BB994394
E_ET_psgV_27B03_F_0	BB989652
E_ET_psgV_27B03_R_0	BB994395
E_ET_psgV_27B04_F_0	BB989653
E_ET_psgV_27B04_R_0	BB994396
E_ET_psgV_27B05_F_0	BB989654
E_ET_psgV_27B05_R_0	BB994397
E_ET_psgV_27B06_F_0	BB989655
E_ET_psgV_27B06_R_0	BB994398
E_ET_psgV_27B07_F_0	BB989656
E_ET_psgV_27B08_F_0	BB989657
E_ET_psgV_27B08_R_0	BB994399
E_ET_psgV_27B09_F_0	BB989658
E_ET_psgV_27B10_F_0	BB989659
E_ET_psgV_27B10_R_0	BB994400
E_ET_psgV_27B12_F_0	BB989660
E_ET_psgV_27B12_R_0	BB994401
E_ET_psgV_27C01_F_0	BB989661
E_ET_psgV_27C01_R_0	BB994402
E_ET_psgV_27C02_F_0	BB989662
E_ET_psgV_27C03_F_0	BB989663
E_ET_psgV_27C03_R_0	BB994403
E_ET_psgV_27C04_R_0	BB994404
E_ET_psgV_27C05_F_0	BB989664
E_ET_psgV_27C05_R_0	BB994405
E_ET_psgV_27C06_F_0	BB989665
E_ET_psgV_27C06_R_0	BB994406
E_ET_psgV_27C07_F_0	BB989666
E_ET_psgV_27C07_R_0	BB994407
E_ET_psgV_27C08_F_0	BB989667
E_ET_psgV_27C08_R_0	BB994408
E_ET_psgV_27C09_F_0	BB989668
E_ET_psgV_27C10_F_0	BB989669
E_ET_psgV_27C10_R_0	BB994409
E_ET_psgV_27C11_F_0	BB989670
E_ET_psgV_27C11_R_0	BB994410
E_ET_psgV_27C12_F_0	BB989671
E_ET_psgV_27C12_R_0	BB994411
E_ET_psgV_27D01_F_0	BB989672
E_ET_psgV_27D02_F_0	BB989673
E_ET_psgV_27D03_F_0	BB989674
E_ET_psgV_27D04_F_0	BB989675
E_ET_psgV_27D04_R_0	BB994412
E_ET_psgV_27D05_R_0	BB994413
E_ET_psgV_27D06_F_0	BB989676
E_ET_psgV_27D06_R_0	BB994414
E_ET_psgV_27D07_F_0	BB989677
E_ET_psgV_27D07_R_0	BB994415

E_ET_psgV_27D08_F_0	BB989678
E_ET_psgV_27D08_R_0	BB994416
E_ET_psgV_27D09_F_0	BB989679
E_ET_psgV_27D09_R_0	BB994417
E_ET_psgV_27D10_F_0	BB989680
E_ET_psgV_27D10_R_0	BB994418
E_ET_psgV_27D11_F_0	BB989681
E_ET_psgV_27D11_R_0	BB994419
E_ET_psgV_27D12_F_0	BB989682
E_ET_psgV_27D12_R_0	BB994420
E_ET_psgV_27E01_F_0	BB989683
E_ET_psgV_27E02_F_0	BB989684
E_ET_psgV_27E02_R_0	BB994421
E_ET_psgV_27E03_F_0	BB989685
E_ET_psgV_27E03_R_0	BB994422
E_ET_psgV_27E04_F_0	BB989686
E_ET_psgV_27E06_F_0	BB989687
E_ET_psgV_27E06_R_0	BB994423
E_ET_psgV_27E07_F_0	BB989688
E_ET_psgV_27E07_R_0	BB994424
E_ET_psgV_27E08_F_0	BB989689
E_ET_psgV_27E08_R_0	BB994425
E_ET_psgV_27E09_F_0	BB989690
E_ET_psgV_27E09_R_0	BB994426
E_ET_psgV_27E10_R_0	BB994427
E_ET_psgV_27E12_F_0	BB989691
E_ET_psgV_27E12_R_0	BB994428
E_ET_psgV_27F01_F_0	BB989692
E_ET_psgV_27F01_R_0	BB994429
E_ET_psgV_27F02_F_0	BB989693
E_ET_psgV_27F02_R_0	BB994430
E_ET_psgV_27F03_F_0	BB989694
E_ET_psgV_27F03_R_0	BB994431
E_ET_psgV_27F04_F_0	BB989695
E_ET_psgV_27F05_F_0	BB989696
E_ET_psgV_27F05_R_0	BB994432
E_ET_psgV_27F06_F_0	BB989697
E_ET_psgV_27F06_R_0	BB994433
E_ET_psgV_27F07_F_0	BB989698
E_ET_psgV_27F08_F_0	BB989699
E_ET_psgV_27F08_R_0	BB994434
E_ET_psgV_27F09_F_0	BB989700
E_ET_psgV_27F09_R_0	BB994435
E_ET_psgV_27F10_F_0	BB989701
E_ET_psgV_27F11_F_0	BB989702
E_ET_psgV_27F11_R_0	BB994436
E_ET_psgV_27F12_F_0	BB989703
E_ET_psgV_27F12_R_0	BB994437
E_ET_psgV_27G01_F_0	BB989704
E_ET_psgV_27G01_R_0	BB994438
E_ET_psgV_27G02_F_0	BB989705
E_ET_psgV_27G02_R_0	BB994439
E_ET_psgV_27G03_F_0	BB989706
E_ET_psgV_27G03_R_0	BB994440

E_ET_psgV_27G04_F_0	BB989707
E_ET_psgV_27G04_R_0	BB994441
E_ET_psgV_27G05_F_0	BB989708
E_ET_psgV_27G05_R_0	BB994442
E_ET_psgV_27G06_F_0	BB989709
E_ET_psgV_27G06_R_0	BB994443
E_ET_psgV_27G07_F_0	BB989710
E_ET_psgV_27G07_R_0	BB994444
E_ET_psgV_27G08_F_0	BB989711
E_ET_psgV_27G08_R_0	BB994445
E_ET_psgV_27G09_F_0	BB989712
E_ET_psgV_27G09_R_0	BB994446
E_ET_psgV_27G10_F_0	BB989713
E_ET_psgV_27G10_R_0	BB994447
E_ET_psgV_27G12_F_0	BB989714
E_ET_psgV_27G12_R_0	BB994448
E_ET_psgV_27H01_F_0	BB989715
E_ET_psgV_27H01_R_0	BB994449
E_ET_psgV_27H02_F_0	BB989716
E_ET_psgV_27H03_F_0	BB989717
E_ET_psgV_27H03_R_0	BB994450
E_ET_psgV_27H04_F_0	BB989718
E_ET_psgV_27H05_F_0	BB989719
E_ET_psgV_27H05_R_0	BB994451
E_ET_psgV_27H06_F_0	BB989720
E_ET_psgV_27H06_R_0	BB994452
E_ET_psgV_27H07_F_0	BB989721
E_ET_psgV_27H07_R_0	BB994453
E_ET_psgV_27H08_F_0	BB989722
E_ET_psgV_27H08_R_0	BB994454
E_ET_psgV_27H09_F_0	BB989723
E_ET_psgV_27H10_F_0	BB989724
E_ET_psgV_27H10_R_0	BB994455
E_ET_psgV_27H11_F_0	BB989725
E_ET_psgV_27H11_R_0	BB994456
E_ET_psgV_27H12_F_0	BB989726
E_ET_psgV_28A02_F_0	BB989727
E_ET_psgV_28A03_F_0	BB989728
E_ET_psgV_28A04_F_0	BB989729
E_ET_psgV_28A06_F_0	BB989730
E_ET_psgV_28A09_F_0	BB989731
E_ET_psgV_28A10_F_0	BB989732
E_ET_psgV_28A11_R_0	BB994457
E_ET_psgV_28A12_F_0	BB989733
E_ET_psgV_28A12_R_0	BB994458
E_ET_psgV_28B01_F_0	BB989734
E_ET_psgV_28B02_F_0	BB989735
E_ET_psgV_28B03_F_0	BB989736
E_ET_psgV_28B03_R_0	BB994459
E_ET_psgV_28B04_F_0	BB989737
E_ET_psgV_28B04_R_0	BB994460
E_ET_psgV_28B05_F_0	BB989738
E_ET_psgV_28B06_F_0	BB989739
E_ET_psgV_28B06_R_0	BB994461

E_ET_psgV_28B07_F_0	BB989740
E_ET_psgV_28B08_F_0	BB989741
E_ET_psgV_28B09_F_0	BB989742
E_ET_psgV_28B10_F_0	BB989743
E_ET_psgV_28B12_F_0	BB989744
E_ET_psgV_28B12_R_0	BB994462
E_ET_psgV_28C01_R_0	BB994463
E_ET_psgV_28C02_F_0	BB989745
E_ET_psgV_28C03_F_0	BB989746
E_ET_psgV_28C03_R_0	BB994464
E_ET_psgV_28C06_F_0	BB989747
E_ET_psgV_28C06_R_0	BB994465
E_ET_psgV_28C07_F_0	BB989748
E_ET_psgV_28C07_R_0	BB994466
E_ET_psgV_28C10_F_0	BB989749
E_ET_psgV_28C11_F_0	BB989750
E_ET_psgV_28C11_R_0	BB994467
E_ET_psgV_28C12_F_0	BB989751
E_ET_psgV_28C12_R_0	BB994468
E_ET_psgV_28D01_F_0	BB989752
E_ET_psgV_28D01_R_0	BB994469
E_ET_psgV_28D02_F_0	BB989753
E_ET_psgV_28D02_R_0	BB994470
E_ET_psgV_28D04_F_0	BB989754
E_ET_psgV_28D04_R_0	BB994471
E_ET_psgV_28D05_F_0	BB989755
E_ET_psgV_28D05_R_0	BB994472
E_ET_psgV_28D06_F_0	BB989756
E_ET_psgV_28D06_R_0	BB994473
E_ET_psgV_28D08_F_0	BB989757
E_ET_psgV_28D08_R_0	BB994474
E_ET_psgV_28D09_F_0	BB989758
E_ET_psgV_28D09_R_0	BB994475
E_ET_psgV_28D10_F_0	BB989759
E_ET_psgV_28D10_R_0	BB994476
E_ET_psgV_28D11_F_0	BB989760
E_ET_psgV_28D12_F_0	BB989761
E_ET_psgV_28D12_R_0	BB994477
E_ET_psgV_28E01_F_0	BB989762
E_ET_psgV_28E01_R_0	BB994478
E_ET_psgV_28E02_F_0	BB989763
E_ET_psgV_28E02_R_0	BB994479
E_ET_psgV_28E03_F_0	BB989764
E_ET_psgV_28E03_R_0	BB994480
E_ET_psgV_28E04_F_0	BB989765
E_ET_psgV_28E04_R_0	BB994481
E_ET_psgV_28E05_R_0	BB994482
E_ET_psgV_28E06_F_0	BB989766
E_ET_psgV_28E06_R_0	BB994483
E_ET_psgV_28E07_F_0	BB989767
E_ET_psgV_28E09_F_0	BB989768
E_ET_psgV_28E09_R_0	BB994484
E_ET_psgV_28E10_F_0	BB989769
E_ET_psgV_28E10_R_0	BB994485

E_ET_psgV_28E11_F_0	BB989770
E_ET_psgV_28E11_R_0	BB994486
E_ET_psgV_28E12_F_0	BB989771
E_ET_psgV_28E12_R_0	BB994487
E_ET_psgV_28F01_F_0	BB989772
E_ET_psgV_28F01_R_0	BB994488
E_ET_psgV_28F02_F_0	BB989773
E_ET_psgV_28F03_F_0	BB989774
E_ET_psgV_28F03_R_0	BB994489
E_ET_psgV_28F04_F_0	BB989775
E_ET_psgV_28F05_F_0	BB989776
E_ET_psgV_28F05_R_0	BB994490
E_ET_psgV_28F06_F_0	BB989777
E_ET_psgV_28F07_F_0	BB989778
E_ET_psgV_28F07_R_0	BB994491
E_ET_psgV_28F08_F_0	BB989779
E_ET_psgV_28F09_F_0	BB989780
E_ET_psgV_28F09_R_0	BB994492
E_ET_psgV_28F10_F_0	BB989781
E_ET_psgV_28F10_R_0	BB994493
E_ET_psgV_28F11_F_0	BB989782
E_ET_psgV_28F11_R_0	BB994494
E_ET_psgV_28F12_F_0	BB989783
E_ET_psgV_28G02_F_0	BB989784
E_ET_psgV_28G02_R_0	BB994495
E_ET_psgV_28G03_F_0	BB989785
E_ET_psgV_28G03_R_0	BB994496
E_ET_psgV_28G04_F_0	BB989786
E_ET_psgV_28G04_R_0	BB994497
E_ET_psgV_28G05_F_0	BB989787
E_ET_psgV_28G05_R_0	BB994498
E_ET_psgV_28G06_F_0	BB989788
E_ET_psgV_28G07_F_0	BB989789
E_ET_psgV_28G07_R_0	BB994499
E_ET_psgV_28G08_F_0	BB989790
E_ET_psgV_28G08_R_0	BB994500
E_ET_psgV_28G09_F_0	BB989791
E_ET_psgV_28G09_R_0	BB994501
E_ET_psgV_28G10_F_0	BB989792
E_ET_psgV_28G10_R_0	BB994502
E_ET_psgV_28G11_F_0	BB989793
E_ET_psgV_28G11_R_0	BB994503
E_ET_psgV_28G12_F_0	BB989794
E_ET_psgV_28G12_R_0	BB994504
E_ET_psgV_28H01_F_0	BB989795
E_ET_psgV_28H02_F_0	BB989796
E_ET_psgV_28H02_R_0	BB994505
E_ET_psgV_28H03_F_0	BB989797
E_ET_psgV_28H03_R_0	BB994506
E_ET_psgV_28H04_F_0	BB989798
E_ET_psgV_28H04_R_0	BB994507
E_ET_psgV_28H05_F_0	BB989799
E_ET_psgV_28H05_R_0	BB994508
E_ET_psgV_28H07_F_0	BB989800

E_ET_psgV_28H09_F_0	BB989801
E_ET_psgV_28H10_F_0	BB989802
E_ET_psgV_28H10_R_0	BB994509
E_ET_psgV_28H11_F_0	BB989803
E_ET_psgV_28H11_R_0	BB994510
E_ET_psgV_28H12_F_0	BB989804
E_ET_psgV_28H12_R_0	BB994511
E_ET_psgV_29A01_F_0	BB989805
E_ET_psgV_29A01_R_0	BB994512
E_ET_psgV_29A03_F_0	BB989806
E_ET_psgV_29A03_R_0	BB994513
E_ET_psgV_29A04_F_0	BB989807
E_ET_psgV_29A04_R_0	BB994514
E_ET_psgV_29A05_R_0	BB994515
E_ET_psgV_29A06_F_0	BB989808
E_ET_psgV_29A06_R_0	BB994516
E_ET_psgV_29A07_R_0	BB994517
E_ET_psgV_29A08_F_0	BB989809
E_ET_psgV_29A08_R_0	BB994518
E_ET_psgV_29A09_F_0	BB989810
E_ET_psgV_29A09_R_0	BB994519
E_ET_psgV_29A10_F_0	BB989811
E_ET_psgV_29A10_R_0	BB994520
E_ET_psgV_29A11_F_0	BB989812
E_ET_psgV_29A11_R_0	BB994521
E_ET_psgV_29A12_F_0	BB989813
E_ET_psgV_29B01_F_0	BB989814
E_ET_psgV_29B02_F_0	BB989815
E_ET_psgV_29B02_R_0	BB994522
E_ET_psgV_29B03_F_0	BB989816
E_ET_psgV_29B03_R_0	BB994523
E_ET_psgV_29B04_F_0	BB989817
E_ET_psgV_29B04_R_0	BB994524
E_ET_psgV_29B05_F_0	BB989818
E_ET_psgV_29B05_R_0	BB994525
E_ET_psgV_29B06_F_0	BB989819
E_ET_psgV_29B06_R_0	BB994526
E_ET_psgV_29B07_F_0	BB989820
E_ET_psgV_29B07_R_0	BB994527
E_ET_psgV_29B08_F_0	BB989821
E_ET_psgV_29B08_R_0	BB994528
E_ET_psgV_29B09_F_0	BB989822
E_ET_psgV_29B09_R_0	BB994529
E_ET_psgV_29B10_F_0	BB989823
E_ET_psgV_29B10_R_0	BB994530
E_ET_psgV_29B11_F_0	BB989824
E_ET_psgV_29B11_R_0	BB994531
E_ET_psgV_29B12_F_0	BB989825
E_ET_psgV_29C01_F_0	BB989826
E_ET_psgV_29C01_R_0	BB994532
E_ET_psgV_29C02_F_0	BB989827
E_ET_psgV_29C04_F_0	BB989828
E_ET_psgV_29C05_F_0	BB989829
E_ET_psgV_29C05_R_0	BB994533

E_ET_psgV_29C06_F_0	BB989830
E_ET_psgV_29C06_R_0	BB994534
E_ET_psgV_29C07_F_0	BB989831
E_ET_psgV_29C08_F_0	BB989832
E_ET_psgV_29C08_R_0	BB994535
E_ET_psgV_29C09_F_0	BB989833
E_ET_psgV_29C09_R_0	BB994536
E_ET_psgV_29C10_F_0	BB989834
E_ET_psgV_29C10_R_0	BB994537
E_ET_psgV_29C12_F_0	BB989835
E_ET_psgV_29C12_R_0	BB994538
E_ET_psgV_29D01_F_0	BB989836
E_ET_psgV_29D01_R_0	BB994539
E_ET_psgV_29D02_F_0	BB989837
E_ET_psgV_29D02_R_0	BB994540
E_ET_psgV_29D03_F_0	BB989838
E_ET_psgV_29D03_R_0	BB994541
E_ET_psgV_29D04_F_0	BB989839
E_ET_psgV_29D04_R_0	BB994542
E_ET_psgV_29D05_F_0	BB989840
E_ET_psgV_29D05_R_0	BB994543
E_ET_psgV_29D06_F_0	BB989841
E_ET_psgV_29D06_R_0	BB994544
E_ET_psgV_29D07_F_0	BB989842
E_ET_psgV_29D07_R_0	BB994545
E_ET_psgV_29D08_F_0	BB989843
E_ET_psgV_29D09_F_0	BB989844
E_ET_psgV_29D09_R_0	BB994546
E_ET_psgV_29D10_F_0	BB989845
E_ET_psgV_29D10_R_0	BB994547
E_ET_psgV_29D12_F_0	BB989846
E_ET_psgV_29D12_R_0	BB994548
E_ET_psgV_29E01_F_0	BB989847
E_ET_psgV_29E03_F_0	BB989848
E_ET_psgV_29E03_R_0	BB994549
E_ET_psgV_29E04_F_0	BB989849
E_ET_psgV_29E05_F_0	BB989850
E_ET_psgV_29E05_R_0	BB994550
E_ET_psgV_29E06_F_0	BB989851
E_ET_psgV_29E07_R_0	BB994551
E_ET_psgV_29E09_F_0	BB989852
E_ET_psgV_29E10_F_0	BB989853
E_ET_psgV_29E10_R_0	BB994552
E_ET_psgV_29E11_F_0	BB989854
E_ET_psgV_29E11_R_0	BB994553
E_ET_psgV_29E12_F_0	BB989855
E_ET_psgV_29E12_R_0	BB994554
E_ET_psgV_29F01_R_0	BB994555
E_ET_psgV_29F02_F_0	BB989856
E_ET_psgV_29F02_R_0	BB994556
E_ET_psgV_29F03_F_0	BB989857
E_ET_psgV_29F04_F_0	BB989858
E_ET_psgV_29F04_R_0	BB994557
E_ET_psgV_29F05_F_0	BB989859

E_ET_psgV_29F05_R_0	BB994558
E_ET_psgV_29F06_F_0	BB989860
E_ET_psgV_29F06_R_0	BB994559
E_ET_psgV_29F07_F_0	BB989861
E_ET_psgV_29F07_R_0	BB994560
E_ET_psgV_29F08_R_0	BB994561
E_ET_psgV_29F09_F_0	BB989862
E_ET_psgV_29F09_R_0	BB994562
E_ET_psgV_29F10_F_0	BB989863
E_ET_psgV_29F11_F_0	BB989864
E_ET_psgV_29F11_R_0	BB994563
E_ET_psgV_29F12_F_0	BB989865
E_ET_psgV_29G01_F_0	BB989866
E_ET_psgV_29G01_R_0	BB994564
E_ET_psgV_29G02_F_0	BB989867
E_ET_psgV_29G02_R_0	BB994565
E_ET_psgV_29G03_F_0	BB989868
E_ET_psgV_29G03_R_0	BB994566
E_ET_psgV_29G04_F_0	BB989869
E_ET_psgV_29G04_R_0	BB994567
E_ET_psgV_29G05_F_0	BB989870
E_ET_psgV_29G05_R_0	BB994568
E_ET_psgV_29G06_F_0	BB989871
E_ET_psgV_29G07_F_0	BB989872
E_ET_psgV_29G08_F_0	BB989873
E_ET_psgV_29G08_R_0	BB994569
E_ET_psgV_29G10_F_0	BB989874
E_ET_psgV_29G11_F_0	BB989875
E_ET_psgV_29G11_R_0	BB994570
E_ET_psgV_29G12_F_0	BB989876
E_ET_psgV_29G12_R_0	BB994571
E_ET_psgV_29H01_F_0	BB989877
E_ET_psgV_29H01_R_0	BB994572
E_ET_psgV_29H02_F_0	BB989878
E_ET_psgV_29H02_R_0	BB994573
E_ET_psgV_29H03_F_0	BB989879
E_ET_psgV_29H03_R_0	BB994574
E_ET_psgV_29H04_F_0	BB989880
E_ET_psgV_29H04_R_0	BB994575
E_ET_psgV_29H05_F_0	BB989881
E_ET_psgV_29H05_R_0	BB994576
E_ET_psgV_29H07_F_0	BB989882
E_ET_psgV_29H07_R_0	BB994577
E_ET_psgV_29H09_F_0	BB989883
E_ET_psgV_29H09_R_0	BB994578
E_ET_psgV_29H10_F_0	BB989884
E_ET_psgV_29H11_F_0	BB989885
E_ET_psgV_29H11_R_0	BB994579
E_ET_psgV_29H12_F_0	BB989886
E_ET_psgV_30A01_F_0	BB989887
E_ET_psgV_30A01_R_0	BB994580
E_ET_psgV_30A03_F_0	BB989888
E_ET_psgV_30A03_R_0	BB994581
E_ET_psgV_30A04_F_0	BB989889

E_ET_psgV_30A04_R_0	BB994582
E_ET_psgV_30A05_F_0	BB989890
E_ET_psgV_30A05_R_0	BB994583
E_ET_psgV_30A06_F_0	BB989891
E_ET_psgV_30A06_R_0	BB994584
E_ET_psgV_30A07_F_0	BB989892
E_ET_psgV_30A07_R_0	BB994585
E_ET_psgV_30A08_F_0	BB989893
E_ET_psgV_30A09_F_0	BB989894
E_ET_psgV_30A09_R_0	BB994586
E_ET_psgV_30A10_F_0	BB989895
E_ET_psgV_30A11_F_0	BB989896
E_ET_psgV_30A12_F_0	BB989897
E_ET_psgV_30A12_R_0	BB994587
E_ET_psgV_30B01_R_0	BB994588
E_ET_psgV_30B02_R_0	BB994589
E_ET_psgV_30B03_F_0	BB989898
E_ET_psgV_30B03_R_0	BB994590
E_ET_psgV_30B04_F_0	BB989899
E_ET_psgV_30B05_F_0	BB989900
E_ET_psgV_30B05_R_0	BB994591
E_ET_psgV_30B06_F_0	BB989901
E_ET_psgV_30B06_R_0	BB994592
E_ET_psgV_30B07_F_0	BB989902
E_ET_psgV_30B07_R_0	BB994593
E_ET_psgV_30B08_F_0	BB989903
E_ET_psgV_30B08_R_0	BB994594
E_ET_psgV_30B09_F_0	BB989904
E_ET_psgV_30B09_R_0	BB994595
E_ET_psgV_30B10_R_0	BB994596
E_ET_psgV_30B11_F_0	BB989905
E_ET_psgV_30B12_F_0	BB989906
E_ET_psgV_30B12_R_0	BB994597
E_ET_psgV_30C01_F_0	BB989907
E_ET_psgV_30C02_F_0	BB989908
E_ET_psgV_30C02_R_0	BB994598
E_ET_psgV_30C03_F_0	BB989909
E_ET_psgV_30C03_R_0	BB994599
E_ET_psgV_30C04_F_0	BB989910
E_ET_psgV_30C04_R_0	BB994600
E_ET_psgV_30C05_F_0	BB989911
E_ET_psgV_30C05_R_0	BB994601
E_ET_psgV_30C08_F_0	BB989912
E_ET_psgV_30C08_R_0	BB994602
E_ET_psgV_30C09_F_0	BB989913
E_ET_psgV_30C09_R_0	BB994603
E_ET_psgV_30C10_F_0	BB989914
E_ET_psgV_30C10_R_0	BB994604
E_ET_psgV_30C11_R_0	BB994605
E_ET_psgV_30C12_F_0	BB989915
E_ET_psgV_30C12_R_0	BB994606
E_ET_psgV_30D01_F_0	BB989916
E_ET_psgV_30D01_R_0	BB994607
E_ET_psgV_30D03_R_0	BB994608

E_ET_psgV_30D04_F_0	BB989917
E_ET_psgV_30D04_R_0	BB994609
E_ET_psgV_30D05_F_0	BB989918
E_ET_psgV_30D05_R_0	BB994610
E_ET_psgV_30D06_F_0	BB989919
E_ET_psgV_30D06_R_0	BB994611
E_ET_psgV_30D07_F_0	BB989920
E_ET_psgV_30D07_R_0	BB994612
E_ET_psgV_30D08_F_0	BB989921
E_ET_psgV_30D08_R_0	BB994613
E_ET_psgV_30D09_F_0	BB989922
E_ET_psgV_30D09_R_0	BB994614
E_ET_psgV_30D10_F_0	BB989923
E_ET_psgV_30D10_R_0	BB994615
E_ET_psgV_30D11_F_0	BB989924
E_ET_psgV_30D11_R_0	BB994616
E_ET_psgV_30D12_F_0	BB989925
E_ET_psgV_30D12_R_0	BB994617
E_ET_psgV_30E01_F_0	BB989926
E_ET_psgV_30E01_R_0	BB994618
E_ET_psgV_30E02_F_0	BB989927
E_ET_psgV_30E03_F_0	BB989928
E_ET_psgV_30E03_R_0	BB994619
E_ET_psgV_30E06_R_0	BB994620
E_ET_psgV_30E07_F_0	BB989929
E_ET_psgV_30E08_F_0	BB989930
E_ET_psgV_30E08_R_0	BB994621
E_ET_psgV_30E09_F_0	BB989931
E_ET_psgV_30E10_F_0	BB989932
E_ET_psgV_30E10_R_0	BB994622
E_ET_psgV_30E11_R_0	BB994623
E_ET_psgV_30E12_F_0	BB989933
E_ET_psgV_30E12_R_0	BB994624
E_ET_psgV_30F01_F_0	BB989934
E_ET_psgV_30F01_R_0	BB994625
E_ET_psgV_30F02_F_0	BB989935
E_ET_psgV_30F02_R_0	BB994626
E_ET_psgV_30F04_R_0	BB994627
E_ET_psgV_30F05_F_0	BB989936
E_ET_psgV_30F05_R_0	BB994628
E_ET_psgV_30F06_F_0	BB989937
E_ET_psgV_30F06_R_0	BB994629
E_ET_psgV_30F07_F_0	BB989938
E_ET_psgV_30F07_R_0	BB994630
E_ET_psgV_30F08_F_0	BB989939
E_ET_psgV_30F08_R_0	BB994631
E_ET_psgV_30F10_F_0	BB989940
E_ET_psgV_30F11_F_0	BB989941
E_ET_psgV_30F11_R_0	BB994632
E_ET_psgV_30F12_F_0	BB989942
E_ET_psgV_30F12_R_0	BB994633
E_ET_psgV_30G01_F_0	BB989943
E_ET_psgV_30G01_R_0	BB994634
E_ET_psgV_30G02_F_0	BB989944

E_ET_psgV_30G03_F_0	BB989945
E_ET_psgV_30G03_R_0	BB994635
E_ET_psgV_30G04_F_0	BB989946
E_ET_psgV_30G05_F_0	BB989947
E_ET_psgV_30G05_R_0	BB994636
E_ET_psgV_30G06_F_0	BB989948
E_ET_psgV_30G06_R_0	BB994637
E_ET_psgV_30G07_F_0	BB989949
E_ET_psgV_30G07_R_0	BB994638
E_ET_psgV_30G08_F_0	BB989950
E_ET_psgV_30G08_R_0	BB994639
E_ET_psgV_30G10_F_0	BB989951
E_ET_psgV_30G10_R_0	BB994640
E_ET_psgV_30G11_F_0	BB989952
E_ET_psgV_30G11_R_0	BB994641
E_ET_psgV_30G12_F_0	BB989953
E_ET_psgV_30G12_R_0	BB994642
E_ET_psgV_30H01_F_0	BB989954
E_ET_psgV_30H01_R_0	BB994643
E_ET_psgV_30H02_F_0	BB989955
E_ET_psgV_30H02_R_0	BB994644
E_ET_psgV_30H03_R_0	BB994645
E_ET_psgV_30H06_F_0	BB989956
E_ET_psgV_30H06_R_0	BB994646
E_ET_psgV_30H07_F_0	BB989957
E_ET_psgV_30H07_R_0	BB994647
E_ET_psgV_30H08_F_0	BB989958
E_ET_psgV_30H08_R_0	BB994648
E_ET_psgV_30H09_F_0	BB989959
E_ET_psgV_30H09_R_0	BB994649
E_ET_psgV_30H10_F_0	BB989960
E_ET_psgV_30H10_R_0	BB994650
E_ET_psgV_30H11_F_0	BB989961
E_ET_psgV_30H11_R_0	BB994651
E_ET_psgV_30H12_R_0	BB994652
E_ET_psgV_31A01_F_0	BB989962
E_ET_psgV_31A02_F_0	BB989963
E_ET_psgV_31A03_F_0	BB989964
E_ET_psgV_31A04_F_0	BB989965
E_ET_psgV_31A04_R_0	BB994653
E_ET_psgV_31A05_F_0	BB989966
E_ET_psgV_31A06_F_0	BB989967
E_ET_psgV_31A06_R_0	BB994654
E_ET_psgV_31A07_F_0	BB989968
E_ET_psgV_31A07_R_0	BB994655
E_ET_psgV_31A08_F_0	BB989969
E_ET_psgV_31A09_F_0	BB989970
E_ET_psgV_31A10_F_0	BB989971
E_ET_psgV_31A10_R_0	BB994656
E_ET_psgV_31A11_F_0	BB989972
E_ET_psgV_31A11_R_0	BB994657
E_ET_psgV_31A12_F_0	BB989973
E_ET_psgV_31B02_F_0	BB989974
E_ET_psgV_31B02_R_0	BB994658

E_ET_psgV_31B03_F_0	BB989975
E_ET_psgV_31B03_R_0	BB994659
E_ET_psgV_31B04_F_0	BB989976
E_ET_psgV_31B04_R_0	BB994660
E_ET_psgV_31B05_F_0	BB989977
E_ET_psgV_31B05_R_0	BB994661
E_ET_psgV_31B06_R_0	BB994662
E_ET_psgV_31B07_F_0	BB989978
E_ET_psgV_31B07_R_0	BB994663
E_ET_psgV_31B08_F_0	BB989979
E_ET_psgV_31B08_R_0	BB994664
E_ET_psgV_31B09_F_0	BB989980
E_ET_psgV_31B09_R_0	BB994665
E_ET_psgV_31B10_F_0	BB989981
E_ET_psgV_31B10_R_0	BB994666
E_ET_psgV_31B11_F_0	BB989982
E_ET_psgV_31B11_R_0	BB994667
E_ET_psgV_31B12_F_0	BB989983
E_ET_psgV_31B12_R_0	BB994668
E_ET_psgV_31C01_F_0	BB989984
E_ET_psgV_31C01_R_0	BB994669
E_ET_psgV_31C02_R_0	BB994670
E_ET_psgV_31C03_F_0	BB989985
E_ET_psgV_31C03_R_0	BB994671
E_ET_psgV_31C04_F_0	BB989986
E_ET_psgV_31C05_F_0	BB989987
E_ET_psgV_31C05_R_0	BB994672
E_ET_psgV_31C06_F_0	BB989988
E_ET_psgV_31C06_R_0	BB994673
E_ET_psgV_31C07_R_0	BB994674
E_ET_psgV_31C08_F_0	BB989989
E_ET_psgV_31C08_R_0	BB994675
E_ET_psgV_31C09_F_0	BB989990
E_ET_psgV_31C09_R_0	BB994676
E_ET_psgV_31C10_F_0	BB989991
E_ET_psgV_31C10_R_0	BB994677
E_ET_psgV_31C11_F_0	BB989992
E_ET_psgV_31C11_R_0	BB994678
E_ET_psgV_31C12_F_0	BB989993
E_ET_psgV_31C12_R_0	BB994679
E_ET_psgV_31D01_F_0	BB989994
E_ET_psgV_31D01_R_0	BB994680
E_ET_psgV_31D02_F_0	BB989995
E_ET_psgV_31D02_R_0	BB994681
E_ET_psgV_31D03_R_0	BB994682
E_ET_psgV_31D04_F_0	BB989996
E_ET_psgV_31D04_R_0	BB994683
E_ET_psgV_31D05_F_0	BB989997
E_ET_psgV_31D06_F_0	BB989998
E_ET_psgV_31D06_R_0	BB994684
E_ET_psgV_31D07_F_0	BB989999
E_ET_psgV_31D07_R_0	BB994685
E_ET_psgV_31D08_F_0	BB990000
E_ET_psgV_31D08_R_0	BB994686

E_ET_psgV_31D10_F_0	BB990001
E_ET_psgV_31D10_R_0	BB994687
E_ET_psgV_31D11_F_0	BB990002
E_ET_psgV_31D12_F_0	BB990003
E_ET_psgV_31D12_R_0	BB994688
E_ET_psgV_31E01_F_0	BB990004
E_ET_psgV_31E01_R_0	BB994689
E_ET_psgV_31E02_F_0	BB990005
E_ET_psgV_31E02_R_0	BB994690
E_ET_psgV_31E03_R_0	BB994691
E_ET_psgV_31E04_R_0	BB994692
E_ET_psgV_31E05_F_0	BB990006
E_ET_psgV_31E05_R_0	BB994693
E_ET_psgV_31E06_R_0	BB994694
E_ET_psgV_31E07_F_0	BB990007
E_ET_psgV_31E07_R_0	BB994695
E_ET_psgV_31E08_F_0	BB990008
E_ET_psgV_31E08_R_0	BB994696
E_ET_psgV_31E09_F_0	BB990009
E_ET_psgV_31E09_R_0	BB994697
E_ET_psgV_31E10_R_0	BB994698
E_ET_psgV_31E11_F_0	BB990010
E_ET_psgV_31E11_R_0	BB994699
E_ET_psgV_31F01_F_0	BB990011
E_ET_psgV_31F01_R_0	BB994700
E_ET_psgV_31F02_F_0	BB990012
E_ET_psgV_31F02_R_0	BB994701
E_ET_psgV_31F03_R_0	BB994702
E_ET_psgV_31F04_R_0	BB994703
E_ET_psgV_31F06_F_0	BB990013
E_ET_psgV_31F06_R_0	BB994704
E_ET_psgV_31F07_F_0	BB990014
E_ET_psgV_31F07_R_0	BB994705
E_ET_psgV_31F08_F_0	BB990015
E_ET_psgV_31F08_R_0	BB994706
E_ET_psgV_31F09_F_0	BB990016
E_ET_psgV_31F10_F_0	BB990017
E_ET_psgV_31F10_R_0	BB994707
E_ET_psgV_31F11_F_0	BB990018
E_ET_psgV_31F11_R_0	BB994708
E_ET_psgV_31F12_F_0	BB990019
E_ET_psgV_31F12_R_0	BB994709
E_ET_psgV_31G01_F_0	BB990020
E_ET_psgV_31G02_R_0	BB994710
E_ET_psgV_31G03_F_0	BB990021
E_ET_psgV_31G03_R_0	BB994711
E_ET_psgV_31G04_F_0	BB990022
E_ET_psgV_31G04_R_0	BB994712
E_ET_psgV_31G06_F_0	BB990023
E_ET_psgV_31G06_R_0	BB994713
E_ET_psgV_31G08_F_0	BB990024
E_ET_psgV_31G08_R_0	BB994714
E_ET_psgV_31G09_F_0	BB990025
E_ET_psgV_31G09_R_0	BB994715

E_ET_psgV_31G10_F_0	BB990026
E_ET_psgV_31G10_R_0	BB994716
E_ET_psgV_31G11_F_0	BB990027
E_ET_psgV_31G12_F_0	BB990028
E_ET_psgV_31H01_F_0	BB990029
E_ET_psgV_31H02_R_0	BB994717
E_ET_psgV_31H04_F_0	BB990030
E_ET_psgV_31H05_F_0	BB990031
E_ET_psgV_31H06_F_0	BB990032
E_ET_psgV_31H06_R_0	BB994718
E_ET_psgV_31H07_F_0	BB990033
E_ET_psgV_31H08_F_0	BB990034
E_ET_psgV_31H08_R_0	BB994719
E_ET_psgV_31H09_F_0	BB990035
E_ET_psgV_31H10_F_0	BB990036
E_ET_psgV_31H10_R_0	BB994720
E_ET_psgV_31H11_F_0	BB990037
E_ET_psgV_31H11_R_0	BB994721
E_ET_psgV_32A02_F_0	BB990038
E_ET_psgV_32A02_R_0	BB994722
E_ET_psgV_32A03_F_0	BB990039
E_ET_psgV_32A03_R_0	BB994723
E_ET_psgV_32A04_F_0	BB990040
E_ET_psgV_32A04_R_0	BB994724
E_ET_psgV_32A05_F_0	BB990041
E_ET_psgV_32A05_R_0	BB994725
E_ET_psgV_32A06_F_0	BB990042
E_ET_psgV_32A06_R_0	BB994726
E_ET_psgV_32A07_F_0	BB990043
E_ET_psgV_32A07_R_0	BB994727
E_ET_psgV_32A08_F_0	BB990044
E_ET_psgV_32A08_R_0	BB994728
E_ET_psgV_32A09_F_0	BB990045
E_ET_psgV_32A10_F_0	BB990046
E_ET_psgV_32A10_R_0	BB994729
E_ET_psgV_32A11_F_0	BB990047
E_ET_psgV_32A12_F_0	BB990048
E_ET_psgV_32A12_R_0	BB994730
E_ET_psgV_32B01_F_0	BB990049
E_ET_psgV_32B01_R_0	BB994731
E_ET_psgV_32B02_R_0	BB994732
E_ET_psgV_32B03_F_0	BB990050
E_ET_psgV_32B04_F_0	BB990051
E_ET_psgV_32B04_R_0	BB994733
E_ET_psgV_32B05_F_0	BB990052
E_ET_psgV_32B05_R_0	BB994734
E_ET_psgV_32B06_F_0	BB990053
E_ET_psgV_32B06_R_0	BB994735
E_ET_psgV_32B08_F_0	BB990054
E_ET_psgV_32B08_R_0	BB994736
E_ET_psgV_32B09_F_0	BB990055
E_ET_psgV_32B09_R_0	BB994737
E_ET_psgV_32B10_F_0	BB990056
E_ET_psgV_32B10_R_0	BB994738

E_ET_psgV_32B11_F_0	BB990057
E_ET_psgV_32B11_R_0	BB994739
E_ET_psgV_32B12_F_0	BB990058
E_ET_psgV_32B12_R_0	BB994740
E_ET_psgV_32C01_F_0	BB990059
E_ET_psgV_32C01_R_0	BB994741
E_ET_psgV_32C02_F_0	BB990060
E_ET_psgV_32C02_R_0	BB994742
E_ET_psgV_32C04_F_0	BB990061
E_ET_psgV_32C04_R_0	BB994743
E_ET_psgV_32C05_F_0	BB990062
E_ET_psgV_32C05_R_0	BB994744
E_ET_psgV_32C06_F_0	BB990063
E_ET_psgV_32C06_R_0	BB994745
E_ET_psgV_32C07_F_0	BB990064
E_ET_psgV_32C07_R_0	BB994746
E_ET_psgV_32C08_F_0	BB990065
E_ET_psgV_32C09_F_0	BB990066
E_ET_psgV_32C09_R_0	BB994747
E_ET_psgV_32C10_F_0	BB990067
E_ET_psgV_32C10_R_0	BB994748
E_ET_psgV_32C12_F_0	BB990068
E_ET_psgV_32C12_R_0	BB994749
E_ET_psgV_32D01_F_0	BB990069
E_ET_psgV_32D01_R_0	BB994750
E_ET_psgV_32D02_F_0	BB990070
E_ET_psgV_32D02_R_0	BB994751
E_ET_psgV_32D03_R_0	BB994752
E_ET_psgV_32D05_F_0	BB990071
E_ET_psgV_32D05_R_0	BB994753
E_ET_psgV_32D06_F_0	BB990072
E_ET_psgV_32D06_R_0	BB994754
E_ET_psgV_32D07_F_0	BB990073
E_ET_psgV_32D07_R_0	BB994755
E_ET_psgV_32D08_F_0	BB990074
E_ET_psgV_32D08_R_0	BB994756
E_ET_psgV_32D10_F_0	BB990075
E_ET_psgV_32D12_F_0	BB990076
E_ET_psgV_32D12_R_0	BB994757
E_ET_psgV_32E01_R_0	BB994758
E_ET_psgV_32E02_F_0	BB990077
E_ET_psgV_32E02_R_0	BB994759
E_ET_psgV_32E03_F_0	BB990078
E_ET_psgV_32E03_R_0	BB994760
E_ET_psgV_32E04_R_0	BB994761
E_ET_psgV_32E05_R_0	BB994762
E_ET_psgV_32E06_R_0	BB994763
E_ET_psgV_32E07_F_0	BB990079
E_ET_psgV_32E07_R_0	BB994764
E_ET_psgV_32E08_F_0	BB990080
E_ET_psgV_32E08_R_0	BB994765
E_ET_psgV_32E09_R_0	BB994766
E_ET_psgV_32E10_F_0	BB990081
E_ET_psgV_32E10_R_0	BB994767

E_ET_psgV_32E11_F_0	BB990082
E_ET_psgV_32E11_R_0	BB994768
E_ET_psgV_32E12_F_0	BB990083
E_ET_psgV_32F01_F_0	BB990084
E_ET_psgV_32F01_R_0	BB994769
E_ET_psgV_32F02_F_0	BB990085
E_ET_psgV_32F02_R_0	BB994770
E_ET_psgV_32F03_F_0	BB990086
E_ET_psgV_32F03_R_0	BB994771
E_ET_psgV_32F04_F_0	BB990087
E_ET_psgV_32F04_R_0	BB994772
E_ET_psgV_32F05_F_0	BB990088
E_ET_psgV_32F06_F_0	BB990089
E_ET_psgV_32F08_F_0	BB990090
E_ET_psgV_32F08_R_0	BB994773
E_ET_psgV_32F09_F_0	BB990091
E_ET_psgV_32F09_R_0	BB994774
E_ET_psgV_32F10_R_0	BB994775
E_ET_psgV_32F12_F_0	BB990092
E_ET_psgV_32G01_F_0	BB990093
E_ET_psgV_32G01_R_0	BB994776
E_ET_psgV_32G02_F_0	BB990094
E_ET_psgV_32G02_R_0	BB994777
E_ET_psgV_32G03_F_0	BB990095
E_ET_psgV_32G03_R_0	BB994778
E_ET_psgV_32G04_F_0	BB990096
E_ET_psgV_32G04_R_0	BB994779
E_ET_psgV_32G05_R_0	BB994780
E_ET_psgV_32G06_F_0	BB990097
E_ET_psgV_32G06_R_0	BB994781
E_ET_psgV_32G07_R_0	BB994782
E_ET_psgV_32G08_F_0	BB990098
E_ET_psgV_32G08_R_0	BB994783
E_ET_psgV_32G09_F_0	BB990099
E_ET_psgV_32G09_R_0	BB994784
E_ET_psgV_32G10_F_0	BB990100
E_ET_psgV_32G10_R_0	BB994785
E_ET_psgV_32G11_R_0	BB994786
E_ET_psgV_32G12_F_0	BB990101
E_ET_psgV_32G12_R_0	BB994787
E_ET_psgV_32H01_F_0	BB990102
E_ET_psgV_32H01_R_0	BB994788
E_ET_psgV_32H02_F_0	BB990103
E_ET_psgV_32H03_F_0	BB990104
E_ET_psgV_32H03_R_0	BB994789
E_ET_psgV_32H04_F_0	BB990105
E_ET_psgV_32H05_F_0	BB990106
E_ET_psgV_32H05_R_0	BB994790
E_ET_psgV_32H06_F_0	BB990107
E_ET_psgV_32H07_F_0	BB990108
E_ET_psgV_32H07_R_0	BB994791
E_ET_psgV_32H08_F_0	BB990109
E_ET_psgV_32H08_R_0	BB994792
E_ET_psgV_32H09_F_0	BB990110

	E_ET_psgV_32H09_R_0	BB994793
	E_ET_psgV_32H10_F_0	BB990111
	E_ET_psgV_32H10_R_0	BB994794
	E_ET_psgV_32H11_F_0	BB990112
	E_ET_psgV_32H11_R_0	BB994795
	E_ET_psgV_32H12_F_0	BB990113
	E_ET_psgV_32H12_R_0	BB994796
BmN-	E_FL_BmN-_01A02_F_0	BJ979997
	E_FL_BmN-_01A02_R_0	BJ986280
	E_FL_BmN-_01A03_F_0	BJ979998
	E_FL_BmN-_01A03_R_0	BJ986281
	E_FL_BmN-_01A04_F_0	BJ979999
	E_FL_BmN-_01A04_R_0	BJ986282
	E_FL_BmN-_01A05_R_0	BJ986283
	E_FL_BmN-_01A06_F_0	BJ980000
	E_FL_BmN-_01A06_R_0	BJ986284
	E_FL_BmN-_01A07_F_0	BJ980001
	E_FL_BmN-_01A07_R_0	BJ986285
	E_FL_BmN-_01A08_F_0	BJ980002
	E_FL_BmN-_01A08_R_0	BJ986286
	E_FL_BmN-_01A09_F_0	BJ980003
	E_FL_BmN-_01A09_R_0	BJ986287
	E_FL_BmN-_01A10_F_0	BJ980004
	E_FL_BmN-_01A10_R_0	BJ986288
	E_FL_BmN-_01A11_F_0	BJ980005
	E_FL_BmN-_01A11_R_0	BJ986289
	E_FL_BmN-_01A12_F_0	BJ980006
	E_FL_BmN-_01A12_R_0	BJ986290
	E_FL_BmN-_01A13_R_0	BJ986291
	E_FL_BmN-_01A14_F_0	BJ980007
	E_FL_BmN-_01A14_R_0	BJ986292
	E_FL_BmN-_01A15_F_0	BJ980008
	E_FL_BmN-_01A15_R_0	BJ986293
	E_FL_BmN-_01A16_F_0	BJ980009
	E_FL_BmN-_01A16_R_0	BJ986294
	E_FL_BmN-_01A17_F_0	BJ980010
	E_FL_BmN-_01A17_R_0	BJ986295
	E_FL_BmN-_01A18_F_0	BJ980011
	E_FL_BmN-_01A18_R_0	BJ986296
	E_FL_BmN-_01A19_F_0	BJ980012
	E_FL_BmN-_01A19_R_0	BJ986297
	E_FL_BmN-_01A20_F_0	BJ980013
	E_FL_BmN-_01A20_R_0	BJ986298
	E_FL_BmN-_01A21_F_0	BJ980014
	E_FL_BmN-_01A21_R_0	BJ986299
	E_FL_BmN-_01A22_F_0	BJ980015
	E_FL_BmN-_01A22_R_0	BJ986300
	E_FL_BmN-_01A23_F_0	BJ980016
	E_FL_BmN-_01A24_F_0	BJ980017
	E_FL_BmN-_01B01_F_0	BJ980018
	E_FL_BmN-_01B01_R_0	BJ986301
	E_FL_BmN-_01B02_F_0	BJ980019
	E_FL_BmN-_01B02_R_0	BJ986302
	E_FL_BmN-_01B03_F_0	BJ980020

E_FL_BmN-_01B03_R_0	BJ986303
E_FL_BmN-_01B04_F_0	BJ980021
E_FL_BmN-_01B04_R_0	BJ986304
E_FL_BmN-_01B05_F_0	BJ980022
E_FL_BmN-_01B05_R_0	BJ986305
E_FL_BmN-_01B06_R_0	BJ986306
E_FL_BmN-_01B07_R_0	BJ986307
E_FL_BmN-_01B08_F_0	BJ980023
E_FL_BmN-_01B08_R_0	BJ986308
E_FL_BmN-_01B09_F_0	BJ980024
E_FL_BmN-_01B09_R_0	BJ986309
E_FL_BmN-_01B10_F_0	BJ980025
E_FL_BmN-_01B11_F_0	BJ980026
E_FL_BmN-_01B11_R_0	BJ986310
E_FL_BmN-_01B12_F_0	BJ980027
E_FL_BmN-_01B12_R_0	BJ986311
E_FL_BmN-_01B13_R_0	BJ986312
E_FL_BmN-_01B14_F_0	BJ980028
E_FL_BmN-_01B14_R_0	BJ986313
E_FL_BmN-_01B15_F_0	BJ980029
E_FL_BmN-_01B15_R_0	BJ986314
E_FL_BmN-_01B16_F_0	BJ980030
E_FL_BmN-_01B16_R_0	BJ986315
E_FL_BmN-_01B17_F_0	BJ980031
E_FL_BmN-_01B17_R_0	BJ986316
E_FL_BmN-_01B19_F_0	BJ980032
E_FL_BmN-_01B19_R_0	BJ986317
E_FL_BmN-_01B20_F_0	BJ980033
E_FL_BmN-_01B20_R_0	BJ986318
E_FL_BmN-_01B21_F_0	BJ980034
E_FL_BmN-_01B21_R_0	BJ986319
E_FL_BmN-_01B22_F_0	BJ980035
E_FL_BmN-_01B22_R_0	BJ986320
E_FL_BmN-_01B23_F_0	BJ980036
E_FL_BmN-_01B23_R_0	BJ986321
E_FL_BmN-_01B24_F_0	BJ980037
E_FL_BmN-_01B24_R_0	BJ986322
E_FL_BmN-_01C01_F_0	BJ980038
E_FL_BmN-_01C01_R_0	BJ986323
E_FL_BmN-_01C02_F_0	BJ980039
E_FL_BmN-_01C02_R_0	BJ986324
E_FL_BmN-_01C03_F_0	BJ980040
E_FL_BmN-_01C03_R_0	BJ986325
E_FL_BmN-_01C04_F_0	BJ980041
E_FL_BmN-_01C04_R_0	BJ986326
E_FL_BmN-_01C05_F_0	BJ980042
E_FL_BmN-_01C05_R_0	BJ986327
E_FL_BmN-_01C06_F_0	BJ980043
E_FL_BmN-_01C06_R_0	BJ986328
E_FL_BmN-_01C07_F_0	BJ980044
E_FL_BmN-_01C07_R_0	BJ986329
E_FL_BmN-_01C08_R_0	BJ986330
E_FL_BmN-_01C10_F_0	BJ980045
E_FL_BmN-_01C10_R_0	BJ986331

E_FL_BmN-_01C12_R_0	BJ986332
E_FL_BmN-_01C13_F_0	BJ980046
E_FL_BmN-_01C13_R_0	BJ986333
E_FL_BmN-_01C14_F_0	BJ980047
E_FL_BmN-_01C14_R_0	BJ986334
E_FL_BmN-_01C16_F_0	BJ980048
E_FL_BmN-_01C16_R_0	BJ986335
E_FL_BmN-_01C17_F_0	BJ980049
E_FL_BmN-_01C17_R_0	BJ986336
E_FL_BmN-_01C18_F_0	BJ980050
E_FL_BmN-_01C18_R_0	BJ986337
E_FL_BmN-_01C19_F_0	BJ980051
E_FL_BmN-_01C19_R_0	BJ986338
E_FL_BmN-_01C21_F_0	BJ980052
E_FL_BmN-_01C21_R_0	BJ986339
E_FL_BmN-_01C22_F_0	BJ980053
E_FL_BmN-_01C22_R_0	BJ986340
E_FL_BmN-_01D01_F_0	BJ980054
E_FL_BmN-_01D01_R_0	BJ986341
E_FL_BmN-_01D02_F_0	BJ980055
E_FL_BmN-_01D02_R_0	BJ986342
E_FL_BmN-_01D03_F_0	BJ980056
E_FL_BmN-_01D03_R_0	BJ986343
E_FL_BmN-_01D04_F_0	BJ980057
E_FL_BmN-_01D04_R_0	BJ986344
E_FL_BmN-_01D05_F_0	BJ980058
E_FL_BmN-_01D05_R_0	BJ986345
E_FL_BmN-_01D06_F_0	BJ980059
E_FL_BmN-_01D06_R_0	BJ986346
E_FL_BmN-_01D07_F_0	BJ980060
E_FL_BmN-_01D07_R_0	BJ986347
E_FL_BmN-_01D08_F_0	BJ980061
E_FL_BmN-_01D08_R_0	BJ986348
E_FL_BmN-_01D09_F_0	BJ980062
E_FL_BmN-_01D09_R_0	BJ986349
E_FL_BmN-_01D10_F_0	BJ980063
E_FL_BmN-_01D10_R_0	BJ986350
E_FL_BmN-_01D13_R_0	BJ986351
E_FL_BmN-_01D14_F_0	BJ980064
E_FL_BmN-_01D14_R_0	BJ986352
E_FL_BmN-_01D15_F_0	BJ980065
E_FL_BmN-_01D15_R_0	BJ986353
E_FL_BmN-_01D16_F_0	BJ980066
E_FL_BmN-_01D16_R_0	BJ986354
E_FL_BmN-_01D17_F_0	BJ980067
E_FL_BmN-_01D17_R_0	BJ986355
E_FL_BmN-_01D18_F_0	BJ980068
E_FL_BmN-_01D18_R_0	BJ986356
E_FL_BmN-_01D19_F_0	BJ980069
E_FL_BmN-_01D19_R_0	BJ986357
E_FL_BmN-_01D20_F_0	BJ980070
E_FL_BmN-_01D20_R_0	BJ986358
E_FL_BmN-_01D21_F_0	BJ980071
E_FL_BmN-_01D21_R_0	BJ986359

E_FL_BmN-_01D22_F_0	BJ980072
E_FL_BmN-_01D22_R_0	BJ986360
E_FL_BmN-_01D23_F_0	BJ980073
E_FL_BmN-_01D23_R_0	BJ986361
E_FL_BmN-_01D24_F_0	BJ980074
E_FL_BmN-_01D24_R_0	BJ986362
E_FL_BmN-_01E01_F_0	BJ980075
E_FL_BmN-_01E01_R_0	BJ986363
E_FL_BmN-_01E02_F_0	BJ980076
E_FL_BmN-_01E02_R_0	BJ986364
E_FL_BmN-_01E03_F_0	BJ980077
E_FL_BmN-_01E03_R_0	BJ986365
E_FL_BmN-_01E05_F_0	BJ980078
E_FL_BmN-_01E06_R_0	BJ986366
E_FL_BmN-_01E07_F_0	BJ980079
E_FL_BmN-_01E07_R_0	BJ986367
E_FL_BmN-_01E08_F_0	BJ980080
E_FL_BmN-_01E08_R_0	BJ986368
E_FL_BmN-_01E09_F_0	BJ980081
E_FL_BmN-_01E09_R_0	BJ986369
E_FL_BmN-_01E10_F_0	BJ980082
E_FL_BmN-_01E10_R_0	BJ986370
E_FL_BmN-_01E11_F_0	BJ980083
E_FL_BmN-_01E11_R_0	BJ986371
E_FL_BmN-_01E12_F_0	BJ980084
E_FL_BmN-_01E12_R_0	BJ986372
E_FL_BmN-_01E13_F_0	BJ980085
E_FL_BmN-_01E13_R_0	BJ986373
E_FL_BmN-_01E14_F_0	BJ980086
E_FL_BmN-_01E14_R_0	BJ986374
E_FL_BmN-_01E15_F_0	BJ980087
E_FL_BmN-_01E15_R_0	BJ986375
E_FL_BmN-_01E16_F_0	BJ980088
E_FL_BmN-_01E16_R_0	BJ986376
E_FL_BmN-_01E17_R_0	BJ986377
E_FL_BmN-_01E18_F_0	BJ980089
E_FL_BmN-_01E18_R_0	BJ986378
E_FL_BmN-_01E19_F_0	BJ980090
E_FL_BmN-_01E19_R_0	BJ986379
E_FL_BmN-_01E20_F_0	BJ980091
E_FL_BmN-_01E20_R_0	BJ986380
E_FL_BmN-_01E21_F_0	BJ980092
E_FL_BmN-_01E21_R_0	BJ986381
E_FL_BmN-_01E22_F_0	BJ980093
E_FL_BmN-_01E22_R_0	BJ986382
E_FL_BmN-_01E23_F_0	BJ980094
E_FL_BmN-_01E23_R_0	BJ986383
E_FL_BmN-_01E24_F_0	BJ980095
E_FL_BmN-_01F02_R_0	BJ986384
E_FL_BmN-_01F03_F_0	BJ980096
E_FL_BmN-_01F03_R_0	BJ986385
E_FL_BmN-_01F04_F_0	BJ980097
E_FL_BmN-_01F04_R_0	BJ986386
E_FL_BmN-_01F05_F_0	BJ980098

E_FL_BmN-_01F05_R_0	BJ986387
E_FL_BmN-_01F06_F_0	BJ980099
E_FL_BmN-_01F06_R_0	BJ986388
E_FL_BmN-_01F07_R_0	BJ986389
E_FL_BmN-_01F08_F_0	BJ980100
E_FL_BmN-_01F08_R_0	BJ986390
E_FL_BmN-_01F09_F_0	BJ980101
E_FL_BmN-_01F09_R_0	BJ986391
E_FL_BmN-_01F11_F_0	BJ980102
E_FL_BmN-_01F11_R_0	BJ986392
E_FL_BmN-_01F12_F_0	BJ980103
E_FL_BmN-_01F12_R_0	BJ986393
E_FL_BmN-_01F13_R_0	BJ986394
E_FL_BmN-_01F14_F_0	BJ980104
E_FL_BmN-_01F14_R_0	BJ986395
E_FL_BmN-_01F15_F_0	BJ980105
E_FL_BmN-_01F15_R_0	BJ986396
E_FL_BmN-_01F16_F_0	BJ980106
E_FL_BmN-_01F17_F_0	BJ980107
E_FL_BmN-_01F17_R_0	BJ986397
E_FL_BmN-_01F19_F_0	BJ980108
E_FL_BmN-_01F19_R_0	BJ986398
E_FL_BmN-_01F20_R_0	BJ986399
E_FL_BmN-_01F21_F_0	BJ980109
E_FL_BmN-_01F21_R_0	BJ986400
E_FL_BmN-_01F22_F_0	BJ980110
E_FL_BmN-_01F22_R_0	BJ986401
E_FL_BmN-_01F23_F_0	BJ980111
E_FL_BmN-_01F23_R_0	BJ986402
E_FL_BmN-_01F24_F_0	BJ980112
E_FL_BmN-_01F24_R_0	BJ986403
E_FL_BmN-_01G01_F_0	BJ980113
E_FL_BmN-_01G01_R_0	BJ986404
E_FL_BmN-_01G02_F_0	BJ980114
E_FL_BmN-_01G02_R_0	BJ986405
E_FL_BmN-_01G03_F_0	BJ980115
E_FL_BmN-_01G03_R_0	BJ986406
E_FL_BmN-_01G04_F_0	BJ980116
E_FL_BmN-_01G04_R_0	BJ986407
E_FL_BmN-_01G05_F_0	BJ980117
E_FL_BmN-_01G05_R_0	BJ986408
E_FL_BmN-_01G06_F_0	BJ980118
E_FL_BmN-_01G06_R_0	BJ986409
E_FL_BmN-_01G07_F_0	BJ980119
E_FL_BmN-_01G07_R_0	BJ986410
E_FL_BmN-_01G08_F_0	BJ980120
E_FL_BmN-_01G08_R_0	BJ986411
E_FL_BmN-_01G09_F_0	BJ980121
E_FL_BmN-_01G10_F_0	BJ980122
E_FL_BmN-_01G10_R_0	BJ986412
E_FL_BmN-_01G11_F_0	BJ980123
E_FL_BmN-_01G11_R_0	BJ986413
E_FL_BmN-_01G12_R_0	BJ986414
E_FL_BmN-_01G13_F_0	BJ980124

E_FL_BmN-_01G13_R_0	BJ986415
E_FL_BmN-_01G14_F_0	BJ980125
E_FL_BmN-_01G14_R_0	BJ986416
E_FL_BmN-_01G15_F_0	BJ980126
E_FL_BmN-_01G15_R_0	BJ986417
E_FL_BmN-_01G16_F_0	BJ980127
E_FL_BmN-_01G16_R_0	BJ986418
E_FL_BmN-_01G17_F_0	BJ980128
E_FL_BmN-_01G17_R_0	BJ986419
E_FL_BmN-_01G18_R_0	BJ986420
E_FL_BmN-_01G19_F_0	BJ980129
E_FL_BmN-_01G20_F_0	BJ980130
E_FL_BmN-_01G20_R_0	BJ986421
E_FL_BmN-_01G21_F_0	BJ980131
E_FL_BmN-_01G21_R_0	BJ986422
E_FL_BmN-_01G22_F_0	BJ980132
E_FL_BmN-_01G22_R_0	BJ986423
E_FL_BmN-_01G23_F_0	BJ980133
E_FL_BmN-_01G23_R_0	BJ986424
E_FL_BmN-_01G24_F_0	BJ980134
E_FL_BmN-_01G24_R_0	BJ986425
E_FL_BmN-_01H01_F_0	BJ980135
E_FL_BmN-_01H02_F_0	BJ980136
E_FL_BmN-_01H02_R_0	BJ986426
E_FL_BmN-_01H03_F_0	BJ980137
E_FL_BmN-_01H03_R_0	BJ986427
E_FL_BmN-_01H04_F_0	BJ980138
E_FL_BmN-_01H04_R_0	BJ986428
E_FL_BmN-_01H05_F_0	BJ980139
E_FL_BmN-_01H05_R_0	BJ986429
E_FL_BmN-_01H06_F_0	BJ980140
E_FL_BmN-_01H06_R_0	BJ986430
E_FL_BmN-_01H07_F_0	BJ980141
E_FL_BmN-_01H08_F_0	BJ980142
E_FL_BmN-_01H09_F_0	BJ980143
E_FL_BmN-_01H09_R_0	BJ986431
E_FL_BmN-_01H10_F_0	BJ980144
E_FL_BmN-_01H10_R_0	BJ986432
E_FL_BmN-_01H11_F_0	BJ980145
E_FL_BmN-_01H11_R_0	BJ986433
E_FL_BmN-_01H12_F_0	BJ980146
E_FL_BmN-_01H12_R_0	BJ986434
E_FL_BmN-_01H13_F_0	BJ980147
E_FL_BmN-_01H13_R_0	BJ986435
E_FL_BmN-_01H14_F_0	BJ980148
E_FL_BmN-_01H14_R_0	BJ986436
E_FL_BmN-_01H15_F_0	BJ980149
E_FL_BmN-_01H15_R_0	BJ986437
E_FL_BmN-_01H16_F_0	BJ980150
E_FL_BmN-_01H16_R_0	BJ986438
E_FL_BmN-_01H17_F_0	BJ980151
E_FL_BmN-_01H17_R_0	BJ986439
E_FL_BmN-_01H18_F_0	BJ980152
E_FL_BmN-_01H18_R_0	BJ986440

E_FL_BmN-_01H19_F_0	BJ980153
E_FL_BmN-_01H19_R_0	BJ986441
E_FL_BmN-_01H20_F_0	BJ980154
E_FL_BmN-_01H20_R_0	BJ986442
E_FL_BmN-_01H21_F_0	BJ980155
E_FL_BmN-_01H21_R_0	BJ986443
E_FL_BmN-_01H22_F_0	BJ980156
E_FL_BmN-_01H22_R_0	BJ986444
E_FL_BmN-_01H23_F_0	BJ980157
E_FL_BmN-_01H23_R_0	BJ986445
E_FL_BmN-_01H24_F_0	BJ980158
E_FL_BmN-_01I01_F_0	BJ980159
E_FL_BmN-_01I01_R_0	BJ986446
E_FL_BmN-_01I02_F_0	BJ980160
E_FL_BmN-_01I02_R_0	BJ986447
E_FL_BmN-_01I03_F_0	BJ980161
E_FL_BmN-_01I03_R_0	BJ986448
E_FL_BmN-_01I04_F_0	BJ980162
E_FL_BmN-_01I04_R_0	BJ986449
E_FL_BmN-_01I05_F_0	BJ980163
E_FL_BmN-_01I06_R_0	BJ986450
E_FL_BmN-_01I07_F_0	BJ980164
E_FL_BmN-_01I07_R_0	BJ986451
E_FL_BmN-_01I08_F_0	BJ980165
E_FL_BmN-_01I08_R_0	BJ986452
E_FL_BmN-_01I09_F_0	BJ980166
E_FL_BmN-_01I09_R_0	BJ986453
E_FL_BmN-_01I10_F_0	BJ980167
E_FL_BmN-_01I10_R_0	BJ986454
E_FL_BmN-_01I11_F_0	BJ980168
E_FL_BmN-_01I11_R_0	BJ986455
E_FL_BmN-_01I12_F_0	BJ980169
E_FL_BmN-_01I12_R_0	BJ986456
E_FL_BmN-_01I13_F_0	BJ980170
E_FL_BmN-_01I13_R_0	BJ986457
E_FL_BmN-_01I14_F_0	BJ980171
E_FL_BmN-_01I14_R_0	BJ986458
E_FL_BmN-_01I15_F_0	BJ980172
E_FL_BmN-_01I15_R_0	BJ986459
E_FL_BmN-_01I16_F_0	BJ980173
E_FL_BmN-_01I17_R_0	BJ986460
E_FL_BmN-_01I18_F_0	BJ980174
E_FL_BmN-_01I18_R_0	BJ986461
E_FL_BmN-_01I19_F_0	BJ980175
E_FL_BmN-_01I20_F_0	BJ980176
E_FL_BmN-_01I20_R_0	BJ986462
E_FL_BmN-_01I21_F_0	BJ980177
E_FL_BmN-_01I21_R_0	BJ986463
E_FL_BmN-_01I22_F_0	BJ980178
E_FL_BmN-_01I22_R_0	BJ986464
E_FL_BmN-_01I23_F_0	BJ980179
E_FL_BmN-_01I23_R_0	BJ986465
E_FL_BmN-_01I24_F_0	BJ980180
E_FL_BmN-_01I24_R_0	BJ986466

E_FL_BmN-_01J01_F_0	BJ980181
E_FL_BmN-_01J03_F_0	BJ980182
E_FL_BmN-_01J04_F_0	BJ980183
E_FL_BmN-_01J05_F_0	BJ980184
E_FL_BmN-_01J05_R_0	BJ986467
E_FL_BmN-_01J06_F_0	BJ980185
E_FL_BmN-_01J06_R_0	BJ986468
E_FL_BmN-_01J07_F_0	BJ980186
E_FL_BmN-_01J07_R_0	BJ986469
E_FL_BmN-_01J08_F_0	BJ980187
E_FL_BmN-_01J08_R_0	BJ986470
E_FL_BmN-_01J09_F_0	BJ980188
E_FL_BmN-_01J09_R_0	BJ986471
E_FL_BmN-_01J10_F_0	BJ980189
E_FL_BmN-_01J10_R_0	BJ986472
E_FL_BmN-_01J11_F_0	BJ980190
E_FL_BmN-_01J11_R_0	BJ986473
E_FL_BmN-_01J12_F_0	BJ980191
E_FL_BmN-_01J12_R_0	BJ986474
E_FL_BmN-_01J13_F_0	BJ980192
E_FL_BmN-_01J13_R_0	BJ986475
E_FL_BmN-_01J14_F_0	BJ980193
E_FL_BmN-_01J14_R_0	BJ986476
E_FL_BmN-_01J15_F_0	BJ980194
E_FL_BmN-_01J15_R_0	BJ986477
E_FL_BmN-_01J16_F_0	BJ980195
E_FL_BmN-_01J16_R_0	BJ986478
E_FL_BmN-_01J17_F_0	BJ980196
E_FL_BmN-_01J17_R_0	BJ986479
E_FL_BmN-_01J18_F_0	BJ980197
E_FL_BmN-_01J18_R_0	BJ986480
E_FL_BmN-_01J19_F_0	BJ980198
E_FL_BmN-_01J19_R_0	BJ986481
E_FL_BmN-_01J20_F_0	BJ980199
E_FL_BmN-_01J20_R_0	BJ986482
E_FL_BmN-_01J21_F_0	BJ980200
E_FL_BmN-_01J21_R_0	BJ986483
E_FL_BmN-_01J22_F_0	BJ980201
E_FL_BmN-_01J22_R_0	BJ986484
E_FL_BmN-_01J23_F_0	BJ980202
E_FL_BmN-_01J23_R_0	BJ986485
E_FL_BmN-_01J24_R_0	BJ986486
E_FL_BmN-_01K01_F_0	BJ980203
E_FL_BmN-_01K01_R_0	BJ986487
E_FL_BmN-_01K02_F_0	BJ980204
E_FL_BmN-_01K02_R_0	BJ986488
E_FL_BmN-_01K03_F_0	BJ980205
E_FL_BmN-_01K03_R_0	BJ986489
E_FL_BmN-_01K04_R_0	BJ986490
E_FL_BmN-_01K05_F_0	BJ980206
E_FL_BmN-_01K05_R_0	BJ986491
E_FL_BmN-_01K06_R_0	BJ986492
E_FL_BmN-_01K07_F_0	BJ980207
E_FL_BmN-_01K07_R_0	BJ986493

E_FL_BmN-_01K08_F_0	BJ980208
E_FL_BmN-_01K08_R_0	BJ986494
E_FL_BmN-_01K09_F_0	BJ980209
E_FL_BmN-_01K09_R_0	BJ986495
E_FL_BmN-_01K10_F_0	BJ980210
E_FL_BmN-_01K10_R_0	BJ986496
E_FL_BmN-_01K11_F_0	BJ980211
E_FL_BmN-_01K11_R_0	BJ986497
E_FL_BmN-_01K12_R_0	BJ986498
E_FL_BmN-_01K13_F_0	BJ980212
E_FL_BmN-_01K13_R_0	BJ986499
E_FL_BmN-_01K15_F_0	BJ980213
E_FL_BmN-_01K15_R_0	BJ986500
E_FL_BmN-_01K16_F_0	BJ980214
E_FL_BmN-_01K16_R_0	BJ986501
E_FL_BmN-_01K17_F_0	BJ980215
E_FL_BmN-_01K17_R_0	BJ986502
E_FL_BmN-_01K18_F_0	BJ980216
E_FL_BmN-_01K18_R_0	BJ986503
E_FL_BmN-_01K19_F_0	BJ980217
E_FL_BmN-_01K20_F_0	BJ980218
E_FL_BmN-_01K20_R_0	BJ986504
E_FL_BmN-_01K21_F_0	BJ980219
E_FL_BmN-_01K21_R_0	BJ986505
E_FL_BmN-_01K22_F_0	BJ980220
E_FL_BmN-_01K22_R_0	BJ986506
E_FL_BmN-_01K23_F_0	BJ980221
E_FL_BmN-_01K23_R_0	BJ986507
E_FL_BmN-_01K24_F_0	BJ980222
E_FL_BmN-_01K24_R_0	BJ986508
E_FL_BmN-_01L01_F_0	BJ980223
E_FL_BmN-_01L01_R_0	BJ986509
E_FL_BmN-_01L02_F_0	BJ980224
E_FL_BmN-_01L02_R_0	BJ986510
E_FL_BmN-_01L03_F_0	BJ980225
E_FL_BmN-_01L03_R_0	BJ986511
E_FL_BmN-_01L04_F_0	BJ980226
E_FL_BmN-_01L04_R_0	BJ986512
E_FL_BmN-_01L05_F_0	BJ980227
E_FL_BmN-_01L05_R_0	BJ986513
E_FL_BmN-_01L06_F_0	BJ980228
E_FL_BmN-_01L06_R_0	BJ986514
E_FL_BmN-_01L07_R_0	BJ986515
E_FL_BmN-_01L08_F_0	BJ980229
E_FL_BmN-_01L08_R_0	BJ986516
E_FL_BmN-_01L09_F_0	BJ980230
E_FL_BmN-_01L09_R_0	BJ986517
E_FL_BmN-_01L10_F_0	BJ980231
E_FL_BmN-_01L10_R_0	BJ986518
E_FL_BmN-_01L11_F_0	BJ980232
E_FL_BmN-_01L11_R_0	BJ986519
E_FL_BmN-_01L12_F_0	BJ980233
E_FL_BmN-_01L12_R_0	BJ986520
E_FL_BmN-_01L13_F_0	BJ980234

E_FL_BmN-_01L13_R_0	BJ986521
E_FL_BmN-_01L14_F_0	BJ980235
E_FL_BmN-_01L15_F_0	BJ980236
E_FL_BmN-_01L15_R_0	BJ986522
E_FL_BmN-_01L16_F_0	BJ980237
E_FL_BmN-_01L16_R_0	BJ986523
E_FL_BmN-_01L17_F_0	BJ980238
E_FL_BmN-_01L17_R_0	BJ986524
E_FL_BmN-_01L18_F_0	BJ980239
E_FL_BmN-_01L18_R_0	BJ986525
E_FL_BmN-_01L19_F_0	BJ980240
E_FL_BmN-_01L19_R_0	BJ986526
E_FL_BmN-_01L20_F_0	BJ980241
E_FL_BmN-_01L20_R_0	BJ986527
E_FL_BmN-_01L21_F_0	BJ980242
E_FL_BmN-_01L21_R_0	BJ986528
E_FL_BmN-_01L22_F_0	BJ980243
E_FL_BmN-_01L22_R_0	BJ986529
E_FL_BmN-_01L23_F_0	BJ980244
E_FL_BmN-_01L23_R_0	BJ986530
E_FL_BmN-_01L24_R_0	BJ986531
E_FL_BmN-_01M01_F_0	BJ980245
E_FL_BmN-_01M02_F_0	BJ980246
E_FL_BmN-_01M02_R_0	BJ986532
E_FL_BmN-_01M03_F_0	BJ980247
E_FL_BmN-_01M03_R_0	BJ986533
E_FL_BmN-_01M05_F_0	BJ980248
E_FL_BmN-_01M05_R_0	BJ986534
E_FL_BmN-_01M06_F_0	BJ980249
E_FL_BmN-_01M06_R_0	BJ986535
E_FL_BmN-_01M07_F_0	BJ980250
E_FL_BmN-_01M07_R_0	BJ986536
E_FL_BmN-_01M09_F_0	BJ980251
E_FL_BmN-_01M09_R_0	BJ986537
E_FL_BmN-_01M10_F_0	BJ980252
E_FL_BmN-_01M10_R_0	BJ986538
E_FL_BmN-_01M11_F_0	BJ980253
E_FL_BmN-_01M11_R_0	BJ986539
E_FL_BmN-_01M12_F_0	BJ980254
E_FL_BmN-_01M12_R_0	BJ986540
E_FL_BmN-_01M13_F_0	BJ980255
E_FL_BmN-_01M13_R_0	BJ986541
E_FL_BmN-_01M14_F_0	BJ980256
E_FL_BmN-_01M14_R_0	BJ986542
E_FL_BmN-_01M15_F_0	BJ980257
E_FL_BmN-_01M15_R_0	BJ986543
E_FL_BmN-_01M16_F_0	BJ980258
E_FL_BmN-_01M16_R_0	BJ986544
E_FL_BmN-_01M17_F_0	BJ980259
E_FL_BmN-_01M17_R_0	BJ986545
E_FL_BmN-_01M18_R_0	BJ986546
E_FL_BmN-_01M19_F_0	BJ980260
E_FL_BmN-_01M19_R_0	BJ986547
E_FL_BmN-_01M20_F_0	BJ980261

E_FL_BmN-_01M20_R_0	BJ986548
E_FL_BmN-_01M21_F_0	BJ980262
E_FL_BmN-_01M21_R_0	BJ986549
E_FL_BmN-_01M22_F_0	BJ980263
E_FL_BmN-_01M22_R_0	BJ986550
E_FL_BmN-_01M23_F_0	BJ980264
E_FL_BmN-_01M23_R_0	BJ986551
E_FL_BmN-_01M24_R_0	BJ986552
E_FL_BmN-_01N01_F_0	BJ980265
E_FL_BmN-_01N01_R_0	BJ986553
E_FL_BmN-_01N02_F_0	BJ980266
E_FL_BmN-_01N02_R_0	BJ986554
E_FL_BmN-_01N05_F_0	BJ980267
E_FL_BmN-_01N05_R_0	BJ986555
E_FL_BmN-_01N07_F_0	BJ980268
E_FL_BmN-_01N07_R_0	BJ986556
E_FL_BmN-_01N08_F_0	BJ980269
E_FL_BmN-_01N08_R_0	BJ986557
E_FL_BmN-_01N10_F_0	BJ980270
E_FL_BmN-_01N10_R_0	BJ986558
E_FL_BmN-_01N11_F_0	BJ980271
E_FL_BmN-_01N11_R_0	BJ986559
E_FL_BmN-_01N12_F_0	BJ980272
E_FL_BmN-_01N12_R_0	BJ986560
E_FL_BmN-_01N13_F_0	BJ980273
E_FL_BmN-_01N13_R_0	BJ986561
E_FL_BmN-_01N14_F_0	BJ980274
E_FL_BmN-_01N14_R_0	BJ986562
E_FL_BmN-_01N15_F_0	BJ980275
E_FL_BmN-_01N15_R_0	BJ986563
E_FL_BmN-_01N16_F_0	BJ980276
E_FL_BmN-_01N16_R_0	BJ986564
E_FL_BmN-_01N17_F_0	BJ980277
E_FL_BmN-_01N17_R_0	BJ986565
E_FL_BmN-_01N18_F_0	BJ980278
E_FL_BmN-_01N18_R_0	BJ986566
E_FL_BmN-_01N19_F_0	BJ980279
E_FL_BmN-_01N19_R_0	BJ986567
E_FL_BmN-_01N20_F_0	BJ980280
E_FL_BmN-_01N20_R_0	BJ986568
E_FL_BmN-_01N21_F_0	BJ980281
E_FL_BmN-_01N21_R_0	BJ986569
E_FL_BmN-_01N22_F_0	BJ980282
E_FL_BmN-_01N22_R_0	BJ986570
E_FL_BmN-_01N23_F_0	BJ980283
E_FL_BmN-_01N23_R_0	BJ986571
E_FL_BmN-_01N24_F_0	BJ980284
E_FL_BmN-_01N24_R_0	BJ986572
E_FL_BmN-_01O01_F_0	BJ980285
E_FL_BmN-_01O01_R_0	BJ986573
E_FL_BmN-_01O02_F_0	BJ980286
E_FL_BmN-_01O02_R_0	BJ986574
E_FL_BmN-_01O03_F_0	BJ980287
E_FL_BmN-_01O03_R_0	BJ986575

E_FL_BmN-_01O05_F_0	BJ980288
E_FL_BmN-_01O05_R_0	BJ986576
E_FL_BmN-_01O06_F_0	BJ980289
E_FL_BmN-_01O07_F_0	BJ980290
E_FL_BmN-_01O07_R_0	BJ986577
E_FL_BmN-_01O08_F_0	BJ980291
E_FL_BmN-_01O08_R_0	BJ986578
E_FL_BmN-_01O09_R_0	BJ986579
E_FL_BmN-_01O10_F_0	BJ980292
E_FL_BmN-_01O10_R_0	BJ986580
E_FL_BmN-_01O12_F_0	BJ980293
E_FL_BmN-_01O15_R_0	BJ986581
E_FL_BmN-_01O16_F_0	BJ980294
E_FL_BmN-_01O16_R_0	BJ986582
E_FL_BmN-_01O17_F_0	BJ980295
E_FL_BmN-_01O17_R_0	BJ986583
E_FL_BmN-_01O19_F_0	BJ980296
E_FL_BmN-_01O19_R_0	BJ986584
E_FL_BmN-_01O20_F_0	BJ980297
E_FL_BmN-_01O20_R_0	BJ986585
E_FL_BmN-_01O21_F_0	BJ980298
E_FL_BmN-_01O21_R_0	BJ986586
E_FL_BmN-_01O22_F_0	BJ980299
E_FL_BmN-_01O22_R_0	BJ986587
E_FL_BmN-_01O23_F_0	BJ980300
E_FL_BmN-_01O23_R_0	BJ986588
E_FL_BmN-_01O24_R_0	BJ986589
E_FL_BmN-_01P01_F_0	BJ980301
E_FL_BmN-_01P02_F_0	BJ980302
E_FL_BmN-_01P02_R_0	BJ986590
E_FL_BmN-_01P03_F_0	BJ980303
E_FL_BmN-_01P03_R_0	BJ986591
E_FL_BmN-_01P04_F_0	BJ980304
E_FL_BmN-_01P04_R_0	BJ986592
E_FL_BmN-_01P05_F_0	BJ980305
E_FL_BmN-_01P05_R_0	BJ986593
E_FL_BmN-_01P06_F_0	BJ980306
E_FL_BmN-_01P06_R_0	BJ986594
E_FL_BmN-_01P07_F_0	BJ980307
E_FL_BmN-_01P07_R_0	BJ986595
E_FL_BmN-_01P08_F_0	BJ980308
E_FL_BmN-_01P08_R_0	BJ986596
E_FL_BmN-_01P09_F_0	BJ980309
E_FL_BmN-_01P09_R_0	BJ986597
E_FL_BmN-_01P10_F_0	BJ980310
E_FL_BmN-_01P10_R_0	BJ986598
E_FL_BmN-_01P12_F_0	BJ980311
E_FL_BmN-_01P12_R_0	BJ986599
E_FL_BmN-_01P13_F_0	BJ980312
E_FL_BmN-_01P13_R_0	BJ986600
E_FL_BmN-_01P14_F_0	BJ980313
E_FL_BmN-_01P14_R_0	BJ986601
E_FL_BmN-_01P15_F_0	BJ980314
E_FL_BmN-_01P15_R_0	BJ986602

E_FL_BmN-_01P16_F_0	BJ980315
E_FL_BmN-_01P16_R_0	BJ986603
E_FL_BmN-_01P17_F_0	BJ980316
E_FL_BmN-_01P17_R_0	BJ986604
E_FL_BmN-_01P18_F_0	BJ980317
E_FL_BmN-_01P18_R_0	BJ986605
E_FL_BmN-_01P19_F_0	BJ980318
E_FL_BmN-_01P19_R_0	BJ986606
E_FL_BmN-_01P20_F_0	BJ980319
E_FL_BmN-_01P20_R_0	BJ986607
E_FL_BmN-_01P21_F_0	BJ980320
E_FL_BmN-_01P21_R_0	BJ986608
E_FL_BmN-_01P22_F_0	BJ980321
E_FL_BmN-_01P22_R_0	BJ986609
E_FL_BmN-_01P23_R_0	BJ986610
E_FL_BmN-_01P24_F_0	BJ980322
E_FL_BmN-_01P24_R_0	BJ986611
E_FL_BmN-_02A01_F_0	BJ980323
E_FL_BmN-_02A01_R_0	BJ986612
E_FL_BmN-_02A02_F_0	BJ980324
E_FL_BmN-_02A02_R_0	BJ986613
E_FL_BmN-_02A03_F_0	BJ980325
E_FL_BmN-_02A03_R_0	BJ986614
E_FL_BmN-_02A04_F_0	BJ980326
E_FL_BmN-_02A04_R_0	BJ986615
E_FL_BmN-_02A05_F_0	BJ980327
E_FL_BmN-_02A05_R_0	BJ986616
E_FL_BmN-_02A06_F_0	BJ980328
E_FL_BmN-_02A06_R_0	BJ986617
E_FL_BmN-_02A07_F_0	BJ980329
E_FL_BmN-_02A07_R_0	BJ986618
E_FL_BmN-_02A08_F_0	BJ980330
E_FL_BmN-_02A08_R_0	BJ986619
E_FL_BmN-_02A09_R_0	BJ986620
E_FL_BmN-_02A10_F_0	BJ980331
E_FL_BmN-_02A10_R_0	BJ986621
E_FL_BmN-_02A12_F_0	BJ980332
E_FL_BmN-_02A12_R_0	BJ986622
E_FL_BmN-_02A13_F_0	BJ980333
E_FL_BmN-_02A13_R_0	BJ986623
E_FL_BmN-_02A14_F_0	BJ980334
E_FL_BmN-_02A14_R_0	BJ986624
E_FL_BmN-_02A15_F_0	BJ980335
E_FL_BmN-_02A15_R_0	BJ986625
E_FL_BmN-_02A16_F_0	BJ980336
E_FL_BmN-_02A16_R_0	BJ986626
E_FL_BmN-_02A18_F_0	BJ980337
E_FL_BmN-_02A18_R_0	BJ986627
E_FL_BmN-_02A19_F_0	BJ980338
E_FL_BmN-_02A19_R_0	BJ986628
E_FL_BmN-_02A20_F_0	BJ980339
E_FL_BmN-_02A20_R_0	BJ986629
E_FL_BmN-_02A21_F_0	BJ980340
E_FL_BmN-_02A21_R_0	BJ986630

E_FL_BmN-_02A22_F_0	BJ980341
E_FL_BmN-_02A22_R_0	BJ986631
E_FL_BmN-_02A23_F_0	BJ980342
E_FL_BmN-_02A23_R_0	BJ986632
E_FL_BmN-_02A24_F_0	BJ980343
E_FL_BmN-_02A24_R_0	BJ986633
E_FL_BmN-_02B01_R_0	BJ986634
E_FL_BmN-_02B02_F_0	BJ980344
E_FL_BmN-_02B02_R_0	BJ986635
E_FL_BmN-_02B04_F_0	BJ980345
E_FL_BmN-_02B04_R_0	BJ986636
E_FL_BmN-_02B05_F_0	BJ980346
E_FL_BmN-_02B05_R_0	BJ986637
E_FL_BmN-_02B06_R_0	BJ986638
E_FL_BmN-_02B07_F_0	BJ980347
E_FL_BmN-_02B07_R_0	BJ986639
E_FL_BmN-_02B08_F_0	BJ980348
E_FL_BmN-_02B08_R_0	BJ986640
E_FL_BmN-_02B09_F_0	BJ980349
E_FL_BmN-_02B09_R_0	BJ986641
E_FL_BmN-_02B10_F_0	BJ980350
E_FL_BmN-_02B11_F_0	BJ980351
E_FL_BmN-_02B11_R_0	BJ986642
E_FL_BmN-_02B12_F_0	BJ980352
E_FL_BmN-_02B12_R_0	BJ986643
E_FL_BmN-_02B13_F_0	BJ980353
E_FL_BmN-_02B13_R_0	BJ986644
E_FL_BmN-_02B14_F_0	BJ980354
E_FL_BmN-_02B14_R_0	BJ986645
E_FL_BmN-_02B15_F_0	BJ980355
E_FL_BmN-_02B15_R_0	BJ986646
E_FL_BmN-_02B16_F_0	BJ980356
E_FL_BmN-_02B16_R_0	BJ986647
E_FL_BmN-_02B17_F_0	BJ980357
E_FL_BmN-_02B17_R_0	BJ986648
E_FL_BmN-_02B18_F_0	BJ980358
E_FL_BmN-_02B18_R_0	BJ986649
E_FL_BmN-_02B19_R_0	BJ986650
E_FL_BmN-_02B20_F_0	BJ980359
E_FL_BmN-_02B20_R_0	BJ986651
E_FL_BmN-_02B21_F_0	BJ980360
E_FL_BmN-_02B21_R_0	BJ986652
E_FL_BmN-_02B22_F_0	BJ980361
E_FL_BmN-_02B22_R_0	BJ986653
E_FL_BmN-_02B23_F_0	BJ980362
E_FL_BmN-_02B23_R_0	BJ986654
E_FL_BmN-_02B24_F_0	BJ980363
E_FL_BmN-_02B24_R_0	BJ986655
E_FL_BmN-_02C01_F_0	BJ980364
E_FL_BmN-_02C01_R_0	BJ986656
E_FL_BmN-_02C02_F_0	BJ980365
E_FL_BmN-_02C02_R_0	BJ986657
E_FL_BmN-_02C03_F_0	BJ980366
E_FL_BmN-_02C03_R_0	BJ986658

E_FL_BmN-_02C04_F_0	BJ980367
E_FL_BmN-_02C04_R_0	BJ986659
E_FL_BmN-_02C06_F_0	BJ980368
E_FL_BmN-_02C06_R_0	BJ986660
E_FL_BmN-_02C07_F_0	BJ980369
E_FL_BmN-_02C07_R_0	BJ986661
E_FL_BmN-_02C08_F_0	BJ980370
E_FL_BmN-_02C08_R_0	BJ986662
E_FL_BmN-_02C10_F_0	BJ980371
E_FL_BmN-_02C10_R_0	BJ986663
E_FL_BmN-_02C11_F_0	BJ980372
E_FL_BmN-_02C11_R_0	BJ986664
E_FL_BmN-_02C12_R_0	BJ986665
E_FL_BmN-_02C13_F_0	BJ980373
E_FL_BmN-_02C13_R_0	BJ986666
E_FL_BmN-_02C14_R_0	BJ986667
E_FL_BmN-_02C15_F_0	BJ980374
E_FL_BmN-_02C15_R_0	BJ986668
E_FL_BmN-_02C17_F_0	BJ980375
E_FL_BmN-_02C18_F_0	BJ980376
E_FL_BmN-_02C18_R_0	BJ986669
E_FL_BmN-_02C19_F_0	BJ980377
E_FL_BmN-_02C20_F_0	BJ980378
E_FL_BmN-_02C20_R_0	BJ986670
E_FL_BmN-_02C21_F_0	BJ980379
E_FL_BmN-_02C21_R_0	BJ986671
E_FL_BmN-_02C22_F_0	BJ980380
E_FL_BmN-_02C22_R_0	BJ986672
E_FL_BmN-_02C23_F_0	BJ980381
E_FL_BmN-_02C23_R_0	BJ986673
E_FL_BmN-_02D01_F_0	BJ980382
E_FL_BmN-_02D02_F_0	BJ980383
E_FL_BmN-_02D02_R_0	BJ986674
E_FL_BmN-_02D03_F_0	BJ980384
E_FL_BmN-_02D03_R_0	BJ986675
E_FL_BmN-_02D04_F_0	BJ980385
E_FL_BmN-_02D04_R_0	BJ986676
E_FL_BmN-_02D05_F_0	BJ980386
E_FL_BmN-_02D05_R_0	BJ986677
E_FL_BmN-_02D06_F_0	BJ980387
E_FL_BmN-_02D06_R_0	BJ986678
E_FL_BmN-_02D07_F_0	BJ980388
E_FL_BmN-_02D07_R_0	BJ986679
E_FL_BmN-_02D08_F_0	BJ980389
E_FL_BmN-_02D08_R_0	BJ986680
E_FL_BmN-_02D09_F_0	BJ980390
E_FL_BmN-_02D09_R_0	BJ986681
E_FL_BmN-_02D10_F_0	BJ980391
E_FL_BmN-_02D10_R_0	BJ986682
E_FL_BmN-_02D11_F_0	BJ980392
E_FL_BmN-_02D11_R_0	BJ986683
E_FL_BmN-_02D12_F_0	BJ980393
E_FL_BmN-_02D12_R_0	BJ986684
E_FL_BmN-_02D13_F_0	BJ980394

E_FL_BmN-_02D13_R_0	BJ986685
E_FL_BmN-_02D14_F_0	BJ980395
E_FL_BmN-_02D14_R_0	BJ986686
E_FL_BmN-_02D15_F_0	BJ980396
E_FL_BmN-_02D15_R_0	BJ986687
E_FL_BmN-_02D16_F_0	BJ980397
E_FL_BmN-_02D16_R_0	BJ986688
E_FL_BmN-_02D17_F_0	BJ980398
E_FL_BmN-_02D17_R_0	BJ986689
E_FL_BmN-_02D18_R_0	BJ986690
E_FL_BmN-_02D19_F_0	BJ980399
E_FL_BmN-_02D19_R_0	BJ986691
E_FL_BmN-_02D20_F_0	BJ980400
E_FL_BmN-_02D20_R_0	BJ986692
E_FL_BmN-_02D21_F_0	BJ980401
E_FL_BmN-_02D21_R_0	BJ986693
E_FL_BmN-_02D22_F_0	BJ980402
E_FL_BmN-_02D22_R_0	BJ986694
E_FL_BmN-_02D23_F_0	BJ980403
E_FL_BmN-_02D23_R_0	BJ986695
E_FL_BmN-_02D24_R_0	BJ986696
E_FL_BmN-_02E01_F_0	BJ980404
E_FL_BmN-_02E02_F_0	BJ980405
E_FL_BmN-_02E02_R_0	BJ986697
E_FL_BmN-_02E03_F_0	BJ980406
E_FL_BmN-_02E03_R_0	BJ986698
E_FL_BmN-_02E04_F_0	BJ980407
E_FL_BmN-_02E04_R_0	BJ986699
E_FL_BmN-_02E06_R_0	BJ986700
E_FL_BmN-_02E07_F_0	BJ980408
E_FL_BmN-_02E08_F_0	BJ980409
E_FL_BmN-_02E08_R_0	BJ986701
E_FL_BmN-_02E09_F_0	BJ980410
E_FL_BmN-_02E09_R_0	BJ986702
E_FL_BmN-_02E10_F_0	BJ980411
E_FL_BmN-_02E10_R_0	BJ986703
E_FL_BmN-_02E11_F_0	BJ980412
E_FL_BmN-_02E11_R_0	BJ986704
E_FL_BmN-_02E12_F_0	BJ980413
E_FL_BmN-_02E12_R_0	BJ986705
E_FL_BmN-_02E13_F_0	BJ980414
E_FL_BmN-_02E13_R_0	BJ986706
E_FL_BmN-_02E14_F_0	BJ980415
E_FL_BmN-_02E14_R_0	BJ986707
E_FL_BmN-_02E15_F_0	BJ980416
E_FL_BmN-_02E15_R_0	BJ986708
E_FL_BmN-_02E17_F_0	BJ980417
E_FL_BmN-_02E17_R_0	BJ986709
E_FL_BmN-_02E18_F_0	BJ980418
E_FL_BmN-_02E18_R_0	BJ986710
E_FL_BmN-_02E19_F_0	BJ980419
E_FL_BmN-_02E20_F_0	BJ980420
E_FL_BmN-_02E20_R_0	BJ986711
E_FL_BmN-_02E21_F_0	BJ980421

E_FL_BmN-_02E21_R_0	BJ986712
E_FL_BmN-_02E22_F_0	BJ980422
E_FL_BmN-_02E22_R_0	BJ986713
E_FL_BmN-_02E23_F_0	BJ980423
E_FL_BmN-_02E23_R_0	BJ986714
E_FL_BmN-_02E24_F_0	BJ980424
E_FL_BmN-_02E24_R_0	BJ986715
E_FL_BmN-_02F01_R_0	BJ986716
E_FL_BmN-_02F02_R_0	BJ986717
E_FL_BmN-_02F03_F_0	BJ980425
E_FL_BmN-_02F03_R_0	BJ986718
E_FL_BmN-_02F04_F_0	BJ980426
E_FL_BmN-_02F04_R_0	BJ986719
E_FL_BmN-_02F05_F_0	BJ980427
E_FL_BmN-_02F05_R_0	BJ986720
E_FL_BmN-_02F06_F_0	BJ980428
E_FL_BmN-_02F06_R_0	BJ986721
E_FL_BmN-_02F07_F_0	BJ980429
E_FL_BmN-_02F07_R_0	BJ986722
E_FL_BmN-_02F08_F_0	BJ980430
E_FL_BmN-_02F08_R_0	BJ986723
E_FL_BmN-_02F09_F_0	BJ980431
E_FL_BmN-_02F09_R_0	BJ986724
E_FL_BmN-_02F10_F_0	BJ980432
E_FL_BmN-_02F10_R_0	BJ986725
E_FL_BmN-_02F11_F_0	BJ980433
E_FL_BmN-_02F11_R_0	BJ986726
E_FL_BmN-_02F12_F_0	BJ980434
E_FL_BmN-_02F12_R_0	BJ986727
E_FL_BmN-_02F13_F_0	BJ980435
E_FL_BmN-_02F14_F_0	BJ980436
E_FL_BmN-_02F14_R_0	BJ986728
E_FL_BmN-_02F15_F_0	BJ980437
E_FL_BmN-_02F15_R_0	BJ986729
E_FL_BmN-_02F16_F_0	BJ980438
E_FL_BmN-_02F16_R_0	BJ986730
E_FL_BmN-_02F17_F_0	BJ980439
E_FL_BmN-_02F17_R_0	BJ986731
E_FL_BmN-_02F18_F_0	BJ980440
E_FL_BmN-_02F18_R_0	BJ986732
E_FL_BmN-_02F19_F_0	BJ980441
E_FL_BmN-_02F19_R_0	BJ986733
E_FL_BmN-_02F20_F_0	BJ980442
E_FL_BmN-_02F20_R_0	BJ986734
E_FL_BmN-_02F21_F_0	BJ980443
E_FL_BmN-_02F22_F_0	BJ980444
E_FL_BmN-_02F22_R_0	BJ986735
E_FL_BmN-_02F23_F_0	BJ980445
E_FL_BmN-_02F23_R_0	BJ986736
E_FL_BmN-_02F24_F_0	BJ980446
E_FL_BmN-_02F24_R_0	BJ986737
E_FL_BmN-_02G02_F_0	BJ980447
E_FL_BmN-_02G02_R_0	BJ986738
E_FL_BmN-_02G03_F_0	BJ980448

E_FL_BmN-_02G03_R_0	BJ986739
E_FL_BmN-_02G04_F_0	BJ980449
E_FL_BmN-_02G04_R_0	BJ986740
E_FL_BmN-_02G05_F_0	BJ980450
E_FL_BmN-_02G05_R_0	BJ986741
E_FL_BmN-_02G06_F_0	BJ980451
E_FL_BmN-_02G06_R_0	BJ986742
E_FL_BmN-_02G07_F_0	BJ980452
E_FL_BmN-_02G07_R_0	BJ986743
E_FL_BmN-_02G08_F_0	BJ980453
E_FL_BmN-_02G08_R_0	BJ986744
E_FL_BmN-_02G09_F_0	BJ980454
E_FL_BmN-_02G09_R_0	BJ986745
E_FL_BmN-_02G10_F_0	BJ980455
E_FL_BmN-_02G10_R_0	BJ986746
E_FL_BmN-_02G11_F_0	BJ980456
E_FL_BmN-_02G11_R_0	BJ986747
E_FL_BmN-_02G12_F_0	BJ980457
E_FL_BmN-_02G12_R_0	BJ986748
E_FL_BmN-_02G13_F_0	BJ980458
E_FL_BmN-_02G13_R_0	BJ986749
E_FL_BmN-_02G14_F_0	BJ980459
E_FL_BmN-_02G14_R_0	BJ986750
E_FL_BmN-_02G15_F_0	BJ980460
E_FL_BmN-_02G15_R_0	BJ986751
E_FL_BmN-_02G16_F_0	BJ980461
E_FL_BmN-_02G16_R_0	BJ986752
E_FL_BmN-_02G17_F_0	BJ980462
E_FL_BmN-_02G17_R_0	BJ986753
E_FL_BmN-_02G18_F_0	BJ980463
E_FL_BmN-_02G18_R_0	BJ986754
E_FL_BmN-_02G19_R_0	BJ986755
E_FL_BmN-_02G20_F_0	BJ980464
E_FL_BmN-_02G20_R_0	BJ986756
E_FL_BmN-_02G21_F_0	BJ980465
E_FL_BmN-_02G21_R_0	BJ986757
E_FL_BmN-_02G22_F_0	BJ980466
E_FL_BmN-_02G22_R_0	BJ986758
E_FL_BmN-_02G23_F_0	BJ980467
E_FL_BmN-_02G23_R_0	BJ986759
E_FL_BmN-_02G24_R_0	BJ986760
E_FL_BmN-_02H01_F_0	BJ980468
E_FL_BmN-_02H01_R_0	BJ986761
E_FL_BmN-_02H02_F_0	BJ980469
E_FL_BmN-_02H02_R_0	BJ986762
E_FL_BmN-_02H03_F_0	BJ980470
E_FL_BmN-_02H03_R_0	BJ986763
E_FL_BmN-_02H04_F_0	BJ980471
E_FL_BmN-_02H04_R_0	BJ986764
E_FL_BmN-_02H06_F_0	BJ980472
E_FL_BmN-_02H06_R_0	BJ986765
E_FL_BmN-_02H07_F_0	BJ980473
E_FL_BmN-_02H07_R_0	BJ986766
E_FL_BmN-_02H08_F_0	BJ980474

E_FL_BmN-_02H08_R_0	BJ986767
E_FL_BmN-_02H09_F_0	BJ980475
E_FL_BmN-_02H09_R_0	BJ986768
E_FL_BmN-_02H10_F_0	BJ980476
E_FL_BmN-_02H10_R_0	BJ986769
E_FL_BmN-_02H11_F_0	BJ980477
E_FL_BmN-_02H11_R_0	BJ986770
E_FL_BmN-_02H12_F_0	BJ980478
E_FL_BmN-_02H12_R_0	BJ986771
E_FL_BmN-_02H13_F_0	BJ980479
E_FL_BmN-_02H13_R_0	BJ986772
E_FL_BmN-_02H14_F_0	BJ980480
E_FL_BmN-_02H14_R_0	BJ986773
E_FL_BmN-_02H15_F_0	BJ980481
E_FL_BmN-_02H15_R_0	BJ986774
E_FL_BmN-_02H16_F_0	BJ980482
E_FL_BmN-_02H16_R_0	BJ986775
E_FL_BmN-_02H17_F_0	BJ980483
E_FL_BmN-_02H17_R_0	BJ986776
E_FL_BmN-_02H18_R_0	BJ986777
E_FL_BmN-_02H19_R_0	BJ986778
E_FL_BmN-_02H20_F_0	BJ980484
E_FL_BmN-_02H20_R_0	BJ986779
E_FL_BmN-_02H21_F_0	BJ980485
E_FL_BmN-_02H22_F_0	BJ980486
E_FL_BmN-_02H23_F_0	BJ980487
E_FL_BmN-_02H23_R_0	BJ986780
E_FL_BmN-_02H24_F_0	BJ980488
E_FL_BmN-_02H24_R_0	BJ986781
E_FL_BmN-_02I01_F_0	BJ980489
E_FL_BmN-_02I01_R_0	BJ986782
E_FL_BmN-_02I03_F_0	BJ980490
E_FL_BmN-_02I03_R_0	BJ986783
E_FL_BmN-_02I05_F_0	BJ980491
E_FL_BmN-_02I05_R_0	BJ986784
E_FL_BmN-_02I07_F_0	BJ980492
E_FL_BmN-_02I07_R_0	BJ986785
E_FL_BmN-_02I08_F_0	BJ980493
E_FL_BmN-_02I08_R_0	BJ986786
E_FL_BmN-_02I09_F_0	BJ980494
E_FL_BmN-_02I09_R_0	BJ986787
E_FL_BmN-_02I10_F_0	BJ980495
E_FL_BmN-_02I10_R_0	BJ986788
E_FL_BmN-_02I11_F_0	BJ980496
E_FL_BmN-_02I11_R_0	BJ986789
E_FL_BmN-_02I12_F_0	BJ980497
E_FL_BmN-_02I12_R_0	BJ986790
E_FL_BmN-_02I13_F_0	BJ980498
E_FL_BmN-_02I13_R_0	BJ986791
E_FL_BmN-_02I14_F_0	BJ980499
E_FL_BmN-_02I15_F_0	BJ980500
E_FL_BmN-_02I15_R_0	BJ986792
E_FL_BmN-_02I16_F_0	BJ980501
E_FL_BmN-_02I17_F_0	BJ980502

E_FL_BmN-_02I17_R_0	BJ986793
E_FL_BmN-_02I18_F_0	BJ980503
E_FL_BmN-_02I18_R_0	BJ986794
E_FL_BmN-_02I20_F_0	BJ980504
E_FL_BmN-_02I20_R_0	BJ986795
E_FL_BmN-_02I21_F_0	BJ980505
E_FL_BmN-_02I21_R_0	BJ986796
E_FL_BmN-_02I22_F_0	BJ980506
E_FL_BmN-_02I22_R_0	BJ986797
E_FL_BmN-_02I24_R_0	BJ986798
E_FL_BmN-_02J01_F_0	BJ980507
E_FL_BmN-_02J01_R_0	BJ986799
E_FL_BmN-_02J02_F_0	BJ980508
E_FL_BmN-_02J02_R_0	BJ986800
E_FL_BmN-_02J03_F_0	BJ980509
E_FL_BmN-_02J03_R_0	BJ986801
E_FL_BmN-_02J04_F_0	BJ980510
E_FL_BmN-_02J04_R_0	BJ986802
E_FL_BmN-_02J05_F_0	BJ980511
E_FL_BmN-_02J05_R_0	BJ986803
E_FL_BmN-_02J06_F_0	BJ980512
E_FL_BmN-_02J06_R_0	BJ986804
E_FL_BmN-_02J07_F_0	BJ980513
E_FL_BmN-_02J07_R_0	BJ986805
E_FL_BmN-_02J08_F_0	BJ980514
E_FL_BmN-_02J08_R_0	BJ986806
E_FL_BmN-_02J09_F_0	BJ980515
E_FL_BmN-_02J10_F_0	BJ980516
E_FL_BmN-_02J10_R_0	BJ986807
E_FL_BmN-_02J11_F_0	BJ980517
E_FL_BmN-_02J11_R_0	BJ986808
E_FL_BmN-_02J12_R_0	BJ986809
E_FL_BmN-_02J13_F_0	BJ980518
E_FL_BmN-_02J13_R_0	BJ986810
E_FL_BmN-_02J14_F_0	BJ980519
E_FL_BmN-_02J14_R_0	BJ986811
E_FL_BmN-_02J15_F_0	BJ980520
E_FL_BmN-_02J15_R_0	BJ986812
E_FL_BmN-_02J16_F_0	BJ980521
E_FL_BmN-_02J16_R_0	BJ986813
E_FL_BmN-_02J17_F_0	BJ980522
E_FL_BmN-_02J17_R_0	BJ986814
E_FL_BmN-_02J18_R_0	BJ986815
E_FL_BmN-_02J19_F_0	BJ980523
E_FL_BmN-_02J19_R_0	BJ986816
E_FL_BmN-_02J20_F_0	BJ980524
E_FL_BmN-_02J20_R_0	BJ986817
E_FL_BmN-_02J21_F_0	BJ980525
E_FL_BmN-_02J21_R_0	BJ986818
E_FL_BmN-_02J22_F_0	BJ980526
E_FL_BmN-_02J22_R_0	BJ986819
E_FL_BmN-_02J23_F_0	BJ980527
E_FL_BmN-_02J23_R_0	BJ986820
E_FL_BmN-_02J24_R_0	BJ986821

E_FL_BmN-_02K01_F_0	BJ980528
E_FL_BmN-_02K02_F_0	BJ980529
E_FL_BmN-_02K02_R_0	BJ986822
E_FL_BmN-_02K03_F_0	BJ980530
E_FL_BmN-_02K03_R_0	BJ986823
E_FL_BmN-_02K04_F_0	BJ980531
E_FL_BmN-_02K04_R_0	BJ986824
E_FL_BmN-_02K05_F_0	BJ980532
E_FL_BmN-_02K05_R_0	BJ986825
E_FL_BmN-_02K06_F_0	BJ980533
E_FL_BmN-_02K06_R_0	BJ986826
E_FL_BmN-_02K07_F_0	BJ980534
E_FL_BmN-_02K07_R_0	BJ986827
E_FL_BmN-_02K08_F_0	BJ980535
E_FL_BmN-_02K08_R_0	BJ986828
E_FL_BmN-_02K09_F_0	BJ980536
E_FL_BmN-_02K09_R_0	BJ986829
E_FL_BmN-_02K10_F_0	BJ980537
E_FL_BmN-_02K10_R_0	BJ986830
E_FL_BmN-_02K11_F_0	BJ980538
E_FL_BmN-_02K11_R_0	BJ986831
E_FL_BmN-_02K12_F_0	BJ980539
E_FL_BmN-_02K12_R_0	BJ986832
E_FL_BmN-_02K13_F_0	BJ980540
E_FL_BmN-_02K13_R_0	BJ986833
E_FL_BmN-_02K14_F_0	BJ980541
E_FL_BmN-_02K14_R_0	BJ986834
E_FL_BmN-_02K15_F_0	BJ980542
E_FL_BmN-_02K15_R_0	BJ986835
E_FL_BmN-_02K16_F_0	BJ980543
E_FL_BmN-_02K16_R_0	BJ986836
E_FL_BmN-_02K17_F_0	BJ980544
E_FL_BmN-_02K17_R_0	BJ986837
E_FL_BmN-_02K18_F_0	BJ980545
E_FL_BmN-_02K18_R_0	BJ986838
E_FL_BmN-_02K19_F_0	BJ980546
E_FL_BmN-_02K19_R_0	BJ986839
E_FL_BmN-_02K20_F_0	BJ980547
E_FL_BmN-_02K20_R_0	BJ986840
E_FL_BmN-_02K21_F_0	BJ980548
E_FL_BmN-_02K21_R_0	BJ986841
E_FL_BmN-_02K22_R_0	BJ986842
E_FL_BmN-_02K23_F_0	BJ980549
E_FL_BmN-_02K23_R_0	BJ986843
E_FL_BmN-_02K24_F_0	BJ980550
E_FL_BmN-_02K24_R_0	BJ986844
E_FL_BmN-_02L01_R_0	BJ986845
E_FL_BmN-_02L02_F_0	BJ980551
E_FL_BmN-_02L02_R_0	BJ986846
E_FL_BmN-_02L03_F_0	BJ980552
E_FL_BmN-_02L03_R_0	BJ986847
E_FL_BmN-_02L04_F_0	BJ980553
E_FL_BmN-_02L04_R_0	BJ986848
E_FL_BmN-_02L05_F_0	BJ980554

E_FL_BmN-_02L05_R_0	BJ986849
E_FL_BmN-_02L06_F_0	BJ980555
E_FL_BmN-_02L06_R_0	BJ986850
E_FL_BmN-_02L07_F_0	BJ980556
E_FL_BmN-_02L07_R_0	BJ986851
E_FL_BmN-_02L08_F_0	BJ980557
E_FL_BmN-_02L08_R_0	BJ986852
E_FL_BmN-_02L09_F_0	BJ980558
E_FL_BmN-_02L09_R_0	BJ986853
E_FL_BmN-_02L11_F_0	BJ980559
E_FL_BmN-_02L11_R_0	BJ986854
E_FL_BmN-_02L12_R_0	BJ986855
E_FL_BmN-_02L13_F_0	BJ980560
E_FL_BmN-_02L13_R_0	BJ986856
E_FL_BmN-_02L14_F_0	BJ980561
E_FL_BmN-_02L14_R_0	BJ986857
E_FL_BmN-_02L15_F_0	BJ980562
E_FL_BmN-_02L15_R_0	BJ986858
E_FL_BmN-_02L16_F_0	BJ980563
E_FL_BmN-_02L16_R_0	BJ986859
E_FL_BmN-_02L17_F_0	BJ980564
E_FL_BmN-_02L17_R_0	BJ986860
E_FL_BmN-_02L18_F_0	BJ980565
E_FL_BmN-_02L18_R_0	BJ986861
E_FL_BmN-_02L19_F_0	BJ980566
E_FL_BmN-_02L19_R_0	BJ986862
E_FL_BmN-_02L20_F_0	BJ980567
E_FL_BmN-_02L20_R_0	BJ986863
E_FL_BmN-_02L21_F_0	BJ980568
E_FL_BmN-_02L21_R_0	BJ986864
E_FL_BmN-_02L22_F_0	BJ980569
E_FL_BmN-_02L22_R_0	BJ986865
E_FL_BmN-_02L23_F_0	BJ980570
E_FL_BmN-_02L23_R_0	BJ986866
E_FL_BmN-_02L24_F_0	BJ980571
E_FL_BmN-_02L24_R_0	BJ986867
E_FL_BmN-_02M01_F_0	BJ980572
E_FL_BmN-_02M01_R_0	BJ986868
E_FL_BmN-_02M02_F_0	BJ980573
E_FL_BmN-_02M02_R_0	BJ986869
E_FL_BmN-_02M03_R_0	BJ986870
E_FL_BmN-_02M04_F_0	BJ980574
E_FL_BmN-_02M04_R_0	BJ986871
E_FL_BmN-_02M05_F_0	BJ980575
E_FL_BmN-_02M05_R_0	BJ986872
E_FL_BmN-_02M06_R_0	BJ986873
E_FL_BmN-_02M07_F_0	BJ980576
E_FL_BmN-_02M08_F_0	BJ980577
E_FL_BmN-_02M08_R_0	BJ986874
E_FL_BmN-_02M10_F_0	BJ980578
E_FL_BmN-_02M10_R_0	BJ986875
E_FL_BmN-_02M11_F_0	BJ980579
E_FL_BmN-_02M12_F_0	BJ980580
E_FL_BmN-_02M12_R_0	BJ986876

E_FL_BmN-_02M13_F_0	BJ980581
E_FL_BmN-_02M13_R_0	BJ986877
E_FL_BmN-_02M14_F_0	BJ980582
E_FL_BmN-_02M14_R_0	BJ986878
E_FL_BmN-_02M15_F_0	BJ980583
E_FL_BmN-_02M15_R_0	BJ986879
E_FL_BmN-_02M16_F_0	BJ980584
E_FL_BmN-_02M16_R_0	BJ986880
E_FL_BmN-_02M17_F_0	BJ980585
E_FL_BmN-_02M17_R_0	BJ986881
E_FL_BmN-_02M18_R_0	BJ986882
E_FL_BmN-_02M19_F_0	BJ980586
E_FL_BmN-_02M19_R_0	BJ986883
E_FL_BmN-_02M20_F_0	BJ980587
E_FL_BmN-_02M20_R_0	BJ986884
E_FL_BmN-_02M21_F_0	BJ980588
E_FL_BmN-_02M21_R_0	BJ986885
E_FL_BmN-_02M22_F_0	BJ980589
E_FL_BmN-_02M22_R_0	BJ986886
E_FL_BmN-_02M23_F_0	BJ980590
E_FL_BmN-_02M23_R_0	BJ986887
E_FL_BmN-_02M24_F_0	BJ980591
E_FL_BmN-_02M24_R_0	BJ986888
E_FL_BmN-_02N01_F_0	BJ980592
E_FL_BmN-_02N01_R_0	BJ986889
E_FL_BmN-_02N03_R_0	BJ986890
E_FL_BmN-_02N04_F_0	BJ980593
E_FL_BmN-_02N04_R_0	BJ986891
E_FL_BmN-_02N05_F_0	BJ980594
E_FL_BmN-_02N05_R_0	BJ986892
E_FL_BmN-_02N06_F_0	BJ980595
E_FL_BmN-_02N06_R_0	BJ986893
E_FL_BmN-_02N07_F_0	BJ980596
E_FL_BmN-_02N07_R_0	BJ986894
E_FL_BmN-_02N08_R_0	BJ986895
E_FL_BmN-_02N09_F_0	BJ980597
E_FL_BmN-_02N10_F_0	BJ980598
E_FL_BmN-_02N10_R_0	BJ986896
E_FL_BmN-_02N11_F_0	BJ980599
E_FL_BmN-_02N11_R_0	BJ986897
E_FL_BmN-_02N13_F_0	BJ980600
E_FL_BmN-_02N13_R_0	BJ986898
E_FL_BmN-_02N14_F_0	BJ980601
E_FL_BmN-_02N14_R_0	BJ986899
E_FL_BmN-_02N15_F_0	BJ980602
E_FL_BmN-_02N15_R_0	BJ986900
E_FL_BmN-_02N16_F_0	BJ980603
E_FL_BmN-_02N16_R_0	BJ986901
E_FL_BmN-_02N17_F_0	BJ980604
E_FL_BmN-_02N17_R_0	BJ986902
E_FL_BmN-_02N18_R_0	BJ986903
E_FL_BmN-_02N19_F_0	BJ980605
E_FL_BmN-_02N19_R_0	BJ986904
E_FL_BmN-_02N20_F_0	BJ980606

E_FL_BmN-_02N20_R_0	BJ986905
E_FL_BmN-_02N21_F_0	BJ980607
E_FL_BmN-_02N21_R_0	BJ986906
E_FL_BmN-_02N22_F_0	BJ980608
E_FL_BmN-_02N22_R_0	BJ986907
E_FL_BmN-_02N23_F_0	BJ980609
E_FL_BmN-_02N23_R_0	BJ986908
E_FL_BmN-_02N24_R_0	BJ986909
E_FL_BmN-_02O01_F_0	BJ980610
E_FL_BmN-_02O01_R_0	BJ986910
E_FL_BmN-_02O02_F_0	BJ980611
E_FL_BmN-_02O02_R_0	BJ986911
E_FL_BmN-_02O03_F_0	BJ980612
E_FL_BmN-_02O03_R_0	BJ986912
E_FL_BmN-_02O04_F_0	BJ980613
E_FL_BmN-_02O05_F_0	BJ980614
E_FL_BmN-_02O05_R_0	BJ986913
E_FL_BmN-_02O06_F_0	BJ980615
E_FL_BmN-_02O06_R_0	BJ986914
E_FL_BmN-_02O07_F_0	BJ980616
E_FL_BmN-_02O07_R_0	BJ986915
E_FL_BmN-_02O08_F_0	BJ980617
E_FL_BmN-_02O08_R_0	BJ986916
E_FL_BmN-_02O09_F_0	BJ980618
E_FL_BmN-_02O09_R_0	BJ986917
E_FL_BmN-_02O10_F_0	BJ980619
E_FL_BmN-_02O10_R_0	BJ986918
E_FL_BmN-_02O11_F_0	BJ980620
E_FL_BmN-_02O11_R_0	BJ986919
E_FL_BmN-_02O12_F_0	BJ980621
E_FL_BmN-_02O12_R_0	BJ986920
E_FL_BmN-_02O13_F_0	BJ980622
E_FL_BmN-_02O13_R_0	BJ986921
E_FL_BmN-_02O15_F_0	BJ980623
E_FL_BmN-_02O15_R_0	BJ986922
E_FL_BmN-_02O16_F_0	BJ980624
E_FL_BmN-_02O16_R_0	BJ986923
E_FL_BmN-_02O17_F_0	BJ980625
E_FL_BmN-_02O17_R_0	BJ986924
E_FL_BmN-_02O18_F_0	BJ980626
E_FL_BmN-_02O18_R_0	BJ986925
E_FL_BmN-_02O19_F_0	BJ980627
E_FL_BmN-_02O19_R_0	BJ986926
E_FL_BmN-_02O20_F_0	BJ980628
E_FL_BmN-_02O20_R_0	BJ986927
E_FL_BmN-_02O21_F_0	BJ980629
E_FL_BmN-_02O21_R_0	BJ986928
E_FL_BmN-_02O22_F_0	BJ980630
E_FL_BmN-_02O22_R_0	BJ986929
E_FL_BmN-_02O24_F_0	BJ980631
E_FL_BmN-_02O24_R_0	BJ986930
E_FL_BmN-_02P01_F_0	BJ980632
E_FL_BmN-_02P01_R_0	BJ986931
E_FL_BmN-_02P02_F_0	BJ980633

E_FL_BmN-_02P02_R_0	BJ986932
E_FL_BmN-_02P03_F_0	BJ980634
E_FL_BmN-_02P03_R_0	BJ986933
E_FL_BmN-_02P04_F_0	BJ980635
E_FL_BmN-_02P04_R_0	BJ986934
E_FL_BmN-_02P05_R_0	BJ986935
E_FL_BmN-_02P06_F_0	BJ980636
E_FL_BmN-_02P06_R_0	BJ986936
E_FL_BmN-_02P07_F_0	BJ980637
E_FL_BmN-_02P07_R_0	BJ986937
E_FL_BmN-_02P08_F_0	BJ980638
E_FL_BmN-_02P08_R_0	BJ986938
E_FL_BmN-_02P09_F_0	BJ980639
E_FL_BmN-_02P09_R_0	BJ986939
E_FL_BmN-_02P10_F_0	BJ980640
E_FL_BmN-_02P10_R_0	BJ986940
E_FL_BmN-_02P11_R_0	BJ986941
E_FL_BmN-_02P12_F_0	BJ980641
E_FL_BmN-_02P12_R_0	BJ986942
E_FL_BmN-_02P13_F_0	BJ980642
E_FL_BmN-_02P13_R_0	BJ986943
E_FL_BmN-_02P14_F_0	BJ980643
E_FL_BmN-_02P14_R_0	BJ986944
E_FL_BmN-_02P15_F_0	BJ980644
E_FL_BmN-_02P15_R_0	BJ986945
E_FL_BmN-_02P16_F_0	BJ980645
E_FL_BmN-_02P16_R_0	BJ986946
E_FL_BmN-_02P17_F_0	BJ980646
E_FL_BmN-_02P17_R_0	BJ986947
E_FL_BmN-_02P18_F_0	BJ980647
E_FL_BmN-_02P18_R_0	BJ986948
E_FL_BmN-_02P19_F_0	BJ980648
E_FL_BmN-_02P19_R_0	BJ986949
E_FL_BmN-_02P20_F_0	BJ980649
E_FL_BmN-_02P20_R_0	BJ986950
E_FL_BmN-_02P21_F_0	BJ980650
E_FL_BmN-_02P21_R_0	BJ986951
E_FL_BmN-_02P22_F_0	BJ980651
E_FL_BmN-_02P22_R_0	BJ986952
E_FL_BmN-_02P24_F_0	BJ980652
E_FL_BmN-_02P24_R_0	BJ986953
E_FL_BmN-_03A01_F_0	BJ980653
E_FL_BmN-_03A01_R_0	BJ986954
E_FL_BmN-_03A03_F_0	BJ980654
E_FL_BmN-_03A03_R_0	BJ986955
E_FL_BmN-_03A04_F_0	BJ980655
E_FL_BmN-_03A04_R_0	BJ986956
E_FL_BmN-_03A05_F_0	BJ980656
E_FL_BmN-_03A05_R_0	BJ986957
E_FL_BmN-_03A06_F_0	BJ980657
E_FL_BmN-_03A06_R_0	BJ986958
E_FL_BmN-_03A07_F_0	BJ980658
E_FL_BmN-_03A09_F_0	BJ980659
E_FL_BmN-_03A09_R_0	BJ986959

E_FL_BmN-_03A10_F_0	BJ980660
E_FL_BmN-_03A10_R_0	BJ986960
E_FL_BmN-_03A11_F_0	BJ980661
E_FL_BmN-_03A11_R_0	BJ986961
E_FL_BmN-_03A12_F_0	BJ980662
E_FL_BmN-_03A12_R_0	BJ986962
E_FL_BmN-_03A13_F_0	BJ980663
E_FL_BmN-_03A14_F_0	BJ980664
E_FL_BmN-_03A14_R_0	BJ986963
E_FL_BmN-_03A15_F_0	BJ980665
E_FL_BmN-_03A15_R_0	BJ986964
E_FL_BmN-_03A16_F_0	BJ980666
E_FL_BmN-_03A17_F_0	BJ980667
E_FL_BmN-_03A17_R_0	BJ986965
E_FL_BmN-_03A18_F_0	BJ980668
E_FL_BmN-_03A18_R_0	BJ986966
E_FL_BmN-_03A19_F_0	BJ980669
E_FL_BmN-_03A21_F_0	BJ980670
E_FL_BmN-_03A21_R_0	BJ986967
E_FL_BmN-_03A22_F_0	BJ980671
E_FL_BmN-_03A23_F_0	BJ980672
E_FL_BmN-_03A23_R_0	BJ986968
E_FL_BmN-_03A24_F_0	BJ980673
E_FL_BmN-_03A24_R_0	BJ986969
E_FL_BmN-_03B01_F_0	BJ980674
E_FL_BmN-_03B02_F_0	BJ980675
E_FL_BmN-_03B03_F_0	BJ980676
E_FL_BmN-_03B03_R_0	BJ986970
E_FL_BmN-_03B04_F_0	BJ980677
E_FL_BmN-_03B04_R_0	BJ986971
E_FL_BmN-_03B06_F_0	BJ980678
E_FL_BmN-_03B07_F_0	BJ980679
E_FL_BmN-_03B09_F_0	BJ980680
E_FL_BmN-_03B09_R_0	BJ986972
E_FL_BmN-_03B10_F_0	BJ980681
E_FL_BmN-_03B11_F_0	BJ980682
E_FL_BmN-_03B11_R_0	BJ986973
E_FL_BmN-_03B12_F_0	BJ980683
E_FL_BmN-_03B13_F_0	BJ980684
E_FL_BmN-_03B13_R_0	BJ986974
E_FL_BmN-_03B14_F_0	BJ980685
E_FL_BmN-_03B14_R_0	BJ986975
E_FL_BmN-_03B15_F_0	BJ980686
E_FL_BmN-_03B15_R_0	BJ986976
E_FL_BmN-_03B16_F_0	BJ980687
E_FL_BmN-_03B16_R_0	BJ986977
E_FL_BmN-_03B17_F_0	BJ980688
E_FL_BmN-_03B17_R_0	BJ986978
E_FL_BmN-_03B18_F_0	BJ980689
E_FL_BmN-_03B19_F_0	BJ980690
E_FL_BmN-_03B19_R_0	BJ986979
E_FL_BmN-_03B20_F_0	BJ980691
E_FL_BmN-_03B20_R_0	BJ986980
E_FL_BmN-_03B21_F_0	BJ980692

E_FL_BmN-_03B21_R_0	BJ986981
E_FL_BmN-_03B22_F_0	BJ980693
E_FL_BmN-_03B22_R_0	BJ986982
E_FL_BmN-_03B23_F_0	BJ980694
E_FL_BmN-_03B23_R_0	BJ986983
E_FL_BmN-_03B24_F_0	BJ980695
E_FL_BmN-_03B24_R_0	BJ986984
E_FL_BmN-_03C01_F_0	BJ980696
E_FL_BmN-_03C01_R_0	BJ986985
E_FL_BmN-_03C02_F_0	BJ980697
E_FL_BmN-_03C02_R_0	BJ986986
E_FL_BmN-_03C03_F_0	BJ980698
E_FL_BmN-_03C03_R_0	BJ986987
E_FL_BmN-_03C04_F_0	BJ980699
E_FL_BmN-_03C04_R_0	BJ986988
E_FL_BmN-_03C05_F_0	BJ980700
E_FL_BmN-_03C05_R_0	BJ986989
E_FL_BmN-_03C06_F_0	BJ980701
E_FL_BmN-_03C06_R_0	BJ986990
E_FL_BmN-_03C07_F_0	BJ980702
E_FL_BmN-_03C07_R_0	BJ986991
E_FL_BmN-_03C08_F_0	BJ980703
E_FL_BmN-_03C08_R_0	BJ986992
E_FL_BmN-_03C09_F_0	BJ980704
E_FL_BmN-_03C09_R_0	BJ986993
E_FL_BmN-_03C10_F_0	BJ980705
E_FL_BmN-_03C10_R_0	BJ986994
E_FL_BmN-_03C12_F_0	BJ980706
E_FL_BmN-_03C13_F_0	BJ980707
E_FL_BmN-_03C13_R_0	BJ986995
E_FL_BmN-_03C14_F_0	BJ980708
E_FL_BmN-_03C14_R_0	BJ986996
E_FL_BmN-_03C15_R_0	BJ986997
E_FL_BmN-_03C16_F_0	BJ980709
E_FL_BmN-_03C16_R_0	BJ986998
E_FL_BmN-_03C17_F_0	BJ980710
E_FL_BmN-_03C17_R_0	BJ986999
E_FL_BmN-_03C18_F_0	BJ980711
E_FL_BmN-_03C19_R_0	BJ987000
E_FL_BmN-_03C20_F_0	BJ980712
E_FL_BmN-_03C20_R_0	BJ987001
E_FL_BmN-_03C21_F_0	BJ980713
E_FL_BmN-_03C21_R_0	BJ987002
E_FL_BmN-_03C23_F_0	BJ980714
E_FL_BmN-_03C23_R_0	BJ987003
E_FL_BmN-_03C24_F_0	BJ980715
E_FL_BmN-_03C24_R_0	BJ987004
E_FL_BmN-_03D02_F_0	BJ980716
E_FL_BmN-_03D02_R_0	BJ987005
E_FL_BmN-_03D03_F_0	BJ980717
E_FL_BmN-_03D03_R_0	BJ987006
E_FL_BmN-_03D04_F_0	BJ980718
E_FL_BmN-_03D04_R_0	BJ987007
E_FL_BmN-_03D05_F_0	BJ980719

E_FL_BmN-_03D05_R_0	BJ987008
E_FL_BmN-_03D06_F_0	BJ980720
E_FL_BmN-_03D06_R_0	BJ987009
E_FL_BmN-_03D07_F_0	BJ980721
E_FL_BmN-_03D07_R_0	BJ987010
E_FL_BmN-_03D08_F_0	BJ980722
E_FL_BmN-_03D08_R_0	BJ987011
E_FL_BmN-_03D09_F_0	BJ980723
E_FL_BmN-_03D09_R_0	BJ987012
E_FL_BmN-_03D10_F_0	BJ980724
E_FL_BmN-_03D10_R_0	BJ987013
E_FL_BmN-_03D11_F_0	BJ980725
E_FL_BmN-_03D11_R_0	BJ987014
E_FL_BmN-_03D12_F_0	BJ980726
E_FL_BmN-_03D13_F_0	BJ980727
E_FL_BmN-_03D13_R_0	BJ987015
E_FL_BmN-_03D14_F_0	BJ980728
E_FL_BmN-_03D14_R_0	BJ987016
E_FL_BmN-_03D15_F_0	BJ980729
E_FL_BmN-_03D15_R_0	BJ987017
E_FL_BmN-_03D16_F_0	BJ980730
E_FL_BmN-_03D17_F_0	BJ980731
E_FL_BmN-_03D17_R_0	BJ987018
E_FL_BmN-_03D18_F_0	BJ980732
E_FL_BmN-_03D18_R_0	BJ987019
E_FL_BmN-_03D19_F_0	BJ980733
E_FL_BmN-_03D20_F_0	BJ980734
E_FL_BmN-_03D20_R_0	BJ987020
E_FL_BmN-_03D21_F_0	BJ980735
E_FL_BmN-_03D21_R_0	BJ987021
E_FL_BmN-_03D22_F_0	BJ980736
E_FL_BmN-_03D23_F_0	BJ980737
E_FL_BmN-_03D23_R_0	BJ987022
E_FL_BmN-_03E01_F_0	BJ980738
E_FL_BmN-_03E01_R_0	BJ987023
E_FL_BmN-_03E02_F_0	BJ980739
E_FL_BmN-_03E02_R_0	BJ987024
E_FL_BmN-_03E03_F_0	BJ980740
E_FL_BmN-_03E03_R_0	BJ987025
E_FL_BmN-_03E04_F_0	BJ980741
E_FL_BmN-_03E04_R_0	BJ987026
E_FL_BmN-_03E05_F_0	BJ980742
E_FL_BmN-_03E05_R_0	BJ987027
E_FL_BmN-_03E06_F_0	BJ980743
E_FL_BmN-_03E06_R_0	BJ987028
E_FL_BmN-_03E08_F_0	BJ980744
E_FL_BmN-_03E08_R_0	BJ987029
E_FL_BmN-_03E09_F_0	BJ980745
E_FL_BmN-_03E09_R_0	BJ987030
E_FL_BmN-_03E10_F_0	BJ980746
E_FL_BmN-_03E10_R_0	BJ987031
E_FL_BmN-_03E11_R_0	BJ987032
E_FL_BmN-_03E12_F_0	BJ980747
E_FL_BmN-_03E12_R_0	BJ987033

E_FL_BmN-_03E13_F_0	BJ980748
E_FL_BmN-_03E13_R_0	BJ987034
E_FL_BmN-_03E14_F_0	BJ980749
E_FL_BmN-_03E14_R_0	BJ987035
E_FL_BmN-_03E15_F_0	BJ980750
E_FL_BmN-_03E16_F_0	BJ980751
E_FL_BmN-_03E16_R_0	BJ987036
E_FL_BmN-_03E17_F_0	BJ980752
E_FL_BmN-_03E17_R_0	BJ987037
E_FL_BmN-_03E18_F_0	BJ980753
E_FL_BmN-_03E18_R_0	BJ987038
E_FL_BmN-_03E19_F_0	BJ980754
E_FL_BmN-_03E20_F_0	BJ980755
E_FL_BmN-_03E20_R_0	BJ987039
E_FL_BmN-_03E22_F_0	BJ980756
E_FL_BmN-_03E22_R_0	BJ987040
E_FL_BmN-_03E23_F_0	BJ980757
E_FL_BmN-_03E23_R_0	BJ987041
E_FL_BmN-_03E24_F_0	BJ980758
E_FL_BmN-_03E24_R_0	BJ987042
E_FL_BmN-_03F01_F_0	BJ980759
E_FL_BmN-_03F01_R_0	BJ987043
E_FL_BmN-_03F02_F_0	BJ980760
E_FL_BmN-_03F02_R_0	BJ987044
E_FL_BmN-_03F03_F_0	BJ980761
E_FL_BmN-_03F03_R_0	BJ987045
E_FL_BmN-_03F04_F_0	BJ980762
E_FL_BmN-_03F04_R_0	BJ987046
E_FL_BmN-_03F05_F_0	BJ980763
E_FL_BmN-_03F05_R_0	BJ987047
E_FL_BmN-_03F06_F_0	BJ980764
E_FL_BmN-_03F06_R_0	BJ987048
E_FL_BmN-_03F07_F_0	BJ980765
E_FL_BmN-_03F08_F_0	BJ980766
E_FL_BmN-_03F08_R_0	BJ987049
E_FL_BmN-_03F09_F_0	BJ980767
E_FL_BmN-_03F09_R_0	BJ987050
E_FL_BmN-_03F10_F_0	BJ980768
E_FL_BmN-_03F10_R_0	BJ987051
E_FL_BmN-_03F11_F_0	BJ980769
E_FL_BmN-_03F12_F_0	BJ980770
E_FL_BmN-_03F12_R_0	BJ987052
E_FL_BmN-_03F13_F_0	BJ980771
E_FL_BmN-_03F13_R_0	BJ987053
E_FL_BmN-_03F14_F_0	BJ980772
E_FL_BmN-_03F14_R_0	BJ987054
E_FL_BmN-_03F15_F_0	BJ980773
E_FL_BmN-_03F15_R_0	BJ987055
E_FL_BmN-_03F16_F_0	BJ980774
E_FL_BmN-_03F16_R_0	BJ987056
E_FL_BmN-_03F17_F_0	BJ980775
E_FL_BmN-_03F17_R_0	BJ987057
E_FL_BmN-_03F18_F_0	BJ980776
E_FL_BmN-_03F18_R_0	BJ987058

E_FL_BmN-_03F19_F_0	BJ980777
E_FL_BmN-_03F19_R_0	BJ987059
E_FL_BmN-_03F20_F_0	BJ980778
E_FL_BmN-_03F20_R_0	BJ987060
E_FL_BmN-_03F21_F_0	BJ980779
E_FL_BmN-_03F21_R_0	BJ987061
E_FL_BmN-_03F22_F_0	BJ980780
E_FL_BmN-_03F22_R_0	BJ987062
E_FL_BmN-_03F23_F_0	BJ980781
E_FL_BmN-_03F23_R_0	BJ987063
E_FL_BmN-_03G01_F_0	BJ980782
E_FL_BmN-_03G02_F_0	BJ980783
E_FL_BmN-_03G02_R_0	BJ987064
E_FL_BmN-_03G03_F_0	BJ980784
E_FL_BmN-_03G03_R_0	BJ987065
E_FL_BmN-_03G04_F_0	BJ980785
E_FL_BmN-_03G05_F_0	BJ980786
E_FL_BmN-_03G05_R_0	BJ987066
E_FL_BmN-_03G06_F_0	BJ980787
E_FL_BmN-_03G07_R_0	BJ987067
E_FL_BmN-_03G08_F_0	BJ980788
E_FL_BmN-_03G08_R_0	BJ987068
E_FL_BmN-_03G09_F_0	BJ980789
E_FL_BmN-_03G09_R_0	BJ987069
E_FL_BmN-_03G11_F_0	BJ980790
E_FL_BmN-_03G11_R_0	BJ987070
E_FL_BmN-_03G12_F_0	BJ980791
E_FL_BmN-_03G13_F_0	BJ980792
E_FL_BmN-_03G14_F_0	BJ980793
E_FL_BmN-_03G14_R_0	BJ987071
E_FL_BmN-_03G15_F_0	BJ980794
E_FL_BmN-_03G15_R_0	BJ987072
E_FL_BmN-_03G17_F_0	BJ980795
E_FL_BmN-_03G17_R_0	BJ987073
E_FL_BmN-_03G18_F_0	BJ980796
E_FL_BmN-_03G19_F_0	BJ980797
E_FL_BmN-_03G19_R_0	BJ987074
E_FL_BmN-_03G20_F_0	BJ980798
E_FL_BmN-_03G20_R_0	BJ987075
E_FL_BmN-_03G21_F_0	BJ980799
E_FL_BmN-_03G21_R_0	BJ987076
E_FL_BmN-_03G22_F_0	BJ980800
E_FL_BmN-_03G22_R_0	BJ987077
E_FL_BmN-_03G23_F_0	BJ980801
E_FL_BmN-_03G23_R_0	BJ987078
E_FL_BmN-_03G24_F_0	BJ980802
E_FL_BmN-_03H01_F_0	BJ980803
E_FL_BmN-_03H01_R_0	BJ987079
E_FL_BmN-_03H02_F_0	BJ980804
E_FL_BmN-_03H02_R_0	BJ987080
E_FL_BmN-_03H03_F_0	BJ980805
E_FL_BmN-_03H03_R_0	BJ987081
E_FL_BmN-_03H04_F_0	BJ980806
E_FL_BmN-_03H04_R_0	BJ987082

E_FL_BmN-_03H05_F_0	BJ980807
E_FL_BmN-_03H05_R_0	BJ987083
E_FL_BmN-_03H06_F_0	BJ980808
E_FL_BmN-_03H07_F_0	BJ980809
E_FL_BmN-_03H07_R_0	BJ987084
E_FL_BmN-_03H08_F_0	BJ980810
E_FL_BmN-_03H08_R_0	BJ987085
E_FL_BmN-_03H09_F_0	BJ980811
E_FL_BmN-_03H09_R_0	BJ987086
E_FL_BmN-_03H10_F_0	BJ980812
E_FL_BmN-_03H10_R_0	BJ987087
E_FL_BmN-_03H11_F_0	BJ980813
E_FL_BmN-_03H11_R_0	BJ987088
E_FL_BmN-_03H12_F_0	BJ980814
E_FL_BmN-_03H13_F_0	BJ980815
E_FL_BmN-_03H13_R_0	BJ987089
E_FL_BmN-_03H14_F_0	BJ980816
E_FL_BmN-_03H14_R_0	BJ987090
E_FL_BmN-_03H15_F_0	BJ980817
E_FL_BmN-_03H15_R_0	BJ987091
E_FL_BmN-_03H16_F_0	BJ980818
E_FL_BmN-_03H16_R_0	BJ987092
E_FL_BmN-_03H17_F_0	BJ980819
E_FL_BmN-_03H17_R_0	BJ987093
E_FL_BmN-_03H18_F_0	BJ980820
E_FL_BmN-_03H19_F_0	BJ980821
E_FL_BmN-_03H19_R_0	BJ987094
E_FL_BmN-_03H20_F_0	BJ980822
E_FL_BmN-_03H20_R_0	BJ987095
E_FL_BmN-_03H22_F_0	BJ980823
E_FL_BmN-_03H22_R_0	BJ987096
E_FL_BmN-_03H23_F_0	BJ980824
E_FL_BmN-_03H23_R_0	BJ987097
E_FL_BmN-_03H24_F_0	BJ980825
E_FL_BmN-_03H24_R_0	BJ987098
E_FL_BmN-_03I02_F_0	BJ980826
E_FL_BmN-_03I02_R_0	BJ987099
E_FL_BmN-_03I03_F_0	BJ980827
E_FL_BmN-_03I03_R_0	BJ987100
E_FL_BmN-_03I04_F_0	BJ980828
E_FL_BmN-_03I04_R_0	BJ987101
E_FL_BmN-_03I05_F_0	BJ980829
E_FL_BmN-_03I05_R_0	BJ987102
E_FL_BmN-_03I06_F_0	BJ980830
E_FL_BmN-_03I06_R_0	BJ987103
E_FL_BmN-_03I07_F_0	BJ980831
E_FL_BmN-_03I07_R_0	BJ987104
E_FL_BmN-_03I08_F_0	BJ980832
E_FL_BmN-_03I08_R_0	BJ987105
E_FL_BmN-_03I09_F_0	BJ980833
E_FL_BmN-_03I09_R_0	BJ987106
E_FL_BmN-_03I10_F_0	BJ980834
E_FL_BmN-_03I10_R_0	BJ987107
E_FL_BmN-_03I11_F_0	BJ980835

E_FL_BmN-_03I11_R_0	BJ987108
E_FL_BmN-_03I12_F_0	BJ980836
E_FL_BmN-_03I12_R_0	BJ987109
E_FL_BmN-_03I13_F_0	BJ980837
E_FL_BmN-_03I13_R_0	BJ987110
E_FL_BmN-_03I14_F_0	BJ980838
E_FL_BmN-_03I14_R_0	BJ987111
E_FL_BmN-_03I15_F_0	BJ980839
E_FL_BmN-_03I15_R_0	BJ987112
E_FL_BmN-_03I16_F_0	BJ980840
E_FL_BmN-_03I16_R_0	BJ987113
E_FL_BmN-_03I17_R_0	BJ987114
E_FL_BmN-_03I18_F_0	BJ980841
E_FL_BmN-_03I18_R_0	BJ987115
E_FL_BmN-_03I19_R_0	BJ987116
E_FL_BmN-_03I20_R_0	BJ987117
E_FL_BmN-_03I21_F_0	BJ980842
E_FL_BmN-_03I21_R_0	BJ987118
E_FL_BmN-_03I22_F_0	BJ980843
E_FL_BmN-_03I22_R_0	BJ987119
E_FL_BmN-_03I24_F_0	BJ980844
E_FL_BmN-_03J02_F_0	BJ980845
E_FL_BmN-_03J02_R_0	BJ987120
E_FL_BmN-_03J03_F_0	BJ980846
E_FL_BmN-_03J03_R_0	BJ987121
E_FL_BmN-_03J04_F_0	BJ980847
E_FL_BmN-_03J04_R_0	BJ987122
E_FL_BmN-_03J05_F_0	BJ980848
E_FL_BmN-_03J05_R_0	BJ987123
E_FL_BmN-_03J06_F_0	BJ980849
E_FL_BmN-_03J07_F_0	BJ980850
E_FL_BmN-_03J08_F_0	BJ980851
E_FL_BmN-_03J08_R_0	BJ987124
E_FL_BmN-_03J09_F_0	BJ980852
E_FL_BmN-_03J09_R_0	BJ987125
E_FL_BmN-_03J11_F_0	BJ980853
E_FL_BmN-_03J11_R_0	BJ987126
E_FL_BmN-_03J12_F_0	BJ980854
E_FL_BmN-_03J12_R_0	BJ987127
E_FL_BmN-_03J13_F_0	BJ980855
E_FL_BmN-_03J13_R_0	BJ987128
E_FL_BmN-_03J14_F_0	BJ980856
E_FL_BmN-_03J14_R_0	BJ987129
E_FL_BmN-_03J15_F_0	BJ980857
E_FL_BmN-_03J15_R_0	BJ987130
E_FL_BmN-_03J16_F_0	BJ980858
E_FL_BmN-_03J16_R_0	BJ987131
E_FL_BmN-_03J17_F_0	BJ980859
E_FL_BmN-_03J17_R_0	BJ987132
E_FL_BmN-_03J18_F_0	BJ980860
E_FL_BmN-_03J18_R_0	BJ987133
E_FL_BmN-_03J19_F_0	BJ980861
E_FL_BmN-_03J20_F_0	BJ980862
E_FL_BmN-_03J20_R_0	BJ987134

E_FL_BmN-_03J21_F_0	BJ980863
E_FL_BmN-_03J21_R_0	BJ987135
E_FL_BmN-_03J22_F_0	BJ980864
E_FL_BmN-_03J22_R_0	BJ987136
E_FL_BmN-_03J23_F_0	BJ980865
E_FL_BmN-_03J23_R_0	BJ987137
E_FL_BmN-_03J24_F_0	BJ980866
E_FL_BmN-_03K02_F_0	BJ980867
E_FL_BmN-_03K02_R_0	BJ987138
E_FL_BmN-_03K03_F_0	BJ980868
E_FL_BmN-_03K03_R_0	BJ987139
E_FL_BmN-_03K04_F_0	BJ980869
E_FL_BmN-_03K04_R_0	BJ987140
E_FL_BmN-_03K05_F_0	BJ980870
E_FL_BmN-_03K05_R_0	BJ987141
E_FL_BmN-_03K06_F_0	BJ980871
E_FL_BmN-_03K06_R_0	BJ987142
E_FL_BmN-_03K07_F_0	BJ980872
E_FL_BmN-_03K07_R_0	BJ987143
E_FL_BmN-_03K08_F_0	BJ980873
E_FL_BmN-_03K08_R_0	BJ987144
E_FL_BmN-_03K09_F_0	BJ980874
E_FL_BmN-_03K09_R_0	BJ987145
E_FL_BmN-_03K10_F_0	BJ980875
E_FL_BmN-_03K11_F_0	BJ980876
E_FL_BmN-_03K11_R_0	BJ987146
E_FL_BmN-_03K12_F_0	BJ980877
E_FL_BmN-_03K12_R_0	BJ987147
E_FL_BmN-_03K13_F_0	BJ980878
E_FL_BmN-_03K13_R_0	BJ987148
E_FL_BmN-_03K14_F_0	BJ980879
E_FL_BmN-_03K14_R_0	BJ987149
E_FL_BmN-_03K15_F_0	BJ980880
E_FL_BmN-_03K15_R_0	BJ987150
E_FL_BmN-_03K16_F_0	BJ980881
E_FL_BmN-_03K16_R_0	BJ987151
E_FL_BmN-_03K17_F_0	BJ980882
E_FL_BmN-_03K17_R_0	BJ987152
E_FL_BmN-_03K18_F_0	BJ980883
E_FL_BmN-_03K18_R_0	BJ987153
E_FL_BmN-_03K19_F_0	BJ980884
E_FL_BmN-_03K19_R_0	BJ987154
E_FL_BmN-_03K20_F_0	BJ980885
E_FL_BmN-_03K20_R_0	BJ987155
E_FL_BmN-_03K21_F_0	BJ980886
E_FL_BmN-_03K21_R_0	BJ987156
E_FL_BmN-_03K22_F_0	BJ980887
E_FL_BmN-_03K22_R_0	BJ987157
E_FL_BmN-_03K23_F_0	BJ980888
E_FL_BmN-_03K23_R_0	BJ987158
E_FL_BmN-_03K24_F_0	BJ980889
E_FL_BmN-_03K24_R_0	BJ987159
E_FL_BmN-_03L02_F_0	BJ980890
E_FL_BmN-_03L04_F_0	BJ980891

E_FL_BmN-_03L05_F_0	BJ980892
E_FL_BmN-_03L05_R_0	BJ987160
E_FL_BmN-_03L06_F_0	BJ980893
E_FL_BmN-_03L06_R_0	BJ987161
E_FL_BmN-_03L07_F_0	BJ980894
E_FL_BmN-_03L07_R_0	BJ987162
E_FL_BmN-_03L08_F_0	BJ980895
E_FL_BmN-_03L08_R_0	BJ987163
E_FL_BmN-_03L09_F_0	BJ980896
E_FL_BmN-_03L09_R_0	BJ987164
E_FL_BmN-_03L11_F_0	BJ980897
E_FL_BmN-_03L11_R_0	BJ987165
E_FL_BmN-_03L12_F_0	BJ980898
E_FL_BmN-_03L13_F_0	BJ980899
E_FL_BmN-_03L13_R_0	BJ987166
E_FL_BmN-_03L14_F_0	BJ980900
E_FL_BmN-_03L14_R_0	BJ987167
E_FL_BmN-_03L15_F_0	BJ980901
E_FL_BmN-_03L15_R_0	BJ987168
E_FL_BmN-_03L16_F_0	BJ980902
E_FL_BmN-_03L17_F_0	BJ980903
E_FL_BmN-_03L17_R_0	BJ987169
E_FL_BmN-_03L18_F_0	BJ980904
E_FL_BmN-_03L18_R_0	BJ987170
E_FL_BmN-_03L19_F_0	BJ980905
E_FL_BmN-_03L20_F_0	BJ980906
E_FL_BmN-_03L20_R_0	BJ987171
E_FL_BmN-_03L21_F_0	BJ980907
E_FL_BmN-_03L21_R_0	BJ987172
E_FL_BmN-_03L22_F_0	BJ980908
E_FL_BmN-_03L22_R_0	BJ987173
E_FL_BmN-_03L23_F_0	BJ980909
E_FL_BmN-_03L24_F_0	BJ980910
E_FL_BmN-_03L24_R_0	BJ987174
E_FL_BmN-_03M01_R_0	BJ987175
E_FL_BmN-_03M02_F_0	BJ980911
E_FL_BmN-_03M02_R_0	BJ987176
E_FL_BmN-_03M03_F_0	BJ980912
E_FL_BmN-_03M03_R_0	BJ987177
E_FL_BmN-_03M04_F_0	BJ980913
E_FL_BmN-_03M04_R_0	BJ987178
E_FL_BmN-_03M06_F_0	BJ980914
E_FL_BmN-_03M07_F_0	BJ980915
E_FL_BmN-_03M07_R_0	BJ987179
E_FL_BmN-_03M08_F_0	BJ980916
E_FL_BmN-_03M08_R_0	BJ987180
E_FL_BmN-_03M09_F_0	BJ980917
E_FL_BmN-_03M09_R_0	BJ987181
E_FL_BmN-_03M10_F_0	BJ980918
E_FL_BmN-_03M10_R_0	BJ987182
E_FL_BmN-_03M11_F_0	BJ980919
E_FL_BmN-_03M11_R_0	BJ987183
E_FL_BmN-_03M12_F_0	BJ980920
E_FL_BmN-_03M12_R_0	BJ987184

E_FL_BmN-_03M13_F_0	BJ980921
E_FL_BmN-_03M13_R_0	BJ987185
E_FL_BmN-_03M14_F_0	BJ980922
E_FL_BmN-_03M14_R_0	BJ987186
E_FL_BmN-_03M15_F_0	BJ980923
E_FL_BmN-_03M15_R_0	BJ987187
E_FL_BmN-_03M17_F_0	BJ980924
E_FL_BmN-_03M17_R_0	BJ987188
E_FL_BmN-_03M18_F_0	BJ980925
E_FL_BmN-_03M21_F_0	BJ980926
E_FL_BmN-_03M21_R_0	BJ987189
E_FL_BmN-_03M22_F_0	BJ980927
E_FL_BmN-_03M22_R_0	BJ987190
E_FL_BmN-_03M23_F_0	BJ980928
E_FL_BmN-_03M23_R_0	BJ987191
E_FL_BmN-_03M24_F_0	BJ980929
E_FL_BmN-_03N01_F_0	BJ980930
E_FL_BmN-_03N01_R_0	BJ987192
E_FL_BmN-_03N02_F_0	BJ980931
E_FL_BmN-_03N02_R_0	BJ987193
E_FL_BmN-_03N03_F_0	BJ980932
E_FL_BmN-_03N03_R_0	BJ987194
E_FL_BmN-_03N04_F_0	BJ980933
E_FL_BmN-_03N05_F_0	BJ980934
E_FL_BmN-_03N05_R_0	BJ987195
E_FL_BmN-_03N06_F_0	BJ980935
E_FL_BmN-_03N06_R_0	BJ987196
E_FL_BmN-_03N07_F_0	BJ980936
E_FL_BmN-_03N07_R_0	BJ987197
E_FL_BmN-_03N08_F_0	BJ980937
E_FL_BmN-_03N08_R_0	BJ987198
E_FL_BmN-_03N10_F_0	BJ980938
E_FL_BmN-_03N10_R_0	BJ987199
E_FL_BmN-_03N11_F_0	BJ980939
E_FL_BmN-_03N11_R_0	BJ987200
E_FL_BmN-_03N12_F_0	BJ980940
E_FL_BmN-_03N12_R_0	BJ987201
E_FL_BmN-_03N13_R_0	BJ987202
E_FL_BmN-_03N14_F_0	BJ980941
E_FL_BmN-_03N14_R_0	BJ987203
E_FL_BmN-_03N16_F_0	BJ980942
E_FL_BmN-_03N16_R_0	BJ987204
E_FL_BmN-_03N17_F_0	BJ980943
E_FL_BmN-_03N17_R_0	BJ987205
E_FL_BmN-_03N18_F_0	BJ980944
E_FL_BmN-_03N19_F_0	BJ980945
E_FL_BmN-_03N20_F_0	BJ980946
E_FL_BmN-_03N20_R_0	BJ987206
E_FL_BmN-_03N21_F_0	BJ980947
E_FL_BmN-_03N21_R_0	BJ987207
E_FL_BmN-_03N22_F_0	BJ980948
E_FL_BmN-_03N22_R_0	BJ987208
E_FL_BmN-_03N23_F_0	BJ980949
E_FL_BmN-_03N23_R_0	BJ987209

E_FL_BmN-_03N24_F_0	BJ980950
E_FL_BmN-_03O01_F_0	BJ980951
E_FL_BmN-_03O01_R_0	BJ987210
E_FL_BmN-_03O02_F_0	BJ980952
E_FL_BmN-_03O02_R_0	BJ987211
E_FL_BmN-_03O03_F_0	BJ980953
E_FL_BmN-_03O03_R_0	BJ987212
E_FL_BmN-_03O04_F_0	BJ980954
E_FL_BmN-_03O04_R_0	BJ987213
E_FL_BmN-_03O05_F_0	BJ980955
E_FL_BmN-_03O05_R_0	BJ987214
E_FL_BmN-_03O06_F_0	BJ980956
E_FL_BmN-_03O06_R_0	BJ987215
E_FL_BmN-_03O07_F_0	BJ980957
E_FL_BmN-_03O07_R_0	BJ987216
E_FL_BmN-_03O08_F_0	BJ980958
E_FL_BmN-_03O08_R_0	BJ987217
E_FL_BmN-_03O09_F_0	BJ980959
E_FL_BmN-_03O10_F_0	BJ980960
E_FL_BmN-_03O10_R_0	BJ987218
E_FL_BmN-_03O11_F_0	BJ980961
E_FL_BmN-_03O11_R_0	BJ987219
E_FL_BmN-_03O12_F_0	BJ980962
E_FL_BmN-_03O13_F_0	BJ980963
E_FL_BmN-_03O13_R_0	BJ987220
E_FL_BmN-_03O14_F_0	BJ980964
E_FL_BmN-_03O15_F_0	BJ980965
E_FL_BmN-_03O15_R_0	BJ987221
E_FL_BmN-_03O17_F_0	BJ980966
E_FL_BmN-_03O17_R_0	BJ987222
E_FL_BmN-_03O18_F_0	BJ980967
E_FL_BmN-_03O18_R_0	BJ987223
E_FL_BmN-_03O19_F_0	BJ980968
E_FL_BmN-_03O19_R_0	BJ987224
E_FL_BmN-_03O20_F_0	BJ980969
E_FL_BmN-_03O20_R_0	BJ987225
E_FL_BmN-_03O21_F_0	BJ980970
E_FL_BmN-_03O21_R_0	BJ987226
E_FL_BmN-_03O22_F_0	BJ980971
E_FL_BmN-_03O22_R_0	BJ987227
E_FL_BmN-_03O23_F_0	BJ980972
E_FL_BmN-_03O24_F_0	BJ980973
E_FL_BmN-_03O24_R_0	BJ987228
E_FL_BmN-_03P01_F_0	BJ980974
E_FL_BmN-_03P01_R_0	BJ987229
E_FL_BmN-_03P03_F_0	BJ980975
E_FL_BmN-_03P03_R_0	BJ987230
E_FL_BmN-_03P04_F_0	BJ980976
E_FL_BmN-_03P04_R_0	BJ987231
E_FL_BmN-_03P05_F_0	BJ980977
E_FL_BmN-_03P05_R_0	BJ987232
E_FL_BmN-_03P06_F_0	BJ980978
E_FL_BmN-_03P07_F_0	BJ980979
E_FL_BmN-_03P07_R_0	BJ987233

E_FL_BmN-_03P08_F_0	BJ980980
E_FL_BmN-_03P09_F_0	BJ980981
E_FL_BmN-_03P09_R_0	BJ987234
E_FL_BmN-_03P10_F_0	BJ980982
E_FL_BmN-_03P11_F_0	BJ980983
E_FL_BmN-_03P13_F_0	BJ980984
E_FL_BmN-_03P13_R_0	BJ987235
E_FL_BmN-_03P15_F_0	BJ980985
E_FL_BmN-_03P15_R_0	BJ987236
E_FL_BmN-_03P16_F_0	BJ980986
E_FL_BmN-_03P16_R_0	BJ987237
E_FL_BmN-_03P17_F_0	BJ980987
E_FL_BmN-_03P17_R_0	BJ987238
E_FL_BmN-_03P19_F_0	BJ980988
E_FL_BmN-_03P19_R_0	BJ987239
E_FL_BmN-_03P21_F_0	BJ980989
E_FL_BmN-_03P21_R_0	BJ987240
E_FL_BmN-_03P22_F_0	BJ980990
E_FL_BmN-_03P22_R_0	BJ987241
E_FL_BmN-_03P23_F_0	BJ980991
E_FL_BmN-_03P24_F_0	BJ980992
E_FL_BmN-_03P24_R_0	BJ987242
E_FL_BmN-_04A01_F_0	BJ980993
E_FL_BmN-_04A02_F_0	BJ980994
E_FL_BmN-_04A04_F_0	BJ980995
E_FL_BmN-_04A04_R_0	BJ987243
E_FL_BmN-_04A05_F_0	BJ980996
E_FL_BmN-_04A05_R_0	BJ987244
E_FL_BmN-_04A06_F_0	BJ980997
E_FL_BmN-_04A06_R_0	BJ987245
E_FL_BmN-_04A07_F_0	BJ980998
E_FL_BmN-_04A07_R_0	BJ987246
E_FL_BmN-_04A08_F_0	BJ980999
E_FL_BmN-_04A08_R_0	BJ987247
E_FL_BmN-_04A09_F_0	BJ981000
E_FL_BmN-_04A09_R_0	BJ987248
E_FL_BmN-_04A10_F_0	BJ981001
E_FL_BmN-_04A10_R_0	BJ987249
E_FL_BmN-_04A11_F_0	BJ981002
E_FL_BmN-_04A11_R_0	BJ987250
E_FL_BmN-_04A12_F_0	BJ981003
E_FL_BmN-_04A12_R_0	BJ987251
E_FL_BmN-_04A14_F_0	BJ981004
E_FL_BmN-_04A14_R_0	BJ987252
E_FL_BmN-_04A15_F_0	BJ981005
E_FL_BmN-_04A15_R_0	BJ987253
E_FL_BmN-_04A16_F_0	BJ981006
E_FL_BmN-_04A16_R_0	BJ987254
E_FL_BmN-_04A17_F_0	BJ981007
E_FL_BmN-_04A17_R_0	BJ987255
E_FL_BmN-_04A18_F_0	BJ981008
E_FL_BmN-_04A18_R_0	BJ987256
E_FL_BmN-_04A19_F_0	BJ981009
E_FL_BmN-_04A19_R_0	BJ987257

E_FL_BmN-_04A20_R_0	BJ987258
E_FL_BmN-_04A22_F_0	BJ981010
E_FL_BmN-_04A22_R_0	BJ987259
E_FL_BmN-_04A23_F_0	BJ981011
E_FL_BmN-_04A23_R_0	BJ987260
E_FL_BmN-_04A24_F_0	BJ981012
E_FL_BmN-_04A24_R_0	BJ987261
E_FL_BmN-_04B01_F_0	BJ981013
E_FL_BmN-_04B02_F_0	BJ981014
E_FL_BmN-_04B02_R_0	BJ987262
E_FL_BmN-_04B03_F_0	BJ981015
E_FL_BmN-_04B03_R_0	BJ987263
E_FL_BmN-_04B04_F_0	BJ981016
E_FL_BmN-_04B04_R_0	BJ987264
E_FL_BmN-_04B05_F_0	BJ981017
E_FL_BmN-_04B05_R_0	BJ987265
E_FL_BmN-_04B06_F_0	BJ981018
E_FL_BmN-_04B06_R_0	BJ987266
E_FL_BmN-_04B07_F_0	BJ981019
E_FL_BmN-_04B08_F_0	BJ981020
E_FL_BmN-_04B08_R_0	BJ987267
E_FL_BmN-_04B09_F_0	BJ981021
E_FL_BmN-_04B09_R_0	BJ987268
E_FL_BmN-_04B10_F_0	BJ981022
E_FL_BmN-_04B10_R_0	BJ987269
E_FL_BmN-_04B11_F_0	BJ981023
E_FL_BmN-_04B11_R_0	BJ987270
E_FL_BmN-_04B12_F_0	BJ981024
E_FL_BmN-_04B12_R_0	BJ987271
E_FL_BmN-_04B15_F_0	BJ981025
E_FL_BmN-_04B15_R_0	BJ987272
E_FL_BmN-_04B16_F_0	BJ981026
E_FL_BmN-_04B16_R_0	BJ987273
E_FL_BmN-_04B17_F_0	BJ981027
E_FL_BmN-_04B17_R_0	BJ987274
E_FL_BmN-_04B18_F_0	BJ981028
E_FL_BmN-_04B19_F_0	BJ981029
E_FL_BmN-_04B20_F_0	BJ981030
E_FL_BmN-_04B21_F_0	BJ981031
E_FL_BmN-_04B21_R_0	BJ987275
E_FL_BmN-_04B22_F_0	BJ981032
E_FL_BmN-_04B22_R_0	BJ987276
E_FL_BmN-_04B23_F_0	BJ981033
E_FL_BmN-_04B23_R_0	BJ987277
E_FL_BmN-_04B24_F_0	BJ981034
E_FL_BmN-_04B24_R_0	BJ987278
E_FL_BmN-_04C01_F_0	BJ981035
E_FL_BmN-_04C01_R_0	BJ987279
E_FL_BmN-_04C02_F_0	BJ981036
E_FL_BmN-_04C02_R_0	BJ987280
E_FL_BmN-_04C03_F_0	BJ981037
E_FL_BmN-_04C03_R_0	BJ987281
E_FL_BmN-_04C04_F_0	BJ981038
E_FL_BmN-_04C04_R_0	BJ987282

E_FL_BmN-_04C05_F_0	BJ981039
E_FL_BmN-_04C05_R_0	BJ987283
E_FL_BmN-_04C06_F_0	BJ981040
E_FL_BmN-_04C06_R_0	BJ987284
E_FL_BmN-_04C07_F_0	BJ981041
E_FL_BmN-_04C07_R_0	BJ987285
E_FL_BmN-_04C08_F_0	BJ981042
E_FL_BmN-_04C08_R_0	BJ987286
E_FL_BmN-_04C09_F_0	BJ981043
E_FL_BmN-_04C09_R_0	BJ987287
E_FL_BmN-_04C10_F_0	BJ981044
E_FL_BmN-_04C10_R_0	BJ987288
E_FL_BmN-_04C11_F_0	BJ981045
E_FL_BmN-_04C11_R_0	BJ987289
E_FL_BmN-_04C12_F_0	BJ981046
E_FL_BmN-_04C13_F_0	BJ981047
E_FL_BmN-_04C13_R_0	BJ987290
E_FL_BmN-_04C14_F_0	BJ981048
E_FL_BmN-_04C14_R_0	BJ987291
E_FL_BmN-_04C15_F_0	BJ981049
E_FL_BmN-_04C15_R_0	BJ987292
E_FL_BmN-_04C16_F_0	BJ981050
E_FL_BmN-_04C16_R_0	BJ987293
E_FL_BmN-_04C17_F_0	BJ981051
E_FL_BmN-_04C17_R_0	BJ987294
E_FL_BmN-_04C18_F_0	BJ981052
E_FL_BmN-_04C18_R_0	BJ987295
E_FL_BmN-_04C19_F_0	BJ981053
E_FL_BmN-_04C20_F_0	BJ981054
E_FL_BmN-_04C20_R_0	BJ987296
E_FL_BmN-_04C21_F_0	BJ981055
E_FL_BmN-_04C21_R_0	BJ987297
E_FL_BmN-_04C22_F_0	BJ981056
E_FL_BmN-_04C23_F_0	BJ981057
E_FL_BmN-_04C23_R_0	BJ987298
E_FL_BmN-_04C24_F_0	BJ981058
E_FL_BmN-_04C24_R_0	BJ987299
E_FL_BmN-_04D01_F_0	BJ981059
E_FL_BmN-_04D03_F_0	BJ981060
E_FL_BmN-_04D03_R_0	BJ987300
E_FL_BmN-_04D04_F_0	BJ981061
E_FL_BmN-_04D05_F_0	BJ981062
E_FL_BmN-_04D06_F_0	BJ981063
E_FL_BmN-_04D06_R_0	BJ987301
E_FL_BmN-_04D07_F_0	BJ981064
E_FL_BmN-_04D07_R_0	BJ987302
E_FL_BmN-_04D08_F_0	BJ981065
E_FL_BmN-_04D08_R_0	BJ987303
E_FL_BmN-_04D09_F_0	BJ981066
E_FL_BmN-_04D09_R_0	BJ987304
E_FL_BmN-_04D10_F_0	BJ981067
E_FL_BmN-_04D10_R_0	BJ987305
E_FL_BmN-_04D11_F_0	BJ981068
E_FL_BmN-_04D11_R_0	BJ987306

E_FL_BmN-_04D12_F_0	BJ981069
E_FL_BmN-_04D12_R_0	BJ987307
E_FL_BmN-_04D13_F_0	BJ981070
E_FL_BmN-_04D13_R_0	BJ987308
E_FL_BmN-_04D14_F_0	BJ981071
E_FL_BmN-_04D15_F_0	BJ981072
E_FL_BmN-_04D15_R_0	BJ987309
E_FL_BmN-_04D16_F_0	BJ981073
E_FL_BmN-_04D16_R_0	BJ987310
E_FL_BmN-_04D17_F_0	BJ981074
E_FL_BmN-_04D17_R_0	BJ987311
E_FL_BmN-_04D18_F_0	BJ981075
E_FL_BmN-_04D19_F_0	BJ981076
E_FL_BmN-_04D19_R_0	BJ987312
E_FL_BmN-_04D20_F_0	BJ981077
E_FL_BmN-_04D20_R_0	BJ987313
E_FL_BmN-_04D21_F_0	BJ981078
E_FL_BmN-_04D21_R_0	BJ987314
E_FL_BmN-_04D22_F_0	BJ981079
E_FL_BmN-_04D22_R_0	BJ987315
E_FL_BmN-_04D23_F_0	BJ981080
E_FL_BmN-_04D24_F_0	BJ981081
E_FL_BmN-_04D24_R_0	BJ987316
E_FL_BmN-_04E01_F_0	BJ981082
E_FL_BmN-_04E02_F_0	BJ981083
E_FL_BmN-_04E02_R_0	BJ987317
E_FL_BmN-_04E03_F_0	BJ981084
E_FL_BmN-_04E04_F_0	BJ981085
E_FL_BmN-_04E04_R_0	BJ987318
E_FL_BmN-_04E05_F_0	BJ981086
E_FL_BmN-_04E05_R_0	BJ987319
E_FL_BmN-_04E06_F_0	BJ981087
E_FL_BmN-_04E06_R_0	BJ987320
E_FL_BmN-_04E07_F_0	BJ981088
E_FL_BmN-_04E07_R_0	BJ987321
E_FL_BmN-_04E08_F_0	BJ981089
E_FL_BmN-_04E08_R_0	BJ987322
E_FL_BmN-_04E09_F_0	BJ981090
E_FL_BmN-_04E09_R_0	BJ987323
E_FL_BmN-_04E10_F_0	BJ981091
E_FL_BmN-_04E10_R_0	BJ987324
E_FL_BmN-_04E11_F_0	BJ981092
E_FL_BmN-_04E11_R_0	BJ987325
E_FL_BmN-_04E12_F_0	BJ981093
E_FL_BmN-_04E12_R_0	BJ987326
E_FL_BmN-_04E13_F_0	BJ981094
E_FL_BmN-_04E13_R_0	BJ987327
E_FL_BmN-_04E14_F_0	BJ981095
E_FL_BmN-_04E14_R_0	BJ987328
E_FL_BmN-_04E15_F_0	BJ981096
E_FL_BmN-_04E15_R_0	BJ987329
E_FL_BmN-_04E16_F_0	BJ981097
E_FL_BmN-_04E16_R_0	BJ987330
E_FL_BmN-_04E17_F_0	BJ981098

E_FL_BmN-_04E17_R_0	BJ987331
E_FL_BmN-_04E18_F_0	BJ981099
E_FL_BmN-_04E20_F_0	BJ981100
E_FL_BmN-_04E20_R_0	BJ987332
E_FL_BmN-_04E21_F_0	BJ981101
E_FL_BmN-_04E21_R_0	BJ987333
E_FL_BmN-_04E22_F_0	BJ981102
E_FL_BmN-_04E22_R_0	BJ987334
E_FL_BmN-_04E23_F_0	BJ981103
E_FL_BmN-_04E23_R_0	BJ987335
E_FL_BmN-_04E24_F_0	BJ981104
E_FL_BmN-_04E24_R_0	BJ987336
E_FL_BmN-_04F01_F_0	BJ981105
E_FL_BmN-_04F02_F_0	BJ981106
E_FL_BmN-_04F02_R_0	BJ987337
E_FL_BmN-_04F03_F_0	BJ981107
E_FL_BmN-_04F03_R_0	BJ987338
E_FL_BmN-_04F04_F_0	BJ981108
E_FL_BmN-_04F04_R_0	BJ987339
E_FL_BmN-_04F05_F_0	BJ981109
E_FL_BmN-_04F05_R_0	BJ987340
E_FL_BmN-_04F07_F_0	BJ981110
E_FL_BmN-_04F08_F_0	BJ981111
E_FL_BmN-_04F08_R_0	BJ987341
E_FL_BmN-_04F09_F_0	BJ981112
E_FL_BmN-_04F09_R_0	BJ987342
E_FL_BmN-_04F10_F_0	BJ981113
E_FL_BmN-_04F10_R_0	BJ987343
E_FL_BmN-_04F11_F_0	BJ981114
E_FL_BmN-_04F11_R_0	BJ987344
E_FL_BmN-_04F12_F_0	BJ981115
E_FL_BmN-_04F12_R_0	BJ987345
E_FL_BmN-_04F13_F_0	BJ981116
E_FL_BmN-_04F15_F_0	BJ981117
E_FL_BmN-_04F15_R_0	BJ987346
E_FL_BmN-_04F16_F_0	BJ981118
E_FL_BmN-_04F16_R_0	BJ987347
E_FL_BmN-_04F17_F_0	BJ981119
E_FL_BmN-_04F17_R_0	BJ987348
E_FL_BmN-_04F19_F_0	BJ981120
E_FL_BmN-_04F20_F_0	BJ981121
E_FL_BmN-_04F21_F_0	BJ981122
E_FL_BmN-_04F22_F_0	BJ981123
E_FL_BmN-_04F22_R_0	BJ987349
E_FL_BmN-_04F23_F_0	BJ981124
E_FL_BmN-_04F23_R_0	BJ987350
E_FL_BmN-_04F24_F_0	BJ981125
E_FL_BmN-_04G01_F_0	BJ981126
E_FL_BmN-_04G01_R_0	BJ987351
E_FL_BmN-_04G02_F_0	BJ981127
E_FL_BmN-_04G02_R_0	BJ987352
E_FL_BmN-_04G03_F_0	BJ981128
E_FL_BmN-_04G03_R_0	BJ987353
E_FL_BmN-_04G05_F_0	BJ981129

E_FL_BmN-_04G05_R_0	BJ987354
E_FL_BmN-_04G06_F_0	BJ981130
E_FL_BmN-_04G06_R_0	BJ987355
E_FL_BmN-_04G07_F_0	BJ981131
E_FL_BmN-_04G07_R_0	BJ987356
E_FL_BmN-_04G08_F_0	BJ981132
E_FL_BmN-_04G08_R_0	BJ987357
E_FL_BmN-_04G09_R_0	BJ987358
E_FL_BmN-_04G10_F_0	BJ981133
E_FL_BmN-_04G10_R_0	BJ987359
E_FL_BmN-_04G11_F_0	BJ981134
E_FL_BmN-_04G11_R_0	BJ987360
E_FL_BmN-_04G12_F_0	BJ981135
E_FL_BmN-_04G12_R_0	BJ987361
E_FL_BmN-_04G13_F_0	BJ981136
E_FL_BmN-_04G13_R_0	BJ987362
E_FL_BmN-_04G14_F_0	BJ981137
E_FL_BmN-_04G14_R_0	BJ987363
E_FL_BmN-_04G15_F_0	BJ981138
E_FL_BmN-_04G16_F_0	BJ981139
E_FL_BmN-_04G17_F_0	BJ981140
E_FL_BmN-_04G18_F_0	BJ981141
E_FL_BmN-_04G19_R_0	BJ987364
E_FL_BmN-_04G20_F_0	BJ981142
E_FL_BmN-_04G20_R_0	BJ987365
E_FL_BmN-_04G21_F_0	BJ981143
E_FL_BmN-_04G21_R_0	BJ987366
E_FL_BmN-_04G22_F_0	BJ981144
E_FL_BmN-_04G22_R_0	BJ987367
E_FL_BmN-_04G24_F_0	BJ981145
E_FL_BmN-_04H01_F_0	BJ981146
E_FL_BmN-_04H02_F_0	BJ981147
E_FL_BmN-_04H02_R_0	BJ987368
E_FL_BmN-_04H03_F_0	BJ981148
E_FL_BmN-_04H03_R_0	BJ987369
E_FL_BmN-_04H04_F_0	BJ981149
E_FL_BmN-_04H04_R_0	BJ987370
E_FL_BmN-_04H05_F_0	BJ981150
E_FL_BmN-_04H05_R_0	BJ987371
E_FL_BmN-_04H07_F_0	BJ981151
E_FL_BmN-_04H08_F_0	BJ981152
E_FL_BmN-_04H10_R_0	BJ987372
E_FL_BmN-_04H11_F_0	BJ981153
E_FL_BmN-_04H13_F_0	BJ981154
E_FL_BmN-_04H14_F_0	BJ981155
E_FL_BmN-_04H14_R_0	BJ987373
E_FL_BmN-_04H15_F_0	BJ981156
E_FL_BmN-_04H15_R_0	BJ987374
E_FL_BmN-_04H16_F_0	BJ981157
E_FL_BmN-_04H17_F_0	BJ981158
E_FL_BmN-_04H18_F_0	BJ981159
E_FL_BmN-_04H18_R_0	BJ987375
E_FL_BmN-_04H19_F_0	BJ981160
E_FL_BmN-_04H20_F_0	BJ981161

E_FL_BmN-_04H20_R_0	BJ987376
E_FL_BmN-_04H23_F_0	BJ981162
E_FL_BmN-_04H23_R_0	BJ987377
E_FL_BmN-_04H24_F_0	BJ981163
E_FL_BmN-_04H24_R_0	BJ987378
E_FL_BmN-_04I01_F_0	BJ981164
E_FL_BmN-_04I02_F_0	BJ981165
E_FL_BmN-_04I02_R_0	BJ987379
E_FL_BmN-_04I03_F_0	BJ981166
E_FL_BmN-_04I03_R_0	BJ987380
E_FL_BmN-_04I04_F_0	BJ981167
E_FL_BmN-_04I04_R_0	BJ987381
E_FL_BmN-_04I05_F_0	BJ981168
E_FL_BmN-_04I05_R_0	BJ987382
E_FL_BmN-_04I06_F_0	BJ981169
E_FL_BmN-_04I08_F_0	BJ981170
E_FL_BmN-_04I08_R_0	BJ987383
E_FL_BmN-_04I09_F_0	BJ981171
E_FL_BmN-_04I09_R_0	BJ987384
E_FL_BmN-_04I10_F_0	BJ981172
E_FL_BmN-_04I10_R_0	BJ987385
E_FL_BmN-_04I11_F_0	BJ981173
E_FL_BmN-_04I11_R_0	BJ987386
E_FL_BmN-_04I12_F_0	BJ981174
E_FL_BmN-_04I14_F_0	BJ981175
E_FL_BmN-_04I14_R_0	BJ987387
E_FL_BmN-_04I15_F_0	BJ981176
E_FL_BmN-_04I16_F_0	BJ981177
E_FL_BmN-_04I16_R_0	BJ987388
E_FL_BmN-_04I17_F_0	BJ981178
E_FL_BmN-_04I17_R_0	BJ987389
E_FL_BmN-_04I18_F_0	BJ981179
E_FL_BmN-_04I18_R_0	BJ987390
E_FL_BmN-_04I19_R_0	BJ987391
E_FL_BmN-_04I20_F_0	BJ981180
E_FL_BmN-_04I20_R_0	BJ987392
E_FL_BmN-_04I21_F_0	BJ981181
E_FL_BmN-_04I21_R_0	BJ987393
E_FL_BmN-_04I23_F_0	BJ981182
E_FL_BmN-_04I23_R_0	BJ987394
E_FL_BmN-_04I24_F_0	BJ981183
E_FL_BmN-_04I24_R_0	BJ987395
E_FL_BmN-_04J01_R_0	BJ987396
E_FL_BmN-_04J02_F_0	BJ981184
E_FL_BmN-_04J02_R_0	BJ987397
E_FL_BmN-_04J03_F_0	BJ981185
E_FL_BmN-_04J03_R_0	BJ987398
E_FL_BmN-_04J05_F_0	BJ981186
E_FL_BmN-_04J05_R_0	BJ987399
E_FL_BmN-_04J06_F_0	BJ981187
E_FL_BmN-_04J07_F_0	BJ981188
E_FL_BmN-_04J08_F_0	BJ981189
E_FL_BmN-_04J08_R_0	BJ987400
E_FL_BmN-_04J09_F_0	BJ981190

E_FL_BmN-_04J09_R_0	BJ987401
E_FL_BmN-_04J11_F_0	BJ981191
E_FL_BmN-_04J12_F_0	BJ981192
E_FL_BmN-_04J13_F_0	BJ981193
E_FL_BmN-_04J13_R_0	BJ987402
E_FL_BmN-_04J15_F_0	BJ981194
E_FL_BmN-_04J15_R_0	BJ987403
E_FL_BmN-_04J16_F_0	BJ981195
E_FL_BmN-_04J16_R_0	BJ987404
E_FL_BmN-_04J17_F_0	BJ981196
E_FL_BmN-_04J17_R_0	BJ987405
E_FL_BmN-_04J18_F_0	BJ981197
E_FL_BmN-_04J19_F_0	BJ981198
E_FL_BmN-_04J19_R_0	BJ987406
E_FL_BmN-_04J20_F_0	BJ981199
E_FL_BmN-_04J20_R_0	BJ987407
E_FL_BmN-_04J21_F_0	BJ981200
E_FL_BmN-_04J21_R_0	BJ987408
E_FL_BmN-_04J22_F_0	BJ981201
E_FL_BmN-_04J22_R_0	BJ987409
E_FL_BmN-_04J23_F_0	BJ981202
E_FL_BmN-_04J23_R_0	BJ987410
E_FL_BmN-_04J24_F_0	BJ981203
E_FL_BmN-_04J24_R_0	BJ987411
E_FL_BmN-_04K01_F_0	BJ981204
E_FL_BmN-_04K01_R_0	BJ987412
E_FL_BmN-_04K02_F_0	BJ981205
E_FL_BmN-_04K02_R_0	BJ987413
E_FL_BmN-_04K03_F_0	BJ981206
E_FL_BmN-_04K03_R_0	BJ987414
E_FL_BmN-_04K04_F_0	BJ981207
E_FL_BmN-_04K04_R_0	BJ987415
E_FL_BmN-_04K05_F_0	BJ981208
E_FL_BmN-_04K05_R_0	BJ987416
E_FL_BmN-_04K06_F_0	BJ981209
E_FL_BmN-_04K06_R_0	BJ987417
E_FL_BmN-_04K07_F_0	BJ981210
E_FL_BmN-_04K07_R_0	BJ987418
E_FL_BmN-_04K09_F_0	BJ981211
E_FL_BmN-_04K09_R_0	BJ987419
E_FL_BmN-_04K10_F_0	BJ981212
E_FL_BmN-_04K10_R_0	BJ987420
E_FL_BmN-_04K11_F_0	BJ981213
E_FL_BmN-_04K11_R_0	BJ987421
E_FL_BmN-_04K12_F_0	BJ981214
E_FL_BmN-_04K13_F_0	BJ981215
E_FL_BmN-_04K13_R_0	BJ987422
E_FL_BmN-_04K14_F_0	BJ981216
E_FL_BmN-_04K14_R_0	BJ987423
E_FL_BmN-_04K15_F_0	BJ981217
E_FL_BmN-_04K15_R_0	BJ987424
E_FL_BmN-_04K16_F_0	BJ981218
E_FL_BmN-_04K16_R_0	BJ987425
E_FL_BmN-_04K17_F_0	BJ981219

E_FL_BmN-_04K17_R_0	BJ987426
E_FL_BmN-_04K18_F_0	BJ981220
E_FL_BmN-_04K18_R_0	BJ987427
E_FL_BmN-_04K19_F_0	BJ981221
E_FL_BmN-_04K19_R_0	BJ987428
E_FL_BmN-_04K20_F_0	BJ981222
E_FL_BmN-_04K21_F_0	BJ981223
E_FL_BmN-_04K21_R_0	BJ987429
E_FL_BmN-_04K22_F_0	BJ981224
E_FL_BmN-_04K22_R_0	BJ987430
E_FL_BmN-_04K23_F_0	BJ981225
E_FL_BmN-_04K23_R_0	BJ987431
E_FL_BmN-_04K24_F_0	BJ981226
E_FL_BmN-_04K24_R_0	BJ987432
E_FL_BmN-_04L01_F_0	BJ981227
E_FL_BmN-_04L01_R_0	BJ987433
E_FL_BmN-_04L02_F_0	BJ981228
E_FL_BmN-_04L03_F_0	BJ981229
E_FL_BmN-_04L03_R_0	BJ987434
E_FL_BmN-_04L04_F_0	BJ981230
E_FL_BmN-_04L04_R_0	BJ987435
E_FL_BmN-_04L05_F_0	BJ981231
E_FL_BmN-_04L06_F_0	BJ981232
E_FL_BmN-_04L06_R_0	BJ987436
E_FL_BmN-_04L07_F_0	BJ981233
E_FL_BmN-_04L07_R_0	BJ987437
E_FL_BmN-_04L08_F_0	BJ981234
E_FL_BmN-_04L08_R_0	BJ987438
E_FL_BmN-_04L09_F_0	BJ981235
E_FL_BmN-_04L10_F_0	BJ981236
E_FL_BmN-_04L11_F_0	BJ981237
E_FL_BmN-_04L11_R_0	BJ987439
E_FL_BmN-_04L12_F_0	BJ981238
E_FL_BmN-_04L13_F_0	BJ981239
E_FL_BmN-_04L13_R_0	BJ987440
E_FL_BmN-_04L14_F_0	BJ981240
E_FL_BmN-_04L14_R_0	BJ987441
E_FL_BmN-_04L15_F_0	BJ981241
E_FL_BmN-_04L15_R_0	BJ987442
E_FL_BmN-_04L17_F_0	BJ981242
E_FL_BmN-_04L18_F_0	BJ981243
E_FL_BmN-_04L18_R_0	BJ987443
E_FL_BmN-_04L19_F_0	BJ981244
E_FL_BmN-_04L20_F_0	BJ981245
E_FL_BmN-_04L20_R_0	BJ987444
E_FL_BmN-_04L21_F_0	BJ981246
E_FL_BmN-_04L21_R_0	BJ987445
E_FL_BmN-_04L22_F_0	BJ981247
E_FL_BmN-_04L22_R_0	BJ987446
E_FL_BmN-_04L23_F_0	BJ981248
E_FL_BmN-_04L23_R_0	BJ987447
E_FL_BmN-_04L24_F_0	BJ981249
E_FL_BmN-_04L24_R_0	BJ987448
E_FL_BmN-_04M01_F_0	BJ981250

E_FL_BmN-_04M01_R_0	BJ987449
E_FL_BmN-_04M03_F_0	BJ981251
E_FL_BmN-_04M03_R_0	BJ987450
E_FL_BmN-_04M04_F_0	BJ981252
E_FL_BmN-_04M04_R_0	BJ987451
E_FL_BmN-_04M05_F_0	BJ981253
E_FL_BmN-_04M05_R_0	BJ987452
E_FL_BmN-_04M06_F_0	BJ981254
E_FL_BmN-_04M07_F_0	BJ981255
E_FL_BmN-_04M07_R_0	BJ987453
E_FL_BmN-_04M08_F_0	BJ981256
E_FL_BmN-_04M08_R_0	BJ987454
E_FL_BmN-_04M09_F_0	BJ981257
E_FL_BmN-_04M09_R_0	BJ987455
E_FL_BmN-_04M10_F_0	BJ981258
E_FL_BmN-_04M10_R_0	BJ987456
E_FL_BmN-_04M11_F_0	BJ981259
E_FL_BmN-_04M11_R_0	BJ987457
E_FL_BmN-_04M12_F_0	BJ981260
E_FL_BmN-_04M12_R_0	BJ987458
E_FL_BmN-_04M13_R_0	BJ987459
E_FL_BmN-_04M14_F_0	BJ981261
E_FL_BmN-_04M14_R_0	BJ987460
E_FL_BmN-_04M15_F_0	BJ981262
E_FL_BmN-_04M15_R_0	BJ987461
E_FL_BmN-_04M16_F_0	BJ981263
E_FL_BmN-_04M16_R_0	BJ987462
E_FL_BmN-_04M17_F_0	BJ981264
E_FL_BmN-_04M17_R_0	BJ987463
E_FL_BmN-_04M18_F_0	BJ981265
E_FL_BmN-_04M18_R_0	BJ987464
E_FL_BmN-_04M19_F_0	BJ981266
E_FL_BmN-_04M19_R_0	BJ987465
E_FL_BmN-_04M20_F_0	BJ981267
E_FL_BmN-_04M21_F_0	BJ981268
E_FL_BmN-_04M22_F_0	BJ981269
E_FL_BmN-_04M22_R_0	BJ987466
E_FL_BmN-_04M23_F_0	BJ981270
E_FL_BmN-_04M24_F_0	BJ981271
E_FL_BmN-_04M24_R_0	BJ987467
E_FL_BmN-_04N01_F_0	BJ981272
E_FL_BmN-_04N01_R_0	BJ987468
E_FL_BmN-_04N02_F_0	BJ981273
E_FL_BmN-_04N03_F_0	BJ981274
E_FL_BmN-_04N03_R_0	BJ987469
E_FL_BmN-_04N04_F_0	BJ981275
E_FL_BmN-_04N05_F_0	BJ981276
E_FL_BmN-_04N05_R_0	BJ987470
E_FL_BmN-_04N06_F_0	BJ981277
E_FL_BmN-_04N06_R_0	BJ987471
E_FL_BmN-_04N07_F_0	BJ981278
E_FL_BmN-_04N07_R_0	BJ987472
E_FL_BmN-_04N08_F_0	BJ981279
E_FL_BmN-_04N08_R_0	BJ987473

E_FL_BmN-_04N09_F_0	BJ981280
E_FL_BmN-_04N10_F_0	BJ981281
E_FL_BmN-_04N12_F_0	BJ981282
E_FL_BmN-_04N12_R_0	BJ987474
E_FL_BmN-_04N13_F_0	BJ981283
E_FL_BmN-_04N14_F_0	BJ981284
E_FL_BmN-_04N14_R_0	BJ987475
E_FL_BmN-_04N15_F_0	BJ981285
E_FL_BmN-_04N15_R_0	BJ987476
E_FL_BmN-_04N16_F_0	BJ981286
E_FL_BmN-_04N17_F_0	BJ981287
E_FL_BmN-_04N17_R_0	BJ987477
E_FL_BmN-_04N18_F_0	BJ981288
E_FL_BmN-_04N19_F_0	BJ981289
E_FL_BmN-_04N19_R_0	BJ987478
E_FL_BmN-_04N20_F_0	BJ981290
E_FL_BmN-_04N21_F_0	BJ981291
E_FL_BmN-_04N21_R_0	BJ987479
E_FL_BmN-_04N22_F_0	BJ981292
E_FL_BmN-_04N22_R_0	BJ987480
E_FL_BmN-_04N23_F_0	BJ981293
E_FL_BmN-_04N23_R_0	BJ987481
E_FL_BmN-_04N24_F_0	BJ981294
E_FL_BmN-_04N24_R_0	BJ987482
E_FL_BmN-_04O01_F_0	BJ981295
E_FL_BmN-_04O01_R_0	BJ987483
E_FL_BmN-_04O02_F_0	BJ981296
E_FL_BmN-_04O02_R_0	BJ987484
E_FL_BmN-_04O03_F_0	BJ981297
E_FL_BmN-_04O03_R_0	BJ987485
E_FL_BmN-_04O04_F_0	BJ981298
E_FL_BmN-_04O04_R_0	BJ987486
E_FL_BmN-_04O05_F_0	BJ981299
E_FL_BmN-_04O06_F_0	BJ981300
E_FL_BmN-_04O06_R_0	BJ987487
E_FL_BmN-_04O07_F_0	BJ981301
E_FL_BmN-_04O07_R_0	BJ987488
E_FL_BmN-_04O08_F_0	BJ981302
E_FL_BmN-_04O09_F_0	BJ981303
E_FL_BmN-_04O09_R_0	BJ987489
E_FL_BmN-_04O10_F_0	BJ981304
E_FL_BmN-_04O10_R_0	BJ987490
E_FL_BmN-_04O12_F_0	BJ981305
E_FL_BmN-_04O12_R_0	BJ987491
E_FL_BmN-_04O13_F_0	BJ981306
E_FL_BmN-_04O14_F_0	BJ981307
E_FL_BmN-_04O14_R_0	BJ987492
E_FL_BmN-_04O15_F_0	BJ981308
E_FL_BmN-_04O15_R_0	BJ987493
E_FL_BmN-_04O16_F_0	BJ981309
E_FL_BmN-_04O16_R_0	BJ987494
E_FL_BmN-_04O17_F_0	BJ981310
E_FL_BmN-_04O17_R_0	BJ987495
E_FL_BmN-_04O18_F_0	BJ981311

E_FL_BmN-_04O18_R_0	BJ987496
E_FL_BmN-_04O19_F_0	BJ981312
E_FL_BmN-_04O19_R_0	BJ987497
E_FL_BmN-_04O20_F_0	BJ981313
E_FL_BmN-_04O20_R_0	BJ987498
E_FL_BmN-_04O21_F_0	BJ981314
E_FL_BmN-_04O21_R_0	BJ987499
E_FL_BmN-_04O22_F_0	BJ981315
E_FL_BmN-_04O22_R_0	BJ987500
E_FL_BmN-_04O24_F_0	BJ981316
E_FL_BmN-_04P01_F_0	BJ981317
E_FL_BmN-_04P01_R_0	BJ987501
E_FL_BmN-_04P03_F_0	BJ981318
E_FL_BmN-_04P03_R_0	BJ987502
E_FL_BmN-_04P04_F_0	BJ981319
E_FL_BmN-_04P04_R_0	BJ987503
E_FL_BmN-_04P05_F_0	BJ981320
E_FL_BmN-_04P05_R_0	BJ987504
E_FL_BmN-_04P07_F_0	BJ981321
E_FL_BmN-_04P07_R_0	BJ987505
E_FL_BmN-_04P09_F_0	BJ981322
E_FL_BmN-_04P09_R_0	BJ987506
E_FL_BmN-_04P10_F_0	BJ981323
E_FL_BmN-_04P10_R_0	BJ987507
E_FL_BmN-_04P11_F_0	BJ981324
E_FL_BmN-_04P12_F_0	BJ981325
E_FL_BmN-_04P12_R_0	BJ987508
E_FL_BmN-_04P13_F_0	BJ981326
E_FL_BmN-_04P13_R_0	BJ987509
E_FL_BmN-_04P14_F_0	BJ981327
E_FL_BmN-_04P14_R_0	BJ987510
E_FL_BmN-_04P15_F_0	BJ981328
E_FL_BmN-_04P16_F_0	BJ981329
E_FL_BmN-_04P16_R_0	BJ987511
E_FL_BmN-_04P17_F_0	BJ981330
E_FL_BmN-_04P18_F_0	BJ981331
E_FL_BmN-_04P18_R_0	BJ987512
E_FL_BmN-_04P19_F_0	BJ981332
E_FL_BmN-_04P19_R_0	BJ987513
E_FL_BmN-_04P20_F_0	BJ981333
E_FL_BmN-_04P20_R_0	BJ987514
E_FL_BmN-_04P21_F_0	BJ981334
E_FL_BmN-_04P21_R_0	BJ987515
E_FL_BmN-_04P22_F_0	BJ981335
E_FL_BmN-_04P22_R_0	BJ987516
E_FL_BmN-_04P23_F_0	BJ981336
E_FL_BmN-_04P23_R_0	BJ987517
E_FL_BmN-_04P24_F_0	BJ981337
E_FL_BmN-_04P24_R_0	BJ987518
E_FL_BmN-_05A02_F_0	BJ981338
E_FL_BmN-_05A02_R_0	BJ987519
E_FL_BmN-_05A03_F_0	BJ981339
E_FL_BmN-_05A03_R_0	BJ987520
E_FL_BmN-_05A04_F_0	BJ981340

E_FL_BmN-_05A04_R_0	BJ987521
E_FL_BmN-_05A05_F_0	BJ981341
E_FL_BmN-_05A05_R_0	BJ987522
E_FL_BmN-_05A06_F_0	BJ981342
E_FL_BmN-_05A06_R_0	BJ987523
E_FL_BmN-_05A07_F_0	BJ981343
E_FL_BmN-_05A07_R_0	BJ987524
E_FL_BmN-_05A08_F_0	BJ981344
E_FL_BmN-_05A08_R_0	BJ987525
E_FL_BmN-_05A09_F_0	BJ981345
E_FL_BmN-_05A10_F_0	BJ981346
E_FL_BmN-_05A10_R_0	BJ987526
E_FL_BmN-_05A11_F_0	BJ981347
E_FL_BmN-_05A11_R_0	BJ987527
E_FL_BmN-_05A12_F_0	BJ981348
E_FL_BmN-_05A12_R_0	BJ987528
E_FL_BmN-_05A13_F_0	BJ981349
E_FL_BmN-_05A13_R_0	BJ987529
E_FL_BmN-_05A15_F_0	BJ981350
E_FL_BmN-_05A15_R_0	BJ987530
E_FL_BmN-_05A16_F_0	BJ981351
E_FL_BmN-_05A17_F_0	BJ981352
E_FL_BmN-_05A17_R_0	BJ987531
E_FL_BmN-_05A18_F_0	BJ981353
E_FL_BmN-_05A18_R_0	BJ987532
E_FL_BmN-_05A19_F_0	BJ981354
E_FL_BmN-_05A20_F_0	BJ981355
E_FL_BmN-_05A21_F_0	BJ981356
E_FL_BmN-_05A21_R_0	BJ987533
E_FL_BmN-_05A22_F_0	BJ981357
E_FL_BmN-_05A22_R_0	BJ987534
E_FL_BmN-_05A23_F_0	BJ981358
E_FL_BmN-_05A23_R_0	BJ987535
E_FL_BmN-_05A24_F_0	BJ981359
E_FL_BmN-_05A24_R_0	BJ987536
E_FL_BmN-_05B01_F_0	BJ981360
E_FL_BmN-_05B01_R_0	BJ987537
E_FL_BmN-_05B03_F_0	BJ981361
E_FL_BmN-_05B03_R_0	BJ987538
E_FL_BmN-_05B04_F_0	BJ981362
E_FL_BmN-_05B04_R_0	BJ987539
E_FL_BmN-_05B05_F_0	BJ981363
E_FL_BmN-_05B05_R_0	BJ987540
E_FL_BmN-_05B06_F_0	BJ981364
E_FL_BmN-_05B06_R_0	BJ987541
E_FL_BmN-_05B07_F_0	BJ981365
E_FL_BmN-_05B07_R_0	BJ987542
E_FL_BmN-_05B08_F_0	BJ981366
E_FL_BmN-_05B08_R_0	BJ987543
E_FL_BmN-_05B09_R_0	BJ987544
E_FL_BmN-_05B10_F_0	BJ981367
E_FL_BmN-_05B10_R_0	BJ987545
E_FL_BmN-_05B11_F_0	BJ981368
E_FL_BmN-_05B11_R_0	BJ987546

E_FL_BmN-_05B12_F_0	BJ981369
E_FL_BmN-_05B12_R_0	BJ987547
E_FL_BmN-_05B13_F_0	BJ981370
E_FL_BmN-_05B13_R_0	BJ987548
E_FL_BmN-_05B14_F_0	BJ981371
E_FL_BmN-_05B14_R_0	BJ987549
E_FL_BmN-_05B15_F_0	BJ981372
E_FL_BmN-_05B15_R_0	BJ987550
E_FL_BmN-_05B16_F_0	BJ981373
E_FL_BmN-_05B16_R_0	BJ987551
E_FL_BmN-_05B17_F_0	BJ981374
E_FL_BmN-_05B17_R_0	BJ987552
E_FL_BmN-_05B19_F_0	BJ981375
E_FL_BmN-_05B19_R_0	BJ987553
E_FL_BmN-_05B20_F_0	BJ981376
E_FL_BmN-_05B20_R_0	BJ987554
E_FL_BmN-_05B21_F_0	BJ981377
E_FL_BmN-_05B21_R_0	BJ987555
E_FL_BmN-_05B23_F_0	BJ981378
E_FL_BmN-_05B23_R_0	BJ987556
E_FL_BmN-_05B24_F_0	BJ981379
E_FL_BmN-_05B24_R_0	BJ987557
E_FL_BmN-_05C01_F_0	BJ981380
E_FL_BmN-_05C02_F_0	BJ981381
E_FL_BmN-_05C02_R_0	BJ987558
E_FL_BmN-_05C03_F_0	BJ981382
E_FL_BmN-_05C03_R_0	BJ987559
E_FL_BmN-_05C04_F_0	BJ981383
E_FL_BmN-_05C05_F_0	BJ981384
E_FL_BmN-_05C05_R_0	BJ987560
E_FL_BmN-_05C06_R_0	BJ987561
E_FL_BmN-_05C07_F_0	BJ981385
E_FL_BmN-_05C08_F_0	BJ981386
E_FL_BmN-_05C08_R_0	BJ987562
E_FL_BmN-_05C09_F_0	BJ981387
E_FL_BmN-_05C09_R_0	BJ987563
E_FL_BmN-_05C10_F_0	BJ981388
E_FL_BmN-_05C10_R_0	BJ987564
E_FL_BmN-_05C11_F_0	BJ981389
E_FL_BmN-_05C11_R_0	BJ987565
E_FL_BmN-_05C12_R_0	BJ987566
E_FL_BmN-_05C13_F_0	BJ981390
E_FL_BmN-_05C14_F_0	BJ981391
E_FL_BmN-_05C15_F_0	BJ981392
E_FL_BmN-_05C16_F_0	BJ981393
E_FL_BmN-_05C16_R_0	BJ987567
E_FL_BmN-_05C17_F_0	BJ981394
E_FL_BmN-_05C17_R_0	BJ987568
E_FL_BmN-_05C18_R_0	BJ987569
E_FL_BmN-_05C19_F_0	BJ981395
E_FL_BmN-_05C20_F_0	BJ981396
E_FL_BmN-_05C21_F_0	BJ981397
E_FL_BmN-_05C21_R_0	BJ987570
E_FL_BmN-_05C22_R_0	BJ987571

E_FL_BmN-_05C23_F_0	BJ981398
E_FL_BmN-_05C23_R_0	BJ987572
E_FL_BmN-_05C24_R_0	BJ987573
E_FL_BmN-_05D01_F_0	BJ981399
E_FL_BmN-_05D01_R_0	BJ987574
E_FL_BmN-_05D02_F_0	BJ981400
E_FL_BmN-_05D02_R_0	BJ987575
E_FL_BmN-_05D03_F_0	BJ981401
E_FL_BmN-_05D03_R_0	BJ987576
E_FL_BmN-_05D04_F_0	BJ981402
E_FL_BmN-_05D04_R_0	BJ987577
E_FL_BmN-_05D05_F_0	BJ981403
E_FL_BmN-_05D05_R_0	BJ987578
E_FL_BmN-_05D06_R_0	BJ987579
E_FL_BmN-_05D07_F_0	BJ981404
E_FL_BmN-_05D07_R_0	BJ987580
E_FL_BmN-_05D08_F_0	BJ981405
E_FL_BmN-_05D09_F_0	BJ981406
E_FL_BmN-_05D09_R_0	BJ987581
E_FL_BmN-_05D10_F_0	BJ981407
E_FL_BmN-_05D10_R_0	BJ987582
E_FL_BmN-_05D11_F_0	BJ981408
E_FL_BmN-_05D11_R_0	BJ987583
E_FL_BmN-_05D12_F_0	BJ981409
E_FL_BmN-_05D12_R_0	BJ987584
E_FL_BmN-_05D13_F_0	BJ981410
E_FL_BmN-_05D13_R_0	BJ987585
E_FL_BmN-_05D14_R_0	BJ987586
E_FL_BmN-_05D15_F_0	BJ981411
E_FL_BmN-_05D15_R_0	BJ987587
E_FL_BmN-_05D16_F_0	BJ981412
E_FL_BmN-_05D16_R_0	BJ987588
E_FL_BmN-_05D17_F_0	BJ981413
E_FL_BmN-_05D17_R_0	BJ987589
E_FL_BmN-_05D18_R_0	BJ987590
E_FL_BmN-_05D19_F_0	BJ981414
E_FL_BmN-_05D19_R_0	BJ987591
E_FL_BmN-_05D20_R_0	BJ987592
E_FL_BmN-_05D21_R_0	BJ987593
E_FL_BmN-_05D22_F_0	BJ981415
E_FL_BmN-_05D22_R_0	BJ987594
E_FL_BmN-_05D23_F_0	BJ981416
E_FL_BmN-_05D23_R_0	BJ987595
E_FL_BmN-_05D24_F_0	BJ981417
E_FL_BmN-_05D24_R_0	BJ987596
E_FL_BmN-_05E01_F_0	BJ981418
E_FL_BmN-_05E01_R_0	BJ987597
E_FL_BmN-_05E02_F_0	BJ981419
E_FL_BmN-_05E02_R_0	BJ987598
E_FL_BmN-_05E03_F_0	BJ981420
E_FL_BmN-_05E03_R_0	BJ987599
E_FL_BmN-_05E04_F_0	BJ981421
E_FL_BmN-_05E05_F_0	BJ981422
E_FL_BmN-_05E05_R_0	BJ987600

E_FL_BmN-_05E06_F_0	BJ981423
E_FL_BmN-_05E06_R_0	BJ987601
E_FL_BmN-_05E07_F_0	BJ981424
E_FL_BmN-_05E08_R_0	BJ987602
E_FL_BmN-_05E09_F_0	BJ981425
E_FL_BmN-_05E10_F_0	BJ981426
E_FL_BmN-_05E11_F_0	BJ981427
E_FL_BmN-_05E11_R_0	BJ987603
E_FL_BmN-_05E12_F_0	BJ981428
E_FL_BmN-_05E12_R_0	BJ987604
E_FL_BmN-_05E13_F_0	BJ981429
E_FL_BmN-_05E14_F_0	BJ981430
E_FL_BmN-_05E14_R_0	BJ987605
E_FL_BmN-_05E15_F_0	BJ981431
E_FL_BmN-_05E15_R_0	BJ987606
E_FL_BmN-_05E16_F_0	BJ981432
E_FL_BmN-_05E16_R_0	BJ987607
E_FL_BmN-_05E17_F_0	BJ981433
E_FL_BmN-_05E18_F_0	BJ981434
E_FL_BmN-_05E19_F_0	BJ981435
E_FL_BmN-_05E19_R_0	BJ987608
E_FL_BmN-_05E20_F_0	BJ981436
E_FL_BmN-_05E20_R_0	BJ987609
E_FL_BmN-_05E21_F_0	BJ981437
E_FL_BmN-_05E21_R_0	BJ987610
E_FL_BmN-_05E22_F_0	BJ981438
E_FL_BmN-_05E22_R_0	BJ987611
E_FL_BmN-_05E23_F_0	BJ981439
E_FL_BmN-_05E23_R_0	BJ987612
E_FL_BmN-_05E24_F_0	BJ981440
E_FL_BmN-_05E24_R_0	BJ987613
E_FL_BmN-_05F01_F_0	BJ981441
E_FL_BmN-_05F01_R_0	BJ987614
E_FL_BmN-_05F02_R_0	BJ987615
E_FL_BmN-_05F03_R_0	BJ987616
E_FL_BmN-_05F04_F_0	BJ981442
E_FL_BmN-_05F04_R_0	BJ987617
E_FL_BmN-_05F05_F_0	BJ981443
E_FL_BmN-_05F05_R_0	BJ987618
E_FL_BmN-_05F06_F_0	BJ981444
E_FL_BmN-_05F06_R_0	BJ987619
E_FL_BmN-_05F07_F_0	BJ981445
E_FL_BmN-_05F07_R_0	BJ987620
E_FL_BmN-_05F08_F_0	BJ981446
E_FL_BmN-_05F08_R_0	BJ987621
E_FL_BmN-_05F09_R_0	BJ987622
E_FL_BmN-_05F10_F_0	BJ981447
E_FL_BmN-_05F10_R_0	BJ987623
E_FL_BmN-_05F11_F_0	BJ981448
E_FL_BmN-_05F11_R_0	BJ987624
E_FL_BmN-_05F12_F_0	BJ981449
E_FL_BmN-_05F12_R_0	BJ987625
E_FL_BmN-_05F13_F_0	BJ981450
E_FL_BmN-_05F13_R_0	BJ987626

E_FL_BmN-_05F14_F_0	BJ981451
E_FL_BmN-_05F14_R_0	BJ987627
E_FL_BmN-_05F15_F_0	BJ981452
E_FL_BmN-_05F15_R_0	BJ987628
E_FL_BmN-_05F16_F_0	BJ981453
E_FL_BmN-_05F16_R_0	BJ987629
E_FL_BmN-_05F17_F_0	BJ981454
E_FL_BmN-_05F17_R_0	BJ987630
E_FL_BmN-_05F18_F_0	BJ981455
E_FL_BmN-_05F18_R_0	BJ987631
E_FL_BmN-_05F19_F_0	BJ981456
E_FL_BmN-_05F19_R_0	BJ987632
E_FL_BmN-_05F20_R_0	BJ987633
E_FL_BmN-_05F22_F_0	BJ981457
E_FL_BmN-_05F22_R_0	BJ987634
E_FL_BmN-_05F23_F_0	BJ981458
E_FL_BmN-_05F23_R_0	BJ987635
E_FL_BmN-_05F24_F_0	BJ981459
E_FL_BmN-_05F24_R_0	BJ987636
E_FL_BmN-_05G01_F_0	BJ981460
E_FL_BmN-_05G01_R_0	BJ987637
E_FL_BmN-_05G02_F_0	BJ981461
E_FL_BmN-_05G02_R_0	BJ987638
E_FL_BmN-_05G03_F_0	BJ981462
E_FL_BmN-_05G03_R_0	BJ987639
E_FL_BmN-_05G04_F_0	BJ981463
E_FL_BmN-_05G04_R_0	BJ987640
E_FL_BmN-_05G05_F_0	BJ981464
E_FL_BmN-_05G05_R_0	BJ987641
E_FL_BmN-_05G06_F_0	BJ981465
E_FL_BmN-_05G06_R_0	BJ987642
E_FL_BmN-_05G07_F_0	BJ981466
E_FL_BmN-_05G08_R_0	BJ987643
E_FL_BmN-_05G09_F_0	BJ981467
E_FL_BmN-_05G09_R_0	BJ987644
E_FL_BmN-_05G10_F_0	BJ981468
E_FL_BmN-_05G10_R_0	BJ987645
E_FL_BmN-_05G11_F_0	BJ981469
E_FL_BmN-_05G11_R_0	BJ987646
E_FL_BmN-_05G12_F_0	BJ981470
E_FL_BmN-_05G13_F_0	BJ981471
E_FL_BmN-_05G14_F_0	BJ981472
E_FL_BmN-_05G14_R_0	BJ987647
E_FL_BmN-_05G15_F_0	BJ981473
E_FL_BmN-_05G15_R_0	BJ987648
E_FL_BmN-_05G16_F_0	BJ981474
E_FL_BmN-_05G16_R_0	BJ987649
E_FL_BmN-_05G17_F_0	BJ981475
E_FL_BmN-_05G17_R_0	BJ987650
E_FL_BmN-_05G19_F_0	BJ981476
E_FL_BmN-_05G19_R_0	BJ987651
E_FL_BmN-_05G20_F_0	BJ981477
E_FL_BmN-_05G20_R_0	BJ987652
E_FL_BmN-_05G21_F_0	BJ981478

E_FL_BmN-_05G21_R_0	BJ987653
E_FL_BmN-_05G22_F_0	BJ981479
E_FL_BmN-_05G22_R_0	BJ987654
E_FL_BmN-_05G23_F_0	BJ981480
E_FL_BmN-_05G23_R_0	BJ987655
E_FL_BmN-_05G24_F_0	BJ981481
E_FL_BmN-_05G24_R_0	BJ987656
E_FL_BmN-_05H01_F_0	BJ981482
E_FL_BmN-_05H01_R_0	BJ987657
E_FL_BmN-_05H02_F_0	BJ981483
E_FL_BmN-_05H02_R_0	BJ987658
E_FL_BmN-_05H03_F_0	BJ981484
E_FL_BmN-_05H03_R_0	BJ987659
E_FL_BmN-_05H04_F_0	BJ981485
E_FL_BmN-_05H04_R_0	BJ987660
E_FL_BmN-_05H05_F_0	BJ981486
E_FL_BmN-_05H05_R_0	BJ987661
E_FL_BmN-_05H06_F_0	BJ981487
E_FL_BmN-_05H06_R_0	BJ987662
E_FL_BmN-_05H07_F_0	BJ981488
E_FL_BmN-_05H07_R_0	BJ987663
E_FL_BmN-_05H08_F_0	BJ981489
E_FL_BmN-_05H08_R_0	BJ987664
E_FL_BmN-_05H09_F_0	BJ981490
E_FL_BmN-_05H09_R_0	BJ987665
E_FL_BmN-_05H10_R_0	BJ987666
E_FL_BmN-_05H11_F_0	BJ981491
E_FL_BmN-_05H11_R_0	BJ987667
E_FL_BmN-_05H12_R_0	BJ987668
E_FL_BmN-_05H13_F_0	BJ981492
E_FL_BmN-_05H13_R_0	BJ987669
E_FL_BmN-_05H14_F_0	BJ981493
E_FL_BmN-_05H14_R_0	BJ987670
E_FL_BmN-_05H16_F_0	BJ981494
E_FL_BmN-_05H16_R_0	BJ987671
E_FL_BmN-_05H17_F_0	BJ981495
E_FL_BmN-_05H17_R_0	BJ987672
E_FL_BmN-_05H19_F_0	BJ981496
E_FL_BmN-_05H19_R_0	BJ987673
E_FL_BmN-_05H20_F_0	BJ981497
E_FL_BmN-_05H20_R_0	BJ987674
E_FL_BmN-_05H21_F_0	BJ981498
E_FL_BmN-_05H21_R_0	BJ987675
E_FL_BmN-_05H22_F_0	BJ981499
E_FL_BmN-_05H22_R_0	BJ987676
E_FL_BmN-_05H23_F_0	BJ981500
E_FL_BmN-_05H23_R_0	BJ987677
E_FL_BmN-_05H24_F_0	BJ981501
E_FL_BmN-_05H24_R_0	BJ987678
E_FL_BmN-_05I02_F_0	BJ981502
E_FL_BmN-_05I02_R_0	BJ987679
E_FL_BmN-_05I03_F_0	BJ981503
E_FL_BmN-_05I03_R_0	BJ987680
E_FL_BmN-_05I04_F_0	BJ981504

E_FL_BmN-_05I04_R_0	BJ987681
E_FL_BmN-_05I05_F_0	BJ981505
E_FL_BmN-_05I05_R_0	BJ987682
E_FL_BmN-_05I06_F_0	BJ981506
E_FL_BmN-_05I06_R_0	BJ987683
E_FL_BmN-_05I07_F_0	BJ981507
E_FL_BmN-_05I08_F_0	BJ981508
E_FL_BmN-_05I08_R_0	BJ987684
E_FL_BmN-_05I09_F_0	BJ981509
E_FL_BmN-_05I09_R_0	BJ987685
E_FL_BmN-_05I10_F_0	BJ981510
E_FL_BmN-_05I11_F_0	BJ981511
E_FL_BmN-_05I11_R_0	BJ987686
E_FL_BmN-_05I12_F_0	BJ981512
E_FL_BmN-_05I12_R_0	BJ987687
E_FL_BmN-_05I13_R_0	BJ987688
E_FL_BmN-_05I14_F_0	BJ981513
E_FL_BmN-_05I14_R_0	BJ987689
E_FL_BmN-_05I15_F_0	BJ981514
E_FL_BmN-_05I15_R_0	BJ987690
E_FL_BmN-_05I16_F_0	BJ981515
E_FL_BmN-_05I16_R_0	BJ987691
E_FL_BmN-_05I17_F_0	BJ981516
E_FL_BmN-_05I18_F_0	BJ981517
E_FL_BmN-_05I18_R_0	BJ987692
E_FL_BmN-_05I19_F_0	BJ981518
E_FL_BmN-_05I20_F_0	BJ981519
E_FL_BmN-_05I20_R_0	BJ987693
E_FL_BmN-_05I21_F_0	BJ981520
E_FL_BmN-_05I21_R_0	BJ987694
E_FL_BmN-_05I23_F_0	BJ981521
E_FL_BmN-_05I23_R_0	BJ987695
E_FL_BmN-_05I24_F_0	BJ981522
E_FL_BmN-_05I24_R_0	BJ987696
E_FL_BmN-_05J01_F_0	BJ981523
E_FL_BmN-_05J01_R_0	BJ987697
E_FL_BmN-_05J02_F_0	BJ981524
E_FL_BmN-_05J02_R_0	BJ987698
E_FL_BmN-_05J03_F_0	BJ981525
E_FL_BmN-_05J03_R_0	BJ987699
E_FL_BmN-_05J04_F_0	BJ981526
E_FL_BmN-_05J04_R_0	BJ987700
E_FL_BmN-_05J05_F_0	BJ981527
E_FL_BmN-_05J05_R_0	BJ987701
E_FL_BmN-_05J06_F_0	BJ981528
E_FL_BmN-_05J06_R_0	BJ987702
E_FL_BmN-_05J07_F_0	BJ981529
E_FL_BmN-_05J08_F_0	BJ981530
E_FL_BmN-_05J08_R_0	BJ987703
E_FL_BmN-_05J09_F_0	BJ981531
E_FL_BmN-_05J09_R_0	BJ987704
E_FL_BmN-_05J10_F_0	BJ981532
E_FL_BmN-_05J10_R_0	BJ987705
E_FL_BmN-_05J11_F_0	BJ981533

E_FL_BmN-_05J11_R_0	BJ987706
E_FL_BmN-_05J12_F_0	BJ981534
E_FL_BmN-_05J12_R_0	BJ987707
E_FL_BmN-_05J13_F_0	BJ981535
E_FL_BmN-_05J14_R_0	BJ987708
E_FL_BmN-_05J15_F_0	BJ981536
E_FL_BmN-_05J15_R_0	BJ987709
E_FL_BmN-_05J16_F_0	BJ981537
E_FL_BmN-_05J16_R_0	BJ987710
E_FL_BmN-_05J17_F_0	BJ981538
E_FL_BmN-_05J17_R_0	BJ987711
E_FL_BmN-_05J18_F_0	BJ981539
E_FL_BmN-_05J18_R_0	BJ987712
E_FL_BmN-_05J20_F_0	BJ981540
E_FL_BmN-_05J20_R_0	BJ987713
E_FL_BmN-_05J22_F_0	BJ981541
E_FL_BmN-_05J22_R_0	BJ987714
E_FL_BmN-_05J23_F_0	BJ981542
E_FL_BmN-_05J23_R_0	BJ987715
E_FL_BmN-_05J24_F_0	BJ981543
E_FL_BmN-_05J24_R_0	BJ987716
E_FL_BmN-_05K01_F_0	BJ981544
E_FL_BmN-_05K01_R_0	BJ987717
E_FL_BmN-_05K02_F_0	BJ981545
E_FL_BmN-_05K02_R_0	BJ987718
E_FL_BmN-_05K04_F_0	BJ981546
E_FL_BmN-_05K04_R_0	BJ987719
E_FL_BmN-_05K05_F_0	BJ981547
E_FL_BmN-_05K06_R_0	BJ987720
E_FL_BmN-_05K07_F_0	BJ981548
E_FL_BmN-_05K08_R_0	BJ987721
E_FL_BmN-_05K09_F_0	BJ981549
E_FL_BmN-_05K09_R_0	BJ987722
E_FL_BmN-_05K10_F_0	BJ981550
E_FL_BmN-_05K10_R_0	BJ987723
E_FL_BmN-_05K11_F_0	BJ981551
E_FL_BmN-_05K11_R_0	BJ987724
E_FL_BmN-_05K12_F_0	BJ981552
E_FL_BmN-_05K12_R_0	BJ987725
E_FL_BmN-_05K13_F_0	BJ981553
E_FL_BmN-_05K14_F_0	BJ981554
E_FL_BmN-_05K14_R_0	BJ987726
E_FL_BmN-_05K15_F_0	BJ981555
E_FL_BmN-_05K15_R_0	BJ987727
E_FL_BmN-_05K16_F_0	BJ981556
E_FL_BmN-_05K16_R_0	BJ987728
E_FL_BmN-_05K17_F_0	BJ981557
E_FL_BmN-_05K17_R_0	BJ987729
E_FL_BmN-_05K19_F_0	BJ981558
E_FL_BmN-_05K19_R_0	BJ987730
E_FL_BmN-_05K20_F_0	BJ981559
E_FL_BmN-_05K20_R_0	BJ987731
E_FL_BmN-_05K21_F_0	BJ981560
E_FL_BmN-_05K21_R_0	BJ987732

E_FL_BmN-_05K22_F_0	BJ981561
E_FL_BmN-_05K22_R_0	BJ987733
E_FL_BmN-_05K23_F_0	BJ981562
E_FL_BmN-_05K23_R_0	BJ987734
E_FL_BmN-_05K24_F_0	BJ981563
E_FL_BmN-_05K24_R_0	BJ987735
E_FL_BmN-_05L01_F_0	BJ981564
E_FL_BmN-_05L01_R_0	BJ987736
E_FL_BmN-_05L02_F_0	BJ981565
E_FL_BmN-_05L02_R_0	BJ987737
E_FL_BmN-_05L03_F_0	BJ981566
E_FL_BmN-_05L03_R_0	BJ987738
E_FL_BmN-_05L04_F_0	BJ981567
E_FL_BmN-_05L04_R_0	BJ987739
E_FL_BmN-_05L05_F_0	BJ981568
E_FL_BmN-_05L05_R_0	BJ987740
E_FL_BmN-_05L06_R_0	BJ987741
E_FL_BmN-_05L07_F_0	BJ981569
E_FL_BmN-_05L07_R_0	BJ987742
E_FL_BmN-_05L08_F_0	BJ981570
E_FL_BmN-_05L09_F_0	BJ981571
E_FL_BmN-_05L09_R_0	BJ987743
E_FL_BmN-_05L10_F_0	BJ981572
E_FL_BmN-_05L10_R_0	BJ987744
E_FL_BmN-_05L11_R_0	BJ987745
E_FL_BmN-_05L12_R_0	BJ987746
E_FL_BmN-_05L13_F_0	BJ981573
E_FL_BmN-_05L13_R_0	BJ987747
E_FL_BmN-_05L14_F_0	BJ981574
E_FL_BmN-_05L14_R_0	BJ987748
E_FL_BmN-_05L15_F_0	BJ981575
E_FL_BmN-_05L15_R_0	BJ987749
E_FL_BmN-_05L17_R_0	BJ987750
E_FL_BmN-_05L19_F_0	BJ981576
E_FL_BmN-_05L19_R_0	BJ987751
E_FL_BmN-_05L21_F_0	BJ981577
E_FL_BmN-_05L21_R_0	BJ987752
E_FL_BmN-_05L22_F_0	BJ981578
E_FL_BmN-_05L22_R_0	BJ987753
E_FL_BmN-_05L23_F_0	BJ981579
E_FL_BmN-_05L23_R_0	BJ987754
E_FL_BmN-_05L24_F_0	BJ981580
E_FL_BmN-_05L24_R_0	BJ987755
E_FL_BmN-_05M02_F_0	BJ981581
E_FL_BmN-_05M02_R_0	BJ987756
E_FL_BmN-_05M03_F_0	BJ981582
E_FL_BmN-_05M03_R_0	BJ987757
E_FL_BmN-_05M04_F_0	BJ981583
E_FL_BmN-_05M04_R_0	BJ987758
E_FL_BmN-_05M05_F_0	BJ981584
E_FL_BmN-_05M05_R_0	BJ987759
E_FL_BmN-_05M06_F_0	BJ981585
E_FL_BmN-_05M06_R_0	BJ987760
E_FL_BmN-_05M07_F_0	BJ981586

E_FL_BmN-_05M07_R_0	BJ987761
E_FL_BmN-_05M08_F_0	BJ981587
E_FL_BmN-_05M08_R_0	BJ987762
E_FL_BmN-_05M09_F_0	BJ981588
E_FL_BmN-_05M09_R_0	BJ987763
E_FL_BmN-_05M10_F_0	BJ981589
E_FL_BmN-_05M10_R_0	BJ987764
E_FL_BmN-_05M11_F_0	BJ981590
E_FL_BmN-_05M11_R_0	BJ987765
E_FL_BmN-_05M12_R_0	BJ987766
E_FL_BmN-_05M13_F_0	BJ981591
E_FL_BmN-_05M14_F_0	BJ981592
E_FL_BmN-_05M14_R_0	BJ987767
E_FL_BmN-_05M15_F_0	BJ981593
E_FL_BmN-_05M15_R_0	BJ987768
E_FL_BmN-_05M16_F_0	BJ981594
E_FL_BmN-_05M16_R_0	BJ987769
E_FL_BmN-_05M17_F_0	BJ981595
E_FL_BmN-_05M17_R_0	BJ987770
E_FL_BmN-_05M19_F_0	BJ981596
E_FL_BmN-_05M20_F_0	BJ981597
E_FL_BmN-_05M20_R_0	BJ987771
E_FL_BmN-_05M21_F_0	BJ981598
E_FL_BmN-_05M21_R_0	BJ987772
E_FL_BmN-_05M22_F_0	BJ981599
E_FL_BmN-_05M22_R_0	BJ987773
E_FL_BmN-_05M23_F_0	BJ981600
E_FL_BmN-_05M23_R_0	BJ987774
E_FL_BmN-_05M24_F_0	BJ981601
E_FL_BmN-_05M24_R_0	BJ987775
E_FL_BmN-_05N01_R_0	BJ987776
E_FL_BmN-_05N02_F_0	BJ981602
E_FL_BmN-_05N02_R_0	BJ987777
E_FL_BmN-_05N03_F_0	BJ981603
E_FL_BmN-_05N03_R_0	BJ987778
E_FL_BmN-_05N04_F_0	BJ981604
E_FL_BmN-_05N04_R_0	BJ987779
E_FL_BmN-_05N05_F_0	BJ981605
E_FL_BmN-_05N05_R_0	BJ987780
E_FL_BmN-_05N06_F_0	BJ981606
E_FL_BmN-_05N06_R_0	BJ987781
E_FL_BmN-_05N07_F_0	BJ981607
E_FL_BmN-_05N07_R_0	BJ987782
E_FL_BmN-_05N08_F_0	BJ981608
E_FL_BmN-_05N08_R_0	BJ987783
E_FL_BmN-_05N09_F_0	BJ981609
E_FL_BmN-_05N09_R_0	BJ987784
E_FL_BmN-_05N10_F_0	BJ981610
E_FL_BmN-_05N10_R_0	BJ987785
E_FL_BmN-_05N11_R_0	BJ987786
E_FL_BmN-_05N12_F_0	BJ981611
E_FL_BmN-_05N12_R_0	BJ987787
E_FL_BmN-_05N13_F_0	BJ981612
E_FL_BmN-_05N13_R_0	BJ987788

E_FL_BmN-_05N14_F_0	BJ981613
E_FL_BmN-_05N14_R_0	BJ987789
E_FL_BmN-_05N15_F_0	BJ981614
E_FL_BmN-_05N15_R_0	BJ987790
E_FL_BmN-_05N16_F_0	BJ981615
E_FL_BmN-_05N16_R_0	BJ987791
E_FL_BmN-_05N17_R_0	BJ987792
E_FL_BmN-_05N18_R_0	BJ987793
E_FL_BmN-_05N19_R_0	BJ987794
E_FL_BmN-_05N20_F_0	BJ981616
E_FL_BmN-_05N20_R_0	BJ987795
E_FL_BmN-_05N21_F_0	BJ981617
E_FL_BmN-_05N21_R_0	BJ987796
E_FL_BmN-_05N22_F_0	BJ981618
E_FL_BmN-_05N22_R_0	BJ987797
E_FL_BmN-_05N23_F_0	BJ981619
E_FL_BmN-_05N23_R_0	BJ987798
E_FL_BmN-_05N24_F_0	BJ981620
E_FL_BmN-_05N24_R_0	BJ987799
E_FL_BmN-_05O02_F_0	BJ981621
E_FL_BmN-_05O02_R_0	BJ987800
E_FL_BmN-_05O03_F_0	BJ981622
E_FL_BmN-_05O03_R_0	BJ987801
E_FL_BmN-_05O05_F_0	BJ981623
E_FL_BmN-_05O05_R_0	BJ987802
E_FL_BmN-_05O06_R_0	BJ987803
E_FL_BmN-_05O07_F_0	BJ981624
E_FL_BmN-_05O09_F_0	BJ981625
E_FL_BmN-_05O09_R_0	BJ987804
E_FL_BmN-_05O10_F_0	BJ981626
E_FL_BmN-_05O10_R_0	BJ987805
E_FL_BmN-_05O11_R_0	BJ987806
E_FL_BmN-_05O12_F_0	BJ981627
E_FL_BmN-_05O12_R_0	BJ987807
E_FL_BmN-_05O13_F_0	BJ981628
E_FL_BmN-_05O14_F_0	BJ981629
E_FL_BmN-_05O14_R_0	BJ987808
E_FL_BmN-_05O15_F_0	BJ981630
E_FL_BmN-_05O15_R_0	BJ987809
E_FL_BmN-_05O16_F_0	BJ981631
E_FL_BmN-_05O16_R_0	BJ987810
E_FL_BmN-_05O17_R_0	BJ987811
E_FL_BmN-_05O18_R_0	BJ987812
E_FL_BmN-_05O19_F_0	BJ981632
E_FL_BmN-_05O19_R_0	BJ987813
E_FL_BmN-_05O20_F_0	BJ981633
E_FL_BmN-_05O21_F_0	BJ981634
E_FL_BmN-_05O21_R_0	BJ987814
E_FL_BmN-_05O22_F_0	BJ981635
E_FL_BmN-_05O22_R_0	BJ987815
E_FL_BmN-_05O23_F_0	BJ981636
E_FL_BmN-_05O23_R_0	BJ987816
E_FL_BmN-_05O24_R_0	BJ987817
E_FL_BmN-_05P01_F_0	BJ981637

E_FL_BmN-_05P01_R_0	BJ987818
E_FL_BmN-_05P02_F_0	BJ981638
E_FL_BmN-_05P02_R_0	BJ987819
E_FL_BmN-_05P03_F_0	BJ981639
E_FL_BmN-_05P03_R_0	BJ987820
E_FL_BmN-_05P04_R_0	BJ987821
E_FL_BmN-_05P05_F_0	BJ981640
E_FL_BmN-_05P05_R_0	BJ987822
E_FL_BmN-_05P06_F_0	BJ981641
E_FL_BmN-_05P06_R_0	BJ987823
E_FL_BmN-_05P07_F_0	BJ981642
E_FL_BmN-_05P07_R_0	BJ987824
E_FL_BmN-_05P08_F_0	BJ981643
E_FL_BmN-_05P08_R_0	BJ987825
E_FL_BmN-_05P09_F_0	BJ981644
E_FL_BmN-_05P09_R_0	BJ987826
E_FL_BmN-_05P10_R_0	BJ987827
E_FL_BmN-_05P11_R_0	BJ987828
E_FL_BmN-_05P12_F_0	BJ981645
E_FL_BmN-_05P12_R_0	BJ987829
E_FL_BmN-_05P13_F_0	BJ981646
E_FL_BmN-_05P13_R_0	BJ987830
E_FL_BmN-_05P14_F_0	BJ981647
E_FL_BmN-_05P14_R_0	BJ987831
E_FL_BmN-_05P15_F_0	BJ981648
E_FL_BmN-_05P15_R_0	BJ987832
E_FL_BmN-_05P17_F_0	BJ981649
E_FL_BmN-_05P17_R_0	BJ987833
E_FL_BmN-_05P18_F_0	BJ981650
E_FL_BmN-_05P18_R_0	BJ987834
E_FL_BmN-_05P19_R_0	BJ987835
E_FL_BmN-_05P20_F_0	BJ981651
E_FL_BmN-_05P20_R_0	BJ987836
E_FL_BmN-_05P21_F_0	BJ981652
E_FL_BmN-_05P21_R_0	BJ987837
E_FL_BmN-_05P22_F_0	BJ981653
E_FL_BmN-_05P22_R_0	BJ987838
E_FL_BmN-_05P23_F_0	BJ981654
E_FL_BmN-_05P23_R_0	BJ987839
E_FL_BmN-_05P24_F_0	BJ981655
E_FL_BmN-_05P24_R_0	BJ987840
E_FL_BmN-_06A01_F_0	BJ981656
E_FL_BmN-_06A02_F_0	BJ981657
E_FL_BmN-_06A02_R_0	BJ987841
E_FL_BmN-_06A03_F_0	BJ981658
E_FL_BmN-_06A04_F_0	BJ981659
E_FL_BmN-_06A04_R_0	BJ987842
E_FL_BmN-_06A05_F_0	BJ981660
E_FL_BmN-_06A06_F_0	BJ981661
E_FL_BmN-_06A06_R_0	BJ987843
E_FL_BmN-_06A08_R_0	BJ987844
E_FL_BmN-_06A09_F_0	BJ981662
E_FL_BmN-_06A10_F_0	BJ981663
E_FL_BmN-_06A10_R_0	BJ987845

E_FL_BmN-_06A12_F_0	BJ981664
E_FL_BmN-_06A12_R_0	BJ987846
E_FL_BmN-_06A13_F_0	BJ981665
E_FL_BmN-_06A13_R_0	BJ987847
E_FL_BmN-_06A14_F_0	BJ981666
E_FL_BmN-_06A14_R_0	BJ987848
E_FL_BmN-_06A15_R_0	BJ987849
E_FL_BmN-_06A16_F_0	BJ981667
E_FL_BmN-_06A16_R_0	BJ987850
E_FL_BmN-_06A17_F_0	BJ981668
E_FL_BmN-_06A17_R_0	BJ987851
E_FL_BmN-_06A18_F_0	BJ981669
E_FL_BmN-_06A18_R_0	BJ987852
E_FL_BmN-_06A20_F_0	BJ981670
E_FL_BmN-_06A20_R_0	BJ987853
E_FL_BmN-_06A21_F_0	BJ981671
E_FL_BmN-_06A21_R_0	BJ987854
E_FL_BmN-_06A22_F_0	BJ981672
E_FL_BmN-_06A22_R_0	BJ987855
E_FL_BmN-_06A23_F_0	BJ981673
E_FL_BmN-_06A24_R_0	BJ987856
E_FL_BmN-_06B01_F_0	BJ981674
E_FL_BmN-_06B01_R_0	BJ987857
E_FL_BmN-_06B02_R_0	BJ987858
E_FL_BmN-_06B03_F_0	BJ981675
E_FL_BmN-_06B03_R_0	BJ987859
E_FL_BmN-_06B04_F_0	BJ981676
E_FL_BmN-_06B04_R_0	BJ987860
E_FL_BmN-_06B05_R_0	BJ987861
E_FL_BmN-_06B06_R_0	BJ987862
E_FL_BmN-_06B08_R_0	BJ987863
E_FL_BmN-_06B09_F_0	BJ981677
E_FL_BmN-_06B09_R_0	BJ987864
E_FL_BmN-_06B10_F_0	BJ981678
E_FL_BmN-_06B10_R_0	BJ987865
E_FL_BmN-_06B11_F_0	BJ981679
E_FL_BmN-_06B11_R_0	BJ987866
E_FL_BmN-_06B12_R_0	BJ987867
E_FL_BmN-_06B13_F_0	BJ981680
E_FL_BmN-_06B15_F_0	BJ981681
E_FL_BmN-_06B15_R_0	BJ987868
E_FL_BmN-_06B16_F_0	BJ981682
E_FL_BmN-_06B16_R_0	BJ987869
E_FL_BmN-_06B17_R_0	BJ987870
E_FL_BmN-_06B18_R_0	BJ987871
E_FL_BmN-_06B19_F_0	BJ981683
E_FL_BmN-_06B19_R_0	BJ987872
E_FL_BmN-_06B20_F_0	BJ981684
E_FL_BmN-_06B20_R_0	BJ987873
E_FL_BmN-_06B21_F_0	BJ981685
E_FL_BmN-_06B21_R_0	BJ987874
E_FL_BmN-_06B22_F_0	BJ981686
E_FL_BmN-_06B22_R_0	BJ987875
E_FL_BmN-_06B23_F_0	BJ981687

E_FL_BmN-_06B23_R_0	BJ987876
E_FL_BmN-_06B24_F_0	BJ981688
E_FL_BmN-_06B24_R_0	BJ987877
E_FL_BmN-_06C01_F_0	BJ981689
E_FL_BmN-_06C02_F_0	BJ981690
E_FL_BmN-_06C02_R_0	BJ987878
E_FL_BmN-_06C04_F_0	BJ981691
E_FL_BmN-_06C04_R_0	BJ987879
E_FL_BmN-_06C05_F_0	BJ981692
E_FL_BmN-_06C05_R_0	BJ987880
E_FL_BmN-_06C06_R_0	BJ987881
E_FL_BmN-_06C07_F_0	BJ981693
E_FL_BmN-_06C07_R_0	BJ987882
E_FL_BmN-_06C08_F_0	BJ981694
E_FL_BmN-_06C08_R_0	BJ987883
E_FL_BmN-_06C12_R_0	BJ987884
E_FL_BmN-_06C15_F_0	BJ981695
E_FL_BmN-_06C16_F_0	BJ981696
E_FL_BmN-_06C16_R_0	BJ987885
E_FL_BmN-_06C18_R_0	BJ987886
E_FL_BmN-_06C19_F_0	BJ981697
E_FL_BmN-_06C20_F_0	BJ981698
E_FL_BmN-_06C20_R_0	BJ987887
E_FL_BmN-_06C21_F_0	BJ981699
E_FL_BmN-_06C21_R_0	BJ987888
E_FL_BmN-_06C22_F_0	BJ981700
E_FL_BmN-_06C22_R_0	BJ987889
E_FL_BmN-_06C23_F_0	BJ981701
E_FL_BmN-_06C24_R_0	BJ987890
E_FL_BmN-_06D01_F_0	BJ981702
E_FL_BmN-_06D01_R_0	BJ987891
E_FL_BmN-_06D02_R_0	BJ987892
E_FL_BmN-_06D03_F_0	BJ981703
E_FL_BmN-_06D03_R_0	BJ987893
E_FL_BmN-_06D04_F_0	BJ981704
E_FL_BmN-_06D04_R_0	BJ987894
E_FL_BmN-_06D05_F_0	BJ981705
E_FL_BmN-_06D05_R_0	BJ987895
E_FL_BmN-_06D07_F_0	BJ981706
E_FL_BmN-_06D08_F_0	BJ981707
E_FL_BmN-_06D08_R_0	BJ987896
E_FL_BmN-_06D09_R_0	BJ987897
E_FL_BmN-_06D10_R_0	BJ987898
E_FL_BmN-_06D11_F_0	BJ981708
E_FL_BmN-_06D11_R_0	BJ987899
E_FL_BmN-_06D12_F_0	BJ981709
E_FL_BmN-_06D12_R_0	BJ987900
E_FL_BmN-_06D13_F_0	BJ981710
E_FL_BmN-_06D13_R_0	BJ987901
E_FL_BmN-_06D14_F_0	BJ981711
E_FL_BmN-_06D14_R_0	BJ987902
E_FL_BmN-_06D15_F_0	BJ981712
E_FL_BmN-_06D15_R_0	BJ987903
E_FL_BmN-_06D16_F_0	BJ981713

E_FL_BmN-_06D16_R_0	BJ987904
E_FL_BmN-_06D17_F_0	BJ981714
E_FL_BmN-_06D17_R_0	BJ987905
E_FL_BmN-_06D18_R_0	BJ987906
E_FL_BmN-_06D19_F_0	BJ981715
E_FL_BmN-_06D19_R_0	BJ987907
E_FL_BmN-_06D20_F_0	BJ981716
E_FL_BmN-_06D20_R_0	BJ987908
E_FL_BmN-_06D21_F_0	BJ981717
E_FL_BmN-_06D22_R_0	BJ987909
E_FL_BmN-_06D23_F_0	BJ981718
E_FL_BmN-_06D23_R_0	BJ987910
E_FL_BmN-_06D24_F_0	BJ981719
E_FL_BmN-_06D24_R_0	BJ987911
E_FL_BmN-_06E02_F_0	BJ981720
E_FL_BmN-_06E02_R_0	BJ987912
E_FL_BmN-_06E03_F_0	BJ981721
E_FL_BmN-_06E03_R_0	BJ987913
E_FL_BmN-_06E04_F_0	BJ981722
E_FL_BmN-_06E05_F_0	BJ981723
E_FL_BmN-_06E05_R_0	BJ987914
E_FL_BmN-_06E06_F_0	BJ981724
E_FL_BmN-_06E06_R_0	BJ987915
E_FL_BmN-_06E07_F_0	BJ981725
E_FL_BmN-_06E08_F_0	BJ981726
E_FL_BmN-_06E08_R_0	BJ987916
E_FL_BmN-_06E10_F_0	BJ981727
E_FL_BmN-_06E10_R_0	BJ987917
E_FL_BmN-_06E11_R_0	BJ987918
E_FL_BmN-_06E12_F_0	BJ981728
E_FL_BmN-_06E12_R_0	BJ987919
E_FL_BmN-_06E13_F_0	BJ981729
E_FL_BmN-_06E14_F_0	BJ981730
E_FL_BmN-_06E14_R_0	BJ987920
E_FL_BmN-_06E15_F_0	BJ981731
E_FL_BmN-_06E15_R_0	BJ987921
E_FL_BmN-_06E16_F_0	BJ981732
E_FL_BmN-_06E17_F_0	BJ981733
E_FL_BmN-_06E17_R_0	BJ987922
E_FL_BmN-_06E18_F_0	BJ981734
E_FL_BmN-_06E18_R_0	BJ987923
E_FL_BmN-_06E19_F_0	BJ981735
E_FL_BmN-_06E20_R_0	BJ987924
E_FL_BmN-_06E22_F_0	BJ981736
E_FL_BmN-_06E22_R_0	BJ987925
E_FL_BmN-_06E23_F_0	BJ981737
E_FL_BmN-_06E23_R_0	BJ987926
E_FL_BmN-_06E24_F_0	BJ981738
E_FL_BmN-_06E24_R_0	BJ987927
E_FL_BmN-_06F02_F_0	BJ981739
E_FL_BmN-_06F02_R_0	BJ987928
E_FL_BmN-_06F03_F_0	BJ981740
E_FL_BmN-_06F03_R_0	BJ987929
E_FL_BmN-_06F04_F_0	BJ981741

E_FL_BmN-_06F04_R_0	BJ987930
E_FL_BmN-_06F06_F_0	BJ981742
E_FL_BmN-_06F06_R_0	BJ987931
E_FL_BmN-_06F07_F_0	BJ981743
E_FL_BmN-_06F07_R_0	BJ987932
E_FL_BmN-_06F08_R_0	BJ987933
E_FL_BmN-_06F09_F_0	BJ981744
E_FL_BmN-_06F09_R_0	BJ987934
E_FL_BmN-_06F10_R_0	BJ987935
E_FL_BmN-_06F11_F_0	BJ981745
E_FL_BmN-_06F11_R_0	BJ987936
E_FL_BmN-_06F12_F_0	BJ981746
E_FL_BmN-_06F12_R_0	BJ987937
E_FL_BmN-_06F13_F_0	BJ981747
E_FL_BmN-_06F13_R_0	BJ987938
E_FL_BmN-_06F14_R_0	BJ987939
E_FL_BmN-_06F15_F_0	BJ981748
E_FL_BmN-_06F15_R_0	BJ987940
E_FL_BmN-_06F16_F_0	BJ981749
E_FL_BmN-_06F16_R_0	BJ987941
E_FL_BmN-_06F17_F_0	BJ981750
E_FL_BmN-_06F17_R_0	BJ987942
E_FL_BmN-_06F18_R_0	BJ987943
E_FL_BmN-_06F20_F_0	BJ981751
E_FL_BmN-_06F20_R_0	BJ987944
E_FL_BmN-_06F21_F_0	BJ981752
E_FL_BmN-_06F21_R_0	BJ987945
E_FL_BmN-_06F22_F_0	BJ981753
E_FL_BmN-_06F23_F_0	BJ981754
E_FL_BmN-_06F23_R_0	BJ987946
E_FL_BmN-_06F24_F_0	BJ981755
E_FL_BmN-_06F24_R_0	BJ987947
E_FL_BmN-_06G02_F_0	BJ981756
E_FL_BmN-_06G02_R_0	BJ987948
E_FL_BmN-_06G04_F_0	BJ981757
E_FL_BmN-_06G04_R_0	BJ987949
E_FL_BmN-_06G05_F_0	BJ981758
E_FL_BmN-_06G05_R_0	BJ987950
E_FL_BmN-_06G06_F_0	BJ981759
E_FL_BmN-_06G06_R_0	BJ987951
E_FL_BmN-_06G07_F_0	BJ981760
E_FL_BmN-_06G07_R_0	BJ987952
E_FL_BmN-_06G08_F_0	BJ981761
E_FL_BmN-_06G08_R_0	BJ987953
E_FL_BmN-_06G09_R_0	BJ987954
E_FL_BmN-_06G10_F_0	BJ981762
E_FL_BmN-_06G10_R_0	BJ987955
E_FL_BmN-_06G12_F_0	BJ981763
E_FL_BmN-_06G12_R_0	BJ987956
E_FL_BmN-_06G14_F_0	BJ981764
E_FL_BmN-_06G14_R_0	BJ987957
E_FL_BmN-_06G15_F_0	BJ981765
E_FL_BmN-_06G15_R_0	BJ987958
E_FL_BmN-_06G16_F_0	BJ981766

E_FL_BmN-_06G16_R_0	BJ987959
E_FL_BmN-_06G17_F_0	BJ981767
E_FL_BmN-_06G17_R_0	BJ987960
E_FL_BmN-_06G18_R_0	BJ987961
E_FL_BmN-_06G19_R_0	BJ987962
E_FL_BmN-_06G20_F_0	BJ981768
E_FL_BmN-_06G20_R_0	BJ987963
E_FL_BmN-_06G21_F_0	BJ981769
E_FL_BmN-_06G21_R_0	BJ987964
E_FL_BmN-_06G22_F_0	BJ981770
E_FL_BmN-_06G22_R_0	BJ987965
E_FL_BmN-_06G23_F_0	BJ981771
E_FL_BmN-_06G23_R_0	BJ987966
E_FL_BmN-_06G24_F_0	BJ981772
E_FL_BmN-_06G24_R_0	BJ987967
E_FL_BmN-_06H02_R_0	BJ987968
E_FL_BmN-_06H03_F_0	BJ981773
E_FL_BmN-_06H03_R_0	BJ987969
E_FL_BmN-_06H04_F_0	BJ981774
E_FL_BmN-_06H04_R_0	BJ987970
E_FL_BmN-_06H06_F_0	BJ981775
E_FL_BmN-_06H06_R_0	BJ987971
E_FL_BmN-_06H07_F_0	BJ981776
E_FL_BmN-_06H07_R_0	BJ987972
E_FL_BmN-_06H08_R_0	BJ987973
E_FL_BmN-_06H09_F_0	BJ981777
E_FL_BmN-_06H09_R_0	BJ987974
E_FL_BmN-_06H10_F_0	BJ981778
E_FL_BmN-_06H10_R_0	BJ987975
E_FL_BmN-_06H11_F_0	BJ981779
E_FL_BmN-_06H11_R_0	BJ987976
E_FL_BmN-_06H12_R_0	BJ987977
E_FL_BmN-_06H13_F_0	BJ981780
E_FL_BmN-_06H13_R_0	BJ987978
E_FL_BmN-_06H14_R_0	BJ987979
E_FL_BmN-_06H15_F_0	BJ981781
E_FL_BmN-_06H15_R_0	BJ987980
E_FL_BmN-_06H16_F_0	BJ981782
E_FL_BmN-_06H16_R_0	BJ987981
E_FL_BmN-_06H17_F_0	BJ981783
E_FL_BmN-_06H17_R_0	BJ987982
E_FL_BmN-_06H18_R_0	BJ987983
E_FL_BmN-_06H19_F_0	BJ981784
E_FL_BmN-_06H19_R_0	BJ987984
E_FL_BmN-_06H20_F_0	BJ981785
E_FL_BmN-_06H20_R_0	BJ987985
E_FL_BmN-_06H21_F_0	BJ981786
E_FL_BmN-_06H21_R_0	BJ987986
E_FL_BmN-_06H22_F_0	BJ981787
E_FL_BmN-_06H22_R_0	BJ987987
E_FL_BmN-_06H23_F_0	BJ981788
E_FL_BmN-_06H23_R_0	BJ987988
E_FL_BmN-_06I01_F_0	BJ981789
E_FL_BmN-_06I01_R_0	BJ987989

E_FL_BmN-_06I02_F_0	BJ981790
E_FL_BmN-_06I02_R_0	BJ987990
E_FL_BmN-_06I03_F_0	BJ981791
E_FL_BmN-_06I03_R_0	BJ987991
E_FL_BmN-_06I04_F_0	BJ981792
E_FL_BmN-_06I04_R_0	BJ987992
E_FL_BmN-_06I05_R_0	BJ987993
E_FL_BmN-_06I06_F_0	BJ981793
E_FL_BmN-_06I06_R_0	BJ987994
E_FL_BmN-_06I08_F_0	BJ981794
E_FL_BmN-_06I08_R_0	BJ987995
E_FL_BmN-_06I09_F_0	BJ981795
E_FL_BmN-_06I09_R_0	BJ987996
E_FL_BmN-_06I10_F_0	BJ981796
E_FL_BmN-_06I10_R_0	BJ987997
E_FL_BmN-_06I11_R_0	BJ987998
E_FL_BmN-_06I12_F_0	BJ981797
E_FL_BmN-_06I12_R_0	BJ987999
E_FL_BmN-_06I14_F_0	BJ981798
E_FL_BmN-_06I14_R_0	BJ988000
E_FL_BmN-_06I15_F_0	BJ981799
E_FL_BmN-_06I15_R_0	BJ988001
E_FL_BmN-_06I16_F_0	BJ981800
E_FL_BmN-_06I16_R_0	BJ988002
E_FL_BmN-_06I17_F_0	BJ981801
E_FL_BmN-_06I17_R_0	BJ988003
E_FL_BmN-_06I18_F_0	BJ981802
E_FL_BmN-_06I18_R_0	BJ988004
E_FL_BmN-_06I19_F_0	BJ981803
E_FL_BmN-_06I19_R_0	BJ988005
E_FL_BmN-_06I20_F_0	BJ981804
E_FL_BmN-_06I20_R_0	BJ988006
E_FL_BmN-_06I21_F_0	BJ981805
E_FL_BmN-_06I21_R_0	BJ988007
E_FL_BmN-_06I22_F_0	BJ981806
E_FL_BmN-_06I22_R_0	BJ988008
E_FL_BmN-_06I23_F_0	BJ981807
E_FL_BmN-_06I24_R_0	BJ988009
E_FL_BmN-_06J01_F_0	BJ981808
E_FL_BmN-_06J01_R_0	BJ988010
E_FL_BmN-_06J02_F_0	BJ981809
E_FL_BmN-_06J02_R_0	BJ988011
E_FL_BmN-_06J03_F_0	BJ981810
E_FL_BmN-_06J03_R_0	BJ988012
E_FL_BmN-_06J04_F_0	BJ981811
E_FL_BmN-_06J04_R_0	BJ988013
E_FL_BmN-_06J05_F_0	BJ981812
E_FL_BmN-_06J05_R_0	BJ988014
E_FL_BmN-_06J06_F_0	BJ981813
E_FL_BmN-_06J06_R_0	BJ988015
E_FL_BmN-_06J07_F_0	BJ981814
E_FL_BmN-_06J07_R_0	BJ988016
E_FL_BmN-_06J08_F_0	BJ981815
E_FL_BmN-_06J08_R_0	BJ988017

E_FL_BmN-_06J09_R_0	BJ988018
E_FL_BmN-_06J10_R_0	BJ988019
E_FL_BmN-_06J11_F_0	BJ981816
E_FL_BmN-_06J11_R_0	BJ988020
E_FL_BmN-_06J12_F_0	BJ981817
E_FL_BmN-_06J12_R_0	BJ988021
E_FL_BmN-_06J13_F_0	BJ981818
E_FL_BmN-_06J13_R_0	BJ988022
E_FL_BmN-_06J14_R_0	BJ988023
E_FL_BmN-_06J15_F_0	BJ981819
E_FL_BmN-_06J15_R_0	BJ988024
E_FL_BmN-_06J16_F_0	BJ981820
E_FL_BmN-_06J16_R_0	BJ988025
E_FL_BmN-_06J17_F_0	BJ981821
E_FL_BmN-_06J17_R_0	BJ988026
E_FL_BmN-_06J18_R_0	BJ988027
E_FL_BmN-_06J19_F_0	BJ981822
E_FL_BmN-_06J19_R_0	BJ988028
E_FL_BmN-_06J21_F_0	BJ981823
E_FL_BmN-_06J21_R_0	BJ988029
E_FL_BmN-_06J22_F_0	BJ981824
E_FL_BmN-_06J22_R_0	BJ988030
E_FL_BmN-_06J23_F_0	BJ981825
E_FL_BmN-_06J23_R_0	BJ988031
E_FL_BmN-_06J24_F_0	BJ981826
E_FL_BmN-_06J24_R_0	BJ988032
E_FL_BmN-_06K01_F_0	BJ981827
E_FL_BmN-_06K01_R_0	BJ988033
E_FL_BmN-_06K02_F_0	BJ981828
E_FL_BmN-_06K02_R_0	BJ988034
E_FL_BmN-_06K03_F_0	BJ981829
E_FL_BmN-_06K03_R_0	BJ988035
E_FL_BmN-_06K04_F_0	BJ981830
E_FL_BmN-_06K04_R_0	BJ988036
E_FL_BmN-_06K05_F_0	BJ981831
E_FL_BmN-_06K05_R_0	BJ988037
E_FL_BmN-_06K06_F_0	BJ981832
E_FL_BmN-_06K06_R_0	BJ988038
E_FL_BmN-_06K08_F_0	BJ981833
E_FL_BmN-_06K08_R_0	BJ988039
E_FL_BmN-_06K09_F_0	BJ981834
E_FL_BmN-_06K09_R_0	BJ988040
E_FL_BmN-_06K10_F_0	BJ981835
E_FL_BmN-_06K10_R_0	BJ988041
E_FL_BmN-_06K11_R_0	BJ988042
E_FL_BmN-_06K12_F_0	BJ981836
E_FL_BmN-_06K12_R_0	BJ988043
E_FL_BmN-_06K13_F_0	BJ981837
E_FL_BmN-_06K13_R_0	BJ988044
E_FL_BmN-_06K14_F_0	BJ981838
E_FL_BmN-_06K14_R_0	BJ988045
E_FL_BmN-_06K15_F_0	BJ981839
E_FL_BmN-_06K16_F_0	BJ981840
E_FL_BmN-_06K16_R_0	BJ988046

E_FL_BmN-_06K17_F_0	BJ981841
E_FL_BmN-_06K17_R_0	BJ988047
E_FL_BmN-_06K18_F_0	BJ981842
E_FL_BmN-_06K18_R_0	BJ988048
E_FL_BmN-_06K19_R_0	BJ988049
E_FL_BmN-_06K20_F_0	BJ981843
E_FL_BmN-_06K20_R_0	BJ988050
E_FL_BmN-_06K21_F_0	BJ981844
E_FL_BmN-_06K21_R_0	BJ988051
E_FL_BmN-_06K23_F_0	BJ981845
E_FL_BmN-_06K23_R_0	BJ988052
E_FL_BmN-_06K24_F_0	BJ981846
E_FL_BmN-_06K24_R_0	BJ988053
E_FL_BmN-_06L01_F_0	BJ981847
E_FL_BmN-_06L01_R_0	BJ988054
E_FL_BmN-_06L02_R_0	BJ988055
E_FL_BmN-_06L03_F_0	BJ981848
E_FL_BmN-_06L03_R_0	BJ988056
E_FL_BmN-_06L05_R_0	BJ988057
E_FL_BmN-_06L06_F_0	BJ981849
E_FL_BmN-_06L06_R_0	BJ988058
E_FL_BmN-_06L07_F_0	BJ981850
E_FL_BmN-_06L07_R_0	BJ988059
E_FL_BmN-_06L08_R_0	BJ988060
E_FL_BmN-_06L09_F_0	BJ981851
E_FL_BmN-_06L09_R_0	BJ988061
E_FL_BmN-_06L10_F_0	BJ981852
E_FL_BmN-_06L10_R_0	BJ988062
E_FL_BmN-_06L11_F_0	BJ981853
E_FL_BmN-_06L11_R_0	BJ988063
E_FL_BmN-_06L12_R_0	BJ988064
E_FL_BmN-_06L13_F_0	BJ981854
E_FL_BmN-_06L13_R_0	BJ988065
E_FL_BmN-_06L14_R_0	BJ988066
E_FL_BmN-_06L15_F_0	BJ981855
E_FL_BmN-_06L15_R_0	BJ988067
E_FL_BmN-_06L16_F_0	BJ981856
E_FL_BmN-_06L16_R_0	BJ988068
E_FL_BmN-_06L17_F_0	BJ981857
E_FL_BmN-_06L17_R_0	BJ988069
E_FL_BmN-_06L20_R_0	BJ988070
E_FL_BmN-_06L21_F_0	BJ981858
E_FL_BmN-_06L21_R_0	BJ988071
E_FL_BmN-_06L22_F_0	BJ981859
E_FL_BmN-_06L22_R_0	BJ988072
E_FL_BmN-_06L23_F_0	BJ981860
E_FL_BmN-_06L23_R_0	BJ988073
E_FL_BmN-_06L24_F_0	BJ981861
E_FL_BmN-_06L24_R_0	BJ988074
E_FL_BmN-_06M01_F_0	BJ981862
E_FL_BmN-_06M01_R_0	BJ988075
E_FL_BmN-_06M02_F_0	BJ981863
E_FL_BmN-_06M02_R_0	BJ988076
E_FL_BmN-_06M03_F_0	BJ981864

E_FL_BmN-_06M03_R_0	BJ988077
E_FL_BmN-_06M04_F_0	BJ981865
E_FL_BmN-_06M04_R_0	BJ988078
E_FL_BmN-_06M05_F_0	BJ981866
E_FL_BmN-_06M05_R_0	BJ988079
E_FL_BmN-_06M07_F_0	BJ981867
E_FL_BmN-_06M07_R_0	BJ988080
E_FL_BmN-_06M08_F_0	BJ981868
E_FL_BmN-_06M08_R_0	BJ988081
E_FL_BmN-_06M09_F_0	BJ981869
E_FL_BmN-_06M09_R_0	BJ988082
E_FL_BmN-_06M10_F_0	BJ981870
E_FL_BmN-_06M10_R_0	BJ988083
E_FL_BmN-_06M11_R_0	BJ988084
E_FL_BmN-_06M12_F_0	BJ981871
E_FL_BmN-_06M12_R_0	BJ988085
E_FL_BmN-_06M13_F_0	BJ981872
E_FL_BmN-_06M14_F_0	BJ981873
E_FL_BmN-_06M14_R_0	BJ988086
E_FL_BmN-_06M15_F_0	BJ981874
E_FL_BmN-_06M16_F_0	BJ981875
E_FL_BmN-_06M16_R_0	BJ988087
E_FL_BmN-_06M17_F_0	BJ981876
E_FL_BmN-_06M17_R_0	BJ988088
E_FL_BmN-_06M18_F_0	BJ981877
E_FL_BmN-_06M18_R_0	BJ988089
E_FL_BmN-_06M19_R_0	BJ988090
E_FL_BmN-_06M20_F_0	BJ981878
E_FL_BmN-_06M20_R_0	BJ988091
E_FL_BmN-_06M21_F_0	BJ981879
E_FL_BmN-_06M21_R_0	BJ988092
E_FL_BmN-_06M22_F_0	BJ981880
E_FL_BmN-_06M22_R_0	BJ988093
E_FL_BmN-_06M23_F_0	BJ981881
E_FL_BmN-_06M23_R_0	BJ988094
E_FL_BmN-_06M24_F_0	BJ981882
E_FL_BmN-_06M24_R_0	BJ988095
E_FL_BmN-_06N01_F_0	BJ981883
E_FL_BmN-_06N01_R_0	BJ988096
E_FL_BmN-_06N02_F_0	BJ981884
E_FL_BmN-_06N02_R_0	BJ988097
E_FL_BmN-_06N03_F_0	BJ981885
E_FL_BmN-_06N03_R_0	BJ988098
E_FL_BmN-_06N04_F_0	BJ981886
E_FL_BmN-_06N04_R_0	BJ988099
E_FL_BmN-_06N05_F_0	BJ981887
E_FL_BmN-_06N05_R_0	BJ988100
E_FL_BmN-_06N07_F_0	BJ981888
E_FL_BmN-_06N07_R_0	BJ988101
E_FL_BmN-_06N08_F_0	BJ981889
E_FL_BmN-_06N08_R_0	BJ988102
E_FL_BmN-_06N09_R_0	BJ988103
E_FL_BmN-_06N10_F_0	BJ981890
E_FL_BmN-_06N10_R_0	BJ988104

E_FL_BmN-_06N11_F_0	BJ981891
E_FL_BmN-_06N11_R_0	BJ988105
E_FL_BmN-_06N12_F_0	BJ981892
E_FL_BmN-_06N12_R_0	BJ988106
E_FL_BmN-_06N13_F_0	BJ981893
E_FL_BmN-_06N13_R_0	BJ988107
E_FL_BmN-_06N14_F_0	BJ981894
E_FL_BmN-_06N14_R_0	BJ988108
E_FL_BmN-_06N15_F_0	BJ981895
E_FL_BmN-_06N15_R_0	BJ988109
E_FL_BmN-_06N16_F_0	BJ981896
E_FL_BmN-_06N16_R_0	BJ988110
E_FL_BmN-_06N17_F_0	BJ981897
E_FL_BmN-_06N17_R_0	BJ988111
E_FL_BmN-_06N18_F_0	BJ981898
E_FL_BmN-_06N18_R_0	BJ988112
E_FL_BmN-_06N20_F_0	BJ981899
E_FL_BmN-_06N20_R_0	BJ988113
E_FL_BmN-_06N21_F_0	BJ981900
E_FL_BmN-_06N21_R_0	BJ988114
E_FL_BmN-_06N24_F_0	BJ981901
E_FL_BmN-_06N24_R_0	BJ988115
E_FL_BmN-_06O01_F_0	BJ981902
E_FL_BmN-_06O01_R_0	BJ988116
E_FL_BmN-_06O02_F_0	BJ981903
E_FL_BmN-_06O02_R_0	BJ988117
E_FL_BmN-_06O03_F_0	BJ981904
E_FL_BmN-_06O03_R_0	BJ988118
E_FL_BmN-_06O04_F_0	BJ981905
E_FL_BmN-_06O04_R_0	BJ988119
E_FL_BmN-_06O05_F_0	BJ981906
E_FL_BmN-_06O05_R_0	BJ988120
E_FL_BmN-_06O06_R_0	BJ988121
E_FL_BmN-_06O07_R_0	BJ988122
E_FL_BmN-_06O08_R_0	BJ988123
E_FL_BmN-_06O09_F_0	BJ981907
E_FL_BmN-_06O10_F_0	BJ981908
E_FL_BmN-_06O10_R_0	BJ988124
E_FL_BmN-_06O11_F_0	BJ981909
E_FL_BmN-_06O12_F_0	BJ981910
E_FL_BmN-_06O12_R_0	BJ988125
E_FL_BmN-_06O14_F_0	BJ981911
E_FL_BmN-_06O14_R_0	BJ988126
E_FL_BmN-_06O15_F_0	BJ981912
E_FL_BmN-_06O15_R_0	BJ988127
E_FL_BmN-_06O17_F_0	BJ981913
E_FL_BmN-_06O19_R_0	BJ988128
E_FL_BmN-_06O20_F_0	BJ981914
E_FL_BmN-_06O20_R_0	BJ988129
E_FL_BmN-_06O21_F_0	BJ981915
E_FL_BmN-_06O21_R_0	BJ988130
E_FL_BmN-_06O22_F_0	BJ981916
E_FL_BmN-_06O22_R_0	BJ988131
E_FL_BmN-_06O23_F_0	BJ981917

E_FL_BmN-_06O23_R_0	BJ988132
E_FL_BmN-_06O24_R_0	BJ988133
E_FL_BmN-_06P01_F_0	BJ981918
E_FL_BmN-_06P01_R_0	BJ988134
E_FL_BmN-_06P02_F_0	BJ981919
E_FL_BmN-_06P02_R_0	BJ988135
E_FL_BmN-_06P03_F_0	BJ981920
E_FL_BmN-_06P03_R_0	BJ988136
E_FL_BmN-_06P04_F_0	BJ981921
E_FL_BmN-_06P04_R_0	BJ988137
E_FL_BmN-_06P05_R_0	BJ988138
E_FL_BmN-_06P06_F_0	BJ981922
E_FL_BmN-_06P06_R_0	BJ988139
E_FL_BmN-_06P07_F_0	BJ981923
E_FL_BmN-_06P07_R_0	BJ988140
E_FL_BmN-_06P08_F_0	BJ981924
E_FL_BmN-_06P08_R_0	BJ988141
E_FL_BmN-_06P09_F_0	BJ981925
E_FL_BmN-_06P09_R_0	BJ988142
E_FL_BmN-_06P10_R_0	BJ988143
E_FL_BmN-_06P12_F_0	BJ981926
E_FL_BmN-_06P12_R_0	BJ988144
E_FL_BmN-_06P13_F_0	BJ981927
E_FL_BmN-_06P13_R_0	BJ988145
E_FL_BmN-_06P14_F_0	BJ981928
E_FL_BmN-_06P14_R_0	BJ988146
E_FL_BmN-_06P15_F_0	BJ981929
E_FL_BmN-_06P15_R_0	BJ988147
E_FL_BmN-_06P17_R_0	BJ988148
E_FL_BmN-_06P18_F_0	BJ981930
E_FL_BmN-_06P18_R_0	BJ988149
E_FL_BmN-_06P19_F_0	BJ981931
E_FL_BmN-_06P20_F_0	BJ981932
E_FL_BmN-_06P20_R_0	BJ988150
E_FL_BmN-_06P21_R_0	BJ988151
E_FL_BmN-_06P22_F_0	BJ981933
E_FL_BmN-_06P22_R_0	BJ988152
E_FL_BmN-_06P23_F_0	BJ981934
E_FL_BmN-_06P23_R_0	BJ988153
E_FL_BmN-_06P24_R_0	BJ988154
E_FL_BmN-_07A02_F_0	BJ981935
E_FL_BmN-_07A03_F_0	BJ981936
E_FL_BmN-_07A03_R_0	BJ988155
E_FL_BmN-_07A04_F_0	BJ981937
E_FL_BmN-_07A05_F_0	BJ981938
E_FL_BmN-_07A05_R_0	BJ988156
E_FL_BmN-_07A06_F_0	BJ981939
E_FL_BmN-_07A06_R_0	BJ988157
E_FL_BmN-_07A07_F_0	BJ981940
E_FL_BmN-_07A08_R_0	BJ988158
E_FL_BmN-_07A10_F_0	BJ981941
E_FL_BmN-_07A10_R_0	BJ988159
E_FL_BmN-_07A11_F_0	BJ981942
E_FL_BmN-_07A11_R_0	BJ988160

E_FL_BmN-_07A12_F_0	BJ981943
E_FL_BmN-_07A12_R_0	BJ988161
E_FL_BmN-_07A13_F_0	BJ981944
E_FL_BmN-_07A14_F_0	BJ981945
E_FL_BmN-_07A14_R_0	BJ988162
E_FL_BmN-_07A15_F_0	BJ981946
E_FL_BmN-_07A15_R_0	BJ988163
E_FL_BmN-_07A16_F_0	BJ981947
E_FL_BmN-_07A16_R_0	BJ988164
E_FL_BmN-_07A17_F_0	BJ981948
E_FL_BmN-_07A17_R_0	BJ988165
E_FL_BmN-_07A18_F_0	BJ981949
E_FL_BmN-_07A18_R_0	BJ988166
E_FL_BmN-_07A19_F_0	BJ981950
E_FL_BmN-_07A20_F_0	BJ981951
E_FL_BmN-_07A20_R_0	BJ988167
E_FL_BmN-_07A21_R_0	BJ988168
E_FL_BmN-_07A22_F_0	BJ981952
E_FL_BmN-_07A23_R_0	BJ988169
E_FL_BmN-_07A24_R_0	BJ988170
E_FL_BmN-_07B01_F_0	BJ981953
E_FL_BmN-_07B01_R_0	BJ988171
E_FL_BmN-_07B02_R_0	BJ988172
E_FL_BmN-_07B03_F_0	BJ981954
E_FL_BmN-_07B03_R_0	BJ988173
E_FL_BmN-_07B04_F_0	BJ981955
E_FL_BmN-_07B04_R_0	BJ988174
E_FL_BmN-_07B05_F_0	BJ981956
E_FL_BmN-_07B05_R_0	BJ988175
E_FL_BmN-_07B06_F_0	BJ981957
E_FL_BmN-_07B06_R_0	BJ988176
E_FL_BmN-_07B07_F_0	BJ981958
E_FL_BmN-_07B07_R_0	BJ988177
E_FL_BmN-_07B08_F_0	BJ981959
E_FL_BmN-_07B08_R_0	BJ988178
E_FL_BmN-_07B09_R_0	BJ988179
E_FL_BmN-_07B10_R_0	BJ988180
E_FL_BmN-_07B12_R_0	BJ988181
E_FL_BmN-_07B13_F_0	BJ981960
E_FL_BmN-_07B13_R_0	BJ988182
E_FL_BmN-_07B15_F_0	BJ981961
E_FL_BmN-_07B15_R_0	BJ988183
E_FL_BmN-_07B17_F_0	BJ981962
E_FL_BmN-_07B17_R_0	BJ988184
E_FL_BmN-_07B18_F_0	BJ981963
E_FL_BmN-_07B19_F_0	BJ981964
E_FL_BmN-_07B20_F_0	BJ981965
E_FL_BmN-_07B20_R_0	BJ988185
E_FL_BmN-_07B21_F_0	BJ981966
E_FL_BmN-_07B21_R_0	BJ988186
E_FL_BmN-_07B22_F_0	BJ981967
E_FL_BmN-_07B22_R_0	BJ988187
E_FL_BmN-_07B23_F_0	BJ981968
E_FL_BmN-_07B23_R_0	BJ988188

E_FL_BmN-_07B24_F_0	BJ981969
E_FL_BmN-_07B24_R_0	BJ988189
E_FL_BmN-_07C01_F_0	BJ981970
E_FL_BmN-_07C01_R_0	BJ988190
E_FL_BmN-_07C03_F_0	BJ981971
E_FL_BmN-_07C03_R_0	BJ988191
E_FL_BmN-_07C04_F_0	BJ981972
E_FL_BmN-_07C04_R_0	BJ988192
E_FL_BmN-_07C05_F_0	BJ981973
E_FL_BmN-_07C05_R_0	BJ988193
E_FL_BmN-_07C07_F_0	BJ981974
E_FL_BmN-_07C07_R_0	BJ988194
E_FL_BmN-_07C08_F_0	BJ981975
E_FL_BmN-_07C08_R_0	BJ988195
E_FL_BmN-_07C09_F_0	BJ981976
E_FL_BmN-_07C09_R_0	BJ988196
E_FL_BmN-_07C10_F_0	BJ981977
E_FL_BmN-_07C10_R_0	BJ988197
E_FL_BmN-_07C11_F_0	BJ981978
E_FL_BmN-_07C11_R_0	BJ988198
E_FL_BmN-_07C12_R_0	BJ988199
E_FL_BmN-_07C13_F_0	BJ981979
E_FL_BmN-_07C13_R_0	BJ988200
E_FL_BmN-_07C14_F_0	BJ981980
E_FL_BmN-_07C14_R_0	BJ988201
E_FL_BmN-_07C15_F_0	BJ981981
E_FL_BmN-_07C15_R_0	BJ988202
E_FL_BmN-_07C16_F_0	BJ981982
E_FL_BmN-_07C16_R_0	BJ988203
E_FL_BmN-_07C17_F_0	BJ981983
E_FL_BmN-_07C17_R_0	BJ988204
E_FL_BmN-_07C18_R_0	BJ988205
E_FL_BmN-_07C19_F_0	BJ981984
E_FL_BmN-_07C19_R_0	BJ988206
E_FL_BmN-_07C20_F_0	BJ981985
E_FL_BmN-_07C20_R_0	BJ988207
E_FL_BmN-_07C21_F_0	BJ981986
E_FL_BmN-_07C22_F_0	BJ981987
E_FL_BmN-_07C22_R_0	BJ988208
E_FL_BmN-_07C23_F_0	BJ981988
E_FL_BmN-_07C23_R_0	BJ988209
E_FL_BmN-_07C24_R_0	BJ988210
E_FL_BmN-_07D01_F_0	BJ981989
E_FL_BmN-_07D01_R_0	BJ988211
E_FL_BmN-_07D02_F_0	BJ981990
E_FL_BmN-_07D03_F_0	BJ981991
E_FL_BmN-_07D03_R_0	BJ988212
E_FL_BmN-_07D04_F_0	BJ981992
E_FL_BmN-_07D04_R_0	BJ988213
E_FL_BmN-_07D05_F_0	BJ981993
E_FL_BmN-_07D05_R_0	BJ988214
E_FL_BmN-_07D06_F_0	BJ981994
E_FL_BmN-_07D06_R_0	BJ988215
E_FL_BmN-_07D07_F_0	BJ981995

E_FL_BmN-_07D07_R_0	BJ988216
E_FL_BmN-_07D08_R_0	BJ988217
E_FL_BmN-_07D09_F_0	BJ981996
E_FL_BmN-_07D10_F_0	BJ981997
E_FL_BmN-_07D11_F_0	BJ981998
E_FL_BmN-_07D11_R_0	BJ988218
E_FL_BmN-_07D12_R_0	BJ988219
E_FL_BmN-_07D13_F_0	BJ981999
E_FL_BmN-_07D13_R_0	BJ988220
E_FL_BmN-_07D14_R_0	BJ988221
E_FL_BmN-_07D15_F_0	BJ982000
E_FL_BmN-_07D15_R_0	BJ988222
E_FL_BmN-_07D17_R_0	BJ988223
E_FL_BmN-_07D20_F_0	BJ982001
E_FL_BmN-_07D20_R_0	BJ988224
E_FL_BmN-_07D22_F_0	BJ982002
E_FL_BmN-_07D23_F_0	BJ982003
E_FL_BmN-_07D23_R_0	BJ988225
E_FL_BmN-_07D24_F_0	BJ982004
E_FL_BmN-_07D24_R_0	BJ988226
E_FL_BmN-_07E01_F_0	BJ982005
E_FL_BmN-_07E01_R_0	BJ988227
E_FL_BmN-_07E02_F_0	BJ982006
E_FL_BmN-_07E02_R_0	BJ988228
E_FL_BmN-_07E03_F_0	BJ982007
E_FL_BmN-_07E03_R_0	BJ988229
E_FL_BmN-_07E04_F_0	BJ982008
E_FL_BmN-_07E04_R_0	BJ988230
E_FL_BmN-_07E06_F_0	BJ982009
E_FL_BmN-_07E07_F_0	BJ982010
E_FL_BmN-_07E07_R_0	BJ988231
E_FL_BmN-_07E08_F_0	BJ982011
E_FL_BmN-_07E08_R_0	BJ988232
E_FL_BmN-_07E09_F_0	BJ982012
E_FL_BmN-_07E09_R_0	BJ988233
E_FL_BmN-_07E10_F_0	BJ982013
E_FL_BmN-_07E10_R_0	BJ988234
E_FL_BmN-_07E11_F_0	BJ982014
E_FL_BmN-_07E11_R_0	BJ988235
E_FL_BmN-_07E12_F_0	BJ982015
E_FL_BmN-_07E12_R_0	BJ988236
E_FL_BmN-_07E14_F_0	BJ982016
E_FL_BmN-_07E15_F_0	BJ982017
E_FL_BmN-_07E15_R_0	BJ988237
E_FL_BmN-_07E16_F_0	BJ982018
E_FL_BmN-_07E16_R_0	BJ988238
E_FL_BmN-_07E17_F_0	BJ982019
E_FL_BmN-_07E18_F_0	BJ982020
E_FL_BmN-_07E18_R_0	BJ988239
E_FL_BmN-_07E19_F_0	BJ982021
E_FL_BmN-_07E19_R_0	BJ988240
E_FL_BmN-_07E20_F_0	BJ982022
E_FL_BmN-_07E20_R_0	BJ988241
E_FL_BmN-_07E21_F_0	BJ982023

E_FL_BmN-_07E21_R_0	BJ988242
E_FL_BmN-_07E22_F_0	BJ982024
E_FL_BmN-_07E22_R_0	BJ988243
E_FL_BmN-_07E23_F_0	BJ982025
E_FL_BmN-_07E23_R_0	BJ988244
E_FL_BmN-_07E24_F_0	BJ982026
E_FL_BmN-_07E24_R_0	BJ988245
E_FL_BmN-_07F01_F_0	BJ982027
E_FL_BmN-_07F01_R_0	BJ988246
E_FL_BmN-_07F02_F_0	BJ982028
E_FL_BmN-_07F02_R_0	BJ988247
E_FL_BmN-_07F03_R_0	BJ988248
E_FL_BmN-_07F04_F_0	BJ982029
E_FL_BmN-_07F04_R_0	BJ988249
E_FL_BmN-_07F06_F_0	BJ982030
E_FL_BmN-_07F06_R_0	BJ988250
E_FL_BmN-_07F07_F_0	BJ982031
E_FL_BmN-_07F07_R_0	BJ988251
E_FL_BmN-_07F08_F_0	BJ982032
E_FL_BmN-_07F08_R_0	BJ988252
E_FL_BmN-_07F09_R_0	BJ988253
E_FL_BmN-_07F11_R_0	BJ988254
E_FL_BmN-_07F13_F_0	BJ982033
E_FL_BmN-_07F13_R_0	BJ988255
E_FL_BmN-_07F14_R_0	BJ988256
E_FL_BmN-_07F15_F_0	BJ982034
E_FL_BmN-_07F15_R_0	BJ988257
E_FL_BmN-_07F16_F_0	BJ982035
E_FL_BmN-_07F16_R_0	BJ988258
E_FL_BmN-_07F17_F_0	BJ982036
E_FL_BmN-_07F17_R_0	BJ988259
E_FL_BmN-_07F18_R_0	BJ988260
E_FL_BmN-_07F19_F_0	BJ982037
E_FL_BmN-_07F20_R_0	BJ988261
E_FL_BmN-_07F21_R_0	BJ988262
E_FL_BmN-_07F22_F_0	BJ982038
E_FL_BmN-_07F22_R_0	BJ988263
E_FL_BmN-_07F23_F_0	BJ982039
E_FL_BmN-_07G01_R_0	BJ988264
E_FL_BmN-_07G02_F_0	BJ982040
E_FL_BmN-_07G03_F_0	BJ982041
E_FL_BmN-_07G03_R_0	BJ988265
E_FL_BmN-_07G04_F_0	BJ982042
E_FL_BmN-_07G04_R_0	BJ988266
E_FL_BmN-_07G05_R_0	BJ988267
E_FL_BmN-_07G06_F_0	BJ982043
E_FL_BmN-_07G06_R_0	BJ988268
E_FL_BmN-_07G07_F_0	BJ982044
E_FL_BmN-_07G07_R_0	BJ988269
E_FL_BmN-_07G08_F_0	BJ982045
E_FL_BmN-_07G08_R_0	BJ988270
E_FL_BmN-_07G09_F_0	BJ982046
E_FL_BmN-_07G09_R_0	BJ988271
E_FL_BmN-_07G10_F_0	BJ982047

E_FL_BmN-_07G10_R_0	BJ988272
E_FL_BmN-_07G11_F_0	BJ982048
E_FL_BmN-_07G11_R_0	BJ988273
E_FL_BmN-_07G12_F_0	BJ982049
E_FL_BmN-_07G13_F_0	BJ982050
E_FL_BmN-_07G14_F_0	BJ982051
E_FL_BmN-_07G15_F_0	BJ982052
E_FL_BmN-_07G15_R_0	BJ988274
E_FL_BmN-_07G16_F_0	BJ982053
E_FL_BmN-_07G16_R_0	BJ988275
E_FL_BmN-_07G17_F_0	BJ982054
E_FL_BmN-_07G18_F_0	BJ982055
E_FL_BmN-_07G18_R_0	BJ988276
E_FL_BmN-_07G19_F_0	BJ982056
E_FL_BmN-_07G20_R_0	BJ988277
E_FL_BmN-_07G21_F_0	BJ982057
E_FL_BmN-_07G23_F_0	BJ982058
E_FL_BmN-_07G23_R_0	BJ988278
E_FL_BmN-_07G24_F_0	BJ982059
E_FL_BmN-_07G24_R_0	BJ988279
E_FL_BmN-_07H01_F_0	BJ982060
E_FL_BmN-_07H01_R_0	BJ988280
E_FL_BmN-_07H03_F_0	BJ982061
E_FL_BmN-_07H03_R_0	BJ988281
E_FL_BmN-_07H04_F_0	BJ982062
E_FL_BmN-_07H04_R_0	BJ988282
E_FL_BmN-_07H05_F_0	BJ982063
E_FL_BmN-_07H05_R_0	BJ988283
E_FL_BmN-_07H06_R_0	BJ988284
E_FL_BmN-_07H07_F_0	BJ982064
E_FL_BmN-_07H07_R_0	BJ988285
E_FL_BmN-_07H08_R_0	BJ988286
E_FL_BmN-_07H09_F_0	BJ982065
E_FL_BmN-_07H09_R_0	BJ988287
E_FL_BmN-_07H10_F_0	BJ982066
E_FL_BmN-_07H10_R_0	BJ988288
E_FL_BmN-_07H11_F_0	BJ982067
E_FL_BmN-_07H11_R_0	BJ988289
E_FL_BmN-_07H12_F_0	BJ982068
E_FL_BmN-_07H12_R_0	BJ988290
E_FL_BmN-_07H13_F_0	BJ982069
E_FL_BmN-_07H14_R_0	BJ988291
E_FL_BmN-_07H15_F_0	BJ982070
E_FL_BmN-_07H15_R_0	BJ988292
E_FL_BmN-_07H17_F_0	BJ982071
E_FL_BmN-_07H17_R_0	BJ988293
E_FL_BmN-_07H18_R_0	BJ988294
E_FL_BmN-_07H19_F_0	BJ982072
E_FL_BmN-_07H21_F_0	BJ982073
E_FL_BmN-_07H21_R_0	BJ988295
E_FL_BmN-_07H22_F_0	BJ982074
E_FL_BmN-_07H22_R_0	BJ988296
E_FL_BmN-_07H23_F_0	BJ982075
E_FL_BmN-_07H23_R_0	BJ988297

E_FL_BmN-_07H24_F_0	BJ982076
E_FL_BmN-_07H24_R_0	BJ988298
E_FL_BmN-_07I02_R_0	BJ988299
E_FL_BmN-_07I03_F_0	BJ982077
E_FL_BmN-_07I03_R_0	BJ988300
E_FL_BmN-_07I04_F_0	BJ982078
E_FL_BmN-_07I04_R_0	BJ988301
E_FL_BmN-_07I05_F_0	BJ982079
E_FL_BmN-_07I05_R_0	BJ988302
E_FL_BmN-_07I06_R_0	BJ988303
E_FL_BmN-_07I07_R_0	BJ988304
E_FL_BmN-_07I08_F_0	BJ982080
E_FL_BmN-_07I08_R_0	BJ988305
E_FL_BmN-_07I09_F_0	BJ982081
E_FL_BmN-_07I09_R_0	BJ988306
E_FL_BmN-_07I10_F_0	BJ982082
E_FL_BmN-_07I10_R_0	BJ988307
E_FL_BmN-_07I11_F_0	BJ982083
E_FL_BmN-_07I11_R_0	BJ988308
E_FL_BmN-_07I12_R_0	BJ988309
E_FL_BmN-_07I14_F_0	BJ982084
E_FL_BmN-_07I14_R_0	BJ988310
E_FL_BmN-_07I15_F_0	BJ982085
E_FL_BmN-_07I15_R_0	BJ988311
E_FL_BmN-_07I16_F_0	BJ982086
E_FL_BmN-_07I16_R_0	BJ988312
E_FL_BmN-_07I17_F_0	BJ982087
E_FL_BmN-_07I17_R_0	BJ988313
E_FL_BmN-_07I18_F_0	BJ982088
E_FL_BmN-_07I18_R_0	BJ988314
E_FL_BmN-_07I19_R_0	BJ988315
E_FL_BmN-_07I20_R_0	BJ988316
E_FL_BmN-_07I21_F_0	BJ982089
E_FL_BmN-_07I21_R_0	BJ988317
E_FL_BmN-_07I22_F_0	BJ982090
E_FL_BmN-_07I22_R_0	BJ988318
E_FL_BmN-_07I23_F_0	BJ982091
E_FL_BmN-_07I23_R_0	BJ988319
E_FL_BmN-_07I24_F_0	BJ982092
E_FL_BmN-_07I24_R_0	BJ988320
E_FL_BmN-_07J01_F_0	BJ982093
E_FL_BmN-_07J01_R_0	BJ988321
E_FL_BmN-_07J03_F_0	BJ982094
E_FL_BmN-_07J03_R_0	BJ988322
E_FL_BmN-_07J04_F_0	BJ982095
E_FL_BmN-_07J04_R_0	BJ988323
E_FL_BmN-_07J05_F_0	BJ982096
E_FL_BmN-_07J05_R_0	BJ988324
E_FL_BmN-_07J06_F_0	BJ982097
E_FL_BmN-_07J06_R_0	BJ988325
E_FL_BmN-_07J07_F_0	BJ982098
E_FL_BmN-_07J07_R_0	BJ988326
E_FL_BmN-_07J08_F_0	BJ982099
E_FL_BmN-_07J09_F_0	BJ982100

E_FL_BmN-_07J09_R_0	BJ988327
E_FL_BmN-_07J10_R_0	BJ988328
E_FL_BmN-_07J11_F_0	BJ982101
E_FL_BmN-_07J11_R_0	BJ988329
E_FL_BmN-_07J12_F_0	BJ982102
E_FL_BmN-_07J12_R_0	BJ988330
E_FL_BmN-_07J13_F_0	BJ982103
E_FL_BmN-_07J14_F_0	BJ982104
E_FL_BmN-_07J14_R_0	BJ988331
E_FL_BmN-_07J15_F_0	BJ982105
E_FL_BmN-_07J16_F_0	BJ982106
E_FL_BmN-_07J16_R_0	BJ988332
E_FL_BmN-_07J17_F_0	BJ982107
E_FL_BmN-_07J17_R_0	BJ988333
E_FL_BmN-_07J18_R_0	BJ988334
E_FL_BmN-_07J19_F_0	BJ982108
E_FL_BmN-_07J21_F_0	BJ982109
E_FL_BmN-_07J21_R_0	BJ988335
E_FL_BmN-_07J22_F_0	BJ982110
E_FL_BmN-_07J23_F_0	BJ982111
E_FL_BmN-_07J23_R_0	BJ988336
E_FL_BmN-_07J24_F_0	BJ982112
E_FL_BmN-_07J24_R_0	BJ988337
E_FL_BmN-_07K01_F_0	BJ982113
E_FL_BmN-_07K02_F_0	BJ982114
E_FL_BmN-_07K02_R_0	BJ988338
E_FL_BmN-_07K03_R_0	BJ988339
E_FL_BmN-_07K04_F_0	BJ982115
E_FL_BmN-_07K04_R_0	BJ988340
E_FL_BmN-_07K05_F_0	BJ982116
E_FL_BmN-_07K05_R_0	BJ988341
E_FL_BmN-_07K06_F_0	BJ982117
E_FL_BmN-_07K06_R_0	BJ988342
E_FL_BmN-_07K07_F_0	BJ982118
E_FL_BmN-_07K07_R_0	BJ988343
E_FL_BmN-_07K08_F_0	BJ982119
E_FL_BmN-_07K08_R_0	BJ988344
E_FL_BmN-_07K09_F_0	BJ982120
E_FL_BmN-_07K09_R_0	BJ988345
E_FL_BmN-_07K10_F_0	BJ982121
E_FL_BmN-_07K11_F_0	BJ982122
E_FL_BmN-_07K11_R_0	BJ988346
E_FL_BmN-_07K12_F_0	BJ982123
E_FL_BmN-_07K12_R_0	BJ988347
E_FL_BmN-_07K13_F_0	BJ982124
E_FL_BmN-_07K13_R_0	BJ988348
E_FL_BmN-_07K14_F_0	BJ982125
E_FL_BmN-_07K14_R_0	BJ988349
E_FL_BmN-_07K15_F_0	BJ982126
E_FL_BmN-_07K15_R_0	BJ988350
E_FL_BmN-_07K16_F_0	BJ982127
E_FL_BmN-_07K16_R_0	BJ988351
E_FL_BmN-_07K17_R_0	BJ988352
E_FL_BmN-_07K18_R_0	BJ988353

E_FL_BmN-_07K19_F_0	BJ982128
E_FL_BmN-_07K19_R_0	BJ988354
E_FL_BmN-_07K20_F_0	BJ982129
E_FL_BmN-_07K20_R_0	BJ988355
E_FL_BmN-_07K21_F_0	BJ982130
E_FL_BmN-_07K21_R_0	BJ988356
E_FL_BmN-_07K22_F_0	BJ982131
E_FL_BmN-_07K22_R_0	BJ988357
E_FL_BmN-_07K23_F_0	BJ982132
E_FL_BmN-_07K23_R_0	BJ988358
E_FL_BmN-_07K24_F_0	BJ982133
E_FL_BmN-_07K24_R_0	BJ988359
E_FL_BmN-_07L01_F_0	BJ982134
E_FL_BmN-_07L01_R_0	BJ988360
E_FL_BmN-_07L02_F_0	BJ982135
E_FL_BmN-_07L02_R_0	BJ988361
E_FL_BmN-_07L03_F_0	BJ982136
E_FL_BmN-_07L03_R_0	BJ988362
E_FL_BmN-_07L04_F_0	BJ982137
E_FL_BmN-_07L05_R_0	BJ988363
E_FL_BmN-_07L06_F_0	BJ982138
E_FL_BmN-_07L07_F_0	BJ982139
E_FL_BmN-_07L07_R_0	BJ988364
E_FL_BmN-_07L08_F_0	BJ982140
E_FL_BmN-_07L08_R_0	BJ988365
E_FL_BmN-_07L09_F_0	BJ982141
E_FL_BmN-_07L09_R_0	BJ988366
E_FL_BmN-_07L10_F_0	BJ982142
E_FL_BmN-_07L10_R_0	BJ988367
E_FL_BmN-_07L11_F_0	BJ982143
E_FL_BmN-_07L11_R_0	BJ988368
E_FL_BmN-_07L12_F_0	BJ982144
E_FL_BmN-_07L13_F_0	BJ982145
E_FL_BmN-_07L13_R_0	BJ988369
E_FL_BmN-_07L14_R_0	BJ988370
E_FL_BmN-_07L15_F_0	BJ982146
E_FL_BmN-_07L15_R_0	BJ988371
E_FL_BmN-_07L16_F_0	BJ982147
E_FL_BmN-_07L16_R_0	BJ988372
E_FL_BmN-_07L18_F_0	BJ982148
E_FL_BmN-_07L19_F_0	BJ982149
E_FL_BmN-_07L19_R_0	BJ988373
E_FL_BmN-_07L20_R_0	BJ988374
E_FL_BmN-_07L21_R_0	BJ988375
E_FL_BmN-_07L22_F_0	BJ982150
E_FL_BmN-_07L22_R_0	BJ988376
E_FL_BmN-_07L23_F_0	BJ982151
E_FL_BmN-_07L23_R_0	BJ988377
E_FL_BmN-_07L24_F_0	BJ982152
E_FL_BmN-_07M01_F_0	BJ982153
E_FL_BmN-_07M03_F_0	BJ982154
E_FL_BmN-_07M03_R_0	BJ988378
E_FL_BmN-_07M04_F_0	BJ982155
E_FL_BmN-_07M05_R_0	BJ988379

E_FL_BmN-_07M06_F_0	BJ982156
E_FL_BmN-_07M06_R_0	BJ988380
E_FL_BmN-_07M07_F_0	BJ982157
E_FL_BmN-_07M07_R_0	BJ988381
E_FL_BmN-_07M08_F_0	BJ982158
E_FL_BmN-_07M08_R_0	BJ988382
E_FL_BmN-_07M09_F_0	BJ982159
E_FL_BmN-_07M09_R_0	BJ988383
E_FL_BmN-_07M10_F_0	BJ982160
E_FL_BmN-_07M10_R_0	BJ988384
E_FL_BmN-_07M11_F_0	BJ982161
E_FL_BmN-_07M11_R_0	BJ988385
E_FL_BmN-_07M12_F_0	BJ982162
E_FL_BmN-_07M12_R_0	BJ988386
E_FL_BmN-_07M13_F_0	BJ982163
E_FL_BmN-_07M13_R_0	BJ988387
E_FL_BmN-_07M14_F_0	BJ982164
E_FL_BmN-_07M14_R_0	BJ988388
E_FL_BmN-_07M15_F_0	BJ982165
E_FL_BmN-_07M16_F_0	BJ982166
E_FL_BmN-_07M16_R_0	BJ988389
E_FL_BmN-_07M17_F_0	BJ982167
E_FL_BmN-_07M17_R_0	BJ988390
E_FL_BmN-_07M18_F_0	BJ982168
E_FL_BmN-_07M18_R_0	BJ988391
E_FL_BmN-_07M19_F_0	BJ982169
E_FL_BmN-_07M19_R_0	BJ988392
E_FL_BmN-_07M20_F_0	BJ982170
E_FL_BmN-_07M20_R_0	BJ988393
E_FL_BmN-_07M21_F_0	BJ982171
E_FL_BmN-_07M21_R_0	BJ988394
E_FL_BmN-_07M22_F_0	BJ982172
E_FL_BmN-_07M22_R_0	BJ988395
E_FL_BmN-_07M23_F_0	BJ982173
E_FL_BmN-_07M23_R_0	BJ988396
E_FL_BmN-_07M24_F_0	BJ982174
E_FL_BmN-_07M24_R_0	BJ988397
E_FL_BmN-_07N01_F_0	BJ982175
E_FL_BmN-_07N01_R_0	BJ988398
E_FL_BmN-_07N02_F_0	BJ982176
E_FL_BmN-_07N02_R_0	BJ988399
E_FL_BmN-_07N03_F_0	BJ982177
E_FL_BmN-_07N04_F_0	BJ982178
E_FL_BmN-_07N04_R_0	BJ988400
E_FL_BmN-_07N05_R_0	BJ988401
E_FL_BmN-_07N06_F_0	BJ982179
E_FL_BmN-_07N06_R_0	BJ988402
E_FL_BmN-_07N07_F_0	BJ982180
E_FL_BmN-_07N07_R_0	BJ988403
E_FL_BmN-_07N08_F_0	BJ982181
E_FL_BmN-_07N08_R_0	BJ988404
E_FL_BmN-_07N09_F_0	BJ982182
E_FL_BmN-_07N09_R_0	BJ988405
E_FL_BmN-_07N10_F_0	BJ982183

E_FL_BmN-_07N10_R_0	BJ988406
E_FL_BmN-_07N11_F_0	BJ982184
E_FL_BmN-_07N11_R_0	BJ988407
E_FL_BmN-_07N12_R_0	BJ988408
E_FL_BmN-_07N13_F_0	BJ982185
E_FL_BmN-_07N14_F_0	BJ982186
E_FL_BmN-_07N14_R_0	BJ988409
E_FL_BmN-_07N15_F_0	BJ982187
E_FL_BmN-_07N15_R_0	BJ988410
E_FL_BmN-_07N16_F_0	BJ982188
E_FL_BmN-_07N16_R_0	BJ988411
E_FL_BmN-_07N17_R_0	BJ988412
E_FL_BmN-_07N18_R_0	BJ988413
E_FL_BmN-_07N19_F_0	BJ982189
E_FL_BmN-_07N19_R_0	BJ988414
E_FL_BmN-_07N20_F_0	BJ982190
E_FL_BmN-_07N22_F_0	BJ982191
E_FL_BmN-_07N22_R_0	BJ988415
E_FL_BmN-_07N23_F_0	BJ982192
E_FL_BmN-_07N23_R_0	BJ988416
E_FL_BmN-_07O01_F_0	BJ982193
E_FL_BmN-_07O01_R_0	BJ988417
E_FL_BmN-_07O02_F_0	BJ982194
E_FL_BmN-_07O03_F_0	BJ982195
E_FL_BmN-_07O03_R_0	BJ988418
E_FL_BmN-_07O06_R_0	BJ988419
E_FL_BmN-_07O07_F_0	BJ982196
E_FL_BmN-_07O07_R_0	BJ988420
E_FL_BmN-_07O08_F_0	BJ982197
E_FL_BmN-_07O08_R_0	BJ988421
E_FL_BmN-_07O09_F_0	BJ982198
E_FL_BmN-_07O09_R_0	BJ988422
E_FL_BmN-_07O10_F_0	BJ982199
E_FL_BmN-_07O11_R_0	BJ988423
E_FL_BmN-_07O12_R_0	BJ988424
E_FL_BmN-_07O13_F_0	BJ982200
E_FL_BmN-_07O13_R_0	BJ988425
E_FL_BmN-_07O14_F_0	BJ982201
E_FL_BmN-_07O14_R_0	BJ988426
E_FL_BmN-_07O16_F_0	BJ982202
E_FL_BmN-_07O16_R_0	BJ988427
E_FL_BmN-_07O17_R_0	BJ988428
E_FL_BmN-_07O18_R_0	BJ988429
E_FL_BmN-_07O19_R_0	BJ988430
E_FL_BmN-_07O20_F_0	BJ982203
E_FL_BmN-_07O20_R_0	BJ988431
E_FL_BmN-_07O21_F_0	BJ982204
E_FL_BmN-_07O21_R_0	BJ988432
E_FL_BmN-_07O22_F_0	BJ982205
E_FL_BmN-_07O22_R_0	BJ988433
E_FL_BmN-_07O23_F_0	BJ982206
E_FL_BmN-_07O23_R_0	BJ988434
E_FL_BmN-_07P01_F_0	BJ982207
E_FL_BmN-_07P01_R_0	BJ988435

E_FL_BmN-_07P02_F_0	BJ982208
E_FL_BmN-_07P03_F_0	BJ982209
E_FL_BmN-_07P03_R_0	BJ988436
E_FL_BmN-_07P04_F_0	BJ982210
E_FL_BmN-_07P04_R_0	BJ988437
E_FL_BmN-_07P05_R_0	BJ988438
E_FL_BmN-_07P06_F_0	BJ982211
E_FL_BmN-_07P06_R_0	BJ988439
E_FL_BmN-_07P07_F_0	BJ982212
E_FL_BmN-_07P07_R_0	BJ988440
E_FL_BmN-_07P09_F_0	BJ982213
E_FL_BmN-_07P09_R_0	BJ988441
E_FL_BmN-_07P10_R_0	BJ988442
E_FL_BmN-_07P11_R_0	BJ988443
E_FL_BmN-_07P12_R_0	BJ988444
E_FL_BmN-_07P13_R_0	BJ988445
E_FL_BmN-_07P14_F_0	BJ982214
E_FL_BmN-_07P15_F_0	BJ982215
E_FL_BmN-_07P15_R_0	BJ988446
E_FL_BmN-_07P16_F_0	BJ982216
E_FL_BmN-_07P16_R_0	BJ988447
E_FL_BmN-_07P17_R_0	BJ988448
E_FL_BmN-_07P18_R_0	BJ988449
E_FL_BmN-_07P19_R_0	BJ988450
E_FL_BmN-_07P20_F_0	BJ982217
E_FL_BmN-_07P20_R_0	BJ988451
E_FL_BmN-_07P21_F_0	BJ982218
E_FL_BmN-_07P21_R_0	BJ988452
E_FL_BmN-_07P22_F_0	BJ982219
E_FL_BmN-_07P22_R_0	BJ988453
E_FL_BmN-_08A01_F_0	BJ982220
E_FL_BmN-_08A01_R_0	BJ988454
E_FL_BmN-_08A03_F_0	BJ982221
E_FL_BmN-_08A03_R_0	BJ988455
E_FL_BmN-_08A04_F_0	BJ982222
E_FL_BmN-_08A04_R_0	BJ988456
E_FL_BmN-_08A05_F_0	BJ982223
E_FL_BmN-_08A05_R_0	BJ988457
E_FL_BmN-_08A06_F_0	BJ982224
E_FL_BmN-_08A06_R_0	BJ988458
E_FL_BmN-_08A07_F_0	BJ982225
E_FL_BmN-_08A07_R_0	BJ988459
E_FL_BmN-_08A09_F_0	BJ982226
E_FL_BmN-_08A09_R_0	BJ988460
E_FL_BmN-_08A10_F_0	BJ982227
E_FL_BmN-_08A10_R_0	BJ988461
E_FL_BmN-_08A11_F_0	BJ982228
E_FL_BmN-_08A11_R_0	BJ988462
E_FL_BmN-_08A12_F_0	BJ982229
E_FL_BmN-_08A12_R_0	BJ988463
E_FL_BmN-_08A13_F_0	BJ982230
E_FL_BmN-_08A14_F_0	BJ982231
E_FL_BmN-_08A14_R_0	BJ988464
E_FL_BmN-_08A15_F_0	BJ982232

E_FL_BmN-_08A15_R_0	BJ988465
E_FL_BmN-_08A16_F_0	BJ982233
E_FL_BmN-_08A17_F_0	BJ982234
E_FL_BmN-_08A17_R_0	BJ988466
E_FL_BmN-_08A18_F_0	BJ982235
E_FL_BmN-_08A19_F_0	BJ982236
E_FL_BmN-_08A19_R_0	BJ988467
E_FL_BmN-_08A21_F_0	BJ982237
E_FL_BmN-_08A22_F_0	BJ982238
E_FL_BmN-_08A22_R_0	BJ988468
E_FL_BmN-_08A23_F_0	BJ982239
E_FL_BmN-_08A23_R_0	BJ988469
E_FL_BmN-_08A24_F_0	BJ982240
E_FL_BmN-_08A24_R_0	BJ988470
E_FL_BmN-_08B01_F_0	BJ982241
E_FL_BmN-_08B01_R_0	BJ988471
E_FL_BmN-_08B02_F_0	BJ982242
E_FL_BmN-_08B02_R_0	BJ988472
E_FL_BmN-_08B03_F_0	BJ982243
E_FL_BmN-_08B03_R_0	BJ988473
E_FL_BmN-_08B04_F_0	BJ982244
E_FL_BmN-_08B04_R_0	BJ988474
E_FL_BmN-_08B06_R_0	BJ988475
E_FL_BmN-_08B07_F_0	BJ982245
E_FL_BmN-_08B07_R_0	BJ988476
E_FL_BmN-_08B09_F_0	BJ982246
E_FL_BmN-_08B09_R_0	BJ988477
E_FL_BmN-_08B10_F_0	BJ982247
E_FL_BmN-_08B10_R_0	BJ988478
E_FL_BmN-_08B11_F_0	BJ982248
E_FL_BmN-_08B13_F_0	BJ982249
E_FL_BmN-_08B13_R_0	BJ988479
E_FL_BmN-_08B14_F_0	BJ982250
E_FL_BmN-_08B15_F_0	BJ982251
E_FL_BmN-_08B15_R_0	BJ988480
E_FL_BmN-_08B16_F_0	BJ982252
E_FL_BmN-_08B16_R_0	BJ988481
E_FL_BmN-_08B17_R_0	BJ988482
E_FL_BmN-_08B18_F_0	BJ982253
E_FL_BmN-_08B18_R_0	BJ988483
E_FL_BmN-_08B19_F_0	BJ982254
E_FL_BmN-_08B19_R_0	BJ988484
E_FL_BmN-_08B20_F_0	BJ982255
E_FL_BmN-_08B20_R_0	BJ988485
E_FL_BmN-_08B21_R_0	BJ988486
E_FL_BmN-_08B22_F_0	BJ982256
E_FL_BmN-_08B22_R_0	BJ988487
E_FL_BmN-_08B23_F_0	BJ982257
E_FL_BmN-_08B23_R_0	BJ988488
E_FL_BmN-_08B24_F_0	BJ982258
E_FL_BmN-_08C01_F_0	BJ982259
E_FL_BmN-_08C01_R_0	BJ988489
E_FL_BmN-_08C02_F_0	BJ982260
E_FL_BmN-_08C02_R_0	BJ988490

E_FL_BmN-_08C03_F_0	BJ982261
E_FL_BmN-_08C03_R_0	BJ988491
E_FL_BmN-_08C04_F_0	BJ982262
E_FL_BmN-_08C04_R_0	BJ988492
E_FL_BmN-_08C06_R_0	BJ988493
E_FL_BmN-_08C08_F_0	BJ982263
E_FL_BmN-_08C08_R_0	BJ988494
E_FL_BmN-_08C09_F_0	BJ982264
E_FL_BmN-_08C09_R_0	BJ988495
E_FL_BmN-_08C10_F_0	BJ982265
E_FL_BmN-_08C10_R_0	BJ988496
E_FL_BmN-_08C11_F_0	BJ982266
E_FL_BmN-_08C11_R_0	BJ988497
E_FL_BmN-_08C12_R_0	BJ988498
E_FL_BmN-_08C13_F_0	BJ982267
E_FL_BmN-_08C13_R_0	BJ988499
E_FL_BmN-_08C15_F_0	BJ982268
E_FL_BmN-_08C15_R_0	BJ988500
E_FL_BmN-_08C16_F_0	BJ982269
E_FL_BmN-_08C16_R_0	BJ988501
E_FL_BmN-_08C17_R_0	BJ988502
E_FL_BmN-_08C18_R_0	BJ988503
E_FL_BmN-_08C19_F_0	BJ982270
E_FL_BmN-_08C19_R_0	BJ988504
E_FL_BmN-_08C20_R_0	BJ988505
E_FL_BmN-_08C21_F_0	BJ982271
E_FL_BmN-_08C21_R_0	BJ988506
E_FL_BmN-_08C22_F_0	BJ982272
E_FL_BmN-_08C22_R_0	BJ988507
E_FL_BmN-_08C23_F_0	BJ982273
E_FL_BmN-_08C23_R_0	BJ988508
E_FL_BmN-_08C24_R_0	BJ988509
E_FL_BmN-_08D01_F_0	BJ982274
E_FL_BmN-_08D02_F_0	BJ982275
E_FL_BmN-_08D02_R_0	BJ988510
E_FL_BmN-_08D03_F_0	BJ982276
E_FL_BmN-_08D04_F_0	BJ982277
E_FL_BmN-_08D05_F_0	BJ982278
E_FL_BmN-_08D06_R_0	BJ988511
E_FL_BmN-_08D07_F_0	BJ982279
E_FL_BmN-_08D07_R_0	BJ988512
E_FL_BmN-_08D08_F_0	BJ982280
E_FL_BmN-_08D08_R_0	BJ988513
E_FL_BmN-_08D09_F_0	BJ982281
E_FL_BmN-_08D09_R_0	BJ988514
E_FL_BmN-_08D11_R_0	BJ988515
E_FL_BmN-_08D13_R_0	BJ988516
E_FL_BmN-_08D14_R_0	BJ988517
E_FL_BmN-_08D15_F_0	BJ982282
E_FL_BmN-_08D15_R_0	BJ988518
E_FL_BmN-_08D16_F_0	BJ982283
E_FL_BmN-_08D16_R_0	BJ988519
E_FL_BmN-_08D17_F_0	BJ982284
E_FL_BmN-_08D19_F_0	BJ982285

E_FL_BmN-_08D19_R_0	BJ988520
E_FL_BmN-_08D20_F_0	BJ982286
E_FL_BmN-_08D21_F_0	BJ982287
E_FL_BmN-_08D21_R_0	BJ988521
E_FL_BmN-_08D22_F_0	BJ982288
E_FL_BmN-_08D22_R_0	BJ988522
E_FL_BmN-_08D23_F_0	BJ982289
E_FL_BmN-_08D23_R_0	BJ988523
E_FL_BmN-_08D24_F_0	BJ982290
E_FL_BmN-_08D24_R_0	BJ988524
E_FL_BmN-_08E02_F_0	BJ982291
E_FL_BmN-_08E02_R_0	BJ988525
E_FL_BmN-_08E03_F_0	BJ982292
E_FL_BmN-_08E03_R_0	BJ988526
E_FL_BmN-_08E04_F_0	BJ982293
E_FL_BmN-_08E04_R_0	BJ988527
E_FL_BmN-_08E05_F_0	BJ982294
E_FL_BmN-_08E05_R_0	BJ988528
E_FL_BmN-_08E06_F_0	BJ982295
E_FL_BmN-_08E06_R_0	BJ988529
E_FL_BmN-_08E08_R_0	BJ988530
E_FL_BmN-_08E09_F_0	BJ982296
E_FL_BmN-_08E09_R_0	BJ988531
E_FL_BmN-_08E11_F_0	BJ982297
E_FL_BmN-_08E11_R_0	BJ988532
E_FL_BmN-_08E12_R_0	BJ988533
E_FL_BmN-_08E13_F_0	BJ982298
E_FL_BmN-_08E13_R_0	BJ988534
E_FL_BmN-_08E14_R_0	BJ988535
E_FL_BmN-_08E15_F_0	BJ982299
E_FL_BmN-_08E15_R_0	BJ988536
E_FL_BmN-_08E16_F_0	BJ982300
E_FL_BmN-_08E17_F_0	BJ982301
E_FL_BmN-_08E17_R_0	BJ988537
E_FL_BmN-_08E18_F_0	BJ982302
E_FL_BmN-_08E19_F_0	BJ982303
E_FL_BmN-_08E19_R_0	BJ988538
E_FL_BmN-_08E20_R_0	BJ988539
E_FL_BmN-_08E21_F_0	BJ982304
E_FL_BmN-_08E21_R_0	BJ988540
E_FL_BmN-_08E22_F_0	BJ982305
E_FL_BmN-_08E22_R_0	BJ988541
E_FL_BmN-_08E23_F_0	BJ982306
E_FL_BmN-_08E23_R_0	BJ988542
E_FL_BmN-_08E24_F_0	BJ982307
E_FL_BmN-_08E24_R_0	BJ988543
E_FL_BmN-_08F01_F_0	BJ982308
E_FL_BmN-_08F01_R_0	BJ988544
E_FL_BmN-_08F02_F_0	BJ982309
E_FL_BmN-_08F02_R_0	BJ988545
E_FL_BmN-_08F03_F_0	BJ982310
E_FL_BmN-_08F03_R_0	BJ988546
E_FL_BmN-_08F04_F_0	BJ982311
E_FL_BmN-_08F04_R_0	BJ988547

E_FL_BmN-_08F05_R_0	BJ988548
E_FL_BmN-_08F06_R_0	BJ988549
E_FL_BmN-_08F07_F_0	BJ982312
E_FL_BmN-_08F07_R_0	BJ988550
E_FL_BmN-_08F08_F_0	BJ982313
E_FL_BmN-_08F09_R_0	BJ988551
E_FL_BmN-_08F10_R_0	BJ988552
E_FL_BmN-_08F11_F_0	BJ982314
E_FL_BmN-_08F11_R_0	BJ988553
E_FL_BmN-_08F12_R_0	BJ988554
E_FL_BmN-_08F13_R_0	BJ988555
E_FL_BmN-_08F15_F_0	BJ982315
E_FL_BmN-_08F15_R_0	BJ988556
E_FL_BmN-_08F16_F_0	BJ982316
E_FL_BmN-_08F16_R_0	BJ988557
E_FL_BmN-_08F17_F_0	BJ982317
E_FL_BmN-_08F17_R_0	BJ988558
E_FL_BmN-_08F18_R_0	BJ988559
E_FL_BmN-_08F19_F_0	BJ982318
E_FL_BmN-_08F19_R_0	BJ988560
E_FL_BmN-_08F20_R_0	BJ988561
E_FL_BmN-_08F21_R_0	BJ988562
E_FL_BmN-_08F22_F_0	BJ982319
E_FL_BmN-_08F22_R_0	BJ988563
E_FL_BmN-_08F23_F_0	BJ982320
E_FL_BmN-_08F23_R_0	BJ988564
E_FL_BmN-_08F24_F_0	BJ982321
E_FL_BmN-_08F24_R_0	BJ988565
E_FL_BmN-_08G02_F_0	BJ982322
E_FL_BmN-_08G02_R_0	BJ988566
E_FL_BmN-_08G03_F_0	BJ982323
E_FL_BmN-_08G03_R_0	BJ988567
E_FL_BmN-_08G04_F_0	BJ982324
E_FL_BmN-_08G04_R_0	BJ988568
E_FL_BmN-_08G06_F_0	BJ982325
E_FL_BmN-_08G06_R_0	BJ988569
E_FL_BmN-_08G07_F_0	BJ982326
E_FL_BmN-_08G08_F_0	BJ982327
E_FL_BmN-_08G08_R_0	BJ988570
E_FL_BmN-_08G09_F_0	BJ982328
E_FL_BmN-_08G09_R_0	BJ988571
E_FL_BmN-_08G11_F_0	BJ982329
E_FL_BmN-_08G11_R_0	BJ988572
E_FL_BmN-_08G13_F_0	BJ982330
E_FL_BmN-_08G13_R_0	BJ988573
E_FL_BmN-_08G14_F_0	BJ982331
E_FL_BmN-_08G14_R_0	BJ988574
E_FL_BmN-_08G15_F_0	BJ982332
E_FL_BmN-_08G15_R_0	BJ988575
E_FL_BmN-_08G16_F_0	BJ982333
E_FL_BmN-_08G16_R_0	BJ988576
E_FL_BmN-_08G17_F_0	BJ982334
E_FL_BmN-_08G17_R_0	BJ988577
E_FL_BmN-_08G18_F_0	BJ982335

E_FL_BmN-_08G18_R_0	BJ988578
E_FL_BmN-_08G19_F_0	BJ982336
E_FL_BmN-_08G19_R_0	BJ988579
E_FL_BmN-_08G20_F_0	BJ982337
E_FL_BmN-_08G20_R_0	BJ988580
E_FL_BmN-_08G21_F_0	BJ982338
E_FL_BmN-_08G21_R_0	BJ988581
E_FL_BmN-_08G22_F_0	BJ982339
E_FL_BmN-_08G24_F_0	BJ982340
E_FL_BmN-_08G24_R_0	BJ988582
E_FL_BmN-_08H01_F_0	BJ982341
E_FL_BmN-_08H02_F_0	BJ982342
E_FL_BmN-_08H02_R_0	BJ988583
E_FL_BmN-_08H03_F_0	BJ982343
E_FL_BmN-_08H03_R_0	BJ988584
E_FL_BmN-_08H04_F_0	BJ982344
E_FL_BmN-_08H05_R_0	BJ988585
E_FL_BmN-_08H06_R_0	BJ988586
E_FL_BmN-_08H07_F_0	BJ982345
E_FL_BmN-_08H07_R_0	BJ988587
E_FL_BmN-_08H08_R_0	BJ988588
E_FL_BmN-_08H09_R_0	BJ988589
E_FL_BmN-_08H11_F_0	BJ982346
E_FL_BmN-_08H12_R_0	BJ988590
E_FL_BmN-_08H13_F_0	BJ982347
E_FL_BmN-_08H13_R_0	BJ988591
E_FL_BmN-_08H14_F_0	BJ982348
E_FL_BmN-_08H14_R_0	BJ988592
E_FL_BmN-_08H15_F_0	BJ982349
E_FL_BmN-_08H15_R_0	BJ988593
E_FL_BmN-_08H17_F_0	BJ982350
E_FL_BmN-_08H17_R_0	BJ988594
E_FL_BmN-_08H19_F_0	BJ982351
E_FL_BmN-_08H19_R_0	BJ988595
E_FL_BmN-_08H20_R_0	BJ988596
E_FL_BmN-_08H21_F_0	BJ982352
E_FL_BmN-_08H21_R_0	BJ988597
E_FL_BmN-_08H22_F_0	BJ982353
E_FL_BmN-_08H22_R_0	BJ988598
E_FL_BmN-_08H24_F_0	BJ982354
E_FL_BmN-_08H24_R_0	BJ988599
E_FL_BmN-_08I01_F_0	BJ982355
E_FL_BmN-_08I01_R_0	BJ988600
E_FL_BmN-_08I02_F_0	BJ982356
E_FL_BmN-_08I02_R_0	BJ988601
E_FL_BmN-_08I03_F_0	BJ982357
E_FL_BmN-_08I03_R_0	BJ988602
E_FL_BmN-_08I04_F_0	BJ982358
E_FL_BmN-_08I04_R_0	BJ988603
E_FL_BmN-_08I05_F_0	BJ982359
E_FL_BmN-_08I05_R_0	BJ988604
E_FL_BmN-_08I06_F_0	BJ982360
E_FL_BmN-_08I06_R_0	BJ988605
E_FL_BmN-_08I07_F_0	BJ982361

E_FL_BmN-_08I08_F_0	BJ982362
E_FL_BmN-_08I08_R_0	BJ988606
E_FL_BmN-_08I09_F_0	BJ982363
E_FL_BmN-_08I10_F_0	BJ982364
E_FL_BmN-_08I10_R_0	BJ988607
E_FL_BmN-_08I11_F_0	BJ982365
E_FL_BmN-_08I11_R_0	BJ988608
E_FL_BmN-_08I12_R_0	BJ988609
E_FL_BmN-_08I14_R_0	BJ988610
E_FL_BmN-_08I15_F_0	BJ982366
E_FL_BmN-_08I16_F_0	BJ982367
E_FL_BmN-_08I16_R_0	BJ988611
E_FL_BmN-_08I17_F_0	BJ982368
E_FL_BmN-_08I17_R_0	BJ988612
E_FL_BmN-_08I18_R_0	BJ988613
E_FL_BmN-_08I19_F_0	BJ982369
E_FL_BmN-_08I19_R_0	BJ988614
E_FL_BmN-_08I20_F_0	BJ982370
E_FL_BmN-_08I20_R_0	BJ988615
E_FL_BmN-_08I21_F_0	BJ982371
E_FL_BmN-_08I21_R_0	BJ988616
E_FL_BmN-_08I22_F_0	BJ982372
E_FL_BmN-_08I22_R_0	BJ988617
E_FL_BmN-_08I24_R_0	BJ988618
E_FL_BmN-_08J01_F_0	BJ982373
E_FL_BmN-_08J01_R_0	BJ988619
E_FL_BmN-_08J02_R_0	BJ988620
E_FL_BmN-_08J03_F_0	BJ982374
E_FL_BmN-_08J03_R_0	BJ988621
E_FL_BmN-_08J04_F_0	BJ982375
E_FL_BmN-_08J04_R_0	BJ988622
E_FL_BmN-_08J05_F_0	BJ982376
E_FL_BmN-_08J05_R_0	BJ988623
E_FL_BmN-_08J06_F_0	BJ982377
E_FL_BmN-_08J06_R_0	BJ988624
E_FL_BmN-_08J07_F_0	BJ982378
E_FL_BmN-_08J07_R_0	BJ988625
E_FL_BmN-_08J08_F_0	BJ982379
E_FL_BmN-_08J08_R_0	BJ988626
E_FL_BmN-_08J09_R_0	BJ988627
E_FL_BmN-_08J10_F_0	BJ982380
E_FL_BmN-_08J10_R_0	BJ988628
E_FL_BmN-_08J11_R_0	BJ988629
E_FL_BmN-_08J13_F_0	BJ982381
E_FL_BmN-_08J13_R_0	BJ988630
E_FL_BmN-_08J15_R_0	BJ988631
E_FL_BmN-_08J16_F_0	BJ982382
E_FL_BmN-_08J18_R_0	BJ988632
E_FL_BmN-_08J19_F_0	BJ982383
E_FL_BmN-_08J19_R_0	BJ988633
E_FL_BmN-_08J20_F_0	BJ982384
E_FL_BmN-_08J20_R_0	BJ988634
E_FL_BmN-_08J21_F_0	BJ982385
E_FL_BmN-_08J21_R_0	BJ988635

E_FL_BmN-_08J22_F_0	BJ982386
E_FL_BmN-_08J22_R_0	BJ988636
E_FL_BmN-_08J23_F_0	BJ982387
E_FL_BmN-_08J23_R_0	BJ988637
E_FL_BmN-_08J24_R_0	BJ988638
E_FL_BmN-_08K01_F_0	BJ982388
E_FL_BmN-_08K01_R_0	BJ988639
E_FL_BmN-_08K02_F_0	BJ982389
E_FL_BmN-_08K02_R_0	BJ988640
E_FL_BmN-_08K03_F_0	BJ982390
E_FL_BmN-_08K03_R_0	BJ988641
E_FL_BmN-_08K04_F_0	BJ982391
E_FL_BmN-_08K04_R_0	BJ988642
E_FL_BmN-_08K05_F_0	BJ982392
E_FL_BmN-_08K05_R_0	BJ988643
E_FL_BmN-_08K06_F_0	BJ982393
E_FL_BmN-_08K06_R_0	BJ988644
E_FL_BmN-_08K07_F_0	BJ982394
E_FL_BmN-_08K08_F_0	BJ982395
E_FL_BmN-_08K08_R_0	BJ988645
E_FL_BmN-_08K09_F_0	BJ982396
E_FL_BmN-_08K10_F_0	BJ982397
E_FL_BmN-_08K10_R_0	BJ988646
E_FL_BmN-_08K11_F_0	BJ982398
E_FL_BmN-_08K11_R_0	BJ988647
E_FL_BmN-_08K12_F_0	BJ982399
E_FL_BmN-_08K12_R_0	BJ988648
E_FL_BmN-_08K13_F_0	BJ982400
E_FL_BmN-_08K13_R_0	BJ988649
E_FL_BmN-_08K14_F_0	BJ982401
E_FL_BmN-_08K14_R_0	BJ988650
E_FL_BmN-_08K15_F_0	BJ982402
E_FL_BmN-_08K15_R_0	BJ988651
E_FL_BmN-_08K16_F_0	BJ982403
E_FL_BmN-_08K16_R_0	BJ988652
E_FL_BmN-_08K17_R_0	BJ988653
E_FL_BmN-_08K18_R_0	BJ988654
E_FL_BmN-_08K19_F_0	BJ982404
E_FL_BmN-_08K19_R_0	BJ988655
E_FL_BmN-_08K20_F_0	BJ982405
E_FL_BmN-_08K20_R_0	BJ988656
E_FL_BmN-_08K22_F_0	BJ982406
E_FL_BmN-_08K23_F_0	BJ982407
E_FL_BmN-_08K23_R_0	BJ988657
E_FL_BmN-_08K24_F_0	BJ982408
E_FL_BmN-_08K24_R_0	BJ988658
E_FL_BmN-_08L01_R_0	BJ988659
E_FL_BmN-_08L02_F_0	BJ982409
E_FL_BmN-_08L02_R_0	BJ988660
E_FL_BmN-_08L03_F_0	BJ982410
E_FL_BmN-_08L03_R_0	BJ988661
E_FL_BmN-_08L04_F_0	BJ982411
E_FL_BmN-_08L04_R_0	BJ988662
E_FL_BmN-_08L05_F_0	BJ982412

E_FL_BmN-_08L05_R_0	BJ988663
E_FL_BmN-_08L07_F_0	BJ982413
E_FL_BmN-_08L07_R_0	BJ988664
E_FL_BmN-_08L08_R_0	BJ988665
E_FL_BmN-_08L09_R_0	BJ988666
E_FL_BmN-_08L10_F_0	BJ982414
E_FL_BmN-_08L10_R_0	BJ988667
E_FL_BmN-_08L11_R_0	BJ988668
E_FL_BmN-_08L12_F_0	BJ982415
E_FL_BmN-_08L12_R_0	BJ988669
E_FL_BmN-_08L13_R_0	BJ988670
E_FL_BmN-_08L14_F_0	BJ982416
E_FL_BmN-_08L14_R_0	BJ988671
E_FL_BmN-_08L15_F_0	BJ982417
E_FL_BmN-_08L15_R_0	BJ988672
E_FL_BmN-_08L16_F_0	BJ982418
E_FL_BmN-_08L16_R_0	BJ988673
E_FL_BmN-_08L17_R_0	BJ988674
E_FL_BmN-_08L19_R_0	BJ988675
E_FL_BmN-_08L20_F_0	BJ982419
E_FL_BmN-_08L21_F_0	BJ982420
E_FL_BmN-_08L21_R_0	BJ988676
E_FL_BmN-_08L22_R_0	BJ988677
E_FL_BmN-_08L23_F_0	BJ982421
E_FL_BmN-_08L23_R_0	BJ988678
E_FL_BmN-_08L24_F_0	BJ982422
E_FL_BmN-_08L24_R_0	BJ988679
E_FL_BmN-_08M01_F_0	BJ982423
E_FL_BmN-_08M01_R_0	BJ988680
E_FL_BmN-_08M02_F_0	BJ982424
E_FL_BmN-_08M02_R_0	BJ988681
E_FL_BmN-_08M03_F_0	BJ982425
E_FL_BmN-_08M03_R_0	BJ988682
E_FL_BmN-_08M04_F_0	BJ982426
E_FL_BmN-_08M04_R_0	BJ988683
E_FL_BmN-_08M05_F_0	BJ982427
E_FL_BmN-_08M05_R_0	BJ988684
E_FL_BmN-_08M06_F_0	BJ982428
E_FL_BmN-_08M06_R_0	BJ988685
E_FL_BmN-_08M07_R_0	BJ988686
E_FL_BmN-_08M08_F_0	BJ982429
E_FL_BmN-_08M08_R_0	BJ988687
E_FL_BmN-_08M10_F_0	BJ982430
E_FL_BmN-_08M11_F_0	BJ982431
E_FL_BmN-_08M11_R_0	BJ988688
E_FL_BmN-_08M12_R_0	BJ988689
E_FL_BmN-_08M13_F_0	BJ982432
E_FL_BmN-_08M14_F_0	BJ982433
E_FL_BmN-_08M15_F_0	BJ982434
E_FL_BmN-_08M15_R_0	BJ988690
E_FL_BmN-_08M16_F_0	BJ982435
E_FL_BmN-_08M16_R_0	BJ988691
E_FL_BmN-_08M17_F_0	BJ982436
E_FL_BmN-_08M17_R_0	BJ988692

E_FL_BmN-_08M18_R_0	BJ988693
E_FL_BmN-_08M19_F_0	BJ982437
E_FL_BmN-_08M20_F_0	BJ982438
E_FL_BmN-_08M20_R_0	BJ988694
E_FL_BmN-_08M21_F_0	BJ982439
E_FL_BmN-_08M21_R_0	BJ988695
E_FL_BmN-_08M22_F_0	BJ982440
E_FL_BmN-_08M22_R_0	BJ988696
E_FL_BmN-_08M23_F_0	BJ982441
E_FL_BmN-_08M23_R_0	BJ988697
E_FL_BmN-_08M24_F_0	BJ982442
E_FL_BmN-_08M24_R_0	BJ988698
E_FL_BmN-_08N01_F_0	BJ982443
E_FL_BmN-_08N01_R_0	BJ988699
E_FL_BmN-_08N02_F_0	BJ982444
E_FL_BmN-_08N02_R_0	BJ988700
E_FL_BmN-_08N03_F_0	BJ982445
E_FL_BmN-_08N03_R_0	BJ988701
E_FL_BmN-_08N04_F_0	BJ982446
E_FL_BmN-_08N05_F_0	BJ982447
E_FL_BmN-_08N06_F_0	BJ982448
E_FL_BmN-_08N06_R_0	BJ988702
E_FL_BmN-_08N07_F_0	BJ982449
E_FL_BmN-_08N07_R_0	BJ988703
E_FL_BmN-_08N08_F_0	BJ982450
E_FL_BmN-_08N08_R_0	BJ988704
E_FL_BmN-_08N09_F_0	BJ982451
E_FL_BmN-_08N10_F_0	BJ982452
E_FL_BmN-_08N10_R_0	BJ988705
E_FL_BmN-_08N12_R_0	BJ988706
E_FL_BmN-_08N13_F_0	BJ982453
E_FL_BmN-_08N13_R_0	BJ988707
E_FL_BmN-_08N14_F_0	BJ982454
E_FL_BmN-_08N15_F_0	BJ982455
E_FL_BmN-_08N15_R_0	BJ988708
E_FL_BmN-_08N16_R_0	BJ988709
E_FL_BmN-_08N17_R_0	BJ988710
E_FL_BmN-_08N18_F_0	BJ982456
E_FL_BmN-_08N18_R_0	BJ988711
E_FL_BmN-_08N19_R_0	BJ988712
E_FL_BmN-_08N20_F_0	BJ982457
E_FL_BmN-_08N20_R_0	BJ988713
E_FL_BmN-_08N21_F_0	BJ982458
E_FL_BmN-_08N21_R_0	BJ988714
E_FL_BmN-_08N23_R_0	BJ988715
E_FL_BmN-_08N24_F_0	BJ982459
E_FL_BmN-_08N24_R_0	BJ988716
E_FL_BmN-_08O02_F_0	BJ982460
E_FL_BmN-_08O02_R_0	BJ988717
E_FL_BmN-_08O03_F_0	BJ982461
E_FL_BmN-_08O03_R_0	BJ988718
E_FL_BmN-_08O04_F_0	BJ982462
E_FL_BmN-_08O04_R_0	BJ988719
E_FL_BmN-_08O05_F_0	BJ982463

E_FL_BmN-_08O05_R_0	BJ988720
E_FL_BmN-_08O06_R_0	BJ988721
E_FL_BmN-_08O07_F_0	BJ982464
E_FL_BmN-_08O08_F_0	BJ982465
E_FL_BmN-_08O08_R_0	BJ988722
E_FL_BmN-_08O09_F_0	BJ982466
E_FL_BmN-_08O09_R_0	BJ988723
E_FL_BmN-_08O10_F_0	BJ982467
E_FL_BmN-_08O10_R_0	BJ988724
E_FL_BmN-_08O11_R_0	BJ988725
E_FL_BmN-_08O12_R_0	BJ988726
E_FL_BmN-_08O13_F_0	BJ982468
E_FL_BmN-_08O13_R_0	BJ988727
E_FL_BmN-_08O14_F_0	BJ982469
E_FL_BmN-_08O14_R_0	BJ988728
E_FL_BmN-_08O15_F_0	BJ982470
E_FL_BmN-_08O15_R_0	BJ988729
E_FL_BmN-_08O16_R_0	BJ988730
E_FL_BmN-_08O17_F_0	BJ982471
E_FL_BmN-_08O17_R_0	BJ988731
E_FL_BmN-_08O18_R_0	BJ988732
E_FL_BmN-_08O19_R_0	BJ988733
E_FL_BmN-_08O20_F_0	BJ982472
E_FL_BmN-_08O20_R_0	BJ988734
E_FL_BmN-_08O21_F_0	BJ982473
E_FL_BmN-_08O21_R_0	BJ988735
E_FL_BmN-_08O22_R_0	BJ988736
E_FL_BmN-_08O23_R_0	BJ988737
E_FL_BmN-_08O24_R_0	BJ988738
E_FL_BmN-_08P01_F_0	BJ982474
E_FL_BmN-_08P01_R_0	BJ988739
E_FL_BmN-_08P02_F_0	BJ982475
E_FL_BmN-_08P02_R_0	BJ988740
E_FL_BmN-_08P04_F_0	BJ982476
E_FL_BmN-_08P04_R_0	BJ988741
E_FL_BmN-_08P05_R_0	BJ988742
E_FL_BmN-_08P07_F_0	BJ982477
E_FL_BmN-_08P07_R_0	BJ988743
E_FL_BmN-_08P08_F_0	BJ982478
E_FL_BmN-_08P08_R_0	BJ988744
E_FL_BmN-_08P09_R_0	BJ988745
E_FL_BmN-_08P10_F_0	BJ982479
E_FL_BmN-_08P10_R_0	BJ988746
E_FL_BmN-_08P11_R_0	BJ988747
E_FL_BmN-_08P12_F_0	BJ982480
E_FL_BmN-_08P12_R_0	BJ988748
E_FL_BmN-_08P13_R_0	BJ988749
E_FL_BmN-_08P14_F_0	BJ982481
E_FL_BmN-_08P14_R_0	BJ988750
E_FL_BmN-_08P15_F_0	BJ982482
E_FL_BmN-_08P15_R_0	BJ988751
E_FL_BmN-_08P16_F_0	BJ982483
E_FL_BmN-_08P16_R_0	BJ988752
E_FL_BmN-_08P17_R_0	BJ988753

E_FL_BmN-_08P18_R_0	BJ988754
E_FL_BmN-_08P19_F_0	BJ982484
E_FL_BmN-_08P19_R_0	BJ988755
E_FL_BmN-_08P20_F_0	BJ982485
E_FL_BmN-_08P21_F_0	BJ982486
E_FL_BmN-_08P21_R_0	BJ988756
E_FL_BmN-_08P22_F_0	BJ982487
E_FL_BmN-_08P22_R_0	BJ988757
E_FL_BmN-_08P23_R_0	BJ988758
E_FL_BmN-_08P24_F_0	BJ982488
E_FL_BmN-_08P24_R_0	BJ988759
E_FL_BmN-_09A01_R_0	BJ988760
E_FL_BmN-_09A02_F_0	BJ982489
E_FL_BmN-_09A02_R_0	BJ988761
E_FL_BmN-_09A03_R_0	BJ988762
E_FL_BmN-_09A04_F_0	BJ982490
E_FL_BmN-_09A04_R_0	BJ988763
E_FL_BmN-_09A05_F_0	BJ982491
E_FL_BmN-_09A05_R_0	BJ988764
E_FL_BmN-_09A06_F_0	BJ982492
E_FL_BmN-_09A06_R_0	BJ988765
E_FL_BmN-_09A07_R_0	BJ988766
E_FL_BmN-_09A08_F_0	BJ982493
E_FL_BmN-_09A08_R_0	BJ988767
E_FL_BmN-_09A09_R_0	BJ988768
E_FL_BmN-_09A12_R_0	BJ988769
E_FL_BmN-_09A13_R_0	BJ988770
E_FL_BmN-_09A14_F_0	BJ982494
E_FL_BmN-_09A14_R_0	BJ988771
E_FL_BmN-_09A15_F_0	BJ982495
E_FL_BmN-_09A15_R_0	BJ988772
E_FL_BmN-_09A16_F_0	BJ982496
E_FL_BmN-_09A16_R_0	BJ988773
E_FL_BmN-_09A18_F_0	BJ982497
E_FL_BmN-_09A18_R_0	BJ988774
E_FL_BmN-_09A19_R_0	BJ988775
E_FL_BmN-_09A20_F_0	BJ982498
E_FL_BmN-_09A20_R_0	BJ988776
E_FL_BmN-_09A21_R_0	BJ988777
E_FL_BmN-_09A22_F_0	BJ982499
E_FL_BmN-_09A22_R_0	BJ988778
E_FL_BmN-_09A23_F_0	BJ982500
E_FL_BmN-_09A23_R_0	BJ988779
E_FL_BmN-_09B01_F_0	BJ982501
E_FL_BmN-_09B02_F_0	BJ982502
E_FL_BmN-_09B02_R_0	BJ988780
E_FL_BmN-_09B03_R_0	BJ988781
E_FL_BmN-_09B04_F_0	BJ982503
E_FL_BmN-_09B04_R_0	BJ988782
E_FL_BmN-_09B05_R_0	BJ988783
E_FL_BmN-_09B06_F_0	BJ982504
E_FL_BmN-_09B06_R_0	BJ988784
E_FL_BmN-_09B07_F_0	BJ982505
E_FL_BmN-_09B07_R_0	BJ988785

E_FL_BmN-_09B09_F_0	BJ982506
E_FL_BmN-_09B09_R_0	BJ988786
E_FL_BmN-_09B10_F_0	BJ982507
E_FL_BmN-_09B10_R_0	BJ988787
E_FL_BmN-_09B11_F_0	BJ982508
E_FL_BmN-_09B11_R_0	BJ988788
E_FL_BmN-_09B12_F_0	BJ982509
E_FL_BmN-_09B13_F_0	BJ982510
E_FL_BmN-_09B13_R_0	BJ988789
E_FL_BmN-_09B15_F_0	BJ982511
E_FL_BmN-_09B15_R_0	BJ988790
E_FL_BmN-_09B17_F_0	BJ982512
E_FL_BmN-_09B17_R_0	BJ988791
E_FL_BmN-_09B18_F_0	BJ982513
E_FL_BmN-_09B18_R_0	BJ988792
E_FL_BmN-_09B19_F_0	BJ982514
E_FL_BmN-_09B20_F_0	BJ982515
E_FL_BmN-_09B20_R_0	BJ988793
E_FL_BmN-_09B21_F_0	BJ982516
E_FL_BmN-_09B21_R_0	BJ988794
E_FL_BmN-_09B22_R_0	BJ988795
E_FL_BmN-_09B23_F_0	BJ982517
E_FL_BmN-_09B23_R_0	BJ988796
E_FL_BmN-_09B24_F_0	BJ982518
E_FL_BmN-_09B24_R_0	BJ988797
E_FL_BmN-_09C01_F_0	BJ982519
E_FL_BmN-_09C01_R_0	BJ988798
E_FL_BmN-_09C03_F_0	BJ982520
E_FL_BmN-_09C03_R_0	BJ988799
E_FL_BmN-_09C04_F_0	BJ982521
E_FL_BmN-_09C04_R_0	BJ988800
E_FL_BmN-_09C05_F_0	BJ982522
E_FL_BmN-_09C05_R_0	BJ988801
E_FL_BmN-_09C06_F_0	BJ982523
E_FL_BmN-_09C06_R_0	BJ988802
E_FL_BmN-_09C09_F_0	BJ982524
E_FL_BmN-_09C09_R_0	BJ988803
E_FL_BmN-_09C10_F_0	BJ982525
E_FL_BmN-_09C10_R_0	BJ988804
E_FL_BmN-_09C11_F_0	BJ982526
E_FL_BmN-_09C11_R_0	BJ988805
E_FL_BmN-_09C12_F_0	BJ982527
E_FL_BmN-_09C12_R_0	BJ988806
E_FL_BmN-_09C13_F_0	BJ982528
E_FL_BmN-_09C14_R_0	BJ988807
E_FL_BmN-_09C15_F_0	BJ982529
E_FL_BmN-_09C15_R_0	BJ988808
E_FL_BmN-_09C16_F_0	BJ982530
E_FL_BmN-_09C17_F_0	BJ982531
E_FL_BmN-_09C17_R_0	BJ988809
E_FL_BmN-_09C18_F_0	BJ982532
E_FL_BmN-_09C18_R_0	BJ988810
E_FL_BmN-_09C19_F_0	BJ982533
E_FL_BmN-_09C19_R_0	BJ988811

E_FL_BmN-_09C21_F_0	BJ982534
E_FL_BmN-_09C22_F_0	BJ982535
E_FL_BmN-_09C22_R_0	BJ988812
E_FL_BmN-_09C23_F_0	BJ982536
E_FL_BmN-_09C23_R_0	BJ988813
E_FL_BmN-_09C24_R_0	BJ988814
E_FL_BmN-_09D01_F_0	BJ982537
E_FL_BmN-_09D02_R_0	BJ988815
E_FL_BmN-_09D03_F_0	BJ982538
E_FL_BmN-_09D03_R_0	BJ988816
E_FL_BmN-_09D04_F_0	BJ982539
E_FL_BmN-_09D04_R_0	BJ988817
E_FL_BmN-_09D05_F_0	BJ982540
E_FL_BmN-_09D05_R_0	BJ988818
E_FL_BmN-_09D06_F_0	BJ982541
E_FL_BmN-_09D07_R_0	BJ988819
E_FL_BmN-_09D08_F_0	BJ982542
E_FL_BmN-_09D08_R_0	BJ988820
E_FL_BmN-_09D09_F_0	BJ982543
E_FL_BmN-_09D09_R_0	BJ988821
E_FL_BmN-_09D10_F_0	BJ982544
E_FL_BmN-_09D11_F_0	BJ982545
E_FL_BmN-_09D11_R_0	BJ988822
E_FL_BmN-_09D12_F_0	BJ982546
E_FL_BmN-_09D12_R_0	BJ988823
E_FL_BmN-_09D13_F_0	BJ982547
E_FL_BmN-_09D13_R_0	BJ988824
E_FL_BmN-_09D14_F_0	BJ982548
E_FL_BmN-_09D14_R_0	BJ988825
E_FL_BmN-_09D15_R_0	BJ988826
E_FL_BmN-_09D16_R_0	BJ988827
E_FL_BmN-_09D17_F_0	BJ982549
E_FL_BmN-_09D17_R_0	BJ988828
E_FL_BmN-_09D18_F_0	BJ982550
E_FL_BmN-_09D18_R_0	BJ988829
E_FL_BmN-_09D19_F_0	BJ982551
E_FL_BmN-_09D19_R_0	BJ988830
E_FL_BmN-_09D20_R_0	BJ988831
E_FL_BmN-_09D21_R_0	BJ988832
E_FL_BmN-_09D22_R_0	BJ988833
E_FL_BmN-_09D23_F_0	BJ982552
E_FL_BmN-_09D23_R_0	BJ988834
E_FL_BmN-_09D24_F_0	BJ982553
E_FL_BmN-_09E01_F_0	BJ982554
E_FL_BmN-_09E02_F_0	BJ982555
E_FL_BmN-_09E02_R_0	BJ988835
E_FL_BmN-_09E04_F_0	BJ982556
E_FL_BmN-_09E04_R_0	BJ988836
E_FL_BmN-_09E05_F_0	BJ982557
E_FL_BmN-_09E05_R_0	BJ988837
E_FL_BmN-_09E06_F_0	BJ982558
E_FL_BmN-_09E06_R_0	BJ988838
E_FL_BmN-_09E07_F_0	BJ982559
E_FL_BmN-_09E07_R_0	BJ988839

E_FL_BmN-_09E08_R_0	BJ988840
E_FL_BmN-_09E09_R_0	BJ988841
E_FL_BmN-_09E10_F_0	BJ982560
E_FL_BmN-_09E10_R_0	BJ988842
E_FL_BmN-_09E11_R_0	BJ988843
E_FL_BmN-_09E12_F_0	BJ982561
E_FL_BmN-_09E12_R_0	BJ988844
E_FL_BmN-_09E14_R_0	BJ988845
E_FL_BmN-_09E15_F_0	BJ982562
E_FL_BmN-_09E15_R_0	BJ988846
E_FL_BmN-_09E16_F_0	BJ982563
E_FL_BmN-_09E16_R_0	BJ988847
E_FL_BmN-_09E17_R_0	BJ988848
E_FL_BmN-_09E18_F_0	BJ982564
E_FL_BmN-_09E18_R_0	BJ988849
E_FL_BmN-_09E19_F_0	BJ982565
E_FL_BmN-_09E19_R_0	BJ988850
E_FL_BmN-_09E20_R_0	BJ988851
E_FL_BmN-_09E21_R_0	BJ988852
E_FL_BmN-_09E22_F_0	BJ982566
E_FL_BmN-_09E22_R_0	BJ988853
E_FL_BmN-_09E23_F_0	BJ982567
E_FL_BmN-_09E23_R_0	BJ988854
E_FL_BmN-_09E24_F_0	BJ982568
E_FL_BmN-_09F01_F_0	BJ982569
E_FL_BmN-_09F02_F_0	BJ982570
E_FL_BmN-_09F02_R_0	BJ988855
E_FL_BmN-_09F03_F_0	BJ982571
E_FL_BmN-_09F03_R_0	BJ988856
E_FL_BmN-_09F04_R_0	BJ988857
E_FL_BmN-_09F05_R_0	BJ988858
E_FL_BmN-_09F06_R_0	BJ988859
E_FL_BmN-_09F07_F_0	BJ982572
E_FL_BmN-_09F08_F_0	BJ982573
E_FL_BmN-_09F08_R_0	BJ988860
E_FL_BmN-_09F09_F_0	BJ982574
E_FL_BmN-_09F09_R_0	BJ988861
E_FL_BmN-_09F10_R_0	BJ988862
E_FL_BmN-_09F11_R_0	BJ988863
E_FL_BmN-_09F12_F_0	BJ982575
E_FL_BmN-_09F12_R_0	BJ988864
E_FL_BmN-_09F14_F_0	BJ982576
E_FL_BmN-_09F14_R_0	BJ988865
E_FL_BmN-_09F15_F_0	BJ982577
E_FL_BmN-_09F15_R_0	BJ988866
E_FL_BmN-_09F16_F_0	BJ982578
E_FL_BmN-_09F16_R_0	BJ988867
E_FL_BmN-_09F17_R_0	BJ988868
E_FL_BmN-_09F18_R_0	BJ988869
E_FL_BmN-_09F19_F_0	BJ982579
E_FL_BmN-_09F19_R_0	BJ988870
E_FL_BmN-_09F20_F_0	BJ982580
E_FL_BmN-_09F20_R_0	BJ988871
E_FL_BmN-_09F21_R_0	BJ988872

E_FL_BmN-_09F22_R_0	BJ988873
E_FL_BmN-_09F23_F_0	BJ982581
E_FL_BmN-_09F23_R_0	BJ988874
E_FL_BmN-_09F24_F_0	BJ982582
E_FL_BmN-_09F24_R_0	BJ988875
E_FL_BmN-_09G01_F_0	BJ982583
E_FL_BmN-_09G01_R_0	BJ988876
E_FL_BmN-_09G02_F_0	BJ982584
E_FL_BmN-_09G02_R_0	BJ988877
E_FL_BmN-_09G03_F_0	BJ982585
E_FL_BmN-_09G03_R_0	BJ988878
E_FL_BmN-_09G04_R_0	BJ988879
E_FL_BmN-_09G05_F_0	BJ982586
E_FL_BmN-_09G05_R_0	BJ988880
E_FL_BmN-_09G06_R_0	BJ988881
E_FL_BmN-_09G07_F_0	BJ982587
E_FL_BmN-_09G07_R_0	BJ988882
E_FL_BmN-_09G08_R_0	BJ988883
E_FL_BmN-_09G09_F_0	BJ982588
E_FL_BmN-_09G09_R_0	BJ988884
E_FL_BmN-_09G10_R_0	BJ988885
E_FL_BmN-_09G11_F_0	BJ982589
E_FL_BmN-_09G11_R_0	BJ988886
E_FL_BmN-_09G12_F_0	BJ982590
E_FL_BmN-_09G12_R_0	BJ988887
E_FL_BmN-_09G14_F_0	BJ982591
E_FL_BmN-_09G14_R_0	BJ988888
E_FL_BmN-_09G15_F_0	BJ982592
E_FL_BmN-_09G15_R_0	BJ988889
E_FL_BmN-_09G16_F_0	BJ982593
E_FL_BmN-_09G16_R_0	BJ988890
E_FL_BmN-_09G17_F_0	BJ982594
E_FL_BmN-_09G17_R_0	BJ988891
E_FL_BmN-_09G18_F_0	BJ982595
E_FL_BmN-_09G18_R_0	BJ988892
E_FL_BmN-_09G19_F_0	BJ982596
E_FL_BmN-_09G19_R_0	BJ988893
E_FL_BmN-_09G20_R_0	BJ988894
E_FL_BmN-_09G21_F_0	BJ982597
E_FL_BmN-_09G21_R_0	BJ988895
E_FL_BmN-_09G22_R_0	BJ988896
E_FL_BmN-_09G23_F_0	BJ982598
E_FL_BmN-_09G23_R_0	BJ988897
E_FL_BmN-_09G24_F_0	BJ982599
E_FL_BmN-_09G24_R_0	BJ988898
E_FL_BmN-_09H01_F_0	BJ982600
E_FL_BmN-_09H01_R_0	BJ988899
E_FL_BmN-_09H03_F_0	BJ982601
E_FL_BmN-_09H03_R_0	BJ988900
E_FL_BmN-_09H04_R_0	BJ988901
E_FL_BmN-_09H05_F_0	BJ982602
E_FL_BmN-_09H05_R_0	BJ988902
E_FL_BmN-_09H06_F_0	BJ982603
E_FL_BmN-_09H07_F_0	BJ982604

E_FL_BmN-_09H07_R_0	BJ988903
E_FL_BmN-_09H09_F_0	BJ982605
E_FL_BmN-_09H09_R_0	BJ988904
E_FL_BmN-_09H10_F_0	BJ982606
E_FL_BmN-_09H10_R_0	BJ988905
E_FL_BmN-_09H11_F_0	BJ982607
E_FL_BmN-_09H11_R_0	BJ988906
E_FL_BmN-_09H12_F_0	BJ982608
E_FL_BmN-_09H12_R_0	BJ988907
E_FL_BmN-_09H13_F_0	BJ982609
E_FL_BmN-_09H13_R_0	BJ988908
E_FL_BmN-_09H14_F_0	BJ982610
E_FL_BmN-_09H14_R_0	BJ988909
E_FL_BmN-_09H15_F_0	BJ982611
E_FL_BmN-_09H15_R_0	BJ988910
E_FL_BmN-_09H16_F_0	BJ982612
E_FL_BmN-_09H17_F_0	BJ982613
E_FL_BmN-_09H17_R_0	BJ988911
E_FL_BmN-_09H18_F_0	BJ982614
E_FL_BmN-_09H19_F_0	BJ982615
E_FL_BmN-_09H20_F_0	BJ982616
E_FL_BmN-_09H20_R_0	BJ988912
E_FL_BmN-_09H21_R_0	BJ988913
E_FL_BmN-_09H22_F_0	BJ982617
E_FL_BmN-_09H22_R_0	BJ988914
E_FL_BmN-_09H23_F_0	BJ982618
E_FL_BmN-_09H23_R_0	BJ988915
E_FL_BmN-_09H24_F_0	BJ982619
E_FL_BmN-_09I01_F_0	BJ982620
E_FL_BmN-_09I01_R_0	BJ988916
E_FL_BmN-_09I02_F_0	BJ982621
E_FL_BmN-_09I02_R_0	BJ988917
E_FL_BmN-_09I04_F_0	BJ982622
E_FL_BmN-_09I04_R_0	BJ988918
E_FL_BmN-_09I05_F_0	BJ982623
E_FL_BmN-_09I05_R_0	BJ988919
E_FL_BmN-_09I06_F_0	BJ982624
E_FL_BmN-_09I06_R_0	BJ988920
E_FL_BmN-_09I07_F_0	BJ982625
E_FL_BmN-_09I07_R_0	BJ988921
E_FL_BmN-_09I08_R_0	BJ988922
E_FL_BmN-_09I09_F_0	BJ982626
E_FL_BmN-_09I09_R_0	BJ988923
E_FL_BmN-_09I10_F_0	BJ982627
E_FL_BmN-_09I10_R_0	BJ988924
E_FL_BmN-_09I11_F_0	BJ982628
E_FL_BmN-_09I11_R_0	BJ988925
E_FL_BmN-_09I12_F_0	BJ982629
E_FL_BmN-_09I12_R_0	BJ988926
E_FL_BmN-_09I13_R_0	BJ988927
E_FL_BmN-_09I14_F_0	BJ982630
E_FL_BmN-_09I14_R_0	BJ988928
E_FL_BmN-_09I15_F_0	BJ982631
E_FL_BmN-_09I15_R_0	BJ988929

E_FL_BmN-_09I16_F_0	BJ982632
E_FL_BmN-_09I16_R_0	BJ988930
E_FL_BmN-_09I17_F_0	BJ982633
E_FL_BmN-_09I17_R_0	BJ988931
E_FL_BmN-_09I18_R_0	BJ988932
E_FL_BmN-_09I19_F_0	BJ982634
E_FL_BmN-_09I19_R_0	BJ988933
E_FL_BmN-_09I20_R_0	BJ988934
E_FL_BmN-_09I22_F_0	BJ982635
E_FL_BmN-_09I22_R_0	BJ988935
E_FL_BmN-_09I23_F_0	BJ982636
E_FL_BmN-_09I23_R_0	BJ988936
E_FL_BmN-_09I24_F_0	BJ982637
E_FL_BmN-_09I24_R_0	BJ988937
E_FL_BmN-_09J01_F_0	BJ982638
E_FL_BmN-_09J01_R_0	BJ988938
E_FL_BmN-_09J02_F_0	BJ982639
E_FL_BmN-_09J02_R_0	BJ988939
E_FL_BmN-_09J03_R_0	BJ988940
E_FL_BmN-_09J04_R_0	BJ988941
E_FL_BmN-_09J05_F_0	BJ982640
E_FL_BmN-_09J07_F_0	BJ982641
E_FL_BmN-_09J07_R_0	BJ988942
E_FL_BmN-_09J08_F_0	BJ982642
E_FL_BmN-_09J08_R_0	BJ988943
E_FL_BmN-_09J09_F_0	BJ982643
E_FL_BmN-_09J09_R_0	BJ988944
E_FL_BmN-_09J10_R_0	BJ988945
E_FL_BmN-_09J11_F_0	BJ982644
E_FL_BmN-_09J12_F_0	BJ982645
E_FL_BmN-_09J12_R_0	BJ988946
E_FL_BmN-_09J13_F_0	BJ982646
E_FL_BmN-_09J13_R_0	BJ988947
E_FL_BmN-_09J14_F_0	BJ982647
E_FL_BmN-_09J14_R_0	BJ988948
E_FL_BmN-_09J15_F_0	BJ982648
E_FL_BmN-_09J15_R_0	BJ988949
E_FL_BmN-_09J16_F_0	BJ982649
E_FL_BmN-_09J16_R_0	BJ988950
E_FL_BmN-_09J17_F_0	BJ982650
E_FL_BmN-_09J17_R_0	BJ988951
E_FL_BmN-_09J18_F_0	BJ982651
E_FL_BmN-_09J19_F_0	BJ982652
E_FL_BmN-_09J19_R_0	BJ988952
E_FL_BmN-_09J20_F_0	BJ982653
E_FL_BmN-_09J21_R_0	BJ988953
E_FL_BmN-_09J22_R_0	BJ988954
E_FL_BmN-_09J23_F_0	BJ982654
E_FL_BmN-_09J23_R_0	BJ988955
E_FL_BmN-_09J24_F_0	BJ982655
E_FL_BmN-_09J24_R_0	BJ988956
E_FL_BmN-_09K01_F_0	BJ982656
E_FL_BmN-_09K01_R_0	BJ988957
E_FL_BmN-_09K02_F_0	BJ982657

E_FL_BmN-_09K02_R_0	BJ988958
E_FL_BmN-_09K03_F_0	BJ982658
E_FL_BmN-_09K03_R_0	BJ988959
E_FL_BmN-_09K04_F_0	BJ982659
E_FL_BmN-_09K04_R_0	BJ988960
E_FL_BmN-_09K05_F_0	BJ982660
E_FL_BmN-_09K05_R_0	BJ988961
E_FL_BmN-_09K06_R_0	BJ988962
E_FL_BmN-_09K07_R_0	BJ988963
E_FL_BmN-_09K08_R_0	BJ988964
E_FL_BmN-_09K09_F_0	BJ982661
E_FL_BmN-_09K09_R_0	BJ988965
E_FL_BmN-_09K14_F_0	BJ982662
E_FL_BmN-_09K15_F_0	BJ982663
E_FL_BmN-_09K15_R_0	BJ988966
E_FL_BmN-_09K16_F_0	BJ982664
E_FL_BmN-_09K16_R_0	BJ988967
E_FL_BmN-_09K17_F_0	BJ982665
E_FL_BmN-_09K17_R_0	BJ988968
E_FL_BmN-_09K18_R_0	BJ988969
E_FL_BmN-_09K20_R_0	BJ988970
E_FL_BmN-_09K21_F_0	BJ982666
E_FL_BmN-_09K21_R_0	BJ988971
E_FL_BmN-_09K22_F_0	BJ982667
E_FL_BmN-_09K22_R_0	BJ988972
E_FL_BmN-_09K23_F_0	BJ982668
E_FL_BmN-_09K23_R_0	BJ988973
E_FL_BmN-_09K24_R_0	BJ988974
E_FL_BmN-_09L01_F_0	BJ982669
E_FL_BmN-_09L01_R_0	BJ988975
E_FL_BmN-_09L02_F_0	BJ982670
E_FL_BmN-_09L03_F_0	BJ982671
E_FL_BmN-_09L03_R_0	BJ988976
E_FL_BmN-_09L04_F_0	BJ982672
E_FL_BmN-_09L04_R_0	BJ988977
E_FL_BmN-_09L05_F_0	BJ982673
E_FL_BmN-_09L05_R_0	BJ988978
E_FL_BmN-_09L06_R_0	BJ988979
E_FL_BmN-_09L07_F_0	BJ982674
E_FL_BmN-_09L07_R_0	BJ988980
E_FL_BmN-_09L08_F_0	BJ982675
E_FL_BmN-_09L08_R_0	BJ988981
E_FL_BmN-_09L09_R_0	BJ988982
E_FL_BmN-_09L10_R_0	BJ988983
E_FL_BmN-_09L11_F_0	BJ982676
E_FL_BmN-_09L11_R_0	BJ988984
E_FL_BmN-_09L14_F_0	BJ982677
E_FL_BmN-_09L14_R_0	BJ988985
E_FL_BmN-_09L15_R_0	BJ988986
E_FL_BmN-_09L16_F_0	BJ982678
E_FL_BmN-_09L16_R_0	BJ988987
E_FL_BmN-_09L17_F_0	BJ982679
E_FL_BmN-_09L17_R_0	BJ988988
E_FL_BmN-_09L18_R_0	BJ988989

E_FL_BmN-_09L19_F_0	BJ982680
E_FL_BmN-_09L19_R_0	BJ988990
E_FL_BmN-_09L20_F_0	BJ982681
E_FL_BmN-_09L20_R_0	BJ988991
E_FL_BmN-_09L21_F_0	BJ982682
E_FL_BmN-_09L21_R_0	BJ988992
E_FL_BmN-_09L22_F_0	BJ982683
E_FL_BmN-_09L22_R_0	BJ988993
E_FL_BmN-_09L23_F_0	BJ982684
E_FL_BmN-_09L23_R_0	BJ988994
E_FL_BmN-_09L24_R_0	BJ988995
E_FL_BmN-_09M03_F_0	BJ982685
E_FL_BmN-_09M03_R_0	BJ988996
E_FL_BmN-_09M04_F_0	BJ982686
E_FL_BmN-_09M04_R_0	BJ988997
E_FL_BmN-_09M05_F_0	BJ982687
E_FL_BmN-_09M06_F_0	BJ982688
E_FL_BmN-_09M06_R_0	BJ988998
E_FL_BmN-_09M07_F_0	BJ982689
E_FL_BmN-_09M08_R_0	BJ988999
E_FL_BmN-_09M09_F_0	BJ982690
E_FL_BmN-_09M10_F_0	BJ982691
E_FL_BmN-_09M10_R_0	BJ989000
E_FL_BmN-_09M11_F_0	BJ982692
E_FL_BmN-_09M12_F_0	BJ982693
E_FL_BmN-_09M12_R_0	BJ989001
E_FL_BmN-_09M14_F_0	BJ982694
E_FL_BmN-_09M14_R_0	BJ989002
E_FL_BmN-_09M17_F_0	BJ982695
E_FL_BmN-_09M17_R_0	BJ989003
E_FL_BmN-_09M18_F_0	BJ982696
E_FL_BmN-_09M18_R_0	BJ989004
E_FL_BmN-_09M19_F_0	BJ982697
E_FL_BmN-_09M19_R_0	BJ989005
E_FL_BmN-_09M20_R_0	BJ989006
E_FL_BmN-_09M21_F_0	BJ982698
E_FL_BmN-_09M21_R_0	BJ989007
E_FL_BmN-_09M22_F_0	BJ982699
E_FL_BmN-_09M22_R_0	BJ989008
E_FL_BmN-_09M23_F_0	BJ982700
E_FL_BmN-_09M23_R_0	BJ989009
E_FL_BmN-_09M24_F_0	BJ982701
E_FL_BmN-_09M24_R_0	BJ989010
E_FL_BmN-_09N01_F_0	BJ982702
E_FL_BmN-_09N02_F_0	BJ982703
E_FL_BmN-_09N02_R_0	BJ989011
E_FL_BmN-_09N03_F_0	BJ982704
E_FL_BmN-_09N03_R_0	BJ989012
E_FL_BmN-_09N04_F_0	BJ982705
E_FL_BmN-_09N04_R_0	BJ989013
E_FL_BmN-_09N05_F_0	BJ982706
E_FL_BmN-_09N05_R_0	BJ989014
E_FL_BmN-_09N06_R_0	BJ989015
E_FL_BmN-_09N07_F_0	BJ982707

E_FL_BmN-_09N07_R_0	BJ989016
E_FL_BmN-_09N08_F_0	BJ982708
E_FL_BmN-_09N08_R_0	BJ989017
E_FL_BmN-_09N09_F_0	BJ982709
E_FL_BmN-_09N09_R_0	BJ989018
E_FL_BmN-_09N10_F_0	BJ982710
E_FL_BmN-_09N10_R_0	BJ989019
E_FL_BmN-_09N11_F_0	BJ982711
E_FL_BmN-_09N11_R_0	BJ989020
E_FL_BmN-_09N12_R_0	BJ989021
E_FL_BmN-_09N13_F_0	BJ982712
E_FL_BmN-_09N13_R_0	BJ989022
E_FL_BmN-_09N14_F_0	BJ982713
E_FL_BmN-_09N14_R_0	BJ989023
E_FL_BmN-_09N15_F_0	BJ982714
E_FL_BmN-_09N15_R_0	BJ989024
E_FL_BmN-_09N16_F_0	BJ982715
E_FL_BmN-_09N16_R_0	BJ989025
E_FL_BmN-_09N17_F_0	BJ982716
E_FL_BmN-_09N18_R_0	BJ989026
E_FL_BmN-_09N19_F_0	BJ982717
E_FL_BmN-_09N19_R_0	BJ989027
E_FL_BmN-_09N21_F_0	BJ982718
E_FL_BmN-_09N21_R_0	BJ989028
E_FL_BmN-_09N22_F_0	BJ982719
E_FL_BmN-_09N22_R_0	BJ989029
E_FL_BmN-_09N23_F_0	BJ982720
E_FL_BmN-_09N23_R_0	BJ989030
E_FL_BmN-_09N24_R_0	BJ989031
E_FL_BmN-_09O01_F_0	BJ982721
E_FL_BmN-_09O01_R_0	BJ989032
E_FL_BmN-_09O02_R_0	BJ989033
E_FL_BmN-_09O03_F_0	BJ982722
E_FL_BmN-_09O03_R_0	BJ989034
E_FL_BmN-_09O04_F_0	BJ982723
E_FL_BmN-_09O04_R_0	BJ989035
E_FL_BmN-_09O05_F_0	BJ982724
E_FL_BmN-_09O05_R_0	BJ989036
E_FL_BmN-_09O06_F_0	BJ982725
E_FL_BmN-_09O06_R_0	BJ989037
E_FL_BmN-_09O07_R_0	BJ989038
E_FL_BmN-_09O08_R_0	BJ989039
E_FL_BmN-_09O09_F_0	BJ982726
E_FL_BmN-_09O09_R_0	BJ989040
E_FL_BmN-_09O10_F_0	BJ982727
E_FL_BmN-_09O10_R_0	BJ989041
E_FL_BmN-_09O11_F_0	BJ982728
E_FL_BmN-_09O11_R_0	BJ989042
E_FL_BmN-_09O12_F_0	BJ982729
E_FL_BmN-_09O13_F_0	BJ982730
E_FL_BmN-_09O13_R_0	BJ989043
E_FL_BmN-_09O14_F_0	BJ982731
E_FL_BmN-_09O14_R_0	BJ989044
E_FL_BmN-_09O15_F_0	BJ982732

E_FL_BmN-_09O15_R_0	BJ989045
E_FL_BmN-_09O17_R_0	BJ989046
E_FL_BmN-_09O18_F_0	BJ982733
E_FL_BmN-_09O18_R_0	BJ989047
E_FL_BmN-_09O19_R_0	BJ989048
E_FL_BmN-_09O20_F_0	BJ982734
E_FL_BmN-_09O20_R_0	BJ989049
E_FL_BmN-_09O21_F_0	BJ982735
E_FL_BmN-_09O21_R_0	BJ989050
E_FL_BmN-_09O22_F_0	BJ982736
E_FL_BmN-_09O23_F_0	BJ982737
E_FL_BmN-_09O23_R_0	BJ989051
E_FL_BmN-_09O24_F_0	BJ982738
E_FL_BmN-_09O24_R_0	BJ989052
E_FL_BmN-_09P01_F_0	BJ982739
E_FL_BmN-_09P01_R_0	BJ989053
E_FL_BmN-_09P02_F_0	BJ982740
E_FL_BmN-_09P02_R_0	BJ989054
E_FL_BmN-_09P03_F_0	BJ982741
E_FL_BmN-_09P03_R_0	BJ989055
E_FL_BmN-_09P04_F_0	BJ982742
E_FL_BmN-_09P04_R_0	BJ989056
E_FL_BmN-_09P05_F_0	BJ982743
E_FL_BmN-_09P05_R_0	BJ989057
E_FL_BmN-_09P06_F_0	BJ982744
E_FL_BmN-_09P07_F_0	BJ982745
E_FL_BmN-_09P08_F_0	BJ982746
E_FL_BmN-_09P08_R_0	BJ989058
E_FL_BmN-_09P09_R_0	BJ989059
E_FL_BmN-_09P10_F_0	BJ982747
E_FL_BmN-_09P11_F_0	BJ982748
E_FL_BmN-_09P11_R_0	BJ989060
E_FL_BmN-_09P12_F_0	BJ982749
E_FL_BmN-_09P13_F_0	BJ982750
E_FL_BmN-_09P13_R_0	BJ989061
E_FL_BmN-_09P14_F_0	BJ982751
E_FL_BmN-_09P14_R_0	BJ989062
E_FL_BmN-_09P15_F_0	BJ982752
E_FL_BmN-_09P16_F_0	BJ982753
E_FL_BmN-_09P17_F_0	BJ982754
E_FL_BmN-_09P17_R_0	BJ989063
E_FL_BmN-_09P18_F_0	BJ982755
E_FL_BmN-_09P18_R_0	BJ989064
E_FL_BmN-_09P19_F_0	BJ982756
E_FL_BmN-_09P19_R_0	BJ989065
E_FL_BmN-_09P20_F_0	BJ982757
E_FL_BmN-_09P20_R_0	BJ989066
E_FL_BmN-_09P22_F_0	BJ982758
E_FL_BmN-_09P22_R_0	BJ989067
E_FL_BmN-_09P23_F_0	BJ982759
E_FL_BmN-_09P23_R_0	BJ989068
E_FL_BmN-_09P24_F_0	BJ982760
E_FL_BmN-_09P24_R_0	BJ989069
E_FL_BmN-_10A02_F_0	BJ982761

E_FL_BmN-_10A03_F_0	BJ982762
E_FL_BmN-_10A03_R_0	BJ989070
E_FL_BmN-_10A04_F_0	BJ982763
E_FL_BmN-_10A05_F_0	BJ982764
E_FL_BmN-_10A05_R_0	BJ989071
E_FL_BmN-_10A06_F_0	BJ982765
E_FL_BmN-_10A08_F_0	BJ982766
E_FL_BmN-_10A09_F_0	BJ982767
E_FL_BmN-_10A09_R_0	BJ989072
E_FL_BmN-_10A10_F_0	BJ982768
E_FL_BmN-_10A10_R_0	BJ989073
E_FL_BmN-_10A11_F_0	BJ982769
E_FL_BmN-_10A11_R_0	BJ989074
E_FL_BmN-_10A12_F_0	BJ982770
E_FL_BmN-_10A15_R_0	BJ989075
E_FL_BmN-_10A17_F_0	BJ982771
E_FL_BmN-_10A17_R_0	BJ989076
E_FL_BmN-_10A18_F_0	BJ982772
E_FL_BmN-_10A19_R_0	BJ989077
E_FL_BmN-_10A23_F_0	BJ982773
E_FL_BmN-_10A23_R_0	BJ989078
E_FL_BmN-_10A24_F_0	BJ982774
E_FL_BmN-_10A24_R_0	BJ989079
E_FL_BmN-_10B01_F_0	BJ982775
E_FL_BmN-_10B01_R_0	BJ989080
E_FL_BmN-_10B02_F_0	BJ982776
E_FL_BmN-_10B02_R_0	BJ989081
E_FL_BmN-_10B03_F_0	BJ982777
E_FL_BmN-_10B04_F_0	BJ982778
E_FL_BmN-_10B04_R_0	BJ989082
E_FL_BmN-_10B05_R_0	BJ989083
E_FL_BmN-_10B06_F_0	BJ982779
E_FL_BmN-_10B07_F_0	BJ982780
E_FL_BmN-_10B07_R_0	BJ989084
E_FL_BmN-_10B08_F_0	BJ982781
E_FL_BmN-_10B08_R_0	BJ989085
E_FL_BmN-_10B09_R_0	BJ989086
E_FL_BmN-_10B10_F_0	BJ982782
E_FL_BmN-_10B10_R_0	BJ989087
E_FL_BmN-_10B11_F_0	BJ982783
E_FL_BmN-_10B11_R_0	BJ989088
E_FL_BmN-_10B12_F_0	BJ982784
E_FL_BmN-_10B12_R_0	BJ989089
E_FL_BmN-_10B13_R_0	BJ989090
E_FL_BmN-_10B14_F_0	BJ982785
E_FL_BmN-_10B14_R_0	BJ989091
E_FL_BmN-_10B15_F_0	BJ982786
E_FL_BmN-_10B15_R_0	BJ989092
E_FL_BmN-_10B16_F_0	BJ982787
E_FL_BmN-_10B16_R_0	BJ989093
E_FL_BmN-_10B17_R_0	BJ989094
E_FL_BmN-_10B18_R_0	BJ989095
E_FL_BmN-_10B19_F_0	BJ982788
E_FL_BmN-_10B19_R_0	BJ989096

E_FL_BmN-_10B20_F_0	BJ982789
E_FL_BmN-_10B20_R_0	BJ989097
E_FL_BmN-_10B22_R_0	BJ989098
E_FL_BmN-_10B23_F_0	BJ982790
E_FL_BmN-_10B23_R_0	BJ989099
E_FL_BmN-_10B24_F_0	BJ982791
E_FL_BmN-_10B24_R_0	BJ989100
E_FL_BmN-_10C01_R_0	BJ989101
E_FL_BmN-_10C02_F_0	BJ982792
E_FL_BmN-_10C03_F_0	BJ982793
E_FL_BmN-_10C03_R_0	BJ989102
E_FL_BmN-_10C04_F_0	BJ982794
E_FL_BmN-_10C04_R_0	BJ989103
E_FL_BmN-_10C05_F_0	BJ982795
E_FL_BmN-_10C05_R_0	BJ989104
E_FL_BmN-_10C06_F_0	BJ982796
E_FL_BmN-_10C07_F_0	BJ982797
E_FL_BmN-_10C07_R_0	BJ989105
E_FL_BmN-_10C09_F_0	BJ982798
E_FL_BmN-_10C09_R_0	BJ989106
E_FL_BmN-_10C10_F_0	BJ982799
E_FL_BmN-_10C10_R_0	BJ989107
E_FL_BmN-_10C11_R_0	BJ989108
E_FL_BmN-_10C13_R_0	BJ989109
E_FL_BmN-_10C14_R_0	BJ989110
E_FL_BmN-_10C15_F_0	BJ982800
E_FL_BmN-_10C15_R_0	BJ989111
E_FL_BmN-_10C16_F_0	BJ982801
E_FL_BmN-_10C16_R_0	BJ989112
E_FL_BmN-_10C17_F_0	BJ982802
E_FL_BmN-_10C18_F_0	BJ982803
E_FL_BmN-_10C19_R_0	BJ989113
E_FL_BmN-_10C20_R_0	BJ989114
E_FL_BmN-_10C21_R_0	BJ989115
E_FL_BmN-_10C22_F_0	BJ982804
E_FL_BmN-_10C22_R_0	BJ989116
E_FL_BmN-_10C23_F_0	BJ982805
E_FL_BmN-_10C24_F_0	BJ982806
E_FL_BmN-_10D01_F_0	BJ982807
E_FL_BmN-_10D02_F_0	BJ982808
E_FL_BmN-_10D02_R_0	BJ989117
E_FL_BmN-_10D03_R_0	BJ989118
E_FL_BmN-_10D04_F_0	BJ982809
E_FL_BmN-_10D04_R_0	BJ989119
E_FL_BmN-_10D05_F_0	BJ982810
E_FL_BmN-_10D06_F_0	BJ982811
E_FL_BmN-_10D07_F_0	BJ982812
E_FL_BmN-_10D08_F_0	BJ982813
E_FL_BmN-_10D11_F_0	BJ982814
E_FL_BmN-_10D12_F_0	BJ982815
E_FL_BmN-_10D13_F_0	BJ982816
E_FL_BmN-_10D13_R_0	BJ989120
E_FL_BmN-_10D14_F_0	BJ982817
E_FL_BmN-_10D14_R_0	BJ989121

E_FL_BmN-_10D15_R_0	BJ989122
E_FL_BmN-_10D16_F_0	BJ982818
E_FL_BmN-_10D16_R_0	BJ989123
E_FL_BmN-_10D17_F_0	BJ982819
E_FL_BmN-_10D19_F_0	BJ982820
E_FL_BmN-_10D19_R_0	BJ989124
E_FL_BmN-_10D20_F_0	BJ982821
E_FL_BmN-_10D20_R_0	BJ989125
E_FL_BmN-_10D23_F_0	BJ982822
E_FL_BmN-_10D23_R_0	BJ989126
E_FL_BmN-_10D24_F_0	BJ982823
E_FL_BmN-_10D24_R_0	BJ989127
E_FL_BmN-_10E01_F_0	BJ982824
E_FL_BmN-_10E02_R_0	BJ989128
E_FL_BmN-_10E03_F_0	BJ982825
E_FL_BmN-_10E04_F_0	BJ982826
E_FL_BmN-_10E06_F_0	BJ982827
E_FL_BmN-_10E06_R_0	BJ989129
E_FL_BmN-_10E07_F_0	BJ982828
E_FL_BmN-_10E07_R_0	BJ989130
E_FL_BmN-_10E08_F_0	BJ982829
E_FL_BmN-_10E08_R_0	BJ989131
E_FL_BmN-_10E09_F_0	BJ982830
E_FL_BmN-_10E09_R_0	BJ989132
E_FL_BmN-_10E10_R_0	BJ989133
E_FL_BmN-_10E11_F_0	BJ982831
E_FL_BmN-_10E11_R_0	BJ989134
E_FL_BmN-_10E12_F_0	BJ982832
E_FL_BmN-_10E12_R_0	BJ989135
E_FL_BmN-_10E13_F_0	BJ982833
E_FL_BmN-_10E13_R_0	BJ989136
E_FL_BmN-_10E14_R_0	BJ989137
E_FL_BmN-_10E15_F_0	BJ982834
E_FL_BmN-_10E15_R_0	BJ989138
E_FL_BmN-_10E16_F_0	BJ982835
E_FL_BmN-_10E16_R_0	BJ989139
E_FL_BmN-_10E17_F_0	BJ982836
E_FL_BmN-_10E17_R_0	BJ989140
E_FL_BmN-_10E18_F_0	BJ982837
E_FL_BmN-_10E18_R_0	BJ989141
E_FL_BmN-_10E19_R_0	BJ989142
E_FL_BmN-_10E21_R_0	BJ989143
E_FL_BmN-_10E22_R_0	BJ989144
E_FL_BmN-_10E23_F_0	BJ982838
E_FL_BmN-_10E24_F_0	BJ982839
E_FL_BmN-_10E24_R_0	BJ989145
E_FL_BmN-_10F01_F_0	BJ982840
E_FL_BmN-_10F01_R_0	BJ989146
E_FL_BmN-_10F02_F_0	BJ982841
E_FL_BmN-_10F02_R_0	BJ989147
E_FL_BmN-_10F03_F_0	BJ982842
E_FL_BmN-_10F04_F_0	BJ982843
E_FL_BmN-_10F04_R_0	BJ989148
E_FL_BmN-_10F06_F_0	BJ982844

E_FL_BmN-_10F07_F_0	BJ982845
E_FL_BmN-_10F08_F_0	BJ982846
E_FL_BmN-_10F08_R_0	BJ989149
E_FL_BmN-_10F10_R_0	BJ989150
E_FL_BmN-_10F11_F_0	BJ982847
E_FL_BmN-_10F13_F_0	BJ982848
E_FL_BmN-_10F13_R_0	BJ989151
E_FL_BmN-_10F14_F_0	BJ982849
E_FL_BmN-_10F14_R_0	BJ989152
E_FL_BmN-_10F15_R_0	BJ989153
E_FL_BmN-_10F16_F_0	BJ982850
E_FL_BmN-_10F16_R_0	BJ989154
E_FL_BmN-_10F19_F_0	BJ982851
E_FL_BmN-_10F19_R_0	BJ989155
E_FL_BmN-_10F20_F_0	BJ982852
E_FL_BmN-_10F20_R_0	BJ989156
E_FL_BmN-_10F21_R_0	BJ989157
E_FL_BmN-_10F22_R_0	BJ989158
E_FL_BmN-_10F23_F_0	BJ982853
E_FL_BmN-_10F24_F_0	BJ982854
E_FL_BmN-_10F24_R_0	BJ989159
E_FL_BmN-_10G01_F_0	BJ982855
E_FL_BmN-_10G02_F_0	BJ982856
E_FL_BmN-_10G02_R_0	BJ989160
E_FL_BmN-_10G03_F_0	BJ982857
E_FL_BmN-_10G03_R_0	BJ989161
E_FL_BmN-_10G04_F_0	BJ982858
E_FL_BmN-_10G04_R_0	BJ989162
E_FL_BmN-_10G05_F_0	BJ982859
E_FL_BmN-_10G06_F_0	BJ982860
E_FL_BmN-_10G07_F_0	BJ982861
E_FL_BmN-_10G08_R_0	BJ989163
E_FL_BmN-_10G09_F_0	BJ982862
E_FL_BmN-_10G09_R_0	BJ989164
E_FL_BmN-_10G10_F_0	BJ982863
E_FL_BmN-_10G11_F_0	BJ982864
E_FL_BmN-_10G11_R_0	BJ989165
E_FL_BmN-_10G13_F_0	BJ982865
E_FL_BmN-_10G13_R_0	BJ989166
E_FL_BmN-_10G14_F_0	BJ982866
E_FL_BmN-_10G14_R_0	BJ989167
E_FL_BmN-_10G16_F_0	BJ982867
E_FL_BmN-_10G16_R_0	BJ989168
E_FL_BmN-_10G17_F_0	BJ982868
E_FL_BmN-_10G17_R_0	BJ989169
E_FL_BmN-_10G18_F_0	BJ982869
E_FL_BmN-_10G19_R_0	BJ989170
E_FL_BmN-_10G20_R_0	BJ989171
E_FL_BmN-_10G21_F_0	BJ982870
E_FL_BmN-_10G21_R_0	BJ989172
E_FL_BmN-_10G22_F_0	BJ982871
E_FL_BmN-_10G22_R_0	BJ989173
E_FL_BmN-_10G23_F_0	BJ982872
E_FL_BmN-_10G24_F_0	BJ982873

E_FL_BmN-_10G24_R_0	BJ989174
E_FL_BmN-_10H01_F_0	BJ982874
E_FL_BmN-_10H01_R_0	BJ989175
E_FL_BmN-_10H02_F_0	BJ982875
E_FL_BmN-_10H02_R_0	BJ989176
E_FL_BmN-_10H03_F_0	BJ982876
E_FL_BmN-_10H03_R_0	BJ989177
E_FL_BmN-_10H04_R_0	BJ989178
E_FL_BmN-_10H07_F_0	BJ982877
E_FL_BmN-_10H08_F_0	BJ982878
E_FL_BmN-_10H08_R_0	BJ989179
E_FL_BmN-_10H10_F_0	BJ982879
E_FL_BmN-_10H10_R_0	BJ989180
E_FL_BmN-_10H12_F_0	BJ982880
E_FL_BmN-_10H12_R_0	BJ989181
E_FL_BmN-_10H14_F_0	BJ982881
E_FL_BmN-_10H14_R_0	BJ989182
E_FL_BmN-_10H15_F_0	BJ982882
E_FL_BmN-_10H15_R_0	BJ989183
E_FL_BmN-_10H16_F_0	BJ982883
E_FL_BmN-_10H16_R_0	BJ989184
E_FL_BmN-_10H18_F_0	BJ982884
E_FL_BmN-_10H19_F_0	BJ982885
E_FL_BmN-_10H19_R_0	BJ989185
E_FL_BmN-_10H20_F_0	BJ982886
E_FL_BmN-_10H20_R_0	BJ989186
E_FL_BmN-_10H21_F_0	BJ982887
E_FL_BmN-_10H21_R_0	BJ989187
E_FL_BmN-_10H22_F_0	BJ982888
E_FL_BmN-_10H23_F_0	BJ982889
E_FL_BmN-_10H24_F_0	BJ982890
E_FL_BmN-_10H24_R_0	BJ989188
E_FL_BmN-_10I01_F_0	BJ982891
E_FL_BmN-_10I01_R_0	BJ989189
E_FL_BmN-_10I02_F_0	BJ982892
E_FL_BmN-_10I03_F_0	BJ982893
E_FL_BmN-_10I03_R_0	BJ989190
E_FL_BmN-_10I04_F_0	BJ982894
E_FL_BmN-_10I04_R_0	BJ989191
E_FL_BmN-_10I05_F_0	BJ982895
E_FL_BmN-_10I06_F_0	BJ982896
E_FL_BmN-_10I07_F_0	BJ982897
E_FL_BmN-_10I07_R_0	BJ989192
E_FL_BmN-_10I08_F_0	BJ982898
E_FL_BmN-_10I08_R_0	BJ989193
E_FL_BmN-_10I09_F_0	BJ982899
E_FL_BmN-_10I09_R_0	BJ989194
E_FL_BmN-_10I10_F_0	BJ982900
E_FL_BmN-_10I11_F_0	BJ982901
E_FL_BmN-_10I11_R_0	BJ989195
E_FL_BmN-_10I13_F_0	BJ982902
E_FL_BmN-_10I13_R_0	BJ989196
E_FL_BmN-_10I14_F_0	BJ982903
E_FL_BmN-_10I14_R_0	BJ989197

E_FL_BmN-_10I15_F_0	BJ982904
E_FL_BmN-_10I16_F_0	BJ982905
E_FL_BmN-_10I16_R_0	BJ989198
E_FL_BmN-_10I17_R_0	BJ989199
E_FL_BmN-_10I18_F_0	BJ982906
E_FL_BmN-_10I19_R_0	BJ989200
E_FL_BmN-_10I20_R_0	BJ989201
E_FL_BmN-_10I21_F_0	BJ982907
E_FL_BmN-_10I21_R_0	BJ989202
E_FL_BmN-_10I22_F_0	BJ982908
E_FL_BmN-_10I22_R_0	BJ989203
E_FL_BmN-_10I23_F_0	BJ982909
E_FL_BmN-_10I24_F_0	BJ982910
E_FL_BmN-_10J01_F_0	BJ982911
E_FL_BmN-_10J01_R_0	BJ989204
E_FL_BmN-_10J02_F_0	BJ982912
E_FL_BmN-_10J02_R_0	BJ989205
E_FL_BmN-_10J03_F_0	BJ982913
E_FL_BmN-_10J03_R_0	BJ989206
E_FL_BmN-_10J04_R_0	BJ989207
E_FL_BmN-_10J06_F_0	BJ982914
E_FL_BmN-_10J08_R_0	BJ989208
E_FL_BmN-_10J09_R_0	BJ989209
E_FL_BmN-_10J10_F_0	BJ982915
E_FL_BmN-_10J10_R_0	BJ989210
E_FL_BmN-_10J12_F_0	BJ982916
E_FL_BmN-_10J12_R_0	BJ989211
E_FL_BmN-_10J13_F_0	BJ982917
E_FL_BmN-_10J13_R_0	BJ989212
E_FL_BmN-_10J14_F_0	BJ982918
E_FL_BmN-_10J14_R_0	BJ989213
E_FL_BmN-_10J15_F_0	BJ982919
E_FL_BmN-_10J15_R_0	BJ989214
E_FL_BmN-_10J16_F_0	BJ982920
E_FL_BmN-_10J16_R_0	BJ989215
E_FL_BmN-_10J17_F_0	BJ982921
E_FL_BmN-_10J17_R_0	BJ989216
E_FL_BmN-_10J18_F_0	BJ982922
E_FL_BmN-_10J19_F_0	BJ982923
E_FL_BmN-_10J19_R_0	BJ989217
E_FL_BmN-_10J20_R_0	BJ989218
E_FL_BmN-_10J21_F_0	BJ982924
E_FL_BmN-_10J21_R_0	BJ989219
E_FL_BmN-_10J22_R_0	BJ989220
E_FL_BmN-_10J23_F_0	BJ982925
E_FL_BmN-_10J23_R_0	BJ989221
E_FL_BmN-_10J24_F_0	BJ982926
E_FL_BmN-_10K01_F_0	BJ982927
E_FL_BmN-_10K02_F_0	BJ982928
E_FL_BmN-_10K02_R_0	BJ989222
E_FL_BmN-_10K03_F_0	BJ982929
E_FL_BmN-_10K04_F_0	BJ982930
E_FL_BmN-_10K04_R_0	BJ989223
E_FL_BmN-_10K05_F_0	BJ982931

E_FL_BmN-_10K05_R_0	BJ989224
E_FL_BmN-_10K07_R_0	BJ989225
E_FL_BmN-_10K08_F_0	BJ982932
E_FL_BmN-_10K08_R_0	BJ989226
E_FL_BmN-_10K10_F_0	BJ982933
E_FL_BmN-_10K10_R_0	BJ989227
E_FL_BmN-_10K11_F_0	BJ982934
E_FL_BmN-_10K13_F_0	BJ982935
E_FL_BmN-_10K13_R_0	BJ989228
E_FL_BmN-_10K14_R_0	BJ989229
E_FL_BmN-_10K15_F_0	BJ982936
E_FL_BmN-_10K15_R_0	BJ989230
E_FL_BmN-_10K16_F_0	BJ982937
E_FL_BmN-_10K16_R_0	BJ989231
E_FL_BmN-_10K19_R_0	BJ989232
E_FL_BmN-_10K20_R_0	BJ989233
E_FL_BmN-_10K21_F_0	BJ982938
E_FL_BmN-_10K21_R_0	BJ989234
E_FL_BmN-_10K23_F_0	BJ982939
E_FL_BmN-_10K24_R_0	BJ989235
E_FL_BmN-_10L01_F_0	BJ982940
E_FL_BmN-_10L01_R_0	BJ989236
E_FL_BmN-_10L02_F_0	BJ982941
E_FL_BmN-_10L02_R_0	BJ989237
E_FL_BmN-_10L03_F_0	BJ982942
E_FL_BmN-_10L04_R_0	BJ989238
E_FL_BmN-_10L05_F_0	BJ982943
E_FL_BmN-_10L07_F_0	BJ982944
E_FL_BmN-_10L07_R_0	BJ989239
E_FL_BmN-_10L08_F_0	BJ982945
E_FL_BmN-_10L11_F_0	BJ982946
E_FL_BmN-_10L13_F_0	BJ982947
E_FL_BmN-_10L13_R_0	BJ989240
E_FL_BmN-_10L14_F_0	BJ982948
E_FL_BmN-_10L16_R_0	BJ989241
E_FL_BmN-_10L17_F_0	BJ982949
E_FL_BmN-_10L17_R_0	BJ989242
E_FL_BmN-_10L19_F_0	BJ982950
E_FL_BmN-_10L19_R_0	BJ989243
E_FL_BmN-_10L20_R_0	BJ989244
E_FL_BmN-_10L22_R_0	BJ989245
E_FL_BmN-_10L23_F_0	BJ982951
E_FL_BmN-_10L24_R_0	BJ989246
E_FL_BmN-_10M01_F_0	BJ982952
E_FL_BmN-_10M02_R_0	BJ989247
E_FL_BmN-_10M03_F_0	BJ982953
E_FL_BmN-_10M03_R_0	BJ989248
E_FL_BmN-_10M04_F_0	BJ982954
E_FL_BmN-_10M04_R_0	BJ989249
E_FL_BmN-_10M06_F_0	BJ982955
E_FL_BmN-_10M07_F_0	BJ982956
E_FL_BmN-_10M08_F_0	BJ982957
E_FL_BmN-_10M08_R_0	BJ989250
E_FL_BmN-_10M09_F_0	BJ982958

E_FL_BmN-_10M09_R_0	BJ989251
E_FL_BmN-_10M10_F_0	BJ982959
E_FL_BmN-_10M10_R_0	BJ989252
E_FL_BmN-_10M11_F_0	BJ982960
E_FL_BmN-_10M11_R_0	BJ989253
E_FL_BmN-_10M12_F_0	BJ982961
E_FL_BmN-_10M12_R_0	BJ989254
E_FL_BmN-_10M13_F_0	BJ982962
E_FL_BmN-_10M13_R_0	BJ989255
E_FL_BmN-_10M14_F_0	BJ982963
E_FL_BmN-_10M14_R_0	BJ989256
E_FL_BmN-_10M15_R_0	BJ989257
E_FL_BmN-_10M16_F_0	BJ982964
E_FL_BmN-_10M18_F_0	BJ982965
E_FL_BmN-_10M19_R_0	BJ989258
E_FL_BmN-_10M20_R_0	BJ989259
E_FL_BmN-_10M21_F_0	BJ982966
E_FL_BmN-_10M21_R_0	BJ989260
E_FL_BmN-_10M22_F_0	BJ982967
E_FL_BmN-_10M23_F_0	BJ982968
E_FL_BmN-_10M24_F_0	BJ982969
E_FL_BmN-_10M24_R_0	BJ989261
E_FL_BmN-_10N01_F_0	BJ982970
E_FL_BmN-_10N01_R_0	BJ989262
E_FL_BmN-_10N02_F_0	BJ982971
E_FL_BmN-_10N02_R_0	BJ989263
E_FL_BmN-_10N03_R_0	BJ989264
E_FL_BmN-_10N04_F_0	BJ982972
E_FL_BmN-_10N04_R_0	BJ989265
E_FL_BmN-_10N05_F_0	BJ982973
E_FL_BmN-_10N05_R_0	BJ989266
E_FL_BmN-_10N07_F_0	BJ982974
E_FL_BmN-_10N07_R_0	BJ989267
E_FL_BmN-_10N08_F_0	BJ982975
E_FL_BmN-_10N08_R_0	BJ989268
E_FL_BmN-_10N10_R_0	BJ989269
E_FL_BmN-_10N11_F_0	BJ982976
E_FL_BmN-_10N13_F_0	BJ982977
E_FL_BmN-_10N13_R_0	BJ989270
E_FL_BmN-_10N14_F_0	BJ982978
E_FL_BmN-_10N14_R_0	BJ989271
E_FL_BmN-_10N15_F_0	BJ982979
E_FL_BmN-_10N15_R_0	BJ989272
E_FL_BmN-_10N16_F_0	BJ982980
E_FL_BmN-_10N18_R_0	BJ989273
E_FL_BmN-_10N19_F_0	BJ982981
E_FL_BmN-_10N19_R_0	BJ989274
E_FL_BmN-_10N20_F_0	BJ982982
E_FL_BmN-_10N20_R_0	BJ989275
E_FL_BmN-_10N21_R_0	BJ989276
E_FL_BmN-_10N22_F_0	BJ982983
E_FL_BmN-_10O01_F_0	BJ982984
E_FL_BmN-_10O02_F_0	BJ982985
E_FL_BmN-_10O02_R_0	BJ989277

E_FL_BmN-_10O03_F_0	BJ982986
E_FL_BmN-_10O04_F_0	BJ982987
E_FL_BmN-_10O04_R_0	BJ989278
E_FL_BmN-_10O05_F_0	BJ982988
E_FL_BmN-_10O05_R_0	BJ989279
E_FL_BmN-_10O06_F_0	BJ982989
E_FL_BmN-_10O07_R_0	BJ989280
E_FL_BmN-_10O08_F_0	BJ982990
E_FL_BmN-_10O08_R_0	BJ989281
E_FL_BmN-_10O09_F_0	BJ982991
E_FL_BmN-_10O09_R_0	BJ989282
E_FL_BmN-_10O10_F_0	BJ982992
E_FL_BmN-_10O11_F_0	BJ982993
E_FL_BmN-_10O11_R_0	BJ989283
E_FL_BmN-_10O12_F_0	BJ982994
E_FL_BmN-_10O13_F_0	BJ982995
E_FL_BmN-_10O13_R_0	BJ989284
E_FL_BmN-_10O14_F_0	BJ982996
E_FL_BmN-_10O14_R_0	BJ989285
E_FL_BmN-_10O15_F_0	BJ982997
E_FL_BmN-_10O15_R_0	BJ989286
E_FL_BmN-_10O18_R_0	BJ989287
E_FL_BmN-_10O19_R_0	BJ989288
E_FL_BmN-_10O20_R_0	BJ989289
E_FL_BmN-_10O21_F_0	BJ982998
E_FL_BmN-_10O21_R_0	BJ989290
E_FL_BmN-_10O22_F_0	BJ982999
E_FL_BmN-_10O22_R_0	BJ989291
E_FL_BmN-_10O23_F_0	BJ983000
E_FL_BmN-_10O24_F_0	BJ983001
E_FL_BmN-_10P01_F_0	BJ983002
E_FL_BmN-_10P03_R_0	BJ989292
E_FL_BmN-_10P04_F_0	BJ983003
E_FL_BmN-_10P05_F_0	BJ983004
E_FL_BmN-_10P05_R_0	BJ989293
E_FL_BmN-_10P07_F_0	BJ983005
E_FL_BmN-_10P08_F_0	BJ983006
E_FL_BmN-_10P10_F_0	BJ983007
E_FL_BmN-_10P10_R_0	BJ989294
E_FL_BmN-_10P12_F_0	BJ983008
E_FL_BmN-_10P12_R_0	BJ989295
E_FL_BmN-_10P13_F_0	BJ983009
E_FL_BmN-_10P13_R_0	BJ989296
E_FL_BmN-_10P14_F_0	BJ983010
E_FL_BmN-_10P14_R_0	BJ989297
E_FL_BmN-_10P15_F_0	BJ983011
E_FL_BmN-_10P15_R_0	BJ989298
E_FL_BmN-_10P16_F_0	BJ983012
E_FL_BmN-_10P16_R_0	BJ989299
E_FL_BmN-_10P17_F_0	BJ983013
E_FL_BmN-_10P18_F_0	BJ983014
E_FL_BmN-_10P18_R_0	BJ989300
E_FL_BmN-_10P19_F_0	BJ983015
E_FL_BmN-_10P19_R_0	BJ989301

E_FL_BmN-_10P20_F_0	BJ983016
E_FL_BmN-_10P20_R_0	BJ989302
E_FL_BmN-_10P21_R_0	BJ989303
E_FL_BmN-_10P22_F_0	BJ983017
E_FL_BmN-_10P22_R_0	BJ989304
E_FL_BmN-_10P23_F_0	BJ983018
E_FL_BmN-_11A02_F_0	BJ983019
E_FL_BmN-_11A03_F_0	BJ983020
E_FL_BmN-_11A03_R_0	BJ989305
E_FL_BmN-_11A04_F_0	BJ983021
E_FL_BmN-_11A04_R_0	BJ989306
E_FL_BmN-_11A05_F_0	BJ983022
E_FL_BmN-_11A05_R_0	BJ989307
E_FL_BmN-_11A06_F_0	BJ983023
E_FL_BmN-_11A07_F_0	BJ983024
E_FL_BmN-_11A07_R_0	BJ989308
E_FL_BmN-_11A08_F_0	BJ983025
E_FL_BmN-_11A09_F_0	BJ983026
E_FL_BmN-_11A09_R_0	BJ989309
E_FL_BmN-_11A11_F_0	BJ983027
E_FL_BmN-_11A11_R_0	BJ989310
E_FL_BmN-_11A12_F_0	BJ983028
E_FL_BmN-_11A13_R_0	BJ989311
E_FL_BmN-_11A15_F_0	BJ983029
E_FL_BmN-_11A15_R_0	BJ989312
E_FL_BmN-_11A18_F_0	BJ983030
E_FL_BmN-_11A19_R_0	BJ989313
E_FL_BmN-_11A21_F_0	BJ983031
E_FL_BmN-_11A22_F_0	BJ983032
E_FL_BmN-_11A23_F_0	BJ983033
E_FL_BmN-_11A23_R_0	BJ989314
E_FL_BmN-_11A24_F_0	BJ983034
E_FL_BmN-_11B01_F_0	BJ983035
E_FL_BmN-_11B01_R_0	BJ989315
E_FL_BmN-_11B02_F_0	BJ983036
E_FL_BmN-_11B02_R_0	BJ989316
E_FL_BmN-_11B03_F_0	BJ983037
E_FL_BmN-_11B03_R_0	BJ989317
E_FL_BmN-_11B04_R_0	BJ989318
E_FL_BmN-_11B08_F_0	BJ983038
E_FL_BmN-_11B09_F_0	BJ983039
E_FL_BmN-_11B11_R_0	BJ989319
E_FL_BmN-_11B12_F_0	BJ983040
E_FL_BmN-_11B12_R_0	BJ989320
E_FL_BmN-_11B13_R_0	BJ989321
E_FL_BmN-_11B14_F_0	BJ983041
E_FL_BmN-_11B15_R_0	BJ989322
E_FL_BmN-_11B16_F_0	BJ983042
E_FL_BmN-_11B16_R_0	BJ989323
E_FL_BmN-_11B17_R_0	BJ989324
E_FL_BmN-_11B18_F_0	BJ983043
E_FL_BmN-_11B19_F_0	BJ983044
E_FL_BmN-_11B19_R_0	BJ989325
E_FL_BmN-_11B20_F_0	BJ983045

E_FL_BmN-_11B21_F_0	BJ983046
E_FL_BmN-_11B21_R_0	BJ989326
E_FL_BmN-_11B22_R_0	BJ989327
E_FL_BmN-_11B23_F_0	BJ983047
E_FL_BmN-_11B23_R_0	BJ989328
E_FL_BmN-_11B24_F_0	BJ983048
E_FL_BmN-_11C01_F_0	BJ983049
E_FL_BmN-_11C01_R_0	BJ989329
E_FL_BmN-_11C03_F_0	BJ983050
E_FL_BmN-_11C03_R_0	BJ989330
E_FL_BmN-_11C04_F_0	BJ983051
E_FL_BmN-_11C05_F_0	BJ983052
E_FL_BmN-_11C06_F_0	BJ983053
E_FL_BmN-_11C07_R_0	BJ989331
E_FL_BmN-_11C08_F_0	BJ983054
E_FL_BmN-_11C09_F_0	BJ983055
E_FL_BmN-_11C09_R_0	BJ989332
E_FL_BmN-_11C10_F_0	BJ983056
E_FL_BmN-_11C11_F_0	BJ983057
E_FL_BmN-_11C12_F_0	BJ983058
E_FL_BmN-_11C13_R_0	BJ989333
E_FL_BmN-_11C14_F_0	BJ983059
E_FL_BmN-_11C15_F_0	BJ983060
E_FL_BmN-_11C15_R_0	BJ989334
E_FL_BmN-_11C17_F_0	BJ983061
E_FL_BmN-_11C18_F_0	BJ983062
E_FL_BmN-_11C19_R_0	BJ989335
E_FL_BmN-_11C20_F_0	BJ983063
E_FL_BmN-_11C20_R_0	BJ989336
E_FL_BmN-_11C21_F_0	BJ983064
E_FL_BmN-_11C21_R_0	BJ989337
E_FL_BmN-_11C22_F_0	BJ983065
E_FL_BmN-_11C22_R_0	BJ989338
E_FL_BmN-_11C23_F_0	BJ983066
E_FL_BmN-_11C23_R_0	BJ989339
E_FL_BmN-_11D01_F_0	BJ983067
E_FL_BmN-_11D01_R_0	BJ989340
E_FL_BmN-_11D02_F_0	BJ983068
E_FL_BmN-_11D03_R_0	BJ989341
E_FL_BmN-_11D04_F_0	BJ983069
E_FL_BmN-_11D07_F_0	BJ983070
E_FL_BmN-_11D08_F_0	BJ983071
E_FL_BmN-_11D08_R_0	BJ989342
E_FL_BmN-_11D09_F_0	BJ983072
E_FL_BmN-_11D09_R_0	BJ989343
E_FL_BmN-_11D10_F_0	BJ983073
E_FL_BmN-_11D10_R_0	BJ989344
E_FL_BmN-_11D11_F_0	BJ983074
E_FL_BmN-_11D12_F_0	BJ983075
E_FL_BmN-_11D12_R_0	BJ989345
E_FL_BmN-_11D13_F_0	BJ983076
E_FL_BmN-_11D13_R_0	BJ989346
E_FL_BmN-_11D14_F_0	BJ983077
E_FL_BmN-_11D15_F_0	BJ983078

E_FL_BmN-_11D15_R_0	BJ989347
E_FL_BmN-_11D16_R_0	BJ989348
E_FL_BmN-_11D17_F_0	BJ983079
E_FL_BmN-_11D19_F_0	BJ983080
E_FL_BmN-_11D19_R_0	BJ989349
E_FL_BmN-_11D20_F_0	BJ983081
E_FL_BmN-_11D20_R_0	BJ989350
E_FL_BmN-_11D21_F_0	BJ983082
E_FL_BmN-_11D22_F_0	BJ983083
E_FL_BmN-_11D22_R_0	BJ989351
E_FL_BmN-_11D23_F_0	BJ983084
E_FL_BmN-_11D23_R_0	BJ989352
E_FL_BmN-_11D24_F_0	BJ983085
E_FL_BmN-_11E03_F_0	BJ983086
E_FL_BmN-_11E03_R_0	BJ989353
E_FL_BmN-_11E04_R_0	BJ989354
E_FL_BmN-_11E05_F_0	BJ983087
E_FL_BmN-_11E05_R_0	BJ989355
E_FL_BmN-_11E07_F_0	BJ983088
E_FL_BmN-_11E07_R_0	BJ989356
E_FL_BmN-_11E08_F_0	BJ983089
E_FL_BmN-_11E09_R_0	BJ989357
E_FL_BmN-_11E10_F_0	BJ983090
E_FL_BmN-_11E10_R_0	BJ989358
E_FL_BmN-_11E11_F_0	BJ983091
E_FL_BmN-_11E13_F_0	BJ983092
E_FL_BmN-_11E13_R_0	BJ989359
E_FL_BmN-_11E14_F_0	BJ983093
E_FL_BmN-_11E14_R_0	BJ989360
E_FL_BmN-_11E15_F_0	BJ983094
E_FL_BmN-_11E15_R_0	BJ989361
E_FL_BmN-_11E17_F_0	BJ983095
E_FL_BmN-_11E17_R_0	BJ989362
E_FL_BmN-_11E19_R_0	BJ989363
E_FL_BmN-_11E20_F_0	BJ983096
E_FL_BmN-_11E20_R_0	BJ989364
E_FL_BmN-_11E21_F_0	BJ983097
E_FL_BmN-_11E21_R_0	BJ989365
E_FL_BmN-_11E22_F_0	BJ983098
E_FL_BmN-_11E23_F_0	BJ983099
E_FL_BmN-_11E24_F_0	BJ983100
E_FL_BmN-_11F01_R_0	BJ989366
E_FL_BmN-_11F02_F_0	BJ983101
E_FL_BmN-_11F03_F_0	BJ983102
E_FL_BmN-_11F05_F_0	BJ983103
E_FL_BmN-_11F07_F_0	BJ983104
E_FL_BmN-_11F07_R_0	BJ989367
E_FL_BmN-_11F08_F_0	BJ983105
E_FL_BmN-_11F08_R_0	BJ989368
E_FL_BmN-_11F09_F_0	BJ983106
E_FL_BmN-_11F09_R_0	BJ989369
E_FL_BmN-_11F10_F_0	BJ983107
E_FL_BmN-_11F10_R_0	BJ989370
E_FL_BmN-_11F11_F_0	BJ983108

E_FL_BmN-_11F11_R_0	BJ989371
E_FL_BmN-_11F13_F_0	BJ983109
E_FL_BmN-_11F13_R_0	BJ989372
E_FL_BmN-_11F15_F_0	BJ983110
E_FL_BmN-_11F15_R_0	BJ989373
E_FL_BmN-_11F16_R_0	BJ989374
E_FL_BmN-_11F17_F_0	BJ983111
E_FL_BmN-_11F20_R_0	BJ989375
E_FL_BmN-_11F21_R_0	BJ989376
E_FL_BmN-_11F22_R_0	BJ989377
E_FL_BmN-_11F24_F_0	BJ983112
E_FL_BmN-_11G01_F_0	BJ983113
E_FL_BmN-_11G01_R_0	BJ989378
E_FL_BmN-_11G03_R_0	BJ989379
E_FL_BmN-_11G05_F_0	BJ983114
E_FL_BmN-_11G05_R_0	BJ989380
E_FL_BmN-_11G09_R_0	BJ989381
E_FL_BmN-_11G10_R_0	BJ989382
E_FL_BmN-_11G11_F_0	BJ983115
E_FL_BmN-_11G12_R_0	BJ989383
E_FL_BmN-_11G13_F_0	BJ983116
E_FL_BmN-_11G13_R_0	BJ989384
E_FL_BmN-_11G14_R_0	BJ989385
E_FL_BmN-_11G15_R_0	BJ989386
E_FL_BmN-_11G17_F_0	BJ983117
E_FL_BmN-_11G17_R_0	BJ989387
E_FL_BmN-_11G19_F_0	BJ983118
E_FL_BmN-_11G19_R_0	BJ989388
E_FL_BmN-_11G20_F_0	BJ983119
E_FL_BmN-_11G20_R_0	BJ989389
E_FL_BmN-_11G21_F_0	BJ983120
E_FL_BmN-_11G21_R_0	BJ989390
E_FL_BmN-_11G22_F_0	BJ983121
E_FL_BmN-_11G24_F_0	BJ983122
E_FL_BmN-_11G24_R_0	BJ989391
E_FL_BmN-_11H01_F_0	BJ983123
E_FL_BmN-_11H02_F_0	BJ983124
E_FL_BmN-_11H02_R_0	BJ989392
E_FL_BmN-_11H03_R_0	BJ989393
E_FL_BmN-_11H05_F_0	BJ983125
E_FL_BmN-_11H06_F_0	BJ983126
E_FL_BmN-_11H07_F_0	BJ983127
E_FL_BmN-_11H08_F_0	BJ983128
E_FL_BmN-_11H10_F_0	BJ983129
E_FL_BmN-_11H10_R_0	BJ989394
E_FL_BmN-_11H12_F_0	DB668631
E_FL_BmN-_11H13_F_0	DB668632
E_FL_BmN-_11H14_F_0	DB668633
E_FL_BmN-_11H17_F_0	DB668634
E_FL_BmN-_11H18_F_0	DB668635
E_FL_BmN-_11H19_F_0	DB668636
E_FL_BmN-_11H20_F_0	DB668637
E_FL_BmN-_11H21_F_0	DB668638
E_FL_BmN-_11H24_F_0	DB668639

E_FL_BmN-_11I01_F_0	DB668640
E_FL_BmN-_11I04_F_0	DB668641
E_FL_BmN-_11I05_F_0	DB668642
E_FL_BmN-_11I07_F_0	DB668643
E_FL_BmN-_11I08_F_0	DB668644
E_FL_BmN-_11I09_F_0	DB668645
E_FL_BmN-_11I10_F_0	DB668646
E_FL_BmN-_11I11_F_0	DB668647
E_FL_BmN-_11I14_F_0	DB668648
E_FL_BmN-_11I15_F_0	DB668649
E_FL_BmN-_11I16_F_0	DB668650
E_FL_BmN-_11I17_F_0	DB668651
E_FL_BmN-_11I19_F_0	DB668652
E_FL_BmN-_11I20_F_0	DB668653
E_FL_BmN-_11I21_F_0	DB668654
E_FL_BmN-_11I23_F_0	DB668655
E_FL_BmN-_11J07_F_0	DB668656
E_FL_BmN-_11J08_F_0	DB668657
E_FL_BmN-_11J09_F_0	DB668658
E_FL_BmN-_11J10_F_0	DB668659
E_FL_BmN-_11J12_F_0	DB668660
E_FL_BmN-_11J13_F_0	DB668661
E_FL_BmN-_11J18_F_0	DB668662
E_FL_BmN-_11J20_F_0	DB668663
E_FL_BmN-_11J23_F_0	DB668664
E_FL_BmN-_11J24_F_0	DB668665
E_FL_BmN-_11K03_F_0	DB668666
E_FL_BmN-_11K07_F_0	DB668667
E_FL_BmN-_11K09_F_0	DB668668
E_FL_BmN-_11K10_F_0	DB668669
E_FL_BmN-_11K11_F_0	DB668670
E_FL_BmN-_11K13_F_0	DB668671
E_FL_BmN-_11K14_F_0	DB668672
E_FL_BmN-_11K15_F_0	DB668673
E_FL_BmN-_11K19_F_0	DB668674
E_FL_BmN-_11K21_F_0	DB668675
E_FL_BmN-_11K23_F_0	DB668676
E_FL_BmN-_11L01_F_0	DB668677
E_FL_BmN-_11L02_F_0	DB668678
E_FL_BmN-_11L04_F_0	DB668679
E_FL_BmN-_11L07_F_0	DB668680
E_FL_BmN-_11L08_F_0	DB668681
E_FL_BmN-_11L10_F_0	DB668682
E_FL_BmN-_11L13_F_0	DB668683
E_FL_BmN-_11L14_F_0	DB668684
E_FL_BmN-_11L17_F_0	DB668685
E_FL_BmN-_11L19_F_0	DB668686
E_FL_BmN-_11L23_F_0	DB668687
E_FL_BmN-_11M01_F_0	DB668688
E_FL_BmN-_11M02_F_0	DB668689
E_FL_BmN-_11M03_F_0	DB668690
E_FL_BmN-_11M05_F_0	DB668691
E_FL_BmN-_11M07_F_0	DB668692
E_FL_BmN-_11M08_F_0	DB668693

E_FL_BmN-_11M09_F_0	DB668694
E_FL_BmN-_11M11_F_0	DB668695
E_FL_BmN-_11M12_F_0	DB668696
E_FL_BmN-_11M16_F_0	DB668697
E_FL_BmN-_11M17_F_0	DB668698
E_FL_BmN-_11M19_F_0	DB668699
E_FL_BmN-_11M21_F_0	DB668700
E_FL_BmN-_11M23_F_0	DB668701
E_FL_BmN-_11N01_F_0	DB668702
E_FL_BmN-_11N07_F_0	DB668703
E_FL_BmN-_11N09_F_0	DB668704
E_FL_BmN-_11N11_F_0	DB668705
E_FL_BmN-_11N13_F_0	DB668706
E_FL_BmN-_11N16_F_0	DB668707
E_FL_BmN-_11N17_F_0	DB668708
E_FL_BmN-_11N19_F_0	DB668709
E_FL_BmN-_11N20_F_0	DB668710
E_FL_BmN-_11N23_F_0	DB668711
E_FL_BmN-_11O02_F_0	DB668712
E_FL_BmN-_11O03_F_0	DB668713
E_FL_BmN-_11O05_F_0	DB668714
E_FL_BmN-_11O07_F_0	DB668715
E_FL_BmN-_11O11_F_0	DB668716
E_FL_BmN-_11O13_F_0	DB668717
E_FL_BmN-_11O15_F_0	DB668718
E_FL_BmN-_11O17_F_0	DB668719
E_FL_BmN-_11O18_F_0	DB668720
E_FL_BmN-_11O19_F_0	DB668721
E_FL_BmN-_11O21_F_0	DB668722
E_FL_BmN-_11O23_F_0	DB668723
E_FL_BmN-_11O24_F_0	DB668724
E_FL_BmN-_11P01_F_0	DB668725
E_FL_BmN-_11P02_F_0	DB668726
E_FL_BmN-_11P03_F_0	DB668727
E_FL_BmN-_11P04_F_0	DB668728
E_FL_BmN-_11P07_F_0	DB668729
E_FL_BmN-_11P08_F_0	DB668730
E_FL_BmN-_11P09_F_0	DB668731
E_FL_BmN-_11P10_F_0	DB668732
E_FL_BmN-_11P12_F_0	DB668733
E_FL_BmN-_11P13_F_0	DB668734
E_FL_BmN-_11P14_F_0	DB668735
E_FL_BmN-_11P15_F_0	DB668736
E_FL_BmN-_11P16_F_0	DB668737
E_FL_BmN-_11P17_F_0	DB668738
E_FL_BmN-_11P18_F_0	DB668739
E_FL_BmN-_11P19_F_0	DB668740
E_FL_BmN-_11P21_F_0	DB668741
E_FL_BmN-_11P22_F_0	DB668742
E_FL_BmN-_11P23_F_0	DB668743
E_FL_BmN-_12A02_F_0	DB668744
E_FL_BmN-_12A04_F_0	DB668745
E_FL_BmN-_12A05_F_0	DB668746
E_FL_BmN-_12A06_F_0	DB668747

	E_FL_BmN-_12A12_F_0	DB668748
	E_FL_BmN-_12A14_F_0	DB668749
	E_FL_BmN-_12A18_F_0	DB668750
	E_FL_BmN-_12A20_F_0	DB668751
	E_FL_BmN-_12A23_F_0	DB668752
	E_FL_BmN-_12A24_F_0	DB668753
	E_FL_BmN-_12B01_F_0	DB668754
	E_FL_BmN-_12B02_F_0	DB668755
	E_FL_BmN-_12B03_F_0	DB668756
	E_FL_BmN-_12B04_F_0	DB668757
	E_FL_BmN-_12B05_F_0	DB668758
	E_FL_BmN-_12B06_F_0	DB668759
	E_FL_BmN-_12B07_F_0	DB668760
	E_FL_BmN-_12B08_F_0	DB668761
	E_FL_BmN-_12B10_F_0	DB668762
	E_FL_BmN-_12B11_F_0	DB668763
	E_FL_BmN-_12B12_F_0	DB668764
	E_FL_BmN-_12B13_F_0	DB668765
	E_FL_BmN-_12B14_F_0	DB668766
	E_FL_BmN-_12B16_F_0	DB668767
	E_FL_BmN-_12B17_F_0	DB668768
	E_FL_BmN-_12B18_F_0	DB668769
	E_FL_BmN-_12B19_F_0	DB668770
	E_FL_BmN-_12B20_F_0	DB668771
	E_FL_BmN-_12B21_F_0	DB668772
	E_FL_BmN-_12B22_F_0	DB668773
e100	E_FL_e100_01A05_F_0	BY937393
	E_FL_e100_01A05_R_0	BY942315
	E_FL_e100_01A06_F_0	BY937394
	E_FL_e100_01A07_R_0	BY942316
	E_FL_e100_01A09_R_0	BY942317
	E_FL_e100_01A10_R_0	BY942318
	E_FL_e100_01A12_F_0	BY937395
	E_FL_e100_01A12_R_0	BY942319
	E_FL_e100_01A13_F_0	BY937396
	E_FL_e100_01A13_R_0	BY942320
	E_FL_e100_01A15_F_0	BY937397
	E_FL_e100_01A15_R_0	BY942321
	E_FL_e100_01A16_R_0	BY942322
	E_FL_e100_01A18_F_0	BY937398
	E_FL_e100_01A18_R_0	BY942323
	E_FL_e100_01A22_F_0	BY937399
	E_FL_e100_01B01_F_0	BY937400
	E_FL_e100_01B01_R_0	BY942324
	E_FL_e100_01B02_R_0	BY942325
	E_FL_e100_01B03_F_0	BY937401
	E_FL_e100_01B03_R_0	BY942326
	E_FL_e100_01B04_F_0	BY937402
	E_FL_e100_01B04_R_0	BY942327
	E_FL_e100_01B05_F_0	BY937403
	E_FL_e100_01B08_F_0	BY937404
	E_FL_e100_01B10_F_0	BY937405
	E_FL_e100_01B12_F_0	BY937406
	E_FL_e100_01B12_R_0	BY942328

E_FL_e100_01B13_F_0	BY937407
E_FL_e100_01B13_R_0	BY942329
E_FL_e100_01B14_F_0	BY937408
E_FL_e100_01B14_R_0	BY942330
E_FL_e100_01B17_F_0	BY937409
E_FL_e100_01B17_R_0	BY942331
E_FL_e100_01B18_F_0	BY937410
E_FL_e100_01B18_R_0	BY942332
E_FL_e100_01B20_F_0	BY937411
E_FL_e100_01B20_R_0	BY942333
E_FL_e100_01B22_F_0	BY937412
E_FL_e100_01C01_F_0	BY937413
E_FL_e100_01C01_R_0	BY942334
E_FL_e100_01C02_F_0	BY937414
E_FL_e100_01C03_F_0	BY937415
E_FL_e100_01C04_F_0	BY937416
E_FL_e100_01C05_F_0	BY937417
E_FL_e100_01C07_F_0	BY937418
E_FL_e100_01C07_R_0	BY942335
E_FL_e100_01C08_F_0	BY937419
E_FL_e100_01C09_R_0	BY942336
E_FL_e100_01C10_F_0	BY937420
E_FL_e100_01C10_R_0	BY942337
E_FL_e100_01C11_F_0	BY937421
E_FL_e100_01C12_R_0	BY942338
E_FL_e100_01C13_R_0	BY942339
E_FL_e100_01C14_F_0	BY937422
E_FL_e100_01C14_R_0	BY942340
E_FL_e100_01C15_F_0	BY937423
E_FL_e100_01C16_F_0	BY937424
E_FL_e100_01C16_R_0	BY942341
E_FL_e100_01C18_F_0	BY937425
E_FL_e100_01C19_F_0	BY937426
E_FL_e100_01C19_R_0	BY942342
E_FL_e100_01C20_F_0	BY937427
E_FL_e100_01C20_R_0	BY942343
E_FL_e100_01C21_F_0	BY937428
E_FL_e100_01C21_R_0	BY942344
E_FL_e100_01C24_F_0	BY937429
E_FL_e100_01C24_R_0	BY942345
E_FL_e100_01D01_F_0	BY937430
E_FL_e100_01D02_F_0	BY937431
E_FL_e100_01D03_F_0	BY937432
E_FL_e100_01D05_F_0	BY937433
E_FL_e100_01D07_F_0	BY937434
E_FL_e100_01D07_R_0	BY942346
E_FL_e100_01D08_F_0	BY937435
E_FL_e100_01D10_F_0	BY937436
E_FL_e100_01D11_F_0	BY937437
E_FL_e100_01D13_F_0	BY937438
E_FL_e100_01D13_R_0	BY942347
E_FL_e100_01D14_F_0	BY937439
E_FL_e100_01D14_R_0	BY942348
E_FL_e100_01D16_R_0	BY942349

E_FL_e100_01D17_F_0	BY937440
E_FL_e100_01D18_F_0	BY937441
E_FL_e100_01D18_R_0	BY942350
E_FL_e100_01D19_F_0	BY937442
E_FL_e100_01D19_R_0	BY942351
E_FL_e100_01D20_F_0	BY937443
E_FL_e100_01D20_R_0	BY942352
E_FL_e100_01D22_F_0	BY937444
E_FL_e100_01D22_R_0	BY942353
E_FL_e100_01D23_F_0	BY937445
E_FL_e100_01D24_F_0	BY937446
E_FL_e100_01E02_F_0	BY937447
E_FL_e100_01E02_R_0	BY942354
E_FL_e100_01E04_F_0	BY937448
E_FL_e100_01E04_R_0	BY942355
E_FL_e100_01E06_F_0	BY937449
E_FL_e100_01E10_F_0	BY937450
E_FL_e100_01E11_F_0	BY937451
E_FL_e100_01E13_F_0	BY937452
E_FL_e100_01E14_R_0	BY942356
E_FL_e100_01E15_F_0	BY937453
E_FL_e100_01E21_F_0	BY937454
E_FL_e100_01E22_F_0	BY937455
E_FL_e100_01E22_R_0	BY942357
E_FL_e100_01E24_F_0	BY937456
E_FL_e100_01E24_R_0	BY942358
E_FL_e100_01F01_F_0	BY937457
E_FL_e100_01F01_R_0	BY942359
E_FL_e100_01F02_F_0	BY937458
E_FL_e100_01F03_R_0	BY942360
E_FL_e100_01F04_F_0	BY937459
E_FL_e100_01F04_R_0	BY942361
E_FL_e100_01F06_F_0	BY937460
E_FL_e100_01F06_R_0	BY942362
E_FL_e100_01F08_F_0	BY937461
E_FL_e100_01F09_F_0	BY937462
E_FL_e100_01F09_R_0	BY942363
E_FL_e100_01F10_F_0	BY937463
E_FL_e100_01F10_R_0	BY942364
E_FL_e100_01F11_F_0	BY937464
E_FL_e100_01F12_F_0	BY937465
E_FL_e100_01F12_R_0	BY942365
E_FL_e100_01F13_R_0	BY942366
E_FL_e100_01F14_F_0	BY937466
E_FL_e100_01F14_R_0	BY942367
E_FL_e100_01F16_F_0	BY937467
E_FL_e100_01F16_R_0	BY942368
E_FL_e100_01F17_F_0	BY937468
E_FL_e100_01F20_R_0	BY942369
E_FL_e100_01F21_F_0	BY937469
E_FL_e100_01F22_F_0	BY937470
E_FL_e100_01F22_R_0	BY942370
E_FL_e100_01F23_F_0	BY937471
E_FL_e100_01F23_R_0	BY942371

E_FL_e100_01F24_F_0	BY937472
E_FL_e100_01F24_R_0	BY942372
E_FL_e100_01G01_F_0	BY937473
E_FL_e100_01G03_F_0	BY937474
E_FL_e100_01G03_R_0	BY942373
E_FL_e100_01G05_F_0	BY937475
E_FL_e100_01G07_F_0	BY937476
E_FL_e100_01G08_F_0	BY937477
E_FL_e100_01G11_F_0	BY937478
E_FL_e100_01G11_R_0	BY942374
E_FL_e100_01G13_F_0	BY937479
E_FL_e100_01G14_F_0	BY937480
E_FL_e100_01G18_F_0	BY937481
E_FL_e100_01G18_R_0	BY942375
E_FL_e100_01G19_F_0	BY937482
E_FL_e100_01G19_R_0	BY942376
E_FL_e100_01G20_F_0	BY937483
E_FL_e100_01G20_R_0	BY942377
E_FL_e100_01G21_F_0	BY937484
E_FL_e100_01G21_R_0	BY942378
E_FL_e100_01G23_F_0	BY937485
E_FL_e100_01G23_R_0	BY942379
E_FL_e100_01H02_F_0	BY937486
E_FL_e100_01H02_R_0	BY942380
E_FL_e100_01H03_F_0	BY937487
E_FL_e100_01H03_R_0	BY942381
E_FL_e100_01H04_F_0	BY937488
E_FL_e100_01H04_R_0	BY942382
E_FL_e100_01H05_F_0	BY937489
E_FL_e100_01H08_F_0	BY937490
E_FL_e100_01H09_R_0	BY942383
E_FL_e100_01H11_F_0	BY937491
E_FL_e100_01H13_R_0	BY942384
E_FL_e100_01H14_F_0	BY937492
E_FL_e100_01H14_R_0	BY942385
E_FL_e100_01H15_R_0	BY942386
E_FL_e100_01H16_F_0	BY937493
E_FL_e100_01H16_R_0	BY942387
E_FL_e100_01H18_F_0	BY937494
E_FL_e100_01H19_F_0	BY937495
E_FL_e100_01H20_F_0	BY937496
E_FL_e100_01H21_F_0	BY937497
E_FL_e100_01H22_F_0	BY937498
E_FL_e100_01I01_F_0	BY937499
E_FL_e100_01I01_R_0	BY942388
E_FL_e100_01I05_F_0	BY937500
E_FL_e100_01I07_R_0	BY942389
E_FL_e100_01I08_F_0	BY937501
E_FL_e100_01I09_F_0	BY937502
E_FL_e100_01I10_F_0	BY937503
E_FL_e100_01I10_R_0	BY942390
E_FL_e100_01I12_R_0	BY942391
E_FL_e100_01I14_R_0	BY942392
E_FL_e100_01I15_F_0	BY937504

E_FL_e100_01I16_F_0	BY937505
E_FL_e100_01I16_R_0	BY942393
E_FL_e100_01I18_F_0	BY937506
E_FL_e100_01I18_R_0	BY942394
E_FL_e100_01I22_F_0	BY937507
E_FL_e100_01I22_R_0	BY942395
E_FL_e100_01I23_F_0	BY937508
E_FL_e100_01I24_F_0	BY937509
E_FL_e100_01I24_R_0	BY942396
E_FL_e100_01J02_F_0	BY937510
E_FL_e100_01J02_R_0	BY942397
E_FL_e100_01J05_F_0	BY937511
E_FL_e100_01J06_F_0	BY937512
E_FL_e100_01J07_F_0	BY937513
E_FL_e100_01J07_R_0	BY942398
E_FL_e100_01J08_F_0	BY937514
E_FL_e100_01J08_R_0	BY942399
E_FL_e100_01J09_F_0	BY937515
E_FL_e100_01J10_F_0	BY937516
E_FL_e100_01J11_F_0	BY937517
E_FL_e100_01J11_R_0	BY942400
E_FL_e100_01J17_F_0	BY937518
E_FL_e100_01J18_F_0	BY937519
E_FL_e100_01J19_F_0	BY937520
E_FL_e100_01J20_F_0	BY937521
E_FL_e100_01J20_R_0	BY942401
E_FL_e100_01J21_F_0	BY937522
E_FL_e100_01J21_R_0	BY942402
E_FL_e100_01J22_F_0	BY937523
E_FL_e100_01J22_R_0	BY942403
E_FL_e100_01J24_F_0	BY937524
E_FL_e100_01K01_R_0	BY942404
E_FL_e100_01K03_F_0	BY937525
E_FL_e100_01K04_F_0	BY937526
E_FL_e100_01K04_R_0	BY942405
E_FL_e100_01K05_F_0	BY937527
E_FL_e100_01K05_R_0	BY942406
E_FL_e100_01K07_F_0	BY937528
E_FL_e100_01K11_R_0	BY942407
E_FL_e100_01K13_F_0	BY937529
E_FL_e100_01K14_F_0	BY937530
E_FL_e100_01K15_F_0	BY937531
E_FL_e100_01K17_F_0	BY937532
E_FL_e100_01K17_R_0	BY942408
E_FL_e100_01K19_F_0	BY937533
E_FL_e100_01K20_F_0	BY937534
E_FL_e100_01K20_R_0	BY942409
E_FL_e100_01K21_F_0	BY937535
E_FL_e100_01K21_R_0	BY942410
E_FL_e100_01K22_F_0	BY937536
E_FL_e100_01K22_R_0	BY942411
E_FL_e100_01K24_R_0	BY942412
E_FL_e100_01L01_F_0	BY937537
E_FL_e100_01L03_F_0	BY937538

E_FL_e100_01L05_F_0	BY937539
E_FL_e100_01L05_R_0	BY942413
E_FL_e100_01L06_F_0	BY937540
E_FL_e100_01L06_R_0	BY942414
E_FL_e100_01L07_F_0	BY937541
E_FL_e100_01L07_R_0	BY942415
E_FL_e100_01L08_F_0	BY937542
E_FL_e100_01L12_F_0	BY937543
E_FL_e100_01L12_R_0	BY942416
E_FL_e100_01L13_F_0	BY937544
E_FL_e100_01L13_R_0	BY942417
E_FL_e100_01L16_F_0	BY937545
E_FL_e100_01L16_R_0	BY942418
E_FL_e100_01L17_F_0	BY937546
E_FL_e100_01L18_F_0	BY937547
E_FL_e100_01L18_R_0	BY942419
E_FL_e100_01L19_F_0	BY937548
E_FL_e100_01L20_F_0	BY937549
E_FL_e100_01L20_R_0	BY942420
E_FL_e100_01L22_F_0	BY937550
E_FL_e100_01L22_R_0	BY942421
E_FL_e100_01M02_F_0	BY937551
E_FL_e100_01M02_R_0	BY942422
E_FL_e100_01M03_F_0	BY937552
E_FL_e100_01M04_F_0	BY937553
E_FL_e100_01M04_R_0	BY942423
E_FL_e100_01M05_F_0	BY937554
E_FL_e100_01M08_F_0	BY937555
E_FL_e100_01M09_F_0	BY937556
E_FL_e100_01M09_R_0	BY942424
E_FL_e100_01M11_F_0	BY937557
E_FL_e100_01M11_R_0	BY942425
E_FL_e100_01M12_F_0	BY937558
E_FL_e100_01M13_F_0	BY937559
E_FL_e100_01M13_R_0	BY942426
E_FL_e100_01M14_F_0	BY937560
E_FL_e100_01M14_R_0	BY942427
E_FL_e100_01M16_F_0	BY937561
E_FL_e100_01M19_F_0	BY937562
E_FL_e100_01M19_R_0	BY942428
E_FL_e100_01M21_F_0	BY937563
E_FL_e100_01M21_R_0	BY942429
E_FL_e100_01M22_F_0	BY937564
E_FL_e100_01M22_R_0	BY942430
E_FL_e100_01M23_F_0	BY937565
E_FL_e100_01M23_R_0	BY942431
E_FL_e100_01M24_F_0	BY937566
E_FL_e100_01N05_R_0	BY942432
E_FL_e100_01N10_F_0	BY937567
E_FL_e100_01N11_F_0	BY937568
E_FL_e100_01N13_R_0	BY942433
E_FL_e100_01N14_F_0	BY937569
E_FL_e100_01N15_F_0	BY937570
E_FL_e100_01N15_R_0	BY942434

E_FL_e100_01N22_F_0	BY937571
E_FL_e100_01O01_F_0	BY937572
E_FL_e100_01O01_R_0	BY942435
E_FL_e100_01O03_F_0	BY937573
E_FL_e100_01O03_R_0	BY942436
E_FL_e100_01O04_F_0	BY937574
E_FL_e100_01O06_F_0	BY937575
E_FL_e100_01O06_R_0	BY942437
E_FL_e100_01O07_F_0	BY937576
E_FL_e100_01O07_R_0	BY942438
E_FL_e100_01O08_F_0	BY937577
E_FL_e100_01O10_F_0	BY937578
E_FL_e100_01O11_F_0	BY937579
E_FL_e100_01O11_R_0	BY942439
E_FL_e100_01O12_F_0	BY937580
E_FL_e100_01O12_R_0	BY942440
E_FL_e100_01O13_F_0	BY937581
E_FL_e100_01O13_R_0	BY942441
E_FL_e100_01O14_R_0	BY942442
E_FL_e100_01O15_R_0	BY942443
E_FL_e100_01O16_F_0	BY937582
E_FL_e100_01O16_R_0	BY942444
E_FL_e100_01O17_F_0	BY937583
E_FL_e100_01O18_R_0	BY942445
E_FL_e100_01O19_R_0	BY942446
E_FL_e100_01O20_F_0	BY937584
E_FL_e100_01O21_F_0	BY937585
E_FL_e100_01O22_F_0	BY937586
E_FL_e100_01O22_R_0	BY942447
E_FL_e100_01P01_F_0	BY937587
E_FL_e100_01P02_R_0	BY942448
E_FL_e100_01P03_F_0	BY937588
E_FL_e100_01P04_F_0	BY937589
E_FL_e100_01P04_R_0	BY942449
E_FL_e100_01P05_F_0	BY937590
E_FL_e100_01P06_F_0	BY937591
E_FL_e100_01P08_F_0	BY937592
E_FL_e100_01P09_F_0	BY937593
E_FL_e100_01P09_R_0	BY942450
E_FL_e100_01P11_F_0	BY937594
E_FL_e100_01P19_F_0	BY937595
E_FL_e100_01P19_R_0	BY942451
E_FL_e100_01P20_F_0	BY937596
E_FL_e100_01P22_F_0	BY937597
E_FL_e100_01P22_R_0	BY942452
E_FL_e100_01P24_F_0	BY937598
E_FL_e100_02A01_F_0	BY937599
E_FL_e100_02A02_F_0	BY937600
E_FL_e100_02A02_R_0	BY942453
E_FL_e100_02A03_F_0	BY937601
E_FL_e100_02A04_F_0	BY937602
E_FL_e100_02A04_R_0	BY942454
E_FL_e100_02A09_R_0	BY942455
E_FL_e100_02A10_F_0	BY937603

E_FL_e100_02A11_F_0	BY937604
E_FL_e100_02A12_F_0	BY937605
E_FL_e100_02A12_R_0	BY942456
E_FL_e100_02A14_F_0	BY937606
E_FL_e100_02A15_F_0	BY937607
E_FL_e100_02A15_R_0	BY942457
E_FL_e100_02A16_F_0	BY937608
E_FL_e100_02A16_R_0	BY942458
E_FL_e100_02A17_F_0	BY937609
E_FL_e100_02A17_R_0	BY942459
E_FL_e100_02A22_F_0	BY937610
E_FL_e100_02A22_R_0	BY942460
E_FL_e100_02B01_F_0	BY937611
E_FL_e100_02B02_F_0	BY937612
E_FL_e100_02B04_F_0	BY937613
E_FL_e100_02B04_R_0	BY942461
E_FL_e100_02B05_F_0	BY937614
E_FL_e100_02B06_F_0	BY937615
E_FL_e100_02B06_R_0	BY942462
E_FL_e100_02B07_R_0	BY942463
E_FL_e100_02B08_F_0	BY937616
E_FL_e100_02B09_F_0	BY937617
E_FL_e100_02B09_R_0	BY942464
E_FL_e100_02B11_F_0	BY937618
E_FL_e100_02B11_R_0	BY942465
E_FL_e100_02B12_F_0	BY937619
E_FL_e100_02B14_F_0	BY937620
E_FL_e100_02B15_F_0	BY937621
E_FL_e100_02B15_R_0	BY942466
E_FL_e100_02B17_F_0	BY937622
E_FL_e100_02B18_F_0	BY937623
E_FL_e100_02B20_F_0	BY937624
E_FL_e100_02B23_F_0	BY937625
E_FL_e100_02B23_R_0	BY942467
E_FL_e100_02B24_F_0	BY937626
E_FL_e100_02C01_F_0	BY937627
E_FL_e100_02C01_R_0	BY942468
E_FL_e100_02C02_F_0	BY937628
E_FL_e100_02C04_R_0	BY942469
E_FL_e100_02C05_F_0	BY937629
E_FL_e100_02C09_F_0	BY937630
E_FL_e100_02C11_F_0	BY937631
E_FL_e100_02C13_F_0	BY937632
E_FL_e100_02C17_F_0	BY937633
E_FL_e100_02C17_R_0	BY942470
E_FL_e100_02C18_R_0	BY942471
E_FL_e100_02C20_F_0	BY937634
E_FL_e100_02C20_R_0	BY942472
E_FL_e100_02C21_F_0	BY937635
E_FL_e100_02C23_F_0	BY937636
E_FL_e100_02C23_R_0	BY942473
E_FL_e100_02C24_R_0	BY942474
E_FL_e100_02D02_F_0	BY937637
E_FL_e100_02D04_F_0	BY937638

E_FL_e100_02D11_F_0	BY937639
E_FL_e100_02D12_F_0	BY937640
E_FL_e100_02D13_F_0	BY937641
E_FL_e100_02D14_F_0	BY937642
E_FL_e100_02D14_R_0	BY942475
E_FL_e100_02D16_F_0	BY937643
E_FL_e100_02D16_R_0	BY942476
E_FL_e100_02D17_F_0	BY937644
E_FL_e100_02D17_R_0	BY942477
E_FL_e100_02D18_F_0	BY937645
E_FL_e100_02D22_F_0	BY937646
E_FL_e100_02D23_F_0	BY937647
E_FL_e100_02D24_F_0	BY937648
E_FL_e100_02D24_R_0	BY942478
E_FL_e100_02E04_F_0	BY937649
E_FL_e100_02E05_F_0	BY937650
E_FL_e100_02E05_R_0	BY942479
E_FL_e100_02E07_F_0	BY937651
E_FL_e100_02E08_F_0	BY937652
E_FL_e100_02E09_F_0	BY937653
E_FL_e100_02E10_F_0	BY937654
E_FL_e100_02E11_F_0	BY937655
E_FL_e100_02E12_F_0	BY937656
E_FL_e100_02E14_F_0	BY937657
E_FL_e100_02E14_R_0	BY942480
E_FL_e100_02E15_F_0	BY937658
E_FL_e100_02E16_F_0	BY937659
E_FL_e100_02E16_R_0	BY942481
E_FL_e100_02E17_F_0	BY937660
E_FL_e100_02E18_F_0	BY937661
E_FL_e100_02E19_F_0	BY937662
E_FL_e100_02E19_R_0	BY942482
E_FL_e100_02E21_F_0	BY937663
E_FL_e100_02E21_R_0	BY942483
E_FL_e100_02E22_F_0	BY937664
E_FL_e100_02E23_F_0	BY937665
E_FL_e100_02E23_R_0	BY942484
E_FL_e100_02F03_R_0	BY942485
E_FL_e100_02F04_F_0	BY937666
E_FL_e100_02F06_F_0	BY937667
E_FL_e100_02F06_R_0	BY942486
E_FL_e100_02F08_F_0	BY937668
E_FL_e100_02F08_R_0	BY942487
E_FL_e100_02F09_R_0	BY942488
E_FL_e100_02F12_F_0	BY937669
E_FL_e100_02F15_F_0	BY937670
E_FL_e100_02F15_R_0	BY942489
E_FL_e100_02F16_F_0	BY937671
E_FL_e100_02F16_R_0	BY942490
E_FL_e100_02F19_F_0	BY937672
E_FL_e100_02F19_R_0	BY942491
E_FL_e100_02F21_F_0	BY937673
E_FL_e100_02F21_R_0	BY942492
E_FL_e100_02F22_F_0	BY937674

E_FL_e100_02F22_R_0	BY942493
E_FL_e100_02F23_F_0	BY937675
E_FL_e100_02F24_F_0	BY937676
E_FL_e100_02G02_F_0	BY937677
E_FL_e100_02G02_R_0	BY942494
E_FL_e100_02G03_F_0	BY937678
E_FL_e100_02G05_F_0	BY937679
E_FL_e100_02G09_F_0	BY937680
E_FL_e100_02G09_R_0	BY942495
E_FL_e100_02G10_F_0	BY937681
E_FL_e100_02G10_R_0	BY942496
E_FL_e100_02G11_F_0	BY937682
E_FL_e100_02G11_R_0	BY942497
E_FL_e100_02G14_R_0	BY942498
E_FL_e100_02G15_F_0	BY937683
E_FL_e100_02G15_R_0	BY942499
E_FL_e100_02G16_F_0	BY937684
E_FL_e100_02G17_F_0	BY937685
E_FL_e100_02G18_F_0	BY937686
E_FL_e100_02G23_F_0	BY937687
E_FL_e100_02G23_R_0	BY942500
E_FL_e100_02G24_F_0	BY937688
E_FL_e100_02H01_F_0	BY937689
E_FL_e100_02H02_F_0	BY937690
E_FL_e100_02H03_F_0	BY937691
E_FL_e100_02H04_F_0	BY937692
E_FL_e100_02H04_R_0	BY942501
E_FL_e100_02H05_F_0	BY937693
E_FL_e100_02H05_R_0	BY942502
E_FL_e100_02H07_F_0	BY937694
E_FL_e100_02H07_R_0	BY942503
E_FL_e100_02H08_F_0	BY937695
E_FL_e100_02H08_R_0	BY942504
E_FL_e100_02H12_F_0	BY937696
E_FL_e100_02H13_F_0	BY937697
E_FL_e100_02H15_F_0	BY937698
E_FL_e100_02H15_R_0	BY942505
E_FL_e100_02H16_F_0	BY937699
E_FL_e100_02H16_R_0	BY942506
E_FL_e100_02H17_F_0	BY937700
E_FL_e100_02H17_R_0	BY942507
E_FL_e100_02H19_F_0	BY937701
E_FL_e100_02H19_R_0	BY942508
E_FL_e100_02H22_F_0	BY937702
E_FL_e100_02H22_R_0	BY942509
E_FL_e100_02H23_F_0	BY937703
E_FL_e100_02H23_R_0	BY942510
E_FL_e100_02H24_F_0	BY937704
E_FL_e100_02H24_R_0	BY942511
E_FL_e100_02I01_F_0	BY937705
E_FL_e100_02I02_F_0	BY937706
E_FL_e100_02I03_F_0	BY937707
E_FL_e100_02I05_R_0	BY942512
E_FL_e100_02I07_F_0	BY937708

E_FL_e100_02I07_R_0	BY942513
E_FL_e100_02I08_F_0	BY937709
E_FL_e100_02I08_R_0	BY942514
E_FL_e100_02I09_F_0	BY937710
E_FL_e100_02I11_F_0	BY937711
E_FL_e100_02I12_F_0	BY937712
E_FL_e100_02I12_R_0	BY942515
E_FL_e100_02I14_R_0	BY942516
E_FL_e100_02I15_F_0	BY937713
E_FL_e100_02I15_R_0	BY942517
E_FL_e100_02I18_F_0	BY937714
E_FL_e100_02I19_F_0	BY937715
E_FL_e100_02I20_F_0	BY937716
E_FL_e100_02I20_R_0	BY942518
E_FL_e100_02I23_F_0	BY937717
E_FL_e100_02J01_F_0	BY937718
E_FL_e100_02J05_F_0	BY937719
E_FL_e100_02J05_R_0	BY942519
E_FL_e100_02J08_F_0	BY937720
E_FL_e100_02J08_R_0	BY942520
E_FL_e100_02J09_F_0	BY937721
E_FL_e100_02J13_F_0	BY937722
E_FL_e100_02J13_R_0	BY942521
E_FL_e100_02J16_F_0	BY937723
E_FL_e100_02J17_F_0	BY937724
E_FL_e100_02J17_R_0	BY942522
E_FL_e100_02J18_F_0	BY937725
E_FL_e100_02J19_R_0	BY942523
E_FL_e100_02J20_F_0	BY937726
E_FL_e100_02J20_R_0	BY942524
E_FL_e100_02J23_F_0	BY937727
E_FL_e100_02J23_R_0	BY942525
E_FL_e100_02K01_R_0	BY942526
E_FL_e100_02K04_F_0	BY937728
E_FL_e100_02K05_F_0	BY937729
E_FL_e100_02K06_F_0	BY937730
E_FL_e100_02K06_R_0	BY942527
E_FL_e100_02K09_F_0	BY937731
E_FL_e100_02K10_F_0	BY937732
E_FL_e100_02K14_F_0	BY937733
E_FL_e100_02K16_F_0	BY937734
E_FL_e100_02K17_F_0	BY937735
E_FL_e100_02K18_F_0	BY937736
E_FL_e100_02K18_R_0	BY942528
E_FL_e100_02K20_F_0	BY937737
E_FL_e100_02K20_R_0	BY942529
E_FL_e100_02K21_F_0	BY937738
E_FL_e100_02L05_F_0	BY937739
E_FL_e100_02L05_R_0	BY942530
E_FL_e100_02L06_F_0	BY937740
E_FL_e100_02L06_R_0	BY942531
E_FL_e100_02L11_F_0	BY937741
E_FL_e100_02L17_F_0	BY937742
E_FL_e100_02L18_F_0	BY937743

E_FL_e100_02L18_R_0	BY942532
E_FL_e100_02M03_F_0	BY937744
E_FL_e100_02M03_R_0	BY942533
E_FL_e100_02M04_R_0	BY942534
E_FL_e100_02M05_F_0	BY937745
E_FL_e100_02M09_F_0	BY937746
E_FL_e100_02M11_F_0	BY937747
E_FL_e100_02M11_R_0	BY942535
E_FL_e100_02M12_F_0	BY937748
E_FL_e100_02M13_F_0	BY937749
E_FL_e100_02M16_F_0	BY937750
E_FL_e100_02M17_F_0	BY937751
E_FL_e100_02M17_R_0	BY942536
E_FL_e100_02M20_F_0	BY937752
E_FL_e100_02M20_R_0	BY942537
E_FL_e100_02M22_F_0	BY937753
E_FL_e100_02M24_F_0	BY937754
E_FL_e100_02N04_F_0	BY937755
E_FL_e100_02N05_F_0	BY937756
E_FL_e100_02N05_R_0	BY942538
E_FL_e100_02N06_F_0	BY937757
E_FL_e100_02N06_R_0	BY942539
E_FL_e100_02N11_F_0	BY937758
E_FL_e100_02N12_F_0	BY937759
E_FL_e100_02N14_F_0	BY937760
E_FL_e100_02N14_R_0	BY942540
E_FL_e100_02N17_F_0	BY937761
E_FL_e100_02N21_F_0	BY937762
E_FL_e100_02N21_R_0	BY942541
E_FL_e100_02N24_F_0	BY937763
E_FL_e100_02O01_F_0	BY937764
E_FL_e100_02O03_F_0	BY937765
E_FL_e100_02O03_R_0	BY942542
E_FL_e100_02O04_R_0	BY942543
E_FL_e100_02O05_F_0	BY937766
E_FL_e100_02O07_F_0	BY937767
E_FL_e100_02O07_R_0	BY942544
E_FL_e100_02O10_F_0	BY937768
E_FL_e100_02O10_R_0	BY942545
E_FL_e100_02O12_R_0	BY942546
E_FL_e100_02O14_R_0	BY942547
E_FL_e100_02O18_R_0	BY942548
E_FL_e100_02O19_F_0	BY937769
E_FL_e100_02O20_F_0	BY937770
E_FL_e100_02O20_R_0	BY942549
E_FL_e100_02O22_F_0	BY937771
E_FL_e100_02O23_F_0	BY937772
E_FL_e100_02O23_R_0	BY942550
E_FL_e100_02O24_F_0	BY937773
E_FL_e100_02O24_R_0	BY942551
E_FL_e100_02P02_F_0	BY937774
E_FL_e100_02P03_F_0	BY937775
E_FL_e100_02P04_F_0	BY937776
E_FL_e100_02P04_R_0	BY942552

E_FL_e100_02P07_F_0	BY937777
E_FL_e100_02P08_F_0	BY937778
E_FL_e100_02P08_R_0	BY942553
E_FL_e100_02P09_R_0	BY942554
E_FL_e100_02P10_F_0	BY937779
E_FL_e100_02P11_F_0	BY937780
E_FL_e100_02P11_R_0	BY942555
E_FL_e100_02P16_R_0	BY942556
E_FL_e100_02P17_F_0	BY937781
E_FL_e100_02P17_R_0	BY942557
E_FL_e100_02P18_F_0	BY937782
E_FL_e100_02P19_F_0	BY937783
E_FL_e100_02P19_R_0	BY942558
E_FL_e100_02P20_F_0	BY937784
E_FL_e100_02P20_R_0	BY942559
E_FL_e100_02P22_F_0	BY937785
E_FL_e100_02P24_F_0	BY937786
E_FL_e100_03A02_F_0	BY937787
E_FL_e100_03A07_F_0	BY937788
E_FL_e100_03A07_R_0	BY942560
E_FL_e100_03A11_F_0	BY937789
E_FL_e100_03A15_R_0	BY942561
E_FL_e100_03A16_F_0	BY937790
E_FL_e100_03A19_R_0	BY942562
E_FL_e100_03A20_R_0	BY942563
E_FL_e100_03A23_F_0	BY937791
E_FL_e100_03A24_F_0	BY937792
E_FL_e100_03B01_R_0	BY942564
E_FL_e100_03B02_F_0	BY937793
E_FL_e100_03B05_F_0	BY937794
E_FL_e100_03B05_R_0	BY942565
E_FL_e100_03B09_F_0	BY937795
E_FL_e100_03B09_R_0	BY942566
E_FL_e100_03B10_R_0	BY942567
E_FL_e100_03B12_F_0	BY937796
E_FL_e100_03B12_R_0	BY942568
E_FL_e100_03B15_F_0	BY937797
E_FL_e100_03B15_R_0	BY942569
E_FL_e100_03B19_F_0	BY937798
E_FL_e100_03B20_F_0	BY937799
E_FL_e100_03B22_R_0	BY942570
E_FL_e100_03B23_F_0	BY937800
E_FL_e100_03B23_R_0	BY942571
E_FL_e100_03C02_F_0	BY937801
E_FL_e100_03C05_F_0	BY937802
E_FL_e100_03C05_R_0	BY942572
E_FL_e100_03C06_F_0	BY937803
E_FL_e100_03C08_F_0	BY937804
E_FL_e100_03C08_R_0	BY942573
E_FL_e100_03C09_R_0	BY942574
E_FL_e100_03C10_F_0	BY937805
E_FL_e100_03C11_F_0	BY937806
E_FL_e100_03C11_R_0	BY942575
E_FL_e100_03C13_F_0	BY937807

E_FL_e100_03C13_R_0	BY942576
E_FL_e100_03C14_F_0	BY937808
E_FL_e100_03C16_R_0	BY942577
E_FL_e100_03C17_F_0	BY937809
E_FL_e100_03C17_R_0	BY942578
E_FL_e100_03C18_F_0	BY937810
E_FL_e100_03C22_R_0	BY942579
E_FL_e100_03C23_F_0	BY937811
E_FL_e100_03C23_R_0	BY942580
E_FL_e100_03D01_R_0	BY942581
E_FL_e100_03D03_F_0	BY937812
E_FL_e100_03D03_R_0	BY942582
E_FL_e100_03D04_F_0	BY937813
E_FL_e100_03D05_F_0	BY937814
E_FL_e100_03D05_R_0	BY942583
E_FL_e100_03D06_F_0	BY937815
E_FL_e100_03D12_F_0	BY937816
E_FL_e100_03D12_R_0	BY942584
E_FL_e100_03D13_F_0	BY937817
E_FL_e100_03D14_F_0	BY937818
E_FL_e100_03D15_F_0	BY937819
E_FL_e100_03D15_R_0	BY942585
E_FL_e100_03D17_F_0	BY937820
E_FL_e100_03D17_R_0	BY942586
E_FL_e100_03D18_F_0	BY937821
E_FL_e100_03D18_R_0	BY942587
E_FL_e100_03D19_F_0	BY937822
E_FL_e100_03D20_F_0	BY937823
E_FL_e100_03D20_R_0	BY942588
E_FL_e100_03D21_F_0	BY937824
E_FL_e100_03D21_R_0	BY942589
E_FL_e100_03D22_F_0	BY937825
E_FL_e100_03D22_R_0	BY942590
E_FL_e100_03E03_F_0	BY937826
E_FL_e100_03E06_F_0	BY937827
E_FL_e100_03E06_R_0	BY942591
E_FL_e100_03E08_F_0	BY937828
E_FL_e100_03E09_F_0	BY937829
E_FL_e100_03E09_R_0	BY942592
E_FL_e100_03E11_F_0	BY937830
E_FL_e100_03E11_R_0	BY942593
E_FL_e100_03E12_F_0	BY937831
E_FL_e100_03E12_R_0	BY942594
E_FL_e100_03E14_F_0	BY937832
E_FL_e100_03E15_F_0	BY937833
E_FL_e100_03E16_F_0	BY937834
E_FL_e100_03E16_R_0	BY942595
E_FL_e100_03E17_F_0	BY937835
E_FL_e100_03E17_R_0	BY942596
E_FL_e100_03E18_F_0	BY937836
E_FL_e100_03E18_R_0	BY942597
E_FL_e100_03E19_F_0	BY937837
E_FL_e100_03E21_F_0	BY937838
E_FL_e100_03E21_R_0	BY942598

E_FL_e100_03F03_F_0	BY937839
E_FL_e100_03F03_R_0	BY942599
E_FL_e100_03F06_F_0	BY937840
E_FL_e100_03F08_F_0	BY937841
E_FL_e100_03F08_R_0	BY942600
E_FL_e100_03F09_F_0	BY937842
E_FL_e100_03F10_F_0	BY937843
E_FL_e100_03F12_F_0	BY937844
E_FL_e100_03F14_F_0	BY937845
E_FL_e100_03F14_R_0	BY942601
E_FL_e100_03F16_F_0	BY937846
E_FL_e100_03F16_R_0	BY942602
E_FL_e100_03F17_F_0	BY937847
E_FL_e100_03F18_F_0	BY937848
E_FL_e100_03F18_R_0	BY942603
E_FL_e100_03F20_F_0	BY937849
E_FL_e100_03F21_F_0	BY937850
E_FL_e100_03F22_F_0	BY937851
E_FL_e100_03F23_F_0	BY937852
E_FL_e100_03F23_R_0	BY942604
E_FL_e100_03F24_F_0	BY937853
E_FL_e100_03F24_R_0	BY942605
E_FL_e100_03G01_F_0	BY937854
E_FL_e100_03G01_R_0	BY942606
E_FL_e100_03G02_F_0	BY937855
E_FL_e100_03G02_R_0	BY942607
E_FL_e100_03G03_F_0	BY937856
E_FL_e100_03G04_F_0	BY937857
E_FL_e100_03G04_R_0	BY942608
E_FL_e100_03G06_F_0	BY937858
E_FL_e100_03G07_F_0	BY937859
E_FL_e100_03G08_R_0	BY942609
E_FL_e100_03G10_R_0	BY942610
E_FL_e100_03G11_F_0	BY937860
E_FL_e100_03G14_F_0	BY937861
E_FL_e100_03G14_R_0	BY942611
E_FL_e100_03G15_F_0	BY937862
E_FL_e100_03G15_R_0	BY942612
E_FL_e100_03G16_F_0	BY937863
E_FL_e100_03G20_R_0	BY942613
E_FL_e100_03G21_F_0	BY937864
E_FL_e100_03G21_R_0	BY942614
E_FL_e100_03G22_F_0	BY937865
E_FL_e100_03G24_F_0	BY937866
E_FL_e100_03G24_R_0	BY942615
E_FL_e100_03H01_F_0	BY937867
E_FL_e100_03H02_F_0	BY937868
E_FL_e100_03H02_R_0	BY942616
E_FL_e100_03H03_F_0	BY937869
E_FL_e100_03H04_R_0	BY942617
E_FL_e100_03H05_R_0	BY942618
E_FL_e100_03H06_F_0	BY937870
E_FL_e100_03H06_R_0	BY942619
E_FL_e100_03H10_F_0	BY937871

E_FL_e100_03H10_R_0	BY942620
E_FL_e100_03H11_F_0	BY937872
E_FL_e100_03H11_R_0	BY942621
E_FL_e100_03H13_F_0	BY937873
E_FL_e100_03H13_R_0	BY942622
E_FL_e100_03H14_F_0	BY937874
E_FL_e100_03H14_R_0	BY942623
E_FL_e100_03H17_F_0	BY937875
E_FL_e100_03H17_R_0	BY942624
E_FL_e100_03H19_F_0	BY937876
E_FL_e100_03H19_R_0	BY942625
E_FL_e100_03H20_F_0	BY937877
E_FL_e100_03H20_R_0	BY942626
E_FL_e100_03H22_F_0	BY937878
E_FL_e100_03H24_F_0	BY937879
E_FL_e100_03I02_F_0	BY937880
E_FL_e100_03I03_F_0	BY937881
E_FL_e100_03I03_R_0	BY942627
E_FL_e100_03I04_F_0	BY937882
E_FL_e100_03I05_F_0	BY937883
E_FL_e100_03I05_R_0	BY942628
E_FL_e100_03I06_F_0	BY937884
E_FL_e100_03I06_R_0	BY942629
E_FL_e100_03I10_F_0	BY937885
E_FL_e100_03I10_R_0	BY942630
E_FL_e100_03I12_R_0	BY942631
E_FL_e100_03I13_R_0	BY942632
E_FL_e100_03I15_F_0	BY937886
E_FL_e100_03I16_F_0	BY937887
E_FL_e100_03I16_R_0	BY942633
E_FL_e100_03I17_F_0	BY937888
E_FL_e100_03I17_R_0	BY942634
E_FL_e100_03I18_F_0	BY937889
E_FL_e100_03I18_R_0	BY942635
E_FL_e100_03I21_F_0	BY937890
E_FL_e100_03I22_F_0	BY937891
E_FL_e100_03J02_F_0	BY937892
E_FL_e100_03J02_R_0	BY942636
E_FL_e100_03J05_F_0	BY937893
E_FL_e100_03J05_R_0	BY942637
E_FL_e100_03J06_F_0	BY937894
E_FL_e100_03J06_R_0	BY942638
E_FL_e100_03J08_F_0	BY937895
E_FL_e100_03J08_R_0	BY942639
E_FL_e100_03J09_F_0	BY937896
E_FL_e100_03J09_R_0	BY942640
E_FL_e100_03J10_F_0	BY937897
E_FL_e100_03J10_R_0	BY942641
E_FL_e100_03J11_F_0	BY937898
E_FL_e100_03J14_F_0	BY937899
E_FL_e100_03J14_R_0	BY942642
E_FL_e100_03J15_F_0	BY937900
E_FL_e100_03J20_F_0	BY937901
E_FL_e100_03J21_F_0	BY937902

E_FL_e100_03J21_R_0	BY942643
E_FL_e100_03J22_F_0	BY937903
E_FL_e100_03J22_R_0	BY942644
E_FL_e100_03J23_F_0	BY937904
E_FL_e100_03J23_R_0	BY942645
E_FL_e100_03J24_F_0	BY937905
E_FL_e100_03K01_F_0	BY937906
E_FL_e100_03K05_F_0	BY937907
E_FL_e100_03K05_R_0	BY942646
E_FL_e100_03K07_F_0	BY937908
E_FL_e100_03K07_R_0	BY942647
E_FL_e100_03K08_F_0	BY937909
E_FL_e100_03K11_F_0	BY937910
E_FL_e100_03K11_R_0	BY942648
E_FL_e100_03K14_F_0	BY937911
E_FL_e100_03K16_F_0	BY937912
E_FL_e100_03K17_F_0	BY937913
E_FL_e100_03K17_R_0	BY942649
E_FL_e100_03K20_R_0	BY942650
E_FL_e100_03K21_F_0	BY937914
E_FL_e100_03K21_R_0	BY942651
E_FL_e100_03K22_F_0	BY937915
E_FL_e100_03K22_R_0	BY942652
E_FL_e100_03L01_F_0	BY937916
E_FL_e100_03L01_R_0	BY942653
E_FL_e100_03L05_R_0	BY942654
E_FL_e100_03L06_F_0	BY937917
E_FL_e100_03L08_F_0	BY937918
E_FL_e100_03L10_F_0	BY937919
E_FL_e100_03L10_R_0	BY942655
E_FL_e100_03L12_F_0	BY937920
E_FL_e100_03L12_R_0	BY942656
E_FL_e100_03L13_R_0	BY942657
E_FL_e100_03L14_F_0	BY937921
E_FL_e100_03L15_F_0	BY937922
E_FL_e100_03L15_R_0	BY942658
E_FL_e100_03L16_F_0	BY937923
E_FL_e100_03L16_R_0	BY942659
E_FL_e100_03L17_F_0	BY937924
E_FL_e100_03L19_F_0	BY937925
E_FL_e100_03L20_F_0	BY937926
E_FL_e100_03L21_F_0	BY937927
E_FL_e100_03L21_R_0	BY942660
E_FL_e100_03L22_F_0	BY937928
E_FL_e100_03L22_R_0	BY942661
E_FL_e100_03M01_R_0	BY942662
E_FL_e100_03M09_F_0	BY937929
E_FL_e100_03M09_R_0	BY942663
E_FL_e100_03M10_R_0	BY942664
E_FL_e100_03M11_F_0	BY937930
E_FL_e100_03M11_R_0	BY942665
E_FL_e100_03M13_R_0	BY942666
E_FL_e100_03M15_F_0	BY937931
E_FL_e100_03M15_R_0	BY942667

E_FL_e100_03M16_R_0	BY942668
E_FL_e100_03M22_F_0	BY937932
E_FL_e100_03M23_F_0	BY937933
E_FL_e100_03M23_R_0	BY942669
E_FL_e100_03N02_F_0	BY937934
E_FL_e100_03N04_F_0	BY937935
E_FL_e100_03N05_R_0	BY942670
E_FL_e100_03N07_F_0	BY937936
E_FL_e100_03N08_F_0	BY937937
E_FL_e100_03N12_F_0	BY937938
E_FL_e100_03N15_F_0	BY937939
E_FL_e100_03N15_R_0	BY942671
E_FL_e100_03N17_F_0	BY937940
E_FL_e100_03N17_R_0	BY942672
E_FL_e100_03N18_F_0	BY937941
E_FL_e100_03N19_F_0	BY937942
E_FL_e100_03N19_R_0	BY942673
E_FL_e100_03N21_R_0	BY942674
E_FL_e100_03N23_F_0	BY937943
E_FL_e100_03N24_F_0	BY937944
E_FL_e100_03O01_F_0	BY937945
E_FL_e100_03O01_R_0	BY942675
E_FL_e100_03O04_F_0	BY937946
E_FL_e100_03O04_R_0	BY942676
E_FL_e100_03O06_F_0	BY937947
E_FL_e100_03O06_R_0	BY942677
E_FL_e100_03O08_R_0	BY942678
E_FL_e100_03O09_F_0	BY937948
E_FL_e100_03O10_F_0	BY937949
E_FL_e100_03O10_R_0	BY942679
E_FL_e100_03O12_F_0	BY937950
E_FL_e100_03O12_R_0	BY942680
E_FL_e100_03O13_R_0	BY942681
E_FL_e100_03O16_F_0	BY937951
E_FL_e100_03O19_F_0	BY937952
E_FL_e100_03O21_F_0	BY937953
E_FL_e100_03O23_F_0	BY937954
E_FL_e100_03O23_R_0	BY942682
E_FL_e100_03O24_F_0	BY937955
E_FL_e100_03O24_R_0	BY942683
E_FL_e100_03P02_F_0	BY937956
E_FL_e100_03P02_R_0	BY942684
E_FL_e100_03P03_F_0	BY937957
E_FL_e100_03P03_R_0	BY942685
E_FL_e100_03P04_F_0	BY937958
E_FL_e100_03P07_F_0	BY937959
E_FL_e100_03P07_R_0	BY942686
E_FL_e100_03P08_F_0	BY937960
E_FL_e100_03P08_R_0	BY942687
E_FL_e100_03P09_R_0	BY942688
E_FL_e100_03P10_F_0	BY937961
E_FL_e100_03P10_R_0	BY942689
E_FL_e100_03P12_F_0	BY937962
E_FL_e100_03P12_R_0	BY942690

E_FL_e100_03P13_F_0	BY937963
E_FL_e100_03P13_R_0	BY942691
E_FL_e100_03P14_F_0	BY937964
E_FL_e100_03P15_F_0	BY937965
E_FL_e100_03P16_F_0	BY937966
E_FL_e100_03P16_R_0	BY942692
E_FL_e100_03P17_F_0	BY937967
E_FL_e100_03P19_F_0	BY937968
E_FL_e100_03P19_R_0	BY942693
E_FL_e100_03P20_F_0	BY937969
E_FL_e100_03P21_F_0	BY937970
E_FL_e100_03P21_R_0	BY942694
E_FL_e100_03P23_F_0	BY937971
E_FL_e100_03P24_F_0	BY937972
E_FL_e100_03P24_R_0	BY942695
E_FL_e100_04A02_F_0	BY937973
E_FL_e100_04A03_F_0	BY937974
E_FL_e100_04A03_R_0	BY942696
E_FL_e100_04A04_F_0	BY937975
E_FL_e100_04A08_F_0	BY937976
E_FL_e100_04A09_R_0	BY942697
E_FL_e100_04A10_F_0	BY937977
E_FL_e100_04A11_F_0	BY937978
E_FL_e100_04A11_R_0	BY942698
E_FL_e100_04A12_F_0	BY937979
E_FL_e100_04A12_R_0	BY942699
E_FL_e100_04A14_F_0	BY937980
E_FL_e100_04A20_F_0	BY937981
E_FL_e100_04A20_R_0	BY942700
E_FL_e100_04A21_F_0	BY937982
E_FL_e100_04A21_R_0	BY942701
E_FL_e100_04A22_F_0	BY937983
E_FL_e100_04A22_R_0	BY942702
E_FL_e100_04A23_F_0	BY937984
E_FL_e100_04A23_R_0	BY942703
E_FL_e100_04B02_F_0	BY937985
E_FL_e100_04B03_R_0	BY942704
E_FL_e100_04B05_F_0	BY937986
E_FL_e100_04B05_R_0	BY942705
E_FL_e100_04B06_F_0	BY937987
E_FL_e100_04B06_R_0	BY942706
E_FL_e100_04B07_F_0	BY937988
E_FL_e100_04B08_F_0	BY937989
E_FL_e100_04B08_R_0	BY942707
E_FL_e100_04B10_R_0	BY942708
E_FL_e100_04B12_F_0	BY937990
E_FL_e100_04B12_R_0	BY942709
E_FL_e100_04B14_F_0	BY937991
E_FL_e100_04B15_F_0	BY937992
E_FL_e100_04B16_F_0	BY937993
E_FL_e100_04B16_R_0	BY942710
E_FL_e100_04B17_F_0	BY937994
E_FL_e100_04B17_R_0	BY942711
E_FL_e100_04B18_F_0	BY937995

E_FL_e100_04B18_R_0	BY942712
E_FL_e100_04B19_F_0	BY937996
E_FL_e100_04B19_R_0	BY942713
E_FL_e100_04B20_R_0	BY942714
E_FL_e100_04B21_F_0	BY937997
E_FL_e100_04B22_F_0	BY937998
E_FL_e100_04B22_R_0	BY942715
E_FL_e100_04B23_F_0	BY937999
E_FL_e100_04B23_R_0	BY942716
E_FL_e100_04B24_F_0	BY938000
E_FL_e100_04B24_R_0	BY942717
E_FL_e100_04C01_F_0	BY938001
E_FL_e100_04C02_F_0	BY938002
E_FL_e100_04C02_R_0	BY942718
E_FL_e100_04C05_F_0	BY938003
E_FL_e100_04C06_F_0	BY938004
E_FL_e100_04C08_F_0	BY938005
E_FL_e100_04C08_R_0	BY942719
E_FL_e100_04C09_F_0	BY938006
E_FL_e100_04C09_R_0	BY942720
E_FL_e100_04C12_F_0	BY938007
E_FL_e100_04C14_R_0	BY942721
E_FL_e100_04C16_F_0	BY938008
E_FL_e100_04C16_R_0	BY942722
E_FL_e100_04C18_F_0	BY938009
E_FL_e100_04C19_F_0	BY938010
E_FL_e100_04C20_F_0	BY938011
E_FL_e100_04C20_R_0	BY942723
E_FL_e100_04C21_F_0	BY938012
E_FL_e100_04C23_R_0	BY942724
E_FL_e100_04D02_R_0	BY942725
E_FL_e100_04D04_F_0	BY938013
E_FL_e100_04D04_R_0	BY942726
E_FL_e100_04D06_R_0	BY942727
E_FL_e100_04D08_F_0	BY938014
E_FL_e100_04D08_R_0	BY942728
E_FL_e100_04D09_F_0	BY938015
E_FL_e100_04D10_F_0	BY938016
E_FL_e100_04D11_F_0	BY938017
E_FL_e100_04D11_R_0	BY942729
E_FL_e100_04D12_R_0	BY942730
E_FL_e100_04D13_F_0	BY938018
E_FL_e100_04D14_F_0	BY938019
E_FL_e100_04D14_R_0	BY942731
E_FL_e100_04D15_F_0	BY938020
E_FL_e100_04D15_R_0	BY942732
E_FL_e100_04D17_F_0	BY938021
E_FL_e100_04D17_R_0	BY942733
E_FL_e100_04D18_F_0	BY938022
E_FL_e100_04D19_F_0	BY938023
E_FL_e100_04D19_R_0	BY942734
E_FL_e100_04D21_F_0	BY938024
E_FL_e100_04D22_F_0	BY938025
E_FL_e100_04D24_F_0	BY938026

E_FL_e100_04D24_R_0	BY942735
E_FL_e100_04E02_F_0	BY938027
E_FL_e100_04E04_F_0	BY938028
E_FL_e100_04E04_R_0	BY942736
E_FL_e100_04E05_F_0	BY938029
E_FL_e100_04E07_F_0	BY938030
E_FL_e100_04E07_R_0	BY942737
E_FL_e100_04E09_F_0	BY938031
E_FL_e100_04E10_F_0	BY938032
E_FL_e100_04E11_F_0	BY938033
E_FL_e100_04E11_R_0	BY942738
E_FL_e100_04E12_R_0	BY942739
E_FL_e100_04E15_F_0	BY938034
E_FL_e100_04E15_R_0	BY942740
E_FL_e100_04E16_F_0	BY938035
E_FL_e100_04E16_R_0	BY942741
E_FL_e100_04E18_R_0	BY942742
E_FL_e100_04E21_F_0	BY938036
E_FL_e100_04E23_F_0	BY938037
E_FL_e100_04F03_R_0	BY942743
E_FL_e100_04F04_F_0	BY938038
E_FL_e100_04F04_R_0	BY942744
E_FL_e100_04F05_R_0	BY942745
E_FL_e100_04F06_F_0	BY938039
E_FL_e100_04F10_F_0	BY938040
E_FL_e100_04F11_F_0	BY938041
E_FL_e100_04F12_F_0	BY938042
E_FL_e100_04F14_F_0	BY938043
E_FL_e100_04F17_F_0	BY938044
E_FL_e100_04F20_F_0	BY938045
E_FL_e100_04F21_F_0	BY938046
E_FL_e100_04F23_F_0	BY938047
E_FL_e100_04G03_F_0	BY938048
E_FL_e100_04G04_F_0	BY938049
E_FL_e100_04G04_R_0	BY942746
E_FL_e100_04G05_F_0	BY938050
E_FL_e100_04G06_F_0	BY938051
E_FL_e100_04G07_F_0	BY938052
E_FL_e100_04G08_F_0	BY938053
E_FL_e100_04G08_R_0	BY942747
E_FL_e100_04G09_F_0	BY938054
E_FL_e100_04G10_F_0	BY938055
E_FL_e100_04G10_R_0	BY942748
E_FL_e100_04G12_F_0	BY938056
E_FL_e100_04G15_F_0	BY938057
E_FL_e100_04G16_F_0	BY938058
E_FL_e100_04G16_R_0	BY942749
E_FL_e100_04G18_F_0	BY938059
E_FL_e100_04G20_F_0	BY938060
E_FL_e100_04G21_F_0	BY938061
E_FL_e100_04G22_F_0	BY938062
E_FL_e100_04G23_F_0	BY938063
E_FL_e100_04G24_F_0	BY938064
E_FL_e100_04G24_R_0	BY942750

E_FL_e100_04H03_F_0	BY938065
E_FL_e100_04H04_F_0	BY938066
E_FL_e100_04H08_F_0	BY938067
E_FL_e100_04H08_R_0	BY942751
E_FL_e100_04H09_F_0	BY938068
E_FL_e100_04H10_F_0	BY938069
E_FL_e100_04H12_F_0	BY938070
E_FL_e100_04H13_F_0	BY938071
E_FL_e100_04H13_R_0	BY942752
E_FL_e100_04H14_F_0	BY938072
E_FL_e100_04H14_R_0	BY942753
E_FL_e100_04H15_F_0	BY938073
E_FL_e100_04H17_F_0	BY938074
E_FL_e100_04H17_R_0	BY942754
E_FL_e100_04H18_F_0	BY938075
E_FL_e100_04H18_R_0	BY942755
E_FL_e100_04H19_F_0	BY938076
E_FL_e100_04H20_F_0	BY938077
E_FL_e100_04H22_F_0	BY938078
E_FL_e100_04H22_R_0	BY942756
E_FL_e100_04H23_F_0	BY938079
E_FL_e100_04H23_R_0	BY942757
E_FL_e100_04H24_F_0	BY938080
E_FL_e100_04H24_R_0	BY942758
E_FL_e100_04I01_F_0	BY938081
E_FL_e100_04I03_R_0	BY942759
E_FL_e100_04I04_F_0	BY938082
E_FL_e100_04I05_R_0	BY942760
E_FL_e100_04I06_F_0	BY938083
E_FL_e100_04I06_R_0	BY942761
E_FL_e100_04I08_R_0	BY942762
E_FL_e100_04I09_F_0	BY938084
E_FL_e100_04I09_R_0	BY942763
E_FL_e100_04I10_F_0	BY938085
E_FL_e100_04I10_R_0	BY942764
E_FL_e100_04I11_F_0	BY938086
E_FL_e100_04I11_R_0	BY942765
E_FL_e100_04I13_F_0	BY938087
E_FL_e100_04I13_R_0	BY942766
E_FL_e100_04I16_F_0	BY938088
E_FL_e100_04I16_R_0	BY942767
E_FL_e100_04I17_F_0	BY938089
E_FL_e100_04I17_R_0	BY942768
E_FL_e100_04I19_F_0	BY938090
E_FL_e100_04I19_R_0	BY942769
E_FL_e100_04I20_R_0	BY942770
E_FL_e100_04I21_F_0	BY938091
E_FL_e100_04I21_R_0	BY942771
E_FL_e100_04I22_F_0	BY938092
E_FL_e100_04I23_F_0	BY938093
E_FL_e100_04I23_R_0	BY942772
E_FL_e100_04J02_F_0	BY938094
E_FL_e100_04J03_F_0	BY938095
E_FL_e100_04J03_R_0	BY942773

E_FL_e100_04J04_F_0	BY938096
E_FL_e100_04J04_R_0	BY942774
E_FL_e100_04J05_F_0	BY938097
E_FL_e100_04J05_R_0	BY942775
E_FL_e100_04J06_F_0	BY938098
E_FL_e100_04J07_F_0	BY938099
E_FL_e100_04J10_F_0	BY938100
E_FL_e100_04J11_F_0	BY938101
E_FL_e100_04J12_R_0	BY942776
E_FL_e100_04J13_F_0	BY938102
E_FL_e100_04J14_F_0	BY938103
E_FL_e100_04J15_F_0	BY938104
E_FL_e100_04J15_R_0	BY942777
E_FL_e100_04J18_F_0	BY938105
E_FL_e100_04J18_R_0	BY942778
E_FL_e100_04J19_F_0	BY938106
E_FL_e100_04J21_F_0	BY938107
E_FL_e100_04J23_F_0	BY938108
E_FL_e100_04J23_R_0	BY942779
E_FL_e100_04J24_F_0	BY938109
E_FL_e100_04K02_F_0	BY938110
E_FL_e100_04K03_F_0	BY938111
E_FL_e100_04K03_R_0	BY942780
E_FL_e100_04K05_F_0	BY938112
E_FL_e100_04K10_F_0	BY938113
E_FL_e100_04K11_F_0	BY938114
E_FL_e100_04K11_R_0	BY942781
E_FL_e100_04K12_R_0	BY942782
E_FL_e100_04K13_R_0	BY942783
E_FL_e100_04K15_F_0	BY938115
E_FL_e100_04K16_F_0	BY938116
E_FL_e100_04K17_F_0	BY938117
E_FL_e100_04K17_R_0	BY942784
E_FL_e100_04K20_F_0	BY938118
E_FL_e100_04K20_R_0	BY942785
E_FL_e100_04K21_F_0	BY938119
E_FL_e100_04K21_R_0	BY942786
E_FL_e100_04K24_F_0	BY938120
E_FL_e100_04L01_F_0	BY938121
E_FL_e100_04L01_R_0	BY942787
E_FL_e100_04L02_F_0	BY938122
E_FL_e100_04L02_R_0	BY942788
E_FL_e100_04L03_F_0	BY938123
E_FL_e100_04L03_R_0	BY942789
E_FL_e100_04L04_F_0	BY938124
E_FL_e100_04L04_R_0	BY942790
E_FL_e100_04L05_F_0	BY938125
E_FL_e100_04L05_R_0	BY942791
E_FL_e100_04L06_F_0	BY938126
E_FL_e100_04L07_F_0	BY938127
E_FL_e100_04L07_R_0	BY942792
E_FL_e100_04L08_R_0	BY942793
E_FL_e100_04L09_F_0	BY938128
E_FL_e100_04L09_R_0	BY942794

E_FL_e100_04L10_F_0	BY938129
E_FL_e100_04L11_F_0	BY938130
E_FL_e100_04L13_F_0	BY938131
E_FL_e100_04L17_F_0	BY938132
E_FL_e100_04L18_F_0	BY938133
E_FL_e100_04L18_R_0	BY942795
E_FL_e100_04L24_F_0	BY938134
E_FL_e100_04M01_F_0	BY938135
E_FL_e100_04M01_R_0	BY942796
E_FL_e100_04M04_F_0	BY938136
E_FL_e100_04M04_R_0	BY942797
E_FL_e100_04M09_F_0	BY938137
E_FL_e100_04M10_F_0	BY938138
E_FL_e100_04M13_R_0	BY942798
E_FL_e100_04M14_F_0	BY938139
E_FL_e100_04M14_R_0	BY942799
E_FL_e100_04M15_F_0	BY938140
E_FL_e100_04M15_R_0	BY942800
E_FL_e100_04M16_F_0	BY938141
E_FL_e100_04M19_F_0	BY938142
E_FL_e100_04M21_F_0	BY938143
E_FL_e100_04M21_R_0	BY942801
E_FL_e100_04M22_F_0	BY938144
E_FL_e100_04M24_F_0	BY938145
E_FL_e100_04N03_F_0	BY938146
E_FL_e100_04N05_F_0	BY938147
E_FL_e100_04N06_F_0	BY938148
E_FL_e100_04N06_R_0	BY942802
E_FL_e100_04N07_R_0	BY942803
E_FL_e100_04N09_F_0	BY938149
E_FL_e100_04N09_R_0	BY942804
E_FL_e100_04N10_R_0	BY942805
E_FL_e100_04N13_F_0	BY938150
E_FL_e100_04N13_R_0	BY942806
E_FL_e100_04N14_F_0	BY938151
E_FL_e100_04N15_F_0	BY938152
E_FL_e100_04N15_R_0	BY942807
E_FL_e100_04N17_F_0	BY938153
E_FL_e100_04N19_F_0	BY938154
E_FL_e100_04N19_R_0	BY942808
E_FL_e100_04N20_F_0	BY938155
E_FL_e100_04N23_R_0	BY942809
E_FL_e100_04O01_F_0	BY938156
E_FL_e100_04O06_F_0	BY938157
E_FL_e100_04O06_R_0	BY942810
E_FL_e100_04O07_F_0	BY938158
E_FL_e100_04O07_R_0	BY942811
E_FL_e100_04O08_F_0	BY938159
E_FL_e100_04O09_R_0	BY942812
E_FL_e100_04O10_F_0	BY938160
E_FL_e100_04O11_F_0	BY938161
E_FL_e100_04O11_R_0	BY942813
E_FL_e100_04O13_F_0	BY938162
E_FL_e100_04O14_F_0	BY938163

E_FL_e100_04O14_R_0	BY942814
E_FL_e100_04O15_F_0	BY938164
E_FL_e100_04O15_R_0	BY942815
E_FL_e100_04O17_R_0	BY942816
E_FL_e100_04O18_R_0	BY942817
E_FL_e100_04O19_F_0	BY938165
E_FL_e100_04O19_R_0	BY942818
E_FL_e100_04O20_F_0	BY938166
E_FL_e100_04O23_F_0	BY938167
E_FL_e100_04P01_F_0	BY938168
E_FL_e100_04P01_R_0	BY942819
E_FL_e100_04P02_F_0	BY938169
E_FL_e100_04P02_R_0	BY942820
E_FL_e100_04P04_F_0	BY938170
E_FL_e100_04P05_F_0	BY938171
E_FL_e100_04P06_F_0	BY938172
E_FL_e100_04P06_R_0	BY942821
E_FL_e100_04P07_F_0	BY938173
E_FL_e100_04P07_R_0	BY942822
E_FL_e100_04P11_R_0	BY942823
E_FL_e100_04P14_F_0	BY938174
E_FL_e100_04P15_R_0	BY942824
E_FL_e100_04P16_R_0	BY942825
E_FL_e100_04P18_F_0	BY938175
E_FL_e100_04P18_R_0	BY942826
E_FL_e100_04P19_F_0	BY938176
E_FL_e100_04P20_F_0	BY938177
E_FL_e100_04P20_R_0	BY942827
E_FL_e100_04P21_F_0	BY938178
E_FL_e100_04P21_R_0	BY942828
E_FL_e100_04P22_F_0	BY938179
E_FL_e100_04P22_R_0	BY942829
E_FL_e100_04P23_F_0	BY938180
E_FL_e100_04P23_R_0	BY942830
E_FL_e100_04P24_F_0	BY938181
E_FL_e100_05A04_F_0	BY938182
E_FL_e100_05A06_F_0	BY938183
E_FL_e100_05A08_F_0	BY938184
E_FL_e100_05A08_R_0	BY942831
E_FL_e100_05A10_F_0	BY938185
E_FL_e100_05A10_R_0	BY942832
E_FL_e100_05A11_R_0	BY942833
E_FL_e100_05A12_F_0	BY938186
E_FL_e100_05A13_F_0	BY938187
E_FL_e100_05A13_R_0	BY942834
E_FL_e100_05A15_F_0	BY938188
E_FL_e100_05A15_R_0	BY942835
E_FL_e100_05A16_F_0	BY938189
E_FL_e100_05A16_R_0	BY942836
E_FL_e100_05A19_R_0	BY942837
E_FL_e100_05A20_F_0	BY938190
E_FL_e100_05A21_F_0	BY938191
E_FL_e100_05A23_F_0	BY938192
E_FL_e100_05A23_R_0	BY942838

E_FL_e100_05A24_F_0	BY938193
E_FL_e100_05A24_R_0	BY942839
E_FL_e100_05B01_F_0	BY938194
E_FL_e100_05B03_F_0	BY938195
E_FL_e100_05B03_R_0	BY942840
E_FL_e100_05B05_F_0	BY938196
E_FL_e100_05B05_R_0	BY942841
E_FL_e100_05B08_F_0	BY938197
E_FL_e100_05B09_F_0	BY938198
E_FL_e100_05B10_F_0	BY938199
E_FL_e100_05B11_F_0	BY938200
E_FL_e100_05B11_R_0	BY942842
E_FL_e100_05B12_F_0	BY938201
E_FL_e100_05B12_R_0	BY942843
E_FL_e100_05B14_F_0	BY938202
E_FL_e100_05B16_F_0	BY938203
E_FL_e100_05B19_F_0	BY938204
E_FL_e100_05B19_R_0	BY942844
E_FL_e100_05B20_F_0	BY938205
E_FL_e100_05B21_F_0	BY938206
E_FL_e100_05B21_R_0	BY942845
E_FL_e100_05B22_F_0	BY938207
E_FL_e100_05B22_R_0	BY942846
E_FL_e100_05B23_F_0	BY938208
E_FL_e100_05B23_R_0	BY942847
E_FL_e100_05B24_F_0	BY938209
E_FL_e100_05B24_R_0	BY942848
E_FL_e100_05C03_F_0	BY938210
E_FL_e100_05C03_R_0	BY942849
E_FL_e100_05C04_F_0	BY938211
E_FL_e100_05C04_R_0	BY942850
E_FL_e100_05C05_R_0	BY942851
E_FL_e100_05C07_F_0	BY938212
E_FL_e100_05C07_R_0	BY942852
E_FL_e100_05C09_F_0	BY938213
E_FL_e100_05C09_R_0	BY942853
E_FL_e100_05C10_F_0	BY938214
E_FL_e100_05C11_F_0	BY938215
E_FL_e100_05C13_F_0	BY938216
E_FL_e100_05C13_R_0	BY942854
E_FL_e100_05C19_F_0	BY938217
E_FL_e100_05C19_R_0	BY942855
E_FL_e100_05C20_F_0	BY938218
E_FL_e100_05C21_F_0	BY938219
E_FL_e100_05C22_F_0	BY938220
E_FL_e100_05C22_R_0	BY942856
E_FL_e100_05C23_F_0	BY938221
E_FL_e100_05C23_R_0	BY942857
E_FL_e100_05C24_R_0	BY942858
E_FL_e100_05D01_F_0	BY938222
E_FL_e100_05D01_R_0	BY942859
E_FL_e100_05D02_F_0	BY938223
E_FL_e100_05D07_F_0	BY938224
E_FL_e100_05D07_R_0	BY942860

E_FL_e100_05D10_F_0	BY938225
E_FL_e100_05D11_F_0	BY938226
E_FL_e100_05D11_R_0	BY942861
E_FL_e100_05D13_F_0	BY938227
E_FL_e100_05D13_R_0	BY942862
E_FL_e100_05D14_F_0	BY938228
E_FL_e100_05D16_F_0	BY938229
E_FL_e100_05D18_F_0	BY938230
E_FL_e100_05D18_R_0	BY942863
E_FL_e100_05D19_F_0	BY938231
E_FL_e100_05D21_F_0	BY938232
E_FL_e100_05D22_F_0	BY938233
E_FL_e100_05D22_R_0	BY942864
E_FL_e100_05D23_F_0	BY938234
E_FL_e100_05D23_R_0	BY942865
E_FL_e100_05D24_R_0	BY942866
E_FL_e100_05E02_F_0	BY938235
E_FL_e100_05E04_F_0	BY938236
E_FL_e100_05E04_R_0	BY942867
E_FL_e100_05E08_F_0	BY938237
E_FL_e100_05E08_R_0	BY942868
E_FL_e100_05E09_F_0	BY938238
E_FL_e100_05E09_R_0	BY942869
E_FL_e100_05E10_F_0	BY938239
E_FL_e100_05E10_R_0	BY942870
E_FL_e100_05E12_F_0	BY938240
E_FL_e100_05E13_F_0	BY938241
E_FL_e100_05E13_R_0	BY942871
E_FL_e100_05E14_F_0	BY938242
E_FL_e100_05E14_R_0	BY942872
E_FL_e100_05E17_R_0	BY942873
E_FL_e100_05E18_F_0	BY938243
E_FL_e100_05E18_R_0	BY942874
E_FL_e100_05E20_F_0	BY938244
E_FL_e100_05E20_R_0	BY942875
E_FL_e100_05E21_F_0	BY938245
E_FL_e100_05E21_R_0	BY942876
E_FL_e100_05F01_F_0	BY938246
E_FL_e100_05F01_R_0	BY942877
E_FL_e100_05F02_F_0	BY938247
E_FL_e100_05F02_R_0	BY942878
E_FL_e100_05F03_F_0	BY938248
E_FL_e100_05F04_R_0	BY942879
E_FL_e100_05F07_F_0	BY938249
E_FL_e100_05F09_F_0	BY938250
E_FL_e100_05F10_F_0	BY938251
E_FL_e100_05F10_R_0	BY942880
E_FL_e100_05F11_F_0	BY938252
E_FL_e100_05F11_R_0	BY942881
E_FL_e100_05F12_F_0	BY938253
E_FL_e100_05F13_F_0	BY938254
E_FL_e100_05F18_F_0	BY938255
E_FL_e100_05F18_R_0	BY942882
E_FL_e100_05F19_F_0	BY938256

E_FL_e100_05F19_R_0	BY942883
E_FL_e100_05F20_F_0	BY938257
E_FL_e100_05F20_R_0	BY942884
E_FL_e100_05F22_F_0	BY938258
E_FL_e100_05F22_R_0	BY942885
E_FL_e100_05F23_F_0	BY938259
E_FL_e100_05F24_R_0	BY942886
E_FL_e100_05G01_F_0	BY938260
E_FL_e100_05G03_F_0	BY938261
E_FL_e100_05G04_F_0	BY938262
E_FL_e100_05G04_R_0	BY942887
E_FL_e100_05G05_F_0	BY938263
E_FL_e100_05G08_F_0	BY938264
E_FL_e100_05G08_R_0	BY942888
E_FL_e100_05G09_F_0	BY938265
E_FL_e100_05G11_F_0	BY938266
E_FL_e100_05G13_F_0	BY938267
E_FL_e100_05G17_F_0	BY938268
E_FL_e100_05G17_R_0	BY942889
E_FL_e100_05G19_F_0	BY938269
E_FL_e100_05G20_F_0	BY938270
E_FL_e100_05G21_F_0	BY938271
E_FL_e100_05G22_F_0	BY938272
E_FL_e100_05H01_F_0	BY938273
E_FL_e100_05H01_R_0	BY942890
E_FL_e100_05H02_F_0	BY938274
E_FL_e100_05H02_R_0	BY942891
E_FL_e100_05H03_F_0	BY938275
E_FL_e100_05H06_F_0	BY938276
E_FL_e100_05H06_R_0	BY942892
E_FL_e100_05H07_F_0	BY938277
E_FL_e100_05H07_R_0	BY942893
E_FL_e100_05H08_F_0	BY938278
E_FL_e100_05H08_R_0	BY942894
E_FL_e100_05H09_F_0	BY938279
E_FL_e100_05H10_F_0	BY938280
E_FL_e100_05H11_F_0	BY938281
E_FL_e100_05H12_F_0	BY938282
E_FL_e100_05H12_R_0	BY942895
E_FL_e100_05H14_F_0	BY938283
E_FL_e100_05H14_R_0	BY942896
E_FL_e100_05H15_F_0	BY938284
E_FL_e100_05H16_F_0	BY938285
E_FL_e100_05H16_R_0	BY942897
E_FL_e100_05H17_F_0	BY938286
E_FL_e100_05H17_R_0	BY942898
E_FL_e100_05H24_F_0	BY938287
E_FL_e100_05I01_F_0	BY938288
E_FL_e100_05I01_R_0	BY942899
E_FL_e100_05I03_F_0	BY938289
E_FL_e100_05I03_R_0	BY942900
E_FL_e100_05I04_F_0	BY938290
E_FL_e100_05I04_R_0	BY942901
E_FL_e100_05I06_F_0	BY938291

E_FL_e100_05I09_F_0	BY938292
E_FL_e100_05I09_R_0	BY942902
E_FL_e100_05I10_F_0	BY938293
E_FL_e100_05I13_F_0	BY938294
E_FL_e100_05I13_R_0	BY942903
E_FL_e100_05I14_F_0	BY938295
E_FL_e100_05I15_F_0	BY938296
E_FL_e100_05I17_F_0	BY938297
E_FL_e100_05I18_F_0	BY938298
E_FL_e100_05I21_F_0	BY938299
E_FL_e100_05I22_F_0	BY938300
E_FL_e100_05I23_F_0	BY938301
E_FL_e100_05I23_R_0	BY942904
E_FL_e100_05I24_F_0	BY938302
E_FL_e100_05J01_F_0	BY938303
E_FL_e100_05J01_R_0	BY942905
E_FL_e100_05J05_F_0	BY938304
E_FL_e100_05J05_R_0	BY942906
E_FL_e100_05J06_F_0	BY938305
E_FL_e100_05J07_F_0	BY938306
E_FL_e100_05J07_R_0	BY942907
E_FL_e100_05J08_F_0	BY938307
E_FL_e100_05J08_R_0	BY942908
E_FL_e100_05J09_F_0	BY938308
E_FL_e100_05J11_F_0	BY938309
E_FL_e100_05J13_F_0	BY938310
E_FL_e100_05J13_R_0	BY942909
E_FL_e100_05J15_F_0	BY938311
E_FL_e100_05J15_R_0	BY942910
E_FL_e100_05J16_F_0	BY938312
E_FL_e100_05J17_F_0	BY938313
E_FL_e100_05J19_R_0	BY942911
E_FL_e100_05J20_F_0	BY938314
E_FL_e100_05J20_R_0	BY942912
E_FL_e100_05J21_F_0	BY938315
E_FL_e100_05J21_R_0	BY942913
E_FL_e100_05K02_F_0	BY938316
E_FL_e100_05K03_F_0	BY938317
E_FL_e100_05K04_F_0	BY938318
E_FL_e100_05K04_R_0	BY942914
E_FL_e100_05K06_F_0	BY938319
E_FL_e100_05K07_R_0	BY942915
E_FL_e100_05K11_F_0	BY938320
E_FL_e100_05K11_R_0	BY942916
E_FL_e100_05K12_F_0	BY938321
E_FL_e100_05K16_F_0	BY938322
E_FL_e100_05K17_F_0	BY938323
E_FL_e100_05K20_F_0	BY938324
E_FL_e100_05K21_F_0	BY938325
E_FL_e100_05K21_R_0	BY942917
E_FL_e100_05K24_F_0	BY938326
E_FL_e100_05K24_R_0	BY942918
E_FL_e100_05L01_F_0	BY938327
E_FL_e100_05L04_F_0	BY938328

E_FL_e100_05L04_R_0	BY942919
E_FL_e100_05L08_F_0	BY938329
E_FL_e100_05L09_F_0	BY938330
E_FL_e100_05L10_F_0	BY938331
E_FL_e100_05L10_R_0	BY942920
E_FL_e100_05L11_F_0	BY938332
E_FL_e100_05L12_F_0	BY938333
E_FL_e100_05L12_R_0	BY942921
E_FL_e100_05L13_F_0	BY938334
E_FL_e100_05L16_F_0	BY938335
E_FL_e100_05L18_R_0	BY942922
E_FL_e100_05L20_F_0	BY938336
E_FL_e100_05L21_F_0	BY938337
E_FL_e100_05L21_R_0	BY942923
E_FL_e100_05L22_R_0	BY942924
E_FL_e100_05L24_F_0	BY938338
E_FL_e100_05L24_R_0	BY942925
E_FL_e100_05M01_F_0	BY938339
E_FL_e100_05M02_F_0	BY938340
E_FL_e100_05M04_F_0	BY938341
E_FL_e100_05M04_R_0	BY942926
E_FL_e100_05M06_F_0	BY938342
E_FL_e100_05M08_F_0	BY938343
E_FL_e100_05M08_R_0	BY942927
E_FL_e100_05M11_F_0	BY938344
E_FL_e100_05M11_R_0	BY942928
E_FL_e100_05M15_F_0	BY938345
E_FL_e100_05M15_R_0	BY942929
E_FL_e100_05M16_F_0	BY938346
E_FL_e100_05M16_R_0	BY942930
E_FL_e100_05M17_R_0	BY942931
E_FL_e100_05M19_F_0	BY938347
E_FL_e100_05M22_F_0	BY938348
E_FL_e100_05M22_R_0	BY942932
E_FL_e100_05M24_F_0	BY938349
E_FL_e100_05N02_F_0	BY938350
E_FL_e100_05N02_R_0	BY942933
E_FL_e100_05N03_F_0	BY938351
E_FL_e100_05N03_R_0	BY942934
E_FL_e100_05N07_F_0	BY938352
E_FL_e100_05N07_R_0	BY942935
E_FL_e100_05N09_F_0	BY938353
E_FL_e100_05N09_R_0	BY942936
E_FL_e100_05N10_F_0	BY938354
E_FL_e100_05N10_R_0	BY942937
E_FL_e100_05N11_F_0	BY938355
E_FL_e100_05N12_F_0	BY938356
E_FL_e100_05N12_R_0	BY942938
E_FL_e100_05N13_R_0	BY942939
E_FL_e100_05N19_F_0	BY938357
E_FL_e100_05N19_R_0	BY942940
E_FL_e100_05N20_F_0	BY938358
E_FL_e100_05N21_F_0	BY938359
E_FL_e100_05N21_R_0	BY942941

E_FL_e100_05N22_F_0	BY938360
E_FL_e100_05N24_F_0	BY938361
E_FL_e100_05O01_F_0	BY938362
E_FL_e100_05O02_F_0	BY938363
E_FL_e100_05O04_F_0	BY938364
E_FL_e100_05O04_R_0	BY942942
E_FL_e100_05O05_F_0	BY938365
E_FL_e100_05O05_R_0	BY942943
E_FL_e100_05O07_F_0	BY938366
E_FL_e100_05O07_R_0	BY942944
E_FL_e100_05O09_F_0	BY938367
E_FL_e100_05O11_F_0	BY938368
E_FL_e100_05O12_F_0	BY938369
E_FL_e100_05O12_R_0	BY942945
E_FL_e100_05O15_F_0	BY938370
E_FL_e100_05O16_F_0	BY938371
E_FL_e100_05O16_R_0	BY942946
E_FL_e100_05O17_F_0	BY938372
E_FL_e100_05O22_F_0	BY938373
E_FL_e100_05O24_F_0	BY938374
E_FL_e100_05O24_R_0	BY942947
E_FL_e100_05P04_F_0	BY938375
E_FL_e100_05P04_R_0	BY942948
E_FL_e100_05P06_F_0	BY938376
E_FL_e100_05P06_R_0	BY942949
E_FL_e100_05P08_F_0	BY938377
E_FL_e100_05P08_R_0	BY942950
E_FL_e100_05P10_F_0	BY938378
E_FL_e100_05P10_R_0	BY942951
E_FL_e100_05P12_F_0	BY938379
E_FL_e100_05P13_F_0	BY938380
E_FL_e100_05P14_F_0	BY938381
E_FL_e100_05P14_R_0	BY942952
E_FL_e100_05P15_F_0	BY938382
E_FL_e100_05P15_R_0	BY942953
E_FL_e100_05P16_F_0	BY938383
E_FL_e100_05P17_R_0	BY942954
E_FL_e100_05P18_F_0	BY938384
E_FL_e100_05P19_F_0	BY938385
E_FL_e100_05P19_R_0	BY942955
E_FL_e100_05P22_R_0	BY942956
E_FL_e100_05P23_F_0	BY938386
E_FL_e100_05P23_R_0	BY942957
E_FL_e100_06A01_R_0	BY942958
E_FL_e100_06A03_F_0	BY938387
E_FL_e100_06A03_R_0	BY942959
E_FL_e100_06A05_F_0	BY938388
E_FL_e100_06A10_F_0	BY938389
E_FL_e100_06A11_F_0	BY938390
E_FL_e100_06A11_R_0	BY942960
E_FL_e100_06A12_R_0	BY942961
E_FL_e100_06A13_F_0	BY938391
E_FL_e100_06A14_F_0	BY938392
E_FL_e100_06A15_F_0	BY938393

E_FL_e100_06A16_F_0	BY938394
E_FL_e100_06A16_R_0	BY942962
E_FL_e100_06A17_F_0	BY938395
E_FL_e100_06A18_F_0	BY938396
E_FL_e100_06A18_R_0	BY942963
E_FL_e100_06A19_F_0	BY938397
E_FL_e100_06A19_R_0	BY942964
E_FL_e100_06A20_F_0	BY938398
E_FL_e100_06A21_F_0	BY938399
E_FL_e100_06A21_R_0	BY942965
E_FL_e100_06A22_F_0	BY938400
E_FL_e100_06A22_R_0	BY942966
E_FL_e100_06A23_F_0	BY938401
E_FL_e100_06A23_R_0	BY942967
E_FL_e100_06A24_F_0	BY938402
E_FL_e100_06A24_R_0	BY942968
E_FL_e100_06B02_R_0	BY942969
E_FL_e100_06B03_F_0	BY938403
E_FL_e100_06B03_R_0	BY942970
E_FL_e100_06B05_F_0	BY938404
E_FL_e100_06B07_F_0	BY938405
E_FL_e100_06B07_R_0	BY942971
E_FL_e100_06B10_R_0	BY942972
E_FL_e100_06B12_F_0	BY938406
E_FL_e100_06B14_F_0	BY938407
E_FL_e100_06B14_R_0	BY942973
E_FL_e100_06B15_F_0	BY938408
E_FL_e100_06B16_F_0	BY938409
E_FL_e100_06B16_R_0	BY942974
E_FL_e100_06B18_F_0	BY938410
E_FL_e100_06B19_F_0	BY938411
E_FL_e100_06B20_F_0	BY938412
E_FL_e100_06B21_F_0	BY938413
E_FL_e100_06B21_R_0	BY942975
E_FL_e100_06B22_F_0	BY938414
E_FL_e100_06C01_F_0	BY938415
E_FL_e100_06C01_R_0	BY942976
E_FL_e100_06C02_F_0	BY938416
E_FL_e100_06C03_F_0	BY938417
E_FL_e100_06C08_F_0	BY938418
E_FL_e100_06C09_F_0	BY938419
E_FL_e100_06C09_R_0	BY942977
E_FL_e100_06C10_R_0	BY942978
E_FL_e100_06C11_F_0	BY938420
E_FL_e100_06C13_F_0	BY938421
E_FL_e100_06C13_R_0	BY942979
E_FL_e100_06C14_F_0	BY938422
E_FL_e100_06C14_R_0	BY942980
E_FL_e100_06C15_F_0	BY938423
E_FL_e100_06C19_F_0	BY938424
E_FL_e100_06C20_F_0	BY938425
E_FL_e100_06C22_F_0	BY938426
E_FL_e100_06D02_F_0	BY938427
E_FL_e100_06D02_R_0	BY942981

E_FL_e100_06D03_F_0	BY938428
E_FL_e100_06D03_R_0	BY942982
E_FL_e100_06D04_F_0	BY938429
E_FL_e100_06D05_F_0	BY938430
E_FL_e100_06D05_R_0	BY942983
E_FL_e100_06D09_R_0	BY942984
E_FL_e100_06D13_F_0	BY938431
E_FL_e100_06D13_R_0	BY942985
E_FL_e100_06D14_F_0	BY938432
E_FL_e100_06D18_F_0	BY938433
E_FL_e100_06D19_F_0	BY938434
E_FL_e100_06D20_F_0	BY938435
E_FL_e100_06D22_F_0	BY938436
E_FL_e100_06D22_R_0	BY942986
E_FL_e100_06D24_F_0	BY938437
E_FL_e100_06E01_F_0	BY938438
E_FL_e100_06E03_F_0	BY938439
E_FL_e100_06E05_F_0	BY938440
E_FL_e100_06E05_R_0	BY942987
E_FL_e100_06E07_F_0	BY938441
E_FL_e100_06E09_F_0	BY938442
E_FL_e100_06E09_R_0	BY942988
E_FL_e100_06E11_F_0	BY938443
E_FL_e100_06E12_F_0	BY938444
E_FL_e100_06E12_R_0	BY942989
E_FL_e100_06E15_F_0	BY938445
E_FL_e100_06E15_R_0	BY942990
E_FL_e100_06E20_F_0	BY938446
E_FL_e100_06E20_R_0	BY942991
E_FL_e100_06F01_F_0	BY938447
E_FL_e100_06F01_R_0	BY942992
E_FL_e100_06F03_F_0	BY938448
E_FL_e100_06F03_R_0	BY942993
E_FL_e100_06F04_F_0	BY938449
E_FL_e100_06F05_F_0	BY938450
E_FL_e100_06F07_F_0	BY938451
E_FL_e100_06F07_R_0	BY942994
E_FL_e100_06F12_F_0	BY938452
E_FL_e100_06F13_F_0	BY938453
E_FL_e100_06F13_R_0	BY942995
E_FL_e100_06F14_F_0	BY938454
E_FL_e100_06F14_R_0	BY942996
E_FL_e100_06F16_F_0	BY938455
E_FL_e100_06F16_R_0	BY942997
E_FL_e100_06F17_F_0	BY938456
E_FL_e100_06F18_F_0	BY938457
E_FL_e100_06F19_F_0	BY938458
E_FL_e100_06F19_R_0	BY942998
E_FL_e100_06F23_F_0	BY938459
E_FL_e100_06F24_F_0	BY938460
E_FL_e100_06G01_R_0	BY942999
E_FL_e100_06G03_F_0	BY938461
E_FL_e100_06G04_R_0	BY943000
E_FL_e100_06G08_R_0	BY943001

E_FL_e100_06G09_F_0	BY938462
E_FL_e100_06G09_R_0	BY943002
E_FL_e100_06G13_R_0	BY943003
E_FL_e100_06G14_F_0	BY938463
E_FL_e100_06G15_F_0	BY938464
E_FL_e100_06G16_F_0	BY938465
E_FL_e100_06G16_R_0	BY943004
E_FL_e100_06G17_F_0	BY938466
E_FL_e100_06G17_R_0	BY943005
E_FL_e100_06G19_F_0	BY938467
E_FL_e100_06G19_R_0	BY943006
E_FL_e100_06G22_R_0	BY943007
E_FL_e100_06G23_F_0	BY938468
E_FL_e100_06H04_F_0	BY938469
E_FL_e100_06H04_R_0	BY943008
E_FL_e100_06H05_F_0	BY938470
E_FL_e100_06H05_R_0	BY943009
E_FL_e100_06H06_F_0	BY938471
E_FL_e100_06H06_R_0	BY943010
E_FL_e100_06H08_F_0	BY938472
E_FL_e100_06H08_R_0	BY943011
E_FL_e100_06H10_F_0	BY938473
E_FL_e100_06H11_F_0	BY938474
E_FL_e100_06H11_R_0	BY943012
E_FL_e100_06H13_F_0	BY938475
E_FL_e100_06H13_R_0	BY943013
E_FL_e100_06H14_F_0	BY938476
E_FL_e100_06H15_F_0	BY938477
E_FL_e100_06H16_R_0	BY943014
E_FL_e100_06H19_F_0	BY938478
E_FL_e100_06H20_F_0	BY938479
E_FL_e100_06H20_R_0	BY943015
E_FL_e100_06H22_F_0	BY938480
E_FL_e100_06H23_F_0	BY938481
E_FL_e100_06I02_F_0	BY938482
E_FL_e100_06I03_F_0	BY938483
E_FL_e100_06I03_R_0	BY943016
E_FL_e100_06I05_F_0	BY938484
E_FL_e100_06I05_R_0	BY943017
E_FL_e100_06I07_F_0	BY938485
E_FL_e100_06I10_F_0	BY938486
E_FL_e100_06I10_R_0	BY943018
E_FL_e100_06I11_F_0	BY938487
E_FL_e100_06I11_R_0	BY943019
E_FL_e100_06I17_F_0	BY938488
E_FL_e100_06I17_R_0	BY943020
E_FL_e100_06I21_F_0	BY938489
E_FL_e100_06I21_R_0	BY943021
E_FL_e100_06I22_F_0	BY938490
E_FL_e100_06I23_F_0	BY938491
E_FL_e100_06I23_R_0	BY943022
E_FL_e100_06I24_F_0	BY938492
E_FL_e100_06J02_F_0	BY938493
E_FL_e100_06J04_F_0	BY938494

E_FL_e100_06J07_F_0	BY938495
E_FL_e100_06J08_R_0	BY943023
E_FL_e100_06J09_F_0	BY938496
E_FL_e100_06J09_R_0	BY943024
E_FL_e100_06J10_F_0	BY938497
E_FL_e100_06J10_R_0	BY943025
E_FL_e100_06J12_F_0	BY938498
E_FL_e100_06J12_R_0	BY943026
E_FL_e100_06J16_F_0	BY938499
E_FL_e100_06J16_R_0	BY943027
E_FL_e100_06J20_R_0	BY943028
E_FL_e100_06J21_F_0	BY938500
E_FL_e100_06J23_F_0	BY938501
E_FL_e100_06J24_F_0	BY938502
E_FL_e100_06K01_F_0	BY938503
E_FL_e100_06K01_R_0	BY943029
E_FL_e100_06K03_F_0	BY938504
E_FL_e100_06K03_R_0	BY943030
E_FL_e100_06K05_F_0	BY938505
E_FL_e100_06K06_R_0	BY943031
E_FL_e100_06K08_F_0	BY938506
E_FL_e100_06K09_F_0	BY938507
E_FL_e100_06K13_F_0	BY938508
E_FL_e100_06K16_F_0	BY938509
E_FL_e100_06K16_R_0	BY943032
E_FL_e100_06K17_F_0	BY938510
E_FL_e100_06K18_F_0	BY938511
E_FL_e100_06K18_R_0	BY943033
E_FL_e100_06K19_F_0	BY938512
E_FL_e100_06K20_F_0	BY938513
E_FL_e100_06K20_R_0	BY943034
E_FL_e100_06K21_F_0	BY938514
E_FL_e100_06K22_F_0	BY938515
E_FL_e100_06K23_F_0	BY938516
E_FL_e100_06K24_F_0	BY938517
E_FL_e100_06L01_F_0	BY938518
E_FL_e100_06L01_R_0	BY943035
E_FL_e100_06L03_F_0	BY938519
E_FL_e100_06L04_F_0	BY938520
E_FL_e100_06L04_R_0	BY943036
E_FL_e100_06L05_F_0	BY938521
E_FL_e100_06L05_R_0	BY943037
E_FL_e100_06L06_R_0	BY943038
E_FL_e100_06L07_F_0	BY938522
E_FL_e100_06L08_R_0	BY943039
E_FL_e100_06L09_F_0	BY938523
E_FL_e100_06L10_F_0	BY938524
E_FL_e100_06L12_F_0	BY938525
E_FL_e100_06L12_R_0	BY943040
E_FL_e100_06L13_F_0	BY938526
E_FL_e100_06L13_R_0	BY943041
E_FL_e100_06L16_F_0	BY938527
E_FL_e100_06L21_F_0	BY938528
E_FL_e100_06L22_F_0	BY938529

E_FL_e100_06L22_R_0	BY943042
E_FL_e100_06M01_F_0	BY938530
E_FL_e100_06M01_R_0	BY943043
E_FL_e100_06M03_F_0	BY938531
E_FL_e100_06M05_F_0	BY938532
E_FL_e100_06M05_R_0	BY943044
E_FL_e100_06M09_F_0	BY938533
E_FL_e100_06M10_F_0	BY938534
E_FL_e100_06M13_F_0	BY938535
E_FL_e100_06M15_F_0	BY938536
E_FL_e100_06M16_F_0	BY938537
E_FL_e100_06M19_R_0	BY943045
E_FL_e100_06M20_F_0	BY938538
E_FL_e100_06M22_R_0	BY943046
E_FL_e100_06N02_F_0	BY938539
E_FL_e100_06N03_F_0	BY938540
E_FL_e100_06N03_R_0	BY943047
E_FL_e100_06N05_F_0	BY938541
E_FL_e100_06N07_F_0	BY938542
E_FL_e100_06N07_R_0	BY943048
E_FL_e100_06N10_F_0	BY938543
E_FL_e100_06N11_F_0	BY938544
E_FL_e100_06N12_F_0	BY938545
E_FL_e100_06N12_R_0	BY943049
E_FL_e100_06N14_F_0	BY938546
E_FL_e100_06N15_F_0	BY938547
E_FL_e100_06N16_F_0	BY938548
E_FL_e100_06N17_R_0	BY943050
E_FL_e100_06N18_F_0	BY938549
E_FL_e100_06N19_F_0	BY938550
E_FL_e100_06N19_R_0	BY943051
E_FL_e100_06N20_F_0	BY938551
E_FL_e100_06N20_R_0	BY943052
E_FL_e100_06N22_F_0	BY938552
E_FL_e100_06N22_R_0	BY943053
E_FL_e100_06N23_F_0	BY938553
E_FL_e100_06O01_F_0	BY938554
E_FL_e100_06O01_R_0	BY943054
E_FL_e100_06O02_F_0	BY938555
E_FL_e100_06O03_R_0	BY943055
E_FL_e100_06O04_R_0	BY943056
E_FL_e100_06O06_F_0	BY938556
E_FL_e100_06O07_F_0	BY938557
E_FL_e100_06O08_F_0	BY938558
E_FL_e100_06O11_F_0	BY938559
E_FL_e100_06O12_F_0	BY938560
E_FL_e100_06O12_R_0	BY943057
E_FL_e100_06O15_F_0	BY938561
E_FL_e100_06O15_R_0	BY943058
E_FL_e100_06O17_F_0	BY938562
E_FL_e100_06O18_R_0	BY943059
E_FL_e100_06O19_R_0	BY943060
E_FL_e100_06O20_F_0	BY938563
E_FL_e100_06O20_R_0	BY943061

E_FL_e100_06O21_F_0	BY938564
E_FL_e100_06O24_F_0	BY938565
E_FL_e100_06P04_F_0	BY938566
E_FL_e100_06P05_F_0	BY938567
E_FL_e100_06P05_R_0	BY943062
E_FL_e100_06P06_F_0	BY938568
E_FL_e100_06P07_F_0	BY938569
E_FL_e100_06P07_R_0	BY943063
E_FL_e100_06P09_F_0	BY938570
E_FL_e100_06P11_F_0	BY938571
E_FL_e100_06P13_F_0	BY938572
E_FL_e100_06P13_R_0	BY943064
E_FL_e100_06P14_F_0	BY938573
E_FL_e100_06P14_R_0	BY943065
E_FL_e100_06P16_F_0	BY938574
E_FL_e100_06P18_R_0	BY943066
E_FL_e100_06P21_F_0	BY938575
E_FL_e100_06P24_F_0	BY938576
E_FL_e100_07A03_F_0	BY938577
E_FL_e100_07A05_F_0	BY938578
E_FL_e100_07A08_F_0	BY938579
E_FL_e100_07A09_F_0	BY938580
E_FL_e100_07A10_R_0	BY943067
E_FL_e100_07A11_F_0	BY938581
E_FL_e100_07A13_F_0	BY938582
E_FL_e100_07A16_F_0	BY938583
E_FL_e100_07A20_F_0	BY938584
E_FL_e100_07A21_F_0	BY938585
E_FL_e100_07A22_F_0	BY938586
E_FL_e100_07A22_R_0	BY943068
E_FL_e100_07B02_F_0	BY938587
E_FL_e100_07B02_R_0	BY943069
E_FL_e100_07B03_F_0	BY938588
E_FL_e100_07B04_F_0	BY938589
E_FL_e100_07B04_R_0	BY943070
E_FL_e100_07B05_F_0	BY938590
E_FL_e100_07B08_F_0	BY938591
E_FL_e100_07B08_R_0	BY943071
E_FL_e100_07B11_F_0	BY938592
E_FL_e100_07B12_F_0	BY938593
E_FL_e100_07B13_F_0	BY938594
E_FL_e100_07B13_R_0	BY943072
E_FL_e100_07B14_F_0	BY938595
E_FL_e100_07B15_R_0	BY943073
E_FL_e100_07B17_F_0	BY938596
E_FL_e100_07B20_F_0	BY938597
E_FL_e100_07B20_R_0	BY943074
E_FL_e100_07B21_F_0	BY938598
E_FL_e100_07B22_F_0	BY938599
E_FL_e100_07B24_F_0	BY938600
E_FL_e100_07B24_R_0	BY943075
E_FL_e100_07C03_F_0	BY938601
E_FL_e100_07C03_R_0	BY943076
E_FL_e100_07C04_F_0	BY938602

E_FL_e100_07C04_R_0	BY943077
E_FL_e100_07C05_F_0	BY938603
E_FL_e100_07C05_R_0	BY943078
E_FL_e100_07C13_F_0	BY938604
E_FL_e100_07C15_F_0	BY938605
E_FL_e100_07C16_F_0	BY938606
E_FL_e100_07C20_F_0	BY938607
E_FL_e100_07C22_F_0	BY938608
E_FL_e100_07C22_R_0	BY943079
E_FL_e100_07C23_F_0	BY938609
E_FL_e100_07C24_F_0	BY938610
E_FL_e100_07D02_F_0	BY938611
E_FL_e100_07D02_R_0	BY943080
E_FL_e100_07D03_F_0	BY938612
E_FL_e100_07D06_F_0	BY938613
E_FL_e100_07D06_R_0	BY943081
E_FL_e100_07D07_R_0	BY943082
E_FL_e100_07D08_F_0	BY938614
E_FL_e100_07D08_R_0	BY943083
E_FL_e100_07D09_F_0	BY938615
E_FL_e100_07D09_R_0	BY943084
E_FL_e100_07D10_F_0	BY938616
E_FL_e100_07D11_F_0	BY938617
E_FL_e100_07D11_R_0	BY943085
E_FL_e100_07D14_F_0	BY938618
E_FL_e100_07D14_R_0	BY943086
E_FL_e100_07D16_F_0	BY938619
E_FL_e100_07D19_F_0	BY938620
E_FL_e100_07D19_R_0	BY943087
E_FL_e100_07D20_F_0	BY938621
E_FL_e100_07D20_R_0	BY943088
E_FL_e100_07D21_F_0	BY938622
E_FL_e100_07D21_R_0	BY943089
E_FL_e100_07D22_F_0	BY938623
E_FL_e100_07D23_F_0	BY938624
E_FL_e100_07D23_R_0	BY943090
E_FL_e100_07E03_F_0	BY938625
E_FL_e100_07E04_F_0	BY938626
E_FL_e100_07E04_R_0	BY943091
E_FL_e100_07E06_R_0	BY943092
E_FL_e100_07E07_F_0	BY938627
E_FL_e100_07E07_R_0	BY943093
E_FL_e100_07E08_F_0	BY938628
E_FL_e100_07E10_F_0	BY938629
E_FL_e100_07E10_R_0	BY943094
E_FL_e100_07E13_F_0	BY938630
E_FL_e100_07E14_F_0	BY938631
E_FL_e100_07E16_F_0	BY938632
E_FL_e100_07E16_R_0	BY943095
E_FL_e100_07E17_F_0	BY938633
E_FL_e100_07E23_F_0	BY938634
E_FL_e100_07F01_F_0	BY938635
E_FL_e100_07F04_F_0	BY938636
E_FL_e100_07F06_F_0	BY938637

E_FL_e100_07F07_R_0	BY943096
E_FL_e100_07F09_F_0	BY938638
E_FL_e100_07F09_R_0	BY943097
E_FL_e100_07F13_F_0	BY938639
E_FL_e100_07F13_R_0	BY943098
E_FL_e100_07F16_F_0	BY938640
E_FL_e100_07F16_R_0	BY943099
E_FL_e100_07F19_F_0	BY938641
E_FL_e100_07F19_R_0	BY943100
E_FL_e100_07F20_F_0	BY938642
E_FL_e100_07F22_F_0	BY938643
E_FL_e100_07F22_R_0	BY943101
E_FL_e100_07F23_F_0	BY938644
E_FL_e100_07G05_F_0	BY938645
E_FL_e100_07G05_R_0	BY943102
E_FL_e100_07G06_F_0	BY938646
E_FL_e100_07G07_F_0	BY938647
E_FL_e100_07G08_F_0	BY938648
E_FL_e100_07G09_F_0	BY938649
E_FL_e100_07G10_F_0	BY938650
E_FL_e100_07G13_F_0	BY938651
E_FL_e100_07G14_F_0	BY938652
E_FL_e100_07G16_F_0	BY938653
E_FL_e100_07G18_R_0	BY943103
E_FL_e100_07G20_F_0	BY938654
E_FL_e100_07G21_F_0	BY938655
E_FL_e100_07G21_R_0	BY943104
E_FL_e100_07G22_F_0	BY938656
E_FL_e100_07G22_R_0	BY943105
E_FL_e100_07H02_F_0	BY938657
E_FL_e100_07H03_F_0	BY938658
E_FL_e100_07H05_F_0	BY938659
E_FL_e100_07H05_R_0	BY943106
E_FL_e100_07H06_F_0	BY938660
E_FL_e100_07H06_R_0	BY943107
E_FL_e100_07H08_F_0	BY938661
E_FL_e100_07H09_F_0	BY938662
E_FL_e100_07H09_R_0	BY943108
E_FL_e100_07H11_F_0	BY938663
E_FL_e100_07H11_R_0	BY943109
E_FL_e100_07H13_F_0	BY938664
E_FL_e100_07H13_R_0	BY943110
E_FL_e100_07H14_F_0	BY938665
E_FL_e100_07H15_F_0	BY938666
E_FL_e100_07H15_R_0	BY943111
E_FL_e100_07H17_F_0	BY938667
E_FL_e100_07H17_R_0	BY943112
E_FL_e100_07H20_F_0	BY938668
E_FL_e100_07H20_R_0	BY943113
E_FL_e100_07H21_F_0	BY938669
E_FL_e100_07H22_F_0	BY938670
E_FL_e100_07H22_R_0	BY943114
E_FL_e100_07H24_F_0	BY938671
E_FL_e100_07H24_R_0	BY943115

E_FL_e100_07I01_F_0	BY938672
E_FL_e100_07I03_F_0	BY938673
E_FL_e100_07I08_F_0	BY938674
E_FL_e100_07I10_F_0	BY938675
E_FL_e100_07I11_F_0	BY938676
E_FL_e100_07I13_F_0	BY938677
E_FL_e100_07I13_R_0	BY943116
E_FL_e100_07I14_F_0	BY938678
E_FL_e100_07I16_F_0	BY938679
E_FL_e100_07I17_F_0	BY938680
E_FL_e100_07I18_F_0	BY938681
E_FL_e100_07I18_R_0	BY943117
E_FL_e100_07J04_F_0	BY938682
E_FL_e100_07J04_R_0	BY943118
E_FL_e100_07J05_F_0	BY938683
E_FL_e100_07J06_F_0	BY938684
E_FL_e100_07J06_R_0	BY943119
E_FL_e100_07J08_F_0	BY938685
E_FL_e100_07J08_R_0	BY943120
E_FL_e100_07J10_F_0	BY938686
E_FL_e100_07J10_R_0	BY943121
E_FL_e100_07J11_F_0	BY938687
E_FL_e100_07J11_R_0	BY943122
E_FL_e100_07J12_F_0	BY938688
E_FL_e100_07J12_R_0	BY943123
E_FL_e100_07J13_F_0	BY938689
E_FL_e100_07J13_R_0	BY943124
E_FL_e100_07J14_F_0	BY938690
E_FL_e100_07J14_R_0	BY943125
E_FL_e100_07J15_F_0	BY938691
E_FL_e100_07J16_F_0	BY938692
E_FL_e100_07J16_R_0	BY943126
E_FL_e100_07J20_F_0	BY938693
E_FL_e100_07J20_R_0	BY943127
E_FL_e100_07J21_F_0	BY938694
E_FL_e100_07J21_R_0	BY943128
E_FL_e100_07J23_F_0	BY938695
E_FL_e100_07J24_F_0	BY938696
E_FL_e100_07J24_R_0	BY943129
E_FL_e100_07K01_F_0	BY938697
E_FL_e100_07K01_R_0	BY943130
E_FL_e100_07K02_F_0	BY938698
E_FL_e100_07K03_F_0	BY938699
E_FL_e100_07K04_F_0	BY938700
E_FL_e100_07K05_F_0	BY938701
E_FL_e100_07K05_R_0	BY943131
E_FL_e100_07K06_F_0	BY938702
E_FL_e100_07K07_F_0	BY938703
E_FL_e100_07K10_F_0	BY938704
E_FL_e100_07K10_R_0	BY943132
E_FL_e100_07K13_F_0	BY938705
E_FL_e100_07K16_F_0	BY938706
E_FL_e100_07K17_F_0	BY938707
E_FL_e100_07K21_F_0	BY938708

E_FL_e100_07K22_F_0	BY938709
E_FL_e100_07K22_R_0	BY943133
E_FL_e100_07K23_F_0	BY938710
E_FL_e100_07L01_F_0	BY938711
E_FL_e100_07L01_R_0	BY943134
E_FL_e100_07L03_F_0	BY938712
E_FL_e100_07L03_R_0	BY943135
E_FL_e100_07L06_F_0	BY938713
E_FL_e100_07L08_F_0	BY938714
E_FL_e100_07L09_F_0	BY938715
E_FL_e100_07L09_R_0	BY943136
E_FL_e100_07L10_F_0	BY938716
E_FL_e100_07L10_R_0	BY943137
E_FL_e100_07L13_F_0	BY938717
E_FL_e100_07L15_F_0	BY938718
E_FL_e100_07L16_F_0	BY938719
E_FL_e100_07L16_R_0	BY943138
E_FL_e100_07L17_F_0	BY938720
E_FL_e100_07L17_R_0	BY943139
E_FL_e100_07L18_F_0	BY938721
E_FL_e100_07L22_F_0	BY938722
E_FL_e100_07L22_R_0	BY943140
E_FL_e100_07M01_F_0	BY938723
E_FL_e100_07M02_F_0	BY938724
E_FL_e100_07M03_F_0	BY938725
E_FL_e100_07M03_R_0	BY943141
E_FL_e100_07M06_F_0	BY938726
E_FL_e100_07M10_F_0	BY938727
E_FL_e100_07M10_R_0	BY943142
E_FL_e100_07M11_F_0	BY938728
E_FL_e100_07M11_R_0	BY943143
E_FL_e100_07M14_F_0	BY938729
E_FL_e100_07M14_R_0	BY943144
E_FL_e100_07M16_F_0	BY938730
E_FL_e100_07M16_R_0	BY943145
E_FL_e100_07M17_F_0	BY938731
E_FL_e100_07M17_R_0	BY943146
E_FL_e100_07M21_F_0	BY938732
E_FL_e100_07M21_R_0	BY943147
E_FL_e100_07M22_F_0	BY938733
E_FL_e100_07M23_F_0	BY938734
E_FL_e100_07M23_R_0	BY943148
E_FL_e100_07M24_F_0	BY938735
E_FL_e100_07N02_F_0	BY938736
E_FL_e100_07N04_F_0	BY938737
E_FL_e100_07N04_R_0	BY943149
E_FL_e100_07N06_F_0	BY938738
E_FL_e100_07N07_F_0	BY938739
E_FL_e100_07N07_R_0	BY943150
E_FL_e100_07N08_F_0	BY938740
E_FL_e100_07N08_R_0	BY943151
E_FL_e100_07N09_F_0	BY938741
E_FL_e100_07N09_R_0	BY943152
E_FL_e100_07N10_F_0	BY938742

E_FL_e100_07N12_F_0	BY938743
E_FL_e100_07N13_F_0	BY938744
E_FL_e100_07N13_R_0	BY943153
E_FL_e100_07N16_F_0	BY938745
E_FL_e100_07N16_R_0	BY943154
E_FL_e100_07N19_F_0	BY938746
E_FL_e100_07N21_F_0	BY938747
E_FL_e100_07N22_F_0	BY938748
E_FL_e100_07N22_R_0	BY943155
E_FL_e100_07N24_F_0	BY938749
E_FL_e100_07O02_F_0	BY938750
E_FL_e100_07O06_F_0	BY938751
E_FL_e100_07O06_R_0	BY943156
E_FL_e100_07O07_F_0	BY938752
E_FL_e100_07O08_F_0	BY938753
E_FL_e100_07O08_R_0	BY943157
E_FL_e100_07O10_F_0	BY938754
E_FL_e100_07O10_R_0	BY943158
E_FL_e100_07O12_F_0	BY938755
E_FL_e100_07O15_F_0	BY938756
E_FL_e100_07O17_F_0	BY938757
E_FL_e100_07O17_R_0	BY943159
E_FL_e100_07O18_F_0	BY938758
E_FL_e100_07O19_F_0	BY938759
E_FL_e100_07O19_R_0	BY943160
E_FL_e100_07O20_F_0	BY938760
E_FL_e100_07O20_R_0	BY943161
E_FL_e100_07O21_F_0	BY938761
E_FL_e100_07O23_F_0	BY938762
E_FL_e100_07O24_F_0	BY938763
E_FL_e100_07O24_R_0	BY943162
E_FL_e100_07P01_F_0	BY938764
E_FL_e100_07P05_F_0	BY938765
E_FL_e100_07P08_F_0	BY938766
E_FL_e100_07P13_F_0	BY938767
E_FL_e100_07P13_R_0	BY943163
E_FL_e100_07P15_R_0	BY943164
E_FL_e100_07P16_F_0	BY938768
E_FL_e100_07P16_R_0	BY943165
E_FL_e100_07P17_F_0	BY938769
E_FL_e100_07P18_F_0	BY938770
E_FL_e100_07P18_R_0	BY943166
E_FL_e100_07P19_F_0	BY938771
E_FL_e100_07P19_R_0	BY943167
E_FL_e100_07P21_F_0	BY938772
E_FL_e100_07P21_R_0	BY943168
E_FL_e100_07P22_F_0	BY938773
E_FL_e100_07P22_R_0	BY943169
E_FL_e100_08A02_F_0	BY938774
E_FL_e100_08A05_F_0	BY938775
E_FL_e100_08A05_R_0	BY943170
E_FL_e100_08A06_F_0	BY938776
E_FL_e100_08A11_F_0	BY938777
E_FL_e100_08A14_F_0	BY938778

E_FL_e100_08A15_F_0	BY938779
E_FL_e100_08A16_R_0	BY943171
E_FL_e100_08A23_F_0	BY938780
E_FL_e100_08A23_R_0	BY943172
E_FL_e100_08B01_F_0	BY938781
E_FL_e100_08B01_R_0	BY943173
E_FL_e100_08B04_F_0	BY938782
E_FL_e100_08B05_R_0	BY943174
E_FL_e100_08B06_F_0	BY938783
E_FL_e100_08B06_R_0	BY943175
E_FL_e100_08B08_F_0	BY938784
E_FL_e100_08B12_F_0	BY938785
E_FL_e100_08B13_F_0	BY938786
E_FL_e100_08B14_F_0	BY938787
E_FL_e100_08B14_R_0	BY943176
E_FL_e100_08B17_F_0	BY938788
E_FL_e100_08B18_F_0	BY938789
E_FL_e100_08B18_R_0	BY943177
E_FL_e100_08B19_F_0	BY938790
E_FL_e100_08B19_R_0	BY943178
E_FL_e100_08C02_R_0	BY943179
E_FL_e100_08C03_F_0	BY938791
E_FL_e100_08C05_F_0	BY938792
E_FL_e100_08C06_F_0	BY938793
E_FL_e100_08C06_R_0	BY943180
E_FL_e100_08C08_R_0	BY943181
E_FL_e100_08C14_F_0	BY938794
E_FL_e100_08C14_R_0	BY943182
E_FL_e100_08C16_F_0	BY938795
E_FL_e100_08C17_F_0	BY938796
E_FL_e100_08C17_R_0	BY943183
E_FL_e100_08C18_F_0	BY938797
E_FL_e100_08C19_R_0	BY943184
E_FL_e100_08C20_F_0	BY938798
E_FL_e100_08C20_R_0	BY943185
E_FL_e100_08C24_F_0	BY938799
E_FL_e100_08C24_R_0	BY943186
E_FL_e100_08D02_F_0	BY938800
E_FL_e100_08D02_R_0	BY943187
E_FL_e100_08D03_F_0	BY938801
E_FL_e100_08D03_R_0	BY943188
E_FL_e100_08D04_F_0	BY938802
E_FL_e100_08D04_R_0	BY943189
E_FL_e100_08D08_F_0	BY938803
E_FL_e100_08D09_F_0	BY938804
E_FL_e100_08D10_F_0	BY938805
E_FL_e100_08D10_R_0	BY943190
E_FL_e100_08D11_F_0	BY938806
E_FL_e100_08D11_R_0	BY943191
E_FL_e100_08D13_R_0	BY943192
E_FL_e100_08D15_F_0	BY938807
E_FL_e100_08D15_R_0	BY943193
E_FL_e100_08D17_R_0	BY943194
E_FL_e100_08D19_F_0	BY938808

E_FL_e100_08D19_R_0	BY943195
E_FL_e100_08D20_F_0	BY938809
E_FL_e100_08D22_F_0	BY938810
E_FL_e100_08D24_F_0	BY938811
E_FL_e100_08E01_F_0	BY938812
E_FL_e100_08E01_R_0	BY943196
E_FL_e100_08E02_F_0	BY938813
E_FL_e100_08E02_R_0	BY943197
E_FL_e100_08E05_F_0	BY938814
E_FL_e100_08E05_R_0	BY943198
E_FL_e100_08E06_F_0	BY938815
E_FL_e100_08E09_R_0	BY943199
E_FL_e100_08E10_F_0	BY938816
E_FL_e100_08E12_F_0	BY938817
E_FL_e100_08E12_R_0	BY943200
E_FL_e100_08E13_F_0	BY938818
E_FL_e100_08E16_F_0	BY938819
E_FL_e100_08E18_F_0	BY938820
E_FL_e100_08E18_R_0	BY943201
E_FL_e100_08E19_F_0	BY938821
E_FL_e100_08E21_F_0	BY938822
E_FL_e100_08E21_R_0	BY943202
E_FL_e100_08E22_F_0	BY938823
E_FL_e100_08E22_R_0	BY943203
E_FL_e100_08E23_R_0	BY943204
E_FL_e100_08E24_F_0	BY938824
E_FL_e100_08E24_R_0	BY943205
E_FL_e100_08F01_F_0	BY938825
E_FL_e100_08F01_R_0	BY943206
E_FL_e100_08F02_F_0	BY938826
E_FL_e100_08F02_R_0	BY943207
E_FL_e100_08F03_F_0	BY938827
E_FL_e100_08F03_R_0	BY943208
E_FL_e100_08F04_F_0	BY938828
E_FL_e100_08F08_F_0	BY938829
E_FL_e100_08F08_R_0	BY943209
E_FL_e100_08F09_F_0	BY938830
E_FL_e100_08F10_F_0	BY938831
E_FL_e100_08F11_F_0	BY938832
E_FL_e100_08F11_R_0	BY943210
E_FL_e100_08F13_F_0	BY938833
E_FL_e100_08F14_F_0	BY938834
E_FL_e100_08F15_F_0	BY938835
E_FL_e100_08F16_F_0	BY938836
E_FL_e100_08F17_F_0	BY938837
E_FL_e100_08F17_R_0	BY943211
E_FL_e100_08F18_F_0	BY938838
E_FL_e100_08F18_R_0	BY943212
E_FL_e100_08F19_F_0	BY938839
E_FL_e100_08F19_R_0	BY943213
E_FL_e100_08F21_F_0	BY938840
E_FL_e100_08F21_R_0	BY943214
E_FL_e100_08F22_F_0	BY938841
E_FL_e100_08F22_R_0	BY943215

E_FL_e100_08G02_F_0	BY938842
E_FL_e100_08G02_R_0	BY943216
E_FL_e100_08G03_F_0	BY938843
E_FL_e100_08G07_R_0	BY943217
E_FL_e100_08G08_F_0	BY938844
E_FL_e100_08G09_F_0	BY938845
E_FL_e100_08G09_R_0	BY943218
E_FL_e100_08G10_F_0	BY938846
E_FL_e100_08G10_R_0	BY943219
E_FL_e100_08G13_R_0	BY943220
E_FL_e100_08G15_F_0	BY938847
E_FL_e100_08G15_R_0	BY943221
E_FL_e100_08G16_F_0	BY938848
E_FL_e100_08G18_F_0	BY938849
E_FL_e100_08G19_F_0	BY938850
E_FL_e100_08G20_F_0	BY938851
E_FL_e100_08G21_F_0	BY938852
E_FL_e100_08G21_R_0	BY943222
E_FL_e100_08G22_F_0	BY938853
E_FL_e100_08G23_F_0	BY938854
E_FL_e100_08H01_F_0	BY938855
E_FL_e100_08H01_R_0	BY943223
E_FL_e100_08H06_F_0	BY938856
E_FL_e100_08H06_R_0	BY943224
E_FL_e100_08H07_F_0	BY938857
E_FL_e100_08H09_F_0	BY938858
E_FL_e100_08H10_F_0	BY938859
E_FL_e100_08H10_R_0	BY943225
E_FL_e100_08H13_F_0	BY938860
E_FL_e100_08H14_F_0	BY938861
E_FL_e100_08H14_R_0	BY943226
E_FL_e100_08H15_F_0	BY938862
E_FL_e100_08H15_R_0	BY943227
E_FL_e100_08H18_F_0	BY938863
E_FL_e100_08H20_F_0	BY938864
E_FL_e100_08H20_R_0	BY943228
E_FL_e100_08H21_F_0	BY938865
E_FL_e100_08H21_R_0	BY943229
E_FL_e100_08I01_F_0	BY938866
E_FL_e100_08I02_F_0	BY938867
E_FL_e100_08I02_R_0	BY943230
E_FL_e100_08I04_F_0	BY938868
E_FL_e100_08I10_F_0	BY938869
E_FL_e100_08I11_F_0	BY938870
E_FL_e100_08I13_F_0	BY938871
E_FL_e100_08I13_R_0	BY943231
E_FL_e100_08I14_R_0	BY943232
E_FL_e100_08I15_F_0	BY938872
E_FL_e100_08I18_F_0	BY938873
E_FL_e100_08I18_R_0	BY943233
E_FL_e100_08I19_F_0	BY938874
E_FL_e100_08I20_F_0	BY938875
E_FL_e100_08I20_R_0	BY943234
E_FL_e100_08J01_F_0	BY938876

E_FL_e100_08J02_F_0	BY938877
E_FL_e100_08J02_R_0	BY943235
E_FL_e100_08J03_F_0	BY938878
E_FL_e100_08J03_R_0	BY943236
E_FL_e100_08J04_F_0	BY938879
E_FL_e100_08J04_R_0	BY943237
E_FL_e100_08J05_F_0	BY938880
E_FL_e100_08J05_R_0	BY943238
E_FL_e100_08J07_F_0	BY938881
E_FL_e100_08J09_F_0	BY938882
E_FL_e100_08J10_F_0	BY938883
E_FL_e100_08J10_R_0	BY943239
E_FL_e100_08J11_F_0	BY938884
E_FL_e100_08J12_F_0	BY938885
E_FL_e100_08J12_R_0	BY943240
E_FL_e100_08J14_R_0	BY943241
E_FL_e100_08J15_F_0	BY938886
E_FL_e100_08J15_R_0	BY943242
E_FL_e100_08J16_F_0	BY938887
E_FL_e100_08J18_F_0	BY938888
E_FL_e100_08J19_F_0	BY938889
E_FL_e100_08J21_F_0	BY938890
E_FL_e100_08J22_F_0	BY938891
E_FL_e100_08J24_F_0	BY938892
E_FL_e100_08K01_F_0	BY938893
E_FL_e100_08K02_F_0	BY938894
E_FL_e100_08K03_F_0	BY938895
E_FL_e100_08K04_F_0	BY938896
E_FL_e100_08K07_F_0	BY938897
E_FL_e100_08K08_F_0	BY938898
E_FL_e100_08K08_R_0	BY943243
E_FL_e100_08K09_F_0	BY938899
E_FL_e100_08K12_F_0	BY938900
E_FL_e100_08K14_F_0	BY938901
E_FL_e100_08K14_R_0	BY943244
E_FL_e100_08K17_F_0	BY938902
E_FL_e100_08K20_F_0	BY938903
E_FL_e100_08K20_R_0	BY943245
E_FL_e100_08K23_F_0	BY938904
E_FL_e100_08K24_F_0	BY938905
E_FL_e100_08L08_F_0	BY938906
E_FL_e100_08L08_R_0	BY943246
E_FL_e100_08L09_F_0	BY938907
E_FL_e100_08L09_R_0	BY943247
E_FL_e100_08L10_F_0	BY938908
E_FL_e100_08L11_F_0	BY938909
E_FL_e100_08L11_R_0	BY943248
E_FL_e100_08L12_F_0	BY938910
E_FL_e100_08L13_F_0	BY938911
E_FL_e100_08L13_R_0	BY943249
E_FL_e100_08L14_F_0	BY938912
E_FL_e100_08L17_F_0	BY938913
E_FL_e100_08L19_F_0	BY938914
E_FL_e100_08L19_R_0	BY943250

E_FL_e100_08L20_F_0	BY938915
E_FL_e100_08L20_R_0	BY943251
E_FL_e100_08L21_F_0	BY938916
E_FL_e100_08L21_R_0	BY943252
E_FL_e100_08L22_F_0	BY938917
E_FL_e100_08L22_R_0	BY943253
E_FL_e100_08L23_F_0	BY938918
E_FL_e100_08L23_R_0	BY943254
E_FL_e100_08L24_F_0	BY938919
E_FL_e100_08M01_F_0	BY938920
E_FL_e100_08M02_F_0	BY938921
E_FL_e100_08M02_R_0	BY943255
E_FL_e100_08M04_F_0	BY938922
E_FL_e100_08M07_F_0	BY938923
E_FL_e100_08M09_F_0	BY938924
E_FL_e100_08M09_R_0	BY943256
E_FL_e100_08M10_F_0	BY938925
E_FL_e100_08M13_F_0	BY938926
E_FL_e100_08M14_F_0	BY938927
E_FL_e100_08M14_R_0	BY943257
E_FL_e100_08M19_F_0	BY938928
E_FL_e100_08M20_F_0	BY938929
E_FL_e100_08M21_F_0	BY938930
E_FL_e100_08M21_R_0	BY943258
E_FL_e100_08M22_F_0	BY938931
E_FL_e100_08N02_F_0	BY938932
E_FL_e100_08N02_R_0	BY943259
E_FL_e100_08N03_F_0	BY938933
E_FL_e100_08N03_R_0	BY943260
E_FL_e100_08N04_F_0	BY938934
E_FL_e100_08N05_F_0	BY938935
E_FL_e100_08N05_R_0	BY943261
E_FL_e100_08N07_F_0	BY938936
E_FL_e100_08N07_R_0	BY943262
E_FL_e100_08N08_F_0	BY938937
E_FL_e100_08N08_R_0	BY943263
E_FL_e100_08N09_F_0	BY938938
E_FL_e100_08N10_F_0	BY938939
E_FL_e100_08N11_F_0	BY938940
E_FL_e100_08N12_F_0	BY938941
E_FL_e100_08N14_F_0	BY938942
E_FL_e100_08N16_F_0	BY938943
E_FL_e100_08N17_F_0	BY938944
E_FL_e100_08N18_F_0	BY938945
E_FL_e100_08N19_F_0	BY938946
E_FL_e100_08N19_R_0	BY943264
E_FL_e100_08N20_F_0	BY938947
E_FL_e100_08N20_R_0	BY943265
E_FL_e100_08N21_F_0	BY938948
E_FL_e100_08N21_R_0	BY943266
E_FL_e100_08N22_F_0	BY938949
E_FL_e100_08N24_F_0	BY938950
E_FL_e100_08O03_F_0	BY938951
E_FL_e100_08O04_F_0	BY938952

E_FL_e100_08O05_R_0	BY943267
E_FL_e100_08O07_F_0	BY938953
E_FL_e100_08O07_R_0	BY943268
E_FL_e100_08O09_F_0	BY938954
E_FL_e100_08O09_R_0	BY943269
E_FL_e100_08O18_F_0	BY938955
E_FL_e100_08O20_F_0	BY938956
E_FL_e100_08O20_R_0	BY943270
E_FL_e100_08O21_F_0	BY938957
E_FL_e100_08O21_R_0	BY943271
E_FL_e100_08O23_F_0	BY938958
E_FL_e100_08O23_R_0	BY943272
E_FL_e100_08P02_F_0	BY938959
E_FL_e100_08P02_R_0	BY943273
E_FL_e100_08P03_F_0	BY938960
E_FL_e100_08P05_F_0	BY938961
E_FL_e100_08P06_F_0	BY938962
E_FL_e100_08P06_R_0	BY943274
E_FL_e100_08P08_F_0	BY938963
E_FL_e100_08P10_F_0	BY938964
E_FL_e100_08P12_R_0	BY943275
E_FL_e100_08P13_F_0	BY938965
E_FL_e100_08P13_R_0	BY943276
E_FL_e100_08P15_F_0	BY938966
E_FL_e100_08P15_R_0	BY943277
E_FL_e100_08P17_F_0	BY938967
E_FL_e100_08P18_F_0	BY938968
E_FL_e100_08P19_F_0	BY938969
E_FL_e100_08P19_R_0	BY943278
E_FL_e100_08P24_F_0	BY938970
E_FL_e100_08P24_R_0	BY943279
E_FL_e100_09A01_F_0	BY940475
E_FL_e100_09A02_R_0	BY944199
E_FL_e100_09A03_F_0	BY940476
E_FL_e100_09A09_F_0	BY940477
E_FL_e100_09A10_R_0	BY944200
E_FL_e100_09A12_F_0	BY940478
E_FL_e100_09A13_F_0	BY940479
E_FL_e100_09A14_R_0	BY944201
E_FL_e100_09A15_F_0	BY940480
E_FL_e100_09A16_F_0	BY940481
E_FL_e100_09A16_R_0	BY944202
E_FL_e100_09A18_F_0	BY940482
E_FL_e100_09A20_F_0	BY940483
E_FL_e100_09A23_F_0	BY940484
E_FL_e100_09A23_R_0	BY944203
E_FL_e100_09A24_F_0	BY940485
E_FL_e100_09B02_F_0	BY940486
E_FL_e100_09B02_R_0	BY944204
E_FL_e100_09B03_F_0	BY940487
E_FL_e100_09B03_R_0	BY944205
E_FL_e100_09B05_F_0	BY940488
E_FL_e100_09B05_R_0	BY944206
E_FL_e100_09B11_F_0	BY940489

E_FL_e100_09B11_R_0	BY944207
E_FL_e100_09B12_F_0	BY940490
E_FL_e100_09B14_F_0	BY940491
E_FL_e100_09B14_R_0	BY944208
E_FL_e100_09B16_R_0	BY944209
E_FL_e100_09B17_F_0	BY940492
E_FL_e100_09B18_F_0	BY940493
E_FL_e100_09B18_R_0	BY944210
E_FL_e100_09B19_F_0	BY940494
E_FL_e100_09B22_F_0	BY940495
E_FL_e100_09B24_R_0	BY944211
E_FL_e100_09C01_F_0	BY940496
E_FL_e100_09C03_F_0	BY940497
E_FL_e100_09C03_R_0	BY944212
E_FL_e100_09C04_F_0	BY940498
E_FL_e100_09C05_F_0	BY940499
E_FL_e100_09C07_F_0	BY940500
E_FL_e100_09C08_F_0	BY940501
E_FL_e100_09C09_F_0	BY940502
E_FL_e100_09C09_R_0	BY944213
E_FL_e100_09C10_F_0	BY940503
E_FL_e100_09C13_R_0	BY944214
E_FL_e100_09C17_R_0	BY944215
E_FL_e100_09C19_F_0	BY940504
E_FL_e100_09C19_R_0	BY944216
E_FL_e100_09C20_F_0	BY940505
E_FL_e100_09C22_F_0	BY940506
E_FL_e100_09C22_R_0	BY944217
E_FL_e100_09C24_F_0	BY940507
E_FL_e100_09C24_R_0	BY944218
E_FL_e100_09D01_F_0	BY940508
E_FL_e100_09D01_R_0	BY944219
E_FL_e100_09D06_F_0	BY940509
E_FL_e100_09D06_R_0	BY944220
E_FL_e100_09D10_F_0	BY940510
E_FL_e100_09D10_R_0	BY944221
E_FL_e100_09D11_F_0	BY940511
E_FL_e100_09D12_F_0	BY940512
E_FL_e100_09D12_R_0	BY944222
E_FL_e100_09D13_F_0	BY940513
E_FL_e100_09D13_R_0	BY944223
E_FL_e100_09D15_R_0	BY944224
E_FL_e100_09D16_F_0	BY940514
E_FL_e100_09D16_R_0	BY944225
E_FL_e100_09D17_F_0	BY940515
E_FL_e100_09D17_R_0	BY944226
E_FL_e100_09D18_F_0	BY940516
E_FL_e100_09D19_F_0	BY940517
E_FL_e100_09D21_F_0	BY940518
E_FL_e100_09D21_R_0	BY944227
E_FL_e100_09D24_F_0	BY940519
E_FL_e100_09D24_R_0	BY944228
E_FL_e100_09E01_R_0	BY944229
E_FL_e100_09E04_F_0	BY940520

E_FL_e100_09E04_R_0	BY944230
E_FL_e100_09E05_F_0	BY940521
E_FL_e100_09E08_F_0	BY940522
E_FL_e100_09E08_R_0	BY944231
E_FL_e100_09E09_R_0	BY944232
E_FL_e100_09E11_F_0	BY940523
E_FL_e100_09E11_R_0	BY944233
E_FL_e100_09E13_F_0	BY940524
E_FL_e100_09E13_R_0	BY944234
E_FL_e100_09E14_F_0	BY940525
E_FL_e100_09E14_R_0	BY944235
E_FL_e100_09E15_F_0	BY940526
E_FL_e100_09E15_R_0	BY944236
E_FL_e100_09E19_F_0	BY940527
E_FL_e100_09E19_R_0	BY944237
E_FL_e100_09E20_F_0	BY940528
E_FL_e100_09E21_R_0	BY944238
E_FL_e100_09E22_F_0	BY940529
E_FL_e100_09F02_F_0	BY940530
E_FL_e100_09F03_F_0	BY940531
E_FL_e100_09F03_R_0	BY944239
E_FL_e100_09F04_F_0	BY940532
E_FL_e100_09F04_R_0	BY944240
E_FL_e100_09F05_F_0	BY940533
E_FL_e100_09F05_R_0	BY944241
E_FL_e100_09F09_F_0	BY940534
E_FL_e100_09F09_R_0	BY944242
E_FL_e100_09F13_R_0	BY944243
E_FL_e100_09F14_F_0	BY940535
E_FL_e100_09F14_R_0	BY944244
E_FL_e100_09F15_F_0	BY940536
E_FL_e100_09F15_R_0	BY944245
E_FL_e100_09F17_F_0	BY940537
E_FL_e100_09F20_F_0	BY940538
E_FL_e100_09F23_F_0	BY940539
E_FL_e100_09F23_R_0	BY944246
E_FL_e100_09G01_F_0	BY940540
E_FL_e100_09G02_F_0	BY940541
E_FL_e100_09G03_F_0	BY940542
E_FL_e100_09G04_F_0	BY940543
E_FL_e100_09G04_R_0	BY944247
E_FL_e100_09G06_F_0	BY940544
E_FL_e100_09G07_F_0	BY940545
E_FL_e100_09G08_R_0	BY944248
E_FL_e100_09G09_F_0	BY940546
E_FL_e100_09G10_F_0	BY940547
E_FL_e100_09G13_F_0	BY940548
E_FL_e100_09G14_F_0	BY940549
E_FL_e100_09G16_F_0	BY940550
E_FL_e100_09G16_R_0	BY944249
E_FL_e100_09G17_F_0	BY940551
E_FL_e100_09G20_F_0	BY940552
E_FL_e100_09G22_F_0	BY940553
E_FL_e100_09G24_F_0	BY940554

E_FL_e100_09G24_R_0	BY944250
E_FL_e100_09H02_F_0	BY940555
E_FL_e100_09H02_R_0	BY944251
E_FL_e100_09H03_R_0	BY944252
E_FL_e100_09H05_F_0	BY940556
E_FL_e100_09H07_F_0	BY940557
E_FL_e100_09H09_F_0	BY940558
E_FL_e100_09H10_F_0	BY940559
E_FL_e100_09H12_F_0	BY940560
E_FL_e100_09H14_F_0	BY940561
E_FL_e100_09H14_R_0	BY944253
E_FL_e100_09H17_F_0	BY940562
E_FL_e100_09H20_F_0	BY940563
E_FL_e100_09H23_F_0	BY940564
E_FL_e100_09H24_F_0	BY940565
E_FL_e100_09I01_F_0	BY940566
E_FL_e100_09I03_F_0	BY940567
E_FL_e100_09I05_F_0	BY940568
E_FL_e100_09I06_F_0	BY940569
E_FL_e100_09I09_F_0	BY940570
E_FL_e100_09I10_F_0	BY940571
E_FL_e100_09I10_R_0	BY944254
E_FL_e100_09I13_F_0	BY940572
E_FL_e100_09I13_R_0	BY944255
E_FL_e100_09I14_F_0	BY940573
E_FL_e100_09I16_F_0	BY940574
E_FL_e100_09I18_F_0	BY940575
E_FL_e100_09I19_F_0	BY940576
E_FL_e100_09I22_F_0	BY940577
E_FL_e100_09I22_R_0	BY944256
E_FL_e100_09I23_F_0	BY940578
E_FL_e100_09I23_R_0	BY944257
E_FL_e100_09J01_F_0	BY940579
E_FL_e100_09J01_R_0	BY944258
E_FL_e100_09J02_F_0	BY940580
E_FL_e100_09J04_F_0	BY940581
E_FL_e100_09J04_R_0	BY944259
E_FL_e100_09J05_F_0	BY940582
E_FL_e100_09J05_R_0	BY944260
E_FL_e100_09J06_F_0	BY940583
E_FL_e100_09J08_F_0	BY940584
E_FL_e100_09J10_F_0	BY940585
E_FL_e100_09J10_R_0	BY944261
E_FL_e100_09J12_F_0	BY940586
E_FL_e100_09J12_R_0	BY944262
E_FL_e100_09J18_F_0	BY940587
E_FL_e100_09J20_F_0	BY940588
E_FL_e100_09J20_R_0	BY944263
E_FL_e100_09J21_F_0	BY940589
E_FL_e100_09J21_R_0	BY944264
E_FL_e100_09J22_F_0	BY940590
E_FL_e100_09J22_R_0	BY944265
E_FL_e100_09J23_F_0	BY940591
E_FL_e100_09J23_R_0	BY944266

E_FL_e100_09J24_F_0	BY940592
E_FL_e100_09J24_R_0	BY944267
E_FL_e100_09K02_F_0	BY940593
E_FL_e100_09K03_F_0	BY940594
E_FL_e100_09K03_R_0	BY944268
E_FL_e100_09K04_F_0	BY940595
E_FL_e100_09K04_R_0	BY944269
E_FL_e100_09K05_F_0	BY940596
E_FL_e100_09K05_R_0	BY944270
E_FL_e100_09K07_F_0	BY940597
E_FL_e100_09K09_F_0	BY940598
E_FL_e100_09K09_R_0	BY944271
E_FL_e100_09K14_F_0	BY940599
E_FL_e100_09K15_F_0	BY940600
E_FL_e100_09K16_R_0	BY944272
E_FL_e100_09K22_R_0	BY944273
E_FL_e100_09K23_F_0	BY940601
E_FL_e100_09K24_F_0	BY940602
E_FL_e100_09L01_F_0	BY940603
E_FL_e100_09L01_R_0	BY944274
E_FL_e100_09L02_F_0	BY940604
E_FL_e100_09L04_R_0	BY944275
E_FL_e100_09L06_F_0	BY940605
E_FL_e100_09L07_F_0	BY940606
E_FL_e100_09L07_R_0	BY944276
E_FL_e100_09L09_F_0	BY940607
E_FL_e100_09L09_R_0	BY944277
E_FL_e100_09L10_F_0	BY940608
E_FL_e100_09L13_R_0	BY944278
E_FL_e100_09L15_F_0	BY940609
E_FL_e100_09L16_F_0	BY940610
E_FL_e100_09L17_F_0	BY940611
E_FL_e100_09L17_R_0	BY944279
E_FL_e100_09L18_F_0	BY940612
E_FL_e100_09L19_F_0	BY940613
E_FL_e100_09L19_R_0	BY944280
E_FL_e100_09L21_F_0	BY940614
E_FL_e100_09L21_R_0	BY944281
E_FL_e100_09L22_F_0	BY940615
E_FL_e100_09L22_R_0	BY944282
E_FL_e100_09L23_F_0	BY940616
E_FL_e100_09L23_R_0	BY944283
E_FL_e100_09M02_F_0	BY940617
E_FL_e100_09M04_F_0	BY940618
E_FL_e100_09M04_R_0	BY944284
E_FL_e100_09M05_F_0	BY940619
E_FL_e100_09M05_R_0	BY944285
E_FL_e100_09M07_F_0	BY940620
E_FL_e100_09M07_R_0	BY944286
E_FL_e100_09M09_F_0	BY940621
E_FL_e100_09M10_F_0	BY940622
E_FL_e100_09M11_F_0	BY940623
E_FL_e100_09M12_F_0	BY940624
E_FL_e100_09M12_R_0	BY944287

E_FL_e100_09M13_R_0	BY944288
E_FL_e100_09M15_F_0	BY940625
E_FL_e100_09M15_R_0	BY944289
E_FL_e100_09M16_F_0	BY940626
E_FL_e100_09M16_R_0	BY944290
E_FL_e100_09M19_F_0	BY940627
E_FL_e100_09M21_F_0	BY940628
E_FL_e100_09M21_R_0	BY944291
E_FL_e100_09N02_F_0	BY940629
E_FL_e100_09N02_R_0	BY944292
E_FL_e100_09N05_F_0	BY940630
E_FL_e100_09N08_F_0	BY940631
E_FL_e100_09N08_R_0	BY944293
E_FL_e100_09N12_F_0	BY940632
E_FL_e100_09N13_F_0	BY940633
E_FL_e100_09N16_R_0	BY944294
E_FL_e100_09N17_R_0	BY944295
E_FL_e100_09N19_F_0	BY940634
E_FL_e100_09N22_F_0	BY940635
E_FL_e100_09N23_F_0	BY940636
E_FL_e100_09O01_F_0	BY940637
E_FL_e100_09O01_R_0	BY944296
E_FL_e100_09O03_F_0	BY940638
E_FL_e100_09O04_F_0	BY940639
E_FL_e100_09O04_R_0	BY944297
E_FL_e100_09O05_F_0	BY940640
E_FL_e100_09O05_R_0	BY944298
E_FL_e100_09O06_F_0	BY940641
E_FL_e100_09O09_F_0	BY940642
E_FL_e100_09O10_F_0	BY940643
E_FL_e100_09O10_R_0	BY944299
E_FL_e100_09O15_F_0	BY940644
E_FL_e100_09O15_R_0	BY944300
E_FL_e100_09O16_F_0	BY940645
E_FL_e100_09O17_F_0	BY940646
E_FL_e100_09O19_F_0	BY940647
E_FL_e100_09O23_F_0	BY940648
E_FL_e100_09O24_F_0	BY940649
E_FL_e100_09P01_R_0	BY944301
E_FL_e100_09P02_F_0	BY940650
E_FL_e100_09P04_F_0	BY940651
E_FL_e100_09P04_R_0	BY944302
E_FL_e100_09P09_F_0	BY940652
E_FL_e100_09P13_R_0	BY944303
E_FL_e100_09P14_F_0	BY940653
E_FL_e100_09P15_F_0	BY940654
E_FL_e100_09P15_R_0	BY944304
E_FL_e100_09P21_R_0	BY944305
E_FL_e100_09P23_F_0	BY940655
E_FL_e100_10A03_F_0	BY940656
E_FL_e100_10A04_R_0	BY944306
E_FL_e100_10A05_F_0	BY940657
E_FL_e100_10A08_F_0	BY940658
E_FL_e100_10A09_F_0	BY940659

E_FL_e100_10A09_R_0	BY944307
E_FL_e100_10A11_F_0	BY940660
E_FL_e100_10A12_F_0	BY940661
E_FL_e100_10A12_R_0	BY944308
E_FL_e100_10A13_F_0	BY940662
E_FL_e100_10A14_F_0	BY940663
E_FL_e100_10A14_R_0	BY944309
E_FL_e100_10A15_F_0	BY940664
E_FL_e100_10A16_F_0	BY940665
E_FL_e100_10A17_F_0	BY940666
E_FL_e100_10A19_F_0	BY940667
E_FL_e100_10A20_F_0	BY940668
E_FL_e100_10A23_F_0	BY940669
E_FL_e100_10A23_R_0	BY944310
E_FL_e100_10B02_F_0	BY940670
E_FL_e100_10B05_F_0	BY940671
E_FL_e100_10B06_F_0	BY940672
E_FL_e100_10B06_R_0	BY944311
E_FL_e100_10B08_F_0	BY940673
E_FL_e100_10B10_F_0	BY940674
E_FL_e100_10B10_R_0	BY944312
E_FL_e100_10B11_F_0	BY940675
E_FL_e100_10B12_F_0	BY940676
E_FL_e100_10B12_R_0	BY944313
E_FL_e100_10B13_R_0	BY944314
E_FL_e100_10B14_F_0	BY940677
E_FL_e100_10B15_F_0	BY940678
E_FL_e100_10B15_R_0	BY944315
E_FL_e100_10B18_F_0	BY940679
E_FL_e100_10B18_R_0	BY944316
E_FL_e100_10B21_F_0	BY940680
E_FL_e100_10B21_R_0	BY944317
E_FL_e100_10C01_F_0	BY940681
E_FL_e100_10C02_F_0	BY940682
E_FL_e100_10C04_F_0	BY940683
E_FL_e100_10C09_R_0	BY944318
E_FL_e100_10C10_F_0	BY940684
E_FL_e100_10C10_R_0	BY944319
E_FL_e100_10C13_F_0	BY940685
E_FL_e100_10C13_R_0	BY944320
E_FL_e100_10C20_F_0	BY940686
E_FL_e100_10C21_F_0	BY940687
E_FL_e100_10C21_R_0	BY944321
E_FL_e100_10C22_F_0	BY940688
E_FL_e100_10D02_F_0	BY940689
E_FL_e100_10D04_F_0	BY940690
E_FL_e100_10D04_R_0	BY944322
E_FL_e100_10D06_F_0	BY940691
E_FL_e100_10D08_F_0	BY940692
E_FL_e100_10D09_F_0	BY940693
E_FL_e100_10D13_F_0	BY940694
E_FL_e100_10D13_R_0	BY944323
E_FL_e100_10D15_F_0	BY940695
E_FL_e100_10D15_R_0	BY944324

E_FL_e100_10D16_F_0	BY940696
E_FL_e100_10D19_F_0	BY940697
E_FL_e100_10D20_F_0	BY940698
E_FL_e100_10D23_F_0	BY940699
E_FL_e100_10D24_F_0	BY940700
E_FL_e100_10D24_R_0	BY944325
E_FL_e100_10E02_F_0	BY940701
E_FL_e100_10E02_R_0	BY944326
E_FL_e100_10E03_F_0	BY940702
E_FL_e100_10E03_R_0	BY944327
E_FL_e100_10E05_F_0	BY940703
E_FL_e100_10E11_F_0	BY940704
E_FL_e100_10E12_F_0	BY940705
E_FL_e100_10E13_F_0	BY940706
E_FL_e100_10E13_R_0	BY944328
E_FL_e100_10E16_F_0	BY940707
E_FL_e100_10E17_F_0	BY940708
E_FL_e100_10E17_R_0	BY944329
E_FL_e100_10E19_F_0	BY940709
E_FL_e100_10E22_F_0	BY940710
E_FL_e100_10E22_R_0	BY944330
E_FL_e100_10E24_F_0	BY940711
E_FL_e100_10F01_F_0	BY940712
E_FL_e100_10F02_F_0	BY940713
E_FL_e100_10F03_F_0	BY940714
E_FL_e100_10F04_F_0	BY940715
E_FL_e100_10F05_F_0	BY940716
E_FL_e100_10F07_F_0	BY940717
E_FL_e100_10F08_F_0	BY940718
E_FL_e100_10F08_R_0	BY944331
E_FL_e100_10F09_F_0	BY940719
E_FL_e100_10F09_R_0	BY944332
E_FL_e100_10F11_F_0	BY940720
E_FL_e100_10F11_R_0	BY944333
E_FL_e100_10F13_F_0	BY940721
E_FL_e100_10F13_R_0	BY944334
E_FL_e100_10F14_F_0	BY940722
E_FL_e100_10F14_R_0	BY944335
E_FL_e100_10F15_F_0	BY940723
E_FL_e100_10F15_R_0	BY944336
E_FL_e100_10F22_F_0	BY940724
E_FL_e100_10F22_R_0	BY944337
E_FL_e100_10F23_F_0	BY940725
E_FL_e100_10F24_F_0	BY940726
E_FL_e100_10G01_F_0	BY940727
E_FL_e100_10G01_R_0	BY944338
E_FL_e100_10G07_F_0	BY940728
E_FL_e100_10G07_R_0	BY944339
E_FL_e100_10G08_F_0	BY940729
E_FL_e100_10G11_F_0	BY940730
E_FL_e100_10G13_F_0	BY940731
E_FL_e100_10G13_R_0	BY944340
E_FL_e100_10G15_F_0	BY940732
E_FL_e100_10G16_F_0	BY940733

E_FL_e100_10G17_F_0	BY940734
E_FL_e100_10G18_F_0	BY940735
E_FL_e100_10G19_F_0	BY940736
E_FL_e100_10G19_R_0	BY944341
E_FL_e100_10G20_F_0	BY940737
E_FL_e100_10G21_F_0	BY940738
E_FL_e100_10G21_R_0	BY944342
E_FL_e100_10G23_F_0	BY940739
E_FL_e100_10G23_R_0	BY944343
E_FL_e100_10H01_R_0	BY944344
E_FL_e100_10H02_F_0	BY940740
E_FL_e100_10H02_R_0	BY944345
E_FL_e100_10H03_F_0	BY940741
E_FL_e100_10H04_F_0	BY940742
E_FL_e100_10H04_R_0	BY944346
E_FL_e100_10H06_F_0	BY940743
E_FL_e100_10H06_R_0	BY944347
E_FL_e100_10H08_F_0	BY940744
E_FL_e100_10H08_R_0	BY944348
E_FL_e100_10H09_F_0	BY940745
E_FL_e100_10H10_R_0	BY944349
E_FL_e100_10H12_F_0	BY940746
E_FL_e100_10H12_R_0	BY944350
E_FL_e100_10H13_F_0	BY940747
E_FL_e100_10H14_F_0	BY940748
E_FL_e100_10H14_R_0	BY944351
E_FL_e100_10H15_F_0	BY940749
E_FL_e100_10H15_R_0	BY944352
E_FL_e100_10H16_F_0	BY940750
E_FL_e100_10H16_R_0	BY944353
E_FL_e100_10H17_F_0	BY940751
E_FL_e100_10H17_R_0	BY944354
E_FL_e100_10H18_F_0	BY940752
E_FL_e100_10H19_F_0	BY940753
E_FL_e100_10H21_F_0	BY940754
E_FL_e100_10H22_F_0	BY940755
E_FL_e100_10H22_R_0	BY944355
E_FL_e100_10H23_F_0	BY940756
E_FL_e100_10H24_F_0	BY940757
E_FL_e100_10H24_R_0	BY944356
E_FL_e100_10I01_F_0	BY940758
E_FL_e100_10I04_F_0	BY940759
E_FL_e100_10I04_R_0	BY944357
E_FL_e100_10I07_F_0	BY940760
E_FL_e100_10I10_F_0	BY940761
E_FL_e100_10I12_F_0	BY940762
E_FL_e100_10I12_R_0	BY944358
E_FL_e100_10I13_R_0	BY944359
E_FL_e100_10I14_F_0	BY940763
E_FL_e100_10I15_R_0	BY944360
E_FL_e100_10I18_F_0	BY940764
E_FL_e100_10I19_R_0	BY944361
E_FL_e100_10I22_F_0	BY940765
E_FL_e100_10I22_R_0	BY944362

E_FL_e100_10I23_F_0	BY940766
E_FL_e100_10I23_R_0	BY944363
E_FL_e100_10J01_F_0	BY940767
E_FL_e100_10J01_R_0	BY944364
E_FL_e100_10J02_F_0	BY940768
E_FL_e100_10J02_R_0	BY944365
E_FL_e100_10J03_F_0	BY940769
E_FL_e100_10J04_F_0	BY940770
E_FL_e100_10J04_R_0	BY944366
E_FL_e100_10J06_F_0	BY940771
E_FL_e100_10J08_F_0	BY940772
E_FL_e100_10J11_F_0	BY940773
E_FL_e100_10J12_F_0	BY940774
E_FL_e100_10J12_R_0	BY944367
E_FL_e100_10J13_F_0	BY940775
E_FL_e100_10J13_R_0	BY944368
E_FL_e100_10J18_F_0	BY940776
E_FL_e100_10J18_R_0	BY944369
E_FL_e100_10J20_F_0	BY940777
E_FL_e100_10J20_R_0	BY944370
E_FL_e100_10J21_R_0	BY944371
E_FL_e100_10J22_F_0	BY940778
E_FL_e100_10J23_F_0	BY940779
E_FL_e100_10J23_R_0	BY944372
E_FL_e100_10K01_F_0	BY940780
E_FL_e100_10K07_F_0	BY940781
E_FL_e100_10K07_R_0	BY944373
E_FL_e100_10K09_F_0	BY940782
E_FL_e100_10K09_R_0	BY944374
E_FL_e100_10K13_F_0	BY940783
E_FL_e100_10K14_F_0	BY940784
E_FL_e100_10K18_F_0	BY940785
E_FL_e100_10K19_F_0	BY940786
E_FL_e100_10K19_R_0	BY944375
E_FL_e100_10K21_F_0	BY940787
E_FL_e100_10K24_F_0	BY940788
E_FL_e100_10L04_F_0	BY940789
E_FL_e100_10L06_F_0	BY940790
E_FL_e100_10L07_F_0	BY940791
E_FL_e100_10L07_R_0	BY944376
E_FL_e100_10L09_F_0	BY940792
E_FL_e100_10L11_F_0	BY940793
E_FL_e100_10L11_R_0	BY944377
E_FL_e100_10L13_F_0	BY940794
E_FL_e100_10L16_F_0	BY940795
E_FL_e100_10L16_R_0	BY944378
E_FL_e100_10L21_F_0	BY940796
E_FL_e100_10L22_F_0	BY940797
E_FL_e100_10L22_R_0	BY944379
E_FL_e100_10L23_F_0	BY940798
E_FL_e100_10L24_F_0	BY940799
E_FL_e100_10M01_F_0	BY940800
E_FL_e100_10M08_R_0	BY944380
E_FL_e100_10M09_F_0	BY940801

E_FL_e100_10M09_R_0	BY944381
E_FL_e100_10M11_F_0	BY940802
E_FL_e100_10M11_R_0	BY944382
E_FL_e100_10M13_F_0	BY940803
E_FL_e100_10M15_F_0	BY940804
E_FL_e100_10M15_R_0	BY944383
E_FL_e100_10M16_F_0	BY940805
E_FL_e100_10M16_R_0	BY944384
E_FL_e100_10M18_F_0	BY940806
E_FL_e100_10M19_F_0	BY940807
E_FL_e100_10M19_R_0	BY944385
E_FL_e100_10M20_F_0	BY940808
E_FL_e100_10M20_R_0	BY944386
E_FL_e100_10M21_F_0	BY940809
E_FL_e100_10M22_F_0	BY940810
E_FL_e100_10M22_R_0	BY944387
E_FL_e100_10N03_F_0	BY940811
E_FL_e100_10N03_R_0	BY944388
E_FL_e100_10N04_F_0	BY940812
E_FL_e100_10N08_F_0	BY940813
E_FL_e100_10N08_R_0	BY944389
E_FL_e100_10N09_F_0	BY940814
E_FL_e100_10N10_R_0	BY944390
E_FL_e100_10N11_F_0	BY940815
E_FL_e100_10N11_R_0	BY944391
E_FL_e100_10N14_F_0	BY940816
E_FL_e100_10N18_F_0	BY940817
E_FL_e100_10N19_F_0	BY940818
E_FL_e100_10N19_R_0	BY944392
E_FL_e100_10N21_F_0	BY940819
E_FL_e100_10N22_F_0	BY940820
E_FL_e100_10N22_R_0	BY944393
E_FL_e100_10N24_F_0	BY940821
E_FL_e100_10O02_F_0	BY940822
E_FL_e100_10O05_F_0	BY940823
E_FL_e100_10O05_R_0	BY944394
E_FL_e100_10O06_F_0	BY940824
E_FL_e100_10O06_R_0	BY944395
E_FL_e100_10O07_F_0	BY940825
E_FL_e100_10O07_R_0	BY944396
E_FL_e100_10O09_R_0	BY944397
E_FL_e100_10O13_F_0	BY940826
E_FL_e100_10O13_R_0	BY944398
E_FL_e100_10O14_F_0	BY940827
E_FL_e100_10O15_F_0	BY940828
E_FL_e100_10O15_R_0	BY944399
E_FL_e100_10O16_F_0	BY940829
E_FL_e100_10O16_R_0	BY944400
E_FL_e100_10O17_F_0	BY940830
E_FL_e100_10O18_F_0	BY940831
E_FL_e100_10O19_R_0	BY944401
E_FL_e100_10O20_F_0	BY940832
E_FL_e100_10O20_R_0	BY944402
E_FL_e100_10O22_F_0	BY940833

E_FL_e100_10O22_R_0	BY944403
E_FL_e100_10O24_F_0	BY940834
E_FL_e100_10P01_F_0	BY940835
E_FL_e100_10P02_F_0	BY940836
E_FL_e100_10P03_F_0	BY940837
E_FL_e100_10P04_F_0	BY940838
E_FL_e100_10P04_R_0	BY944404
E_FL_e100_10P07_F_0	BY940839
E_FL_e100_10P10_F_0	BY940840
E_FL_e100_10P11_F_0	BY940841
E_FL_e100_10P11_R_0	BY944405
E_FL_e100_10P14_F_0	BY940842
E_FL_e100_10P14_R_0	BY944406
E_FL_e100_10P19_F_0	BY940843
E_FL_e100_10P19_R_0	BY944407
E_FL_e100_10P20_F_0	BY940844
E_FL_e100_10P20_R_0	BY944408
E_FL_e100_10P21_F_0	BY940845
E_FL_e100_10P21_R_0	BY944409
E_FL_e100_10P22_F_0	BY940846
E_FL_e100_11A04_R_0	BY944410
E_FL_e100_11A06_F_0	BY940847
E_FL_e100_11A07_F_0	BY940848
E_FL_e100_11A07_R_0	BY944411
E_FL_e100_11A14_F_0	BY940849
E_FL_e100_11A15_F_0	BY940850
E_FL_e100_11A18_F_0	BY940851
E_FL_e100_11A20_F_0	BY940852
E_FL_e100_11A20_R_0	BY944412
E_FL_e100_11A21_R_0	BY944413
E_FL_e100_11A22_R_0	BY944414
E_FL_e100_11A23_R_0	BY944415
E_FL_e100_11A24_F_0	BY940853
E_FL_e100_11A24_R_0	BY944416
E_FL_e100_11B06_F_0	BY940854
E_FL_e100_11B06_R_0	BY944417
E_FL_e100_11B07_R_0	BY944418
E_FL_e100_11B09_F_0	BY940855
E_FL_e100_11B10_F_0	BY940856
E_FL_e100_11B10_R_0	BY944419
E_FL_e100_11B11_F_0	BY940857
E_FL_e100_11B12_F_0	BY940858
E_FL_e100_11B12_R_0	BY944420
E_FL_e100_11B15_F_0	BY940859
E_FL_e100_11B17_F_0	BY940860
E_FL_e100_11B18_F_0	BY940861
E_FL_e100_11B18_R_0	BY944421
E_FL_e100_11B19_F_0	BY940862
E_FL_e100_11B23_F_0	BY940863
E_FL_e100_11B23_R_0	BY944422
E_FL_e100_11C02_F_0	BY940864
E_FL_e100_11C05_F_0	BY940865
E_FL_e100_11C05_R_0	BY944423
E_FL_e100_11C08_F_0	BY940866

E_FL_e100_11C12_F_0	BY940867
E_FL_e100_11C13_F_0	BY940868
E_FL_e100_11C15_F_0	BY940869
E_FL_e100_11C15_R_0	BY944424
E_FL_e100_11C16_F_0	BY940870
E_FL_e100_11C16_R_0	BY944425
E_FL_e100_11C19_F_0	BY940871
E_FL_e100_11C20_R_0	BY944426
E_FL_e100_11C22_F_0	BY940872
E_FL_e100_11C22_R_0	BY944427
E_FL_e100_11D02_F_0	BY940873
E_FL_e100_11D02_R_0	BY944428
E_FL_e100_11D04_F_0	BY940874
E_FL_e100_11D04_R_0	BY944429
E_FL_e100_11D05_F_0	BY940875
E_FL_e100_11D05_R_0	BY944430
E_FL_e100_11D06_F_0	BY940876
E_FL_e100_11D07_F_0	BY940877
E_FL_e100_11D08_F_0	BY940878
E_FL_e100_11D08_R_0	BY944431
E_FL_e100_11D09_F_0	BY940879
E_FL_e100_11D09_R_0	BY944432
E_FL_e100_11D10_F_0	BY940880
E_FL_e100_11D12_F_0	BY940881
E_FL_e100_11D13_F_0	BY940882
E_FL_e100_11D13_R_0	BY944433
E_FL_e100_11D15_F_0	BY940883
E_FL_e100_11D15_R_0	BY944434
E_FL_e100_11D16_F_0	BY940884
E_FL_e100_11D16_R_0	BY944435
E_FL_e100_11D17_F_0	BY940885
E_FL_e100_11D18_F_0	BY940886
E_FL_e100_11D18_R_0	BY944436
E_FL_e100_11D21_F_0	BY940887
E_FL_e100_11D21_R_0	BY944437
E_FL_e100_11D22_F_0	BY940888
E_FL_e100_11D23_F_0	BY940889
E_FL_e100_11D23_R_0	BY944438
E_FL_e100_11E02_F_0	BY940890
E_FL_e100_11E03_R_0	BY944439
E_FL_e100_11E04_F_0	BY940891
E_FL_e100_11E06_F_0	BY940892
E_FL_e100_11E06_R_0	BY944440
E_FL_e100_11E10_F_0	BY940893
E_FL_e100_11E13_F_0	BY940894
E_FL_e100_11E16_F_0	BY940895
E_FL_e100_11E17_F_0	BY940896
E_FL_e100_11E17_R_0	BY944441
E_FL_e100_11E19_F_0	BY940897
E_FL_e100_11E21_F_0	BY940898
E_FL_e100_11E21_R_0	BY944442
E_FL_e100_11E23_F_0	BY940899
E_FL_e100_11E23_R_0	BY944443
E_FL_e100_11F02_F_0	BY940900

E_FL_e100_11F03_F_0	BY940901
E_FL_e100_11F05_F_0	BY940902
E_FL_e100_11F06_F_0	BY940903
E_FL_e100_11F06_R_0	BY944444
E_FL_e100_11F07_F_0	BY940904
E_FL_e100_11F08_F_0	BY940905
E_FL_e100_11F09_F_0	BY940906
E_FL_e100_11F09_R_0	BY944445
E_FL_e100_11F10_F_0	BY940907
E_FL_e100_11F11_F_0	BY940908
E_FL_e100_11F12_F_0	BY940909
E_FL_e100_11F15_R_0	BY944446
E_FL_e100_11F16_F_0	BY940910
E_FL_e100_11F16_R_0	BY944447
E_FL_e100_11F17_F_0	BY940911
E_FL_e100_11F18_F_0	BY940912
E_FL_e100_11F18_R_0	BY944448
E_FL_e100_11F19_F_0	BY940913
E_FL_e100_11G02_F_0	BY940914
E_FL_e100_11G04_F_0	BY940915
E_FL_e100_11G09_F_0	BY940916
E_FL_e100_11G10_F_0	BY940917
E_FL_e100_11G13_F_0	BY940918
E_FL_e100_11G16_F_0	BY940919
E_FL_e100_11G16_R_0	BY944449
E_FL_e100_11G18_F_0	BY940920
E_FL_e100_11G19_F_0	BY940921
E_FL_e100_11G22_F_0	BY940922
E_FL_e100_11G22_R_0	BY944450
E_FL_e100_11G23_F_0	BY940923
E_FL_e100_11G24_F_0	BY940924
E_FL_e100_11H01_F_0	BY940925
E_FL_e100_11H03_F_0	BY940926
E_FL_e100_11H03_R_0	BY944451
E_FL_e100_11H06_F_0	BY940927
E_FL_e100_11H06_R_0	BY944452
E_FL_e100_11H09_F_0	BY940928
E_FL_e100_11H09_R_0	BY944453
E_FL_e100_11H13_F_0	BY940929
E_FL_e100_11H13_R_0	BY944454
E_FL_e100_11H14_F_0	BY940930
E_FL_e100_11H14_R_0	BY944455
E_FL_e100_11H15_F_0	BY940931
E_FL_e100_11H15_R_0	BY944456
E_FL_e100_11H16_F_0	BY940932
E_FL_e100_11H16_R_0	BY944457
E_FL_e100_11H17_F_0	BY940933
E_FL_e100_11H17_R_0	BY944458
E_FL_e100_11H18_F_0	BY940934
E_FL_e100_11H21_F_0	BY940935
E_FL_e100_11H21_R_0	BY944459
E_FL_e100_11H22_F_0	BY940936
E_FL_e100_11H22_R_0	BY944460
E_FL_e100_11I01_R_0	BY944461

E_FL_e100_11I04_F_0	BY940937
E_FL_e100_11I06_F_0	BY940938
E_FL_e100_11I07_F_0	BY940939
E_FL_e100_11I10_F_0	BY940940
E_FL_e100_11I14_F_0	BY940941
E_FL_e100_11I16_F_0	BY940942
E_FL_e100_11I16_R_0	BY944462
E_FL_e100_11I18_F_0	BY940943
E_FL_e100_11I23_F_0	BY940944
E_FL_e100_11I24_F_0	BY940945
E_FL_e100_11J02_F_0	BY940946
E_FL_e100_11J05_F_0	BY940947
E_FL_e100_11J06_F_0	BY940948
E_FL_e100_11J07_F_0	BY940949
E_FL_e100_11J09_F_0	BY940950
E_FL_e100_11J09_R_0	BY944463
E_FL_e100_11J10_F_0	BY940951
E_FL_e100_11J11_F_0	BY940952
E_FL_e100_11J11_R_0	BY944464
E_FL_e100_11J12_F_0	BY940953
E_FL_e100_11J14_F_0	BY940954
E_FL_e100_11J15_F_0	BY940955
E_FL_e100_11J15_R_0	BY944465
E_FL_e100_11J16_F_0	BY940956
E_FL_e100_11J17_R_0	BY944466
E_FL_e100_11J18_F_0	BY940957
E_FL_e100_11J19_F_0	BY940958
E_FL_e100_11J20_F_0	BY940959
E_FL_e100_11J21_R_0	BY944467
E_FL_e100_11J22_F_0	BY940960
E_FL_e100_11J23_F_0	BY940961
E_FL_e100_11J23_R_0	BY944468
E_FL_e100_11J24_F_0	BY940962
E_FL_e100_11K01_F_0	BY940963
E_FL_e100_11K01_R_0	BY944469
E_FL_e100_11K02_R_0	BY944470
E_FL_e100_11K03_F_0	BY940964
E_FL_e100_11K03_R_0	BY944471
E_FL_e100_11K04_F_0	BY940965
E_FL_e100_11K04_R_0	BY944472
E_FL_e100_11K05_F_0	BY940966
E_FL_e100_11K07_F_0	BY940967
E_FL_e100_11K09_F_0	BY940968
E_FL_e100_11K09_R_0	BY944473
E_FL_e100_11K11_R_0	BY944474
E_FL_e100_11K12_F_0	BY940969
E_FL_e100_11K12_R_0	BY944475
E_FL_e100_11K14_F_0	BY940970
E_FL_e100_11K16_F_0	BY940971
E_FL_e100_11K21_R_0	BY944476
E_FL_e100_11K24_F_0	BY940972
E_FL_e100_11L02_F_0	BY940973
E_FL_e100_11L04_F_0	BY940974
E_FL_e100_11L04_R_0	BY944477

E_FL_e100_11L07_F_0	BY940975
E_FL_e100_11L08_R_0	BY944478
E_FL_e100_11L09_F_0	BY940976
E_FL_e100_11L09_R_0	BY944479
E_FL_e100_11L13_F_0	BY940977
E_FL_e100_11L13_R_0	BY944480
E_FL_e100_11L14_F_0	BY940978
E_FL_e100_11L14_R_0	BY944481
E_FL_e100_11L15_F_0	BY940979
E_FL_e100_11L17_F_0	BY940980
E_FL_e100_11L18_F_0	BY940981
E_FL_e100_11L19_F_0	BY940982
E_FL_e100_11L19_R_0	BY944482
E_FL_e100_11L21_F_0	BY940983
E_FL_e100_11L21_R_0	BY944483
E_FL_e100_11L23_F_0	BY940984
E_FL_e100_11M02_F_0	BY940985
E_FL_e100_11M03_R_0	BY944484
E_FL_e100_11M04_F_0	BY940986
E_FL_e100_11M04_R_0	BY944485
E_FL_e100_11M05_F_0	BY940987
E_FL_e100_11M06_F_0	BY940988
E_FL_e100_11M10_F_0	BY940989
E_FL_e100_11M10_R_0	BY944486
E_FL_e100_11M11_F_0	BY940990
E_FL_e100_11M13_F_0	BY940991
E_FL_e100_11M14_F_0	BY940992
E_FL_e100_11M14_R_0	BY944487
E_FL_e100_11M16_F_0	BY940993
E_FL_e100_11M17_F_0	BY940994
E_FL_e100_11M18_F_0	BY940995
E_FL_e100_11M18_R_0	BY944488
E_FL_e100_11M20_F_0	BY940996
E_FL_e100_11M20_R_0	BY944489
E_FL_e100_11M21_F_0	BY940997
E_FL_e100_11M22_F_0	BY940998
E_FL_e100_11M22_R_0	BY944490
E_FL_e100_11M24_F_0	BY940999
E_FL_e100_11N02_F_0	BY941000
E_FL_e100_11N02_R_0	BY944491
E_FL_e100_11N03_F_0	BY941001
E_FL_e100_11N03_R_0	BY944492
E_FL_e100_11N04_F_0	BY941002
E_FL_e100_11N05_F_0	BY941003
E_FL_e100_11N05_R_0	BY944493
E_FL_e100_11N06_F_0	BY941004
E_FL_e100_11N06_R_0	BY944494
E_FL_e100_11N08_F_0	BY941005
E_FL_e100_11N10_F_0	BY941006
E_FL_e100_11N11_F_0	BY941007
E_FL_e100_11N12_F_0	BY941008
E_FL_e100_11N13_F_0	BY941009
E_FL_e100_11N13_R_0	BY944495
E_FL_e100_11N16_F_0	BY941010

E_FL_e100_11N16_R_0	BY944496
E_FL_e100_11N17_F_0	BY941011
E_FL_e100_11N17_R_0	BY944497
E_FL_e100_11N18_F_0	BY941012
E_FL_e100_11N19_F_0	BY941013
E_FL_e100_11N20_F_0	BY941014
E_FL_e100_11N20_R_0	BY944498
E_FL_e100_11N21_F_0	BY941015
E_FL_e100_11N21_R_0	BY944499
E_FL_e100_11N24_F_0	BY941016
E_FL_e100_11O01_F_0	BY941017
E_FL_e100_11O02_F_0	BY941018
E_FL_e100_11O03_F_0	BY941019
E_FL_e100_11O03_R_0	BY944500
E_FL_e100_11O04_F_0	BY941020
E_FL_e100_11O04_R_0	BY944501
E_FL_e100_11O05_F_0	BY941021
E_FL_e100_11O06_F_0	BY941022
E_FL_e100_11O06_R_0	BY944502
E_FL_e100_11O07_F_0	BY941023
E_FL_e100_11O07_R_0	BY944503
E_FL_e100_11O09_F_0	BY941024
E_FL_e100_11O10_F_0	BY941025
E_FL_e100_11O10_R_0	BY944504
E_FL_e100_11O13_F_0	BY941026
E_FL_e100_11O13_R_0	BY944505
E_FL_e100_11O14_F_0	BY941027
E_FL_e100_11O14_R_0	BY944506
E_FL_e100_11O15_F_0	BY941028
E_FL_e100_11O16_F_0	BY941029
E_FL_e100_11O23_R_0	BY944507
E_FL_e100_11O24_F_0	BY941030
E_FL_e100_11O24_R_0	BY944508
E_FL_e100_11P04_F_0	BY941031
E_FL_e100_11P04_R_0	BY944509
E_FL_e100_11P06_F_0	BY941032
E_FL_e100_11P07_R_0	BY944510
E_FL_e100_11P09_F_0	BY941033
E_FL_e100_11P09_R_0	BY944511
E_FL_e100_11P10_F_0	BY941034
E_FL_e100_11P10_R_0	BY944512
E_FL_e100_11P11_F_0	BY941035
E_FL_e100_11P11_R_0	BY944513
E_FL_e100_11P13_F_0	BY941036
E_FL_e100_11P14_R_0	BY944514
E_FL_e100_11P18_F_0	BY941037
E_FL_e100_11P19_F_0	BY941038
E_FL_e100_11P20_F_0	BY941039
E_FL_e100_11P20_R_0	BY944515
E_FL_e100_11P21_R_0	BY944516
E_FL_e100_11P23_F_0	BY941040
E_FL_e100_11P23_R_0	BY944517
E_FL_e100_12A03_F_0	BY941041
E_FL_e100_12A03_R_0	BY944518

E_FL_e100_12A04_F_0	BY941042
E_FL_e100_12A10_F_0	BY941043
E_FL_e100_12A10_R_0	BY944519
E_FL_e100_12A11_F_0	BY941044
E_FL_e100_12A15_F_0	BY941045
E_FL_e100_12A17_F_0	BY941046
E_FL_e100_12A19_F_0	BY941047
E_FL_e100_12A20_F_0	BY941048
E_FL_e100_12A24_F_0	BY941049
E_FL_e100_12A24_R_0	BY944520
E_FL_e100_12B01_F_0	BY941050
E_FL_e100_12B01_R_0	BY944521
E_FL_e100_12B02_F_0	BY941051
E_FL_e100_12B02_R_0	BY944522
E_FL_e100_12B03_F_0	BY941052
E_FL_e100_12B03_R_0	BY944523
E_FL_e100_12B05_F_0	BY941053
E_FL_e100_12B06_F_0	BY941054
E_FL_e100_12B06_R_0	BY944524
E_FL_e100_12B09_F_0	BY941055
E_FL_e100_12B11_F_0	BY941056
E_FL_e100_12B13_F_0	BY941057
E_FL_e100_12B14_F_0	BY941058
E_FL_e100_12B16_F_0	BY941059
E_FL_e100_12B16_R_0	BY944525
E_FL_e100_12B19_F_0	BY941060
E_FL_e100_12B19_R_0	BY944526
E_FL_e100_12B20_F_0	BY941061
E_FL_e100_12B20_R_0	BY944527
E_FL_e100_12B21_F_0	BY941062
E_FL_e100_12B22_F_0	BY941063
E_FL_e100_12B22_R_0	BY944528
E_FL_e100_12B23_F_0	BY941064
E_FL_e100_12B24_F_0	BY941065
E_FL_e100_12C05_F_0	BY941066
E_FL_e100_12C08_F_0	BY941067
E_FL_e100_12C12_F_0	BY941068
E_FL_e100_12C13_F_0	BY941069
E_FL_e100_12C14_F_0	BY941070
E_FL_e100_12C14_R_0	BY944529
E_FL_e100_12C16_F_0	BY941071
E_FL_e100_12C16_R_0	BY944530
E_FL_e100_12C18_F_0	BY941072
E_FL_e100_12C20_F_0	BY941073
E_FL_e100_12C21_F_0	BY941074
E_FL_e100_12C21_R_0	BY944531
E_FL_e100_12C22_F_0	BY941075
E_FL_e100_12C22_R_0	BY944532
E_FL_e100_12C23_F_0	BY941076
E_FL_e100_12D03_F_0	BY941077
E_FL_e100_12D04_F_0	BY941078
E_FL_e100_12D08_F_0	BY941079
E_FL_e100_12D09_F_0	BY941080
E_FL_e100_12D10_F_0	BY941081

E_FL_e100_12D11_F_0	BY941082
E_FL_e100_12D11_R_0	BY944533
E_FL_e100_12D15_F_0	BY941083
E_FL_e100_12D15_R_0	BY944534
E_FL_e100_12D16_F_0	BY941084
E_FL_e100_12D17_F_0	BY941085
E_FL_e100_12D18_R_0	BY944535
E_FL_e100_12D20_F_0	BY941086
E_FL_e100_12D20_R_0	BY944536
E_FL_e100_12D21_F_0	BY941087
E_FL_e100_12D22_F_0	BY941088
E_FL_e100_12D23_F_0	BY941089
E_FL_e100_12D23_R_0	BY944537
E_FL_e100_12D24_F_0	BY941090
E_FL_e100_12D24_R_0	BY944538
E_FL_e100_12E01_R_0	BY944539
E_FL_e100_12E06_F_0	BY941091
E_FL_e100_12E06_R_0	BY944540
E_FL_e100_12E08_F_0	BY941092
E_FL_e100_12E10_F_0	BY941093
E_FL_e100_12E11_F_0	BY941094
E_FL_e100_12E12_F_0	BY941095
E_FL_e100_12E13_F_0	BY941096
E_FL_e100_12E15_F_0	BY941097
E_FL_e100_12E16_F_0	BY941098
E_FL_e100_12E18_F_0	BY941099
E_FL_e100_12E18_R_0	BY944541
E_FL_e100_12E19_F_0	BY941100
E_FL_e100_12E20_F_0	BY941101
E_FL_e100_12E20_R_0	BY944542
E_FL_e100_12E21_R_0	BY944543
E_FL_e100_12E22_F_0	BY941102
E_FL_e100_12F05_F_0	BY941103
E_FL_e100_12F06_F_0	BY941104
E_FL_e100_12F07_R_0	BY944544
E_FL_e100_12F11_F_0	BY941105
E_FL_e100_12F12_F_0	BY941106
E_FL_e100_12F14_F_0	BY941107
E_FL_e100_12F16_F_0	BY941108
E_FL_e100_12F17_F_0	BY941109
E_FL_e100_12F18_F_0	BY941110
E_FL_e100_12F19_F_0	BY941111
E_FL_e100_12F19_R_0	BY944545
E_FL_e100_12G02_F_0	BY941112
E_FL_e100_12G04_F_0	BY941113
E_FL_e100_12G04_R_0	BY944546
E_FL_e100_12G05_F_0	BY941114
E_FL_e100_12G07_F_0	BY941115
E_FL_e100_12G09_F_0	BY941116
E_FL_e100_12G10_F_0	BY941117
E_FL_e100_12G10_R_0	BY944547
E_FL_e100_12G11_F_0	BY941118
E_FL_e100_12G13_F_0	BY941119
E_FL_e100_12G15_F_0	BY941120

E_FL_e100_12G15_R_0	BY944548
E_FL_e100_12G17_F_0	BY941121
E_FL_e100_12G18_F_0	BY941122
E_FL_e100_12G19_F_0	BY941123
E_FL_e100_12G23_F_0	BY941124
E_FL_e100_12H01_F_0	BY941125
E_FL_e100_12H01_R_0	BY944549
E_FL_e100_12H02_F_0	BY941126
E_FL_e100_12H02_R_0	BY944550
E_FL_e100_12H03_F_0	BY941127
E_FL_e100_12H05_F_0	BY941128
E_FL_e100_12H08_F_0	BY941129
E_FL_e100_12H11_F_0	BY941130
E_FL_e100_12H12_F_0	BY941131
E_FL_e100_12H14_F_0	BY941132
E_FL_e100_12H15_F_0	BY941133
E_FL_e100_12H19_F_0	BY941134
E_FL_e100_12H19_R_0	BY944551
E_FL_e100_12H20_F_0	BY941135
E_FL_e100_12H21_F_0	BY941136
E_FL_e100_12H21_R_0	BY944552
E_FL_e100_12H23_F_0	BY941137
E_FL_e100_12I01_R_0	BY944553
E_FL_e100_12I05_F_0	BY941138
E_FL_e100_12I06_F_0	BY941139
E_FL_e100_12I07_R_0	BY944554
E_FL_e100_12I08_F_0	BY941140
E_FL_e100_12I10_F_0	BY941141
E_FL_e100_12I13_F_0	BY941142
E_FL_e100_12I14_R_0	BY944555
E_FL_e100_12I15_F_0	BY941143
E_FL_e100_12I15_R_0	BY944556
E_FL_e100_12I16_F_0	BY941144
E_FL_e100_12I17_F_0	BY941145
E_FL_e100_12I22_F_0	BY941146
E_FL_e100_12J01_F_0	BY941147
E_FL_e100_12J02_F_0	BY941148
E_FL_e100_12J03_F_0	BY941149
E_FL_e100_12J03_R_0	BY944557
E_FL_e100_12J04_F_0	BY941150
E_FL_e100_12J05_F_0	BY941151
E_FL_e100_12J06_F_0	BY941152
E_FL_e100_12J06_R_0	BY944558
E_FL_e100_12J08_F_0	BY941153
E_FL_e100_12J10_F_0	BY941154
E_FL_e100_12J11_F_0	BY941155
E_FL_e100_12J13_F_0	BY941156
E_FL_e100_12J14_F_0	BY941157
E_FL_e100_12J14_R_0	BY944559
E_FL_e100_12J16_F_0	BY941158
E_FL_e100_12J18_F_0	BY941159
E_FL_e100_12J18_R_0	BY944560
E_FL_e100_12J19_F_0	BY941160
E_FL_e100_12J21_F_0	BY941161

E_FL_e100_12J22_F_0	BY941162
E_FL_e100_12J23_F_0	BY941163
E_FL_e100_12J23_R_0	BY944561
E_FL_e100_12J24_F_0	BY941164
E_FL_e100_12K04_F_0	BY941165
E_FL_e100_12K04_R_0	BY944562
E_FL_e100_12K06_R_0	BY944563
E_FL_e100_12K08_F_0	BY941166
E_FL_e100_12K10_R_0	BY944564
E_FL_e100_12K12_F_0	BY941167
E_FL_e100_12K18_F_0	BY941168
E_FL_e100_12K20_R_0	BY944565
E_FL_e100_12K22_F_0	BY941169
E_FL_e100_12K23_F_0	BY941170
E_FL_e100_12K24_R_0	BY944566
E_FL_e100_12L01_F_0	BY941171
E_FL_e100_12L01_R_0	BY944567
E_FL_e100_12L03_F_0	BY941172
E_FL_e100_12L03_R_0	BY944568
E_FL_e100_12L04_F_0	BY941173
E_FL_e100_12L05_F_0	BY941174
E_FL_e100_12L05_R_0	BY944569
E_FL_e100_12L06_F_0	BY941175
E_FL_e100_12L07_F_0	BY941176
E_FL_e100_12L09_F_0	BY941177
E_FL_e100_12L09_R_0	BY944570
E_FL_e100_12L12_R_0	BY944571
E_FL_e100_12L13_F_0	BY941178
E_FL_e100_12L14_F_0	BY941179
E_FL_e100_12L15_F_0	BY941180
E_FL_e100_12L18_F_0	BY941181
E_FL_e100_12L19_R_0	BY944572
E_FL_e100_12L21_F_0	BY941182
E_FL_e100_12L21_R_0	BY944573
E_FL_e100_12L23_F_0	BY941183
E_FL_e100_12M02_F_0	BY941184
E_FL_e100_12M02_R_0	BY944574
E_FL_e100_12M07_F_0	BY941185
E_FL_e100_12M09_F_0	BY941186
E_FL_e100_12M10_F_0	BY941187
E_FL_e100_12M12_F_0	BY941188
E_FL_e100_12M13_F_0	BY941189
E_FL_e100_12M14_F_0	BY941190
E_FL_e100_12M16_F_0	BY941191
E_FL_e100_12M17_F_0	BY941192
E_FL_e100_12M20_F_0	BY941193
E_FL_e100_12M20_R_0	BY944575
E_FL_e100_12M21_F_0	BY941194
E_FL_e100_12M21_R_0	BY944576
E_FL_e100_12M24_F_0	BY941195
E_FL_e100_12N04_F_0	BY941196
E_FL_e100_12N04_R_0	BY944577
E_FL_e100_12N06_F_0	BY941197
E_FL_e100_12N07_F_0	BY941198

E_FL_e100_12N07_R_0	BY944578
E_FL_e100_12N08_F_0	BY941199
E_FL_e100_12N09_F_0	BY941200
E_FL_e100_12N09_R_0	BY944579
E_FL_e100_12N10_R_0	BY944580
E_FL_e100_12N12_R_0	BY944581
E_FL_e100_12N13_R_0	BY944582
E_FL_e100_12N14_F_0	BY941201
E_FL_e100_12N14_R_0	BY944583
E_FL_e100_12N15_F_0	BY941202
E_FL_e100_12N17_F_0	BY941203
E_FL_e100_12N21_F_0	BY941204
E_FL_e100_12N21_R_0	BY944584
E_FL_e100_12N23_F_0	BY941205
E_FL_e100_12N24_R_0	BY944585
E_FL_e100_12O01_F_0	BY941206
E_FL_e100_12O04_R_0	BY944586
E_FL_e100_12O05_F_0	BY941207
E_FL_e100_12O05_R_0	BY944587
E_FL_e100_12O07_F_0	BY941208
E_FL_e100_12O07_R_0	BY944588
E_FL_e100_12O09_R_0	BY944589
E_FL_e100_12O10_F_0	BY941209
E_FL_e100_12O12_F_0	BY941210
E_FL_e100_12O13_F_0	BY941211
E_FL_e100_12O13_R_0	BY944590
E_FL_e100_12O14_F_0	BY941212
E_FL_e100_12O14_R_0	BY944591
E_FL_e100_12O15_F_0	BY941213
E_FL_e100_12O17_R_0	BY944592
E_FL_e100_12O19_F_0	BY941214
E_FL_e100_12O19_R_0	BY944593
E_FL_e100_12O22_R_0	BY944594
E_FL_e100_12P01_F_0	BY941215
E_FL_e100_12P03_F_0	BY941216
E_FL_e100_12P03_R_0	BY944595
E_FL_e100_12P04_F_0	BY941217
E_FL_e100_12P04_R_0	BY944596
E_FL_e100_12P05_F_0	BY941218
E_FL_e100_12P05_R_0	BY944597
E_FL_e100_12P06_F_0	BY941219
E_FL_e100_12P09_F_0	BY941220
E_FL_e100_12P10_F_0	BY941221
E_FL_e100_12P11_F_0	BY941222
E_FL_e100_12P12_F_0	BY941223
E_FL_e100_12P13_F_0	BY941224
E_FL_e100_12P15_F_0	BY941225
E_FL_e100_12P17_F_0	BY941226
E_FL_e100_12P18_F_0	BY941227
E_FL_e100_12P19_F_0	BY941228
E_FL_e100_12P20_F_0	BY941229
E_FL_e100_12P20_R_0	BY944598
E_FL_e100_12P21_F_0	BY941230
E_FL_e100_12P22_F_0	BY941231

E_FL_e100_12P22_R_0	BY944599
E_FL_e100_12P23_F_0	BY941232
E_FL_e100_13A05_F_0	BY941233
E_FL_e100_13A06_F_0	BY941234
E_FL_e100_13A07_F_0	BY941235
E_FL_e100_13A10_F_0	BY941236
E_FL_e100_13A12_F_0	BY941237
E_FL_e100_13A14_F_0	BY941238
E_FL_e100_13A14_R_0	BY944600
E_FL_e100_13A15_F_0	BY941239
E_FL_e100_13A15_R_0	BY944601
E_FL_e100_13A16_R_0	BY944602
E_FL_e100_13A17_F_0	BY941240
E_FL_e100_13A18_F_0	BY941241
E_FL_e100_13A19_F_0	BY941242
E_FL_e100_13A20_F_0	BY941243
E_FL_e100_13A20_R_0	BY944603
E_FL_e100_13A23_R_0	BY944604
E_FL_e100_13B01_F_0	BY941244
E_FL_e100_13B01_R_0	BY944605
E_FL_e100_13B03_F_0	BY941245
E_FL_e100_13B03_R_0	BY944606
E_FL_e100_13B06_F_0	BY941246
E_FL_e100_13B06_R_0	BY944607
E_FL_e100_13B10_F_0	BY941247
E_FL_e100_13B10_R_0	BY944608
E_FL_e100_13B11_F_0	BY941248
E_FL_e100_13B12_F_0	BY941249
E_FL_e100_13B12_R_0	BY944609
E_FL_e100_13B13_F_0	BY941250
E_FL_e100_13B14_F_0	BY941251
E_FL_e100_13B15_F_0	BY941252
E_FL_e100_13B15_R_0	BY944610
E_FL_e100_13B16_F_0	BY941253
E_FL_e100_13B16_R_0	BY944611
E_FL_e100_13B17_F_0	BY941254
E_FL_e100_13B17_R_0	BY944612
E_FL_e100_13B18_F_0	BY941255
E_FL_e100_13B18_R_0	BY944613
E_FL_e100_13C02_F_0	BY941256
E_FL_e100_13C03_F_0	BY941257
E_FL_e100_13C06_F_0	BY941258
E_FL_e100_13C08_F_0	BY941259
E_FL_e100_13C08_R_0	BY944614
E_FL_e100_13C09_F_0	BY941260
E_FL_e100_13C10_F_0	BY941261
E_FL_e100_13C10_R_0	BY944615
E_FL_e100_13C12_F_0	BY941262
E_FL_e100_13C14_F_0	BY941263
E_FL_e100_13C17_F_0	BY941264
E_FL_e100_13C22_F_0	BY941265
E_FL_e100_13D01_F_0	BY941266
E_FL_e100_13D02_F_0	BY941267
E_FL_e100_13D02_R_0	BY944616

E_FL_e100_13D03_F_0	BY941268
E_FL_e100_13D03_R_0	BY944617
E_FL_e100_13D04_F_0	BY941269
E_FL_e100_13D04_R_0	BY944618
E_FL_e100_13D06_F_0	BY941270
E_FL_e100_13D06_R_0	BY944619
E_FL_e100_13D07_R_0	BY944620
E_FL_e100_13D08_F_0	BY941271
E_FL_e100_13D09_F_0	BY941272
E_FL_e100_13D09_R_0	BY944621
E_FL_e100_13D10_F_0	BY941273
E_FL_e100_13D12_F_0	BY941274
E_FL_e100_13D15_R_0	BY944622
E_FL_e100_13D16_F_0	BY941275
E_FL_e100_13D17_F_0	BY941276
E_FL_e100_13D17_R_0	BY944623
E_FL_e100_13D18_R_0	BY944624
E_FL_e100_13D19_F_0	BY941277
E_FL_e100_13D20_R_0	BY944625
E_FL_e100_13D22_F_0	BY941278
E_FL_e100_13D22_R_0	BY944626
E_FL_e100_13D23_F_0	BY941279
E_FL_e100_13D23_R_0	BY944627
E_FL_e100_13E01_F_0	BY941280
E_FL_e100_13E06_F_0	BY941281
E_FL_e100_13E09_F_0	BY941282
E_FL_e100_13E10_F_0	BY941283
E_FL_e100_13E11_F_0	BY941284
E_FL_e100_13E11_R_0	BY944628
E_FL_e100_13E13_F_0	BY941285
E_FL_e100_13E15_F_0	BY941286
E_FL_e100_13E16_R_0	BY944629
E_FL_e100_13E17_F_0	BY941287
E_FL_e100_13E18_F_0	BY941288
E_FL_e100_13E19_F_0	BY941289
E_FL_e100_13E19_R_0	BY944630
E_FL_e100_13E20_F_0	BY941290
E_FL_e100_13E20_R_0	BY944631
E_FL_e100_13E24_F_0	BY941291
E_FL_e100_13F02_F_0	BY941292
E_FL_e100_13F04_F_0	BY941293
E_FL_e100_13F06_F_0	BY941294
E_FL_e100_13F07_F_0	BY941295
E_FL_e100_13F07_R_0	BY944632
E_FL_e100_13F08_F_0	BY941296
E_FL_e100_13F09_F_0	BY941297
E_FL_e100_13F09_R_0	BY944633
E_FL_e100_13F11_F_0	BY941298
E_FL_e100_13F11_R_0	BY944634
E_FL_e100_13F12_R_0	BY944635
E_FL_e100_13F13_F_0	BY941299
E_FL_e100_13F14_R_0	BY944636
E_FL_e100_13F15_F_0	BY941300
E_FL_e100_13F15_R_0	BY944637

E_FL_e100_13F17_F_0	BY941301
E_FL_e100_13F17_R_0	BY944638
E_FL_e100_13F22_F_0	BY941302
E_FL_e100_13F22_R_0	BY944639
E_FL_e100_13F24_F_0	BY941303
E_FL_e100_13G02_F_0	BY941304
E_FL_e100_13G02_R_0	BY944640
E_FL_e100_13G05_F_0	BY941305
E_FL_e100_13G07_F_0	BY941306
E_FL_e100_13G08_F_0	BY941307
E_FL_e100_13G08_R_0	BY944641
E_FL_e100_13G10_F_0	BY941308
E_FL_e100_13G10_R_0	BY944642
E_FL_e100_13G11_R_0	BY944643
E_FL_e100_13G13_F_0	BY941309
E_FL_e100_13G16_F_0	BY941310
E_FL_e100_13G16_R_0	BY944644
E_FL_e100_13G17_F_0	BY941311
E_FL_e100_13G17_R_0	BY944645
E_FL_e100_13G20_F_0	BY941312
E_FL_e100_13G20_R_0	BY944646
E_FL_e100_13H01_F_0	BY941313
E_FL_e100_13H01_R_0	BY944647
E_FL_e100_13H02_F_0	BY941314
E_FL_e100_13H02_R_0	BY944648
E_FL_e100_13H03_F_0	BY941315
E_FL_e100_13H03_R_0	BY944649
E_FL_e100_13H05_R_0	BY944650
E_FL_e100_13H06_F_0	BY941316
E_FL_e100_13H06_R_0	BY944651
E_FL_e100_13H10_F_0	BY941317
E_FL_e100_13H11_F_0	BY941318
E_FL_e100_13H12_F_0	BY941319
E_FL_e100_13H12_R_0	BY944652
E_FL_e100_13H13_F_0	BY941320
E_FL_e100_13H13_R_0	BY944653
E_FL_e100_13H16_F_0	BY941321
E_FL_e100_13H17_F_0	BY941322
E_FL_e100_13H17_R_0	BY944654
E_FL_e100_13H18_F_0	BY941323
E_FL_e100_13H19_F_0	BY941324
E_FL_e100_13H22_F_0	BY941325
E_FL_e100_13H23_F_0	BY941326
E_FL_e100_13H23_R_0	BY944655
E_FL_e100_13H24_F_0	BY941327
E_FL_e100_13H24_R_0	BY944656
E_FL_e100_13I01_F_0	BY941328
E_FL_e100_13I01_R_0	BY944657
E_FL_e100_13I02_F_0	BY941329
E_FL_e100_13I03_R_0	BY944658
E_FL_e100_13I07_F_0	BY941330
E_FL_e100_13I08_F_0	BY941331
E_FL_e100_13I09_F_0	BY941332
E_FL_e100_13I09_R_0	BY944659

E_FL_e100_13I11_F_0	BY941333
E_FL_e100_13I11_R_0	BY944660
E_FL_e100_13I12_F_0	BY941334
E_FL_e100_13I12_R_0	BY944661
E_FL_e100_13I13_F_0	BY941335
E_FL_e100_13I15_F_0	BY941336
E_FL_e100_13I15_R_0	BY944662
E_FL_e100_13I16_F_0	BY941337
E_FL_e100_13I18_F_0	BY941338
E_FL_e100_13I19_F_0	BY941339
E_FL_e100_13J02_F_0	BY941340
E_FL_e100_13J02_R_0	BY944663
E_FL_e100_13J03_F_0	BY941341
E_FL_e100_13J04_F_0	BY941342
E_FL_e100_13J06_F_0	BY941343
E_FL_e100_13J07_F_0	BY941344
E_FL_e100_13J07_R_0	BY944664
E_FL_e100_13J09_F_0	BY941345
E_FL_e100_13J09_R_0	BY944665
E_FL_e100_13J10_F_0	BY941346
E_FL_e100_13J13_F_0	BY941347
E_FL_e100_13J13_R_0	BY944666
E_FL_e100_13J15_F_0	BY941348
E_FL_e100_13J16_F_0	BY941349
E_FL_e100_13J17_F_0	BY941350
E_FL_e100_13J18_F_0	BY941351
E_FL_e100_13J18_R_0	BY944667
E_FL_e100_13K03_F_0	BY941352
E_FL_e100_13K04_F_0	BY941353
E_FL_e100_13K06_F_0	BY941354
E_FL_e100_13K08_F_0	BY941355
E_FL_e100_13K09_F_0	BY941356
E_FL_e100_13K09_R_0	BY944668
E_FL_e100_13K10_R_0	BY944669
E_FL_e100_13K11_F_0	BY941357
E_FL_e100_13K13_F_0	BY941358
E_FL_e100_13K14_F_0	BY941359
E_FL_e100_13K15_R_0	BY944670
E_FL_e100_13K17_F_0	BY941360
E_FL_e100_13K19_F_0	BY941361
E_FL_e100_13K19_R_0	BY944671
E_FL_e100_13L01_F_0	BY941362
E_FL_e100_13L01_R_0	BY944672
E_FL_e100_13L02_F_0	BY941363
E_FL_e100_13L03_F_0	BY941364
E_FL_e100_13L10_F_0	BY941365
E_FL_e100_13L10_R_0	BY944673
E_FL_e100_13L12_F_0	BY941366
E_FL_e100_13L13_F_0	BY941367
E_FL_e100_13L14_F_0	BY941368
E_FL_e100_13L17_F_0	BY941369
E_FL_e100_13L17_R_0	BY944674
E_FL_e100_13M02_F_0	BY941370
E_FL_e100_13M02_R_0	BY944675

E_FL_e100_13M03_F_0	BY941371
E_FL_e100_13M03_R_0	BY944676
E_FL_e100_13M08_F_0	BY941372
E_FL_e100_13M08_R_0	BY944677
E_FL_e100_13M10_F_0	BY941373
E_FL_e100_13M10_R_0	BY944678
E_FL_e100_13M13_F_0	BY941374
E_FL_e100_13M13_R_0	BY944679
E_FL_e100_13M15_F_0	BY941375
E_FL_e100_13M15_R_0	BY944680
E_FL_e100_13M16_F_0	BY941376
E_FL_e100_13M16_R_0	BY944681
E_FL_e100_13M17_F_0	BY941377
E_FL_e100_13M17_R_0	BY944682
E_FL_e100_13M19_F_0	BY941378
E_FL_e100_13M23_F_0	BY941379
E_FL_e100_13N05_F_0	BY941380
E_FL_e100_13N07_R_0	BY944683
E_FL_e100_13N08_F_0	BY941381
E_FL_e100_13N08_R_0	BY944684
E_FL_e100_13N09_F_0	BY941382
E_FL_e100_13N13_R_0	BY944685
E_FL_e100_13N14_F_0	BY941383
E_FL_e100_13N14_R_0	BY944686
E_FL_e100_13N15_R_0	BY944687
E_FL_e100_13N17_F_0	BY941384
E_FL_e100_13N18_R_0	BY944688
E_FL_e100_13N19_F_0	BY941385
E_FL_e100_13N19_R_0	BY944689
E_FL_e100_13N20_F_0	BY941386
E_FL_e100_13N21_F_0	BY941387
E_FL_e100_13N21_R_0	BY944690
E_FL_e100_13N22_F_0	BY941388
E_FL_e100_13N22_R_0	BY944691
E_FL_e100_13O02_F_0	BY941389
E_FL_e100_13O04_F_0	BY941390
E_FL_e100_13O07_F_0	BY941391
E_FL_e100_13O09_F_0	BY941392
E_FL_e100_13O11_F_0	BY941393
E_FL_e100_13O14_F_0	BY941394
E_FL_e100_13O14_R_0	BY944692
E_FL_e100_13O17_F_0	BY941395
E_FL_e100_13O19_F_0	BY941396
E_FL_e100_13O20_F_0	BY941397
E_FL_e100_13O22_F_0	BY941398
E_FL_e100_13O23_F_0	BY941399
E_FL_e100_13O23_R_0	BY944693
E_FL_e100_13P07_F_0	BY941400
E_FL_e100_13P08_F_0	BY941401
E_FL_e100_13P08_R_0	BY944694
E_FL_e100_13P10_F_0	BY941402
E_FL_e100_13P12_F_0	BY941403
E_FL_e100_13P13_F_0	BY941404
E_FL_e100_13P15_F_0	BY941405

E_FL_e100_13P15_R_0	BY944695
E_FL_e100_13P19_F_0	BY941406
E_FL_e100_13P23_F_0	BY941407
E_FL_e100_14A02_F_0	BY941408
E_FL_e100_14A05_F_0	BY941409
E_FL_e100_14A06_F_0	BY941410
E_FL_e100_14A07_F_0	BY941411
E_FL_e100_14A09_F_0	BY941412
E_FL_e100_14A10_F_0	BY941413
E_FL_e100_14A11_F_0	BY941414
E_FL_e100_14A14_F_0	BY941415
E_FL_e100_14A15_F_0	BY941416
E_FL_e100_14A15_R_0	BY944696
E_FL_e100_14A16_F_0	BY941417
E_FL_e100_14A19_F_0	BY941418
E_FL_e100_14A20_R_0	BY944697
E_FL_e100_14A21_F_0	BY941419
E_FL_e100_14A21_R_0	BY944698
E_FL_e100_14A22_F_0	BY941420
E_FL_e100_14A22_R_0	BY944699
E_FL_e100_14B02_F_0	BY941421
E_FL_e100_14B02_R_0	BY944700
E_FL_e100_14B09_F_0	BY941422
E_FL_e100_14B12_F_0	BY941423
E_FL_e100_14B12_R_0	BY944701
E_FL_e100_14B13_F_0	BY941424
E_FL_e100_14B13_R_0	BY944702
E_FL_e100_14B14_R_0	BY944703
E_FL_e100_14B15_F_0	BY941425
E_FL_e100_14B15_R_0	BY944704
E_FL_e100_14B19_F_0	BY941426
E_FL_e100_14B19_R_0	BY944705
E_FL_e100_14B20_F_0	BY941427
E_FL_e100_14B20_R_0	BY944706
E_FL_e100_14B22_F_0	BY941428
E_FL_e100_14B24_F_0	BY941429
E_FL_e100_14B24_R_0	BY944707
E_FL_e100_14C02_F_0	BY941430
E_FL_e100_14C02_R_0	BY944708
E_FL_e100_14C04_R_0	BY944709
E_FL_e100_14C05_F_0	BY941431
E_FL_e100_14C07_F_0	BY941432
E_FL_e100_14C09_F_0	BY941433
E_FL_e100_14C10_F_0	BY941434
E_FL_e100_14C11_F_0	BY941435
E_FL_e100_14C12_F_0	BY941436
E_FL_e100_14C15_F_0	BY941437
E_FL_e100_14C15_R_0	BY944710
E_FL_e100_14C16_F_0	BY941438
E_FL_e100_14C17_F_0	BY941439
E_FL_e100_14C18_F_0	BY941440
E_FL_e100_14C19_F_0	BY941441
E_FL_e100_14D02_F_0	BY941442
E_FL_e100_14D02_R_0	BY944711

E_FL_e100_14D03_F_0	BY941443
E_FL_e100_14D03_R_0	BY944712
E_FL_e100_14D05_F_0	BY941444
E_FL_e100_14D06_F_0	BY941445
E_FL_e100_14D07_F_0	BY941446
E_FL_e100_14D10_F_0	BY941447
E_FL_e100_14D11_F_0	BY941448
E_FL_e100_14D11_R_0	BY944713
E_FL_e100_14D12_F_0	BY941449
E_FL_e100_14D13_F_0	BY941450
E_FL_e100_14D14_F_0	BY941451
E_FL_e100_14D16_F_0	BY941452
E_FL_e100_14D16_R_0	BY944714
E_FL_e100_14D17_F_0	BY941453
E_FL_e100_14D18_F_0	BY941454
E_FL_e100_14D22_F_0	BY941455
E_FL_e100_14D23_F_0	BY941456
E_FL_e100_14D23_R_0	BY944715
E_FL_e100_14D24_R_0	BY944716
E_FL_e100_14E01_F_0	BY941457
E_FL_e100_14E01_R_0	BY944717
E_FL_e100_14E02_F_0	BY941458
E_FL_e100_14E03_F_0	BY941459
E_FL_e100_14E04_F_0	BY941460
E_FL_e100_14E06_R_0	BY944718
E_FL_e100_14E07_F_0	BY941461
E_FL_e100_14E07_R_0	BY944719
E_FL_e100_14E08_R_0	BY944720
E_FL_e100_14E09_F_0	BY941462
E_FL_e100_14E09_R_0	BY944721
E_FL_e100_14E10_F_0	BY941463
E_FL_e100_14E12_F_0	BY941464
E_FL_e100_14E12_R_0	BY944722
E_FL_e100_14E14_F_0	BY941465
E_FL_e100_14E15_F_0	BY941466
E_FL_e100_14E15_R_0	BY944723
E_FL_e100_14E17_F_0	BY941467
E_FL_e100_14E17_R_0	BY944724
E_FL_e100_14E18_R_0	BY944725
E_FL_e100_14E19_F_0	BY941468
E_FL_e100_14E19_R_0	BY944726
E_FL_e100_14E21_F_0	BY941469
E_FL_e100_14E22_F_0	BY941470
E_FL_e100_14E23_F_0	BY941471
E_FL_e100_14E24_F_0	BY941472
E_FL_e100_14E24_R_0	BY944727
E_FL_e100_14F01_F_0	BY941473
E_FL_e100_14F02_F_0	BY941474
E_FL_e100_14F02_R_0	BY944728
E_FL_e100_14F05_F_0	BY941475
E_FL_e100_14F06_F_0	BY941476
E_FL_e100_14F10_F_0	BY941477
E_FL_e100_14F11_F_0	BY941478
E_FL_e100_14F11_R_0	BY944729

E_FL_e100_14F15_F_0	BY941479
E_FL_e100_14F15_R_0	BY944730
E_FL_e100_14F16_R_0	BY944731
E_FL_e100_14F17_F_0	BY941480
E_FL_e100_14F20_F_0	BY941481
E_FL_e100_14F21_F_0	BY941482
E_FL_e100_14F22_F_0	BY941483
E_FL_e100_14F22_R_0	BY944732
E_FL_e100_14F23_F_0	BY941484
E_FL_e100_14F23_R_0	BY944733
E_FL_e100_14G02_F_0	BY941485
E_FL_e100_14G03_R_0	BY944734
E_FL_e100_14G04_F_0	BY941486
E_FL_e100_14G13_F_0	BY941487
E_FL_e100_14G14_F_0	BY941488
E_FL_e100_14G15_F_0	BY941489
E_FL_e100_14H01_F_0	BY941490
E_FL_e100_14H02_R_0	BY944735
E_FL_e100_14H03_F_0	BY941491
E_FL_e100_14H06_F_0	BY941492
E_FL_e100_14H06_R_0	BY944736
E_FL_e100_14H08_F_0	BY941493
E_FL_e100_14H10_F_0	BY941494
E_FL_e100_14H11_F_0	BY941495
E_FL_e100_14H14_F_0	BY941496
E_FL_e100_14H14_R_0	BY944737
E_FL_e100_14H15_F_0	BY941497
E_FL_e100_14H18_F_0	BY941498
E_FL_e100_14H21_R_0	BY944738
E_FL_e100_14H22_F_0	BY941499
E_FL_e100_14I01_F_0	BY941500
E_FL_e100_14I02_F_0	BY941501
E_FL_e100_14I02_R_0	BY944739
E_FL_e100_14I05_F_0	BY941502
E_FL_e100_14I05_R_0	BY944740
E_FL_e100_14I06_F_0	BY941503
E_FL_e100_14I07_F_0	BY941504
E_FL_e100_14I08_F_0	BY941505
E_FL_e100_14I09_F_0	BY941506
E_FL_e100_14I09_R_0	BY944741
E_FL_e100_14I11_F_0	BY941507
E_FL_e100_14I11_R_0	BY944742
E_FL_e100_14I12_F_0	BY941508
E_FL_e100_14I12_R_0	BY944743
E_FL_e100_14I13_F_0	BY941509
E_FL_e100_14I14_F_0	BY941510
E_FL_e100_14I14_R_0	BY944744
E_FL_e100_14I15_F_0	BY941511
E_FL_e100_14I15_R_0	BY944745
E_FL_e100_14I16_F_0	BY941512
E_FL_e100_14I16_R_0	BY944746
E_FL_e100_14I17_F_0	BY941513
E_FL_e100_14I18_F_0	BY941514
E_FL_e100_14I19_F_0	BY941515

E_FL_e100_14J01_F_0	BY941516
E_FL_e100_14J01_R_0	BY944747
E_FL_e100_14J02_F_0	BY941517
E_FL_e100_14J02_R_0	BY944748
E_FL_e100_14J03_R_0	BY944749
E_FL_e100_14J04_F_0	BY941518
E_FL_e100_14J05_F_0	BY941519
E_FL_e100_14J05_R_0	BY944750
E_FL_e100_14J11_F_0	BY941520
E_FL_e100_14J14_F_0	BY941521
E_FL_e100_14J15_F_0	BY941522
E_FL_e100_14J15_R_0	BY944751
E_FL_e100_14J16_F_0	BY941523
E_FL_e100_14J17_F_0	BY941524
E_FL_e100_14J18_F_0	BY941525
E_FL_e100_14J18_R_0	BY944752
E_FL_e100_14J20_F_0	BY941526
E_FL_e100_14J20_R_0	BY944753
E_FL_e100_14J21_F_0	BY941527
E_FL_e100_14J23_F_0	BY941528
E_FL_e100_14J24_F_0	BY941529
E_FL_e100_14J24_R_0	BY944754
E_FL_e100_14K02_F_0	BY941530
E_FL_e100_14K02_R_0	BY944755
E_FL_e100_14K04_F_0	BY941531
E_FL_e100_14K04_R_0	BY944756
E_FL_e100_14K05_F_0	BY941532
E_FL_e100_14K07_F_0	BY941533
E_FL_e100_14K08_F_0	BY941534
E_FL_e100_14K11_F_0	BY941535
E_FL_e100_14K13_F_0	BY941536
E_FL_e100_14K15_F_0	BY941537
E_FL_e100_14K16_F_0	BY941538
E_FL_e100_14K16_R_0	BY944757
E_FL_e100_14K18_F_0	BY941539
E_FL_e100_14K19_F_0	BY941540
E_FL_e100_14K19_R_0	BY944758
E_FL_e100_14K21_F_0	BY941541
E_FL_e100_14K22_R_0	BY944759
E_FL_e100_14K23_F_0	BY941542
E_FL_e100_14L01_F_0	BY941543
E_FL_e100_14L01_R_0	BY944760
E_FL_e100_14L02_F_0	BY941544
E_FL_e100_14L03_F_0	BY941545
E_FL_e100_14L03_R_0	BY944761
E_FL_e100_14L04_F_0	BY941546
E_FL_e100_14L04_R_0	BY944762
E_FL_e100_14L05_F_0	BY941547
E_FL_e100_14L05_R_0	BY944763
E_FL_e100_14L06_F_0	BY941548
E_FL_e100_14L06_R_0	BY944764
E_FL_e100_14L09_F_0	BY941549
E_FL_e100_14L10_F_0	BY941550
E_FL_e100_14L13_F_0	BY941551

E_FL_e100_14L14_F_0	BY941552
E_FL_e100_14L15_F_0	BY941553
E_FL_e100_14L23_F_0	BY941554
E_FL_e100_14L23_R_0	BY944765
E_FL_e100_14L24_F_0	BY941555
E_FL_e100_14L24_R_0	BY944766
E_FL_e100_14M02_F_0	BY941556
E_FL_e100_14M03_F_0	BY941557
E_FL_e100_14M03_R_0	BY944767
E_FL_e100_14M09_F_0	BY941558
E_FL_e100_14M09_R_0	BY944768
E_FL_e100_14M11_F_0	BY941559
E_FL_e100_14M11_R_0	BY944769
E_FL_e100_14M14_F_0	BY941560
E_FL_e100_14M16_F_0	BY941561
E_FL_e100_14M17_F_0	BY941562
E_FL_e100_14M19_F_0	BY941563
E_FL_e100_14M20_F_0	BY941564
E_FL_e100_14M20_R_0	BY944770
E_FL_e100_14M21_F_0	BY941565
E_FL_e100_14M21_R_0	BY944771
E_FL_e100_14M22_F_0	BY941566
E_FL_e100_14M23_F_0	BY941567
E_FL_e100_14M23_R_0	BY944772
E_FL_e100_14N01_F_0	BY941568
E_FL_e100_14N02_F_0	BY941569
E_FL_e100_14N02_R_0	BY944773
E_FL_e100_14N03_F_0	BY941570
E_FL_e100_14N03_R_0	BY944774
E_FL_e100_14N05_F_0	BY941571
E_FL_e100_14N05_R_0	BY944775
E_FL_e100_14N06_R_0	BY944776
E_FL_e100_14N07_F_0	BY941572
E_FL_e100_14N09_F_0	BY941573
E_FL_e100_14N10_F_0	BY941574
E_FL_e100_14N11_F_0	BY941575
E_FL_e100_14N11_R_0	BY944777
E_FL_e100_14N14_F_0	BY941576
E_FL_e100_14N15_F_0	BY941577
E_FL_e100_14N15_R_0	BY944778
E_FL_e100_14N16_F_0	BY941578
E_FL_e100_14N16_R_0	BY944779
E_FL_e100_14N17_F_0	BY941579
E_FL_e100_14N17_R_0	BY944780
E_FL_e100_14N19_F_0	BY941580
E_FL_e100_14N20_F_0	BY941581
E_FL_e100_14N22_F_0	BY941582
E_FL_e100_14N24_R_0	BY944781
E_FL_e100_14O02_F_0	BY941583
E_FL_e100_14O03_F_0	BY941584
E_FL_e100_14O03_R_0	BY944782
E_FL_e100_14O07_F_0	BY941585
E_FL_e100_14O08_F_0	BY941586
E_FL_e100_14O09_F_0	BY941587

E_FL_e100_14O09_R_0	BY944783
E_FL_e100_14O11_F_0	BY941588
E_FL_e100_14O13_F_0	BY941589
E_FL_e100_14O16_R_0	BY944784
E_FL_e100_14O17_F_0	BY941590
E_FL_e100_14O19_F_0	BY941591
E_FL_e100_14O19_R_0	BY944785
E_FL_e100_14O20_F_0	BY941592
E_FL_e100_14O21_F_0	BY941593
E_FL_e100_14O22_F_0	BY941594
E_FL_e100_14O22_R_0	BY944786
E_FL_e100_14O23_F_0	BY941595
E_FL_e100_14P01_F_0	BY941596
E_FL_e100_14P01_R_0	BY944787
E_FL_e100_14P02_F_0	BY941597
E_FL_e100_14P04_F_0	BY941598
E_FL_e100_14P05_F_0	BY941599
E_FL_e100_14P06_F_0	BY941600
E_FL_e100_14P07_F_0	BY941601
E_FL_e100_14P09_R_0	BY944788
E_FL_e100_14P10_F_0	BY941602
E_FL_e100_14P13_F_0	BY941603
E_FL_e100_14P14_F_0	BY941604
E_FL_e100_14P14_R_0	BY944789
E_FL_e100_14P15_F_0	BY941605
E_FL_e100_14P16_F_0	BY941606
E_FL_e100_14P17_F_0	BY941607
E_FL_e100_14P18_F_0	BY941608
E_FL_e100_14P19_F_0	BY941609
E_FL_e100_14P19_R_0	BY944790
E_FL_e100_14P20_F_0	BY941610
E_FL_e100_14P22_F_0	BY941611
E_FL_e100_14P24_R_0	BY944791
E_FL_e100_15A04_F_0	BY941612
E_FL_e100_15A04_R_0	BY944792
E_FL_e100_15A05_F_0	BY941613
E_FL_e100_15A05_R_0	BY944793
E_FL_e100_15A06_R_0	BY944794
E_FL_e100_15A07_F_0	BY941614
E_FL_e100_15A09_F_0	BY941615
E_FL_e100_15A13_R_0	BY944795
E_FL_e100_15A15_F_0	BY941616
E_FL_e100_15A16_F_0	BY941617
E_FL_e100_15A16_R_0	BY944796
E_FL_e100_15A18_F_0	BY941618
E_FL_e100_15A19_F_0	BY941619
E_FL_e100_15A20_F_0	BY941620
E_FL_e100_15A20_R_0	BY944797
E_FL_e100_15A23_F_0	BY941621
E_FL_e100_15A24_F_0	BY941622
E_FL_e100_15B03_F_0	BY941623
E_FL_e100_15B03_R_0	BY944798
E_FL_e100_15B05_F_0	BY941624
E_FL_e100_15B06_F_0	BY941625

E_FL_e100_15B07_F_0	BY941626
E_FL_e100_15B09_F_0	BY941627
E_FL_e100_15B09_R_0	BY944799
E_FL_e100_15B10_F_0	BY941628
E_FL_e100_15B10_R_0	BY944800
E_FL_e100_15B12_F_0	BY941629
E_FL_e100_15B15_F_0	BY941630
E_FL_e100_15B16_F_0	BY941631
E_FL_e100_15B16_R_0	BY944801
E_FL_e100_15B19_F_0	BY941632
E_FL_e100_15B21_F_0	BY941633
E_FL_e100_15B21_R_0	BY944802
E_FL_e100_15B23_R_0	BY944803
E_FL_e100_15B24_F_0	BY941634
E_FL_e100_15B24_R_0	BY944804
E_FL_e100_15C01_F_0	BY941635
E_FL_e100_15C03_F_0	BY941636
E_FL_e100_15C03_R_0	BY944805
E_FL_e100_15C04_F_0	BY941637
E_FL_e100_15C05_F_0	BY941638
E_FL_e100_15C07_F_0	BY941639
E_FL_e100_15C07_R_0	BY944806
E_FL_e100_15C08_R_0	BY944807
E_FL_e100_15C09_F_0	BY941640
E_FL_e100_15C09_R_0	BY944808
E_FL_e100_15C11_F_0	BY941641
E_FL_e100_15C11_R_0	BY944809
E_FL_e100_15C15_F_0	BY941642
E_FL_e100_15C15_R_0	BY944810
E_FL_e100_15C16_F_0	BY941643
E_FL_e100_15C17_F_0	BY941644
E_FL_e100_15C19_F_0	BY941645
E_FL_e100_15C23_R_0	BY944811
E_FL_e100_15C24_F_0	BY941646
E_FL_e100_15D01_F_0	BY941647
E_FL_e100_15D03_F_0	BY941648
E_FL_e100_15D05_F_0	BY941649
E_FL_e100_15D05_R_0	BY944812
E_FL_e100_15D08_F_0	BY941650
E_FL_e100_15D08_R_0	BY944813
E_FL_e100_15D10_F_0	BY941651
E_FL_e100_15D11_F_0	BY941652
E_FL_e100_15D12_F_0	BY941653
E_FL_e100_15D13_F_0	BY941654
E_FL_e100_15D15_F_0	BY941655
E_FL_e100_15D19_F_0	BY941656
E_FL_e100_15D20_R_0	BY944814
E_FL_e100_15D21_R_0	BY944815
E_FL_e100_15D22_F_0	BY941657
E_FL_e100_15D23_F_0	BY941658
E_FL_e100_15D23_R_0	BY944816
E_FL_e100_15E04_F_0	BY941659
E_FL_e100_15E07_F_0	BY941660
E_FL_e100_15E08_F_0	BY941661

E_FL_e100_15E08_R_0	BY944817
E_FL_e100_15E09_F_0	BY941662
E_FL_e100_15E13_F_0	BY941663
E_FL_e100_15E15_F_0	BY941664
E_FL_e100_15E16_F_0	BY941665
E_FL_e100_15E16_R_0	BY944818
E_FL_e100_15E23_F_0	BY941666
E_FL_e100_15F01_F_0	BY941667
E_FL_e100_15F01_R_0	BY944819
E_FL_e100_15F02_F_0	BY941668
E_FL_e100_15F02_R_0	BY944820
E_FL_e100_15F03_F_0	BY941669
E_FL_e100_15F03_R_0	BY944821
E_FL_e100_15F06_F_0	BY941670
E_FL_e100_15F08_F_0	BY941671
E_FL_e100_15F08_R_0	BY944822
E_FL_e100_15F11_F_0	BY941672
E_FL_e100_15F11_R_0	BY944823
E_FL_e100_15F12_F_0	BY941673
E_FL_e100_15F13_F_0	BY941674
E_FL_e100_15F14_F_0	BY941675
E_FL_e100_15F14_R_0	BY944824
E_FL_e100_15F15_F_0	BY941676
E_FL_e100_15F16_F_0	BY941677
E_FL_e100_15F16_R_0	BY944825
E_FL_e100_15F19_F_0	BY941678
E_FL_e100_15F20_F_0	BY941679
E_FL_e100_15F20_R_0	BY944826
E_FL_e100_15G03_F_0	BY941680
E_FL_e100_15G04_F_0	BY941681
E_FL_e100_15G04_R_0	BY944827
E_FL_e100_15G08_F_0	BY941682
E_FL_e100_15G10_F_0	BY941683
E_FL_e100_15G11_F_0	BY941684
E_FL_e100_15G11_R_0	BY944828
E_FL_e100_15G14_F_0	BY941685
E_FL_e100_15G14_R_0	BY944829
E_FL_e100_15G16_F_0	BY941686
E_FL_e100_15G17_F_0	BY941687
E_FL_e100_15G19_R_0	BY944830
E_FL_e100_15G21_F_0	BY941688
E_FL_e100_15G21_R_0	BY944831
E_FL_e100_15G22_F_0	BY941689
E_FL_e100_15G24_R_0	BY944832
E_FL_e100_15H01_F_0	BY941690
E_FL_e100_15H02_F_0	BY941691
E_FL_e100_15H03_F_0	BY941692
E_FL_e100_15H03_R_0	BY944833
E_FL_e100_15H05_F_0	BY941693
E_FL_e100_15H05_R_0	BY944834
E_FL_e100_15H13_F_0	BY941694
E_FL_e100_15H16_F_0	BY941695
E_FL_e100_15H16_R_0	BY944835
E_FL_e100_15H17_R_0	BY944836

E_FL_e100_15H19_F_0	BY941696
E_FL_e100_15H19_R_0	BY944837
E_FL_e100_15I02_F_0	BY941697
E_FL_e100_15I02_R_0	BY944838
E_FL_e100_15I03_F_0	BY941698
E_FL_e100_15I03_R_0	BY944839
E_FL_e100_15I04_F_0	BY941699
E_FL_e100_15I08_F_0	BY941700
E_FL_e100_15I08_R_0	BY944840
E_FL_e100_15I09_F_0	BY941701
E_FL_e100_15I09_R_0	BY944841
E_FL_e100_15I11_F_0	BY941702
E_FL_e100_15I11_R_0	BY944842
E_FL_e100_15I12_R_0	BY944843
E_FL_e100_15I13_F_0	BY941703
E_FL_e100_15I16_R_0	BY944844
E_FL_e100_15I17_F_0	BY941704
E_FL_e100_15I17_R_0	BY944845
E_FL_e100_15I19_F_0	BY941705
E_FL_e100_15I21_F_0	BY941706
E_FL_e100_15I22_F_0	BY941707
E_FL_e100_15I24_F_0	BY941708
E_FL_e100_15J02_F_0	BY941709
E_FL_e100_15J06_F_0	BY941710
E_FL_e100_15J08_F_0	BY941711
E_FL_e100_15J08_R_0	BY944846
E_FL_e100_15J10_F_0	BY941712
E_FL_e100_15J10_R_0	BY944847
E_FL_e100_15J11_F_0	BY941713
E_FL_e100_15J13_F_0	BY941714
E_FL_e100_15J13_R_0	BY944848
E_FL_e100_15J14_F_0	BY941715
E_FL_e100_15J14_R_0	BY944849
E_FL_e100_15J15_F_0	BY941716
E_FL_e100_15J15_R_0	BY944850
E_FL_e100_15J16_F_0	BY941717
E_FL_e100_15J17_F_0	BY941718
E_FL_e100_15J17_R_0	BY944851
E_FL_e100_15J18_F_0	BY941719
E_FL_e100_15J19_F_0	BY941720
E_FL_e100_15J19_R_0	BY944852
E_FL_e100_15J22_F_0	BY941721
E_FL_e100_15J23_F_0	BY941722
E_FL_e100_15J24_F_0	BY941723
E_FL_e100_15J24_R_0	BY944853
E_FL_e100_15K04_F_0	BY941724
E_FL_e100_15K04_R_0	BY944854
E_FL_e100_15K07_F_0	BY941725
E_FL_e100_15K07_R_0	BY944855
E_FL_e100_15K08_F_0	BY941726
E_FL_e100_15K08_R_0	BY944856
E_FL_e100_15K14_F_0	BY941727
E_FL_e100_15K14_R_0	BY944857
E_FL_e100_15K15_R_0	BY944858

E_FL_e100_15K16_F_0	BY941728
E_FL_e100_15K16_R_0	BY944859
E_FL_e100_15K19_F_0	BY941729
E_FL_e100_15K20_F_0	BY941730
E_FL_e100_15K20_R_0	BY944860
E_FL_e100_15K22_R_0	BY944861
E_FL_e100_15L01_F_0	BY941731
E_FL_e100_15L03_F_0	BY941732
E_FL_e100_15L04_F_0	BY941733
E_FL_e100_15L04_R_0	BY944862
E_FL_e100_15L07_F_0	BY941734
E_FL_e100_15L11_F_0	BY941735
E_FL_e100_15L11_R_0	BY944863
E_FL_e100_15L13_F_0	BY941736
E_FL_e100_15L13_R_0	BY944864
E_FL_e100_15L14_F_0	BY941737
E_FL_e100_15L14_R_0	BY944865
E_FL_e100_15L19_F_0	BY941738
E_FL_e100_15L19_R_0	BY944866
E_FL_e100_15L21_F_0	BY941739
E_FL_e100_15L21_R_0	BY944867
E_FL_e100_15L23_F_0	BY941740
E_FL_e100_15L23_R_0	BY944868
E_FL_e100_15M01_F_0	BY941741
E_FL_e100_15M02_F_0	BY941742
E_FL_e100_15M03_F_0	BY941743
E_FL_e100_15M03_R_0	BY944869
E_FL_e100_15M04_F_0	BY941744
E_FL_e100_15M05_F_0	BY941745
E_FL_e100_15M08_F_0	BY941746
E_FL_e100_15M09_F_0	BY941747
E_FL_e100_15M09_R_0	BY944870
E_FL_e100_15M11_F_0	BY941748
E_FL_e100_15M13_R_0	BY944871
E_FL_e100_15M22_F_0	BY941749
E_FL_e100_15M24_R_0	BY944872
E_FL_e100_15N03_F_0	BY941750
E_FL_e100_15N04_F_0	BY941751
E_FL_e100_15N07_F_0	BY941752
E_FL_e100_15N10_F_0	BY941753
E_FL_e100_15N13_F_0	BY941754
E_FL_e100_15N13_R_0	BY944873
E_FL_e100_15N16_F_0	BY941755
E_FL_e100_15N20_F_0	BY941756
E_FL_e100_15N20_R_0	BY944874
E_FL_e100_15N21_F_0	BY941757
E_FL_e100_15N21_R_0	BY944875
E_FL_e100_15N22_F_0	BY941758
E_FL_e100_15N22_R_0	BY944876
E_FL_e100_15N23_F_0	BY941759
E_FL_e100_15O01_F_0	BY941760
E_FL_e100_15O01_R_0	BY944877
E_FL_e100_15O03_F_0	BY941761
E_FL_e100_15O03_R_0	BY944878

E_FL_e100_15O04_F_0	BY941762
E_FL_e100_15O04_R_0	BY944879
E_FL_e100_15O05_F_0	BY941763
E_FL_e100_15O07_F_0	BY941764
E_FL_e100_15O08_F_0	BY941765
E_FL_e100_15O08_R_0	BY944880
E_FL_e100_15O10_F_0	BY941766
E_FL_e100_15O10_R_0	BY944881
E_FL_e100_15O11_F_0	BY941767
E_FL_e100_15O11_R_0	BY944882
E_FL_e100_15O13_R_0	BY944883
E_FL_e100_15O14_F_0	BY941768
E_FL_e100_15O14_R_0	BY944884
E_FL_e100_15O18_R_0	BY944885
E_FL_e100_15O19_F_0	BY941769
E_FL_e100_15O19_R_0	BY944886
E_FL_e100_15O20_F_0	BY941770
E_FL_e100_15O22_F_0	BY941771
E_FL_e100_15O22_R_0	BY944887
E_FL_e100_15O23_F_0	BY941772
E_FL_e100_15P01_F_0	BY941773
E_FL_e100_15P01_R_0	BY944888
E_FL_e100_15P02_F_0	BY941774
E_FL_e100_15P03_F_0	BY941775
E_FL_e100_15P05_F_0	BY941776
E_FL_e100_15P08_F_0	BY941777
E_FL_e100_15P09_F_0	BY941778
E_FL_e100_15P09_R_0	BY944889
E_FL_e100_15P10_F_0	BY941779
E_FL_e100_15P10_R_0	BY944890
E_FL_e100_15P11_F_0	BY941780
E_FL_e100_15P12_F_0	BY941781
E_FL_e100_15P13_F_0	BY941782
E_FL_e100_15P15_F_0	BY941783
E_FL_e100_15P16_F_0	BY941784
E_FL_e100_15P21_F_0	BY941785
E_FL_e100_15P21_R_0	BY944891
E_FL_e100_15P22_F_0	BY941786
E_FL_e100_15P22_R_0	BY944892
E_FL_e100_15P23_F_0	BY941787
E_FL_e100_15P24_F_0	BY941788
E_FL_e100_16A03_F_0	BY941789
E_FL_e100_16A06_F_0	BY941790
E_FL_e100_16A08_F_0	BY941791
E_FL_e100_16A08_R_0	BY944893
E_FL_e100_16A11_F_0	BY941792
E_FL_e100_16A11_R_0	BY944894
E_FL_e100_16A12_R_0	BY944895
E_FL_e100_16A13_F_0	BY941793
E_FL_e100_16A14_F_0	BY941794
E_FL_e100_16A14_R_0	BY944896
E_FL_e100_16A16_F_0	BY941795
E_FL_e100_16A16_R_0	BY944897
E_FL_e100_16A18_F_0	BY941796

E_FL_e100_16A18_R_0	BY944898
E_FL_e100_16A19_F_0	BY941797
E_FL_e100_16A20_F_0	BY941798
E_FL_e100_16A22_R_0	BY944899
E_FL_e100_16A23_F_0	BY941799
E_FL_e100_16A24_F_0	BY941800
E_FL_e100_16A24_R_0	BY944900
E_FL_e100_16B01_F_0	BY941801
E_FL_e100_16B01_R_0	BY944901
E_FL_e100_16B02_F_0	BY941802
E_FL_e100_16B02_R_0	BY944902
E_FL_e100_16B04_F_0	BY941803
E_FL_e100_16B04_R_0	BY944903
E_FL_e100_16B06_R_0	BY944904
E_FL_e100_16B12_F_0	BY941804
E_FL_e100_16B12_R_0	BY944905
E_FL_e100_16B14_F_0	BY941805
E_FL_e100_16B14_R_0	BY944906
E_FL_e100_16B16_F_0	BY941806
E_FL_e100_16B16_R_0	BY944907
E_FL_e100_16B17_F_0	BY941807
E_FL_e100_16B17_R_0	BY944908
E_FL_e100_16B18_R_0	BY944909
E_FL_e100_16B19_F_0	BY941808
E_FL_e100_16B20_F_0	BY941809
E_FL_e100_16B21_F_0	BY941810
E_FL_e100_16B21_R_0	BY944910
E_FL_e100_16B22_F_0	BY941811
E_FL_e100_16B23_F_0	BY941812
E_FL_e100_16B23_R_0	BY944911
E_FL_e100_16B24_F_0	BY941813
E_FL_e100_16B24_R_0	BY944912
E_FL_e100_16C02_F_0	BY941814
E_FL_e100_16C03_F_0	BY941815
E_FL_e100_16C07_F_0	BY941816
E_FL_e100_16C07_R_0	BY944913
E_FL_e100_16C09_F_0	BY941817
E_FL_e100_16C10_F_0	BY941818
E_FL_e100_16C11_F_0	BY941819
E_FL_e100_16C11_R_0	BY944914
E_FL_e100_16C12_F_0	BY941820
E_FL_e100_16C14_F_0	BY941821
E_FL_e100_16C15_F_0	BY941822
E_FL_e100_16C16_F_0	BY941823
E_FL_e100_16C19_F_0	BY941824
E_FL_e100_16C20_F_0	BY941825
E_FL_e100_16C23_F_0	BY941826
E_FL_e100_16D01_F_0	BY941827
E_FL_e100_16D03_F_0	BY941828
E_FL_e100_16D04_F_0	BY941829
E_FL_e100_16D05_F_0	BY941830
E_FL_e100_16D05_R_0	BY944915
E_FL_e100_16D06_F_0	BY941831
E_FL_e100_16D07_F_0	BY941832

E_FL_e100_16D07_R_0	BY944916
E_FL_e100_16D09_F_0	BY941833
E_FL_e100_16D10_F_0	BY941834
E_FL_e100_16D10_R_0	BY944917
E_FL_e100_16D12_F_0	BY941835
E_FL_e100_16D12_R_0	BY944918
E_FL_e100_16D14_F_0	BY941836
E_FL_e100_16D14_R_0	BY944919
E_FL_e100_16D15_F_0	BY941837
E_FL_e100_16D15_R_0	BY944920
E_FL_e100_16D16_F_0	BY941838
E_FL_e100_16D18_F_0	BY941839
E_FL_e100_16D18_R_0	BY944921
E_FL_e100_16D20_F_0	BY941840
E_FL_e100_16D21_F_0	BY941841
E_FL_e100_16D21_R_0	BY944922
E_FL_e100_16D22_F_0	BY941842
E_FL_e100_16D22_R_0	BY944923
E_FL_e100_16D24_F_0	BY941843
E_FL_e100_16D24_R_0	BY944924
E_FL_e100_16E01_F_0	BY941844
E_FL_e100_16E02_F_0	BY941845
E_FL_e100_16E03_F_0	BY941846
E_FL_e100_16E04_F_0	BY941847
E_FL_e100_16E07_F_0	BY941848
E_FL_e100_16E07_R_0	BY944925
E_FL_e100_16E08_F_0	BY941849
E_FL_e100_16E09_F_0	BY941850
E_FL_e100_16E16_F_0	BY941851
E_FL_e100_16E16_R_0	BY944926
E_FL_e100_16E18_F_0	BY941852
E_FL_e100_16E18_R_0	BY944927
E_FL_e100_16E19_F_0	BY941853
E_FL_e100_16E21_F_0	BY941854
E_FL_e100_16E22_F_0	BY941855
E_FL_e100_16E22_R_0	BY944928
E_FL_e100_16E23_F_0	BY941856
E_FL_e100_16E24_F_0	BY941857
E_FL_e100_16F02_F_0	BY941858
E_FL_e100_16F02_R_0	BY944929
E_FL_e100_16F03_F_0	BY941859
E_FL_e100_16F03_R_0	BY944930
E_FL_e100_16F05_F_0	BY941860
E_FL_e100_16F05_R_0	BY944931
E_FL_e100_16F06_F_0	BY941861
E_FL_e100_16F06_R_0	BY944932
E_FL_e100_16F07_F_0	BY941862
E_FL_e100_16F08_F_0	BY941863
E_FL_e100_16F08_R_0	BY944933
E_FL_e100_16F09_F_0	BY941864
E_FL_e100_16F14_F_0	BY941865
E_FL_e100_16F15_R_0	BY944934
E_FL_e100_16F16_R_0	BY944935
E_FL_e100_16F19_F_0	BY941866

E_FL_e100_16F20_F_0	BY941867
E_FL_e100_16F20_R_0	BY944936
E_FL_e100_16F24_F_0	BY941868
E_FL_e100_16F24_R_0	BY944937
E_FL_e100_16G03_F_0	BY941869
E_FL_e100_16G04_F_0	BY941870
E_FL_e100_16G04_R_0	BY944938
E_FL_e100_16G06_R_0	BY944939
E_FL_e100_16G09_F_0	BY941871
E_FL_e100_16G10_F_0	BY941872
E_FL_e100_16G13_F_0	BY941873
E_FL_e100_16G15_R_0	BY944940
E_FL_e100_16G16_F_0	BY941874
E_FL_e100_16G17_F_0	BY941875
E_FL_e100_16G17_R_0	BY944941
E_FL_e100_16G22_F_0	BY941876
E_FL_e100_16G24_R_0	BY944942
E_FL_e100_16H05_R_0	BY944943
E_FL_e100_16H08_R_0	BY944944
E_FL_e100_16H09_F_0	BY941877
E_FL_e100_16H10_R_0	BY944945
E_FL_e100_16H11_R_0	BY944946
E_FL_e100_16H12_F_0	BY941878
E_FL_e100_16H12_R_0	BY944947
E_FL_e100_16H15_F_0	BY941879
E_FL_e100_16H15_R_0	BY944948
E_FL_e100_16H16_F_0	BY941880
E_FL_e100_16H16_R_0	BY944949
E_FL_e100_16H17_F_0	BY941881
E_FL_e100_16H17_R_0	BY944950
E_FL_e100_16H18_F_0	BY941882
E_FL_e100_16H18_R_0	BY944951
E_FL_e100_16H22_F_0	BY941883
E_FL_e100_16H24_F_0	BY941884
E_FL_e100_16H24_R_0	BY944952
E_FL_e100_16I03_R_0	BY944953
E_FL_e100_16I04_F_0	BY941885
E_FL_e100_16I06_F_0	BY941886
E_FL_e100_16I06_R_0	BY944954
E_FL_e100_16I12_F_0	BY941887
E_FL_e100_16I12_R_0	BY944955
E_FL_e100_16I14_F_0	BY941888
E_FL_e100_16I16_F_0	BY941889
E_FL_e100_16I19_F_0	BY941890
E_FL_e100_16I19_R_0	BY944956
E_FL_e100_16I21_F_0	BY941891
E_FL_e100_16I22_F_0	BY941892
E_FL_e100_16J02_R_0	BY944957
E_FL_e100_16J06_F_0	BY941893
E_FL_e100_16J07_F_0	BY941894
E_FL_e100_16J07_R_0	BY944958
E_FL_e100_16J09_F_0	BY941895
E_FL_e100_16J12_F_0	BY941896
E_FL_e100_16J15_F_0	BY941897

E_FL_e100_16J16_F_0	BY941898
E_FL_e100_16J18_F_0	BY941899
E_FL_e100_16J18_R_0	BY944959
E_FL_e100_16J19_F_0	BY941900
E_FL_e100_16J21_F_0	BY941901
E_FL_e100_16J22_F_0	BY941902
E_FL_e100_16J22_R_0	BY944960
E_FL_e100_16K01_F_0	BY941903
E_FL_e100_16K02_F_0	BY941904
E_FL_e100_16K03_F_0	BY941905
E_FL_e100_16K07_F_0	BY941906
E_FL_e100_16K09_F_0	BY941907
E_FL_e100_16K11_R_0	BY944961
E_FL_e100_16K15_R_0	BY944962
E_FL_e100_16K19_F_0	BY941908
E_FL_e100_16K23_F_0	BY941909
E_FL_e100_16L01_F_0	BY941910
E_FL_e100_16L01_R_0	BY944963
E_FL_e100_16L06_F_0	BY941911
E_FL_e100_16L08_F_0	BY941912
E_FL_e100_16L08_R_0	BY944964
E_FL_e100_16L12_F_0	BY941913
E_FL_e100_16L13_F_0	BY941914
E_FL_e100_16L13_R_0	BY944965
E_FL_e100_16L14_F_0	BY941915
E_FL_e100_16L14_R_0	BY944966
E_FL_e100_16L15_F_0	BY941916
E_FL_e100_16L15_R_0	BY944967
E_FL_e100_16L16_F_0	BY941917
E_FL_e100_16L16_R_0	BY944968
E_FL_e100_16L17_F_0	BY941918
E_FL_e100_16L17_R_0	BY944969
E_FL_e100_16L19_F_0	BY941919
E_FL_e100_16L22_F_0	BY941920
E_FL_e100_16L23_F_0	BY941921
E_FL_e100_16L24_F_0	BY941922
E_FL_e100_16M01_F_0	BY941923
E_FL_e100_16M03_F_0	BY941924
E_FL_e100_16M07_F_0	BY941925
E_FL_e100_16M07_R_0	BY944970
E_FL_e100_16M09_F_0	BY941926
E_FL_e100_16M13_F_0	BY941927
E_FL_e100_16M21_F_0	BY941928
E_FL_e100_16N01_R_0	BY944971
E_FL_e100_16N04_F_0	BY941929
E_FL_e100_16N04_R_0	BY944972
E_FL_e100_16N05_F_0	BY941930
E_FL_e100_16N05_R_0	BY944973
E_FL_e100_16N08_F_0	BY941931
E_FL_e100_16N08_R_0	BY944974
E_FL_e100_16N09_F_0	BY941932
E_FL_e100_16N09_R_0	BY944975
E_FL_e100_16N11_F_0	BY941933
E_FL_e100_16N11_R_0	BY944976

E_FL_e100_16N17_F_0	BY941934
E_FL_e100_16N17_R_0	BY944977
E_FL_e100_16N19_F_0	BY941935
E_FL_e100_16N22_F_0	BY941936
E_FL_e100_16N23_F_0	BY941937
E_FL_e100_16O01_F_0	BY941938
E_FL_e100_16O02_F_0	BY941939
E_FL_e100_16O03_F_0	BY941940
E_FL_e100_16O04_F_0	BY941941
E_FL_e100_16O04_R_0	BY944978
E_FL_e100_16O05_F_0	BY941942
E_FL_e100_16O05_R_0	BY944979
E_FL_e100_16O07_F_0	BY941943
E_FL_e100_16O09_F_0	BY941944
E_FL_e100_16O11_F_0	BY941945
E_FL_e100_16O13_F_0	BY941946
E_FL_e100_16O16_F_0	BY941947
E_FL_e100_16O17_F_0	BY941948
E_FL_e100_16O18_R_0	BY944980
E_FL_e100_16O22_F_0	BY941949
E_FL_e100_16O22_R_0	BY944981
E_FL_e100_16P02_F_0	BY941950
E_FL_e100_16P05_F_0	BY941951
E_FL_e100_16P05_R_0	BY944982
E_FL_e100_16P07_F_0	BY941952
E_FL_e100_16P07_R_0	BY944983
E_FL_e100_16P08_F_0	BY941953
E_FL_e100_16P09_F_0	BY941954
E_FL_e100_16P09_R_0	BY944984
E_FL_e100_16P11_F_0	BY941955
E_FL_e100_16P12_F_0	BY941956
E_FL_e100_16P14_F_0	BY941957
E_FL_e100_16P16_F_0	BY941958
E_FL_e100_16P17_F_0	BY941959
E_FL_e100_16P18_F_0	BY941960
E_FL_e100_16P19_F_0	BY941961
E_FL_e100_16P20_F_0	BY941962
E_FL_e100_16P21_F_0	BY941963
E_FL_e100_17A01_F_0	BY941964
E_FL_e100_17A01_R_0	BY944985
E_FL_e100_17A03_R_0	BY944986
E_FL_e100_17A10_R_0	BY944987
E_FL_e100_17A13_F_0	BY941965
E_FL_e100_17A17_F_0	BY941966
E_FL_e100_17A19_F_0	BY941967
E_FL_e100_17A22_F_0	BY941968
E_FL_e100_17A22_R_0	BY944988
E_FL_e100_17A23_F_0	BY941969
E_FL_e100_17A24_F_0	BY941970
E_FL_e100_17A24_R_0	BY944989
E_FL_e100_17B02_F_0	BY941971
E_FL_e100_17B02_R_0	BY944990
E_FL_e100_17B03_F_0	BY941972
E_FL_e100_17B04_F_0	BY941973

E_FL_e100_17B05_R_0	BY944991
E_FL_e100_17B06_R_0	BY944992
E_FL_e100_17B07_F_0	BY941974
E_FL_e100_17B11_R_0	BY944993
E_FL_e100_17B14_F_0	BY941975
E_FL_e100_17B14_R_0	BY944994
E_FL_e100_17B17_R_0	BY944995
E_FL_e100_17B19_F_0	BY941976
E_FL_e100_17B21_F_0	BY941977
E_FL_e100_17B21_R_0	BY944996
E_FL_e100_17B22_F_0	BY941978
E_FL_e100_17B22_R_0	BY944997
E_FL_e100_17B23_F_0	BY941979
E_FL_e100_17B23_R_0	BY944998
E_FL_e100_17B24_F_0	BY941980
E_FL_e100_17B24_R_0	BY944999
E_FL_e100_17C02_R_0	BY945000
E_FL_e100_17C05_F_0	BY941981
E_FL_e100_17C10_R_0	BY945001
E_FL_e100_17C11_F_0	BY941982
E_FL_e100_17C12_F_0	BY941983
E_FL_e100_17C15_R_0	BY945002
E_FL_e100_17C16_F_0	BY941984
E_FL_e100_17C17_F_0	BY941985
E_FL_e100_17C17_R_0	BY945003
E_FL_e100_17C19_F_0	BY941986
E_FL_e100_17D01_F_0	BY941987
E_FL_e100_17D03_F_0	BY941988
E_FL_e100_17D04_F_0	BY941989
E_FL_e100_17D04_R_0	BY945004
E_FL_e100_17D05_F_0	BY941990
E_FL_e100_17D07_F_0	BY941991
E_FL_e100_17D08_F_0	BY941992
E_FL_e100_17D13_F_0	BY941993
E_FL_e100_17D14_F_0	BY941994
E_FL_e100_17D14_R_0	BY945005
E_FL_e100_17D16_F_0	BY941995
E_FL_e100_17D16_R_0	BY945006
E_FL_e100_17D21_F_0	BY941996
E_FL_e100_17D21_R_0	BY945007
E_FL_e100_17D22_F_0	BY941997
E_FL_e100_17D23_F_0	BY941998
E_FL_e100_17D23_R_0	BY945008
E_FL_e100_17D24_F_0	BY941999
E_FL_e100_17E01_F_0	BY942000
E_FL_e100_17E02_R_0	BY945009
E_FL_e100_17E06_F_0	BY942001
E_FL_e100_17E06_R_0	BY945010
E_FL_e100_17E10_R_0	BY945011
E_FL_e100_17E14_R_0	BY945012
E_FL_e100_17E15_R_0	BY945013
E_FL_e100_17E18_F_0	BY942002
E_FL_e100_17E19_F_0	BY942003
E_FL_e100_17E20_F_0	BY942004

E_FL_e100_17E23_F_0	BY942005
E_FL_e100_17E23_R_0	BY945014
E_FL_e100_17E24_F_0	BY942006
E_FL_e100_17F01_F_0	BY942007
E_FL_e100_17F02_F_0	BY942008
E_FL_e100_17F02_R_0	BY945015
E_FL_e100_17F03_F_0	BY942009
E_FL_e100_17F04_F_0	BY942010
E_FL_e100_17F07_F_0	BY942011
E_FL_e100_17F12_R_0	BY945016
E_FL_e100_17F13_F_0	BY942012
E_FL_e100_17F14_F_0	BY942013
E_FL_e100_17F15_F_0	BY942014
E_FL_e100_17F17_F_0	BY942015
E_FL_e100_17F17_R_0	BY945017
E_FL_e100_17F18_F_0	BY942016
E_FL_e100_17F18_R_0	BY945018
E_FL_e100_17F20_F_0	BY942017
E_FL_e100_17F20_R_0	BY945019
E_FL_e100_17F21_F_0	BY942018
E_FL_e100_17F23_F_0	BY942019
E_FL_e100_17F23_R_0	BY945020
E_FL_e100_17F24_R_0	BY945021
E_FL_e100_17G15_R_0	BY945022
E_FL_e100_17G19_F_0	BY942020
E_FL_e100_17G23_F_0	BY942021
E_FL_e100_17G23_R_0	BY945023
E_FL_e100_17G24_F_0	BY942022
E_FL_e100_17H01_F_0	BY942023
E_FL_e100_17H03_R_0	BY945024
E_FL_e100_17H07_F_0	BY942024
E_FL_e100_17H13_F_0	BY942025
E_FL_e100_17H14_F_0	BY942026
E_FL_e100_17H14_R_0	BY945025
E_FL_e100_17H16_F_0	BY942027
E_FL_e100_17H19_F_0	BY942028
E_FL_e100_17H19_R_0	BY945026
E_FL_e100_17H24_F_0	BY942029
E_FL_e100_17H24_R_0	BY945027
E_FL_e100_17I03_R_0	BY945028
E_FL_e100_17I10_F_0	BY942030
E_FL_e100_17I10_R_0	BY945029
E_FL_e100_17I11_F_0	BY942031
E_FL_e100_17I11_R_0	BY945030
E_FL_e100_17I17_F_0	BY942032
E_FL_e100_17I17_R_0	BY945031
E_FL_e100_17I18_R_0	BY945032
E_FL_e100_17I20_F_0	BY942033
E_FL_e100_17I21_F_0	BY942034
E_FL_e100_17I21_R_0	BY945033
E_FL_e100_17I24_F_0	BY942035
E_FL_e100_17J01_F_0	BY942036
E_FL_e100_17J01_R_0	BY945034
E_FL_e100_17J02_R_0	BY945035

E_FL_e100_17J04_F_0	BY942037
E_FL_e100_17J04_R_0	BY945036
E_FL_e100_17J05_R_0	BY945037
E_FL_e100_17J08_F_0	BY942038
E_FL_e100_17J08_R_0	BY945038
E_FL_e100_17J11_R_0	BY945039
E_FL_e100_17J14_F_0	BY942039
E_FL_e100_17J14_R_0	BY945040
E_FL_e100_17J18_F_0	BY942040
E_FL_e100_17J18_R_0	BY945041
E_FL_e100_17J19_F_0	BY942041
E_FL_e100_17J20_F_0	BY942042
E_FL_e100_17J20_R_0	BY945042
E_FL_e100_17J23_R_0	BY945043
E_FL_e100_17K08_R_0	BY945044
E_FL_e100_17K09_F_0	BY942043
E_FL_e100_17K16_F_0	BY942044
E_FL_e100_17K17_F_0	BY942045
E_FL_e100_17K17_R_0	BY945045
E_FL_e100_17K21_F_0	BY942046
E_FL_e100_17K24_R_0	BY945046
E_FL_e100_17L06_R_0	BY945047
E_FL_e100_17L11_F_0	BY942047
E_FL_e100_17L11_R_0	BY945048
E_FL_e100_17L13_F_0	BY942048
E_FL_e100_17L13_R_0	BY945049
E_FL_e100_17L15_F_0	BY942049
E_FL_e100_17L19_F_0	BY942050
E_FL_e100_17L19_R_0	BY945050
E_FL_e100_17L20_F_0	BY942051
E_FL_e100_17L20_R_0	BY945051
E_FL_e100_17M08_R_0	BY945052
E_FL_e100_17M16_F_0	BY942052
E_FL_e100_17M17_F_0	BY942053
E_FL_e100_17M17_R_0	BY945053
E_FL_e100_17M20_F_0	BY942054
E_FL_e100_17M22_F_0	BY942055
E_FL_e100_17M23_R_0	BY945054
E_FL_e100_17N02_F_0	BY942056
E_FL_e100_17N02_R_0	BY945055
E_FL_e100_17N03_F_0	BY942057
E_FL_e100_17N05_R_0	BY945056
E_FL_e100_17N06_R_0	BY945057
E_FL_e100_17N07_F_0	BY942058
E_FL_e100_17N08_F_0	BY942059
E_FL_e100_17N08_R_0	BY945058
E_FL_e100_17N12_R_0	BY945059
E_FL_e100_17N17_F_0	BY942060
E_FL_e100_17N17_R_0	BY945060
E_FL_e100_17N19_F_0	BY942061
E_FL_e100_17N20_R_0	BY945061
E_FL_e100_17N21_F_0	BY942062
E_FL_e100_17N22_F_0	BY942063
E_FL_e100_17O03_F_0	BY942064

E_FL_e100_17O03_R_0	BY945062
E_FL_e100_17O05_F_0	BY942065
E_FL_e100_17O06_F_0	BY942066
E_FL_e100_17O08_R_0	BY945063
E_FL_e100_17O09_F_0	BY942067
E_FL_e100_17O12_F_0	BY942068
E_FL_e100_17O12_R_0	BY945064
E_FL_e100_17O13_R_0	BY945065
E_FL_e100_17O17_R_0	BY945066
E_FL_e100_17O20_R_0	BY945067
E_FL_e100_17O22_F_0	BY942069
E_FL_e100_17O22_R_0	BY945068
E_FL_e100_17P05_F_0	BY942070
E_FL_e100_17P05_R_0	BY945069
E_FL_e100_17P06_F_0	BY942071
E_FL_e100_17P07_F_0	BY942072
E_FL_e100_17P09_F_0	BY942073
E_FL_e100_17P09_R_0	BY945070
E_FL_e100_17P11_F_0	BY942074
E_FL_e100_17P12_F_0	BY942075
E_FL_e100_17P17_F_0	BY942076
E_FL_e100_17P17_R_0	BY945071
E_FL_e100_17P18_F_0	BY942077
E_FL_e100_17P18_R_0	BY945072
E_FL_e100_17P21_F_0	BY942078
E_FL_e100_17P21_R_0	BY945073
E_FL_e100_17P23_R_0	BY945074
E_FL_e100_17P24_F_0	BY942079
E_FL_e100_18A03_F_0	BY942080
E_FL_e100_18A05_F_0	BY942081
E_FL_e100_18A05_R_0	BY945075
E_FL_e100_18A07_R_0	BY945076
E_FL_e100_18A08_F_0	BY942082
E_FL_e100_18A08_R_0	BY945077
E_FL_e100_18A10_F_0	BY942083
E_FL_e100_18A16_R_0	BY945078
E_FL_e100_18A18_F_0	BY942084
E_FL_e100_18A18_R_0	BY945079
E_FL_e100_18A19_F_0	BY942085
E_FL_e100_18A19_R_0	BY945080
E_FL_e100_18A20_F_0	BY942086
E_FL_e100_18A21_R_0	BY945081
E_FL_e100_18A23_F_0	BY942087
E_FL_e100_18A23_R_0	BY945082
E_FL_e100_18A24_F_0	BY942088
E_FL_e100_18B03_F_0	BY942089
E_FL_e100_18B07_F_0	BY942090
E_FL_e100_18B11_R_0	BY945083
E_FL_e100_18B12_F_0	BY942091
E_FL_e100_18B15_F_0	BY942092
E_FL_e100_18B15_R_0	BY945084
E_FL_e100_18B16_F_0	BY942093
E_FL_e100_18B17_F_0	BY942094
E_FL_e100_18B17_R_0	BY945085

E_FL_e100_18B19_F_0	BY942095
E_FL_e100_18B19_R_0	BY945086
E_FL_e100_18B20_F_0	BY942096
E_FL_e100_18B21_F_0	BY942097
E_FL_e100_18B21_R_0	BY945087
E_FL_e100_18B24_F_0	BY942098
E_FL_e100_18C10_F_0	BY942099
E_FL_e100_18C10_R_0	BY945088
E_FL_e100_18C12_F_0	BY942100
E_FL_e100_18C18_F_0	BY942101
E_FL_e100_18C19_R_0	BY945089
E_FL_e100_18C20_F_0	BY942102
E_FL_e100_18C20_R_0	BY945090
E_FL_e100_18C22_F_0	BY942103
E_FL_e100_18C24_F_0	BY942104
E_FL_e100_18D01_F_0	BY942105
E_FL_e100_18D04_F_0	BY942106
E_FL_e100_18D04_R_0	BY945091
E_FL_e100_18D06_F_0	BY942107
E_FL_e100_18D06_R_0	BY945092
E_FL_e100_18D07_R_0	BY945093
E_FL_e100_18D09_F_0	BY942108
E_FL_e100_18D09_R_0	BY945094
E_FL_e100_18D10_R_0	BY945095
E_FL_e100_18D11_F_0	BY942109
E_FL_e100_18D13_F_0	BY942110
E_FL_e100_18D13_R_0	BY945096
E_FL_e100_18D15_F_0	BY942111
E_FL_e100_18D15_R_0	BY945097
E_FL_e100_18D19_F_0	BY942112
E_FL_e100_18D20_F_0	BY942113
E_FL_e100_18D20_R_0	BY945098
E_FL_e100_18D22_R_0	BY945099
E_FL_e100_18D23_F_0	BY942114
E_FL_e100_18D23_R_0	BY945100
E_FL_e100_18E02_F_0	BY942115
E_FL_e100_18E03_R_0	BY945101
E_FL_e100_18E04_F_0	BY942116
E_FL_e100_18E05_F_0	BY942117
E_FL_e100_18E06_F_0	BY942118
E_FL_e100_18E06_R_0	BY945102
E_FL_e100_18E07_F_0	BY942119
E_FL_e100_18E07_R_0	BY945103
E_FL_e100_18E08_F_0	BY942120
E_FL_e100_18E08_R_0	BY945104
E_FL_e100_18E11_F_0	BY942121
E_FL_e100_18E12_F_0	BY942122
E_FL_e100_18E12_R_0	BY945105
E_FL_e100_18E13_R_0	BY945106
E_FL_e100_18E15_R_0	BY945107
E_FL_e100_18E17_F_0	BY942123
E_FL_e100_18E17_R_0	BY945108
E_FL_e100_18E20_F_0	BY942124
E_FL_e100_18E21_F_0	BY942125

E_FL_e100_18E21_R_0	BY945109
E_FL_e100_18E22_F_0	BY942126
E_FL_e100_18E22_R_0	BY945110
E_FL_e100_18E23_F_0	BY942127
E_FL_e100_18F01_F_0	BY942128
E_FL_e100_18F01_R_0	BY945111
E_FL_e100_18F03_F_0	BY942129
E_FL_e100_18F03_R_0	BY945112
E_FL_e100_18F04_F_0	BY942130
E_FL_e100_18F04_R_0	BY945113
E_FL_e100_18F06_F_0	BY942131
E_FL_e100_18F06_R_0	BY945114
E_FL_e100_18F08_F_0	BY942132
E_FL_e100_18F08_R_0	BY945115
E_FL_e100_18F09_F_0	BY942133
E_FL_e100_18F09_R_0	BY945116
E_FL_e100_18F13_F_0	BY942134
E_FL_e100_18F14_F_0	BY942135
E_FL_e100_18F18_R_0	BY945117
E_FL_e100_18F19_R_0	BY945118
E_FL_e100_18F22_F_0	BY942136
E_FL_e100_18F22_R_0	BY945119
E_FL_e100_18F23_F_0	BY942137
E_FL_e100_18F23_R_0	BY945120
E_FL_e100_18F24_F_0	BY942138
E_FL_e100_18F24_R_0	BY945121
E_FL_e100_18G02_F_0	BY942139
E_FL_e100_18G02_R_0	BY945122
E_FL_e100_18G03_F_0	BY942140
E_FL_e100_18G04_F_0	BY942141
E_FL_e100_18G04_R_0	BY945123
E_FL_e100_18G05_F_0	BY942142
E_FL_e100_18G05_R_0	BY945124
E_FL_e100_18G06_F_0	BY942143
E_FL_e100_18G08_F_0	BY942144
E_FL_e100_18G08_R_0	BY945125
E_FL_e100_18G13_R_0	BY945126
E_FL_e100_18G20_F_0	BY942145
E_FL_e100_18G20_R_0	BY945127
E_FL_e100_18G21_F_0	BY942146
E_FL_e100_18H01_F_0	BY942147
E_FL_e100_18H01_R_0	BY945128
E_FL_e100_18H02_F_0	BY942148
E_FL_e100_18H02_R_0	BY945129
E_FL_e100_18H05_F_0	BY942149
E_FL_e100_18H07_F_0	BY942150
E_FL_e100_18H10_F_0	BY942151
E_FL_e100_18H10_R_0	BY945130
E_FL_e100_18H14_F_0	BY942152
E_FL_e100_18H19_F_0	BY942153
E_FL_e100_18H23_F_0	BY942154
E_FL_e100_18H24_F_0	BY942155
E_FL_e100_18H24_R_0	BY945131
E_FL_e100_18I02_F_0	BY942156

E_FL_e100_18I05_F_0	BY942157
E_FL_e100_18I05_R_0	BY945132
E_FL_e100_18I07_F_0	BY942158
E_FL_e100_18I10_F_0	BY942159
E_FL_e100_18I15_R_0	BY945133
E_FL_e100_18I16_R_0	BY945134
E_FL_e100_18I21_F_0	BY942160
E_FL_e100_18I21_R_0	BY945135
E_FL_e100_18I22_F_0	BY942161
E_FL_e100_18I22_R_0	BY945136
E_FL_e100_18I23_F_0	BY942162
E_FL_e100_18I23_R_0	BY945137
E_FL_e100_18J01_F_0	BY942163
E_FL_e100_18J04_F_0	BY942164
E_FL_e100_18J04_R_0	BY945138
E_FL_e100_18J05_F_0	BY942165
E_FL_e100_18J05_R_0	BY945139
E_FL_e100_18J07_F_0	BY942166
E_FL_e100_18J07_R_0	BY945140
E_FL_e100_18J08_F_0	BY942167
E_FL_e100_18J09_F_0	BY942168
E_FL_e100_18J13_F_0	BY942169
E_FL_e100_18J14_F_0	BY942170
E_FL_e100_18J14_R_0	BY945141
E_FL_e100_18J16_F_0	BY942171
E_FL_e100_18J17_F_0	BY942172
E_FL_e100_18J18_F_0	BY942173
E_FL_e100_18J21_R_0	BY945142
E_FL_e100_18J23_F_0	BY942174
E_FL_e100_18J23_R_0	BY945143
E_FL_e100_18K01_F_0	BY942175
E_FL_e100_18K01_R_0	BY945144
E_FL_e100_18K04_F_0	BY942176
E_FL_e100_18K04_R_0	BY945145
E_FL_e100_18K05_R_0	BY945146
E_FL_e100_18K07_F_0	BY942177
E_FL_e100_18K07_R_0	BY945147
E_FL_e100_18K08_F_0	BY942178
E_FL_e100_18K08_R_0	BY945148
E_FL_e100_18K09_R_0	BY945149
E_FL_e100_18K10_F_0	BY942179
E_FL_e100_18K10_R_0	BY945150
E_FL_e100_18K15_F_0	BY942180
E_FL_e100_18K16_F_0	BY942181
E_FL_e100_18K16_R_0	BY945151
E_FL_e100_18K21_F_0	BY942182
E_FL_e100_18K22_F_0	BY942183
E_FL_e100_18K23_F_0	BY942184
E_FL_e100_18K23_R_0	BY945152
E_FL_e100_18L01_F_0	BY942185
E_FL_e100_18L03_F_0	BY942186
E_FL_e100_18L05_F_0	BY942187
E_FL_e100_18L06_R_0	BY945153
E_FL_e100_18L08_F_0	BY942188

E_FL_e100_18L11_F_0	BY942189
E_FL_e100_18L11_R_0	BY945154
E_FL_e100_18L13_F_0	BY942190
E_FL_e100_18L19_F_0	BY942191
E_FL_e100_18L20_F_0	BY942192
E_FL_e100_18L20_R_0	BY945155
E_FL_e100_18L23_F_0	BY942193
E_FL_e100_18M01_F_0	BY942194
E_FL_e100_18M01_R_0	BY945156
E_FL_e100_18M03_F_0	BY942195
E_FL_e100_18M03_R_0	BY945157
E_FL_e100_18M04_F_0	BY942196
E_FL_e100_18M05_R_0	BY945158
E_FL_e100_18M08_F_0	BY942197
E_FL_e100_18M11_F_0	BY942198
E_FL_e100_18M12_F_0	BY942199
E_FL_e100_18M13_R_0	BY945159
E_FL_e100_18M15_R_0	BY945160
E_FL_e100_18M18_F_0	BY942200
E_FL_e100_18M20_F_0	BY942201
E_FL_e100_18M20_R_0	BY945161
E_FL_e100_18M21_F_0	BY942202
E_FL_e100_18M21_R_0	BY945162
E_FL_e100_18M22_F_0	BY942203
E_FL_e100_18M23_F_0	BY942204
E_FL_e100_18M23_R_0	BY945163
E_FL_e100_18N01_F_0	BY942205
E_FL_e100_18N03_F_0	BY942206
E_FL_e100_18N03_R_0	BY945164
E_FL_e100_18N04_R_0	BY945165
E_FL_e100_18N06_R_0	BY945166
E_FL_e100_18N07_F_0	BY942207
E_FL_e100_18N08_F_0	BY942208
E_FL_e100_18N08_R_0	BY945167
E_FL_e100_18N09_F_0	BY942209
E_FL_e100_18N09_R_0	BY945168
E_FL_e100_18N10_F_0	BY942210
E_FL_e100_18N10_R_0	BY945169
E_FL_e100_18N11_F_0	BY942211
E_FL_e100_18N11_R_0	BY945170
E_FL_e100_18N12_R_0	BY945171
E_FL_e100_18N14_F_0	BY942212
E_FL_e100_18N14_R_0	BY945172
E_FL_e100_18N15_F_0	BY942213
E_FL_e100_18N17_F_0	BY942214
E_FL_e100_18N18_R_0	BY945173
E_FL_e100_18N19_F_0	BY942215
E_FL_e100_18N20_F_0	BY942216
E_FL_e100_18N20_R_0	BY945174
E_FL_e100_18O01_F_0	BY942217
E_FL_e100_18O01_R_0	BY945175
E_FL_e100_18O02_F_0	BY942218
E_FL_e100_18O02_R_0	BY945176
E_FL_e100_18O04_F_0	BY942219

E_FL_e100_18O12_F_0	BY942220
E_FL_e100_18O14_R_0	BY945177
E_FL_e100_18O16_F_0	BY942221
E_FL_e100_18O16_R_0	BY945178
E_FL_e100_18O17_F_0	BY942222
E_FL_e100_18O21_F_0	BY942223
E_FL_e100_18O22_F_0	BY942224
E_FL_e100_18O23_F_0	BY942225
E_FL_e100_18O24_F_0	BY942226
E_FL_e100_18P01_F_0	BY942227
E_FL_e100_18P02_F_0	BY942228
E_FL_e100_18P02_R_0	BY945179
E_FL_e100_18P03_F_0	BY942229
E_FL_e100_18P05_F_0	BY942230
E_FL_e100_18P10_F_0	BY942231
E_FL_e100_18P12_F_0	BY942232
E_FL_e100_18P12_R_0	BY945180
E_FL_e100_18P13_F_0	BY942233
E_FL_e100_18P13_R_0	BY945181
E_FL_e100_18P14_F_0	BY942234
E_FL_e100_18P15_F_0	BY942235
E_FL_e100_18P15_R_0	BY945182
E_FL_e100_18P21_F_0	BY942236
E_FL_e100_18P21_R_0	BY945183
E_FL_e100_18P22_F_0	BY942237
E_FL_e100_18P22_R_0	BY945184
E_FL_e100_19A02_F_0	BY942238
E_FL_e100_19A03_F_0	BY942239
E_FL_e100_19A04_F_0	BY942240
E_FL_e100_19A05_F_0	BY942241
E_FL_e100_19A05_R_0	BY945185
E_FL_e100_19A08_F_0	BY942242
E_FL_e100_19A08_R_0	BY945186
E_FL_e100_19A10_F_0	BY942243
E_FL_e100_19A10_R_0	BY945187
E_FL_e100_19A11_F_0	BY942244
E_FL_e100_19A11_R_0	BY945188
E_FL_e100_19A13_R_0	BY945189
E_FL_e100_19A14_R_0	BY945190
E_FL_e100_19A19_F_0	BY942245
E_FL_e100_19A19_R_0	BY945191
E_FL_e100_19A22_F_0	BY942246
E_FL_e100_19A24_R_0	BY945192
E_FL_e100_19B01_F_0	BY942247
E_FL_e100_19B01_R_0	BY945193
E_FL_e100_19B03_F_0	BY942248
E_FL_e100_19B03_R_0	BY945194
E_FL_e100_19B05_F_0	BY942249
E_FL_e100_19B05_R_0	BY945195
E_FL_e100_19B08_F_0	BY942250
E_FL_e100_19B08_R_0	BY945196
E_FL_e100_19B12_F_0	BY942251
E_FL_e100_19B12_R_0	BY945197
E_FL_e100_19B14_F_0	BY942252

E_FL_e100_19B15_R_0	BY945198
E_FL_e100_19B16_R_0	BY945199
E_FL_e100_19B17_F_0	BY942253
E_FL_e100_19B17_R_0	BY945200
E_FL_e100_19B19_F_0	BY942254
E_FL_e100_19B20_R_0	BY945201
E_FL_e100_19B22_F_0	BY942255
E_FL_e100_19C01_F_0	BY942256
E_FL_e100_19C02_F_0	BY942257
E_FL_e100_19C02_R_0	BY945202
E_FL_e100_19C03_F_0	BY942258
E_FL_e100_19C04_F_0	BY942259
E_FL_e100_19C06_F_0	BY942260
E_FL_e100_19C08_F_0	BY942261
E_FL_e100_19C10_F_0	BY942262
E_FL_e100_19C16_F_0	BY942263
E_FL_e100_19C18_F_0	BY942264
E_FL_e100_19C19_F_0	BY942265
E_FL_e100_19C19_R_0	BY945203
E_FL_e100_19C21_F_0	BY942266
E_FL_e100_19C21_R_0	BY945204
E_FL_e100_19C22_F_0	BY942267
E_FL_e100_19D02_F_0	BY942268
E_FL_e100_19D06_F_0	BY942269
E_FL_e100_19D06_R_0	BY945205
E_FL_e100_19D08_F_0	BY942270
E_FL_e100_19D08_R_0	BY945206
E_FL_e100_19D09_F_0	BY942271
E_FL_e100_19D09_R_0	BY945207
E_FL_e100_19D10_R_0	BY945208
E_FL_e100_19D15_F_0	BY942272
E_FL_e100_19D15_R_0	BY945209
E_FL_e100_19D17_R_0	BY945210
E_FL_e100_19D18_F_0	BY942273
E_FL_e100_19D18_R_0	BY945211
E_FL_e100_19D20_F_0	BY942274
E_FL_e100_19E01_F_0	BY942275
E_FL_e100_19E02_F_0	BY942276
E_FL_e100_19E02_R_0	BY945212
E_FL_e100_19E03_F_0	BY942277
E_FL_e100_19E04_F_0	BY942278
E_FL_e100_19E04_R_0	BY945213
E_FL_e100_19E06_F_0	BY942279
E_FL_e100_19E06_R_0	BY945214
E_FL_e100_19E08_R_0	BY945215
E_FL_e100_19E10_F_0	BY942280
E_FL_e100_19E10_R_0	BY945216
E_FL_e100_19E13_F_0	BY942281
E_FL_e100_19E14_R_0	BY945217
E_FL_e100_19E18_F_0	BY942282
E_FL_e100_19E19_F_0	BY942283
E_FL_e100_19E21_F_0	BY942284
E_FL_e100_19E22_F_0	BY942285
E_FL_e100_19F03_F_0	BY942286

E_FL_e100_19F03_R_0	BY945218
E_FL_e100_19F07_F_0	BY942287
E_FL_e100_19F10_F_0	BY942288
E_FL_e100_19F13_F_0	BY942289
E_FL_e100_19F14_F_0	BY942290
E_FL_e100_19F15_R_0	BY945219
E_FL_e100_19F19_F_0	BY942291
E_FL_e100_19F22_R_0	BY945220
E_FL_e100_19F23_F_0	BY942292
E_FL_e100_19F23_R_0	BY945221
E_FL_e100_19F24_F_0	BY942293
E_FL_e100_19G03_F_0	BY942294
E_FL_e100_19G05_F_0	BY942295
E_FL_e100_19G05_R_0	BY945222
E_FL_e100_19G09_F_0	BY942296
E_FL_e100_19G09_R_0	BY945223
E_FL_e100_19G11_F_0	BY942297
E_FL_e100_19G12_F_0	BY942298
E_FL_e100_19G16_F_0	BY942299
E_FL_e100_19G22_F_0	BY942300
E_FL_e100_19G23_F_0	BY942301
E_FL_e100_19G23_R_0	BY945224
E_FL_e100_19G24_F_0	BY942302
E_FL_e100_19G24_R_0	BY945225
E_FL_e100_19H03_R_0	BY945226
E_FL_e100_19H06_F_0	BY942303
E_FL_e100_19H07_F_0	BY942304
E_FL_e100_19H07_R_0	BY945227
E_FL_e100_19H11_F_0	BY942305
E_FL_e100_19H11_R_0	BY945228
E_FL_e100_19H13_F_0	BY942306
E_FL_e100_19H15_F_0	BY942307
E_FL_e100_19H15_R_0	BY945229
E_FL_e100_19H16_F_0	BY942308
E_FL_e100_19H19_F_0	BY942309
E_FL_e100_19H23_F_0	BY942310
E_FL_e100_19I02_F_0	BY942311
E_FL_e100_19I02_R_0	BY945230
E_FL_e100_19I03_F_0	BY942312
E_FL_e100_19I06_R_0	BY945231
E_FL_e100_19I07_F_0	BY942313
E_FL_e100_19I12_F_0	BY942314
E_FL_e100_19I12_R_0	BY943280
E_FL_e100_19I15_F_0	BY938971
E_FL_e100_19I15_R_0	BY943281
E_FL_e100_19I18_F_0	BY938972
E_FL_e100_19I19_F_0	BY938973
E_FL_e100_19I21_F_0	BY938974
E_FL_e100_19I21_R_0	BY943282
E_FL_e100_19I22_F_0	BY938975
E_FL_e100_19I22_R_0	BY943283
E_FL_e100_19J10_R_0	BY943284
E_FL_e100_19J12_F_0	BY938976
E_FL_e100_19J13_F_0	BY938977

E_FL_e100_19J16_R_0	BY943285
E_FL_e100_19J19_F_0	BY938978
E_FL_e100_19J23_F_0	BY938979
E_FL_e100_19J23_R_0	BY943286
E_FL_e100_19K03_F_0	BY938980
E_FL_e100_19K03_R_0	BY943287
E_FL_e100_19K05_F_0	BY938981
E_FL_e100_19K05_R_0	BY943288
E_FL_e100_19K07_F_0	BY938982
E_FL_e100_19K10_F_0	BY938983
E_FL_e100_19K11_F_0	BY938984
E_FL_e100_19K11_R_0	BY943289
E_FL_e100_19K14_F_0	BY938985
E_FL_e100_19K14_R_0	BY943290
E_FL_e100_19K15_R_0	BY943291
E_FL_e100_19K19_F_0	BY938986
E_FL_e100_19K20_F_0	BY938987
E_FL_e100_19K21_R_0	BY943292
E_FL_e100_19K22_R_0	BY943293
E_FL_e100_19K23_F_0	BY938988
E_FL_e100_19K23_R_0	BY943294
E_FL_e100_19L03_F_0	BY938989
E_FL_e100_19L05_F_0	BY938990
E_FL_e100_19L07_F_0	BY938991
E_FL_e100_19L07_R_0	BY943295
E_FL_e100_19L10_F_0	BY938992
E_FL_e100_19L10_R_0	BY943296
E_FL_e100_19L13_R_0	BY943297
E_FL_e100_19L15_F_0	BY938993
E_FL_e100_19L19_F_0	BY938994
E_FL_e100_19L20_F_0	BY938995
E_FL_e100_19L20_R_0	BY943298
E_FL_e100_19L21_F_0	BY938996
E_FL_e100_19L21_R_0	BY943299
E_FL_e100_19L23_F_0	BY938997
E_FL_e100_19M01_F_0	BY938998
E_FL_e100_19M02_F_0	BY938999
E_FL_e100_19M02_R_0	BY943300
E_FL_e100_19M03_F_0	BY939000
E_FL_e100_19M03_R_0	BY943301
E_FL_e100_19M04_R_0	BY943302
E_FL_e100_19M05_F_0	BY939001
E_FL_e100_19M06_F_0	BY939002
E_FL_e100_19M08_F_0	BY939003
E_FL_e100_19M08_R_0	BY943303
E_FL_e100_19M11_F_0	BY939004
E_FL_e100_19M11_R_0	BY943304
E_FL_e100_19M14_R_0	BY943305
E_FL_e100_19M16_F_0	BY939005
E_FL_e100_19M16_R_0	BY943306
E_FL_e100_19M20_R_0	BY943307
E_FL_e100_19M21_F_0	BY939006
E_FL_e100_19M21_R_0	BY943308
E_FL_e100_19N01_F_0	BY939007

E_FL_e100_19N01_R_0	BY943309
E_FL_e100_19N02_F_0	BY939008
E_FL_e100_19N02_R_0	BY943310
E_FL_e100_19N06_R_0	BY943311
E_FL_e100_19N07_F_0	BY939009
E_FL_e100_19N07_R_0	BY943312
E_FL_e100_19N08_F_0	BY939010
E_FL_e100_19N08_R_0	BY943313
E_FL_e100_19N09_F_0	BY939011
E_FL_e100_19N13_F_0	BY939012
E_FL_e100_19N14_F_0	BY939013
E_FL_e100_19N14_R_0	BY943314
E_FL_e100_19N16_F_0	BY939014
E_FL_e100_19N20_F_0	BY939015
E_FL_e100_19N21_F_0	BY939016
E_FL_e100_19N21_R_0	BY943315
E_FL_e100_19N23_F_0	BY939017
E_FL_e100_19O01_F_0	BY939018
E_FL_e100_19O01_R_0	BY943316
E_FL_e100_19O02_F_0	BY939019
E_FL_e100_19O03_R_0	BY943317
E_FL_e100_19O04_F_0	BY939020
E_FL_e100_19O07_F_0	BY939021
E_FL_e100_19O10_F_0	BY939022
E_FL_e100_19O10_R_0	BY943318
E_FL_e100_19O12_F_0	BY939023
E_FL_e100_19O14_F_0	BY939024
E_FL_e100_19O14_R_0	BY943319
E_FL_e100_19O16_F_0	BY939025
E_FL_e100_19O19_F_0	BY939026
E_FL_e100_19O19_R_0	BY943320
E_FL_e100_19O22_F_0	BY939027
E_FL_e100_19O24_F_0	BY939028
E_FL_e100_19P01_F_0	BY939029
E_FL_e100_19P01_R_0	BY943321
E_FL_e100_19P03_F_0	BY939030
E_FL_e100_19P03_R_0	BY943322
E_FL_e100_19P07_F_0	BY939031
E_FL_e100_19P08_F_0	BY939032
E_FL_e100_19P08_R_0	BY943323
E_FL_e100_19P09_F_0	BY939033
E_FL_e100_19P09_R_0	BY943324
E_FL_e100_19P10_F_0	BY939034
E_FL_e100_19P11_F_0	BY939035
E_FL_e100_19P11_R_0	BY943325
E_FL_e100_19P13_F_0	BY939036
E_FL_e100_19P13_R_0	BY943326
E_FL_e100_19P16_R_0	BY943327
E_FL_e100_19P17_F_0	BY939037
E_FL_e100_19P17_R_0	BY943328
E_FL_e100_19P18_F_0	BY939038
E_FL_e100_19P19_F_0	BY939039
E_FL_e100_19P21_F_0	BY939040
E_FL_e100_19P23_F_0	BY939041

E_FL_e100_19P24_F_0	BY939042
E_FL_e100_20A01_F_0	BY939043
E_FL_e100_20A01_R_0	BY943329
E_FL_e100_20A07_F_0	BY939044
E_FL_e100_20A07_R_0	BY943330
E_FL_e100_20A08_R_0	BY943331
E_FL_e100_20A10_F_0	BY939045
E_FL_e100_20A10_R_0	BY943332
E_FL_e100_20A12_F_0	BY939046
E_FL_e100_20A12_R_0	BY943333
E_FL_e100_20A13_F_0	BY939047
E_FL_e100_20A13_R_0	BY943334
E_FL_e100_20A14_R_0	BY943335
E_FL_e100_20A17_F_0	BY939048
E_FL_e100_20A17_R_0	BY943336
E_FL_e100_20A18_F_0	BY939049
E_FL_e100_20A23_F_0	BY939050
E_FL_e100_20B01_F_0	BY939051
E_FL_e100_20B02_F_0	BY939052
E_FL_e100_20B02_R_0	BY943337
E_FL_e100_20B03_F_0	BY939053
E_FL_e100_20B06_F_0	BY939054
E_FL_e100_20B06_R_0	BY943338
E_FL_e100_20B07_F_0	BY939055
E_FL_e100_20B08_F_0	BY939056
E_FL_e100_20B09_F_0	BY939057
E_FL_e100_20B12_F_0	BY939058
E_FL_e100_20B17_F_0	BY939059
E_FL_e100_20B17_R_0	BY943339
E_FL_e100_20B18_F_0	BY939060
E_FL_e100_20B22_F_0	BY939061
E_FL_e100_20B22_R_0	BY943340
E_FL_e100_20B23_F_0	BY939062
E_FL_e100_20B23_R_0	BY943341
E_FL_e100_20C01_F_0	BY939063
E_FL_e100_20C03_F_0	BY939064
E_FL_e100_20C04_F_0	BY939065
E_FL_e100_20C06_F_0	BY939066
E_FL_e100_20C10_R_0	BY943342
E_FL_e100_20C11_F_0	BY939067
E_FL_e100_20C11_R_0	BY943343
E_FL_e100_20C12_F_0	BY939068
E_FL_e100_20C14_F_0	BY939069
E_FL_e100_20C14_R_0	BY943344
E_FL_e100_20C15_F_0	BY939070
E_FL_e100_20C16_F_0	BY939071
E_FL_e100_20C17_F_0	BY939072
E_FL_e100_20C18_F_0	BY939073
E_FL_e100_20C23_F_0	BY939074
E_FL_e100_20C23_R_0	BY943345
E_FL_e100_20C24_F_0	BY939075
E_FL_e100_20C24_R_0	BY943346
E_FL_e100_20D03_F_0	BY939076
E_FL_e100_20D03_R_0	BY943347

E_FL_e100_20D04_F_0	BY939077
E_FL_e100_20D13_F_0	BY939078
E_FL_e100_20D13_R_0	BY943348
E_FL_e100_20D14_F_0	BY939079
E_FL_e100_20D14_R_0	BY943349
E_FL_e100_20D18_F_0	BY939080
E_FL_e100_20D19_R_0	BY943350
E_FL_e100_20D20_F_0	BY939081
E_FL_e100_20D23_F_0	BY939082
E_FL_e100_20D24_F_0	BY939083
E_FL_e100_20E01_F_0	BY939084
E_FL_e100_20E03_F_0	BY939085
E_FL_e100_20E04_R_0	BY943351
E_FL_e100_20E06_R_0	BY943352
E_FL_e100_20E07_F_0	BY939086
E_FL_e100_20E07_R_0	BY943353
E_FL_e100_20E08_F_0	BY939087
E_FL_e100_20E09_F_0	BY939088
E_FL_e100_20E09_R_0	BY943354
E_FL_e100_20E10_F_0	BY939089
E_FL_e100_20E10_R_0	BY943355
E_FL_e100_20E11_F_0	BY939090
E_FL_e100_20E12_F_0	BY939091
E_FL_e100_20E13_F_0	BY939092
E_FL_e100_20E14_F_0	BY939093
E_FL_e100_20E14_R_0	BY943356
E_FL_e100_20E16_F_0	BY939094
E_FL_e100_20E17_R_0	BY943357
E_FL_e100_20E18_F_0	BY939095
E_FL_e100_20E19_F_0	BY939096
E_FL_e100_20E19_R_0	BY943358
E_FL_e100_20E20_R_0	BY943359
E_FL_e100_20E22_R_0	BY943360
E_FL_e100_20E23_F_0	BY939097
E_FL_e100_20E24_F_0	BY939098
E_FL_e100_20E24_R_0	BY943361
E_FL_e100_20F02_F_0	BY939099
E_FL_e100_20F05_F_0	BY939100
E_FL_e100_20F05_R_0	BY943362
E_FL_e100_20F07_F_0	BY939101
E_FL_e100_20F08_F_0	BY939102
E_FL_e100_20F08_R_0	BY943363
E_FL_e100_20F09_R_0	BY943364
E_FL_e100_20F10_F_0	BY939103
E_FL_e100_20F10_R_0	BY943365
E_FL_e100_20F11_F_0	BY939104
E_FL_e100_20F14_F_0	BY939105
E_FL_e100_20F15_R_0	BY943366
E_FL_e100_20F18_R_0	BY943367
E_FL_e100_20F19_F_0	BY939106
E_FL_e100_20F19_R_0	BY943368
E_FL_e100_20F23_F_0	BY939107
E_FL_e100_20F23_R_0	BY943369
E_FL_e100_20F24_F_0	BY939108

E_FL_e100_20F24_R_0	BY943370
E_FL_e100_20G01_F_0	BY939109
E_FL_e100_20G02_R_0	BY943371
E_FL_e100_20G11_F_0	BY939110
E_FL_e100_20G11_R_0	BY943372
E_FL_e100_20G12_F_0	BY939111
E_FL_e100_20G13_R_0	BY943373
E_FL_e100_20G14_F_0	BY939112
E_FL_e100_20G16_F_0	BY939113
E_FL_e100_20G18_F_0	BY939114
E_FL_e100_20G19_F_0	BY939115
E_FL_e100_20G19_R_0	BY943374
E_FL_e100_20G21_F_0	BY939116
E_FL_e100_20G23_R_0	BY943375
E_FL_e100_20H01_F_0	BY939117
E_FL_e100_20H02_F_0	BY939118
E_FL_e100_20H02_R_0	BY943376
E_FL_e100_20H03_F_0	BY939119
E_FL_e100_20H03_R_0	BY943377
E_FL_e100_20H04_R_0	BY943378
E_FL_e100_20H05_F_0	BY939120
E_FL_e100_20H05_R_0	BY943379
E_FL_e100_20H09_F_0	BY939121
E_FL_e100_20H09_R_0	BY943380
E_FL_e100_20H11_R_0	BY943381
E_FL_e100_20H14_F_0	BY939122
E_FL_e100_20H19_F_0	BY939123
E_FL_e100_20H20_F_0	BY939124
E_FL_e100_20H20_R_0	BY943382
E_FL_e100_20H21_F_0	BY939125
E_FL_e100_20H23_F_0	BY939126
E_FL_e100_20H23_R_0	BY943383
E_FL_e100_20H24_F_0	BY939127
E_FL_e100_20I01_R_0	BY943384
E_FL_e100_20I02_R_0	BY943385
E_FL_e100_20I03_F_0	BY939128
E_FL_e100_20I03_R_0	BY943386
E_FL_e100_20I04_R_0	BY943387
E_FL_e100_20I06_F_0	BY939129
E_FL_e100_20I06_R_0	BY943388
E_FL_e100_20I07_F_0	BY939130
E_FL_e100_20I09_F_0	BY939131
E_FL_e100_20I10_F_0	BY939132
E_FL_e100_20I10_R_0	BY943389
E_FL_e100_20I12_F_0	BY939133
E_FL_e100_20I13_F_0	BY939134
E_FL_e100_20I15_F_0	BY939135
E_FL_e100_20I15_R_0	BY943390
E_FL_e100_20I16_F_0	BY939136
E_FL_e100_20I17_F_0	BY939137
E_FL_e100_20I17_R_0	BY943391
E_FL_e100_20I18_F_0	BY939138
E_FL_e100_20I19_R_0	BY943392
E_FL_e100_20I21_F_0	BY939139

E_FL_e100_20I21_R_0	BY943393
E_FL_e100_20I22_F_0	BY939140
E_FL_e100_20I22_R_0	BY943394
E_FL_e100_20I24_F_0	BY939141
E_FL_e100_20I24_R_0	BY943395
E_FL_e100_20J01_F_0	BY939142
E_FL_e100_20J02_F_0	BY939143
E_FL_e100_20J03_F_0	BY939144
E_FL_e100_20J04_F_0	BY939145
E_FL_e100_20J04_R_0	BY943396
E_FL_e100_20J05_F_0	BY939146
E_FL_e100_20J05_R_0	BY943397
E_FL_e100_20J06_F_0	BY939147
E_FL_e100_20J06_R_0	BY943398
E_FL_e100_20J07_F_0	BY939148
E_FL_e100_20J07_R_0	BY943399
E_FL_e100_20J08_F_0	BY939149
E_FL_e100_20J12_F_0	BY939150
E_FL_e100_20J13_F_0	BY939151
E_FL_e100_20J13_R_0	BY943400
E_FL_e100_20J14_R_0	BY943401
E_FL_e100_20J20_F_0	BY939152
E_FL_e100_20J22_F_0	BY939153
E_FL_e100_20J22_R_0	BY943402
E_FL_e100_20J24_F_0	BY939154
E_FL_e100_20K04_F_0	BY939155
E_FL_e100_20K04_R_0	BY943403
E_FL_e100_20K09_F_0	BY939156
E_FL_e100_20K09_R_0	BY943404
E_FL_e100_20K11_F_0	BY939157
E_FL_e100_20K13_F_0	BY939158
E_FL_e100_20K13_R_0	BY943405
E_FL_e100_20K14_R_0	BY943406
E_FL_e100_20K16_R_0	BY943407
E_FL_e100_20L01_F_0	BY939159
E_FL_e100_20L01_R_0	BY943408
E_FL_e100_20L03_F_0	BY939160
E_FL_e100_20L05_F_0	BY939161
E_FL_e100_20L05_R_0	BY943409
E_FL_e100_20L07_F_0	BY939162
E_FL_e100_20L07_R_0	BY943410
E_FL_e100_20L08_F_0	BY939163
E_FL_e100_20L08_R_0	BY943411
E_FL_e100_20L11_F_0	BY939164
E_FL_e100_20L14_F_0	BY939165
E_FL_e100_20L15_F_0	BY939166
E_FL_e100_20L15_R_0	BY943412
E_FL_e100_20L16_F_0	BY939167
E_FL_e100_20L16_R_0	BY943413
E_FL_e100_20L19_F_0	BY939168
E_FL_e100_20L19_R_0	BY943414
E_FL_e100_20L20_F_0	BY939169
E_FL_e100_20L20_R_0	BY943415
E_FL_e100_20L21_F_0	BY939170

E_FL_e100_20L21_R_0	BY943416
E_FL_e100_20L23_F_0	BY939171
E_FL_e100_20L23_R_0	BY943417
E_FL_e100_20M05_F_0	BY939172
E_FL_e100_20M05_R_0	BY943418
E_FL_e100_20M06_F_0	BY939173
E_FL_e100_20M07_F_0	BY939174
E_FL_e100_20M08_F_0	BY939175
E_FL_e100_20M08_R_0	BY943419
E_FL_e100_20M10_R_0	BY943420
E_FL_e100_20M12_F_0	BY939176
E_FL_e100_20M14_F_0	BY939177
E_FL_e100_20M14_R_0	BY943421
E_FL_e100_20M15_F_0	BY939178
E_FL_e100_20M15_R_0	BY943422
E_FL_e100_20M16_F_0	BY939179
E_FL_e100_20M16_R_0	BY943423
E_FL_e100_20M17_F_0	BY939180
E_FL_e100_20M17_R_0	BY943424
E_FL_e100_20M19_R_0	BY943425
E_FL_e100_20M22_R_0	BY943426
E_FL_e100_20M24_F_0	BY939181
E_FL_e100_20N01_F_0	BY939182
E_FL_e100_20N01_R_0	BY943427
E_FL_e100_20N02_F_0	BY939183
E_FL_e100_20N02_R_0	BY943428
E_FL_e100_20N05_F_0	BY939184
E_FL_e100_20N05_R_0	BY943429
E_FL_e100_20N06_R_0	BY943430
E_FL_e100_20N07_F_0	BY939185
E_FL_e100_20N11_F_0	BY939186
E_FL_e100_20N11_R_0	BY943431
E_FL_e100_20N12_R_0	BY943432
E_FL_e100_20N13_F_0	BY939187
E_FL_e100_20N13_R_0	BY943433
E_FL_e100_20N17_F_0	BY939188
E_FL_e100_20N20_F_0	BY939189
E_FL_e100_20N20_R_0	BY943434
E_FL_e100_20N23_F_0	BY939190
E_FL_e100_20N24_R_0	BY943435
E_FL_e100_20O04_F_0	BY939191
E_FL_e100_20O04_R_0	BY943436
E_FL_e100_20O06_F_0	BY939192
E_FL_e100_20O09_F_0	BY939193
E_FL_e100_20O09_R_0	BY943437
E_FL_e100_20O13_F_0	BY939194
E_FL_e100_20O13_R_0	BY943438
E_FL_e100_20O14_F_0	BY939195
E_FL_e100_20O15_R_0	BY943439
E_FL_e100_20O16_F_0	BY939196
E_FL_e100_20O16_R_0	BY943440
E_FL_e100_20O17_R_0	BY943441
E_FL_e100_20O18_F_0	BY939197
E_FL_e100_20O23_F_0	BY939198

E_FL_e100_20O24_F_0	BY939199
E_FL_e100_20P01_F_0	BY939200
E_FL_e100_20P02_F_0	BY939201
E_FL_e100_20P02_R_0	BY943442
E_FL_e100_20P03_F_0	BY939202
E_FL_e100_20P04_F_0	BY939203
E_FL_e100_20P06_R_0	BY943443
E_FL_e100_20P07_F_0	BY939204
E_FL_e100_20P07_R_0	BY943444
E_FL_e100_20P08_F_0	BY939205
E_FL_e100_20P09_F_0	BY939206
E_FL_e100_20P12_F_0	BY939207
E_FL_e100_20P13_F_0	BY939208
E_FL_e100_20P13_R_0	BY943445
E_FL_e100_20P15_F_0	BY939209
E_FL_e100_20P18_F_0	BY939210
E_FL_e100_20P18_R_0	BY943446
E_FL_e100_20P19_F_0	BY939211
E_FL_e100_20P24_F_0	BY939212
E_FL_e100_21A01_F_0	BY939213
E_FL_e100_21A01_R_0	BY943447
E_FL_e100_21A03_F_0	BY939214
E_FL_e100_21A03_R_0	BY943448
E_FL_e100_21A05_F_0	BY939215
E_FL_e100_21A05_R_0	BY943449
E_FL_e100_21A06_F_0	BY939216
E_FL_e100_21A06_R_0	BY943450
E_FL_e100_21A07_F_0	BY939217
E_FL_e100_21A07_R_0	BY943451
E_FL_e100_21A10_R_0	BY943452
E_FL_e100_21A12_F_0	BY939218
E_FL_e100_21A13_F_0	BY939219
E_FL_e100_21A14_F_0	BY939220
E_FL_e100_21A14_R_0	BY943453
E_FL_e100_21A15_F_0	BY939221
E_FL_e100_21A19_R_0	BY943454
E_FL_e100_21B01_F_0	BY939222
E_FL_e100_21B04_F_0	BY939223
E_FL_e100_21B04_R_0	BY943455
E_FL_e100_21B05_F_0	BY939224
E_FL_e100_21B07_F_0	BY939225
E_FL_e100_21B08_F_0	BY939226
E_FL_e100_21B10_F_0	BY939227
E_FL_e100_21B13_F_0	BY939228
E_FL_e100_21B15_F_0	BY939229
E_FL_e100_21B19_F_0	BY939230
E_FL_e100_21B19_R_0	BY943456
E_FL_e100_21B20_F_0	BY939231
E_FL_e100_21B20_R_0	BY943457
E_FL_e100_21B22_F_0	BY939232
E_FL_e100_21B24_F_0	BY939233
E_FL_e100_21C07_F_0	BY939234
E_FL_e100_21C07_R_0	BY943458
E_FL_e100_21C08_R_0	BY943459

E_FL_e100_21C12_F_0	BY939235
E_FL_e100_21C12_R_0	BY943460
E_FL_e100_21C15_F_0	BY939236
E_FL_e100_21C15_R_0	BY943461
E_FL_e100_21C17_F_0	BY939237
E_FL_e100_21C17_R_0	BY943462
E_FL_e100_21C19_R_0	BY943463
E_FL_e100_21C20_F_0	BY939238
E_FL_e100_21C23_R_0	BY943464
E_FL_e100_21C24_F_0	BY939239
E_FL_e100_21D01_F_0	BY939240
E_FL_e100_21D03_F_0	BY939241
E_FL_e100_21D05_F_0	BY939242
E_FL_e100_21D05_R_0	BY943465
E_FL_e100_21D07_F_0	BY939243
E_FL_e100_21D07_R_0	BY943466
E_FL_e100_21D10_F_0	BY939244
E_FL_e100_21D13_F_0	BY939245
E_FL_e100_21D14_F_0	BY939246
E_FL_e100_21D14_R_0	BY943467
E_FL_e100_21D15_F_0	BY939247
E_FL_e100_21D15_R_0	BY943468
E_FL_e100_21D17_F_0	BY939248
E_FL_e100_21D17_R_0	BY943469
E_FL_e100_21D18_F_0	BY939249
E_FL_e100_21D18_R_0	BY943470
E_FL_e100_21D22_F_0	BY939250
E_FL_e100_21D24_F_0	BY939251
E_FL_e100_21D24_R_0	BY943471
E_FL_e100_21E01_R_0	BY943472
E_FL_e100_21E03_F_0	BY939252
E_FL_e100_21E04_F_0	BY939253
E_FL_e100_21E05_F_0	BY939254
E_FL_e100_21E06_F_0	BY939255
E_FL_e100_21E06_R_0	BY943473
E_FL_e100_21E08_F_0	BY939256
E_FL_e100_21E11_R_0	BY943474
E_FL_e100_21E12_F_0	BY939257
E_FL_e100_21E12_R_0	BY943475
E_FL_e100_21E13_F_0	BY939258
E_FL_e100_21E14_F_0	BY939259
E_FL_e100_21E15_R_0	BY943476
E_FL_e100_21E21_R_0	BY943477
E_FL_e100_21F02_F_0	BY939260
E_FL_e100_21F02_R_0	BY943478
E_FL_e100_21F04_F_0	BY939261
E_FL_e100_21F07_F_0	BY939262
E_FL_e100_21F07_R_0	BY943479
E_FL_e100_21F08_F_0	BY939263
E_FL_e100_21F09_F_0	BY939264
E_FL_e100_21F09_R_0	BY943480
E_FL_e100_21F10_F_0	BY939265
E_FL_e100_21F10_R_0	BY943481
E_FL_e100_21F11_F_0	BY939266

E_FL_e100_21F11_R_0	BY943482
E_FL_e100_21F12_F_0	BY939267
E_FL_e100_21F15_R_0	BY943483
E_FL_e100_21F16_F_0	BY939268
E_FL_e100_21F16_R_0	BY943484
E_FL_e100_21F17_R_0	BY943485
E_FL_e100_21F20_F_0	BY939269
E_FL_e100_21F20_R_0	BY943486
E_FL_e100_21F21_F_0	BY939270
E_FL_e100_21F22_F_0	BY939271
E_FL_e100_21F23_R_0	BY943487
E_FL_e100_21G04_F_0	BY939272
E_FL_e100_21G05_F_0	BY939273
E_FL_e100_21G05_R_0	BY943488
E_FL_e100_21G07_F_0	BY939274
E_FL_e100_21G07_R_0	BY943489
E_FL_e100_21G11_R_0	BY943490
E_FL_e100_21G13_F_0	BY939275
E_FL_e100_21G13_R_0	BY943491
E_FL_e100_21G16_F_0	BY939276
E_FL_e100_21G16_R_0	BY943492
E_FL_e100_21G17_R_0	BY943493
E_FL_e100_21G19_F_0	BY939277
E_FL_e100_21G20_F_0	BY939278
E_FL_e100_21G20_R_0	BY943494
E_FL_e100_21G21_R_0	BY943495
E_FL_e100_21G23_F_0	BY939279
E_FL_e100_21G24_F_0	BY939280
E_FL_e100_21H01_F_0	BY939281
E_FL_e100_21H02_R_0	BY943496
E_FL_e100_21H03_R_0	BY943497
E_FL_e100_21H04_F_0	BY939282
E_FL_e100_21H05_F_0	BY939283
E_FL_e100_21H05_R_0	BY943498
E_FL_e100_21H06_F_0	BY939284
E_FL_e100_21H06_R_0	BY943499
E_FL_e100_21H07_F_0	BY939285
E_FL_e100_21H07_R_0	BY943500
E_FL_e100_21H08_F_0	BY939286
E_FL_e100_21H08_R_0	BY943501
E_FL_e100_21H11_F_0	BY939287
E_FL_e100_21H11_R_0	BY943502
E_FL_e100_21H14_F_0	BY939288
E_FL_e100_21H16_F_0	BY939289
E_FL_e100_21H16_R_0	BY943503
E_FL_e100_21H17_F_0	BY939290
E_FL_e100_21H18_F_0	BY939291
E_FL_e100_21H19_F_0	BY939292
E_FL_e100_21H20_F_0	BY939293
E_FL_e100_21H22_F_0	BY939294
E_FL_e100_21H22_R_0	BY943504
E_FL_e100_21H23_F_0	BY939295
E_FL_e100_21H23_R_0	BY943505
E_FL_e100_21I01_F_0	BY939296

E_FL_e100_21I02_F_0	BY939297
E_FL_e100_21I03_F_0	BY939298
E_FL_e100_21I03_R_0	BY943506
E_FL_e100_21I04_F_0	BY939299
E_FL_e100_21I04_R_0	BY943507
E_FL_e100_21I06_R_0	BY943508
E_FL_e100_21I07_F_0	BY939300
E_FL_e100_21I10_R_0	BY943509
E_FL_e100_21I15_F_0	BY939301
E_FL_e100_21I15_R_0	BY943510
E_FL_e100_21I16_F_0	BY939302
E_FL_e100_21I16_R_0	BY943511
E_FL_e100_21I19_F_0	BY939303
E_FL_e100_21I19_R_0	BY943512
E_FL_e100_21I20_R_0	BY943513
E_FL_e100_21I23_F_0	BY939304
E_FL_e100_21I23_R_0	BY943514
E_FL_e100_21J01_F_0	BY939305
E_FL_e100_21J02_F_0	BY939306
E_FL_e100_21J02_R_0	BY943515
E_FL_e100_21J04_F_0	BY939307
E_FL_e100_21J04_R_0	BY943516
E_FL_e100_21J05_F_0	BY939308
E_FL_e100_21J05_R_0	BY943517
E_FL_e100_21J06_F_0	BY939309
E_FL_e100_21J09_F_0	BY939310
E_FL_e100_21J09_R_0	BY943518
E_FL_e100_21J10_R_0	BY943519
E_FL_e100_21J13_F_0	BY939311
E_FL_e100_21J19_F_0	BY939312
E_FL_e100_21J19_R_0	BY943520
E_FL_e100_21J20_F_0	BY939313
E_FL_e100_21J20_R_0	BY943521
E_FL_e100_21K01_F_0	BY939314
E_FL_e100_21K01_R_0	BY943522
E_FL_e100_21K02_F_0	BY939315
E_FL_e100_21K02_R_0	BY943523
E_FL_e100_21K03_F_0	BY939316
E_FL_e100_21K03_R_0	BY943524
E_FL_e100_21K05_F_0	BY939317
E_FL_e100_21K05_R_0	BY943525
E_FL_e100_21K14_R_0	BY943526
E_FL_e100_21K16_F_0	BY939318
E_FL_e100_21K16_R_0	BY943527
E_FL_e100_21L01_F_0	BY939319
E_FL_e100_21L01_R_0	BY943528
E_FL_e100_21L02_F_0	BY939320
E_FL_e100_21L02_R_0	BY943529
E_FL_e100_21L03_F_0	BY939321
E_FL_e100_21L07_F_0	BY939322
E_FL_e100_21L09_F_0	BY939323
E_FL_e100_21L10_F_0	BY939324
E_FL_e100_21L13_F_0	BY939325
E_FL_e100_21L14_F_0	BY939326

E_FL_e100_21L15_F_0	BY939327
E_FL_e100_21L15_R_0	BY943530
E_FL_e100_21L17_F_0	BY939328
E_FL_e100_21L17_R_0	BY943531
E_FL_e100_21L19_F_0	BY939329
E_FL_e100_21L19_R_0	BY943532
E_FL_e100_21L20_F_0	BY939330
E_FL_e100_21L20_R_0	BY943533
E_FL_e100_21L22_F_0	BY939331
E_FL_e100_21L22_R_0	BY943534
E_FL_e100_21M01_F_0	BY939332
E_FL_e100_21M05_F_0	BY939333
E_FL_e100_21M05_R_0	BY943535
E_FL_e100_21M06_F_0	BY939334
E_FL_e100_21M10_R_0	BY943536
E_FL_e100_21M11_F_0	BY939335
E_FL_e100_21M11_R_0	BY943537
E_FL_e100_21M12_F_0	BY939336
E_FL_e100_21M13_R_0	BY943538
E_FL_e100_21M14_F_0	BY939337
E_FL_e100_21M17_F_0	BY939338
E_FL_e100_21M17_R_0	BY943539
E_FL_e100_21M18_F_0	BY939339
E_FL_e100_21M20_R_0	BY943540
E_FL_e100_21M23_F_0	BY939340
E_FL_e100_21M23_R_0	BY943541
E_FL_e100_21N01_F_0	BY939341
E_FL_e100_21N02_F_0	BY939342
E_FL_e100_21N03_F_0	BY939343
E_FL_e100_21N03_R_0	BY943542
E_FL_e100_21N04_F_0	BY939344
E_FL_e100_21N04_R_0	BY943543
E_FL_e100_21N05_F_0	BY939345
E_FL_e100_21N06_R_0	BY943544
E_FL_e100_21N08_F_0	BY939346
E_FL_e100_21N08_R_0	BY943545
E_FL_e100_21N10_F_0	BY939347
E_FL_e100_21N13_F_0	BY939348
E_FL_e100_21N19_F_0	BY939349
E_FL_e100_21N19_R_0	BY943546
E_FL_e100_21N20_F_0	BY939350
E_FL_e100_21N20_R_0	BY943547
E_FL_e100_21N21_F_0	BY939351
E_FL_e100_21N22_F_0	BY939352
E_FL_e100_21O01_F_0	BY939353
E_FL_e100_21O03_R_0	BY943548
E_FL_e100_21O04_F_0	BY939354
E_FL_e100_21O08_R_0	BY943549
E_FL_e100_21O11_F_0	BY939355
E_FL_e100_21O13_R_0	BY943550
E_FL_e100_21O14_F_0	BY939356
E_FL_e100_21O16_F_0	BY939357
E_FL_e100_21O18_F_0	BY939358
E_FL_e100_21O21_R_0	BY943551

E_FL_e100_21O22_R_0	BY943552
E_FL_e100_21P02_F_0	BY939359
E_FL_e100_21P02_R_0	BY943553
E_FL_e100_21P03_R_0	BY943554
E_FL_e100_21P07_F_0	BY939360
E_FL_e100_21P08_F_0	BY939361
E_FL_e100_21P08_R_0	BY943555
E_FL_e100_21P10_F_0	BY939362
E_FL_e100_21P10_R_0	BY943556
E_FL_e100_21P12_R_0	BY943557
E_FL_e100_21P13_F_0	BY939363
E_FL_e100_21P14_F_0	BY939364
E_FL_e100_21P14_R_0	BY943558
E_FL_e100_21P19_F_0	BY939365
E_FL_e100_21P19_R_0	BY943559
E_FL_e100_21P20_F_0	BY939366
E_FL_e100_21P23_F_0	BY939367
E_FL_e100_22A02_F_0	BY939368
E_FL_e100_22A07_F_0	BY939369
E_FL_e100_22A10_F_0	BY939370
E_FL_e100_22A14_F_0	BY939371
E_FL_e100_22A15_F_0	BY939372
E_FL_e100_22A16_F_0	BY939373
E_FL_e100_22A17_F_0	BY939374
E_FL_e100_22A19_F_0	BY939375
E_FL_e100_22A21_F_0	BY939376
E_FL_e100_22A22_F_0	BY939377
E_FL_e100_22A24_F_0	BY939378
E_FL_e100_22B01_F_0	BY939379
E_FL_e100_22B01_R_0	BY943560
E_FL_e100_22B02_F_0	BY939380
E_FL_e100_22B02_R_0	BY943561
E_FL_e100_22B03_F_0	BY939381
E_FL_e100_22B03_R_0	BY943562
E_FL_e100_22B10_F_0	BY939382
E_FL_e100_22B10_R_0	BY943563
E_FL_e100_22B12_F_0	BY939383
E_FL_e100_22B12_R_0	BY943564
E_FL_e100_22B13_F_0	BY939384
E_FL_e100_22B14_F_0	BY939385
E_FL_e100_22B15_F_0	BY939386
E_FL_e100_22B15_R_0	BY943565
E_FL_e100_22B16_F_0	BY939387
E_FL_e100_22B16_R_0	BY943566
E_FL_e100_22B17_F_0	BY939388
E_FL_e100_22B17_R_0	BY943567
E_FL_e100_22B19_F_0	BY939389
E_FL_e100_22B20_F_0	BY939390
E_FL_e100_22B21_F_0	BY939391
E_FL_e100_22B21_R_0	BY943568
E_FL_e100_22B23_F_0	BY939392
E_FL_e100_22B23_R_0	BY943569
E_FL_e100_22B24_F_0	BY939393
E_FL_e100_22C02_F_0	BY939394

E_FL_e100_22C03_F_0	BY939395
E_FL_e100_22C04_F_0	BY939396
E_FL_e100_22C07_F_0	BY939397
E_FL_e100_22C07_R_0	BY943570
E_FL_e100_22C08_F_0	BY939398
E_FL_e100_22C11_F_0	BY939399
E_FL_e100_22C11_R_0	BY943571
E_FL_e100_22C12_F_0	BY939400
E_FL_e100_22C12_R_0	BY943572
E_FL_e100_22C16_F_0	BY939401
E_FL_e100_22C17_F_0	BY939402
E_FL_e100_22C18_R_0	BY943573
E_FL_e100_22C19_F_0	BY939403
E_FL_e100_22C21_F_0	BY939404
E_FL_e100_22C21_R_0	BY943574
E_FL_e100_22C22_F_0	BY939405
E_FL_e100_22C22_R_0	BY943575
E_FL_e100_22C23_F_0	BY939406
E_FL_e100_22C24_F_0	BY939407
E_FL_e100_22D02_F_0	BY939408
E_FL_e100_22D02_R_0	BY943576
E_FL_e100_22D05_F_0	BY939409
E_FL_e100_22D06_F_0	BY939410
E_FL_e100_22D06_R_0	BY943577
E_FL_e100_22D07_F_0	BY939411
E_FL_e100_22D08_R_0	BY943578
E_FL_e100_22D09_F_0	BY939412
E_FL_e100_22D09_R_0	BY943579
E_FL_e100_22D10_R_0	BY943580
E_FL_e100_22D11_F_0	BY939413
E_FL_e100_22D12_F_0	BY939414
E_FL_e100_22D14_F_0	BY939415
E_FL_e100_22D15_F_0	BY939416
E_FL_e100_22D15_R_0	BY943581
E_FL_e100_22D17_F_0	BY939417
E_FL_e100_22D17_R_0	BY943582
E_FL_e100_22D19_F_0	BY939418
E_FL_e100_22D21_F_0	BY939419
E_FL_e100_22D22_F_0	BY939420
E_FL_e100_22D23_F_0	BY939421
E_FL_e100_22D23_R_0	BY943583
E_FL_e100_22D24_F_0	BY939422
E_FL_e100_22D24_R_0	BY943584
E_FL_e100_22E01_F_0	BY939423
E_FL_e100_22E02_F_0	BY939424
E_FL_e100_22E04_F_0	BY939425
E_FL_e100_22E07_F_0	BY939426
E_FL_e100_22E07_R_0	BY943585
E_FL_e100_22E08_F_0	BY939427
E_FL_e100_22E09_F_0	BY939428
E_FL_e100_22E12_F_0	BY939429
E_FL_e100_22E12_R_0	BY943586
E_FL_e100_22E13_F_0	BY939430
E_FL_e100_22E14_F_0	BY939431

E_FL_e100_22E16_F_0	BY939432
E_FL_e100_22E18_F_0	BY939433
E_FL_e100_22E22_F_0	BY939434
E_FL_e100_22E24_F_0	BY939435
E_FL_e100_22E24_R_0	BY943587
E_FL_e100_22F03_F_0	BY939436
E_FL_e100_22F03_R_0	BY943588
E_FL_e100_22F04_F_0	BY939437
E_FL_e100_22F04_R_0	BY943589
E_FL_e100_22F06_F_0	BY939438
E_FL_e100_22F06_R_0	BY943590
E_FL_e100_22F09_F_0	BY939439
E_FL_e100_22F09_R_0	BY943591
E_FL_e100_22F10_F_0	BY939440
E_FL_e100_22F10_R_0	BY943592
E_FL_e100_22F12_F_0	BY939441
E_FL_e100_22F12_R_0	BY943593
E_FL_e100_22F13_F_0	BY939442
E_FL_e100_22F13_R_0	BY943594
E_FL_e100_22F14_F_0	BY939443
E_FL_e100_22F14_R_0	BY943595
E_FL_e100_22F18_F_0	BY939444
E_FL_e100_22F20_F_0	BY939445
E_FL_e100_22F20_R_0	BY943596
E_FL_e100_22F21_F_0	BY939446
E_FL_e100_22F21_R_0	BY943597
E_FL_e100_22F22_F_0	BY939447
E_FL_e100_22F22_R_0	BY943598
E_FL_e100_22F24_F_0	BY939448
E_FL_e100_22G01_F_0	BY939449
E_FL_e100_22G04_F_0	BY939450
E_FL_e100_22G04_R_0	BY943599
E_FL_e100_22G05_F_0	BY939451
E_FL_e100_22G05_R_0	BY943600
E_FL_e100_22G07_F_0	BY939452
E_FL_e100_22G09_F_0	BY939453
E_FL_e100_22G10_F_0	BY939454
E_FL_e100_22G10_R_0	BY943601
E_FL_e100_22G13_F_0	BY939455
E_FL_e100_22G15_F_0	BY939456
E_FL_e100_22G16_F_0	BY939457
E_FL_e100_22G18_F_0	BY939458
E_FL_e100_22G18_R_0	BY943602
E_FL_e100_22G19_F_0	BY939459
E_FL_e100_22G21_F_0	BY939460
E_FL_e100_22G21_R_0	BY943603
E_FL_e100_22G24_F_0	BY939461
E_FL_e100_22G24_R_0	BY943604
E_FL_e100_22H02_F_0	BY939462
E_FL_e100_22H02_R_0	BY943605
E_FL_e100_22H03_F_0	BY939463
E_FL_e100_22H03_R_0	BY943606
E_FL_e100_22H05_F_0	BY939464
E_FL_e100_22H06_F_0	BY939465

E_FL_e100_22H06_R_0	BY943607
E_FL_e100_22H08_F_0	BY939466
E_FL_e100_22H08_R_0	BY943608
E_FL_e100_22H09_F_0	BY939467
E_FL_e100_22H09_R_0	BY943609
E_FL_e100_22H10_F_0	BY939468
E_FL_e100_22H12_F_0	BY939469
E_FL_e100_22H12_R_0	BY943610
E_FL_e100_22H15_F_0	BY939470
E_FL_e100_22H18_F_0	BY939471
E_FL_e100_22H21_F_0	BY939472
E_FL_e100_22H22_F_0	BY939473
E_FL_e100_22H23_F_0	BY939474
E_FL_e100_22H24_F_0	BY939475
E_FL_e100_22H24_R_0	BY943611
E_FL_e100_22I01_F_0	BY939476
E_FL_e100_22I04_F_0	BY939477
E_FL_e100_22I04_R_0	BY943612
E_FL_e100_22I05_F_0	BY939478
E_FL_e100_22I05_R_0	BY943613
E_FL_e100_22I07_F_0	BY939479
E_FL_e100_22I07_R_0	BY943614
E_FL_e100_22I08_F_0	BY939480
E_FL_e100_22I09_F_0	BY939481
E_FL_e100_22I14_F_0	BY939482
E_FL_e100_22I15_F_0	BY939483
E_FL_e100_22I16_F_0	BY939484
E_FL_e100_22I17_F_0	BY939485
E_FL_e100_22I17_R_0	BY943615
E_FL_e100_22I18_F_0	BY939486
E_FL_e100_22I18_R_0	BY943616
E_FL_e100_22I19_F_0	BY939487
E_FL_e100_22I20_F_0	BY939488
E_FL_e100_22I21_F_0	BY939489
E_FL_e100_22I23_F_0	BY939490
E_FL_e100_22I24_F_0	BY939491
E_FL_e100_22J02_F_0	BY939492
E_FL_e100_22J03_F_0	BY939493
E_FL_e100_22J05_F_0	BY939494
E_FL_e100_22J11_F_0	BY939495
E_FL_e100_22J12_F_0	BY939496
E_FL_e100_22J15_F_0	BY939497
E_FL_e100_22J15_R_0	BY943617
E_FL_e100_22J17_F_0	BY939498
E_FL_e100_22J17_R_0	BY943618
E_FL_e100_22J18_F_0	BY939499
E_FL_e100_22J18_R_0	BY943619
E_FL_e100_22J19_F_0	BY939500
E_FL_e100_22J19_R_0	BY943620
E_FL_e100_22J20_F_0	BY939501
E_FL_e100_22J20_R_0	BY943621
E_FL_e100_22J21_F_0	BY939502
E_FL_e100_22J23_F_0	BY939503
E_FL_e100_22J24_F_0	BY939504

E_FL_e100_22K02_F_0	BY939505
E_FL_e100_22K05_F_0	BY939506
E_FL_e100_22K07_F_0	BY939507
E_FL_e100_22K07_R_0	BY943622
E_FL_e100_22K08_F_0	BY939508
E_FL_e100_22K08_R_0	BY943623
E_FL_e100_22K10_F_0	BY939509
E_FL_e100_22K10_R_0	BY943624
E_FL_e100_22K13_F_0	BY939510
E_FL_e100_22K15_F_0	BY939511
E_FL_e100_22K16_F_0	BY939512
E_FL_e100_22K19_F_0	BY939513
E_FL_e100_22K19_R_0	BY943625
E_FL_e100_22K21_F_0	BY939514
E_FL_e100_22K21_R_0	BY943626
E_FL_e100_22K23_F_0	BY939515
E_FL_e100_22K24_F_0	BY939516
E_FL_e100_22L01_R_0	BY943627
E_FL_e100_22L02_F_0	BY939517
E_FL_e100_22L03_F_0	BY939518
E_FL_e100_22L03_R_0	BY943628
E_FL_e100_22L04_F_0	BY939519
E_FL_e100_22L04_R_0	BY943629
E_FL_e100_22L05_F_0	BY939520
E_FL_e100_22L05_R_0	BY943630
E_FL_e100_22L08_F_0	BY939521
E_FL_e100_22L08_R_0	BY943631
E_FL_e100_22L09_F_0	BY939522
E_FL_e100_22L09_R_0	BY943632
E_FL_e100_22L11_F_0	BY939523
E_FL_e100_22L11_R_0	BY943633
E_FL_e100_22L15_F_0	BY939524
E_FL_e100_22L15_R_0	BY943634
E_FL_e100_22L16_F_0	BY939525
E_FL_e100_22L16_R_0	BY943635
E_FL_e100_22L17_F_0	BY939526
E_FL_e100_22L17_R_0	BY943636
E_FL_e100_22L20_F_0	BY939527
E_FL_e100_22L21_F_0	BY939528
E_FL_e100_22L21_R_0	BY943637
E_FL_e100_22L24_F_0	BY939529
E_FL_e100_22M01_F_0	BY939530
E_FL_e100_22M06_F_0	BY939531
E_FL_e100_22M06_R_0	BY943638
E_FL_e100_22M07_F_0	BY939532
E_FL_e100_22M10_F_0	BY939533
E_FL_e100_22M12_R_0	BY943639
E_FL_e100_22M13_F_0	BY939534
E_FL_e100_22M16_F_0	BY939535
E_FL_e100_22M17_F_0	BY939536
E_FL_e100_22M20_F_0	BY939537
E_FL_e100_22M21_F_0	BY939538
E_FL_e100_22M22_F_0	BY939539
E_FL_e100_22M22_R_0	BY943640

E_FL_e100_22M24_F_0	BY939540
E_FL_e100_22N01_F_0	BY939541
E_FL_e100_22N04_F_0	BY939542
E_FL_e100_22N05_F_0	BY939543
E_FL_e100_22N07_F_0	BY939544
E_FL_e100_22N07_R_0	BY943641
E_FL_e100_22N08_F_0	BY939545
E_FL_e100_22N09_F_0	BY939546
E_FL_e100_22N10_F_0	BY939547
E_FL_e100_22N11_F_0	BY939548
E_FL_e100_22N18_F_0	BY939549
E_FL_e100_22N20_F_0	BY939550
E_FL_e100_22N22_F_0	BY939551
E_FL_e100_22N23_R_0	BY943642
E_FL_e100_22N24_F_0	BY939552
E_FL_e100_22O04_F_0	BY939553
E_FL_e100_22O09_R_0	BY943643
E_FL_e100_22O11_F_0	BY939554
E_FL_e100_22O13_F_0	BY939555
E_FL_e100_22O14_F_0	BY939556
E_FL_e100_22O16_F_0	BY939557
E_FL_e100_22O17_R_0	BY943644
E_FL_e100_22O18_F_0	BY939558
E_FL_e100_22O18_R_0	BY943645
E_FL_e100_22O19_F_0	BY939559
E_FL_e100_22O19_R_0	BY943646
E_FL_e100_22O20_F_0	BY939560
E_FL_e100_22O22_F_0	BY939561
E_FL_e100_22O24_R_0	BY943647
E_FL_e100_22P01_F_0	BY939562
E_FL_e100_22P02_F_0	BY939563
E_FL_e100_22P02_R_0	BY943648
E_FL_e100_22P04_F_0	BY939564
E_FL_e100_22P04_R_0	BY943649
E_FL_e100_22P08_F_0	BY939565
E_FL_e100_22P10_F_0	BY939566
E_FL_e100_22P14_F_0	BY939567
E_FL_e100_22P16_F_0	BY939568
E_FL_e100_22P17_F_0	BY939569
E_FL_e100_22P22_F_0	BY939570
E_FL_e100_22P22_R_0	BY943650
E_FL_e100_22P23_F_0	BY939571
E_FL_e100_22P23_R_0	BY943651
E_FL_e100_23A02_F_0	BY939572
E_FL_e100_23A02_R_0	BY943652
E_FL_e100_23A03_F_0	BY939573
E_FL_e100_23A04_F_0	BY939574
E_FL_e100_23A05_R_0	BY943653
E_FL_e100_23A07_F_0	BY939575
E_FL_e100_23A07_R_0	BY943654
E_FL_e100_23A11_R_0	BY943655
E_FL_e100_23A12_R_0	BY943656
E_FL_e100_23A16_R_0	BY943657
E_FL_e100_23A19_F_0	BY939576

E_FL_e100_23A20_F_0	BY939577
E_FL_e100_23A24_F_0	BY939578
E_FL_e100_23B01_F_0	BY939579
E_FL_e100_23B01_R_0	BY943658
E_FL_e100_23B02_R_0	BY943659
E_FL_e100_23B03_R_0	BY943660
E_FL_e100_23B04_F_0	BY939580
E_FL_e100_23B04_R_0	BY943661
E_FL_e100_23B06_R_0	BY943662
E_FL_e100_23B07_F_0	BY939581
E_FL_e100_23B07_R_0	BY943663
E_FL_e100_23B08_R_0	BY943664
E_FL_e100_23B09_F_0	BY939582
E_FL_e100_23B09_R_0	BY943665
E_FL_e100_23B10_F_0	BY939583
E_FL_e100_23B10_R_0	BY943666
E_FL_e100_23B12_F_0	BY939584
E_FL_e100_23B12_R_0	BY943667
E_FL_e100_23B13_F_0	BY939585
E_FL_e100_23B13_R_0	BY943668
E_FL_e100_23B14_F_0	BY939586
E_FL_e100_23B14_R_0	BY943669
E_FL_e100_23B18_F_0	BY939587
E_FL_e100_23B18_R_0	BY943670
E_FL_e100_23B19_F_0	BY939588
E_FL_e100_23B19_R_0	BY943671
E_FL_e100_23B20_F_0	BY939589
E_FL_e100_23B20_R_0	BY943672
E_FL_e100_23B22_F_0	BY939590
E_FL_e100_23B24_F_0	BY939591
E_FL_e100_23B24_R_0	BY943673
E_FL_e100_23C01_F_0	BY939592
E_FL_e100_23C03_R_0	BY943674
E_FL_e100_23C05_F_0	BY939593
E_FL_e100_23C08_F_0	BY939594
E_FL_e100_23C10_R_0	BY943675
E_FL_e100_23C11_R_0	BY943676
E_FL_e100_23C12_F_0	BY939595
E_FL_e100_23C13_F_0	BY939596
E_FL_e100_23C17_R_0	BY943677
E_FL_e100_23C18_F_0	BY939597
E_FL_e100_23C19_F_0	BY939598
E_FL_e100_23C19_R_0	BY943678
E_FL_e100_23C20_F_0	BY939599
E_FL_e100_23C22_R_0	BY943679
E_FL_e100_23D01_F_0	BY939600
E_FL_e100_23D01_R_0	BY943680
E_FL_e100_23D02_R_0	BY943681
E_FL_e100_23D03_F_0	BY939601
E_FL_e100_23D03_R_0	BY943682
E_FL_e100_23D04_F_0	BY939602
E_FL_e100_23D04_R_0	BY943683
E_FL_e100_23D07_F_0	BY939603
E_FL_e100_23D07_R_0	BY943684

E_FL_e100_23D08_F_0	BY939604
E_FL_e100_23D09_F_0	BY939605
E_FL_e100_23D10_F_0	BY939606
E_FL_e100_23D10_R_0	BY943685
E_FL_e100_23D12_F_0	BY939607
E_FL_e100_23D12_R_0	BY943686
E_FL_e100_23D13_F_0	BY939608
E_FL_e100_23D13_R_0	BY943687
E_FL_e100_23D14_R_0	BY943688
E_FL_e100_23D15_F_0	BY939609
E_FL_e100_23D15_R_0	BY943689
E_FL_e100_23D16_R_0	BY943690
E_FL_e100_23D19_F_0	BY939610
E_FL_e100_23D19_R_0	BY943691
E_FL_e100_23D21_R_0	BY943692
E_FL_e100_23D22_F_0	BY939611
E_FL_e100_23D23_F_0	BY939612
E_FL_e100_23D23_R_0	BY943693
E_FL_e100_23D24_R_0	BY943694
E_FL_e100_23E03_F_0	BY939613
E_FL_e100_23E03_R_0	BY943695
E_FL_e100_23E05_F_0	BY939614
E_FL_e100_23E08_F_0	BY939615
E_FL_e100_23E09_F_0	BY939616
E_FL_e100_23E12_R_0	BY943696
E_FL_e100_23E13_F_0	BY939617
E_FL_e100_23E13_R_0	BY943697
E_FL_e100_23E18_F_0	BY939618
E_FL_e100_23E18_R_0	BY943698
E_FL_e100_23E21_F_0	BY939619
E_FL_e100_23E24_R_0	BY943699
E_FL_e100_23F04_F_0	BY939620
E_FL_e100_23F06_F_0	BY939621
E_FL_e100_23F06_R_0	BY943700
E_FL_e100_23F08_F_0	BY939622
E_FL_e100_23F09_F_0	BY939623
E_FL_e100_23F09_R_0	BY943701
E_FL_e100_23F10_F_0	BY939624
E_FL_e100_23F11_R_0	BY943702
E_FL_e100_23F13_F_0	BY939625
E_FL_e100_23F13_R_0	BY943703
E_FL_e100_23F14_F_0	BY939626
E_FL_e100_23F14_R_0	BY943704
E_FL_e100_23F19_F_0	BY939627
E_FL_e100_23F21_F_0	BY939628
E_FL_e100_23F21_R_0	BY943705
E_FL_e100_23F24_F_0	BY939629
E_FL_e100_23G06_F_0	BY939630
E_FL_e100_23G06_R_0	BY943706
E_FL_e100_23G08_F_0	BY939631
E_FL_e100_23G11_R_0	BY943707
E_FL_e100_23G12_F_0	BY939632
E_FL_e100_23G12_R_0	BY943708
E_FL_e100_23G16_R_0	BY943709

E_FL_e100_23G18_F_0	BY939633
E_FL_e100_23G19_R_0	BY943710
E_FL_e100_23G24_F_0	BY939634
E_FL_e100_23G24_R_0	BY943711
E_FL_e100_23H01_F_0	BY939635
E_FL_e100_23H02_F_0	BY939636
E_FL_e100_23H03_F_0	BY939637
E_FL_e100_23H04_F_0	BY939638
E_FL_e100_23H04_R_0	BY943712
E_FL_e100_23H07_F_0	BY939639
E_FL_e100_23H07_R_0	BY943713
E_FL_e100_23H08_F_0	BY939640
E_FL_e100_23H09_F_0	BY939641
E_FL_e100_23H16_F_0	BY939642
E_FL_e100_23H19_F_0	BY939643
E_FL_e100_23H22_F_0	BY939644
E_FL_e100_23H22_R_0	BY943714
E_FL_e100_23I01_F_0	BY939645
E_FL_e100_23I05_F_0	BY939646
E_FL_e100_23I05_R_0	BY943715
E_FL_e100_23I06_F_0	BY939647
E_FL_e100_23I06_R_0	BY943716
E_FL_e100_23I07_F_0	BY939648
E_FL_e100_23I07_R_0	BY943717
E_FL_e100_23I08_F_0	BY939649
E_FL_e100_23I10_R_0	BY943718
E_FL_e100_23I11_R_0	BY943719
E_FL_e100_23I12_R_0	BY943720
E_FL_e100_23I13_R_0	BY943721
E_FL_e100_23I14_F_0	BY939650
E_FL_e100_23I15_R_0	BY943722
E_FL_e100_23I19_F_0	BY939651
E_FL_e100_23I19_R_0	BY943723
E_FL_e100_23I23_F_0	BY939652
E_FL_e100_23I23_R_0	BY943724
E_FL_e100_23J03_F_0	BY939653
E_FL_e100_23J03_R_0	BY943725
E_FL_e100_23J04_F_0	BY939654
E_FL_e100_23J04_R_0	BY943726
E_FL_e100_23J05_F_0	BY939655
E_FL_e100_23J05_R_0	BY943727
E_FL_e100_23J06_R_0	BY943728
E_FL_e100_23J07_F_0	BY939656
E_FL_e100_23J07_R_0	BY943729
E_FL_e100_23J10_F_0	BY939657
E_FL_e100_23J10_R_0	BY943730
E_FL_e100_23J12_F_0	BY939658
E_FL_e100_23J12_R_0	BY943731
E_FL_e100_23J13_F_0	BY939659
E_FL_e100_23J13_R_0	BY943732
E_FL_e100_23J14_F_0	BY939660
E_FL_e100_23J16_F_0	BY939661
E_FL_e100_23J18_F_0	BY939662
E_FL_e100_23J20_F_0	BY939663

E_FL_e100_23J20_R_0	BY943733
E_FL_e100_23J21_F_0	BY939664
E_FL_e100_23J22_F_0	BY939665
E_FL_e100_23J22_R_0	BY943734
E_FL_e100_23J23_F_0	BY939666
E_FL_e100_23J23_R_0	BY943735
E_FL_e100_23J24_R_0	BY943736
E_FL_e100_23K01_F_0	BY939667
E_FL_e100_23K02_F_0	BY939668
E_FL_e100_23K02_R_0	BY943737
E_FL_e100_23K03_F_0	BY939669
E_FL_e100_23K04_F_0	BY939670
E_FL_e100_23K05_F_0	BY939671
E_FL_e100_23K10_R_0	BY943738
E_FL_e100_23K17_F_0	BY939672
E_FL_e100_23K23_F_0	BY939673
E_FL_e100_23L04_F_0	BY939674
E_FL_e100_23L07_F_0	BY939675
E_FL_e100_23L07_R_0	BY943739
E_FL_e100_23L09_F_0	BY939676
E_FL_e100_23L09_R_0	BY943740
E_FL_e100_23L11_F_0	BY939677
E_FL_e100_23L11_R_0	BY943741
E_FL_e100_23L14_F_0	BY939678
E_FL_e100_23L14_R_0	BY943742
E_FL_e100_23L17_F_0	BY939679
E_FL_e100_23L17_R_0	BY943743
E_FL_e100_23L19_F_0	BY939680
E_FL_e100_23L19_R_0	BY943744
E_FL_e100_23L20_F_0	BY939681
E_FL_e100_23L20_R_0	BY943745
E_FL_e100_23L21_F_0	BY939682
E_FL_e100_23L21_R_0	BY943746
E_FL_e100_23L22_R_0	BY943747
E_FL_e100_23L23_F_0	BY939683
E_FL_e100_23L23_R_0	BY943748
E_FL_e100_23M01_F_0	BY939684
E_FL_e100_23M02_R_0	BY943749
E_FL_e100_23M03_F_0	BY939685
E_FL_e100_23M05_F_0	BY939686
E_FL_e100_23M05_R_0	BY943750
E_FL_e100_23M06_R_0	BY943751
E_FL_e100_23M07_F_0	BY939687
E_FL_e100_23M09_R_0	BY943752
E_FL_e100_23M11_R_0	BY943753
E_FL_e100_23M13_F_0	BY939688
E_FL_e100_23M13_R_0	BY943754
E_FL_e100_23M20_F_0	BY939689
E_FL_e100_23M20_R_0	BY943755
E_FL_e100_23M21_F_0	BY939690
E_FL_e100_23N01_R_0	BY943756
E_FL_e100_23N02_F_0	BY939691
E_FL_e100_23N02_R_0	BY943757
E_FL_e100_23N03_F_0	BY939692

E_FL_e100_23N03_R_0	BY943758
E_FL_e100_23N04_F_0	BY939693
E_FL_e100_23N04_R_0	BY943759
E_FL_e100_23N05_F_0	BY939694
E_FL_e100_23N07_F_0	BY939695
E_FL_e100_23N08_F_0	BY939696
E_FL_e100_23N11_F_0	BY939697
E_FL_e100_23N14_F_0	BY939698
E_FL_e100_23N16_F_0	BY939699
E_FL_e100_23N16_R_0	BY943760
E_FL_e100_23N19_F_0	BY939700
E_FL_e100_23N19_R_0	BY943761
E_FL_e100_23N21_F_0	BY939701
E_FL_e100_23N21_R_0	BY943762
E_FL_e100_23N22_F_0	BY939702
E_FL_e100_23O01_F_0	BY939703
E_FL_e100_23O02_F_0	BY939704
E_FL_e100_23O03_R_0	BY943763
E_FL_e100_23O04_F_0	BY939705
E_FL_e100_23O06_F_0	BY939706
E_FL_e100_23O06_R_0	BY943764
E_FL_e100_23O12_F_0	BY939707
E_FL_e100_23O12_R_0	BY943765
E_FL_e100_23O15_R_0	BY943766
E_FL_e100_23O17_F_0	BY939708
E_FL_e100_23O20_R_0	BY943767
E_FL_e100_23O24_F_0	BY939709
E_FL_e100_23O24_R_0	BY943768
E_FL_e100_23P01_F_0	BY939710
E_FL_e100_23P01_R_0	BY943769
E_FL_e100_23P04_F_0	BY939711
E_FL_e100_23P04_R_0	BY943770
E_FL_e100_23P05_F_0	BY939712
E_FL_e100_23P05_R_0	BY943771
E_FL_e100_23P10_R_0	BY943772
E_FL_e100_23P13_F_0	BY939713
E_FL_e100_23P13_R_0	BY943773
E_FL_e100_23P14_F_0	BY939714
E_FL_e100_23P17_F_0	BY939715
E_FL_e100_23P18_F_0	BY939716
E_FL_e100_23P18_R_0	BY943774
E_FL_e100_23P19_R_0	BY943775
E_FL_e100_23P21_F_0	BY939717
E_FL_e100_24A03_F_0	BY939718
E_FL_e100_24A05_F_0	BY939719
E_FL_e100_24A06_F_0	BY939720
E_FL_e100_24A08_R_0	BY943776
E_FL_e100_24A09_F_0	BY939721
E_FL_e100_24A10_F_0	BY939722
E_FL_e100_24A13_F_0	BY939723
E_FL_e100_24A13_R_0	BY943777
E_FL_e100_24A15_F_0	BY939724
E_FL_e100_24A16_F_0	BY939725
E_FL_e100_24A16_R_0	BY943778

E_FL_e100_24A18_F_0	BY939726
E_FL_e100_24A18_R_0	BY943779
E_FL_e100_24A20_F_0	BY939727
E_FL_e100_24A24_F_0	BY939728
E_FL_e100_24A24_R_0	BY943780
E_FL_e100_24B01_F_0	BY939729
E_FL_e100_24B03_F_0	BY939730
E_FL_e100_24B03_R_0	BY943781
E_FL_e100_24B06_F_0	BY939731
E_FL_e100_24B06_R_0	BY943782
E_FL_e100_24B08_F_0	BY939732
E_FL_e100_24B11_F_0	BY939733
E_FL_e100_24B11_R_0	BY943783
E_FL_e100_24B14_F_0	BY939734
E_FL_e100_24B14_R_0	BY943784
E_FL_e100_24B15_F_0	BY939735
E_FL_e100_24B16_F_0	BY939736
E_FL_e100_24B16_R_0	BY943785
E_FL_e100_24B17_F_0	BY939737
E_FL_e100_24B18_F_0	BY939738
E_FL_e100_24B19_R_0	BY943786
E_FL_e100_24B22_F_0	BY939739
E_FL_e100_24B22_R_0	BY943787
E_FL_e100_24B23_F_0	BY939740
E_FL_e100_24B24_F_0	BY939741
E_FL_e100_24C02_F_0	BY939742
E_FL_e100_24C03_F_0	BY939743
E_FL_e100_24C05_F_0	BY939744
E_FL_e100_24C07_R_0	BY943788
E_FL_e100_24C08_F_0	BY939745
E_FL_e100_24C13_F_0	BY939746
E_FL_e100_24C13_R_0	BY943789
E_FL_e100_24C15_R_0	BY943790
E_FL_e100_24C19_F_0	BY939747
E_FL_e100_24C22_F_0	BY939748
E_FL_e100_24C24_R_0	BY943791
E_FL_e100_24D01_F_0	BY939749
E_FL_e100_24D01_R_0	BY943792
E_FL_e100_24D02_F_0	BY939750
E_FL_e100_24D03_F_0	BY939751
E_FL_e100_24D03_R_0	BY943793
E_FL_e100_24D04_R_0	BY943794
E_FL_e100_24D06_F_0	BY939752
E_FL_e100_24D06_R_0	BY943795
E_FL_e100_24D07_F_0	BY939753
E_FL_e100_24D07_R_0	BY943796
E_FL_e100_24D08_F_0	BY939754
E_FL_e100_24D10_F_0	BY939755
E_FL_e100_24D11_F_0	BY939756
E_FL_e100_24D13_F_0	BY939757
E_FL_e100_24D13_R_0	BY943797
E_FL_e100_24D15_F_0	BY939758
E_FL_e100_24D15_R_0	BY943798
E_FL_e100_24D18_F_0	BY939759

E_FL_e100_24D19_F_0	BY939760
E_FL_e100_24D19_R_0	BY943799
E_FL_e100_24D23_R_0	BY943800
E_FL_e100_24D24_F_0	BY939761
E_FL_e100_24D24_R_0	BY943801
E_FL_e100_24E06_F_0	BY939762
E_FL_e100_24E06_R_0	BY943802
E_FL_e100_24E07_F_0	BY939763
E_FL_e100_24E07_R_0	BY943803
E_FL_e100_24E09_F_0	BY939764
E_FL_e100_24E11_F_0	BY939765
E_FL_e100_24E12_F_0	BY939766
E_FL_e100_24E12_R_0	BY943804
E_FL_e100_24E15_F_0	BY939767
E_FL_e100_24E15_R_0	BY943805
E_FL_e100_24E17_F_0	BY939768
E_FL_e100_24E19_F_0	BY939769
E_FL_e100_24E20_F_0	BY939770
E_FL_e100_24E21_F_0	BY939771
E_FL_e100_24E22_F_0	BY939772
E_FL_e100_24E23_F_0	BY939773
E_FL_e100_24E23_R_0	BY943806
E_FL_e100_24E24_F_0	BY939774
E_FL_e100_24F01_F_0	BY939775
E_FL_e100_24F03_F_0	BY939776
E_FL_e100_24F06_R_0	BY943807
E_FL_e100_24F07_F_0	BY939777
E_FL_e100_24F08_F_0	BY939778
E_FL_e100_24F08_R_0	BY943808
E_FL_e100_24F09_R_0	BY943809
E_FL_e100_24F11_F_0	BY939779
E_FL_e100_24F11_R_0	BY943810
E_FL_e100_24F13_F_0	BY939780
E_FL_e100_24F13_R_0	BY943811
E_FL_e100_24F14_F_0	BY939781
E_FL_e100_24F14_R_0	BY943812
E_FL_e100_24F15_F_0	BY939782
E_FL_e100_24F15_R_0	BY943813
E_FL_e100_24F20_F_0	BY939783
E_FL_e100_24F21_F_0	BY939784
E_FL_e100_24F21_R_0	BY943814
E_FL_e100_24F22_F_0	BY939785
E_FL_e100_24F22_R_0	BY943815
E_FL_e100_24F23_F_0	BY939786
E_FL_e100_24F23_R_0	BY943816
E_FL_e100_24G05_F_0	BY939787
E_FL_e100_24G05_R_0	BY943817
E_FL_e100_24G06_F_0	BY939788
E_FL_e100_24G06_R_0	BY943818
E_FL_e100_24G07_R_0	BY943819
E_FL_e100_24G08_F_0	BY939789
E_FL_e100_24G11_F_0	BY939790
E_FL_e100_24G12_F_0	BY939791
E_FL_e100_24G13_F_0	BY939792

E_FL_e100_24G14_F_0	BY939793
E_FL_e100_24G14_R_0	BY943820
E_FL_e100_24G16_F_0	BY939794
E_FL_e100_24G17_F_0	BY939795
E_FL_e100_24G19_F_0	BY939796
E_FL_e100_24G20_F_0	BY939797
E_FL_e100_24G22_F_0	BY939798
E_FL_e100_24G22_R_0	BY943821
E_FL_e100_24G23_F_0	BY939799
E_FL_e100_24H03_F_0	BY939800
E_FL_e100_24H03_R_0	BY943822
E_FL_e100_24H04_F_0	BY939801
E_FL_e100_24H05_F_0	BY939802
E_FL_e100_24H07_F_0	BY939803
E_FL_e100_24H09_F_0	BY939804
E_FL_e100_24H10_F_0	BY939805
E_FL_e100_24H10_R_0	BY943823
E_FL_e100_24H11_F_0	BY939806
E_FL_e100_24H12_F_0	BY939807
E_FL_e100_24H14_F_0	BY939808
E_FL_e100_24H15_F_0	BY939809
E_FL_e100_24H21_F_0	BY939810
E_FL_e100_24H21_R_0	BY943824
E_FL_e100_24H22_F_0	BY939811
E_FL_e100_24H23_F_0	BY939812
E_FL_e100_24H24_R_0	BY943825
E_FL_e100_24I03_F_0	BY939813
E_FL_e100_24I04_F_0	BY939814
E_FL_e100_24I05_F_0	BY939815
E_FL_e100_24I05_R_0	BY943826
E_FL_e100_24I13_F_0	BY939816
E_FL_e100_24I14_F_0	BY939817
E_FL_e100_24I14_R_0	BY943827
E_FL_e100_24I15_F_0	BY939818
E_FL_e100_24I16_F_0	BY939819
E_FL_e100_24I18_F_0	BY939820
E_FL_e100_24I21_F_0	BY939821
E_FL_e100_24I22_F_0	BY939822
E_FL_e100_24I23_F_0	BY939823
E_FL_e100_24J03_F_0	BY939824
E_FL_e100_24J04_F_0	BY939825
E_FL_e100_24J04_R_0	BY943828
E_FL_e100_24J05_F_0	BY939826
E_FL_e100_24J05_R_0	BY943829
E_FL_e100_24J06_R_0	BY943830
E_FL_e100_24J07_F_0	BY939827
E_FL_e100_24J09_F_0	BY939828
E_FL_e100_24J11_F_0	BY939829
E_FL_e100_24J12_F_0	BY939830
E_FL_e100_24J12_R_0	BY943831
E_FL_e100_24J13_F_0	BY939831
E_FL_e100_24J13_R_0	BY943832
E_FL_e100_24J14_F_0	BY939832
E_FL_e100_24J14_R_0	BY943833

E_FL_e100_24J16_F_0	BY939833
E_FL_e100_24J18_F_0	BY939834
E_FL_e100_24J19_F_0	BY939835
E_FL_e100_24J19_R_0	BY943834
E_FL_e100_24J21_R_0	BY943835
E_FL_e100_24J23_F_0	BY939836
E_FL_e100_24J24_F_0	BY939837
E_FL_e100_24J24_R_0	BY943836
E_FL_e100_24K01_F_0	BY939838
E_FL_e100_24K02_F_0	BY939839
E_FL_e100_24K03_F_0	BY939840
E_FL_e100_24K04_F_0	BY939841
E_FL_e100_24K05_F_0	BY939842
E_FL_e100_24K05_R_0	BY943837
E_FL_e100_24K06_F_0	BY939843
E_FL_e100_24K08_F_0	BY939844
E_FL_e100_24K11_F_0	BY939845
E_FL_e100_24K14_F_0	BY939846
E_FL_e100_24K15_F_0	BY939847
E_FL_e100_24K23_F_0	BY939848
E_FL_e100_24K23_R_0	BY943838
E_FL_e100_24K24_F_0	BY939849
E_FL_e100_24L01_F_0	BY939850
E_FL_e100_24L03_F_0	BY939851
E_FL_e100_24L03_R_0	BY943839
E_FL_e100_24L07_F_0	BY939852
E_FL_e100_24L07_R_0	BY943840
E_FL_e100_24L10_F_0	BY939853
E_FL_e100_24L11_F_0	BY939854
E_FL_e100_24L14_F_0	BY939855
E_FL_e100_24L14_R_0	BY943841
E_FL_e100_24L15_F_0	BY939856
E_FL_e100_24L15_R_0	BY943842
E_FL_e100_24L16_F_0	BY939857
E_FL_e100_24L16_R_0	BY943843
E_FL_e100_24L18_F_0	BY939858
E_FL_e100_24L22_F_0	BY939859
E_FL_e100_24L22_R_0	BY943844
E_FL_e100_24L23_F_0	BY939860
E_FL_e100_24M01_F_0	BY939861
E_FL_e100_24M04_F_0	BY939862
E_FL_e100_24M05_F_0	BY939863
E_FL_e100_24M05_R_0	BY943845
E_FL_e100_24M06_F_0	BY939864
E_FL_e100_24M06_R_0	BY943846
E_FL_e100_24M07_F_0	BY939865
E_FL_e100_24M08_F_0	BY939866
E_FL_e100_24M15_F_0	BY939867
E_FL_e100_24M15_R_0	BY943847
E_FL_e100_24M17_F_0	BY939868
E_FL_e100_24M22_F_0	BY939869
E_FL_e100_24M23_F_0	BY939870
E_FL_e100_24M23_R_0	BY943848
E_FL_e100_24N01_R_0	BY943849

E_FL_e100_24N02_F_0	BY939871
E_FL_e100_24N02_R_0	BY943850
E_FL_e100_24N04_F_0	BY939872
E_FL_e100_24N04_R_0	BY943851
E_FL_e100_24N08_F_0	BY939873
E_FL_e100_24N08_R_0	BY943852
E_FL_e100_24N10_F_0	BY939874
E_FL_e100_24N10_R_0	BY943853
E_FL_e100_24N11_F_0	BY939875
E_FL_e100_24N11_R_0	BY943854
E_FL_e100_24N15_F_0	BY939876
E_FL_e100_24N22_F_0	BY939877
E_FL_e100_24N22_R_0	BY943855
E_FL_e100_24O02_F_0	BY939878
E_FL_e100_24O03_F_0	BY939879
E_FL_e100_24O04_F_0	BY939880
E_FL_e100_24O05_F_0	BY939881
E_FL_e100_24O05_R_0	BY943856
E_FL_e100_24O06_F_0	BY939882
E_FL_e100_24O06_R_0	BY943857
E_FL_e100_24O11_F_0	BY939883
E_FL_e100_24O11_R_0	BY943858
E_FL_e100_24O13_F_0	BY939884
E_FL_e100_24O13_R_0	BY943859
E_FL_e100_24O14_F_0	BY939885
E_FL_e100_24O18_F_0	BY939886
E_FL_e100_24O19_F_0	BY939887
E_FL_e100_24O20_F_0	BY939888
E_FL_e100_24O21_F_0	BY939889
E_FL_e100_24P01_F_0	BY939890
E_FL_e100_24P01_R_0	BY943860
E_FL_e100_24P02_F_0	BY939891
E_FL_e100_24P02_R_0	BY943861
E_FL_e100_24P03_F_0	BY939892
E_FL_e100_24P03_R_0	BY943862
E_FL_e100_24P04_F_0	BY939893
E_FL_e100_24P04_R_0	BY943863
E_FL_e100_24P05_F_0	BY939894
E_FL_e100_24P05_R_0	BY943864
E_FL_e100_24P06_F_0	BY939895
E_FL_e100_24P08_R_0	BY943865
E_FL_e100_24P10_F_0	BY939896
E_FL_e100_24P11_R_0	BY943866
E_FL_e100_24P13_F_0	BY939897
E_FL_e100_24P13_R_0	BY943867
E_FL_e100_24P16_R_0	BY943868
E_FL_e100_24P17_F_0	BY939898
E_FL_e100_24P20_F_0	BY939899
E_FL_e100_24P20_R_0	BY943869
E_FL_e100_24P23_F_0	BY939900
E_FL_e100_24P24_F_0	BY939901
E_FL_e100_24P24_R_0	BY943870
E_FL_e100_25A02_F_0	BY939902
E_FL_e100_25A03_F_0	BY939903

E_FL_e100_25A06_F_0	BY939904
E_FL_e100_25A06_R_0	BY943871
E_FL_e100_25A07_F_0	BY939905
E_FL_e100_25A07_R_0	BY943872
E_FL_e100_25A08_F_0	BY939906
E_FL_e100_25A09_F_0	BY939907
E_FL_e100_25A12_F_0	BY939908
E_FL_e100_25A12_R_0	BY943873
E_FL_e100_25A13_F_0	BY939909
E_FL_e100_25A14_F_0	BY939910
E_FL_e100_25A17_F_0	BY939911
E_FL_e100_25A18_F_0	BY939912
E_FL_e100_25A20_F_0	BY939913
E_FL_e100_25A22_F_0	BY939914
E_FL_e100_25A23_F_0	BY939915
E_FL_e100_25A23_R_0	BY943874
E_FL_e100_25A24_F_0	BY939916
E_FL_e100_25A24_R_0	BY943875
E_FL_e100_25B02_F_0	BY939917
E_FL_e100_25B04_F_0	BY939918
E_FL_e100_25B04_R_0	BY943876
E_FL_e100_25B07_F_0	BY939919
E_FL_e100_25B07_R_0	BY943877
E_FL_e100_25B08_F_0	BY939920
E_FL_e100_25B08_R_0	BY943878
E_FL_e100_25B09_F_0	BY939921
E_FL_e100_25B10_F_0	BY939922
E_FL_e100_25B10_R_0	BY943879
E_FL_e100_25B12_F_0	BY939923
E_FL_e100_25B12_R_0	BY943880
E_FL_e100_25B14_F_0	BY939924
E_FL_e100_25B15_F_0	BY939925
E_FL_e100_25B15_R_0	BY943881
E_FL_e100_25B23_F_0	BY939926
E_FL_e100_25B23_R_0	BY943882
E_FL_e100_25C04_F_0	BY939927
E_FL_e100_25C04_R_0	BY943883
E_FL_e100_25C05_F_0	BY939928
E_FL_e100_25C07_F_0	BY939929
E_FL_e100_25C09_F_0	BY939930
E_FL_e100_25C12_F_0	BY939931
E_FL_e100_25C13_F_0	BY939932
E_FL_e100_25C16_F_0	BY939933
E_FL_e100_25C17_F_0	BY939934
E_FL_e100_25C18_F_0	BY939935
E_FL_e100_25C19_F_0	BY939936
E_FL_e100_25C20_F_0	BY939937
E_FL_e100_25C24_F_0	BY939938
E_FL_e100_25D01_F_0	BY939939
E_FL_e100_25D05_F_0	BY939940
E_FL_e100_25D06_F_0	BY939941
E_FL_e100_25D06_R_0	BY943884
E_FL_e100_25D09_F_0	BY939942
E_FL_e100_25D10_F_0	BY939943

E_FL_e100_25D14_R_0	BY943885
E_FL_e100_25D15_F_0	BY939944
E_FL_e100_25D15_R_0	BY943886
E_FL_e100_25D16_F_0	BY939945
E_FL_e100_25D16_R_0	BY943887
E_FL_e100_25D17_F_0	BY939946
E_FL_e100_25D17_R_0	BY943888
E_FL_e100_25D18_F_0	BY939947
E_FL_e100_25D18_R_0	BY943889
E_FL_e100_25D20_F_0	BY939948
E_FL_e100_25D20_R_0	BY943890
E_FL_e100_25D21_F_0	BY939949
E_FL_e100_25D22_F_0	BY939950
E_FL_e100_25D22_R_0	BY943891
E_FL_e100_25E01_F_0	BY939951
E_FL_e100_25E02_F_0	BY939952
E_FL_e100_25E02_R_0	BY943892
E_FL_e100_25E04_F_0	BY939953
E_FL_e100_25E05_F_0	BY939954
E_FL_e100_25E05_R_0	BY943893
E_FL_e100_25E07_F_0	BY939955
E_FL_e100_25E08_F_0	BY939956
E_FL_e100_25E12_F_0	BY939957
E_FL_e100_25E12_R_0	BY943894
E_FL_e100_25E14_F_0	BY939958
E_FL_e100_25E16_F_0	BY939959
E_FL_e100_25E17_F_0	BY939960
E_FL_e100_25E21_F_0	BY939961
E_FL_e100_25E24_F_0	BY939962
E_FL_e100_25E24_R_0	BY943895
E_FL_e100_25F01_F_0	BY939963
E_FL_e100_25F02_F_0	BY939964
E_FL_e100_25F03_R_0	BY943896
E_FL_e100_25F05_F_0	BY939965
E_FL_e100_25F05_R_0	BY943897
E_FL_e100_25F07_R_0	BY943898
E_FL_e100_25F08_F_0	BY939966
E_FL_e100_25F10_F_0	BY939967
E_FL_e100_25F10_R_0	BY943899
E_FL_e100_25F11_F_0	BY939968
E_FL_e100_25F13_F_0	BY939969
E_FL_e100_25F14_F_0	BY939970
E_FL_e100_25F14_R_0	BY943900
E_FL_e100_25F15_F_0	BY939971
E_FL_e100_25F15_R_0	BY943901
E_FL_e100_25F16_F_0	BY939972
E_FL_e100_25F16_R_0	BY943902
E_FL_e100_25F18_F_0	BY939973
E_FL_e100_25F18_R_0	BY943903
E_FL_e100_25F20_F_0	BY939974
E_FL_e100_25F22_F_0	BY939975
E_FL_e100_25F24_F_0	BY939976
E_FL_e100_25F24_R_0	BY943904
E_FL_e100_25G05_F_0	BY939977

E_FL_e100_25G05_R_0	BY943905
E_FL_e100_25G06_R_0	BY943906
E_FL_e100_25G07_F_0	BY939978
E_FL_e100_25G07_R_0	BY943907
E_FL_e100_25G08_F_0	BY939979
E_FL_e100_25G09_F_0	BY939980
E_FL_e100_25G11_F_0	BY939981
E_FL_e100_25G14_F_0	BY939982
E_FL_e100_25G15_F_0	BY939983
E_FL_e100_25G15_R_0	BY943908
E_FL_e100_25G17_F_0	BY939984
E_FL_e100_25G17_R_0	BY943909
E_FL_e100_25G20_F_0	BY939985
E_FL_e100_25G22_F_0	BY939986
E_FL_e100_25G24_F_0	BY939987
E_FL_e100_25H01_F_0	BY939988
E_FL_e100_25H03_R_0	BY943910
E_FL_e100_25H04_F_0	BY939989
E_FL_e100_25H07_F_0	BY939990
E_FL_e100_25H08_R_0	BY943911
E_FL_e100_25H09_F_0	BY939991
E_FL_e100_25H09_R_0	BY943912
E_FL_e100_25H12_F_0	BY939992
E_FL_e100_25H13_F_0	BY939993
E_FL_e100_25H14_F_0	BY939994
E_FL_e100_25H14_R_0	BY943913
E_FL_e100_25H17_F_0	BY939995
E_FL_e100_25H17_R_0	BY943914
E_FL_e100_25H18_F_0	BY939996
E_FL_e100_25H18_R_0	BY943915
E_FL_e100_25H19_F_0	BY939997
E_FL_e100_25H19_R_0	BY943916
E_FL_e100_25H20_F_0	BY939998
E_FL_e100_25H20_R_0	BY943917
E_FL_e100_25H21_F_0	BY939999
E_FL_e100_25H21_R_0	BY943918
E_FL_e100_25H22_F_0	BY940000
E_FL_e100_25H23_F_0	BY940001
E_FL_e100_25H24_F_0	BY940002
E_FL_e100_25H24_R_0	BY943919
E_FL_e100_25I01_F_0	BY940003
E_FL_e100_25I01_R_0	BY943920
E_FL_e100_25I04_F_0	BY940004
E_FL_e100_25I04_R_0	BY943921
E_FL_e100_25I05_F_0	BY940005
E_FL_e100_25I05_R_0	BY943922
E_FL_e100_25I06_F_0	BY940006
E_FL_e100_25I11_F_0	BY940007
E_FL_e100_25I12_F_0	BY940008
E_FL_e100_25I13_F_0	BY940009
E_FL_e100_25I14_F_0	BY940010
E_FL_e100_25I15_F_0	BY940011
E_FL_e100_25I15_R_0	BY943923
E_FL_e100_25I17_F_0	BY940012

E_FL_e100_25I17_R_0	BY943924
E_FL_e100_25I18_R_0	BY943925
E_FL_e100_25I19_F_0	BY940013
E_FL_e100_25I21_F_0	BY940014
E_FL_e100_25I22_F_0	BY940015
E_FL_e100_25I23_R_0	BY943926
E_FL_e100_25J02_F_0	BY940016
E_FL_e100_25J02_R_0	BY943927
E_FL_e100_25J04_F_0	BY940017
E_FL_e100_25J06_F_0	BY940018
E_FL_e100_25J06_R_0	BY943928
E_FL_e100_25J08_F_0	BY940019
E_FL_e100_25J09_R_0	BY943929
E_FL_e100_25J10_F_0	BY940020
E_FL_e100_25J10_R_0	BY943930
E_FL_e100_25J12_F_0	BY940021
E_FL_e100_25J13_F_0	BY940022
E_FL_e100_25J14_F_0	BY940023
E_FL_e100_25J14_R_0	BY943931
E_FL_e100_25J17_F_0	BY940024
E_FL_e100_25J17_R_0	BY943932
E_FL_e100_25J18_F_0	BY940025
E_FL_e100_25J18_R_0	BY943933
E_FL_e100_25J19_R_0	BY943934
E_FL_e100_25J20_F_0	BY940026
E_FL_e100_25J21_F_0	BY940027
E_FL_e100_25J22_F_0	BY940028
E_FL_e100_25J22_R_0	BY943935
E_FL_e100_25J24_F_0	BY940029
E_FL_e100_25J24_R_0	BY943936
E_FL_e100_25K02_F_0	BY940030
E_FL_e100_25K05_F_0	BY940031
E_FL_e100_25K05_R_0	BY943937
E_FL_e100_25K11_F_0	BY940032
E_FL_e100_25K12_F_0	BY940033
E_FL_e100_25K16_F_0	BY940034
E_FL_e100_25K17_F_0	BY940035
E_FL_e100_25K17_R_0	BY943938
E_FL_e100_25K18_F_0	BY940036
E_FL_e100_25K20_F_0	BY940037
E_FL_e100_25K21_F_0	BY940038
E_FL_e100_25L01_F_0	BY940039
E_FL_e100_25L01_R_0	BY943939
E_FL_e100_25L04_F_0	BY940040
E_FL_e100_25L06_F_0	BY940041
E_FL_e100_25L07_F_0	BY940042
E_FL_e100_25L08_F_0	BY940043
E_FL_e100_25L08_R_0	BY943940
E_FL_e100_25L10_F_0	BY940044
E_FL_e100_25L10_R_0	BY943941
E_FL_e100_25L11_F_0	BY940045
E_FL_e100_25L11_R_0	BY943942
E_FL_e100_25L12_F_0	BY940046
E_FL_e100_25L13_R_0	BY943943

E_FL_e100_25L14_F_0	BY940047
E_FL_e100_25L15_F_0	BY940048
E_FL_e100_25L15_R_0	BY943944
E_FL_e100_25L17_F_0	BY940049
E_FL_e100_25L18_F_0	BY940050
E_FL_e100_25L23_F_0	BY940051
E_FL_e100_25L23_R_0	BY943945
E_FL_e100_25L24_F_0	BY940052
E_FL_e100_25M01_R_0	BY943946
E_FL_e100_25M02_F_0	BY940053
E_FL_e100_25M04_R_0	BY943947
E_FL_e100_25M05_F_0	BY940054
E_FL_e100_25M05_R_0	BY943948
E_FL_e100_25M07_F_0	BY940055
E_FL_e100_25M07_R_0	BY943949
E_FL_e100_25M08_F_0	BY940056
E_FL_e100_25M09_F_0	BY940057
E_FL_e100_25M11_F_0	BY940058
E_FL_e100_25M11_R_0	BY943950
E_FL_e100_25M12_F_0	BY940059
E_FL_e100_25M12_R_0	BY943951
E_FL_e100_25M14_F_0	BY940060
E_FL_e100_25M18_F_0	BY940061
E_FL_e100_25M18_R_0	BY943952
E_FL_e100_25M21_F_0	BY940062
E_FL_e100_25N01_F_0	BY940063
E_FL_e100_25N02_F_0	BY940064
E_FL_e100_25N04_F_0	BY940065
E_FL_e100_25N04_R_0	BY943953
E_FL_e100_25N05_F_0	BY940066
E_FL_e100_25N11_R_0	BY943954
E_FL_e100_25N14_F_0	BY940067
E_FL_e100_25N14_R_0	BY943955
E_FL_e100_25N15_F_0	BY940068
E_FL_e100_25N15_R_0	BY943956
E_FL_e100_25N16_F_0	BY940069
E_FL_e100_25N16_R_0	BY943957
E_FL_e100_25N18_F_0	BY940070
E_FL_e100_25N19_F_0	BY940071
E_FL_e100_25N19_R_0	BY943958
E_FL_e100_25N20_F_0	BY940072
E_FL_e100_25N20_R_0	BY943959
E_FL_e100_25N21_F_0	BY940073
E_FL_e100_25N21_R_0	BY943960
E_FL_e100_25N22_F_0	BY940074
E_FL_e100_25O02_F_0	BY940075
E_FL_e100_25O04_F_0	BY940076
E_FL_e100_25O04_R_0	BY943961
E_FL_e100_25O05_F_0	BY940077
E_FL_e100_25O06_R_0	BY943962
E_FL_e100_25O07_F_0	BY940078
E_FL_e100_25O08_F_0	BY940079
E_FL_e100_25O10_F_0	BY940080
E_FL_e100_25O11_F_0	BY940081

E_FL_e100_25O14_F_0	BY940082
E_FL_e100_25O15_F_0	BY940083
E_FL_e100_25O18_F_0	BY940084
E_FL_e100_25O19_F_0	BY940085
E_FL_e100_25O22_F_0	BY940086
E_FL_e100_25O22_R_0	BY943963
E_FL_e100_25O24_F_0	BY940087
E_FL_e100_25O24_R_0	BY943964
E_FL_e100_25P01_F_0	BY940088
E_FL_e100_25P01_R_0	BY943965
E_FL_e100_25P02_F_0	BY940089
E_FL_e100_25P04_F_0	BY940090
E_FL_e100_25P04_R_0	BY943966
E_FL_e100_25P05_F_0	BY940091
E_FL_e100_25P18_F_0	BY940092
E_FL_e100_25P20_F_0	BY940093
E_FL_e100_25P21_F_0	BY940094
E_FL_e100_25P22_F_0	BY940095
E_FL_e100_25P22_R_0	BY943967
E_FL_e100_25P24_F_0	BY940096
E_FL_e100_26A03_F_0	BY940097
E_FL_e100_26A04_F_0	BY940098
E_FL_e100_26A05_F_0	BY940099
E_FL_e100_26A06_F_0	BY940100
E_FL_e100_26A06_R_0	BY943968
E_FL_e100_26A08_F_0	BY940101
E_FL_e100_26A08_R_0	BY943969
E_FL_e100_26A10_F_0	BY940102
E_FL_e100_26A12_F_0	BY940103
E_FL_e100_26A14_F_0	BY940104
E_FL_e100_26A15_F_0	BY940105
E_FL_e100_26A15_R_0	BY943970
E_FL_e100_26A17_F_0	BY940106
E_FL_e100_26A19_F_0	BY940107
E_FL_e100_26A23_F_0	BY940108
E_FL_e100_26A23_R_0	BY943971
E_FL_e100_26B02_F_0	BY940109
E_FL_e100_26B03_F_0	BY940110
E_FL_e100_26B03_R_0	BY943972
E_FL_e100_26B04_F_0	BY940111
E_FL_e100_26B06_R_0	BY943973
E_FL_e100_26B07_R_0	BY943974
E_FL_e100_26B09_F_0	BY940112
E_FL_e100_26B09_R_0	BY943975
E_FL_e100_26B10_F_0	BY940113
E_FL_e100_26B10_R_0	BY943976
E_FL_e100_26B11_F_0	BY940114
E_FL_e100_26B11_R_0	BY943977
E_FL_e100_26B12_F_0	BY940115
E_FL_e100_26B12_R_0	BY943978
E_FL_e100_26B15_F_0	BY940116
E_FL_e100_26B15_R_0	BY943979
E_FL_e100_26B16_F_0	BY940117
E_FL_e100_26B17_F_0	BY940118

E_FL_e100_26B20_F_0	BY940119
E_FL_e100_26B20_R_0	BY943980
E_FL_e100_26B21_F_0	BY940120
E_FL_e100_26B21_R_0	BY943981
E_FL_e100_26C01_F_0	BY940121
E_FL_e100_26C03_F_0	BY940122
E_FL_e100_26C04_F_0	BY940123
E_FL_e100_26C04_R_0	BY943982
E_FL_e100_26C05_F_0	BY940124
E_FL_e100_26C06_F_0	BY940125
E_FL_e100_26C06_R_0	BY943983
E_FL_e100_26C07_F_0	BY940126
E_FL_e100_26C07_R_0	BY943984
E_FL_e100_26C09_F_0	BY940127
E_FL_e100_26C10_F_0	BY940128
E_FL_e100_26C10_R_0	BY943985
E_FL_e100_26C11_R_0	BY943986
E_FL_e100_26C12_R_0	BY943987
E_FL_e100_26C13_F_0	BY940129
E_FL_e100_26C13_R_0	BY943988
E_FL_e100_26C14_F_0	BY940130
E_FL_e100_26C14_R_0	BY943989
E_FL_e100_26C15_F_0	BY940131
E_FL_e100_26C15_R_0	BY943990
E_FL_e100_26C16_R_0	BY943991
E_FL_e100_26C17_F_0	BY940132
E_FL_e100_26C17_R_0	BY943992
E_FL_e100_26C19_F_0	BY940133
E_FL_e100_26C19_R_0	BY943993
E_FL_e100_26C21_F_0	BY940134
E_FL_e100_26C22_F_0	BY940135
E_FL_e100_26C22_R_0	BY943994
E_FL_e100_26D01_F_0	BY940136
E_FL_e100_26D02_F_0	BY940137
E_FL_e100_26D03_F_0	BY940138
E_FL_e100_26D04_F_0	BY940139
E_FL_e100_26D04_R_0	BY943995
E_FL_e100_26D05_F_0	BY940140
E_FL_e100_26D05_R_0	BY943996
E_FL_e100_26D08_F_0	BY940141
E_FL_e100_26D12_F_0	BY940142
E_FL_e100_26D13_F_0	BY940143
E_FL_e100_26D13_R_0	BY943997
E_FL_e100_26D14_F_0	BY940144
E_FL_e100_26D16_F_0	BY940145
E_FL_e100_26D16_R_0	BY943998
E_FL_e100_26D17_F_0	BY940146
E_FL_e100_26D17_R_0	BY943999
E_FL_e100_26D20_F_0	BY940147
E_FL_e100_26D21_F_0	BY940148
E_FL_e100_26D21_R_0	BY944000
E_FL_e100_26D22_F_0	BY940149
E_FL_e100_26D22_R_0	BY944001
E_FL_e100_26D23_F_0	BY940150

E_FL_e100_26D24_F_0	BY940151
E_FL_e100_26E02_F_0	BY940152
E_FL_e100_26E03_F_0	BY940153
E_FL_e100_26E05_F_0	BY940154
E_FL_e100_26E05_R_0	BY944002
E_FL_e100_26E06_F_0	BY940155
E_FL_e100_26E08_F_0	BY940156
E_FL_e100_26E12_F_0	BY940157
E_FL_e100_26E13_R_0	BY944003
E_FL_e100_26E15_F_0	BY940158
E_FL_e100_26E15_R_0	BY944004
E_FL_e100_26E16_F_0	BY940159
E_FL_e100_26E16_R_0	BY944005
E_FL_e100_26E19_F_0	BY940160
E_FL_e100_26E19_R_0	BY944006
E_FL_e100_26E21_F_0	BY940161
E_FL_e100_26E24_F_0	BY940162
E_FL_e100_26F01_F_0	BY940163
E_FL_e100_26F01_R_0	BY944007
E_FL_e100_26F02_F_0	BY940164
E_FL_e100_26F04_F_0	BY940165
E_FL_e100_26F04_R_0	BY944008
E_FL_e100_26F08_F_0	BY940166
E_FL_e100_26F08_R_0	BY944009
E_FL_e100_26F09_F_0	BY940167
E_FL_e100_26F14_F_0	BY940168
E_FL_e100_26F16_F_0	BY940169
E_FL_e100_26F16_R_0	BY944010
E_FL_e100_26F17_R_0	BY944011
E_FL_e100_26F19_F_0	BY940170
E_FL_e100_26F19_R_0	BY944012
E_FL_e100_26F20_F_0	BY940171
E_FL_e100_26F21_F_0	BY940172
E_FL_e100_26F22_F_0	BY940173
E_FL_e100_26F22_R_0	BY944013
E_FL_e100_26F23_F_0	BY940174
E_FL_e100_26F23_R_0	BY944014
E_FL_e100_26F24_F_0	BY940175
E_FL_e100_26G01_F_0	BY940176
E_FL_e100_26G01_R_0	BY944015
E_FL_e100_26G02_F_0	BY940177
E_FL_e100_26G03_F_0	BY940178
E_FL_e100_26G04_F_0	BY940179
E_FL_e100_26G04_R_0	BY944016
E_FL_e100_26G05_F_0	BY940180
E_FL_e100_26G07_F_0	BY940181
E_FL_e100_26G07_R_0	BY944017
E_FL_e100_26G08_F_0	BY940182
E_FL_e100_26G08_R_0	BY944018
E_FL_e100_26G09_F_0	BY940183
E_FL_e100_26G11_F_0	BY940184
E_FL_e100_26G11_R_0	BY944019
E_FL_e100_26G12_F_0	BY940185
E_FL_e100_26G19_F_0	BY940186

E_FL_e100_26G23_F_0	BY940187
E_FL_e100_26G23_R_0	BY944020
E_FL_e100_26H01_F_0	BY940188
E_FL_e100_26H03_F_0	BY940189
E_FL_e100_26H03_R_0	BY944021
E_FL_e100_26H06_F_0	BY940190
E_FL_e100_26H06_R_0	BY944022
E_FL_e100_26H07_F_0	BY940191
E_FL_e100_26H08_F_0	BY940192
E_FL_e100_26H09_R_0	BY944023
E_FL_e100_26H13_F_0	BY940193
E_FL_e100_26H14_R_0	BY944024
E_FL_e100_26H15_F_0	BY940194
E_FL_e100_26H15_R_0	BY944025
E_FL_e100_26H17_F_0	BY940195
E_FL_e100_26H20_F_0	BY940196
E_FL_e100_26H21_F_0	BY940197
E_FL_e100_26H22_F_0	BY940198
E_FL_e100_26H22_R_0	BY944026
E_FL_e100_26H23_F_0	BY940199
E_FL_e100_26I02_R_0	BY944027
E_FL_e100_26I03_F_0	BY940200
E_FL_e100_26I04_F_0	BY940201
E_FL_e100_26I04_R_0	BY944028
E_FL_e100_26I05_F_0	BY940202
E_FL_e100_26I07_F_0	BY940203
E_FL_e100_26I09_F_0	BY940204
E_FL_e100_26I09_R_0	BY944029
E_FL_e100_26I10_F_0	BY940205
E_FL_e100_26I10_R_0	BY944030
E_FL_e100_26I12_F_0	BY940206
E_FL_e100_26I13_R_0	BY944031
E_FL_e100_26I21_F_0	BY940207
E_FL_e100_26I22_F_0	BY940208
E_FL_e100_26I24_R_0	BY944032
E_FL_e100_26J02_F_0	BY940209
E_FL_e100_26J03_F_0	BY940210
E_FL_e100_26J04_F_0	BY940211
E_FL_e100_26J04_R_0	BY944033
E_FL_e100_26J05_F_0	BY940212
E_FL_e100_26J06_F_0	BY940213
E_FL_e100_26J06_R_0	BY944034
E_FL_e100_26J07_F_0	BY940214
E_FL_e100_26J07_R_0	BY944035
E_FL_e100_26J08_F_0	BY940215
E_FL_e100_26J09_F_0	BY940216
E_FL_e100_26J11_F_0	BY940217
E_FL_e100_26J14_F_0	BY940218
E_FL_e100_26J14_R_0	BY944036
E_FL_e100_26J15_F_0	BY940219
E_FL_e100_26J15_R_0	BY944037
E_FL_e100_26J16_F_0	BY940220
E_FL_e100_26J17_F_0	BY940221
E_FL_e100_26J18_F_0	BY940222

E_FL_e100_26J18_R_0	BY944038
E_FL_e100_26J19_F_0	BY940223
E_FL_e100_26J19_R_0	BY944039
E_FL_e100_26J21_F_0	BY940224
E_FL_e100_26K01_F_0	BY940225
E_FL_e100_26K01_R_0	BY944040
E_FL_e100_26K03_F_0	BY940226
E_FL_e100_26K04_F_0	BY940227
E_FL_e100_26K04_R_0	BY944041
E_FL_e100_26K06_R_0	BY944042
E_FL_e100_26K07_F_0	BY940228
E_FL_e100_26K08_F_0	BY940229
E_FL_e100_26K09_F_0	BY940230
E_FL_e100_26K09_R_0	BY944043
E_FL_e100_26K10_F_0	BY940231
E_FL_e100_26K10_R_0	BY944044
E_FL_e100_26K11_F_0	BY940232
E_FL_e100_26K11_R_0	BY944045
E_FL_e100_26K12_F_0	BY940233
E_FL_e100_26K18_R_0	BY944046
E_FL_e100_26K19_F_0	BY940234
E_FL_e100_26K21_F_0	BY940235
E_FL_e100_26K21_R_0	BY944047
E_FL_e100_26K23_F_0	BY940236
E_FL_e100_26K24_F_0	BY940237
E_FL_e100_26K24_R_0	BY944048
E_FL_e100_26L01_F_0	BY940238
E_FL_e100_26L01_R_0	BY944049
E_FL_e100_26L02_F_0	BY940239
E_FL_e100_26L02_R_0	BY944050
E_FL_e100_26L03_R_0	BY944051
E_FL_e100_26L04_F_0	BY940240
E_FL_e100_26L04_R_0	BY944052
E_FL_e100_26L06_F_0	BY940241
E_FL_e100_26L08_F_0	BY940242
E_FL_e100_26L09_F_0	BY940243
E_FL_e100_26L13_F_0	BY940244
E_FL_e100_26L14_F_0	BY940245
E_FL_e100_26L15_R_0	BY944053
E_FL_e100_26L17_F_0	BY940246
E_FL_e100_26L17_R_0	BY944054
E_FL_e100_26L20_F_0	BY940247
E_FL_e100_26L20_R_0	BY944055
E_FL_e100_26L21_F_0	BY940248
E_FL_e100_26L21_R_0	BY944056
E_FL_e100_26L22_F_0	BY940249
E_FL_e100_26L22_R_0	BY944057
E_FL_e100_26L23_F_0	BY940250
E_FL_e100_26L24_F_0	BY940251
E_FL_e100_26L24_R_0	BY944058
E_FL_e100_26M04_F_0	BY940252
E_FL_e100_26M06_F_0	BY940253
E_FL_e100_26M06_R_0	BY944059
E_FL_e100_26M09_F_0	BY940254

E_FL_e100_26M10_F_0	BY940255
E_FL_e100_26M12_F_0	BY940256
E_FL_e100_26M14_F_0	BY940257
E_FL_e100_26M14_R_0	BY944060
E_FL_e100_26M16_F_0	BY940258
E_FL_e100_26M16_R_0	BY944061
E_FL_e100_26M17_F_0	BY940259
E_FL_e100_26M17_R_0	BY944062
E_FL_e100_26M18_F_0	BY940260
E_FL_e100_26M19_R_0	BY944063
E_FL_e100_26M21_F_0	BY940261
E_FL_e100_26M21_R_0	BY944064
E_FL_e100_26M22_F_0	BY940262
E_FL_e100_26M22_R_0	BY944065
E_FL_e100_26N02_F_0	BY940263
E_FL_e100_26N04_F_0	BY940264
E_FL_e100_26N04_R_0	BY944066
E_FL_e100_26N07_F_0	BY940265
E_FL_e100_26N08_F_0	BY940266
E_FL_e100_26N10_F_0	BY940267
E_FL_e100_26N10_R_0	BY944067
E_FL_e100_26N11_F_0	BY940268
E_FL_e100_26N11_R_0	BY944068
E_FL_e100_26N13_F_0	BY940269
E_FL_e100_26N13_R_0	BY944069
E_FL_e100_26N14_F_0	BY940270
E_FL_e100_26N14_R_0	BY944070
E_FL_e100_26N16_F_0	BY940271
E_FL_e100_26N17_F_0	BY940272
E_FL_e100_26N21_F_0	BY940273
E_FL_e100_26N23_R_0	BY944071
E_FL_e100_26N24_F_0	BY940274
E_FL_e100_26N24_R_0	BY944072
E_FL_e100_26O03_F_0	BY940275
E_FL_e100_26O03_R_0	BY944073
E_FL_e100_26O04_F_0	BY940276
E_FL_e100_26O05_F_0	BY940277
E_FL_e100_26O06_F_0	BY940278
E_FL_e100_26O06_R_0	BY944074
E_FL_e100_26O07_F_0	BY940279
E_FL_e100_26O07_R_0	BY944075
E_FL_e100_26O08_F_0	BY940280
E_FL_e100_26O08_R_0	BY944076
E_FL_e100_26O09_F_0	BY940281
E_FL_e100_26O12_F_0	BY940282
E_FL_e100_26O14_F_0	BY940283
E_FL_e100_26O14_R_0	BY944077
E_FL_e100_26O15_F_0	BY940284
E_FL_e100_26O15_R_0	BY944078
E_FL_e100_26O16_F_0	BY940285
E_FL_e100_26O18_F_0	BY940286
E_FL_e100_26O18_R_0	BY944079
E_FL_e100_26O19_F_0	BY940287
E_FL_e100_26O19_R_0	BY944080

E_FL_e100_26O20_R_0	BY944081
E_FL_e100_26O23_R_0	BY944082
E_FL_e100_26O24_F_0	BY940288
E_FL_e100_26P02_F_0	BY940289
E_FL_e100_26P03_F_0	BY940290
E_FL_e100_26P04_F_0	BY940291
E_FL_e100_26P04_R_0	BY944083
E_FL_e100_26P07_F_0	BY940292
E_FL_e100_26P08_F_0	BY940293
E_FL_e100_26P10_F_0	BY940294
E_FL_e100_26P10_R_0	BY944084
E_FL_e100_26P11_F_0	BY940295
E_FL_e100_26P13_F_0	BY940296
E_FL_e100_26P13_R_0	BY944085
E_FL_e100_26P15_F_0	BY940297
E_FL_e100_26P16_F_0	BY940298
E_FL_e100_26P16_R_0	BY944086
E_FL_e100_26P17_F_0	BY940299
E_FL_e100_26P17_R_0	BY944087
E_FL_e100_26P20_F_0	BY940300
E_FL_e100_26P20_R_0	BY944088
E_FL_e100_26P21_F_0	BY940301
E_FL_e100_26P21_R_0	BY944089
E_FL_e100_26P22_R_0	BY944090
E_FL_e100_26P23_F_0	BY940302
E_FL_e100_26P24_F_0	BY940303
E_FL_e100_27A02_F_0	BY940304
E_FL_e100_27A06_F_0	BY940305
E_FL_e100_27A08_F_0	BY940306
E_FL_e100_27A10_F_0	BY940307
E_FL_e100_27A10_R_0	BY944091
E_FL_e100_27A12_R_0	BY944092
E_FL_e100_27A14_F_0	BY940308
E_FL_e100_27A14_R_0	BY944093
E_FL_e100_27A19_F_0	BY940309
E_FL_e100_27A20_F_0	BY940310
E_FL_e100_27A20_R_0	BY944094
E_FL_e100_27A22_F_0	BY940311
E_FL_e100_27A23_F_0	BY940312
E_FL_e100_27B01_F_0	BY940313
E_FL_e100_27B05_R_0	BY944095
E_FL_e100_27B07_F_0	BY940314
E_FL_e100_27B08_F_0	BY940315
E_FL_e100_27B08_R_0	BY944096
E_FL_e100_27B09_F_0	BY940316
E_FL_e100_27B10_F_0	BY940317
E_FL_e100_27B12_F_0	BY940318
E_FL_e100_27B12_R_0	BY944097
E_FL_e100_27B13_F_0	BY940319
E_FL_e100_27B15_F_0	BY940320
E_FL_e100_27B15_R_0	BY944098
E_FL_e100_27B16_F_0	BY940321
E_FL_e100_27B18_F_0	BY940322
E_FL_e100_27B18_R_0	BY944099

E_FL_e100_27B19_F_0	BY940323
E_FL_e100_27B19_R_0	BY944100
E_FL_e100_27B22_F_0	BY940324
E_FL_e100_27B22_R_0	BY944101
E_FL_e100_27B23_F_0	BY940325
E_FL_e100_27B23_R_0	BY944102
E_FL_e100_27C02_F_0	BY940326
E_FL_e100_27C02_R_0	BY944103
E_FL_e100_27C03_F_0	BY940327
E_FL_e100_27C03_R_0	BY944104
E_FL_e100_27C04_F_0	BY940328
E_FL_e100_27C04_R_0	BY944105
E_FL_e100_27C05_F_0	BY940329
E_FL_e100_27C05_R_0	BY944106
E_FL_e100_27C07_F_0	BY940330
E_FL_e100_27C11_F_0	BY940331
E_FL_e100_27C12_F_0	BY940332
E_FL_e100_27C13_F_0	BY940333
E_FL_e100_27C14_F_0	BY940334
E_FL_e100_27C15_R_0	BY944107
E_FL_e100_27C16_F_0	BY940335
E_FL_e100_27C16_R_0	BY944108
E_FL_e100_27C17_R_0	BY944109
E_FL_e100_27C19_F_0	BY940336
E_FL_e100_27C21_F_0	BY940337
E_FL_e100_27C22_F_0	BY940338
E_FL_e100_27C22_R_0	BY944110
E_FL_e100_27C24_F_0	BY940339
E_FL_e100_27D03_F_0	BY940340
E_FL_e100_27D05_F_0	BY940341
E_FL_e100_27D06_F_0	BY940342
E_FL_e100_27D06_R_0	BY944111
E_FL_e100_27D10_F_0	BY940343
E_FL_e100_27D10_R_0	BY944112
E_FL_e100_27D13_F_0	BY940344
E_FL_e100_27D13_R_0	BY944113
E_FL_e100_27D14_F_0	BY940345
E_FL_e100_27D14_R_0	BY944114
E_FL_e100_27D15_F_0	BY940346
E_FL_e100_27D16_F_0	BY940347
E_FL_e100_27D16_R_0	BY944115
E_FL_e100_27D18_F_0	BY940348
E_FL_e100_27D19_F_0	BY940349
E_FL_e100_27D19_R_0	BY944116
E_FL_e100_27D23_F_0	BY940350
E_FL_e100_27D24_F_0	BY940351
E_FL_e100_27E09_F_0	BY940352
E_FL_e100_27E11_F_0	BY940353
E_FL_e100_27E11_R_0	BY944117
E_FL_e100_27E14_F_0	BY940354
E_FL_e100_27E14_R_0	BY944118
E_FL_e100_27E19_R_0	BY944119
E_FL_e100_27E20_R_0	BY944120
E_FL_e100_27E21_F_0	BY940355

E_FL_e100_27E22_R_0	BY944121
E_FL_e100_27F02_F_0	BY940356
E_FL_e100_27F02_R_0	BY944122
E_FL_e100_27F03_F_0	BY940357
E_FL_e100_27F04_F_0	BY940358
E_FL_e100_27F05_F_0	BY940359
E_FL_e100_27F05_R_0	BY944123
E_FL_e100_27F07_F_0	BY940360
E_FL_e100_27F08_R_0	BY944124
E_FL_e100_27F09_F_0	BY940361
E_FL_e100_27F09_R_0	BY944125
E_FL_e100_27F10_F_0	BY940362
E_FL_e100_27F10_R_0	BY944126
E_FL_e100_27F11_F_0	BY940363
E_FL_e100_27F11_R_0	BY944127
E_FL_e100_27F13_F_0	BY940364
E_FL_e100_27F15_F_0	BY940365
E_FL_e100_27F16_F_0	BY940366
E_FL_e100_27F16_R_0	BY944128
E_FL_e100_27F18_F_0	BY940367
E_FL_e100_27F20_F_0	BY940368
E_FL_e100_27F21_F_0	BY940369
E_FL_e100_27F21_R_0	BY944129
E_FL_e100_27F23_F_0	BY940370
E_FL_e100_27F24_F_0	BY940371
E_FL_e100_27F24_R_0	BY944130
E_FL_e100_27G03_F_0	BY940372
E_FL_e100_27G04_F_0	BY940373
E_FL_e100_27G04_R_0	BY944131
E_FL_e100_27G05_F_0	BY940374
E_FL_e100_27G05_R_0	BY944132
E_FL_e100_27G08_F_0	BY940375
E_FL_e100_27G08_R_0	BY944133
E_FL_e100_27G09_F_0	BY940376
E_FL_e100_27G09_R_0	BY944134
E_FL_e100_27G15_F_0	BY940377
E_FL_e100_27G17_F_0	BY940378
E_FL_e100_27G20_R_0	BY944135
E_FL_e100_27G21_R_0	BY944136
E_FL_e100_27G22_R_0	BY944137
E_FL_e100_27G23_R_0	BY944138
E_FL_e100_27G24_F_0	BY940379
E_FL_e100_27H03_F_0	BY940380
E_FL_e100_27H04_F_0	BY940381
E_FL_e100_27H04_R_0	BY944139
E_FL_e100_27H05_R_0	BY944140
E_FL_e100_27H12_F_0	BY940382
E_FL_e100_27H12_R_0	BY944141
E_FL_e100_27H13_F_0	BY940383
E_FL_e100_27H16_F_0	BY940384
E_FL_e100_27H16_R_0	BY944142
E_FL_e100_27H20_F_0	BY940385
E_FL_e100_27H21_F_0	BY940386
E_FL_e100_27H21_R_0	BY944143

E_FL_e100_27H22_F_0	BY940387
E_FL_e100_27H24_R_0	BY944144
E_FL_e100_27I03_F_0	BY940388
E_FL_e100_27I03_R_0	BY944145
E_FL_e100_27I06_F_0	BY940389
E_FL_e100_27I06_R_0	BY944146
E_FL_e100_27I08_F_0	BY940390
E_FL_e100_27I09_F_0	BY940391
E_FL_e100_27I09_R_0	BY944147
E_FL_e100_27I11_F_0	BY940392
E_FL_e100_27I11_R_0	BY944148
E_FL_e100_27I15_F_0	BY940393
E_FL_e100_27I17_F_0	BY940394
E_FL_e100_27I17_R_0	BY944149
E_FL_e100_27I21_F_0	BY940395
E_FL_e100_27I21_R_0	BY944150
E_FL_e100_27I22_F_0	BY940396
E_FL_e100_27I23_F_0	BY940397
E_FL_e100_27I23_R_0	BY944151
E_FL_e100_27J02_F_0	BY940398
E_FL_e100_27J04_R_0	BY944152
E_FL_e100_27J07_F_0	BY940399
E_FL_e100_27J08_F_0	BY940400
E_FL_e100_27J08_R_0	BY944153
E_FL_e100_27J10_F_0	BY940401
E_FL_e100_27J10_R_0	BY944154
E_FL_e100_27J14_F_0	BY940402
E_FL_e100_27J16_F_0	BY940403
E_FL_e100_27J16_R_0	BY944155
E_FL_e100_27J17_F_0	BY940404
E_FL_e100_27J19_F_0	BY940405
E_FL_e100_27J20_F_0	BY940406
E_FL_e100_27J21_F_0	BY940407
E_FL_e100_27K03_F_0	BY940408
E_FL_e100_27K07_F_0	BY940409
E_FL_e100_27K08_F_0	BY940410
E_FL_e100_27K08_R_0	BY944156
E_FL_e100_27K09_R_0	BY944157
E_FL_e100_27K13_F_0	BY940411
E_FL_e100_27K14_F_0	BY940412
E_FL_e100_27K14_R_0	BY944158
E_FL_e100_27K16_F_0	BY940413
E_FL_e100_27K16_R_0	BY944159
E_FL_e100_27K19_F_0	BY940414
E_FL_e100_27K19_R_0	BY944160
E_FL_e100_27K20_F_0	BY940415
E_FL_e100_27K22_F_0	BY940416
E_FL_e100_27K22_R_0	BY944161
E_FL_e100_27L01_R_0	BY944162
E_FL_e100_27L03_F_0	BY940417
E_FL_e100_27L04_F_0	BY940418
E_FL_e100_27L04_R_0	BY944163
E_FL_e100_27L06_F_0	BY940419
E_FL_e100_27L06_R_0	BY944164

E_FL_e100_27L07_F_0	BY940420
E_FL_e100_27L08_F_0	BY940421
E_FL_e100_27L08_R_0	BY944165
E_FL_e100_27L14_F_0	BY940422
E_FL_e100_27L14_R_0	BY944166
E_FL_e100_27L19_F_0	BY940423
E_FL_e100_27L20_R_0	BY944167
E_FL_e100_27L23_F_0	BY940424
E_FL_e100_27M01_R_0	BY944168
E_FL_e100_27M03_F_0	BY940425
E_FL_e100_27M04_F_0	BY940426
E_FL_e100_27M05_F_0	BY940427
E_FL_e100_27M05_R_0	BY944169
E_FL_e100_27M07_F_0	BY940428
E_FL_e100_27M07_R_0	BY944170
E_FL_e100_27M09_F_0	BY940429
E_FL_e100_27M10_F_0	BY940430
E_FL_e100_27M10_R_0	BY944171
E_FL_e100_27M11_F_0	BY940431
E_FL_e100_27M12_F_0	BY940432
E_FL_e100_27M12_R_0	BY944172
E_FL_e100_27M13_R_0	BY944173
E_FL_e100_27M16_F_0	BY940433
E_FL_e100_27M16_R_0	BY944174
E_FL_e100_27M17_F_0	BY940434
E_FL_e100_27M22_F_0	BY940435
E_FL_e100_27M22_R_0	BY944175
E_FL_e100_27M24_F_0	BY940436
E_FL_e100_27M24_R_0	BY944176
E_FL_e100_27N02_F_0	BY940437
E_FL_e100_27N02_R_0	BY944177
E_FL_e100_27N03_F_0	BY940438
E_FL_e100_27N04_F_0	BY940439
E_FL_e100_27N05_F_0	BY940440
E_FL_e100_27N05_R_0	BY944178
E_FL_e100_27N06_F_0	BY940441
E_FL_e100_27N06_R_0	BY944179
E_FL_e100_27N08_F_0	BY940442
E_FL_e100_27N10_F_0	BY940443
E_FL_e100_27N10_R_0	BY944180
E_FL_e100_27N11_F_0	BY940444
E_FL_e100_27N11_R_0	BY944181
E_FL_e100_27N12_F_0	BY940445
E_FL_e100_27N12_R_0	BY944182
E_FL_e100_27N14_F_0	BY940446
E_FL_e100_27N14_R_0	BY944183
E_FL_e100_27N15_R_0	BY944184
E_FL_e100_27N16_F_0	BY940447
E_FL_e100_27N20_F_0	BY940448
E_FL_e100_27N22_F_0	BY940449
E_FL_e100_27N23_F_0	BY940450
E_FL_e100_27N23_R_0	BY944185
E_FL_e100_27O03_F_0	BY940451
E_FL_e100_27O03_R_0	BY944186

	E_FL_e100_27O07_F_0	BY940452
	E_FL_e100_27O08_F_0	BY940453
	E_FL_e100_27O08_R_0	BY944187
	E_FL_e100_27O10_F_0	BY940454
	E_FL_e100_27O10_R_0	BY944188
	E_FL_e100_27O11_F_0	BY940455
	E_FL_e100_27O13_F_0	BY940456
	E_FL_e100_27O14_F_0	BY940457
	E_FL_e100_27O15_F_0	BY940458
	E_FL_e100_27O16_F_0	BY940459
	E_FL_e100_27O16_R_0	BY944189
	E_FL_e100_27O17_R_0	BY944190
	E_FL_e100_27O20_F_0	BY940460
	E_FL_e100_27O23_F_0	BY940461
	E_FL_e100_27P01_F_0	BY940462
	E_FL_e100_27P01_R_0	BY944191
	E_FL_e100_27P02_F_0	BY940463
	E_FL_e100_27P02_R_0	BY944192
	E_FL_e100_27P03_F_0	BY940464
	E_FL_e100_27P04_F_0	BY940465
	E_FL_e100_27P05_F_0	BY940466
	E_FL_e100_27P05_R_0	BY944193
	E_FL_e100_27P06_F_0	BY940467
	E_FL_e100_27P06_R_0	BY944194
	E_FL_e100_27P07_F_0	BY940468
	E_FL_e100_27P07_R_0	BY944195
	E_FL_e100_27P13_F_0	BY940469
	E_FL_e100_27P13_R_0	BY944196
	E_FL_e100_27P14_F_0	BY940470
	E_FL_e100_27P14_R_0	BY944197
	E_FL_e100_27P16_F_0	BY940471
	E_FL_e100_27P19_F_0	BY940472
	E_FL_e100_27P19_R_0	BY944198
	E_FL_e100_27P21_F_0	BY940473
	E_FL_e100_27P24_F_0	BY940474
mxg-	E_FL_mxg-_01A02_F_0	BB990114
	E_FL_mxg-_01A05_F_0	BB990115
	E_FL_mxg-_01A06_F_0	BB990116
	E_FL_mxg-_01A09_F_0	BB990117
	E_FL_mxg-_01A10_F_0	BB990118
	E_FL_mxg-_01A12_F_0	BB990119
	E_FL_mxg-_01A13_F_0	BB990120
	E_FL_mxg-_01A16_F_0	BB990121
	E_FL_mxg-_01A18_F_0	BB990122
	E_FL_mxg-_01A23_F_0	BB990123
	E_FL_mxg-_01B08_F_0	BB990124
	E_FL_mxg-_01B10_F_0	BB990125
	E_FL_mxg-_01B12_F_0	BB990126
	E_FL_mxg-_01B13_F_0	BB990127
	E_FL_mxg-_01B17_F_0	BB990128
	E_FL_mxg-_01B18_F_0	BB990129
	E_FL_mxg-_01B20_F_0	BB990130
	E_FL_mxg-_01B21_F_0	BB990131
	E_FL_mxg-_01B22_F_0	BB990132

E_FL_mxg-_01C01_F_0	BB990133
E_FL_mxg-_01C04_F_0	BB990134
E_FL_mxg-_01C07_F_0	BB990135
E_FL_mxg-_01C10_F_0	BB990136
E_FL_mxg-_01C11_F_0	BB990137
E_FL_mxg-_01C12_F_0	BB990138
E_FL_mxg-_01C13_F_0	BB990139
E_FL_mxg-_01C17_F_0	BB990140
E_FL_mxg-_01C19_F_0	BB990141
E_FL_mxg-_01C21_F_0	BB990142
E_FL_mxg-_01C23_F_0	BB990143
E_FL_mxg-_01D02_F_0	BB990144
E_FL_mxg-_01D03_F_0	BB990145
E_FL_mxg-_01D06_F_0	BB990146
E_FL_mxg-_01D07_F_0	BB990147
E_FL_mxg-_01D08_F_0	BB990148
E_FL_mxg-_01D09_F_0	BB990149
E_FL_mxg-_01D15_F_0	BB990150
E_FL_mxg-_01D17_F_0	BB990151
E_FL_mxg-_01D19_F_0	BB990152
E_FL_mxg-_01D20_F_0	BB990153
E_FL_mxg-_01D21_F_0	BB990154
E_FL_mxg-_01D22_F_0	BB990155
E_FL_mxg-_01E05_F_0	BB990156
E_FL_mxg-_01E06_F_0	BB990157
E_FL_mxg-_01E07_F_0	BB990158
E_FL_mxg-_01E10_F_0	BB990159
E_FL_mxg-_01E14_F_0	BB990160
E_FL_mxg-_01E16_F_0	BB990161
E_FL_mxg-_01E20_F_0	BB990162
E_FL_mxg-_01E21_F_0	BB990163
E_FL_mxg-_01E23_F_0	BB990164
E_FL_mxg-_01E24_F_0	BB990165
E_FL_mxg-_01F03_F_0	BB990166
E_FL_mxg-_01F04_F_0	BB990167
E_FL_mxg-_01F07_F_0	BB990168
E_FL_mxg-_01F08_F_0	BB990169
E_FL_mxg-_01F09_F_0	BB990170
E_FL_mxg-_01F10_F_0	BB990171
E_FL_mxg-_01F11_F_0	BB990172
E_FL_mxg-_01F13_F_0	BB990173
E_FL_mxg-_01F15_F_0	BB990174
E_FL_mxg-_01F17_F_0	BB990175
E_FL_mxg-_01F18_F_0	BB990176
E_FL_mxg-_01F20_F_0	BB990177
E_FL_mxg-_01F23_F_0	BB990178
E_FL_mxg-_01G01_F_0	BB990179
E_FL_mxg-_01G02_F_0	BB990180
E_FL_mxg-_01G05_F_0	BB990181
E_FL_mxg-_01G08_F_0	BB990182
E_FL_mxg-_01G09_F_0	BB990183
E_FL_mxg-_01G11_F_0	BB990184
E_FL_mxg-_01G21_F_0	BB990185
E_FL_mxg-_01G22_F_0	BB990186

E_FL_mxg-_01G24_F_0	BB990187
E_FL_mxg-_01H07_F_0	BB990188
E_FL_mxg-_01H08_F_0	BB990189
E_FL_mxg-_01H09_F_0	BB990190
E_FL_mxg-_01H13_F_0	BB990191
E_FL_mxg-_01H18_F_0	BB990192
E_FL_mxg-_01H19_F_0	BB990193
E_FL_mxg-_01H20_F_0	BB990194
E_FL_mxg-_01H23_F_0	BB990195
E_FL_mxg-_01H24_F_0	BB990196
E_FL_mxg-_01I02_F_0	BB990197
E_FL_mxg-_01I04_F_0	BB990198
E_FL_mxg-_01I05_F_0	BB990199
E_FL_mxg-_01I07_F_0	BB990200
E_FL_mxg-_01I08_F_0	BB990201
E_FL_mxg-_01I11_F_0	BB990202
E_FL_mxg-_01I13_F_0	BB990203
E_FL_mxg-_01I14_F_0	BB990204
E_FL_mxg-_01I15_F_0	BB990205
E_FL_mxg-_01I17_F_0	BB990206
E_FL_mxg-_01I21_F_0	BB990207
E_FL_mxg-_01I23_F_0	BB990208
E_FL_mxg-_01J01_F_0	BB990209
E_FL_mxg-_01J02_F_0	BB990210
E_FL_mxg-_01J06_F_0	BB990211
E_FL_mxg-_01J10_F_0	BB990212
E_FL_mxg-_01J11_F_0	BB990213
E_FL_mxg-_01J16_F_0	BB990214
E_FL_mxg-_01J17_F_0	BB990215
E_FL_mxg-_01J22_F_0	BB990216
E_FL_mxg-_01J24_F_0	BB990217
E_FL_mxg-_01K04_F_0	BB990218
E_FL_mxg-_01K06_F_0	BB990219
E_FL_mxg-_01K08_F_0	BB990220
E_FL_mxg-_01K09_F_0	BB990221
E_FL_mxg-_01K11_F_0	BB990222
E_FL_mxg-_01K13_F_0	BB990223
E_FL_mxg-_01K14_F_0	BB990224
E_FL_mxg-_01K15_F_0	BB990225
E_FL_mxg-_01K17_F_0	BB990226
E_FL_mxg-_01K18_F_0	BB990227
E_FL_mxg-_01K21_F_0	BB990228
E_FL_mxg-_01K22_F_0	BB990229
E_FL_mxg-_01L02_F_0	BB990230
E_FL_mxg-_01L06_F_0	BB990231
E_FL_mxg-_01L10_F_0	BB990232
E_FL_mxg-_01L12_F_0	BB990233
E_FL_mxg-_01L14_F_0	BB990234
E_FL_mxg-_01L15_F_0	BB990235
E_FL_mxg-_01L16_F_0	BB990236
E_FL_mxg-_01L21_F_0	BB990237
E_FL_mxg-_01L22_F_0	BB990238
E_FL_mxg-_01L24_F_0	BB990239
E_FL_mxg-_01M01_F_0	BB990240

E_FL_mxg-_01M03_F_0	BB990241
E_FL_mxg-_01M05_F_0	BB990242
E_FL_mxg-_01M07_F_0	BB990243
E_FL_mxg-_01M08_F_0	BB990244
E_FL_mxg-_01M09_F_0	BB990245
E_FL_mxg-_01M10_F_0	BB990246
E_FL_mxg-_01M19_F_0	BB990247
E_FL_mxg-_01M21_F_0	BB990248
E_FL_mxg-_01M23_F_0	BB990249
E_FL_mxg-_01N04_F_0	BB990250
E_FL_mxg-_01N05_F_0	BB990251
E_FL_mxg-_01N06_F_0	BB990252
E_FL_mxg-_01N08_F_0	BB990253
E_FL_mxg-_01N10_F_0	BB990254
E_FL_mxg-_01N11_F_0	BB990255
E_FL_mxg-_01N12_F_0	BB990256
E_FL_mxg-_01N15_F_0	BB990257
E_FL_mxg-_01N16_F_0	BB990258
E_FL_mxg-_01N17_F_0	BB990259
E_FL_mxg-_01N18_F_0	BB990260
E_FL_mxg-_01N24_F_0	BB990261
E_FL_mxg-_01O04_F_0	BB990262
E_FL_mxg-_01O06_F_0	BB990263
E_FL_mxg-_01O09_F_0	BB990264
E_FL_mxg-_01O10_F_0	BB990265
E_FL_mxg-_01O12_F_0	BB990266
E_FL_mxg-_01O13_F_0	BB990267
E_FL_mxg-_01O14_F_0	BB990268
E_FL_mxg-_01O15_F_0	BB990269
E_FL_mxg-_01O16_F_0	BB990270
E_FL_mxg-_01O17_F_0	BB990271
E_FL_mxg-_01O19_F_0	BB990272
E_FL_mxg-_01O21_F_0	BB990273
E_FL_mxg-_01O22_F_0	BB990274
E_FL_mxg-_01O23_F_0	BB990275
E_FL_mxg-_01O24_F_0	BB990276
E_FL_mxg-_01P02_F_0	BB990277
E_FL_mxg-_01P04_F_0	BB990278
E_FL_mxg-_01P05_F_0	BB990279
E_FL_mxg-_01P08_F_0	BB990280
E_FL_mxg-_01P10_F_0	BB990281
E_FL_mxg-_01P12_F_0	BB990282
E_FL_mxg-_01P13_F_0	BB990283
E_FL_mxg-_01P15_F_0	BB990284
E_FL_mxg-_01P16_F_0	BB990285
E_FL_mxg-_01P17_F_0	BB990286
E_FL_mxg-_01P18_F_0	BB990287
E_FL_mxg-_01P19_F_0	BB990288
E_FL_mxg-_01P20_F_0	BB990289
E_FL_mxg-_01P21_F_0	BB990290
E_FL_mxg-_02A01_F_0	BB990291
E_FL_mxg-_02A02_F_0	BB990292
E_FL_mxg-_02A03_F_0	BB990293
E_FL_mxg-_02A06_F_0	BB990294

E_FL_mxg-_02A07_F_0	BB990295
E_FL_mxg-_02A08_F_0	BB990296
E_FL_mxg-_02A10_F_0	BB990297
E_FL_mxg-_02A11_F_0	BB990298
E_FL_mxg-_02A12_F_0	BB990299
E_FL_mxg-_02A13_F_0	BB990300
E_FL_mxg-_02A18_F_0	BB990301
E_FL_mxg-_02A21_F_0	BB990302
E_FL_mxg-_02A22_F_0	BB990303
E_FL_mxg-_02B03_F_0	BB990304
E_FL_mxg-_02B05_F_0	BB990305
E_FL_mxg-_02B06_F_0	BB990306
E_FL_mxg-_02B10_F_0	BB990307
E_FL_mxg-_02B11_F_0	BB990308
E_FL_mxg-_02B12_F_0	BB990309
E_FL_mxg-_02B15_F_0	BB990310
E_FL_mxg-_02B20_F_0	BB990311
E_FL_mxg-_02B21_F_0	BB990312
E_FL_mxg-_02B24_F_0	BB990313
E_FL_mxg-_02C04_F_0	BB990314
E_FL_mxg-_02C05_F_0	BB990315
E_FL_mxg-_02C06_F_0	BB990316
E_FL_mxg-_02C08_F_0	BB990317
E_FL_mxg-_02C10_F_0	BB990318
E_FL_mxg-_02C16_F_0	BB990319
E_FL_mxg-_02C18_F_0	BB990320
E_FL_mxg-_02C20_F_0	BB990321
E_FL_mxg-_02C21_F_0	BB990322
E_FL_mxg-_02C22_F_0	BB990323
E_FL_mxg-_02D01_F_0	BB990324
E_FL_mxg-_02D09_F_0	BB990325
E_FL_mxg-_02D11_F_0	BB990326
E_FL_mxg-_02D12_F_0	BB990327
E_FL_mxg-_02D15_F_0	BB990328
E_FL_mxg-_02D21_F_0	BB990329
E_FL_mxg-_02D24_F_0	BB990330
E_FL_mxg-_02E01_F_0	BB990331
E_FL_mxg-_02E03_F_0	BB990332
E_FL_mxg-_02E04_F_0	BB990333
E_FL_mxg-_02E05_F_0	BB990334
E_FL_mxg-_02E08_F_0	BB990335
E_FL_mxg-_02E10_F_0	BB990336
E_FL_mxg-_02E15_F_0	BB990337
E_FL_mxg-_02E18_F_0	BB990338
E_FL_mxg-_02E21_F_0	BB990339
E_FL_mxg-_02E23_F_0	BB990340
E_FL_mxg-_02F01_F_0	BB990341
E_FL_mxg-_02F02_F_0	BB990342
E_FL_mxg-_02F07_F_0	BB990343
E_FL_mxg-_02F08_F_0	BB990344
E_FL_mxg-_02F12_F_0	BB990345
E_FL_mxg-_02F13_F_0	BB990346
E_FL_mxg-_02F14_F_0	BB990347
E_FL_mxg-_02F19_F_0	BB990348

E_FL_mxg-_02F21_F_0	BB990349
E_FL_mxg-_02F23_F_0	BB990350
E_FL_mxg-_02G04_F_0	BB990351
E_FL_mxg-_02G05_F_0	BB990352
E_FL_mxg-_02G11_F_0	BB990353
E_FL_mxg-_02G12_F_0	BB990354
E_FL_mxg-_02G16_F_0	BB990355
E_FL_mxg-_02G17_F_0	BB990356
E_FL_mxg-_02G18_F_0	BB990357
E_FL_mxg-_02G20_F_0	BB990358
E_FL_mxg-_02H02_F_0	BB990359
E_FL_mxg-_02H04_F_0	BB990360
E_FL_mxg-_02H09_F_0	BB990361
E_FL_mxg-_02H10_F_0	BB990362
E_FL_mxg-_02H12_F_0	BB990363
E_FL_mxg-_02H13_F_0	BB990364
E_FL_mxg-_02H15_F_0	BB990365
E_FL_mxg-_02H16_F_0	BB990366
E_FL_mxg-_02H18_F_0	BB990367
E_FL_mxg-_02I01_F_0	BB990368
E_FL_mxg-_02I03_F_0	BB990369
E_FL_mxg-_02I05_F_0	BB990370
E_FL_mxg-_02I09_F_0	BB990371
E_FL_mxg-_02I10_F_0	BB990372
E_FL_mxg-_02I12_F_0	BB990373
E_FL_mxg-_02I14_F_0	BB990374
E_FL_mxg-_02I15_F_0	BB990375
E_FL_mxg-_02I17_F_0	BB990376
E_FL_mxg-_02I20_F_0	BB990377
E_FL_mxg-_02I21_F_0	BB990378
E_FL_mxg-_02I22_F_0	BB990379
E_FL_mxg-_02J05_F_0	BB990380
E_FL_mxg-_02J06_F_0	BB990381
E_FL_mxg-_02J09_F_0	BB990382
E_FL_mxg-_02J11_F_0	BB990383
E_FL_mxg-_02J13_F_0	BB990384
E_FL_mxg-_02J17_F_0	BB990385
E_FL_mxg-_02J19_F_0	BB990386
E_FL_mxg-_02J20_F_0	BB990387
E_FL_mxg-_02J21_F_0	BB990388
E_FL_mxg-_02J22_F_0	BB990389
E_FL_mxg-_02J24_F_0	BB990390
E_FL_mxg-_02K01_F_0	BB990391
E_FL_mxg-_02K03_F_0	BB990392
E_FL_mxg-_02K04_F_0	BB990393
E_FL_mxg-_02K07_F_0	BB990394
E_FL_mxg-_02K09_F_0	BB990395
E_FL_mxg-_02K10_F_0	BB990396
E_FL_mxg-_02K11_F_0	BB990397
E_FL_mxg-_02K12_F_0	BB990398
E_FL_mxg-_02K13_F_0	BB990399
E_FL_mxg-_02K15_F_0	BB990400
E_FL_mxg-_02K18_F_0	BB990401
E_FL_mxg-_02K19_F_0	BB990402

E_FL_mxg-_02K20_F_0	BB990403
E_FL_mxg-_02L01_F_0	BB990404
E_FL_mxg-_02L03_F_0	BB990405
E_FL_mxg-_02L07_F_0	BB990406
E_FL_mxg-_02L09_F_0	BB990407
E_FL_mxg-_02L13_F_0	BB990408
E_FL_mxg-_02L15_F_0	BB990409
E_FL_mxg-_02L17_F_0	BB990410
E_FL_mxg-_02L20_F_0	BB990411
E_FL_mxg-_02L21_F_0	BB990412
E_FL_mxg-_02L23_F_0	BB990413
E_FL_mxg-_02M02_F_0	BB990414
E_FL_mxg-_02M05_F_0	BB990415
E_FL_mxg-_02M07_F_0	BB990416
E_FL_mxg-_02M08_F_0	BB990417
E_FL_mxg-_02M12_F_0	BB990418
E_FL_mxg-_02M13_F_0	BB990419
E_FL_mxg-_02M15_F_0	BB990420
E_FL_mxg-_02M16_F_0	BB990421
E_FL_mxg-_02M19_F_0	BB990422
E_FL_mxg-_02N01_F_0	BB990423
E_FL_mxg-_02N02_F_0	BB990424
E_FL_mxg-_02N06_F_0	BB990425
E_FL_mxg-_02N07_F_0	BB990426
E_FL_mxg-_02N09_F_0	BB990427
E_FL_mxg-_02N10_F_0	BB990428
E_FL_mxg-_02N11_F_0	BB990429
E_FL_mxg-_02N12_F_0	BB990430
E_FL_mxg-_02N15_F_0	BB990431
E_FL_mxg-_02N17_F_0	BB990432
E_FL_mxg-_02N23_F_0	BB990433
E_FL_mxg-_02N24_F_0	BB990434
E_FL_mxg-_02O02_F_0	BB990435
E_FL_mxg-_02O04_F_0	BB990436
E_FL_mxg-_02O09_F_0	BB990437
E_FL_mxg-_02O10_F_0	BB990438
E_FL_mxg-_02O12_F_0	BB990439
E_FL_mxg-_02O13_F_0	BB990440
E_FL_mxg-_02O14_F_0	BB990441
E_FL_mxg-_02O16_F_0	BB990442
E_FL_mxg-_02O18_F_0	BB990443
E_FL_mxg-_02O20_F_0	BB990444
E_FL_mxg-_02O22_F_0	BB990445
E_FL_mxg-_02O23_F_0	BB990446
E_FL_mxg-_02O24_F_0	BB990447
E_FL_mxg-_02P03_F_0	BB990448
E_FL_mxg-_02P04_F_0	BB990449
E_FL_mxg-_02P06_F_0	BB990450
E_FL_mxg-_02P09_F_0	BB990451
E_FL_mxg-_02P10_F_0	BB990452
E_FL_mxg-_02P15_F_0	BB990453
E_FL_mxg-_02P16_F_0	BB990454
E_FL_mxg-_02P18_F_0	BB990455
E_FL_mxg-_02P20_F_0	BB990456

E_FL_mxg-_02P22_F_0	BB990457
E_FL_mxg-_02P23_F_0	BB990458
E_FL_mxg-_03A03_F_0	BB990459
E_FL_mxg-_03A04_F_0	BB990460
E_FL_mxg-_03A06_F_0	BB990461
E_FL_mxg-_03A10_F_0	BB990462
E_FL_mxg-_03A12_F_0	BB990463
E_FL_mxg-_03A13_F_0	BB990464
E_FL_mxg-_03A15_F_0	BB990465
E_FL_mxg-_03A16_F_0	BB990466
E_FL_mxg-_03A20_F_0	BB990467
E_FL_mxg-_03A22_F_0	BB990468
E_FL_mxg-_03B02_F_0	BB990469
E_FL_mxg-_03B04_F_0	BB990470
E_FL_mxg-_03B08_F_0	BB990471
E_FL_mxg-_03B09_F_0	BB990472
E_FL_mxg-_03B12_F_0	BB990473
E_FL_mxg-_03B14_F_0	BB990474
E_FL_mxg-_03B15_F_0	BB990475
E_FL_mxg-_03B18_F_0	BB990476
E_FL_mxg-_03B20_F_0	BB990477
E_FL_mxg-_03C02_F_0	BB990478
E_FL_mxg-_03C07_F_0	BB990479
E_FL_mxg-_03C09_F_0	BB990480
E_FL_mxg-_03C10_F_0	BB990481
E_FL_mxg-_03C12_F_0	BB990482
E_FL_mxg-_03C13_F_0	BB990483
E_FL_mxg-_03C14_F_0	BB990484
E_FL_mxg-_03C20_F_0	BB990485
E_FL_mxg-_03C21_F_0	BB990486
E_FL_mxg-_03C22_F_0	BB990487
E_FL_mxg-_03C24_F_0	BB990488
E_FL_mxg-_03D01_F_0	BB990489
E_FL_mxg-_03D02_F_0	BB990490
E_FL_mxg-_03D04_F_0	BB990491
E_FL_mxg-_03D05_F_0	BB990492
E_FL_mxg-_03D09_F_0	BB990493
E_FL_mxg-_03D12_F_0	BB990494
E_FL_mxg-_03D14_F_0	BB990495
E_FL_mxg-_03D15_F_0	BB990496
E_FL_mxg-_03D17_F_0	BB990497
E_FL_mxg-_03D18_F_0	BB990498
E_FL_mxg-_03D19_F_0	BB990499
E_FL_mxg-_03D22_F_0	BB990500
E_FL_mxg-_03E01_F_0	BB990501
E_FL_mxg-_03E02_F_0	BB990502
E_FL_mxg-_03E04_F_0	BB990503
E_FL_mxg-_03E05_F_0	BB990504
E_FL_mxg-_03E07_F_0	BB990505
E_FL_mxg-_03E09_F_0	BB990506
E_FL_mxg-_03E10_F_0	BB990507
E_FL_mxg-_03E12_F_0	BB990508
E_FL_mxg-_03E13_F_0	BB990509
E_FL_mxg-_03E15_F_0	BB990510

E_FL_mxg-_03E19_F_0	BB990511
E_FL_mxg-_03E22_F_0	BB990512
E_FL_mxg-_03E23_F_0	BB990513
E_FL_mxg-_03F01_F_0	BB990514
E_FL_mxg-_03F03_F_0	BB990515
E_FL_mxg-_03F04_F_0	BB990516
E_FL_mxg-_03F05_F_0	BB990517
E_FL_mxg-_03F12_F_0	BB990518
E_FL_mxg-_03F14_F_0	BB990519
E_FL_mxg-_03F17_F_0	BB990520
E_FL_mxg-_03F19_F_0	BB990521
E_FL_mxg-_03F20_F_0	BB990522
E_FL_mxg-_03F22_F_0	BB990523
E_FL_mxg-_03G01_F_0	BB990524
E_FL_mxg-_03G02_F_0	BB990525
E_FL_mxg-_03G05_F_0	BB990526
E_FL_mxg-_03G08_F_0	BB990527
E_FL_mxg-_03G13_F_0	BB990528
E_FL_mxg-_03G14_F_0	BB990529
E_FL_mxg-_03G16_F_0	BB990530
E_FL_mxg-_03G17_F_0	BB990531
E_FL_mxg-_03G18_F_0	BB990532
E_FL_mxg-_03G19_F_0	BB990533
E_FL_mxg-_03G22_F_0	BB990534
E_FL_mxg-_03G23_F_0	BB990535
E_FL_mxg-_03G24_F_0	BB990536
E_FL_mxg-_03H02_F_0	BB990537
E_FL_mxg-_03H05_F_0	BB990538
E_FL_mxg-_03H09_F_0	BB990539
E_FL_mxg-_03H10_F_0	BB990540
E_FL_mxg-_03H14_F_0	BB990541
E_FL_mxg-_03H16_F_0	BB990542
E_FL_mxg-_03H19_F_0	BB990543
E_FL_mxg-_03H20_F_0	BB990544
E_FL_mxg-_03H22_F_0	BB990545
E_FL_mxg-_03H23_F_0	BB990546
E_FL_mxg-_03H24_F_0	BB990547
E_FL_mxg-_03I01_F_0	BB990548
E_FL_mxg-_03I04_F_0	BB990549
E_FL_mxg-_03I07_F_0	BB990550
E_FL_mxg-_03I12_F_0	BB990551
E_FL_mxg-_03I15_F_0	BB990552
E_FL_mxg-_03I17_F_0	BB990553
E_FL_mxg-_03I20_F_0	BB990554
E_FL_mxg-_03I21_F_0	BB990555
E_FL_mxg-_03I22_F_0	BB990556
E_FL_mxg-_03I23_F_0	BB990557
E_FL_mxg-_03I24_F_0	BB990558
E_FL_mxg-_03J01_F_0	BB990559
E_FL_mxg-_03J02_F_0	BB990560
E_FL_mxg-_03J03_F_0	BB990561
E_FL_mxg-_03J04_F_0	BB990562
E_FL_mxg-_03J07_F_0	BB990563
E_FL_mxg-_03J09_F_0	BB990564

E_FL_mxg-_03J12_F_0	BB990565
E_FL_mxg-_03J14_F_0	BB990566
E_FL_mxg-_03J15_F_0	BB990567
E_FL_mxg-_03J17_F_0	BB990568
E_FL_mxg-_03J21_F_0	BB990569
E_FL_mxg-_03K01_F_0	BB990570
E_FL_mxg-_03K02_F_0	BB990571
E_FL_mxg-_03K05_F_0	BB990572
E_FL_mxg-_03K06_F_0	BB990573
E_FL_mxg-_03K07_F_0	BB990574
E_FL_mxg-_03K12_F_0	BB990575
E_FL_mxg-_03K15_F_0	BB990576
E_FL_mxg-_03K16_F_0	BB990577
E_FL_mxg-_03K17_F_0	BB990578
E_FL_mxg-_03L04_F_0	BB990579
E_FL_mxg-_03L05_F_0	BB990580
E_FL_mxg-_03L08_F_0	BB990581
E_FL_mxg-_03L09_F_0	BB990582
E_FL_mxg-_03L10_F_0	BB990583
E_FL_mxg-_03L11_F_0	BB990584
E_FL_mxg-_03L12_F_0	BB990585
E_FL_mxg-_03L14_F_0	BB990586
E_FL_mxg-_03L15_F_0	BB990587
E_FL_mxg-_03L16_F_0	BB990588
E_FL_mxg-_03L17_F_0	BB990589
E_FL_mxg-_03L18_F_0	BB990590
E_FL_mxg-_03L20_F_0	BB990591
E_FL_mxg-_03L24_F_0	BB990592
E_FL_mxg-_03M04_F_0	BB990593
E_FL_mxg-_03M06_F_0	BB990594
E_FL_mxg-_03M10_F_0	BB990595
E_FL_mxg-_03M11_F_0	BB990596
E_FL_mxg-_03M14_F_0	BB990597
E_FL_mxg-_03M16_F_0	BB990598
E_FL_mxg-_03M18_F_0	BB990599
E_FL_mxg-_03M23_F_0	BB990600
E_FL_mxg-_03N04_F_0	BB990601
E_FL_mxg-_03N06_F_0	BB990602
E_FL_mxg-_03N08_F_0	BB990603
E_FL_mxg-_03N11_F_0	BB990604
E_FL_mxg-_03N12_F_0	BB990605
E_FL_mxg-_03N14_F_0	BB990606
E_FL_mxg-_03N18_F_0	BB990607
E_FL_mxg-_03N20_F_0	BB990608
E_FL_mxg-_03N21_F_0	BB990609
E_FL_mxg-_03N22_F_0	BB990610
E_FL_mxg-_03O02_F_0	BB990611
E_FL_mxg-_03O03_F_0	BB990612
E_FL_mxg-_03O04_F_0	BB990613
E_FL_mxg-_03O05_F_0	BB990614
E_FL_mxg-_03O06_F_0	BB990615
E_FL_mxg-_03O08_F_0	BB990616
E_FL_mxg-_03O09_F_0	BB990617
E_FL_mxg-_03O10_F_0	BB990618

E_FL_mxg-_03O14_F_0	BB990619
E_FL_mxg-_03O16_F_0	BB990620
E_FL_mxg-_03O17_F_0	BB990621
E_FL_mxg-_03O18_F_0	BB990622
E_FL_mxg-_03O19_F_0	BB990623
E_FL_mxg-_03O20_F_0	BB990624
E_FL_mxg-_03O21_F_0	BB990625
E_FL_mxg-_03O22_F_0	BB990626
E_FL_mxg-_03O23_F_0	BB990627
E_FL_mxg-_03O24_F_0	BB990628
E_FL_mxg-_03P02_F_0	BB990629
E_FL_mxg-_03P04_F_0	BB990630
E_FL_mxg-_03P09_F_0	BB990631
E_FL_mxg-_03P14_F_0	BB990632
E_FL_mxg-_03P15_F_0	BB990633
E_FL_mxg-_03P16_F_0	BB990634
E_FL_mxg-_03P19_F_0	BB990635
E_FL_mxg-_03P20_F_0	BB990636
E_FL_mxg-_03P21_F_0	BB990637
E_FL_mxg-_04A03_F_0	BB990638
E_FL_mxg-_04A05_F_0	BB990639
E_FL_mxg-_04A06_F_0	BB990640
E_FL_mxg-_04A09_F_0	BB990641
E_FL_mxg-_04A11_F_0	BB990642
E_FL_mxg-_04A14_F_0	BB990643
E_FL_mxg-_04A16_F_0	BB990644
E_FL_mxg-_04A17_F_0	BB990645
E_FL_mxg-_04A20_F_0	BB990646
E_FL_mxg-_04B08_F_0	BB990647
E_FL_mxg-_04B13_F_0	BB990648
E_FL_mxg-_04B16_F_0	BB990649
E_FL_mxg-_04B17_F_0	BB990650
E_FL_mxg-_04B21_F_0	BB990651
E_FL_mxg-_04B22_F_0	BB990652
E_FL_mxg-_04B24_F_0	BB990653
E_FL_mxg-_04C02_F_0	BB990654
E_FL_mxg-_04C03_F_0	BB990655
E_FL_mxg-_04C06_F_0	BB990656
E_FL_mxg-_04C07_F_0	BB990657
E_FL_mxg-_04C08_F_0	BB990658
E_FL_mxg-_04C09_F_0	BB990659
E_FL_mxg-_04C10_F_0	BB990660
E_FL_mxg-_04C11_F_0	BB990661
E_FL_mxg-_04C12_F_0	BB990662
E_FL_mxg-_04C13_F_0	BB990663
E_FL_mxg-_04C17_F_0	BB990664
E_FL_mxg-_04C20_F_0	BB990665
E_FL_mxg-_04C24_F_0	BB990666
E_FL_mxg-_04D01_F_0	BB990667
E_FL_mxg-_04D09_F_0	BB990668
E_FL_mxg-_04D11_F_0	BB990669
E_FL_mxg-_04D12_F_0	BB990670
E_FL_mxg-_04D13_F_0	BB990671
E_FL_mxg-_04D14_F_0	BB990672

E_FL_mxg-_04D15_F_0	BB990673
E_FL_mxg-_04D20_F_0	BB990674
E_FL_mxg-_04D22_F_0	BB990675
E_FL_mxg-_04D24_F_0	BB990676
E_FL_mxg-_04E01_F_0	BB990677
E_FL_mxg-_04E04_F_0	BB990678
E_FL_mxg-_04E06_F_0	BB990679
E_FL_mxg-_04E07_F_0	BB990680
E_FL_mxg-_04E08_F_0	BB990681
E_FL_mxg-_04E09_F_0	BB990682
E_FL_mxg-_04E10_F_0	BB990683
E_FL_mxg-_04E11_F_0	BB990684
E_FL_mxg-_04E12_F_0	BB990685
E_FL_mxg-_04E18_F_0	BB990686
E_FL_mxg-_04E19_F_0	BB990687
E_FL_mxg-_04E20_F_0	BJ983130
E_FL_mxg-_04E21_F_0	BJ983131
E_FL_mxg-_04E22_F_0	BJ983132
E_FL_mxg-_04F02_F_0	BJ983133
E_FL_mxg-_04F03_F_0	BJ983134
E_FL_mxg-_04F05_F_0	BJ983135
E_FL_mxg-_04F07_F_0	BJ983136
E_FL_mxg-_04F11_F_0	BJ983137
E_FL_mxg-_04F17_F_0	BJ983138
E_FL_mxg-_04F19_F_0	BJ983139
E_FL_mxg-_04F20_F_0	BJ983140
E_FL_mxg-_04F21_F_0	BJ983141
E_FL_mxg-_04F22_F_0	BJ983142
E_FL_mxg-_04G03_F_0	BJ983143
E_FL_mxg-_04G05_F_0	BJ983144
E_FL_mxg-_04G07_F_0	BJ983145
E_FL_mxg-_04G11_F_0	BJ983146
E_FL_mxg-_04G13_F_0	BJ983147
E_FL_mxg-_04G14_F_0	BJ983148
E_FL_mxg-_04G15_F_0	BJ983149
E_FL_mxg-_04G16_F_0	BJ983150
E_FL_mxg-_04G17_F_0	BJ983151
E_FL_mxg-_04G19_F_0	BJ983152
E_FL_mxg-_04G20_F_0	BJ983153
E_FL_mxg-_04G23_F_0	BJ983154
E_FL_mxg-_04H02_F_0	BJ983155
E_FL_mxg-_04H05_F_0	BJ983156
E_FL_mxg-_04H07_F_0	BJ983157
E_FL_mxg-_04H11_F_0	BJ983158
E_FL_mxg-_04H12_F_0	BJ983159
E_FL_mxg-_04H13_F_0	BJ983160
E_FL_mxg-_04H14_F_0	BJ983161
E_FL_mxg-_04H15_F_0	BJ983162
E_FL_mxg-_04H16_F_0	BJ983163
E_FL_mxg-_04H17_F_0	BJ983164
E_FL_mxg-_04H20_F_0	BJ983165
E_FL_mxg-_04H21_F_0	BJ983166
E_FL_mxg-_04I03_F_0	BJ983167
E_FL_mxg-_04I05_F_0	BJ983168

E_FL_mxg-_04I06_F_0	BJ983169
E_FL_mxg-_04I08_F_0	BJ983170
E_FL_mxg-_04I09_F_0	BJ983171
E_FL_mxg-_04I11_F_0	BJ983172
E_FL_mxg-_04I13_F_0	BJ983173
E_FL_mxg-_04I14_F_0	BJ983174
E_FL_mxg-_04I19_F_0	BJ983175
E_FL_mxg-_04I20_F_0	BJ983176
E_FL_mxg-_04I22_F_0	BJ983177
E_FL_mxg-_04I23_F_0	BJ983178
E_FL_mxg-_04I24_F_0	BJ983179
E_FL_mxg-_04J02_F_0	BJ983180
E_FL_mxg-_04J05_F_0	BJ983181
E_FL_mxg-_04J12_F_0	BJ983182
E_FL_mxg-_04J13_F_0	BJ983183
E_FL_mxg-_04J14_F_0	BJ983184
E_FL_mxg-_04J16_F_0	BJ983185
E_FL_mxg-_04J19_F_0	BJ983186
E_FL_mxg-_04J20_F_0	BJ983187
E_FL_mxg-_04J24_F_0	BJ983188
E_FL_mxg-_04K07_F_0	BJ983189
E_FL_mxg-_04K12_F_0	BJ983190
E_FL_mxg-_04K15_F_0	BJ983191
E_FL_mxg-_04K18_F_0	BJ983192
E_FL_mxg-_04K20_F_0	BJ983193
E_FL_mxg-_04K22_F_0	BJ983194
E_FL_mxg-_04L05_F_0	BJ983195
E_FL_mxg-_04L06_F_0	BJ983196
E_FL_mxg-_04L07_F_0	BJ983197
E_FL_mxg-_04L16_F_0	BJ983198
E_FL_mxg-_04L17_F_0	BJ983199
E_FL_mxg-_04L20_F_0	BJ983200
E_FL_mxg-_04L21_F_0	BJ983201
E_FL_mxg-_04L22_F_0	BJ983202
E_FL_mxg-_04L23_F_0	BJ983203
E_FL_mxg-_04L24_F_0	BJ983204
E_FL_mxg-_04M01_F_0	BJ983205
E_FL_mxg-_04M03_F_0	BJ983206
E_FL_mxg-_04M05_F_0	BJ983207
E_FL_mxg-_04M08_F_0	BJ983208
E_FL_mxg-_04M12_F_0	BJ983209
E_FL_mxg-_04M13_F_0	BJ983210
E_FL_mxg-_04M14_F_0	BJ983211
E_FL_mxg-_04M16_F_0	BJ983212
E_FL_mxg-_04M18_F_0	BJ983213
E_FL_mxg-_04M19_F_0	BJ983214
E_FL_mxg-_04M21_F_0	BJ983215
E_FL_mxg-_04M23_F_0	BJ983216
E_FL_mxg-_04N03_F_0	BJ983217
E_FL_mxg-_04N10_F_0	BJ983218
E_FL_mxg-_04N12_F_0	BJ983219
E_FL_mxg-_04N14_F_0	BJ983220
E_FL_mxg-_04N16_F_0	BJ983221
E_FL_mxg-_04N18_F_0	BJ983222

E_FL_mxg-_04N19_F_0	BJ983223
E_FL_mxg-_04N20_F_0	BJ983224
E_FL_mxg-_04N24_F_0	BJ983225
E_FL_mxg-_04O03_F_0	BJ983226
E_FL_mxg-_04O09_F_0	BJ983227
E_FL_mxg-_04O11_F_0	BJ983228
E_FL_mxg-_04O12_F_0	BJ983229
E_FL_mxg-_04O13_F_0	BJ983230
E_FL_mxg-_04O20_F_0	BJ983231
E_FL_mxg-_04O21_F_0	BJ983232
E_FL_mxg-_04O22_F_0	BJ983233
E_FL_mxg-_04O24_F_0	BJ983234
E_FL_mxg-_04P02_F_0	BJ983235
E_FL_mxg-_04P06_F_0	BJ983236
E_FL_mxg-_04P21_F_0	BJ983237
E_FL_mxg-_05A02_F_0	BJ983238
E_FL_mxg-_05A03_F_0	BJ983239
E_FL_mxg-_05A06_F_0	BJ983240
E_FL_mxg-_05A08_F_0	BJ983241
E_FL_mxg-_05A12_F_0	BJ983242
E_FL_mxg-_05A15_F_0	BJ983243
E_FL_mxg-_05A18_F_0	BJ983244
E_FL_mxg-_05A19_F_0	BJ983245
E_FL_mxg-_05A22_F_0	BJ983246
E_FL_mxg-_05A23_F_0	BJ983247
E_FL_mxg-_05B04_F_0	BJ983248
E_FL_mxg-_05B07_F_0	BJ983249
E_FL_mxg-_05B08_F_0	BJ983250
E_FL_mxg-_05B09_F_0	BJ983251
E_FL_mxg-_05B10_F_0	BJ983252
E_FL_mxg-_05B12_F_0	BJ983253
E_FL_mxg-_05B13_F_0	BJ983254
E_FL_mxg-_05B16_F_0	BJ983255
E_FL_mxg-_05B17_F_0	BJ983256
E_FL_mxg-_05B18_F_0	BJ983257
E_FL_mxg-_05B21_F_0	BJ983258
E_FL_mxg-_05C01_F_0	BJ983259
E_FL_mxg-_05C02_F_0	BJ983260
E_FL_mxg-_05C06_F_0	BJ983261
E_FL_mxg-_05C08_F_0	BJ983262
E_FL_mxg-_05C09_F_0	BJ983263
E_FL_mxg-_05C12_F_0	BJ983264
E_FL_mxg-_05C13_F_0	BJ983265
E_FL_mxg-_05C14_F_0	BJ983266
E_FL_mxg-_05C18_F_0	BJ983267
E_FL_mxg-_05C19_F_0	BJ983268
E_FL_mxg-_05C22_F_0	BJ983269
E_FL_mxg-_05D03_F_0	BJ983270
E_FL_mxg-_05D04_F_0	BJ983271
E_FL_mxg-_05D11_F_0	BJ983272
E_FL_mxg-_05D14_F_0	BJ983273
E_FL_mxg-_05D15_F_0	BJ983274
E_FL_mxg-_05D17_F_0	BJ983275
E_FL_mxg-_05D21_F_0	BJ983276

E_FL_mxg-_05D22_F_0	BJ983277
E_FL_mxg-_05D24_F_0	BJ983278
E_FL_mxg-_05E02_F_0	BJ983279
E_FL_mxg-_05E03_F_0	BJ983280
E_FL_mxg-_05E06_F_0	BJ983281
E_FL_mxg-_05E08_F_0	BJ983282
E_FL_mxg-_05E14_F_0	BJ983283
E_FL_mxg-_05E17_F_0	BJ983284
E_FL_mxg-_05E18_F_0	BJ983285
E_FL_mxg-_05E19_F_0	BJ983286
E_FL_mxg-_05E20_F_0	BJ983287
E_FL_mxg-_05E23_F_0	BJ983288
E_FL_mxg-_05E24_F_0	BJ983289
E_FL_mxg-_05F05_F_0	BJ983290
E_FL_mxg-_05F07_F_0	BJ983291
E_FL_mxg-_05F08_F_0	BJ983292
E_FL_mxg-_05F10_F_0	BJ983293
E_FL_mxg-_05F11_F_0	BJ983294
E_FL_mxg-_05F13_F_0	BJ983295
E_FL_mxg-_05F16_F_0	BJ983296
E_FL_mxg-_05F20_F_0	BJ983297
E_FL_mxg-_05F21_F_0	BJ983298
E_FL_mxg-_05F22_F_0	BJ983299
E_FL_mxg-_05G01_F_0	BJ983300
E_FL_mxg-_05G03_F_0	BJ983301
E_FL_mxg-_05G06_F_0	BJ983302
E_FL_mxg-_05G11_F_0	BJ983303
E_FL_mxg-_05G13_F_0	BJ983304
E_FL_mxg-_05G14_F_0	BJ983305
E_FL_mxg-_05G15_F_0	BJ983306
E_FL_mxg-_05G17_F_0	BJ983307
E_FL_mxg-_05G20_F_0	BJ983308
E_FL_mxg-_05G22_F_0	BJ983309
E_FL_mxg-_05G23_F_0	BJ983310
E_FL_mxg-_05H03_F_0	BJ983311
E_FL_mxg-_05H06_F_0	BJ983312
E_FL_mxg-_05H07_F_0	BJ983313
E_FL_mxg-_05H08_F_0	BJ983314
E_FL_mxg-_05H14_F_0	BJ983315
E_FL_mxg-_05H17_F_0	BJ983316
E_FL_mxg-_05H20_F_0	BJ983317
E_FL_mxg-_05I07_F_0	BJ983318
E_FL_mxg-_05I09_F_0	BJ983319
E_FL_mxg-_05I11_F_0	BJ983320
E_FL_mxg-_05I12_F_0	BJ983321
E_FL_mxg-_05I13_F_0	BJ983322
E_FL_mxg-_05I15_F_0	BJ983323
E_FL_mxg-_05I18_F_0	BJ983324
E_FL_mxg-_05I19_F_0	BJ983325
E_FL_mxg-_05I20_F_0	BJ983326
E_FL_mxg-_05I21_F_0	BJ983327
E_FL_mxg-_05I22_F_0	BJ983328
E_FL_mxg-_05I23_F_0	BJ983329
E_FL_mxg-_05J02_F_0	BJ983330

E_FL_mxg-_05J04_F_0	BJ983331
E_FL_mxg-_05J05_F_0	BJ983332
E_FL_mxg-_05J06_F_0	BJ983333
E_FL_mxg-_05J07_F_0	BJ983334
E_FL_mxg-_05J08_F_0	BJ983335
E_FL_mxg-_05J12_F_0	BJ983336
E_FL_mxg-_05J13_F_0	BJ983337
E_FL_mxg-_05J14_F_0	BJ983338
E_FL_mxg-_05J20_F_0	BJ983339
E_FL_mxg-_05J24_F_0	BJ983340
E_FL_mxg-_05K04_F_0	BJ983341
E_FL_mxg-_05K06_F_0	BJ983342
E_FL_mxg-_05K07_F_0	BJ983343
E_FL_mxg-_05K10_F_0	BJ983344
E_FL_mxg-_05K15_F_0	BJ983345
E_FL_mxg-_05K18_F_0	BJ983346
E_FL_mxg-_05K22_F_0	BJ983347
E_FL_mxg-_05L01_F_0	BJ983348
E_FL_mxg-_05L02_F_0	BJ983349
E_FL_mxg-_05L03_F_0	BJ983350
E_FL_mxg-_05L05_F_0	BJ983351
E_FL_mxg-_05L06_F_0	BJ983352
E_FL_mxg-_05L07_F_0	BJ983353
E_FL_mxg-_05L09_F_0	BJ983354
E_FL_mxg-_05L10_F_0	BJ983355
E_FL_mxg-_05L11_F_0	BJ983356
E_FL_mxg-_05L14_F_0	BJ983357
E_FL_mxg-_05L15_F_0	BJ983358
E_FL_mxg-_05L19_F_0	BJ983359
E_FL_mxg-_05L20_F_0	BJ983360
E_FL_mxg-_05L23_F_0	BJ983361
E_FL_mxg-_05L24_F_0	BJ983362
E_FL_mxg-_05M03_F_0	BJ983363
E_FL_mxg-_05M04_F_0	BJ983364
E_FL_mxg-_05M05_F_0	BJ983365
E_FL_mxg-_05M06_F_0	BJ983366
E_FL_mxg-_05M07_F_0	BJ983367
E_FL_mxg-_05M15_F_0	BJ983368
E_FL_mxg-_05M20_F_0	BJ983369
E_FL_mxg-_05M24_F_0	BJ983370
E_FL_mxg-_05N03_F_0	BJ983371
E_FL_mxg-_05N04_F_0	BJ983372
E_FL_mxg-_05N05_F_0	BJ983373
E_FL_mxg-_05N12_F_0	BJ983374
E_FL_mxg-_05N13_F_0	BJ983375
E_FL_mxg-_05N15_F_0	BJ983376
E_FL_mxg-_05N16_F_0	BJ983377
E_FL_mxg-_05N18_F_0	BJ983378
E_FL_mxg-_05N22_F_0	BJ983379
E_FL_mxg-_05N24_F_0	BJ983380
E_FL_mxg-_05O02_F_0	BJ983381
E_FL_mxg-_05O05_F_0	BJ983382
E_FL_mxg-_05O06_F_0	BJ983383
E_FL_mxg-_05O07_F_0	BJ983384

E_FL_mxg-_05O09_F_0	BJ983385
E_FL_mxg-_05O15_F_0	BJ983386
E_FL_mxg-_05O16_F_0	BJ983387
E_FL_mxg-_05O17_F_0	BJ983388
E_FL_mxg-_05O19_F_0	BJ983389
E_FL_mxg-_05O20_F_0	BJ983390
E_FL_mxg-_05O21_F_0	BJ983391
E_FL_mxg-_05O22_F_0	BJ983392
E_FL_mxg-_05O23_F_0	BJ983393
E_FL_mxg-_05O24_F_0	BJ983394
E_FL_mxg-_05P01_F_0	BJ983395
E_FL_mxg-_05P03_F_0	BJ983396
E_FL_mxg-_05P04_F_0	BJ983397
E_FL_mxg-_05P08_F_0	BJ983398
E_FL_mxg-_05P12_F_0	BJ983399
E_FL_mxg-_05P16_F_0	BJ983400
E_FL_mxg-_05P24_F_0	BJ983401
E_FL_mxg-_06A02_F_0	BJ983402
E_FL_mxg-_06A05_F_0	BJ983403
E_FL_mxg-_06A07_F_0	BJ983404
E_FL_mxg-_06A08_F_0	BJ983405
E_FL_mxg-_06A10_F_0	BJ983406
E_FL_mxg-_06A11_F_0	BJ983407
E_FL_mxg-_06A15_F_0	BJ983408
E_FL_mxg-_06A16_F_0	BJ983409
E_FL_mxg-_06A19_F_0	BJ983410
E_FL_mxg-_06A20_F_0	BJ983411
E_FL_mxg-_06A22_F_0	BJ983412
E_FL_mxg-_06A23_F_0	BJ983413
E_FL_mxg-_06B02_F_0	BJ983414
E_FL_mxg-_06B04_F_0	BJ983415
E_FL_mxg-_06B05_F_0	BJ983416
E_FL_mxg-_06B06_F_0	BJ983417
E_FL_mxg-_06B08_F_0	BJ983418
E_FL_mxg-_06B10_F_0	BJ983419
E_FL_mxg-_06B14_F_0	BJ983420
E_FL_mxg-_06B16_F_0	BJ983421
E_FL_mxg-_06B19_F_0	BJ983422
E_FL_mxg-_06B24_F_0	BJ983423
E_FL_mxg-_06C01_F_0	BJ983424
E_FL_mxg-_06C02_F_0	BJ983425
E_FL_mxg-_06C03_F_0	BJ983426
E_FL_mxg-_06C05_F_0	BJ983427
E_FL_mxg-_06C08_F_0	BJ983428
E_FL_mxg-_06C09_F_0	BJ983429
E_FL_mxg-_06C11_F_0	BJ983430
E_FL_mxg-_06C12_F_0	BJ983431
E_FL_mxg-_06C17_F_0	BJ983432
E_FL_mxg-_06C20_F_0	BJ983433
E_FL_mxg-_06C24_F_0	BJ983434
E_FL_mxg-_06D03_F_0	BJ983435
E_FL_mxg-_06D04_F_0	BJ983436
E_FL_mxg-_06D05_F_0	BJ983437
E_FL_mxg-_06D06_F_0	BJ983438

E_FL_mxg-_06D08_F_0	BJ983439
E_FL_mxg-_06D11_F_0	BJ983440
E_FL_mxg-_06D14_F_0	BJ983441
E_FL_mxg-_06D15_F_0	BJ983442
E_FL_mxg-_06D17_F_0	BJ983443
E_FL_mxg-_06D18_F_0	BJ983444
E_FL_mxg-_06D19_F_0	BJ983445
E_FL_mxg-_06D21_F_0	BJ983446
E_FL_mxg-_06D22_F_0	BJ983447
E_FL_mxg-_06E05_F_0	BJ983448
E_FL_mxg-_06E06_F_0	BJ983449
E_FL_mxg-_06E07_F_0	BJ983450
E_FL_mxg-_06E08_F_0	BJ983451
E_FL_mxg-_06E11_F_0	BJ983452
E_FL_mxg-_06E14_F_0	BJ983453
E_FL_mxg-_06E15_F_0	BJ983454
E_FL_mxg-_06E16_F_0	BJ983455
E_FL_mxg-_06E17_F_0	BJ983456
E_FL_mxg-_06E21_F_0	BJ983457
E_FL_mxg-_06E22_F_0	BJ983458
E_FL_mxg-_06E24_F_0	BJ983459
E_FL_mxg-_06F03_F_0	BJ983460
E_FL_mxg-_06F04_F_0	BJ983461
E_FL_mxg-_06F07_F_0	BJ983462
E_FL_mxg-_06F09_F_0	BJ983463
E_FL_mxg-_06F12_F_0	BJ983464
E_FL_mxg-_06F14_F_0	BJ983465
E_FL_mxg-_06F15_F_0	BJ983466
E_FL_mxg-_06F16_F_0	BJ983467
E_FL_mxg-_06F17_F_0	BJ983468
E_FL_mxg-_06F18_F_0	BJ983469
E_FL_mxg-_06F20_F_0	BJ983470
E_FL_mxg-_06F21_F_0	BJ983471
E_FL_mxg-_06F23_F_0	BJ983472
E_FL_mxg-_06F24_F_0	BJ983473
E_FL_mxg-_06G02_F_0	BJ983474
E_FL_mxg-_06G05_F_0	BJ983475
E_FL_mxg-_06G07_F_0	BJ983476
E_FL_mxg-_06G08_F_0	BJ983477
E_FL_mxg-_06G09_F_0	BJ983478
E_FL_mxg-_06G16_F_0	BJ983479
E_FL_mxg-_06G17_F_0	BJ983480
E_FL_mxg-_06G20_F_0	BJ983481
E_FL_mxg-_06G22_F_0	BJ983482
E_FL_mxg-_06G23_F_0	BJ983483
E_FL_mxg-_06G24_F_0	BJ983484
E_FL_mxg-_06H02_F_0	BJ983485
E_FL_mxg-_06H05_F_0	BJ983486
E_FL_mxg-_06H08_F_0	BJ983487
E_FL_mxg-_06H09_F_0	BJ983488
E_FL_mxg-_06H17_F_0	BJ983489
E_FL_mxg-_06H20_F_0	BJ983490
E_FL_mxg-_06I02_F_0	BJ983491
E_FL_mxg-_06I04_F_0	BJ983492

E_FL_mxg-_06I07_F_0	BJ983493
E_FL_mxg-_06I08_F_0	BJ983494
E_FL_mxg-_06I09_F_0	BJ983495
E_FL_mxg-_06I12_F_0	BJ983496
E_FL_mxg-_06I13_F_0	BJ983497
E_FL_mxg-_06I18_F_0	BJ983498
E_FL_mxg-_06I21_F_0	BJ983499
E_FL_mxg-_06I22_F_0	BJ983500
E_FL_mxg-_06I23_F_0	BJ983501
E_FL_mxg-_06I24_F_0	BJ983502
E_FL_mxg-_06J01_F_0	BJ983503
E_FL_mxg-_06J05_F_0	BJ983504
E_FL_mxg-_06J06_F_0	BJ983505
E_FL_mxg-_06J07_F_0	BJ983506
E_FL_mxg-_06J08_F_0	BJ983507
E_FL_mxg-_06J10_F_0	BJ983508
E_FL_mxg-_06J13_F_0	BJ983509
E_FL_mxg-_06J18_F_0	BJ983510
E_FL_mxg-_06J19_F_0	BJ983511
E_FL_mxg-_06J21_F_0	BJ983512
E_FL_mxg-_06J22_F_0	BJ983513
E_FL_mxg-_06J23_F_0	BJ983514
E_FL_mxg-_06K02_F_0	BJ983515
E_FL_mxg-_06K03_F_0	BJ983516
E_FL_mxg-_06K08_F_0	BJ983517
E_FL_mxg-_06K11_F_0	BJ983518
E_FL_mxg-_06K12_F_0	BJ983519
E_FL_mxg-_06K17_F_0	BJ983520
E_FL_mxg-_06K20_F_0	BJ983521
E_FL_mxg-_06K21_F_0	BJ983522
E_FL_mxg-_06K22_F_0	BJ983523
E_FL_mxg-_06L01_F_0	BJ983524
E_FL_mxg-_06L03_F_0	BJ983525
E_FL_mxg-_06L04_F_0	BJ983526
E_FL_mxg-_06L05_F_0	BJ983527
E_FL_mxg-_06L07_F_0	BJ983528
E_FL_mxg-_06L08_F_0	BJ983529
E_FL_mxg-_06L09_F_0	BJ983530
E_FL_mxg-_06L11_F_0	BJ983531
E_FL_mxg-_06L14_F_0	BJ983532
E_FL_mxg-_06L19_F_0	BJ983533
E_FL_mxg-_06M01_F_0	BJ983534
E_FL_mxg-_06M02_F_0	BJ983535
E_FL_mxg-_06M03_F_0	BJ983536
E_FL_mxg-_06M07_F_0	BJ983537
E_FL_mxg-_06M09_F_0	BJ983538
E_FL_mxg-_06M10_F_0	BJ983539
E_FL_mxg-_06M11_F_0	BJ983540
E_FL_mxg-_06M13_F_0	BJ983541
E_FL_mxg-_06M14_F_0	BJ983542
E_FL_mxg-_06M15_F_0	BJ983543
E_FL_mxg-_06M16_F_0	BJ983544
E_FL_mxg-_06M17_F_0	BJ983545
E_FL_mxg-_06M19_F_0	BJ983546

E_FL_mxg-_06M23_F_0	BJ983547
E_FL_mxg-_06M24_F_0	BJ983548
E_FL_mxg-_06N01_F_0	BJ983549
E_FL_mxg-_06N04_F_0	BJ983550
E_FL_mxg-_06N06_F_0	BJ983551
E_FL_mxg-_06N08_F_0	BJ983552
E_FL_mxg-_06N16_F_0	BJ983553
E_FL_mxg-_06N17_F_0	BJ983554
E_FL_mxg-_06N18_F_0	BJ983555
E_FL_mxg-_06N21_F_0	BJ983556
E_FL_mxg-_06N22_F_0	BJ983557
E_FL_mxg-_06O01_F_0	BJ983558
E_FL_mxg-_06O02_F_0	BJ983559
E_FL_mxg-_06O04_F_0	BJ983560
E_FL_mxg-_06O05_F_0	BJ983561
E_FL_mxg-_06O08_F_0	BJ983562
E_FL_mxg-_06O09_F_0	BJ983563
E_FL_mxg-_06O13_F_0	BJ983564
E_FL_mxg-_06O15_F_0	BJ983565
E_FL_mxg-_06O22_F_0	BJ983566
E_FL_mxg-_06O24_F_0	BJ983567
E_FL_mxg-_06P03_F_0	BJ983568
E_FL_mxg-_06P04_F_0	BJ983569
E_FL_mxg-_06P05_F_0	BJ983570
E_FL_mxg-_06P08_F_0	BJ983571
E_FL_mxg-_06P10_F_0	BJ983572
E_FL_mxg-_06P13_F_0	BJ983573
E_FL_mxg-_06P15_F_0	BJ983574
E_FL_mxg-_06P16_F_0	BJ983575
E_FL_mxg-_06P17_F_0	BJ983576
E_FL_mxg-_06P18_F_0	BJ983577
E_FL_mxg-_06P19_F_0	BJ983578
E_FL_mxg-_06P20_F_0	BJ983579
E_FL_mxg-_06P22_F_0	BJ983580
E_FL_mxg-_07A02_F_0	BJ983581
E_FL_mxg-_07A07_F_0	BJ983582
E_FL_mxg-_07A08_F_0	BJ983583
E_FL_mxg-_07A09_F_0	BJ983584
E_FL_mxg-_07A10_F_0	BJ983585
E_FL_mxg-_07A11_F_0	BJ983586
E_FL_mxg-_07A12_F_0	BJ983587
E_FL_mxg-_07A13_F_0	BJ983588
E_FL_mxg-_07A16_F_0	BJ983589
E_FL_mxg-_07A19_F_0	BJ983590
E_FL_mxg-_07A21_F_0	BJ983591
E_FL_mxg-_07B02_F_0	BJ983592
E_FL_mxg-_07B03_F_0	BJ983593
E_FL_mxg-_07B05_F_0	BJ983594
E_FL_mxg-_07B08_F_0	BJ983595
E_FL_mxg-_07B10_F_0	BJ983596
E_FL_mxg-_07B12_F_0	BJ983597
E_FL_mxg-_07B15_F_0	BJ983598
E_FL_mxg-_07B17_F_0	BJ983599
E_FL_mxg-_07B23_F_0	BJ983600

E_FL_mxg-_07B24_F_0	BJ983601
E_FL_mxg-_07C04_F_0	BJ983602
E_FL_mxg-_07C10_F_0	BJ983603
E_FL_mxg-_07C13_F_0	BJ983604
E_FL_mxg-_07C19_F_0	BJ983605
E_FL_mxg-_07C22_F_0	BJ983606
E_FL_mxg-_07C23_F_0	BJ983607
E_FL_mxg-_07D01_F_0	BJ983608
E_FL_mxg-_07D12_F_0	BJ983609
E_FL_mxg-_07D13_F_0	BJ983610
E_FL_mxg-_07D16_F_0	BJ983611
E_FL_mxg-_07D19_F_0	BJ983612
E_FL_mxg-_07D24_F_0	BJ983613
E_FL_mxg-_07E05_F_0	BJ983614
E_FL_mxg-_07E07_F_0	BJ983615
E_FL_mxg-_07E08_F_0	BJ983616
E_FL_mxg-_07E09_F_0	BJ983617
E_FL_mxg-_07E11_F_0	BJ983618
E_FL_mxg-_07E13_F_0	BJ983619
E_FL_mxg-_07E17_F_0	BJ983620
E_FL_mxg-_07E19_F_0	BJ983621
E_FL_mxg-_07E21_F_0	BJ983622
E_FL_mxg-_07E22_F_0	BJ983623
E_FL_mxg-_07E23_F_0	BJ983624
E_FL_mxg-_07F03_F_0	BJ983625
E_FL_mxg-_07F04_F_0	BJ983626
E_FL_mxg-_07F06_F_0	BJ983627
E_FL_mxg-_07F10_F_0	BJ983628
E_FL_mxg-_07F13_F_0	BJ983629
E_FL_mxg-_07F14_F_0	BJ983630
E_FL_mxg-_07F16_F_0	BJ983631
E_FL_mxg-_07F17_F_0	BJ983632
E_FL_mxg-_07F20_F_0	BJ983633
E_FL_mxg-_07F21_F_0	BJ983634
E_FL_mxg-_07F22_F_0	BJ983635
E_FL_mxg-_07F24_F_0	BJ983636
E_FL_mxg-_07G02_F_0	BJ983637
E_FL_mxg-_07G04_F_0	BJ983638
E_FL_mxg-_07G06_F_0	BJ983639
E_FL_mxg-_07G07_F_0	BJ983640
E_FL_mxg-_07G08_F_0	BJ983641
E_FL_mxg-_07G10_F_0	BJ983642
E_FL_mxg-_07G14_F_0	BJ983643
E_FL_mxg-_07G15_F_0	BJ983644
E_FL_mxg-_07G16_F_0	BJ983645
E_FL_mxg-_07G20_F_0	BJ983646
E_FL_mxg-_07G21_F_0	BJ983647
E_FL_mxg-_07G22_F_0	BJ983648
E_FL_mxg-_07H01_F_0	BJ983649
E_FL_mxg-_07H05_F_0	BJ983650
E_FL_mxg-_07H09_F_0	BJ983651
E_FL_mxg-_07H10_F_0	BJ983652
E_FL_mxg-_07H11_F_0	BJ983653
E_FL_mxg-_07H12_F_0	BJ983654

E_FL_mxg-_07H13_F_0	BJ983655
E_FL_mxg-_07H16_F_0	BJ983656
E_FL_mxg-_07H17_F_0	BJ983657
E_FL_mxg-_07H19_F_0	BJ983658
E_FL_mxg-_07H20_F_0	BJ983659
E_FL_mxg-_07H21_F_0	BJ983660
E_FL_mxg-_07H23_F_0	BJ983661
E_FL_mxg-_07I01_F_0	BJ983662
E_FL_mxg-_07I02_F_0	BJ983663
E_FL_mxg-_07I05_F_0	BJ983664
E_FL_mxg-_07I07_F_0	BJ983665
E_FL_mxg-_07I08_F_0	BJ983666
E_FL_mxg-_07I12_F_0	BJ983667
E_FL_mxg-_07I13_F_0	BJ983668
E_FL_mxg-_07I17_F_0	BJ983669
E_FL_mxg-_07I23_F_0	BJ983670
E_FL_mxg-_07I24_F_0	BJ983671
E_FL_mxg-_07J02_F_0	BJ983672
E_FL_mxg-_07J03_F_0	BJ983673
E_FL_mxg-_07J04_F_0	BJ983674
E_FL_mxg-_07J06_F_0	BJ983675
E_FL_mxg-_07J07_F_0	BJ983676
E_FL_mxg-_07J09_F_0	BJ983677
E_FL_mxg-_07J10_F_0	BJ983678
E_FL_mxg-_07J11_F_0	BJ983679
E_FL_mxg-_07J13_F_0	BJ983680
E_FL_mxg-_07J14_F_0	BJ983681
E_FL_mxg-_07J16_F_0	BJ983682
E_FL_mxg-_07J17_F_0	BJ983683
E_FL_mxg-_07J19_F_0	BJ983684
E_FL_mxg-_07J22_F_0	BJ983685
E_FL_mxg-_07J23_F_0	BJ983686
E_FL_mxg-_07J24_F_0	BJ983687
E_FL_mxg-_07K03_F_0	BJ983688
E_FL_mxg-_07K05_F_0	BJ983689
E_FL_mxg-_07K06_F_0	BJ983690
E_FL_mxg-_07K08_F_0	BJ983691
E_FL_mxg-_07K10_F_0	BJ983692
E_FL_mxg-_07K12_F_0	BJ983693
E_FL_mxg-_07K13_F_0	BJ983694
E_FL_mxg-_07K18_F_0	BJ983695
E_FL_mxg-_07K19_F_0	BJ983696
E_FL_mxg-_07K22_F_0	BJ983697
E_FL_mxg-_07K23_F_0	BJ983698
E_FL_mxg-_07K24_F_0	BJ983699
E_FL_mxg-_07L04_F_0	BJ983700
E_FL_mxg-_07L05_F_0	BJ983701
E_FL_mxg-_07L06_F_0	BJ983702
E_FL_mxg-_07L07_F_0	BJ983703
E_FL_mxg-_07L08_F_0	BJ983704
E_FL_mxg-_07L09_F_0	BJ983705
E_FL_mxg-_07L10_F_0	BJ983706
E_FL_mxg-_07L11_F_0	BJ983707
E_FL_mxg-_07L14_F_0	BJ983708

E_FL_mxg-_07L15_F_0	BJ983709
E_FL_mxg-_07L17_F_0	BJ983710
E_FL_mxg-_07L19_F_0	BJ983711
E_FL_mxg-_07L20_F_0	BJ983712
E_FL_mxg-_07L22_F_0	BJ983713
E_FL_mxg-_07L24_F_0	BJ983714
E_FL_mxg-_07M01_F_0	BJ983715
E_FL_mxg-_07M02_F_0	BJ983716
E_FL_mxg-_07M03_F_0	BJ983717
E_FL_mxg-_07M08_F_0	BJ983718
E_FL_mxg-_07M10_F_0	BJ983719
E_FL_mxg-_07M13_F_0	BJ983720
E_FL_mxg-_07M16_F_0	BJ983721
E_FL_mxg-_07M17_F_0	BJ983722
E_FL_mxg-_07M23_F_0	BJ983723
E_FL_mxg-_07N03_F_0	BJ983724
E_FL_mxg-_07N04_F_0	BJ983725
E_FL_mxg-_07N05_F_0	BJ983726
E_FL_mxg-_07N08_F_0	BJ983727
E_FL_mxg-_07N16_F_0	BJ983728
E_FL_mxg-_07N17_F_0	BJ983729
E_FL_mxg-_07N18_F_0	BJ983730
E_FL_mxg-_07N20_F_0	BJ983731
E_FL_mxg-_07N24_F_0	BJ983732
E_FL_mxg-_07O01_F_0	BJ983733
E_FL_mxg-_07O02_F_0	BJ983734
E_FL_mxg-_07O04_F_0	BJ983735
E_FL_mxg-_07O06_F_0	BJ983736
E_FL_mxg-_07O09_F_0	BJ983737
E_FL_mxg-_07O10_F_0	BJ983738
E_FL_mxg-_07O11_F_0	BJ983739
E_FL_mxg-_07O12_F_0	BJ983740
E_FL_mxg-_07O15_F_0	BJ983741
E_FL_mxg-_07O16_F_0	BJ983742
E_FL_mxg-_07O17_F_0	BJ983743
E_FL_mxg-_07O18_F_0	BJ983744
E_FL_mxg-_07O20_F_0	BJ983745
E_FL_mxg-_07O23_F_0	BJ983746
E_FL_mxg-_07P04_F_0	BJ983747
E_FL_mxg-_07P10_F_0	BJ983748
E_FL_mxg-_07P11_F_0	BJ983749
E_FL_mxg-_07P15_F_0	BJ983750
E_FL_mxg-_07P21_F_0	BJ983751
E_FL_mxg-_07P23_F_0	BJ983752
E_FL_mxg-_08A02_F_0	BJ983753
E_FL_mxg-_08A03_F_0	BJ983754
E_FL_mxg-_08A04_F_0	BJ983755
E_FL_mxg-_08A08_F_0	BJ983756
E_FL_mxg-_08A12_F_0	BJ983757
E_FL_mxg-_08A14_F_0	BJ983758
E_FL_mxg-_08A21_F_0	BJ983759
E_FL_mxg-_08A23_F_0	BJ983760
E_FL_mxg-_08B02_F_0	BJ983761
E_FL_mxg-_08B06_F_0	BJ983762

E_FL_mxg-_08B07_F_0	BJ983763
E_FL_mxg-_08B09_F_0	BJ983764
E_FL_mxg-_08B10_F_0	BJ983765
E_FL_mxg-_08B12_F_0	BJ983766
E_FL_mxg-_08B14_F_0	BJ983767
E_FL_mxg-_08B18_F_0	BJ983768
E_FL_mxg-_08B19_F_0	BJ983769
E_FL_mxg-_08B21_F_0	BJ983770
E_FL_mxg-_08C01_F_0	BJ983771
E_FL_mxg-_08C03_F_0	BJ983772
E_FL_mxg-_08C05_F_0	BJ983773
E_FL_mxg-_08C06_F_0	BJ983774
E_FL_mxg-_08C07_F_0	BJ983775
E_FL_mxg-_08C10_F_0	BJ983776
E_FL_mxg-_08C11_F_0	BJ983777
E_FL_mxg-_08C12_F_0	BJ983778
E_FL_mxg-_08C13_F_0	BJ983779
E_FL_mxg-_08C15_F_0	BJ983780
E_FL_mxg-_08C17_F_0	BJ983781
E_FL_mxg-_08C18_F_0	BJ983782
E_FL_mxg-_08C19_F_0	BJ983783
E_FL_mxg-_08D01_F_0	BJ983784
E_FL_mxg-_08D03_F_0	BJ983785
E_FL_mxg-_08D05_F_0	BJ983786
E_FL_mxg-_08D07_F_0	BJ983787
E_FL_mxg-_08D08_F_0	BJ983788
E_FL_mxg-_08D10_F_0	BJ983789
E_FL_mxg-_08D12_F_0	BJ983790
E_FL_mxg-_08D14_F_0	BJ983791
E_FL_mxg-_08D15_F_0	BJ983792
E_FL_mxg-_08D16_F_0	BJ983793
E_FL_mxg-_08D18_F_0	BJ983794
E_FL_mxg-_08D23_F_0	BJ983795
E_FL_mxg-_08D24_F_0	BJ983796
E_FL_mxg-_08E01_F_0	BJ983797
E_FL_mxg-_08E04_F_0	BJ983798
E_FL_mxg-_08E06_F_0	BJ983799
E_FL_mxg-_08E09_F_0	BJ983800
E_FL_mxg-_08E12_F_0	BJ983801
E_FL_mxg-_08E18_F_0	BJ983802
E_FL_mxg-_08E19_F_0	BJ983803
E_FL_mxg-_08E20_F_0	BJ983804
E_FL_mxg-_08E23_F_0	BJ983805
E_FL_mxg-_08F03_F_0	BJ983806
E_FL_mxg-_08F05_F_0	BJ983807
E_FL_mxg-_08F06_F_0	BJ983808
E_FL_mxg-_08F08_F_0	BJ983809
E_FL_mxg-_08F09_F_0	BJ983810
E_FL_mxg-_08F10_F_0	BJ983811
E_FL_mxg-_08F12_F_0	BJ983812
E_FL_mxg-_08F16_F_0	BJ983813
E_FL_mxg-_08F18_F_0	BJ983814
E_FL_mxg-_08F21_F_0	BJ983815
E_FL_mxg-_08F22_F_0	BJ983816

E_FL_mxg-_08F23_F_0	BJ983817
E_FL_mxg-_08G03_F_0	BJ983818
E_FL_mxg-_08G06_F_0	BJ983819
E_FL_mxg-_08G07_F_0	BJ983820
E_FL_mxg-_08G08_F_0	BJ983821
E_FL_mxg-_08G09_F_0	BJ983822
E_FL_mxg-_08G10_F_0	BJ983823
E_FL_mxg-_08G12_F_0	BJ983824
E_FL_mxg-_08G17_F_0	BJ983825
E_FL_mxg-_08G20_F_0	BJ983826
E_FL_mxg-_08H02_F_0	BJ983827
E_FL_mxg-_08H04_F_0	BJ983828
E_FL_mxg-_08H05_F_0	BJ983829
E_FL_mxg-_08H06_F_0	BJ983830
E_FL_mxg-_08H08_F_0	BJ983831
E_FL_mxg-_08H09_F_0	BJ983832
E_FL_mxg-_08H13_F_0	BJ983833
E_FL_mxg-_08H16_F_0	BJ983834
E_FL_mxg-_08H17_F_0	BJ983835
E_FL_mxg-_08H18_F_0	BJ983836
E_FL_mxg-_08H19_F_0	BJ983837
E_FL_mxg-_08H22_F_0	BJ983838
E_FL_mxg-_08H24_F_0	BJ983839
E_FL_mxg-_08I04_F_0	BJ983840
E_FL_mxg-_08I05_F_0	BJ983841
E_FL_mxg-_08I08_F_0	BJ983842
E_FL_mxg-_08I11_F_0	BJ983843
E_FL_mxg-_08I15_F_0	BJ983844
E_FL_mxg-_08I20_F_0	BJ983845
E_FL_mxg-_08I22_F_0	BJ983846
E_FL_mxg-_08I23_F_0	BJ983847
E_FL_mxg-_08J01_F_0	BJ983848
E_FL_mxg-_08J02_F_0	BJ983849
E_FL_mxg-_08J03_F_0	BJ983850
E_FL_mxg-_08J06_F_0	BJ983851
E_FL_mxg-_08J07_F_0	BJ983852
E_FL_mxg-_08J11_F_0	BJ983853
E_FL_mxg-_08J14_F_0	BJ983854
E_FL_mxg-_08J16_F_0	BJ983855
E_FL_mxg-_08J20_F_0	BJ983856
E_FL_mxg-_08J22_F_0	BJ983857
E_FL_mxg-_08J24_F_0	BJ983858
E_FL_mxg-_08K04_F_0	BJ983859
E_FL_mxg-_08K05_F_0	BJ983860
E_FL_mxg-_08K06_F_0	BJ983861
E_FL_mxg-_08K08_F_0	BJ983862
E_FL_mxg-_08K09_F_0	BJ983863
E_FL_mxg-_08K15_F_0	BJ983864
E_FL_mxg-_08K16_F_0	BJ983865
E_FL_mxg-_08K18_F_0	BJ983866
E_FL_mxg-_08K21_F_0	BJ983867
E_FL_mxg-_08K22_F_0	BJ983868
E_FL_mxg-_08K23_F_0	BJ983869
E_FL_mxg-_08L01_F_0	BJ983870

E_FL_mxg-_08L02_F_0	BJ983871
E_FL_mxg-_08L03_F_0	BJ983872
E_FL_mxg-_08L04_F_0	BJ983873
E_FL_mxg-_08L05_F_0	BJ983874
E_FL_mxg-_08L08_F_0	BJ983875
E_FL_mxg-_08L09_F_0	BJ983876
E_FL_mxg-_08L12_F_0	BJ983877
E_FL_mxg-_08L13_F_0	BJ983878
E_FL_mxg-_08L15_F_0	BJ983879
E_FL_mxg-_08L18_F_0	BJ983880
E_FL_mxg-_08L20_F_0	BJ983881
E_FL_mxg-_08L22_F_0	BJ983882
E_FL_mxg-_08L23_F_0	BJ983883
E_FL_mxg-_08L24_F_0	BJ983884
E_FL_mxg-_08M02_F_0	BJ983885
E_FL_mxg-_08M05_F_0	BJ983886
E_FL_mxg-_08M06_F_0	BJ983887
E_FL_mxg-_08M10_F_0	BJ983888
E_FL_mxg-_08M11_F_0	BJ983889
E_FL_mxg-_08M15_F_0	BJ983890
E_FL_mxg-_08M16_F_0	BJ983891
E_FL_mxg-_08M18_F_0	BJ983892
E_FL_mxg-_08M19_F_0	BJ983893
E_FL_mxg-_08M20_F_0	BJ983894
E_FL_mxg-_08M22_F_0	BJ983895
E_FL_mxg-_08M24_F_0	BJ983896
E_FL_mxg-_08N03_F_0	BJ983897
E_FL_mxg-_08N04_F_0	BJ983898
E_FL_mxg-_08N06_F_0	BJ983899
E_FL_mxg-_08N11_F_0	BJ983900
E_FL_mxg-_08N16_F_0	BJ983901
E_FL_mxg-_08N17_F_0	BJ983902
E_FL_mxg-_08N18_F_0	BJ983903
E_FL_mxg-_08N21_F_0	BJ983904
E_FL_mxg-_08N23_F_0	BJ983905
E_FL_mxg-_08O01_F_0	BJ983906
E_FL_mxg-_08O02_F_0	BJ983907
E_FL_mxg-_08O07_F_0	BJ983908
E_FL_mxg-_08O09_F_0	BJ983909
E_FL_mxg-_08O12_F_0	BJ983910
E_FL_mxg-_08O14_F_0	BJ983911
E_FL_mxg-_08O15_F_0	BJ983912
E_FL_mxg-_08O18_F_0	BJ983913
E_FL_mxg-_08O20_F_0	BJ983914
E_FL_mxg-_08O23_F_0	BJ983915
E_FL_mxg-_08P02_F_0	BJ983916
E_FL_mxg-_08P05_F_0	BJ983917
E_FL_mxg-_08P07_F_0	BJ983918
E_FL_mxg-_08P08_F_0	BJ983919
E_FL_mxg-_08P13_F_0	BJ983920
E_FL_mxg-_08P17_F_0	BJ983921
E_FL_mxg-_08P18_F_0	BJ983922
E_FL_mxg-_08P20_F_0	BJ983923
E_FL_mxg-_08P22_F_0	BJ983924

E_FL_mxg-_09A04_F_0	BJ983925
E_FL_mxg-_09A05_F_0	BJ983926
E_FL_mxg-_09A07_F_0	BJ983927
E_FL_mxg-_09A11_F_0	BJ983928
E_FL_mxg-_09A13_F_0	BJ983929
E_FL_mxg-_09A23_F_0	BJ983930
E_FL_mxg-_09B04_F_0	BJ983931
E_FL_mxg-_09B06_F_0	BJ983932
E_FL_mxg-_09B10_F_0	BJ983933
E_FL_mxg-_09B11_F_0	BJ983934
E_FL_mxg-_09B12_F_0	BJ983935
E_FL_mxg-_09B14_F_0	BJ983936
E_FL_mxg-_09B16_F_0	BJ983937
E_FL_mxg-_09B18_F_0	BJ983938
E_FL_mxg-_09B22_F_0	BJ983939
E_FL_mxg-_09C02_F_0	BJ983940
E_FL_mxg-_09C08_F_0	BJ983941
E_FL_mxg-_09C09_F_0	BJ983942
E_FL_mxg-_09C10_F_0	BJ983943
E_FL_mxg-_09C12_F_0	BJ983944
E_FL_mxg-_09C14_F_0	BJ983945
E_FL_mxg-_09C21_F_0	BJ983946
E_FL_mxg-_09C23_F_0	BJ983947
E_FL_mxg-_09C24_F_0	BJ983948
E_FL_mxg-_09D01_F_0	BJ983949
E_FL_mxg-_09D09_F_0	BJ983950
E_FL_mxg-_09D14_F_0	BJ983951
E_FL_mxg-_09D21_F_0	BJ983952
E_FL_mxg-_09D22_F_0	BJ983953
E_FL_mxg-_09D23_F_0	BJ983954
E_FL_mxg-_09E03_F_0	BJ983955
E_FL_mxg-_09E05_F_0	BJ983956
E_FL_mxg-_09E09_F_0	BJ983957
E_FL_mxg-_09E10_F_0	BJ983958
E_FL_mxg-_09E16_F_0	BJ983959
E_FL_mxg-_09E17_F_0	BJ983960
E_FL_mxg-_09F04_F_0	BJ983961
E_FL_mxg-_09F06_F_0	BJ983962
E_FL_mxg-_09F07_F_0	BJ983963
E_FL_mxg-_09F11_F_0	BJ983964
E_FL_mxg-_09F14_F_0	BJ983965
E_FL_mxg-_09F16_F_0	BJ983966
E_FL_mxg-_09F22_F_0	BJ983967
E_FL_mxg-_09G01_F_0	BJ983968
E_FL_mxg-_09G02_F_0	BJ983969
E_FL_mxg-_09G03_F_0	BJ983970
E_FL_mxg-_09G04_F_0	BJ983971
E_FL_mxg-_09G05_F_0	BJ983972
E_FL_mxg-_09G06_F_0	BJ983973
E_FL_mxg-_09G09_F_0	BJ983974
E_FL_mxg-_09G12_F_0	BJ983975
E_FL_mxg-_09G15_F_0	BJ983976
E_FL_mxg-_09G17_F_0	BJ983977
E_FL_mxg-_09G18_F_0	BJ983978

E_FL_mxg-_09G19_F_0	BJ983979
E_FL_mxg-_09G24_F_0	BJ983980
E_FL_mxg-_09H04_F_0	BJ983981
E_FL_mxg-_09H05_F_0	BJ983982
E_FL_mxg-_09H06_F_0	BJ983983
E_FL_mxg-_09H07_F_0	BJ983984
E_FL_mxg-_09H08_F_0	BJ983985
E_FL_mxg-_09H09_F_0	BJ983986
E_FL_mxg-_09H10_F_0	BJ983987
E_FL_mxg-_09H15_F_0	BJ983988
E_FL_mxg-_09H16_F_0	BJ983989
E_FL_mxg-_09H19_F_0	BJ983990
E_FL_mxg-_09H20_F_0	BJ983991
E_FL_mxg-_09H21_F_0	BJ983992
E_FL_mxg-_09H24_F_0	BJ983993
E_FL_mxg-_09I05_F_0	BJ983994
E_FL_mxg-_09I08_F_0	BJ983995
E_FL_mxg-_09I09_F_0	BJ983996
E_FL_mxg-_09I12_F_0	BJ983997
E_FL_mxg-_09I13_F_0	BJ983998
E_FL_mxg-_09I14_F_0	BJ983999
E_FL_mxg-_09I15_F_0	BJ984000
E_FL_mxg-_09I16_F_0	BJ984001
E_FL_mxg-_09I17_F_0	BJ984002
E_FL_mxg-_09I18_F_0	BJ984003
E_FL_mxg-_09I21_F_0	BJ984004
E_FL_mxg-_09I22_F_0	BJ984005
E_FL_mxg-_09J02_F_0	BJ984006
E_FL_mxg-_09J03_F_0	BJ984007
E_FL_mxg-_09J04_F_0	BJ984008
E_FL_mxg-_09J05_F_0	BJ984009
E_FL_mxg-_09J09_F_0	BJ984010
E_FL_mxg-_09J10_F_0	BJ984011
E_FL_mxg-_09J12_F_0	BJ984012
E_FL_mxg-_09J13_F_0	BJ984013
E_FL_mxg-_09J14_F_0	BJ984014
E_FL_mxg-_09J15_F_0	BJ984015
E_FL_mxg-_09J18_F_0	BJ984016
E_FL_mxg-_09J19_F_0	BJ984017
E_FL_mxg-_09J20_F_0	BJ984018
E_FL_mxg-_09J24_F_0	BJ984019
E_FL_mxg-_09K04_F_0	BJ984020
E_FL_mxg-_09K06_F_0	BJ984021
E_FL_mxg-_09K08_F_0	BJ984022
E_FL_mxg-_09K10_F_0	BJ984023
E_FL_mxg-_09K12_F_0	BJ984024
E_FL_mxg-_09K13_F_0	BJ984025
E_FL_mxg-_09K14_F_0	BJ984026
E_FL_mxg-_09K15_F_0	BJ984027
E_FL_mxg-_09K17_F_0	BJ984028
E_FL_mxg-_09K19_F_0	BJ984029
E_FL_mxg-_09K20_F_0	BJ984030
E_FL_mxg-_09K21_F_0	BJ984031
E_FL_mxg-_09K24_F_0	BJ984032

E_FL_mxg-_09L02_F_0	BJ984033
E_FL_mxg-_09L04_F_0	BJ984034
E_FL_mxg-_09L06_F_0	BJ984035
E_FL_mxg-_09L08_F_0	BJ984036
E_FL_mxg-_09L09_F_0	BJ984037
E_FL_mxg-_09L12_F_0	BJ984038
E_FL_mxg-_09L15_F_0	BJ984039
E_FL_mxg-_09L16_F_0	BJ984040
E_FL_mxg-_09L18_F_0	BJ984041
E_FL_mxg-_09L19_F_0	BJ984042
E_FL_mxg-_09L20_F_0	BJ984043
E_FL_mxg-_09L22_F_0	BJ984044
E_FL_mxg-_09L23_F_0	BJ984045
E_FL_mxg-_09L24_F_0	BJ984046
E_FL_mxg-_09M02_F_0	BJ984047
E_FL_mxg-_09M05_F_0	BJ984048
E_FL_mxg-_09M10_F_0	BJ984049
E_FL_mxg-_09M11_F_0	BJ984050
E_FL_mxg-_09M12_F_0	BJ984051
E_FL_mxg-_09M13_F_0	BJ984052
E_FL_mxg-_09M16_F_0	BJ984053
E_FL_mxg-_09M17_F_0	BJ984054
E_FL_mxg-_09M18_F_0	BJ984055
E_FL_mxg-_09M19_F_0	BJ984056
E_FL_mxg-_09M20_F_0	BJ984057
E_FL_mxg-_09M24_F_0	BJ984058
E_FL_mxg-_09N04_F_0	BJ984059
E_FL_mxg-_09N07_F_0	BJ984060
E_FL_mxg-_09N08_F_0	BJ984061
E_FL_mxg-_09N10_F_0	BJ984062
E_FL_mxg-_09N11_F_0	BJ984063
E_FL_mxg-_09N15_F_0	BJ984064
E_FL_mxg-_09N17_F_0	BJ984065
E_FL_mxg-_09N20_F_0	BJ984066
E_FL_mxg-_09N21_F_0	BJ984067
E_FL_mxg-_09N22_F_0	BJ984068
E_FL_mxg-_09N24_F_0	BJ984069
E_FL_mxg-_09O01_F_0	BJ984070
E_FL_mxg-_09O04_F_0	BJ984071
E_FL_mxg-_09O05_F_0	BJ984072
E_FL_mxg-_09O09_F_0	BJ984073
E_FL_mxg-_09O11_F_0	BJ984074
E_FL_mxg-_09O12_F_0	BJ984075
E_FL_mxg-_09O13_F_0	BJ984076
E_FL_mxg-_09O15_F_0	BJ984077
E_FL_mxg-_09O16_F_0	BJ984078
E_FL_mxg-_09O17_F_0	BJ984079
E_FL_mxg-_09O20_F_0	BJ984080
E_FL_mxg-_09O24_F_0	BJ984081
E_FL_mxg-_09P01_F_0	BJ984082
E_FL_mxg-_09P03_F_0	BJ984083
E_FL_mxg-_09P07_F_0	BJ984084
E_FL_mxg-_09P11_F_0	BJ984085
E_FL_mxg-_09P13_F_0	BJ984086

E_FL_mxg-_09P14_F_0	BJ984087
E_FL_mxg-_09P16_F_0	BJ984088
E_FL_mxg-_09P18_F_0	BJ984089
E_FL_mxg-_09P20_F_0	BJ984090
E_FL_mxg-_09P22_F_0	BJ984091
E_FL_mxg-_09P23_F_0	BJ984092
E_FL_mxg-_09P24_F_0	BJ984093
E_FL_mxg-_10A03_F_0	BJ984094
E_FL_mxg-_10A04_F_0	BJ984095
E_FL_mxg-_10A06_F_0	BJ984096
E_FL_mxg-_10A10_F_0	BJ984097
E_FL_mxg-_10A21_F_0	BJ984098
E_FL_mxg-_10A22_F_0	BJ984099
E_FL_mxg-_10A23_F_0	BJ984100
E_FL_mxg-_10B03_F_0	BJ984101
E_FL_mxg-_10B07_F_0	BJ984102
E_FL_mxg-_10B08_F_0	BJ984103
E_FL_mxg-_10B11_F_0	BJ984104
E_FL_mxg-_10B14_F_0	BJ984105
E_FL_mxg-_10B15_F_0	BJ984106
E_FL_mxg-_10B17_F_0	BJ984107
E_FL_mxg-_10B18_F_0	BJ984108
E_FL_mxg-_10B19_F_0	BJ984109
E_FL_mxg-_10B23_F_0	BJ984110
E_FL_mxg-_10C04_F_0	BJ984111
E_FL_mxg-_10C05_F_0	BJ984112
E_FL_mxg-_10C10_F_0	BJ984113
E_FL_mxg-_10C11_F_0	BJ984114
E_FL_mxg-_10C12_F_0	BJ984115
E_FL_mxg-_10C13_F_0	BJ984116
E_FL_mxg-_10C16_F_0	BJ984117
E_FL_mxg-_10C18_F_0	BJ984118
E_FL_mxg-_10C20_F_0	BJ984119
E_FL_mxg-_10C22_F_0	BJ984120
E_FL_mxg-_10D02_F_0	BJ984121
E_FL_mxg-_10D03_F_0	BJ984122
E_FL_mxg-_10D05_F_0	BJ984123
E_FL_mxg-_10D06_F_0	BJ984124
E_FL_mxg-_10D07_F_0	BJ984125
E_FL_mxg-_10D15_F_0	BJ984126
E_FL_mxg-_10D16_F_0	BJ984127
E_FL_mxg-_10D20_F_0	BJ984128
E_FL_mxg-_10D21_F_0	BJ984129
E_FL_mxg-_10D22_F_0	BJ984130
E_FL_mxg-_10D23_F_0	BJ984131
E_FL_mxg-_10E01_F_0	BJ984132
E_FL_mxg-_10E04_F_0	BJ984133
E_FL_mxg-_10E05_F_0	BJ984134
E_FL_mxg-_10E08_F_0	BJ984135
E_FL_mxg-_10E11_F_0	BJ984136
E_FL_mxg-_10E12_F_0	BJ984137
E_FL_mxg-_10E14_F_0	BJ984138
E_FL_mxg-_10E17_F_0	BJ984139
E_FL_mxg-_10E22_F_0	BJ984140

E_FL_mxg-_10F01_F_0	BJ984141
E_FL_mxg-_10F03_F_0	BJ984142
E_FL_mxg-_10F04_F_0	BJ984143
E_FL_mxg-_10F06_F_0	BJ984144
E_FL_mxg-_10F07_F_0	BJ984145
E_FL_mxg-_10F08_F_0	BJ984146
E_FL_mxg-_10F11_F_0	BJ984147
E_FL_mxg-_10F12_F_0	BJ984148
E_FL_mxg-_10F14_F_0	BJ984149
E_FL_mxg-_10F18_F_0	BJ984150
E_FL_mxg-_10F19_F_0	BJ984151
E_FL_mxg-_10F20_F_0	BJ984152
E_FL_mxg-_10F22_F_0	BJ984153
E_FL_mxg-_10G01_F_0	BJ984154
E_FL_mxg-_10G02_F_0	BJ984155
E_FL_mxg-_10G03_F_0	BJ984156
E_FL_mxg-_10G05_F_0	BJ984157
E_FL_mxg-_10G09_F_0	BJ984158
E_FL_mxg-_10G10_F_0	BJ984159
E_FL_mxg-_10G20_F_0	BJ984160
E_FL_mxg-_10G21_F_0	BJ984161
E_FL_mxg-_10G22_F_0	BJ984162
E_FL_mxg-_10G23_F_0	BJ984163
E_FL_mxg-_10H02_F_0	BJ984164
E_FL_mxg-_10H10_F_0	BJ984165
E_FL_mxg-_10H11_F_0	BJ984166
E_FL_mxg-_10H12_F_0	BJ984167
E_FL_mxg-_10H14_F_0	BJ984168
E_FL_mxg-_10H15_F_0	BJ984169
E_FL_mxg-_10H17_F_0	BJ984170
E_FL_mxg-_10H18_F_0	BJ984171
E_FL_mxg-_10H22_F_0	BJ984172
E_FL_mxg-_10H24_F_0	BJ984173
E_FL_mxg-_10I03_F_0	BJ984174
E_FL_mxg-_10I04_F_0	BJ984175
E_FL_mxg-_10I08_F_0	BJ984176
E_FL_mxg-_10I09_F_0	BJ984177
E_FL_mxg-_10I10_F_0	BJ984178
E_FL_mxg-_10I12_F_0	BJ984179
E_FL_mxg-_10I17_F_0	BJ984180
E_FL_mxg-_10I18_F_0	BJ984181
E_FL_mxg-_10I20_F_0	BJ984182
E_FL_mxg-_10I21_F_0	BJ984183
E_FL_mxg-_10I24_F_0	BJ984184
E_FL_mxg-_10J03_F_0	BJ984185
E_FL_mxg-_10J04_F_0	BJ984186
E_FL_mxg-_10J05_F_0	BJ984187
E_FL_mxg-_10J06_F_0	BJ984188
E_FL_mxg-_10J11_F_0	BJ984189
E_FL_mxg-_10J15_F_0	BJ984190
E_FL_mxg-_10J16_F_0	BJ984191
E_FL_mxg-_10J18_F_0	BJ984192
E_FL_mxg-_10K04_F_0	BJ984193
E_FL_mxg-_10K06_F_0	BJ984194

E_FL_mxg-_10K07_F_0	BJ984195
E_FL_mxg-_10K08_F_0	BJ984196
E_FL_mxg-_10K10_F_0	BJ984197
E_FL_mxg-_10K11_F_0	BJ984198
E_FL_mxg-_10K13_F_0	BJ984199
E_FL_mxg-_10K18_F_0	BJ984200
E_FL_mxg-_10K22_F_0	BJ984201
E_FL_mxg-_10K23_F_0	BJ984202
E_FL_mxg-_10L02_F_0	BJ984203
E_FL_mxg-_10L06_F_0	BJ984204
E_FL_mxg-_10L07_F_0	BJ984205
E_FL_mxg-_10L08_F_0	BJ984206
E_FL_mxg-_10L09_F_0	BJ984207
E_FL_mxg-_10L10_F_0	BJ984208
E_FL_mxg-_10L12_F_0	BJ984209
E_FL_mxg-_10L13_F_0	BJ984210
E_FL_mxg-_10L23_F_0	BJ984211
E_FL_mxg-_10M02_F_0	BJ984212
E_FL_mxg-_10M09_F_0	BJ984213
E_FL_mxg-_10M11_F_0	BJ984214
E_FL_mxg-_10M15_F_0	BJ984215
E_FL_mxg-_10M16_F_0	BJ984216
E_FL_mxg-_10M17_F_0	BJ984217
E_FL_mxg-_10M18_F_0	BJ984218
E_FL_mxg-_10M19_F_0	BJ984219
E_FL_mxg-_10M22_F_0	BJ984220
E_FL_mxg-_10M23_F_0	BJ984221
E_FL_mxg-_10N02_F_0	BJ984222
E_FL_mxg-_10N03_F_0	BJ984223
E_FL_mxg-_10N08_F_0	BJ984224
E_FL_mxg-_10N09_F_0	BJ984225
E_FL_mxg-_10N13_F_0	BJ984226
E_FL_mxg-_10N14_F_0	BJ984227
E_FL_mxg-_10N18_F_0	BJ984228
E_FL_mxg-_10N20_F_0	BJ984229
E_FL_mxg-_10O03_F_0	BJ984230
E_FL_mxg-_10O04_F_0	BJ984231
E_FL_mxg-_10O05_F_0	BJ984232
E_FL_mxg-_10O08_F_0	BJ984233
E_FL_mxg-_10O09_F_0	BJ984234
E_FL_mxg-_10O10_F_0	BJ984235
E_FL_mxg-_10O11_F_0	BJ984236
E_FL_mxg-_10O14_F_0	BJ984237
E_FL_mxg-_10O19_F_0	BJ984238
E_FL_mxg-_10O21_F_0	BJ984239
E_FL_mxg-_10O22_F_0	BJ984240
E_FL_mxg-_10O23_F_0	BJ984241
E_FL_mxg-_10P01_F_0	BJ984242
E_FL_mxg-_10P02_F_0	BJ984243
E_FL_mxg-_10P04_F_0	BJ984244
E_FL_mxg-_10P05_F_0	BJ984245
E_FL_mxg-_10P06_F_0	BJ984246
E_FL_mxg-_10P08_F_0	BJ984247
E_FL_mxg-_10P09_F_0	BJ984248

E_FL_mxg-_10P10_F_0	BJ984249
E_FL_mxg-_10P11_F_0	BJ984250
E_FL_mxg-_10P13_F_0	BJ984251
E_FL_mxg-_10P15_F_0	BJ984252
E_FL_mxg-_10P16_F_0	BJ984253
E_FL_mxg-_10P21_F_0	BJ984254
E_FL_mxg-_10P22_F_0	BJ984255
E_FL_mxg-_11A02_F_0	BJ984256
E_FL_mxg-_11A04_F_0	BJ984257
E_FL_mxg-_11A05_F_0	BJ984258
E_FL_mxg-_11A06_F_0	BJ984259
E_FL_mxg-_11A08_F_0	BJ984260
E_FL_mxg-_11A09_F_0	BJ984261
E_FL_mxg-_11A10_F_0	BJ984262
E_FL_mxg-_11A11_F_0	BJ984263
E_FL_mxg-_11A12_F_0	BJ984264
E_FL_mxg-_11A15_F_0	BJ984265
E_FL_mxg-_11A18_F_0	BJ984266
E_FL_mxg-_11A19_F_0	BJ984267
E_FL_mxg-_11A20_F_0	BJ984268
E_FL_mxg-_11A21_F_0	BJ984269
E_FL_mxg-_11A22_F_0	BJ984270
E_FL_mxg-_11A23_F_0	BJ984271
E_FL_mxg-_11A24_F_0	BJ984272
E_FL_mxg-_11B06_F_0	BJ984273
E_FL_mxg-_11B07_F_0	BJ984274
E_FL_mxg-_11B09_F_0	BJ984275
E_FL_mxg-_11B10_F_0	BJ984276
E_FL_mxg-_11B11_F_0	BJ984277
E_FL_mxg-_11B13_F_0	BJ984278
E_FL_mxg-_11B19_F_0	BJ984279
E_FL_mxg-_11B21_F_0	BJ984280
E_FL_mxg-_11B22_F_0	BJ984281
E_FL_mxg-_11B23_F_0	BJ984282
E_FL_mxg-_11C04_F_0	BJ984283
E_FL_mxg-_11C13_F_0	BJ984284
E_FL_mxg-_11C15_F_0	BJ984285
E_FL_mxg-_11C16_F_0	BJ984286
E_FL_mxg-_11C17_F_0	BJ984287
E_FL_mxg-_11C19_F_0	BJ984288
E_FL_mxg-_11C22_F_0	BJ984289
E_FL_mxg-_11C24_F_0	BJ984290
E_FL_mxg-_11D01_F_0	BJ984291
E_FL_mxg-_11D02_F_0	BJ984292
E_FL_mxg-_11D03_F_0	BJ984293
E_FL_mxg-_11D07_F_0	BJ984294
E_FL_mxg-_11D08_F_0	BJ984295
E_FL_mxg-_11D10_F_0	BJ984296
E_FL_mxg-_11D13_F_0	BJ984297
E_FL_mxg-_11D14_F_0	BJ984298
E_FL_mxg-_11D17_F_0	BJ984299
E_FL_mxg-_11D18_F_0	BJ984300
E_FL_mxg-_11D19_F_0	BJ984301
E_FL_mxg-_11D22_F_0	BJ984302

E_FL_mxg-_11D24_F_0	BJ984303
E_FL_mxg-_11E04_F_0	BJ984304
E_FL_mxg-_11E05_F_0	BJ984305
E_FL_mxg-_11E06_F_0	BJ984306
E_FL_mxg-_11E10_F_0	BJ984307
E_FL_mxg-_11E11_F_0	BJ984308
E_FL_mxg-_11E14_F_0	BJ984309
E_FL_mxg-_11E15_F_0	BJ984310
E_FL_mxg-_11E16_F_0	BJ984311
E_FL_mxg-_11E19_F_0	BJ984312
E_FL_mxg-_11E20_F_0	BJ984313
E_FL_mxg-_11E22_F_0	BJ984314
E_FL_mxg-_11E24_F_0	BJ984315
E_FL_mxg-_11F01_F_0	BJ984316
E_FL_mxg-_11F02_F_0	BJ984317
E_FL_mxg-_11F04_F_0	BJ984318
E_FL_mxg-_11F05_F_0	BJ984319
E_FL_mxg-_11F07_F_0	BJ984320
E_FL_mxg-_11F08_F_0	BJ984321
E_FL_mxg-_11F09_F_0	BJ984322
E_FL_mxg-_11F11_F_0	BJ984323
E_FL_mxg-_11F12_F_0	BJ984324
E_FL_mxg-_11F15_F_0	BJ984325
E_FL_mxg-_11F20_F_0	BJ984326
E_FL_mxg-_11F23_F_0	BJ984327
E_FL_mxg-_11F24_F_0	BJ984328
E_FL_mxg-_11G04_F_0	BJ984329
E_FL_mxg-_11G05_F_0	BJ984330
E_FL_mxg-_11G06_F_0	BJ984331
E_FL_mxg-_11G09_F_0	BJ984332
E_FL_mxg-_11G10_F_0	BJ984333
E_FL_mxg-_11G12_F_0	BJ984334
E_FL_mxg-_11G13_F_0	BJ984335
E_FL_mxg-_11G14_F_0	BJ984336
E_FL_mxg-_11G15_F_0	BJ984337
E_FL_mxg-_11G21_F_0	BJ984338
E_FL_mxg-_11G22_F_0	BJ984339
E_FL_mxg-_11G23_F_0	BJ984340
E_FL_mxg-_11G24_F_0	BJ984341
E_FL_mxg-_11H02_F_0	BJ984342
E_FL_mxg-_11H03_F_0	BJ984343
E_FL_mxg-_11H04_F_0	BJ984344
E_FL_mxg-_11H07_F_0	BJ984345
E_FL_mxg-_11H08_F_0	BJ984346
E_FL_mxg-_11H09_F_0	BJ984347
E_FL_mxg-_11H12_F_0	BJ984348
E_FL_mxg-_11H13_F_0	BJ984349
E_FL_mxg-_11H14_F_0	BJ984350
E_FL_mxg-_11H16_F_0	BJ984351
E_FL_mxg-_11H19_F_0	BJ984352
E_FL_mxg-_11H20_F_0	BJ984353
E_FL_mxg-_11H22_F_0	BJ984354
E_FL_mxg-_11H24_F_0	BJ984355
E_FL_mxg-_11I01_F_0	BJ984356

E_FL_mxg-_11I03_F_0	BJ984357
E_FL_mxg-_11I05_F_0	BJ984358
E_FL_mxg-_11I06_F_0	BJ984359
E_FL_mxg-_11I08_F_0	BJ984360
E_FL_mxg-_11I09_F_0	BJ984361
E_FL_mxg-_11I11_F_0	BJ984362
E_FL_mxg-_11I15_F_0	BJ984363
E_FL_mxg-_11I16_F_0	BJ984364
E_FL_mxg-_11I17_F_0	BJ984365
E_FL_mxg-_11I19_F_0	BJ984366
E_FL_mxg-_11I20_F_0	BJ984367
E_FL_mxg-_11I21_F_0	BJ984368
E_FL_mxg-_11I22_F_0	BJ984369
E_FL_mxg-_11I24_F_0	BJ984370
E_FL_mxg-_11J01_F_0	BJ984371
E_FL_mxg-_11J03_F_0	BJ984372
E_FL_mxg-_11J04_F_0	BJ984373
E_FL_mxg-_11J05_F_0	BJ984374
E_FL_mxg-_11J09_F_0	BJ984375
E_FL_mxg-_11J12_F_0	BJ984376
E_FL_mxg-_11J15_F_0	BJ984377
E_FL_mxg-_11J18_F_0	BJ984378
E_FL_mxg-_11J19_F_0	BJ984379
E_FL_mxg-_11J21_F_0	BJ984380
E_FL_mxg-_11J22_F_0	BJ984381
E_FL_mxg-_11J23_F_0	BJ984382
E_FL_mxg-_11K03_F_0	BJ984383
E_FL_mxg-_11K04_F_0	BJ984384
E_FL_mxg-_11K07_F_0	BJ984385
E_FL_mxg-_11K09_F_0	BJ984386
E_FL_mxg-_11K12_F_0	BJ984387
E_FL_mxg-_11K14_F_0	BJ984388
E_FL_mxg-_11K18_F_0	BJ984389
E_FL_mxg-_11K19_F_0	BJ984390
E_FL_mxg-_11K22_F_0	BJ984391
E_FL_mxg-_11L04_F_0	BJ984392
E_FL_mxg-_11L05_F_0	BJ984393
E_FL_mxg-_11L09_F_0	BJ984394
E_FL_mxg-_11L10_F_0	BJ984395
E_FL_mxg-_11L11_F_0	BJ984396
E_FL_mxg-_11L15_F_0	BJ984397
E_FL_mxg-_11L17_F_0	BJ984398
E_FL_mxg-_11L18_F_0	BJ984399
E_FL_mxg-_11L19_F_0	BJ984400
E_FL_mxg-_11L22_F_0	BJ984401
E_FL_mxg-_11L24_F_0	BJ984402
E_FL_mxg-_11M01_F_0	BJ984403
E_FL_mxg-_11M02_F_0	BJ984404
E_FL_mxg-_11M05_F_0	BJ984405
E_FL_mxg-_11M06_F_0	BJ984406
E_FL_mxg-_11M11_F_0	BJ984407
E_FL_mxg-_11M14_F_0	BJ984408
E_FL_mxg-_11M15_F_0	BJ984409
E_FL_mxg-_11M17_F_0	BJ984410

E_FL_mxg-_11M22_F_0	BJ984411
E_FL_mxg-_11M24_F_0	BJ984412
E_FL_mxg-_11N08_F_0	BJ984413
E_FL_mxg-_11N10_F_0	BJ984414
E_FL_mxg-_11N11_F_0	BJ984415
E_FL_mxg-_11N14_F_0	BJ984416
E_FL_mxg-_11N15_F_0	BJ984417
E_FL_mxg-_11N16_F_0	BJ984418
E_FL_mxg-_11N17_F_0	BJ984419
E_FL_mxg-_11N18_F_0	BJ984420
E_FL_mxg-_11N20_F_0	BJ984421
E_FL_mxg-_11N23_F_0	BJ984422
E_FL_mxg-_11O01_F_0	BJ984423
E_FL_mxg-_11O02_F_0	BJ984424
E_FL_mxg-_11O03_F_0	BJ984425
E_FL_mxg-_11O05_F_0	BJ984426
E_FL_mxg-_11O07_F_0	BJ984427
E_FL_mxg-_11O11_F_0	BJ984428
E_FL_mxg-_11O13_F_0	BJ984429
E_FL_mxg-_11O15_F_0	BJ984430
E_FL_mxg-_11O16_F_0	BJ984431
E_FL_mxg-_11O17_F_0	BJ984432
E_FL_mxg-_11O18_F_0	BJ984433
E_FL_mxg-_11O19_F_0	BJ984434
E_FL_mxg-_11O20_F_0	BJ984435
E_FL_mxg-_11O21_F_0	BJ984436
E_FL_mxg-_11O22_F_0	BJ984437
E_FL_mxg-_11O23_F_0	BJ984438
E_FL_mxg-_11P02_F_0	BJ984439
E_FL_mxg-_11P04_F_0	BJ984440
E_FL_mxg-_11P05_F_0	BJ984441
E_FL_mxg-_11P06_F_0	BJ984442
E_FL_mxg-_11P07_F_0	BJ984443
E_FL_mxg-_11P10_F_0	BJ984444
E_FL_mxg-_11P11_F_0	BJ984445
E_FL_mxg-_11P12_F_0	BJ984446
E_FL_mxg-_11P13_F_0	BJ984447
E_FL_mxg-_11P17_F_0	BJ984448
E_FL_mxg-_11P18_F_0	BJ984449
E_FL_mxg-_11P19_F_0	BJ984450
E_FL_mxg-_11P21_F_0	BJ984451
E_FL_mxg-_11P22_F_0	BJ984452
E_FL_mxg-_12A01_F_0	BJ984453
E_FL_mxg-_12A02_F_0	BJ984454
E_FL_mxg-_12A04_F_0	BJ984455
E_FL_mxg-_12A07_F_0	BJ984456
E_FL_mxg-_12A08_F_0	BJ984457
E_FL_mxg-_12A09_F_0	BJ984458
E_FL_mxg-_12A10_F_0	BJ984459
E_FL_mxg-_12A11_F_0	BJ984460
E_FL_mxg-_12A15_F_0	BJ984461
E_FL_mxg-_12A16_F_0	BJ984462
E_FL_mxg-_12A18_F_0	BJ984463
E_FL_mxg-_12A22_F_0	BJ984464

E_FL_mxg-_12A23_F_0	BJ984465
E_FL_mxg-_12A24_F_0	BJ984466
E_FL_mxg-_12B01_F_0	BJ984467
E_FL_mxg-_12B02_F_0	BJ984468
E_FL_mxg-_12B07_F_0	BJ984469
E_FL_mxg-_12B10_F_0	BJ984470
E_FL_mxg-_12B12_F_0	BJ984471
E_FL_mxg-_12B13_F_0	BJ984472
E_FL_mxg-_12B14_F_0	BJ984473
E_FL_mxg-_12B15_F_0	BJ984474
E_FL_mxg-_12B18_F_0	BJ984475
E_FL_mxg-_12B21_F_0	BJ984476
E_FL_mxg-_12B22_F_0	BJ984477
E_FL_mxg-_12C02_F_0	BJ984478
E_FL_mxg-_12C08_F_0	BJ984479
E_FL_mxg-_12C09_F_0	BJ984480
E_FL_mxg-_12C10_F_0	BJ984481
E_FL_mxg-_12C12_F_0	BJ984482
E_FL_mxg-_12C15_F_0	BJ984483
E_FL_mxg-_12C17_F_0	BJ984484
E_FL_mxg-_12C18_F_0	BJ984485
E_FL_mxg-_12C19_F_0	BJ984486
E_FL_mxg-_12C20_F_0	BJ984487
E_FL_mxg-_12C21_F_0	BJ984488
E_FL_mxg-_12D01_F_0	BJ984489
E_FL_mxg-_12D04_F_0	BJ984490
E_FL_mxg-_12D05_F_0	BJ984491
E_FL_mxg-_12D07_F_0	BJ984492
E_FL_mxg-_12D10_F_0	BJ984493
E_FL_mxg-_12D13_F_0	BJ984494
E_FL_mxg-_12D15_F_0	BJ984495
E_FL_mxg-_12D16_F_0	BJ984496
E_FL_mxg-_12D18_F_0	BJ984497
E_FL_mxg-_12E01_F_0	BJ984498
E_FL_mxg-_12E03_F_0	BJ984499
E_FL_mxg-_12E04_F_0	BJ984500
E_FL_mxg-_12E06_F_0	BJ984501
E_FL_mxg-_12E07_F_0	BJ984502
E_FL_mxg-_12E08_F_0	BJ984503
E_FL_mxg-_12E11_F_0	BJ984504
E_FL_mxg-_12E12_F_0	BJ984505
E_FL_mxg-_12E14_F_0	BJ984506
E_FL_mxg-_12E15_F_0	BJ984507
E_FL_mxg-_12E17_F_0	BJ984508
E_FL_mxg-_12E20_F_0	BJ984509
E_FL_mxg-_12E21_F_0	BJ984510
E_FL_mxg-_12E22_F_0	BJ984511
E_FL_mxg-_12E23_F_0	BJ984512
E_FL_mxg-_12F04_F_0	BJ984513
E_FL_mxg-_12F05_F_0	BJ984514
E_FL_mxg-_12F07_F_0	BJ984515
E_FL_mxg-_12F08_F_0	BJ984516
E_FL_mxg-_12F09_F_0	BJ984517
E_FL_mxg-_12F13_F_0	BJ984518

E_FL_mxg-_12F15_F_0	BJ984519
E_FL_mxg-_12F17_F_0	BJ984520
E_FL_mxg-_12F22_F_0	BJ984521
E_FL_mxg-_12F23_F_0	BJ984522
E_FL_mxg-_12G01_F_0	BJ984523
E_FL_mxg-_12G02_F_0	BJ984524
E_FL_mxg-_12G11_F_0	BJ984525
E_FL_mxg-_12G16_F_0	BJ984526
E_FL_mxg-_12G17_F_0	BJ984527
E_FL_mxg-_12G19_F_0	BJ984528
E_FL_mxg-_12G20_F_0	BJ984529
E_FL_mxg-_12G22_F_0	BJ984530
E_FL_mxg-_12G24_F_0	BJ984531
E_FL_mxg-_12H02_F_0	BJ984532
E_FL_mxg-_12H05_F_0	BJ984533
E_FL_mxg-_12H06_F_0	BJ984534
E_FL_mxg-_12H11_F_0	BJ984535
E_FL_mxg-_12H13_F_0	BJ984536
E_FL_mxg-_12H23_F_0	BJ984537
E_FL_mxg-_12I01_F_0	BJ984538
E_FL_mxg-_12I02_F_0	BJ984539
E_FL_mxg-_12I04_F_0	BJ984540
E_FL_mxg-_12I06_F_0	BJ984541
E_FL_mxg-_12I07_F_0	BJ984542
E_FL_mxg-_12I09_F_0	BJ984543
E_FL_mxg-_12I10_F_0	BJ984544
E_FL_mxg-_12I15_F_0	BJ984545
E_FL_mxg-_12I18_F_0	BJ984546
E_FL_mxg-_12I19_F_0	BJ984547
E_FL_mxg-_12I21_F_0	BJ984548
E_FL_mxg-_12I23_F_0	BJ984549
E_FL_mxg-_12J01_F_0	BJ984550
E_FL_mxg-_12J02_F_0	BJ984551
E_FL_mxg-_12J03_F_0	BJ984552
E_FL_mxg-_12J04_F_0	BJ984553
E_FL_mxg-_12J05_F_0	BJ984554
E_FL_mxg-_12J07_F_0	BJ984555
E_FL_mxg-_12J09_F_0	BJ984556
E_FL_mxg-_12J10_F_0	BJ984557
E_FL_mxg-_12J11_F_0	BJ984558
E_FL_mxg-_12J12_F_0	BJ984559
E_FL_mxg-_12J13_F_0	BJ984560
E_FL_mxg-_12J14_F_0	BJ984561
E_FL_mxg-_12J17_F_0	BJ984562
E_FL_mxg-_12J19_F_0	BJ984563
E_FL_mxg-_12J21_F_0	BJ984564
E_FL_mxg-_12J22_F_0	BJ984565
E_FL_mxg-_12J23_F_0	BJ984566
E_FL_mxg-_12K01_F_0	BJ984567
E_FL_mxg-_12K03_F_0	BJ984568
E_FL_mxg-_12K05_F_0	BJ984569
E_FL_mxg-_12K07_F_0	BJ984570
E_FL_mxg-_12K08_F_0	BJ984571
E_FL_mxg-_12K11_F_0	BJ984572

E_FL_mxg-_12K12_F_0	BJ984573
E_FL_mxg-_12K13_F_0	BJ984574
E_FL_mxg-_12K14_F_0	BJ984575
E_FL_mxg-_12K17_F_0	BJ984576
E_FL_mxg-_12K18_F_0	BJ984577
E_FL_mxg-_12K21_F_0	BJ984578
E_FL_mxg-_12K22_F_0	BJ984579
E_FL_mxg-_12K23_F_0	BJ984580
E_FL_mxg-_12L02_F_0	BJ984581
E_FL_mxg-_12L03_F_0	BJ984582
E_FL_mxg-_12L06_F_0	BJ984583
E_FL_mxg-_12L08_F_0	BJ984584
E_FL_mxg-_12L14_F_0	BJ984585
E_FL_mxg-_12L16_F_0	BJ984586
E_FL_mxg-_12L17_F_0	BJ984587
E_FL_mxg-_12L20_F_0	BJ984588
E_FL_mxg-_12L22_F_0	BJ984589
E_FL_mxg-_12L24_F_0	BJ984590
E_FL_mxg-_12M03_F_0	BJ984591
E_FL_mxg-_12M04_F_0	BJ984592
E_FL_mxg-_12M05_F_0	BJ984593
E_FL_mxg-_12M06_F_0	BJ984594
E_FL_mxg-_12M07_F_0	BJ984595
E_FL_mxg-_12M09_F_0	BJ984596
E_FL_mxg-_12M12_F_0	BJ984597
E_FL_mxg-_12M13_F_0	BJ984598
E_FL_mxg-_12M17_F_0	BJ984599
E_FL_mxg-_12M19_F_0	BJ984600
E_FL_mxg-_12M21_F_0	BJ984601
E_FL_mxg-_12M23_F_0	BJ984602
E_FL_mxg-_12M24_F_0	BJ984603
E_FL_mxg-_12N02_F_0	BJ984604
E_FL_mxg-_12N05_F_0	BJ984605
E_FL_mxg-_12N06_F_0	BJ984606
E_FL_mxg-_12N08_F_0	BJ984607
E_FL_mxg-_12N09_F_0	BJ984608
E_FL_mxg-_12N11_F_0	BJ984609
E_FL_mxg-_12N12_F_0	BJ984610
E_FL_mxg-_12N13_F_0	BJ984611
E_FL_mxg-_12N15_F_0	BJ984612
E_FL_mxg-_12N16_F_0	BJ984613
E_FL_mxg-_12N17_F_0	BJ984614
E_FL_mxg-_12N20_F_0	BJ984615
E_FL_mxg-_12N21_F_0	BJ984616
E_FL_mxg-_12O01_F_0	BJ984617
E_FL_mxg-_12O05_F_0	BJ984618
E_FL_mxg-_12O07_F_0	BJ984619
E_FL_mxg-_12O12_F_0	BJ984620
E_FL_mxg-_12O15_F_0	BJ984621
E_FL_mxg-_12O18_F_0	BJ984622
E_FL_mxg-_12O20_F_0	BJ984623
E_FL_mxg-_12O24_F_0	BJ984624
E_FL_mxg-_12P02_F_0	BJ984625
E_FL_mxg-_12P05_F_0	BJ984626

E_FL_mxg-_12P11_F_0	BJ984627
E_FL_mxg-_12P12_F_0	BJ984628
E_FL_mxg-_12P13_F_0	BJ984629
E_FL_mxg-_12P16_F_0	BJ984630
E_FL_mxg-_12P20_F_0	BJ984631
E_FL_mxg-_12P22_F_0	BJ984632
E_FL_mxg-_12P23_F_0	BJ984633
E_FL_mxg-_13A06_F_0	BJ984634
E_FL_mxg-_13A12_F_0	BJ984635
E_FL_mxg-_13A14_F_0	BJ984636
E_FL_mxg-_13A17_F_0	BJ984637
E_FL_mxg-_13A19_F_0	BJ984638
E_FL_mxg-_13A20_F_0	BJ984639
E_FL_mxg-_13B02_F_0	BJ984640
E_FL_mxg-_13B04_F_0	BJ984641
E_FL_mxg-_13B07_F_0	BJ984642
E_FL_mxg-_13B08_F_0	BJ984643
E_FL_mxg-_13B11_F_0	BJ984644
E_FL_mxg-_13B14_F_0	BJ984645
E_FL_mxg-_13B15_F_0	BJ984646
E_FL_mxg-_13B17_F_0	BJ984647
E_FL_mxg-_13B18_F_0	BJ984648
E_FL_mxg-_13B19_F_0	BJ984649
E_FL_mxg-_13B22_F_0	BJ984650
E_FL_mxg-_13B23_F_0	BJ984651
E_FL_mxg-_13B24_F_0	BJ984652
E_FL_mxg-_13C01_F_0	BJ984653
E_FL_mxg-_13C03_F_0	BJ984654
E_FL_mxg-_13C04_F_0	BJ984655
E_FL_mxg-_13C07_F_0	BJ984656
E_FL_mxg-_13C09_F_0	BJ984657
E_FL_mxg-_13C14_F_0	BJ984658
E_FL_mxg-_13C16_F_0	BJ984659
E_FL_mxg-_13C17_F_0	BJ984660
E_FL_mxg-_13C18_F_0	BJ984661
E_FL_mxg-_13C23_F_0	BJ984662
E_FL_mxg-_13D01_F_0	BJ984663
E_FL_mxg-_13D02_F_0	BJ984664
E_FL_mxg-_13D04_F_0	BJ984665
E_FL_mxg-_13D05_F_0	BJ984666
E_FL_mxg-_13D07_F_0	BJ984667
E_FL_mxg-_13D10_F_0	BJ984668
E_FL_mxg-_13D20_F_0	BJ984669
E_FL_mxg-_13D21_F_0	BJ984670
E_FL_mxg-_13D23_F_0	BJ984671
E_FL_mxg-_13E02_F_0	BJ984672
E_FL_mxg-_13E12_F_0	BJ984673
E_FL_mxg-_13E14_F_0	BJ984674
E_FL_mxg-_13E16_F_0	BJ984675
E_FL_mxg-_13E18_F_0	BJ984676
E_FL_mxg-_13E20_F_0	BJ984677
E_FL_mxg-_13E22_F_0	BJ984678
E_FL_mxg-_13F02_F_0	BJ984679
E_FL_mxg-_13F03_F_0	BJ984680

E_FL_mxg-_13F08_F_0	BJ984681
E_FL_mxg-_13F10_F_0	BJ984682
E_FL_mxg-_13F12_F_0	BJ984683
E_FL_mxg-_13F13_F_0	BJ984684
E_FL_mxg-_13F15_F_0	BJ984685
E_FL_mxg-_13F21_F_0	BJ984686
E_FL_mxg-_13F22_F_0	BJ984687
E_FL_mxg-_13F23_F_0	BJ984688
E_FL_mxg-_13F24_F_0	BJ984689
E_FL_mxg-_13G02_F_0	BJ984690
E_FL_mxg-_13G03_F_0	BJ984691
E_FL_mxg-_13G04_F_0	BJ984692
E_FL_mxg-_13G05_F_0	BJ984693
E_FL_mxg-_13G08_F_0	BJ984694
E_FL_mxg-_13G10_F_0	BJ984695
E_FL_mxg-_13G12_F_0	BJ984696
E_FL_mxg-_13G15_F_0	BJ984697
E_FL_mxg-_13G16_F_0	BJ984698
E_FL_mxg-_13G19_F_0	BJ984699
E_FL_mxg-_13G21_F_0	BJ984700
E_FL_mxg-_13G22_F_0	BJ984701
E_FL_mxg-_13H01_F_0	BJ984702
E_FL_mxg-_13H04_F_0	BJ984703
E_FL_mxg-_13H09_F_0	BJ984704
E_FL_mxg-_13H10_F_0	BJ984705
E_FL_mxg-_13H11_F_0	BJ984706
E_FL_mxg-_13H12_F_0	BJ984707
E_FL_mxg-_13H14_F_0	BJ984708
E_FL_mxg-_13H15_F_0	BJ984709
E_FL_mxg-_13H19_F_0	BJ984710
E_FL_mxg-_13H24_F_0	BJ984711
E_FL_mxg-_13I02_F_0	BJ984712
E_FL_mxg-_13I03_F_0	BJ984713
E_FL_mxg-_13I06_F_0	BJ984714
E_FL_mxg-_13I07_F_0	BJ984715
E_FL_mxg-_13I08_F_0	BJ984716
E_FL_mxg-_13I09_F_0	BJ984717
E_FL_mxg-_13I13_F_0	BJ984718
E_FL_mxg-_13I14_F_0	BJ984719
E_FL_mxg-_13I15_F_0	BJ984720
E_FL_mxg-_13I16_F_0	BJ984721
E_FL_mxg-_13I24_F_0	BJ984722
E_FL_mxg-_13J01_F_0	BJ984723
E_FL_mxg-_13J02_F_0	BJ984724
E_FL_mxg-_13J03_F_0	BJ984725
E_FL_mxg-_13J04_F_0	BJ984726
E_FL_mxg-_13J06_F_0	BJ984727
E_FL_mxg-_13J08_F_0	BJ984728
E_FL_mxg-_13J12_F_0	BJ984729
E_FL_mxg-_13J13_F_0	BJ984730
E_FL_mxg-_13J14_F_0	BJ984731
E_FL_mxg-_13J16_F_0	BJ984732
E_FL_mxg-_13J18_F_0	BJ984733
E_FL_mxg-_13J19_F_0	BJ984734

E_FL_mxg-_13J24_F_0	BJ984735
E_FL_mxg-_13K04_F_0	BJ984736
E_FL_mxg-_13K06_F_0	BJ984737
E_FL_mxg-_13K07_F_0	BJ984738
E_FL_mxg-_13K11_F_0	BJ984739
E_FL_mxg-_13K17_F_0	BJ984740
E_FL_mxg-_13K18_F_0	BJ984741
E_FL_mxg-_13K21_F_0	BJ984742
E_FL_mxg-_13K22_F_0	BJ984743
E_FL_mxg-_13K24_F_0	BJ984744
E_FL_mxg-_13L05_F_0	BJ984745
E_FL_mxg-_13L06_F_0	BJ984746
E_FL_mxg-_13L08_F_0	BJ984747
E_FL_mxg-_13L18_F_0	BJ984748
E_FL_mxg-_13L24_F_0	BJ984749
E_FL_mxg-_13M02_F_0	BJ984750
E_FL_mxg-_13M08_F_0	BJ984751
E_FL_mxg-_13M09_F_0	BJ984752
E_FL_mxg-_13M13_F_0	BJ984753
E_FL_mxg-_13M14_F_0	BJ984754
E_FL_mxg-_13M16_F_0	BJ984755
E_FL_mxg-_13M17_F_0	BJ984756
E_FL_mxg-_13M18_F_0	BJ984757
E_FL_mxg-_13M21_F_0	BJ984758
E_FL_mxg-_13M22_F_0	BJ984759
E_FL_mxg-_13M23_F_0	BJ984760
E_FL_mxg-_13M24_F_0	BJ984761
E_FL_mxg-_13N01_F_0	BJ984762
E_FL_mxg-_13N02_F_0	BJ984763
E_FL_mxg-_13N04_F_0	BJ984764
E_FL_mxg-_13N05_F_0	BJ984765
E_FL_mxg-_13N06_F_0	BJ984766
E_FL_mxg-_13N07_F_0	BJ984767
E_FL_mxg-_13N08_F_0	BJ984768
E_FL_mxg-_13N10_F_0	BJ984769
E_FL_mxg-_13N13_F_0	BJ984770
E_FL_mxg-_13N16_F_0	BJ984771
E_FL_mxg-_13N17_F_0	BJ984772
E_FL_mxg-_13N20_F_0	BJ984773
E_FL_mxg-_13N21_F_0	BJ984774
E_FL_mxg-_13N22_F_0	BJ984775
E_FL_mxg-_13O02_F_0	BJ984776
E_FL_mxg-_13O05_F_0	BJ984777
E_FL_mxg-_13O07_F_0	BJ984778
E_FL_mxg-_13O11_F_0	BJ984779
E_FL_mxg-_13O12_F_0	BJ984780
E_FL_mxg-_13O13_F_0	BJ984781
E_FL_mxg-_13O14_F_0	BJ984782
E_FL_mxg-_13O17_F_0	BJ984783
E_FL_mxg-_13O20_F_0	BJ984784
E_FL_mxg-_13O21_F_0	BJ984785
E_FL_mxg-_13O23_F_0	BJ984786
E_FL_mxg-_13O24_F_0	BJ984787
E_FL_mxg-_13P02_F_0	BJ984788

E_FL_mxg-_13P03_F_0	BJ984789
E_FL_mxg-_13P04_F_0	BJ984790
E_FL_mxg-_13P05_F_0	BJ984791
E_FL_mxg-_13P08_F_0	BJ984792
E_FL_mxg-_13P09_F_0	BJ984793
E_FL_mxg-_13P10_F_0	BJ984794
E_FL_mxg-_13P13_F_0	BJ984795
E_FL_mxg-_13P15_F_0	BJ984796
E_FL_mxg-_13P17_F_0	BJ984797
E_FL_mxg-_13P18_F_0	BJ984798
E_FL_mxg-_13P21_F_0	BJ984799
E_FL_mxg-_14A05_F_0	BJ984800
E_FL_mxg-_14A06_F_0	BJ984801
E_FL_mxg-_14A08_F_0	BJ984802
E_FL_mxg-_14A09_F_0	BJ984803
E_FL_mxg-_14A11_F_0	BJ984804
E_FL_mxg-_14A15_F_0	BJ984805
E_FL_mxg-_14A16_F_0	BJ984806
E_FL_mxg-_14B02_F_0	BJ984807
E_FL_mxg-_14B06_F_0	BJ984808
E_FL_mxg-_14B08_F_0	BJ984809
E_FL_mxg-_14B09_F_0	BJ984810
E_FL_mxg-_14B16_F_0	BJ984811
E_FL_mxg-_14B17_F_0	BJ984812
E_FL_mxg-_14B23_F_0	BJ984813
E_FL_mxg-_14B24_F_0	BJ984814
E_FL_mxg-_14C03_F_0	BJ984815
E_FL_mxg-_14C06_F_0	BJ984816
E_FL_mxg-_14C07_F_0	BJ984817
E_FL_mxg-_14C09_F_0	BJ984818
E_FL_mxg-_14C10_F_0	BJ984819
E_FL_mxg-_14C13_F_0	BJ984820
E_FL_mxg-_14C18_F_0	BJ984821
E_FL_mxg-_14C23_F_0	BJ984822
E_FL_mxg-_14D02_F_0	BJ984823
E_FL_mxg-_14D03_F_0	BJ984824
E_FL_mxg-_14D06_F_0	BJ984825
E_FL_mxg-_14D07_F_0	BJ984826
E_FL_mxg-_14D09_F_0	BJ984827
E_FL_mxg-_14D11_F_0	BJ984828
E_FL_mxg-_14D12_F_0	BJ984829
E_FL_mxg-_14D13_F_0	BJ984830
E_FL_mxg-_14D14_F_0	BJ984831
E_FL_mxg-_14D17_F_0	BJ984832
E_FL_mxg-_14D24_F_0	BJ984833
E_FL_mxg-_14E02_F_0	BJ984834
E_FL_mxg-_14E03_F_0	BJ984835
E_FL_mxg-_14E05_F_0	BJ984836
E_FL_mxg-_14E06_F_0	BJ984837
E_FL_mxg-_14E07_F_0	BJ984838
E_FL_mxg-_14E08_F_0	BJ984839
E_FL_mxg-_14E12_F_0	BJ984840
E_FL_mxg-_14E14_F_0	BJ984841
E_FL_mxg-_14E15_F_0	BJ984842

E_FL_mxg-_14E16_F_0	BJ984843
E_FL_mxg-_14E20_F_0	BJ984844
E_FL_mxg-_14E24_F_0	BJ984845
E_FL_mxg-_14F01_F_0	BJ984846
E_FL_mxg-_14F02_F_0	BJ984847
E_FL_mxg-_14F05_F_0	BJ984848
E_FL_mxg-_14F06_F_0	BJ984849
E_FL_mxg-_14F07_F_0	BJ984850
E_FL_mxg-_14F11_F_0	BJ984851
E_FL_mxg-_14F15_F_0	BJ984852
E_FL_mxg-_14F19_F_0	BJ984853
E_FL_mxg-_14G03_F_0	BJ984854
E_FL_mxg-_14G08_F_0	BJ984855
E_FL_mxg-_14G09_F_0	BJ984856
E_FL_mxg-_14G10_F_0	BJ984857
E_FL_mxg-_14G11_F_0	BJ984858
E_FL_mxg-_14G17_F_0	BJ984859
E_FL_mxg-_14G18_F_0	BJ984860
E_FL_mxg-_14G19_F_0	BJ984861
E_FL_mxg-_14G21_F_0	BJ984862
E_FL_mxg-_14G24_F_0	BJ984863
E_FL_mxg-_14H04_F_0	BJ984864
E_FL_mxg-_14H05_F_0	BJ984865
E_FL_mxg-_14H11_F_0	BJ984866
E_FL_mxg-_14H13_F_0	BJ984867
E_FL_mxg-_14H16_F_0	BJ984868
E_FL_mxg-_14H17_F_0	BJ984869
E_FL_mxg-_14H20_F_0	BJ984870
E_FL_mxg-_14H23_F_0	BJ984871
E_FL_mxg-_14I03_F_0	BJ984872
E_FL_mxg-_14I04_F_0	BJ984873
E_FL_mxg-_14I06_F_0	BJ984874
E_FL_mxg-_14I07_F_0	BJ984875
E_FL_mxg-_14I08_F_0	BJ984876
E_FL_mxg-_14I10_F_0	BJ984877
E_FL_mxg-_14I11_F_0	BJ984878
E_FL_mxg-_14I12_F_0	BJ984879
E_FL_mxg-_14I13_F_0	BJ984880
E_FL_mxg-_14I17_F_0	BJ984881
E_FL_mxg-_14I19_F_0	BJ984882
E_FL_mxg-_14I20_F_0	BJ984883
E_FL_mxg-_14I22_F_0	BJ984884
E_FL_mxg-_14I24_F_0	BJ984885
E_FL_mxg-_14J04_F_0	BJ984886
E_FL_mxg-_14J08_F_0	BJ984887
E_FL_mxg-_14J09_F_0	BJ984888
E_FL_mxg-_14J10_F_0	BJ984889
E_FL_mxg-_14J11_F_0	BJ984890
E_FL_mxg-_14J13_F_0	BJ984891
E_FL_mxg-_14J14_F_0	BJ984892
E_FL_mxg-_14J15_F_0	BJ984893
E_FL_mxg-_14J17_F_0	BJ984894
E_FL_mxg-_14J18_F_0	BJ984895
E_FL_mxg-_14J19_F_0	BJ984896

E_FL_mxg-_14J21_F_0	BJ984897
E_FL_mxg-_14K04_F_0	BJ984898
E_FL_mxg-_14K07_F_0	BJ984899
E_FL_mxg-_14K08_F_0	BJ984900
E_FL_mxg-_14K09_F_0	BJ984901
E_FL_mxg-_14K10_F_0	BJ984902
E_FL_mxg-_14K11_F_0	BJ984903
E_FL_mxg-_14K12_F_0	BJ984904
E_FL_mxg-_14K13_F_0	BJ984905
E_FL_mxg-_14K14_F_0	BJ984906
E_FL_mxg-_14K15_F_0	BJ984907
E_FL_mxg-_14K17_F_0	BJ984908
E_FL_mxg-_14K18_F_0	BJ984909
E_FL_mxg-_14K19_F_0	BJ984910
E_FL_mxg-_14K20_F_0	BJ984911
E_FL_mxg-_14K21_F_0	BJ984912
E_FL_mxg-_14K23_F_0	BJ984913
E_FL_mxg-_14K24_F_0	BJ984914
E_FL_mxg-_14L03_F_0	BJ984915
E_FL_mxg-_14L04_F_0	BJ984916
E_FL_mxg-_14L10_F_0	BJ984917
E_FL_mxg-_14L12_F_0	BJ984918
E_FL_mxg-_14L16_F_0	BJ984919
E_FL_mxg-_14L18_F_0	BJ984920
E_FL_mxg-_14L20_F_0	BJ984921
E_FL_mxg-_14M01_F_0	BJ984922
E_FL_mxg-_14M03_F_0	BJ984923
E_FL_mxg-_14M08_F_0	BJ984924
E_FL_mxg-_14M09_F_0	BJ984925
E_FL_mxg-_14M12_F_0	BJ984926
E_FL_mxg-_14M13_F_0	BJ984927
E_FL_mxg-_14M15_F_0	BJ984928
E_FL_mxg-_14M17_F_0	BJ984929
E_FL_mxg-_14M18_F_0	BJ984930
E_FL_mxg-_14M19_F_0	BJ984931
E_FL_mxg-_14M22_F_0	BJ984932
E_FL_mxg-_14N06_F_0	BJ984933
E_FL_mxg-_14N07_F_0	BJ984934
E_FL_mxg-_14N11_F_0	BJ984935
E_FL_mxg-_14N16_F_0	BJ984936
E_FL_mxg-_14N17_F_0	BJ984937
E_FL_mxg-_14N20_F_0	BJ984938
E_FL_mxg-_14N21_F_0	BJ984939
E_FL_mxg-_14N23_F_0	BJ984940
E_FL_mxg-_14N24_F_0	BJ984941
E_FL_mxg-_14O03_F_0	BJ984942
E_FL_mxg-_14O08_F_0	BJ984943
E_FL_mxg-_14O13_F_0	BJ984944
E_FL_mxg-_14O14_F_0	BJ984945
E_FL_mxg-_14O16_F_0	BJ984946
E_FL_mxg-_14O18_F_0	BJ984947
E_FL_mxg-_14O19_F_0	BJ984948
E_FL_mxg-_14O20_F_0	BJ984949
E_FL_mxg-_14O23_F_0	BJ984950

E_FL_mxg-_14O24_F_0	BJ984951
E_FL_mxg-_14P17_F_0	BJ984952
E_FL_mxg-_14P19_F_0	BJ984953
E_FL_mxg-_14P20_F_0	BJ984954
E_FL_mxg-_14P21_F_0	BJ984955
E_FL_mxg-_14P23_F_0	BJ984956
E_FL_mxg-_15A01_F_0	BJ984957
E_FL_mxg-_15A06_F_0	BJ984958
E_FL_mxg-_15A16_F_0	BJ984959
E_FL_mxg-_15A18_F_0	BJ984960
E_FL_mxg-_15A22_F_0	BJ984961
E_FL_mxg-_15A23_F_0	BJ984962
E_FL_mxg-_15B02_F_0	BJ984963
E_FL_mxg-_15B08_F_0	BJ984964
E_FL_mxg-_15B09_F_0	BJ984965
E_FL_mxg-_15B11_F_0	BJ984966
E_FL_mxg-_15B13_F_0	BJ984967
E_FL_mxg-_15B15_F_0	BJ984968
E_FL_mxg-_15B18_F_0	BJ984969
E_FL_mxg-_15B19_F_0	BJ984970
E_FL_mxg-_15B20_F_0	BJ984971
E_FL_mxg-_15C03_F_0	BJ984972
E_FL_mxg-_15C04_F_0	BJ984973
E_FL_mxg-_15C05_F_0	BJ984974
E_FL_mxg-_15C06_F_0	BJ984975
E_FL_mxg-_15C11_F_0	BJ984976
E_FL_mxg-_15C12_F_0	BJ984977
E_FL_mxg-_15C17_F_0	BJ984978
E_FL_mxg-_15C20_F_0	BJ984979
E_FL_mxg-_15C22_F_0	BJ984980
E_FL_mxg-_15C23_F_0	BJ984981
E_FL_mxg-_15C24_F_0	BJ984982
E_FL_mxg-_15D01_F_0	BJ984983
E_FL_mxg-_15D02_F_0	BJ984984
E_FL_mxg-_15D03_F_0	BJ984985
E_FL_mxg-_15D04_F_0	BJ984986
E_FL_mxg-_15D05_F_0	BJ984987
E_FL_mxg-_15D07_F_0	BJ984988
E_FL_mxg-_15D09_F_0	BJ984989
E_FL_mxg-_15D10_F_0	BJ984990
E_FL_mxg-_15D11_F_0	BJ984991
E_FL_mxg-_15D12_F_0	BJ984992
E_FL_mxg-_15D13_F_0	BJ984993
E_FL_mxg-_15D17_F_0	BJ984994
E_FL_mxg-_15D19_F_0	BJ984995
E_FL_mxg-_15D20_F_0	BJ984996
E_FL_mxg-_15D21_F_0	BJ984997
E_FL_mxg-_15D23_F_0	BJ984998
E_FL_mxg-_15D24_F_0	BJ984999
E_FL_mxg-_15E03_F_0	BJ985000
E_FL_mxg-_15E04_F_0	BJ985001
E_FL_mxg-_15E09_F_0	BJ985002
E_FL_mxg-_15E10_F_0	BJ985003
E_FL_mxg-_15E12_F_0	BJ985004

E_FL_mxg-_15E16_F_0	BJ985005
E_FL_mxg-_15E17_F_0	BJ985006
E_FL_mxg-_15E18_F_0	BJ985007
E_FL_mxg-_15E22_F_0	BJ985008
E_FL_mxg-_15E24_F_0	BJ985009
E_FL_mxg-_15F03_F_0	BJ985010
E_FL_mxg-_15F06_F_0	BJ985011
E_FL_mxg-_15F07_F_0	BJ985012
E_FL_mxg-_15F09_F_0	BJ985013
E_FL_mxg-_15F16_F_0	BJ985014
E_FL_mxg-_15F18_F_0	BJ985015
E_FL_mxg-_15F19_F_0	BJ985016
E_FL_mxg-_15F20_F_0	BJ985017
E_FL_mxg-_15F22_F_0	BJ985018
E_FL_mxg-_15F23_F_0	BJ985019
E_FL_mxg-_15F24_F_0	BJ985020
E_FL_mxg-_15G03_F_0	BJ985021
E_FL_mxg-_15G04_F_0	BJ985022
E_FL_mxg-_15G05_F_0	BJ985023
E_FL_mxg-_15G06_F_0	BJ985024
E_FL_mxg-_15G09_F_0	BJ985025
E_FL_mxg-_15G10_F_0	BJ985026
E_FL_mxg-_15G12_F_0	BJ985027
E_FL_mxg-_15G13_F_0	BJ985028
E_FL_mxg-_15G16_F_0	BJ985029
E_FL_mxg-_15G17_F_0	BJ985030
E_FL_mxg-_15G18_F_0	BJ985031
E_FL_mxg-_15G19_F_0	BJ985032
E_FL_mxg-_15G20_F_0	BJ985033
E_FL_mxg-_15G23_F_0	BJ985034
E_FL_mxg-_15G24_F_0	BJ985035
E_FL_mxg-_15H01_F_0	BJ985036
E_FL_mxg-_15H03_F_0	BJ985037
E_FL_mxg-_15H07_F_0	BJ985038
E_FL_mxg-_15H08_F_0	BJ985039
E_FL_mxg-_15H09_F_0	BJ985040
E_FL_mxg-_15H10_F_0	BJ985041
E_FL_mxg-_15H12_F_0	BJ985042
E_FL_mxg-_15H14_F_0	BJ985043
E_FL_mxg-_15H19_F_0	BJ985044
E_FL_mxg-_15H22_F_0	BJ985045
E_FL_mxg-_15H23_F_0	BJ985046
E_FL_mxg-_15I02_F_0	BJ985047
E_FL_mxg-_15I05_F_0	BJ985048
E_FL_mxg-_15I06_F_0	BJ985049
E_FL_mxg-_15I07_F_0	BJ985050
E_FL_mxg-_15I08_F_0	BJ985051
E_FL_mxg-_15I19_F_0	BJ985052
E_FL_mxg-_15I20_F_0	BJ985053
E_FL_mxg-_15I23_F_0	BJ985054
E_FL_mxg-_15J05_F_0	BJ985055
E_FL_mxg-_15J06_F_0	BJ985056
E_FL_mxg-_15J09_F_0	BJ985057
E_FL_mxg-_15J11_F_0	BJ985058

E_FL_mxg-_15J14_F_0	BJ985059
E_FL_mxg-_15J17_F_0	BJ985060
E_FL_mxg-_15J18_F_0	BJ985061
E_FL_mxg-_15K01_F_0	BJ985062
E_FL_mxg-_15K02_F_0	BJ985063
E_FL_mxg-_15K04_F_0	BJ985064
E_FL_mxg-_15K05_F_0	BJ985065
E_FL_mxg-_15K06_F_0	BJ985066
E_FL_mxg-_15K08_F_0	BJ985067
E_FL_mxg-_15K12_F_0	BJ985068
E_FL_mxg-_15K14_F_0	BJ985069
E_FL_mxg-_15K15_F_0	BJ985070
E_FL_mxg-_15K16_F_0	BJ985071
E_FL_mxg-_15K17_F_0	BJ985072
E_FL_mxg-_15K19_F_0	BJ985073
E_FL_mxg-_15K24_F_0	BJ985074
E_FL_mxg-_15L01_F_0	BJ985075
E_FL_mxg-_15L02_F_0	BJ985076
E_FL_mxg-_15L04_F_0	BJ985077
E_FL_mxg-_15L08_F_0	BJ985078
E_FL_mxg-_15L10_F_0	BJ985079
E_FL_mxg-_15L11_F_0	BJ985080
E_FL_mxg-_15L14_F_0	BJ985081
E_FL_mxg-_15L15_F_0	BJ985082
E_FL_mxg-_15L16_F_0	BJ985083
E_FL_mxg-_15L17_F_0	BJ985084
E_FL_mxg-_15L24_F_0	BJ985085
E_FL_mxg-_15M01_F_0	BJ985086
E_FL_mxg-_15M02_F_0	BJ985087
E_FL_mxg-_15M04_F_0	BJ985088
E_FL_mxg-_15M05_F_0	BJ985089
E_FL_mxg-_15M07_F_0	BJ985090
E_FL_mxg-_15M08_F_0	BJ985091
E_FL_mxg-_15M09_F_0	BJ985092
E_FL_mxg-_15M11_F_0	BJ985093
E_FL_mxg-_15M12_F_0	BJ985094
E_FL_mxg-_15M17_F_0	BJ985095
E_FL_mxg-_15M20_F_0	BJ985096
E_FL_mxg-_15M22_F_0	BJ985097
E_FL_mxg-_15M23_F_0	BJ985098
E_FL_mxg-_15M24_F_0	BJ985099
E_FL_mxg-_15N02_F_0	BJ985100
E_FL_mxg-_15N03_F_0	BJ985101
E_FL_mxg-_15N04_F_0	BJ985102
E_FL_mxg-_15N08_F_0	BJ985103
E_FL_mxg-_15N10_F_0	BJ985104
E_FL_mxg-_15N11_F_0	BJ985105
E_FL_mxg-_15N16_F_0	BJ985106
E_FL_mxg-_15N17_F_0	BJ985107
E_FL_mxg-_15N19_F_0	BJ985108
E_FL_mxg-_15O04_F_0	BJ985109
E_FL_mxg-_15O06_F_0	BJ985110
E_FL_mxg-_15O08_F_0	BJ985111
E_FL_mxg-_15O11_F_0	BJ985112

E_FL_mxg-_15O12_F_0	BJ985113
E_FL_mxg-_15O13_F_0	BJ985114
E_FL_mxg-_15O15_F_0	BJ985115
E_FL_mxg-_15O18_F_0	BJ985116
E_FL_mxg-_15O19_F_0	BJ985117
E_FL_mxg-_15O23_F_0	BJ985118
E_FL_mxg-_15P02_F_0	BJ985119
E_FL_mxg-_15P07_F_0	BJ985120
E_FL_mxg-_15P08_F_0	BJ985121
E_FL_mxg-_15P10_F_0	BJ985122
E_FL_mxg-_15P14_F_0	BJ985123
E_FL_mxg-_15P18_F_0	BJ985124
E_FL_mxg-_15P19_F_0	BJ985125
E_FL_mxg-_15P21_F_0	BJ985126
E_FL_mxg-_16A03_F_0	BJ985127
E_FL_mxg-_16A04_F_0	BJ985128
E_FL_mxg-_16A07_F_0	BJ985129
E_FL_mxg-_16A08_F_0	BJ985130
E_FL_mxg-_16A09_F_0	BJ985131
E_FL_mxg-_16A12_F_0	BJ985132
E_FL_mxg-_16A13_F_0	BJ985133
E_FL_mxg-_16A15_F_0	BJ985134
E_FL_mxg-_16A16_F_0	BJ985135
E_FL_mxg-_16A17_F_0	BJ985136
E_FL_mxg-_16A18_F_0	BJ985137
E_FL_mxg-_16A20_F_0	BJ985138
E_FL_mxg-_16A23_F_0	BJ985139
E_FL_mxg-_16A24_F_0	BJ985140
E_FL_mxg-_16B01_F_0	BJ985141
E_FL_mxg-_16B02_F_0	BJ985142
E_FL_mxg-_16B10_F_0	BJ985143
E_FL_mxg-_16B11_F_0	BJ985144
E_FL_mxg-_16B14_F_0	BJ985145
E_FL_mxg-_16B18_F_0	BJ985146
E_FL_mxg-_16B20_F_0	BJ985147
E_FL_mxg-_16B21_F_0	BJ985148
E_FL_mxg-_16B22_F_0	BJ985149
E_FL_mxg-_16B23_F_0	BJ985150
E_FL_mxg-_16B24_F_0	BJ985151
E_FL_mxg-_16C06_F_0	BJ985152
E_FL_mxg-_16C07_F_0	BJ985153
E_FL_mxg-_16C08_F_0	BJ985154
E_FL_mxg-_16C09_F_0	BJ985155
E_FL_mxg-_16C12_F_0	BJ985156
E_FL_mxg-_16C16_F_0	BJ985157
E_FL_mxg-_16C20_F_0	BJ985158
E_FL_mxg-_16C21_F_0	BJ985159
E_FL_mxg-_16C23_F_0	BJ985160
E_FL_mxg-_16D03_F_0	BJ985161
E_FL_mxg-_16D05_F_0	BJ985162
E_FL_mxg-_16D08_F_0	BJ985163
E_FL_mxg-_16D09_F_0	BJ985164
E_FL_mxg-_16D10_F_0	BJ985165
E_FL_mxg-_16D11_F_0	BJ985166

E_FL_mxg-_16D12_F_0	BJ985167
E_FL_mxg-_16D15_F_0	BJ985168
E_FL_mxg-_16D16_F_0	BJ985169
E_FL_mxg-_16D17_F_0	BJ985170
E_FL_mxg-_16D18_F_0	BJ985171
E_FL_mxg-_16D20_F_0	BJ985172
E_FL_mxg-_16D21_F_0	BJ985173
E_FL_mxg-_16E01_F_0	BJ985174
E_FL_mxg-_16E02_F_0	BJ985175
E_FL_mxg-_16E08_F_0	BJ985176
E_FL_mxg-_16E09_F_0	BJ985177
E_FL_mxg-_16E11_F_0	BJ985178
E_FL_mxg-_16E12_F_0	BJ985179
E_FL_mxg-_16E17_F_0	BJ985180
E_FL_mxg-_16E20_F_0	BJ985181
E_FL_mxg-_16E22_F_0	BJ985182
E_FL_mxg-_16E23_F_0	BJ985183
E_FL_mxg-_16F01_F_0	BJ985184
E_FL_mxg-_16F03_F_0	BJ985185
E_FL_mxg-_16F06_F_0	BJ985186
E_FL_mxg-_16F08_F_0	BJ985187
E_FL_mxg-_16F11_F_0	BJ985188
E_FL_mxg-_16F12_F_0	BJ985189
E_FL_mxg-_16F14_F_0	BJ985190
E_FL_mxg-_16F15_F_0	BJ985191
E_FL_mxg-_16F16_F_0	BJ985192
E_FL_mxg-_16F20_F_0	BJ985193
E_FL_mxg-_16F22_F_0	BJ985194
E_FL_mxg-_16F24_F_0	BJ985195
E_FL_mxg-_16G01_F_0	BJ985196
E_FL_mxg-_16G02_F_0	BJ985197
E_FL_mxg-_16G04_F_0	BJ985198
E_FL_mxg-_16G05_F_0	BJ985199
E_FL_mxg-_16G06_F_0	BJ985200
E_FL_mxg-_16G08_F_0	BJ985201
E_FL_mxg-_16G12_F_0	BJ985202
E_FL_mxg-_16G13_F_0	BJ985203
E_FL_mxg-_16G14_F_0	BJ985204
E_FL_mxg-_16G16_F_0	BJ985205
E_FL_mxg-_16G18_F_0	BJ985206
E_FL_mxg-_16G19_F_0	BJ985207
E_FL_mxg-_16G21_F_0	BJ985208
E_FL_mxg-_16G23_F_0	BJ985209
E_FL_mxg-_16G24_F_0	BJ985210
E_FL_mxg-_16H01_F_0	BJ985211
E_FL_mxg-_16H02_F_0	BJ985212
E_FL_mxg-_16H05_F_0	BJ985213
E_FL_mxg-_16H06_F_0	BJ985214
E_FL_mxg-_16H07_F_0	BJ985215
E_FL_mxg-_16H10_F_0	BJ985216
E_FL_mxg-_16H11_F_0	BJ985217
E_FL_mxg-_16H12_F_0	BJ985218
E_FL_mxg-_16H13_F_0	BJ985219
E_FL_mxg-_16H16_F_0	BJ985220

E_FL_mxg-_16H17_F_0	BJ985221
E_FL_mxg-_16H18_F_0	BJ985222
E_FL_mxg-_16H19_F_0	BJ985223
E_FL_mxg-_16H20_F_0	BJ985224
E_FL_mxg-_16H22_F_0	BJ985225
E_FL_mxg-_16H23_F_0	BJ985226
E_FL_mxg-_16I01_F_0	BJ985227
E_FL_mxg-_16I04_F_0	BJ985228
E_FL_mxg-_16I07_F_0	BJ985229
E_FL_mxg-_16I10_F_0	BJ985230
E_FL_mxg-_16I11_F_0	BJ985231
E_FL_mxg-_16I12_F_0	BJ985232
E_FL_mxg-_16I13_F_0	BJ985233
E_FL_mxg-_16I14_F_0	BJ985234
E_FL_mxg-_16I20_F_0	BJ985235
E_FL_mxg-_16I21_F_0	BJ985236
E_FL_mxg-_16I22_F_0	BJ985237
E_FL_mxg-_16J03_F_0	BJ985238
E_FL_mxg-_16J04_F_0	BJ985239
E_FL_mxg-_16J06_F_0	BJ985240
E_FL_mxg-_16J07_F_0	BJ985241
E_FL_mxg-_16J08_F_0	BJ985242
E_FL_mxg-_16J12_F_0	BJ985243
E_FL_mxg-_16J13_F_0	BJ985244
E_FL_mxg-_16J17_F_0	BJ985245
E_FL_mxg-_16J19_F_0	BJ985246
E_FL_mxg-_16J20_F_0	BJ985247
E_FL_mxg-_16J21_F_0	BJ985248
E_FL_mxg-_16J24_F_0	BJ985249
E_FL_mxg-_16K04_F_0	BJ985250
E_FL_mxg-_16K05_F_0	BJ985251
E_FL_mxg-_16K06_F_0	BJ985252
E_FL_mxg-_16K07_F_0	BJ985253
E_FL_mxg-_16K10_F_0	BJ985254
E_FL_mxg-_16K12_F_0	BJ985255
E_FL_mxg-_16K15_F_0	BJ985256
E_FL_mxg-_16K16_F_0	BJ985257
E_FL_mxg-_16K19_F_0	BJ985258
E_FL_mxg-_16K21_F_0	BJ985259
E_FL_mxg-_16K22_F_0	BJ985260
E_FL_mxg-_16K23_F_0	BJ985261
E_FL_mxg-_16L01_F_0	BJ985262
E_FL_mxg-_16L02_F_0	BJ985263
E_FL_mxg-_16L05_F_0	BJ985264
E_FL_mxg-_16L06_F_0	BJ985265
E_FL_mxg-_16L09_F_0	BJ985266
E_FL_mxg-_16L11_F_0	BJ985267
E_FL_mxg-_16L14_F_0	BJ985268
E_FL_mxg-_16L16_F_0	BJ985269
E_FL_mxg-_16L21_F_0	BJ985270
E_FL_mxg-_16L23_F_0	BJ985271
E_FL_mxg-_16M02_F_0	BJ985272
E_FL_mxg-_16M05_F_0	BJ985273
E_FL_mxg-_16M07_F_0	BJ985274

E_FL_mxg-_16M10_F_0	BJ985275
E_FL_mxg-_16M12_F_0	BJ985276
E_FL_mxg-_16M15_F_0	BJ985277
E_FL_mxg-_16M18_F_0	BJ985278
E_FL_mxg-_16M19_F_0	BJ985279
E_FL_mxg-_16M20_F_0	BJ985280
E_FL_mxg-_16M23_F_0	BJ985281
E_FL_mxg-_16N01_F_0	BJ985282
E_FL_mxg-_16N02_F_0	BJ985283
E_FL_mxg-_16N04_F_0	BJ985284
E_FL_mxg-_16N11_F_0	BJ985285
E_FL_mxg-_16N12_F_0	BJ985286
E_FL_mxg-_16N17_F_0	BJ985287
E_FL_mxg-_16N18_F_0	BJ985288
E_FL_mxg-_16N20_F_0	BJ985289
E_FL_mxg-_16N22_F_0	BJ985290
E_FL_mxg-_16N23_F_0	BJ985291
E_FL_mxg-_16O01_F_0	BJ985292
E_FL_mxg-_16O03_F_0	BJ985293
E_FL_mxg-_16O07_F_0	BJ985294
E_FL_mxg-_16O08_F_0	BJ985295
E_FL_mxg-_16O11_F_0	BJ985296
E_FL_mxg-_16O12_F_0	BJ985297
E_FL_mxg-_16O16_F_0	BJ985298
E_FL_mxg-_16O18_F_0	BJ985299
E_FL_mxg-_16O19_F_0	BJ985300
E_FL_mxg-_16P03_F_0	BJ985301
E_FL_mxg-_16P04_F_0	BJ985302
E_FL_mxg-_16P06_F_0	BJ985303
E_FL_mxg-_16P08_F_0	BJ985304
E_FL_mxg-_16P09_F_0	BJ985305
E_FL_mxg-_16P11_F_0	BJ985306
E_FL_mxg-_16P13_F_0	BJ985307
E_FL_mxg-_16P15_F_0	BJ985308
E_FL_mxg-_16P16_F_0	BJ985309
E_FL_mxg-_16P18_F_0	BJ985310
E_FL_mxg-_16P19_F_0	BJ985311
E_FL_mxg-_17A04_F_0	BJ985312
E_FL_mxg-_17A05_F_0	BJ985313
E_FL_mxg-_17A07_F_0	BJ985314
E_FL_mxg-_17A09_F_0	BJ985315
E_FL_mxg-_17A15_F_0	BJ985316
E_FL_mxg-_17A16_F_0	BJ985317
E_FL_mxg-_17A17_F_0	BJ985318
E_FL_mxg-_17A19_F_0	BJ985319
E_FL_mxg-_17B01_F_0	BJ985320
E_FL_mxg-_17B03_F_0	BJ985321
E_FL_mxg-_17B07_F_0	BJ985322
E_FL_mxg-_17B09_F_0	BJ985323
E_FL_mxg-_17B12_F_0	BJ985324
E_FL_mxg-_17B14_F_0	BJ985325
E_FL_mxg-_17B15_F_0	BJ985326
E_FL_mxg-_17B16_F_0	BJ985327
E_FL_mxg-_17B17_F_0	BJ985328

E_FL_mxg-_17B19_F_0	BJ985329
E_FL_mxg-_17B23_F_0	BJ985330
E_FL_mxg-_17C02_F_0	BJ985331
E_FL_mxg-_17C07_F_0	BJ985332
E_FL_mxg-_17C09_F_0	BJ985333
E_FL_mxg-_17C10_F_0	BJ985334
E_FL_mxg-_17C13_F_0	BJ985335
E_FL_mxg-_17C19_F_0	BJ985336
E_FL_mxg-_17C23_F_0	BJ985337
E_FL_mxg-_17D02_F_0	BJ985338
E_FL_mxg-_17D07_F_0	BJ985339
E_FL_mxg-_17D09_F_0	BJ985340
E_FL_mxg-_17D11_F_0	BJ985341
E_FL_mxg-_17D16_F_0	BJ985342
E_FL_mxg-_17D17_F_0	BJ985343
E_FL_mxg-_17D18_F_0	BJ985344
E_FL_mxg-_17D20_F_0	BJ985345
E_FL_mxg-_17D21_F_0	BJ985346
E_FL_mxg-_17D22_F_0	BJ985347
E_FL_mxg-_17E01_F_0	BJ985348
E_FL_mxg-_17E02_F_0	BJ985349
E_FL_mxg-_17E04_F_0	BJ985350
E_FL_mxg-_17E06_F_0	BJ985351
E_FL_mxg-_17E07_F_0	BJ985352
E_FL_mxg-_17E08_F_0	BJ985353
E_FL_mxg-_17E09_F_0	BJ985354
E_FL_mxg-_17E13_F_0	BJ985355
E_FL_mxg-_17E14_F_0	BJ985356
E_FL_mxg-_17E16_F_0	BJ985357
E_FL_mxg-_17E20_F_0	BJ985358
E_FL_mxg-_17E21_F_0	BJ985359
E_FL_mxg-_17E22_F_0	BJ985360
E_FL_mxg-_17E23_F_0	BJ985361
E_FL_mxg-_17E24_F_0	BJ985362
E_FL_mxg-_17F02_F_0	BJ985363
E_FL_mxg-_17F03_F_0	BJ985364
E_FL_mxg-_17F04_F_0	BJ985365
E_FL_mxg-_17F05_F_0	BJ985366
E_FL_mxg-_17F06_F_0	BJ985367
E_FL_mxg-_17F07_F_0	BJ985368
E_FL_mxg-_17F09_F_0	BJ985369
E_FL_mxg-_17F10_F_0	BJ985370
E_FL_mxg-_17F12_F_0	BJ985371
E_FL_mxg-_17F13_F_0	BJ985372
E_FL_mxg-_17F19_F_0	BJ985373
E_FL_mxg-_17F21_F_0	BJ985374
E_FL_mxg-_17F23_F_0	BJ985375
E_FL_mxg-_17G01_F_0	BJ985376
E_FL_mxg-_17G07_F_0	BJ985377
E_FL_mxg-_17G09_F_0	BJ985378
E_FL_mxg-_17G11_F_0	BJ985379
E_FL_mxg-_17G15_F_0	BJ985380
E_FL_mxg-_17G17_F_0	BJ985381
E_FL_mxg-_17G18_F_0	BJ985382

E_FL_mxg-_17G20_F_0	BJ985383
E_FL_mxg-_17G21_F_0	BJ985384
E_FL_mxg-_17G24_F_0	BJ985385
E_FL_mxg-_17H04_F_0	BJ985386
E_FL_mxg-_17H07_F_0	BJ985387
E_FL_mxg-_17H08_F_0	BJ985388
E_FL_mxg-_17H09_F_0	BJ985389
E_FL_mxg-_17H10_F_0	BJ985390
E_FL_mxg-_17H12_F_0	BJ985391
E_FL_mxg-_17H13_F_0	BJ985392
E_FL_mxg-_17H15_F_0	BJ985393
E_FL_mxg-_17H18_F_0	BJ985394
E_FL_mxg-_17H19_F_0	BJ985395
E_FL_mxg-_17H20_F_0	BJ985396
E_FL_mxg-_17H21_F_0	BJ985397
E_FL_mxg-_17H22_F_0	BJ985398
E_FL_mxg-_17H23_F_0	BJ985399
E_FL_mxg-_17I03_F_0	BJ985400
E_FL_mxg-_17I04_F_0	BJ985401
E_FL_mxg-_17I07_F_0	BJ985402
E_FL_mxg-_17I08_F_0	BJ985403
E_FL_mxg-_17I09_F_0	BJ985404
E_FL_mxg-_17I13_F_0	BJ985405
E_FL_mxg-_17I14_F_0	BJ985406
E_FL_mxg-_17I16_F_0	BJ985407
E_FL_mxg-_17I23_F_0	BJ985408
E_FL_mxg-_17J06_F_0	BJ985409
E_FL_mxg-_17J07_F_0	BJ985410
E_FL_mxg-_17J08_F_0	BJ985411
E_FL_mxg-_17J09_F_0	BJ985412
E_FL_mxg-_17J14_F_0	BJ985413
E_FL_mxg-_17J15_F_0	BJ985414
E_FL_mxg-_17J16_F_0	BJ985415
E_FL_mxg-_17J17_F_0	BJ985416
E_FL_mxg-_17J18_F_0	BJ985417
E_FL_mxg-_17J21_F_0	BJ985418
E_FL_mxg-_17J24_F_0	BJ985419
E_FL_mxg-_17K01_F_0	BJ985420
E_FL_mxg-_17K03_F_0	BJ985421
E_FL_mxg-_17K04_F_0	BJ985422
E_FL_mxg-_17K05_F_0	BJ985423
E_FL_mxg-_17K07_F_0	BJ985424
E_FL_mxg-_17K08_F_0	BJ985425
E_FL_mxg-_17K09_F_0	BJ985426
E_FL_mxg-_17K10_F_0	BJ985427
E_FL_mxg-_17K13_F_0	BJ985428
E_FL_mxg-_17K14_F_0	BJ985429
E_FL_mxg-_17K15_F_0	BJ985430
E_FL_mxg-_17K17_F_0	BJ985431
E_FL_mxg-_17K21_F_0	BJ985432
E_FL_mxg-_17K24_F_0	BJ985433
E_FL_mxg-_17L04_F_0	BJ985434
E_FL_mxg-_17L07_F_0	BJ985435
E_FL_mxg-_17L09_F_0	BJ985436

E_FL_mxg-_17L11_F_0	BJ985437
E_FL_mxg-_17L12_F_0	BJ985438
E_FL_mxg-_17L13_F_0	BJ985439
E_FL_mxg-_17L14_F_0	BJ985440
E_FL_mxg-_17L16_F_0	BJ985441
E_FL_mxg-_17L17_F_0	BJ985442
E_FL_mxg-_17L19_F_0	BJ985443
E_FL_mxg-_17L20_F_0	BJ985444
E_FL_mxg-_17L24_F_0	BJ985445
E_FL_mxg-_17M01_F_0	BJ985446
E_FL_mxg-_17M02_F_0	BJ985447
E_FL_mxg-_17M04_F_0	BJ985448
E_FL_mxg-_17M09_F_0	BJ985449
E_FL_mxg-_17M11_F_0	BJ985450
E_FL_mxg-_17M13_F_0	BJ985451
E_FL_mxg-_17M14_F_0	BJ985452
E_FL_mxg-_17M15_F_0	BJ985453
E_FL_mxg-_17M20_F_0	BJ985454
E_FL_mxg-_17N01_F_0	BJ985455
E_FL_mxg-_17N02_F_0	BJ985456
E_FL_mxg-_17N04_F_0	BJ985457
E_FL_mxg-_17N06_F_0	BJ985458
E_FL_mxg-_17N07_F_0	BJ985459
E_FL_mxg-_17N10_F_0	BJ985460
E_FL_mxg-_17N11_F_0	BJ985461
E_FL_mxg-_17N12_F_0	BJ985462
E_FL_mxg-_17N13_F_0	BJ985463
E_FL_mxg-_17N15_F_0	BJ985464
E_FL_mxg-_17N16_F_0	BJ985465
E_FL_mxg-_17N17_F_0	BJ985466
E_FL_mxg-_17N18_F_0	BJ985467
E_FL_mxg-_17N20_F_0	BJ985468
E_FL_mxg-_17N21_F_0	BJ985469
E_FL_mxg-_17N24_F_0	BJ985470
E_FL_mxg-_17O04_F_0	BJ985471
E_FL_mxg-_17O08_F_0	BJ985472
E_FL_mxg-_17O10_F_0	BJ985473
E_FL_mxg-_17O13_F_0	BJ985474
E_FL_mxg-_17O21_F_0	BJ985475
E_FL_mxg-_17O22_F_0	BJ985476
E_FL_mxg-_17P03_F_0	BJ985477
E_FL_mxg-_17P04_F_0	BJ985478
E_FL_mxg-_17P07_F_0	BJ985479
E_FL_mxg-_17P12_F_0	BJ985480
E_FL_mxg-_17P13_F_0	BJ985481
E_FL_mxg-_17P14_F_0	BJ985482
E_FL_mxg-_17P16_F_0	BJ985483
E_FL_mxg-_17P17_F_0	BJ985484
E_FL_mxg-_17P21_F_0	BJ985485
E_FL_mxg-_17P22_F_0	BJ985486
E_FL_mxg-_17P23_F_0	BJ985487
E_FL_mxg-_18A01_F_0	BJ985488
E_FL_mxg-_18A02_F_0	BJ985489
E_FL_mxg-_18A04_F_0	BJ985490

E_FL_mxg-_18A08_F_0	BJ985491
E_FL_mxg-_18A10_F_0	BJ985492
E_FL_mxg-_18A12_F_0	BJ985493
E_FL_mxg-_18A19_F_0	BJ985494
E_FL_mxg-_18A22_F_0	BJ985495
E_FL_mxg-_18B01_F_0	BJ985496
E_FL_mxg-_18B02_F_0	BJ985497
E_FL_mxg-_18B03_F_0	BJ985498
E_FL_mxg-_18B04_F_0	BJ985499
E_FL_mxg-_18B06_F_0	BJ985500
E_FL_mxg-_18B10_F_0	BJ985501
E_FL_mxg-_18B11_F_0	BJ985502
E_FL_mxg-_18B18_F_0	BJ985503
E_FL_mxg-_18B19_F_0	BJ985504
E_FL_mxg-_18B20_F_0	BJ985505
E_FL_mxg-_18C02_F_0	BJ985506
E_FL_mxg-_18C03_F_0	BJ985507
E_FL_mxg-_18C05_F_0	BJ985508
E_FL_mxg-_18C07_F_0	BJ985509
E_FL_mxg-_18C08_F_0	BJ985510
E_FL_mxg-_18C09_F_0	BJ985511
E_FL_mxg-_18C16_F_0	BJ985512
E_FL_mxg-_18C17_F_0	BJ985513
E_FL_mxg-_18C19_F_0	BJ985514
E_FL_mxg-_18C20_F_0	BJ985515
E_FL_mxg-_18C21_F_0	BJ985516
E_FL_mxg-_18C22_F_0	BJ985517
E_FL_mxg-_18D04_F_0	BJ985518
E_FL_mxg-_18D05_F_0	BJ985519
E_FL_mxg-_18D06_F_0	BJ985520
E_FL_mxg-_18D08_F_0	BJ985521
E_FL_mxg-_18D09_F_0	BJ985522
E_FL_mxg-_18D12_F_0	BJ985523
E_FL_mxg-_18D15_F_0	BJ985524
E_FL_mxg-_18D16_F_0	BJ985525
E_FL_mxg-_18D20_F_0	BJ985526
E_FL_mxg-_18D21_F_0	BJ985527
E_FL_mxg-_18D22_F_0	BJ985528
E_FL_mxg-_18D23_F_0	BJ985529
E_FL_mxg-_18E03_F_0	BJ985530
E_FL_mxg-_18E06_F_0	BJ985531
E_FL_mxg-_18E09_F_0	BJ985532
E_FL_mxg-_18E10_F_0	BJ985533
E_FL_mxg-_18E11_F_0	BJ985534
E_FL_mxg-_18E12_F_0	BJ985535
E_FL_mxg-_18E13_F_0	BJ985536
E_FL_mxg-_18E14_F_0	BJ985537
E_FL_mxg-_18E20_F_0	BJ985538
E_FL_mxg-_18E21_F_0	BJ985539
E_FL_mxg-_18E22_F_0	BJ985540
E_FL_mxg-_18E24_F_0	BJ985541
E_FL_mxg-_18F02_F_0	BJ985542
E_FL_mxg-_18F09_F_0	BJ985543
E_FL_mxg-_18F11_F_0	BJ985544

E_FL_mxg-_18F12_F_0	BJ985545
E_FL_mxg-_18F14_F_0	BJ985546
E_FL_mxg-_18F16_F_0	BJ985547
E_FL_mxg-_18F18_F_0	BJ985548
E_FL_mxg-_18F19_F_0	BJ985549
E_FL_mxg-_18F21_F_0	BJ985550
E_FL_mxg-_18F22_F_0	BJ985551
E_FL_mxg-_18G01_F_0	BJ985552
E_FL_mxg-_18G05_F_0	BJ985553
E_FL_mxg-_18G08_F_0	BJ985554
E_FL_mxg-_18G17_F_0	BJ985555
E_FL_mxg-_18G19_F_0	BJ985556
E_FL_mxg-_18G20_F_0	BJ985557
E_FL_mxg-_18G21_F_0	BJ985558
E_FL_mxg-_18G22_F_0	BJ985559
E_FL_mxg-_18G23_F_0	BJ985560
E_FL_mxg-_18H01_F_0	BJ985561
E_FL_mxg-_18H02_F_0	BJ985562
E_FL_mxg-_18H06_F_0	BJ985563
E_FL_mxg-_18H08_F_0	BJ985564
E_FL_mxg-_18H10_F_0	BJ985565
E_FL_mxg-_18H11_F_0	BJ985566
E_FL_mxg-_18H13_F_0	BJ985567
E_FL_mxg-_18H14_F_0	BJ985568
E_FL_mxg-_18H15_F_0	BJ985569
E_FL_mxg-_18H18_F_0	BJ985570
E_FL_mxg-_18H19_F_0	BJ985571
E_FL_mxg-_18H21_F_0	BJ985572
E_FL_mxg-_18H23_F_0	BJ985573
E_FL_mxg-_18H24_F_0	BJ985574
E_FL_mxg-_18I01_F_0	BJ985575
E_FL_mxg-_18I02_F_0	BJ985576
E_FL_mxg-_18I04_F_0	BJ985577
E_FL_mxg-_18I06_F_0	BJ985578
E_FL_mxg-_18I10_F_0	BJ985579
E_FL_mxg-_18I11_F_0	BJ985580
E_FL_mxg-_18I13_F_0	BJ985581
E_FL_mxg-_18I15_F_0	BJ985582
E_FL_mxg-_18I18_F_0	BJ985583
E_FL_mxg-_18I22_F_0	BJ985584
E_FL_mxg-_18I23_F_0	BJ985585
E_FL_mxg-_18J01_F_0	BJ985586
E_FL_mxg-_18J02_F_0	BJ985587
E_FL_mxg-_18J03_F_0	BJ985588
E_FL_mxg-_18J10_F_0	BJ985589
E_FL_mxg-_18J11_F_0	BJ985590
E_FL_mxg-_18J14_F_0	BJ985591
E_FL_mxg-_18J18_F_0	BJ985592
E_FL_mxg-_18J21_F_0	BJ985593
E_FL_mxg-_18J23_F_0	BJ985594
E_FL_mxg-_18K01_F_0	BJ985595
E_FL_mxg-_18K05_F_0	BJ985596
E_FL_mxg-_18K06_F_0	BJ985597
E_FL_mxg-_18K08_F_0	BJ985598

E_FL_mxg-_18K09_F_0	BJ985599
E_FL_mxg-_18K12_F_0	BJ985600
E_FL_mxg-_18K15_F_0	BJ985601
E_FL_mxg-_18K17_F_0	BJ985602
E_FL_mxg-_18K18_F_0	BJ985603
E_FL_mxg-_18K21_F_0	BJ985604
E_FL_mxg-_18K23_F_0	BJ985605
E_FL_mxg-_18L02_F_0	BJ985606
E_FL_mxg-_18L03_F_0	BJ985607
E_FL_mxg-_18L04_F_0	BJ985608
E_FL_mxg-_18L05_F_0	BJ985609
E_FL_mxg-_18L11_F_0	BJ985610
E_FL_mxg-_18L12_F_0	BJ985611
E_FL_mxg-_18L16_F_0	BJ985612
E_FL_mxg-_18L20_F_0	BJ985613
E_FL_mxg-_18L24_F_0	BJ985614
E_FL_mxg-_18M05_F_0	BJ985615
E_FL_mxg-_18M08_F_0	BJ985616
E_FL_mxg-_18M09_F_0	BJ985617
E_FL_mxg-_18M11_F_0	BJ985618
E_FL_mxg-_18M12_F_0	BJ985619
E_FL_mxg-_18M13_F_0	BJ985620
E_FL_mxg-_18M15_F_0	BJ985621
E_FL_mxg-_18M17_F_0	BJ985622
E_FL_mxg-_18M18_F_0	BJ985623
E_FL_mxg-_18M19_F_0	BJ985624
E_FL_mxg-_18M23_F_0	BJ985625
E_FL_mxg-_18M24_F_0	BJ985626
E_FL_mxg-_18N04_F_0	BJ985627
E_FL_mxg-_18N06_F_0	BJ985628
E_FL_mxg-_18N08_F_0	BJ985629
E_FL_mxg-_18N10_F_0	BJ985630
E_FL_mxg-_18N13_F_0	BJ985631
E_FL_mxg-_18N14_F_0	BJ985632
E_FL_mxg-_18N19_F_0	BJ985633
E_FL_mxg-_18N20_F_0	BJ985634
E_FL_mxg-_18N23_F_0	BJ985635
E_FL_mxg-_18O02_F_0	BJ985636
E_FL_mxg-_18O05_F_0	BJ985637
E_FL_mxg-_18O09_F_0	BJ985638
E_FL_mxg-_18O10_F_0	BJ985639
E_FL_mxg-_18O12_F_0	BJ985640
E_FL_mxg-_18O15_F_0	BJ985641
E_FL_mxg-_18O16_F_0	BJ985642
E_FL_mxg-_18O23_F_0	BJ985643
E_FL_mxg-_18P02_F_0	BJ985644
E_FL_mxg-_18P03_F_0	BJ985645
E_FL_mxg-_18P06_F_0	BJ985646
E_FL_mxg-_18P09_F_0	BJ985647
E_FL_mxg-_18P10_F_0	BJ985648
E_FL_mxg-_18P13_F_0	BJ985649
E_FL_mxg-_18P15_F_0	BJ985650
E_FL_mxg-_18P16_F_0	BJ985651
E_FL_mxg-_18P17_F_0	BJ985652

E_FL_mxg-_18P20_F_0	BJ985653
E_FL_mxg-_19A05_F_0	BJ985654
E_FL_mxg-_19A07_F_0	BJ985655
E_FL_mxg-_19A11_F_0	BJ985656
E_FL_mxg-_19A13_F_0	BJ985657
E_FL_mxg-_19A17_F_0	BJ985658
E_FL_mxg-_19A18_F_0	BJ985659
E_FL_mxg-_19A19_F_0	BJ985660
E_FL_mxg-_19A20_F_0	BJ985661
E_FL_mxg-_19A21_F_0	BJ985662
E_FL_mxg-_19A22_F_0	BJ985663
E_FL_mxg-_19A23_F_0	BJ985664
E_FL_mxg-_19B01_F_0	BJ985665
E_FL_mxg-_19B02_F_0	BJ985666
E_FL_mxg-_19B03_F_0	BJ985667
E_FL_mxg-_19B05_F_0	BJ985668
E_FL_mxg-_19B06_F_0	BJ985669
E_FL_mxg-_19B07_F_0	BJ985670
E_FL_mxg-_19B09_F_0	BJ985671
E_FL_mxg-_19B11_F_0	BJ985672
E_FL_mxg-_19B12_F_0	BJ985673
E_FL_mxg-_19B21_F_0	BJ985674
E_FL_mxg-_19B24_F_0	BJ985675
E_FL_mxg-_19C02_F_0	BJ985676
E_FL_mxg-_19C03_F_0	BJ985677
E_FL_mxg-_19C07_F_0	BJ985678
E_FL_mxg-_19C12_F_0	BJ985679
E_FL_mxg-_19C13_F_0	BJ985680
E_FL_mxg-_19C14_F_0	BJ985681
E_FL_mxg-_19C16_F_0	BJ985682
E_FL_mxg-_19C17_F_0	BJ985683
E_FL_mxg-_19C19_F_0	BJ985684
E_FL_mxg-_19C20_F_0	BJ985685
E_FL_mxg-_19C22_F_0	BJ985686
E_FL_mxg-_19C24_F_0	BJ985687
E_FL_mxg-_19D03_F_0	BJ985688
E_FL_mxg-_19D05_F_0	BJ985689
E_FL_mxg-_19D06_F_0	BJ985690
E_FL_mxg-_19D07_F_0	BJ985691
E_FL_mxg-_19D14_F_0	BJ985692
E_FL_mxg-_19D15_F_0	BJ985693
E_FL_mxg-_19D17_F_0	BJ985694
E_FL_mxg-_19D19_F_0	BJ985695
E_FL_mxg-_19D20_F_0	BJ985696
E_FL_mxg-_19E01_F_0	BJ985697
E_FL_mxg-_19E02_F_0	BJ985698
E_FL_mxg-_19E05_F_0	BJ985699
E_FL_mxg-_19E06_F_0	BJ985700
E_FL_mxg-_19E07_F_0	BJ985701
E_FL_mxg-_19E09_F_0	BJ985702
E_FL_mxg-_19E11_F_0	BJ985703
E_FL_mxg-_19E13_F_0	BJ985704
E_FL_mxg-_19E19_F_0	BJ985705
E_FL_mxg-_19E22_F_0	BJ985706

E_FL_mxg-_19E23_F_0	BJ985707
E_FL_mxg-_19F01_F_0	BJ985708
E_FL_mxg-_19F02_F_0	BJ985709
E_FL_mxg-_19F04_F_0	BJ985710
E_FL_mxg-_19F07_F_0	BJ985711
E_FL_mxg-_19F09_F_0	BJ985712
E_FL_mxg-_19F10_F_0	BJ985713
E_FL_mxg-_19F11_F_0	BJ985714
E_FL_mxg-_19F14_F_0	BJ985715
E_FL_mxg-_19F15_F_0	BJ985716
E_FL_mxg-_19F16_F_0	BJ985717
E_FL_mxg-_19F18_F_0	BJ985718
E_FL_mxg-_19F19_F_0	BJ985719
E_FL_mxg-_19F22_F_0	BJ985720
E_FL_mxg-_19F24_F_0	BJ985721
E_FL_mxg-_19G02_F_0	BJ985722
E_FL_mxg-_19G03_F_0	BJ985723
E_FL_mxg-_19G05_F_0	BJ985724
E_FL_mxg-_19G06_F_0	BJ985725
E_FL_mxg-_19G11_F_0	BJ985726
E_FL_mxg-_19G12_F_0	BJ985727
E_FL_mxg-_19G14_F_0	BJ985728
E_FL_mxg-_19G15_F_0	BJ985729
E_FL_mxg-_19G16_F_0	BJ985730
E_FL_mxg-_19G21_F_0	BJ985731
E_FL_mxg-_19G23_F_0	BJ985732
E_FL_mxg-_19H02_F_0	BJ985733
E_FL_mxg-_19H06_F_0	BJ985734
E_FL_mxg-_19H07_F_0	BJ985735
E_FL_mxg-_19H12_F_0	BJ985736
E_FL_mxg-_19H15_F_0	BJ985737
E_FL_mxg-_19H16_F_0	BJ985738
E_FL_mxg-_19H18_F_0	BJ985739
E_FL_mxg-_19H19_F_0	BJ985740
E_FL_mxg-_19H20_F_0	BJ985741
E_FL_mxg-_19I01_F_0	BJ985742
E_FL_mxg-_19I04_F_0	BJ985743
E_FL_mxg-_19I05_F_0	BJ985744
E_FL_mxg-_19I07_F_0	BJ985745
E_FL_mxg-_19I09_F_0	BJ985746
E_FL_mxg-_19I11_F_0	BJ985747
E_FL_mxg-_19I12_F_0	BJ985748
E_FL_mxg-_19I14_F_0	BJ985749
E_FL_mxg-_19I15_F_0	BJ985750
E_FL_mxg-_19I16_F_0	BJ985751
E_FL_mxg-_19I17_F_0	BJ985752
E_FL_mxg-_19I23_F_0	BJ985753
E_FL_mxg-_19J01_F_0	BJ985754
E_FL_mxg-_19J02_F_0	BJ985755
E_FL_mxg-_19J05_F_0	BJ985756
E_FL_mxg-_19J07_F_0	BJ985757
E_FL_mxg-_19J09_F_0	BJ985758
E_FL_mxg-_19J10_F_0	BJ985759
E_FL_mxg-_19J11_F_0	BJ985760

E_FL_mxg-_19J13_F_0	BJ985761
E_FL_mxg-_19J17_F_0	BJ985762
E_FL_mxg-_19J21_F_0	BJ985763
E_FL_mxg-_19K01_F_0	BJ985764
E_FL_mxg-_19K04_F_0	BJ985765
E_FL_mxg-_19K05_F_0	BJ985766
E_FL_mxg-_19K07_F_0	BJ985767
E_FL_mxg-_19K08_F_0	BJ985768
E_FL_mxg-_19K09_F_0	BJ985769
E_FL_mxg-_19K10_F_0	BJ985770
E_FL_mxg-_19K12_F_0	BJ985771
E_FL_mxg-_19K13_F_0	BJ985772
E_FL_mxg-_19K14_F_0	BJ985773
E_FL_mxg-_19K15_F_0	BJ985774
E_FL_mxg-_19K17_F_0	BJ985775
E_FL_mxg-_19K22_F_0	BJ985776
E_FL_mxg-_19K24_F_0	BJ985777
E_FL_mxg-_19L01_F_0	BJ985778
E_FL_mxg-_19L02_F_0	BJ985779
E_FL_mxg-_19L04_F_0	BJ985780
E_FL_mxg-_19L06_F_0	BJ985781
E_FL_mxg-_19L10_F_0	BJ985782
E_FL_mxg-_19L14_F_0	BJ985783
E_FL_mxg-_19L15_F_0	BJ985784
E_FL_mxg-_19L19_F_0	BJ985785
E_FL_mxg-_19L20_F_0	BJ985786
E_FL_mxg-_19L21_F_0	BJ985787
E_FL_mxg-_19M03_F_0	BJ985788
E_FL_mxg-_19M05_F_0	BJ985789
E_FL_mxg-_19M06_F_0	BJ985790
E_FL_mxg-_19M09_F_0	BJ985791
E_FL_mxg-_19M10_F_0	BJ985792
E_FL_mxg-_19M13_F_0	BJ985793
E_FL_mxg-_19N01_F_0	BJ985794
E_FL_mxg-_19N02_F_0	BJ985795
E_FL_mxg-_19N05_F_0	BJ985796
E_FL_mxg-_19N09_F_0	BJ985797
E_FL_mxg-_19N12_F_0	BJ985798
E_FL_mxg-_19N13_F_0	BJ985799
E_FL_mxg-_19N16_F_0	BJ985800
E_FL_mxg-_19N17_F_0	BJ985801
E_FL_mxg-_19N24_F_0	BJ985802
E_FL_mxg-_19O01_F_0	BJ985803
E_FL_mxg-_19O02_F_0	BJ985804
E_FL_mxg-_19O05_F_0	BJ985805
E_FL_mxg-_19O07_F_0	BJ985806
E_FL_mxg-_19O10_F_0	BJ985807
E_FL_mxg-_19O14_F_0	BJ985808
E_FL_mxg-_19O24_F_0	BJ985809
E_FL_mxg-_19P04_F_0	BJ985810
E_FL_mxg-_19P05_F_0	BJ985811
E_FL_mxg-_19P06_F_0	BJ985812
E_FL_mxg-_19P08_F_0	BJ985813
E_FL_mxg-_19P09_F_0	BJ985814

E_FL_mxg-_19P10_F_0	BJ985815
E_FL_mxg-_19P14_F_0	BJ985816
E_FL_mxg-_19P16_F_0	BJ985817
E_FL_mxg-_19P18_F_0	BJ985818
E_FL_mxg-_19P19_F_0	BJ985819
E_FL_mxg-_19P23_F_0	BJ985820
E_FL_mxg-_20A03_F_0	BJ985821
E_FL_mxg-_20A04_F_0	BJ985822
E_FL_mxg-_20A06_F_0	BJ985823
E_FL_mxg-_20A07_F_0	BJ985824
E_FL_mxg-_20A10_F_0	BJ985825
E_FL_mxg-_20A16_F_0	BJ985826
E_FL_mxg-_20A17_F_0	BJ985827
E_FL_mxg-_20A18_F_0	BJ985828
E_FL_mxg-_20A19_F_0	BJ985829
E_FL_mxg-_20A20_F_0	BJ985830
E_FL_mxg-_20B03_F_0	BJ985831
E_FL_mxg-_20B04_F_0	BJ985832
E_FL_mxg-_20B05_F_0	BJ985833
E_FL_mxg-_20B12_F_0	BJ985834
E_FL_mxg-_20B13_F_0	BJ985835
E_FL_mxg-_20B16_F_0	BJ985836
E_FL_mxg-_20B18_F_0	BJ985837
E_FL_mxg-_20B19_F_0	BJ985838
E_FL_mxg-_20B22_F_0	BJ985839
E_FL_mxg-_20C02_F_0	BJ985840
E_FL_mxg-_20C03_F_0	BJ985841
E_FL_mxg-_20C06_F_0	BJ985842
E_FL_mxg-_20C07_F_0	BJ985843
E_FL_mxg-_20C09_F_0	BJ985844
E_FL_mxg-_20C12_F_0	BJ985845
E_FL_mxg-_20C15_F_0	BJ985846
E_FL_mxg-_20C17_F_0	BJ985847
E_FL_mxg-_20C21_F_0	BJ985848
E_FL_mxg-_20C22_F_0	BJ985849
E_FL_mxg-_20D01_F_0	BJ985850
E_FL_mxg-_20D07_F_0	BJ985851
E_FL_mxg-_20D10_F_0	BJ985852
E_FL_mxg-_20D21_F_0	BJ985853
E_FL_mxg-_20D22_F_0	BJ985854
E_FL_mxg-_20E02_F_0	BJ985855
E_FL_mxg-_20E04_F_0	BJ985856
E_FL_mxg-_20E05_F_0	BJ985857
E_FL_mxg-_20E06_F_0	BJ985858
E_FL_mxg-_20E08_F_0	BJ985859
E_FL_mxg-_20E11_F_0	BJ985860
E_FL_mxg-_20E12_F_0	BJ985861
E_FL_mxg-_20E13_F_0	BJ985862
E_FL_mxg-_20E15_F_0	BJ985863
E_FL_mxg-_20E16_F_0	BJ985864
E_FL_mxg-_20E20_F_0	BJ985865
E_FL_mxg-_20E22_F_0	BJ985866
E_FL_mxg-_20E23_F_0	BJ985867
E_FL_mxg-_20F05_F_0	BJ985868

E_FL_mxg-_20F09_F_0	BJ985869
E_FL_mxg-_20F10_F_0	BJ985870
E_FL_mxg-_20F15_F_0	BJ985871
E_FL_mxg-_20F17_F_0	BJ985872
E_FL_mxg-_20F18_F_0	BJ985873
E_FL_mxg-_20F23_F_0	BJ985874
E_FL_mxg-_20G03_F_0	BJ985875
E_FL_mxg-_20G04_F_0	BJ985876
E_FL_mxg-_20G05_F_0	BJ985877
E_FL_mxg-_20G10_F_0	BJ985878
E_FL_mxg-_20G11_F_0	BJ985879
E_FL_mxg-_20G13_F_0	BJ985880
E_FL_mxg-_20G15_F_0	BJ985881
E_FL_mxg-_20G21_F_0	BJ985882
E_FL_mxg-_20G22_F_0	BJ985883
E_FL_mxg-_20G23_F_0	BJ985884
E_FL_mxg-_20H02_F_0	BJ985885
E_FL_mxg-_20H03_F_0	BJ985886
E_FL_mxg-_20H04_F_0	BJ985887
E_FL_mxg-_20H07_F_0	BJ985888
E_FL_mxg-_20H08_F_0	BJ985889
E_FL_mxg-_20H09_F_0	BJ985890
E_FL_mxg-_20H14_F_0	BJ985891
E_FL_mxg-_20H16_F_0	BJ985892
E_FL_mxg-_20H18_F_0	BJ985893
E_FL_mxg-_20H19_F_0	BJ985894
E_FL_mxg-_20H22_F_0	BJ985895
E_FL_mxg-_20I01_F_0	BJ985896
E_FL_mxg-_20I02_F_0	BJ985897
E_FL_mxg-_20I03_F_0	BJ985898
E_FL_mxg-_20I06_F_0	BJ985899
E_FL_mxg-_20I07_F_0	BJ985900
E_FL_mxg-_20I11_F_0	BJ985901
E_FL_mxg-_20I13_F_0	BJ985902
E_FL_mxg-_20I14_F_0	BJ985903
E_FL_mxg-_20I16_F_0	BJ985904
E_FL_mxg-_20I17_F_0	BJ985905
E_FL_mxg-_20I20_F_0	BJ985906
E_FL_mxg-_20I21_F_0	BJ985907
E_FL_mxg-_20I24_F_0	BJ985908
E_FL_mxg-_20J07_F_0	BJ985909
E_FL_mxg-_20J08_F_0	BJ985910
E_FL_mxg-_20J09_F_0	BJ985911
E_FL_mxg-_20J10_F_0	BJ985912
E_FL_mxg-_20J11_F_0	BJ985913
E_FL_mxg-_20J15_F_0	BJ985914
E_FL_mxg-_20J16_F_0	BJ985915
E_FL_mxg-_20J17_F_0	BJ985916
E_FL_mxg-_20J19_F_0	BJ985917
E_FL_mxg-_20J20_F_0	BJ985918
E_FL_mxg-_20J22_F_0	BJ985919
E_FL_mxg-_20J23_F_0	BJ985920
E_FL_mxg-_20J24_F_0	BJ985921
E_FL_mxg-_20K01_F_0	BJ985922

E_FL_mxg-_20K02_F_0	BJ985923
E_FL_mxg-_20K03_F_0	BJ985924
E_FL_mxg-_20K04_F_0	BJ985925
E_FL_mxg-_20K06_F_0	BJ985926
E_FL_mxg-_20K09_F_0	BJ985927
E_FL_mxg-_20K10_F_0	BJ985928
E_FL_mxg-_20K11_F_0	BJ985929
E_FL_mxg-_20K13_F_0	BJ985930
E_FL_mxg-_20K19_F_0	BJ985931
E_FL_mxg-_20K20_F_0	BJ985932
E_FL_mxg-_20L01_F_0	BJ985933
E_FL_mxg-_20L02_F_0	BJ985934
E_FL_mxg-_20L05_F_0	BJ985935
E_FL_mxg-_20L07_F_0	BJ985936
E_FL_mxg-_20L08_F_0	BJ985937
E_FL_mxg-_20L09_F_0	BJ985938
E_FL_mxg-_20L10_F_0	BJ985939
E_FL_mxg-_20L11_F_0	BJ985940
E_FL_mxg-_20L13_F_0	BJ985941
E_FL_mxg-_20L16_F_0	BJ985942
E_FL_mxg-_20L18_F_0	BJ985943
E_FL_mxg-_20L22_F_0	BJ985944
E_FL_mxg-_20L23_F_0	BJ985945
E_FL_mxg-_20M01_F_0	BJ985946
E_FL_mxg-_20M07_F_0	BJ985947
E_FL_mxg-_20M08_F_0	BJ985948
E_FL_mxg-_20M09_F_0	BJ985949
E_FL_mxg-_20M12_F_0	BJ985950
E_FL_mxg-_20M14_F_0	BJ985951
E_FL_mxg-_20M19_F_0	BJ985952
E_FL_mxg-_20M23_F_0	BJ985953
E_FL_mxg-_20N04_F_0	BJ985954
E_FL_mxg-_20N07_F_0	BJ985955
E_FL_mxg-_20N09_F_0	BJ985956
E_FL_mxg-_20N11_F_0	BJ985957
E_FL_mxg-_20N12_F_0	BJ985958
E_FL_mxg-_20N14_F_0	BJ985959
E_FL_mxg-_20N20_F_0	BJ985960
E_FL_mxg-_20N23_F_0	BJ985961
E_FL_mxg-_20O01_F_0	BJ985962
E_FL_mxg-_20O02_F_0	BJ985963
E_FL_mxg-_20O04_F_0	BJ985964
E_FL_mxg-_20O05_F_0	BJ985965
E_FL_mxg-_20O10_F_0	BJ985966
E_FL_mxg-_20O14_F_0	BJ985967
E_FL_mxg-_20O16_F_0	BJ985968
E_FL_mxg-_20O17_F_0	BJ985969
E_FL_mxg-_20O20_F_0	BJ985970
E_FL_mxg-_20O22_F_0	BJ985971
E_FL_mxg-_20P07_F_0	BJ985972
E_FL_mxg-_20P11_F_0	BJ985973
E_FL_mxg-_20P12_F_0	BJ985974
E_FL_mxg-_20P14_F_0	BJ985975
E_FL_mxg-_20P18_F_0	BJ985976

E_FL_mxg-_20P21_F_0	BJ985977
E_FL_mxg-_20P22_F_0	BJ985978
E_FL_mxg-_21A03_F_0	BJ985979
E_FL_mxg-_21A11_F_0	BJ985980
E_FL_mxg-_21A19_F_0	BJ985981
E_FL_mxg-_21B04_F_0	BJ985982
E_FL_mxg-_21B10_F_0	BJ985983
E_FL_mxg-_21B11_F_0	BJ985984
E_FL_mxg-_21B12_F_0	BJ985985
E_FL_mxg-_21B13_F_0	BJ985986
E_FL_mxg-_21B18_F_0	BJ985987
E_FL_mxg-_21C01_F_0	BJ985988
E_FL_mxg-_21C10_F_0	BJ985989
E_FL_mxg-_21C13_F_0	BJ985990
E_FL_mxg-_21D01_F_0	BJ985991
E_FL_mxg-_21D02_F_0	BJ985992
E_FL_mxg-_21D09_F_0	BJ985993
E_FL_mxg-_21E21_F_0	BJ985994
E_FL_mxg-_21F09_F_0	BJ985995
E_FL_mxg-_21F12_F_0	BJ985996
E_FL_mxg-_21F15_F_0	BJ985997
E_FL_mxg-_21G13_F_0	BJ985998
E_FL_mxg-_21G22_F_0	BJ985999
E_FL_mxg-_21G23_F_0	BJ986000
E_FL_mxg-_21H01_F_0	BJ986001
E_FL_mxg-_21H12_F_0	BJ986002
E_FL_mxg-_21H14_F_0	BJ986003
E_FL_mxg-_21H16_F_0	BJ986004
E_FL_mxg-_21H22_F_0	BJ986005
E_FL_mxg-_21H23_F_0	BJ986006
E_FL_mxg-_21H24_F_0	BJ986007
E_FL_mxg-_21I22_F_0	BJ986008
E_FL_mxg-_21I23_F_0	BJ986009
E_FL_mxg-_21I24_F_0	BJ986010
E_FL_mxg-_21J01_F_0	BJ986011
E_FL_mxg-_21J02_F_0	BJ986012
E_FL_mxg-_21J18_F_0	BJ986013
E_FL_mxg-_21J23_F_0	BJ986014
E_FL_mxg-_21K15_F_0	BJ986015
E_FL_mxg-_21L15_F_0	BJ986016
E_FL_mxg-_21L21_F_0	BJ986017
E_FL_mxg-_21L23_F_0	BJ986018
E_FL_mxg-_21M09_F_0	BJ986019
E_FL_mxg-_21N01_F_0	BJ986020
E_FL_mxg-_21N02_F_0	BJ986021
E_FL_mxg-_21N19_F_0	BJ986022
E_FL_mxg-_21O02_F_0	BJ986023
E_FL_mxg-_21O09_F_0	BJ986024
E_FL_mxg-_21O16_F_0	BJ986025
E_FL_mxg-_21O21_F_0	BJ986026
E_FL_mxg-_21P05_F_0	BJ986027
E_FL_mxg-_21P21_F_0	BJ986028
E_FL_mxg-_21P22_F_0	BJ986029
E_FL_mxg-_22A04_F_0	BJ986030

E_FL_mxg-_22A05_F_0	BJ986031
E_FL_mxg-_22A06_F_0	BJ986032
E_FL_mxg-_22A10_F_0	BJ986033
E_FL_mxg-_22A14_F_0	BJ986034
E_FL_mxg-_22A17_F_0	BJ986035
E_FL_mxg-_22A19_F_0	BJ986036
E_FL_mxg-_22B01_F_0	BJ986037
E_FL_mxg-_22B03_F_0	BJ986038
E_FL_mxg-_22B06_F_0	BJ986039
E_FL_mxg-_22B10_F_0	BJ986040
E_FL_mxg-_22B12_F_0	BJ986041
E_FL_mxg-_22B17_F_0	BJ986042
E_FL_mxg-_22B18_F_0	BJ986043
E_FL_mxg-_22C09_F_0	BJ986044
E_FL_mxg-_22C13_F_0	BJ986045
E_FL_mxg-_22D19_F_0	BJ986046
E_FL_mxg-_22F11_F_0	BJ986047
E_FL_mxg-_22F16_F_0	BJ986048
E_FL_mxg-_22G11_F_0	BJ986049
E_FL_mxg-_22G12_F_0	BJ986050
E_FL_mxg-_22G23_F_0	BJ986051
E_FL_mxg-_22H11_F_0	BJ986052
E_FL_mxg-_22H21_F_0	BJ986053
E_FL_mxg-_22H23_F_0	BJ986054
E_FL_mxg-_22I17_F_0	BJ986055
E_FL_mxg-_22I18_F_0	BJ986056
E_FL_mxg-_22I21_F_0	BJ986057
E_FL_mxg-_22I24_F_0	BJ986058
E_FL_mxg-_22J02_F_0	BJ986059
E_FL_mxg-_22J21_F_0	BJ986060
E_FL_mxg-_22J23_F_0	BJ986061
E_FL_mxg-_22K22_F_0	BJ986062
E_FL_mxg-_22N09_F_0	BJ986063
E_FL_mxg-_22O02_F_0	BJ986064
E_FL_mxg-_22O07_F_0	BJ986065
E_FL_mxg-_22O09_F_0	BJ986066
E_FL_mxg-_22O10_F_0	BJ986067
E_FL_mxg-_22O12_F_0	BJ986068
E_FL_mxg-_22P20_F_0	BJ986069
E_FL_mxg-_22P21_F_0	BJ986070
E_FL_mxg-_23A05_F_0	BJ986071
E_FL_mxg-_23A06_F_0	BJ986072
E_FL_mxg-_23A10_F_0	BJ986073
E_FL_mxg-_23A12_F_0	BJ986074
E_FL_mxg-_23A13_F_0	BJ986075
E_FL_mxg-_23A14_F_0	BJ986076
E_FL_mxg-_23A20_F_0	BJ986077
E_FL_mxg-_23A21_F_0	BJ986078
E_FL_mxg-_23A23_F_0	BJ986079
E_FL_mxg-_23B06_F_0	BJ986080
E_FL_mxg-_23B10_F_0	BJ986081
E_FL_mxg-_23B15_F_0	BJ986082
E_FL_mxg-_23B17_F_0	BJ986083
E_FL_mxg-_23B18_F_0	BJ986084

E_FL_mxg-_23B19_F_0	BJ986085
E_FL_mxg-_23B21_F_0	BJ986086
E_FL_mxg-_23B23_F_0	BJ986087
E_FL_mxg-_23C01_F_0	BJ986088
E_FL_mxg-_23C15_F_0	BJ986089
E_FL_mxg-_23C19_F_0	BJ986090
E_FL_mxg-_23C23_F_0	BJ986091
E_FL_mxg-_23D03_F_0	BJ986092
E_FL_mxg-_23E01_F_0	BJ986093
E_FL_mxg-_23E03_F_0	BJ986094
E_FL_mxg-_23E04_F_0	BJ986095
E_FL_mxg-_23E05_F_0	BJ986096
E_FL_mxg-_23E12_F_0	BJ986097
E_FL_mxg-_23E19_F_0	BJ986098
E_FL_mxg-_23E21_F_0	BJ986099
E_FL_mxg-_23E22_F_0	BJ986100
E_FL_mxg-_23F11_F_0	BJ986101
E_FL_mxg-_23F20_F_0	BJ986102
E_FL_mxg-_23F21_F_0	BJ986103
E_FL_mxg-_23G02_F_0	BJ986104
E_FL_mxg-_23G03_F_0	BJ986105
E_FL_mxg-_23G10_F_0	BJ986106
E_FL_mxg-_23G11_F_0	BJ986107
E_FL_mxg-_23G17_F_0	BJ986108
E_FL_mxg-_23G18_F_0	BJ986109
E_FL_mxg-_23G21_F_0	BJ986110
E_FL_mxg-_23G23_F_0	BJ986111
E_FL_mxg-_23H13_F_0	BJ986112
E_FL_mxg-_23H16_F_0	BJ986113
E_FL_mxg-_23H21_F_0	BJ986114
E_FL_mxg-_23H22_F_0	BJ986115
E_FL_mxg-_23I01_F_0	BJ986116
E_FL_mxg-_23I02_F_0	BJ986117
E_FL_mxg-_23I09_F_0	BJ986118
E_FL_mxg-_23I10_F_0	BJ986119
E_FL_mxg-_23I11_F_0	BJ986120
E_FL_mxg-_23I14_F_0	BJ986121
E_FL_mxg-_23I17_F_0	BJ986122
E_FL_mxg-_23I18_F_0	BJ986123
E_FL_mxg-_23I21_F_0	BJ986124
E_FL_mxg-_23J09_F_0	BJ986125
E_FL_mxg-_23J10_F_0	BJ986126
E_FL_mxg-_23J12_F_0	BJ986127
E_FL_mxg-_23J20_F_0	BJ986128
E_FL_mxg-_23J22_F_0	BJ986129
E_FL_mxg-_23J24_F_0	BJ986130
E_FL_mxg-_23K03_F_0	BJ986131
E_FL_mxg-_23K14_F_0	BJ986132
E_FL_mxg-_23K18_F_0	BJ986133
E_FL_mxg-_23K19_F_0	BJ986134
E_FL_mxg-_23K22_F_0	BJ986135
E_FL_mxg-_23L03_F_0	BJ986136
E_FL_mxg-_23L09_F_0	BJ986137
E_FL_mxg-_23L13_F_0	BJ986138

E_FL_mxg-_23L15_F_0	BJ986139
E_FL_mxg-_23L17_F_0	BJ986140
E_FL_mxg-_23L18_F_0	BJ986141
E_FL_mxg-_23M01_F_0	BJ986142
E_FL_mxg-_23M05_F_0	BJ986143
E_FL_mxg-_23M10_F_0	BJ986144
E_FL_mxg-_23M13_F_0	BJ986145
E_FL_mxg-_23M14_F_0	BJ986146
E_FL_mxg-_23M15_F_0	BJ986147
E_FL_mxg-_23M18_F_0	BJ986148
E_FL_mxg-_23N01_F_0	BJ986149
E_FL_mxg-_23N07_F_0	BJ986150
E_FL_mxg-_23N08_F_0	BJ986151
E_FL_mxg-_23N21_F_0	BJ986152
E_FL_mxg-_23N23_F_0	BJ986153
E_FL_mxg-_23O01_F_0	BJ986154
E_FL_mxg-_23O02_F_0	BJ986155
E_FL_mxg-_23O05_F_0	BJ986156
E_FL_mxg-_23O07_F_0	BJ986157
E_FL_mxg-_23O15_F_0	BJ986158
E_FL_mxg-_23P02_F_0	BJ986159
E_FL_mxg-_23P03_F_0	BJ986160
E_FL_mxg-_23P05_F_0	BJ986161
E_FL_mxg-_23P06_F_0	BJ986162
E_FL_mxg-_23P09_F_0	BJ986163
E_FL_mxg-_23P10_F_0	BJ986164
E_FL_mxg-_23P12_F_0	BJ986165
E_FL_mxg-_23P13_F_0	BJ986166
E_FL_mxg-_23P17_F_0	BJ986167
E_FL_mxg-_23P19_F_0	BJ986168
E_FL_mxg-_23P20_F_0	BJ986169
E_FL_mxg-_24A09_F_0	BJ986170
E_FL_mxg-_24A10_F_0	BJ986171
E_FL_mxg-_24A12_F_0	BJ986172
E_FL_mxg-_24A18_F_0	BJ986173
E_FL_mxg-_24B06_F_0	BJ986174
E_FL_mxg-_24B08_F_0	BJ986175
E_FL_mxg-_24B10_F_0	BJ986176
E_FL_mxg-_24B18_F_0	BJ986177
E_FL_mxg-_24C19_F_0	BJ986178
E_FL_mxg-_24D09_F_0	BJ986179
E_FL_mxg-_24D10_F_0	BJ986180
E_FL_mxg-_24G24_F_0	BJ986181
E_FL_mxg-_24I21_F_0	BJ986182
E_FL_mxg-_24J22_F_0	BJ986183
E_FL_mxg-_24J23_F_0	BJ986184
E_FL_mxg-_24J24_F_0	BJ986185
E_FL_mxg-_24M02_F_0	BJ986186
E_FL_mxg-_24M03_F_0	BJ986187
E_FL_mxg-_24O01_F_0	BJ986188
E_FL_mxg-_24O18_F_0	BJ986189
E_FL_mxg-_24O21_F_0	BJ986190
E_FL_mxg-_24O22_F_0	BJ986191
E_FL_mxg-_24P22_F_0	BJ986192

E_FL_mxg-_25A05_F_0	BJ986193
E_FL_mxg-_25A19_F_0	BJ986194
E_FL_mxg-_25A21_F_0	BJ986195
E_FL_mxg-_25B03_F_0	BJ986196
E_FL_mxg-_25B04_F_0	BJ986197
E_FL_mxg-_25B06_F_0	BJ986198
E_FL_mxg-_25B09_F_0	BJ986199
E_FL_mxg-_25B10_F_0	BJ986200
E_FL_mxg-_25B18_F_0	BJ986201
E_FL_mxg-_25C17_F_0	BJ986202
E_FL_mxg-_25E15_F_0	BJ986203
E_FL_mxg-_25F09_F_0	BJ986204
E_FL_mxg-_25F11_F_0	BJ986205
E_FL_mxg-_25G11_F_0	BJ986206
E_FL_mxg-_25G24_F_0	BJ986207
E_FL_mxg-_25I01_F_0	BJ986208
E_FL_mxg-_25I11_F_0	BJ986209
E_FL_mxg-_25I13_F_0	BJ986210
E_FL_mxg-_25I19_F_0	BJ986211
E_FL_mxg-_25I21_F_0	BJ986212
E_FL_mxg-_25J24_F_0	BJ986213
E_FL_mxg-_25K22_F_0	BJ986214
E_FL_mxg-_25L21_F_0	BJ986215
E_FL_mxg-_25L22_F_0	BJ986216
E_FL_mxg-_25M03_F_0	BJ986217
E_FL_mxg-_25M07_F_0	BJ986218
E_FL_mxg-_25N15_F_0	BJ986219
E_FL_mxg-_25O03_F_0	BJ986220
E_FL_mxg-_25O15_F_0	BJ986221
E_FL_mxg-_25O17_F_0	BJ986222
E_FL_mxg-_25O21_F_0	BJ986223
E_FL_mxg-_25P21_F_0	BJ986224
E_FL_mxg-_25P22_F_0	BJ986225
E_FL_mxg-_26A03_F_0	BJ986226
E_FL_mxg-_26A06_F_0	BJ986227
E_FL_mxg-_26A10_F_0	BJ986228
E_FL_mxg-_26B05_F_0	BJ986229
E_FL_mxg-_26B06_F_0	BJ986230
E_FL_mxg-_26B09_F_0	BJ986231
E_FL_mxg-_26B10_F_0	BJ986232
E_FL_mxg-_26D09_F_0	BJ986233
E_FL_mxg-_26F12_F_0	BJ986234
E_FL_mxg-_26G11_F_0	BJ986235
E_FL_mxg-_26I18_F_0	BJ986236
E_FL_mxg-_26I21_F_0	BJ986237
E_FL_mxg-_26K22_F_0	BJ986238
E_FL_mxg-_26L09_F_0	BJ986239
E_FL_mxg-_26L15_F_0	BJ986240
E_FL_mxg-_26M22_F_0	BJ986241
E_FL_mxg-_26N03_F_0	BJ986242
E_FL_mxg-_26O17_F_0	BJ986243
E_FL_mxg-_26O22_F_0	BJ986244
E_FL_mxg-_26P01_F_0	BJ986245
E_FL_mxg-_26P02_F_0	BJ986246

	E_FL_mxg-_26P09_F_0	BJ986247
	E_FL_mxg-_27A03_F_0	BJ986248
	E_FL_mxg-_27A05_F_0	BJ986249
	E_FL_mxg-_27A10_F_0	BJ986250
	E_FL_mxg-_27A11_F_0	BJ986251
	E_FL_mxg-_27A18_F_0	BJ986252
	E_FL_mxg-_27B11_F_0	BJ986253
	E_FL_mxg-_27B17_F_0	BJ986254
	E_FL_mxg-_27C10_F_0	BJ986255
	E_FL_mxg-_27E22_F_0	BJ986256
	E_FL_mxg-_27G13_F_0	BJ986257
	E_FL_mxg-_27G21_F_0	BJ986258
	E_FL_mxg-_27G22_F_0	BJ986259
	E_FL_mxg-_27H12_F_0	BJ986260
	E_FL_mxg-_27H19_F_0	BJ986261
	E_FL_mxg-_27H21_F_0	BJ986262
	E_FL_mxg-_27H22_F_0	BJ986263
	E_FL_mxg-_27J01_F_0	BJ986264
	E_FL_mxg-_27J17_F_0	BJ986265
	E_FL_mxg-_27J21_F_0	BJ986266
	E_FL_mxg-_27J22_F_0	BJ986267
	E_FL_mxg-_27K21_F_0	BJ986268
	E_FL_mxg-_27L22_F_0	BJ986269
	E_FL_mxg-_27M09_F_0	BJ986270
	E_FL_mxg-_27N10_F_0	BJ986271
	E_FL_mxg-_27N11_F_0	BJ986272
	E_FL_mxg-_27O01_F_0	BJ986273
	E_FL_mxg-_27O11_F_0	BJ986274
	E_FL_mxg-_27O20_F_0	BJ986275
	E_FL_mxg-_27O21_F_0	BJ986276
	E_FL_mxg-_27P16_F_0	BJ986277
	E_FL_mxg-_27P17_F_0	BJ986278
	E_FL_mxg-_27P21_F_0	BJ986279
prW-	E_FL_prW-_01A03_F_0	BY934602
	E_FL_prW-_01A04_F_0	BY934603
	E_FL_prW-_01A05_F_0	BY934604
	E_FL_prW-_01A06_F_0	BY934605
	E_FL_prW-_01A08_F_0	BY934606
	E_FL_prW-_01A09_F_0	BY934607
	E_FL_prW-_01A10_F_0	BY934608
	E_FL_prW-_01A11_F_0	BY934609
	E_FL_prW-_01A12_F_0	BY934610
	E_FL_prW-_01A15_F_0	BY934611
	E_FL_prW-_01A16_F_0	BY934612
	E_FL_prW-_01A17_F_0	BY934613
	E_FL_prW-_01A19_F_0	BY934614
	E_FL_prW-_01A21_F_0	BY934615
	E_FL_prW-_01A23_F_0	BY934616
	E_FL_prW-_01A24_F_0	BY934617
	E_FL_prW-_01B01_F_0	BY934618
	E_FL_prW-_01B03_F_0	BY934619
	E_FL_prW-_01B05_F_0	BY934620
	E_FL_prW-_01B07_F_0	BY934621
	E_FL_prW-_01B08_F_0	BY934622

E_FL_prW-_01B11_F_0	BY934623
E_FL_prW-_01B12_F_0	BY934624
E_FL_prW-_01B13_F_0	BY934625
E_FL_prW-_01B14_F_0	BY934626
E_FL_prW-_01B15_F_0	BY934627
E_FL_prW-_01B16_F_0	BY934628
E_FL_prW-_01B17_F_0	BY934629
E_FL_prW-_01B18_F_0	BY934630
E_FL_prW-_01B19_F_0	BY934631
E_FL_prW-_01B20_F_0	BY934632
E_FL_prW-_01B21_F_0	BY934633
E_FL_prW-_01B22_F_0	BY934634
E_FL_prW-_01C05_F_0	BY934635
E_FL_prW-_01C09_F_0	BY934636
E_FL_prW-_01C11_F_0	BY934637
E_FL_prW-_01C13_F_0	BY934638
E_FL_prW-_01C14_F_0	BY934639
E_FL_prW-_01C16_F_0	BY934640
E_FL_prW-_01C17_F_0	BY934641
E_FL_prW-_01C18_F_0	BY934642
E_FL_prW-_01C19_F_0	BY934643
E_FL_prW-_01C21_F_0	BY934644
E_FL_prW-_01D01_F_0	BY934645
E_FL_prW-_01D05_F_0	BY934646
E_FL_prW-_01D06_F_0	BY934647
E_FL_prW-_01D08_F_0	BY934648
E_FL_prW-_01D09_F_0	BY934649
E_FL_prW-_01D10_F_0	BY934650
E_FL_prW-_01D11_F_0	BY934651
E_FL_prW-_01D13_F_0	BY934652
E_FL_prW-_01D14_F_0	BY934653
E_FL_prW-_01D15_F_0	BY934654
E_FL_prW-_01D17_F_0	BY934655
E_FL_prW-_01D18_F_0	BY934656
E_FL_prW-_01D19_F_0	BY934657
E_FL_prW-_01D21_F_0	BY934658
E_FL_prW-_01D22_F_0	BY934659
E_FL_prW-_01E02_F_0	BY934660
E_FL_prW-_01E03_F_0	BY934661
E_FL_prW-_01E04_F_0	BY934662
E_FL_prW-_01E05_F_0	BY934663
E_FL_prW-_01E06_F_0	BY934664
E_FL_prW-_01E09_F_0	BY934665
E_FL_prW-_01E11_F_0	BY934666
E_FL_prW-_01E13_F_0	BY934667
E_FL_prW-_01E15_F_0	BY934668
E_FL_prW-_01E17_F_0	BY934669
E_FL_prW-_01E18_F_0	BY934670
E_FL_prW-_01E20_F_0	BY934671
E_FL_prW-_01E22_F_0	BY934672
E_FL_prW-_01E24_F_0	BY934673
E_FL_prW-_01F04_F_0	BY934674
E_FL_prW-_01F06_F_0	BY934675
E_FL_prW-_01F07_F_0	BY934676

E_FL_prW-_01F08_F_0	BY934677
E_FL_prW-_01F10_F_0	BY934678
E_FL_prW-_01F11_F_0	BY934679
E_FL_prW-_01F13_F_0	BY934680
E_FL_prW-_01F14_F_0	BY934681
E_FL_prW-_01F17_F_0	BY934682
E_FL_prW-_01F19_F_0	BY934683
E_FL_prW-_01F20_F_0	BY934684
E_FL_prW-_01F22_F_0	BY934685
E_FL_prW-_01F23_F_0	BY934686
E_FL_prW-_01F24_F_0	BY934687
E_FL_prW-_01G01_F_0	BY934688
E_FL_prW-_01G02_F_0	BY934689
E_FL_prW-_01G03_F_0	BY934690
E_FL_prW-_01G05_F_0	BY934691
E_FL_prW-_01G06_F_0	BY934692
E_FL_prW-_01G07_F_0	BY934693
E_FL_prW-_01G08_F_0	BY934694
E_FL_prW-_01G09_F_0	BY934695
E_FL_prW-_01G11_F_0	BY934696
E_FL_prW-_01G13_F_0	BY934697
E_FL_prW-_01G14_F_0	BY934698
E_FL_prW-_01G16_F_0	BY934699
E_FL_prW-_01G17_F_0	BY934700
E_FL_prW-_01G19_F_0	BY934701
E_FL_prW-_01G21_F_0	BY934702
E_FL_prW-_01G22_F_0	BY934703
E_FL_prW-_01G23_F_0	BY934704
E_FL_prW-_01H01_F_0	BY934705
E_FL_prW-_01H03_F_0	BY934706
E_FL_prW-_01H04_F_0	BY934707
E_FL_prW-_01H06_F_0	BY934708
E_FL_prW-_01H08_F_0	BY934709
E_FL_prW-_01H11_F_0	BY934710
E_FL_prW-_01H15_F_0	BY934711
E_FL_prW-_01H18_F_0	BY934712
E_FL_prW-_01H19_F_0	BY934713
E_FL_prW-_01H20_F_0	BY934714
E_FL_prW-_01H21_F_0	BY934715
E_FL_prW-_01H24_F_0	BY934716
E_FL_prW-_01I01_F_0	BY934717
E_FL_prW-_01I05_F_0	BY934718
E_FL_prW-_01I06_F_0	BY934719
E_FL_prW-_01I08_F_0	BY934720
E_FL_prW-_01I09_F_0	BY934721
E_FL_prW-_01I10_F_0	BY934722
E_FL_prW-_01I17_F_0	BY934723
E_FL_prW-_01I18_F_0	BY934724
E_FL_prW-_01I21_F_0	BY934725
E_FL_prW-_01I22_F_0	BY934726
E_FL_prW-_01J04_F_0	BY934727
E_FL_prW-_01J08_F_0	BY934728
E_FL_prW-_01J09_F_0	BY934729
E_FL_prW-_01J10_F_0	BY934730

E_FL_prW-_01J11_F_0	BY934731
E_FL_prW-_01J12_F_0	BY934732
E_FL_prW-_01J13_F_0	BY934733
E_FL_prW-_01J15_F_0	BY934734
E_FL_prW-_01J16_F_0	BY934735
E_FL_prW-_01J22_F_0	BY934736
E_FL_prW-_01J23_F_0	BY934737
E_FL_prW-_01K02_F_0	BY934738
E_FL_prW-_01K03_F_0	BY934739
E_FL_prW-_01K04_F_0	BY934740
E_FL_prW-_01K05_F_0	BY934741
E_FL_prW-_01K06_F_0	BY934742
E_FL_prW-_01K07_F_0	BY934743
E_FL_prW-_01K08_F_0	BY934744
E_FL_prW-_01K09_F_0	BY934745
E_FL_prW-_01K10_F_0	BY934746
E_FL_prW-_01K11_F_0	BY934747
E_FL_prW-_01K13_F_0	BY934748
E_FL_prW-_01K14_F_0	BY934749
E_FL_prW-_01K15_F_0	BY934750
E_FL_prW-_01K17_F_0	BY934751
E_FL_prW-_01K18_F_0	BY934752
E_FL_prW-_01K19_F_0	BY934753
E_FL_prW-_01K23_F_0	BY934754
E_FL_prW-_01L06_F_0	BY934755
E_FL_prW-_01L07_F_0	BY934756
E_FL_prW-_01L09_F_0	BY934757
E_FL_prW-_01L12_F_0	BY934758
E_FL_prW-_01L13_F_0	BY934759
E_FL_prW-_01L15_F_0	BY934760
E_FL_prW-_01L16_F_0	BY934761
E_FL_prW-_01L18_F_0	BY934762
E_FL_prW-_01L19_F_0	BY934763
E_FL_prW-_01L21_F_0	BY934764
E_FL_prW-_01L22_F_0	BY934765
E_FL_prW-_01L23_F_0	BY934766
E_FL_prW-_01M02_F_0	BY934767
E_FL_prW-_01M04_F_0	BY934768
E_FL_prW-_01M05_F_0	BY934769
E_FL_prW-_01M08_F_0	BY934770
E_FL_prW-_01M11_F_0	BY934771
E_FL_prW-_01M15_F_0	BY934772
E_FL_prW-_01M16_F_0	BY934773
E_FL_prW-_01M21_F_0	BY934774
E_FL_prW-_01M22_F_0	BY934775
E_FL_prW-_01M24_F_0	BY934776
E_FL_prW-_01N01_F_0	BY934777
E_FL_prW-_01N04_F_0	BY934778
E_FL_prW-_01N08_F_0	BY934779
E_FL_prW-_01N09_F_0	BY934780
E_FL_prW-_01N12_F_0	BY934781
E_FL_prW-_01N13_F_0	BY934782
E_FL_prW-_01N14_F_0	BY934783
E_FL_prW-_01N15_F_0	BY934784

E_FL_prW-_01N17_F_0	BY934785
E_FL_prW-_01N21_F_0	BY934786
E_FL_prW-_01N23_F_0	BY934787
E_FL_prW-_01N24_F_0	BY934788
E_FL_prW-_01O01_F_0	BY934789
E_FL_prW-_01O03_F_0	BY934790
E_FL_prW-_01O06_F_0	BY934791
E_FL_prW-_01O08_F_0	BY934792
E_FL_prW-_01O11_F_0	BY934793
E_FL_prW-_01O12_F_0	BY934794
E_FL_prW-_01O13_F_0	BY934795
E_FL_prW-_01O14_F_0	BY934796
E_FL_prW-_01O16_F_0	BY934797
E_FL_prW-_01O20_F_0	BY934798
E_FL_prW-_01O22_F_0	BY934799
E_FL_prW-_01O23_F_0	BY934800
E_FL_prW-_01P01_F_0	BY934801
E_FL_prW-_01P03_F_0	BY934802
E_FL_prW-_01P04_F_0	BY934803
E_FL_prW-_01P05_F_0	BY934804
E_FL_prW-_01P06_F_0	BY934805
E_FL_prW-_01P08_F_0	BY934806
E_FL_prW-_01P10_F_0	BY934807
E_FL_prW-_01P14_F_0	BY934808
E_FL_prW-_01P15_F_0	BY934809
E_FL_prW-_01P17_F_0	BY934810
E_FL_prW-_01P19_F_0	BY934811
E_FL_prW-_01P20_F_0	BY934812
E_FL_prW-_01P21_F_0	BY934813
E_FL_prW-_02A05_F_0	BY934814
E_FL_prW-_02A06_F_0	BY934815
E_FL_prW-_02A08_F_0	BY934816
E_FL_prW-_02A10_F_0	BY934817
E_FL_prW-_02A14_F_0	BY934818
E_FL_prW-_02A16_F_0	BY934819
E_FL_prW-_02A17_F_0	BY934820
E_FL_prW-_02A19_F_0	BY934821
E_FL_prW-_02A20_F_0	BY934822
E_FL_prW-_02A21_F_0	BY934823
E_FL_prW-_02A23_F_0	BY934824
E_FL_prW-_02B03_F_0	BY934825
E_FL_prW-_02B05_F_0	BY934826
E_FL_prW-_02B07_F_0	BY934827
E_FL_prW-_02B08_F_0	BY934828
E_FL_prW-_02B09_F_0	BY934829
E_FL_prW-_02B10_F_0	BY934830
E_FL_prW-_02B11_F_0	BY934831
E_FL_prW-_02B13_F_0	BY934832
E_FL_prW-_02B15_F_0	BY934833
E_FL_prW-_02B18_F_0	BY934834
E_FL_prW-_02B19_F_0	BY934835
E_FL_prW-_02B20_F_0	BY934836
E_FL_prW-_02B21_F_0	BY934837
E_FL_prW-_02B22_F_0	BY934838

E_FL_prW-_02C01_F_0	BY934839
E_FL_prW-_02C04_F_0	BY934840
E_FL_prW-_02C07_F_0	BY934841
E_FL_prW-_02C08_F_0	BY934842
E_FL_prW-_02C10_F_0	BY934843
E_FL_prW-_02C12_F_0	BY934844
E_FL_prW-_02C13_F_0	BY934845
E_FL_prW-_02C15_F_0	BY934846
E_FL_prW-_02C17_F_0	BY934847
E_FL_prW-_02C19_F_0	BY934848
E_FL_prW-_02C21_F_0	BY934849
E_FL_prW-_02C22_F_0	BY934850
E_FL_prW-_02D02_F_0	BY934851
E_FL_prW-_02D04_F_0	BY934852
E_FL_prW-_02D05_F_0	BY934853
E_FL_prW-_02D07_F_0	BY934854
E_FL_prW-_02D08_F_0	BY934855
E_FL_prW-_02D10_F_0	BY934856
E_FL_prW-_02D11_F_0	BY934857
E_FL_prW-_02D13_F_0	BY934858
E_FL_prW-_02D14_F_0	BY934859
E_FL_prW-_02D15_F_0	BY934860
E_FL_prW-_02D16_F_0	BY934861
E_FL_prW-_02D17_F_0	BY934862
E_FL_prW-_02D19_F_0	BY934863
E_FL_prW-_02D21_F_0	BY934864
E_FL_prW-_02E02_F_0	BY934865
E_FL_prW-_02E05_F_0	BY934866
E_FL_prW-_02E06_F_0	BY934867
E_FL_prW-_02E08_F_0	BY934868
E_FL_prW-_02E10_F_0	BY934869
E_FL_prW-_02E11_F_0	BY934870
E_FL_prW-_02E13_F_0	BY934871
E_FL_prW-_02E14_F_0	BY934872
E_FL_prW-_02E16_F_0	BY934873
E_FL_prW-_02E19_F_0	BY934874
E_FL_prW-_02E20_F_0	BY934875
E_FL_prW-_02E22_F_0	BY934876
E_FL_prW-_02E23_F_0	BY934877
E_FL_prW-_02F02_F_0	BY934878
E_FL_prW-_02F05_F_0	BY934879
E_FL_prW-_02F08_F_0	BY934880
E_FL_prW-_02F09_F_0	BY934881
E_FL_prW-_02F11_F_0	BY934882
E_FL_prW-_02F12_F_0	BY934883
E_FL_prW-_02F15_F_0	BY934884
E_FL_prW-_02F16_F_0	BY934885
E_FL_prW-_02F17_F_0	BY934886
E_FL_prW-_02F20_F_0	BY934887
E_FL_prW-_02F21_F_0	BY934888
E_FL_prW-_02F22_F_0	BY934889
E_FL_prW-_02F23_F_0	BY934890
E_FL_prW-_02F24_F_0	BY934891
E_FL_prW-_02G01_F_0	BY934892

E_FL_prW-_02G02_F_0	BY934893
E_FL_prW-_02G03_F_0	BY934894
E_FL_prW-_02G05_F_0	BY934895
E_FL_prW-_02G07_F_0	BY934896
E_FL_prW-_02G08_F_0	BY934897
E_FL_prW-_02G09_F_0	BY934898
E_FL_prW-_02G14_F_0	BY934899
E_FL_prW-_02G16_F_0	BY934900
E_FL_prW-_02G20_F_0	BY934901
E_FL_prW-_02G21_F_0	BY934902
E_FL_prW-_02G22_F_0	BY934903
E_FL_prW-_02G23_F_0	BY934904
E_FL_prW-_02H02_F_0	BY934905
E_FL_prW-_02H03_F_0	BY934906
E_FL_prW-_02H04_F_0	BY934907
E_FL_prW-_02H05_F_0	BY934908
E_FL_prW-_02H06_F_0	BY934909
E_FL_prW-_02H07_F_0	BY934910
E_FL_prW-_02H08_F_0	BY934911
E_FL_prW-_02H10_F_0	BY934912
E_FL_prW-_02H11_F_0	BY934913
E_FL_prW-_02H14_F_0	BY934914
E_FL_prW-_02H15_F_0	BY934915
E_FL_prW-_02H16_F_0	BY934916
E_FL_prW-_02H21_F_0	BY934917
E_FL_prW-_02H22_F_0	BY934918
E_FL_prW-_02H23_F_0	BY934919
E_FL_prW-_02H24_F_0	BY934920
E_FL_prW-_02I01_F_0	BY934921
E_FL_prW-_02I03_F_0	BY934922
E_FL_prW-_02I04_F_0	BY934923
E_FL_prW-_02I05_F_0	BY934924
E_FL_prW-_02I08_F_0	BY934925
E_FL_prW-_02I09_F_0	BY934926
E_FL_prW-_02I10_F_0	BY934927
E_FL_prW-_02I11_F_0	BY934928
E_FL_prW-_02I12_F_0	BY934929
E_FL_prW-_02I13_F_0	BY934930
E_FL_prW-_02I15_F_0	BY934931
E_FL_prW-_02I18_F_0	BY934932
E_FL_prW-_02I21_F_0	BY934933
E_FL_prW-_02I23_F_0	BY934934
E_FL_prW-_02J01_F_0	BY934935
E_FL_prW-_02J03_F_0	BY934936
E_FL_prW-_02J04_F_0	BY934937
E_FL_prW-_02J05_F_0	BY934938
E_FL_prW-_02J06_F_0	BY934939
E_FL_prW-_02J08_F_0	BY934940
E_FL_prW-_02J10_F_0	BY934941
E_FL_prW-_02J15_F_0	BY934942
E_FL_prW-_02J22_F_0	BY934943
E_FL_prW-_02J23_F_0	BY934944
E_FL_prW-_02J24_F_0	BY934945
E_FL_prW-_02K01_F_0	BY934946

E_FL_prW-_02K05_F_0	BY934947
E_FL_prW-_02K06_F_0	BY934948
E_FL_prW-_02K07_F_0	BY934949
E_FL_prW-_02K09_F_0	BY934950
E_FL_prW-_02K10_F_0	BY934951
E_FL_prW-_02K11_F_0	BY934952
E_FL_prW-_02K13_F_0	BY934953
E_FL_prW-_02K14_F_0	BY934954
E_FL_prW-_02K19_F_0	BY934955
E_FL_prW-_02K21_F_0	BY934956
E_FL_prW-_02K23_F_0	BY934957
E_FL_prW-_02L03_F_0	BY934958
E_FL_prW-_02L04_F_0	BY934959
E_FL_prW-_02L05_F_0	BY934960
E_FL_prW-_02L07_F_0	BY934961
E_FL_prW-_02L08_F_0	BY934962
E_FL_prW-_02L09_F_0	BY934963
E_FL_prW-_02L13_F_0	BY934964
E_FL_prW-_02L14_F_0	BY934965
E_FL_prW-_02L16_F_0	BY934966
E_FL_prW-_02L17_F_0	BY934967
E_FL_prW-_02L22_F_0	BY934968
E_FL_prW-_02L23_F_0	BY934969
E_FL_prW-_02L24_F_0	BY934970
E_FL_prW-_02M03_F_0	BY934971
E_FL_prW-_02M05_F_0	BY934972
E_FL_prW-_02M09_F_0	BY934973
E_FL_prW-_02M10_F_0	BY934974
E_FL_prW-_02M11_F_0	BY934975
E_FL_prW-_02M12_F_0	BY934976
E_FL_prW-_02M13_F_0	BY934977
E_FL_prW-_02M15_F_0	BY934978
E_FL_prW-_02M16_F_0	BY934979
E_FL_prW-_02M20_F_0	BY934980
E_FL_prW-_02M21_F_0	BY934981
E_FL_prW-_02M23_F_0	BY934982
E_FL_prW-_02M24_F_0	BY934983
E_FL_prW-_02N04_F_0	BY934984
E_FL_prW-_02N05_F_0	BY934985
E_FL_prW-_02N07_F_0	BY934986
E_FL_prW-_02N08_F_0	BY934987
E_FL_prW-_02N09_F_0	BY934988
E_FL_prW-_02N11_F_0	BY934989
E_FL_prW-_02N12_F_0	BY934990
E_FL_prW-_02N14_F_0	BY934991
E_FL_prW-_02N16_F_0	BY934992
E_FL_prW-_02N17_F_0	BY934993
E_FL_prW-_02N23_F_0	BY934994
E_FL_prW-_02N24_F_0	BY934995
E_FL_prW-_02O01_F_0	BY934996
E_FL_prW-_02O04_F_0	BY934997
E_FL_prW-_02O05_F_0	BY934998
E_FL_prW-_02O09_F_0	BY934999
E_FL_prW-_02O10_F_0	BY935000

E_FL_prW-_02O15_F_0	BY935001
E_FL_prW-_02O16_F_0	BY935002
E_FL_prW-_02O17_F_0	BY935003
E_FL_prW-_02O18_F_0	BY935004
E_FL_prW-_02O20_F_0	BY935005
E_FL_prW-_02O23_F_0	BY935006
E_FL_prW-_02P01_F_0	BY935007
E_FL_prW-_02P02_F_0	BY935008
E_FL_prW-_02P04_F_0	BY935009
E_FL_prW-_02P06_F_0	BY935010
E_FL_prW-_02P09_F_0	BY935011
E_FL_prW-_02P10_F_0	BY935012
E_FL_prW-_02P12_F_0	BY935013
E_FL_prW-_02P14_F_0	BY935014
E_FL_prW-_02P15_F_0	BY935015
E_FL_prW-_02P19_F_0	BY935016
E_FL_prW-_02P20_F_0	BY935017
E_FL_prW-_02P24_F_0	BY935018
E_FL_prW-_03A01_F_0	BY935019
E_FL_prW-_03A02_F_0	BY935020
E_FL_prW-_03A04_F_0	BY935021
E_FL_prW-_03A06_F_0	BY935022
E_FL_prW-_03A07_F_0	BY935023
E_FL_prW-_03A09_F_0	BY935024
E_FL_prW-_03A10_F_0	BY935025
E_FL_prW-_03A11_F_0	BY935026
E_FL_prW-_03A13_F_0	BY935027
E_FL_prW-_03A14_F_0	BY935028
E_FL_prW-_03A15_F_0	BY935029
E_FL_prW-_03A16_F_0	BY935030
E_FL_prW-_03A17_F_0	BY935031
E_FL_prW-_03A18_F_0	BY935032
E_FL_prW-_03A20_F_0	BY935033
E_FL_prW-_03A22_F_0	BY935034
E_FL_prW-_03A23_F_0	BY935035
E_FL_prW-_03B01_F_0	BY935036
E_FL_prW-_03B02_F_0	BY935037
E_FL_prW-_03B03_F_0	BY935038
E_FL_prW-_03B04_F_0	BY935039
E_FL_prW-_03B05_F_0	BY935040
E_FL_prW-_03B06_F_0	BY935041
E_FL_prW-_03B08_F_0	BY935042
E_FL_prW-_03B09_F_0	BY935043
E_FL_prW-_03B11_F_0	BY935044
E_FL_prW-_03B13_F_0	BY935045
E_FL_prW-_03B14_F_0	BY935046
E_FL_prW-_03B17_F_0	BY935047
E_FL_prW-_03B18_F_0	BY935048
E_FL_prW-_03B19_F_0	BY935049
E_FL_prW-_03B21_F_0	BY935050
E_FL_prW-_03B22_F_0	BY935051
E_FL_prW-_03B24_F_0	BY935052
E_FL_prW-_03C02_F_0	BY935053
E_FL_prW-_03C05_F_0	BY935054

E_FL_prW-_03C07_F_0	BY935055
E_FL_prW-_03C10_F_0	BY935056
E_FL_prW-_03C11_F_0	BY935057
E_FL_prW-_03C12_F_0	BY935058
E_FL_prW-_03C13_F_0	BY935059
E_FL_prW-_03C14_F_0	BY935060
E_FL_prW-_03C15_F_0	BY935061
E_FL_prW-_03C16_F_0	BY935062
E_FL_prW-_03C19_F_0	BY935063
E_FL_prW-_03C21_F_0	BY935064
E_FL_prW-_03C23_F_0	BY935065
E_FL_prW-_03D01_F_0	BY935066
E_FL_prW-_03D02_F_0	BY935067
E_FL_prW-_03D03_F_0	BY935068
E_FL_prW-_03D04_F_0	BY935069
E_FL_prW-_03D05_F_0	BY935070
E_FL_prW-_03D06_F_0	BY935071
E_FL_prW-_03D07_F_0	BY935072
E_FL_prW-_03D11_F_0	BY935073
E_FL_prW-_03D12_F_0	BY935074
E_FL_prW-_03D13_F_0	BY935075
E_FL_prW-_03D16_F_0	BY935076
E_FL_prW-_03D17_F_0	BY935077
E_FL_prW-_03D21_F_0	BY935078
E_FL_prW-_03D23_F_0	BY935079
E_FL_prW-_03E04_F_0	BY935080
E_FL_prW-_03E05_F_0	BY935081
E_FL_prW-_03E09_F_0	BY935082
E_FL_prW-_03E10_F_0	BY935083
E_FL_prW-_03E11_F_0	BY935084
E_FL_prW-_03E12_F_0	BY935085
E_FL_prW-_03E13_F_0	BY935086
E_FL_prW-_03E14_F_0	BY935087
E_FL_prW-_03E15_F_0	BY935088
E_FL_prW-_03E16_F_0	BY935089
E_FL_prW-_03E17_F_0	BY935090
E_FL_prW-_03E18_F_0	BY935091
E_FL_prW-_03E21_F_0	BY935092
E_FL_prW-_03E22_F_0	BY935093
E_FL_prW-_03E24_F_0	BY935094
E_FL_prW-_03F01_F_0	BY935095
E_FL_prW-_03F05_F_0	BY935096
E_FL_prW-_03F06_F_0	BY935097
E_FL_prW-_03F09_F_0	BY935098
E_FL_prW-_03F14_F_0	BY935099
E_FL_prW-_03F15_F_0	BY935100
E_FL_prW-_03F23_F_0	BY935101
E_FL_prW-_03F24_F_0	BY935102
E_FL_prW-_03G02_F_0	BY935103
E_FL_prW-_03G03_F_0	BY935104
E_FL_prW-_03G04_F_0	BY935105
E_FL_prW-_03G08_F_0	BY935106
E_FL_prW-_03G09_F_0	BY935107
E_FL_prW-_03G11_F_0	BY935108

E_FL_prW-_03G12_F_0	BY935109
E_FL_prW-_03G14_F_0	BY935110
E_FL_prW-_03G15_F_0	BY935111
E_FL_prW-_03G19_F_0	BY935112
E_FL_prW-_03G20_F_0	BY935113
E_FL_prW-_03G22_F_0	BY935114
E_FL_prW-_03G24_F_0	BY935115
E_FL_prW-_03H01_F_0	BY935116
E_FL_prW-_03H04_F_0	BY935117
E_FL_prW-_03H05_F_0	BY935118
E_FL_prW-_03H06_F_0	BY935119
E_FL_prW-_03H09_F_0	BY935120
E_FL_prW-_03H10_F_0	BY935121
E_FL_prW-_03H11_F_0	BY935122
E_FL_prW-_03H12_F_0	BY935123
E_FL_prW-_03H13_F_0	BY935124
E_FL_prW-_03H14_F_0	BY935125
E_FL_prW-_03H17_F_0	BY935126
E_FL_prW-_03H18_F_0	BY935127
E_FL_prW-_03H21_F_0	BY935128
E_FL_prW-_03H22_F_0	BY935129
E_FL_prW-_03H23_F_0	BY935130
E_FL_prW-_03I04_F_0	BY935131
E_FL_prW-_03I05_F_0	BY935132
E_FL_prW-_03I06_F_0	BY935133
E_FL_prW-_03I10_F_0	BY935134
E_FL_prW-_03I11_F_0	BY935135
E_FL_prW-_03I13_F_0	BY935136
E_FL_prW-_03I15_F_0	BY935137
E_FL_prW-_03I16_F_0	BY935138
E_FL_prW-_03I17_F_0	BY935139
E_FL_prW-_03I19_F_0	BY935140
E_FL_prW-_03I20_F_0	BY935141
E_FL_prW-_03I21_F_0	BY935142
E_FL_prW-_03I24_F_0	BY935143
E_FL_prW-_03J01_F_0	BY935144
E_FL_prW-_03J02_F_0	BY935145
E_FL_prW-_03J04_F_0	BY935146
E_FL_prW-_03J05_F_0	BY935147
E_FL_prW-_03J06_F_0	BY935148
E_FL_prW-_03J08_F_0	BY935149
E_FL_prW-_03J09_F_0	BY935150
E_FL_prW-_03J10_F_0	BY935151
E_FL_prW-_03J11_F_0	BY935152
E_FL_prW-_03J15_F_0	BY935153
E_FL_prW-_03J17_F_0	BY935154
E_FL_prW-_03J19_F_0	BY935155
E_FL_prW-_03J21_F_0	BY935156
E_FL_prW-_03J22_F_0	BY935157
E_FL_prW-_03K01_F_0	BY935158
E_FL_prW-_03K02_F_0	BY935159
E_FL_prW-_03K03_F_0	BY935160
E_FL_prW-_03K06_F_0	BY935161
E_FL_prW-_03K07_F_0	BY935162

E_FL_prW-_03K08_F_0	BY935163
E_FL_prW-_03K09_F_0	BY935164
E_FL_prW-_03K11_F_0	BY935165
E_FL_prW-_03K12_F_0	BY935166
E_FL_prW-_03K15_F_0	BY935167
E_FL_prW-_03K16_F_0	BY935168
E_FL_prW-_03K17_F_0	BY935169
E_FL_prW-_03K23_F_0	BY935170
E_FL_prW-_03L02_F_0	BY935171
E_FL_prW-_03L03_F_0	BY935172
E_FL_prW-_03L05_F_0	BY935173
E_FL_prW-_03L06_F_0	BY935174
E_FL_prW-_03L07_F_0	BY935175
E_FL_prW-_03L08_F_0	BY935176
E_FL_prW-_03L09_F_0	BY935177
E_FL_prW-_03L10_F_0	BY935178
E_FL_prW-_03L12_F_0	BY935179
E_FL_prW-_03L15_F_0	BY935180
E_FL_prW-_03L16_F_0	BY935181
E_FL_prW-_03L17_F_0	BY935182
E_FL_prW-_03L18_F_0	BY935183
E_FL_prW-_03L19_F_0	BY935184
E_FL_prW-_03L22_F_0	BY935185
E_FL_prW-_03L24_F_0	BY935186
E_FL_prW-_03M03_F_0	BY935187
E_FL_prW-_03M05_F_0	BY935188
E_FL_prW-_03M06_F_0	BY935189
E_FL_prW-_03M07_F_0	BY935190
E_FL_prW-_03M08_F_0	BY935191
E_FL_prW-_03M09_F_0	BY935192
E_FL_prW-_03M10_F_0	BY935193
E_FL_prW-_03M12_F_0	BY935194
E_FL_prW-_03M15_F_0	BY935195
E_FL_prW-_03M16_F_0	BY935196
E_FL_prW-_03M19_F_0	BY935197
E_FL_prW-_03M24_F_0	BY935198
E_FL_prW-_03N01_F_0	BY935199
E_FL_prW-_03N02_F_0	BY935200
E_FL_prW-_03N03_F_0	BY935201
E_FL_prW-_03N08_F_0	BY935202
E_FL_prW-_03N09_F_0	BY935203
E_FL_prW-_03N10_F_0	BY935204
E_FL_prW-_03N13_F_0	BY935205
E_FL_prW-_03N14_F_0	BY935206
E_FL_prW-_03N17_F_0	BY935207
E_FL_prW-_03N19_F_0	BY935208
E_FL_prW-_03N20_F_0	BY935209
E_FL_prW-_03N21_F_0	BY935210
E_FL_prW-_03N23_F_0	BY935211
E_FL_prW-_03O01_F_0	BY935212
E_FL_prW-_03O02_F_0	BY935213
E_FL_prW-_03O04_F_0	BY935214
E_FL_prW-_03O06_F_0	BY935215
E_FL_prW-_03O07_F_0	BY935216

E_FL_prW-_03O08_F_0	BY935217
E_FL_prW-_03O09_F_0	BY935218
E_FL_prW-_03O10_F_0	BY935219
E_FL_prW-_03O13_F_0	BY935220
E_FL_prW-_03O15_F_0	BY935221
E_FL_prW-_03O16_F_0	BY935222
E_FL_prW-_03O17_F_0	BY935223
E_FL_prW-_03O18_F_0	BY935224
E_FL_prW-_03O19_F_0	BY935225
E_FL_prW-_03O20_F_0	BY935226
E_FL_prW-_03O22_F_0	BY935227
E_FL_prW-_03P01_F_0	BY935228
E_FL_prW-_03P03_F_0	BY935229
E_FL_prW-_03P04_F_0	BY935230
E_FL_prW-_03P07_F_0	BY935231
E_FL_prW-_03P08_F_0	BY935232
E_FL_prW-_03P10_F_0	BY935233
E_FL_prW-_03P11_F_0	BY935234
E_FL_prW-_03P12_F_0	BY935235
E_FL_prW-_03P13_F_0	BY935236
E_FL_prW-_03P16_F_0	BY935237
E_FL_prW-_03P17_F_0	BY935238
E_FL_prW-_03P21_F_0	BY935239
E_FL_prW-_03P22_F_0	BY935240
E_FL_prW-_03P24_F_0	BY935241
E_FL_prW-_04A02_F_0	BY935242
E_FL_prW-_04A07_F_0	BY935243
E_FL_prW-_04A08_F_0	BY935244
E_FL_prW-_04A09_F_0	BY935245
E_FL_prW-_04A10_F_0	BY935246
E_FL_prW-_04A12_F_0	BY935247
E_FL_prW-_04A15_F_0	BY935248
E_FL_prW-_04A17_F_0	BY935249
E_FL_prW-_04A19_F_0	BY935250
E_FL_prW-_04A20_F_0	BY935251
E_FL_prW-_04A22_F_0	BY935252
E_FL_prW-_04A23_F_0	BY935253
E_FL_prW-_04A24_F_0	BY935254
E_FL_prW-_04B01_F_0	BY935255
E_FL_prW-_04B02_F_0	BY935256
E_FL_prW-_04B03_F_0	BY935257
E_FL_prW-_04B05_F_0	BY935258
E_FL_prW-_04B06_F_0	BY935259
E_FL_prW-_04B08_F_0	BY935260
E_FL_prW-_04B10_F_0	BY935261
E_FL_prW-_04B11_F_0	BY935262
E_FL_prW-_04B13_F_0	BY935263
E_FL_prW-_04B15_F_0	BY935264
E_FL_prW-_04B17_F_0	BY935265
E_FL_prW-_04B18_F_0	BY935266
E_FL_prW-_04B19_F_0	BY935267
E_FL_prW-_04B21_F_0	BY935268
E_FL_prW-_04B22_F_0	BY935269
E_FL_prW-_04C01_F_0	BY935270

E_FL_prW-_04C02_F_0	BY935271
E_FL_prW-_04C07_F_0	BY935272
E_FL_prW-_04C08_F_0	BY935273
E_FL_prW-_04C09_F_0	BY935274
E_FL_prW-_04C10_F_0	BY935275
E_FL_prW-_04C12_F_0	BY935276
E_FL_prW-_04C14_F_0	BY935277
E_FL_prW-_04C15_F_0	BY935278
E_FL_prW-_04C16_F_0	BY935279
E_FL_prW-_04C17_F_0	BY935280
E_FL_prW-_04C20_F_0	BY935281
E_FL_prW-_04C21_F_0	BY935282
E_FL_prW-_04D03_F_0	BY935283
E_FL_prW-_04D04_F_0	BY935284
E_FL_prW-_04D06_F_0	BY935285
E_FL_prW-_04D08_F_0	BY935286
E_FL_prW-_04D09_F_0	BY935287
E_FL_prW-_04D10_F_0	BY935288
E_FL_prW-_04D11_F_0	BY935289
E_FL_prW-_04D12_F_0	BY935290
E_FL_prW-_04D13_F_0	BY935291
E_FL_prW-_04D15_F_0	BY935292
E_FL_prW-_04D16_F_0	BY935293
E_FL_prW-_04D17_F_0	BY935294
E_FL_prW-_04D18_F_0	BY935295
E_FL_prW-_04D19_F_0	BY935296
E_FL_prW-_04D20_F_0	BY935297
E_FL_prW-_04D22_F_0	BY935298
E_FL_prW-_04D23_F_0	BY935299
E_FL_prW-_04E02_F_0	BY935300
E_FL_prW-_04E03_F_0	BY935301
E_FL_prW-_04E04_F_0	BY935302
E_FL_prW-_04E06_F_0	BY935303
E_FL_prW-_04E08_F_0	BY935304
E_FL_prW-_04E10_F_0	BY935305
E_FL_prW-_04E11_F_0	BY935306
E_FL_prW-_04E12_F_0	BY935307
E_FL_prW-_04E13_F_0	BY935308
E_FL_prW-_04E14_F_0	BY935309
E_FL_prW-_04E15_F_0	BY935310
E_FL_prW-_04E16_F_0	BY935311
E_FL_prW-_04E17_F_0	BY935312
E_FL_prW-_04E18_F_0	BY935313
E_FL_prW-_04E20_F_0	BY935314
E_FL_prW-_04E23_F_0	BY935315
E_FL_prW-_04F01_F_0	BY935316
E_FL_prW-_04F03_F_0	BY935317
E_FL_prW-_04F09_F_0	BY935318
E_FL_prW-_04F11_F_0	BY935319
E_FL_prW-_04F13_F_0	BY935320
E_FL_prW-_04F15_F_0	BY935321
E_FL_prW-_04F17_F_0	BY935322
E_FL_prW-_04F18_F_0	BY935323
E_FL_prW-_04F20_F_0	BY935324

E_FL_prW-_04F24_F_0	BY935325
E_FL_prW-_04G03_F_0	BY935326
E_FL_prW-_04G04_F_0	BY935327
E_FL_prW-_04G05_F_0	BY935328
E_FL_prW-_04G10_F_0	BY935329
E_FL_prW-_04G11_F_0	BY935330
E_FL_prW-_04G13_F_0	BY935331
E_FL_prW-_04G14_F_0	BY935332
E_FL_prW-_04G16_F_0	BY935333
E_FL_prW-_04G17_F_0	BY935334
E_FL_prW-_04G22_F_0	BY935335
E_FL_prW-_04G24_F_0	BY935336
E_FL_prW-_04H02_F_0	BY935337
E_FL_prW-_04H04_F_0	BY935338
E_FL_prW-_04H05_F_0	BY935339
E_FL_prW-_04H06_F_0	BY935340
E_FL_prW-_04H08_F_0	BY935341
E_FL_prW-_04H10_F_0	BY935342
E_FL_prW-_04H14_F_0	BY935343
E_FL_prW-_04H15_F_0	BY935344
E_FL_prW-_04H19_F_0	BY935345
E_FL_prW-_04H20_F_0	BY935346
E_FL_prW-_04H21_F_0	BY935347
E_FL_prW-_04H22_F_0	BY935348
E_FL_prW-_04H23_F_0	BY935349
E_FL_prW-_04I03_F_0	BY935350
E_FL_prW-_04I04_F_0	BY935351
E_FL_prW-_04I05_F_0	BY935352
E_FL_prW-_04I10_F_0	BY935353
E_FL_prW-_04I12_F_0	BY935354
E_FL_prW-_04I16_F_0	BY935355
E_FL_prW-_04I17_F_0	BY935356
E_FL_prW-_04I19_F_0	BY935357
E_FL_prW-_04I21_F_0	BY935358
E_FL_prW-_04I22_F_0	BY935359
E_FL_prW-_04I23_F_0	BY935360
E_FL_prW-_04I24_F_0	BY935361
E_FL_prW-_04J02_F_0	BY935362
E_FL_prW-_04J05_F_0	BY935363
E_FL_prW-_04J07_F_0	BY935364
E_FL_prW-_04J08_F_0	BY935365
E_FL_prW-_04J09_F_0	BY935366
E_FL_prW-_04J10_F_0	BY935367
E_FL_prW-_04J13_F_0	BY935368
E_FL_prW-_04J14_F_0	BY935369
E_FL_prW-_04J15_F_0	BY935370
E_FL_prW-_04J16_F_0	BY935371
E_FL_prW-_04J17_F_0	BY935372
E_FL_prW-_04J21_F_0	BY935373
E_FL_prW-_04J22_F_0	BY935374
E_FL_prW-_04J24_F_0	BY935375
E_FL_prW-_04K03_F_0	BY935376
E_FL_prW-_04K04_F_0	BY935377
E_FL_prW-_04K06_F_0	BY935378

E_FL_prW-_04K08_F_0	BY935379
E_FL_prW-_04K10_F_0	BY935380
E_FL_prW-_04K11_F_0	BY935381
E_FL_prW-_04K14_F_0	BY935382
E_FL_prW-_04K15_F_0	BY935383
E_FL_prW-_04K17_F_0	BY935384
E_FL_prW-_04K20_F_0	BY935385
E_FL_prW-_04K21_F_0	BY935386
E_FL_prW-_04K22_F_0	BY935387
E_FL_prW-_04L01_F_0	BY935388
E_FL_prW-_04L02_F_0	BY935389
E_FL_prW-_04L03_F_0	BY935390
E_FL_prW-_04L04_F_0	BY935391
E_FL_prW-_04L06_F_0	BY935392
E_FL_prW-_04L10_F_0	BY935393
E_FL_prW-_04L13_F_0	BY935394
E_FL_prW-_04L15_F_0	BY935395
E_FL_prW-_04L17_F_0	BY935396
E_FL_prW-_04L18_F_0	BY935397
E_FL_prW-_04L19_F_0	BY935398
E_FL_prW-_04L20_F_0	BY935399
E_FL_prW-_04L23_F_0	BY935400
E_FL_prW-_04L24_F_0	BY935401
E_FL_prW-_04M02_F_0	BY935402
E_FL_prW-_04M03_F_0	BY935403
E_FL_prW-_04M05_F_0	BY935404
E_FL_prW-_04M06_F_0	BY935405
E_FL_prW-_04M08_F_0	BY935406
E_FL_prW-_04M12_F_0	BY935407
E_FL_prW-_04M14_F_0	BY935408
E_FL_prW-_04M15_F_0	BY935409
E_FL_prW-_04M16_F_0	BY935410
E_FL_prW-_04M17_F_0	BY935411
E_FL_prW-_04M18_F_0	BY935412
E_FL_prW-_04N01_F_0	BY935413
E_FL_prW-_04N03_F_0	BY935414
E_FL_prW-_04N04_F_0	BY935415
E_FL_prW-_04N05_F_0	BY935416
E_FL_prW-_04N06_F_0	BY935417
E_FL_prW-_04N08_F_0	BY935418
E_FL_prW-_04N10_F_0	BY935419
E_FL_prW-_04N12_F_0	BY935420
E_FL_prW-_04N14_F_0	BY935421
E_FL_prW-_04N15_F_0	BY935422
E_FL_prW-_04N16_F_0	BY935423
E_FL_prW-_04N18_F_0	BY935424
E_FL_prW-_04N21_F_0	BY935425
E_FL_prW-_04N22_F_0	BY935426
E_FL_prW-_04N23_F_0	BY935427
E_FL_prW-_04N24_F_0	BY935428
E_FL_prW-_04O01_F_0	BY935429
E_FL_prW-_04O02_F_0	BY935430
E_FL_prW-_04O03_F_0	BY935431
E_FL_prW-_04O04_F_0	BY935432

E_FL_prW-_04O05_F_0	BY935433
E_FL_prW-_04O09_F_0	BY935434
E_FL_prW-_04O11_F_0	BY935435
E_FL_prW-_04O14_F_0	BY935436
E_FL_prW-_04O15_F_0	BY935437
E_FL_prW-_04O16_F_0	BY935438
E_FL_prW-_04O18_F_0	BY935439
E_FL_prW-_04O21_F_0	BY935440
E_FL_prW-_04P01_F_0	BY935441
E_FL_prW-_04P02_F_0	BY935442
E_FL_prW-_04P03_F_0	BY935443
E_FL_prW-_04P06_F_0	BY935444
E_FL_prW-_04P08_F_0	BY935445
E_FL_prW-_04P09_F_0	BY935446
E_FL_prW-_04P10_F_0	BY935447
E_FL_prW-_04P11_F_0	BY935448
E_FL_prW-_04P18_F_0	BY935449
E_FL_prW-_04P20_F_0	BY935450
E_FL_prW-_04P21_F_0	BY935451
E_FL_prW-_04P22_F_0	BY935452
E_FL_prW-_04P23_F_0	BY935453
E_FL_prW-_05A01_F_0	BY935454
E_FL_prW-_05A02_F_0	BY935455
E_FL_prW-_05A03_F_0	BY935456
E_FL_prW-_05A04_F_0	BY935457
E_FL_prW-_05A06_F_0	BY935458
E_FL_prW-_05A11_F_0	BY935459
E_FL_prW-_05A14_F_0	BY935460
E_FL_prW-_05A15_F_0	BY935461
E_FL_prW-_05A16_F_0	BY935462
E_FL_prW-_05A19_F_0	BY935463
E_FL_prW-_05A22_F_0	BY935464
E_FL_prW-_05A23_F_0	BY935465
E_FL_prW-_05A24_F_0	BY935466
E_FL_prW-_05B01_F_0	BY935467
E_FL_prW-_05B02_F_0	BY935468
E_FL_prW-_05B03_F_0	BY935469
E_FL_prW-_05B04_F_0	BY935470
E_FL_prW-_05B05_F_0	BY935471
E_FL_prW-_05B06_F_0	BY935472
E_FL_prW-_05B07_F_0	BY935473
E_FL_prW-_05B09_F_0	BY935474
E_FL_prW-_05B10_F_0	BY935475
E_FL_prW-_05B12_F_0	BY935476
E_FL_prW-_05B13_F_0	BY935477
E_FL_prW-_05B15_F_0	BY935478
E_FL_prW-_05B16_F_0	BY935479
E_FL_prW-_05B19_F_0	BY935480
E_FL_prW-_05B20_F_0	BY935481
E_FL_prW-_05B21_F_0	BY935482
E_FL_prW-_05B22_F_0	BY935483
E_FL_prW-_05B23_F_0	BY935484
E_FL_prW-_05B24_F_0	BY935485
E_FL_prW-_05C01_F_0	BY935486

E_FL_prW-_05C07_F_0	BY935487
E_FL_prW-_05C10_F_0	BY935488
E_FL_prW-_05C11_F_0	BY935489
E_FL_prW-_05C12_F_0	BY935490
E_FL_prW-_05C13_F_0	BY935491
E_FL_prW-_05C15_F_0	BY935492
E_FL_prW-_05C16_F_0	BY935493
E_FL_prW-_05C17_F_0	BY935494
E_FL_prW-_05C18_F_0	BY935495
E_FL_prW-_05C23_F_0	BY935496
E_FL_prW-_05D01_F_0	BY935497
E_FL_prW-_05D03_F_0	BY935498
E_FL_prW-_05D05_F_0	BY935499
E_FL_prW-_05D06_F_0	BY935500
E_FL_prW-_05D07_F_0	BY935501
E_FL_prW-_05D08_F_0	BY935502
E_FL_prW-_05D10_F_0	BY935503
E_FL_prW-_05D14_F_0	BY935504
E_FL_prW-_05D16_F_0	BY935505
E_FL_prW-_05D17_F_0	BY935506
E_FL_prW-_05D18_F_0	BY935507
E_FL_prW-_05D20_F_0	BY935508
E_FL_prW-_05D21_F_0	BY935509
E_FL_prW-_05D22_F_0	BY935510
E_FL_prW-_05E01_F_0	BY935511
E_FL_prW-_05E02_F_0	BY935512
E_FL_prW-_05E04_F_0	BY935513
E_FL_prW-_05E06_F_0	BY935514
E_FL_prW-_05E07_F_0	BY935515
E_FL_prW-_05E08_F_0	BY935516
E_FL_prW-_05E09_F_0	BY935517
E_FL_prW-_05E11_F_0	BY935518
E_FL_prW-_05E12_F_0	BY935519
E_FL_prW-_05E15_F_0	BY935520
E_FL_prW-_05E16_F_0	BY935521
E_FL_prW-_05E17_F_0	BY935522
E_FL_prW-_05E18_F_0	BY935523
E_FL_prW-_05E22_F_0	BY935524
E_FL_prW-_05E23_F_0	BY935525
E_FL_prW-_05F01_F_0	BY935526
E_FL_prW-_05F02_F_0	BY935527
E_FL_prW-_05F04_F_0	BY935528
E_FL_prW-_05F06_F_0	BY935529
E_FL_prW-_05F08_F_0	BY935530
E_FL_prW-_05F09_F_0	BY935531
E_FL_prW-_05F12_F_0	BY935532
E_FL_prW-_05F16_F_0	BY935533
E_FL_prW-_05F20_F_0	BY935534
E_FL_prW-_05F23_F_0	BY935535
E_FL_prW-_05G04_F_0	BY935536
E_FL_prW-_05G05_F_0	BY935537
E_FL_prW-_05G07_F_0	BY935538
E_FL_prW-_05G09_F_0	BY935539
E_FL_prW-_05G10_F_0	BY935540

E_FL_prW-_05G12_F_0	BY935541
E_FL_prW-_05G14_F_0	BY935542
E_FL_prW-_05G19_F_0	BY935543
E_FL_prW-_05G21_F_0	BY935544
E_FL_prW-_05G22_F_0	BY935545
E_FL_prW-_05G23_F_0	BY935546
E_FL_prW-_05H01_F_0	BY935547
E_FL_prW-_05H04_F_0	BY935548
E_FL_prW-_05H05_F_0	BY935549
E_FL_prW-_05H07_F_0	BY935550
E_FL_prW-_05H08_F_0	BY935551
E_FL_prW-_05H10_F_0	BY935552
E_FL_prW-_05H12_F_0	BY935553
E_FL_prW-_05H14_F_0	BY935554
E_FL_prW-_05H18_F_0	BY935555
E_FL_prW-_05H20_F_0	BY935556
E_FL_prW-_05H21_F_0	BY935557
E_FL_prW-_05H23_F_0	BY935558
E_FL_prW-_05I04_F_0	BY935559
E_FL_prW-_05I05_F_0	BY935560
E_FL_prW-_05I06_F_0	BY935561
E_FL_prW-_05I12_F_0	BY935562
E_FL_prW-_05I17_F_0	BY935563
E_FL_prW-_05I21_F_0	BY935564
E_FL_prW-_05I22_F_0	BY935565
E_FL_prW-_05I23_F_0	BY935566
E_FL_prW-_05J04_F_0	BY935567
E_FL_prW-_05J07_F_0	BY935568
E_FL_prW-_05J09_F_0	BY935569
E_FL_prW-_05J10_F_0	BY935570
E_FL_prW-_05J11_F_0	BY935571
E_FL_prW-_05J12_F_0	BY935572
E_FL_prW-_05J13_F_0	BY935573
E_FL_prW-_05J15_F_0	BY935574
E_FL_prW-_05J17_F_0	BY935575
E_FL_prW-_05J19_F_0	BY935576
E_FL_prW-_05J21_F_0	BY935577
E_FL_prW-_05J22_F_0	BY935578
E_FL_prW-_05K02_F_0	BY935579
E_FL_prW-_05K03_F_0	BY935580
E_FL_prW-_05K07_F_0	BY935581
E_FL_prW-_05K08_F_0	BY935582
E_FL_prW-_05K10_F_0	BY935583
E_FL_prW-_05K12_F_0	BY935584
E_FL_prW-_05K13_F_0	BY935585
E_FL_prW-_05K17_F_0	BY935586
E_FL_prW-_05K23_F_0	BY935587
E_FL_prW-_05L01_F_0	BY935588
E_FL_prW-_05L05_F_0	BY935589
E_FL_prW-_05L06_F_0	BY935590
E_FL_prW-_05L08_F_0	BY935591
E_FL_prW-_05L09_F_0	BY935592
E_FL_prW-_05L10_F_0	BY935593
E_FL_prW-_05L12_F_0	BY935594

E_FL_prW-_05L13_F_0	BY935595
E_FL_prW-_05L14_F_0	BY935596
E_FL_prW-_05L16_F_0	BY935597
E_FL_prW-_05L17_F_0	BY935598
E_FL_prW-_05L21_F_0	BY935599
E_FL_prW-_05L23_F_0	BY935600
E_FL_prW-_05L24_F_0	BY935601
E_FL_prW-_05M03_F_0	BY935602
E_FL_prW-_05M04_F_0	BY935603
E_FL_prW-_05M07_F_0	BY935604
E_FL_prW-_05M08_F_0	BY935605
E_FL_prW-_05M09_F_0	BY935606
E_FL_prW-_05M10_F_0	BY935607
E_FL_prW-_05M12_F_0	BY935608
E_FL_prW-_05M13_F_0	BY935609
E_FL_prW-_05M14_F_0	BY935610
E_FL_prW-_05M15_F_0	BY935611
E_FL_prW-_05M16_F_0	BY935612
E_FL_prW-_05M17_F_0	BY935613
E_FL_prW-_05M21_F_0	BY935614
E_FL_prW-_05M22_F_0	BY935615
E_FL_prW-_05M24_F_0	BY935616
E_FL_prW-_05N01_F_0	BY935617
E_FL_prW-_05N03_F_0	BY935618
E_FL_prW-_05N05_F_0	BY935619
E_FL_prW-_05N06_F_0	BY935620
E_FL_prW-_05N07_F_0	BY935621
E_FL_prW-_05N10_F_0	BY935622
E_FL_prW-_05N14_F_0	BY935623
E_FL_prW-_05N18_F_0	BY935624
E_FL_prW-_05N19_F_0	BY935625
E_FL_prW-_05N20_F_0	BY935626
E_FL_prW-_05N21_F_0	BY935627
E_FL_prW-_05O03_F_0	BY935628
E_FL_prW-_05O04_F_0	BY935629
E_FL_prW-_05O05_F_0	BY935630
E_FL_prW-_05O09_F_0	BY935631
E_FL_prW-_05O12_F_0	BY935632
E_FL_prW-_05O16_F_0	BY935633
E_FL_prW-_05O20_F_0	BY935634
E_FL_prW-_05O21_F_0	BY935635
E_FL_prW-_05O23_F_0	BY935636
E_FL_prW-_05P02_F_0	BY935637
E_FL_prW-_05P04_F_0	BY935638
E_FL_prW-_05P05_F_0	BY935639
E_FL_prW-_05P06_F_0	BY935640
E_FL_prW-_05P07_F_0	BY935641
E_FL_prW-_05P08_F_0	BY935642
E_FL_prW-_05P09_F_0	BY935643
E_FL_prW-_05P10_F_0	BY935644
E_FL_prW-_05P12_F_0	BY935645
E_FL_prW-_05P13_F_0	BY935646
E_FL_prW-_05P17_F_0	BY935647
E_FL_prW-_05P24_F_0	BY935648

E_FL_prW-_06A01_F_0	BY935649
E_FL_prW-_06A02_F_0	BY935650
E_FL_prW-_06A04_F_0	BY935651
E_FL_prW-_06A08_F_0	BY935652
E_FL_prW-_06A09_F_0	BY935653
E_FL_prW-_06A12_F_0	BY935654
E_FL_prW-_06A14_F_0	BY935655
E_FL_prW-_06A15_F_0	BY935656
E_FL_prW-_06A16_F_0	BY935657
E_FL_prW-_06A19_F_0	BY935658
E_FL_prW-_06A20_F_0	BY935659
E_FL_prW-_06A21_F_0	BY935660
E_FL_prW-_06A22_F_0	BY935661
E_FL_prW-_06A23_F_0	BY935662
E_FL_prW-_06A24_F_0	BY935663
E_FL_prW-_06B02_F_0	BY935664
E_FL_prW-_06B05_F_0	BY935665
E_FL_prW-_06B07_F_0	BY935666
E_FL_prW-_06B11_F_0	BY935667
E_FL_prW-_06B13_F_0	BY935668
E_FL_prW-_06B14_F_0	BY935669
E_FL_prW-_06B16_F_0	BY935670
E_FL_prW-_06B19_F_0	BY935671
E_FL_prW-_06B21_F_0	BY935672
E_FL_prW-_06B22_F_0	BY935673
E_FL_prW-_06B23_F_0	BY935674
E_FL_prW-_06B24_F_0	BY935675
E_FL_prW-_06C01_F_0	BY935676
E_FL_prW-_06C05_F_0	BY935677
E_FL_prW-_06C06_F_0	BY935678
E_FL_prW-_06C07_F_0	BY935679
E_FL_prW-_06C08_F_0	BY935680
E_FL_prW-_06C09_F_0	BY935681
E_FL_prW-_06C11_F_0	BY935682
E_FL_prW-_06C13_F_0	BY935683
E_FL_prW-_06C14_F_0	BY935684
E_FL_prW-_06C15_F_0	BY935685
E_FL_prW-_06C22_F_0	BY935686
E_FL_prW-_06D01_F_0	BY935687
E_FL_prW-_06D03_F_0	BY935688
E_FL_prW-_06D05_F_0	BY935689
E_FL_prW-_06D06_F_0	BY935690
E_FL_prW-_06D08_F_0	BY935691
E_FL_prW-_06D09_F_0	BY935692
E_FL_prW-_06D11_F_0	BY935693
E_FL_prW-_06D12_F_0	BY935694
E_FL_prW-_06D13_F_0	BY935695
E_FL_prW-_06D14_F_0	BY935696
E_FL_prW-_06D16_F_0	BY935697
E_FL_prW-_06D17_F_0	BY935698
E_FL_prW-_06D18_F_0	BY935699
E_FL_prW-_06D21_F_0	BY935700
E_FL_prW-_06D22_F_0	BY935701
E_FL_prW-_06D24_F_0	BY935702

E_FL_prW-_06E01_F_0	BY935703
E_FL_prW-_06E02_F_0	BY935704
E_FL_prW-_06E04_F_0	BY935705
E_FL_prW-_06E08_F_0	BY935706
E_FL_prW-_06E10_F_0	BY935707
E_FL_prW-_06E11_F_0	BY935708
E_FL_prW-_06E12_F_0	BY935709
E_FL_prW-_06E16_F_0	BY935710
E_FL_prW-_06E17_F_0	BY935711
E_FL_prW-_06E18_F_0	BY935712
E_FL_prW-_06E19_F_0	BY935713
E_FL_prW-_06E20_F_0	BY935714
E_FL_prW-_06E21_F_0	BY935715
E_FL_prW-_06E23_F_0	BY935716
E_FL_prW-_06F02_F_0	BY935717
E_FL_prW-_06F04_F_0	BY935718
E_FL_prW-_06F08_F_0	BY935719
E_FL_prW-_06F09_F_0	BY935720
E_FL_prW-_06F10_F_0	BY935721
E_FL_prW-_06F11_F_0	BY935722
E_FL_prW-_06F12_F_0	BY935723
E_FL_prW-_06F15_F_0	BY935724
E_FL_prW-_06F17_F_0	BY935725
E_FL_prW-_06F18_F_0	BY935726
E_FL_prW-_06F20_F_0	BY935727
E_FL_prW-_06F24_F_0	BY935728
E_FL_prW-_06G02_F_0	BY935729
E_FL_prW-_06G03_F_0	BY935730
E_FL_prW-_06G04_F_0	BY935731
E_FL_prW-_06G05_F_0	BY935732
E_FL_prW-_06G08_F_0	BY935733
E_FL_prW-_06G14_F_0	BY935734
E_FL_prW-_06G15_F_0	BY935735
E_FL_prW-_06G19_F_0	BY935736
E_FL_prW-_06G20_F_0	BY935737
E_FL_prW-_06G21_F_0	BY935738
E_FL_prW-_06H01_F_0	BY935739
E_FL_prW-_06H03_F_0	BY935740
E_FL_prW-_06H04_F_0	BY935741
E_FL_prW-_06H05_F_0	BY935742
E_FL_prW-_06H06_F_0	BY935743
E_FL_prW-_06H08_F_0	BY935744
E_FL_prW-_06H10_F_0	BY935745
E_FL_prW-_06H11_F_0	BY935746
E_FL_prW-_06H13_F_0	BY935747
E_FL_prW-_06H15_F_0	BY935748
E_FL_prW-_06H16_F_0	BY935749
E_FL_prW-_06H17_F_0	BY935750
E_FL_prW-_06H18_F_0	BY935751
E_FL_prW-_06H19_F_0	BY935752
E_FL_prW-_06H21_F_0	BY935753
E_FL_prW-_06H22_F_0	BY935754
E_FL_prW-_06H23_F_0	BY935755
E_FL_prW-_06I02_F_0	BY935756

E_FL_prW-_06I04_F_0	BY935757
E_FL_prW-_06I05_F_0	BY935758
E_FL_prW-_06I06_F_0	BY935759
E_FL_prW-_06I07_F_0	BY935760
E_FL_prW-_06I08_F_0	BY935761
E_FL_prW-_06I10_F_0	BY935762
E_FL_prW-_06I12_F_0	BY935763
E_FL_prW-_06I13_F_0	BY935764
E_FL_prW-_06I17_F_0	BY935765
E_FL_prW-_06I18_F_0	BY935766
E_FL_prW-_06I19_F_0	BY935767
E_FL_prW-_06I22_F_0	BY935768
E_FL_prW-_06I24_F_0	BY935769
E_FL_prW-_06J01_F_0	BY935770
E_FL_prW-_06J02_F_0	BY935771
E_FL_prW-_06J09_F_0	BY935772
E_FL_prW-_06J13_F_0	BY935773
E_FL_prW-_06J14_F_0	BY935774
E_FL_prW-_06J15_F_0	BY935775
E_FL_prW-_06J17_F_0	BY935776
E_FL_prW-_06J19_F_0	BY935777
E_FL_prW-_06J20_F_0	BY935778
E_FL_prW-_06J23_F_0	BY935779
E_FL_prW-_06K01_F_0	BY935780
E_FL_prW-_06K02_F_0	BY935781
E_FL_prW-_06K03_F_0	BY935782
E_FL_prW-_06K05_F_0	BY935783
E_FL_prW-_06K07_F_0	BY935784
E_FL_prW-_06K08_F_0	BY935785
E_FL_prW-_06K09_F_0	BY935786
E_FL_prW-_06K10_F_0	BY935787
E_FL_prW-_06K12_F_0	BY935788
E_FL_prW-_06K13_F_0	BY935789
E_FL_prW-_06K15_F_0	BY935790
E_FL_prW-_06K18_F_0	BY935791
E_FL_prW-_06L02_F_0	BY935792
E_FL_prW-_06L03_F_0	BY935793
E_FL_prW-_06L04_F_0	BY935794
E_FL_prW-_06L06_F_0	BY935795
E_FL_prW-_06L09_F_0	BY935796
E_FL_prW-_06L10_F_0	BY935797
E_FL_prW-_06L11_F_0	BY935798
E_FL_prW-_06L13_F_0	BY935799
E_FL_prW-_06L14_F_0	BY935800
E_FL_prW-_06L16_F_0	BY935801
E_FL_prW-_06L18_F_0	BY935802
E_FL_prW-_06L19_F_0	BY935803
E_FL_prW-_06L22_F_0	BY935804
E_FL_prW-_06L23_F_0	BY935805
E_FL_prW-_06M03_F_0	BY935806
E_FL_prW-_06M04_F_0	BY935807
E_FL_prW-_06M06_F_0	BY935808
E_FL_prW-_06M07_F_0	BY935809
E_FL_prW-_06M08_F_0	BY935810

E_FL_prW-_06M09_F_0	BY935811
E_FL_prW-_06M13_F_0	BY935812
E_FL_prW-_06M16_F_0	BY935813
E_FL_prW-_06M17_F_0	BY935814
E_FL_prW-_06M21_F_0	BY935815
E_FL_prW-_06M23_F_0	BY935816
E_FL_prW-_06N01_F_0	BY935817
E_FL_prW-_06N02_F_0	BY935818
E_FL_prW-_06N08_F_0	BY935819
E_FL_prW-_06N10_F_0	BY935820
E_FL_prW-_06N11_F_0	BY935821
E_FL_prW-_06N13_F_0	BY935822
E_FL_prW-_06N14_F_0	BY935823
E_FL_prW-_06N15_F_0	BY935824
E_FL_prW-_06N16_F_0	BY935825
E_FL_prW-_06N19_F_0	BY935826
E_FL_prW-_06N20_F_0	BY935827
E_FL_prW-_06N21_F_0	BY935828
E_FL_prW-_06N22_F_0	BY935829
E_FL_prW-_06O01_F_0	BY935830
E_FL_prW-_06O03_F_0	BY935831
E_FL_prW-_06O07_F_0	BY935832
E_FL_prW-_06O08_F_0	BY935833
E_FL_prW-_06O09_F_0	BY935834
E_FL_prW-_06O10_F_0	BY935835
E_FL_prW-_06O13_F_0	BY935836
E_FL_prW-_06O14_F_0	BY935837
E_FL_prW-_06O15_F_0	BY935838
E_FL_prW-_06O16_F_0	BY935839
E_FL_prW-_06O19_F_0	BY935840
E_FL_prW-_06O20_F_0	BY935841
E_FL_prW-_06O21_F_0	BY935842
E_FL_prW-_06P01_F_0	BY935843
E_FL_prW-_06P02_F_0	BY935844
E_FL_prW-_06P03_F_0	BY935845
E_FL_prW-_06P04_F_0	BY935846
E_FL_prW-_06P06_F_0	BY935847
E_FL_prW-_06P09_F_0	BY935848
E_FL_prW-_06P10_F_0	BY935849
E_FL_prW-_06P11_F_0	BY935850
E_FL_prW-_06P13_F_0	BY935851
E_FL_prW-_06P14_F_0	BY935852
E_FL_prW-_06P15_F_0	BY935853
E_FL_prW-_06P16_F_0	BY935854
E_FL_prW-_06P18_F_0	BY935855
E_FL_prW-_06P19_F_0	BY935856
E_FL_prW-_06P21_F_0	BY935857
E_FL_prW-_06P22_F_0	BY935858
E_FL_prW-_06P24_F_0	BY935859
E_FL_prW-_07A03_F_0	BY935860
E_FL_prW-_07A07_F_0	BY935861
E_FL_prW-_07A10_F_0	BY935862
E_FL_prW-_07A15_F_0	BY935863
E_FL_prW-_07A17_F_0	BY935864

E_FL_prW-_07A18_F_0	BY935865
E_FL_prW-_07A20_F_0	BY935866
E_FL_prW-_07A21_F_0	BY935867
E_FL_prW-_07A23_F_0	BY935868
E_FL_prW-_07A24_F_0	BY935869
E_FL_prW-_07B01_F_0	BY935870
E_FL_prW-_07B03_F_0	BY935871
E_FL_prW-_07B04_F_0	BY935872
E_FL_prW-_07B06_F_0	BY935873
E_FL_prW-_07B07_F_0	BY935874
E_FL_prW-_07B08_F_0	BY935875
E_FL_prW-_07B09_F_0	BY935876
E_FL_prW-_07B10_F_0	BY935877
E_FL_prW-_07B11_F_0	BY935878
E_FL_prW-_07B12_F_0	BY935879
E_FL_prW-_07B13_F_0	BY935880
E_FL_prW-_07B14_F_0	BY935881
E_FL_prW-_07B16_F_0	BY935882
E_FL_prW-_07B19_F_0	BY935883
E_FL_prW-_07B20_F_0	BY935884
E_FL_prW-_07B22_F_0	BY935885
E_FL_prW-_07B23_F_0	BY935886
E_FL_prW-_07C02_F_0	BY935887
E_FL_prW-_07C04_F_0	BY935888
E_FL_prW-_07C05_F_0	BY935889
E_FL_prW-_07C07_F_0	BY935890
E_FL_prW-_07C09_F_0	BY935891
E_FL_prW-_07C10_F_0	BY935892
E_FL_prW-_07C12_F_0	BY935893
E_FL_prW-_07C14_F_0	BY935894
E_FL_prW-_07C16_F_0	BY935895
E_FL_prW-_07C17_F_0	BY935896
E_FL_prW-_07C20_F_0	BY935897
E_FL_prW-_07C22_F_0	BY935898
E_FL_prW-_07D01_F_0	BY935899
E_FL_prW-_07D02_F_0	BY935900
E_FL_prW-_07D03_F_0	BY935901
E_FL_prW-_07D05_F_0	BY935902
E_FL_prW-_07D06_F_0	BY935903
E_FL_prW-_07D07_F_0	BY935904
E_FL_prW-_07D09_F_0	BY935905
E_FL_prW-_07D11_F_0	BY935906
E_FL_prW-_07D12_F_0	BY935907
E_FL_prW-_07D13_F_0	BY935908
E_FL_prW-_07D14_F_0	BY935909
E_FL_prW-_07D15_F_0	BY935910
E_FL_prW-_07D16_F_0	BY935911
E_FL_prW-_07D18_F_0	BY935912
E_FL_prW-_07D20_F_0	BY935913
E_FL_prW-_07D21_F_0	BY935914
E_FL_prW-_07D22_F_0	BY935915
E_FL_prW-_07E01_F_0	BY935916
E_FL_prW-_07E02_F_0	BY935917
E_FL_prW-_07E03_F_0	BY935918

E_FL_prW-_07E04_F_0	BY935919
E_FL_prW-_07E05_F_0	BY935920
E_FL_prW-_07E07_F_0	BY935921
E_FL_prW-_07E08_F_0	BY935922
E_FL_prW-_07E09_F_0	BY935923
E_FL_prW-_07E15_F_0	BY935924
E_FL_prW-_07E16_F_0	BY935925
E_FL_prW-_07E17_F_0	BY935926
E_FL_prW-_07E18_F_0	BY935927
E_FL_prW-_07E20_F_0	BY935928
E_FL_prW-_07E21_F_0	BY935929
E_FL_prW-_07E22_F_0	BY935930
E_FL_prW-_07E24_F_0	BY935931
E_FL_prW-_07F01_F_0	BY935932
E_FL_prW-_07F02_F_0	BY935933
E_FL_prW-_07F04_F_0	BY935934
E_FL_prW-_07F05_F_0	BY935935
E_FL_prW-_07F06_F_0	BY935936
E_FL_prW-_07F13_F_0	BY935937
E_FL_prW-_07F14_F_0	BY935938
E_FL_prW-_07F17_F_0	BY935939
E_FL_prW-_07F18_F_0	BY935940
E_FL_prW-_07F19_F_0	BY935941
E_FL_prW-_07F20_F_0	BY935942
E_FL_prW-_07F22_F_0	BY935943
E_FL_prW-_07F24_F_0	BY935944
E_FL_prW-_07G01_F_0	BY935945
E_FL_prW-_07G04_F_0	BY935946
E_FL_prW-_07G05_F_0	BY935947
E_FL_prW-_07G07_F_0	BY935948
E_FL_prW-_07G08_F_0	BY935949
E_FL_prW-_07G10_F_0	BY935950
E_FL_prW-_07G11_F_0	BY935951
E_FL_prW-_07G14_F_0	BY935952
E_FL_prW-_07G19_F_0	BY935953
E_FL_prW-_07G20_F_0	BY935954
E_FL_prW-_07G21_F_0	BY935955
E_FL_prW-_07G23_F_0	BY935956
E_FL_prW-_07H02_F_0	BY935957
E_FL_prW-_07H03_F_0	BY935958
E_FL_prW-_07H04_F_0	BY935959
E_FL_prW-_07H12_F_0	BY935960
E_FL_prW-_07H13_F_0	BY935961
E_FL_prW-_07H15_F_0	BY935962
E_FL_prW-_07H16_F_0	BY935963
E_FL_prW-_07H17_F_0	BY935964
E_FL_prW-_07H20_F_0	BY935965
E_FL_prW-_07H21_F_0	BY935966
E_FL_prW-_07H22_F_0	BY935967
E_FL_prW-_07H23_F_0	BY935968
E_FL_prW-_07H24_F_0	BY935969
E_FL_prW-_07I03_F_0	BY935970
E_FL_prW-_07I04_F_0	BY935971
E_FL_prW-_07I05_F_0	BY935972

E_FL_prW-_07I08_F_0	BY935973
E_FL_prW-_07I10_F_0	BY935974
E_FL_prW-_07I12_F_0	BY935975
E_FL_prW-_07I14_F_0	BY935976
E_FL_prW-_07I15_F_0	BY935977
E_FL_prW-_07I16_F_0	BY935978
E_FL_prW-_07I20_F_0	BY935979
E_FL_prW-_07I21_F_0	BY935980
E_FL_prW-_07I22_F_0	BY935981
E_FL_prW-_07J01_F_0	BY935982
E_FL_prW-_07J02_F_0	BY935983
E_FL_prW-_07J04_F_0	BY935984
E_FL_prW-_07J06_F_0	BY935985
E_FL_prW-_07J07_F_0	BY935986
E_FL_prW-_07J08_F_0	BY935987
E_FL_prW-_07J10_F_0	BY935988
E_FL_prW-_07J14_F_0	BY935989
E_FL_prW-_07J15_F_0	BY935990
E_FL_prW-_07J16_F_0	BY935991
E_FL_prW-_07J17_F_0	BY935992
E_FL_prW-_07J19_F_0	BY935993
E_FL_prW-_07J21_F_0	BY935994
E_FL_prW-_07J22_F_0	BY935995
E_FL_prW-_07J24_F_0	BY935996
E_FL_prW-_07K01_F_0	BY935997
E_FL_prW-_07K03_F_0	BY935998
E_FL_prW-_07K05_F_0	BY935999
E_FL_prW-_07K06_F_0	BY936000
E_FL_prW-_07K09_F_0	BY936001
E_FL_prW-_07K13_F_0	BY936002
E_FL_prW-_07K15_F_0	BY936003
E_FL_prW-_07K17_F_0	BY936004
E_FL_prW-_07K18_F_0	BY936005
E_FL_prW-_07K19_F_0	BY936006
E_FL_prW-_07K20_F_0	BY936007
E_FL_prW-_07K22_F_0	BY936008
E_FL_prW-_07L01_F_0	BY936009
E_FL_prW-_07L02_F_0	BY936010
E_FL_prW-_07L03_F_0	BY936011
E_FL_prW-_07L05_F_0	BY936012
E_FL_prW-_07L07_F_0	BY936013
E_FL_prW-_07L08_F_0	BY936014
E_FL_prW-_07L09_F_0	BY936015
E_FL_prW-_07L11_F_0	BY936016
E_FL_prW-_07L14_F_0	BY936017
E_FL_prW-_07L15_F_0	BY936018
E_FL_prW-_07M01_F_0	BY936019
E_FL_prW-_07M02_F_0	BY936020
E_FL_prW-_07M03_F_0	BY936021
E_FL_prW-_07M04_F_0	BY936022
E_FL_prW-_07M05_F_0	BY936023
E_FL_prW-_07M06_F_0	BY936024
E_FL_prW-_07M07_F_0	BY936025
E_FL_prW-_07M09_F_0	BY936026

E_FL_prW-_07M10_F_0	BY936027
E_FL_prW-_07M12_F_0	BY936028
E_FL_prW-_07M13_F_0	BY936029
E_FL_prW-_07M14_F_0	BY936030
E_FL_prW-_07M15_F_0	BY936031
E_FL_prW-_07M17_F_0	BY936032
E_FL_prW-_07M18_F_0	BY936033
E_FL_prW-_07M20_F_0	BY936034
E_FL_prW-_07M21_F_0	BY936035
E_FL_prW-_07M23_F_0	BY936036
E_FL_prW-_07N07_F_0	BY936037
E_FL_prW-_07N09_F_0	BY936038
E_FL_prW-_07N10_F_0	BY936039
E_FL_prW-_07N11_F_0	BY936040
E_FL_prW-_07N14_F_0	BY936041
E_FL_prW-_07N16_F_0	BY936042
E_FL_prW-_07N17_F_0	BY936043
E_FL_prW-_07N20_F_0	BY936044
E_FL_prW-_07N22_F_0	BY936045
E_FL_prW-_07N23_F_0	BY936046
E_FL_prW-_07O03_F_0	BY936047
E_FL_prW-_07O07_F_0	BY936048
E_FL_prW-_07O08_F_0	BY936049
E_FL_prW-_07O10_F_0	BY936050
E_FL_prW-_07O14_F_0	BY936051
E_FL_prW-_07O20_F_0	BY936052
E_FL_prW-_07O21_F_0	BY936053
E_FL_prW-_07O22_F_0	BY936054
E_FL_prW-_07P02_F_0	BY936055
E_FL_prW-_07P09_F_0	BY936056
E_FL_prW-_07P11_F_0	BY936057
E_FL_prW-_07P12_F_0	BY936058
E_FL_prW-_07P17_F_0	BY936059
E_FL_prW-_07P21_F_0	BY936060
E_FL_prW-_07P23_F_0	BY936061
E_FL_prW-_07P24_F_0	BY936062
E_FL_prW-_08A02_F_0	BY936063
E_FL_prW-_08A04_F_0	BY936064
E_FL_prW-_08A05_F_0	BY936065
E_FL_prW-_08A06_F_0	BY936066
E_FL_prW-_08A09_F_0	BY936067
E_FL_prW-_08A10_F_0	BY936068
E_FL_prW-_08A11_F_0	BY936069
E_FL_prW-_08A12_F_0	BY936070
E_FL_prW-_08A14_F_0	BY936071
E_FL_prW-_08A15_F_0	BY936072
E_FL_prW-_08A16_F_0	BY936073
E_FL_prW-_08A18_F_0	BY936074
E_FL_prW-_08A20_F_0	BY936075
E_FL_prW-_08A21_F_0	BY936076
E_FL_prW-_08A22_F_0	BY936077
E_FL_prW-_08A23_F_0	BY936078
E_FL_prW-_08A24_F_0	BY936079
E_FL_prW-_08B01_F_0	BY936080

E_FL_prW-_08B03_F_0	BY936081
E_FL_prW-_08B04_F_0	BY936082
E_FL_prW-_08B08_F_0	BY936083
E_FL_prW-_08B09_F_0	BY936084
E_FL_prW-_08B10_F_0	BY936085
E_FL_prW-_08B11_F_0	BY936086
E_FL_prW-_08B12_F_0	BY936087
E_FL_prW-_08B13_F_0	BY936088
E_FL_prW-_08B16_F_0	BY936089
E_FL_prW-_08B17_F_0	BY936090
E_FL_prW-_08B18_F_0	BY936091
E_FL_prW-_08B19_F_0	BY936092
E_FL_prW-_08B21_F_0	BY936093
E_FL_prW-_08B22_F_0	BY936094
E_FL_prW-_08B23_F_0	BY936095
E_FL_prW-_08C01_F_0	BY936096
E_FL_prW-_08C03_F_0	BY936097
E_FL_prW-_08C08_F_0	BY936098
E_FL_prW-_08C11_F_0	BY936099
E_FL_prW-_08C12_F_0	BY936100
E_FL_prW-_08C13_F_0	BY936101
E_FL_prW-_08C14_F_0	BY936102
E_FL_prW-_08C15_F_0	BY936103
E_FL_prW-_08C17_F_0	BY936104
E_FL_prW-_08C19_F_0	BY936105
E_FL_prW-_08C20_F_0	BY936106
E_FL_prW-_08C21_F_0	BY936107
E_FL_prW-_08C23_F_0	BY936108
E_FL_prW-_08C24_F_0	BY936109
E_FL_prW-_08D02_F_0	BY936110
E_FL_prW-_08D03_F_0	BY936111
E_FL_prW-_08D05_F_0	BY936112
E_FL_prW-_08D09_F_0	BY936113
E_FL_prW-_08D12_F_0	BY936114
E_FL_prW-_08D14_F_0	BY936115
E_FL_prW-_08D16_F_0	BY936116
E_FL_prW-_08D17_F_0	BY936117
E_FL_prW-_08D19_F_0	BY936118
E_FL_prW-_08D22_F_0	BY936119
E_FL_prW-_08D23_F_0	BY936120
E_FL_prW-_08E01_F_0	BY936121
E_FL_prW-_08E06_F_0	BY936122
E_FL_prW-_08E07_F_0	BY936123
E_FL_prW-_08E09_F_0	BY936124
E_FL_prW-_08E10_F_0	BY936125
E_FL_prW-_08E12_F_0	BY936126
E_FL_prW-_08E13_F_0	BY936127
E_FL_prW-_08E14_F_0	BY936128
E_FL_prW-_08E15_F_0	BY936129
E_FL_prW-_08E17_F_0	BY936130
E_FL_prW-_08E18_F_0	BY936131
E_FL_prW-_08E21_F_0	BY936132
E_FL_prW-_08E22_F_0	BY936133
E_FL_prW-_08E23_F_0	BY936134

E_FL_prW-_08E24_F_0	BY936135
E_FL_prW-_08F02_F_0	BY936136
E_FL_prW-_08F03_F_0	BY936137
E_FL_prW-_08F04_F_0	BY936138
E_FL_prW-_08F05_F_0	BY936139
E_FL_prW-_08F06_F_0	BY936140
E_FL_prW-_08F09_F_0	BY936141
E_FL_prW-_08F10_F_0	BY936142
E_FL_prW-_08F12_F_0	BY936143
E_FL_prW-_08F13_F_0	BY936144
E_FL_prW-_08F14_F_0	BY936145
E_FL_prW-_08F15_F_0	BY936146
E_FL_prW-_08F17_F_0	BY936147
E_FL_prW-_08F18_F_0	BY936148
E_FL_prW-_08F20_F_0	BY936149
E_FL_prW-_08F22_F_0	BY936150
E_FL_prW-_08F23_F_0	BY936151
E_FL_prW-_08G05_F_0	BY936152
E_FL_prW-_08G07_F_0	BY936153
E_FL_prW-_08G09_F_0	BY936154
E_FL_prW-_08G10_F_0	BY936155
E_FL_prW-_08G11_F_0	BY936156
E_FL_prW-_08G13_F_0	BY936157
E_FL_prW-_08G14_F_0	BY936158
E_FL_prW-_08G16_F_0	BY936159
E_FL_prW-_08G20_F_0	BY936160
E_FL_prW-_08G21_F_0	BY936161
E_FL_prW-_08G22_F_0	BY936162
E_FL_prW-_08G23_F_0	BY936163
E_FL_prW-_08H01_F_0	BY936164
E_FL_prW-_08H02_F_0	BY936165
E_FL_prW-_08H03_F_0	BY936166
E_FL_prW-_08H05_F_0	BY936167
E_FL_prW-_08H10_F_0	BY936168
E_FL_prW-_08H11_F_0	BY936169
E_FL_prW-_08H13_F_0	BY936170
E_FL_prW-_08H23_F_0	BY936171
E_FL_prW-_08I02_F_0	BY936172
E_FL_prW-_08I03_F_0	BY936173
E_FL_prW-_08I04_F_0	BY936174
E_FL_prW-_08I08_F_0	BY936175
E_FL_prW-_08I10_F_0	BY936176
E_FL_prW-_08I11_F_0	BY936177
E_FL_prW-_08I12_F_0	BY936178
E_FL_prW-_08I14_F_0	BY936179
E_FL_prW-_08I15_F_0	BY936180
E_FL_prW-_08I18_F_0	BY936181
E_FL_prW-_08I20_F_0	BY936182
E_FL_prW-_08I22_F_0	BY936183
E_FL_prW-_08I24_F_0	BY936184
E_FL_prW-_08J01_F_0	BY936185
E_FL_prW-_08J02_F_0	BY936186
E_FL_prW-_08J03_F_0	BY936187
E_FL_prW-_08J05_F_0	BY936188

E_FL_prW-_08J07_F_0	BY936189
E_FL_prW-_08J08_F_0	BY936190
E_FL_prW-_08J09_F_0	BY936191
E_FL_prW-_08J10_F_0	BY936192
E_FL_prW-_08J14_F_0	BY936193
E_FL_prW-_08J15_F_0	BY936194
E_FL_prW-_08J16_F_0	BY936195
E_FL_prW-_08J17_F_0	BY936196
E_FL_prW-_08J18_F_0	BY936197
E_FL_prW-_08J21_F_0	BY936198
E_FL_prW-_08J22_F_0	BY936199
E_FL_prW-_08K02_F_0	BY936200
E_FL_prW-_08K03_F_0	BY936201
E_FL_prW-_08K04_F_0	BY936202
E_FL_prW-_08K06_F_0	BY936203
E_FL_prW-_08K11_F_0	BY936204
E_FL_prW-_08K14_F_0	BY936205
E_FL_prW-_08K17_F_0	BY936206
E_FL_prW-_08K19_F_0	BY936207
E_FL_prW-_08K21_F_0	BY936208
E_FL_prW-_08K23_F_0	BY936209
E_FL_prW-_08K24_F_0	BY936210
E_FL_prW-_08L01_F_0	BY936211
E_FL_prW-_08L03_F_0	BY936212
E_FL_prW-_08L08_F_0	BY936213
E_FL_prW-_08L09_F_0	BY936214
E_FL_prW-_08L13_F_0	BY936215
E_FL_prW-_08L14_F_0	BY936216
E_FL_prW-_08L16_F_0	BY936217
E_FL_prW-_08L17_F_0	BY936218
E_FL_prW-_08L18_F_0	BY936219
E_FL_prW-_08L19_F_0	BY936220
E_FL_prW-_08L22_F_0	BY936221
E_FL_prW-_08L23_F_0	BY936222
E_FL_prW-_08M01_F_0	BY936223
E_FL_prW-_08M03_F_0	BY936224
E_FL_prW-_08M04_F_0	BY936225
E_FL_prW-_08M08_F_0	BY936226
E_FL_prW-_08M09_F_0	BY936227
E_FL_prW-_08M10_F_0	BY936228
E_FL_prW-_08M11_F_0	BY936229
E_FL_prW-_08M12_F_0	BY936230
E_FL_prW-_08M13_F_0	BY936231
E_FL_prW-_08M14_F_0	BY936232
E_FL_prW-_08M15_F_0	BY936233
E_FL_prW-_08M17_F_0	BY936234
E_FL_prW-_08M18_F_0	BY936235
E_FL_prW-_08M19_F_0	BY936236
E_FL_prW-_08M22_F_0	BY936237
E_FL_prW-_08N01_F_0	BY936238
E_FL_prW-_08N02_F_0	BY936239
E_FL_prW-_08N03_F_0	BY936240
E_FL_prW-_08N05_F_0	BY936241
E_FL_prW-_08N07_F_0	BY936242

E_FL_prW-_08N11_F_0	BY936243
E_FL_prW-_08N15_F_0	BY936244
E_FL_prW-_08N16_F_0	BY936245
E_FL_prW-_08N17_F_0	BY936246
E_FL_prW-_08N18_F_0	BY936247
E_FL_prW-_08N21_F_0	BY936248
E_FL_prW-_08N23_F_0	BY936249
E_FL_prW-_08O01_F_0	BY936250
E_FL_prW-_08O03_F_0	BY936251
E_FL_prW-_08O04_F_0	BY936252
E_FL_prW-_08O07_F_0	BY936253
E_FL_prW-_08O08_F_0	BY936254
E_FL_prW-_08O09_F_0	BY936255
E_FL_prW-_08O10_F_0	BY936256
E_FL_prW-_08O11_F_0	BY936257
E_FL_prW-_08O16_F_0	BY936258
E_FL_prW-_08O19_F_0	BY936259
E_FL_prW-_08O21_F_0	BY936260
E_FL_prW-_08O22_F_0	BY936261
E_FL_prW-_08P01_F_0	BY936262
E_FL_prW-_08P03_F_0	BY936263
E_FL_prW-_08P06_F_0	BY936264
E_FL_prW-_08P08_F_0	BY936265
E_FL_prW-_08P09_F_0	BY936266
E_FL_prW-_08P11_F_0	BY936267
E_FL_prW-_08P12_F_0	BY936268
E_FL_prW-_08P14_F_0	BY936269
E_FL_prW-_08P15_F_0	BY936270
E_FL_prW-_08P17_F_0	BY936271
E_FL_prW-_08P18_F_0	BY936272
E_FL_prW-_08P19_F_0	BY936273
E_FL_prW-_08P22_F_0	BY936274
E_FL_prW-_08P24_F_0	BY936275
E_FL_prW-_09A04_F_0	BY936276
E_FL_prW-_09A05_F_0	BY936277
E_FL_prW-_09A06_F_0	BY936278
E_FL_prW-_09A08_F_0	BY936279
E_FL_prW-_09A10_F_0	BY936280
E_FL_prW-_09A11_F_0	BY936281
E_FL_prW-_09A12_F_0	BY936282
E_FL_prW-_09A14_F_0	BY936283
E_FL_prW-_09A16_F_0	BY936284
E_FL_prW-_09A17_F_0	BY936285
E_FL_prW-_09A19_F_0	BY936286
E_FL_prW-_09A20_F_0	BY936287
E_FL_prW-_09A21_F_0	BY936288
E_FL_prW-_09A23_F_0	BY936289
E_FL_prW-_09A24_F_0	BY936290
E_FL_prW-_09B02_F_0	BY936291
E_FL_prW-_09B03_F_0	BY936292
E_FL_prW-_09B04_F_0	BY936293
E_FL_prW-_09B06_F_0	BY936294
E_FL_prW-_09B09_F_0	BY936295
E_FL_prW-_09B10_F_0	BY936296

E_FL_prW-_09B13_F_0	BY936297
E_FL_prW-_09B16_F_0	BY936298
E_FL_prW-_09B19_F_0	BY936299
E_FL_prW-_09B23_F_0	BY936300
E_FL_prW-_09B24_F_0	BY936301
E_FL_prW-_09C01_F_0	BY936302
E_FL_prW-_09C02_F_0	BY936303
E_FL_prW-_09C05_F_0	BY936304
E_FL_prW-_09C07_F_0	BY936305
E_FL_prW-_09C10_F_0	BY936306
E_FL_prW-_09C11_F_0	BY936307
E_FL_prW-_09C15_F_0	BY936308
E_FL_prW-_09C16_F_0	BY936309
E_FL_prW-_09C17_F_0	BY936310
E_FL_prW-_09C18_F_0	BY936311
E_FL_prW-_09C22_F_0	BY936312
E_FL_prW-_09C24_F_0	BY936313
E_FL_prW-_09D02_F_0	BY936314
E_FL_prW-_09D04_F_0	BY936315
E_FL_prW-_09D05_F_0	BY936316
E_FL_prW-_09D09_F_0	BY936317
E_FL_prW-_09D11_F_0	BY936318
E_FL_prW-_09D13_F_0	BY936319
E_FL_prW-_09D15_F_0	BY936320
E_FL_prW-_09D17_F_0	BY936321
E_FL_prW-_09D18_F_0	BY936322
E_FL_prW-_09D20_F_0	BY936323
E_FL_prW-_09D21_F_0	BY936324
E_FL_prW-_09D22_F_0	BY936325
E_FL_prW-_09D23_F_0	BY936326
E_FL_prW-_09E03_F_0	BY936327
E_FL_prW-_09E10_F_0	BY936328
E_FL_prW-_09E12_F_0	BY936329
E_FL_prW-_09E16_F_0	BY936330
E_FL_prW-_09E18_F_0	BY936331
E_FL_prW-_09E21_F_0	BY936332
E_FL_prW-_09E22_F_0	BY936333
E_FL_prW-_09E23_F_0	BY936334
E_FL_prW-_09E24_F_0	BY936335
E_FL_prW-_09F03_F_0	BY936336
E_FL_prW-_09F09_F_0	BY936337
E_FL_prW-_09F11_F_0	BY936338
E_FL_prW-_09F15_F_0	BY936339
E_FL_prW-_09F16_F_0	BY936340
E_FL_prW-_09F19_F_0	BY936341
E_FL_prW-_09F21_F_0	BY936342
E_FL_prW-_09G03_F_0	BY936343
E_FL_prW-_09G04_F_0	BY936344
E_FL_prW-_09G05_F_0	BY936345
E_FL_prW-_09G07_F_0	BY936346
E_FL_prW-_09G08_F_0	BY936347
E_FL_prW-_09G10_F_0	BY936348
E_FL_prW-_09G11_F_0	BY936349
E_FL_prW-_09G13_F_0	BY936350

E_FL_prW-_09G15_F_0	BY936351
E_FL_prW-_09G16_F_0	BY936352
E_FL_prW-_09G18_F_0	BY936353
E_FL_prW-_09G19_F_0	BY936354
E_FL_prW-_09G22_F_0	BY936355
E_FL_prW-_09G23_F_0	BY936356
E_FL_prW-_09H01_F_0	BY936357
E_FL_prW-_09H02_F_0	BY936358
E_FL_prW-_09H03_F_0	BY936359
E_FL_prW-_09H04_F_0	BY936360
E_FL_prW-_09H06_F_0	BY936361
E_FL_prW-_09H08_F_0	BY936362
E_FL_prW-_09H09_F_0	BY936363
E_FL_prW-_09H10_F_0	BY936364
E_FL_prW-_09H13_F_0	BY936365
E_FL_prW-_09H14_F_0	BY936366
E_FL_prW-_09H16_F_0	BY936367
E_FL_prW-_09H19_F_0	BY936368
E_FL_prW-_09H20_F_0	BY936369
E_FL_prW-_09H21_F_0	BY936370
E_FL_prW-_09H22_F_0	BY936371
E_FL_prW-_09H24_F_0	BY936372
E_FL_prW-_09I01_F_0	BY936373
E_FL_prW-_09I04_F_0	BY936374
E_FL_prW-_09I06_F_0	BY936375
E_FL_prW-_09I10_F_0	BY936376
E_FL_prW-_09I12_F_0	BY936377
E_FL_prW-_09I21_F_0	BY936378
E_FL_prW-_09I22_F_0	BY936379
E_FL_prW-_09I23_F_0	BY936380
E_FL_prW-_09J03_F_0	BY936381
E_FL_prW-_09J04_F_0	BY936382
E_FL_prW-_09J06_F_0	BY936383
E_FL_prW-_09J07_F_0	BY936384
E_FL_prW-_09J08_F_0	BY936385
E_FL_prW-_09J11_F_0	BY936386
E_FL_prW-_09J14_F_0	BY936387
E_FL_prW-_09J15_F_0	BY936388
E_FL_prW-_09J18_F_0	BY936389
E_FL_prW-_09J19_F_0	BY936390
E_FL_prW-_09J23_F_0	BY936391
E_FL_prW-_09J24_F_0	BY936392
E_FL_prW-_09K05_F_0	BY936393
E_FL_prW-_09K06_F_0	BY936394
E_FL_prW-_09K07_F_0	BY936395
E_FL_prW-_09K08_F_0	BY936396
E_FL_prW-_09K10_F_0	BY936397
E_FL_prW-_09K11_F_0	BY936398
E_FL_prW-_09K13_F_0	BY936399
E_FL_prW-_09K15_F_0	BY936400
E_FL_prW-_09K16_F_0	BY936401
E_FL_prW-_09K17_F_0	BY936402
E_FL_prW-_09K18_F_0	BY936403
E_FL_prW-_09K19_F_0	BY936404

E_FL_prW-_09L01_F_0	BY936405
E_FL_prW-_09L02_F_0	BY936406
E_FL_prW-_09L03_F_0	BY936407
E_FL_prW-_09L07_F_0	BY936408
E_FL_prW-_09L09_F_0	BY936409
E_FL_prW-_09L12_F_0	BY936410
E_FL_prW-_09L14_F_0	BY936411
E_FL_prW-_09L16_F_0	BY936412
E_FL_prW-_09L21_F_0	BY936413
E_FL_prW-_09L22_F_0	BY936414
E_FL_prW-_09L23_F_0	BY936415
E_FL_prW-_09M03_F_0	BY936416
E_FL_prW-_09M04_F_0	BY936417
E_FL_prW-_09M05_F_0	BY936418
E_FL_prW-_09M06_F_0	BY936419
E_FL_prW-_09M07_F_0	BY936420
E_FL_prW-_09M09_F_0	BY936421
E_FL_prW-_09M11_F_0	BY936422
E_FL_prW-_09M14_F_0	BY936423
E_FL_prW-_09M17_F_0	BY936424
E_FL_prW-_09M18_F_0	BY936425
E_FL_prW-_09M19_F_0	BY936426
E_FL_prW-_09M20_F_0	BY936427
E_FL_prW-_09M21_F_0	BY936428
E_FL_prW-_09M22_F_0	BY936429
E_FL_prW-_09N01_F_0	BY936430
E_FL_prW-_09N03_F_0	BY936431
E_FL_prW-_09N04_F_0	BY936432
E_FL_prW-_09N07_F_0	BY936433
E_FL_prW-_09N08_F_0	BY936434
E_FL_prW-_09N11_F_0	BY936435
E_FL_prW-_09N13_F_0	BY936436
E_FL_prW-_09N14_F_0	BY936437
E_FL_prW-_09N15_F_0	BY936438
E_FL_prW-_09N16_F_0	BY936439
E_FL_prW-_09N17_F_0	BY936440
E_FL_prW-_09N18_F_0	BY936441
E_FL_prW-_09N19_F_0	BY936442
E_FL_prW-_09N20_F_0	BY936443
E_FL_prW-_09N21_F_0	BY936444
E_FL_prW-_09N22_F_0	BY936445
E_FL_prW-_09N23_F_0	BY936446
E_FL_prW-_09O02_F_0	BY936447
E_FL_prW-_09O04_F_0	BY936448
E_FL_prW-_09O05_F_0	BY936449
E_FL_prW-_09O14_F_0	BY936450
E_FL_prW-_09O15_F_0	BY936451
E_FL_prW-_09O16_F_0	BY936452
E_FL_prW-_09O19_F_0	BY936453
E_FL_prW-_09O20_F_0	BY936454
E_FL_prW-_09O21_F_0	BY936455
E_FL_prW-_09P04_F_0	BY936456
E_FL_prW-_09P05_F_0	BY936457
E_FL_prW-_09P07_F_0	BY936458

E_FL_prW-_09P09_F_0	BY936459
E_FL_prW-_09P10_F_0	BY936460
E_FL_prW-_09P11_F_0	BY936461
E_FL_prW-_09P15_F_0	BY936462
E_FL_prW-_10A07_F_0	BY936463
E_FL_prW-_10A12_F_0	BY936464
E_FL_prW-_10A21_F_0	BY936465
E_FL_prW-_10A24_F_0	BY936466
E_FL_prW-_10B01_F_0	BY936467
E_FL_prW-_10B02_F_0	BY936468
E_FL_prW-_10B04_F_0	BY936469
E_FL_prW-_10B05_F_0	BY936470
E_FL_prW-_10B06_F_0	BY936471
E_FL_prW-_10B08_F_0	BY936472
E_FL_prW-_10B13_F_0	BY936473
E_FL_prW-_10B15_F_0	BY936474
E_FL_prW-_10B16_F_0	BY936475
E_FL_prW-_10B17_F_0	BY936476
E_FL_prW-_10B18_F_0	BY936477
E_FL_prW-_10B20_F_0	BY936478
E_FL_prW-_10B21_F_0	BY936479
E_FL_prW-_10B22_F_0	BY936480
E_FL_prW-_10C06_F_0	BY936481
E_FL_prW-_10C07_F_0	BY936482
E_FL_prW-_10C19_F_0	BY936483
E_FL_prW-_10D01_F_0	BY936484
E_FL_prW-_10D03_F_0	BY936485
E_FL_prW-_10D05_F_0	BY936486
E_FL_prW-_10D07_F_0	BY936487
E_FL_prW-_10D09_F_0	BY936488
E_FL_prW-_10D11_F_0	BY936489
E_FL_prW-_10D13_F_0	BY936490
E_FL_prW-_10D15_F_0	BY936491
E_FL_prW-_10D16_F_0	BY936492
E_FL_prW-_10D18_F_0	BY936493
E_FL_prW-_10D19_F_0	BY936494
E_FL_prW-_10E07_F_0	BY936495
E_FL_prW-_10E13_F_0	BY936496
E_FL_prW-_10E14_F_0	BY936497
E_FL_prW-_10E18_F_0	BY936498
E_FL_prW-_10E21_F_0	BY936499
E_FL_prW-_10E22_F_0	BY936500
E_FL_prW-_10F02_F_0	BY936501
E_FL_prW-_10F03_F_0	BY936502
E_FL_prW-_10F05_F_0	BY936503
E_FL_prW-_10F06_F_0	BY936504
E_FL_prW-_10F07_F_0	BY936505
E_FL_prW-_10F08_F_0	BY936506
E_FL_prW-_10F11_F_0	BY936507
E_FL_prW-_10F12_F_0	BY936508
E_FL_prW-_10F14_F_0	BY936509
E_FL_prW-_10F17_F_0	BY936510
E_FL_prW-_10F18_F_0	BY936511
E_FL_prW-_10F19_F_0	BY936512

E_FL_prW-_10F20_F_0	BY936513
E_FL_prW-_10F23_F_0	BY936514
E_FL_prW-_10G05_F_0	BY936515
E_FL_prW-_10G07_F_0	BY936516
E_FL_prW-_10G08_F_0	BY936517
E_FL_prW-_10G11_F_0	BY936518
E_FL_prW-_10G13_F_0	BY936519
E_FL_prW-_10G16_F_0	BY936520
E_FL_prW-_10G17_F_0	BY936521
E_FL_prW-_10G18_F_0	BY936522
E_FL_prW-_10G19_F_0	BY936523
E_FL_prW-_10G20_F_0	BY936524
E_FL_prW-_10G22_F_0	BY936525
E_FL_prW-_10H08_F_0	BY936526
E_FL_prW-_10H09_F_0	BY936527
E_FL_prW-_10H12_F_0	BY936528
E_FL_prW-_10H14_F_0	BY936529
E_FL_prW-_10H15_F_0	BY936530
E_FL_prW-_10H17_F_0	BY936531
E_FL_prW-_10H18_F_0	BY936532
E_FL_prW-_10H23_F_0	BY936533
E_FL_prW-_10H24_F_0	BY936534
E_FL_prW-_10I05_F_0	BY936535
E_FL_prW-_10I06_F_0	BY936536
E_FL_prW-_10I07_F_0	BY936537
E_FL_prW-_10I09_F_0	BY936538
E_FL_prW-_10I10_F_0	BY936539
E_FL_prW-_10I11_F_0	BY936540
E_FL_prW-_10I13_F_0	BY936541
E_FL_prW-_10I15_F_0	BY936542
E_FL_prW-_10I16_F_0	BY936543
E_FL_prW-_10I18_F_0	BY936544
E_FL_prW-_10I19_F_0	BY936545
E_FL_prW-_10I22_F_0	BY936546
E_FL_prW-_10I23_F_0	BY936547
E_FL_prW-_10J01_F_0	BY936548
E_FL_prW-_10J02_F_0	BY936549
E_FL_prW-_10J04_F_0	BY936550
E_FL_prW-_10J05_F_0	BY936551
E_FL_prW-_10J07_F_0	BY936552
E_FL_prW-_10J08_F_0	BY936553
E_FL_prW-_10J11_F_0	BY936554
E_FL_prW-_10J12_F_0	BY936555
E_FL_prW-_10J13_F_0	BY936556
E_FL_prW-_10J14_F_0	BY936557
E_FL_prW-_10J15_F_0	BY936558
E_FL_prW-_10J19_F_0	BY936559
E_FL_prW-_10J21_F_0	BY936560
E_FL_prW-_10J24_F_0	BY936561
E_FL_prW-_10K11_F_0	BY936562
E_FL_prW-_10K13_F_0	BY936563
E_FL_prW-_10K14_F_0	BY936564
E_FL_prW-_10K17_F_0	BY936565
E_FL_prW-_10K19_F_0	BY936566

E_FL_prW-_10K22_F_0	BY936567
E_FL_prW-_10K23_F_0	BY936568
E_FL_prW-_10L01_F_0	BY936569
E_FL_prW-_10L03_F_0	BY936570
E_FL_prW-_10L04_F_0	BY936571
E_FL_prW-_10L07_F_0	BY936572
E_FL_prW-_10L08_F_0	BY936573
E_FL_prW-_10L09_F_0	BY936574
E_FL_prW-_10L10_F_0	BY936575
E_FL_prW-_10L11_F_0	BY936576
E_FL_prW-_10L15_F_0	BY936577
E_FL_prW-_10L16_F_0	BY936578
E_FL_prW-_10L19_F_0	BY936579
E_FL_prW-_10L20_F_0	BY936580
E_FL_prW-_10L21_F_0	BY936581
E_FL_prW-_10L22_F_0	BY936582
E_FL_prW-_10M07_F_0	BY936583
E_FL_prW-_10M08_F_0	BY936584
E_FL_prW-_10M13_F_0	BY936585
E_FL_prW-_10M16_F_0	BY936586
E_FL_prW-_10M17_F_0	BY936587
E_FL_prW-_10M19_F_0	BY936588
E_FL_prW-_10M20_F_0	BY936589
E_FL_prW-_10M22_F_0	BY936590
E_FL_prW-_10M23_F_0	BY936591
E_FL_prW-_10M24_F_0	BY936592
E_FL_prW-_10N01_F_0	BY936593
E_FL_prW-_10N02_F_0	BY936594
E_FL_prW-_10N03_F_0	BY936595
E_FL_prW-_10N05_F_0	BY936596
E_FL_prW-_10N07_F_0	BY936597
E_FL_prW-_10N09_F_0	BY936598
E_FL_prW-_10N11_F_0	BY936599
E_FL_prW-_10N15_F_0	BY936600
E_FL_prW-_10N17_F_0	BY936601
E_FL_prW-_10N20_F_0	BY936602
E_FL_prW-_10N23_F_0	BY936603
E_FL_prW-_10O06_F_0	BY936604
E_FL_prW-_10O08_F_0	BY936605
E_FL_prW-_10O12_F_0	BY936606
E_FL_prW-_10O14_F_0	BY936607
E_FL_prW-_10O16_F_0	BY936608
E_FL_prW-_10O17_F_0	BY936609
E_FL_prW-_10O18_F_0	BY936610
E_FL_prW-_10O24_F_0	BY936611
E_FL_prW-_10P01_F_0	BY936612
E_FL_prW-_10P02_F_0	BY936613
E_FL_prW-_10P03_F_0	BY936614
E_FL_prW-_10P04_F_0	BY936615
E_FL_prW-_10P06_F_0	BY936616
E_FL_prW-_10P07_F_0	BY936617
E_FL_prW-_10P09_F_0	BY936618
E_FL_prW-_10P10_F_0	BY936619
E_FL_prW-_10P12_F_0	BY936620

E_FL_prW-_10P13_F_0	BY936621
E_FL_prW-_10P15_F_0	BY936622
E_FL_prW-_10P16_F_0	BY936623
E_FL_prW-_10P17_F_0	BY936624
E_FL_prW-_10P19_F_0	BY936625
E_FL_prW-_10P20_F_0	BY936626
E_FL_prW-_10P24_F_0	BY936627
E_FL_prW-_11A02_F_0	BY936628
E_FL_prW-_11A03_F_0	BY936629
E_FL_prW-_11A06_F_0	BY936630
E_FL_prW-_11A07_F_0	BY936631
E_FL_prW-_11A08_F_0	BY936632
E_FL_prW-_11A10_F_0	BY936633
E_FL_prW-_11A11_F_0	BY936634
E_FL_prW-_11A13_F_0	BY936635
E_FL_prW-_11A20_F_0	BY936636
E_FL_prW-_11A22_F_0	BY936637
E_FL_prW-_11A23_F_0	BY936638
E_FL_prW-_11A24_F_0	BY936639
E_FL_prW-_11B02_F_0	BY936640
E_FL_prW-_11B03_F_0	BY936641
E_FL_prW-_11B05_F_0	BY936642
E_FL_prW-_11B06_F_0	BY936643
E_FL_prW-_11B07_F_0	BY936644
E_FL_prW-_11B08_F_0	BY936645
E_FL_prW-_11B11_F_0	BY936646
E_FL_prW-_11B12_F_0	BY936647
E_FL_prW-_11B15_F_0	BY936648
E_FL_prW-_11B17_F_0	BY936649
E_FL_prW-_11B18_F_0	BY936650
E_FL_prW-_11B19_F_0	BY936651
E_FL_prW-_11B21_F_0	BY936652
E_FL_prW-_11B22_F_0	BY936653
E_FL_prW-_11B23_F_0	BY936654
E_FL_prW-_11B24_F_0	BY936655
E_FL_prW-_11C11_F_0	BY936656
E_FL_prW-_11C13_F_0	BY936657
E_FL_prW-_11C17_F_0	BY936658
E_FL_prW-_11C19_F_0	BY936659
E_FL_prW-_11C23_F_0	BY936660
E_FL_prW-_11D01_F_0	BY936661
E_FL_prW-_11D04_F_0	BY936662
E_FL_prW-_11D05_F_0	BY936663
E_FL_prW-_11D08_F_0	BY936664
E_FL_prW-_11D09_F_0	BY936665
E_FL_prW-_11D10_F_0	BY936666
E_FL_prW-_11D13_F_0	BY936667
E_FL_prW-_11D14_F_0	BY936668
E_FL_prW-_11D15_F_0	BY936669
E_FL_prW-_11D18_F_0	BY936670
E_FL_prW-_11D19_F_0	BY936671
E_FL_prW-_11D21_F_0	BY936672
E_FL_prW-_11D24_F_0	BY936673
E_FL_prW-_11E02_F_0	BY936674

E_FL_prW-_11E03_F_0	BY936675
E_FL_prW-_11E04_F_0	BY936676
E_FL_prW-_11E06_F_0	BY936677
E_FL_prW-_11E10_F_0	BY936678
E_FL_prW-_11E13_F_0	BY936679
E_FL_prW-_11E15_F_0	BY936680
E_FL_prW-_11E17_F_0	BY936681
E_FL_prW-_11E18_F_0	BY936682
E_FL_prW-_11E19_F_0	BY936683
E_FL_prW-_11E20_F_0	BY936684
E_FL_prW-_11E21_F_0	BY936685
E_FL_prW-_11E23_F_0	BY936686
E_FL_prW-_11E24_F_0	BY936687
E_FL_prW-_11F01_F_0	BY936688
E_FL_prW-_11F02_F_0	BY936689
E_FL_prW-_11F04_F_0	BY936690
E_FL_prW-_11F06_F_0	BY936691
E_FL_prW-_11F07_F_0	BY936692
E_FL_prW-_11F08_F_0	BY936693
E_FL_prW-_11F11_F_0	BY936694
E_FL_prW-_11F14_F_0	BY936695
E_FL_prW-_11F15_F_0	BY936696
E_FL_prW-_11F18_F_0	BY936697
E_FL_prW-_11F20_F_0	BY936698
E_FL_prW-_11F23_F_0	BY936699
E_FL_prW-_11F24_F_0	BY936700
E_FL_prW-_11G02_F_0	BY936701
E_FL_prW-_11G06_F_0	BY936702
E_FL_prW-_11G13_F_0	BY936703
E_FL_prW-_11G16_F_0	BY936704
E_FL_prW-_11G19_F_0	BY936705
E_FL_prW-_11G22_F_0	BY936706
E_FL_prW-_11G24_F_0	BY936707
E_FL_prW-_11H01_F_0	BY936708
E_FL_prW-_11H05_F_0	BY936709
E_FL_prW-_11H06_F_0	BY936710
E_FL_prW-_11H07_F_0	BY936711
E_FL_prW-_11H08_F_0	BY936712
E_FL_prW-_11H12_F_0	BY936713
E_FL_prW-_11H14_F_0	BY936714
E_FL_prW-_11H18_F_0	BY936715
E_FL_prW-_11H21_F_0	BY936716
E_FL_prW-_11H23_F_0	BY936717
E_FL_prW-_11I01_F_0	BY936718
E_FL_prW-_11I03_F_0	BY936719
E_FL_prW-_11I04_F_0	BY936720
E_FL_prW-_11I07_F_0	BY936721
E_FL_prW-_11I10_F_0	BY936722
E_FL_prW-_11I11_F_0	BY936723
E_FL_prW-_11I12_F_0	BY936724
E_FL_prW-_11I13_F_0	BY936725
E_FL_prW-_11I15_F_0	BY936726
E_FL_prW-_11I16_F_0	BY936727
E_FL_prW-_11I17_F_0	BY936728

E_FL_prW-_11I18_F_0	BY936729
E_FL_prW-_11I19_F_0	BY936730
E_FL_prW-_11I23_F_0	BY936731
E_FL_prW-_11I24_F_0	BY936732
E_FL_prW-_11J01_F_0	BY936733
E_FL_prW-_11J02_F_0	BY936734
E_FL_prW-_11J04_F_0	BY936735
E_FL_prW-_11J06_F_0	BY936736
E_FL_prW-_11J07_F_0	BY936737
E_FL_prW-_11J14_F_0	BY936738
E_FL_prW-_11J15_F_0	BY936739
E_FL_prW-_11J16_F_0	BY936740
E_FL_prW-_11J20_F_0	BY936741
E_FL_prW-_11J22_F_0	BY936742
E_FL_prW-_11J24_F_0	BY936743
E_FL_prW-_11K01_F_0	BY936744
E_FL_prW-_11K05_F_0	BY936745
E_FL_prW-_11K08_F_0	BY936746
E_FL_prW-_11K10_F_0	BY936747
E_FL_prW-_11K17_F_0	BY936748
E_FL_prW-_11K20_F_0	BY936749
E_FL_prW-_11K22_F_0	BY936750
E_FL_prW-_11K23_F_0	BY936751
E_FL_prW-_11L01_F_0	BY936752
E_FL_prW-_11L04_F_0	BY936753
E_FL_prW-_11L07_F_0	BY936754
E_FL_prW-_11L10_F_0	BY936755
E_FL_prW-_11L13_F_0	BY936756
E_FL_prW-_11L15_F_0	BY936757
E_FL_prW-_11L23_F_0	BY936758
E_FL_prW-_11M01_F_0	BY936759
E_FL_prW-_11M02_F_0	BY936760
E_FL_prW-_11M05_F_0	BY936761
E_FL_prW-_11M09_F_0	BY936762
E_FL_prW-_11M12_F_0	BY936763
E_FL_prW-_11M14_F_0	BY936764
E_FL_prW-_11M15_F_0	BY936765
E_FL_prW-_11M16_F_0	BY936766
E_FL_prW-_11M19_F_0	BY936767
E_FL_prW-_11M22_F_0	BY936768
E_FL_prW-_11M24_F_0	BY936769
E_FL_prW-_11N01_F_0	BY936770
E_FL_prW-_11N03_F_0	BY936771
E_FL_prW-_11N04_F_0	BY936772
E_FL_prW-_11N07_F_0	BY936773
E_FL_prW-_11N08_F_0	BY936774
E_FL_prW-_11N09_F_0	BY936775
E_FL_prW-_11N11_F_0	BY936776
E_FL_prW-_11N15_F_0	BY936777
E_FL_prW-_11N19_F_0	BY936778
E_FL_prW-_11N21_F_0	BY936779
E_FL_prW-_11N22_F_0	BY936780
E_FL_prW-_11N23_F_0	BY936781
E_FL_prW-_11O04_F_0	BY936782

E_FL_prW-_11O06_F_0	BY936783
E_FL_prW-_11O08_F_0	BY936784
E_FL_prW-_11O10_F_0	BY936785
E_FL_prW-_11O12_F_0	BY936786
E_FL_prW-_11O14_F_0	BY936787
E_FL_prW-_11O16_F_0	BY936788
E_FL_prW-_11O17_F_0	BY936789
E_FL_prW-_11O21_F_0	BY936790
E_FL_prW-_11O23_F_0	BY936791
E_FL_prW-_11O24_F_0	BY936792
E_FL_prW-_11P02_F_0	BY936793
E_FL_prW-_11P03_F_0	BY936794
E_FL_prW-_11P05_F_0	BY936795
E_FL_prW-_11P06_F_0	BY936796
E_FL_prW-_11P07_F_0	BY936797
E_FL_prW-_11P08_F_0	BY936798
E_FL_prW-_11P09_F_0	BY936799
E_FL_prW-_11P10_F_0	BY936800
E_FL_prW-_11P12_F_0	BY936801
E_FL_prW-_11P13_F_0	BY936802
E_FL_prW-_11P14_F_0	BY936803
E_FL_prW-_11P16_F_0	BY936804
E_FL_prW-_11P17_F_0	BY936805
E_FL_prW-_11P19_F_0	BY936806
E_FL_prW-_11P20_F_0	BY936807
E_FL_prW-_11P21_F_0	BY936808
E_FL_prW-_11P22_F_0	BY936809
E_FL_prW-_11P23_F_0	BY936810
E_FL_prW-_12A05_F_0	BY936811
E_FL_prW-_12A06_F_0	BY936812
E_FL_prW-_12A11_F_0	BY936813
E_FL_prW-_12A12_F_0	BY936814
E_FL_prW-_12A18_F_0	BY936815
E_FL_prW-_12A19_F_0	BY936816
E_FL_prW-_12A22_F_0	BY936817
E_FL_prW-_12A24_F_0	BY936818
E_FL_prW-_12B01_F_0	BY936819
E_FL_prW-_12B02_F_0	BY936820
E_FL_prW-_12B04_F_0	BY936821
E_FL_prW-_12B05_F_0	BY936822
E_FL_prW-_12B06_F_0	BY936823
E_FL_prW-_12B07_F_0	BY936824
E_FL_prW-_12B08_F_0	BY936825
E_FL_prW-_12B09_F_0	BY936826
E_FL_prW-_12B10_F_0	BY936827
E_FL_prW-_12B11_F_0	BY936828
E_FL_prW-_12B12_F_0	BY936829
E_FL_prW-_12B15_F_0	BY936830
E_FL_prW-_12B16_F_0	BY936831
E_FL_prW-_12B18_F_0	BY936832
E_FL_prW-_12B19_F_0	BY936833
E_FL_prW-_12B20_F_0	BY936834
E_FL_prW-_12B21_F_0	BY936835
E_FL_prW-_12B22_F_0	BY936836

E_FL_prW-_12B24_F_0	BY936837
E_FL_prW-_12C01_F_0	BY936838
E_FL_prW-_12C05_F_0	BY936839
E_FL_prW-_12C06_F_0	BY936840
E_FL_prW-_12C11_F_0	BY936841
E_FL_prW-_12C13_F_0	BY936842
E_FL_prW-_12C15_F_0	BY936843
E_FL_prW-_12C17_F_0	BY936844
E_FL_prW-_12C18_F_0	BY936845
E_FL_prW-_12C21_F_0	BY936846
E_FL_prW-_12C23_F_0	BY936847
E_FL_prW-_12C24_F_0	BY936848
E_FL_prW-_12D03_F_0	BY936849
E_FL_prW-_12D04_F_0	BY936850
E_FL_prW-_12D06_F_0	BY936851
E_FL_prW-_12D08_F_0	BY936852
E_FL_prW-_12D09_F_0	BY936853
E_FL_prW-_12D12_F_0	BY936854
E_FL_prW-_12D13_F_0	BY936855
E_FL_prW-_12D14_F_0	BY936856
E_FL_prW-_12D16_F_0	BY936857
E_FL_prW-_12D17_F_0	BY936858
E_FL_prW-_12D19_F_0	BY936859
E_FL_prW-_12D20_F_0	BY936860
E_FL_prW-_12D21_F_0	BY936861
E_FL_prW-_12D22_F_0	BY936862
E_FL_prW-_12E01_F_0	BY936863
E_FL_prW-_12E03_F_0	BY936864
E_FL_prW-_12E06_F_0	BY936865
E_FL_prW-_12E11_F_0	BY936866
E_FL_prW-_12E12_F_0	BY936867
E_FL_prW-_12E15_F_0	BY936868
E_FL_prW-_12E16_F_0	BY936869
E_FL_prW-_12E17_F_0	BY936870
E_FL_prW-_12E18_F_0	BY936871
E_FL_prW-_12E21_F_0	BY936872
E_FL_prW-_12E22_F_0	BY936873
E_FL_prW-_12E23_F_0	BY936874
E_FL_prW-_12E24_F_0	BY936875
E_FL_prW-_12F01_F_0	BY936876
E_FL_prW-_12F04_F_0	BY936877
E_FL_prW-_12F05_F_0	BY936878
E_FL_prW-_12F07_F_0	BY936879
E_FL_prW-_12F09_F_0	BY936880
E_FL_prW-_12F13_F_0	BY936881
E_FL_prW-_12F14_F_0	BY936882
E_FL_prW-_12F15_F_0	BY936883
E_FL_prW-_12F18_F_0	BY936884
E_FL_prW-_12F19_F_0	BY936885
E_FL_prW-_12G05_F_0	BY936886
E_FL_prW-_12G10_F_0	BY936887
E_FL_prW-_12G13_F_0	BY936888
E_FL_prW-_12G14_F_0	BY936889
E_FL_prW-_12G15_F_0	BY936890

E_FL_prW-_12G16_F_0	BY936891
E_FL_prW-_12G22_F_0	BY936892
E_FL_prW-_12G24_F_0	BY936893
E_FL_prW-_12H01_F_0	BY936894
E_FL_prW-_12H02_F_0	BY936895
E_FL_prW-_12H03_F_0	BY936896
E_FL_prW-_12H04_F_0	BY936897
E_FL_prW-_12H05_F_0	BY936898
E_FL_prW-_12H06_F_0	BY936899
E_FL_prW-_12H08_F_0	BY936900
E_FL_prW-_12H12_F_0	BY936901
E_FL_prW-_12H16_F_0	BY936902
E_FL_prW-_12H18_F_0	BY936903
E_FL_prW-_12H19_F_0	BY936904
E_FL_prW-_12H20_F_0	BY936905
E_FL_prW-_12H21_F_0	BY936906
E_FL_prW-_12H22_F_0	BY936907
E_FL_prW-_12H23_F_0	BY936908
E_FL_prW-_12H24_F_0	BY936909
E_FL_prW-_12I11_F_0	BY936910
E_FL_prW-_12I13_F_0	BY936911
E_FL_prW-_12I15_F_0	BY936912
E_FL_prW-_12I18_F_0	BY936913
E_FL_prW-_12I22_F_0	BY936914
E_FL_prW-_12I24_F_0	BY936915
E_FL_prW-_12J01_F_0	BY936916
E_FL_prW-_12J03_F_0	BY936917
E_FL_prW-_12J06_F_0	BY936918
E_FL_prW-_12J07_F_0	BY936919
E_FL_prW-_12J12_F_0	BY936920
E_FL_prW-_12J13_F_0	BY936921
E_FL_prW-_12J18_F_0	BY936922
E_FL_prW-_12J19_F_0	BY936923
E_FL_prW-_12J20_F_0	BY936924
E_FL_prW-_12J21_F_0	BY936925
E_FL_prW-_12J23_F_0	BY936926
E_FL_prW-_12J24_F_0	BY936927
E_FL_prW-_12K02_F_0	BY936928
E_FL_prW-_12K05_F_0	BY936929
E_FL_prW-_12K10_F_0	BY936930
E_FL_prW-_12K11_F_0	BY936931
E_FL_prW-_12K13_F_0	BY936932
E_FL_prW-_12K15_F_0	BY936933
E_FL_prW-_12K17_F_0	BY936934
E_FL_prW-_12K20_F_0	BY936935
E_FL_prW-_12K22_F_0	BY936936
E_FL_prW-_12K23_F_0	BY936937
E_FL_prW-_12L02_F_0	BY936938
E_FL_prW-_12L03_F_0	BY936939
E_FL_prW-_12L04_F_0	BY936940
E_FL_prW-_12L08_F_0	BY936941
E_FL_prW-_12L09_F_0	BY936942
E_FL_prW-_12L13_F_0	BY936943
E_FL_prW-_12L14_F_0	BY936944

E_FL_prW-_12L17_F_0	BY936945
E_FL_prW-_12L19_F_0	BY936946
E_FL_prW-_12L20_F_0	BY936947
E_FL_prW-_12L21_F_0	BY936948
E_FL_prW-_12L22_F_0	BY936949
E_FL_prW-_12M01_F_0	BY936950
E_FL_prW-_12M10_F_0	BY936951
E_FL_prW-_12M11_F_0	BY936952
E_FL_prW-_12M12_F_0	BY936953
E_FL_prW-_12M15_F_0	BY936954
E_FL_prW-_12M17_F_0	BY936955
E_FL_prW-_12M18_F_0	BY936956
E_FL_prW-_12M22_F_0	BY936957
E_FL_prW-_12N01_F_0	BY936958
E_FL_prW-_12N02_F_0	BY936959
E_FL_prW-_12N03_F_0	BY936960
E_FL_prW-_12N04_F_0	BY936961
E_FL_prW-_12N07_F_0	BY936962
E_FL_prW-_12N08_F_0	BY936963
E_FL_prW-_12N11_F_0	BY936964
E_FL_prW-_12N13_F_0	BY936965
E_FL_prW-_12N14_F_0	BY936966
E_FL_prW-_12N17_F_0	BY936967
E_FL_prW-_12N19_F_0	BY936968
E_FL_prW-_12N20_F_0	BY936969
E_FL_prW-_12N22_F_0	BY936970
E_FL_prW-_12O06_F_0	BY936971
E_FL_prW-_12O10_F_0	BY936972
E_FL_prW-_12O14_F_0	BY936973
E_FL_prW-_12O17_F_0	BY936974
E_FL_prW-_12O18_F_0	BY936975
E_FL_prW-_12O22_F_0	BY936976
E_FL_prW-_12O23_F_0	BY936977
E_FL_prW-_12O24_F_0	BY936978
E_FL_prW-_12P01_F_0	BY936979
E_FL_prW-_12P03_F_0	BY936980
E_FL_prW-_12P05_F_0	BY936981
E_FL_prW-_12P07_F_0	BY936982
E_FL_prW-_12P09_F_0	BY936983
E_FL_prW-_12P10_F_0	BY936984
E_FL_prW-_12P15_F_0	BY936985
E_FL_prW-_12P18_F_0	BY936986
E_FL_prW-_12P20_F_0	BY936987
E_FL_prW-_12P22_F_0	BY936988
E_FL_prW-_12P23_F_0	BY936989
E_FL_prW-_13A05_F_0	BY936990
E_FL_prW-_13A06_F_0	BY936991
E_FL_prW-_13A07_F_0	BY936992
E_FL_prW-_13A17_F_0	BY936993
E_FL_prW-_13A23_F_0	BY936994
E_FL_prW-_13B01_F_0	BY936995
E_FL_prW-_13B03_F_0	BY936996
E_FL_prW-_13B04_F_0	BY936997
E_FL_prW-_13B05_F_0	BY936998

E_FL_prW-_13B07_F_0	BY936999
E_FL_prW-_13B08_F_0	BY937000
E_FL_prW-_13B10_F_0	BY937001
E_FL_prW-_13B11_F_0	BY937002
E_FL_prW-_13B14_F_0	BY937003
E_FL_prW-_13B16_F_0	BY937004
E_FL_prW-_13B17_F_0	BY937005
E_FL_prW-_13B19_F_0	BY937006
E_FL_prW-_13B20_F_0	BY937007
E_FL_prW-_13B23_F_0	BY937008
E_FL_prW-_13B24_F_0	BY937009
E_FL_prW-_13C05_F_0	BY937010
E_FL_prW-_13C06_F_0	BY937011
E_FL_prW-_13C07_F_0	BY937012
E_FL_prW-_13C11_F_0	BY937013
E_FL_prW-_13C18_F_0	BY937014
E_FL_prW-_13C21_F_0	BY937015
E_FL_prW-_13C23_F_0	BY937016
E_FL_prW-_13C24_F_0	BY937017
E_FL_prW-_13D01_F_0	BY937018
E_FL_prW-_13D02_F_0	BY937019
E_FL_prW-_13D04_F_0	BY937020
E_FL_prW-_13D06_F_0	BY937021
E_FL_prW-_13D07_F_0	BY937022
E_FL_prW-_13D08_F_0	BY937023
E_FL_prW-_13D09_F_0	BY937024
E_FL_prW-_13D12_F_0	BY937025
E_FL_prW-_13D13_F_0	BY937026
E_FL_prW-_13D14_F_0	BY937027
E_FL_prW-_13D16_F_0	BY937028
E_FL_prW-_13D17_F_0	BY937029
E_FL_prW-_13D20_F_0	BY937030
E_FL_prW-_13D21_F_0	BY937031
E_FL_prW-_13D22_F_0	BY937032
E_FL_prW-_13D23_F_0	BY937033
E_FL_prW-_13E05_F_0	BY937034
E_FL_prW-_13E06_F_0	BY937035
E_FL_prW-_13E07_F_0	BY937036
E_FL_prW-_13E08_F_0	BY937037
E_FL_prW-_13E10_F_0	BY937038
E_FL_prW-_13E11_F_0	BY937039
E_FL_prW-_13E12_F_0	BY937040
E_FL_prW-_13E17_F_0	BY937041
E_FL_prW-_13E19_F_0	BY937042
E_FL_prW-_13E21_F_0	BY937043
E_FL_prW-_13E23_F_0	BY937044
E_FL_prW-_13E24_F_0	BY937045
E_FL_prW-_13F01_F_0	BY937046
E_FL_prW-_13F02_F_0	BY937047
E_FL_prW-_13F05_F_0	BY937048
E_FL_prW-_13F06_F_0	BY937049
E_FL_prW-_13F07_F_0	BY937050
E_FL_prW-_13F08_F_0	BY937051
E_FL_prW-_13F09_F_0	BY937052

E_FL_prW-_13F10_F_0	BY937053
E_FL_prW-_13F11_F_0	BY937054
E_FL_prW-_13F16_F_0	BY937055
E_FL_prW-_13F18_F_0	BY937056
E_FL_prW-_13F20_F_0	BY937057
E_FL_prW-_13F22_F_0	BY937058
E_FL_prW-_13G05_F_0	BY937059
E_FL_prW-_13G06_F_0	BY937060
E_FL_prW-_13G09_F_0	BY937061
E_FL_prW-_13G10_F_0	BY937062
E_FL_prW-_13G11_F_0	BY937063
E_FL_prW-_13G17_F_0	BY937064
E_FL_prW-_13G19_F_0	BY937065
E_FL_prW-_13G21_F_0	BY937066
E_FL_prW-_13G22_F_0	BY937067
E_FL_prW-_13G23_F_0	BY937068
E_FL_prW-_13H02_F_0	BY937069
E_FL_prW-_13H03_F_0	BY937070
E_FL_prW-_13H07_F_0	BY937071
E_FL_prW-_13H08_F_0	BY937072
E_FL_prW-_13H09_F_0	BY937073
E_FL_prW-_13H10_F_0	BY937074
E_FL_prW-_13H17_F_0	BY937075
E_FL_prW-_13H19_F_0	BY937076
E_FL_prW-_13H21_F_0	BY937077
E_FL_prW-_13H24_F_0	BY937078
E_FL_prW-_13I04_F_0	BY937079
E_FL_prW-_13I05_F_0	BY937080
E_FL_prW-_13I06_F_0	BY937081
E_FL_prW-_13I07_F_0	BY937082
E_FL_prW-_13I08_F_0	BY937083
E_FL_prW-_13I09_F_0	BY937084
E_FL_prW-_13I10_F_0	BY937085
E_FL_prW-_13I12_F_0	BY937086
E_FL_prW-_13I18_F_0	BY937087
E_FL_prW-_13I19_F_0	BY937088
E_FL_prW-_13I20_F_0	BY937089
E_FL_prW-_13I21_F_0	BY937090
E_FL_prW-_13I22_F_0	BY937091
E_FL_prW-_13J02_F_0	BY937092
E_FL_prW-_13J04_F_0	BY937093
E_FL_prW-_13J05_F_0	BY937094
E_FL_prW-_13J07_F_0	BY937095
E_FL_prW-_13J08_F_0	BY937096
E_FL_prW-_13J09_F_0	BY937097
E_FL_prW-_13J12_F_0	BY937098
E_FL_prW-_13J13_F_0	BY937099
E_FL_prW-_13J15_F_0	BY937100
E_FL_prW-_13J17_F_0	BY937101
E_FL_prW-_13J18_F_0	BY937102
E_FL_prW-_13J19_F_0	BY937103
E_FL_prW-_13J22_F_0	BY937104
E_FL_prW-_13J24_F_0	BY937105
E_FL_prW-_13K08_F_0	BY937106

E_FL_prW-_13K10_F_0	BY937107
E_FL_prW-_13K11_F_0	BY937108
E_FL_prW-_13K15_F_0	BY937109
E_FL_prW-_13K16_F_0	BY937110
E_FL_prW-_13K19_F_0	BY937111
E_FL_prW-_13K20_F_0	BY937112
E_FL_prW-_13K21_F_0	BY937113
E_FL_prW-_13K22_F_0	BY937114
E_FL_prW-_13K23_F_0	BY937115
E_FL_prW-_13L01_F_0	BY937116
E_FL_prW-_13L02_F_0	BY937117
E_FL_prW-_13L03_F_0	BY937118
E_FL_prW-_13L09_F_0	BY937119
E_FL_prW-_13L10_F_0	BY937120
E_FL_prW-_13L13_F_0	BY937121
E_FL_prW-_13L14_F_0	BY937122
E_FL_prW-_13L15_F_0	BY937123
E_FL_prW-_13L20_F_0	BY937124
E_FL_prW-_13M06_F_0	BY937125
E_FL_prW-_13M07_F_0	BY937126
E_FL_prW-_13M09_F_0	BY937127
E_FL_prW-_13M11_F_0	BY937128
E_FL_prW-_13M12_F_0	BY937129
E_FL_prW-_13M16_F_0	BY937130
E_FL_prW-_13M18_F_0	BY937131
E_FL_prW-_13M19_F_0	BY937132
E_FL_prW-_13M21_F_0	BY937133
E_FL_prW-_13M23_F_0	BY937134
E_FL_prW-_13N02_F_0	BY937135
E_FL_prW-_13N03_F_0	BY937136
E_FL_prW-_13N05_F_0	BY937137
E_FL_prW-_13N07_F_0	BY937138
E_FL_prW-_13N08_F_0	BY937139
E_FL_prW-_13N09_F_0	BY937140
E_FL_prW-_13N10_F_0	BY937141
E_FL_prW-_13N13_F_0	BY937142
E_FL_prW-_13N16_F_0	BY937143
E_FL_prW-_13N17_F_0	BY937144
E_FL_prW-_13N20_F_0	BY937145
E_FL_prW-_13N21_F_0	BY937146
E_FL_prW-_13N22_F_0	BY937147
E_FL_prW-_13O04_F_0	BY937148
E_FL_prW-_13O06_F_0	BY937149
E_FL_prW-_13O12_F_0	BY937150
E_FL_prW-_13O20_F_0	BY937151
E_FL_prW-_13O22_F_0	BY937152
E_FL_prW-_13O24_F_0	BY937153
E_FL_prW-_13P03_F_0	BY937154
E_FL_prW-_13P04_F_0	BY937155
E_FL_prW-_13P05_F_0	BY937156
E_FL_prW-_13P07_F_0	BY937157
E_FL_prW-_13P08_F_0	BY937158
E_FL_prW-_13P09_F_0	BY937159
E_FL_prW-_13P11_F_0	BY937160

E_FL_prW-_13P13_F_0	BY937161
E_FL_prW-_13P14_F_0	BY937162
E_FL_prW-_13P20_F_0	BY937163
E_FL_prW-_13P22_F_0	BY937164
E_FL_prW-_13P24_F_0	BY937165
E_FL_prW-_14A02_F_0	BY937166
E_FL_prW-_14A03_F_0	BY937167
E_FL_prW-_14A04_F_0	BY937168
E_FL_prW-_14A05_F_0	BY937169
E_FL_prW-_14A06_F_0	BY937170
E_FL_prW-_14A09_F_0	BY937171
E_FL_prW-_14A11_F_0	BY937172
E_FL_prW-_14A19_F_0	BY937173
E_FL_prW-_14A21_F_0	BY937174
E_FL_prW-_14A23_F_0	BY937175
E_FL_prW-_14B02_F_0	BY937176
E_FL_prW-_14B07_F_0	BY937177
E_FL_prW-_14B10_F_0	BY937178
E_FL_prW-_14B11_F_0	BY937179
E_FL_prW-_14B13_F_0	BY937180
E_FL_prW-_14B15_F_0	BY937181
E_FL_prW-_14B16_F_0	BY937182
E_FL_prW-_14B18_F_0	BY937183
E_FL_prW-_14B19_F_0	BY937184
E_FL_prW-_14B21_F_0	BY937185
E_FL_prW-_14B22_F_0	BY937186
E_FL_prW-_14B23_F_0	BY937187
E_FL_prW-_14C01_F_0	BY937188
E_FL_prW-_14C02_F_0	BY937189
E_FL_prW-_14C03_F_0	BY937190
E_FL_prW-_14C05_F_0	BY937191
E_FL_prW-_14C06_F_0	BY937192
E_FL_prW-_14C07_F_0	BY937193
E_FL_prW-_14C08_F_0	BY937194
E_FL_prW-_14C09_F_0	BY937195
E_FL_prW-_14C11_F_0	BY937196
E_FL_prW-_14C12_F_0	BY937197
E_FL_prW-_14C13_F_0	BY937198
E_FL_prW-_14C15_F_0	BY937199
E_FL_prW-_14C16_F_0	BY937200
E_FL_prW-_14C18_F_0	BY937201
E_FL_prW-_14D01_F_0	BY937202
E_FL_prW-_14D03_F_0	BY937203
E_FL_prW-_14D06_F_0	BY937204
E_FL_prW-_14D08_F_0	BY937205
E_FL_prW-_14D09_F_0	BY937206
E_FL_prW-_14D11_F_0	BY937207
E_FL_prW-_14D12_F_0	BY937208
E_FL_prW-_14D15_F_0	BY937209
E_FL_prW-_14D16_F_0	BY937210
E_FL_prW-_14D17_F_0	BY937211
E_FL_prW-_14D19_F_0	BY937212
E_FL_prW-_14D21_F_0	BY937213
E_FL_prW-_14D22_F_0	BY937214

E_FL_prW-_14D23_F_0	BY937215
E_FL_prW-_14E01_F_0	BY937216
E_FL_prW-_14E02_F_0	BY937217
E_FL_prW-_14E04_F_0	BY937218
E_FL_prW-_14E05_F_0	BY937219
E_FL_prW-_14E07_F_0	BY937220
E_FL_prW-_14E08_F_0	BY937221
E_FL_prW-_14E11_F_0	BY937222
E_FL_prW-_14E12_F_0	BY937223
E_FL_prW-_14E13_F_0	BY937224
E_FL_prW-_14E14_F_0	BY937225
E_FL_prW-_14E16_F_0	BY937226
E_FL_prW-_14E17_F_0	BY937227
E_FL_prW-_14E18_F_0	BY937228
E_FL_prW-_14E19_F_0	BY937229
E_FL_prW-_14E21_F_0	BY937230
E_FL_prW-_14E23_F_0	BY937231
E_FL_prW-_14F01_F_0	BY937232
E_FL_prW-_14F02_F_0	BY937233
E_FL_prW-_14F03_F_0	BY937234
E_FL_prW-_14F04_F_0	BY937235
E_FL_prW-_14F05_F_0	BY937236
E_FL_prW-_14F06_F_0	BY937237
E_FL_prW-_14F07_F_0	BY937238
E_FL_prW-_14F08_F_0	BY937239
E_FL_prW-_14F10_F_0	BY937240
E_FL_prW-_14F12_F_0	BY937241
E_FL_prW-_14F16_F_0	BY937242
E_FL_prW-_14F17_F_0	BY937243
E_FL_prW-_14F18_F_0	BY937244
E_FL_prW-_14F19_F_0	BY937245
E_FL_prW-_14F21_F_0	BY937246
E_FL_prW-_14F22_F_0	BY937247
E_FL_prW-_14F23_F_0	BY937248
E_FL_prW-_14G01_F_0	BY937249
E_FL_prW-_14G03_F_0	BY937250
E_FL_prW-_14G04_F_0	BY937251
E_FL_prW-_14G05_F_0	BY937252
E_FL_prW-_14G07_F_0	BY937253
E_FL_prW-_14G08_F_0	BY937254
E_FL_prW-_14G10_F_0	BY937255
E_FL_prW-_14G11_F_0	BY937256
E_FL_prW-_14G12_F_0	BY937257
E_FL_prW-_14G13_F_0	BY937258
E_FL_prW-_14G14_F_0	BY937259
E_FL_prW-_14G16_F_0	BY937260
E_FL_prW-_14G19_F_0	BY937261
E_FL_prW-_14G21_F_0	BY937262
E_FL_prW-_14G22_F_0	BY937263
E_FL_prW-_14G24_F_0	BY937264
E_FL_prW-_14H01_F_0	BY937265
E_FL_prW-_14H02_F_0	BY937266
E_FL_prW-_14H03_F_0	BY937267
E_FL_prW-_14H04_F_0	BY937268

E_FL_prW-_14H05_F_0	BY937269
E_FL_prW-_14H09_F_0	BY937270
E_FL_prW-_14H10_F_0	BY937271
E_FL_prW-_14H11_F_0	BY937272
E_FL_prW-_14H12_F_0	BY937273
E_FL_prW-_14H14_F_0	BY937274
E_FL_prW-_14H17_F_0	BY937275
E_FL_prW-_14H18_F_0	BY937276
E_FL_prW-_14H19_F_0	BY937277
E_FL_prW-_14H20_F_0	BY937278
E_FL_prW-_14H21_F_0	BY937279
E_FL_prW-_14H22_F_0	BY937280
E_FL_prW-_14H23_F_0	BY937281
E_FL_prW-_14H24_F_0	BY937282
E_FL_prW-_14I01_F_0	BY937283
E_FL_prW-_14I02_F_0	BY937284
E_FL_prW-_14I03_F_0	BY937285
E_FL_prW-_14I04_F_0	BY937286
E_FL_prW-_14I07_F_0	BY937287
E_FL_prW-_14I08_F_0	BY937288
E_FL_prW-_14I09_F_0	BY937289
E_FL_prW-_14I12_F_0	BY937290
E_FL_prW-_14I13_F_0	BY937291
E_FL_prW-_14I14_F_0	BY937292
E_FL_prW-_14I17_F_0	BY937293
E_FL_prW-_14I18_F_0	BY937294
E_FL_prW-_14I20_F_0	BY937295
E_FL_prW-_14I21_F_0	BY937296
E_FL_prW-_14I22_F_0	BY937297
E_FL_prW-_14I23_F_0	BY937298
E_FL_prW-_14I24_F_0	BY937299
E_FL_prW-_14J03_F_0	BY937300
E_FL_prW-_14J05_F_0	BY937301
E_FL_prW-_14J06_F_0	BY937302
E_FL_prW-_14J07_F_0	BY937303
E_FL_prW-_14J14_F_0	BY937304
E_FL_prW-_14J16_F_0	BY937305
E_FL_prW-_14J19_F_0	BY937306
E_FL_prW-_14J23_F_0	BY937307
E_FL_prW-_14J24_F_0	BY937308
E_FL_prW-_14K02_F_0	BY937309
E_FL_prW-_14K03_F_0	BY937310
E_FL_prW-_14K07_F_0	BY937311
E_FL_prW-_14K08_F_0	BY937312
E_FL_prW-_14K09_F_0	BY937313
E_FL_prW-_14K13_F_0	BY937314
E_FL_prW-_14K14_F_0	BY937315
E_FL_prW-_14K15_F_0	BY937316
E_FL_prW-_14K17_F_0	BY937317
E_FL_prW-_14K19_F_0	BY937318
E_FL_prW-_14K22_F_0	BY937319
E_FL_prW-_14K23_F_0	BY937320
E_FL_prW-_14K24_F_0	BY937321
E_FL_prW-_14L01_F_0	BY937322

E_FL_prW-_14L02_F_0	BY937323
E_FL_prW-_14L04_F_0	BY937324
E_FL_prW-_14L08_F_0	BY937325
E_FL_prW-_14L12_F_0	BY937326
E_FL_prW-_14L16_F_0	BY937327
E_FL_prW-_14L17_F_0	BY937328
E_FL_prW-_14L19_F_0	BY937329
E_FL_prW-_14L20_F_0	BY937330
E_FL_prW-_14L22_F_0	BY937331
E_FL_prW-_14L23_F_0	BY937332
E_FL_prW-_14M01_F_0	BY937333
E_FL_prW-_14M03_F_0	BY937334
E_FL_prW-_14M04_F_0	BY937335
E_FL_prW-_14M05_F_0	BY937336
E_FL_prW-_14M07_F_0	BY937337
E_FL_prW-_14M10_F_0	BY937338
E_FL_prW-_14M12_F_0	BY937339
E_FL_prW-_14M14_F_0	BY937340
E_FL_prW-_14M15_F_0	BY937341
E_FL_prW-_14M17_F_0	BY937342
E_FL_prW-_14M20_F_0	BY937343
E_FL_prW-_14M22_F_0	BY937344
E_FL_prW-_14N01_F_0	BY937345
E_FL_prW-_14N02_F_0	BY937346
E_FL_prW-_14N03_F_0	BY937347
E_FL_prW-_14N04_F_0	BY937348
E_FL_prW-_14N05_F_0	BY937349
E_FL_prW-_14N06_F_0	BY937350
E_FL_prW-_14N07_F_0	BY937351
E_FL_prW-_14N08_F_0	BY937352
E_FL_prW-_14N09_F_0	BY937353
E_FL_prW-_14N10_F_0	BY937354
E_FL_prW-_14N11_F_0	BY937355
E_FL_prW-_14N14_F_0	BY937356
E_FL_prW-_14N18_F_0	BY937357
E_FL_prW-_14N19_F_0	BY937358
E_FL_prW-_14N21_F_0	BY937359
E_FL_prW-_14N22_F_0	BY937360
E_FL_prW-_14N24_F_0	BY937361
E_FL_prW-_14O01_F_0	BY937362
E_FL_prW-_14O02_F_0	BY937363
E_FL_prW-_14O03_F_0	BY937364
E_FL_prW-_14O04_F_0	BY937365
E_FL_prW-_14O05_F_0	BY937366
E_FL_prW-_14O06_F_0	BY937367
E_FL_prW-_14O09_F_0	BY937368
E_FL_prW-_14O10_F_0	BY937369
E_FL_prW-_14O12_F_0	BY937370
E_FL_prW-_14O16_F_0	BY937371
E_FL_prW-_14O19_F_0	BY937372
E_FL_prW-_14O21_F_0	BY937373
E_FL_prW-_14O22_F_0	BY937374
E_FL_prW-_14O23_F_0	BY937375
E_FL_prW-_14O24_F_0	BY937376

	E_FL_prW-_14P01_F_0	BY937377
	E_FL_prW-_14P02_F_0	BY937378
	E_FL_prW-_14P05_F_0	BY937379
	E_FL_prW-_14P06_F_0	BY937380
	E_FL_prW-_14P08_F_0	BY937381
	E_FL_prW-_14P11_F_0	BY937382
	E_FL_prW-_14P12_F_0	BY937383
	E_FL_prW-_14P15_F_0	BY937384
	E_FL_prW-_14P16_F_0	BY937385
	E_FL_prW-_14P18_F_0	BY937386
	E_FL_prW-_14P19_F_0	BY937387
	E_FL_prW-_14P20_F_0	BY937388
	E_FL_prW-_14P21_F_0	BY937389
	E_FL_prW-_14P22_F_0	BY937390
	E_FL_prW-_14P23_F_0	BY937391
	E_FL_prW-_14P24_F_0	BY937392
epV3	epV30002	BP122595
	epV30003	BP122596
	epV30006	BP122597
	epV30007	BP122598
	epV30008	BP122599
	epV30010	BP122601
	epV30011	BP122602
	epV30012X	BP122603
	epV30014X	BP122604
	epV30015X	BP122605
	epV30017	BP122606
	epV30018	BP122607
	epV30019	BP122608
	epV30020	BP122609
	epV30021	BP122610
	epV30022	BP122611
	epV30023	BP122612
	epV30024X	BP122613
	epV30025	BP122614
	epV30026X	BP122615
	epV30027	BP122616
	epV30028	BP122617
	epV30029	BP122618
	epV30031	BP122619
	epV30033	BP122620
	epV30034	BP122621
	epV30035	BP122622
	epV30036	BP122623
	epV30038X	BP122624
	epV30039X	BP122625
	epV30040	BP122626
	epV30041X	BP122627
	epV30042	BP122628
	epV30044X	BP122630
	epV30045	BP122631
	epV30046X	BP122632
	epV30049	BP122633
	epV30051	BP122634

epV30052X	BP122635
epV30053	BP122636
epV30055	BP122637
epV30056	BP122638
epV30058	BP122640
epV30059X	BP122641
epV30060	BP122642
epV30061	BP122643
epV30063	BP122644
epV30064	BP122645
epV30065	BP122646
epV30066	BP122647
epV30067	BP122648
epV30068	BP122649
epV30069	BP122650
epV30072	BP122652
epV30074X	BP122653
epV30075	BP122654
epV30076	BP122655
epV30077	BP122656
epV30078X	BP122657
epV30080X	BP122658
epV30082X	BP122659
epV30083	BP122660
epV30085X	BP122662
epV30086X	BP122663
epV30087	BP122664
epV30088	BP122665
epV30089	BP122666
epV30090	BP122667
epV30091	BP122668
epV30092	BP122669
epV30093	BP122670
epV30094X	BP122671
epV30095	BP122672
epV30096	BP122673
epV31156	BP123526
epV31157	BP123527
epV31158	BP123528
epV31159	BP123529
epV31160	BP123530
epV31166	BP123532
epV31167	BP123533
epV31169	BP123534
epV31170	BP123535
epV31173	BP123536
epV31174	BP123537
epV31175	BP123538
epV31176	BP123539
epV31180	BP123541
epV31182	BP123542
epV31183	BP123543
epV31184	BP123544
epV31188	BP123545

epV31191	BP123546
epV31193	BP123547
epV31196	BP123549
epV31200	BP123552
epV31205	BP123554
epV31206	BP123555
epV31207	BP123556
epV31208	BP123557
epV31210	BP123558
epV31211	BP123559
epV31212	BP123560
epV31215	BP123562
epV31216	BP123563
epV31218	BP123564
epV31219	BP123565
epV31221	BP123566
epV31222	BP123567
epV31224	BP123568
epV31227	BP123569
epV31228	BP123570
epV31229	BP123571
epV31230	BP123572
epV31231	BP123573
epV31232	BP123574
epV31234	BP123575
epV31235	BP123576
epV31237	BP123578
epV31238	BP123579
epV31239	BP123580
epV31241	BP123581
epV31243	BP123582
epV31245	BP123584
epV31248	BP123586
epV31249	BP123587
epV31250	BP123588
epV31251	BP123589
epV31252	BP123590
epV31254	BP123592
epV31255	BP123593
epV31256	BP123594
epV31258	BP123595
epV31259	BP123596
epV31260	BP123597
epV31262	BP123598
epV31263	BP123599
epV31264	BP123600
epV31265	BP123601
epV31267	BP123603
epV31268	BP123604
epV31269	BP123605
epV31270	BP123606
epV31272	BP123607
epV31273	BP123608
epV31274	BP123609

epV31276	BP123610
epV31277	BP123611
epV31278	BP123612
epV31279	BP123613
epV31280	BP123614
epV31281	BP123615
epV31283	BP123617
epV31285	BP123618
epV31287	BP123619
epV31288	BP123620
epV31289	BP123621
epV31290	BP123622
epV31292	BP123623
epV31293	BP123624
epV31294	BP123625
epV31295	BP123626
epV31298	BP123628
epV31299	BP123629
epV31301	BP123630
epV31306	BP123631
epV31307	BP123632
epV31308	BP123633
epV31309	BP123634
epV31310	BP123635
epV31311	BP123636
epV31312	BP123637
epV31313	BP123638
epV31315	BP123640
epV31316	BP123641
epV31319	BP123642
epV31320	BP123643
epV31321	BP123644
epV31322	BP123645
epV31323	BP123646
epV31324	BP123647
epV31326	BP123649
epV31327	BP123650
epV31330	BP123652
epV31331	BP123653
epV31332	BP123654
epV31333	BP123655
epV31336	BP123658
epV31337	BP123659
epV31338	BP123660
epV31339	BP123661
epV31340	BP123662
epV31341	BP123663
epV31342	BP123664
epV31343	BP123665
epV31344	BP123666
epV31345	BP123667
epV31346	BP123668
epV31348	BP123669
epV31351	BP123671

epV31352	BP123672
epV31354	BP123674
epV31355	BP123675
epV31356	BP123676
epV31357	BP123677
epV31359	BP123679
epV31360	BP123680
epV31361	BP123681
epV31362	BP123682
epV31364	BP123684
epV31365	BP123685
epV31366	BP123686
epV31367	BP123687
epV31368	BP123688
epV31369	BP123689
epV31372	BP123690
epV31373	BP123691
epV31374	BP123692
epV31375	BP123693
epV31376	BP123694
epV31377	BP123695
epV31378	BP123696
epV31380	BP123698
epV31381	BP123699
epV31382	BP123700
epV31383	BP123701
epV31384	BP123702
epV31386	BP123703
epV31387	BP123704
epV31388	BP123705
epV31389	BP123706
epV31390	BP123707
epV31392	BP123708
epV31393	BP123709
epV31394	BP123710
epV31395	BP123711
epV31396	BP123712
epV31398	BP123713
epV31399	BP123714
epV31400	BP123715
epV31401	BP123716
epV31402	BP123717
epV31403	BP123718
epV31404	BP123719
epV31405	BP123720
epV31406	BP123721
epV31409	BP123723
epV31410	BP123724
epV31411	BP123725
epV31412	BP123726
epV31413	BP123727
epV31414	BP123728
epV31416	BP123729
epV31417	BP123730

epV31418	BP123731
epV31419	BP123732
epV31420	BP123733
epV31421	BP123734
epV31422	BP123735
epV31424	BP123737
epV31426	BP123738
epV31429	BP123739
epV31430	BP123740
epV31431	BP123741
epV31432	BP123742
epV31433	BP123743
epV31434	BP123744
epV31435	BP123745
epV31436	BP123746
epV31437	BP123747
epV31438	BP123748
epV31440	BP123750
epV31441	BP123751
epV31443	BP123752
epV31444	BP123753
epV31445	BP123754
epV31446	BP123755
epV31447	BP123756
epV31448	BP123757
epV31449	BP123758
epV31450	BP123759
epV31451	BP123760
epV31452	BP123761
epV31453	BP123762
epV31454	BP123763
epV31455	BP123764
epV31456	BP123765
epV31457	BP123766
epV31458	BP123767
epV31460	BP123768
epV31462	BP123770
epV31463	BP123771
epV31464	BP123772
epV31465	BP123773
epV31466	BP123774
epV31467	BP123775
epV31468	BP123776
epV31469	BP123777
epV31470	BP123778
epV31471	BP123779
epV31472	BP123780
epV31473	BP123781
epV31474	BP123782
epV31475	BP123783
epV31476	BP123784
epV31477	BP123785
epV31478	BP123786
epV31479	BP123787

epV31480	BP123788
epV31481	BP123789
epV31482	BP123790
epV31483	BP123791
epV31484	BP123792
epV31485	BP123793
epV31486	BP123794
epV31487	BP123795
epV31489	BP123796
epV31490	BP123797
epV31491	BP123798
epV31493	BP123799
epV31494	BP123800
epV31495	BP123801
epV31497	BP123802
epV31498	BP123803
epV31499	BP123804
epV31500	BP123805
epV31501	BP123806
epV31502	BP123807
epV31503	BP123808
epV31504	BP123809
epV31505	BP123810
epV31506	BP123811
epV31507	BP123812
epV31508	BP123813
epV31509	BP123814
epV31510	BP123815
epV31512	BP123816
epV31513	BP123817
epV31515	BP123818
epV31516	BP123819
epV31517	BP123820
epV31518	BP123821
epV31519	BP123822
epV31520	BP123823
epV31521	BP123824
epV31522	BP123825
epV31524	BP123826
epV31525	BP123827
epV31526	BP123828
epV31527	BP123829
epV31528	BP123830
epV31529	BP123831
epV31530	BP123832
epV31531	BP123833
epV31533	BP123834
epV31535	BP123836
epV31536	BP123837
epV31537	BP123838
epV31538	BP123839
epV31539	BP123840
epV31540	BP123841
epV31541	BP123842

epV31542	BP123843
epV31544	BP123844
epV31545	BP123845
epV31546	BP123846
epV31547	BP123847
epV31548	BP123848
epV31549	BP123849
epV31550	BP123850
epV31551	BP123851
epV31554	BP123852
epV31555	BP123853
epV31557	BP123854
epV31558	BP123855
epV31559	BP123856
epV31560	BP123857
epV31561	BP123858
epV31562	BP123859
epV31563	BP123860
epV31564	BP123861
epV31565	BP123862
epV31566	BP123863
epV31567	BP123864
epV31568	BP123865
epV31570	BP123866
epV31571	BP123867
epV31573	BP123868
epV31574	BP123869
epV31575	BP123870
epV31576	BP123871
epV31578	BP123873
epV31579	BP123874
epV31580	BP123875
epV31581	BP123876
epV31582	BP123877
epV31583	BP123878
epV31584	BP123879
epV31585	BP123880
epV31586	BP123881
epV31587	BP123882
epV31588	BP123883
epV31589	BP123884
epV31590	BP123885
epV31592	BP123886
epV31593	BP123887
epV31595	BP123888
epV31598	BP123890
epV31599	BP123891
epV31601	BP123892
epV31602	BP123893
epV31603	BP123894
epV31604	BP123895
epV31605	BP123896
epV31608	BP123897
epV31609	BP123898

epV31610	BP123899
epV31611	BP123900
epV31612	BP123901
epV31615	BP123903
epV31616	BP123904
epV31617	BP123905
epV31618	BP123906
epV31619	BP123907
epV31620	BP123908
epV31621	BP123909
epV31622	BP123910
epV31624	BP123911
epV31626	BP123913
epV31628	BP123914
epV31630	BP123915
epV31635	BP123916
epV31636	BP123917
epV31637	BP123918
epV31639	BP123919
epV31640	BP123920
epV31648	BP123922
epV31650	BP123924
epV31651	BP123925
epV31653	BP123926
epV31654	BP123927
epV31656	BP123928
epV31660	BP123930
epV31661	BP123931
epV31663	BP123932
epV31664	BP123933
epV31666	BP123934
epV31668	BP123935
epV31671	BP123936
epV31673	BP123938
epV31676	BP123939
epV31677	BP123940
epV31679	BP123942
epV31680	BP123943
epV31683	BP123944
epV31684	BP123945
epV31686	BP123946
epV31690	BP123947
epV31695	BP123949
epV31697	BP123950
epV31700	BP123951
epV31701	BP123952
epV31703	BP123953
epV31704	BP123954
epV31705	BP123955
epV31708	BP123956
epV31709	BP123957
epV31712	BP123959
epV31714	BP123961
epV31716	BP123962

epV31717	BP123963
epV31718	BP123964
epV31721	BP123966
epV31722	BP123967
epV31723	BP123968
epV31726	BP123970
epV31727	BP123971
epV31728	BP123972
epV31731	BP123973
epV31733	BP123974
epV31734	BP123975
epV31736	BP123976
epV31737	BP123977
epV31738	BP123978
epV31743	BP123979
epV31744	BP123980
epV31745	BP123981
epV31748	BP123982
epV31752	BP123983
epV31755	BP123984
epV31756	BP123985
epV31757	BP123986
epV31758	BP123987
epV31759	BP123988
epV31760	BP123989
epV31762	BP123990
epV31768	BP123992
epV31769	BP123993
epV31770	BP123994
epV31773	BP123996
epV31776	BP123997
epV31779	BP123998
epV31781	BP124000
epV31782	BP124001
epV31783	BP124002
epV31785	BP124003
epV31786	BP124004
epV31787	BP124005
epV31788	BP124006
epV31790	BP124007
epV31792	BP124008
epV31793	BP124009
epV31795	BP124011
epV31797	BP124012
epV31798	BP124013
epV31800	BP124014
epV31803	BP124015
epV31804	BP124016
epV31805	BP124017
epV31807	BP124018
epV31808	BP124019
epV31810	BP124020
epV31811	BP124021
epV31812	BP124022

epV31814	BP124024
epV31815	BP124025
epV31816	BP124026
epV31819	BP124027
epV31820	BP124028
epV31823	BP124029
epV31824	BP124030
epV31825	BP124031
epV31826	BP124032
epV31827	BP124033
epV31828	BP124034
epV31829	BP124035
epV31830	BP124036
epV31831	BP124037
epV31832	BP124038
epV31833	BP124039
epV31834	BP124040
epV31835	BP124041
epV31836	BP124042
epV31837	BP124043
epV31838	BP124044
epV31839	BP124045
epV31843	BP124047
epV31844	BP124048
epV31847	BP124049
epV31848	BP124050
epV31849	BP124051
epV31850	BP124052
epV31851	BP124053
epV31852	BP124054
epV31853	BP124055
epV31855	BP124057
epV31856	BP124058
epV31857	BP124059
epV31858	BP124060
epV31859	BP124061
epV31860	BP124062
epV31861	BP124063
epV31862	BP124064
epV31863	BP124065
epV31865	BP124067
epV31866	BP124068
epV31867	BP124069
epV31868	BP124070
epV31869	BP124071
epV31870	BP124072
epV31871	BP124073
epV31872	BP124074
epV31874	BP124076
epV31875	BP124077
epV31877	BP124078
epV31878	BP124079
epV31879	BP124080
epV31880	BP124081

epV31881	BP124082
epV31882	BP124083
epV31883	BP124084
epV31884	BP124085
epV31885	BP124086
epV31886	BP124087
epV31887	BP124088
epV31888	BP124089
epV31890	BP124090
epV31891	BP124091
epV31892	BP124092
epV31893	BP124093
epV31894	BP124094
epV31896	BP124095
epV31897	BP124096
epV31898	BP124097
epV31899	BP124098
epV31900	BP124099
epV31902	BP124100
epV31903	BP124101
epV31904	BP124102
epV31905	BP124103
epV31906	BP124104
epV31907	BP124105
epV31908	BP124106
epV31909	BP124107
epV31910	BP124108
epV31911	BP124109
epV31913	BP124111
epV31914	BP124112
epV31915	BP124113
epV31917	BP124114
epV31918	BP124115
epV31919	BP124116
epV31920	BP124117
epV31922	BP124118
epV31923	BP124119
epV31924	BP124120
epV31925	BP124121
epV31926	BP124122
epV31927	BP124123
epV31928	BP124124
epV31929	BP124125
epV31932	BP124127
epV31934	BP124128
epV31935	BP124129
epV31936	BP124130
epV31937	BP124131
epV31939	BP124132
epV31940	BP124133
epV31941	BP124134
epV31943	BP124135
epV31945	BP124136
epV31946	BP124137

epV31947	BP124138
epV31948	BP124139
epV31949	BP124140
epV31950	BP124141
epV31951	BP124142
epV31952	BP124143
epV31953	BP124144
epV31954	BP124145
epV31955	BP124146
epV31956	BP124147
epV31957	BP124148
epV31958	BP124149
epV31959	BP124150
epV31960	BP124151
epV31961	BP124152
epV31962	BP124153
epV31963	BP124154
epV31964	BP124155
epV31965	BP124156
epV31966	BP124157
epV31969	BP124158
epV31971	BP124159
epV31972	BP124160
epV31973	BP124161
epV31974	BP124162
epV31978	BP124163
epV31979	BP124164
epV31980	BP124165
epV31981	BP124166
epV31982	BP124167
epV31983	BP124168
epV31984	BP124169
epV31985	BP124170
epV31986	BP124171
epV31987	BP124172
epV31988	BP124173
epV31989	BP124174
epV31990	BP124175
epV31992	BP124176
epV31993	BP124177
epV31994	BP124178
epV31995	BP124179
epV31996	BP124180
epV31997	BP124181
epV31998	BP124182
epV32000	BP124183
epV32001	BP124184
epV32002	BP124185
epV32003	BP124186
epV32004	BP124187
epV32006	BP124188
epV32007	BP124189
epV32008	BP124190
epV32009	BP124191

	epV32010	BP124192
	epV32011	BP124193
	epV32012	BP124194
	epV32013	BP124195
	epV32014	BP124196
	epV32015	BP124197
	epV32018	BP124200
	epV32023	BP124203
	epV32024	BP124204
	epV32025	BP124205
	epV32026	BP124206
	epV32027	BP124207
	epV32028	BP124208
	epV32032	BP124209
	epV32033	BP124210
	epV32034	BP124211
	epV32035	BP124212
	epV32038	BP124215
	epV32039	BP124216
	epV32040	BP124217
	epV32043	BP124218
	epV32044	BP124219
	epV32048	BP124221
	epV32049	BP124222
	epV32050	BP124223
	epV32052	BP124225
	epV32055	BP124226
	epV32056	BP124227
	epV32058	BP124228
	epV32059	BP124229
	epV32060	BP124230
	epV32061	BP124231
	epV32062	BP124232
	epV32063	BP124233
	epV32064	BP124234
	epV32067	BP124235
	epV32068	BP124236
	epV32069	BP124237
	epV32070	BP124238
	epV32073	BP124240
	epV32074	BP124241
	epV32075	BP124242
	epV32076	BP124243
	epV32080	BP124245
	epV32083	BP124247
	epV32086	BP124248
	epV32087	BP124249
	epV32088	BP124250
	epV32095	BP124252
	epV32096	BP124253
	epV32098	BP124255
	epV32110	BP124257
	epV32111	BP124258
fbf	fbf0032f	AU000767

fbf0038f	AU000768
fbf0055f	AU000769
fbf0071f	AU000771
fbf0081f	AU000774
fbf0097f	AU000782
fbf0109f	AU000789
fbf0110f	AU000790
fbf0113f	AU000792
fbf0114f	AU000793
fbf0116f	AU000795
fbf0119f	AU000798
fbf0120f	AU000799
fbf0121f	AU000800
fbf0125f	AU000803
fbf0126f	AU000804
fbf0128f	AU000806
fbf0131f	AU000809
fbf0132f	AU000810
fbf0136f	AU000813
fbf0140f	AU000815
fbf0141f	AU000816
fbf0148f	AU000821
fbf0154f	AU000825
fbf0156f	AU000827
fbf0158f	AU000829
fbf0164f	AU000833
fbf0167f	AU000835
fbf0175f	AU000837
fbf0181f	AU000841
fbf0193f	AU000844
fbf0197f	AU000846
fbf0205f	AU000853
fbf0206f	AU000854
fbf0207f	AU000855
fbf0210f	AU000858
fbf0214f	AU000860
fbf0215f	AU000861
fbf0217f	AU000862
fbf0222f	AU000863
fbf0224f	AU000864
fbf0226f	AU000865
fbf0228f	BY945232
fbf0233f	AU000866
fbf0238f	AU000869
fbf0239f	AU000870
fbf0240f	AU000871
fbf0244f	AU000873
fbf0246f	AU000875
fbf0252f	AU000878
fbf0255f	BY945233
fbf0256f	AU000880
fbf0278f	BY945234
fbf0288f	BY945235
fbf0297f	AU000887

fbf0308f	AU000894
fbf0309f	AU000895
fbf0313f	AU000899
fbf0317f	AU000901
fbf0321f	AU000904
fbf0324f	AU000906
fbf0325f	AU000907
fbf0328f	AU000909
fbf0329f	AU000910
fbf0342f	AU000917
fbf0344f	AU000919
fbf0348f	AU000922
fbf0350f	AU000924
fbf0351f	AU000925
fbf0358f	AU000931
fbf0362f	AU000932
fbf0370f	BY945236
fbf0383f	AU000935
fbf0388f	AU000936
fbf0390f	AU000937
fbf0391f	AU000938
fbf0392f	BY945237
fbf0394f	AU000939
fbf0403f	AU000940
fbf0404f	AU000941
fbf0407f	AU000944
fbf0408f	BY945238
fbf0409f	AU000945
fbf0410f	BY945239
fbf0413f	AU000946
fbf0417f	AU000947
fbf0418f	AU000948
fbf0420f	AU000949
fbf0429f	AU000950
fbf0430f	BY945240
fbf0440f	AU000952
fbf0468f	AU000953
fbf0470f	BY945241
fbf0479f	BY945242
fbf0482f	BY945243
fbf0484f	BY945244
fbf0486f	AU000955
fbf0488f	AU000956
fbf0489f	BY945245
fbf0494f	AU000957
fbf0499f	BY945246
fbf0501f	AU000958
fbf0506f	BY945247
fbf0509f	AU000960
fbf0516f	BY945248
fbf0521f	BY945249
fbf0533f	AU000962
fbf0542f	BY945250
fbf0545f	BY945251

fbf0552f	AU000963
fbf0555f	BY945252
fbf0572f	BY945253
fbf0577f	BY945254
fbf0578f	AU000967
fbf0584f	AU000968
fbf0586f	AU000969
fbf0587f	AU000970
fbf0590f	AU000971
fbf0598f	BY945255
fbf0682f	BY945256
fbf0701f	AU000974
fbf0703f	BY945257
fbf0708f	AU000976
fbf0758f	AU000977
fbf0759f	AU000978
fbf0770f	AU000979
fbf0781f	AU000983
fbf0783f	BY945258
fbf0792f	AU000984
fbf0811f	AU000985
fbf0819f	BY945259
fbf0823f	AU000987
fbf0824f	AU000988
fbf0827f	AU000990
fbf0828f	AU000991
fbf0830f	AU000992
fbf0866f	AU000993
fbf0869f	AU000995
fbf0874f	BY945260
fbf0876f	AU000997
fbf0877f	BY945261
fbf0878f	AU000998
fbf0880f	AU000999
fbf0885f	AU001000
fbf0886f	AU001001
fbf0891f	AU001002
fbf0892f	AU001003
fbf0894f	AU001004
fbf0896f	BY945262
fbf0902f	BY945263
fbf0910f	AU001007
fbf0911f	AU001008
fbf0913f	AU001009
fbf0914f	AU001010
fbf0916f	AU001012
fbf0917f	BY945264
fbf0919f	AU001013
fbf0921f	BY945265
fbf0922f	AU001014
fbf0923f	BY945266
fbf0931f	AU001017
fbf0939f	AU001020
fbf0942f	BY945267

	fbf0945f	AU001021
	fbf0950f	AU001023
	fbf0951f	AU001024
	fbf0952f	BY945268
	fbf0953f	AU001025
	fbf0955f	BY945269
	fbf0966f	BY945270
	fbf0970f	AU001026
	fbf0982f	AU001027
	fbf0988f	AU001029
	fbf0991f	BY945271
	fbf0997f	AU001030
	fbf1001f	AU001031
	fbf1002f	AU001032
	fbf1014f	AU001035
	fbf1015f	BY945272
	fbf1022f	AU001036
	fbf1023f	AU001037
	fbf1026f	AU001038
	fbf1034f	AU001039
	fbf1043f	AU001041
	fbf1047f	AU001042
	fbf1049f	AU001044
	fbf1051f	BY945273
	fbf1056f	AU001045
	fbf1060f	BY945274
	fbf1062f	AU001047
	fbf1064f	AU001048
	fbf1072f	AU001051
	fbf1073f	AU001052
	fbf1074f	AU001053
	fbf1076f	AU001055
	fbf1078f	AU001056
	fbf1079f	BY945275
	fbf1084f	BY945276
	fbf1097f	AU001058
	fbf1110f	AU001059
	fbf1120f	AU001060
	fbf1128f	AU001061
	fbf1136f	AU001064
	fbf1162f	BY945277
	fbf1540f	BY945278
	fbf1543f	BY945279
	fbf1576f	BY945280
	fbf1580f	BY945281
	fbf1581f	BY945282
	fbf1588f	BY945283
	fbf1611f	BY945284
	fbf2451f	BY945285
	fbf2456f	BY945286
fbm	fbm0001f	AU001066
	fbm0002f	AU001067
	fbm0011f	AU001075
	fbm0014f	AU001077

fbm0020f	AU001080
fbm0034f	AU001089
fbm0038f	AU001093
fbm0041f	AU001096
fbm0045f	AU001099
fbm0050f	AU001104
fbm0053f	AU001105
fbm0059f	AU001110
fbm0061f	AU001112
fbm0062f	AU001113
fbm0064f	AU001115
fbm0067f	AU001118
fbm0070f	AU001120
fbm0072f	AU001121
fbm0084f	AU001129
fbm0092f	AU001131
fbm0093f	AU001132
fbm0094f	AU001133
fbm0099f	AU001136
fbm0100f	AU001137
fbm0108f	AU001142
fbm0111f	AU001144
fbm0112f	AU001145
fbm0118f	AU001150
fbm0119f	AU001151
fbm0124f	BY945294
fbm0127f	AU001156
fbm0132f	AU001157
fbm0137f	AU001160
fbm0148f	AU001164
fbm0150f	AU001165
fbm0151f	AU001166
fbm0152f	AU001167
fbm0153f	AU001168
fbm0154f	AU001169
fbm0155f	AU001170
fbm0156f	AU001171
fbm0159f	AU001172
fbm0162f	AU001174
fbm0168f	AU001177
fbm0172f	AU001180
fbm0174f	AU001181
fbm0177f	AU001183
fbm0183f	AU001185
fbm0184f	AU001186
fbm0185f	AU001187
fbm0189f	AU001190
fbm0190f	AU001191
fbm0191f	AU001192
fbm0193f	AU001194
fbm0193r	AU001195
fbm0194r	AU001197
fbm0196r	AU001199
fbm0197f	AU001200

fbm0197r	AU001201
fbm0201f	AU001207
fbm0201r	AU001208
fbm0205f	AU001214
fbm0209r	AU001220
fbm0211r	AU001224
fbm0215f	AU001231
fbm0215r	AU001232
fbm0216r	AU001234
fbm0220f	AU001240
fbm0220r	AU001241
fbm0221f	AU001242
fbm0223f	AU001243
fbm0225f	AU001247
fbm0225r	AU001248
fbm0227f	AU001249
fbm0227r	AU001250
fbm0231r	AU001253
fbm0233f	AU001256
fbm0233r	AU001257
fbm0235f	AU001260
fbm0235r	AU001261
fbm0237f	AU001264
fbm0237r	AU001265
fbm0238f	AU001266
fbm0245f	AU001275
fbm0249f	AU001281
fbm0249r	AU001282
fbm0250f	AU001283
fbm0250r	AU001284
fbm0251f	AU001285
fbm0251r	AU001286
fbm0252f	AU001287
fbm0255r	AU001291
fbm0256f	AU001292
fbm0256r	AU001293
fbm0262f	AU001298
fbm0262r	AU001299
fbm0268f	AU001307
fbm0268r	AU001308
fbm0272f	AU001313
fbm0273f	AU001315
fbm0273r	AU001316
fbm0274f	AU001317
fbm0274r	AU001318
fbm0276f	AU001321
fbm0276r	AU001322
fbm0277r	AU001323
fbm0278f	AU001324
fbm0281f	AU001329
fbm0286f	AU001334
fbm0290f	AU001338
fbm0293f	AU001340
fbm0295f	AU001341

fbm0298f	AU001343
fbm0299f	AU001344
fbm0308f	AU001349
fbm0309f	AU001350
fbm0315f	AU001354
fbm0318f	AU001357
fbm0320f	AU001358
fbm0326f	AU001363
fbm0327f	AU001364
fbm0330f	AU001367
fbm0332f	AU001369
fbm0333f	AU001370
fbm0337f	AU001374
fbm0340f	AU001376
fbm0341f	AU001377
fbm0343f	AU001379
fbm0344f	AU001380
fbm0345f	AU001381
fbm0348f	AU001384
fbm0353f	AU001388
fbm0356f	AU001389
fbm0357f	AU001390
fbm0358f	AU001391
fbm0360f	AU001392
fbm0361f	AU001393
fbm0362f	AU001394
fbm0364f	AU001396
fbm0366f	AU001398
fbm0367f	AU001399
fbm0369f	AU001400
fbm0372f	AU001403
fbm0373f	AU001404
fbm0374f	AU001405
fbm0376f	AU001407
fbm0377f	AU001408
fbm0379f	AU001409
fbm0380f	AU001410
fbm0382f	AU001411
fbm0392f	AU001418
fbm0396f	AU001420
fbm0407f	AU001427
fbm0413f	AU001431
fbm0414f	AU001432
fbm0415f	AU001433
fbm0416f	AU001434
fbm0417f	AU001435
fbm0423f	AU001439
fbm0426f	AU001441
fbm0429f	AU001443
fbm0431f	AU001445
fbm0432f	AU001446
fbm0433f	AU001447
fbm0438f	AU001449
fbm0445f	AU001454

fbm0446f	AU001455
fbm0448f	AU001456
fbm0449f	AU001457
fbm0451f	AU001459
fbm0452f	AU001460
fbm0455f	AU001463
fbm0460f	AU001466
fbm0461f	AU001467
fbm0466f	AU001471
fbm0468f	AU001473
fbm0469f	AU001474
fbm0472f	AU001476
fbm0475f	AU001479
fbm0586f	AU001491
fbm0599f	AU001493
fbm0606f	AU001494
fbm0621f	AU001498
fbm0633f	AU001501
fbm0706f	AU001511
fbm0722f	AU001513
fbm0731f	AU001515
fbm0818f	AU001518
fbm0822f	AU001520
fbm0823f	AU001521
fbm0826f	AU001523
fbm0827f	AU001524
fbm0832f	AU001529
fbm0833f	AU001530
fbm0834f	AU001531
fbm0835f	AU001532
fbm0836f	AU001533
fbm0837f	AU001534
fbm0838f	AU001535
fbm0840f	AU001536
fbm0932f	AU001538
fbm0933f	AU001539
fbm0941f	AU001542
fbm0942f	AU001543
fbm0946f	AU001545
fbm0956f	AU001548
fbm0961f	AU001549
fbm0963f	AU001550
fbm0976f	AU001555
fbm0983f	AU001559
fbm0986f	AU001561
fbm0997f	AU001567
fbm0998f	AU001568
fbm1005f	AU001573
fbm1006f	AU001574
fbm1010f	AU001577
fbm1011f	AU001578
fbm1021f	AU001583
fbm1022f	AU001584
fbm1023f	AU001585

fbm1031f	AU001587
fbm1035f	AU001588
fbm1038f	AU001591
fbm1047f	AU001595
fbm1050f	AU001596
fbm1055f	AU001599
fbm1060f	AU001603
fbm1061f	AU001604
fbm1062f	AU001605
fbm1064f	AU001607
fbm1074f	AU001613
fbm1076f	AU001614
fbm1077f	AU001615
fbm1080f	AU001618
fbm1083f	AU001620
fbm1087f	AU001623
fbm1090f	AU001626
fbm1092f	AU001628
fbm1098f	AU001633
fbm1099f	AU001634
fbm1100f	AU001635
fbm1102f	AU001636
fbm1104f	AU001638
fbm1106f	AU001640
fbm1108f	AU001642
fbm1113f	AU001643
fbm1115f	AU001645
fbm1119f	AU001648
fbm1120f	AU001649
fbm1122f	AU001651
fbm1123f	AU001652
fbm1124f	AU001653
fbm1126f	AU001655
fbm1130f	AU001658
fbm1131f	AU001659
fbm1132f	AU001660
fbm1141f	AU001662
fbm1150f	AU001668
fbm1152f	AU001670
fbm1159f	AU001674
fbm1164f	AU001679
fbm1166f	AU001681
fbm1167f	AU001682
fbm1168f	AU001683
fbm1170f	AU001684
fbm1173f	AU001687
fbm1176f	AU001690
fbm1177f	AU001691
fbm1180f	AU001692
fbm1182f	AU001694
fbm1183f	AU001695
fbm1184f	AU001696
fbm1185f	AU001697
fbm1189f	AU001699

fbm1195f	AU001703
fbm1198f	AU001706
fbm1199f	AU001707
fbm1204f	AU001712
fbm1206f	AU001714
fbm1208f	AU001716
fbm1210f	AU001718
fbm1216f	AU001722
fbm1217f	AU001723
fbm1220f	AU001726
fbm1221f	AU001727
fbm1222f	AU001728
fbm1224f	AU001730
fbm1228f	AU001732
fbm1266f	AU001737
fbm1267f	AU001738
fbm1271f	AU001742
fbm1272f	AU001743
fbm1275f	AU001745
fbm1284f	AU001751
fbm1295f	AU001756
fbm1301f	AU001758
fbm1307f	AU001762
fbm1309f	AU001763
fbm1311f	AU001765
fbm1316f	AU001768
fbm1323f	AU001773
fbm1328f	AU001775
fbm1330f	AU001777
fbm1338f	AU001783
fbm1339f	AU001784
fbm1341f	AU001786
fbm1345f	AU001790
fbm1346f	AU001791
fbm1355f	AU001795
fbm1358f	AU001796
fbm1362f	AU001798
fbm1364f	AU001800
fbm1365f	AU001801
fbm1370f	AU001805
fbm1414f	AU001837
fbm1419f	AU001838
fbm1424f	AU001840
fbm1431f	AU001843
fbm1437f	AU001845
fbm1438f	AU001846
fbm1439f	AU001847
fbm1441f	AU001848
fbm1445f	AU001849
fbm1450f	AU001850
fbm1451f	AU001851
fbm1453f	AU001852
fbm1454f	AU001853
fbm1456f	AU001854

	fbm1457f	AU001855
	fbm1458f	AU001856
	fbm1459f	AU001857
	fbm1466f	BY945287
	fbm1467f	AU001858
	fbm1469f	BY945288
	fbm1471f	AU001859
	fbm1475f	AU001860
	fbm1483f	AU001861
	fbm1485f	AU001862
	fbm1486f	BY945289
	fbm1487f	AU001863
	fbm1491f	AU001864
	fbm1492f	BY945290
	fbm1494f	BY945291
	fbm1498f	AU001865
	fbm1499f	AU001866
	fbm1500f	AU001867
	fbm1580f	BY945292
	fbm1590f	BY945293
fbpv	fbpv0002	BP124846
	fbpv0003	BP124847
	fbpv0007	BP124850
	fbpv0008	BP124851
	fbpv0010	BP124852
	fbpv0011	BP124853
	fbpv0012	BP124854
	fbpv0013	BP124855
	fbpv0014	BP124856
	fbpv0015	BP124857
	fbpv0016	BP124858
	fbpv0017	BP124859
	fbpv0018	BP124860
	fbpv0019	BP124861
	fbpv0020	BP124862
	fbpv0021	BY915277
	fbpv0022	BP124863
	fbpv0023	BP124864
	fbpv0024	BP124865
	fbpv0025	BP124866
	fbpv0026	BP124867
	fbpv0028	BP124869
	fbpv0029	BP124870
	fbpv0031	BP124872
	fbpv0032	BP124873
	fbpv0033	BP124874
	fbpv0034	BP124875
	fbpv0035	BP124876
	fbpv0036_1	BY915278
	fbpv0037	BP124878
	fbpv0038	BP124879
	fbpv0039	BP124880
	fbpv0041	BP124882
	fbpv0042	BP124883

fbpv0043	BP124884
fbpv0044	BP124885
fbpv0045	BP124886
fbpv0046	BP124887
fbpv0047	BP124888
fbpv0048	BP124889
fbpv0049	BP124890
fbpv0050	BP124891
fbpv0052	BP124892
fbpv0055	BP124894
fbpv0056	BP124895
fbpv0057	BP124896
fbpv0058	BP124897
fbpv0059	BP124898
fbpv0060	BP124899
fbpv0061	BP124900
fbpv0063	BP124901
fbpv0064	BP124902
fbpv0065	BP124903
fbpv0066	BP124904
fbpv0067	BP124905
fbpv0068	BP124906
fbpv0069	BP124907
fbpv0071	BP124908
fbpv0073	BP124909
fbpv0074	BP124910
fbpv0075	BP124911
fbpv0076	BP124912
fbpv0077	BP124913
fbpv0079	BP124914
fbpv0081	BY915279
fbpv0083	BP124916
fbpv0084_1	BY915280
fbpv0085	BP124918
fbpv0086	BP124919
fbpv0087	BP124920
fbpv0088	BP124921
fbpv0090	BP124923
fbpv0091	BP124924
fbpv0092	BP124925
fbpv0093	BP124926
fbpv0094	BP124927
fbpv0095	BP124928
fbpv0096	BP124929
fbpv0100	BP124931
fbpv0102	BP124932
fbpv0103	BP124933
fbpv0104	BP124934
fbpv0106	BP124935
fbpv0107	BP124936
fbpv0108	BP124937
fbpv0111	BP124938
fbpv0112	BP124939
fbpv0114	BP124940

fbpv0115	BP124941
fbpv0116	BP124942
fbpv0117	BP124943
fbpv0118	BP124944
fbpv0119	BP124945
fbpv0120	BP124946
fbpv0123	BP124947
fbpv0124	BP124948
fbpv0126	BP124949
fbpv0128	BP124951
fbpv0129	BP124952
fbpv0130	BP124953
fbpv0131	BP124954
fbpv0132	BP124955
fbpv0133	BY915281
fbpv0135	BP124956
fbpv0136	BP124957
fbpv0137	BP124958
fbpv0138	BP124959
fbpv0140	BP124961
fbpv0141	BP124962
fbpv0142	BP124963
fbpv0143	BP124964
fbpv0149	BP124968
fbpv0151	BP124970
fbpv0152	BP124971
fbpv0153	BP124972
fbpv0154	BP124973
fbpv0156	BP124975
fbpv0160	BP124976
fbpv0162	BP124977
fbpv0163	BP124978
fbpv0164	BP124979
fbpv0165	BP124980
fbpv0166	BP124981
fbpv0167	BP124982
fbpv0168	BP124983
fbpv0169	BP124984
fbpv0172	BP124985
fbpv0173	BP124986
fbpv0174	BP124987
fbpv0175	BP124988
fbpv0176	BP124989
fbpv0177	BP124990
fbpv0178	BP124991
fbpv0179	BP124992
fbpv0180	BP124993
fbpv0184	BP124994
fbpv0185	BP124995
fbpv0187	BP124996
fbpv0190	BP124999
fbpv0191	BP125000
fbpv0193	BP125001
fbpv0194	BP125002

fbpv0195	BP125003
fbpv0196	BP125004
fbpv0197	BP125005
fbpv0198	BP125006
fbpv0199	BP125007
fbpv0200	BP125008
fbpv0203	BP125011
fbpv0204	BP125012
fbpv0205	BP125013
fbpv0206	BP125014
fbpv0207	BP125015
fbpv0208	BP125016
fbpv0210	BP125018
fbpv0211	BP125019
fbpv0212	BP125020
fbpv0213	BP125021
fbpv0216	BP125023
fbpv0217	BP125024
fbpv0218	BP125025
fbpv0219	BP125026
fbpv0222	BP125027
fbpv0223	BP125028
fbpv0224	BP125029
fbpv0225	BP125030
fbpv0226	BP125031
fbpv0231	BP125035
fbpv0232	BP125036
fbpv0236	BP125037
fbpv0237	BP125038
fbpv0238	BP125039
fbpv0239	BP125040
fbpv0241	BP125041
fbpv0242	BP125042
fbpv0243	BP125043
fbpv0244	BP125044
fbpv0245	BP125045
fbpv0246	BP125046
fbpv0247	BP125047
fbpv0248	BP125048
fbpv0249	BP125049
fbpv0250	BP125050
fbpv0251	BP125051
fbpv0253	BP125052
fbpv0254	BP125053
fbpv0256	BP125054
fbpv0257	BP125055
fbpv0258	BP125056
fbpv0259	BP125057
fbpv0260	BP125058
fbpv0262	BP125059
fbpv0266	BP125060
fbpv0269	BP125063
fbpv0270	BP125064
fbpv0272	BP125066

fbpv0273	BP125067
fbpv0275	BP125069
fbpv0277	BP125071
fbpv0278	BP125072
fbpv0279	BP125073
fbpv0280	BP125074
fbpv0281	BP125075
fbpv0283	BP125076
fbpv0284	BP125077
fbpv0287	BP125078
fbpv0290	BP125081
fbpv0291	BP125082
fbpv0292	BP125083
fbpv0296	BP125084
fbpv0297	BP125085
fbpv0298	BP125086
fbpv0301	BP125087
fbpv0303	BP125088
fbpv0311	BP125090
fbpv0312	BP125091
fbpv0314	BP125092
fbpv0317	BP125094
fbpv0321	BP125097
fbpv0322	BP125098
fbpv0324	BP125099
fbpv0330	BP125100
fbpv0331	BP125101
fbpv0332	BP125102
fbpv0334	BP125103
fbpv0336	BP125104
fbpv0337	BY915282
fbpv0338	BY915283
fbpv0339	BY915284
fbpv0340	BY915285
fbpv0342	BY915286
fbpv0343	BY915287
fbpv0344	BY915288
fbpv0348	BY915289
fbpv0349	BY915290
fbpv0356	BY915291
fbpv0357	BY915292
fbpv0361	BY915293
fbpv0363	BY915294
fbpv0364	BY915295
fbpv0365	BY915296
fbpv0369	BY915297
fbpv0370	BY915298
fbpv0371	BY915299
fbpv0372	BY915300
fbpv0373	BY915301
fbpv0374	BY915302
fbpv0376	BY915303
fbpv0381	BY915304
fbpv0383	BY915305

fbpv0384	BY915306
fbpv0386	BP125105
fbpv0387	BP125106
fbpv0388	BP125107
fbpv0390	BY915307
fbpv0391	BP125108
fbpv0392	BP125109
fbpv0396	BP125111
fbpv0397	BP125112
fbpv0398	BP125113
fbpv0399	BP125114
fbpv0401	BP125116
fbpv0404	BP125118
fbpv0405	BP125119
fbpv0406	BP125120
fbpv0407	BP125121
fbpv0408	BP125122
fbpv0409	BP125123
fbpv0410	BP125124
fbpv0411	BP125125
fbpv0412	BP125126
fbpv0413	BP125127
fbpv0414	BP125128
fbpv0415	BP125129
fbpv0416	BP125130
fbpv0417	BP125131
fbpv0418	BP125132
fbpv0421	BP125133
fbpv0423	BP125134
fbpv0425	BP125135
fbpv0428	BP125137
fbpv0430	BP125138
fbpv0432	BP125140
fbpv0433	BP125141
fbpv0434	BP125142
fbpv0438	BP125143
fbpv0441	BP125145
fbpv0442	BP125146
fbpv0443	BP125147
fbpv0444	BP125148
fbpv0447	BP125151
fbpv0448	BP125152
fbpv0449	BP125153
fbpv0451	BP125155
fbpv0452	BP125156
fbpv0453	BP125157
fbpv0454	BP125158
fbpv0455	BP125159
fbpv0456	BP125160
fbpv0458	BP125161
fbpv0461	BP125162
fbpv0462	BY915308
fbpv0464	BP125164
fbpv0466	BP125166

fbpv0467	BP125167
fbpv0468	BP125168
fbpv0469	BP125169
fbpv0470	BP125170
fbpv0472	BP125171
fbpv0473	BP125172
fbpv0474	BP125173
fbpv0478	BP125176
fbpv0479	BY915309
fbpv0480	BP125177
fbpv0483	BP125178
fbpv0485	BP125180
fbpv0486	BP125181
fbpv0487	BP125182
fbpv0491	BP125185
fbpv0492	BP125186
fbpv0493	BP125187
fbpv0494	BP125188
fbpv0500	BY915310
fbpv0501	BY915311
fbpv0502	BY915312
fbpv0503	BY915313
fbpv0505	BY915314
fbpv0506	BY915315
fbpv0507	BP125191
fbpv0508	BP125192
fbpv0510	BP125193
fbpv0511	BP125194
fbpv0513	BP125195
fbpv0514	BP125196
fbpv0516	BP125198
fbpv0517	BP125199
fbpv0518	BP125200
fbpv0520	BP125201
fbpv0522	BY915316
fbpv0523	BP125202
fbpv0524	BP125203
fbpv0525	BP125204
fbpv0533	BP125206
fbpv0536	BP125207
fbpv0537	BP125208
fbpv0538	BP125209
fbpv0539	BP125210
fbpv0540	BP125211
fbpv0541	BP125212
fbpv0542	BP125213
fbpv0547	BP125216
fbpv0549	BP125217
fbpv0551	BP125218
fbpv0552	BP125219
fbpv0556	BP125221
fbpv0557	BP125222
fbpv0558	BP125223
fbpv0559	BP125224

fbpv0561	BP125226
fbpv0565	BP125228
fbpv0570	BP125230
fbpv0571	BP125231
fbpv0572	BP125232
fbpv0577	BP125235
fbpv0579	BP125237
fbpv0581	BP125239
fbpv0583	BP125241
fbpv0584	BP125242
fbpv0586	BP125243
fbpv0587	BP125244
fbpv0588	BP125245
fbpv0589	BY915317
fbpv0590	BY915318
fbpv0592	BY915319
fbpv0593	BP125246
fbpv0596	BP125247
fbpv0601	BP125248
fbpv0602	BP125249
fbpv0604	BP125251
fbpv0605	BP125252
fbpv0606	BP125253
fbpv0607	BP125254
fbpv0609	BP125255
fbpv0610	BP125256
fbpv0611	BP125257
fbpv0612	BP125258
fbpv0613	BP125259
fbpv0614	BP125260
fbpv0615	BP125261
fbpv0616	BP125262
fbpv0617	BP125263
fbpv0619	BP125264
fbpv0620	BP125265
fbpv0621	BP125266
fbpv0622	BP125267
fbpv0623	BP125268
fbpv0624	BP125269
fbpv0625	BP125270
fbpv0626	BP125271
fbpv0628	BP125273
fbpv0629	BP125274
fbpv0630	BP125275
fbpv0631	BP125276
fbpv0634	BP125278
fbpv0635	BP125279
fbpv0636	BP125280
fbpv0637	BP125281
fbpv0638	BP125282
fbpv0639	BP125283
fbpv0641	BP125285
fbpv0642	BP125286
fbpv0644	BP125288

fbpv0645	BP125289
fbpv0647	BP125290
fbpv0648	BP125291
fbpv0649	BP125292
fbpv0650	BP125293
fbpv0651	BP125294
fbpv0653	BP125296
fbpv0655	BP125298
fbpv0657	BP125299
fbpv0658	BP125300
fbpv0659	BP125301
fbpv0660	BP125302
fbpv0661	BP125303
fbpv0662	BP125304
fbpv0665	BP125307
fbpv0666	BP125308
fbpv0671	BP125310
fbpv0673	BP125312
fbpv0674	BP125313
fbpv0675	BP125314
fbpv0677	BP125316
fbpv0678	BP125317
fbpv0684	BP125321
fbpv0685	BP125322
fbpv0686	BP125323
fbpv0687	BY915320
fbpv0689	BP125325
fbpv0690	BP125326
fbpv0692	BP125327
fbpv0693	BP125328
fbpv0698	BP125329
fbpv0700	BP125330
fbpv0701	BP125331
fbpv0702	BP125332
fbpv0705	BP125333
fbpv0709	BP125335
fbpv0710	BY915321
fbpv0713	BP125338
fbpv0715	BP125339
fbpv0717	BP125341
fbpv0718	BP125342
fbpv0721	BP125344
fbpv0722	BP125345
fbpv0723	BP125346
fbpv0727	BP125349
fbpv0730	BP125351
fbpv0732	BP125352
fbpv0733	BP125353
fbpv0735	BP125354
fbpv0738	BP125356
fbpv0739	BP125357
fbpv0741	BP125358
fbpv0742	BP125359
fbpv0744	BP125361

fbpv0747	BP125363
fbpv0749	BP125364
fbpv0751	BP125365
fbpv0752	BP125366
fbpv0755	BP125368
fbpv0756	BP125369
fbpv0758	BP125370
fbpv0760	BP125372
fbpv0762	BP125374
fbpv0763	BP125375
fbpv0766	BP125376
fbpv0768	BP125378
fbpv0769	BP125379
fbpv0770	BP125380
fbpv0771	BP125381
fbpv0773	BP125383
fbpv0775	BP125385
fbpv0776	BP125386
fbpv0777	BP125387
fbpv0778	BP125388
fbpv0781	BP125389
fbpv0782	BP125390
fbpv0783	BP125391
fbpv0785	BP125392
fbpv0786	BP125393
fbpv0787	BY915322
fbpv0788	BP125394
fbpv0791	BY915323
fbpv0792	BP125396
fbpv0793	BP125397
fbpv0794	BP125398
fbpv0795	BP125399
fbpv0796	BP125400
fbpv0798	BP125402
fbpv0799	BP125403
fbpv0800	BP125404
fbpv0801	BP125405
fbpv0802	BP125406
fbpv0803	BP125407
fbpv0804	BP125408
fbpv0805	BP125409
fbpv0806	BP125410
fbpv0807	BP125411
fbpv0808	BP125412
fbpv0809	BP125413
fbpv0811	BP125415
fbpv0813	BP125417
fbpv0814	BP125418
fbpv0815	BY915324
fbpv0818	BP125421
fbpv0821	BP125424
fbpv0822	BP125425
fbpv0823	BP125426
fbpv0824	BP125427

fbpv0826	BP125428
fbpv0827	BP125429
fbpv0829	BP125430
fbpv0830	BP125431
fbpv0831	BP125432
fbpv0833	BP125434
fbpv0834	BP125435
fbpv0835	BP125436
fbpv0836	BP125437
fbpv0839	BP125439
fbpv0841	BP125441
fbpv0842	BP125442
fbpv0844	BP125443
fbpv0845	BP125444
fbpv0846	BY915325
fbpv0847	BP125445
fbpv0851	BP125448
fbpv0852	BP125449
fbpv0853	BP125450
fbpv0854	BP125451
fbpv0855	BY915326
fbpv0856	BP125452
fbpv0857	BP125453
fbpv0858	BP125454
fbpv0859	BP125455
fbpv0861	BP125457
fbpv0862	BP125458
fbpv0863	BP125459
fbpv0865	BP125460
fbpv0866_1	BY915327
fbpv0867	BP125462
fbpv0868	BP125463
fbpv0869	BP125464
fbpv0871	BP125465
fbpv0872	BP125466
fbpv0873	BP125467
fbpv0874	BP125468
fbpv0875	BP125469
fbpv0876	BP125470
fbpv0877	BP125471
fbpv0878	BP125472
fbpv0879	BP125473
fbpv0882	BP125474
fbpv0886	BP125476
fbpv0887	BP125477
fbpv0888	BP125478
fbpv0889	BP125479
fbpv0891	BP125481
fbpv0892	BP125482
fbpv0893	BP125483
fbpv0894	BY915328
fbpv0895	BP125484
fbpv0896	BP125485
fbpv0897	BP125486

	fbpv0898	BP125487
	fbpv0899	BY915329
	fbpv0900	BP125488
	fbpv0903	BP125489
	fbpv0905	BP125491
	fbpv0906	BY915330
	fbpv0907	BP125492
	fbpv0908	BP125493
	fbpv0909	BY915331
	fbpv0910	BP125494
	fbpv0911	BY915332
	fbpv0912	BP125495
	fbpv0913	BP125496
	fbpv0914	BP125497
	fbpv0916	BP125499
	fbpv0917	BP125500
	fbpv0918	BP125501
	fbpv0920	BP125502
	fbpv0921	BP125503
	fbpv0922	BP125504
	fbpv0924	BP125506
	fbpv0925	BP125507
	fbpv0926	BP125508
	fbpv0927	BP125509
	fbpv0928	BP125510
	fbpv0929	BP125511
	fbpv0930	BP125512
	fbpv0931	BP125513
	fbpv0933	BY915333
	fbpv0935	BP125514
	fbpv0937	BP125515
	fbpv0938	BP125516
	fbpv0939	BP125517
	fbpv0941	BP125518
	fbpv0942	BP125519
	fbpv0943	BP125520
	fbpv0944	BP125521
	fbpv0945	BP125522
	fbpv0946	BP125523
	fbpv0947	BP125524
	fbpv0950	BP125525
	fbpv0951	BP125526
	fbpv0952	BP125527
	fbpv0954	BP125529
	fbpv0957	BP125530
	fbpv0958	BP125531
	fbpv0959	BP125532
	fbpv0960	BY915334
fbS2	fbS20003	BP124564
	fbS20010	BP124566
	fbS20012	BP124567
	fbS20013	BP124568
	fbS20015	BP124569
	fbS20016	BP124570

fbS20017	BP124571
fbS20019	BP124573
fbS20020	BP124574
fbS20021	BP124575
fbS20022	BP124576
fbS20024	BP124577
fbS20025	BP124578
fbS20026	BP124579
fbS20027	BP124580
fbS20028	BP124581
fbS20029	BP124582
fbS20030	BP124583
fbS20033	BP124585
fbS20034	BP124586
fbS20036	BP124588
fbS20037	BP124589
fbS20038	BP124590
fbS20039	BP124591
fbS20041	BP124593
fbS20042	BP124594
fbS20043	BP124595
fbS20044	BP124596
fbS20046	BP124598
fbS20047	BP124599
fbS20048	BP124600
fbS20049	BP124601
fbS20053	BP124602
fbS20056	BP124603
fbS20057X_1	BY915335
fbS20058	BP124605
fbS20060X_1	BY915336
fbS20061	BP124607
fbS20062	BP124608
fbS20064	BP124609
fbS20065	BP124610
fbS20066	BP124611
fbS20067	BP124612
fbS20068	BP124613
fbS20069	BP124614
fbS20070	BP124615
fbS20072	BP124616
fbS20073	BP124617
fbS20074	BP124618
fbS20075	BP124619
fbS20076	BP124620
fbS20078	BP124622
fbS20079	BP124623
fbS20080	BP124624
fbS20082	BP124626
fbS20083	BP124627
fbS20084	BP124628
fbS20085	BP124629
fbS20086X	BP124630
fbS20087	BP124631

fbS20090	BP124633
fbS20091	BP124634
fbS20092	BP124635
fbS20093	BP124636
fbS20094	BP124637
fbS20095	BP124638
fbS20096	BP124639
fbS20098	BP124641
fbS20099	BP124642
fbS20100	BP124643
fbS20101	BP124644
fbS20102	BP124645
fbS20103	BP124646
fbS20104	BP124647
fbS20105	BP124648
fbS20106	BP124649
fbS20107	BP124650
fbS20108	BP124651
fbS20110	BP124652
fbS20111	BP124653
fbS20112	BP124654
fbS20113	BP124655
fbS20114	BP124656
fbS20115	BP124657
fbS20116	BP124658
fbS20117	BP124659
fbS20118	BP124660
fbS20119	BP124661
fbS20120	BP124662
fbS20121	BP124663
fbS20123	BP124665
fbS20124	BP124666
fbS20125	BP124667
fbS20126	BP124668
fbS20127	BP124669
fbS20128	BP124670
fbS20129	BP124671
fbS20131	BP124672
fbS20132	BP124673
fbS20135	BP124674
fbS20136	BP124675
fbS20137	BP124676
fbS20138	BP124677
fbS20139	BP124678
fbS20141	BP124679
fbS20142	BP124680
fbS20143	BP124681
fbS20144	BP124682
fbS20147	BP124683
fbS20150	BP124684
fbS20151	BP124685
fbS20152	BP124686
fbS20153	BP124687
fbS20154	BP124688

fbS20155	BP124689
fbS20156	BP124690
fbS20158	BP124691
fbS20159	BP124692
fbS20161	BP124693
fbS20162	BP124694
fbS20164	BP124695
fbS20166	BP124696
fbS20167	BP124697
fbS20168	BP124698
fbS20170	BP124699
fbS20175	BP124701
fbS20176	BP124702
fbS20177	BP124703
fbS20180	BP124705
fbS20181X	BP124706
fbS20183	BP124707
fbS20185	BP124708
fbS20186	BP124709
fbS20187	BP124710
fbS20188	BP124711
fbS20189	BP124712
fbS20191	BP124714
fbS20192	BP124715
fbS20193	BP124716
fbS20194	BP124717
fbS20195	BP124718
fbS20197	BP124719
fbS20200	BP124720
fbS20202	BP124722
fbS20203	BP124723
fbS20205	BP124724
fbS20206	BP124725
fbS20211	BP124726
fbS20212	BP124727
fbS20214	BP124728
fbS20215	BP124729
fbS20218	BP124730
fbS20219	BP124731
fbS20220	BP124732
fbS20221	BP124733
fbS20222	BP124734
fbS20228	BP124735
fbS20230	BP124736
fbS20232	BP124738
fbS20233	BP124739
fbS20234	BP124740
fbS20235	BP124741
fbS20236	BP124742
fbS20237	BP124743
fbS20239	BP124744
fbS20243	BP124745
fbS20244	BP124746
fbS20246	BP124748

fbS20247	BP124749
fbS20249	BP124750
fbS20252	BP124751
fbS20256	BP124752
fbS20257	BP124753
fbS20260	BP124754
fbS20263	BP124755
fbS20265	BP124756
fbS20270	BP124757
fbS20272	BP124759
fbS20275	BP124760
fbS20282	BP124762
fbS20283	BP124763
fbS20288	BP124764
fbS20290	BP124765
fbS20292	BP124767
fbS20293	BP124768
fbS20294	BP124769
fbS20295	BP124770
fbS20296	BP124771
fbS20298	BP124773
fbS20299	BP124774
fbS20302	BP124776
fbS20309	BP124777
fbS20310	BP124778
fbS20311	BP124779
fbS20312	BP124780
fbS20318	BP124782
fbS20319	BP124783
fbS20321	BP124784
fbS20323	BP124785
fbS20325	BP124786
fbS20328	BP124787
fbS20329	BP124788
fbS20332	BP124789
fbS20333	BP124790
fbS20334	BP124791
fbS20387	BY915337
fbS20394	BY915338
fbS20396	BY915339
fbS20398	BY915340
fbS20400	BY915341
fbS20406	BY915342
fbS20407	BY915343
fbS20408	BY915344
fbS20414	BY915345
fbS20416	BY915346
fbS20417	BY915347
fbS20418	BY915348
fbS20419	BY915349
fbS20421	BY915350
fbS20422	BY915351
fbS20425	BY915352
fbS20426	BY915353

fbS20427	BY915354
fbS20428	BY915355
fbS20429	BY915356
fbS20430	BY915357
fbS20432	BY915358
fbS20433	BY915359
fbS20436	BY915360
fbS20443	BY915361
fbS20445	BY915362
fbS20446	BY915363
fbS20447	BY915364
fbS20448	BY915365
fbS20450	BY915366
fbS20453	BY915367
fbS20454	BY915368
fbS20455	BY915369
fbS20456	BY915370
fbS20461	BY915371
fbS20466	BY915372
fbS20474	BY915373
fbS20490	BY915374
fbS20491	BY915375
fbS20493	BY915376
fbS20494	BY915377
fbS20495	BY915378
fbS20496	BY915379
fbS20500	BY915380
fbS20504	BY915381
fbS20505	BY915382
fbS20507	BY915383
fbS20510	BY915384
fbS20517	BY915385
fbS20518	BY915386
fbS20520	BY915387
fbS20524	BY915388
fbS20525	BY915389
fbS20526	BY915390
fbS20535	BY915391
fbS20536	BY915392
fbS20537	BY915393
fbS20540	BY915394
fbS20541	BY915395
fbS20544	BY915396
fbS20547	BY915397
fbS20557	BY915398
fbS20574	BY915399
fbS20578	BY915400
fbS20579	BY915401
fbS20585	BY915402
fbS20586	BY915403
fbS20587	BY915404
fbS20591	BY915405
fbS20596	BY915406
fbS20597	BY915407

fbS20600	BY915408
fbS20603	BY915409
fbS20605	BY915410
fbS20606	BY915411
fbS20607	BY915412
fbS20608	BY915413
fbS20609	BY915414
fbS20610	BY915415
fbS20617	BY915416
fbS20619	BY915417
fbS20621	BY915418
fbS20622	BY915419
fbS20625	BY915420
fbS20626	BY915421
fbS20627	BY915422
fbS20628	BY915423
fbS20629	BY915424
fbS20631	BY915425
fbS20632	BY915426
fbS20634	BY915427
fbS20637	BY915428
fbS20641	BY915429
fbS20643	BY915430
fbS20644	BY915431
fbS20646	BY915432
fbS20648	BY915433
fbS20649	BY915434
fbS20650	BY915435
fbS20653	BY915436
fbS20656	BY915437
fbS20657	BY915438
fbS20661	BY915439
fbS20662	BY915440
fbS20667	BY915441
fbS20668	BY915442
fbS20670	BY915443
fbS20675	BY915444
fbS20678	BY915445
fbS20681	BY915446
fbS20682	BY915447
fbS20684	BY915448
fbS20685	BY915449
fbS20686	BY915450
fbS20687	BY915451
fbS20688	BY915452
fbS20691	BY915453
fbS20693	BY915454
fbS20694	BY915455
fbS20695	BY915456
fbS20697	BY915457
fbS20698	BY915458
fbS20699	BY915459
fbS20703	BY915460
fbS20704	BY915461

fbS20705	BY915462
fbS20706	BY915463
fbS20712	BY915464
fbS20713	BY915465
fbS20714	BY915466
fbS20715	BY915467
fbS20716	BY915468
fbS20717	BY915469
fbS20718	BY915470
fbS20719	BY915471
fbS20721	BY915472
fbS20722	BY915473
fbS20724	BY915474
fbS20725	BY915475
fbS20726	BY915476
fbS20727	BY915477
fbS20728	BY915478
fbS20729	BY915479
fbS20730	BY915480
fbS20732	BY915481
fbS20733	BY915482
fbS20734	BY915483
fbS20738	BY915484
fbS20740	BY915485
fbS20741	BY915486
fbS20742	BY915487
fbS20743	BY915488
fbS20745	BY915489
fbS20746	BY915490
fbS20747	BY915491
fbS20750	BY915492
fbS20751	BY915493
fbS20752	BY915494
fbS20754	BY915495
fbS20755	BY915496
fbS20756	BY915497
fbS20757	BY915498
fbS20758	BY915499
fbS20762	BY915500
fbS20763	BY915501
fbS20764	BY915502
fbS20765	BY915503
fbS20766	BY915504
fbS20767	BY915505
fbS20768	BY915506
fbS20773	BP124792
fbS20775	BP124793
fbS20777X	BP124794
fbS20779	BP124795
fbS20781	BP124796
fbS20783	BP124797
fbS20785	BP124798
fbS20788	BP124799
fbS20789	BP124800

	fbS20790	BP124801
	fbS20792	BP124802
	fbS20793	BP124803
	fbS20794	BP124804
	fbS20796	BP124805
	fbS20799	BP124806
	fbS20806	BP124808
	fbS20809	BP124809
	fbS20814	BP124810
	fbS20815	BP124811
	fbS20818	BP124812
	fbS20819	BP124813
	fbS20821	BP124814
	fbS20823	BP124815
	fbS20826	BP124816
	fbS20831	BP124817
	fbS20834	BP124818
	fbS20835	BP124819
	fbS20836	BP124820
	fbS20837	BP124821
	fbS20838	BP124822
	fbS20843	BP124824
	fbS20845	BP124825
	fbS20852	BP124826
	fbS20856	BP124827
	fbS20857	BP124828
	fbS20860	BP124829
	fbS20878	BP124831
	fbS20879	BP124832
	fbS20880	BP124833
	fbS20881	BP124834
	fbS20886	BP124835
	fbS20898	BP124836
	fbS20899	BP124837
	fbS20902	BP124838
	fbS20903	BP124839
	fbS20908	BP124840
	fbS20909	BP124841
	fbS20914	BP124842
	fbS20929	BP124843
	fbS20933	BP124844
heS0	heS00003	AV401650
	heS00004	AV401651
	heS00005	AV401652
	heS00006	AV401653
	heS00007	AV401654
	heS00008	AV401655
	heS00009	AV401656
	heS00011	AV401658
	heS00012X	AV401659
	heS00013	AV401660
	heS00014	AV401661
	heS00015	AV401662
	heS00016	AV401663

heS00018	AV401664
heS00019	AV401665
heS00020	AV401666
heS00023	AV401667
heS00024	AV401668
heS00025	AV401669
heS00026	AV401670
heS00028	AV401672
heS00029	AV401673
heS00030	AV401674
heS00031	AV401675
heS00032	AV401676
heS00033	AV401677
heS00034	AV401678
heS00035	AV401679
heS00036	AV401680
heS00037	AV401681
heS00040	AV401684
heS00041	AV401685
heS00042	AV401686
heS00043	AV401687
heS00045	AV401689
heS00046	AV401690
heS00047	AV401691
heS00049	AV401693
heS00050X	AV401694
heS00052	AV401696
heS00055	AV401698
heS00056	AV401699
heS00057	AV401700
heS00058	AV401701
heS00060	AV401702
heS00061	AV401703
heS00062	AV401704
heS00063	AV401705
heS00064	AV401706
heS00065	AV401707
heS00068	AV401709
heS00070	AV401711
heS00071	AV401712
heS00072	AV401713
heS00073	AV401714
heS00074	AV401715
heS00076	AV401716
heS00077	AV401717
heS00078	AV401718
heS00079	AV401719
heS00081	AV401721
heS00083	AV401723
heS00084X	AV401724
heS00085	AV401725
heS00086	AV401726
heS00087	AV401727
heS00088	AV401728

heS00089	AV401729
heS00091	AV401731
heS00092	AV401732
heS00093	AV401733
heS00095	AV401735
heS00096	AV401736
heS00097	AV401737
heS00098X	AV401738
heS00099	AV401739
heS00100	AV401740
heS00103	AV401742
heS00104	AV401743
heS00105	AV401744
heS00106	AV401745
heS00107	BY915507
heS00108	AV401746
heS00110	AV401747
heS00111	AV401748
heS00112	AV401749
heS00113	AV401750
heS00114X	AV401751
heS00115	AV401752
heS00117	AV401753
heS00120	AV401754
heS00121X	AV401755
heS00122	AV401756
heS00123	AV401757
heS00124X	AV401758
heS00125	AV401759
heS00126	AV401760
heS00127	AV401761
heS00128	AV401762
heS00130	AV401763
heS00131X	AV401764
heS00132	AV401765
heS00133	AV401766
heS00134	AV401767
heS00138	AV401768
heS00139	AV401769
heS00140	AV401770
heS00141	AV401771
heS00142	AV401772
heS00143	AV401773
heS00144	AV401774
heS00147	AV401776
heS00148	AV401777
heS00149	AV401778
heS00150	AV401779
heS00151	AV401780
heS00153	AV401781
heS00154	AV401782
heS00155	AV401783
heS00158	AV401784
heS00159	AV401785

heS00160X	AV401786
heS00162	AV401787
heS00163	AV401788
heS00164	AV401789
heS00165	AV401790
heS00167	AV401792
heS00168	AV401793
heS00169	AV401794
heS00170	AV401795
heS00172	AV401797
heS00173	AV401798
heS00174	AV401799
heS00175	AV401800
heS00176	AV401801
heS00177	AV401802
heS00178	AV401803
heS00179	AV401804
heS00180	AV401805
heS00182	AV401806
heS00183	AV401807
heS00184	AV401808
heS00188	AV401809
heS00191	AV401813
heS00192	AV401814
heS00193	AV401815
heS00194	AV401816
heS00195	AV401817
heS00196	AV401818
heS00202	AV401820
heS00203	AV401821
heS00204	AV401822
heS00205	AV401823
heS00206	AV401824
heS00207	AV401825
heS00209	AV401826
heS00210X	AV401827
heS00211	AV401828
heS00217X	AV401832
heS00218	AV401833
heS00219	AV401834
heS00221	AV401835
heS00222X	AV401836
heS00223	AV401837
heS00224	AV401838
heS00225X	AV401839
heS00230	AV401842
heS00231	AV401843
heS00232	AV401844
heS00236	AV401847
heS00241	AV401850
heS00243	AV401851
heS00245	AV401852
heS00247	AV401854
heS00248X	AV401855

heS00249	AV401856
heS00250	AV401857
heS00251	AV401858
heS00252	AV401859
heS00255	AV401861
heS00256	AV401862
heS00258	AV401864
heS00259	AV401865
heS00260	AV401866
heS00262	AV401867
heS00263	AV401868
heS00264	AV401869
heS00265X	AV401870
heS00267	AV401871
heS00268	AV401872
heS00269	AV401873
heS00271	AV401875
heS00272	AV401876
heS00273	AV401877
heS00274X	AV401878
heS00275	AV401879
heS00276	AV401880
heS00277	AV401881
heS00278	AV401882
heS00279	AV401883
heS00280	AV401884
heS00281	AV401885
heS00282	AV401886
heS00283	AV401887
heS00284	AV401888
heS00285	AV401889
heS00286	AV401890
heS00287	AV401891
heS00288X	AV401892
heS00289	AV401893
heS00290	AV401894
heS00291	AV401895
heS00292	AV401896
heS00293	AV401897
heS00294	AV401898
heS00295	AV401899
heS00296	AV401900
heS00297	AV401901
heS00298	AV401902
heS00299	AV401903
heS00303X	AV401904
heS00305	AV401905
heS00306	AV401906
heS00307	AV401907
heS00308X	AV401908
heS00310	AV401910
heS00312	AV401911
heS00314	AV401912
heS00316X	AV401914

heS00317	AV401915
heS00318	AV401916
heS00319	AV401917
heS00320	AV401918
heS00321X	AV401919
heS00323	AV401921
heS00324X	AV401922
heS00325	AV401923
heS00326	AV401924
heS00327	AV401925
heS00328	AV401926
heS00329	AV401927
heS00330	AV401928
heS00331	AV401929
heS00334X	AV401932
heS00336	AV401934
heS00363X	AV401935
heS00367	AV401937
heS00368	AV401938
heS00369	AV401939
heS00370X	AV401940
heS00371	AV401941
heS00372	AV401942
heS00373	AV401943
heS00374X	AV401944
heS00375	AV401945
heS00377	AV401946
heS00378	AV401947
heS00381	AV401948
heS00382	AV401949
heS00384	AV401950
heS00386	AV401951
heS00389	AV401954
heS00390	AV401955
heS00391	AV401956
heS00392	AV401957
heS00393	AV401958
heS00394	AV401959
heS00395	AV401960
heS00397	AV401961
heS00399	AV401962
heS00400	AV401963
heS00403	AV401964
heS00404	AV401965
heS00406	AV401966
heS00407	AV401967
heS00409	AV401968
heS00410	AV401969
heS00411	AV401970
heS00412X	AV401971
heS00413	AV401972
heS00414	AV401973
heS00415	AV401974
heS00416	AV401975

heS00417	AV401976
heS00419	AV401978
heS00422X	AV401979
heS00423	AV401980
heS00424	AV401981
heS00425	AV401982
heS00426	AV401983
heS00427	AV401984
heS00429	AV401985
heS00430	AV401986
heS00431	AV401987
heS00432	AV401988
heS00433X	AV401989
heS00435X	AV401991
heS00436	AV401992
heS00437	AV401993
heS00438	AV401994
heS00439	AV401995
heS00440	BY915508
heS00441	AV401996
heS00442	AV401997
heS00443	AV401998
heS00444	AV401999
heS00445	AV402000
heS00446X	AV402001
heS00448	AV402002
heS00449	AV402003
heS00450	AV402004
heS00451	AV402005
heS00453	AV402006
heS00455	AV402008
heS00456	AV402009
heS00457	AV402010
heS00458	AV402011
heS00459	AV402012
heS00460	AV402013
heS00461	AV402014
heS00462	AV402015
heS00463	AV402016
heS00464X	AV402017
heS00465	AV402018
heS00466	AV402019
heS00467	AV402020
heS00468	AV402021
heS00469	AV402022
heS00470	AV402023
heS00471	AV402024
heS00472	AV402025
heS00473	AV402026
heS00474	AV402027
heS00476X	AV402028
heS00477	AV402029
heS00478	AV402030
heS00480	AV402032

heS00482	AV402033
heS00483	AV402034
heS00487	AV402036
heS00488	AV402037
heS00489	AV402038
heS00490	AV402039
heS00491	AV402040
heS00493X	AV402041
heS00496	AV402042
heS00497X	AV402043
heS00498	AV402044
heS00501	AV402047
heS00502	AV402048
heS00503	AV402049
heS00504	AV402050
heS00505	AV402051
heS00507	AV402052
heS00509	AV402054
heS00511	AV402056
heS00514	AV402058
heS00515	AV402059
heS00516	AV402060
heS00517	AV402061
heS00518	AV402062
heS00519	AV402063
heS00520	AV402064
heS00521	AV402065
heS00522	AV402066
heS00523	AV402067
heS00524	AV402068
heS00525	AV402069
heS00526	AV402070
heS00527	AV402071
heS00530X	AV402072
heS00531	AV402073
heS00532	AV402074
heS00535	AV402075
heS00536	AV402076
heS00538	AV402078
heS00539	AV402079
heS00540	AV402080
heS00541	AV402081
heS00542	AV402082
heS00543	AV402083
heS00547	AV402085
heS00549	AV402086
heS00551	AV402087
heS00552	AV402088
heS00553	AV402089
heS00554X	AV402090
heS00555	AV402091
heS00556	AV402092
heS00557	AV402093
heS00558	AV402094

heS00560X	AV402096
heS00561	AV402097
heS00562X	AV402098
heS00563	AV402099
heS00565	AV402100
heS00566	AV402101
heS00567	AV402102
heS00568X	AV402103
heS00569	AV402104
heS00570	AV402105
heS00572X	AV402106
heS00573	AV402107
heS00575	AV402108
heS00576	AV402109
heS00577X	AV402110
heS00578X	AV402111
heS00581	AV402113
heS00582	AV402114
heS00584X	AV402116
heS00589X	AV402119
heS00591	AV402121
heS00593	AV402122
heS00594X	AV402123
heS00596	AV402125
heS00601	AV402128
heS00602	AV402129
heS00604	AV402131
heS00606	AV402132
heS00608	AV402134
heS00609	AV402135
heS00611	AV402137
heS00618	AV402140
heS00622	AV402143
heS00623X	AV402144
heS00625	AV402146
heS00627	AV402147
heS00633	AV402151
heS00634	AV402152
heS00635X	AV402153
heS00637X	AV402155
heS00639	AV402156
heS00640	AV402157
heS00641X	AV402158
heS00642	AV402159
heS00643	AV402160
heS00644	AV402161
heS00645	AV402162
heS00646	AV402163
heS00647X	AV402164
heS00648	AV402165
heS00649	AV402166
heS00651	AV402168
heS00652	AV402169
heS00653	AV402170

heS00654	AV402171
heS00655	AV402172
heS00658	AV402173
heS00659	AV402174
heS00660	AV402175
heS00661	AV402176
heS00662	AV402177
heS00663	AV402178
heS00664	AV402179
heS00665	AV402180
heS00666	AV402181
heS00667	AV402182
heS00668X	AV402183
heS00671X	AV402185
heS00672	AV402186
heS00673	AV402187
heS00674X	AV402188
heS00676	AV402190
heS00678	AV402191
heS00679	AV402192
heS00681	AV402194
heS00682	AV402195
heS00683	AV402196
heS00685	AV402198
heS00689	AV402200
heS00690X	AV402201
heS00691	AV402202
heS00692	AV402203
heS00693	AV402204
heS00694X	AV402205
heS00695	AV402206
heS00696	AV402207
heS00697	AV402208
heS00699	AV402209
heS00700X	AV402210
heS00701X	AV402211
heS00702	AV402212
heS00703	AV402213
heS00705	AV402214
heS00708	AV402215
heS00709X	AV402216
heS00710	AV402217
heS00711X	AV402218
heS00712	AV402219
heS00713	AV402220
heS00716	AV402221
heS00717X	AV402222
heS00720	AV402225
heS00770	AV402226
heS00771	AV402227
heS00772X	AV402228
heS00774	AV402229
heS00776	AV402230
heS00777	AV402231

heS00778	AV402232
heS00779	AV402233
heS00780	AV402234
heS00781	AV402235
heS00782	AV402236
heS00783	AV402237
heS00784X	AV402238
heS00786	AV402240
heS00787	AV402241
heS00788	AV402242
heS00789	AV402243
heS00790	AV402244
heS00792	AV402247
heS00793	AV402248
heS00794	AV402249
heS00795	AV402250
heS00796	AV402251
heS00797	AV402252
heS00798	AV402253
heS00799	AV402254
heS00800	AV402255
heS00801	AV402256
heS00802	AV402257
heS00803	AV402258
heS00804	AV402259
heS00805	AV402260
heS00806	BY915509
heS00807	AV402261
heS00808	AV402262
heS00809	AV402263
heS00810	AV402264
heS00811	AV402265
heS00812	AV402266
heS00813	AV402267
heS00814	AV402268
heS00815	AV402269
heS00816	AV402270
heS00817	AV402271
heS00818	AV402272
heS00819	AV402273
heS00820	AV402274
heS00821	AV402275
heS00822	AV402276
heS00823X	AV402277
heS00824	AV402278
heS00825X	AV402279
heS00826X	AV402280
heS00828	AV402281
heS00829	AV402282
heS00830	AV402283
heS00831X	AV402284
heS00832X	AV402285
heS00833	AV402286
heS00834X	AV402287

heS00835X	AV402288
heS00836	AV402289
heS00837X	AV402290
heS00838	AV402291
heS00841	AV402293
heS00842	AV402294
heS00845	AV402295
heS00846	AV402296
heS00848	AV402298
heS00849	AV402299
heS00850	AV402300
heS00851	AV402301
heS00852	AV402302
heS00854	AV402304
heS00855	AV402305
heS00857X	AV402307
heS00858	AV402308
heS00859	AV402309
heS00860	AV402310
heS00863	AV402312
heS00864	AV402313
heS00865X	AV402314
heS00867	AV402315
heS00868	AV402316
heS00869	AV402317
heS00870	AV402318
heS00871	AV402319
heS00872	AV402320
heS00873	AV402321
heS00874X	AV402322
heS00875	AV402323
heS00876	AV402324
heS00877	AV402325
heS00878	AV402326
heS00879	AV402327
heS00880	AV402328
heS00881	AV402329
heS00882	AV402330
heS00883	AV402331
heS00885	AV402332
heS00886	AV402333
heS00887	AV402334
heS00888	AV402335
heS00889	AV402336
heS00890	AV402337
heS00891	AV402338
heS00893	AV402340
heS00894	AV402341
heS00895	AV402342
heS00896	AV402343
heS00898	AV402344
heS00899	AV402345
heS00900	AV402346
heS00901X	AV402347

heS00902	AV402348
heS00904	AV402349
heS00905	AV402350
heS00906	AV402351
heS00907	AV402352
heS00908	AV402353
heS00909	AV402354
heS00910	AV402355
heS00911	AV402356
heS00913	AV402358
heS00915	AV402360
heS00916	AV402361
heS00917	AV402362
heS00918	AV402363
heS00919	AV402364
heS00920X	AV402365
heS00923	AV402366
heS00924	AV402367
heS00926X	AV402368
heS00927	AV402369
heS00929X	AV402370
heS00931	AV402371
heS00935	AV402372
heS00936	AV402373
heS00937	AV402374
heS00939	AV402375
heS00940	AV402376
heS00942	AV402377
heS00945	AV402380
heS00946	AV402381
heS00948	AV402383
heS00949	AV402384
heS00953	AV402387
heS00955	AV402389
heS00956	AV402390
heS00957	AV402391
heS00959	AV402393
heS00960	AV402394
heS00961	AV402395
heS00962	AV402396
heS00964	AV402397
heS00965	AV402398
heS00966	AV402399
heS00967	BY915510
heS00968X	AV402400
heS00969	AV402401
heS00971	AV402403
heS00972	AV402404
heS00973	AV402405
heS00974	AV402406
heS00976	AV402408
heS00977	AV402409
heS00978	AV402410
heS00979	AV402411

heS00980	AV402412
heS00982	AV402413
heS00983	AV402414
heS00984	AV402415
heS00986	AV402416
heS00987	AV402417
heS00990	AV402419
heS00991	AV402420
heS00992	AV402421
heS00995	AV402423
heS00998	AV402425
heS01000	AV402426
heS01001	AV402427
heS01003	AV402428
heS01004	AV402429
heS01005	AV402430
heS01006X	AV402431
heS01007	AV402432
heS01008	AV402433
heS01010X	AV402434
heS01011	AV402435
heS01012	AV402436
heS01013	AV402437
heS01014	AV402438
heS01015	AV402439
heS01016	AV402440
heS01017	AV402441
heS01018	AV402442
heS01019	AV402443
heS01020X	AV402444
heS01022	AV402445
heS01023	AV402446
heS01024	AV402447
heS01026X	AV402449
heS01027	AV402450
heS01028	AV402451
heS01029	AV402452
heS01030	AV402453
heS01031	AV402454
heS01032	AV402455
heS01034	AV402456
heS01035	AV402457
heS01036	AV402458
heS01037	AV402459
heS01039	AV402461
heS01040	AV402462
heS01041	AV402463
heS01042	AV402464
heS01043	AV402465
heS01044	AV402466
heS01047	AV402467
heS01050	AV402468
heS01051	AV402469
heS01052	AV402470

	heS01053	AV402471
	heS01055	AV402473
	heS01056	AV402474
heS3	heS30001	AV402475
	heS30003	AV402476
	heS30005	AV402477
	heS30006	AV402478
	heS30010	AV402481
	heS30011	AV402482
	heS30012	AV402483
	heS30014	AV402485
	heS30015	AV402486
	heS30017	AV402487
	heS30024	AV402490
	heS30027	AV402491
	heS30030	AV402493
	heS30033	AV402494
	heS30035	AV402495
	heS30038	AV402496
	heS30039	AV402497
	heS30043	AV402499
	heS30045	AV402500
	heS30046	AV402501
	heS30047	AV402502
	heS30048	AV402503
	heS30049	AV402504
	heS30050	AV402505
	heS30052	AV402506
	heS30053	AV402507
	heS30057	AV402508
	heS30058	AV402509
	heS30060	AV402510
	heS30061	AV402511
	heS30066	AV402515
	heS30069	AV402517
	heS30071	AV402518
	heS30073	AV402520
	heS30074	AV402521
	heS30075	AV402522
	heS30076	AV402523
	heS30079	AV402525
	heS30081	AV402527
	heS30082	AV402528
	heS30083	AV402529
	heS30084	AV402530
	heS30085	AV402531
	heS30086	AV402532
	heS30087	AV402533
	heS30090	AV402535
	heS30092	AV402537
	heS30093	AV402538
	heS30094	AV402539
	heS30096	AV402541
	heS30097	AV402542

heS30098	AV402543
heS30099	AV402544
heS30103	AV402545
heS30105	AV402546
heS30106	AV402547
heS30107	AV402548
heS30110	AV402549
heS30111	AV402550
heS30113	AV402552
heS30114	AV402553
heS30116	AV402554
heS30118	AV402555
heS30119	AV402556
heS30121	AV402557
heS30125	AV402558
heS30126	AV402559
heS30127	AV402560
heS30128	AV402561
heS30129	AV402562
heS30130	AV402563
heS30131	AV402564
heS30134	AV402565
heS30136	AV402566
heS30138	AV402567
heS30139	AV402568
heS30141	AV402569
heS30142	AV402570
heS30144	AV402572
heS30146	AV402573
heS30147	AV402574
heS30150	AV402575
heS30154	AV402576
heS30156	AV402577
heS30160	AV402579
heS30161	AV402580
heS30162	AV402581
heS30164	AV402582
heS30165	AV402583
heS30166	AV402584
heS30167	AV402585
heS30168	AV402586
heS30170	AV402587
heS30173	AV402589
heS30176	AV402590
heS30178	AV402591
heS30179	AV402592
heS30181	AV402594
heS30182	BY915511
heS30183	AV402595
heS30184	AV402596
heS30186	AV402597
heS30187	AV402598
heS30189	AV402599
heS30190	AV402600

heS30191	AV402601
heS30192	AV402602
heS30193	AV402603
heS30195	AV402604
heS30196	AV402605
heS30198	AV402607
heS30199	AV402608
heS30200	AV402609
heS30202	AV402610
heS30205	AV402612
heS30208	AV402613
heS30209	AV402614
heS30210	AV402615
heS30214	AV402616
heS30215	AV402617
heS30218	AV402618
heS30219	AV402619
heS30220	AV402620
heS30221	AV402621
heS30225	AV402623
heS30228	AV402624
heS30229	AV402625
heS30230	AV402626
heS30233	AV402627
heS30234	AV402628
heS30235	AV402629
heS30236	AV402630
heS30237	AV402631
heS30238	AV402632
heS30240	AV402633
heS30242	AV402634
heS30243	AV402635
heS30245	AV402636
heS30248	AV402637
heS30250	AV402638
heS30251	AV402639
heS30255	AV402640
heS30256	AV402641
heS30260	AV402642
heS30261	AV402643
heS30262	AV402644
heS30263	AV402645
heS30264	AV402646
heS30265	AV402647
heS30267	AV402648
heS30268	AV402649
heS30272	AV402650
heS30274	AV402651
heS30277	AV402652
heS30280	AV402653
heS30281	AV402654
heS30282	AV402655
heS30286	AV402656
heS30287	AV402657

heS30290	AV402658
heS30292	AV402659
heS30293	AV402660
heS30294	AV402661
heS30297	AV402662
heS30298	AV402663
heS30300	AV402664
heS30302	AV402665
heS30303	AV402666
heS30304	AV402667
heS30305	AV402668
heS30306	AV402669
heS30308	AV402670
heS30309	AV402671
heS30311	AV402672
heS30313	AV402674
heS30315	AV402675
heS30316	AV402676
heS30317	AV402677
heS30320	AV402678
heS30321	AV402679
heS30322	AV402680
heS30323	AV402681
heS30325	AV402682
heS30326	AV402683
heS30327	AV402684
heS30328	AV402685
heS30329	AV402686
heS30330	AV402687
heS30331	AV402688
heS30332	AV402689
heS30335	AV402690
heS30336	AV402691
heS30340	AV402693
heS30342	AV402694
heS30346	AV402695
heS30347	AV402696
heS30350	AV402697
heS30351	AV402698
heS30352	AV402699
heS30356	AV402700
heS30357	AV402701
heS30360	AV402702
heS30361	AV402703
heS30362	AV402704
heS30363	AV402705
heS30364	AV402706
heS30365	AV402707
heS30367	AV402708
heS30368	AV402709
heS30369	AV402710
heS30370	AV402711
heS30371	AV402712
heS30375	AV402713

heS30376	AV402714
heS30378	AV402715
heS30381	AV402716
heS30382	AV402717
heS30383	AV402718
heS30385	AV402719
heS30388	AV402720
heS30390	AV402721
heS30393	AV402722
heS30394	AV402723
heS30395	AV402724
heS30398	AV402725
heS30399	AV402726
heS30400	AV402727
heS30403	AV402728
heS30404	AV402729
heS30405	AV402730
heS30407	AV402731
heS30408	AV402732
heS30409	AV402733
heS30410	AV402734
heS30411	AV402735
heS30412	AV402736
heS30413	AV402737
heS30415	AV402738
heS30416	AV402739
heS30417	AV402740
heS30418	AV402741
heS30420	AV402742
heS30421	AV402743
heS30424	AV402744
heS30425	AV402745
heS30428	AV402746
heS30430	AV402747
heS30432	AV402748
heS30437	AV402749
heS30438	AV402750
heS30439	AV402751
heS30444	AV402753
heS30446	AV402754
heS30447	AV402755
heS30450	AV402757
heS30451	AV402758
heS30453	AV402759
heS30454	AV402760
heS30455	AV402761
heS30456	AV402762
heS30459	AV402763
heS30460	AV402764
heS30461	AV402765
heS30462	AV402766
heS30463	AV402767
heS30465	AV402768
heS30466	AV402769

heS30467	AV402770
heS30468	AV402771
heS30469	AV402772
heS30472	AV402773
heS30473	AV402774
heS30474	AV402775
heS30475	AV402776
heS30477	AV402777
heS30478	AV402778
heS30479	AV402779
heS30480	AV402780
heS30508	AV402781
heS30509	AV402782
heS30510	AV402783
heS30511	AV402784
heS30513	AV402785
heS30514	AV402786
heS30516	AV402787
heS30517	AV402788
heS30519	AV402789
heS30523	AV402790
heS30524	AV402791
heS30526	AV402792
heS30527	AV402793
heS30529	AV402794
heS30530	AV402795
heS30531	AV402796
heS30532	AV402797
heS30533	AV402798
heS30534	AV402799
heS30537	AV402800
heS30538	AV402801
heS30539	AV402802
heS30544	AV402803
heS30545	AV402804
heS30547	AV402805
heS30549	AV402806
heS30550	AV402807
heS30552	AV402808
heS30553	AV402809
heS30554	AV402810
heS30556	AV402811
heS30557	AV402812
heS30558	AV402813
heS30559	AV402814
heS30560	AV402815
heS30561	AV402816
heS30562	AV402817
heS30564	AV402818
heS30570	AV402820
heS30572	AV402821
heS30574	AV402822
heS30576	AV402823
heS30580	AV402824

heS30581	AV402825
heS30585	AV402828
heS30587	AV402829
heS30588	AV402830
heS30590	AV402831
heS30591	AV402832
heS30594	AV402834
heS30595	AV402835
heS30596	AV402836
heS30598	AV402837
heS30599	AV402838
heS30600	AV402839
heS30601	AV402840
heS30602	AV402841
heS30603	AV402842
heS30606	AV402844
heS30607	AV402845
heS30609	AV402847
heS30610	AV402848
heS30611	AV402849
heS30612	AV402850
heS30614	AV402851
heS30617	AV402852
heS30619	AV402853
heS30620	AV402854
heS30624	AV402856
heS30627	AV402857
heS30629	AV402858
heS30630	AV402859
heS30631	AV402860
heS30632	AV402861
heS30633	AV402862
heS30634	AV402863
heS30635	AV402864
heS30636	AV402865
heS30637	AV402866
heS30640	AV402867
heS30642	AV402868
heS30644	AV402869
heS30645	AV402870
heS30647	AV402871
heS30649	AV402872
heS30653	AV402873
heS30654	AV402874
heS30655	AV402875
heS30656	AV402876
heS30657	AV402877
heS30659	AV402878
heS30665	AV402881
heS30666	AV402882
heS30670	AV402883
heS30671	AV402884
heS30675	AV402885
heS30677	AV402887

heS30679	AV402888
heS30680	AV402889
heS30681	AV402890
heS30683	AV402891
heS30684	AV402892
heS30685	AV402893
heS30686	AV402894
heS30690	AV402897
heS30691	AV402898
heS30694	AV402900
heS30696	AV402902
heS30698	AV402903
heS30700	AV402904
heS30701	AV402905
heS30702	AV402906
heS30703	AV402907
heS30706	AV402908
heS30710	AV402910
heS30714	AV402912
heS30716	AV402914
heS30718	AV402915
heS30720	AV402916
heS30722	AV402917
heS30723	AV402918
heS30724	AV402919
heS30725	AV402920
heS30727	AV402921
heS30728	AV402922
heS30731	AV402923
heS30733	AV402924
heS30734	AV402925
heS30735	AV402926
heS30736	AV402927
heS30739	AV402928
heS30743	AV402929
heS30744	AV402930
heS30745	AV402931
heS30747	AV402932
heS30750	AV402934
heS30752	AV402936
heS30753	AV402937
heS30754	AV402938
heS30756	AV402939
heS30757	AV402940
heS30759	AV402941
heS30760	AV402942
heS30761	AV402943
heS30769	AV402945
heS30772	AV402947
heS30774	AV402948
heS30778	AV402949
heS30779	AV402950
heS30782	AV402951
heS30783	AV402952

heS30786	AV402953
heS30788	AV402954
heS30791	AV402955
heS30792	AV402956
heS30793	AV402957
heS30795	AV402958
heS30796	AV402959
heS30797	AV402960
heS30798	AV402961
heS30799	AV402962
heS30800	AV402963
heS30805	AV402964
heS30808	AV402966
heS30809	AV402967
heS30812	AV402968
heS30814	AV402970
heS30816	AV402971
heS30817	AV402972
heS30818	AV402973
heS30819	AV402974
heS30820	AV402975
heS30821	AV402976
heS30822	AV402977
heS30824	AV402978
heS30825	AV402979
heS30826	AV402980
heS30827	AV402981
heS30828	AV402982
heS30831	AV402984
heS30832	AV402985
heS30838	AV402988
heS30840	AV402989
heS30845	AV402990
heS30846	AV402991
heS30847	AV402992
heS30849	AV402993
heS30852	AV402994
heS30855	AV402995
heS30856	AV402996
heS30857	AV402997
heS30859	AV402999
heS30860	AV403000
heS30862	AV403001
heS30863	AV403002
heS30865	AV403003
heS30868	AV403004
heS30870	AV403005
heS30872	AV403006
heS30874	AV403007
heS30875	AV403008
heS30876	AV403009
heS30877	AV403010
heS30880	AV403011
heS30881	AV403012

heS30884	AV403013
heS30889	AV403014
heS30897	AV403015
heS30901	AV403016
heS30902	AV403017
heS30904	AV403018
heS30905	AV403019
heS30945	AV403020
heS30946	AV403021
heS30947	AV403022
heS30948	AV403023
heS30949	AV403024
heS30954	AV403027
heS30955	AV403028
heS30956	AV403029
heS30959	AV403031
heS30960	AV403032
heS30961	AV403033
heS30962	AV403034
heS30963	AV403035
heS30965	AV403037
heS30969	AV403038
heS30970	AV403039
heS30972	AV403040
heS30973	AV403041
heS30974	AV403042
heS30975	AV403043
heS30976	AV403044
heS30977	AV403045
heS30979	AV403046
heS30980	AV403047
heS30983	AV403049
heS30988	AV403051
heS30989	AV403052
heS30991	AV403053
heS30992	AV403054
heS30994	AV403055
heS30996	AV403057
heS30997	AV403058
heS30998	AV403059
heS30999	AV403060
heS31000	AV403061
heS31002	AV403062
heS31003	AV403063
heS31004	AV403064
heS31005	AV403065
heS31008	AV403067
heS31009	AV403068
heS31010	AV403069
heS31011	AV403070
heS31012	AV403071
heS31013	AV403072
heS31015	AV403074
heS31017	AV403075

	heS31019	AV403076
	heS31021	AV403077
	heS31023	AV403078
	heS31026	AV403079
	heS31031	AV403080
	heS31034	AV403081
	heS31036	AV403082
	heS31037	AV403083
	heS31038	AV403084
	heS31039	AV403085
	heS31041	AV403086
	heS31046	AV403087
	heS31047	AV403088
	heS31048	AV403089
	heS31050	AV403090
	heS31051	AV403091
	heS31052	AV403092
	heS31053	AV403093
	heS31054	AV403094
	heS31056	AV403095
mg	mg0001	AU001868
	mg0003	AU001870
	mg0004	AU001871
	mg0005	AU001872
	mg0006	AU001873
	mg0008	BY916287
	mg0009	BY916288
	mg0010	BY916289
	mg0011	BY916290
	mg0012	AU001874
	mg0013	BY916291
	mg0014	AU001875
	mg0015	AU001876
	mg0016	BY916292
	mg0017	AU001877
	mg0018	AU001878
	mg0019	AU001879
	mg0021	BY916293
	mg0022	BY916294
	mg0023	AU001880
	mg0025	AU001881
	mg0026	AU001882
	mg0027	AU001883
	mg0028	AU001884
	mg0029	AU001885
	mg0030	AU001886
	mg0031	AU001887
	mg0032	AU001888
	mg0033	BY916295
	mg0035	AU001889
	mg0036	BY916296
	mg0038	AU001891
	mg0039	AU001892
	mg0040	AU001893

mg0041	AU001894
mg0042	AU001895
mg0043	BY916297
mg0044	AU001896
mg0046	AU001897
mg0048	AU001898
mg0049	AU001899
mg0051	AU001901
mg0052	BY916298
mg0054	AU001903
mg0055	BY916299
mg0056	BY916300
mg0057	AU001904
mg0059	AU001905
mg0060	AU001906
mg0061	AU001907
mg0062	AU001908
mg0063	AU001909
mg0065	AU001911
mg0066	AU001912
mg0067	AU001913
mg0069	AU001914
mg0070	BY916301
mg0071	AU001915
mg0072	BY916302
mg0073	AU001916
mg0074	AU001917
mg0075	AU001918
mg0078	AU001920
mg0079	AU001921
mg0081	AU001923
mg0083	AU001925
mg0084	BY916303
mg0085	BY916304
mg0086	AU001926
mg0087	AU001927
mg0089	AU001929
mg0090	AU001930
mg0092	BY916305
mg0093	AU001931
mg0094	BY916306
mg0095	AU001932
mg0096	BY916307
mg0097	BY916308
mg0098	AU001933
mg0099	AU001934
mg0100	AU001935
mg0101	AU001936
mg0102	BY916309
mg0104	AU001938
mg0105	AU001939
mg0106	BY916310
mg0108	BY916311
mg0109	BY916312

mg0110	AU001941
mg0111	AU001942
mg0112	BY916313
mg0113	AU001943
mg0114	AU001944
mg0116	BY916314
mg0117	AU001946
mg0119	AU001947
mg0120	AU001948
mg0121	BY916315
mg0123	AU001949
mg0126	AU001952
mg0127	AU001953
mg0128	AU001954
mg0129	AU001955
mg0130	AU001956
mg0131	BY916316
mg0132	BY916317
mg0133	BY916318
mg0135	AU001958
mg0136	AU001959
mg0138	AU001960
mg0139	AU001961
mg0140	BY916319
mg0141	AU001962
mg0142	BY916320
mg0143	BY916321
mg0144	BY916322
mg0145	BY916323
mg0146	AU001963
mg0147	AU001964
mg0148	BY916324
mg0150	AU001965
mg0151	AU001966
mg0152	BY916325
mg0154	AU001967
mg0155	BY916326
mg0156	AU001968
mg0157	BY916327
mg0159	AU001969
mg0160	AU001970
mg0161	AU001971
mg0163	BY916328
mg0164	BY916329
mg0166	AU001972
mg0167	BY916330
mg0168	BY916331
mg0169	BY916332
mg0173	BY916333
mg0174	AU001974
mg0176	AU001975
mg0177	AU001976
mg0180	AU001978
mg0181	BY916334

mg0182	BY916335
mg0183	AU001979
mg0184	AU001980
mg0186	AU001981
mg0187	BY916336
mg0188	BY916337
mg0189	BY916338
mg0191	BY916339
mg0192	AU001982
mg0194	BY916340
mg0195	BY916341
mg0196	BY916342
mg0198	BY916343
mg0199	BY916344
mg0201	BY916345
mg0202	BY916346
mg0203	BY916347
mg0204	BY916348
mg0206	BY916349
mg0207	BY916350
mg0209	BY916351
mg0210	BY916352
mg0211	BY916353
mg0212	BY916354
mg0213	BY916355
mg0215	BY916356
mg0216	BY916357
mg0219	BY916358
mg0221	BY916359
mg0222	BY916360
mg0223	BY916361
mg0224	BY916362
mg0225	BY916363
mg0226	BY916364
mg0227	BY916365
mg0231	BY916366
mg0232	BY916367
mg0234	BY916368
mg0235	BY916369
mg0236	BY916370
mg0237	BY916371
mg0238	BY916372
mg0240	BY916373
mg0242	BY916374
mg0244	BY916375
mg0245	BY916376
mg0246	BY916377
mg0247	BY916378
mg0248	BY916379
mg0249	BY916380
mg0250	BY916381
mg0251	BY916382
mg0252	BY916383
mg0254	BY916384

mg0255	BY916385
mg0256	BY916386
mg0257	BY916387
mg0259	BY916388
mg0262	BY916389
mg0263	BY916390
mg0264	BY916391
mg0266	BY916392
mg0267	BY916393
mg0268	BY916394
mg0269	BY916395
mg0270	BY916396
mg0271	BY916397
mg0272	BY916398
mg0273	BY916399
mg0274	BY916400
mg0275	BY916401
mg0276	BY916402
mg0278	BY916403
mg0279	BY916404
mg0280	BY916405
mg0281	BY916406
mg0282	BY916407
mg0283	BY916408
mg0284	BY916409
mg0289	AU001983
mg0290	AU001984
mg0291	AU001985
mg0292	AU001986
mg0293	AU001987
mg0294	AU001988
mg0295	AU001989
mg0296	AU001990
mg0297	AU001991
mg0298	AU001992
mg0299	AU001993
mg0304	AU001996
mg0305	AU001997
mg0306	AU001998
mg0307	AU001999
mg0308	AU002000
mg0309	AU002001
mg0311	AU002003
mg0312	AU002004
mg0313	AU002005
mg0314	AU002006
mg0315	AU002007
mg0316	AU002008
mg0317	AU002009
mg0318	AU002010
mg0320	AU002011
mg0321	BY916410
mg0322	AU002012
mg0323	AU002013

mg0324	AU002014
mg0325	AU002015
mg0327	AU002017
mg0328	AU002018
mg0331	BY916411
mg0332	BY916412
mg0333	AU002020
mg0334	AU002021
mg0335	AU002022
mg0336	BY916413
mg0337	AU002023
mg0338	BY916414
mg0339	AU002024
mg0340	AU002025
mg0341	AU002026
mg0343	AU002027
mg0344	AU002028
mg0346	AU002030
mg0347	BY916415
mg0348	BY916416
mg0349	AU002031
mg0350	AU002032
mg0351	AU002033
mg0352	AU002034
mg0353	AU002035
mg0354	AU002036
mg0355	BY916417
mg0358	BY916418
mg0359	AU002038
mg0363	AU002042
mg0364	BY916419
mg0365	AU002043
mg0366	AU002044
mg0368	AU002046
mg0372	AU002049
mg0373	AU002050
mg0374	AU002051
mg0375	AU002052
mg0376	AU002053
mg0378	AU002055
mg0379	AU002056
mg0380	AU002057
mg0383	AU002058
mg0386	BY916420
mg0388	BY916421
mg0390	BY916422
mg0391	AU002062
mg0392	AU002063
mg0393	AU002064
mg0394	AU002065
mg0395	AU002066
mg0397	AU002067
mg0398	AU002068
mg0399	AU002069

mg0400	AU002070
mg0402	AU002071
mg0405	AU002072
mg0408	AU002074
mg0409	BY916423
mg0410	AU002075
mg0411	AU002076
mg0412	AU002077
mg0413	AU002078
mg0414	AU002079
mg0416	AU002080
mg0417	BY916424
mg0418	AU002081
mg0419	BY916425
mg0420	AU002082
mg0421	AU002083
mg0422	BY916426
mg0424	AU002084
mg0425	AU002085
mg0426	AU002086
mg0427	BY916427
mg0428	AU002087
mg0429	BY916428
mg0431	AU002089
mg0432	AU002090
mg0433	AU002091
mg0434	BY916429
mg0437	BY916430
mg0438	BY916431
mg0439	BY916432
mg0441	BY916433
mg0442	BY916434
mg0444	BY916435
mg0445	BY916436
mg0446	BY916437
mg0447	BY916438
mg0448	BY916439
mg0449	BY916440
mg0450	BY916441
mg0451	BY916442
mg0452	BY916443
mg0453	BY916444
mg0454	BY916445
mg0455	BY916446
mg0458	BY916447
mg0459	BY916448
mg0460	BY916449
mg0461	BY916450
mg0464	BY916451
mg0465	BY916452
mg0466	BY916453
mg0467	BY916454
mg0468	BY916455
mg0469	BY916456

mg0470	BY916457
mg0471	BY916458
mg0472	BY916459
mg0473	BY916460
mg0474	BY916461
mg0475	BY916462
mg0476	BY916463
mg0477	BY916464
mg0479	BY916465
mg0480	BY916466
mg0481	AU002092
mg0482	AU002093
mg0483	AU002094
mg0484	AU002095
mg0486	BY916467
mg0487	AU002097
mg0488	AU002098
mg0489	AU002099
mg0491	AU002100
mg0492	AU002101
mg0493	AU002102
mg0494	AU002103
mg0495	BY916468
mg0496	AU002104
mg0497	AU002105
mg0498	AU002106
mg0499	AU002107
mg0501	AU002109
mg0502	AU002110
mg0503	AU002111
mg0505	AU002113
mg0506	AU002114
mg0507	AU002115
mg0508	AU002116
mg0509	AU002117
mg0510	AU002118
mg0511	AU002119
mg0512	AU002120
mg0514	AU002122
mg0515	AU002123
mg0516	AU002124
mg0517	AU002125
mg0518	AU002126
mg0519	AU002127
mg0520	AU002128
mg0521	AU002129
mg0523	AU002131
mg0524	AU002132
mg0525	AU002133
mg0527	AU002134
mg0528	AU002135
mg0529	AU002136
mg0530	BY916469
mg0531	AU002137

mg0535	AU002138
mg0536	BY916470
mg0537	AU002139
mg0540	AU002140
mg0541	AU002141
mg0542	BY916471
mg0545	AU002143
mg0546	AU002144
mg0547	AU002145
mg0548	BY916472
mg0549	AU002146
mg0550	AU002147
mg0551	AU002148
mg0552	AU002149
mg0554	AU002151
mg0556	AU002152
mg0558	AU002153
mg0559	AU002154
mg0561	AU002156
mg0562	AU002157
mg0563	AU002158
mg0566	BY916473
mg0567	AU002161
mg0569	AU002162
mg0570	AU002163
mg0571	AU002164
mg0574	AU002167
mg0575	AU002168
mg0576	AU002169
mg0577	AU002170
mg0578	AU002171
mg0579	AU002172
mg0580	AU002173
mg0582	AU002174
mg0584	AU002176
mg0585	AU002177
mg0586	AU002178
mg0587	AU002179
mg0588	AU002180
mg0589	AU002181
mg0590	AU002182
mg0591	AU002183
mg0592	AU002184
mg0593	AU002185
mg0595	AU002187
mg0596	AU002188
mg0597	AU002189
mg0598	AU002190
mg0599	AU002191
mg0600	AU002192
mg0601	AU002193
mg0602	BY916474
mg0605	AU002195
mg0607	AU002197

mg0612	AU002201
mg0613	AU002202
mg0614	AU002203
mg0615	AU002204
mg0616	AU002205
mg0617	AU002206
mg0618	AU002207
mg0619	AU002208
mg0620	AU002209
mg0621	AU002210
mg0622	BY916475
mg0623	AU002211
mg0624	AU002212
mg0625	AU002213
mg0627	BY916476
mg0628	BY916477
mg0629	AU002214
mg0631	AU002215
mg0632	AU002216
mg0633	AU002217
mg0634_1	BY916478
mg0634	AU002218
mg0635	BY916479
mg0637	AU002219
mg0639_1	BY916480
mg0639	AU002220
mg0642	AU002222
mg0643	AU002223
mg0645	BY916481
mg0646_1	BY916482
mg0646	AU002225
mg0648	AU002227
mg0649_1	BY916483
mg0649	AU002228
mg0650	BY916484
mg0651	AU002229
mg0652	AU002230
mg0653	AU002231
mg0654	AU002232
mg0655	AU002233
mg0656	AU002234
mg0657	AU002235
mg0658	AU002236
mg0659	AU002237
mg0660	AU002238
mg0661	AU002239
mg0662	AU002240
mg0663	AU002241
mg0664	AU002242
mg0665	AU002243
mg0666	BY916485
mg0667	AU002244
mg0668	AU002245
mg0669	AU002246

mg0670	AU002247
mg0671	AU002248
mg0672	AU002249
mg0673	AU002250
mg0674	AU002251
mg0675	AU002252
mg0677	AU002254
mg0678	AU002255
mg0679	AU002256
mg0681	AU002257
mg0683	AU002259
mg0686	AU002262
mg0687	AU002263
mg0689	AU002265
mg0690	AU002266
mg0691	AU002267
mg0692	BY916486
mg0696	BY916487
mg0698	AU002272
mg0700	AU002274
mg0701	AU002275
mg0702	AU002276
mg0703	AU002277
mg0704	BY916488
mg0705	AU002278
mg0707	AU002279
mg0708	AU002280
mg0709	BY916489
mg0710	AU002281
mg0711	AU002282
mg0712	AU002283
mg0713	AU002284
mg0714	AU002285
mg0715	AU002286
mg0716	AU002287
mg0717	AU002288
mg0718	AU002289
mg0719	AU002290
mg0720	AU002291
mg0722	AU002293
mg0723	AU002294
mg0725	AU002295
mg0726	AU002296
mg0727	AU002297
mg0728	AU002298
mg0731	AU002299
mg0732	AU002300
mg0733	BY916490
mg0734	AU002301
mg0735	AU002302
mg0736	AU002303
mg0738	AU002305
mg0739	AU002306
mg0740	AU002307

mg0741	AU002308
mg0742	AU002309
mg0743	AU002310
mg0744	AU002311
mg0746	AU002312
mg0747	AU002313
mg0748	AU002314
mg0749	AU002315
mg0750	AU002316
mg0751	AU002317
mg0752	AU002318
mg0753	AU002319
mg0754	AU002320
mg0755	AU002321
mg0756	AU002322
mg0757	BY916491
mg0758	BY916492
mg0759	BY916493
mg0760	AU002323
mg0761	AU002324
mg0762	AU002325
mg0763	AU002326
mg0765	AU002327
mg0766	AU002328
mg0767	AU002329
mg0768	AU002330
mg0769	AU002331
mg0770	BY916494
mg0771	AU002332
mg0772	AU002333
mg0773	BY916495
mg0774	BY916496
mg0776	BY916497
mg0778	AU002334
mg0779	BY916498
mg0780	AU002335
mg0781	BY916499
mg0782	AU002336
mg0783	BY916500
mg0785	AU002338
mg0786	AU002339
mg0787	BY916501
mg0788	AU002340
mg0789	BY916502
mg0790	AU002341
mg0791	AU002342
mg0793	AU002344
mg0794	BY916503
mg0797	AU002345
mg0799	AU002346
mg0800	AU002347
mg0801	BY916504
mg0802	AU002348
mg0803	BY916505

mg0804	BY916506
mg0805	AU002349
mg0806	AU002350
mg0807	AU002351
mg0808	AU002352
mg0809	AU002353
mg0810	AU002354
mg0811	AU002355
mg0812	AU002356
mg0813	AU002357
mg0814	AU002358
mg0815	AU002359
mg0816	AU002360
mg0818	AU002361
mg0819	AU002362
mg0820	AU002363
mg0822	AU002365
mg0824	AU002366
mg0825	AU002367
mg0826	AU002368
mg0827	AU002369
mg0828	AU002370
mg0830	AU002371
mg0831	AU002372
mg0832	AU002373
mg0834	AU002374
mg0836	AU002376
mg0837	AU002377
mg0839	AU002379
mg0840	AU002380
mg0842	AU002381
mg0843	AU002382
mg0844	AU002383
mg0845	AU002384
mg0846	AU002385
mg0847	AU002386
mg0848	AU002387
mg0849	AU002388
mg0850	AU002389
mg0851	AU002390
mg0852	AU002391
mg0853	BY916507
mg0854	AU002392
mg0855	AU002393
mg0856	AU002394
mg0857	AU002395
mg0858	AU002396
mg0859	AU002397
mg0860	AU002398
mg0861	AU002399
mg0862	AU002400
mg0863	AU002401
mg0864	AU002402
mg0867	AU002403

mg0868	AU002404
mg0869	AU002405
mg0870	AU002406
mg0871	BY916508
mg0872	AU002407
mg0875	AU002408
mg0877	BY916509
mg0881	BY916510
mg0883	AU002409
mg0885	BY916511
mg0886	AU002410
mg0887	AU002411
mg0888	AU002412
mg0889	BY916512
mg0891	BY916513
mg0892	BY916514
mg0896	AU002414
mg0898	BY916515
mg0900	AU002415
mg0903	AU002416
mg0904	BY916516
mg0905	AU002417
mg0906	BY916517
mg0907	AU002418
mg0908	BY916518
mg0909	AU002419
mg0911	AU002420
mg0912	BY916519
mg0913	AU002421
mg0914	BY916520
mg0915	BY916521
mg0916	BY916522
mg0918	BY916523
mg0919	AU002422
mg0922	BY916524
mg0923	AU002423
mg0926	BY916525
mg0927	AU002424
mg0929	BY916526
mg0930	AU002425
mg0931	AU002426
mg0932	AU002427
mg0933	BY916527
mg0934	BY916528
mg0935	BY916529
mg0936	BY916530
mg0937	BY916531
mg0938	BY916532
mg0939	AU002428
mg0940	BY916533
mg0941	AU002429
mg0942	BY916534
mg0943	BY916535
mg0944	AU002430

	mg0945	AU002431
	mg0946	AU002432
	mg0947	BY916536
	mg0948	AU002433
	mg0949	AU002434
	mg0950	AU002435
	mg0951	AU002436
	mg0952	AU002437
	mg0953	AU002438
	mg0955	AU002440
	mg0957	BY916537
	mg0958	AU002441
	mg0959	AU002442
	mg0960	BY916538
	mg0961	BY916539
	mg0964	AU002445
	mg0967	AU002447
	mg0968	AU002448
	mg0969	AU002449
	mg0970	AU002450
	mg0971	AU002451
	mg0972	AU002452
	mg0973	AU002453
	mg0974	AU002454
	mg0975	AU002455
	mg0976	AU002456
	mg0977	AU002457
	mg0978	AU002458
	mg0980	AU002460
	mg0981	AU002461
	mg0982	AU002462
	mg0984	BY916540
	mg0985	BY916541
	mg0987	AU002465
	mg0988	BY916542
	mg0989	BY916543
	mg0990	AU002466
	mg0992	AU002468
	mg0993	AU002469
	mg0997	AU002473
	mg0998	AU002474
	mg1000	AU002476
n	n0001	AU002477
	n0002	AU002478
	n0003	AU002479
	n0004	AU002480
	n0005	AU002481
	n0008	AU002484
	n0009	AU002485
	n0011	AU002486
	n0012	AU002487
	n0013	AU002488
	n0014	AU002489
	n0015	BY916544

n0016	AU002490
n0017	BY916545
n0018	AU002491
n0019	BY916546
n0020	AU002492
n0021	AU002493
n0022	AU002494
n0023	AU002495
n0024	AU002496
n0025	AU002497
n0026	AU002498
n0027	BY916547
n0029	AU002499
n0030	AU002500
n0031	AU002501
n0032	AU002502
n0033	AU002503
n0034	AU002504
n0035	AU002505
n0036	AU002506
n0037	AU002507
n0038	BY916548
n0039	BY916549
n0040	AU002508
n0041	AU002509
n0042	AU002510
n0043	AU002511
n0044	AU002512
n0047	BY916550
n0049	AU002515
n0050	BY916551
n0051	BY916552
n0052	BY916553
n0053	AU002516
n0054	BY916554
n0055	BY916555
n0056	AU002517
n0058	AU002518
n0059	BY916556
n0060	AU002519
n0061	BY916557
n0062	BY916558
n0066	BY916559
n0067	BY916560
n0068	BY916561
n0069	AU002521
n0070	BY916562
n0071	AU002522
n0072	AU002523
n0073	AU002524
n0074	AU002525
n0075	AU002526
n0076	AU002527
n0078	BY916563

n0080	BY916564
n0082	AU002529
n0083	BY916565
n0085	AU002530
n0086	AU002531
n0087	AU002532
n0088	AU002533
n0089	BY916566
n0090	AU002534
n0091	BY916567
n0092	AU002535
n0093	BY916568
n0095	BY916569
n0096	BY916570
n0097	AU002536
n0098	AU002537
n0100	BY916571
n0101	AU002539
n0103	AU002540
n0105	AU002542
n0106	AU002543
n0107	BY916572
n0108	AU002544
n0109	AU002545
n0110	BY916573
n0112	AU002546
n0113	AU002547
n0114	BY916574
n0115	AU002548
n0116	BY916575
n0117	BY916576
n0119	AU002550
n0120	AU002551
n0121	AU002552
n0122	AU002553
n0125	BY916577
n0127	BY916578
n0129	AU002554
n0131	BY916579
n0132	AU002555
n0133	AU002556
n0134	AU002557
n0135	AU002558
n0136	BY916580
n0137	AU002559
n0139	BY916581
n0140	BY916582
n0142	AU002560
n0145	AU002561
n0146	BY916583
n0147	BY916584
n0148	BY916585
n0149	BY916586
n0150	BY916587

n0151	BY916588
n0153	AU002562
n0154	BY916589
n0155	AU002563
n0157	AU002565
n0158	BY916590
n0159	BY916591
n0161	BY916592
n0162	BY916593
n0165	BY916594
n0166	BY916595
n0167	BY916596
n0168	AU002566
n0172	BY916597
n0173	BY916598
n0174	BY916599
n0175	BY916600
n0176	BY916601
n0177	BY916602
n0179	BY916603
n0180	BY916604
n0182	BY916605
n0184	AU002567
n0185	BY916606
n0186	BY916607
n0187	BY916608
n0189	BY916609
n0191	AU002568
n0193	AU002569
n0194	AU002570
n0195	AU002571
n0196	AU002572
n0197	AU002573
n0199	AU002575
n0200	AU002576
n0201	AU002577
n0202	AU002578
n0203	AU002579
n0204	AU002580
n0205	AU002581
n0206	BY916610
n0207	AU002582
n0208	BY916611
n0209	AU002583
n0210	AU002584
n0211	AU002585
n0212	AU002586
n0214	AU002588
n0215	AU002589
n0217	AU002591
n0218	AU002592
n0219	AU002593
n0220	AU002594
n0221	AU002595

n0222	AU002596
n0223	AU002597
n0225	AU002599
n0226	AU002600
n0227	AU002601
n0228	AU002602
n0229	AU002603
n0230	AU002604
n0231	AU002605
n0232	AU002606
n0233	AU002607
n0234	AU002608
n0237	AU002610
n0238	BY916612
n0239	AU002611
n0240	AU002612
n0241	AU002613
n0242	AU002614
n0243	AU002615
n0244	AU002616
n0245	AU002617
n0246	AU002618
n0247	AU002619
n0248	AU002620
n0249	AU002621
n0250	AU002622
n0251	BY916613
n0252	AU002623
n0253	AU002624
n0254	AU002625
n0255	AU002626
n0256	AU002627
n0257	BY916614
n0258	AU002628
n0259	AU002629
n0260	AU002630
n0261	AU002631
n0262	AU002632
n0263	AU002633
n0264	AU002634
n0265	BY916615
n0266	AU002635
n0267	AU002636
n0268	AU002637
n0269	AU002638
n0271	AU002639
n0272	AU002640
n0273	AU002641
n0274	AU002642
n0275	AU002643
n0276	AU002644
n0277	AU002645
n0278	AU002646
n0279	AU002647

n0280	AU002648
n0281	BY916616
n0282	AU002649
n0283	AU002650
n0284	AU002651
n0285	AU002652
n0286	AU002653
n0288	AU002655
n0289	BY916617
n0290	BY916618
n0291	AU002656
n0292	AU002657
n0293	AU002658
n0294	AU002659
n0295	AU002660
n0296	AU002661
n0297	AU002662
n0299	AU002663
n0300	AU002664
n0301	BY916619
n0302	BY916620
n0303	AU002665
n0304	BY916621
n0305	AU002666
n0307	AU002667
n0308	AU002668
n0309	BY916622
n0310	BY916623
n0315	AU002669
n0316	AU002670
n0317	AU002671
n0318	AU002672
n0319	AU002673
n0320	BY916624
n0322	AU002675
n0323	BY916625
n0324	BY916626
n0325	BY916627
n0326	AU002676
n0327	BY916628
n0328	AU002677
n0329	AU002678
n0330	AU002679
n0331	AU002680
n0332	AU002681
n0333	AU002682
n0334	AU002683
n0335	AU002684
n0336	BY916629
n0337	BY916630
n0338	BY916631
n0339	BY916632
n0340	AU002685
n0341	AU002686

n0342	AU002687
n0344	AU002688
n0345	AU002689
n0347	AU002690
n0348	AU002691
n0349	BY916633
n0350	AU002692
n0351	AU002693
n0352	AU002694
n0353	AU002695
n0354	AU002696
n0355	AU002697
n0356	AU002698
n0357	AU002699
n0358	AU002700
n0359	AU002701
n0360	AU002702
n0361	BY916634
n0363	AU002703
n0365	AU002704
n0366	AU002705
n0367	AU002706
n0368	AU002707
n0369	AU002708
n0370	AU002709
n0371	AU002710
n0372	AU002711
n0375	AU002712
n0376	AU002713
n0377	AU002714
n0380	AU002716
n0381	AU002717
n0382	AU002718
n0384	AU002719
n0385	AU002720
n0386	AU002721
n0387	AU002722
n0388	AU002723
n0389	AU002724
n0390	AU002725
n0392	AU002726
n0393	AU002727
n0394	AU002728
n0395	AU002729
n0397	AU002730
n0398	AU002731
n0399	AU002732
n0400	AU002733
n0401	AU002734
n0402	AU002735
n0403	AU002736
n0404	AU002737
n0405	AU002738
n0407	AU002739

n0408	AU002740
n0409	AU002741
n0411	AU002742
n0412	AU002743
n0413	AU002744
n0414	AU002745
n0415	AU002746
n0416	BY916635
n0417	AU002747
n0419	AU002748
n0420	AU002749
n0421	AU002750
n0422	AU002751
n0423	AU002752
n0425	AU002753
n0426	AU002754
n0427	AU002755
n0428	AU002756
n0430	AU002757
n0431	AU002758
n0432	AU002759
n0433	AU002760
n0435	AU002762
n0436	AU002763
n0437	AU002764
n0438	AU002765
n0439	AU002766
n0440	AU002767
n0441	AU002768
n0442	AU002769
n0443	AU002770
n0444	AU002771
n0445	AU002772
n0446	AU002773
n0448	AU002774
n0449	AU002775
n0451	AU002777
n0452	AU002778
n0453	AU002779
n0455	AU002780
n0456	AU002781
n0457	AU002782
n0461	AU002784
n0462	AU002785
n0463	AU002786
n0464	AU002787
n0465	AU002788
n0466	AU002789
n0473	AU002790
n0478	AU002791
n0479	AU002792
n0483	AU002793
n0484	AU002794
n0487	AU002795

n0488	AU002796
n0492	AU002797
n0495	AU002798
n0497	AU002799
n0498	AU002800
n0501	AU002801
n0503	AU002802
n0505	BY916636
n0506	AU002803
n0507	AU002804
n0508	AU002805
n0509	AU002806
n0510	AU002807
n0511	AU002808
n0512	AU002809
n0513	AU002810
n0516	AU002811
n0519	AU002812
n0520	AU002813
n0522	AU002814
n0523	AU002815
n0524	AU002816
n0525	AU002817
n0526	AU002818
n0527	AU002819
n0528	AU002820
n0530	AU002821
n0531	AU002822
n0532	AU002823
n0533	AU002824
n0535	AU002825
n0538	AU002827
n0539	AU002828
n0542	AU002829
n0544	AU002830
n0545	AU002831
n0546	AU002832
n0547	AU002833
n0548	AU002834
n0549	AU002835
n0550	AU002836
n0551	AU002837
n0552	AU002838
n0555	AU002839
n0556	AU002840
n0557	AU002841
n0558	AU002842
n0560	AU002843
n0561	AU002844
n0563	AU002845
n0566	AU002846
n0567	AU002847
n0568	AU002848
n0570	AU002849

n0571	AU002850
n0572	AU002851
n0575	AU002852
n0577	AU002853
n0579	AU002854
n0580	AU002855
n0582	AU002856
n0583	AU002857
n0584	AU002858
n0585	AU002859
n0587	AU002860
n0589	AU002861
n0590	AU002862
n0592	AU002863
n0593	AU002864
n0595	AU002865
n0596	AU002866
n0597	AU002867
n0602	AU002868
n0603	AU002869
n0604	AU002870
n0606	AU002871
n0607	AU002872
n0608	AU002873
n0609	AU002874
n0612	AU002875
n0614	AU002876
n0615	AU002877
n0616	AU002878
n0617	AU002879
n0618	AU002880
n0622	AU002881
n0624	AU002882
n0625X	AU002883
n0626	AU002884
n0627	AU002885
n0628	AU002886
n0629	AU002887
n0630	AU002888
n0631	AU002889
n0636	AU002891
n0637	AU002892
n0639	AU002893
n0640	AU002894
n0641	AU002895
n0643	AU002896
n0645	AU002897
n0646	AU002898
n0647	AU002899
n0650	AU002900
n0651	AU002901
n0653	BY916637
n0655	AU002903
n0657	AU002904

n0658	BY916638
n0659	AU002905
n0660	AU002906
n0661	AU002907
n0662_1	BY916639
n0664	AU002910
n0666	AU002911
n0667	BY916640
n0669	AU002912
n0670	AU002913
n0671	AU002914
n0673	AU002915
n0674	AU002916
n0675	AU002917
n0676	AU002918
n0677	AU002919
n0678	AU002920
n0679	AU002921
n0681	AU002923
n0682	AU002924
n0685	AU002925
n0686	AU002926
n0687	AU002927
n0688	AU002928
n0689	AU002929
n0690	AU002930
n0691	AU002931
n0694	AU002932
n0695	AU002933
n0696	AU002934
n0697	AU002935
n0698	AU002936
n0699	AU002937
n0701	AU002938
n0702	AU002939
n0703	AU002940
n0704	AU002941
n0705	AU002942
n0706	AU002943
n0707	AU002944
n0708	AU002945
n0709	AU002946
n0711	AU002947
n0712	AU002948
n0713	AU002949
n0714	BY916641
n0716	AU002950
n0717	AU002951
n0718	AU002952
n0720	AU002953
n0722	AU002954
n0723	AU002955
n0724	AU002956
n0725	AU002957

n0726	AU002958
n0727	AU002959
n0729	AU002960
n0734	AU002962
n0735	AU002963
n0736	AU002964
n0738	AU002965
n0739	AU002966
n0740	AU002967
n0741	AU002968
n0742	AU002969
n0746	AU002970
n0747	AU002971
n0748	AU002972
n0749	AU002973
n0751	AU002974
n0753	AU002976
n0754	AU002977
n0755	AU002978
n0758	AU002979
n0759	AU002980
n0760	AU002981
n0761	AU002982
n0762	AU002983
n0763	AU002984
n0764	AU002985
n0766	AU002986
n0768	AU002987
n0769	AU002988
n0770	AU002989
n0771	AU002990
n0772	AU002991
n0773	AU002992
n0774	AU002993
n0775	AU002994
n0776	AU002995
n0777	AU002996
n0778	AU002997
n0781	AU002998
n0782	AU002999
n0783	AU003000
n0784	AU003001
n0786	AU003002
n0787	AU003003
n0788	AU003004
n0789	AU003005
n0790	AU003006
n0792	AU003007
n0793	AU003008
n0794	AU003009
n0795	AU003010
n0796	AU003011
n0797	AU003012
n0798	AU003013

n0799	AU003014
n0800	AU003015
n0802	AU003016
n0803	BY916642
n0805	AU003017
n0806	AU003018
n0807	AU003019
n0808	AU003020
n0810	AU003021
n0811	AU003022
n0813	AU003023
n0814	AU003024
n0831	AU003025
n0832	AU003026
n0833	AU003027
n0835	AU003028
n0838	AU003029
n0839	AU003030
n0848	AU003031
n0850	AU003032
n0859	AU003033
n0868	AU003034
n0869	BY916643
n0870	AU003035
n0871	AU003036
n0872	AU003037
n0873	AU003038
n0874	AU003039
n0875	AU003040
n0878	AU003041
n0879	AU003042
n0880	AU003043
n0882	AU003044
n0883	AU003045
n0885	AU003047
n0886	AU003048
n0887	AU003049
n0890	AU003050
n0891	AU003051
n0892	AU003052
n0893	AU003053
n0895	AU003054
n0896	AU003055
n0897	AU003056
n0899	AU003057
n0900	AU003058
n0901	AU003059
n0902	AU003060
n0903	AU003061
n0904	AU003062
n0905	AU003063
n0906	AU003064
n0907	AU003065
n0908	AU003066

n0909	AU003067
n0910	AU003068
n0911	AU003069
n0912	AU003070
n0915	AU003071
n0916	AU003072
n0917	AU003073
n0918	AU003074
n0919	AU003075
n0920	AU003076
n0922	AU003078
n0923	AU003079
n0924	AU003080
n0926	AU003081
n0927	AU003082
n0928	AU003083
n0930	AU003085
n0931	AU003086
n0932	AU003087
n0933	AU003088
n0934	AU003089
n0935	AU003090
n0936	AU003091
n0939	AU003092
n0941	AU003093
n0942	AU003094
n0943	AU003095
n0944	AU003096
n0945	AU003097
n0946	AU003098
n0947	AU003099
n0948	AU003100
n0950	AU003101
n0952	AU003102
n0953	AU003103
n0954	AU003104
n0957	AU003105
n0959	AU003106
n0960	AU003107
n0961	AU003108
n0962	AU003110
n0963	AU003112
n0964	AU003114
n0965	AU003116
n0966	AU003118
n0967	AU003119
n0968	AU003120
n0969	AU003121
n0970	AU003122
n0971	AU003123
n0972	AU003124
n0973	AU003125
n0974	AU003127
n0975	AU003129

n0976	AU003131
n0977	AU003133
n0978	AU003135
n0979	AU003136
n0980	AU003137
n0986	AU003141
n0987	AU003143
n0988	AU003145
n0989	AU003146
n0990	AU003148
n0991	AU003149
n0992	AU003150
n0993	AU003151
n0994	AU003152
n0995	AU003153
n0996	AU003154
n0998	AU003155
n0999	AU003157
n1000	AU003159
n1001	AU003161
n1003	AU003164
n1004	AU003165
n1005	AU003166
n1006	AU003167
n1007	AU003168
n1008	AU003169
n1009	AU003170
n1011	AU003173
n1012	AU003175
n1013	AU003177
n1014	AU003179
n1015	AU003181
n1016	AU003182
n1017	AU003183
n1018	AU003184
n1019	AU003185
n1020	AU003186
n1021	AU003187
n1022	AU003189
n1023	AU003191
n1024	AU003193
n1026	AU003195
n1027	AU003197
n1029	AU003199
n1030	AU003200
n1031	AU003201
n1034	AU003202
n1035	AU003204
n1036	AU003206
n1038	AU003210
n1039	AU003211
n1040	AU003213
n1041	AU003215
n1043	AU003216

	n1044	AU003217
	n1045	AU003218
	n1046	AU003219
	n1047	AU003221
	n1049	AU003224
	n1051	AU003227
	n1052	AU003229
	n1054	AU003231
	n1055	AU003232
	n1056	AU003233
	n1058	AU003234
	n1060	AU003237
	n1062	AU003240
	n1063	AU003242
	n1066	AU003246
	n1067	AU003248
	n1068	AU003250
	n1070	AU003252
	n1071	AU003254
	n1073	AU003257
	n1075	AU003260
	n1076	AU003262
	n1077	AU003264
	n1078	AU003266
	n1079	AU003268
	n1080	AU003270
	n1084	AU003273
	n1085	AU003275
	n1086	AU003276
	n1087	AU003278
	n1089	AU003280
	n1091	AU003284
	n1092	AU003286
	n1093	AU003288
	n1094	AU003290
	n1096	AU003293
	n1097	AU003295
	n1099	AU003297
NV06	NV060053	AV398588
	NV060054	AV398589
	NV060055	AV398590
	NV060056	AV398591
	NV060057	AV398592
	NV060058	BY916867
	NV060065	AV398595
	NV060066	AV398596
	NV060067	AV398597
	NV060068	BY916868
	NV060069	AV398598
	NV060070	AV398599
	NV060071	AV398600
	NV060072	AV398601
	NV060079	AV398604
	NV060081	AV398605

NV060082	AV398606
NV060083	AV398607
NV060086	AV398610
NV060087	AV398611
NV060089	AV398613
NV060090	AV398614
NV060091	AV398615
NV060093	AV398616
NV060094	AV398617
NV060095	AV398618
NV060097	AV398620
NV060099	AV398622
NV060100	AV398623
NV060103	AV398625
NV060104	AV398626
NV060105	AV398627
NV060106	AV398628
NV060109	AV398631
NV060110	AV398632
NV060111	AV398633
NV060115	AV398636
NV060116	AV398637
NV060119	AV398639
NV060121	AV398641
NV060123	AV398643
NV060125	AV398645
NV060126	AV398646
NV060128	AV398648
NV060129	AV398649
NV060131	AV398651
NV060132	AV398652
NV060134	AV398654
NV060136	AV398656
NV060137	AV398657
NV060138	AV398658
NV060142	AV398662
NV060144	AV398663
NV060145	AV398664
NV060146	AV398665
NV060147	AV398666
NV060148	BY916869
NV060149	AV398667
NV060151	AV398669
NV060152	AV398670
NV060153	BY916870
NV060154	AV398671
NV060160	AV398676
NV060164	AV398679
NV060165	AV398680
NV060166	AV398681
NV060167	AV398682
NV060170	AV398684
NV060174	AV398688
NV060176	AV398690

NV060178	AV398692
NV060179	AV398693
NV060182	AV398696
NV060184	AV398698
NV060186	AV398700
NV060187	AV398701
NV060188	AV398702
NV060189	AV398703
NV060190	AV398704
NV060191	AV398705
NV060192	AV398706
NV060245	AV398753
NV060246	AV398754
NV060247	AV398755
NV060248	AV398756
NV060249	AV398757
NV060250	AV398758
NV060251	AV398759
NV060252	AV398760
NV060257	AV398763
NV060258	AV398764
NV060259	AV398765
NV060260	AV398766
NV060262	AV398768
NV060263	AV398769
NV060264	AV398770
NV060270	AV398775
NV060271	AV398776
NV060272	AV398777
NV060273	AV398778
NV060274	AV398779
NV060275	AV398780
NV060276	AV398781
NV060282	AV398787
NV060283	AV398788
NV060285	AV398790
NV060289	AV398793
NV060291	AV398794
NV060292	AV398795
NV060294	AV398797
NV060295	AV398798
NV060297	AV398800
NV060298	AV398801
NV060299	AV398802
NV060300	AV398803
NV060301	AV398804
NV060302	AV398805
NV060303	AV398806
NV060305	AV398808
NV060306	AV398809
NV060307	AV398810
NV060308	AV398811
NV060310	AV398813
NV060311	AV398814

NV060312	AV398815
NV060313	AV398816
NV060314	AV398817
NV060315	AV398818
NV060317	AV398820
NV060318	AV398821
NV060320	AV398822
NV060322	AV398824
NV060323	AV398825
NV060325	AV398826
NV060327	AV398827
NV060330	BY916871
NV060334	AV398832
NV060335	AV398833
NV060336	AV398834
NV060337	AV398835
NV060338	AV398836
NV060339	AV398837
NV060342	AV398839
NV060343	AV398840
NV060345	BY916872
NV060347	AV398843
NV060349	AV398845
NV060350	AV398846
NV060352	AV398848
NV060353	AV398849
NV060354	AV398850
NV060355	AV398851
NV060359	AV398855
NV060360	AV398856
NV060361	AV398857
NV060364	AV398860
NV060365	AV398861
NV060366	AV398862
NV060367	AV398863
NV060369	AV398864
NV060370	AV398865
NV060371	AV398866
NV060372	AV398867
NV060484	AV398968
NV060485	AV398969
NV060486	AV398970
NV060487	AV398971
NV060488	AV398972
NV060489	AV398973
NV060490	AV398974
NV060496	AV398980
NV060497	AV398981
NV060498	AV398982
NV060499	AV398983
NV060500	AV398984
NV060501	AV398985
NV060502	AV398986
NV060503	AV398987

NV060509	AV398989
NV060510	AV398990
NV060511	AV398991
NV060512_1	BY916873
NV060513	AV398993
NV060514	BY916874
NV060515	AV398994
NV060520	BY916875
NV060521	AV398999
NV060522	AV399000
NV060523	AV399001
NV060524	BY916876
NV060525	AV399002
NV060526	AV399003
NV060527	AV399004
NV060528_1	BY916877
NV060533	AV399009
NV060535	AV399011
NV060536	AV399012
NV060537	BY916878
NV060538	AV399013
NV060539	AV399014
NV060544	AV399016
NV060545	AV399017
NV060546	AV399018
NV060547	BY916879
NV060548	BY916880
NV060549	AV399019
NV060551	AV399020
NV060557	AV399021
NV060559	AV399022
NV060561	AV399023
NV060563	BY916881
NV060570	AV399026
NV060574	AV399028
NV060575	AV399029
NV060576	AV399030
NV060577	AV399031
NV060578	AV399032
NV060579	AV399033
NV060580	AV399034
NV060582	AV399035
NV060583	AV399036
NV060585	AV399037
NV060587	AV399039
NV060588	AV399040
NV060589	AV399041
NV060590	AV399042
NV060593	AV399043
NV060594	AV399044
NV060595	AV399045
NV060597	AV399046
NV060598	AV399047
NV060599	AV399048

NV060600	AV399049
NV060601	AV399050
NV060602	AV399051
NV060603	AV399052
NV060604	AV399053
NV060606	BY916882
NV060607	AV399054
NV060608	AV399055
NV060609	AV399056
NV060613	AV399059
NV060614	AV399060
NV060616	AV399061
NV060617	AV399062
NV060618	AV399063
NV060619	AV399064
NV060621	AV399065
NV060622	AV399066
NV060623	AV399067
NV060624	AV399068
NV060626	AV399070
NV060627	AV399071
NV060628	AV399072
NV060629	AV399073
NV060630	AV399074
NV060631	AV399075
NV060632	AV399076
NV060634	BY916883
NV060635	AV399078
NV060636	AV399079
NV060637	AV399080
NV060639	AV399081
NV060640	AV399082
NV060641	AV399083
NV060642	AV399084
NV060645	AV399086
NV060646	AV399087
NV060647	AV399088
NV060648	AV399089
NV060649	AV399090
NV060650	AV399091
NV060651	AV399092
NV060653	BY916884
NV060654	AV399093
NV060655	AV399094
NV060656	AV399095
NV060657	AV399096
NV060660	AV399098
NV060661	AV399099
NV060663	AV399100
NV060664	AV399101
NV060665	AV399102
NV060669	AV399105
NV060670	AV399106
NV060671	AV399107

	NV060672	AV399108
	NV060961	AV399246
	NV060962	AV399247
	NV060963	BY916885
	NV060964	AV399248
	NV060965	AV399249
	NV060966	AV399250
	NV060968	AV399251
	NV060969	BY916886
	NV060970	AV399252
	NV060971	AV399253
	NV060972	AV399254
	NV060973	AV399255
	NV060975	AV399257
	NV060977	AV399259
	NV060978	AV399260
	NV060979	AV399261
	NV060980	AV399262
	NV060981	AV399263
	NV060982	AV399264
	NV060984	AV399265
	NV060986	AV399266
	NV060987	AV399267
	NV060988	BY916887
	NV060989	AV399268
	NV060990	AV399269
	NV060991	AV399270
NV12	NV120002	AV399271
	NV120006	AV399275
	NV120007	AV399276
	NV120008	AV399277
	NV120016	AV399280
	NV120024	AV399286
	NV120036	AV399294
	NV120039	AV399296
	NV120044	AV399301
	NV120048	AV399305
	NV120052	AV399308
	NV120053	AV399309
	NV120056	AV399311
	NV120059	AV399314
	NV120060	AV399315
	NV120062	AV399316
	NV120064	AV399318
	NV120070X	AV399324
	NV120071	AV399325
	NV120072	AV399326
	NV120073	AV399327
	NV120076	AV399329
	NV120077	AV399330
	NV120078	AV399331
	NV120081	AV399334
	NV120083	AV399336
	NV120087	AV399338

NV120092	AV399342
NV120093	AV399343
NV120096	AV399346
NV120097	AV399347
NV120105	AV399355
NV120110	AV399358
NV120111	AV399359
NV120112	AV399360
NV120115	AV399362
NV120117	AV399363
NV120119	AV399364
NV120125	AV399368
NV120126	AV399369
NV120127	AV399370
NV120130	AV399371
NV120137	AV399375
NV120138	AV399376
NV120140	AV399378
NV120143	AV399380
NV120148	AV399382
NV120152	AV399384
NV120154	AV399385
NV120155	AV399386
NV120161	AV399389
NV120165	AV399393
NV120168	AV399395
NV120184	AV399403
NV120189	AV399406
NV120191	AV399407
NV120198	AV399413
NV120200	AV399415
NV120201	AV399416
NV120202	AV399417
NV120203	AV399418
NV120206	AV399421
NV120210	AV399424
NV120211	AV399425
NV120217	AV399430
NV120223	AV399433
NV120224	AV399434
NV120227	AV399436
NV120228	AV399437
NV120234	AV399442
NV120236	AV399443
NV120241	AV399447
NV120245	AV399450
NV120247X	AV399452
NV120248	AV399453
NV120249	AV399454
NV120258	AV399461
NV120259	AV399462
NV120260	AV399463
NV120263	AV399466
NV120265	AV399468

NV120266	AV399469
NV120267	AV399470
NV120271	AV399472
NV120273	AV399474
NV120275	AV399476
NV120279	AV399480
NV120281	AV399481
NV120283	AV399483
NV120286	AV399485
NV120288	AV399486
NV120290	AV399488
NV120294	AV399492
NV120297	AV399495
NV120303	AV399499
NV120304	AV399500
NV120305	AV399501
NV120307	AV399503
NV120311	AV399504
NV120312	AV399505
NV120318	AV399506
NV120321	AV399508
NV120322	AV399509
NV120324	AV399511
NV120331X	AV399516
NV120332	AV399517
NV120341	AV399520
NV120343	AV399522
NV120346	AV399525
NV120347	AV399526
NV120349	AV399528
NV120355	AV399530
NV120358	AV399532
NV120363	AV399535
NV120364	AV399536
NV120366	AV399538
NV120369	AV399539
NV120371	AV399540
NV120372	AV399541
NV120376	AV399542
NV120379	AV399545
NV120382	AV399548
NV120383	AV399549
NV120384	AV399550
NV120387	AV399552
NV120391	AV399554
NV120392	AV399555
NV120398X	AV399558
NV120400	AV399560
NV120401	AV399561
NV120402	AV399562
NV120405	AV399565
NV120409	AV399569
NV120411	AV399571
NV120419	AV399579

NV120420	AV399580
NV120421	AV399581
NV120424	AV399583
NV120430	AV399587
NV120431X	AV399588
NV120435X	AV399592
NV120436X	AV399593
NV120437	AV399594
NV120439	AV399596
NV120440	AV399597
NV120442	AV399599
NV120445	AV399602
NV120447	AV399604
NV120449	AV399606
NV120451	AV399607
NV120453	AV399608
NV120455X	AV399610
NV120460	AV399613
NV120464	AV399617
NV120466	AV399618
NV120469	AV399621
NV120473	AV399625
NV120475	AV399627
NV120479	AV399629
NV120483	AV399630
NV120486	AV399631
NV120491	AV399636
NV120494	AV399638
NV120499	AV399641
NV120503	AV399645
NV120507	AV399647
NV120509	AV399649
NV120511	AV399650
NV120513	AV399651
NV120515	AV399652
NV120518	AV399654
NV120521	AV399656
NV120526	AV399661
NV120531	AV399664
NV120538	AV399669
NV120539X	AV399670
NV120542	AV399672
NV120544	AV399674
NV120549	AV399679
NV120551	AV399681
NV120552	AV399682
NV120554	AV399683
NV120558	AV399687
NV120562X	AV399691
NV120567	AV399693
NV120568	AV399694
NV120569	AV399695
NV120570	AV399696
NV120571	AV399697

NV120575	AV399700
NV120578X	AV399702
NV120582	AV399704
NV120584X	AV399706
NV120589	AV399709
NV120612	BY916888
NV120613	AV399722
NV120614X	BY916889
NV120615	AV399723
NV120620	BY916890
NV120621	AV399726
NV120636	BY916891
NV120638	AV399727
NV120644	AV399729
NV120650	BY916892
NV120653	BY916893
NV120658	AV399735
NV120659	AV399736
NV120660	BY916894
NV120668	BY916895
NV120670	BY916896
NV120671	BY916897
NV120676	AV399740
NV120677	AV399741
NV120681	AV399745
NV120688	BY916898
NV120689	AV399746
NV120690	BY916899
NV120696	AV399750
NV120698	BY916900
NV120702	BY916901
NV120703	AV399752
NV120705	BY916902
NV120710	BY916903
NV120711	AV399754
NV120712	AV399755
NV120718	BY916904
NV120723	BY916905
NV120725	AV399759
NV120728	BY916906
NV120731	AV399761
NV120734	BY916907
NV120736	BY916908
NV120742	AV399764
NV120743	AV399765
NV120750	BY916909
NV120754	AV399771
NV120761	AV399775
NV120764	AV399777
NV120767	AV399778
NV120774	BY916910
NV120775	BY916911
NV120777	AV399781
NV120779X_1	BY916912

NV120788	AV399789
NV120790	AV399791
NV120791	AV399792
NV120797	AV399798
NV120798	AV399799
NV120806	AV399806
NV120811	AV399811
NV120816	AV399814
NV120822	AV399819
NV120823	AV399820
NV120825	AV399822
NV120827	BY916913
NV120830	AV399825
NV120833	AV399827
NV120840	AV399832
NV120843	AV399833
NV120847	AV399835
NV120848	AV399836
NV120850	AV399838
NV120851	AV399839
NV120854	AV399841
NV120855	AV399842
NV120856	AV399843
NV120857	AV399844
NV120860	AV399847
NV120863	AV399850
NV120864	AV399851
NV120865	AV399852
NV120870	AV399856
NV120873	AV399859
NV120874	AV399860
NV120882	AV399865
NV120885	AV399867
NV120891	AV399871
NV120894	AV399874
NV120896	AV399876
NV120898	AV399878
NV120905	AV399884
NV120909	AV399888
NV120912	AV399891
NV120919	AV399897
NV120921	AV399899
NV120922	AV399900
NV120928	BY916914
NV120929	BY916915
NV120930	BY916916
NV120931	BY916917
NV120932	BY916918
NV120939X	BY916919
NV120941	BY916920
NV120944	AV399902
NV120945	AV399903
NV120947	AV399904
NV120950	AV399907

	NV120951	AV399908
	NV120959	AV399915
	NV120960	AV399916
ovS3	ovS3001A02r	BB982032
	ovS3001A03r	BB982033
	ovS3001A04r	BB982034
	ovS3001A05r	BB982035
	ovS3001A06r	BB982036
	ovS3001A08r	BB982037
	ovS3001A09r	BB982038
	ovS3001A12r	BB982039
	ovS3001B03r	BB982040
	ovS3001B04r	BB982041
	ovS3001B05r	BB982042
	ovS3001B06r	BB982043
	ovS3001B07r	BB982044
	ovS3001B08r	BB982045
	ovS3001B09r	BB982046
	ovS3001B11r	BB982047
	ovS3001B12r	BB982048
	ovS3001C02r	BB982049
	ovS3001C03r	BB982050
	ovS3001C04r	BB982051
	ovS3001C05r	BB982052
	ovS3001C06r	BB982053
	ovS3001C07r	BB982054
	ovS3001C08r	BB982055
	ovS3001C09r	BB982056
	ovS3001C10r	BB982057
	ovS3001C11r	BB982058
	ovS3001D02r	BB982059
	ovS3001D03r	BB982060
	ovS3001D04r	BB982061
	ovS3001D06r	BB982062
	ovS3001D07r	BB982063
	ovS3001D08r	BB982064
	ovS3001D09r	BB982065
	ovS3001D10r	BB982066
	ovS3001E02r	BB982067
	ovS3001E03r	BB982068
	ovS3001E04r	BB982069
	ovS3001E05r	BB982070
	ovS3001E08r	BB982071
	ovS3001E09r	BB982072
	ovS3001E10r	BB982073
	ovS3001E12r	BB982074
	ovS3001F01r	BB982075
	ovS3001F02r	BB982076
	ovS3001F03r	BB982077
	ovS3001F05r	BB982078
	ovS3001F06f	BB982079
	ovS3001F06r	BB982080
	ovS3001F07r	BB982081
	ovS3001F08r	BB982082

ovS3001F09r	BB982083
ovS3001F10r	BB982084
ovS3001F11r	BB982085
ovS3001F12r	BB982086
ovS3001G01r	BB982087
ovS3001G02r	BB982088
ovS3001G05r	BB982089
ovS3001G08r	BB982090
ovS3001G09r	BB982091
ovS3001G11r	BB982092
ovS3001G12r	BB982093
ovS3001H01r	BB982094
ovS3001H03r	BB982095
ovS3001H04r	BB982096
ovS3001H05r	BB982097
ovS3001H06r	BB982098
ovS3001H07r	BB982099
ovS3001H08r	BB982100
ovS3001H09r	BB982101
ovS3001H11r	BB982102
ovS3002A01r	BB982103
ovS3002A04r	BB982104
ovS3002A06r	BB982105
ovS3002A07r	BB982106
ovS3002A08r	BB982107
ovS3002A10r	BB982108
ovS3002A11r	BB982109
ovS3002A12r	BB982110
ovS3002B01r	BB982111
ovS3002B02r	BB982112
ovS3002B03r	BB982113
ovS3002B04r	BB982114
ovS3002B07r	BB982115
ovS3002B08r	BB982116
ovS3002B09r	BB982117
ovS3002B11r	BB982118
ovS3002C01r	BB982119
ovS3002C02r	BB982120
ovS3002C03r	BB982121
ovS3002C04r	BB982122
ovS3002C07r	BB982123
ovS3002C08r	BB982124
ovS3002C09r	BB982125
ovS3002C11r	BB982126
ovS3002C12r	BB982127
ovS3002D01r	BB982128
ovS3002D02r	BB982129
ovS3002D03r	BB982130
ovS3002D05r	BB982131
ovS3002D06r	BB982132
ovS3002D07r	BB982133
ovS3002D08r	BB982134
ovS3002D09r	BB982135
ovS3002D10r	BB982136

ovS3002D11r	BB982137
ovS3002E01r	BB982138
ovS3002E02r	BB982139
ovS3002E03r	BB982140
ovS3002E04r	BB982141
ovS3002E05r	BB982142
ovS3002E06r	BB982143
ovS3002E07r	BB982144
ovS3002E08r	BB982145
ovS3002E11r	BB982146
ovS3002E12r	BB982147
ovS3002F01r	BB982148
ovS3002F02r	BB982149
ovS3002F03r	BB982150
ovS3002F04r	BB982151
ovS3002F06r	BB982152
ovS3002F07r	BB982153
ovS3002F08r	BB982154
ovS3002F09r	BB982155
ovS3002F10r	BB982156
ovS3002F12r	BB982157
ovS3002G01r	BB982158
ovS3002G02r	BB982159
ovS3002G04r	BB982160
ovS3002G05r	BB982161
ovS3002G07r	BB982162
ovS3002G08r	BB982163
ovS3002G11r	BB982164
ovS3002H01r	BB982165
ovS3002H02r	BB982166
ovS3002H04r	BB982167
ovS3002H05r	BB982168
ovS3002H06r	BB982169
ovS3002H07r	BB982170
ovS3002H08r	BB982171
ovS3002H10r	BB982172
ovS3002H11r	BB982173
ovS3002H12r	BB982174
ovS3003A02r	BB982175
ovS3003A03r	BB982176
ovS3003A04r	BB982177
ovS3003A05r	BB982178
ovS3003A06r	BB982179
ovS3003A08r	BB982180
ovS3003A09r	BB982181
ovS3003A11r	BB982182
ovS3003A12r	BB982183
ovS3003B01r	BB982184
ovS3003B02r	BB982185
ovS3003B03r	BB982186
ovS3003B04r	BB982187
ovS3003B06r	BB982188
ovS3003B11r	BB982189
ovS3003B12r	BB982190

ovS3003C01r	BB982191
ovS3003C02r	BB982192
ovS3003C03r	BB982193
ovS3003C04r	BB982194
ovS3003C05r	BB982195
ovS3003C07r	BB982196
ovS3003C09r	BB982197
ovS3003C11r	BB982198
ovS3003C12r	BB982199
ovS3003D01r	BB982200
ovS3003D04r	BB982201
ovS3003D05r	BB982202
ovS3003D06r	BB982203
ovS3003D07r	BB982204
ovS3003D08r	BB982205
ovS3003D09r	BB982206
ovS3003D10r	BB982207
ovS3003D11r	BB982208
ovS3003E03r	BB982209
ovS3003E04r	BB982210
ovS3003E07r	BB982211
ovS3003E08r	BB982212
ovS3003E10r	BB982213
ovS3003E11r	BB982214
ovS3003E12r	BB982215
ovS3003F01r	BB982216
ovS3003F02r	BB982217
ovS3003F03r	BB982218
ovS3003F04r	BB982219
ovS3003F05r	BB982220
ovS3003F06r	BB982221
ovS3003F07r	BB982222
ovS3003F09r	BB982223
ovS3003F10r	BB982224
ovS3003F12r	BB982225
ovS3003G02r	BB982226
ovS3003G03r	BB982227
ovS3003G06r	BB982228
ovS3003G07r	BB982229
ovS3003G08r	BB982230
ovS3003G09r	BB982231
ovS3003G10r	BB982232
ovS3003G11r	BB982233
ovS3003G12r	BB982234
ovS3003H01r	BB982235
ovS3003H02r	BB982236
ovS3003H03r	BB982237
ovS3003H04r	BB982238
ovS3003H06r	BB982239
ovS3003H07r	BB982240
ovS3003H08r	BB982241
ovS3003H09r	BB982242
ovS3003H10r	BB982243
ovS3003H12r	BB982244

ovS3004A01r	BB982245
ovS3004A02r	BB982246
ovS3004A03r	BB982247
ovS3004A04r	BB982248
ovS3004A05r	BB982249
ovS3004A06r	BB982250
ovS3004A07r	BB982251
ovS3004A08r	BB982252
ovS3004A11r	BB982253
ovS3004A12r	BB982254
ovS3004B01r	BB982255
ovS3004B02r	BB982256
ovS3004B03r	BB982257
ovS3004B05r	BB982258
ovS3004B07r	BB982259
ovS3004B10r	BB982260
ovS3004B12r	BB982261
ovS3004C01r	BB982262
ovS3004C02r	BB982263
ovS3004C04r	BB982264
ovS3004C05r	BB982265
ovS3004C06r	BB982266
ovS3004C08r	BB982267
ovS3004C11r	BB982268
ovS3004D01r	BB982269
ovS3004D02r	BB982270
ovS3004D03r	BB982271
ovS3004D04r	BB982272
ovS3004D07r	BB982273
ovS3004D08r	BB982274
ovS3004D09r	BB982275
ovS3004D10r	BB982276
ovS3004D11r	BB982277
ovS3004D12r	BB982278
ovS3004E03r	BB982279
ovS3004E04r	BB982280
ovS3004E08r	BB982281
ovS3004E10r	BB982282
ovS3004E12r	BB982283
ovS3004F01r	BB982284
ovS3004F03r	BB982285
ovS3004F04r	BB982286
ovS3004F05r	BB982287
ovS3004F08f	BB982288
ovS3004F09r	BB982289
ovS3004F10r	BB982290
ovS3004G02r	BB982291
ovS3004G04r	BB982292
ovS3004G05r	BB982293
ovS3004G06r	BB982294
ovS3004G07r	BB982295
ovS3004G11r	BB982296
ovS3004G12r	BB982297
ovS3004H01r	BB982298

ovS3004H02r	BB982299
ovS3004H03r	BB982300
ovS3004H05r	BB982301
ovS3004H06r	BB982302
ovS3004H07r	BB982303
ovS3004H08r	BB982304
ovS3004H09r	BB982305
ovS3004H10r	BB982306
ovS3004H11r	BB982307
ovS3004H12r	BB982308
ovS3005A01r	BB982309
ovS3005A02r	BB982310
ovS3005A04r	BB982311
ovS3005A05r	BB982312
ovS3005A06r	BB982313
ovS3005A08r	BB982314
ovS3005A09r	BB982315
ovS3005A10r	BB982316
ovS3005A11r	BB982317
ovS3005A12r	BB982318
ovS3005B02r	BB982319
ovS3005B03r	BB982320
ovS3005B04r	BB982321
ovS3005B05r	BB982322
ovS3005B06r	BB982323
ovS3005B07r	BB982324
ovS3005B08r	BB982325
ovS3005B09r	BB982326
ovS3005B10r	BB982327
ovS3005B11r	BB982328
ovS3005B12r	BB982329
ovS3005C01r	BB982330
ovS3005C02r	BB982331
ovS3005C03r	BB982332
ovS3005C04r	BB982333
ovS3005C06r	BB982334
ovS3005C07r	BB982335
ovS3005C08r	BB982336
ovS3005C09r	BB982337
ovS3005C11r	BB982338
ovS3005C12r	BB982339
ovS3005D01r	BB982340
ovS3005D02r	BB982341
ovS3005D04r	BB982342
ovS3005D06r	BB982343
ovS3005D07r	BB982344
ovS3005D08r	BB982345
ovS3005D09r	BB982346
ovS3005D10r	BB982347
ovS3005D11r	BB982348
ovS3005D12r	BB982349
ovS3005E01r	BB982350
ovS3005E02r	BB982351
ovS3005E03r	BB982352

ovS3005E06r	BB982353
ovS3005E07r	BB982354
ovS3005E08r	BB982355
ovS3005E09r	BB982356
ovS3005E10r	BB982357
ovS3005E11r	BB982358
ovS3005E12r	BB982359
ovS3005F01r	BB982360
ovS3005F02r	BB982361
ovS3005F03r	BB982362
ovS3005F04r	BB982363
ovS3005F06f	BB982364
ovS3005F08r	BB982365
ovS3005F09r	BB982366
ovS3005F10r	BB982367
ovS3005F11r	BB982368
ovS3005F12r	BB982369
ovS3005G01r	BB982370
ovS3005G03r	BB982371
ovS3005G04r	BB982372
ovS3005G05r	BB982373
ovS3005G06r	BB982374
ovS3005G08r	BB982375
ovS3005G09r	BB982376
ovS3005G10r	BB982377
ovS3005G11r	BB982378
ovS3005G12r	BB982379
ovS3005H01r	BB982380
ovS3005H02r	BB982381
ovS3005H03r	BB982382
ovS3005H04r	BB982383
ovS3005H05r	BB982384
ovS3005H06r	BB982385
ovS3005H07r	BB982386
ovS3005H08r	BB982387
ovS3005H10r	BB982388
ovS3005H11r	BB982389
ovS3005H12r	BB982390
ovS3006A03r	BB982391
ovS3006A04r	BB982392
ovS3006A05r	BB982393
ovS3006A07r	BB982394
ovS3006A08r	BB982395
ovS3006A09r	BB982396
ovS3006A11r	BB982397
ovS3006A12r	BB982398
ovS3006B01r	BB982399
ovS3006B03r	BB982400
ovS3006B05r	BB982401
ovS3006B06r	BB982402
ovS3006B07r	BB982403
ovS3006B08r	BB982404
ovS3006B10r	BB982405
ovS3006B11r	BB982406

ovS3006B12r	BB982407
ovS3006C01r	BB982408
ovS3006C02r	BB982409
ovS3006C04r	BB982410
ovS3006C05r	BB982411
ovS3006C07r	BB982412
ovS3006C10r	BB982413
ovS3006C11r	BB982414
ovS3006C12r	BB982415
ovS3006D01r	BB982416
ovS3006D02r	BB982417
ovS3006D03r	BB982418
ovS3006D07r	BB982419
ovS3006D08r	BB982420
ovS3006D09r	BB982421
ovS3006D11r	BB982422
ovS3006D12r	BB982423
ovS3006E01r	BB982424
ovS3006E02r	BB982425
ovS3006E03r	BB982426
ovS3006E04r	BB982427
ovS3006E08r	BB982428
ovS3006E10r	BB982429
ovS3006E11r	BB982430
ovS3006E12r	BB982431
ovS3006F01r	BB982432
ovS3006F02r	BB982433
ovS3006F03r	BB982434
ovS3006F04r	BB982435
ovS3006F07r	BB982436
ovS3006F09r	BB982437
ovS3006F10r	BB982438
ovS3006F11r	BB982439
ovS3006F12r	BB982440
ovS3006G02r	BB982441
ovS3006G03r	BB982442
ovS3006G04r	BB982443
ovS3006G05r	BB982444
ovS3006G06r	BB982445
ovS3006G07r	BB982446
ovS3006G09r	BB982447
ovS3006G10r	BB982448
ovS3006G11r	BB982449
ovS3006G12r	BB982450
ovS3006H01r	BB982451
ovS3006H03r	BB982452
ovS3006H05r	BB982453
ovS3006H07r	BB982454
ovS3006H08r	BB982455
ovS3006H11r	BB982456
ovS3006H12r	BB982457
ovS3007A01r	BB982458
ovS3007A03r	BB982459
ovS3007A04r	BB982460

ovS3007A05r	BB982461
ovS3007A06r	BB982462
ovS3007A07r	BB982463
ovS3007A08r	BB982464
ovS3007A10r	BB982465
ovS3007A12r	BB982466
ovS3007B01r	BB982467
ovS3007B02r	BB982468
ovS3007B03r	BB982469
ovS3007B04r	BB982470
ovS3007B05r	BB982471
ovS3007B06r	BB982472
ovS3007B07r	BB982473
ovS3007B08r	BB982474
ovS3007B10r	BB982475
ovS3007B12r	BB982476
ovS3007C01r	BB982477
ovS3007C02r	BB982478
ovS3007C03r	BB982479
ovS3007C04r	BB982480
ovS3007C07r	BB982481
ovS3007C08r	BB982482
ovS3007C09r	BB982483
ovS3007C10r	BB982484
ovS3007D01r	BB982485
ovS3007D02r	BB982486
ovS3007D03r	BB982487
ovS3007D06r	BB982488
ovS3007D07r	BB982489
ovS3007D08r	BB982490
ovS3007D09r	BB982491
ovS3007D10r	BB982492
ovS3007D11r	BB982493
ovS3007D12r	BB982494
ovS3007E01r	BB982495
ovS3007E02r	BB982496
ovS3007E03r	BB982497
ovS3007E04r	BB982498
ovS3007E05r	BB982499
ovS3007E06r	BB982500
ovS3007E07r	BB982501
ovS3007E08r	BB982502
ovS3007E10r	BB982503
ovS3007E11r	BB982504
ovS3007E12r	BB982505
ovS3007F01r	BB982506
ovS3007F02r	BB982507
ovS3007F03r	BB982508
ovS3007F04r	BB982509
ovS3007F05r	BB982510
ovS3007F06r	BB982511
ovS3007F07r	BB982512
ovS3007F08r	BB982513
ovS3007F10r	BB982514

ovS3007F11r	BB982515
ovS3007G02r	BB982516
ovS3007G03r	BB982517
ovS3007G05r	BB982518
ovS3007G06r	BB982519
ovS3007G08r	BB982520
ovS3007G09r	BB982521
ovS3007G10r	BB982522
ovS3007G11r	BB982523
ovS3007G12r	BB982524
ovS3007H01r	BB982525
ovS3007H02r	BB982526
ovS3007H04r	BB982527
ovS3007H05r	BB982528
ovS3007H06r	BB982529
ovS3007H07r	BB982530
ovS3007H08r	BB982531
ovS3007H09r	BB982532
ovS3007H10r	BB982533
ovS3007H11r	BB982534
ovS3007H12r	BB982535
ovS3008A01r	BB982536
ovS3008A03r	BB982537
ovS3008A06r	BB982538
ovS3008A07r	BB982539
ovS3008A08r	BB982540
ovS3008A09r	BB982541
ovS3008A10r	BB982542
ovS3008A12r	BB982543
ovS3008B02r	BB982544
ovS3008B03r	BB982545
ovS3008B04r	BB982546
ovS3008B05r	BB982547
ovS3008B06r	BB982548
ovS3008B07r	BB982549
ovS3008B09r	BB982550
ovS3008B10r	BB982551
ovS3008B12r	BB982552
ovS3008C01r	BB982553
ovS3008C02r	BB982554
ovS3008C03r	BB982555
ovS3008C04r	BB982556
ovS3008C06r	BB982557
ovS3008C08r	BB982558
ovS3008C09r	BB982559
ovS3008C10r	BB982560
ovS3008C11r	BB982561
ovS3008D01r	BB982562
ovS3008D03r	BB982563
ovS3008D04r	BB982564
ovS3008D06r	BB982565
ovS3008D08r	BB982566
ovS3008D09r	BB982567
ovS3008D10r	BB982568

ovS3008D12r	BB982569
ovS3008E01r	BB982570
ovS3008E02r	BB982571
ovS3008E04r	BB982572
ovS3008E05r	BB982573
ovS3008E08r	BB982574
ovS3008E10r	BB982575
ovS3008E11r	BB982576
ovS3008E12r	BB982577
ovS3008F01r	BB982578
ovS3008F02r	BB982579
ovS3008F03r	BB982580
ovS3008F04r	BB982581
ovS3008F05r	BB982582
ovS3008F06r	BB982583
ovS3008F07r	BB982584
ovS3008F08r	BB982585
ovS3008F09r	BB982586
ovS3008F10r	BB982587
ovS3008F11r	BB982588
ovS3008G01r	BB982589
ovS3008G02r	BB982590
ovS3008G03r	BB982591
ovS3008G04r	BB982592
ovS3008G05r	BB982593
ovS3008G06r	BB982594
ovS3008G07r	BB982595
ovS3008G08r	BB982596
ovS3008G09r	BB982597
ovS3008G10r	BB982598
ovS3008G11r	BB982599
ovS3008G12r	BB982600
ovS3008H01r	BB982601
ovS3008H02r	BB982602
ovS3008H03r	BB982603
ovS3008H04r	BB982604
ovS3008H05r	BB982605
ovS3008H06r	BB982606
ovS3008H07r	BB982607
ovS3008H08r	BB982608
ovS3008H10r	BB982609
ovS3008H11r	BB982610
ovS3008H12r	BB982611
ovS3009A01r	BB982612
ovS3009A02r	BB982613
ovS3009A04r	BB982614
ovS3009A06r	BB982615
ovS3009A07r	BB982616
ovS3009A08r	BB982617
ovS3009A09r	BB982618
ovS3009A10r	BB982619
ovS3009A11r	BB982620
ovS3009A12r	BB982621
ovS3009B01r	BB982622

ovS3009B03r	BB982623
ovS3009B05r	BB982624
ovS3009B06r	BB982625
ovS3009B08r	BB982626
ovS3009B10r	BB982627
ovS3009B12r	BB982628
ovS3009C04r	BB982629
ovS3009C07r	BB982630
ovS3009C08r	BB982631
ovS3009C12r	BB982632
ovS3009D01r	BB982633
ovS3009D02r	BB982634
ovS3009D03r	BB982635
ovS3009D04r	BB982636
ovS3009D06r	BB982637
ovS3009D08r	BB982638
ovS3009D09r	BB982639
ovS3009D10r	BB982640
ovS3009D11r	BB982641
ovS3009D12r	BB982642
ovS3009E01r	BB982643
ovS3009E02r	BB982644
ovS3009E03r	BB982645
ovS3009E11r	BB982646
ovS3009E12r	BB982647
ovS3009F01r	BB982648
ovS3009F02r	BB982649
ovS3009F03r	BB982650
ovS3009F06r	BB982651
ovS3009F07r	BB982652
ovS3009F10r	BB982653
ovS3009F11r	BB982654
ovS3009F12r	BB982655
ovS3009G01r	BB982656
ovS3009G03r	BB982657
ovS3009G04r	BB982658
ovS3009G07r	BB982659
ovS3009G09r	BB982660
ovS3009G12r	BB982661
ovS3009H01r	BB982662
ovS3009H02r	BB982663
ovS3009H03r	BB982664
ovS3009H04r	BB982665
ovS3009H06r	BB982666
ovS3009H07r	BB982667
ovS3009H08r	BB982668
ovS3009H10r	BB982669
ovS3009H11r	BB982670
ovS3009H12r	BB982671
ovS3010A01r	BB982672
ovS3010A03r	BB982673
ovS3010A04r	BB982674
ovS3010A05r	BB982675
ovS3010A06r	BB982676

ovS3010A07r	BB982677
ovS3010A08r	BB982678
ovS3010A09r	BB982679
ovS3010A10r	BB982680
ovS3010A11r	BB982681
ovS3010A12r	BB982682
ovS3010B01r	BB982683
ovS3010B02r	BB982684
ovS3010B03r	BB982685
ovS3010B04r	BB982686
ovS3010B05r	BB982687
ovS3010B06r	BB982688
ovS3010B07r	BB982689
ovS3010B08r	BB982690
ovS3010B09r	BB982691
ovS3010B10r	BB982692
ovS3010B11r	BB982693
ovS3010C01r	BB982694
ovS3010C02r	BB982695
ovS3010C03r	BB982696
ovS3010C06r	BB982697
ovS3010C07r	BB982698
ovS3010C08r	BB982699
ovS3010C10r	BB982700
ovS3010C11r	BB982701
ovS3010D01r	BB982702
ovS3010D02r	BB982703
ovS3010D03r	BB982704
ovS3010D04r	BB982705
ovS3010D05r	BB982706
ovS3010D09r	BB982707
ovS3010D10r	BB982708
ovS3010D12r	BB982709
ovS3010E02r	BB982710
ovS3010E03r	BB982711
ovS3010E04r	BB982712
ovS3010E05r	BB982713
ovS3010E06r	BB982714
ovS3010E07r	BB982715
ovS3010E08r	BB982716
ovS3010E09r	BB982717
ovS3010E10r	BB982718
ovS3010E12r	BB982719
ovS3010F01r	BB982720
ovS3010F02r	BB982721
ovS3010F04r	BB982722
ovS3010F05r	BB982723
ovS3010F06r	BB982724
ovS3010F07r	BB982725
ovS3010F08r	BB982726
ovS3010F10r	BB982727
ovS3010F12r	BB982728
ovS3010G02r	BB982729
ovS3010G03r	BB982730

ovS3010G05r	BB982731
ovS3010G06r	BB982732
ovS3010G07r	BB982733
ovS3010G08r	BB982734
ovS3010G10r	BB982735
ovS3010H03r	BB982736
ovS3010H04r	BB982737
ovS3010H05r	BB982738
ovS3010H07r	BB982739
ovS3010H08r	BB982740
ovS3010H10r	BB982741
ovS3010H12r	BB982742
ovS3011A02r	BB982743
ovS3011A03r	BB982744
ovS3011A04r	BB982745
ovS3011A05r	BB982746
ovS3011A06r	BB982747
ovS3011A07r	BB982748
ovS3011A08r	BB982749
ovS3011A10r	BB982750
ovS3011A12r	BB982751
ovS3011B02r	BB982752
ovS3011B03r	BB982753
ovS3011B04r	BB982754
ovS3011B06r	BB982755
ovS3011B07r	BB982756
ovS3011B08r	BB982757
ovS3011B09r	BB982758
ovS3011B11r	BB982759
ovS3011B12r	BB982760
ovS3011C01r	BB982761
ovS3011C02r	BB982762
ovS3011C05r	BB982763
ovS3011C07r	BB982764
ovS3011C08r	BB982765
ovS3011C09r	BB982766
ovS3011C10r	BB982767
ovS3011C11r	BB982768
ovS3011C12r	BB982769
ovS3011D01r	BB982770
ovS3011D02r	BB982771
ovS3011D04r	BB982772
ovS3011D05r	BB982773
ovS3011D07r	BB982774
ovS3011D09r	BB982775
ovS3011D12r	BB982776
ovS3011E01r	BB982777
ovS3011E02r	BB982778
ovS3011E04r	BB982779
ovS3011E05f	BB982780
ovS3011E06r	BB982781
ovS3011E07r	BB982782
ovS3011E10r	BB982783
ovS3011E11r	BB982784

ovS3011E12r	BB982785
ovS3011F01r	BB982786
ovS3011F02r	BB982787
ovS3011F04r	BB982788
ovS3011F06r	BB982789
ovS3011F07r	BB982790
ovS3011F09r	BB982791
ovS3011F10r	BB982792
ovS3011F11r	BB982793
ovS3011F12r	BB982794
ovS3011G01r	BB982795
ovS3011G02r	BB982796
ovS3011G03r	BB982797
ovS3011G04r	BB982798
ovS3011G05r	BB982799
ovS3011G06r	BB982800
ovS3011G07r	BB982801
ovS3011G08r	BB982802
ovS3011G10r	BB982803
ovS3011G11r	BB982804
ovS3011G12r	BB982805
ovS3011H01r	BB982806
ovS3011H02r	BB982807
ovS3011H03r	BB982808
ovS3011H04r	BB982809
ovS3011H05r	BB982810
ovS3011H06r	BB982811
ovS3011H07r	BB982812
ovS3011H09r	BB982813
ovS3011H12r	BB982814
ovS3012A01f	BB982815
ovS3012A01r	BB982816
ovS3012A02r	BB982817
ovS3012A03r	BB982818
ovS3012A05r	BB982819
ovS3012A07r	BB982820
ovS3012A08r	BB982821
ovS3012A09r	BB982822
ovS3012A10r	BB982823
ovS3012A11r	BB982824
ovS3012B01r	BB982825
ovS3012B04r	BB982826
ovS3012B05r	BB982827
ovS3012B06r	BB982828
ovS3012B07r	BB982829
ovS3012B08r	BB982830
ovS3012B09r	BB982831
ovS3012B10r	BB982832
ovS3012B11r	BB982833
ovS3012B12r	BB982834
ovS3012C01r	BB982835
ovS3012C02r	BB982836
ovS3012C05r	BB982837
ovS3012C07r	BB982838

ovS3012C08r	BB982839
ovS3012C11r	BB982840
ovS3012C12r	BB982841
ovS3012D01r	BB982842
ovS3012D02r	BB982843
ovS3012D03r	BB982844
ovS3012D05r	BB982845
ovS3012D06r	BB982846
ovS3012D07r	BB982847
ovS3012D08r	BB982848
ovS3012D09r	BB982849
ovS3012E01r	BB982850
ovS3012E02r	BB982851
ovS3012E03r	BB982852
ovS3012E04r	BB982853
ovS3012E05r	BB982854
ovS3012E06r	BB982855
ovS3012E07r	BB982856
ovS3012E08r	BB982857
ovS3012E09r	BB982858
ovS3012E11r	BB982859
ovS3012E12r	BB982860
ovS3012F01r	BB982861
ovS3012F02r	BB982862
ovS3012F03r	BB982863
ovS3012F04r	BB982864
ovS3012F06r	BB982865
ovS3012F07r	BB982866
ovS3012F10r	BB982867
ovS3012F11r	BB982868
ovS3012F12r	BB982869
ovS3012G01r	BB982870
ovS3012G02r	BB982871
ovS3012G04r	BB982872
ovS3012G07r	BB982873
ovS3012G08r	BB982874
ovS3012G11r	BB982875
ovS3012G12r	BB982876
ovS3012H01r	BB982877
ovS3012H02r	BB982878
ovS3012H03r	BB982879
ovS3012H04r	BB982880
ovS3012H05r	BB982881
ovS3012H06r	BB982882
ovS3012H08r	BB982883
ovS3012H10r	BB982884
ovS3013A01r	BB982885
ovS3013A02r	BB982886
ovS3013A03r	BB982887
ovS3013A05r	BB982888
ovS3013A07r	BB982889
ovS3013A08r	BB982890
ovS3013A09r	BB982891
ovS3013A10r	BB982892

ovS3013A12r	BB982893
ovS3013B01r	BB982894
ovS3013B03r	BB982895
ovS3013B04r	BB982896
ovS3013B05r	BB982897
ovS3013B06r	BB982898
ovS3013B07r	BB982899
ovS3013B08r	BB982900
ovS3013B09r	BB982901
ovS3013B11r	BB982902
ovS3013C01r	BB982903
ovS3013C03r	BB982904
ovS3013C04r	BB982905
ovS3013C06r	BB982906
ovS3013C08r	BB982907
ovS3013C09r	BB982908
ovS3013C10r	BB982909
ovS3013C12r	BB982910
ovS3013D01r	BB982911
ovS3013D02r	BB982912
ovS3013D03r	BB982913
ovS3013D04r	BB982914
ovS3013D05r	BB982915
ovS3013D12r	BB982916
ovS3013E01r	BB982917
ovS3013E02r	BB982918
ovS3013E03r	BB982919
ovS3013E04r	BB982920
ovS3013E05r	BB982921
ovS3013E06r	BB982922
ovS3013E07r	BB982923
ovS3013E09r	BB982924
ovS3013E10r	BB982925
ovS3013E11r	BB982926
ovS3013E12r	BB982927
ovS3013F01r	BB982928
ovS3013F02r	BB982929
ovS3013F03r	BB982930
ovS3013F04r	BB982931
ovS3013F06r	BB982932
ovS3013F07r	BB982933
ovS3013F08r	BB982934
ovS3013F09r	BB982935
ovS3013F12r	BB982936
ovS3013G01r	BB982937
ovS3013G02r	BB982938
ovS3013G03r	BB982939
ovS3013G04r	BB982940
ovS3013G05r	BB982941
ovS3013G06r	BB982942
ovS3013G07r	BB982943
ovS3013G08r	BB982944
ovS3013G09r	BB982945
ovS3013G11r	BB982946

ovS3013H01r	BB982947
ovS3013H02r	BB982948
ovS3013H03r	BB982949
ovS3013H04r	BB982950
ovS3013H07r	BB982951
ovS3013H08r	BB982952
ovS3013H09r	BB982953
ovS3013H12r	BB982954
ovS3014A03r	BB982955
ovS3014A04r	BB982956
ovS3014A05r	BB982957
ovS3014A06r	BB982958
ovS3014A07r	BB982959
ovS3014A10r	BB982960
ovS3014A12r	BB982961
ovS3014B01r	BB982962
ovS3014B03r	BB982963
ovS3014B04r	BB982964
ovS3014B06r	BB982965
ovS3014B07r	BB982966
ovS3014B08r	BB982967
ovS3014B09r	BB982968
ovS3014B10r	BB982969
ovS3014B12r	BB982970
ovS3014C01r	BB982971
ovS3014C02r	BB982972
ovS3014C03r	BB982973
ovS3014C04r	BB982974
ovS3014C06r	BB982975
ovS3014C07r	BB982976
ovS3014C08r	BB982977
ovS3014C09r	BB982978
ovS3014C10r	BB982979
ovS3014C11r	BB982980
ovS3014C12r	BB982981
ovS3014D01r	BB982982
ovS3014D02r	BB982983
ovS3014D03r	BB982984
ovS3014D04r	BB982985
ovS3014D06r	BB982986
ovS3014D07r	BB982987
ovS3014D08r	BB982988
ovS3014D10r	BB982989
ovS3014D11r	BB982990
ovS3014D12r	BB982991
ovS3014E01r	BB982992
ovS3014E02r	BB982993
ovS3014E03r	BB982994
ovS3014E04r	BB982995
ovS3014E05r	BB982996
ovS3014E06r	BB982997
ovS3014E07r	BB982998
ovS3014E09r	BB982999
ovS3014E10r	BB983000

ovS3014E11r	BB983001
ovS3014E12r	BB983002
ovS3014F01r	BB983003
ovS3014F02r	BB983004
ovS3014F03r	BB983005
ovS3014F04r	BB983006
ovS3014F05r	BB983007
ovS3014F06r	BB983008
ovS3014F07r	BB983009
ovS3014F09r	BB983010
ovS3014F10r	BB983011
ovS3014F11r	BB983012
ovS3014G01r	BB983013
ovS3014G02r	BB983014
ovS3014G03r	BB983015
ovS3014G04r	BB983016
ovS3014G05r	BB983017
ovS3014G06r	BB983018
ovS3014G07r	BB983019
ovS3014G08r	BB983020
ovS3014G09r	BB983021
ovS3014G10r	BB983022
ovS3014G11r	BB983023
ovS3014G12r	BB983024
ovS3014H03r	BB983025
ovS3014H04f	BB983026
ovS3014H04r	BB983027
ovS3014H05r	BB983028
ovS3014H06r	BB983029
ovS3014H07r	BB983030
ovS3014H08r	BB983031
ovS3014H10r	BB983032
ovS3014H12r	BB983033
ovS3015A03r	BB983034
ovS3015A04r	BB983035
ovS3015A05r	BB983036
ovS3015A08r	BB983037
ovS3015A11r	BB983038
ovS3015A12f	BB983039
ovS3015B01r	BB983040
ovS3015B02r	BB983041
ovS3015B03r	BB983042
ovS3015B04r	BB983043
ovS3015B05r	BB983044
ovS3015B07r	BB983045
ovS3015B08r	BB983046
ovS3015B09r	BB983047
ovS3015B10r	BB983048
ovS3015C01r	BB983049
ovS3015C02r	BB983050
ovS3015C03r	BB983051
ovS3015C04r	BB983052
ovS3015C05r	BB983053
ovS3015C06r	BB983054

ovS3015C07r	BB983055
ovS3015C08r	BB983056
ovS3015C09r	BB983057
ovS3015C11r	BB983058
ovS3015D01r	BB983059
ovS3015D02r	BB983060
ovS3015D03r	BB983061
ovS3015D05r	BB983062
ovS3015D06r	BB983063
ovS3015D07r	BB983064
ovS3015D08r	BB983065
ovS3015D09r	BB983066
ovS3015D11r	BB983067
ovS3015D12r	BB983068
ovS3015E01r	BB983069
ovS3015E02r	BB983070
ovS3015E03r	BB983071
ovS3015E04r	BB983072
ovS3015E05r	BB983073
ovS3015E07r	BB983074
ovS3015E08r	BB983075
ovS3015E11r	BB983076
ovS3015E12r	BB983077
ovS3015F01r	BB983078
ovS3015F02r	BB983079
ovS3015F03r	BB983080
ovS3015F04r	BB983081
ovS3015F06r	BB983082
ovS3015F08r	BB983083
ovS3015F09r	BB983084
ovS3015F10r	BB983085
ovS3015F11r	BB983086
ovS3015F12r	BB983087
ovS3015G01r	BB983088
ovS3015G03r	BB983089
ovS3015G05r	BB983090
ovS3015G07r	BB983091
ovS3015G09r	BB983092
ovS3015G12r	BB983093
ovS3015H01r	BB983094
ovS3015H02r	BB983095
ovS3015H03r	BB983096
ovS3015H06r	BB983097
ovS3015H07r	BB983098
ovS3015H08r	BB983099
ovS3015H10r	BB983100
ovS3015H11r	BB983101
ovS3015H12r	BB983102
ovS3016A01r	BB983103
ovS3016A07r	BB983104
ovS3016A08r	BB983105
ovS3016A09r	BB983106
ovS3016A10r	BB983107
ovS3016A11r	BB983108

ovS3016A12r	BB983109
ovS3016B01r	BB983110
ovS3016B02r	BB983111
ovS3016B03r	BB983112
ovS3016B04r	BB983113
ovS3016B07r	BB983114
ovS3016B08r	BB983115
ovS3016B10r	BB983116
ovS3016B11r	BB983117
ovS3016B12r	BB983118
ovS3016C01r	BB983119
ovS3016C02r	BB983120
ovS3016C03r	BB983121
ovS3016C04r	BB983122
ovS3016C06r	BB983123
ovS3016C07r	BB983124
ovS3016C09r	BB983125
ovS3016C12r	BB983126
ovS3016D01r	BB983127
ovS3016D02r	BB983128
ovS3016D03r	BB983129
ovS3016D04r	BB983130
ovS3016D05r	BB983131
ovS3016D06r	BB983132
ovS3016D07r	BB983133
ovS3016D09r	BB983134
ovS3016D10r	BB983135
ovS3016D11r	BB983136
ovS3016D12r	BB983137
ovS3016E01r	BB983138
ovS3016E02r	BB983139
ovS3016E03r	BB983140
ovS3016E04r	BB983141
ovS3016E06r	BB983142
ovS3016E07r	BB983143
ovS3016E08r	BB983144
ovS3016E10r	BB983145
ovS3016E11r	BB983146
ovS3016E12r	BB983147
ovS3016F01r	BB983148
ovS3016F02r	BB983149
ovS3016F03r	BB983150
ovS3016F06r	BB983151
ovS3016F07r	BB983152
ovS3016F08r	BB983153
ovS3016F09r	BB983154
ovS3016F10r	BB983155
ovS3016F11r	BB983156
ovS3016F12r	BB983157
ovS3016G01r	BB983158
ovS3016G02r	BB983159
ovS3016G05r	BB983160
ovS3016G06r	BB983161
ovS3016G07r	BB983162

ovS3016G08r	BB983163
ovS3016G11r	BB983164
ovS3016H01r	BB983165
ovS3016H02r	BB983166
ovS3016H03r	BB983167
ovS3016H04r	BB983168
ovS3016H07r	BB983169
ovS3016H09r	BB983170
ovS3016H10r	BB983171
ovS3016H11r	BB983172
ovS3016H12r	BB983173
ovS3017A01r	BB983174
ovS3017A02r	BB983175
ovS3017A03r	BB983176
ovS3017A06r	BB983177
ovS3017A08r	BB983178
ovS3017A09f	BB983179
ovS3017A09r	BB983180
ovS3017A10r	BB983181
ovS3017A12r	BB983182
ovS3017B02r	BB983183
ovS3017B03r	BB983184
ovS3017B04f	BB983185
ovS3017B04r	BB983186
ovS3017B05r	BB983187
ovS3017B06r	BB983188
ovS3017B07r	BB983189
ovS3017B09r	BB983190
ovS3017B10r	BB983191
ovS3017B11r	BB983192
ovS3017C01r	BB983193
ovS3017C02r	BB983194
ovS3017C04r	BB983195
ovS3017C07r	BB983196
ovS3017C08r	BB983197
ovS3017C09r	BB983198
ovS3017C10r	BB983199
ovS3017C11r	BB983200
ovS3017D01r	BB983201
ovS3017D04r	BB983202
ovS3017D05r	BB983203
ovS3017D06r	BB983204
ovS3017D07r	BB983205
ovS3017D10r	BB983206
ovS3017D12r	BB983207
ovS3017E01f	BB983208
ovS3017E02r	BB983209
ovS3017E03r	BB983210
ovS3017E05r	BB983211
ovS3017E07r	BB983212
ovS3017E08r	BB983213
ovS3017E12r	BB983214
ovS3017F01r	BB983215
ovS3017F02r	BB983216

ovS3017F04r	BB983217
ovS3017F06r	BB983218
ovS3017F07r	BB983219
ovS3017F08r	BB983220
ovS3017F09r	BB983221
ovS3017F10r	BB983222
ovS3017F12r	BB983223
ovS3017G01r	BB983224
ovS3017G02r	BB983225
ovS3017G03r	BB983226
ovS3017G07r	BB983227
ovS3017G08r	BB983228
ovS3017G09r	BB983229
ovS3017G10r	BB983230
ovS3017H01r	BB983231
ovS3017H02r	BB983232
ovS3017H04r	BB983233
ovS3017H06r	BB983234
ovS3017H09r	BB983235
ovS3017H10r	BB983236
ovS3017H11r	BB983237
ovS3017H12r	BB983238
ovS3018A01r	BB983239
ovS3018A03r	BB983240
ovS3018A04r	BB983241
ovS3018A05r	BB983242
ovS3018A07r	BB983243
ovS3018A08r	BB983244
ovS3018A09r	BB983245
ovS3018A10r	BB983246
ovS3018A11r	BB983247
ovS3018A12r	BB983248
ovS3018B01r	BB983249
ovS3018B03r	BB983250
ovS3018B04r	BB983251
ovS3018B05r	BB983252
ovS3018B06r	BB983253
ovS3018B07r	BB983254
ovS3018B08r	BB983255
ovS3018B09r	BB983256
ovS3018B10r	BB983257
ovS3018B11r	BB983258
ovS3018B12r	BB983259
ovS3018C01r	BB983260
ovS3018C02r	BB983261
ovS3018C03r	BB983262
ovS3018C04r	BB983263
ovS3018C05r	BB983264
ovS3018C06r	BB983265
ovS3018C07r	BB983266
ovS3018C08r	BB983267
ovS3018C09r	BB983268
ovS3018C10r	BB983269
ovS3018C11r	BB983270

ovS3018D01r	BB983271
ovS3018D02r	BB983272
ovS3018D03r	BB983273
ovS3018D04r	BB983274
ovS3018D05r	BB983275
ovS3018D06r	BB983276
ovS3018D07f	BB983277
ovS3018D08r	BB983278
ovS3018D10r	BB983279
ovS3018D11r	BB983280
ovS3018E01r	BB983281
ovS3018E03r	BB983282
ovS3018E04r	BB983283
ovS3018E07r	BB983284
ovS3018E09r	BB983285
ovS3018E10r	BB983286
ovS3018E11r	BB983287
ovS3018F01r	BB983288
ovS3018F02r	BB983289
ovS3018F04r	BB983290
ovS3018F05r	BB983291
ovS3018F07r	BB983292
ovS3018F08r	BB983293
ovS3018F09r	BB983294
ovS3018F10r	BB983295
ovS3018F11r	BB983296
ovS3018F12r	BB983297
ovS3018G02r	BB983298
ovS3018G03r	BB983299
ovS3018G04r	BB983300
ovS3018G05r	BB983301
ovS3018G08r	BB983302
ovS3018G09r	BB983303
ovS3018G10r	BB983304
ovS3018G11r	BB983305
ovS3018G12r	BB983306
ovS3018H02r	BB983307
ovS3018H03r	BB983308
ovS3018H04r	BB983309
ovS3018H05r	BB983310
ovS3018H06r	BB983311
ovS3018H07r	BB983312
ovS3018H08r	BB983313
ovS3018H11r	BB983314
ovS3019A01r	BB983315
ovS3019A02r	BB983316
ovS3019A03r	BB983317
ovS3019A06r	BB983318
ovS3019A07r	BB983319
ovS3019A08r	BB983320
ovS3019A09r	BB983321
ovS3019A11r	BB983322
ovS3019B01r	BB983323
ovS3019B03r	BB983324

ovS3019B04r	BB983325
ovS3019B05r	BB983326
ovS3019B07r	BB983327
ovS3019B08r	BB983328
ovS3019B09r	BB983329
ovS3019B10r	BB983330
ovS3019B12r	BB983331
ovS3019C02r	BB983332
ovS3019C03r	BB983333
ovS3019C04r	BB983334
ovS3019C05r	BB983335
ovS3019C06r	BB983336
ovS3019C07r	BB983337
ovS3019C08r	BB983338
ovS3019C10r	BB983339
ovS3019D01r	BB983340
ovS3019D02r	BB983341
ovS3019D03r	BB983342
ovS3019D04r	BB983343
ovS3019D05r	BB983344
ovS3019D06r	BB983345
ovS3019D07r	BB983346
ovS3019D08r	BB983347
ovS3019D10r	BB983348
ovS3019D11r	BB983349
ovS3019D12r	BB983350
ovS3019E01r	BB983351
ovS3019E02r	BB983352
ovS3019E03r	BB983353
ovS3019E05r	BB983354
ovS3019E07r	BB983355
ovS3019E08r	BB983356
ovS3019E09r	BB983357
ovS3019E11r	BB983358
ovS3019E12r	BB983359
ovS3019F01r	BB983360
ovS3019F04r	BB983361
ovS3019F05r	BB983362
ovS3019F08r	BB983363
ovS3019F10r	BB983364
ovS3019F12r	BB983365
ovS3019G02r	BB983366
ovS3019G03r	BB983367
ovS3019G05r	BB983368
ovS3019G07r	BB983369
ovS3019G08r	BB983370
ovS3019G09r	BB983371
ovS3019G10r	BB983372
ovS3019G11r	BB983373
ovS3019G12r	BB983374
ovS3019H01r	BB983375
ovS3019H02r	BB983376
ovS3019H03r	BB983377
ovS3019H04r	BB983378

ovS3019H06r	BB983379
ovS3019H07r	BB983380
ovS3019H08r	BB983381
ovS3019H10r	BB983382
ovS3019H11r	BB983383
ovS3019H12r	BB983384
ovS301A03f	BP179299
ovS301A04f	BP179300
ovS301A05f	BP179301
ovS301A08f	BP179304
ovS301A09f	BP179305
ovS301A12f	BP179307
ovS301B01f	BP179308
ovS301B03f	BP179309
ovS301B05f	BP179310
ovS301B06f	BP179311
ovS301B07f	BP179312
ovS301B08f	BP179313
ovS301B09f	BP179314
ovS301B11f	BP179315
ovS301B12f	BP179316
ovS301C01f	BP179317
ovS301C02f	BP179318
ovS301C04f	BP179320
ovS301C05f	BP179321
ovS301C06f	BP179322
ovS301C07f	BP179323
ovS301C08f	BP179324
ovS301C09f	BP179325
ovS301C10f	BP179326
ovS301C11f	BP179327
ovS301D01f	BP179329
ovS301D04f	BP179331
ovS301D06f	BP179332
ovS301D07f	BP179333
ovS301D08f	BP179334
ovS301D09f	BP179335
ovS301D10f	BP179336
ovS301D11f	BP179337
ovS301D12f	BP179338
ovS301E04f	BP179339
ovS301E05f	BP179340
ovS301E07f	BP179341
ovS301E08f	BP179342
ovS301E09f	BP179343
ovS301E10f	BP179344
ovS301E11f	BP179345
ovS301E12f	BP179346
ovS301F01f	BP179347
ovS301F02f	BP179348
ovS301F03f	BP179349
ovS301F04f	BP179350
ovS301F05f	BP179351
ovS301F07f	BP179352

ovS301F08f	BP179353
ovS301F09f	BP179354
ovS301F10f	BP179355
ovS301F11f	BP179356
ovS301F12f	BP179357
ovS301G01f	BP179358
ovS301G02f	BP179359
ovS301G03f	BP179360
ovS301G04f	BP179361
ovS301G05f	BP179362
ovS301G07f	BP179363
ovS301G10f	BP179364
ovS301G11f	BP179365
ovS301G12f	BP179366
ovS301H01f	BP179367
ovS301H03f	BP179368
ovS301H04f	BP179369
ovS301H05f	BP179370
ovS301H06f	BP179371
ovS301H07f	BP179372
ovS301H08f	BP179373
ovS301H09f	BP179374
ovS301H11f	BP179376
ovS3020A01r	BB983385
ovS3020A02r	BB983386
ovS3020A05r	BB983387
ovS3020A06r	BB983388
ovS3020A07r	BB983389
ovS3020A08r	BB983390
ovS3020A11r	BB983391
ovS3020A12r	BB983392
ovS3020B01r	BB983393
ovS3020B03r	BB983394
ovS3020B04r	BB983395
ovS3020B07r	BB983396
ovS3020B08r	BB983397
ovS3020B09r	BB983398
ovS3020B10r	BB983399
ovS3020B11r	BB983400
ovS3020B12r	BB983401
ovS3020C02r	BB983402
ovS3020C03r	BB983403
ovS3020C05r	BB983404
ovS3020C07r	BB983405
ovS3020C08r	BB983406
ovS3020C09r	BB983407
ovS3020C10r	BB983408
ovS3020C12r	BB983409
ovS3020D01r	BB983410
ovS3020D02r	BB983411
ovS3020D03r	BB983412
ovS3020D04r	BB983413
ovS3020D06r	BB983414
ovS3020D07r	BB983415

ovS3020D08r	BB983416
[truncated: 204,587 more chars]
